# Supplementary material for: Evaluating feature extraction in ovarian cancer cell line co-cultures using deep neural networks
Source: Commun Biol. 2025 Feb 25;8:303. doi: 10.1038/s42003-025-07766-w (PMC11862010; doi:10.1038/s42003-025-07766-w)
Supplement: Supplementary file 1 — Supplementary Information [file 42003_2025_7766_MOESM1_ESM.pdf]

Supplementary Figures for

Evaluating Feature Extraction in Ovarian Cancer Cell Line Co-Cultures Using Deep Neural Networks

Osheen Sharma[1*], Greta Gudoityte[1], Rezan Minozada[1], Olli P. Kallioniemi[1,2], Riku Turkki[2], Lassi Paavolainen[2,3], Brinton Seashore-Ludlow[1**]

[1]Department of Oncology-Pathology, Karolinska Institutet, Science for Life Laboratory, Stockholm, Sweden
[2]Institute for Molecular Medicine Finland (FIMM), HiLIFE, University of Helsinki, Helsinki, Finland
[3]iCAN Digital Precision Cancer Medicine Flagship, University of Helsinki, Helsinki, Finland

[*]Correspondence: osheen.sharma@ki.se
[**]Correspondence: brinton.seashore-ludlow@ki.se

Table of Contents

Supplementary Figure 1
Example of the fluorescent imagining dataset from co-culture assay. 3

Supplementary Figure 2
Cellpose mask for cancer channel (CK8/18). 4

Supplementary Figure 3
PCA plots from negative control wells showing morphological differences across co-culture assays derived from CellProfiler features and EfficientNetB0. 5

Supplementary Figure 4
UMAP plots of cancer cell morphological profiles derived from CellProfiler across co-culture datasets, colored by plate IDs and treatment status. 6

Supplementary Figure 5
UMAP plots of cancer cell morphological profiles derived from unmasked EfficientNetB0 across co-culture datasets, colored by plate IDs and treatment status. 7

Supplementary Figure 6
Histogram representing the number of compounds per mode of action. 8

Supplementary Figure 7
Enrichment radar plots from CellProfiler and unmasked neural networks. 9

Supplementary Figure 8
Activation maps. 10

Supplementary Figure 9
UMAP and PCA plots of morphological features were derived after masking the bounding box area. 11

Supplementary Figure 10
Cell count density plot across five co-culture assays for each MOA vs DMSO. 12

Supplementary Figure 11
Enrichment radar plots from CellProfiler and masked neural networks. 13

Supplementary Figure 12
DSS score box plot for context-dependent cytotoxicity. 14

Supplementary Figure 13
UMAP plots of cancer cell morphological profiles derived from ResNet50 across co-culture datasets, colored by plate IDs and treatment status. 15

Supplementary Figure 14
UMAP plots of cancer cell morphological profiles derived from fine-tuned ResNet50 across co-culture datasets, colored by plate IDs and treatment status. 16

Supplementary Figure 15
OVCAR8 cells demonstrate partial co-expression of Vimentin. 17

Supplementary Figure 1

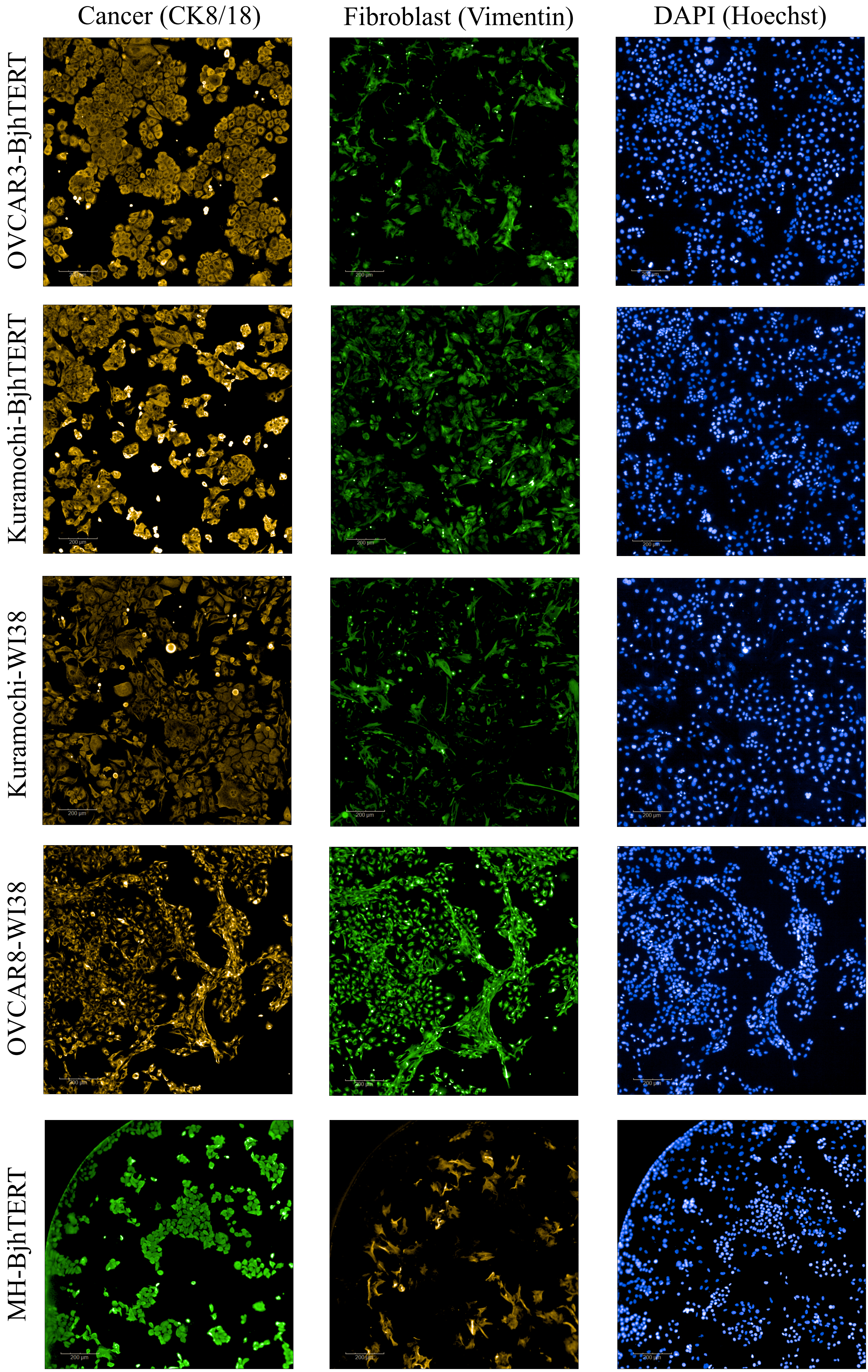

Supplementary Figure 1. Example fluorescent image from one field of view for DMSO treated cells used for standardization from each co-culture combination. The row titles represent co-culture combination names and column titles indicate the channel information and antibody staining.

Supplementary Figure 2

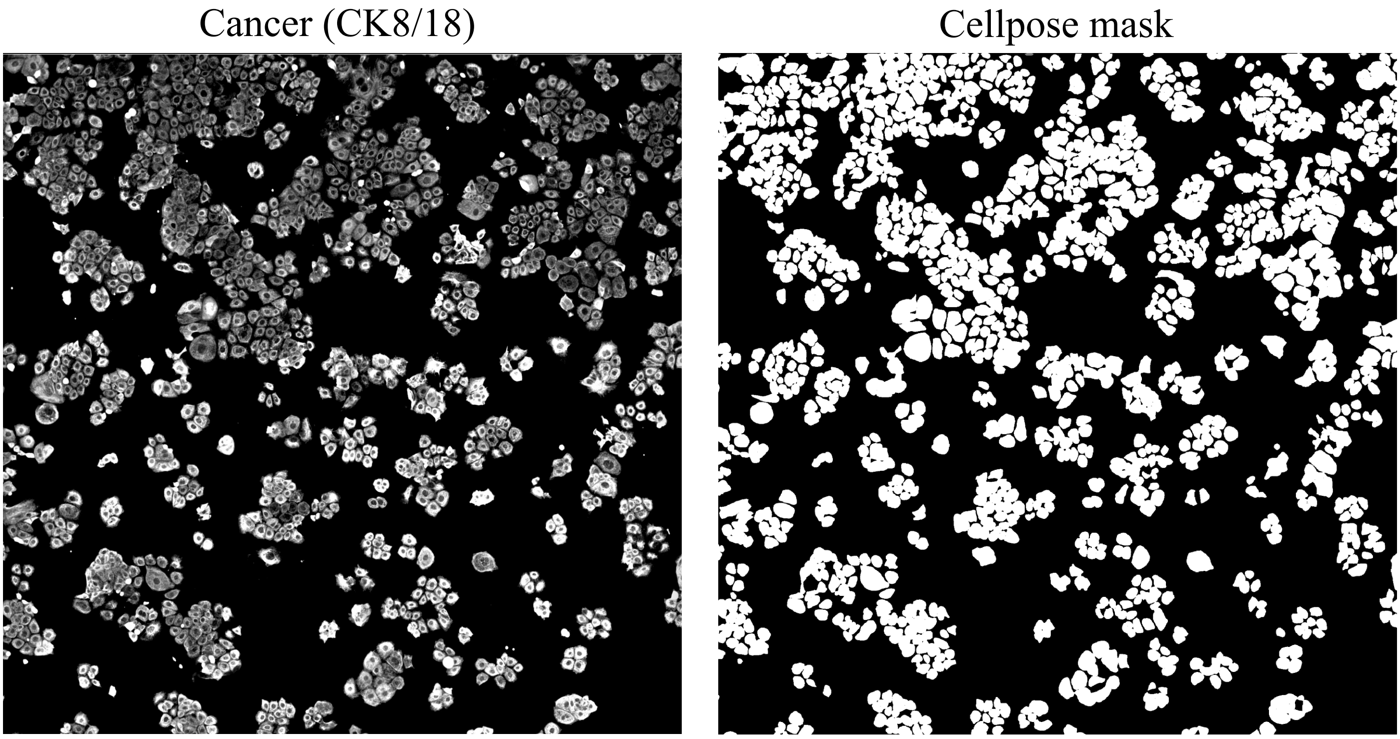

Supplementary Figure 2. Cellpose segmentation mask. Example image from cancer cells stained with CK8/18 antibody (left) and its corresponding Cellpose binary mask (right).

Supplementary Figure 3

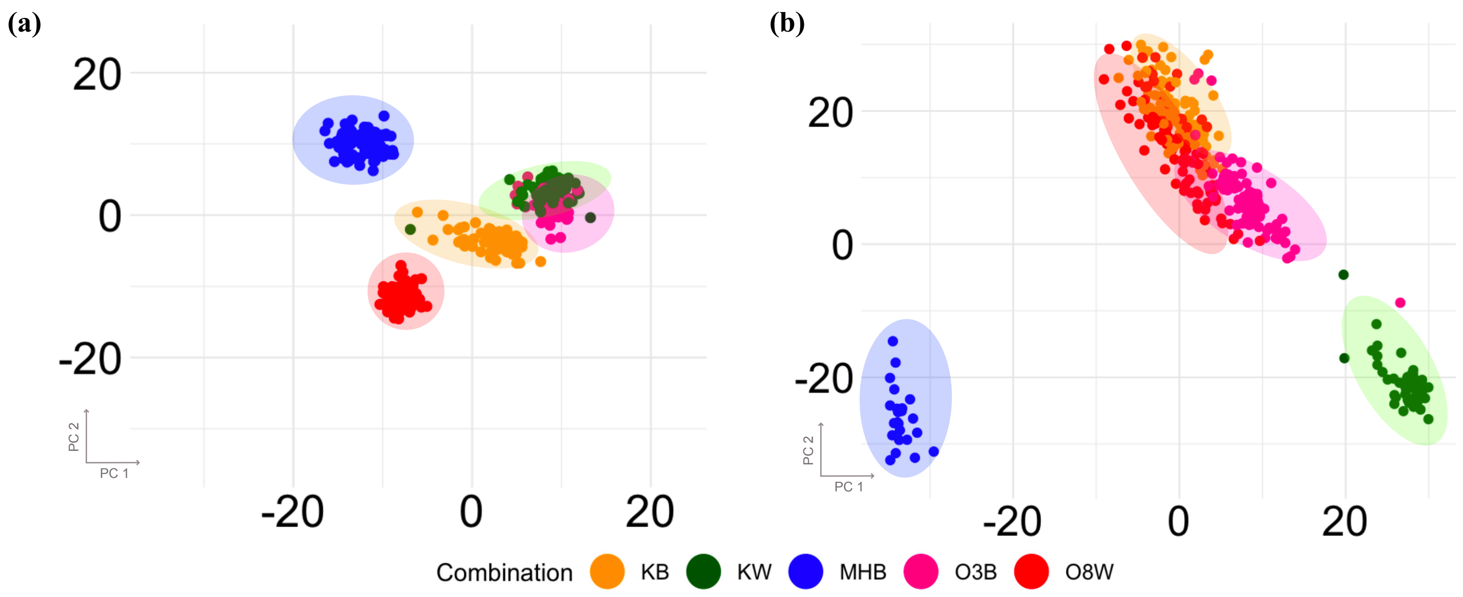

Supplementary Figure 3. Principal Component Analysis (PCA) reveal distinct morphological profiles for different ovarian cancer cell lines and also for same cancer cell line line when cocultured with different fibroblasts. Morphological profiles in DMSO for co-culture combinations (indicated by color shade) **(a)** CP **(b)** Pre-trained baseline EfficientNetB0 model.

Supplementary Figure 4

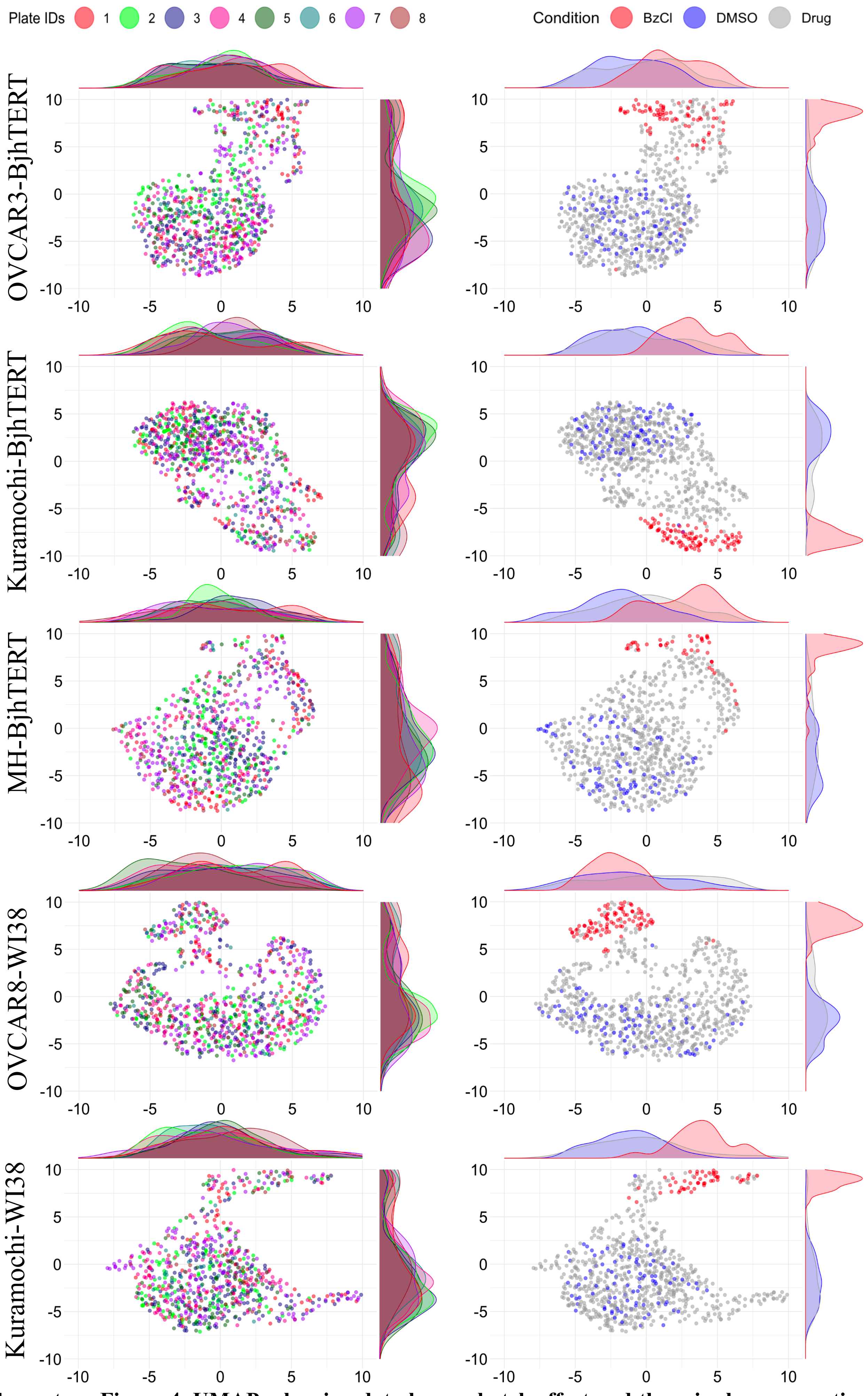

Supplementary Figure 4. UMAPs showing data has no batch effect and there is clear separation between positive and negative controls. UMAP plots of well-level profiles derived from CP for cancer cells across all 5 co-culture combination datasets used in the study (rows), colored by plate IDs (left column) and negative control (DMSO) vs treatment status (incl. BzCl) (right column). The density function on the x and y axes for each color group highlights the spread and clustering pattern of the data.

Supplementary Figure 5

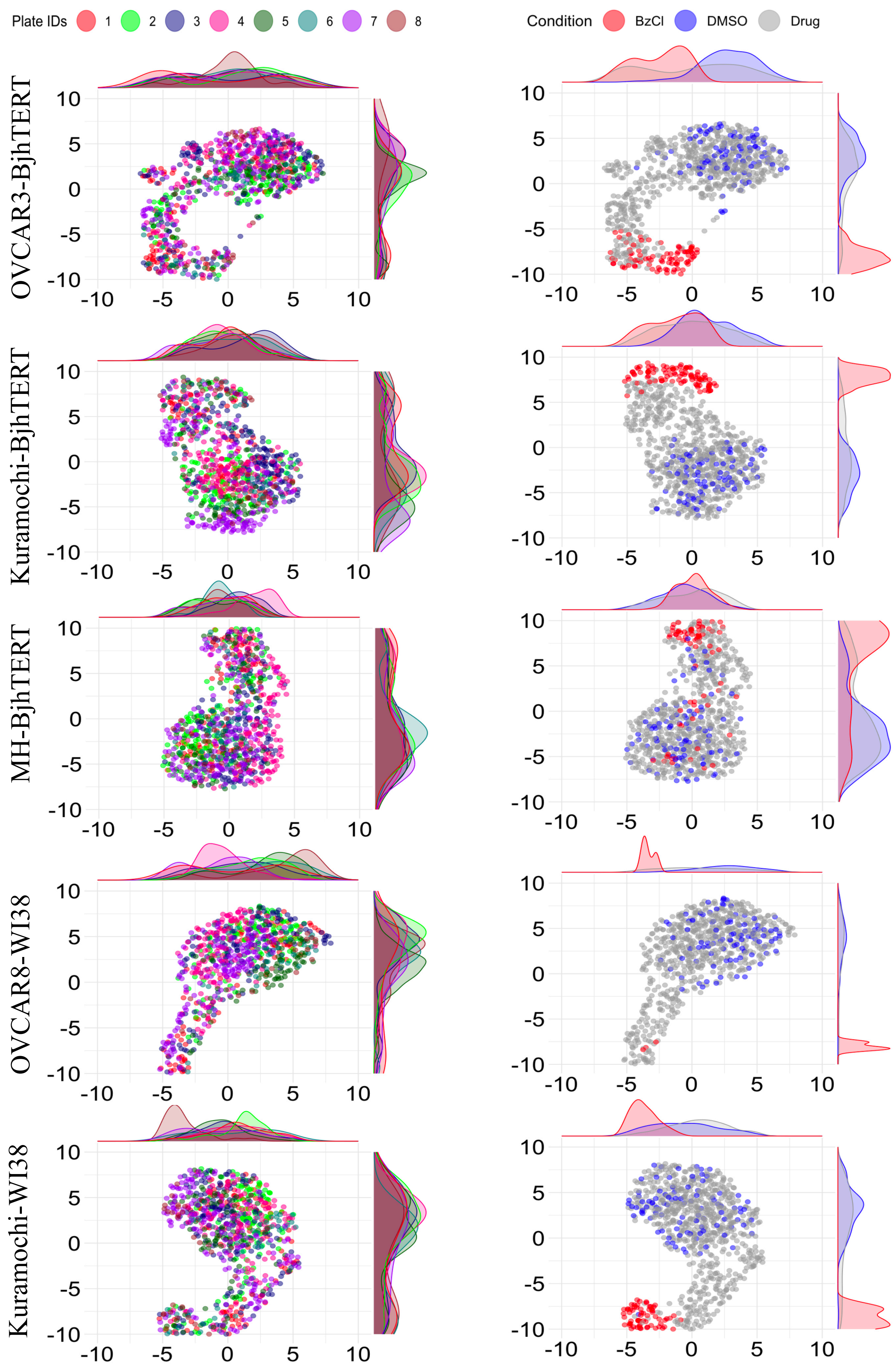

Supplementary Figure 5. UMAPs derived from EfficientNetB0 showing data has no batch effect and there is clear separation between positive and negative controls. UMAP plots of well-level profiles derived from cancer cells across all 5 co-culture combination datasets used in the study (rows), colored by plate IDs (left column) and negative control (DMSO) vs treatment status (incl. BzCl) (right column). The density function on the x and y axes for each color group highlights the spread and clustering pattern of the data.

Supplementary Figure 6

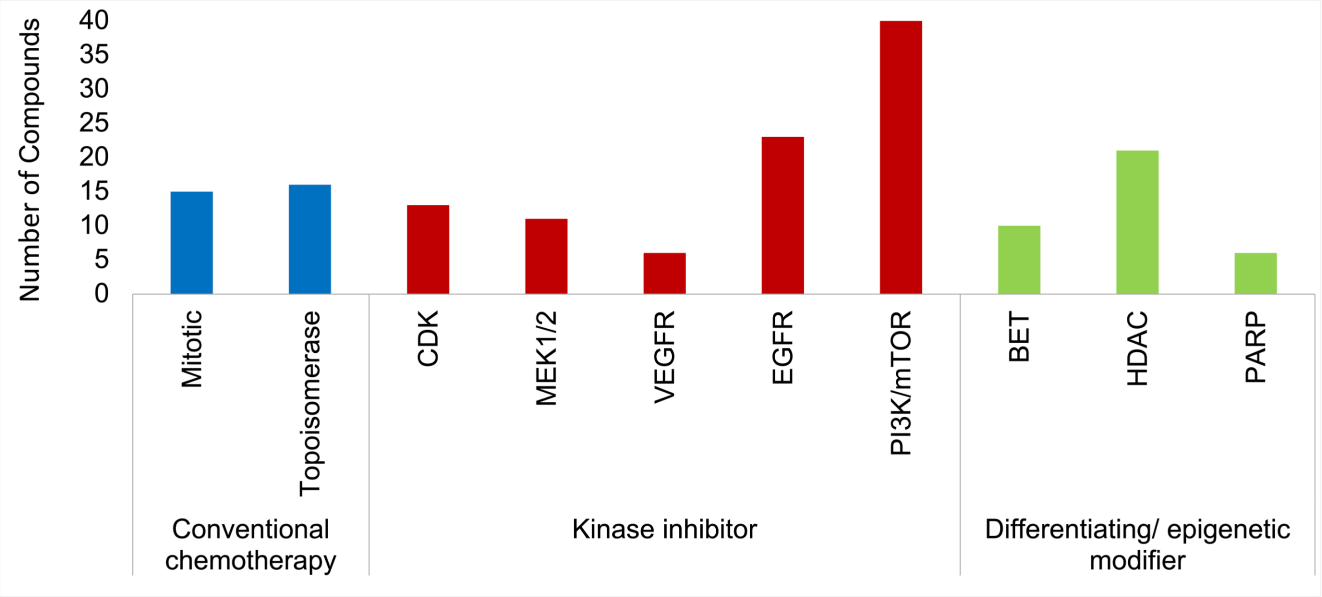

Supplementary Figure 6. Histogram representing compound counts for each MOA.

Supplementary Figure 7

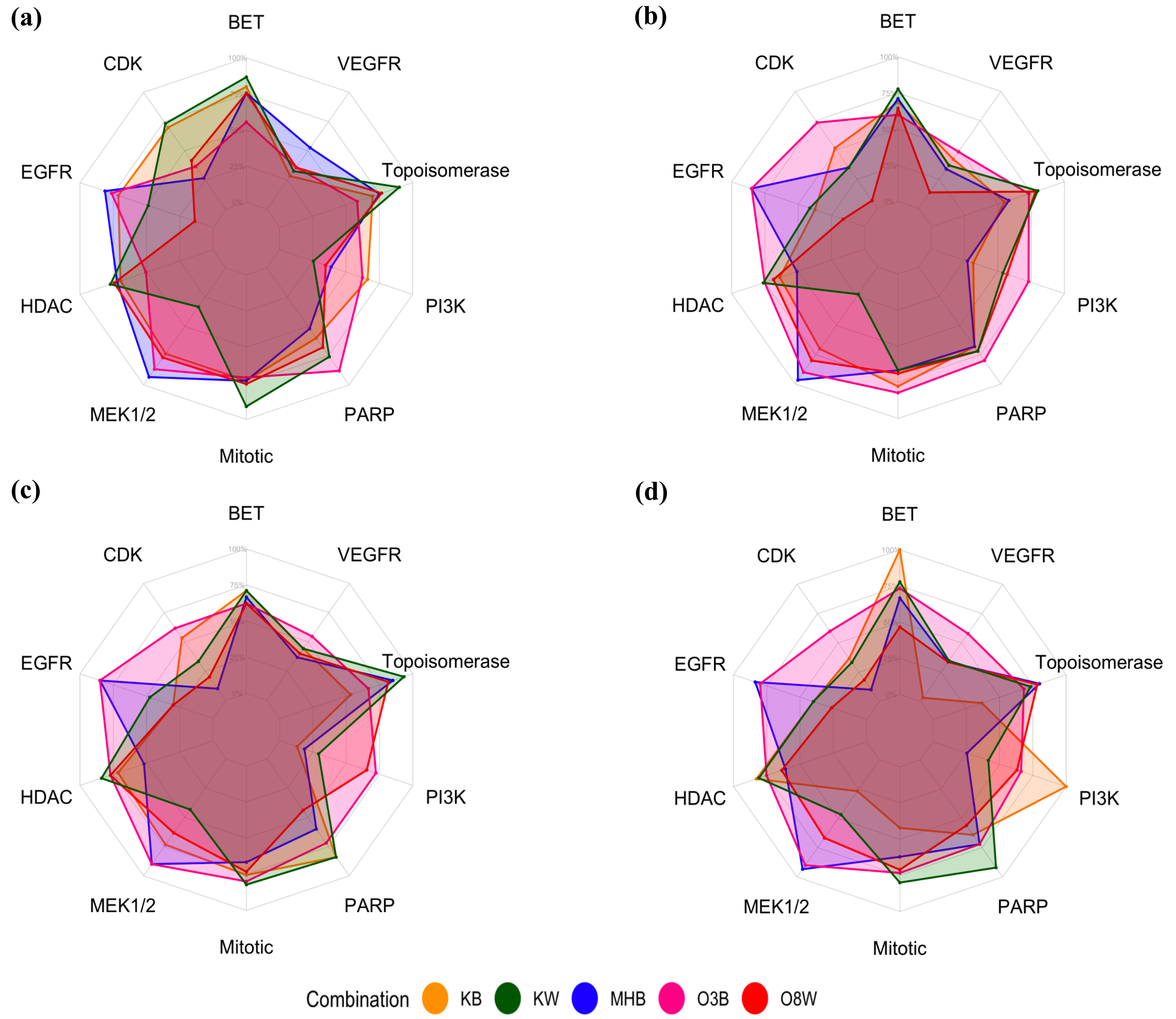

Supplementary Figure 7. Enrichment radar plots showing percentage of enriched features for each MOA. (a) CP features and unmasked bounding box pre-trained baseline **(b)** EfficientNetB0 **(c)** MobileNetV2 **(d)** ResNet50

Supplementary Figure 8

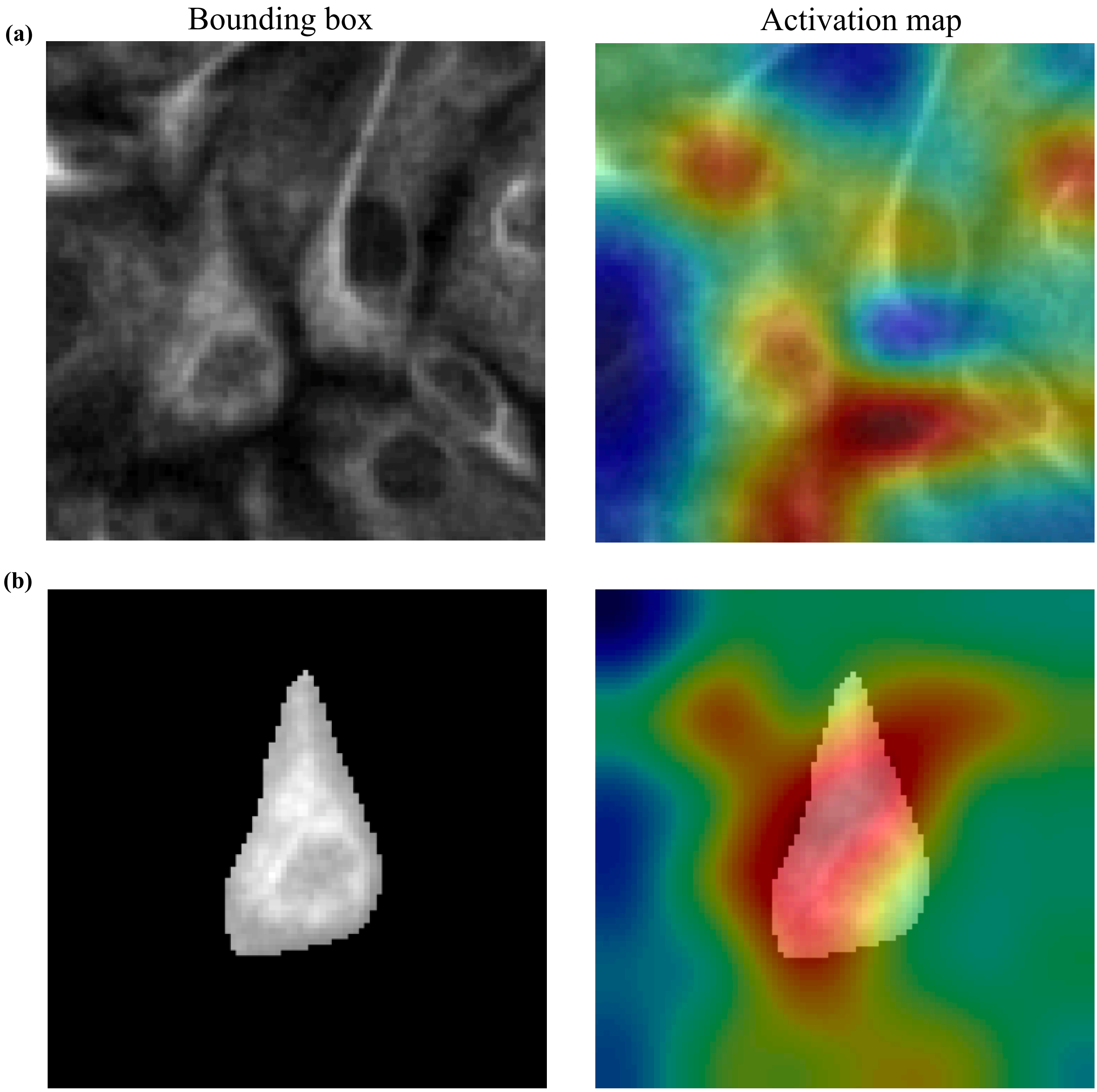

Supplementary Figure 8. Visualization of activation maps for unmasked and masked bounding boxes illustrating the network's focus on image regions to extract relevant features. **(a)** Activation map derived from the unmasked bounding box, **(b)** Activation map derived from the masked bounding box.

Supplementary Figure 9

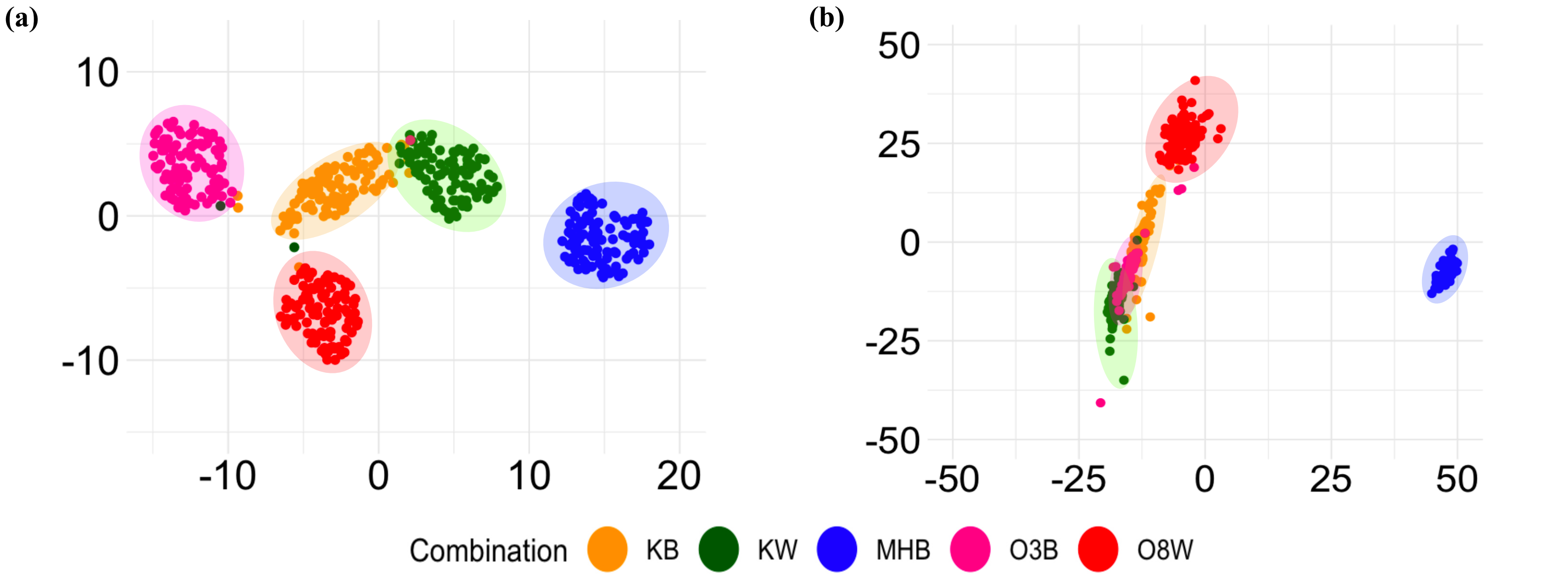

Supplementary Figure 9. Morphological profiles derived from masked bounding box region using pre-trained baseline EfficientNetB0 12 DMSO wells across 40 plates from each assay (a) UMAP **(b)** PCA

Supplementary Figure 10

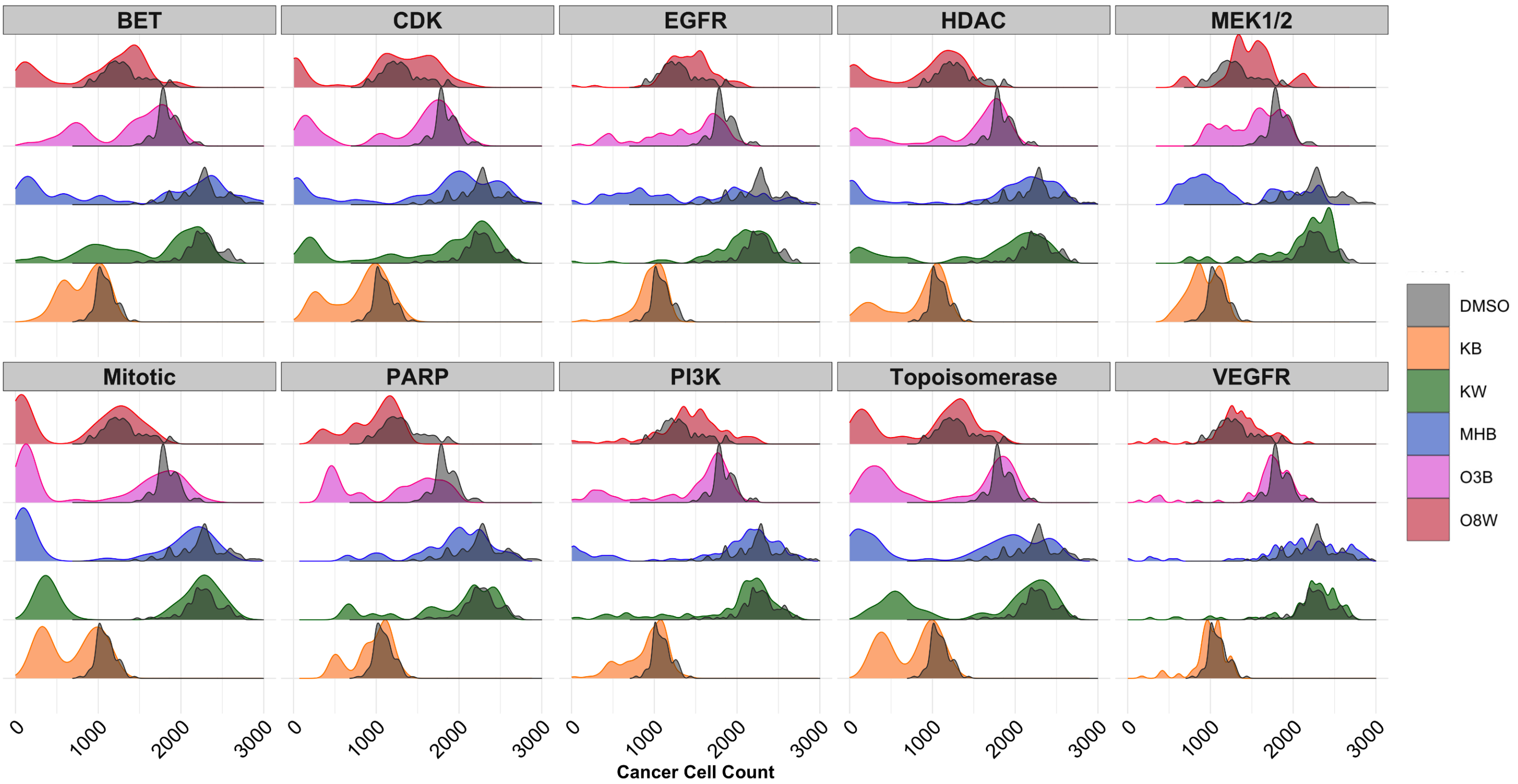

Supplementary Figure 10. Density plots of the cancer cell count across DMSO Controls and drug treatments in five co-culture combinations.

Supplementary Figure 11

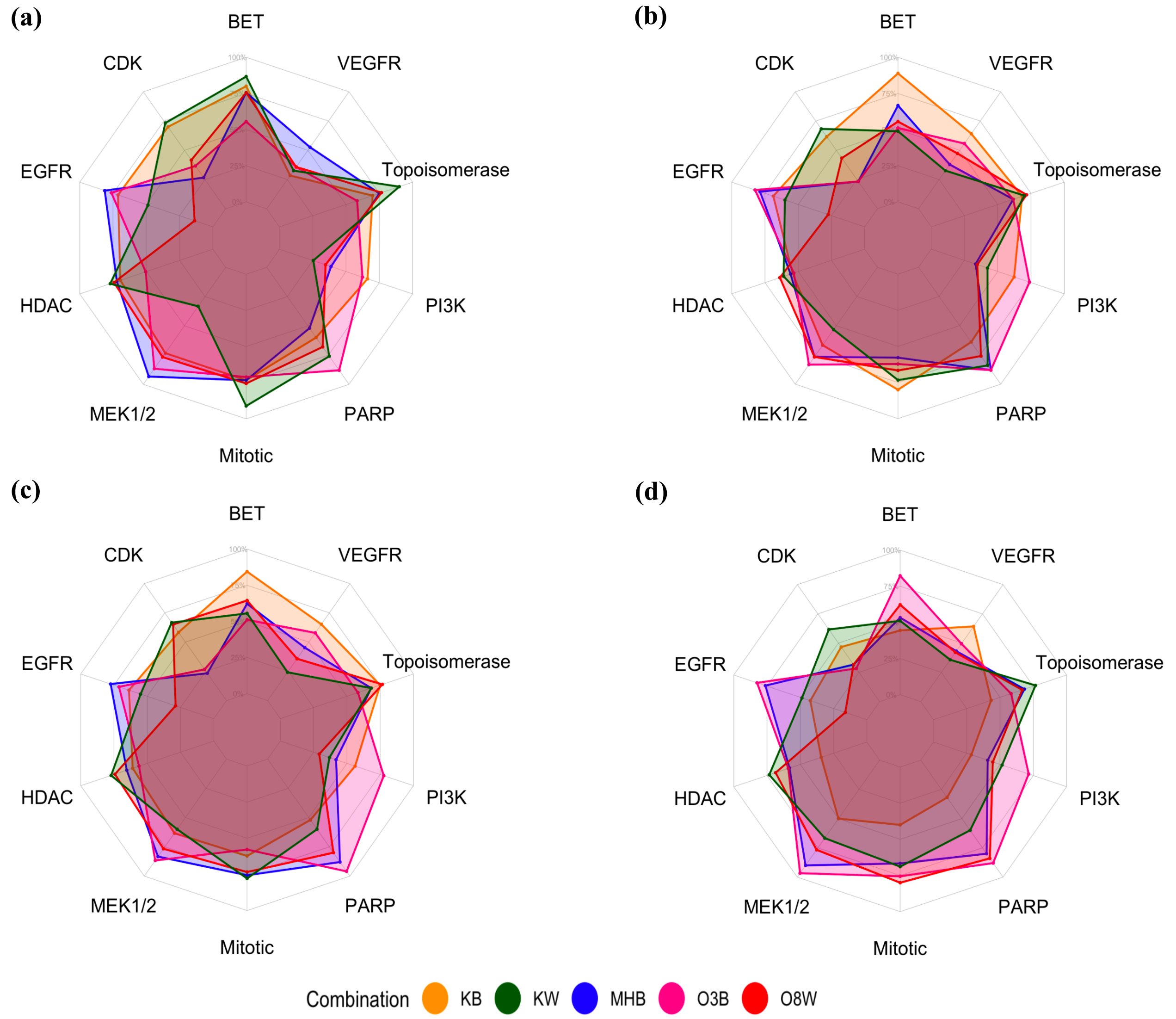

Supplementary Figure 11. Enrichment radar plots showing percentage of enriched features for each MOA. (a) CP features and masked bounding box pre-trained baseline **(b)** EfficientNetB0 **(c)** MobileNetV2 **(d)** ResNet50

Supplementary Figure 12

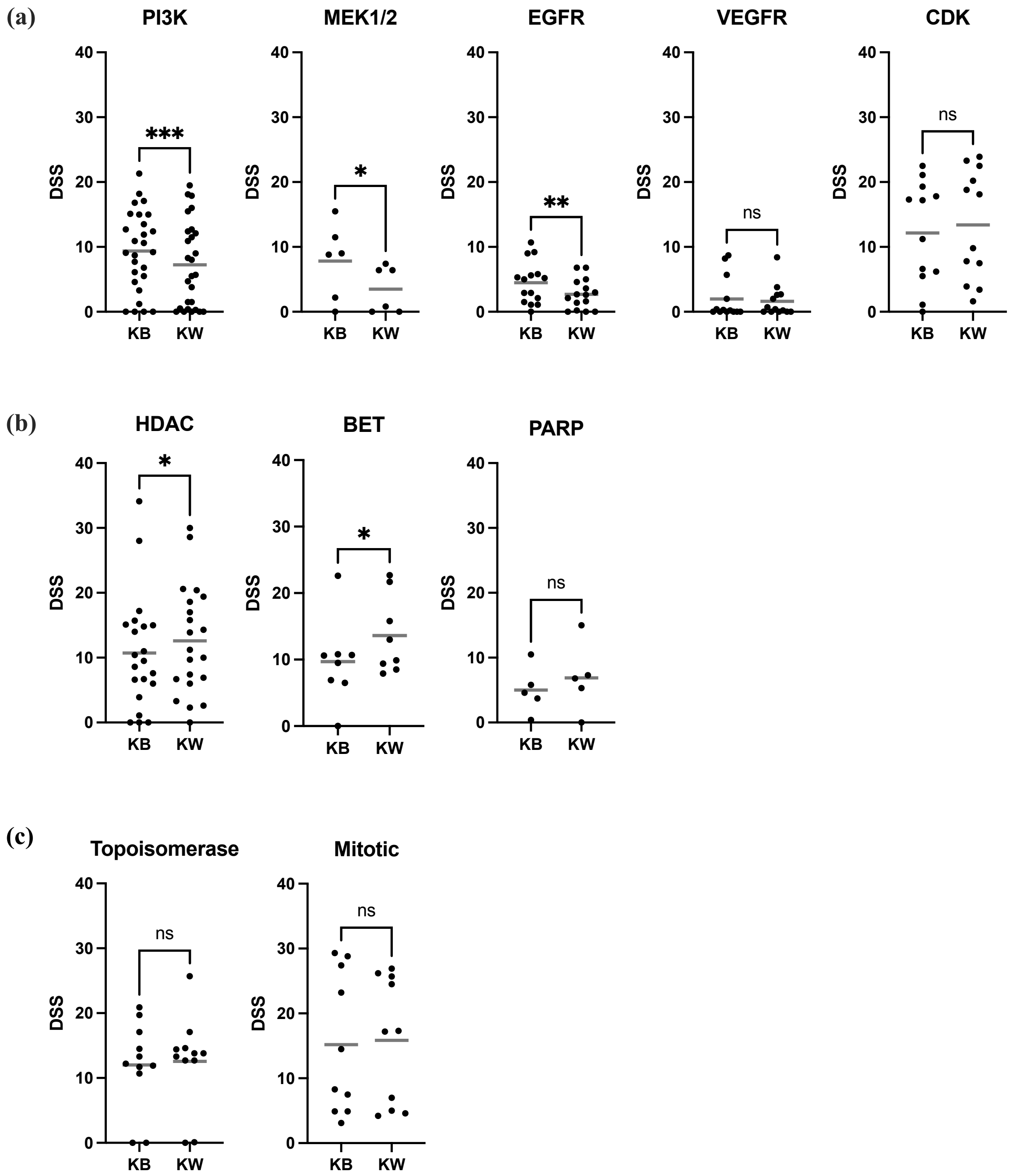

Supplementary Figure 12. Comparison of DSS scores from the KB and KW reveal context-dependent cytotoxicity and impact feature representation. DSS for each compound in KB and KW the **(a)** kinase inhibitors, **(b)** epigenetic modifiers, and **(c)** conventional chemotherapies included in this study. Each point represents a compound in the corresponding MOA class. The grey bar denotes the mean value. * denotes $p < 0.05$, ** $p < 0.01$, *** $p < 0.001$ for paired two-tailed t-test for the DSS of the same inhibitors in the class in KB and KW.

Supplementary Figure 13

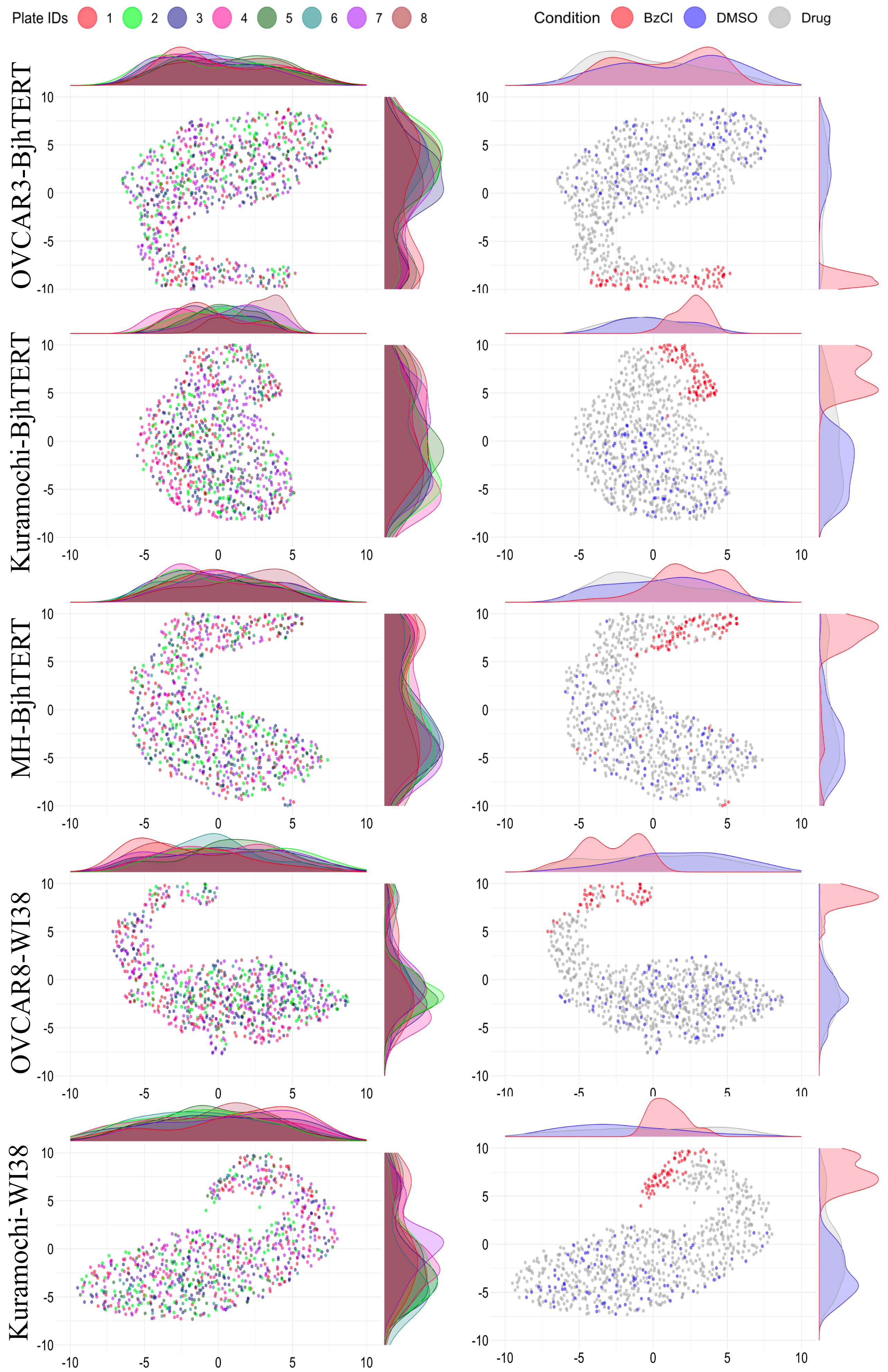

Supplementary Figure 13. UMAPs derived from ResNet50 showing data has no batch effect and there is clear separation between positive and negative controls. UMAP plots of well-level profiles derived from the cancer cells across all five co-culture combination datasets used in the study (rows), colored by plate ids (left column) and negative control (DMSO) vs treatment status (incl. BzCl) (right column). The density function on the x and y axes for each color group highlights the spread and clustering pattern of the data.

Supplementary Figure 14

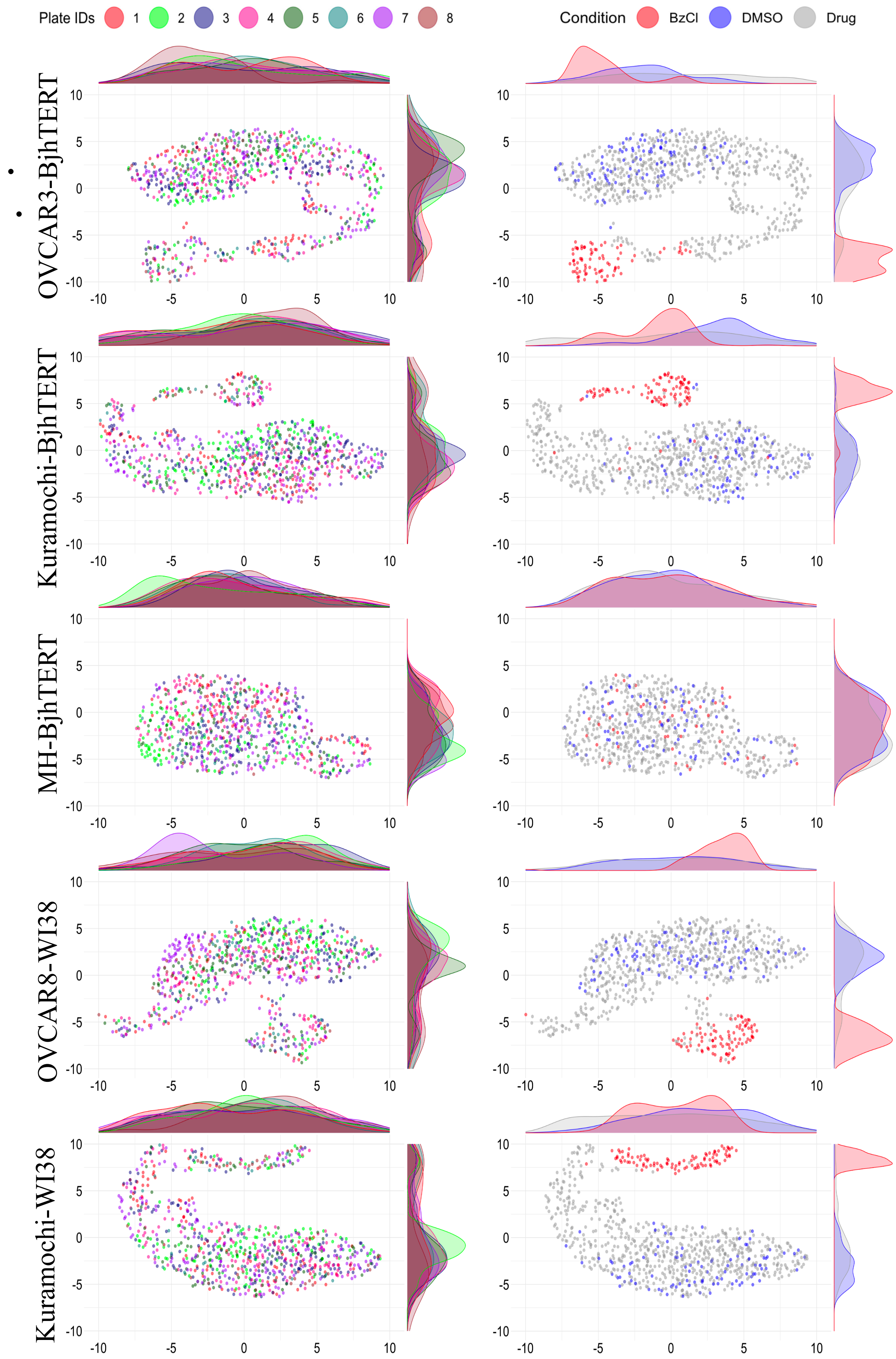

Supplementary Figure 14. UMAP plots of well-level profiles derived from embedding layer of trained (on randomly selected wells for second lowest and second highest dose concentrations across all cell line combinations) ResNet50 model. UMAP from features of cancer cells across all five co-culture combination datasets used in the study (rows), colored by plate ids (left column) and negative control (DMSO) vs treatment status (incl. BzCl) (right column). The density function on the x and y axes for each color group highlights the spread and clustering pattern of the data.

Supplementary Figure 15

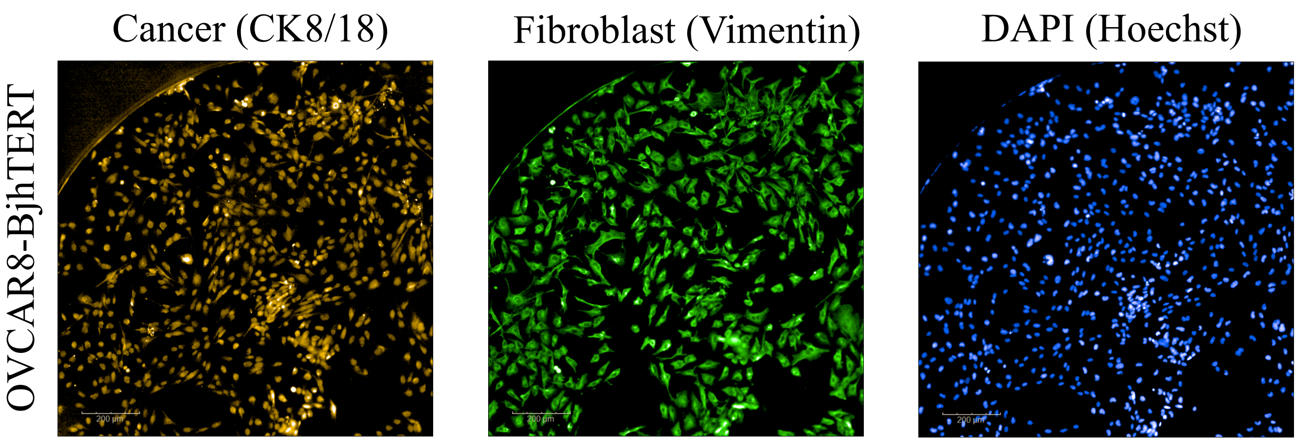

Supplementary Figure 15. Immunofluorescent staining of OVCAR8 ovarian cancer cells (CK8/18) and BJhTERT fibroblasts (Vimentin) in co-culture. OVCAR8 cells demonstrate partial co-expression of Vimentin, consistent with epithelial-mesenchymal transition (EMT). The figure support observations in OVCAR8-WI38 co-culture (Supplementary Figure 1), highlighting the influence of tumor-stromal interactions on marker expression.

Supplementary Tables for

Evaluating Feature Extraction in Ovarian Cancer Cell Line Co-Cultures Using Deep Neural Networks

Osheen Sharma[1*], Greta Gudoityte[1], Rezan Minozada[1], Olli P. Kallioniemi[1,2], Riku Turkki[2], Lassi Paavolainen[2,3], Brinton Seashore-Ludlow[1**]

[1]Department of Oncology-Pathology, Karolinska Institutet, Science for Life Laboratory, Stockholm, Sweden
[2]Institute for Molecular Medicine Finland (FIMM), HiLIFE, University of Helsinki, Helsinki, Finland
[3]iCAN Digital Precision Cancer Medicine Flagship, University of Helsinki, Helsinki, Finland

[*]Correspondence: osheen.sharma@ki.se
[**]Correspondence: brinton.seashore-ludlow@ki.se

Table number	Description
Supplementary Table 1	FIMM Oncology Drug annotation with plate layout
Supplementary Table 2	Z' score for co-culture combinations across different plates
Supplementary Table 3	10 selected mode of actions from 3 different drug classes
Supplementary Table 4	Running Enrichment Score for 10 Selected Modes of Actions from pretrained baseline MobileNetV2 and ResNet50 model
Supplementary Table 5	Running Enrichment Score calculated excluding query compound and its concentrations for 10 Selected Modes of Actions from pretrained baseline models
Supplementary Table 6	Percentage of lowest and highest concentrations enriched from CellProfiler vs EfficientNetB0
Supplementary Table 7	Running Enrichment Score for MHB coculture after finetuning EfficientNetB0 model
Supplementary Table 8	Running Enrichment Score for all cocultures after finetuning ResNet50 model

Supplementary Table 1

Well_number	Compound	MoA	Concentration	Label	Category	Plate
A1	cells	cells	None	cells	Negative Controls	1
A2	BzCl	BzCl	0	BzCl	Miscl.	1
A3	Vorinostat	HDAC inhibitor	10000	Drug	HDAC	1
A4	Bicalutamide	Nonsteriodal antiandrogen	10000	Drug	Miscl.	1
A5	Prednisolone	Glucocorticoid, immunomodulatory agent	10000	Drug	Miscl.	1
A6	Everolimus	binds FKBP12, causes inhibition of mTORC1	100	Drug	Miscl.	1
A7	Carfilzomib	Proteasome inhibitor (20S subunit)	1000	Drug	Miscl.	1
A8	Bortezomib	Proteasome inhibitor (26S subunit)	1000	Drug	Miscl.	1
A9	Auranofin	Antirheumatic agent	2500	Drug	Miscl.	1
A10	Vinorelbine	Mitotic inhibitor. Vinca alkaloid microtubule depolymerizer	10000	Drug	Mitotic	1
A11	Ixazomib	20S proteasome inhibitor	1000	Drug	Miscl.	1
A12	Raltitrexed	DHFR/GARFT/thymidylate synthase inhibitor	1000	Drug	Miscl.	1
A13	Ixabepilone	Mitotic inhibitor. Epothilone microtubule stabilizer.	1000	Drug	Mitotic	1
A14	Raloxifene	Selective estrogen receptor modulator	10000	Drug	Miscl.	1
A15	Pentostatin	Antimetabolite; Purine analog	10000	Drug	Miscl.	1
A16	Mercaptopurine	Antimetabolite	10000	Drug	Miscl.	1
A17	Mitotane	Antineoplastic agent	10000	Drug	Miscl.	1
A18	Paclitaxel	Mitotic inhibitor, taxane microtubule stabilizer	1000	Drug	Mitotic	1
A19	Thalidomide	Immunosuppresant	10000	Drug	Miscl.	1
A20	Allopurinol	Xanthine oxidase inhibitor	10000	Drug	Miscl.	1
A21	Trifluridine	Antimetabolite; Nucleoside analog	10000	Drug	Miscl.	1
A22	Hydroxyurea	Antineoplastic agent	1000000	Drug	Miscl.	1
A23	BzCl	BzCl	0	BzCl	Miscl.	1
A24	BzCl	BzCl	0	BzCl	Miscl.	1
B1	cells	cells	None	cells	Negative Controls	1
B2	Nelarabine	Nucleoside analog, DNA, RNA synth inhibitor	10000	Drug	Miscl.	1
B3	Vorinostat	HDAC inhibitor	1000	Drug	HDAC	1
B4	Bicalutamide	Nonsteriodal antiandrogen	1000	Drug	Miscl.	1
B5	Prednisolone	Glucocorticoid, immunomodulatory agent	1000	Drug	Miscl.	1
B6	Everolimus	binds FKBP12, causes inhibition of mTORC1	10	Drug	Miscl.	1
B7	Carfilzomib	Proteasome inhibitor (20S subunit)	100	Drug	Miscl.	1

B8	Bortezomib	Proteasome inhibitor (26S subunit)	100	Drug	Miscl.	1
B9	DMSO	DMSO	0	DMSO	Negative Controls	1
B10	Vinorelbine	Mitotic inhibitor. Vinca alkaloid microtubule depolymerizer	1000	Drug	Mitotic	1
B11	Ixazomib	20S proteasome inhibitor	100	Drug	Miscl.	1
B12	Raltitrexed	DHFR/GARFT/thymidylate synthase inhibitor	100	Drug	Miscl.	1
B13	Ixabepilone	Mitotic inhibitor. Epothilone microtubule stabilizer.	100	Drug	Mitotic	1
B14	Raloxifene	Selective estrogen receptor modulator	1000	Drug	Miscl.	1
B15	Pentostatin	Antimetabolite; Purine analog	1000	Drug	Miscl.	1
B16	BzCl	BzCl	0	BzCl	Miscl.	1
B17	Mitotane	Antineoplastic agent	1000	Drug	Miscl.	1
B18	Paclitaxel	Mitotic inhibitor, taxane microtubule stabilizer	100	Drug	Mitotic	1
B19	Thalidomide	Immunosuppresant	1000	Drug	Miscl.	1
B20	Allopurinol	Xanthine oxidase inhibitor	1000	Drug	Miscl.	1
B21	Trifluridine	Antimetabolite; Nucleoside analog	1000	Drug	Miscl.	1
B22	Hydroxyurea	Antineoplastic agent	100000	Drug	Miscl.	1
B23	Clofarabine	Antimetabolite; Purine analog	10000	Drug	Miscl.	1
B24	cells	cells	None	cells	Negative Controls	1
C1	cells	cells	None	cells	Negative Controls	1
C2	Nelarabine	Nucleoside analog, DNA, RNA synth inhibitor	1000	Drug	Miscl.	1
C3	Vorinostat	HDAC inhibitor	100	Drug	HDAC	1
C4	Bicalutamide	Nonsteriodal antiandrogen	100	Drug	Miscl.	1
C5	Prednisolone	Glucocorticoid, immunomodulatory agent	100	Drug	Miscl.	1
C6	Everolimus	binds FKBP12, causes inhibition of mTORC1	1	Drug	Miscl.	1
C7	Carfilzomib	Proteasome inhibitor (20S subunit)	10	Drug	Miscl.	1
C8	Bortezomib	Proteasome inhibitor (26S subunit)	10	Drug	Miscl.	1
C9	Auranofin	Antirheumatic agent	250	Drug	Miscl.	1
C10	Vinorelbine	Mitotic inhibitor. Vinca alkaloid microtubule depolymerizer	100	Drug	Mitotic	1
C11	Ixazomib	20S proteasome inhibitor	10	Drug	Miscl.	1
C12	DMSO	DMSO	0	DMSO	Negative Controls	1
C13	Ixabepilone	Mitotic inhibitor. Epothilone microtubule stabilizer.	10	Drug	Mitotic	1
C14	Raloxifene	Selective estrogen receptor modulator	100	Drug	Miscl.	1
C15	Pentostatin	Antimetabolite; Purine analog	100	Drug	Miscl.	1
C16	Mercaptopurine	Antimetabolite	1000	Drug	Miscl.	1

C17	Mitotane	Antineoplastic agent	100	Drug	Miscl.	1
C18	Paclitaxel	Mitotic inhibitor, taxane microtubule stabilizer	10	Drug	Mitotic	1
C19	Thalidomide	Immunosuppresant	100	Drug	Miscl.	1
C20	DMSO	DMSO	0	DMSO	Negative Controls	1
C21	Trifluridine	Antimetabolite; Nucleoside analog	100	Drug	Miscl.	1
C22	Hydroxyurea	Antineoplastic agent	10000	Drug	Miscl.	1
C23	Clofarabine	Antimetabolite; Purine analog	1000	Drug	Miscl.	1
C24	cells	cells	None	cells	Negative Controls	1
D1	cells	cells	None	cells	Negative Controls	1
D2	Nelarabine	Nucleoside analog, DNA, RNA synth inhibitor	100	Drug	Miscl.	1
D3	Vorinostat	HDAC inhibitor	10	Drug	HDAC	1
D4	Bicalutamide	Nonsteriodal antiandrogen	10	Drug	Miscl.	1
D5	DMSO	DMSO	0	DMSO	Negative Controls	1
D6	Everolimus	binds FKBP12, causes inhibition of mTORC1	0.1	Drug	Miscl.	1
D7	Carfilzomib	Proteasome inhibitor (20S subunit)	1	Drug	Miscl.	1
D8	Bortezomib	Proteasome inhibitor (26S subunit)	1	Drug	Miscl.	1
D9	Auranofin	Antirheumatic agent	25	Drug	Miscl.	1
D10	Vinorelbine	Mitotic inhibitor. Vinca alkaloid microtubule depolymerizer	10	Drug	Mitotic	1
D11	Ixazomib	20S proteasome inhibitor	1	Drug	Miscl.	1
D12	Raltitrexed	DHFR/GARFT/thymidylate synthase inhibitor	10	Drug	Miscl.	1
D13	Ixabepilone	Mitotic inhibitor. Epothilone microtubule stabilizer.	1	Drug	Mitotic	1
D14	Raloxifene	Selective estrogen receptor modulator	10	Drug	Miscl.	1
D15	Pentostatin	Antimetabolite; Purine analog	10	Drug	Miscl.	1
D16	Mercaptopurine	Antimetabolite	100	Drug	Miscl.	1
D17	Mitotane	Antineoplastic agent	10	Drug	Miscl.	1
D18	Paclitaxel	Mitotic inhibitor, taxane microtubule stabilizer	1	Drug	Mitotic	1
D19	Thalidomide	Immunosuppresant	10	Drug	Miscl.	1
D20	Allopurinol	Xanthine oxidase inhibitor	100	Drug	Miscl.	1
D21	Trifluridine	Antimetabolite; Nucleoside analog	10	Drug	Miscl.	1
D22	Hydroxyurea	Antineoplastic agent	1000	Drug	Miscl.	1
D23	Clofarabine	Antimetabolite; Purine analog	100	Drug	Miscl.	1
D24	cells	cells	None	cells	Negative Controls	1
E1	cells	cells	None	cells	Negative Controls	1

E2	Nelarabine	Nucleoside analog, DNA, RNA synth inhibitor	10	Drug	Miscl.	1
E3	Vorinostat	HDAC inhibitor	1	Drug	HDAC	1
E4	Bicalutamide	Nonsteriodal antiandrogen	1	Drug	Miscl.	1
E5	Prednisolone	Glucocorticoid, immunomodulatory agent	10	Drug	Miscl.	1
E6	Everolimus	binds FKBP12, causes inhibition of mTORC1	0.01	Drug	Miscl.	1
E7	Carfilzomib	Proteasome inhibitor (20S subunit)	0.1	Drug	Miscl.	1
E8	Bortezomib	Proteasome inhibitor (26S subunit)	0.1	Drug	Miscl.	1
E9	Auranofin	Antirheumatic agent	2.5	Drug	Miscl.	1
E10	Vinorelbine	Mitotic inhibitor. Vinca alkaloid microtubule depolymerizer	1	Drug	Mitotic	1
E11	Ixazomib	20S proteasome inhibitor	0.1	Drug	Miscl.	1
E12	Raltitrexed	DHFR/GARFT/thymidylate synthase inhibitor	1	Drug	Miscl.	1
E13	Ixabepilone	Mitotic inhibitor. Epothilone microtubule stabilizer.	0.1	Drug	Mitotic	1
E14	Raloxifene	Selective estrogen receptor modulator	1	Drug	Miscl.	1
E15	DMSO	DMSO	0	DMSO	Negative Controls	1
E16	Mercaptopurine	Antimetabolite	10	Drug	Miscl.	1
E17	Mitotane	Antineoplastic agent	1	Drug	Miscl.	1
E18	Paclitaxel	Mitotic inhibitor, taxane microtubule stabilizer	0.1	Drug	Mitotic	1
E19	Thalidomide	Immunosuppresant	1	Drug	Miscl.	1
E20	Allopurinol	Xanthine oxidase inhibitor	10	Drug	Miscl.	1
E21	Trifluridine	Antimetabolite; Nucleoside analog	1	Drug	Miscl.	1
E22	Hydroxyurea	Antineoplastic agent	100	Drug	Miscl.	1
E23	Clofarabine	Antimetabolite; Purine analog	10	Drug	Miscl.	1
E24	cells	cells	None	cells	Negative Controls	1
F1	cells	cells	None	cells	Negative Controls	1
F2	Nelarabine	Nucleoside analog, DNA, RNA synth inhibitor	1	Drug	Miscl.	1
F3	Aminoglutethimide	Anti-steroid, aromatase inhibitor	10000	Drug	Miscl.	1
F4	Capecitabine	5-FU prodrug	10000	Drug	Miscl.	1
F5	Prednisolone	Glucocorticoid, immunomodulatory agent	1	Drug	Miscl.	1
F6	Sirolimus	binds FKBP12, causes inhibition of mTORC1	100	Drug	Miscl.	1
F7	Pomalidomide	Immunomodulatory agent, anti-angiogenic	10000	Drug	Miscl.	1
F8	Bimatoprost	Prostaglandin analog	5500	Drug	Miscl.	1
F9	Auranofin	Antirheumatic agent	0.25	Drug	Miscl.	1
F10	BzCl	BzCl	0	BzCl	Miscl.	1

F11	Amsacrine	DNA intercalation, Topo II inhibitor	10000	Drug	Topoisomerase	1
F12	Raltitrexed	DHFR/GARFT/thymidylate synthase inhibitor	0.1	Drug	Miscl.	1
F13	Vinblastine	Mitotic inhibitor. Vinca alkaloid microtubule depolymerizer	1000	Drug	Mitotic	1
F14	Anastrozole	Aromatase inhibitor	10000	Drug	Miscl.	1
F15	Pentostatin	Antimetabolite; Purine analog	1	Drug	Miscl.	1
F16	Mercaptopurine	Antimetabolite	1	Drug	Miscl.	1
F17	Cladribine	Antimetabolite; Purine analog	1000	Drug	Miscl.	1
F18	Floxuridine	Antimetabolite; Analog of 5-fluorouracil	10000	Drug	Miscl.	1
F19	Thioguanine	Antimetabolite; Purine analog	10000	Drug	Miscl.	1
F20	Allopurinol	Xanthine oxidase inhibitor	1	Drug	Miscl.	1
F21	Pirfenidone	Antifibrotic and anti-inflammatory	10000	Drug	Miscl.	1
F22	Goserelin	Gonadotropin releasing hormone superagonist	10000	Drug	Miscl.	1
F23	Clofarabine	Antimetabolite; Purine analog	1	Drug	Miscl.	1
F24	cells	cells	None	cells	Negative Controls	1
G1	cells	cells	None	cells	Negative Controls	1
G2	Decitabine	Nucleoside analog DNA methyl transferase inhibitor	10000	Drug	Miscl.	1
G3	Aminoglutethimide	Anti-steroid, aromatase inhibitor	1000	Drug	Miscl.	1
G4	Capecitabine	5-FU prodrug	1000	Drug	Miscl.	1
G5	Dexamethasone	Glucocorticoid, immunomodulatory agent	10000	Drug	Miscl.	1
G6	BzCl	BzCl	0	BzCl	Miscl.	1
G7	Pomalidomide	Immunomodulatory agent, anti-angiogenic	1000	Drug	Miscl.	1
G8	Bimatoprost	Prostaglandin analog	550	Drug	Miscl.	1
G9	Plicamycin	RNA synthesis inhibitor	10000	Drug	Miscl.	1
G10	Temsirolimus	binds FKBP12, causes inhibition of mTORC1	100	Drug	Miscl.	1
G11	Amsacrine	DNA intercalation, Topo II inhibitor	1000	Drug	Topoisomerase	1
G12	Cabazitaxel	Taxane microtubule stabilizer, antimitotic	1000	Drug	Miscl.	1
G13	Vinblastine	Mitotic inhibitor. Vinca alkaloid microtubule depolymerizer	100	Drug	Mitotic	1
G14	Anastrozole	Aromatase inhibitor	1000	Drug	Miscl.	1
G15	Eribulin	Mitotic inhibitor, microtubule depolymerizer.	1000	Drug	Mitotic	1
G16	Letrozole	Aromatase inhibitor	10000	Drug	Miscl.	1
G17	Cladribine	Antimetabolite; Purine analog	100	Drug	Miscl.	1
G18	Floxuridine	Antimetabolite; Analog of 5-fluorouracil	1000	Drug	Miscl.	1
G19	Thioguanine	Antimetabolite; Purine analog	1000	Drug	Miscl.	1

G20	Epirubicin	Topoisomerase II inhibitor	1000	Drug	Topoisomerase	1
G21	Pirfenidone	Antifibrotic and anti-inflammatory	1000	Drug	Miscl.	1
G22	Goserelin	Gonadotropin releasing hormone superagonist	1000	Drug	Miscl.	1
G23	Fluorouracil	Antimetabolite	10000	Drug	Miscl.	1
G24	cells	cells	None	cells	Negative Controls	1
H1	cells	cells	None	cells	Negative Controls	1
H2	Decitabine	Nucleoside analog DNA methyl transferase inhibitor	1000	Drug	Miscl.	1
H3	Aminoglutethimide	Anti-steroid, aromatase inhibitor	100	Drug	Miscl.	1
H4	Capecitabine	5-FU prodrug	100	Drug	Miscl.	1
H5	Dexamethasone	Glucocorticoid, immunomodulatory agent	1000	Drug	Miscl.	1
H6	Sirolimus	binds FKBP12, causes inhibition of mTORC1	10	Drug	Miscl.	1
H7	Pomalidomide	Immunomodulatory agent, anti-angiogenic	100	Drug	Miscl.	1
H8	Bimatoprost	Prostaglandin analog	55	Drug	Miscl.	1
H9	Plicamycin	RNA synthesis inhibitor	1000	Drug	Miscl.	1
H10	Temsirolimus	binds FKBP12, causes inhibition of mTORC1	10	Drug	Miscl.	1
H11	Amsacrine	DNA intercalation, Topo II inhibitor	100	Drug	Topoisomerase	1
H12	Cabazitaxel	Taxane microtubule stabilizer, antimitotic	100	Drug	Miscl.	1
H13	Vinblastine	Mitotic inhibitor. Vinca alkaloid microtubule depolymerizer	10	Drug	Mitotic	1
H14	Anastrozole	Aromatase inhibitor	100	Drug	Miscl.	1
H15	Eribulin	Mitotic inhibitor, microtubule depolymerizer.	100	Drug	Mitotic	1
H16	Letrozole	Aromatase inhibitor	1000	Drug	Miscl.	1
H17	Cladribine	Antimetabolite; Purine analog	10	Drug	Miscl.	1
H18	Floxuridine	Antimetabolite; Analog of 5-fluorouracil	100	Drug	Miscl.	1
H19	DMSO	DMSO	0	DMSO	Negative Controls	1
H20	Epirubicin	Topoisomerase II inhibitor	100	Drug	Topoisomerase	1
H21	Pirfenidone	Antifibrotic and anti-inflammatory	100	Drug	Miscl.	1
H22	Goserelin	Gonadotropin releasing hormone superagonist	100	Drug	Miscl.	1
H23	Fluorouracil	Antimetabolite	1000	Drug	Miscl.	1
H24	cells	cells	None	cells	Negative Controls	1
I1	cells	cells	None	cells	Negative Controls	1
I2	Decitabine	Nucleoside analog DNA methyl transferase inhibitor	100	Drug	Miscl.	1
I3	Aminoglutethimide	Anti-steroid, aromatase inhibitor	10	Drug	Miscl.	1
I4	Capecitabine	5-FU prodrug	10	Drug	Miscl.	1

I5	Dexamethasone	Glucocorticoid, immunomodulatory agent	100	Drug	Miscl.	1
I6	Sirolimus	binds FKBP12, causes inhibition of mTORC1	1	Drug	Miscl.	1
I7	Pomalidomide	Immunomodulatory agent, anti-angiogenic	10	Drug	Miscl.	1
I8	DMSO	DMSO	0	DMSO	Negative Controls	1
I9	Plicamycin	RNA synthesis inhibitor	100	Drug	Miscl.	1
I10	Temsirolimus	binds FKBP12, causes inhibition of mTORC1	1	Drug	Miscl.	1
I11	Amsacrine	DNA intercalation, Topo II inhibitor	10	Drug	Topoisomerase	1
I12	Cabazitaxel	Taxane microtubule stabilizer, antimitotic	10	Drug	Miscl.	1
I13	Vinblastine	Mitotic inhibitor. Vinca alkaloid microtubule depolymerizer	1	Drug	Mitotic	1
I14	Anastrozole	Aromatase inhibitor	10	Drug	Miscl.	1
I15	Eribulin	Mitotic inhibitor, microtubule depolymerizer.	10	Drug	Mitotic	1
I16	Letrozole	Aromatase inhibitor	100	Drug	Miscl.	1
I17	Cladribine	Antimetabolite; Purine analog	1	Drug	Miscl.	1
I18	Floxuridine	Antimetabolite; Analog of 5-fluorouracil	10	Drug	Miscl.	1
I19	Thioguanine	Antimetabolite; Purine analog	100	Drug	Miscl.	1
I20	Epirubicin	Topoisomerase II inhibitor	10	Drug	Topoisomerase	1
I21	Pirfenidone	Antifibrotic and anti-inflammatory	10	Drug	Miscl.	1
I22	Goserelin	Gonadotropin releasing hormone superagonist	10	Drug	Miscl.	1
I23	Fluorouracil	Antimetabolite	100	Drug	Miscl.	1
I24	cells	cells	None	cells	Negative Controls	1
J1	cells	cells	None	cells	Negative Controls	1
J2	Decitabine	Nucleoside analog DNA methyl transferase inhibitor	10	Drug	Miscl.	1
J3	Aminoglutethimide	Anti-steroid, aromatase inhibitor	1	Drug	Miscl.	1
J4	Capecitabine	5-FU prodrug	1	Drug	Miscl.	1
J5	Dexamethasone	Glucocorticoid, immunomodulatory agent	10	Drug	Miscl.	1
J6	Sirolimus	binds FKBP12, causes inhibition of mTORC1	0.1	Drug	Miscl.	1
J7	Pomalidomide	Immunomodulatory agent, anti-angiogenic	1	Drug	Miscl.	1
J8	Bimatoprost	Prostaglandin analog	5.5	Drug	Miscl.	1
J9	Plicamycin	RNA synthesis inhibitor	10	Drug	Miscl.	1
J10	Temsirolimus	binds FKBP12, causes inhibition of mTORC1	0.1	Drug	Miscl.	1
J11	Amsacrine	DNA intercalation, Topo II inhibitor	1	Drug	Topoisomerase	1
J12	Cabazitaxel	Taxane microtubule stabilizer, antimitotic	1	Drug	Miscl.	1
J13	Vinblastine	Mitotic inhibitor. Vinca alkaloid microtubule depolymerizer	0.1	Drug	Mitotic	1

J14	BzCl	BzCl	0	BzCl	Miscl.	1
J15	Eribulin	Mitotic inhibitor, microtubule depolymerizer.	1	Drug	Mitotic	1
J16	Letrozole	Aromatase inhibitor	10	Drug	Miscl.	1
J17	Cladribine	Antimetabolite; Purine analog	0.1	Drug	Miscl.	1
J18	Floxuridine	Antimetabolite; Analog of 5-fluorouracil	1	Drug	Miscl.	1
J19	Thioguanine	Antimetabolite; Purine analog	10	Drug	Miscl.	1
J20	Epirubicin	Topoisomerase II inhibitor	1	Drug	Topoisomerase	1
J21	Pirfenidone	Antifibrotic and anti-inflammatory	1	Drug	Miscl.	1
J22	Goserelin	Gonadotropin releasing hormone superagonist	1	Drug	Miscl.	1
J23	Fluorouracil	Antimetabolite	10	Drug	Miscl.	1
J24	cells	cells	None	cells	Negative Controls	1
K1	cells	cells	None	cells	Negative Controls	1
K2	Decitabine	Nucleoside analog DNA methyl transferase inhibitor	1	Drug	Miscl.	1
K3	Bexarotene	Antineoplastic agent; retinoid specifically selective for retinoid X receptors	1	Drug	Miscl.	1
K4	Methylprednisolone	Glucocorticoid, immunomodulatory agent	1	Drug	Miscl.	1
K5	Dexamethasone	Glucocorticoid, immunomodulatory agent	1	Drug	Miscl.	1
K6	Sirolimus	binds FKBP12, causes inhibition of mTORC1	0.01	Drug	Miscl.	1
K7	Vincristine	Mitotic inhibitor. Vinca alkaloid microtubule depolymerizer	0.1	Drug	Mitotic	1
K8	Bimatoprost	Prostaglandin analog	0.55000000000000004	Drug	Miscl.	1
K9	Plicamycin	RNA synthesis inhibitor	1	Drug	Miscl.	1
K10	Temsirolimus	binds FKBP12, causes inhibition of mTORC1	0.01	Drug	Miscl.	1
K11	SN-38	Active metabolite of irinotecan. Topoisomerase I inhibitor	1	Drug	Topoisomerase	1
K12	Cabazitaxel	Taxane microtubule stabilizer, antimitotic	0.1	Drug	Miscl.	1
K13	Dactinomycin	RNA and DNA synthesis inhibitor	0.1	Drug	Miscl.	1
K14	Anastrozole	Aromatase inhibitor	1	Drug	Miscl.	1
K15	Eribulin	Mitotic inhibitor, microtubule depolymerizer.	0.1	Drug	Mitotic	1
K16	Letrozole	Aromatase inhibitor	1	Drug	Miscl.	1
K17	Gemcitabine	Antimetabolite; Nucleoside analog	0.1	Drug	Miscl.	1
K18	Azacitidine	Nucleoside analog DNA methyl transferase inhibitor	1	Drug	Miscl.	1
K19	Thioguanine	Antimetabolite; Purine analog	1	Drug	Miscl.	1
K20	Epirubicin	Topoisomerase II inhibitor	0.1	Drug	Topoisomerase	1
K21	BzCl	BzCl	0	BzCl	Miscl.	1
K22	Imiquimod	Immunomodulatory agent, TLR7 agonist	0.25	Drug	Miscl.	1

K23	Fluorouracil	Antimetabolite	1	Drug	Miscl.	1
K24	cells	cells	None	cells	Negative Controls	1
L1	cells	cells	None	cells	Negative Controls	1
L2	Olaparib	PARP inhibitor	1	Drug	PARP	1
L3	Bexarotene	Antineoplastic agent; retinoid specifically selective for retinoid X receptors	10	Drug	Miscl.	1
L4	Methylprednisolone	Glucocorticoid, immunomodulatory agent	10	Drug	Miscl.	1
L5	Tacrolimus	Binds FKBP12, causes inhibition of calcineurin	1	Drug	Miscl.	1
L6	Rucaparib	PARP inhibitor	1	Drug	PARP	1
L7	Vincristine	Mitotic inhibitor. Vinca alkaloid microtubule depolymerizer	1	Drug	Mitotic	1
L8	Bleomycin	Glycopeptide antibiotic; causes DNA breaks	1	Drug	Miscl.	1
L9	Lenalidomide	Immunomodulatory	10	Drug	Miscl.	1
L10	Temozolomide	Alkylating agent	10	Drug	Miscl.	1
L11	SN-38	Active metabolite of irinotecan. Topoisomerase I inhibitor	10	Drug	Topoisomerase	1
L12	Romidepsin	HDAC inhibitor	0.1	Drug	HDAC	1
L13	Dactinomycin	RNA and DNA synthesis inhibitor	1	Drug	Miscl.	1
L14	Topotecan	Topoisomerase I inhibitor. Camptothecin analog	1	Drug	Topoisomerase	1
L15	Mitomycin C	Antineoplastic anatibiotic; DNA crosslinker	1	Drug	Miscl.	1
L16	Cytarabine	Antimetabolite, interferes with DNA synthesis	1	Drug	Miscl.	1
L17	DMSO	DMSO	0	DMSO	Negative Controls	1
L18	Azacitidine	Nucleoside analog DNA methyl transferase inhibitor	10	Drug	Miscl.	1
L19	Tretinoin	Retinoic acid receptor agonist	1	Drug	Miscl.	1
L20	Vinflunine	Mitotic inhibitor. Vinca alkaloid microtubule depolymerizer	0.1	Drug	Mitotic	1
L21	Acitretin	Retinoid receptor agonist	1	Drug	Miscl.	1
L22	Imiquimod	Immunomodulatory agent, TLR7 agonist	2.5	Drug	Miscl.	1
L23	Cytarabine/Idarubicin	Std. Induction therapy combination	0,5/0,05	Drug	Miscl.	1
L24	cells	cells	None	cells	Negative Controls	1
M1	cells	cells	None	cells	Negative Controls	1
M2	Olaparib	PARP inhibitor	10	Drug	PARP	1
M3	Bexarotene	Antineoplastic agent; retinoid specifically selective for retinoid X receptors	100	Drug	Miscl.	1
M4	BzCl	BzCl	0	BzCl	Miscl.	1
M5	Tacrolimus	Binds FKBP12, causes inhibition of calcineurin	10	Drug	Miscl.	1
M6	Rucaparib	PARP inhibitor	10	Drug	PARP	1
M7	Vincristine	Mitotic inhibitor. Vinca alkaloid microtubule depolymerizer	10	Drug	Mitotic	1

M8	Bleomycin	Glycopeptide antibiotic; causes DNA breaks	10	Drug	Miscl.	1
M9	Lenalidomide	Immunomodulatory	100	Drug	Miscl.	1
M10	Temozolomide	Alkylating agent	100	Drug	Miscl.	1
M11	SN-38	Active metabolite of irinotecan. Topoisomerase I inhibitor	100	Drug	Topoisomerase	1
M12	Romidepsin	HDAC inhibitor	1	Drug	HDAC	1
M13	Dactinomycin	RNA and DNA synthesis inhibitor	10	Drug	Miscl.	1
M14	Topotecan	Topoisomerase I inhibitor. Camptothecin analog	10	Drug	Topoisomerase	1
M15	Mitomycin C	Antineoplastic anatibiotic; DNA crosslinker	10	Drug	Miscl.	1
M16	Cytarabine	Antimetabolite, interferes with DNA synthesis	10	Drug	Miscl.	1
M17	Gemcitabine	Antimetabolite; Nucleoside analog	1	Drug	Miscl.	1
M18	Azacitidine	Nucleoside analog DNA methyl transferase inhibitor	100	Drug	Miscl.	1
M19	Tretinoin	Retinoic acid receptor agonist	10	Drug	Miscl.	1
M20	Vinflunine	Mitotic inhibitor. Vinca alkaloid microtubule depolymerizer	1	Drug	Mitotic	1
M21	Acitretin	Retinoid receptor agonist	10	Drug	Miscl.	1
M22	Imiquimod	Immunomodulatory agent, TLR7 agonist	25	Drug	Miscl.	1
M23	Cytarabine/Idarubicin	Std. Induction therapy combination	5/0,5	Drug	Miscl.	1
M24	cells	cells	None	cells	Negative Controls	1
N1	cells	cells	None	cells	Negative Controls	1
N2	Olaparib	PARP inhibitor	100	Drug	PARP	1
N3	Bexarotene	Antineoplastic agent; retinoid specifically selective for retinoid X receptors	1000	Drug	Miscl.	1
N4	Methylprednisolone	Glucocorticoid, immunomodulatory agent	100	Drug	Miscl.	1
N5	Tacrolimus	Binds FKBP12, causes inhibition of calcineurin	100	Drug	Miscl.	1
N6	Rucaparib	PARP inhibitor	100	Drug	PARP	1
N7	DMSO	DMSO	0	DMSO	Negative Controls	1
N8	Bleomycin	Glycopeptide antibiotic; causes DNA breaks	100	Drug	Miscl.	1
N9	Lenalidomide	Immunomodulatory	1000	Drug	Miscl.	1
N10	Temozolomide	Alkylating agent	1000	Drug	Miscl.	1
N11	BzCl	BzCl	0	BzCl	Miscl.	1
N12	Romidepsin	HDAC inhibitor	10	Drug	HDAC	1
N13	Dactinomycin	RNA and DNA synthesis inhibitor	100	Drug	Miscl.	1
N14	Topotecan	Topoisomerase I inhibitor. Camptothecin analog	100	Drug	Topoisomerase	1
N15	Mitomycin C	Antineoplastic anatibiotic; DNA crosslinker	100	Drug	Miscl.	1
N16	Cytarabine	Antimetabolite, interferes with DNA synthesis	100	Drug	Miscl.	1

N17	Gemcitabine	Antimetabolite; Nucleoside analog	10	Drug	Miscl.	1
N18	Azacitidine	Nucleoside analog DNA methyl transferase inhibitor	1000	Drug	Miscl.	1
N19	Tretinoin	Retinoic acid receptor agonist	100	Drug	Miscl.	1
N20	Vinflunine	Mitotic inhibitor. Vinca alkaloid microtubule depolymerizer	10	Drug	Mitotic	1
N21	Acitretin	Retinoid receptor agonist	100	Drug	Miscl.	1
N22	Imiquimod	Immunomodulatory agent, TLR7 agonist	250	Drug	Miscl.	1
N23	Cytarabine/Idarubicin	Std. Induction therapy combination	50/5	Drug	Miscl.	1
N24	cells	cells	None	cells	Negative Controls	1
O1	cells	cells	None	cells	Negative Controls	1
O2	Olaparib	PARP inhibitor	1000	Drug	PARP	1
O3	Bexarotene	Antineoplastic agent; retinoid specifically selective for retinoid X receptors	10000	Drug	Miscl.	1
O4	Methylprednisolone	Glucocorticoid, immunomodulatory agent	1000	Drug	Miscl.	1
O5	Tacrolimus	Binds FKBP12, causes inhibition of calcineurin	1000	Drug	Miscl.	1
O6	Rucaparib	PARP inhibitor	1000	Drug	PARP	1
O7	Vincristine	Mitotic inhibitor. Vinca alkaloid microtubule depolymerizer	100	Drug	Mitotic	1
O8	Bleomycin	Glycopeptide antibiotic; causes DNA breaks	1000	Drug	Miscl.	1
O9	Lenalidomide	Immunomodulatory	10000	Drug	Miscl.	1
O10	Temozolomide	Alkylating agent	10000	Drug	Miscl.	1
O11	SN-38	Active metabolite of irinotecan. Topoisomerase I inhibitor	1000	Drug	Topoisomerase	1
O12	Romidepsin	HDAC inhibitor	100	Drug	HDAC	1
O13	DMSO	DMSO	0	DMSO	Negative Controls	1
O14	Topotecan	Topoisomerase I inhibitor. Camptothecin analog	1000	Drug	Topoisomerase	1
O15	Mitomycin C	Antineoplastic anatibiotic; DNA crosslinker	1000	Drug	Miscl.	1
O16	Cytarabine	Antimetabolite, interferes with DNA synthesis	1000	Drug	Miscl.	1
O17	Gemcitabine	Antimetabolite; Nucleoside analog	100	Drug	Miscl.	1
O18	BzCl	BzCl	0	BzCl	Miscl.	1
O19	Tretinoin	Retinoic acid receptor agonist	1000	Drug	Miscl.	1
O20	Vinflunine	Mitotic inhibitor. Vinca alkaloid microtubule depolymerizer	100	Drug	Mitotic	1
O21	Acitretin	Retinoid receptor agonist	1000	Drug	Miscl.	1
O22	Imiquimod	Immunomodulatory agent, TLR7 agonist	2500	Drug	Miscl.	1
O23	Cytarabine/Idarubicin	Std. Induction therapy combination	500/50	Drug	Miscl.	1
O24	cells	cells	None	cells	Negative Controls	1
P1	cells	cells	None	cells	Negative Controls	1

P2	Olaparib	PARP inhibitor	10000	Drug	PARP	1
P3	DMSO	DMSO	0	DMSO	Negative Controls	1
P4	Methylprednisolone	Glucocorticoid, immunomodulatory agent	10000	Drug	Miscl.	1
P5	Tacrolimus	Binds FKBP12, causes inhibition of calcineurin	10000	Drug	Miscl.	1
P6	Rucaparib	PARP inhibitor	10000	Drug	PARP	1
P7	Vincristine	Mitotic inhibitor. Vinca alkaloid microtubule depolymerizer	1000	Drug	Mitotic	1
P8	Bleomycin	Glycopeptide antibiotic; causes DNA breaks	10000	Drug	Miscl.	1
P9	Lenalidomide	Immunomodulatory	100000	Drug	Miscl.	1
P10	Temozolomide	Alkylating agent	100000	Drug	Miscl.	1
P11	SN-38	Active metabolite of irinotecan. Topoisomerase I inhibitor	10000	Drug	Topoisomerase	1
P12	Romidepsin	HDAC inhibitor	1000	Drug	HDAC	1
P13	Dactinomycin	RNA and DNA synthesis inhibitor	1000	Drug	Miscl.	1
P14	Topotecan	Topoisomerase I inhibitor. Camptothecin analog	10000	Drug	Topoisomerase	1
P15	Mitomycin C	Antineoplastic anatibiotic; DNA crosslinker	10000	Drug	Miscl.	1
P16	Cytarabine	Antimetabolite, interferes with DNA synthesis	10000	Drug	Miscl.	1
P17	Gemcitabine	Antimetabolite; Nucleoside analog	1000	Drug	Miscl.	1
P18	Azacitidine	Nucleoside analog DNA methyl transferase inhibitor	10000	Drug	Miscl.	1
P19	Tretinoin	Retinoic acid receptor agonist	10000	Drug	Miscl.	1
P20	Vinflunine	Mitotic inhibitor. Vinca alkaloid microtubule depolymerizer	1000	Drug	Mitotic	1
P21	Acitretin	Retinoid receptor agonist	10000	Drug	Miscl.	1
P22	DMSO	DMSO	0	DMSO	Negative Controls	1
P23	Cytarabine/Idarubicin	Std. Induction therapy combination	5000/500	Drug	Miscl.	1
P24	BzCl	BzCl	0	BzCl	Miscl.	1
A1	cells	cells	None	cells	Negative Controls	2
A2	BzCl	BzCl	0	BzCl	Miscl.	2
A3	Fingolimod	S1PR antagonist	10000	Drug	Miscl.	2
A4	Toremifene	selective estrogen receptor modulator	10000	Drug	Miscl.	2
A5	Pilocarpine	Non-selective muscarinic receptor agonist	40000	Drug	Miscl.	2
A6	Lasofoxifene	Selective estrogen receptor modulator	1000	Drug	Miscl.	2
A7	Itraconazole	antifungal, hedgehog signaling inhibitor	5000	Drug	Miscl.	2
A8	Exemestane	Aromatase inhibitor	10000	Drug	Miscl.	2
A9	Tamoxifen	Estrogen receptor antagonist	10000	Drug	Miscl.	2
A10	Enzalutamide	AR antagonist	10000	Drug	Miscl.	2

A11	Volasertib	PLK1 inhibitor	1000	Drug	Miscl.	2
A12	Trametinib	MEK1/2 inhibitor	250	Drug	MEK1/2	2
A13	Ibrutinib	Btk inhibitor	1000	Drug	Miscl.	2
A14	Dabrafenib	B-Raf(V600E) inhibitor	2500	Drug	Miscl.	2
A15	Lenvatinib	VEGFR inhibitor	2500	Drug	VEGFR	2
A16	Gefitinib	EGFR inhibitor	10000	Drug	EGFR	2
A17	Nintedanib	VEGFR, PDGFR, FGFR inhibitor	10000	Drug	VEGFR	2
A18	Ceritinib	ALK inhibitor	2500	Drug	Miscl.	2
A19	Erlotinib	EGFR inhibitor	10000	Drug	EGFR	2
A20	Tivozanib	VEGFR1, 2, 3, c-Kit, PDGFRB inhibitor	10000	Drug	VEGFR	2
A21	Saracatinib	Src, Abl inhibitor	10000	Drug	Miscl.	2
A22	AZD1152-HQPA	Aurora B inhibitor	1000	Drug	Miscl.	2
A23	BzCl	BzCl	0	BzCl	Miscl.	2
A24	cells	cells	None	cells	Negative Controls	2
B1	cells	cells	None	cells	Negative Controls	2
B2	Sonidegib	Smothened (Hh) inhib	10000	Drug	Miscl.	2
B3	Fingolimod	S1PR antagonist	1000	Drug	Miscl.	2
B4	Toremifene	selective estrogen receptor modulator	1000	Drug	Miscl.	2
B5	Pilocarpine	Non-selective muscarinic receptor agonist	4000	Drug	Miscl.	2
B6	Lasofoxifene	Selective estrogen receptor modulator	100	Drug	Miscl.	2
B7	Itraconazole	antifungal, hedgehog signaling inhibitor	500	Drug	Miscl.	2
B8	Exemestane	Aromatase inhibitor	1000	Drug	Miscl.	2
B9	DMSO	DMSO	0	DMSO	Negative Controls	2
B10	Enzalutamide	AR antagonist	1000	Drug	Miscl.	2
B11	Volasertib	PLK1 inhibitor	100	Drug	Miscl.	2
B12	Trametinib	MEK1/2 inhibitor	25	Drug	MEK1/2	2
B13	Ibrutinib	Btk inhibitor	100	Drug	Miscl.	2
B14	Dabrafenib	B-Raf(V600E) inhibitor	250	Drug	Miscl.	2
B15	Lenvatinib	VEGFR inhibitor	250	Drug	VEGFR	2
B16	BzCl	BzCl	0	BzCl	Miscl.	2
B17	Nintedanib	VEGFR, PDGFR, FGFR inhibitor	1000	Drug	VEGFR	2
B18	Ceritinib	ALK inhibitor	250	Drug	Miscl.	2
B19	Erlotinib	EGFR inhibitor	1000	Drug	EGFR	2

B20	Tivozanib	VEGFR1, 2, 3, c-Kit, PDGFRB inhibitor	1000	Drug	VEGFR	2
B21	Saracatinib	Src, Abl inhibitor	1000	Drug	Miscl.	2
B22	AZD1152-HQPA	Aurora B inhibitor	100	Drug	Miscl.	2
B23	Midostaurin	Broad TK (FLT3, KIT, RET, JAK, EGFR...) inhibitor	10000	Drug	Miscl.	2
B24	BzCl	BzCl	0	BzCl	Miscl.	2
C1	cells	cells	None	cells	Negative Controls	2
C2	Sonidegib	Smothened (Hh) inhib	1000	Drug	Miscl.	2
C3	Fingolimod	S1PR antagonist	100	Drug	Miscl.	2
C4	Toremifene	selective estrogen receptor modulator	100	Drug	Miscl.	2
C5	Pilocarpine	Non-selective muscarinic receptor agonist	400	Drug	Miscl.	2
C6	Lasofoxifene	Selective estrogen receptor modulator	10	Drug	Miscl.	2
C7	Itraconazole	antifungal, hedgehog signaling inhibitor	50	Drug	Miscl.	2
C8	Exemestane	Aromatase inhibitor	100	Drug	Miscl.	2
C9	Tamoxifen	Estrogen receptor antagonist	1000	Drug	Miscl.	2
C10	Enzalutamide	AR antagonist	100	Drug	Miscl.	2
C11	Volasertib	PLK1 inhibitor	10	Drug	Miscl.	2
C12	DMSO	DMSO	0	DMSO	Negative Controls	2
C13	Ibrutinib	Btk inhibitor	10	Drug	Miscl.	2
C14	Dabrafenib	B-Raf(V600E) inhibitor	25	Drug	Miscl.	2
C15	Lenvatinib	VEGFR inhibitor	25	Drug	VEGFR	2
C16	Gefitinib	EGFR inhibitor	1000	Drug	EGFR	2
C17	Nintedanib	VEGFR, PDGFR, FGFR inhibitor	100	Drug	VEGFR	2
C18	Ceritinib	ALK inhibitor	25	Drug	Miscl.	2
C19	Erlotinib	EGFR inhibitor	100	Drug	EGFR	2
C20	DMSO	DMSO	0	DMSO	Negative Controls	2
C21	Saracatinib	Src, Abl inhibitor	100	Drug	Miscl.	2
C22	AZD1152-HQPA	Aurora B inhibitor	10	Drug	Miscl.	2
C23	Midostaurin	Broad TK (FLT3, KIT, RET, JAK, EGFR...) inhibitor	1000	Drug	Miscl.	2
C24	cells	cells	None	cells	Negative Controls	2
D1	cells	cells	None	cells	Negative Controls	2
D2	Sonidegib	Smothened (Hh) inhib	100	Drug	Miscl.	2
D3	Fingolimod	S1PR antagonist	10	Drug	Miscl.	2
D4	Toremifene	selective estrogen receptor modulator	10	Drug	Miscl.	2

D5	DMSO	DMSO	0	DMSO	Negative Controls	2
D6	Lasofoxifene	Selective estrogen receptor modulator	1	Drug	Miscl.	2
D7	Itraconazole	antifungal, hedgehog signaling inhibitor	5	Drug	Miscl.	2
D8	Exemestane	Aromatase inhibitor	10	Drug	Miscl.	2
D9	Tamoxifen	Estrogen receptor antagonist	100	Drug	Miscl.	2
D10	Enzalutamide	AR antagonist	10	Drug	Miscl.	2
D11	Volasertib	PLK1 inhibitor	1	Drug	Miscl.	2
D12	Trametinib	MEK1/2 inhibitor	2.5	Drug	MEK1/2	2
D13	Ibrutinib	Btk inhibitor	1	Drug	Miscl.	2
D14	Dabrafenib	B-Raf(V600E) inhibitor	2.5	Drug	Miscl.	2
D15	Lenvatinib	VEGFR inhibitor	2.5	Drug	VEGFR	2
D16	Gefitinib	EGFR inhibitor	100	Drug	EGFR	2
D17	Nintedanib	VEGFR, PDGFR, FGFR inhibitor	10	Drug	VEGFR	2
D18	Ceritinib	ALK inhibitor	2.5	Drug	Miscl.	2
D19	Erlotinib	EGFR inhibitor	10	Drug	EGFR	2
D20	Tivozanib	VEGFR1, 2, 3, c-Kit, PDGFRB inhibitor	100	Drug	VEGFR	2
D21	Saracatinib	Src, Abl inhibitor	10	Drug	Miscl.	2
D22	AZD1152-HQPA	Aurora B inhibitor	1	Drug	Miscl.	2
D23	Midostaurin	Broad TK (FLT3, KIT, RET, JAK, EGFR…) inhibitor	100	Drug	Miscl.	2
D24	cells	cells	None	cells	Negative Controls	2
E1	cells	cells	None	cells	Negative Controls	2
E2	Sonidegib	Smothened (Hh) inhib	10	Drug	Miscl.	2
E3	Fingolimod	S1PR antagonist	1	Drug	Miscl.	2
E4	Toremifene	selective estrogen receptor modulator	1	Drug	Miscl.	2
E5	Pilocarpine	Non-selective muscarinic receptor agonist	40	Drug	Miscl.	2
E6	Lasofoxifene	Selective estrogen receptor modulator	0.1	Drug	Miscl.	2
E7	Itraconazole	antifungal, hedgehog signaling inhibitor	0.5	Drug	Miscl.	2
E8	Exemestane	Aromatase inhibitor	1	Drug	Miscl.	2
E9	Tamoxifen	Estrogen receptor antagonist	10	Drug	Miscl.	2
E10	Enzalutamide	AR antagonist	1	Drug	Miscl.	2
E11	Volasertib	PLK1 inhibitor	0.1	Drug	Miscl.	2
E12	Trametinib	MEK1/2 inhibitor	0.25	Drug	MEK1/2	2
E13	Ibrutinib	Btk inhibitor	0.1	Drug	Miscl.	2

E14	Dabrafenib	B-Raf(V600E) inhibitor	0.25	Drug	Miscl.	2
E15	DMSO	DMSO	0	DMSO	Negative Controls	2
E16	Gefitinib	EGFR inhibitor	10	Drug	EGFR	2
E17	Nintedanib	VEGFR, PDGFR, FGFR inhibitor	1	Drug	VEGFR	2
E18	Ceritinib	ALK inhibitor	0.25	Drug	Miscl.	2
E19	Erlotinib	EGFR inhibitor	1	Drug	EGFR	2
E20	Tivozanib	VEGFR1, 2, 3, c-Kit, PDGFRB inhibitor	10	Drug	VEGFR	2
E21	Saracatinib	Src, Abl inhibitor	1	Drug	Miscl.	2
E22	AZD1152-HQPA	Aurora B inhibitor	0.1	Drug	Miscl.	2
E23	Midostaurin	Broad TK (FLT3, KIT, RET, JAK, EGFR…) inhibitor	10	Drug	Miscl.	2
E24	cells	cells	None	cells	Negative Controls	2
F1	cells	cells	None	cells	Negative Controls	2
F2	Sonidegib	Smothened (Hh) inhib	1	Drug	Miscl.	2
F3	Vismodegib	Smothened (Hh) inhibitor	10000	Drug	Miscl.	2
F4	Finasteride	type II 5-alpha reductase inhibitor	10000	Drug	Miscl.	2
F5	Pilocarpine	Non-selective muscarinic receptor agonist	4	Drug	Miscl.	2
F6	Methotrexate	Antimetabolite; Anti-folate agent	5000	Drug	Miscl.	2
F7	Fulvestrant	Estrogen receptor antagonist	1000	Drug	Miscl.	2
F8	Atorvastatin	HMG CoA reductase inhibitor	10000	Drug	Miscl.	2
F9	Tamoxifen	Estrogen receptor antagonist	1	Drug	Miscl.	2
F10	BzCl	BzCl	0	BzCl	Miscl.	2
F11	Imatinib	Abl, Kit, PDGFRB inhibitor	10000	Drug	Miscl.	2
F12	Trametinib	MEK1/2 inhibitor	2.5000000000000001E-2	Drug	MEK1/2	2
F13	Axitinib	VEGFR, PDGFR, KIT inhibitor	10000	Drug	VEGFR	2
F14	Cobimetinib	MEK1/2 inhibitor	1000	Drug	MEK1/2	2
F15	Lenvatinib	VEGFR inhibitor	0.25	Drug	VEGFR	2
F16	Gefitinib	EGFR inhibitor	1	Drug	EGFR	2
F17	Ripasudil	ROCK inhibitor	10000	Drug	Miscl.	2
F18	Vemurafenib	B-Raf(V600E) inhibitor	10000	Drug	Miscl.	2
F19	Regorafenib	B-Raf, c-Kit, VEGFR2 inhibitor	10000	Drug	VEGFR	2
F20	Tivozanib	VEGFR1, 2, 3, c-Kit, PDGFRB inhibitor	1	Drug	VEGFR	2
F21	Vatalanib	VEGFR-1 & -2 inhibitor	10000	Drug	VEGFR	2
F22	Enzastaurin	PKCbeta inhibitor	10000	Drug	Miscl.	2

F23	Midostaurin	Broad TK (FLT3, KIT, RET, JAK, EGFR...) inhibitor	1	Drug	Miscl.	2
F24	cells	cells	None	cells	Negative Controls	2
G1	cells	cells	None	cells	Negative Controls	2
G2	Abiraterone	P450 17alpha-hydroxylase-17,20-lyase inhibitor	5000	Drug	Miscl.	2
G3	Vismodegib	Smothened (Hh) inhibitor	1000	Drug	Miscl.	2
G4	Finasteride	type II 5-alpha reductase inhibitor	1000	Drug	Miscl.	2
G5	Anagrelide	PDE-3, PLA2 inhibitor	10000	Drug	Miscl.	2
G6	BzCl	BzCl	0	BzCl	Miscl.	2
G7	Fulvestrant	Estrogen receptor antagonist	100	Drug	Miscl.	2
G8	Atorvastatin	HMG CoA reductase inhibitor	1000	Drug	Miscl.	2
G9	Celecoxib	Selective COX-2 inhibitor	10000	Drug	Miscl.	2
G10	Apatinib	VEGFR inhibitor	10000	Drug	VEGFR	2
G11	Imatinib	Abl, Kit, PDGFRB inhibitor	1000	Drug	Miscl.	2
G12	Dasatinib	Abl, Src, Kit, EphR... Inhibitor	1000	Drug	Miscl.	2
G13	Axitinib	VEGFR, PDGFR, KIT inhibitor	1000	Drug	VEGFR	2
G14	Cobimetinib	MEK1/2 inhibitor	100	Drug	MEK1/2	2
G15	Alectinib	ALK (incl gatekeeper mut) inhib	1000	Drug	Miscl.	2
G16	Crizotinib	ALK, c-Met inhibitor	1000	Drug	Miscl.	2
G17	Ripasudil	ROCK inhibitor	1000	Drug	Miscl.	2
G18	Vemurafenib	B-Raf(V600E) inhibitor	1000	Drug	Miscl.	2
G19	Regorafenib	B-Raf, c-Kit, VEGFR2 inhibitor	1000	Drug	VEGFR	2
G20	Dovitinib	FGFR inhibitor	10000	Drug	Miscl.	2
G21	Vatalanib	VEGFR-1 & -2 inhibitor	1000	Drug	VEGFR	2
G22	Enzastaurin	PKCbeta inhibitor	1000	Drug	Miscl.	2
G23	Alisertib	Aurora A inhibitor	10000	Drug	Miscl.	2
G24	cells	cells	None	cells	Negative Controls	2
H1	cells	cells	None	cells	Negative Controls	2
H2	Abiraterone	P450 17alpha-hydroxylase-17,20-lyase inhibitor	500	Drug	Miscl.	2
H3	Vismodegib	Smothened (Hh) inhibitor	100	Drug	Miscl.	2
H4	Finasteride	type II 5-alpha reductase inhibitor	100	Drug	Miscl.	2
H5	Anagrelide	PDE-3, PLA2 inhibitor	1000	Drug	Miscl.	2
H6	Methotrexate	Antimetabolite; Anti-folate agent	500	Drug	Miscl.	2
H7	Fulvestrant	Estrogen receptor antagonist	10	Drug	Miscl.	2

H8	Atorvastatin	HMG CoA reductase inhibitor	100	Drug	Miscl.	2
H9	Celecoxib	Selective COX-2 inhibitor	1000	Drug	Miscl.	2
H10	Apatinib	VEGFR inhibitor	1000	Drug	VEGFR	2
H11	Imatinib	Abl, Kit, PDGFRB inhibitor	100	Drug	Miscl.	2
H12	Dasatinib	Abl, Src, Kit, EphR… Inhibitor	100	Drug	Miscl.	2
H13	Axitinib	VEGFR, PDGFR, KIT inhibitor	100	Drug	VEGFR	2
H14	Cobimetinib	MEK1/2 inhibitor	10	Drug	MEK1/2	2
H15	Alectinib	ALK (incl gatekeeper mut) inhib	100	Drug	Miscl.	2
H16	Crizotinib	ALK, c-Met inhibitor	100	Drug	Miscl.	2
H17	Ripasudil	ROCK inhibitor	100	Drug	Miscl.	2
H18	Vemurafenib	B-Raf(V600E) inhibitor	100	Drug	Miscl.	2
H19	DMSO	DMSO	0	DMSO	Negative Controls	2
H20	Dovitinib	FGFR inhibitor	1000	Drug	Miscl.	2
H21	Vatalanib	VEGFR-1 & -2 inhibitor	100	Drug	VEGFR	2
H22	Enzastaurin	PKCbeta inhibitor	100	Drug	Miscl.	2
H23	Alisertib	Aurora A inhibitor	1000	Drug	Miscl.	2
H24	cells	cells	None	cells	Negative Controls	2
I1	cells	cells	None	cells	Negative Controls	2
I2	Abiraterone	P450 17alpha-hydroxylase-17,20-lyase inhibitor	50	Drug	Miscl.	2
I3	Vismodegib	Smothened (Hh) inhibitor	10	Drug	Miscl.	2
I4	Finasteride	type II 5-alpha reductase inhibitor	10	Drug	Miscl.	2
I5	Anagrelide	PDE-3, PLA2 inhibitor	100	Drug	Miscl.	2
I6	Methotrexate	Antimetabolite; Anti-folate agent	50	Drug	Miscl.	2
I7	Fulvestrant	Estrogen receptor antagonist	1	Drug	Miscl.	2
I8	DMSO	DMSO	0	DMSO	Negative Controls	2
I9	Celecoxib	Selective COX-2 inhibitor	100	Drug	Miscl.	2
I10	Apatinib	VEGFR inhibitor	100	Drug	VEGFR	2
I11	Imatinib	Abl, Kit, PDGFRB inhibitor	10	Drug	Miscl.	2
I12	Dasatinib	Abl, Src, Kit, EphR… Inhibitor	10	Drug	Miscl.	2
I13	Axitinib	VEGFR, PDGFR, KIT inhibitor	10	Drug	VEGFR	2
I14	Cobimetinib	MEK1/2 inhibitor	1	Drug	MEK1/2	2
I15	Alectinib	ALK (incl gatekeeper mut) inhib	10	Drug	Miscl.	2
I16	Crizotinib	ALK, c-Met inhibitor	10	Drug	Miscl.	2

I17	Ripasudil	ROCK inhibitor	10	Drug	Miscl.	2
I18	Vemurafenib	B-Raf(V600E) inhibitor	10	Drug	Miscl.	2
I19	Regorafenib	B-Raf, c-Kit, VEGFR2 inhibitor	100	Drug	VEGFR	2
I20	Dovitinib	FGFR inhibitor	100	Drug	Miscl.	2
I21	Vatalanib	VEGFR-1 & -2 inhibitor	10	Drug	VEGFR	2
I22	Enzastaurin	PKCbeta inhibitor	10	Drug	Miscl.	2
I23	Alisertib	Aurora A inhibitor	100	Drug	Miscl.	2
I24	cells	cells	None	cells	Negative Controls	2
J1	cells	cells	None	cells	Negative Controls	2
J2	Abiraterone	P450 17alpha-hydroxylase-17,20-lyase inhibitor	5	Drug	Miscl.	2
J3	Vismodegib	Smothened (Hh) inhibitor	1	Drug	Miscl.	2
J4	Finasteride	type II 5-alpha reductase inhibitor	1	Drug	Miscl.	2
J5	Anagrelide	PDE-3, PLA2 inhibitor	10	Drug	Miscl.	2
J6	Methotrexate	Antimetabolite; Anti-folate agent	5	Drug	Miscl.	2
J7	Fulvestrant	Estrogen receptor antagonist	0.1	Drug	Miscl.	2
J8	Atorvastatin	HMG CoA reductase inhibitor	10	Drug	Miscl.	2
J9	Celecoxib	Selective COX-2 inhibitor	10	Drug	Miscl.	2
J10	Apatinib	VEGFR inhibitor	10	Drug	VEGFR	2
J11	Imatinib	Abl, Kit, PDGFRB inhibitor	1	Drug	Miscl.	2
J12	Dasatinib	Abl, Src, Kit, EphR... Inhibitor	1	Drug	Miscl.	2
J13	Axitinib	VEGFR, PDGFR, KIT inhibitor	1	Drug	VEGFR	2
J14	BzCl	BzCl	0	BzCl	Miscl.	2
J15	Alectinib	ALK (incl gatekeeper mut) inhib	1	Drug	Miscl.	2
J16	Crizotinib	ALK, c-Met inhibitor	1	Drug	Miscl.	2
J17	Ripasudil	ROCK inhibitor	1	Drug	Miscl.	2
J18	Vemurafenib	B-Raf(V600E) inhibitor	1	Drug	Miscl.	2
J19	Regorafenib	B-Raf, c-Kit, VEGFR2 inhibitor	10	Drug	VEGFR	2
J20	Dovitinib	FGFR inhibitor	10	Drug	Miscl.	2
J21	Vatalanib	VEGFR-1 & -2 inhibitor	1	Drug	VEGFR	2
J22	Enzastaurin	PKCbeta inhibitor	1	Drug	Miscl.	2
J23	Alisertib	Aurora A inhibitor	10	Drug	Miscl.	2
J24	cells	cells	None	cells	Negative Controls	2
K1	cells	cells	None	cells	Negative Controls	2

K2	Abiraterone	P450 17alpha-hydroxylase-17,20-lyase inhibitor	0.5	Drug	Miscl.	2
K3	Nilutamide	Nonsteroidal antiandrogen	1	Drug	Miscl.	2
K4	Flutamide	Nonsteroidal antiandrogen	1	Drug	Miscl.	2
K5	Anagrelide	PDE-3, PLA2 inhibitor	1	Drug	Miscl.	2
K6	Methotrexate	Antimetabolite; Anti-folate agent	0.5	Drug	Miscl.	2
K7	Clomifene	Selective estrogen receptor modulator	1	Drug	Miscl.	2
K8	Atorvastatin	HMG CoA reductase inhibitor	1	Drug	Miscl.	2
K9	Celecoxib	Selective COX-2 inhibitor	1	Drug	Miscl.	2
K10	Apatinib	VEGFR inhibitor	1	Drug	VEGFR	2
K11	Afatinib	EGFR inhibitor	0.1	Drug	EGFR	2
K12	Dasatinib	Abl, Src, Kit, EphR… Inhibitor	0.1	Drug	Miscl.	2
K13	Vandetanib	VEGFR,EGFR, RET inhibitor	0.1	Drug	VEGFR	2
K14	Cobimetinib	MEK1/2 inhibitor	0.1	Drug	MEK1/2	2
K15	Alectinib	ALK (incl gatekeeper mut) inhib	0.1	Drug	Miscl.	2
K16	Crizotinib	ALK, c-Met inhibitor	0.1	Drug	Miscl.	2
K17	Pazopanib	VEGFR inhibitor	1	Drug	VEGFR	2
K18	Hydroxyfasudil	ROCK, PKA, PKG, PRK inhibitor	1.9	Drug	Miscl.	2
K19	Regorafenib	B-Raf, c-Kit, VEGFR2 inhibitor	1	Drug	VEGFR	2
K20	Dovitinib	FGFR inhibitor	1	Drug	Miscl.	2
K21	BzCl	BzCl	0	BzCl	Miscl.	2
K22	Ruboxistaurin	PKCbeta inhibitor	1	Drug	Miscl.	2
K23	Alisertib	Aurora A inhibitor	1	Drug	Miscl.	2
K24	cells	cells	None	cells	Negative Controls	2
L1	cells	cells	None	cells	Negative Controls	2
L2	Digoxin	Cardiac glycoside	0.1	Drug	Miscl.	2
L3	Nilutamide	Nonsteroidal antiandrogen	10	Drug	Miscl.	2
L4	Flutamide	Nonsteroidal antiandrogen	10	Drug	Miscl.	2
L5	Simvastatin	HMG CoA reductase inhibitor	1	Drug	Miscl.	2
L6	Lovastatin	HMG-CoA reductase inhibitor	1	Drug	Miscl.	2
L7	Clomifene	Selective estrogen receptor modulator	10	Drug	Miscl.	2
L8	Megestrol acetate	Progestogen	1	Drug	Miscl.	2
L9	Salinomycin	Ionophore	5	Drug	Miscl.	2
L10	Idelalisib	PI3K inhibitor, p110δ-selective	1	Drug	PI3K	2

L11	Afatinib	EGFR inhibitor	1	Drug	EGFR	2
L12	Sorafenib	B-Raf, FGFR-1, VEGFR-2 & -3, PDGFR-beta, KIT, and FLT3 inhib	0.1	Drug	VEGFR	2
L13	Vandetanib	VEGFR,EGFR, RET inhibitor	1	Drug	VEGFR	2
L14	Bosutinib	Abl, Src inhibitor	1	Drug	Miscl.	2
L15	Nilotinib	Abl inhibitor	1	Drug	Miscl.	2
L16	Osimertinib	EGFR(L858R/T790M) inhibitor	0.25	Drug	EGFR	2
L17	DMSO	DMSO	0	DMSO	Negative Controls	2
L18	Hydroxyfasudil	ROCK, PKA, PKG, PRK inhibitor	19	Drug	Miscl.	2
L19	Lapatinib	HER2, EGFR inhibitor	0.1	Drug	EGFR	2
L20	Selumetinib	MEK1/2 inhibitor	1	Drug	MEK1/2	2
L21	Cediranib	KDR/Flt/VEGFR inhibitor	0.1	Drug	VEGFR	2
L22	Ruboxistaurin	PKCbeta inhibitor	10	Drug	Miscl.	2
L23	Masitinib	KIT inhibitor	1	Drug	Miscl.	2
L24	cells	cells	None	cells	Negative Controls	2
M1	cells	cells	None	cells	Negative Controls	2
M2	Digoxin	Cardiac glycoside	1	Drug	Miscl.	2
M3	Nilutamide	Nonsteroidal antiandrogen	100	Drug	Miscl.	2
M4	BzCl	BzCl	0	BzCl	Miscl.	2
M5	Simvastatin	HMG CoA reductase inhibitor	10	Drug	Miscl.	2
M6	Lovastatin	HMG-CoA reductase inhibitor	10	Drug	Miscl.	2
M7	Clomifene	Selective estrogen receptor modulator	100	Drug	Miscl.	2
M8	Megestrol acetate	Progestogen	10	Drug	Miscl.	2
M9	Salinomycin	Ionophore	50	Drug	Miscl.	2
M10	Idelalisib	PI3K inhibitor, p110δ-selective	10	Drug	PI3K	2
M11	Afatinib	EGFR inhibitor	10	Drug	EGFR	2
M12	Sorafenib	B-Raf, FGFR-1, VEGFR-2 & -3, PDGFR-beta, KIT, and FLT3 inhib	1	Drug	VEGFR	2
M13	Vandetanib	VEGFR,EGFR, RET inhibitor	10	Drug	VEGFR	2
M14	Bosutinib	Abl, Src inhibitor	10	Drug	Miscl.	2
M15	Nilotinib	Abl inhibitor	10	Drug	Miscl.	2
M16	Osimertinib	EGFR(L858R/T790M) inhibitor	2.5	Drug	EGFR	2
M17	Pazopanib	VEGFR inhibitor	10	Drug	VEGFR	2
M18	Hydroxyfasudil	ROCK, PKA, PKG, PRK inhibitor	190	Drug	Miscl.	2
M19	Lapatinib	HER2, EGFR inhibitor	1	Drug	EGFR	2

M20	Selumetinib	MEK1/2 inhibitor	10	Drug	MEK1/2	2
M21	Cediranib	KDR/Flt/VEGFR inhibitor	1	Drug	VEGFR	2
M22	Ruboxistaurin	PKCbeta inhibitor	100	Drug	Miscl.	2
M23	Masitinib	KIT inhibitor	10	Drug	Miscl.	2
M24	cells	cells	None	cells	Negative Controls	2
N1	cells	cells	None	cells	Negative Controls	2
N2	Digoxin	Cardiac glycoside	10	Drug	Miscl.	2
N3	Nilutamide	Nonsteroidal antiandrogen	1000	Drug	Miscl.	2
N4	Flutamide	Nonsteroidal antiandrogen	100	Drug	Miscl.	2
N5	Simvastatin	HMG CoA reductase inhibitor	100	Drug	Miscl.	2
N6	Lovastatin	HMG-CoA reductase inhibitor	100	Drug	Miscl.	2
N7	DMSO	DMSO	0	DMSO	Negative Controls	2
N8	Megestrol acetate	Progestogen	100	Drug	Miscl.	2
N9	Salinomycin	Ionophore	500	Drug	Miscl.	2
N10	Idelalisib	PI3K inhibitor, p110δ-selective	100	Drug	PI3K	2
N11	BzCl	BzCl	0	BzCl	Miscl.	2
N12	Sorafenib	B-Raf, FGFR-1, VEGFR-2 & -3, PDGFR-beta, KIT, and FLT3 inhib	10	Drug	VEGFR	2
N13	Vandetanib	VEGFR,EGFR, RET inhibitor	100	Drug	VEGFR	2
N14	Bosutinib	Abl, Src inhibitor	100	Drug	Miscl.	2
N15	Nilotinib	Abl inhibitor	100	Drug	Miscl.	2
N16	Osimertinib	EGFR(L858R/T790M) inhibitor	25	Drug	EGFR	2
N17	Pazopanib	VEGFR inhibitor	100	Drug	VEGFR	2
N18	Hydroxyfasudil	ROCK, PKA, PKG, PRK inhibitor	1900	Drug	Miscl.	2
N19	Lapatinib	HER2, EGFR inhibitor	10	Drug	EGFR	2
N20	Selumetinib	MEK1/2 inhibitor	100	Drug	MEK1/2	2
N21	Cediranib	KDR/Flt/VEGFR inhibitor	10	Drug	VEGFR	2
N22	Ruboxistaurin	PKCbeta inhibitor	1000	Drug	Miscl.	2
N23	Masitinib	KIT inhibitor	100	Drug	Miscl.	2
N24	cells	cells	None	cells	Negative Controls	2
O1	cells	cells	None	cells	Negative Controls	2
O2	Digoxin	Cardiac glycoside	100	Drug	Miscl.	2
O3	Nilutamide	Nonsteroidal antiandrogen	10000	Drug	Miscl.	2
O4	Flutamide	Nonsteroidal antiandrogen	1000	Drug	Miscl.	2

O5	Simvastatin	HMG CoA reductase inhibitor	1000	Drug	Miscl.	2
O6	Lovastatin	HMG-CoA reductase inhibitor	1000	Drug	Miscl.	2
O7	Clomifene	Selective estrogen receptor modulator	1000	Drug	Miscl.	2
O8	Megestrol acetate	Progestogen	1000	Drug	Miscl.	2
O9	Salinomycin	Ionophore	5000	Drug	Miscl.	2
O10	Idelalisib	PI3K inhibitor, p110δ-selective	1000	Drug	PI3K	2
O11	Afatinib	EGFR inhibitor	100	Drug	EGFR	2
O12	Sorafenib	B-Raf, FGFR-1, VEGFR-2 & -3, PDGFR-beta, KIT, and FLT3 inhib	100	Drug	VEGFR	2
O13	DMSO	DMSO	0	DMSO	Negative Controls	2
O14	Bosutinib	Abl, Src inhibitor	1000	Drug	Miscl.	2
O15	Nilotinib	Abl inhibitor	1000	Drug	Miscl.	2
O16	Osimertinib	EGFR(L858R/T790M) inhibitor	250	Drug	EGFR	2
O17	Pazopanib	VEGFR inhibitor	1000	Drug	VEGFR	2
O18	BzCl	BzCl	0	BzCl	Miscl.	2
O19	Lapatinib	HER2, EGFR inhibitor	100	Drug	EGFR	2
O20	Selumetinib	MEK1/2 inhibitor	1000	Drug	MEK1/2	2
O21	Cediranib	KDR/Flt/VEGFR inhibitor	100	Drug	VEGFR	2
O22	Ruboxistaurin	PKCbeta inhibitor	10000	Drug	Miscl.	2
O23	Masitinib	KIT inhibitor	1000	Drug	Miscl.	2
O24	cells	cells	None	cells	Negative Controls	2
P1	cells	cells	None	cells	Negative Controls	2
P2	Digoxin	Cardiac glycoside	1000	Drug	Miscl.	2
P3	DMSO	DMSO	0	DMSO	Negative Controls	2
P4	Flutamide	Nonsteroidal antiandrogen	10000	Drug	Miscl.	2
P5	Simvastatin	HMG CoA reductase inhibitor	10000	Drug	Miscl.	2
P6	Lovastatin	HMG-CoA reductase inhibitor	10000	Drug	Miscl.	2
P7	Clomifene	Selective estrogen receptor modulator	10000	Drug	Miscl.	2
P8	Megestrol acetate	Progestogen	10000	Drug	Miscl.	2
P9	Salinomycin	Ionophore	50000	Drug	Miscl.	2
P10	Idelalisib	PI3K inhibitor, p110δ-selective	10000	Drug	PI3K	2
P11	Afatinib	EGFR inhibitor	1000	Drug	EGFR	2
P12	Sorafenib	B-Raf, FGFR-1, VEGFR-2 & -3, PDGFR-beta, KIT, and FLT3 inhib	1000	Drug	VEGFR	2
P13	Vandetanib	VEGFR,EGFR, RET inhibitor	1000	Drug	VEGFR	2

P14	Bosutinib	Abl, Src inhibitor	10000	Drug	Miscl.	2
P15	Nilotinib	Abl inhibitor	10000	Drug	Miscl.	2
P16	Osimertinib	EGFR(L858R/T790M) inhibitor	2500	Drug	EGFR	2
P17	Pazopanib	VEGFR inhibitor	10000	Drug	VEGFR	2
P18	Hydroxyfasudil	ROCK, PKA, PKG, PRK inhibitor	19000	Drug	Miscl.	2
P19	Lapatinib	HER2, EGFR inhibitor	1000	Drug	EGFR	2
P20	Selumetinib	MEK1/2 inhibitor	10000	Drug	MEK1/2	2
P21	Cediranib	KDR/Flt/VEGFR inhibitor	1000	Drug	VEGFR	2
P22	DMSO	DMSO	0	DMSO	Negative Controls	2
P23	Masitinib	KIT inhibitor	10000	Drug	Miscl.	2
P24	BzCl	BzCl	0	BzCl	Miscl.	2
A1	cells	cells	None	cells	Negative Controls	3
A2	BzCl	BzCl	0	BzCl	Miscl.	3
A3	Cabozantinib	VEGFR2, Met, FLT3, Tie2, Kit and Ret inhibitor	1000	Drug	VEGFR	3
A4	Panobinostat	HDAC inhibitor	1000	Drug	HDAC	3
A5	Omacetaxine	Protein synthesis inhib (80 S ribosome)	10000	Drug	Miscl.	3
A6	Foretinib	MET, VEGFR2 inhibitor	1000	Drug	VEGFR	3
A7	Docetaxel	Mitotic inhibitor, taxane microtubule stabilizer	1000	Drug	Mitotic	3
A8	AZ 3146	Mps1 kinase (TTK) inhibitor	10000	Drug	Miscl.	3
A9	Quizartinib	FLT3 inhibitor	1000	Drug	Miscl.	3
A10	Fludarabine	Antimetabolite; Purine analog	10000	Drug	Miscl.	3
A11	Etoposide	Topoisomerase II inhibitor	10000	Drug	Topoisomerase	3
A12	Pemetrexed	Dihydrofolate reductase inhibitor	10000	Drug	Miscl.	3
A13	Deferoxamine	Iron chelator	10000	Drug	Miscl.	3
A14	Pravastatin	HMG CoA reductase inhibitor	10000	Drug	Miscl.	3
A15	Cisplatin	Platinum-based antineoplastic agent	100000	Drug	Miscl.	3
A16	Perifosine	AKT/PI3K inhibitor	2500	Drug	PI3K	3
A17	Fostamatinib	Syk inhibitor	2500	Drug	Miscl.	3
A18	Linifanib	VEGFR, PDGFR, CSF-1R, FLT3 inhibitor	1000	Drug	VEGFR	3
A19	Dinaciclib	CDK inhibitor	1000	Drug	CDK	3
A20	Pacritinib	FLT3/JAK2	10000	Drug	Miscl.	3
A21	Losmapimod	p38MAPK inhibitor	10000	Drug	Miscl.	3
A22	Gilteritinib	FLT3/AXL inhibitor	1000	Drug	Miscl.	3

A23	BzCl	BzCl	0	BzCl	Miscl.	3
A24	cells	cells	None	cells	Negative Controls	3
B1	cells	cells	None	cells	Negative Controls	3
B2	Ruxolitinib	JAK1&2 inhibitor	10000	Drug	Miscl.	3
B3	Cabozantinib	VEGFR2, Met, FLT3, Tie2, Kit and Ret inhibitor	100	Drug	VEGFR	3
B4	Panobinostat	HDAC inhibitor	100	Drug	HDAC	3
B5	Omacetaxine	Protein synthesis inhib (80 S ribosome)	1000	Drug	Miscl.	3
B6	Foretinib	MET, VEGFR2 inhibitor	100	Drug	VEGFR	3
B7	Docetaxel	Mitotic inhibitor, taxane microtubule stabilizer	100	Drug	Mitotic	3
B8	AZ 3146	Mps1 kinase (TTK) inhibitor	1000	Drug	Miscl.	3
B9	DMSO	DMSO	0	DMSO	Negative Controls	3
B10	Fludarabine	Antimetabolite; Purine analog	1000	Drug	Miscl.	3
B11	Etoposide	Topoisomerase II inhibitor	1000	Drug	Topoisomerase	3
B12	Pemetrexed	Dihydrofolate reductase inhibitor	1000	Drug	Miscl.	3
B13	Deferoxamine	Iron chelator	1000	Drug	Miscl.	3
B14	Pravastatin	HMG CoA reductase inhibitor	1000	Drug	Miscl.	3
B15	Cisplatin	Platinum-based antineoplastic agent	10000	Drug	Miscl.	3
B16	BzCl	BzCl	0	BzCl	Miscl.	3
B17	Fostamatinib	Syk inhibitor	250	Drug	Miscl.	3
B18	Linifanib	VEGFR, PDGFR, CSF-1R, FLT3 inhibitor	100	Drug	VEGFR	3
B19	Dinaciclib	CDK inhibitor	100	Drug	CDK	3
B20	Pacritinib	FLT3/JAK2	1000	Drug	Miscl.	3
B21	Losmapimod	p38MAPK inhibitor	1000	Drug	Miscl.	3
B22	Gilteritinib	FLT3/AXL inhibitor	100	Drug	Miscl.	3
B23	Abemaciclib	CDK4/6 inhibitor	2500	Drug	CDK	3
B24	cells	cells	None	cells	Negative Controls	3
C1	cells	cells	None	cells	Negative Controls	3
C2	Ruxolitinib	JAK1&2 inhibitor	1000	Drug	Miscl.	3
C3	Cabozantinib	VEGFR2, Met, FLT3, Tie2, Kit and Ret inhibitor	10	Drug	VEGFR	3
C4	Panobinostat	HDAC inhibitor	10	Drug	HDAC	3
C5	Omacetaxine	Protein synthesis inhib (80 S ribosome)	100	Drug	Miscl.	3
C6	Foretinib	MET, VEGFR2 inhibitor	10	Drug	VEGFR	3
C7	Docetaxel	Mitotic inhibitor, taxane microtubule stabilizer	10	Drug	Mitotic	3

C8	AZ 3146	Mps1 kinase (TTK) inhibitor	100	Drug	Miscl.	3
C9	Quizartinib	FLT3 inhibitor	100	Drug	Miscl.	3
C10	Fludarabine	Antimetabolite; Purine analog	100	Drug	Miscl.	3
C11	Etoposide	Topoisomerase II inhibitor	100	Drug	Topoisomerase	3
C12	DMSO	DMSO	0	DMSO	Negative Controls	3
C13	Deferoxamine	Iron chelator	100	Drug	Miscl.	3
C14	Pravastatin	HMG CoA reductase inhibitor	100	Drug	Miscl.	3
C15	Cisplatin	Platinum-based antineoplastic agent	1000	Drug	Miscl.	3
C16	Perifosine	AKT/PI3K inhibitor	250	Drug	PI3K	3
C17	Fostamatinib	Syk inhibitor	25	Drug	Miscl.	3
C18	Linifanib	VEGFR, PDGFR, CSF-1R, FLT3 inhibitor	10	Drug	VEGFR	3
C19	Dinaciclib	CDK inhibitor	10	Drug	CDK	3
C20	DMSO	DMSO	0	DMSO	Negative Controls	3
C21	Losmapimod	p38MAPK inhibitor	100	Drug	Miscl.	3
C22	Gilteritinib	FLT3/AXL inhibitor	10	Drug	Miscl.	3
C23	Abemaciclib	CDK4/6 inhibitor	250	Drug	CDK	3
C24	BzCl	BzCl	0	BzCl	Miscl.	3
D1	cells	cells	None	cells	Negative Controls	3
D2	Ruxolitinib	JAK1&2 inhibitor	100	Drug	Miscl.	3
D3	Cabozantinib	VEGFR2, Met, FLT3, Tie2, Kit and Ret inhibitor	1	Drug	VEGFR	3
D4	Panobinostat	HDAC inhibitor	1	Drug	HDAC	3
D5	DMSO	DMSO	0	DMSO	Negative Controls	3
D6	Foretinib	MET, VEGFR2 inhibitor	1	Drug	VEGFR	3
D7	Docetaxel	Mitotic inhibitor, taxane microtubule stabilizer	1	Drug	Mitotic	3
D8	AZ 3146	Mps1 kinase (TTK) inhibitor	10	Drug	Miscl.	3
D9	Quizartinib	FLT3 inhibitor	10	Drug	Miscl.	3
D10	Fludarabine	Antimetabolite; Purine analog	10	Drug	Miscl.	3
D11	Etoposide	Topoisomerase II inhibitor	10	Drug	Topoisomerase	3
D12	Pemetrexed	Dihydrofolate reductase inhibitor	100	Drug	Miscl.	3
D13	Deferoxamine	Iron chelator	10	Drug	Miscl.	3
D14	Pravastatin	HMG CoA reductase inhibitor	10	Drug	Miscl.	3
D15	Cisplatin	Platinum-based antineoplastic agent	100	Drug	Miscl.	3
D16	Perifosine	AKT/PI3K inhibitor	25	Drug	PI3K	3

D17	Fostamatinib	Syk inhibitor	2.5	Drug	Miscl.	3
D18	Linifanib	VEGFR, PDGFR, CSF-1R, FLT3 inhibitor	1	Drug	VEGFR	3
D19	Dinaciclib	CDK inhibitor	1	Drug	CDK	3
D20	Pacritinib	FLT3/JAK2	100	Drug	Miscl.	3
D21	Losmapimod	p38MAPK inhibitor	10	Drug	Miscl.	3
D22	Gilteritinib	FLT3/AXL inhibitor	1	Drug	Miscl.	3
D23	Abemaciclib	CDK4/6 inhibitor	25	Drug	CDK	3
D24	cells	cells	None	cells	Negative Controls	3
E1	cells	cells	None	cells	Negative Controls	3
E2	Ruxolitinib	JAK1&2 inhibitor	10	Drug	Miscl.	3
E3	Cabozantinib	VEGFR2, Met, FLT3, Tie2, Kit and Ret inhibitor	0.1	Drug	VEGFR	3
E4	Panobinostat	HDAC inhibitor	0.1	Drug	HDAC	3
E5	Omacetaxine	Protein synthesis inhib (80 S ribosome)	10	Drug	Miscl.	3
E6	Foretinib	MET, VEGFR2 inhibitor	0.1	Drug	VEGFR	3
E7	Docetaxel	Mitotic inhibitor, taxane microtubule stabilizer	0.1	Drug	Mitotic	3
E8	AZ 3146	Mps1 kinase (TTK) inhibitor	1	Drug	Miscl.	3
E9	Quizartinib	FLT3 inhibitor	1	Drug	Miscl.	3
E10	Fludarabine	Antimetabolite; Purine analog	1	Drug	Miscl.	3
E11	Etoposide	Topoisomerase II inhibitor	1	Drug	Topoisomerase	3
E12	Pemetrexed	Dihydrofolate reductase inhibitor	10	Drug	Miscl.	3
E13	Deferoxamine	Iron chelator	1	Drug	Miscl.	3
E14	Pravastatin	HMG CoA reductase inhibitor	1	Drug	Miscl.	3
E15	DMSO	DMSO	0	DMSO	Negative Controls	3
E16	Perifosine	AKT/PI3K inhibitor	2.5	Drug	PI3K	3
E17	Fostamatinib	Syk inhibitor	0.25	Drug	Miscl.	3
E18	Linifanib	VEGFR, PDGFR, CSF-1R, FLT3 inhibitor	0.1	Drug	VEGFR	3
E19	Dinaciclib	CDK inhibitor	0.1	Drug	CDK	3
E20	Pacritinib	FLT3/JAK2	10	Drug	Miscl.	3
E21	Losmapimod	p38MAPK inhibitor	1	Drug	Miscl.	3
E22	Gilteritinib	FLT3/AXL inhibitor	0.1	Drug	Miscl.	3
E23	Abemaciclib	CDK4/6 inhibitor	2.5	Drug	CDK	3
E24	cells	cells	None	cells	Negative Controls	3
F1	cells	cells	None	cells	Negative Controls	3

F2	Ruxolitinib	JAK1&2 inhibitor	1	Drug	Miscl.	3
F3	Sunitinib	Broad TK inhibitor	1000	Drug	Miscl.	3
F4	Venetoclax	Bcl-2-selective inhibitor	1000	Drug	Miscl.	3
F5	Omacetaxine	Protein synthesis inhib (80 S ribosome)	1	Drug	Miscl.	3
F6	AZD1480	JAK1/2, FGFR inhibitor	1000	Drug	Miscl.	3
F7	Quisinostat	HDAC inhibitor	1000	Drug	HDAC	3
F8	UNC0642	G9a/GLP inhibitor	10000	Drug	Miscl.	3
F9	Quizartinib	FLT3 inhibitor	0.1	Drug	Miscl.	3
F10	BzCl	BzCl	0	BzCl	Miscl.	3
F11	Navitoclax	Bcl-2/Bcl-xL inhibitor	10000	Drug	Miscl.	3
F12	Pemetrexed	Dihydrofolate reductase inhibitor	1	Drug	Miscl.	3
F13	Plerixafor	CXCR4 antagonist	10000	Drug	Miscl.	3
F14	Metformin	AMPK activator	100000	Drug	Miscl.	3
F15	Cisplatin	Platinum-based antineoplastic agent	10	Drug	Miscl.	3
F16	Perifosine	AKT/PI3K inhibitor	0.25	Drug	PI3K	3
F17	Miltefosine	Antimicrobial, inhibits PI3K/AKT	100000	Drug	PI3K	3
F18	Brivanib	VEGFR inhibitor	1000	Drug	VEGFR	3
F19	Duvelisib	PI3K inhibitor	500	Drug	PI3K	3
F20	Pacritinib	FLT3/JAK2	1	Drug	Miscl.	3
F21	Rociletinib	EGFR(L858R/T790M) inhibitor	10000	Drug	EGFR	3
F22	Peficitinb	JAK inhibitor	2500	Drug	Miscl.	3
F23	Abemaciclib	CDK4/6 inhibitor	0.25	Drug	CDK	3
F24	cells	cells	None	cells	Negative Controls	3
G1	cells	cells	None	cells	Negative Controls	3
G2	Tofacitinib	JAK3, JAK2(V617F) inhibitor	5000	Drug	Miscl.	3
G3	Sunitinib	Broad TK inhibitor	100	Drug	Miscl.	3
G4	Venetoclax	Bcl-2-selective inhibitor	100	Drug	Miscl.	3
G5	Momelotinib	JAK1 & 2 inhibitor	10000	Drug	Miscl.	3
G6	BzCl	BzCl	0	BzCl	Miscl.	3
G7	Quisinostat	HDAC inhibitor	100	Drug	HDAC	3
G8	UNC0642	G9a/GLP inhibitor	1000	Drug	Miscl.	3
G9	Daunorubicin	Topoisomerase II inhibitor	1000	Drug	Topoisomerase	3
G10	Teniposide	Topoisomerase II inhibitor	10000	Drug	Topoisomerase	3

G11	Navitoclax	Bcl-2/Bcl-xL inhibitor	1000	Drug	Miscl.	3
G12	Valproic acid	HDAC inhibitor	1000000	Drug	HDAC	3
G13	Plerixafor	CXCR4 antagonist	1000	Drug	Miscl.	3
G14	Metformin	AMPK activator	10000	Drug	Miscl.	3
G15	Oxaliplatin	Platinum-based antineoplastic agent	100000	Drug	Miscl.	3
G16	BMS863233	Cdc7 inhibitor	10000	Drug	Miscl.	3
G17	Miltefosine	Antimicrobial, inhibits PI3K/AKT	10000	Drug	PI3K	3
G18	Brivanib	VEGFR inhibitor	100	Drug	VEGFR	3
G19	Duvelisib	PI3K inhibitor	50	Drug	PI3K	3
G20	Neratinib	EGFR inhibitor	1000	Drug	EGFR	3
G21	Rociletinib	EGFR(L858R/T790M) inhibitor	1000	Drug	EGFR	3
G22	Peficitinb	JAK inhibitor	250	Drug	Miscl.	3
G23	Ensartinib	ALK inhibitor	1000	Drug	Miscl.	3
G24	cells	cells	None	cells	Negative Controls	3
H1	cells	cells	None	cells	Negative Controls	3
H2	Tofacitinib	JAK3, JAK2(V617F) inhibitor	500	Drug	Miscl.	3
H3	Sunitinib	Broad TK inhibitor	10	Drug	Miscl.	3
H4	Venetoclax	Bcl-2-selective inhibitor	10	Drug	Miscl.	3
H5	Momelotinib	JAK1 & 2 inhibitor	1000	Drug	Miscl.	3
H6	AZD1480	JAK1/2, FGFR inhibitor	100	Drug	Miscl.	3
H7	Quisinostat	HDAC inhibitor	10	Drug	HDAC	3
H8	UNC0642	G9a/GLP inhibitor	100	Drug	Miscl.	3
H9	Daunorubicin	Topoisomerase II inhibitor	100	Drug	Topoisomerase	3
H10	Teniposide	Topoisomerase II inhibitor	1000	Drug	Topoisomerase	3
H11	Navitoclax	Bcl-2/Bcl-xL inhibitor	100	Drug	Miscl.	3
H12	Valproic acid	HDAC inhibitor	100000	Drug	HDAC	3
H13	Plerixafor	CXCR4 antagonist	100	Drug	Miscl.	3
H14	Metformin	AMPK activator	1000	Drug	Miscl.	3
H15	Oxaliplatin	Platinum-based antineoplastic agent	10000	Drug	Miscl.	3
H16	BMS863233	Cdc7 inhibitor	1000	Drug	Miscl.	3
H17	Miltefosine	Antimicrobial, inhibits PI3K/AKT	1000	Drug	PI3K	3
H18	Brivanib	VEGFR inhibitor	10	Drug	VEGFR	3
H19	DMSO	DMSO	0	DMSO	Negative Controls	3

H20	Neratinib	EGFR inhibitor	100	Drug	EGFR	3
H21	Rociletinib	EGFR(L858R/T790M) inhibitor	100	Drug	EGFR	3
H22	Peficitinb	JAK inhibitor	25	Drug	Miscl.	3
H23	Ensartinib	ALK inhibitor	100	Drug	Miscl.	3
H24	cells	cells	None	cells	Negative Controls	3
I1	cells	cells	None	cells	Negative Controls	3
I2	Tofacitinib	JAK3, JAK2(V617F) inhibitor	50	Drug	Miscl.	3
I3	Sunitinib	Broad TK inhibitor	1	Drug	Miscl.	3
I4	Venetoclax	Bcl-2-selective inhibitor	1	Drug	Miscl.	3
I5	Momelotinib	JAK1 & 2 inhibitor	100	Drug	Miscl.	3
I6	AZD1480	JAK1/2, FGFR inhibitor	10	Drug	Miscl.	3
I7	Quisinostat	HDAC inhibitor	1	Drug	HDAC	3
I8	DMSO	DMSO	0	DMSO	Negative Controls	3
I9	Daunorubicin	Topoisomerase II inhibitor	10	Drug	Topoisomerase	3
I10	Teniposide	Topoisomerase II inhibitor	100	Drug	Topoisomerase	3
I11	Navitoclax	Bcl-2/Bcl-xL inhibitor	10	Drug	Miscl.	3
I12	Valproic acid	HDAC inhibitor	10000	Drug	HDAC	3
I13	Plerixafor	CXCR4 antagonist	10	Drug	Miscl.	3
I14	Metformin	AMPK activator	100	Drug	Miscl.	3
I15	Oxaliplatin	Platinum-based antineoplastic agent	1000	Drug	Miscl.	3
I16	BMS863233	Cdc7 inhibitor	100	Drug	Miscl.	3
I17	Miltefosine	Antimicrobial, inhibits PI3K/AKT	100	Drug	PI3K	3
I18	Brivanib	VEGFR inhibitor	1	Drug	VEGFR	3
I19	Duvelisib	PI3K inhibitor	5	Drug	PI3K	3
I20	Neratinib	EGFR inhibitor	10	Drug	EGFR	3
I21	Rociletinib	EGFR(L858R/T790M) inhibitor	10	Drug	EGFR	3
I22	Peficitinb	JAK inhibitor	2.5	Drug	Miscl.	3
I23	Ensartinib	ALK inhibitor	10	Drug	Miscl.	3
I24	cells	cells	None	cells	Negative Controls	3
J1	cells	cells	None	cells	Negative Controls	3
J2	Tofacitinib	JAK3, JAK2(V617F) inhibitor	5	Drug	Miscl.	3
J3	Sunitinib	Broad TK inhibitor	0.1	Drug	Miscl.	3
J4	Venetoclax	Bcl-2-selective inhibitor	0.1	Drug	Miscl.	3

J5	Momelotinib	JAK1 & 2 inhibitor	10	Drug	Miscl.	3
J6	AZD1480	JAK1/2, FGFR inhibitor	1	Drug	Miscl.	3
J7	Quisinostat	HDAC inhibitor	0.1	Drug	HDAC	3
J8	UNC0642	G9a/GLP inhibitor	10	Drug	Miscl.	3
J9	Daunorubicin	Topoisomerase II inhibitor	1	Drug	Topoisomerase	3
J10	Teniposide	Topoisomerase II inhibitor	10	Drug	Topoisomerase	3
J11	Navitoclax	Bcl-2/Bcl-xL inhibitor	1	Drug	Miscl.	3
J12	Valproic acid	HDAC inhibitor	1000	Drug	HDAC	3
J13	Plerixafor	CXCR4 antagonist	1	Drug	Miscl.	3
J14	BzCl	BzCl	0	BzCl	Miscl.	3
J15	Oxaliplatin	Platinum-based antineoplastic agent	100	Drug	Miscl.	3
J16	BMS863233	Cdc7 inhibitor	10	Drug	Miscl.	3
J17	Miltefosine	Antimicrobial, inhibits PI3K/AKT	10	Drug	PI3K	3
J18	Brivanib	VEGFR inhibitor	0.1	Drug	VEGFR	3
J19	Duvelisib	PI3K inhibitor	0.5	Drug	PI3K	3
J20	Neratinib	EGFR inhibitor	1	Drug	EGFR	3
J21	Rociletinib	EGFR(L858R/T790M) inhibitor	1	Drug	EGFR	3
J22	Peficitinb	JAK inhibitor	0.25	Drug	Miscl.	3
J23	Ensartinib	ALK inhibitor	1	Drug	Miscl.	3
J24	cells	cells	None	cells	Negative Controls	3
K1	cells	cells	None	cells	Negative Controls	3
K2	Tofacitinib	JAK3, JAK2(V617F) inhibitor	0.5	Drug	Miscl.	3
K3	Belinostat	HDAC inhibitor	1	Drug	HDAC	3
K4	Canertinib	pan-HER inhibitor	1	Drug	EGFR	3
K5	Momelotinib	JAK1 & 2 inhibitor	1	Drug	Miscl.	3
K6	AZD1480	JAK1/2, FGFR inhibitor	0.1	Drug	Miscl.	3
K7	Idarubicin	Topoisomerase II inhibitor	0.1	Drug	Topoisomerase	3
K8	UNC0642	G9a/GLP inhibitor	1	Drug	Miscl.	3
K9	Daunorubicin	Topoisomerase II inhibitor	0.1	Drug	Topoisomerase	3
K10	Teniposide	Topoisomerase II inhibitor	1	Drug	Topoisomerase	3
K11	Chloroquine	Antimalaria agent; chemo/radio sensitizer	10	Drug	Miscl.	3
K12	Valproic acid	HDAC inhibitor	100	Drug	HDAC	3
K13	Mepacrine	Unclear. PLA2 inhibitor. NF-kB inhibitor, p53 activator	5	Drug	Miscl.	3

K14	Metformin	AMPK activator	10	Drug	Miscl.	3
K15	Oxaliplatin	Platinum-based antineoplastic agent	10	Drug	Miscl.	3
K16	BMS863233	Cdc7 inhibitor	1	Drug	Miscl.	3
K17	Palbociclib	CDK4/6 inhibitor	1	Drug	CDK	3
K18	Dacomitinib	pan-HER inhibitor	0.1	Drug	EGFR	3
K19	Duvelisib	PI3K inhibitor	0.05	Drug	PI3K	3
K20	Neratinib	EGFR inhibitor	0.1	Drug	EGFR	3
K21	BzCl	BzCl	0	BzCl	Miscl.	3
K22	Decernotinib	JAK3 inhibitor	1	Drug	Miscl.	3
K23	Ensartinib	ALK inhibitor	0.1	Drug	Miscl.	3
K24	cells	cells	None	cells	Negative Controls	3
L1	cells	cells	None	cells	Negative Controls	3
L2	Ponatinib	Broad TK inhibitor	0.1	Drug	Miscl.	3
L3	Belinostat	HDAC inhibitor	10	Drug	HDAC	3
L4	Canertinib	pan-HER inhibitor	10	Drug	EGFR	3
L5	AZD7762	Chk1 inhibitor	0.1	Drug	Miscl.	3
L6	Doxorubicin	Topoisomerase II inhibitor	0.1	Drug	Topoisomerase	3
L7	Idarubicin	Topoisomerase II inhibitor	1	Drug	Topoisomerase	3
L8	Pictilisib	PI3K inhibitor, pan-class I	1	Drug	PI3K	3
L9	Valrubicin	Topoisomerase II inhibitor	0.5	Drug	Topoisomerase	3
L10	Mitoxantrone	Topoisomerase II inhibitor	0.1	Drug	Topoisomerase	3
L11	Chloroquine	Antimalaria agent; chemo/radio sensitizer	100	Drug	Miscl.	3
L12	Arsenic(III) oxide	Thioredoxin reductase inhibitor; cytotoxic chemotherapeutic	0.25	Drug	Miscl.	3
L13	Mepacrine	Unclear. PLA2 inhibitor. NF-kB inhibitor, p53 activator	50	Drug	Miscl.	3
L14	Carboplatin	Platinum-based antineoplastic agent	10	Drug	Miscl.	3
L15	1-methyl-D-tryptophan	Indolamine 2,3-dioxygenase 1 and 2 inhibitor	0.5	Drug	Miscl.	3
L16	Pixantrone	topoisomerase II inhibitor	1	Drug	Topoisomerase	3
L17	DMSO	DMSO	0	DMSO	Negative Controls	3
L18	Dacomitinib	pan-HER inhibitor	1	Drug	EGFR	3
L19	Ribociclib	CDK4/6 inhibitor	1	Drug	CDK	3
L20	Pexidartinib	KIT, CSF1R, FLT3 inhibitor	1	Drug	Miscl.	3
L21	Taselisib	PI3K alpha, delta, (gamma) selective inhibitor	0.1	Drug	PI3K	3
L22	Decernotinib	JAK3 inhibitor	10	Drug	Miscl.	3

L23	Upadacitinib	JAK1-selective inhibitor	1	Drug	Miscl.	3
L24	cells	cells	None	cells	Negative Controls	3
M1	cells	cells	None	cells	Negative Controls	3
M2	Ponatinib	Broad TK inhibitor	1	Drug	Miscl.	3
M3	Belinostat	HDAC inhibitor	100	Drug	HDAC	3
M4	BzCl	BzCl	0	BzCl	Miscl.	3
M5	AZD7762	Chk1 inhibitor	1	Drug	Miscl.	3
M6	Doxorubicin	Topoisomerase II inhibitor	1	Drug	Topoisomerase	3
M7	Idarubicin	Topoisomerase II inhibitor	10	Drug	Topoisomerase	3
M8	Pictilisib	PI3K inhibitor, pan-class I	10	Drug	PI3K	3
M9	Valrubicin	Topoisomerase II inhibitor	5	Drug	Topoisomerase	3
M10	Mitoxantrone	Topoisomerase II inhibitor	1	Drug	Topoisomerase	3
M11	Chloroquine	Antimalaria agent; chemo/radio sensitizer	1000	Drug	Miscl.	3
M12	Arsenic(III) oxide	Thioredoxin reductase inhibitor; cytotoxic chemotherapeutic	2.5	Drug	Miscl.	3
M13	Mepacrine	Unclear. PLA2 inhibitor. NF-kB inhibitor, p53 activator	500	Drug	Miscl.	3
M14	Carboplatin	Platinum-based antineoplastic agent	100	Drug	Miscl.	3
M15	1-methyl-D-tryptophan	Indolamine 2,3-dioxygenase 1 and 2 inhibitor	5	Drug	Miscl.	3
M16	Pixantrone	topoisomerase II inhibitor	10	Drug	Topoisomerase	3
M17	Palbociclib	CDK4/6 inhibitor	10	Drug	CDK	3
M18	Dacomitinib	pan-HER inhibitor	10	Drug	EGFR	3
M19	Ribociclib	CDK4/6 inhibitor	10	Drug	CDK	3
M20	Pexidartinib	KIT, CSF1R, FLT3 inhibitor	10	Drug	Miscl.	3
M21	Taselisib	PI3K alpha, delta, (gamma) selective inhibitor	1	Drug	PI3K	3
M22	Decernotinib	JAK3 inhibitor	100	Drug	Miscl.	3
M23	Upadacitinib	JAK1-selective inhibitor	10	Drug	Miscl.	3
M24	cells	cells	None	cells	Negative Controls	3
N1	cells	cells	None	cells	Negative Controls	3
N2	Ponatinib	Broad TK inhibitor	10	Drug	Miscl.	3
N3	Belinostat	HDAC inhibitor	1000	Drug	HDAC	3
N4	Canertinib	pan-HER inhibitor	100	Drug	EGFR	3
N5	AZD7762	Chk1 inhibitor	10	Drug	Miscl.	3
N6	Doxorubicin	Topoisomerase II inhibitor	10	Drug	Topoisomerase	3
N7	DMSO	DMSO	0	DMSO	Negative Controls	3

N8	Pictilisib	PI3K inhibitor, pan-class I	100	Drug	PI3K	3
N9	Valrubicin	Topoisomerase II inhibitor	50	Drug	Topoisomerase	3
N10	Mitoxantrone	Topoisomerase II inhibitor	10	Drug	Topoisomerase	3
N11	BzCl	BzCl	0	BzCl	Miscl.	3
N12	Arsenic(III) oxide	Thioredoxin reductase inhibitor; cytotoxic chemotherapeutic	25	Drug	Miscl.	3
N13	Mepacrine	Unclear. PLA2 inhibitor. NF-kB inhibitor, p53 activator	5000	Drug	Miscl.	3
N14	Carboplatin	Platinum-based antineoplastic agent	1000	Drug	Miscl.	3
N15	1-methyl-D-tryptophan	Indolamine 2,3-dioxygenase 1 and 2 inhibitor	50	Drug	Miscl.	3
N16	Pixantrone	topoisomerase II inhibitor	100	Drug	Topoisomerase	3
N17	Palbociclib	CDK4/6 inhibitor	100	Drug	CDK	3
N18	Dacomitinib	pan-HER inhibitor	100	Drug	EGFR	3
N19	Ribociclib	CDK4/6 inhibitor	100	Drug	CDK	3
N20	Pexidartinib	KIT, CSF1R, FLT3 inhibitor	100	Drug	Miscl.	3
N21	Taselisib	PI3K alpha, delta, (gamma) selective inhibitor	10	Drug	PI3K	3
N22	Decernotinib	JAK3 inhibitor	1000	Drug	Miscl.	3
N23	Upadacitinib	JAK1-selective inhibitor	100	Drug	Miscl.	3
N24	cells	cells	None	cells	Negative Controls	3
O1	cells	cells	None	cells	Negative Controls	3
O2	Ponatinib	Broad TK inhibitor	100	Drug	Miscl.	3
O3	Belinostat	HDAC inhibitor	10000	Drug	HDAC	3
O4	Canertinib	pan-HER inhibitor	1000	Drug	EGFR	3
O5	AZD7762	Chk1 inhibitor	100	Drug	Miscl.	3
O6	Doxorubicin	Topoisomerase II inhibitor	100	Drug	Topoisomerase	3
O7	Idarubicin	Topoisomerase II inhibitor	100	Drug	Topoisomerase	3
O8	Pictilisib	PI3K inhibitor, pan-class I	1000	Drug	PI3K	3
O9	Valrubicin	Topoisomerase II inhibitor	500	Drug	Topoisomerase	3
O10	Mitoxantrone	Topoisomerase II inhibitor	100	Drug	Topoisomerase	3
O11	Chloroquine	Antimalaria agent; chemo/radio sensitizer	10000	Drug	Miscl.	3
O12	Arsenic(III) oxide	Thioredoxin reductase inhibitor; cytotoxic chemotherapeutic	250	Drug	Miscl.	3
O13	DMSO	DMSO	0	DMSO	Negative Controls	3
O14	Carboplatin	Platinum-based antineoplastic agent	10000	Drug	Miscl.	3
O15	1-methyl-D-tryptophan	Indolamine 2,3-dioxygenase 1 and 2 inhibitor	500	Drug	Miscl.	3
O16	Pixantrone	topoisomerase II inhibitor	1000	Drug	Topoisomerase	3

O17	Palbociclib	CDK4/6 inhibitor	1000	Drug	CDK	3
O18	BzCl	BzCl	0	BzCl	Miscl.	3
O19	Ribociclib	CDK4/6 inhibitor	1000	Drug	CDK	3
O20	Pexidartinib	KIT, CSF1R, FLT3 inhibitor	1000	Drug	Miscl.	3
O21	Taselisib	PI3K alpha, delta, (gamma) selective inhibitor	100	Drug	PI3K	3
O22	Decernotinib	JAK3 inhibitor	10000	Drug	Miscl.	3
O23	Upadacitinib	JAK1-selective inhibitor	1000	Drug	Miscl.	3
O24	cells	cells	None	cells	Negative Controls	3
P1	cells	cells	None	cells	Negative Controls	3
P2	Ponatinib	Broad TK inhibitor	1000	Drug	Miscl.	3
P3	DMSO	DMSO	0	DMSO	Negative Controls	3
P4	Canertinib	pan-HER inhibitor	10000	Drug	EGFR	3
P5	AZD7762	Chk1 inhibitor	1000	Drug	Miscl.	3
P6	Doxorubicin	Topoisomerase II inhibitor	1000	Drug	Topoisomerase	3
P7	Idarubicin	Topoisomerase II inhibitor	1000	Drug	Topoisomerase	3
P8	Pictilisib	PI3K inhibitor, pan-class I	10000	Drug	PI3K	3
P9	Valrubicin	Topoisomerase II inhibitor	5000	Drug	Topoisomerase	3
P10	Mitoxantrone	Topoisomerase II inhibitor	1000	Drug	Topoisomerase	3
P11	Chloroquine	Antimalaria agent; chemo/radio sensitizer	100000	Drug	Miscl.	3
P12	Arsenic(III) oxide	Thioredoxin reductase inhibitor; cytotoxic chemotherapeutic	2500	Drug	Miscl.	3
P13	Mepacrine	Unclear. PLA2 inhibitor. NF-kB inhibitor, p53 activator	50000	Drug	Miscl.	3
P14	Carboplatin	Platinum-based antineoplastic agent	100000	Drug	Miscl.	3
P15	1-methyl-D-tryptophan	Indolamine 2,3-dioxygenase 1 and 2 inhibitor	5000	Drug	Miscl.	3
P16	Pixantrone	topoisomerase II inhibitor	10000	Drug	Topoisomerase	3
P17	Palbociclib	CDK4/6 inhibitor	10000	Drug	CDK	3
P18	Dacomitinib	pan-HER inhibitor	1000	Drug	EGFR	3
P19	Ribociclib	CDK4/6 inhibitor	10000	Drug	CDK	3
P20	Pexidartinib	KIT, CSF1R, FLT3 inhibitor	10000	Drug	Miscl.	3
P21	Taselisib	PI3K alpha, delta, (gamma) selective inhibitor	1000	Drug	PI3K	3
P22	DMSO	DMSO	0	DMSO	Negative Controls	3
P23	Upadacitinib	JAK1-selective inhibitor	10000	Drug	Miscl.	3
P24	BzCl	BzCl	0	BzCl	Miscl.	3
A1	cells	cells	None	cells	Negative Controls	4

A2	BzCl	BzCl	0	BzCl	Miscl.	4
A3	Brigatinib	ALK inhibitor, including gatekeeper mutant ALK	1000	Drug	Miscl.	4
A4	SNS-032	CDK inhibitor	10000	Drug	CDK	4
A5	Gandotinib	JAK2 inhibitor	10000	Drug	Miscl.	4
A6	Vistusertib	mTOR inhibitor, ATP-competitive	10000	Drug	Miscl.	4
A7	Fedratinib	JAK2-selective inhibitor	10000	Drug	Miscl.	4
A8	Milciclib	CDK2 inhibitor	10000	Drug	CDK	4
A9	BI 2536	PLK1 inhibitor	1000	Drug	Miscl.	4
A10	Binimetinib	MEK1/2 inhibitor	1000	Drug	MEK1/2	4
A11	Rabusertib	Chk1 inhibitor	1000	Drug	Miscl.	4
A12	ENMD-2076	pan-Aurora, VEGFR inhibitor	10000	Drug	VEGFR	4
A13	PD0325901	MEK1/2 inhibitor	1000	Drug	MEK1/2	4
A14	Capmatinib	MET inhibitor	1000	Drug	Miscl.	4
A15	Golvatinib	MET, VEGFR2 inhibitor	2500	Drug	VEGFR	4
A16	CEP-32496	BRAF inhibitor	10000	Drug	Miscl.	4
A17	Silmitasertib	CSNK2A1 inhibitor	10000	Drug	Miscl.	4
A18	Encorafenib	B-Raf(V600E) inhibitor	1000	Drug	Miscl.	4
A19	Alpelisib	PI3Kalpha inhibitor	2500	Drug	PI3K	4
A20	Motesanib	VEGFR, PDGFR, Ret, Kit inhibitor	10000	Drug	VEGFR	4
A21	OTS167	MELK inhibitor	1000	Drug	Miscl.	4
A22	AZD1775	Wee1 inhibitor	10000	Drug	Miscl.	4
A23	BzCl	BzCl	0	BzCl	Miscl.	4
A24	cells	cells	None	cells	Negative Controls	4
B1	cells	cells	None	cells	Negative Controls	4
B2	Radotinib	ABL, PDGFR inhibitor	10000	Drug	Miscl.	4
B3	Brigatinib	ALK inhibitor, including gatekeeper mutant ALK	100	Drug	Miscl.	4
B4	SNS-032	CDK inhibitor	1000	Drug	CDK	4
B5	Gandotinib	JAK2 inhibitor	1000	Drug	Miscl.	4
B6	Vistusertib	mTOR inhibitor, ATP-competitive	1000	Drug	Miscl.	4
B7	Fedratinib	JAK2-selective inhibitor	1000	Drug	Miscl.	4
B8	Milciclib	CDK2 inhibitor	1000	Drug	CDK	4
B9	DMSO	DMSO	0	DMSO	Negative Controls	4
B10	Binimetinib	MEK1/2 inhibitor	100	Drug	MEK1/2	4

B11	Rabusertib	Chk1 inhibitor	100	Drug	Miscl.	4
B12	ENMD-2076	pan-Aurora, VEGFR inhibitor	1000	Drug	VEGFR	4
B13	PD0325901	MEK1/2 inhibitor	100	Drug	MEK1/2	4
B14	Capmatinib	MET inhibitor	100	Drug	Miscl.	4
B15	Golvatinib	MET, VEGFR2 inhibitor	250	Drug	VEGFR	4
B16	BzCl	BzCl	0	BzCl	Miscl.	4
B17	Silmitasertib	CSNK2A1 inhibitor	1000	Drug	Miscl.	4
B18	Encorafenib	B-Raf(V600E) inhibitor	100	Drug	Miscl.	4
B19	Alpelisib	PI3Kalpha inhibitor	250	Drug	PI3K	4
B20	Motesanib	VEGFR, PDGFR, Ret, Kit inhibitor	1000	Drug	VEGFR	4
B21	OTS167	MELK inhibitor	100	Drug	Miscl.	4
B22	AZD1775	Wee1 inhibitor	1000	Drug	Miscl.	4
B23	Ralimetinib	p38MAPK inhibitor	10000	Drug	Miscl.	4
B24	cells	cells	None	cells	Negative Controls	4
C1	cells	cells	None	cells	Negative Controls	4
C2	Radotinib	ABL, PDGFR inhibitor	1000	Drug	Miscl.	4
C3	Brigatinib	ALK inhibitor, including gatekeeper mutant ALK	10	Drug	Miscl.	4
C4	SNS-032	CDK inhibitor	100	Drug	CDK	4
C5	Gandotinib	JAK2 inhibitor	100	Drug	Miscl.	4
C6	Vistusertib	mTOR inhibitor, ATP-competitive	100	Drug	Miscl.	4
C7	Fedratinib	JAK2-selective inhibitor	100	Drug	Miscl.	4
C8	Milciclib	CDK2 inhibitor	100	Drug	CDK	4
C9	BI 2536	PLK1 inhibitor	100	Drug	Miscl.	4
C10	Binimetinib	MEK1/2 inhibitor	10	Drug	MEK1/2	4
C11	Rabusertib	Chk1 inhibitor	10	Drug	Miscl.	4
C12	DMSO	DMSO	0	DMSO	Negative Controls	4
C13	PD0325901	MEK1/2 inhibitor	10	Drug	MEK1/2	4
C14	Capmatinib	MET inhibitor	10	Drug	Miscl.	4
C15	Golvatinib	MET, VEGFR2 inhibitor	25	Drug	VEGFR	4
C16	CEP-32496	BRAF inhibitor	1000	Drug	Miscl.	4
C17	Silmitasertib	CSNK2A1 inhibitor	100	Drug	Miscl.	4
C18	Encorafenib	B-Raf(V600E) inhibitor	10	Drug	Miscl.	4
C19	Alpelisib	PI3Kalpha inhibitor	25	Drug	PI3K	4

C20	DMSO	DMSO	0	DMSO	Negative Controls	4
C21	OTS167	MELK inhibitor	10	Drug	Miscl.	4
C22	AZD1775	Wee1 inhibitor	100	Drug	Miscl.	4
C23	Ralimetinib	p38MAPK inhibitor	1000	Drug	Miscl.	4
C24	cells	cells	None	cells	Negative Controls	4
D1	cells	cells	None	cells	Negative Controls	4
D2	Radotinib	ABL, PDGFR inhibitor	100	Drug	Miscl.	4
D3	Brigatinib	ALK inhibitor, including gatekeeper mutant ALK	1	Drug	Miscl.	4
D4	SNS-032	CDK inhibitor	10	Drug	CDK	4
D5	DMSO	DMSO	0	DMSO	Negative Controls	4
D6	Vistusertib	mTOR inhibitor, ATP-competitive	10	Drug	Miscl.	4
D7	Fedratinib	JAK2-selective inhibitor	10	Drug	Miscl.	4
D8	Milciclib	CDK2 inhibitor	10	Drug	CDK	4
D9	BI 2536	PLK1 inhibitor	10	Drug	Miscl.	4
D10	Binimetinib	MEK1/2 inhibitor	1	Drug	MEK1/2	4
D11	Rabusertib	Chk1 inhibitor	1	Drug	Miscl.	4
D12	ENMD-2076	pan-Aurora, VEGFR inhibitor	100	Drug	VEGFR	4
D13	PD0325901	MEK1/2 inhibitor	1	Drug	MEK1/2	4
D14	Capmatinib	MET inhibitor	1	Drug	Miscl.	4
D15	Golvatinib	MET, VEGFR2 inhibitor	2.5	Drug	VEGFR	4
D16	CEP-32496	BRAF inhibitor	100	Drug	Miscl.	4
D17	Silmitasertib	CSNK2A1 inhibitor	10	Drug	Miscl.	4
D18	Encorafenib	B-Raf(V600E) inhibitor	1	Drug	Miscl.	4
D19	Alpelisib	PI3Kalpha inhibitor	2.5	Drug	PI3K	4
D20	Motesanib	VEGFR, PDGFR, Ret, Kit inhibitor	100	Drug	VEGFR	4
D21	OTS167	MELK inhibitor	1	Drug	Miscl.	4
D22	AZD1775	Wee1 inhibitor	10	Drug	Miscl.	4
D23	Ralimetinib	p38MAPK inhibitor	100	Drug	Miscl.	4
D24	BzCl	BzCl	0	BzCl	Miscl.	4
E1	cells	cells	None	cells	Negative Controls	4
E2	Radotinib	ABL, PDGFR inhibitor	10	Drug	Miscl.	4
E3	Brigatinib	ALK inhibitor, including gatekeeper mutant ALK	0.1	Drug	Miscl.	4
E4	SNS-032	CDK inhibitor	1	Drug	CDK	4

E5	Gandotinib	JAK2 inhibitor	10	Drug	Miscl.	4
E6	Vistusertib	mTOR inhibitor, ATP-competitive	1	Drug	Miscl.	4
E7	Fedratinib	JAK2-selective inhibitor	1	Drug	Miscl.	4
E8	Milciclib	CDK2 inhibitor	1	Drug	CDK	4
E9	BI 2536	PLK1 inhibitor	1	Drug	Miscl.	4
E10	Binimetinib	MEK1/2 inhibitor	0.1	Drug	MEK1/2	4
E11	Rabusertib	Chk1 inhibitor	0.1	Drug	Miscl.	4
E12	ENMD-2076	pan-Aurora, VEGFR inhibitor	10	Drug	VEGFR	4
E13	PD0325901	MEK1/2 inhibitor	0.1	Drug	MEK1/2	4
E14	Capmatinib	MET inhibitor	0.1	Drug	Miscl.	4
E15	DMSO	DMSO	0	DMSO	Negative Controls	4
E16	CEP-32496	BRAF inhibitor	10	Drug	Miscl.	4
E17	Silmitasertib	CSNK2A1 inhibitor	1	Drug	Miscl.	4
E18	Encorafenib	B-Raf(V600E) inhibitor	0.1	Drug	Miscl.	4
E19	Alpelisib	PI3Kalpha inhibitor	0.25	Drug	PI3K	4
E20	Motesanib	VEGFR, PDGFR, Ret, Kit inhibitor	10	Drug	VEGFR	4
E21	OTS167	MELK inhibitor	0.1	Drug	Miscl.	4
E22	AZD1775	Wee1 inhibitor	1	Drug	Miscl.	4
E23	Ralimetinib	p38MAPK inhibitor	10	Drug	Miscl.	4
E24	cells	cells	None	cells	Negative Controls	4
F1	cells	cells	None	cells	Negative Controls	4
F2	Radotinib	ABL, PDGFR inhibitor	1	Drug	Miscl.	4
F3	Linsitinib	IGF1R, IR inhibitor	10000	Drug	Miscl.	4
F4	Seliciclib	CDK2/7/9 inhibitor	10000	Drug	CDK	4
F5	Gandotinib	JAK2 inhibitor	1	Drug	Miscl.	4
F6	Ipatasertib	AKT inhibitor	10000	Drug	Miscl.	4
F7	Amuvatinib	Broad spectrum TK inhib	10000	Drug	Miscl.	4
F8	Tideglusib	GSK3 inhibitor	3000	Drug	Miscl.	4
F9	BI 2536	PLK1 inhibitor	0.1	Drug	Miscl.	4
F10	BzCl	BzCl	0	BzCl	Miscl.	4
F11	NVP-RAF265	"C-Raf" inhibitor, unclear MoA	1000	Drug	Miscl.	4
F12	ENMD-2076	pan-Aurora, VEGFR inhibitor	1	Drug	VEGFR	4
F13	Sapitinib	Pan-HER inhibitor	1000	Drug	EGFR	4

F14	NVP-BGT226	PI3K/mTOR inhibitor	1000	Drug	PI3K	4
F15	Golvatinib	MET, VEGFR2 inhibitor	0.25	Drug	VEGFR	4
F16	CEP-32496	BRAF inhibitor	1	Drug	Miscl.	4
F17	Talmapimod	p38MAPK alpha selective inhibitor	10000	Drug	Miscl.	4
F18	Tanzisertib	JNK inhibitor	10000	Drug	Miscl.	4
F19	Baricitinib	JAK inhibitor	2500	Drug	Miscl.	4
F20	Motesanib	VEGFR, PDGFR, Ret, Kit inhibitor	1	Drug	VEGFR	4
F21	Entospletinib	SYK inhibitor	5000	Drug	Miscl.	4
F22	Alvocidib	CDK inhibitor	10000	Drug	CDK	4
F23	Ralimetinib	p38MAPK inhibitor	1	Drug	Miscl.	4
F24	cells	cells	None	cells	Negative Controls	4
G1	cells	cells	None	cells	Negative Controls	4
G2	TGR-1202	PI3Kdelta inhibitor	2500	Drug	PI3K	4
G3	Linsitinib	IGF1R, IR inhibitor	1000	Drug	Miscl.	4
G4	Seliciclib	CDK2/7/9 inhibitor	1000	Drug	CDK	4
G5	Sonolisib	PI3K inhibitor, pan-class I. Irreversible	10000	Drug	PI3K	4
G6	BzCl	BzCl	0	BzCl	Miscl.	4
G7	Amuvatinib	Broad spectrum TK inhib	1000	Drug	Miscl.	4
G8	Tideglusib	GSK3 inhibitor	300	Drug	Miscl.	4
G9	Tandutinib	FLT3, PDGFR, KIT inhibitor	1000	Drug	Miscl.	4
G10	AZD4547	FGFR inhibitor	1000	Drug	Miscl.	4
G11	NVP-RAF265	"C-Raf" inhibitor, unclear MoA	100	Drug	Miscl.	4
G12	Bafetinib	Abl, Lyn inhibitor	1000	Drug	Miscl.	4
G13	Sapitinib	Pan-HER inhibitor	100	Drug	EGFR	4
G14	NVP-BGT226	PI3K/mTOR inhibitor	100	Drug	PI3K	4
G15	BMS-777607	Met, Axl, Ron and Tyro3 inhibitor	2500	Drug	Miscl.	4
G16	Varlitinib	EGFR HER2 inhibitor	10000	Drug	EGFR	4
G17	Talmapimod	p38MAPK alpha selective inhibitor	1000	Drug	Miscl.	4
G18	Tanzisertib	JNK inhibitor	1000	Drug	Miscl.	4
G19	Baricitinib	JAK inhibitor	250	Drug	Miscl.	4
G20	Buparlisib	PI3K inhibitor, pan-class I	10000	Drug	PI3K	4
G21	Entospletinib	SYK inhibitor	500	Drug	Miscl.	4
G22	Alvocidib	CDK inhibitor	1000	Drug	CDK	4

G23	BMS-754807	IGF1R inhibitor	10000	Drug	Miscl.	4
G24	cells	cells	None	cells	Negative Controls	4
H1	cells	cells	None	cells	Negative Controls	4
H2	TGR-1202	PI3Kdelta inhibitor	250	Drug	PI3K	4
H3	Linsitinib	IGF1R, IR inhibitor	100	Drug	Miscl.	4
H4	Seliciclib	CDK2/7/9 inhibitor	100	Drug	CDK	4
H5	Sonolisib	PI3K inhibitor, pan-class I. Irreversible	1000	Drug	PI3K	4
H6	Ipatasertib	AKT inhibitor	1000	Drug	Miscl.	4
H7	Amuvatinib	Broad spectrum TK inhib	100	Drug	Miscl.	4
H8	Tideglusib	GSK3 inhibitor	30	Drug	Miscl.	4
H9	Tandutinib	FLT3, PDGFR, KIT inhibitor	100	Drug	Miscl.	4
H10	AZD4547	FGFR inhibitor	100	Drug	Miscl.	4
H11	NVP-RAF265	"C-Raf" inhibitor, unclear MoA	10	Drug	Miscl.	4
H12	Bafetinib	Abl, Lyn inhibitor	100	Drug	Miscl.	4
H13	Sapitinib	Pan-HER inhibitor	10	Drug	EGFR	4
H14	NVP-BGT226	PI3K/mTOR inhibitor	10	Drug	PI3K	4
H15	BMS-777607	Met, Axl, Ron and Tyro3 inhibitor	250	Drug	Miscl.	4
H16	Varlitinib	EGFR HER2 inhibitor	1000	Drug	EGFR	4
H17	Talmapimod	p38MAPK alpha selective inhibitor	100	Drug	Miscl.	4
H18	Tanzisertib	JNK inhibitor	100	Drug	Miscl.	4
H19	DMSO	DMSO	0	DMSO	Negative Controls	4
H20	Buparlisib	PI3K inhibitor, pan-class I	1000	Drug	PI3K	4
H21	Entospletinib	SYK inhibitor	50	Drug	Miscl.	4
H22	Alvocidib	CDK inhibitor	100	Drug	CDK	4
H23	BMS-754807	IGF1R inhibitor	1000	Drug	Miscl.	4
H24	cells	cells	None	cells	Negative Controls	4
I1	cells	cells	None	cells	Negative Controls	4
I2	TGR-1202	PI3Kdelta inhibitor	25	Drug	PI3K	4
I3	Linsitinib	IGF1R, IR inhibitor	10	Drug	Miscl.	4
I4	Seliciclib	CDK2/7/9 inhibitor	10	Drug	CDK	4
I5	Sonolisib	PI3K inhibitor, pan-class I. Irreversible	100	Drug	PI3K	4
I6	Ipatasertib	AKT inhibitor	100	Drug	Miscl.	4
I7	Amuvatinib	Broad spectrum TK inhib	10	Drug	Miscl.	4

I8	DMSO	DMSO	0	DMSO	Negative Controls	4
I9	Tandutinib	FLT3, PDGFR, KIT inhibitor	10	Drug	Miscl.	4
I10	AZD4547	FGFR inhibitor	10	Drug	Miscl.	4
I11	NVP-RAF265	"C-Raf" inhibitor, unclear MoA	1	Drug	Miscl.	4
I12	Bafetinib	Abl, Lyn inhibitor	10	Drug	Miscl.	4
I13	Sapitinib	Pan-HER inhibitor	1	Drug	EGFR	4
I14	NVP-BGT226	PI3K/mTOR inhibitor	1	Drug	PI3K	4
I15	BMS-777607	Met, Axl, Ron and Tyro3 inhibitor	25	Drug	Miscl.	4
I16	Varlitinib	EGFR HER2 inhibitor	100	Drug	EGFR	4
I17	Talmapimod	p38MAPK alpha selective inhibitor	10	Drug	Miscl.	4
I18	Tanzisertib	JNK inhibitor	10	Drug	Miscl.	4
I19	Baricitinib	JAK inhibitor	25	Drug	Miscl.	4
I20	Buparlisib	PI3K inhibitor, pan-class I	100	Drug	PI3K	4
I21	Entospletinib	SYK inhibitor	5	Drug	Miscl.	4
I22	Alvocidib	CDK inhibitor	10	Drug	CDK	4
I23	BMS-754807	IGF1R inhibitor	100	Drug	Miscl.	4
I24	cells	cells	None	cells	Negative Controls	4
J1	cells	cells	None	cells	Negative Controls	4
J2	TGR-1202	PI3Kdelta inhibitor	2.5	Drug	PI3K	4
J3	Linsitinib	IGF1R, IR inhibitor	1	Drug	Miscl.	4
J4	Seliciclib	CDK2/7/9 inhibitor	1	Drug	CDK	4
J5	Sonolisib	PI3K inhibitor, pan-class I. Irreversible	10	Drug	PI3K	4
J6	Ipatasertib	AKT inhibitor	10	Drug	Miscl.	4
J7	Amuvatinib	Broad spectrum TK inhib	1	Drug	Miscl.	4
J8	Tideglusib	GSK3 inhibitor	3	Drug	Miscl.	4
J9	Tandutinib	FLT3, PDGFR, KIT inhibitor	1	Drug	Miscl.	4
J10	AZD4547	FGFR inhibitor	1	Drug	Miscl.	4
J11	NVP-RAF265	"C-Raf" inhibitor, unclear MoA	0.1	Drug	Miscl.	4
J12	Bafetinib	Abl, Lyn inhibitor	1	Drug	Miscl.	4
J13	Sapitinib	Pan-HER inhibitor	0.1	Drug	EGFR	4
J14	BzCl	BzCl	0	BzCl	Miscl.	4
J15	BMS-777607	Met, Axl, Ron and Tyro3 inhibitor	2.5	Drug	Miscl.	4
J16	Varlitinib	EGFR HER2 inhibitor	10	Drug	EGFR	4

J17	Talmapimod	p38MAPK alpha selective inhibitor	1	Drug	Miscl.	4
J18	Tanzisertib	JNK inhibitor	1	Drug	Miscl.	4
J19	Baricitinib	JAK inhibitor	2.5	Drug	Miscl.	4
J20	Buparlisib	PI3K inhibitor, pan-class I	10	Drug	PI3K	4
J21	Entospletinib	SYK inhibitor	0.5	Drug	Miscl.	4
J22	Alvocidib	CDK inhibitor	1	Drug	CDK	4
J23	BMS-754807	IGF1R inhibitor	10	Drug	Miscl.	4
J24	cells	cells	None	cells	Negative Controls	4
K1	cells	cells	None	cells	Negative Controls	4
K2	TGR-1202	PI3Kdelta inhibitor	0.25	Drug	PI3K	4
K3	Danusertib	Aurora, Ret, TrkA, FGFR-1 inhibitor	1	Drug	Miscl.	4
K4	Dactolisib	mTOR/(PI3K) inhibitor	0.1	Drug	PI3K	4
K5	Sonolisib	PI3K inhibitor, pan-class I. Irreversible	1	Drug	PI3K	4
K6	Ipatasertib	AKT inhibitor	1	Drug	Miscl.	4
K7	Icotinib	EGFR inhibitor	1	Drug	EGFR	4
K8	Tideglusib	GSK3 inhibitor	0.3	Drug	Miscl.	4
K9	Tandutinib	FLT3, PDGFR, KIT inhibitor	0.1	Drug	Miscl.	4
K10	AZD4547	FGFR inhibitor	0.1	Drug	Miscl.	4
K11	Sotrastaurin	PKC inhibitor	1	Drug	Miscl.	4
K12	Bafetinib	Abl, Lyn inhibitor	0.1	Drug	Miscl.	4
K13	Tesevatinib	EGFR, ERBB2, VEGFR, EPHB4	0.1	Drug	EGFR	4
K14	NVP-BGT226	PI3K/mTOR inhibitor	0.1	Drug	PI3K	4
K15	BMS-777607	Met, Axl, Ron and Tyro3 inhibitor	0.25	Drug	Miscl.	4
K16	Varlitinib	EGFR HER2 inhibitor	1	Drug	EGFR	4
K17	Tozasertib	pan-Aurora inhibitor	1	Drug	Miscl.	4
K18	UCN-01	PKCbeta, PDK1, Chk, Cdk2 inhibitor	1	Drug	Miscl.	4
K19	Baricitinib	JAK inhibitor	0.25	Drug	Miscl.	4
K20	Buparlisib	PI3K inhibitor, pan-class I	1	Drug	PI3K	4
K21	BzCl	BzCl	0	BzCl	Miscl.	4
K22	Neflamapimod	p38MAPK inhibitor	1	Drug	Miscl.	4
K23	BMS-754807	IGF1R inhibitor	1	Drug	Miscl.	4
K24	cells	cells	None	cells	Negative Controls	4
L1	cells	cells	None	cells	Negative Controls	4

L2	Acalabrutinib	BTK inhibitor	0.1	Drug	Miscl.	4
L3	Danusertib	Aurora, Ret, TrkA, FGFR-1 inhibitor	10	Drug	Miscl.	4
L4	Dactolisib	mTOR/(PI3K) inhibitor	1	Drug	PI3K	4
L5	Crenolanib	PDGFRA and PDGFRB inhibitor	1	Drug	Miscl.	4
L6	AZD-5363	AKT inhibitor	1	Drug	Miscl.	4
L7	Icotinib	EGFR inhibitor	10	Drug	EGFR	4
L8	MK-2206	AKT inhibitor	0.1	Drug	Miscl.	4
L9	Tivantinib	MET inhibitor	0.1	Drug	Miscl.	4
L10	Galunisertib	TGF-B/Smad inhibitor	0.1	Drug	Miscl.	4
L11	Sotrastaurin	PKC inhibitor	10	Drug	Miscl.	4
L12	PH-797804	p38MAPK inhibitor	0.1	Drug	Miscl.	4
L13	Tesevatinib	EGFR, ERBB2, VEGFR, EPHB4	1	Drug	EGFR	4
L14	Gedatolisib	PI3K/mTOR inhibitor	0.1	Drug	PI3K	4
L15	TG100-115	PI3K gamma/delta inhibitor	1	Drug	PI3K	4
L16	Telatinib	VEGFR, KIT, PDGFR inhibitor	1	Drug	VEGFR	4
L17	DMSO	DMSO	0	DMSO	Negative Controls	4
L18	UCN-01	PKCbeta, PDK1, Chk, Cdk2 inhibitor	10	Drug	Miscl.	4
L19	GDC-0623	MEK1/2 inhibitor	0.25	Drug	MEK1/2	4
L20	AT9283	Aurora A & B, Jak2, Flt, Abl inhibitor	0.1	Drug	Miscl.	4
L21	Copanlisib	PI3K alpha, delta selective inhibitor	0.1	Drug	PI3K	4
L22	Neflamapimod	p38MAPK inhibitor	10	Drug	Miscl.	4
L23	Triciribine	AKT inhibitor	10	Drug	Miscl.	4
L24	cells	cells	None	cells	Negative Controls	4
M1	cells	cells	None	cells	Negative Controls	4
M2	Acalabrutinib	BTK inhibitor	1	Drug	Miscl.	4
M3	Danusertib	Aurora, Ret, TrkA, FGFR-1 inhibitor	100	Drug	Miscl.	4
M4	BzCl	BzCl	0	BzCl	Miscl.	4
M5	Crenolanib	PDGFRA and PDGFRB inhibitor	10	Drug	Miscl.	4
M6	AZD-5363	AKT inhibitor	10	Drug	Miscl.	4
M7	Icotinib	EGFR inhibitor	100	Drug	EGFR	4
M8	MK-2206	AKT inhibitor	1	Drug	Miscl.	4
M9	Tivantinib	MET inhibitor	1	Drug	Miscl.	4
M10	Galunisertib	TGF-B/Smad inhibitor	1	Drug	Miscl.	4

M11	Sotrastaurin	PKC inhibitor	100	Drug	Miscl.	4
M12	PH-797804	p38MAPK inhibitor	1	Drug	Miscl.	4
M13	Tesevatinib	EGFR, ERBB2, VEGFR, EPHB4	10	Drug	EGFR	4
M14	Gedatolisib	PI3K/mTOR inhibitor	1	Drug	PI3K	4
M15	TG100-115	PI3K gamma/delta inhibitor	10	Drug	PI3K	4
M16	Telatinib	VEGFR, KIT, PDGFR inhibitor	10	Drug	VEGFR	4
M17	Tozasertib	pan-Aurora inhibitor	10	Drug	Miscl.	4
M18	UCN-01	PKCbeta, PDK1, Chk, Cdk2 inhibitor	100	Drug	Miscl.	4
M19	GDC-0623	MEK1/2 inhibitor	2.5	Drug	MEK1/2	4
M20	AT9283	Aurora A & B, Jak2, Flt, Abl inhibitor	1	Drug	Miscl.	4
M21	Copanlisib	PI3K alpha, delta selective inhibitor	1	Drug	PI3K	4
M22	Neflamapimod	p38MAPK inhibitor	100	Drug	Miscl.	4
M23	Triciribine	AKT inhibitor	100	Drug	Miscl.	4
M24	cells	cells	None	cells	Negative Controls	4
N1	cells	cells	None	cells	Negative Controls	4
N2	Acalabrutinib	BTK inhibitor	10	Drug	Miscl.	4
N3	Danusertib	Aurora, Ret, TrkA, FGFR-1 inhibitor	1000	Drug	Miscl.	4
N4	Dactolisib	mTOR/(PI3K) inhibitor	10	Drug	PI3K	4
N5	Crenolanib	PDGFRA and PDGFRB inhibitor	100	Drug	Miscl.	4
N6	AZD-5363	AKT inhibitor	100	Drug	Miscl.	4
N7	DMSO	DMSO	0	DMSO	Negative Controls	4
N8	MK-2206	AKT inhibitor	10	Drug	Miscl.	4
N9	Tivantinib	MET inhibitor	10	Drug	Miscl.	4
N10	Galunisertib	TGF-B/Smad inhibitor	10	Drug	Miscl.	4
N11	BzCl	BzCl	0	BzCl	Miscl.	4
N12	PH-797804	p38MAPK inhibitor	10	Drug	Miscl.	4
N13	Tesevatinib	EGFR, ERBB2, VEGFR, EPHB4	100	Drug	EGFR	4
N14	Gedatolisib	PI3K/mTOR inhibitor	10	Drug	PI3K	4
N15	TG100-115	PI3K gamma/delta inhibitor	100	Drug	PI3K	4
N16	Telatinib	VEGFR, KIT, PDGFR inhibitor	100	Drug	VEGFR	4
N17	Tozasertib	pan-Aurora inhibitor	100	Drug	Miscl.	4
N18	UCN-01	PKCbeta, PDK1, Chk, Cdk2 inhibitor	1000	Drug	Miscl.	4
N19	GDC-0623	MEK1/2 inhibitor	25	Drug	MEK1/2	4

N20	AT9283	Aurora A & B, Jak2, Flt, Abl inhibitor	10	Drug	Miscl.	4
N21	Copanlisib	PI3K alpha, delta selective inhibitor	10	Drug	PI3K	4
N22	Neflamapimod	p38MAPK inhibitor	1000	Drug	Miscl.	4
N23	Triciribine	AKT inhibitor	1000	Drug	Miscl.	4
N24	cells	cells	None	cells	Negative Controls	4
O1	cells	cells	None	cells	Negative Controls	4
O2	Acalabrutinib	BTK inhibitor	100	Drug	Miscl.	4
O3	Danusertib	Aurora, Ret, TrkA, FGFR-1 inhibitor	10000	Drug	Miscl.	4
O4	Dactolisib	mTOR/(PI3K) inhibitor	100	Drug	PI3K	4
O5	Crenolanib	PDGFRA and PDGFRB inhibitor	1000	Drug	Miscl.	4
O6	AZD-5363	AKT inhibitor	1000	Drug	Miscl.	4
O7	Icotinib	EGFR inhibitor	1000	Drug	EGFR	4
O8	MK-2206	AKT inhibitor	100	Drug	Miscl.	4
O9	Tivantinib	MET inhibitor	100	Drug	Miscl.	4
O10	Galunisertib	TGF-B/Smad inhibitor	100	Drug	Miscl.	4
O11	Sotrastaurin	PKC inhibitor	1000	Drug	Miscl.	4
O12	PH-797804	p38MAPK inhibitor	100	Drug	Miscl.	4
O13	DMSO	DMSO	0	DMSO	Negative Controls	4
O14	Gedatolisib	PI3K/mTOR inhibitor	100	Drug	PI3K	4
O15	TG100-115	PI3K gamma/delta inhibitor	1000	Drug	PI3K	4
O16	Telatinib	VEGFR, KIT, PDGFR inhibitor	1000	Drug	VEGFR	4
O17	Tozasertib	pan-Aurora inhibitor	1000	Drug	Miscl.	4
O18	BzCl	BzCl	0	BzCl	Miscl.	4
O19	GDC-0623	MEK1/2 inhibitor	250	Drug	MEK1/2	4
O20	AT9283	Aurora A & B, Jak2, Flt, Abl inhibitor	100	Drug	Miscl.	4
O21	Copanlisib	PI3K alpha, delta selective inhibitor	100	Drug	PI3K	4
O22	Neflamapimod	p38MAPK inhibitor	10000	Drug	Miscl.	4
O23	Triciribine	AKT inhibitor	10000	Drug	Miscl.	4
O24	cells	cells	None	cells	Negative Controls	4
P1	cells	cells	None	cells	Negative Controls	4
P2	Acalabrutinib	BTK inhibitor	1000	Drug	Miscl.	4
P3	DMSO	DMSO	0	DMSO	Negative Controls	4
P4	Dactolisib	mTOR/(PI3K) inhibitor	1000	Drug	PI3K	4

P5	Crenolanib	PDGFRA and PDGFRB inhibitor	10000	Drug	Miscl.	4
P6	AZD-5363	AKT inhibitor	10000	Drug	Miscl.	4
P7	Icotinib	EGFR inhibitor	10000	Drug	EGFR	4
P8	MK-2206	AKT inhibitor	1000	Drug	Miscl.	4
P9	Tivantinib	MET inhibitor	1000	Drug	Miscl.	4
P10	Galunisertib	TGF-B/Smad inhibitor	1000	Drug	Miscl.	4
P11	Sotrastaurin	PKC inhibitor	10000	Drug	Miscl.	4
P12	PH-797804	p38MAPK inhibitor	1000	Drug	Miscl.	4
P13	Tesevatinib	EGFR, ERBB2, VEGFR, EPHB4	1000	Drug	EGFR	4
P14	Gedatolisib	PI3K/mTOR inhibitor	1000	Drug	PI3K	4
P15	TG100-115	PI3K gamma/delta inhibitor	10000	Drug	PI3K	4
P16	Telatinib	VEGFR, KIT, PDGFR inhibitor	10000	Drug	VEGFR	4
P17	Tozasertib	pan-Aurora inhibitor	10000	Drug	Miscl.	4
P18	UCN-01	PKCbeta, PDK1, Chk, Cdk2 inhibitor	10000	Drug	Miscl.	4
P19	GDC-0623	MEK1/2 inhibitor	2500	Drug	MEK1/2	4
P20	AT9283	Aurora A & B, Jak2, Flt, Abl inhibitor	1000	Drug	Miscl.	4
P21	Copanlisib	PI3K alpha, delta selective inhibitor	1000	Drug	PI3K	4
P22	DMSO	DMSO	0	DMSO	Negative Controls	4
P23	Triciribine	AKT inhibitor	100000	Drug	Miscl.	4
P24	BzCl	BzCl	0	BzCl	Miscl.	4
A1	cells	cells	None	cells	Negative Controls	5
A2	BzCl	BzCl	0	BzCl	Miscl.	5
A3	Prexasertib	Chk1 inhibitor	10000	Drug	Miscl.	5
A4	Afuresertib	AKT1-selective inhibitor	1000	Drug	Miscl.	5
A5	PF-06463922	ALK, ROS1 inhibitor	1000	Drug	Miscl.	5
A6	LY3023414	PI3K/mTOR/DNA-PK inhibitor	2500	Drug	PI3K	5
A7	AMG319	PI3Kdelta inhibitor	1000	Drug	PI3K	5
A8	GSK2256098	FAK inhibitor	1000	Drug	Miscl.	5
A9	Larotrectinib	TRK inhibitor	1000	Drug	Miscl.	5
A10	PF06650833	IRAK4 inhibitor	10000	Drug	Miscl.	5
A11	Sapanisertib	mTOR inhibitor	1000	Drug	Miscl.	5
A12	ASP3026	ALK inhibitor	10000	Drug	Miscl.	5
A13	PF-00477736	Chk1 inhibitor	10000	Drug	Miscl.	5

A14	LY-2874455	FGFR inhibitor	1000	Drug	Miscl.	5
A15	Mubritinib	HER2 inhibitor	1000	Drug	Miscl.	5
A16	AZD-6482	PI3Kbeta-selective inhibitor	2500	Drug	PI3K	5
A17	Palomid-529	AKT, MTOR, PI3K inhibitor	10000	Drug	PI3K	5
A18	NVP-AEW541	IGF1R inhibitor	10000	Drug	Miscl.	5
A19	AZD-5438	CDK1,2,9 inhibitor	10000	Drug	CDK	5
A20	AZD8055	mTOR inhibitor	10000	Drug	Miscl.	5
A21	Bryostatin 1	PKC activator	100	Drug	Miscl.	5
A22	CEP-37440	ALK inhibitor	5000	Drug	Miscl.	5
A23	BzCl	BzCl	0	BzCl	Miscl.	5
A24	cells	cells	None	cells	Negative Controls	5
B1	cells	cells	None	cells	Negative Controls	5
B2	KD025	ROCK2 inhibitor	5000	Drug	Miscl.	5
B3	Prexasertib	Chk1 inhibitor	1000	Drug	Miscl.	5
B4	Afuresertib	AKT1-selective inhibitor	100	Drug	Miscl.	5
B5	PF-06463922	ALK, ROS1 inhibitor	100	Drug	Miscl.	5
B6	LY3023414	PI3K/mTOR/DNA-PK inhibitor	250	Drug	PI3K	5
B7	AMG319	PI3Kdelta inhibitor	100	Drug	PI3K	5
B8	GSK2256098	FAK inhibitor	100	Drug	Miscl.	5
B9	DMSO	DMSO	0	DMSO	Negative Controls	5
B10	PF06650833	IRAK4 inhibitor	1000	Drug	Miscl.	5
B11	Sapanisertib	mTOR inhibitor	100	Drug	Miscl.	5
B12	ASP3026	ALK inhibitor	1000	Drug	Miscl.	5
B13	PF-00477736	Chk1 inhibitor	1000	Drug	Miscl.	5
B14	LY-2874455	FGFR inhibitor	100	Drug	Miscl.	5
B15	Mubritinib	HER2 inhibitor	100	Drug	Miscl.	5
B16	BzCl	BzCl	0	BzCl	Miscl.	5
B17	Palomid-529	AKT, MTOR, PI3K inhibitor	1000	Drug	PI3K	5
B18	NVP-AEW541	IGF1R inhibitor	1000	Drug	Miscl.	5
B19	AZD-5438	CDK1,2,9 inhibitor	1000	Drug	CDK	5
B20	AZD8055	mTOR inhibitor	1000	Drug	Miscl.	5
B21	Bryostatin 1	PKC activator	10	Drug	Miscl.	5
B22	CEP-37440	ALK inhibitor	500	Drug	Miscl.	5

B23	PF-03758309	PAK inhibitor	10000	Drug	Miscl.	5
B24	cells	cells	None	cells	Negative Controls	5
C1	cells	cells	None	cells	Negative Controls	5
C2	KD025	ROCK2 inhibitor	500	Drug	Miscl.	5
C3	Prexasertib	Chk1 inhibitor	100	Drug	Miscl.	5
C4	Afuresertib	AKT1-selective inhibitor	10	Drug	Miscl.	5
C5	PF-06463922	ALK, ROS1 inhibitor	10	Drug	Miscl.	5
C6	LY3023414	PI3K/mTOR/DNA-PK inhibitor	25	Drug	PI3K	5
C7	AMG319	PI3Kdelta inhibitor	10	Drug	PI3K	5
C8	GSK2256098	FAK inhibitor	10	Drug	Miscl.	5
C9	Larotrectinib	TRK inhibitor	100	Drug	Miscl.	5
C10	PF06650833	IRAK4 inhibitor	100	Drug	Miscl.	5
C11	Sapanisertib	mTOR inhibitor	10	Drug	Miscl.	5
C12	DMSO	DMSO	0	DMSO	Negative Controls	5
C13	PF-00477736	Chk1 inhibitor	100	Drug	Miscl.	5
C14	LY-2874455	FGFR inhibitor	10	Drug	Miscl.	5
C15	Mubritinib	HER2 inhibitor	10	Drug	Miscl.	5
C16	AZD-6482	PI3Kbeta-selective inhibitor	250	Drug	PI3K	5
C17	Palomid-529	AKT, MTOR, PI3K inhibitor	100	Drug	PI3K	5
C18	NVP-AEW541	IGF1R inhibitor	100	Drug	Miscl.	5
C19	AZD-5438	CDK1,2,9 inhibitor	100	Drug	CDK	5
C20	DMSO	DMSO	0	DMSO	Negative Controls	5
C21	Bryostatin 1	PKC activator	1	Drug	Miscl.	5
C22	CEP-37440	ALK inhibitor	50	Drug	Miscl.	5
C23	PF-03758309	PAK inhibitor	1000	Drug	Miscl.	5
C24	cells	cells	None	cells	Negative Controls	5
D1	cells	cells	None	cells	Negative Controls	5
D2	KD025	ROCK2 inhibitor	50	Drug	Miscl.	5
D3	Prexasertib	Chk1 inhibitor	10	Drug	Miscl.	5
D4	Afuresertib	AKT1-selective inhibitor	1	Drug	Miscl.	5
D5	DMSO	DMSO	0	DMSO	Negative Controls	5
D6	LY3023414	PI3K/mTOR/DNA-PK inhibitor	2.5	Drug	PI3K	5
D7	AMG319	PI3Kdelta inhibitor	1	Drug	PI3K	5

D8	GSK2256098	FAK inhibitor	1	Drug	Miscl.	5
D9	Larotrectinib	TRK inhibitor	10	Drug	Miscl.	5
D10	PF06650833	IRAK4 inhibitor	10	Drug	Miscl.	5
D11	Sapanisertib	mTOR inhibitor	1	Drug	Miscl.	5
D12	ASP3026	ALK inhibitor	100	Drug	Miscl.	5
D13	PF-00477736	Chk1 inhibitor	10	Drug	Miscl.	5
D14	LY-2874455	FGFR inhibitor	1	Drug	Miscl.	5
D15	Mubritinib	HER2 inhibitor	1	Drug	Miscl.	5
D16	AZD-6482	PI3Kbeta-selective inhibitor	25	Drug	PI3K	5
D17	Palomid-529	AKT, MTOR, PI3K inhibitor	10	Drug	PI3K	5
D18	NVP-AEW541	IGF1R inhibitor	10	Drug	Miscl.	5
D19	AZD-5438	CDK1,2,9 inhibitor	10	Drug	CDK	5
D20	AZD8055	mTOR inhibitor	100	Drug	Miscl.	5
D21	Bryostatin 1	PKC activator	0.1	Drug	Miscl.	5
D22	CEP-37440	ALK inhibitor	5	Drug	Miscl.	5
D23	PF-03758309	PAK inhibitor	100	Drug	Miscl.	5
D24	cells	cells	None	cells	Negative Controls	5
E1	cells	cells	None	cells	Negative Controls	5
E2	KD025	ROCK2 inhibitor	5	Drug	Miscl.	5
E3	Prexasertib	Chk1 inhibitor	1	Drug	Miscl.	5
E4	Afuresertib	AKT1-selective inhibitor	0.1	Drug	Miscl.	5
E5	PF-06463922	ALK, ROS1 inhibitor	1	Drug	Miscl.	5
E6	LY3023414	PI3K/mTOR/DNA-PK inhibitor	0.25	Drug	PI3K	5
E7	AMG319	PI3Kdelta inhibitor	0.1	Drug	PI3K	5
E8	GSK2256098	FAK inhibitor	0.1	Drug	Miscl.	5
E9	Larotrectinib	TRK inhibitor	1	Drug	Miscl.	5
E10	PF06650833	IRAK4 inhibitor	1	Drug	Miscl.	5
E11	Sapanisertib	mTOR inhibitor	0.1	Drug	Miscl.	5
E12	ASP3026	ALK inhibitor	10	Drug	Miscl.	5
E13	PF-00477736	Chk1 inhibitor	1	Drug	Miscl.	5
E14	LY-2874455	FGFR inhibitor	0.1	Drug	Miscl.	5
E15	DMSO	DMSO	0	DMSO	Negative Controls	5
E16	AZD-6482	PI3Kbeta-selective inhibitor	2.5	Drug	PI3K	5

E17	Palomid-529	AKT, MTOR, PI3K inhibitor	1	Drug	PI3K	5
E18	NVP-AEW541	IGF1R inhibitor	1	Drug	Miscl.	5
E19	AZD-5438	CDK1,2,9 inhibitor	1	Drug	CDK	5
E20	AZD8055	mTOR inhibitor	10	Drug	Miscl.	5
E21	Bryostatin 1	PKC activator	0.01	Drug	Miscl.	5
E22	CEP-37440	ALK inhibitor	0.5	Drug	Miscl.	5
E23	PF-03758309	PAK inhibitor	10	Drug	Miscl.	5
E24	BzCl	BzCl	0	BzCl	Miscl.	5
F1	cells	cells	None	cells	Negative Controls	5
F2	KD025	ROCK2 inhibitor	0.5	Drug	Miscl.	5
F3	Ulixertinib	ERK inhibitor	10000	Drug	Miscl.	5
F4	Poziotinib	pan-HER inhibitor	1000	Drug	EGFR	5
F5	PF-06463922	ALK, ROS1 inhibitor	0.1	Drug	Miscl.	5
F6	Glesatinib	MET, AXL, TIE, VEGFR, RON inhibitor	2500	Drug	Miscl.	5
F7	AZD3759	EGFR inhibitor, BBB penetrable	1000	Drug	EGFR	5
F8	GDC-0853	BTK inhibitor	1000	Drug	Miscl.	5
F9	Larotrectinib	TRK inhibitor	0.1	Drug	Miscl.	5
F10	BzCl	BzCl	0	BzCl	Miscl.	5
F11	GSK2636771	PI3K beta selective inhibitor	10000	Drug	PI3K	5
F12	ASP3026	ALK inhibitor	1	Drug	Miscl.	5
F13	Tamatinib	Syk inhibitor	10000	Drug	Miscl.	5
F14	GSK-1070916	AURb, AURc inhibitor	1000	Drug	Miscl.	5
F15	Mubritinib	HER2 inhibitor	0.1	Drug	Miscl.	5
F16	AZD-6482	PI3Kbeta-selective inhibitor	0.25	Drug	PI3K	5
F17	PF-00562271	FAK inhibitor	10000	Drug	Miscl.	5
F18	AZD-1080	GSK3 inhibitor	10000	Drug	Miscl.	5
F19	GSK-690693	AKT, PKA, PKC inhibitor	10000	Drug	Miscl.	5
F20	AZD8055	mTOR inhibitor	1	Drug	Miscl.	5
F21	Tepotinib	c-Met inhibitor	1000	Drug	Miscl.	5
F22	BGB324	Axl inhibitor	10000	Drug	Miscl.	5
F23	PF-03758309	PAK inhibitor	1	Drug	Miscl.	5
F24	cells	cells	None	cells	Negative Controls	5
G1	cells	cells	None	cells	Negative Controls	5

G2	Uprosertib	AKT inhibitor	10000	Drug	Miscl.	5
G3	Ulixertinib	ERK inhibitor	1000	Drug	Miscl.	5
G4	Poziotinib	pan-HER inhibitor	100	Drug	EGFR	5
G5	Entrectinib	TRK, ROS1, ALK inhibitor	1000	Drug	Miscl.	5
G6	BzCl	BzCl	0	BzCl	Miscl.	5
G7	AZD3759	EGFR inhibitor, BBB penetrable	100	Drug	EGFR	5
G8	GDC-0853	BTK inhibitor	100	Drug	Miscl.	5
G9	Serabelisib	PI3Kalpha selective inhibitor	10000	Drug	PI3K	5
G10	Amcasertib	Cancer stem cell kinase inhibitor	10000	Drug	Miscl.	5
G11	GSK2636771	PI3K beta selective inhibitor	1000	Drug	PI3K	5
G12	SGI-1776	PIM kinase inhibitor	10000	Drug	Miscl.	5
G13	Tamatinib	Syk inhibitor	1000	Drug	Miscl.	5
G14	GSK-1070916	AURb, AURc inhibitor	100	Drug	Miscl.	5
G15	Tucatinib	HER2 inhibitor	2500	Drug	Miscl.	5
G16	MK-8776	CHEK1 inhibitor	2500	Drug	Miscl.	5
G17	PF-00562271	FAK inhibitor	1000	Drug	Miscl.	5
G18	AZD-1080	GSK3 inhibitor	1000	Drug	Miscl.	5
G19	GSK-690693	AKT, PKA, PKC inhibitor	1000	Drug	Miscl.	5
G20	Infigratinib	FGFR inhibitor	1000	Drug	Miscl.	5
G21	Tepotinib	c-Met inhibitor	100	Drug	Miscl.	5
G22	BGB324	Axl inhibitor	1000	Drug	Miscl.	5
G23	Doramapimod	p38MAPK inhibitor	10000	Drug	Miscl.	5
G24	cells	cells	None	cells	Negative Controls	5
H1	cells	cells	None	cells	Negative Controls	5
H2	Uprosertib	AKT inhibitor	1000	Drug	Miscl.	5
H3	Ulixertinib	ERK inhibitor	100	Drug	Miscl.	5
H4	Poziotinib	pan-HER inhibitor	10	Drug	EGFR	5
H5	Entrectinib	TRK, ROS1, ALK inhibitor	100	Drug	Miscl.	5
H6	Glesatinib	MET, AXL, TIE, VEGFR, RON inhibitor	250	Drug	Miscl.	5
H7	AZD3759	EGFR inhibitor, BBB penetrable	10	Drug	EGFR	5
H8	GDC-0853	BTK inhibitor	10	Drug	Miscl.	5
H9	Serabelisib	PI3Kalpha selective inhibitor	1000	Drug	PI3K	5
H10	Amcasertib	Cancer stem cell kinase inhibitor	1000	Drug	Miscl.	5

H11	GSK2636771	PI3K beta selective inhibitor	100	Drug	PI3K	5
H12	SGI-1776	PIM kinase inhibitor	1000	Drug	Miscl.	5
H13	Tamatinib	Syk inhibitor	100	Drug	Miscl.	5
H14	GSK-1070916	AURb, AURc inhibitor	10	Drug	Miscl.	5
H15	Tucatinib	HER2 inhibitor	250	Drug	Miscl.	5
H16	MK-8776	CHEK1 inhibitor	250	Drug	Miscl.	5
H17	PF-00562271	FAK inhibitor	100	Drug	Miscl.	5
H18	AZD-1080	GSK3 inhibitor	100	Drug	Miscl.	5
H19	DMSO	DMSO	0	DMSO	Negative Controls	5
H20	Infigratinib	FGFR inhibitor	100	Drug	Miscl.	5
H21	Tepotinib	c-Met inhibitor	10	Drug	Miscl.	5
H22	BGB324	Axl inhibitor	100	Drug	Miscl.	5
H23	Doramapimod	p38MAPK inhibitor	1000	Drug	Miscl.	5
H24	cells	cells	None	cells	Negative Controls	5
I1	cells	cells	None	cells	Negative Controls	5
I2	Uprosertib	AKT inhibitor	100	Drug	Miscl.	5
I3	Ulixertinib	ERK inhibitor	10	Drug	Miscl.	5
I4	Poziotinib	pan-HER inhibitor	1	Drug	EGFR	5
I5	Entrectinib	TRK, ROS1, ALK inhibitor	10	Drug	Miscl.	5
I6	Glesatinib	MET, AXL, TIE, VEGFR, RON inhibitor	25	Drug	Miscl.	5
I7	AZD3759	EGFR inhibitor, BBB penetrable	1	Drug	EGFR	5
I8	DMSO	DMSO	0	DMSO	Negative Controls	5
I9	Serabelisib	PI3Kalpha selective inhibitor	100	Drug	PI3K	5
I10	Amcasertib	Cancer stem cell kinase inhibitor	100	Drug	Miscl.	5
I11	GSK2636771	PI3K beta selective inhibitor	10	Drug	PI3K	5
I12	SGI-1776	PIM kinase inhibitor	100	Drug	Miscl.	5
I13	Tamatinib	Syk inhibitor	10	Drug	Miscl.	5
I14	GSK-1070916	AURb, AURc inhibitor	1	Drug	Miscl.	5
I15	Tucatinib	HER2 inhibitor	25	Drug	Miscl.	5
I16	MK-8776	CHEK1 inhibitor	25	Drug	Miscl.	5
I17	PF-00562271	FAK inhibitor	10	Drug	Miscl.	5
I18	AZD-1080	GSK3 inhibitor	10	Drug	Miscl.	5
I19	GSK-690693	AKT, PKA, PKC inhibitor	100	Drug	Miscl.	5

I20	Infigratinib	FGFR inhibitor	10	Drug	Miscl.	5
I21	Tepotinib	c-Met inhibitor	1	Drug	Miscl.	5
I22	BGB324	Axl inhibitor	10	Drug	Miscl.	5
I23	Doramapimod	p38MAPK inhibitor	100	Drug	Miscl.	5
I24	cells	cells	None	cells	Negative Controls	5
J1	cells	cells	None	cells	Negative Controls	5
J2	Uprosertib	AKT inhibitor	10	Drug	Miscl.	5
J3	Ulixertinib	ERK inhibitor	1	Drug	Miscl.	5
J4	Poziotinib	pan-HER inhibitor	0.1	Drug	EGFR	5
J5	Entrectinib	TRK, ROS1, ALK inhibitor	1	Drug	Miscl.	5
J6	Glesatinib	MET, AXL, TIE, VEGFR, RON inhibitor	2.5	Drug	Miscl.	5
J7	AZD3759	EGFR inhibitor, BBB penetrable	0.1	Drug	EGFR	5
J8	GDC-0853	BTK inhibitor	1	Drug	Miscl.	5
J9	Serabelisib	PI3Kalpha selective inhibitor	10	Drug	PI3K	5
J10	Amcasertib	Cancer stem cell kinase inhibitor	10	Drug	Miscl.	5
J11	GSK2636771	PI3K beta selective inhibitor	1	Drug	PI3K	5
J12	SGI-1776	PIM kinase inhibitor	10	Drug	Miscl.	5
J13	Tamatinib	Syk inhibitor	1	Drug	Miscl.	5
J14	BzCl	BzCl	0	BzCl	Miscl.	5
J15	Tucatinib	HER2 inhibitor	2.5	Drug	Miscl.	5
J16	MK-8776	CHEK1 inhibitor	2.5	Drug	Miscl.	5
J17	PF-00562271	FAK inhibitor	1	Drug	Miscl.	5
J18	AZD-1080	GSK3 inhibitor	1	Drug	Miscl.	5
J19	GSK-690693	AKT, PKA, PKC inhibitor	10	Drug	Miscl.	5
J20	Infigratinib	FGFR inhibitor	1	Drug	Miscl.	5
J21	Tepotinib	c-Met inhibitor	0.1	Drug	Miscl.	5
J22	BGB324	Axl inhibitor	1	Drug	Miscl.	5
J23	Doramapimod	p38MAPK inhibitor	10	Drug	Miscl.	5
J24	cells	cells	None	cells	Negative Controls	5
K1	cells	cells	None	cells	Negative Controls	5
K2	Uprosertib	AKT inhibitor	1	Drug	Miscl.	5
K3	Merestinib	Met inhibitor	0.1	Drug	Miscl.	5
K4	Spebrutinib	BTK inhibitor	0.1	Drug	Miscl.	5

K5	Entrectinib	TRK, ROS1, ALK inhibitor	0.1	Drug	Miscl.	5
K6	Glesatinib	MET, AXL, TIE, VEGFR, RON inhibitor	0.25	Drug	Miscl.	5
K7	Olmutinib	EGFR(L858R/T790M) inhibitor	0.1	Drug	EGFR	5
K8	GDC-0853	BTK inhibitor	0.1	Drug	Miscl.	5
K9	Serabelisib	PI3Kalpha selective inhibitor	1	Drug	PI3K	5
K10	Amcasertib	Cancer stem cell kinase inhibitor	1	Drug	Miscl.	5
K11	BMS-911543	JAK2 inhibitor	1	Drug	Miscl.	5
K12	SGI-1776	PIM kinase inhibitor	1	Drug	Miscl.	5
K13	OSU-03012	PDPK1 inhibitor	2.5	Drug	Miscl.	5
K14	GSK-1070916	AURb, AURc inhibitor	0.1	Drug	Miscl.	5
K15	Tucatinib	HER2 inhibitor	0.25	Drug	Miscl.	5
K16	MK-8776	CHEK1 inhibitor	0.25	Drug	Miscl.	5
K17	AT7519	CDK1, 2, 4, 6 and 9 inhibitor	1	Drug	CDK	5
K18	GSK-461364	PLK1 inhibitor	1	Drug	Miscl.	5
K19	GSK-690693	AKT, PKA, PKC inhibitor	1	Drug	Miscl.	5
K20	Infigratinib	FGFR inhibitor	0.1	Drug	Miscl.	5
K21	BzCl	BzCl	0	BzCl	Miscl.	5
K22	AZD1208	PIM1, 2, 3 kinase inhibitor	1	Drug	Miscl.	5
K23	Doramapimod	p38MAPK inhibitor	1	Drug	Miscl.	5
K24	cells	cells	None	cells	Negative Controls	5
L1	cells	cells	None	cells	Negative Controls	5
L2	Filgotinib	JAK1-selective inhibitor	1	Drug	Miscl.	5
L3	Merestinib	Met inhibitor	1	Drug	Miscl.	5
L4	Spebrutinib	BTK inhibitor	1	Drug	Miscl.	5
L5	ABC294640	Sphingosine kinase 2 inhibitor	5	Drug	Miscl.	5
L6	CC-223	mTOR inhibitor	1	Drug	Miscl.	5
L7	Olmutinib	EGFR(L858R/T790M) inhibitor	1	Drug	EGFR	5
L8	Erdafitinib	FGFR inhibitor	0.1	Drug	Miscl.	5
L9	AMG-337	Met inhibitor	1	Drug	Miscl.	5
L10	Selonsertib	ASK1 inhibitor	0.1	Drug	Miscl.	5
L11	BMS-911543	JAK2 inhibitor	10	Drug	Miscl.	5
L12	TAK-901	Aurora, Src family, JAK3, RTK inhibitor	0.1	Drug	Miscl.	5
L13	OSU-03012	PDPK1 inhibitor	25	Drug	Miscl.	5

L14	AZD-8186	PI3Kbeta inhibitor	0.1	Drug	PI3K	5
L15	TAK-285	HER2 inhibitor	0.25	Drug	Miscl.	5
L16	Lucitanib	FGFR1, VEGFR inhibitor	1	Drug	Miscl.	5
L17	DMSO	DMSO	0	DMSO	Negative Controls	5
L18	GSK-461364	PLK1 inhibitor	10	Drug	Miscl.	5
L19	Bentamapimod	JNK inhibitor	1	Drug	Miscl.	5
L20	ZSTK474	PI3K gamma selective inhibitor	1	Drug	PI3K	5
L21	AT13148	p70S6K, PKA, ROCK (AKT) inhibitor	1	Drug	Miscl.	5
L22	AZD1208	PIM1, 2, 3 kinase inhibitor	10	Drug	Miscl.	5
L23	Omipalisib	PI3K/mTOR inhibitor	0.1	Drug	PI3K	5
L24	cells	cells	None	cells	Negative Controls	5
M1	cells	cells	None	cells	Negative Controls	5
M2	Filgotinib	JAK1-selective inhibitor	10	Drug	Miscl.	5
M3	Merestinib	Met inhibitor	10	Drug	Miscl.	5
M4	BzCl	BzCl	0	BzCl	Miscl.	5
M5	ABC294640	Sphingosine kinase 2 inhibitor	50	Drug	Miscl.	5
M6	CC-223	mTOR inhibitor	10	Drug	Miscl.	5
M7	Olmutinib	EGFR(L858R/T790M) inhibitor	10	Drug	EGFR	5
M8	Erdafitinib	FGFR inhibitor	1	Drug	Miscl.	5
M9	AMG-337	Met inhibitor	10	Drug	Miscl.	5
M10	Selonsertib	ASK1 inhibitor	1	Drug	Miscl.	5
M11	BMS-911543	JAK2 inhibitor	100	Drug	Miscl.	5
M12	TAK-901	Aurora, Src family, JAK3, RTK inhibitor	1	Drug	Miscl.	5
M13	OSU-03012	PDPK1 inhibitor	250	Drug	Miscl.	5
M14	AZD-8186	PI3Kbeta inhibitor	1	Drug	PI3K	5
M15	TAK-285	HER2 inhibitor	2.5	Drug	Miscl.	5
M16	Lucitanib	FGFR1, VEGFR inhibitor	10	Drug	Miscl.	5
M17	AT7519	CDK1, 2, 4, 6 and 9 inhibitor	10	Drug	CDK	5
M18	GSK-461364	PLK1 inhibitor	100	Drug	Miscl.	5
M19	Bentamapimod	JNK inhibitor	10	Drug	Miscl.	5
M20	ZSTK474	PI3K gamma selective inhibitor	10	Drug	PI3K	5
M21	AT13148	p70S6K, PKA, ROCK (AKT) inhibitor	10	Drug	Miscl.	5
M22	AZD1208	PIM1, 2, 3 kinase inhibitor	100	Drug	Miscl.	5

M23	Omipalisib	PI3K/mTOR inhibitor	1	Drug	PI3K	5
M24	cells	cells	None	cells	Negative Controls	5
N1	cells	cells	None	cells	Negative Controls	5
N2	Filgotinib	JAK1-selective inhibitor	100	Drug	Miscl.	5
N3	Merestinib	Met inhibitor	100	Drug	Miscl.	5
N4	Spebrutinib	BTK inhibitor	10	Drug	Miscl.	5
N5	ABC294640	Sphingosine kinase 2 inhibitor	500	Drug	Miscl.	5
N6	CC-223	mTOR inhibitor	100	Drug	Miscl.	5
N7	DMSO	DMSO	0	DMSO	Negative Controls	5
N8	Erdafitinib	FGFR inhibitor	10	Drug	Miscl.	5
N9	AMG-337	Met inhibitor	100	Drug	Miscl.	5
N10	Selonsertib	ASK1 inhibitor	10	Drug	Miscl.	5
N11	BzCl	BzCl	0	BzCl	Miscl.	5
N12	TAK-901	Aurora, Src family, JAK3, RTK inhibitor	10	Drug	Miscl.	5
N13	OSU-03012	PDPK1 inhibitor	2500	Drug	Miscl.	5
N14	AZD-8186	PI3Kbeta inhibitor	10	Drug	PI3K	5
N15	TAK-285	HER2 inhibitor	25	Drug	Miscl.	5
N16	Lucitanib	FGFR1, VEGFR inhibitor	100	Drug	Miscl.	5
N17	AT7519	CDK1, 2, 4, 6 and 9 inhibitor	100	Drug	CDK	5
N18	GSK-461364	PLK1 inhibitor	1000	Drug	Miscl.	5
N19	Bentamapimod	JNK inhibitor	100	Drug	Miscl.	5
N20	ZSTK474	PI3K gamma selective inhibitor	100	Drug	PI3K	5
N21	AT13148	p70S6K, PKA, ROCK (AKT) inhibitor	100	Drug	Miscl.	5
N22	AZD1208	PIM1, 2, 3 kinase inhibitor	1000	Drug	Miscl.	5
N23	Omipalisib	PI3K/mTOR inhibitor	10	Drug	PI3K	5
N24	cells	cells	None	cells	Negative Controls	5
O1	cells	cells	None	cells	Negative Controls	5
O2	Filgotinib	JAK1-selective inhibitor	1000	Drug	Miscl.	5
O3	Merestinib	Met inhibitor	1000	Drug	Miscl.	5
O4	Spebrutinib	BTK inhibitor	100	Drug	Miscl.	5
O5	ABC294640	Sphingosine kinase 2 inhibitor	5000	Drug	Miscl.	5
O6	CC-223	mTOR inhibitor	1000	Drug	Miscl.	5
O7	Olmutinib	EGFR(L858R/T790M) inhibitor	100	Drug	EGFR	5

O8	Erdafitinib	FGFR inhibitor	100	Drug	Miscl.	5
O9	AMG-337	Met inhibitor	1000	Drug	Miscl.	5
O10	Selonsertib	ASK1 inhibitor	100	Drug	Miscl.	5
O11	BMS-911543	JAK2 inhibitor	1000	Drug	Miscl.	5
O12	TAK-901	Aurora, Src family, JAK3, RTK inhibitor	100	Drug	Miscl.	5
O13	DMSO	DMSO	0	DMSO	Negative Controls	5
O14	AZD-8186	PI3Kbeta inhibitor	100	Drug	PI3K	5
O15	TAK-285	HER2 inhibitor	250	Drug	Miscl.	5
O16	Lucitanib	FGFR1, VEGFR inhibitor	1000	Drug	Miscl.	5
O17	AT7519	CDK1, 2, 4, 6 and 9 inhibitor	1000	Drug	CDK	5
O18	BzCl	BzCl	0	BzCl	Miscl.	5
O19	Bentamapimod	JNK inhibitor	1000	Drug	Miscl.	5
O20	ZSTK474	PI3K gamma selective inhibitor	1000	Drug	PI3K	5
O21	AT13148	p70S6K, PKA, ROCK (AKT) inhibitor	1000	Drug	Miscl.	5
O22	AZD1208	PIM1, 2, 3 kinase inhibitor	10000	Drug	Miscl.	5
O23	Omipalisib	PI3K/mTOR inhibitor	100	Drug	PI3K	5
O24	cells	cells	None	cells	Negative Controls	5
P1	cells	cells	None	cells	Negative Controls	5
P2	Filgotinib	JAK1-selective inhibitor	10000	Drug	Miscl.	5
P3	DMSO	DMSO	0	DMSO	Negative Controls	5
P4	Spebrutinib	BTK inhibitor	1000	Drug	Miscl.	5
P5	ABC294640	Sphingosine kinase 2 inhibitor	50000	Drug	Miscl.	5
P6	CC-223	mTOR inhibitor	10000	Drug	Miscl.	5
P7	Olmutinib	EGFR(L858R/T790M) inhibitor	1000	Drug	EGFR	5
P8	Erdafitinib	FGFR inhibitor	1000	Drug	Miscl.	5
P9	AMG-337	Met inhibitor	10000	Drug	Miscl.	5
P10	Selonsertib	ASK1 inhibitor	1000	Drug	Miscl.	5
P11	BMS-911543	JAK2 inhibitor	10000	Drug	Miscl.	5
P12	TAK-901	Aurora, Src family, JAK3, RTK inhibitor	1000	Drug	Miscl.	5
P13	OSU-03012	PDPK1 inhibitor	25000	Drug	Miscl.	5
P14	AZD-8186	PI3Kbeta inhibitor	1000	Drug	PI3K	5
P15	TAK-285	HER2 inhibitor	2500	Drug	Miscl.	5
P16	Lucitanib	FGFR1, VEGFR inhibitor	10000	Drug	Miscl.	5

P17	AT7519	CDK1, 2, 4, 6 and 9 inhibitor	10000	Drug	CDK	5
P18	GSK-461364	PLK1 inhibitor	10000	Drug	Miscl.	5
P19	Bentamapimod	JNK inhibitor	10000	Drug	Miscl.	5
P20	ZSTK474	PI3K gamma selective inhibitor	10000	Drug	PI3K	5
P21	AT13148	p70S6K, PKA, ROCK (AKT) inhibitor	10000	Drug	Miscl.	5
P22	DMSO	DMSO	0	DMSO	Negative Controls	5
P23	Omipalisib	PI3K/mTOR inhibitor	1000	Drug	PI3K	5
P24	BzCl	BzCl	0	BzCl	Miscl.	5
A1	cells	cells	None	cells	Negative Controls	6
A2	BzCl	BzCl	0	BzCl	Miscl.	6
A3	TEW-7197	TGF-β receptor ALK4/ALK5 inhibitor	2500	Drug	Miscl.	6
A4	Asciminib	Allosteric ABL inhibitor	1000	Drug	Miscl.	6
A5	CC-115	mTOR/DNA-PK inhibitor	10000	Drug	Miscl.	6
A6	Altiratinib	MET/Tie-2 inhibitor	10000	Drug	Miscl.	6
A7	LY3009120	pan-RAF inhibitor	10000	Drug	Miscl.	6
A8	TGX-221	PI3K beta selective inhibitor	10000	Drug	PI3K	6
A9	PHA 408	IKK-2 inhibitor	10000	Drug	Miscl.	6
A10	VE-821	ATR inihibitor	10000	Drug	Miscl.	6
A11	PF-4800567	CK1epsilon inhibitor	10000	Drug	Miscl.	6
A12	SCH772984	ERK1 & 2 inhibitor	10000	Drug	Miscl.	6
A13	AMG-925	FLT-3, CDK4 inhibitor	1000	Drug	Miscl.	6
A14	GSK2656157	PERK inhibitor	2500	Drug	Miscl.	6
A15	CCT196969	pan-RAF/Src inhibitor	25000	Drug	Miscl.	6
A16	KU-60019	ATM inhibitor	25000	Drug	Miscl.	6
A17	Senexin B	CDK8/19 inhibitor	1000	Drug	CDK	6
A18	Tasquinimod	S100A9 inhibitor, immunomodulatory, anti-angiogenic	10000	Drug	Miscl.	6
A19	ODM-201	AR antagonist	2500	Drug	Miscl.	6
A20	Indibulin	Mitoric inhibitor. Microtubule depolymerizer	10000	Drug	Miscl.	6
A21	Resiquimod	TLR7/TLR8 agonist	10000	Drug	Miscl.	6
A22	Vesatolimod	TLR7 agonist	10000	Drug	Miscl.	6
A23	BzCl	BzCl	0	BzCl	Miscl.	6
A24	cells	cells	None	cells	Negative Controls	6
B1	cells	cells	None	cells	Negative Controls	6

B2	Ravoxertinib	ERK inhibitor	10000	Drug	Miscl.	6
B3	TEW-7197	TGF-β receptor ALK4/ALK5 inhibitor	250	Drug	Miscl.	6
B4	Asciminib	Allosteric ABL inhibitor	100	Drug	Miscl.	6
B5	CC-115	mTOR/DNA-PK inhibitor	1000	Drug	Miscl.	6
B6	Altiratinib	MET/Tie-2 inhibitor	1000	Drug	Miscl.	6
B7	LY3009120	pan-RAF inhibitor	1000	Drug	Miscl.	6
B8	TGX-221	PI3K beta selective inhibitor	1000	Drug	PI3K	6
B9	DMSO	DMSO	0	DMSO	Negative Controls	6
B10	VE-821	ATR inihibitor	1000	Drug	Miscl.	6
B11	PF-4800567	CK1epsilon inhibitor	1000	Drug	Miscl.	6
B12	SCH772984	ERK1 & 2 inhibitor	1000	Drug	Miscl.	6
B13	AMG-925	FLT-3, CDK4 inhibitor	100	Drug	Miscl.	6
B14	GSK2656157	PERK inhibitor	250	Drug	Miscl.	6
B15	CCT196969	pan-RAF/Src inhibitor	2500	Drug	Miscl.	6
B16	BzCl	BzCl	0	BzCl	Miscl.	6
B17	Senexin B	CDK8/19 inhibitor	100	Drug	CDK	6
B18	Tasquinimod	S100A9 inhibitor, immunomodulatory, anti-angiogenic	1000	Drug	Miscl.	6
B19	ODM-201	AR antagonist	250	Drug	Miscl.	6
B20	Indibulin	Mitoric inhibitor. Microtubule depolymerizer	1000	Drug	Miscl.	6
B21	Resiquimod	TLR7/TLR8 agonist	1000	Drug	Miscl.	6
B22	Vesatolimod	TLR7 agonist	1000	Drug	Miscl.	6
B23	Oprozomib	proteasome (20 S) inhibitor	2500	Drug	Miscl.	6
B24	cells	cells	None	cells	Negative Controls	6
C1	cells	cells	None	cells	Negative Controls	6
C2	Ravoxertinib	ERK inhibitor	1000	Drug	Miscl.	6
C3	TEW-7197	TGF-β receptor ALK4/ALK5 inhibitor	25	Drug	Miscl.	6
C4	Asciminib	Allosteric ABL inhibitor	10	Drug	Miscl.	6
C5	CC-115	mTOR/DNA-PK inhibitor	100	Drug	Miscl.	6
C6	Altiratinib	MET/Tie-2 inhibitor	100	Drug	Miscl.	6
C7	LY3009120	pan-RAF inhibitor	100	Drug	Miscl.	6
C8	TGX-221	PI3K beta selective inhibitor	100	Drug	PI3K	6
C9	PHA 408	IKK-2 inhibitor	1000	Drug	Miscl.	6
C10	VE-821	ATR inihibitor	100	Drug	Miscl.	6

C11	PF-4800567	CK1epsilon inhibitor	100	Drug	Miscl.	6
C12	DMSO	DMSO	0	DMSO	Negative Controls	6
C13	AMG-925	FLT-3, CDK4 inhibitor	10	Drug	Miscl.	6
C14	GSK2656157	PERK inhibitor	25	Drug	Miscl.	6
C15	CCT196969	pan-RAF/Src inhibitor	250	Drug	Miscl.	6
C16	KU-60019	ATM inhibitor	2500	Drug	Miscl.	6
C17	Senexin B	CDK8/19 inhibitor	10	Drug	CDK	6
C18	Tasquinimod	S100A9 inhibitor, immunomodulatory, anti-angiogenic	100	Drug	Miscl.	6
C19	ODM-201	AR antagonist	25	Drug	Miscl.	6
C20	DMSO	DMSO	0	DMSO	Negative Controls	6
C21	Resiquimod	TLR7/TLR8 agonist	100	Drug	Miscl.	6
C22	Vesatolimod	TLR7 agonist	100	Drug	Miscl.	6
C23	Oprozomib	proteasome (20 S) inhibitor	250	Drug	Miscl.	6
C24	cells	cells	None	cells	Negative Controls	6
D1	cells	cells	None	cells	Negative Controls	6
D2	Ravoxertinib	ERK inhibitor	100	Drug	Miscl.	6
D3	TEW-7197	TGF-β receptor ALK4/ALK5 inhibitor	2.5	Drug	Miscl.	6
D4	Asciminib	Allosteric ABL inhibitor	1	Drug	Miscl.	6
D5	DMSO	DMSO	0	DMSO	Negative Controls	6
D6	Altiratinib	MET/Tie-2 inhibitor	10	Drug	Miscl.	6
D7	LY3009120	pan-RAF inhibitor	10	Drug	Miscl.	6
D8	TGX-221	PI3K beta selective inhibitor	10	Drug	PI3K	6
D9	PHA 408	IKK-2 inhibitor	100	Drug	Miscl.	6
D10	VE-821	ATR inihibitor	10	Drug	Miscl.	6
D11	PF-4800567	CK1epsilon inhibitor	10	Drug	Miscl.	6
D12	SCH772984	ERK1 & 2 inhibitor	100	Drug	Miscl.	6
D13	AMG-925	FLT-3, CDK4 inhibitor	1	Drug	Miscl.	6
D14	GSK2656157	PERK inhibitor	2.5	Drug	Miscl.	6
D15	CCT196969	pan-RAF/Src inhibitor	25	Drug	Miscl.	6
D16	KU-60019	ATM inhibitor	250	Drug	Miscl.	6
D17	Senexin B	CDK8/19 inhibitor	1	Drug	CDK	6
D18	Tasquinimod	S100A9 inhibitor, immunomodulatory, anti-angiogenic	10	Drug	Miscl.	6
D19	ODM-201	AR antagonist	2.5	Drug	Miscl.	6

D20	Indibulin	Mitoric inhibitor. Microtubule depolymerizer	100	Drug	Miscl.	6
D21	Resiquimod	TLR7/TLR8 agonist	10	Drug	Miscl.	6
D22	Vesatolimod	TLR7 agonist	10	Drug	Miscl.	6
D23	Oprozomib	proteasome (20 S) inhibitor	25	Drug	Miscl.	6
D24	cells	cells	None	cells	Negative Controls	6
E1	cells	cells	None	cells	Negative Controls	6
E2	Ravoxertinib	ERK inhibitor	10	Drug	Miscl.	6
E3	TEW-7197	TGF-β receptor ALK4/ALK5 inhibitor	0.25	Drug	Miscl.	6
E4	Asciminib	Allosteric ABL inhibitor	0.1	Drug	Miscl.	6
E5	CC-115	mTOR/DNA-PK inhibitor	10	Drug	Miscl.	6
E6	Altiratinib	MET/Tie-2 inhibitor	1	Drug	Miscl.	6
E7	LY3009120	pan-RAF inhibitor	1	Drug	Miscl.	6
E8	TGX-221	PI3K beta selective inhibitor	1	Drug	PI3K	6
E9	PHA 408	IKK-2 inhibitor	10	Drug	Miscl.	6
E10	VE-821	ATR inihibitor	1	Drug	Miscl.	6
E11	PF-4800567	CK1epsilon inhibitor	1	Drug	Miscl.	6
E12	SCH772984	ERK1 & 2 inhibitor	10	Drug	Miscl.	6
E13	AMG-925	FLT-3, CDK4 inhibitor	0.1	Drug	Miscl.	6
E14	GSK2656157	PERK inhibitor	0.25	Drug	Miscl.	6
E15	DMSO	DMSO	0	DMSO	Negative Controls	6
E16	KU-60019	ATM inhibitor	25	Drug	Miscl.	6
E17	Senexin B	CDK8/19 inhibitor	0.1	Drug	CDK	6
E18	Tasquinimod	S100A9 inhibitor, immunomodulatory, anti-angiogenic	1	Drug	Miscl.	6
E19	ODM-201	AR antagonist	0.25	Drug	Miscl.	6
E20	Indibulin	Mitoric inhibitor. Microtubule depolymerizer	10	Drug	Miscl.	6
E21	Resiquimod	TLR7/TLR8 agonist	1	Drug	Miscl.	6
E22	Vesatolimod	TLR7 agonist	1	Drug	Miscl.	6
E23	Oprozomib	proteasome (20 S) inhibitor	2.5	Drug	Miscl.	6
E24	cells	cells	None	cells	Negative Controls	6
F1	cells	cells	None	cells	Negative Controls	6
F2	Ravoxertinib	ERK inhibitor	1	Drug	Miscl.	6
F3	RO5126766	dual RAF/MEK inhibitor	1000	Drug	Miscl.	6
F4	Tirabrutinib	BTK inhibitor	1000	Drug	Miscl.	6

F5	CC-115	mTOR/DNA-PK inhibitor	1	Drug	Miscl.	6
F6	PIM-447	PIM1, 2, 3 kinase inhibitor	10000	Drug	Miscl.	6
F7	AZD0156	ATM inihibitor	1000	Drug	Miscl.	6
F8	BX-912	PDK1 inhib	10000	Drug	Miscl.	6
F9	PHA 408	IKK-2 inhibitor	1	Drug	Miscl.	6
F10	BzCl	BzCl	0	BzCl	Miscl.	6
F11	PF-670462	CK1epsilon and CK1delta inhibitor	10000	Drug	Miscl.	6
F12	SCH772984	ERK1 & 2 inhibitor	1	Drug	Miscl.	6
F13	GNE-0877	LRRK2 inhibitor	1000	Drug	Miscl.	6
F14	UNC2881	MER inhibitor	2500	Drug	Miscl.	6
F15	CCT196969	pan-RAF/Src inhibitor	2.5	Drug	Miscl.	6
F16	KU-60019	ATM inhibitor	2.5	Drug	Miscl.	6
F17	Orteronel	CYP17A1, androgen synth inhib.	10000	Drug	Miscl.	6
F18	Ridaforolimus	binds FKBP12, causes inhibition of mTORC1	100	Drug	Miscl.	6
F19	Resatorvid	TLR4 inhibitor	10000	Drug	Miscl.	6
F20	Indibulin	Mitoric inhibitor. Microtubule depolymerizer	1	Drug	Miscl.	6
F21	8-chloro-adenosine	Nucleoside analog, RNA synthesis inhibitor	50000	Drug	Miscl.	6
F22	VGX-1027	Nitric oxide-donating immunomodulator	10000	Drug	Miscl.	6
F23	Oprozomib	proteasome (20 S) inhibitor	0.25	Drug	Miscl.	6
F24	BzCl	BzCl	0	BzCl	Miscl.	6
G1	cells	cells	None	cells	Negative Controls	6
G2	Cerdulatinib	JAK, SYK inhibitor	10000	Drug	Miscl.	6
G3	RO5126766	dual RAF/MEK inhibitor	100	Drug	Miscl.	6
G4	Tirabrutinib	BTK inhibitor	100	Drug	Miscl.	6
G5	LY-2584702	p70S6K inhibitor	10000	Drug	Miscl.	6
G6	BzCl	BzCl	0	BzCl	Miscl.	6
G7	AZD0156	ATM inihibitor	100	Drug	Miscl.	6
G8	BX-912	PDK1 inhib	1000	Drug	Miscl.	6
G9	MK-8745	Aurora A inhibitor	10000	Drug	Miscl.	6
G10	GSK-2334470	PDK1 inhibitor	10000	Drug	Miscl.	6
G11	PF-670462	CK1epsilon and CK1delta inhibitor	1000	Drug	Miscl.	6
G12	AZD7545	PDHK inhibitor	10000	Drug	Miscl.	6
G13	GNE-0877	LRRK2 inhibitor	100	Drug	Miscl.	6

G14	UNC2881	MER inhibitor	250	Drug	Miscl.	6
G15	OTS-964	TOPK inhibitor	2500	Drug	Miscl.	6
G16	DEL-22379	ERK dimerization inhibitor	50000	Drug	Miscl.	6
G17	Orteronel	CYP17A1, androgen synth inhib.	1000	Drug	Miscl.	6
G18	Ridaforolimus	binds FKBP12, causes inhibition of mTORC1	10	Drug	Miscl.	6
G19	Resatorvid	TLR4 inhibitor	1000	Drug	Miscl.	6
G20	Apalutamide	AR antagonist	10000	Drug	Miscl.	6
G21	8-chloro-adenosine	Nucleoside analog, RNA synthesis inhibitor	5000	Drug	Miscl.	6
G22	VGX-1027	Nitric oxide-donating immunomodulator	1000	Drug	Miscl.	6
G23	8-amino-adenosine	Nucleoside analog, RNA synthesis inhibitor	50000	Drug	Miscl.	6
G24	cells	cells	None	cells	Negative Controls	6
H1	cells	cells	None	cells	Negative Controls	6
H2	Cerdulatinib	JAK, SYK inhibitor	1000	Drug	Miscl.	6
H3	RO5126766	dual RAF/MEK inhibitor	10	Drug	Miscl.	6
H4	Tirabrutinib	BTK inhibitor	10	Drug	Miscl.	6
H5	LY-2584702	p70S6K inhibitor	1000	Drug	Miscl.	6
H6	PIM-447	PIM1, 2, 3 kinase inhibitor	1000	Drug	Miscl.	6
H7	AZD0156	ATM inihibitor	10	Drug	Miscl.	6
H8	BX-912	PDK1 inhib	100	Drug	Miscl.	6
H9	MK-8745	Aurora A inhibitor	1000	Drug	Miscl.	6
H10	GSK-2334470	PDK1 inhibitor	1000	Drug	Miscl.	6
H11	PF-670462	CK1epsilon and CK1delta inhibitor	100	Drug	Miscl.	6
H12	AZD7545	PDHK inhibitor	1000	Drug	Miscl.	6
H13	GNE-0877	LRRK2 inhibitor	10	Drug	Miscl.	6
H14	UNC2881	MER inhibitor	25	Drug	Miscl.	6
H15	OTS-964	TOPK inhibitor	250	Drug	Miscl.	6
H16	DEL-22379	ERK dimerization inhibitor	5000	Drug	Miscl.	6
H17	Orteronel	CYP17A1, androgen synth inhib.	100	Drug	Miscl.	6
H18	Ridaforolimus	binds FKBP12, causes inhibition of mTORC1	1	Drug	Miscl.	6
H19	DMSO	DMSO	0	DMSO	Negative Controls	6
H20	Apalutamide	AR antagonist	1000	Drug	Miscl.	6
H21	8-chloro-adenosine	Nucleoside analog, RNA synthesis inhibitor	500	Drug	Miscl.	6
H22	VGX-1027	Nitric oxide-donating immunomodulator	100	Drug	Miscl.	6

H23	8-amino-adenosine	Nucleoside analog, RNA synthesis inhibitor	5000	Drug	Miscl.	6
H24	cells	cells	None	cells	Negative Controls	6
I1	cells	cells	None	cells	Negative Controls	6
I2	Cerdulatinib	JAK, SYK inhibitor	100	Drug	Miscl.	6
I3	RO5126766	dual RAF/MEK inhibitor	1	Drug	Miscl.	6
I4	Tirabrutinib	BTK inhibitor	1	Drug	Miscl.	6
I5	LY-2584702	p70S6K inhibitor	100	Drug	Miscl.	6
I6	PIM-447	PIM1, 2, 3 kinase inhibitor	100	Drug	Miscl.	6
I7	AZD0156	ATM inihibitor	1	Drug	Miscl.	6
I8	DMSO	DMSO	0	DMSO	Negative Controls	6
I9	MK-8745	Aurora A inhibitor	100	Drug	Miscl.	6
I10	GSK-2334470	PDK1 inhibitor	100	Drug	Miscl.	6
I11	PF-670462	CK1epsilon and CK1delta inhibitor	10	Drug	Miscl.	6
I12	AZD7545	PDHK inhibitor	100	Drug	Miscl.	6
I13	GNE-0877	LRRK2 inhibitor	1	Drug	Miscl.	6
I14	UNC2881	MER inhibitor	2.5	Drug	Miscl.	6
I15	OTS-964	TOPK inhibitor	25	Drug	Miscl.	6
I16	DEL-22379	ERK dimerization inhibitor	500	Drug	Miscl.	6
I17	Orteronel	CYP17A1, androgen synth inhib.	10	Drug	Miscl.	6
I18	Ridaforolimus	binds FKBP12, causes inhibition of mTORC1	0.1	Drug	Miscl.	6
I19	Resatorvid	TLR4 inhibitor	100	Drug	Miscl.	6
I20	Apalutamide	AR antagonist	100	Drug	Miscl.	6
I21	8-chloro-adenosine	Nucleoside analog, RNA synthesis inhibitor	50	Drug	Miscl.	6
I22	VGX-1027	Nitric oxide-donating immunomodulator	10	Drug	Miscl.	6
I23	8-amino-adenosine	Nucleoside analog, RNA synthesis inhibitor	500	Drug	Miscl.	6
I24	cells	cells	None	cells	Negative Controls	6
J1	cells	cells	None	cells	Negative Controls	6
J2	Cerdulatinib	JAK, SYK inhibitor	10	Drug	Miscl.	6
J3	RO5126766	dual RAF/MEK inhibitor	0.1	Drug	Miscl.	6
J4	Tirabrutinib	BTK inhibitor	0.1	Drug	Miscl.	6
J5	LY-2584702	p70S6K inhibitor	10	Drug	Miscl.	6
J6	PIM-447	PIM1, 2, 3 kinase inhibitor	10	Drug	Miscl.	6
J7	AZD0156	ATM inihibitor	0.1	Drug	Miscl.	6

J8	BX-912	PDK1 inhib	10	Drug	Miscl.	6
J9	MK-8745	Aurora A inhibitor	10	Drug	Miscl.	6
J10	GSK-2334470	PDK1 inhibitor	10	Drug	Miscl.	6
J11	PF-670462	CK1epsilon and CK1delta inhibitor	1	Drug	Miscl.	6
J12	AZD7545	PDHK inhibitor	10	Drug	Miscl.	6
J13	GNE-0877	LRRK2 inhibitor	0.1	Drug	Miscl.	6
J14	BzCl	BzCl	0	BzCl	Miscl.	6
J15	OTS-964	TOPK inhibitor	2.5	Drug	Miscl.	6
J16	DEL-22379	ERK dimerization inhibitor	50	Drug	Miscl.	6
J17	Orteronel	CYP17A1, androgen synth inhib.	1	Drug	Miscl.	6
J18	Ridaforolimus	binds FKBP12, causes inhibition of mTORC1	0.01	Drug	Miscl.	6
J19	Resatorvid	TLR4 inhibitor	10	Drug	Miscl.	6
J20	Apalutamide	AR antagonist	10	Drug	Miscl.	6
J21	8-chloro-adenosine	Nucleoside analog, RNA synthesis inhibitor	5	Drug	Miscl.	6
J22	VGX-1027	Nitric oxide-donating immunomodulator	1	Drug	Miscl.	6
J23	8-amino-adenosine	Nucleoside analog, RNA synthesis inhibitor	50	Drug	Miscl.	6
J24	cells	cells	None	cells	Negative Controls	6
K1	cells	cells	None	cells	Negative Controls	6
K2	Cerdulatinib	JAK, SYK inhibitor	1	Drug	Miscl.	6
K3	AZD6738	ATR inihibitor	2.5	Drug	Miscl.	6
K4	Sitravatinib	RET, TRK, PDGFR, VEGFR, KIT, DDR… inhibitor	0.25	Drug	Miscl.	6
K5	LY-2584702	p70S6K inhibitor	1	Drug	Miscl.	6
K6	PIM-447	PIM1, 2, 3 kinase inhibitor	1	Drug	Miscl.	6
K7	PF-04708671	p70S6K inhibitor	1	Drug	Miscl.	6
K8	BX-912	PDK1 inhib	1	Drug	Miscl.	6
K9	MK-8745	Aurora A inhibitor	1	Drug	Miscl.	6
K10	GSK-2334470	PDK1 inhibitor	1	Drug	Miscl.	6
K11	GSK269962	ROCK1 and ROCK2 inhibitor	1	Drug	Miscl.	6
K12	AZD7545	PDHK inhibitor	1	Drug	Miscl.	6
K13	GNE-7915	LRRK2 inhibitor	0.1	Drug	Miscl.	6
K14	UNC2881	MER inhibitor	0.25	Drug	Miscl.	6
K15	OTS-964	TOPK inhibitor	0.25	Drug	Miscl.	6
K16	DEL-22379	ERK dimerization inhibitor	5	Drug	Miscl.	6

K17	Aldoxorubicin	Topoisomerase II inhibitor, Albumin binding	0.1	Drug	Miscl.	6
K18	Epacadostat	IDO inhibitor	1	Drug	Miscl.	6
K19	Resatorvid	TLR4 inhibitor	1	Drug	Miscl.	6
K20	Apalutamide	AR antagonist	1	Drug	Miscl.	6
K21	BzCl	BzCl	0	BzCl	Miscl.	6
K22	GDC-0919	IDO inhibitor	1	Drug	Miscl.	6
K23	8-amino-adenosine	Nucleoside analog, RNA synthesis inhibitor	5	Drug	Miscl.	6
K24	cells	cells	None	cells	Negative Controls	6
L1	cells	cells	None	cells	Negative Controls	6
L2	VS-4718	FAK inhibitor	1	Drug	Miscl.	6
L3	AZD6738	ATR inihibitor	25	Drug	Miscl.	6
L4	Sitravatinib	RET, TRK, PDGFR, VEGFR, KIT, DDR... inhibitor	2.5	Drug	Miscl.	6
L5	BGB-283	pan-Raf inhibitor	1	Drug	Miscl.	6
L6	GDC-0084	PI3K/mTOR inhibitor	1	Drug	PI3K	6
L7	PF-04708671	p70S6K inhibitor	10	Drug	Miscl.	6
L8	PS-1145	IKK-2 inhibitor	2.5	Drug	Miscl.	6
L9	AZ191	DYRK1A inhibitor	1	Drug	Miscl.	6
L10	GSK650394	SGK1 & 2 inhibitor	1	Drug	Miscl.	6
L11	GSK269962	ROCK1 and ROCK2 inhibitor	10	Drug	Miscl.	6
L12	FRAX486	PAK1, 2, 3 inhibitor	0.5	Drug	Miscl.	6
L13	GNE-7915	LRRK2 inhibitor	1	Drug	Miscl.	6
L14	A-419259	HCK and other SRC family kinase inhibitor	1	Drug	Miscl.	6
L15	THZ2	CDK7 inhibitor	1	Drug	CDK	6
L16	NVP-BHG712	EphB4 inhibitor	1	Drug	Miscl.	6
L17	DMSO	DMSO	0	DMSO	Negative Controls	6
L18	Epacadostat	IDO inhibitor	10	Drug	Miscl.	6
L19	ABT-751	Mitotic inhibitor. Colchicine site binding microtubule depolymerizer.	1	Drug	Mitotic	6
L20	Motolimod	TLR8 agonist	1	Drug	Miscl.	6
L21	CC122	IMiD immunomodulator	1	Drug	Miscl.	6
L22	GDC-0919	IDO inhibitor	10	Drug	Miscl.	6
L23	4-hydroxytamoxifen	Selective estrogen receptor modulator	1	Drug	Miscl.	6
L24	cells	cells	None	cells	Negative Controls	6
M1	cells	cells	None	cells	Negative Controls	6

M2	VS-4718	FAK inhibitor	10	Drug	Miscl.	6
M3	AZD6738	ATR inihibitor	250	Drug	Miscl.	6
M4	BzCl	BzCl	0	BzCl	Miscl.	6
M5	BGB-283	pan-Raf inhibitor	10	Drug	Miscl.	6
M6	GDC-0084	PI3K/mTOR inhibitor	10	Drug	PI3K	6
M7	PF-04708671	p70S6K inhibitor	100	Drug	Miscl.	6
M8	PS-1145	IKK-2 inhibitor	25	Drug	Miscl.	6
M9	AZ191	DYRK1A inhibitor	10	Drug	Miscl.	6
M10	GSK650394	SGK1 & 2 inhibitor	10	Drug	Miscl.	6
M11	GSK269962	ROCK1 and ROCK2 inhibitor	100	Drug	Miscl.	6
M12	FRAX486	PAK1, 2, 3 inhibitor	5	Drug	Miscl.	6
M13	GNE-7915	LRRK2 inhibitor	10	Drug	Miscl.	6
M14	A-419259	HCK and other SRC family kinase inhibitor	10	Drug	Miscl.	6
M15	THZ2	CDK7 inhibitor	10	Drug	CDK	6
M16	NVP-BHG712	EphB4 inhibitor	10	Drug	Miscl.	6
M17	Aldoxorubicin	Topoisomerase II inhibitor, Albumin binding	1	Drug	Miscl.	6
M18	Epacadostat	IDO inhibitor	100	Drug	Miscl.	6
M19	ABT-751	Mitotic inhibitor. Colchicine site binding microtubule depolymerizer.	10	Drug	Mitotic	6
M20	Motolimod	TLR8 agonist	10	Drug	Miscl.	6
M21	CC122	IMiD immunomodulator	10	Drug	Miscl.	6
M22	GDC-0919	IDO inhibitor	100	Drug	Miscl.	6
M23	4-hydroxytamoxifen	Selective estrogen receptor modulator	10	Drug	Miscl.	6
M24	cells	cells	None	cells	Negative Controls	6
N1	cells	cells	None	cells	Negative Controls	6
N2	VS-4718	FAK inhibitor	100	Drug	Miscl.	6
N3	AZD6738	ATR inihibitor	2500	Drug	Miscl.	6
N4	Sitravatinib	RET, TRK, PDGFR, VEGFR, KIT, DDR... inhibitor	25	Drug	Miscl.	6
N5	BGB-283	pan-Raf inhibitor	100	Drug	Miscl.	6
N6	GDC-0084	PI3K/mTOR inhibitor	100	Drug	PI3K	6
N7	DMSO	DMSO	0	DMSO	Negative Controls	6
N8	PS-1145	IKK-2 inhibitor	250	Drug	Miscl.	6
N9	AZ191	DYRK1A inhibitor	100	Drug	Miscl.	6
N10	GSK650394	SGK1 & 2 inhibitor	100	Drug	Miscl.	6

N11	BzCl	BzCl	0	BzCl	Miscl.	6
N12	FRAX486	PAK1, 2, 3 inhibitor	50	Drug	Miscl.	6
N13	GNE-7915	LRRK2 inhibitor	100	Drug	Miscl.	6
N14	A-419259	HCK and other SRC family kinase inhibitor	100	Drug	Miscl.	6
N15	THZ2	CDK7 inhibitor	100	Drug	CDK	6
N16	NVP-BHG712	EphB4 inhibitor	100	Drug	Miscl.	6
N17	Aldoxorubicin	Topoisomerase II inhibitor, Albumin binding	10	Drug	Miscl.	6
N18	Epacadostat	IDO inhibitor	1000	Drug	Miscl.	6
N19	ABT-751	Mitotic inhibitor. Colchicine site binding microtubule depolymerizer.	100	Drug	Mitotic	6
N20	Motolimod	TLR8 agonist	100	Drug	Miscl.	6
N21	CC122	IMiD immunomodulator	100	Drug	Miscl.	6
N22	GDC-0919	IDO inhibitor	1000	Drug	Miscl.	6
N23	4-hydroxytamoxifen	Selective estrogen receptor modulator	100	Drug	Miscl.	6
N24	cells	cells	None	cells	Negative Controls	6
O1	cells	cells	None	cells	Negative Controls	6
O2	VS-4718	FAK inhibitor	1000	Drug	Miscl.	6
O3	AZD6738	ATR inihibitor	25000	Drug	Miscl.	6
O4	Sitravatinib	RET, TRK, PDGFR, VEGFR, KIT, DDR... inhibitor	250	Drug	Miscl.	6
O5	BGB-283	pan-Raf inhibitor	1000	Drug	Miscl.	6
O6	GDC-0084	PI3K/mTOR inhibitor	1000	Drug	PI3K	6
O7	PF-04708671	p70S6K inhibitor	1000	Drug	Miscl.	6
O8	PS-1145	IKK-2 inhibitor	2500	Drug	Miscl.	6
O9	AZ191	DYRK1A inhibitor	1000	Drug	Miscl.	6
O10	GSK650394	SGK1 & 2 inhibitor	1000	Drug	Miscl.	6
O11	GSK269962	ROCK1 and ROCK2 inhibitor	1000	Drug	Miscl.	6
O12	FRAX486	PAK1, 2, 3 inhibitor	500	Drug	Miscl.	6
O13	DMSO	DMSO	0	DMSO	Negative Controls	6
O14	A-419259	HCK and other SRC family kinase inhibitor	1000	Drug	Miscl.	6
O15	THZ2	CDK7 inhibitor	1000	Drug	CDK	6
O16	NVP-BHG712	EphB4 inhibitor	1000	Drug	Miscl.	6
O17	Aldoxorubicin	Topoisomerase II inhibitor, Albumin binding	100	Drug	Miscl.	6
O18	BzCl	BzCl	0	BzCl	Miscl.	6
O19	ABT-751	Mitotic inhibitor. Colchicine site binding microtubule depolymerizer.	1000	Drug	Mitotic	6

O20	Motolimod	TLR8 agonist	1000	Drug	Miscl.	6
O21	CC122	IMiD immunomodulator	1000	Drug	Miscl.	6
O22	GDC-0919	IDO inhibitor	10000	Drug	Miscl.	6
O23	4-hydroxytamoxifen	Selective estrogen receptor modulator	1000	Drug	Miscl.	6
O24	cells	cells	None	cells	Negative Controls	6
P1	cells	cells	None	cells	Negative Controls	6
P2	VS-4718	FAK inhibitor	10000	Drug	Miscl.	6
P3	DMSO	DMSO	0	DMSO	Negative Controls	6
P4	Sitravatinib	RET, TRK, PDGFR, VEGFR, KIT, DDR… inhibitor	2500	Drug	Miscl.	6
P5	BGB-283	pan-Raf inhibitor	10000	Drug	Miscl.	6
P6	GDC-0084	PI3K/mTOR inhibitor	10000	Drug	PI3K	6
P7	PF-04708671	p70S6K inhibitor	10000	Drug	Miscl.	6
P8	PS-1145	IKK-2 inhibitor	25000	Drug	Miscl.	6
P9	AZ191	DYRK1A inhibitor	10000	Drug	Miscl.	6
P10	GSK650394	SGK1 & 2 inhibitor	10000	Drug	Miscl.	6
P11	GSK269962	ROCK1 and ROCK2 inhibitor	10000	Drug	Miscl.	6
P12	FRAX486	PAK1, 2, 3 inhibitor	5000	Drug	Miscl.	6
P13	GNE-7915	LRRK2 inhibitor	1000	Drug	Miscl.	6
P14	A-419259	HCK and other SRC family kinase inhibitor	10000	Drug	Miscl.	6
P15	THZ2	CDK7 inhibitor	10000	Drug	CDK	6
P16	NVP-BHG712	EphB4 inhibitor	10000	Drug	Miscl.	6
P17	Aldoxorubicin	Topoisomerase II inhibitor, Albumin binding	1000	Drug	Miscl.	6
P18	Epacadostat	IDO inhibitor	10000	Drug	Miscl.	6
P19	ABT-751	Mitotic inhibitor. Colchicine site binding microtubule depolymerizer.	10000	Drug	Mitotic	6
P20	Motolimod	TLR8 agonist	10000	Drug	Miscl.	6
P21	CC122	IMiD immunomodulator	10000	Drug	Miscl.	6
P22	DMSO	DMSO	0	DMSO	Negative Controls	6
P23	4-hydroxytamoxifen	Selective estrogen receptor modulator	10000	Drug	Miscl.	6
P24	BzCl	BzCl	0	BzCl	Miscl.	6
A1	cells	cells	None	cells	Negative Controls	7
A2	BzCl	BzCl	0	BzCl	Miscl.	7
A3	Talazoparib	PARP1/2 inhibitor	1000	Drug	PARP	7
A4	Lonafarnib	Farnesyl transferase inhibitor	100000	Drug	Miscl.	7

A5	Mocetinostat	HDAC inhibitor (HDAC1 & 2-selective)	10000	Drug	HDAC	7
A6	Birinapant	IAPs, SMAC mimetic	1000	Drug	Miscl.	7
A7	CUDC-907	HDAC1/2/3/10, PI3Kalpha inhibitor	10000	Drug	HDAC	7
A8	AT 101	Bcl-2 family inhibitor	100000	Drug	Miscl.	7
A9	Givinostat	HDAC inhibitor	1000	Drug	HDAC	7
A10	Roxadustat	HIF prolyl hydroxylase inhibitor	10000	Drug	Miscl.	7
A11	Eltanexor	XPO1/CRM1 inhibitor	10000	Drug	Miscl.	7
A12	Rocilinostat	HDAC-6 selective inhibitor	10000	Drug	HDAC	7
A13	BAY 87-2243	HIF1alpha inhibitor	1000	Drug	Miscl.	7
A14	BAY-1436032	IDH1 R132H/R132C inhibitor	10000	Drug	Miscl.	7
A15	StemRegenin 1	AHR antagonist, stem cell regenerating	10000	Drug	Miscl.	7
A16	C646	p300/CREB-binding protein (CBP) inhibitor	25000	Drug	Miscl.	7
A17	SGC-CBP30	CREBBP/EP300 bromodomain inhibitor	25000	Drug	Miscl.	7
A18	GSK-J4	JMJD3 (histone demethylase) inhibitor	100000	Drug	Miscl.	7
A19	UNC0638	G9a/GLP inhibitor	10000	Drug	Miscl.	7
A20	EPZ015666	PRMT5 inhibitor	10000	Drug	Miscl.	7
A21	dBET1	BET-targeting PROTAC	10000	Drug	BET	7
A22	PFI-1	BET family inhibitor	30000	Drug	BET	7
A23	BzCl	BzCl	0	BzCl	Miscl.	7
A24	cells	cells	None	cells	Negative Controls	7
B1	cells	cells	None	cells	Negative Controls	7
B2	Veliparib	PARP inhibitor	10000	Drug	PARP	7
B3	Talazoparib	PARP1/2 inhibitor	100	Drug	PARP	7
B4	Lonafarnib	Farnesyl transferase inhibitor	10000	Drug	Miscl.	7
B5	Mocetinostat	HDAC inhibitor (HDAC1 & 2-selective)	1000	Drug	HDAC	7
B6	Birinapant	IAPs, SMAC mimetic	100	Drug	Miscl.	7
B7	CUDC-907	HDAC1/2/3/10, PI3Kalpha inhibitor	1000	Drug	HDAC	7
B8	AT 101	Bcl-2 family inhibitor	10000	Drug	Miscl.	7
B9	DMSO	DMSO	0	DMSO	Negative Controls	7
B10	Roxadustat	HIF prolyl hydroxylase inhibitor	1000	Drug	Miscl.	7
B11	Eltanexor	XPO1/CRM1 inhibitor	1000	Drug	Miscl.	7
B12	Rocilinostat	HDAC-6 selective inhibitor	1000	Drug	HDAC	7
B13	BAY 87-2243	HIF1alpha inhibitor	100	Drug	Miscl.	7

B14	BAY-1436032	IDH1 R132H/R132C inhibitor	1000	Drug	Miscl.	7
B15	StemRegenin 1	AHR antagonist, stem cell regenerating	1000	Drug	Miscl.	7
B16	BzCl	BzCl	0	BzCl	Miscl.	7
B17	SGC-CBP30	CREBBP/EP300 bromodomain inhibitor	2500	Drug	Miscl.	7
B18	GSK-J4	JMJD3 (histone demethylase) inhibitor	10000	Drug	Miscl.	7
B19	UNC0638	G9a/GLP inhibitor	1000	Drug	Miscl.	7
B20	EPZ015666	PRMT5 inhibitor	1000	Drug	Miscl.	7
B21	dBET1	BET-targeting PROTAC	1000	Drug	BET	7
B22	PFI-1	BET family inhibitor	3000	Drug	BET	7
B23	ML390	DHODH inhibitor	50000	Drug	Miscl.	7
B24	cells	cells	None	cells	Negative Controls	7
C1	cells	cells	None	cells	Negative Controls	7
C2	Veliparib	PARP inhibitor	1000	Drug	PARP	7
C3	Talazoparib	PARP1/2 inhibitor	10	Drug	PARP	7
C4	Lonafarnib	Farnesyl transferase inhibitor	1000	Drug	Miscl.	7
C5	Mocetinostat	HDAC inhibitor (HDAC1 & 2-selective)	100	Drug	HDAC	7
C6	Birinapant	IAPs, SMAC mimetic	10	Drug	Miscl.	7
C7	CUDC-907	HDAC1/2/3/10, PI3Kalpha inhibitor	100	Drug	HDAC	7
C8	AT 101	Bcl-2 family inhibitor	1000	Drug	Miscl.	7
C9	Givinostat	HDAC inhibitor	100	Drug	HDAC	7
C10	Roxadustat	HIF prolyl hydroxylase inhibitor	100	Drug	Miscl.	7
C11	Eltanexor	XPO1/CRM1 inhibitor	100	Drug	Miscl.	7
C12	DMSO	DMSO	0	DMSO	Negative Controls	7
C13	BAY 87-2243	HIF1alpha inhibitor	10	Drug	Miscl.	7
C14	BAY-1436032	IDH1 R132H/R132C inhibitor	100	Drug	Miscl.	7
C15	StemRegenin 1	AHR antagonist, stem cell regenerating	100	Drug	Miscl.	7
C16	C646	p300/CREB-binding protein (CBP) inhibitor	2500	Drug	Miscl.	7
C17	SGC-CBP30	CREBBP/EP300 bromodomain inhibitor	250	Drug	Miscl.	7
C18	GSK-J4	JMJD3 (histone demethylase) inhibitor	1000	Drug	Miscl.	7
C19	UNC0638	G9a/GLP inhibitor	100	Drug	Miscl.	7
C20	DMSO	DMSO	0	DMSO	Negative Controls	7
C21	dBET1	BET-targeting PROTAC	100	Drug	BET	7
C22	PFI-1	BET family inhibitor	300	Drug	BET	7

C23	ML390	DHODH inhibitor	5000	Drug	Miscl.	7
C24	cells	cells	None	cells	Negative Controls	7
D1	cells	cells	None	cells	Negative Controls	7
D2	Veliparib	PARP inhibitor	100	Drug	PARP	7
D3	Talazoparib	PARP1/2 inhibitor	1	Drug	PARP	7
D4	Lonafarnib	Farnesyl transferase inhibitor	100	Drug	Miscl.	7
D5	DMSO	DMSO	0	DMSO	Negative Controls	7
D6	Birinapant	IAPs, SMAC mimetic	1	Drug	Miscl.	7
D7	CUDC-907	HDAC1/2/3/10, PI3Kalpha inhibitor	10	Drug	HDAC	7
D8	AT 101	Bcl-2 family inhibitor	100	Drug	Miscl.	7
D9	Givinostat	HDAC inhibitor	10	Drug	HDAC	7
D10	Roxadustat	HIF prolyl hydroxylase inhibitor	10	Drug	Miscl.	7
D11	Eltanexor	XPO1/CRM1 inhibitor	10	Drug	Miscl.	7
D12	Rocilinostat	HDAC-6 selective inhibitor	100	Drug	HDAC	7
D13	BAY 87-2243	HIF1alpha inhibitor	1	Drug	Miscl.	7
D14	BAY-1436032	IDH1 R132H/R132C inhibitor	10	Drug	Miscl.	7
D15	StemRegenin 1	AHR antagonist, stem cell regenerating	10	Drug	Miscl.	7
D16	C646	p300/CREB-binding protein (CBP) inhibitor	250	Drug	Miscl.	7
D17	SGC-CBP30	CREBBP/EP300 bromodomain inhibitor	25	Drug	Miscl.	7
D18	GSK-J4	JMJD3 (histone demethylase) inhibitor	100	Drug	Miscl.	7
D19	UNC0638	G9a/GLP inhibitor	10	Drug	Miscl.	7
D20	EPZ015666	PRMT5 inhibitor	100	Drug	Miscl.	7
D21	dBET1	BET-targeting PROTAC	10	Drug	BET	7
D22	PFI-1	BET family inhibitor	30	Drug	BET	7
D23	ML390	DHODH inhibitor	500	Drug	Miscl.	7
D24	cells	cells	None	cells	Negative Controls	7
E1	cells	cells	None	cells	Negative Controls	7
E2	Veliparib	PARP inhibitor	10	Drug	PARP	7
E3	Talazoparib	PARP1/2 inhibitor	0.1	Drug	PARP	7
E4	Lonafarnib	Farnesyl transferase inhibitor	10	Drug	Miscl.	7
E5	Mocetinostat	HDAC inhibitor (HDAC1 & 2-selective)	10	Drug	HDAC	7
E6	Birinapant	IAPs, SMAC mimetic	0.1	Drug	Miscl.	7
E7	CUDC-907	HDAC1/2/3/10, PI3Kalpha inhibitor	1	Drug	HDAC	7

E8	AT 101	Bcl-2 family inhibitor	10	Drug	Miscl.	7
E9	Givinostat	HDAC inhibitor	1	Drug	HDAC	7
E10	Roxadustat	HIF prolyl hydroxylase inhibitor	1	Drug	Miscl.	7
E11	Eltanexor	XPO1/CRM1 inhibitor	1	Drug	Miscl.	7
E12	Rocilinostat	HDAC-6 selective inhibitor	10	Drug	HDAC	7
E13	BAY 87-2243	HIF1alpha inhibitor	0.1	Drug	Miscl.	7
E14	BAY-1436032	IDH1 R132H/R132C inhibitor	1	Drug	Miscl.	7
E15	DMSO	DMSO	0	DMSO	Negative Controls	7
E16	C646	p300/CREB-binding protein (CBP) inhibitor	25	Drug	Miscl.	7
E17	SGC-CBP30	CREBBP/EP300 bromodomain inhibitor	2.5	Drug	Miscl.	7
E18	GSK-J4	JMJD3 (histone demethylase) inhibitor	10	Drug	Miscl.	7
E19	UNC0638	G9a/GLP inhibitor	1	Drug	Miscl.	7
E20	EPZ015666	PRMT5 inhibitor	10	Drug	Miscl.	7
E21	dBET1	BET-targeting PROTAC	1	Drug	BET	7
E22	PFI-1	BET family inhibitor	3	Drug	BET	7
E23	ML390	DHODH inhibitor	50	Drug	Miscl.	7
E24	cells	cells	None	cells	Negative Controls	7
F1	cells	cells	None	cells	Negative Controls	7
F2	Veliparib	PARP inhibitor	1	Drug	PARP	7
F3	Idasanutlin	MDM2 inhibitor	10000	Drug	Miscl.	7
F4	Sepantronium bromide	Survivin inhibitor	10000	Drug	Miscl.	7
F5	Mocetinostat	HDAC inhibitor (HDAC1 & 2-selective)	1	Drug	HDAC	7
F6	Selinexor	CRM1 inhibitor	10000	Drug	Miscl.	7
F7	Resminostat	HDAC1, 3, 6 inhibitor	10000	Drug	HDAC	7
F8	AMG-232	MDM2 inhibitor	10000	Drug	Miscl.	7
F9	Givinostat	HDAC inhibitor	0.1	Drug	HDAC	7
F10	BzCl	BzCl	0	BzCl	Miscl.	7
F11	Vidofludimus	DHODH inhibitor	10000	Drug	Miscl.	7
F12	Rocilinostat	HDAC-6 selective inhibitor	1	Drug	HDAC	7
F13	GSK2879552	LSD1 inhibitor	100000	Drug	Miscl.	7
F14	XAV-939	Tankyrase-1 and -2	10000	Drug	Miscl.	7
F15	StemRegenin 1	AHR antagonist, stem cell regenerating	1	Drug	Miscl.	7
F16	C646	p300/CREB-binding protein (CBP) inhibitor	2.5	Drug	Miscl.	7

F17	IOX-1	2-Oxoglutarate Oxygenase Inhibitor	100000	Drug	Miscl.	7
F18	UNC1215	L3MBTL3 inhibitor	10000	Drug	Miscl.	7
F19	PCI-34051	HDAC8 inhibitor	10000	Drug	HDAC	7
F20	EPZ015666	PRMT5 inhibitor	1	Drug	Miscl.	7
F21	GSK2801	BAZ2B/A bromodomain inhibitor	10000	Drug	Miscl.	7
F22	A-366	G9a/GLP inhibitor	25000	Drug	Miscl.	7
F23	ML390	DHODH inhibitor	5	Drug	Miscl.	7
F24	cells	cells	None	cells	Negative Controls	7
G1	cells	cells	None	cells	Negative Controls	7
G2	Niraparib	PARP inhibitor	10000	Drug	PARP	7
G3	Idasanutlin	MDM2 inhibitor	1000	Drug	Miscl.	7
G4	Sepantronium bromide	Survivin inhibitor	1000	Drug	Miscl.	7
G5	Lomeguatrib	O6-methylguanine-DNA methyltransferase inhibitor	10000	Drug	Miscl.	7
G6	BzCl	BzCl	0	BzCl	Miscl.	7
G7	Resminostat	HDAC1, 3, 6 inhibitor	1000	Drug	HDAC	7
G8	AMG-232	MDM2 inhibitor	1000	Drug	Miscl.	7
G9	Enasidenib	IDH2-R140Q inhibitor	10000	Drug	Miscl.	7
G10	Birabresib	BET family inhibitor	10000	Drug	BET	7
G11	Vidofludimus	DHODH inhibitor	1000	Drug	Miscl.	7
G12	Pinometostat	DOT1L inhibitor	1000	Drug	Miscl.	7
G13	GSK2879552	LSD1 inhibitor	10000	Drug	Miscl.	7
G14	XAV-939	Tankyrase-1 and -2	1000	Drug	Miscl.	7
G15	I-BET151	BET family inhibitor	10000	Drug	BET	7
G16	IOX-2	PHD2 inhibitor	50000	Drug	Miscl.	7
G17	IOX-1	2-Oxoglutarate Oxygenase Inhibitor	10000	Drug	Miscl.	7
G18	UNC1215	L3MBTL3 inhibitor	1000	Drug	Miscl.	7
G19	PCI-34051	HDAC8 inhibitor	1000	Drug	HDAC	7
G20	UM729	Enhancer of aryl hydrocarbon receptor (AhR) antagonists	10000	Drug	Miscl.	7
G21	GSK2801	BAZ2B/A bromodomain inhibitor	1000	Drug	Miscl.	7
G22	A-366	G9a/GLP inhibitor	2500	Drug	Miscl.	7
G23	GSK343	EZH2 inhibitor	1000	Drug	Miscl.	7
G24	BzCl	BzCl	0	BzCl	Miscl.	7
H1	cells	cells	None	cells	Negative Controls	7

H2	Niraparib	PARP inhibitor	1000	Drug	PARP	7
H3	Idasanutlin	MDM2 inhibitor	100	Drug	Miscl.	7
H4	Sepantronium bromide	Survivin inhibitor	100	Drug	Miscl.	7
H5	Lomeguatrib	O6-methylguanine-DNA methyltransferase inhibitor	1000	Drug	Miscl.	7
H6	Selinexor	CRM1 inhibitor	1000	Drug	Miscl.	7
H7	Resminostat	HDAC1, 3, 6 inhibitor	100	Drug	HDAC	7
H8	AMG-232	MDM2 inhibitor	100	Drug	Miscl.	7
H9	Enasidenib	IDH2-R140Q inhibitor	1000	Drug	Miscl.	7
H10	Birabresib	BET family inhibitor	1000	Drug	BET	7
H11	Vidofludimus	DHODH inhibitor	100	Drug	Miscl.	7
H12	Pinometostat	DOT1L inhibitor	100	Drug	Miscl.	7
H13	GSK2879552	LSD1 inhibitor	1000	Drug	Miscl.	7
H14	XAV-939	Tankyrase-1 and -2	100	Drug	Miscl.	7
H15	I-BET151	BET family inhibitor	1000	Drug	BET	7
H16	IOX-2	PHD2 inhibitor	5000	Drug	Miscl.	7
H17	IOX-1	2-Oxoglutarate Oxygenase Inhibitor	1000	Drug	Miscl.	7
H18	UNC1215	L3MBTL3 inhibitor	100	Drug	Miscl.	7
H19	DMSO	DMSO	0	DMSO	Negative Controls	7
H20	UM729	Enhancer of aryl hydrocarbon receptor (AhR) antagonists	1000	Drug	Miscl.	7
H21	GSK2801	BAZ2B/A bromodomain inhibitor	100	Drug	Miscl.	7
H22	A-366	G9a/GLP inhibitor	250	Drug	Miscl.	7
H23	GSK343	EZH2 inhibitor	100	Drug	Miscl.	7
H24	cells	cells	None	cells	Negative Controls	7
I1	cells	cells	None	cells	Negative Controls	7
I2	Niraparib	PARP inhibitor	100	Drug	PARP	7
I3	Idasanutlin	MDM2 inhibitor	10	Drug	Miscl.	7
I4	Sepantronium bromide	Survivin inhibitor	10	Drug	Miscl.	7
I5	Lomeguatrib	O6-methylguanine-DNA methyltransferase inhibitor	100	Drug	Miscl.	7
I6	Selinexor	CRM1 inhibitor	100	Drug	Miscl.	7
I7	Resminostat	HDAC1, 3, 6 inhibitor	10	Drug	HDAC	7
I8	DMSO	DMSO	0	DMSO	Negative Controls	7
I9	Enasidenib	IDH2-R140Q inhibitor	100	Drug	Miscl.	7
I10	Birabresib	BET family inhibitor	100	Drug	BET	7

I11	Vidofludimus	DHODH inhibitor	10	Drug	Miscl.	7
I12	Pinometostat	DOT1L inhibitor	10	Drug	Miscl.	7
I13	GSK2879552	LSD1 inhibitor	100	Drug	Miscl.	7
I14	XAV-939	Tankyrase-1 and -2	10	Drug	Miscl.	7
I15	I-BET151	BET family inhibitor	100	Drug	BET	7
I16	IOX-2	PHD2 inhibitor	500	Drug	Miscl.	7
I17	IOX-1	2-Oxoglutarate Oxygenase Inhibitor	100	Drug	Miscl.	7
I18	UNC1215	L3MBTL3 inhibitor	10	Drug	Miscl.	7
I19	PCI-34051	HDAC8 inhibitor	100	Drug	HDAC	7
I20	UM729	Enhancer of aryl hydrocarbon receptor (AhR) antagonists	100	Drug	Miscl.	7
I21	GSK2801	BAZ2B/A bromodomain inhibitor	10	Drug	Miscl.	7
I22	A-366	G9a/GLP inhibitor	25	Drug	Miscl.	7
I23	GSK343	EZH2 inhibitor	10	Drug	Miscl.	7
I24	cells	cells	None	cells	Negative Controls	7
J1	cells	cells	None	cells	Negative Controls	7
J2	Niraparib	PARP inhibitor	10	Drug	PARP	7
J3	Idasanutlin	MDM2 inhibitor	1	Drug	Miscl.	7
J4	Sepantronium bromide	Survivin inhibitor	1	Drug	Miscl.	7
J5	Lomeguatrib	O6-methylguanine-DNA methyltransferase inhibitor	10	Drug	Miscl.	7
J6	Selinexor	CRM1 inhibitor	10	Drug	Miscl.	7
J7	Resminostat	HDAC1, 3, 6 inhibitor	1	Drug	HDAC	7
J8	AMG-232	MDM2 inhibitor	10	Drug	Miscl.	7
J9	Enasidenib	IDH2-R140Q inhibitor	10	Drug	Miscl.	7
J10	Birabresib	BET family inhibitor	10	Drug	BET	7
J11	Vidofludimus	DHODH inhibitor	1	Drug	Miscl.	7
J12	Pinometostat	DOT1L inhibitor	1	Drug	Miscl.	7
J13	GSK2879552	LSD1 inhibitor	10	Drug	Miscl.	7
J14	BzCl	BzCl	0	BzCl	Miscl.	7
J15	I-BET151	BET family inhibitor	10	Drug	BET	7
J16	IOX-2	PHD2 inhibitor	50	Drug	Miscl.	7
J17	IOX-1	2-Oxoglutarate Oxygenase Inhibitor	10	Drug	Miscl.	7
J18	UNC1215	L3MBTL3 inhibitor	1	Drug	Miscl.	7
J19	PCI-34051	HDAC8 inhibitor	10	Drug	HDAC	7

J20	UM729	Enhancer of aryl hydrocarbon receptor (AhR) antagonists	10	Drug	Miscl.	7
J21	GSK2801	BAZ2B/A bromodomain inhibitor	1	Drug	Miscl.	7
J22	A-366	G9a/GLP inhibitor	2.5	Drug	Miscl.	7
J23	GSK343	EZH2 inhibitor	1	Drug	Miscl.	7
J24	cells	cells	None	cells	Negative Controls	7
K1	cells	cells	None	cells	Negative Controls	7
K2	Niraparib	PARP inhibitor	1	Drug	PARP	7
K3	Tipifarnib	Farnesyltransferase inhibitor	1	Drug	Miscl.	7
K4	Entinostat	HDAC inhibitor	1	Drug	HDAC	7
K5	Lomeguatrib	O6-methylguanine-DNA methyltransferase inhibitor	1	Drug	Miscl.	7
K6	Selinexor	CRM1 inhibitor	1	Drug	Miscl.	7
K7	NVP-LCL161	IAPs, SMAC mimetic	2.5	Drug	Miscl.	7
K8	AMG-232	MDM2 inhibitor	1	Drug	Miscl.	7
K9	Enasidenib	IDH2-R140Q inhibitor	1	Drug	Miscl.	7
K10	Birabresib	BET family inhibitor	1	Drug	BET	7
K11	AR-42	HDAC inhibitor	1	Drug	HDAC	7
K12	Pinometostat	DOT1L inhibitor	0.1	Drug	Miscl.	7
K13	Mivebresib	BET family inhibitor	1	Drug	BET	7
K14	XAV-939	Tankyrase-1 and -2	1	Drug	Miscl.	7
K15	I-BET151	BET family inhibitor	1	Drug	BET	7
K16	IOX-2	PHD2 inhibitor	5	Drug	Miscl.	7
K17	SGC0946	DOT1L inhibitor	1	Drug	Miscl.	7
K18	Tubacin	HDAC6 inhibitor	1	Drug	HDAC	7
K19	PCI-34051	HDAC8 inhibitor	1	Drug	HDAC	7
K20	UM729	Enhancer of aryl hydrocarbon receptor (AhR) antagonists	1	Drug	Miscl.	7
K21	BzCl	BzCl	0	BzCl	Miscl.	7
K22	EPZ031686	SMYD3 inhibitor	1	Drug	Miscl.	7
K23	GSK343	EZH2 inhibitor	0.1	Drug	Miscl.	7
K24	cells	cells	None	cells	Negative Controls	7
L1	cells	cells	None	cells	Negative Controls	7
L2	Tacedinaline	HDAC inhibitor	0.1	Drug	HDAC	7
L3	Tipifarnib	Farnesyltransferase inhibitor	10	Drug	Miscl.	7
L4	Entinostat	HDAC inhibitor	10	Drug	HDAC	7

L5	Pracinostat	HDAC inhibitor	1	Drug	HDAC	7
L6	Tazemetostat	EZH2 inhibitor	1	Drug	Miscl.	7
L7	NVP-LCL161	IAPs, SMAC mimetic	25	Drug	Miscl.	7
L8	Abexinostat	HDAC1-selective inhibitor	1	Drug	HDAC	7
L9	Ivosidenib	IDH1 R132H/R132C inhibitor	1	Drug	Miscl.	7
L10	Tucidinostat	HDAC1/2/3/10 inhibitor	1	Drug	HDAC	7
L11	AR-42	HDAC inhibitor	10	Drug	HDAC	7
L12	Molibresib	BET family inhibitor	1	Drug	BET	7
L13	Mivebresib	BET family inhibitor	10	Drug	BET	7
L14	Tubastatin A	HDAC6 inhibitor	1	Drug	HDAC	7
L15	EPZ-5687	EZH2 inhibitor	1	Drug	Miscl.	7
L16	RGFP966	HDAC3 inhibitor	1	Drug	HDAC	7
L17	DMSO	DMSO	0	DMSO	Negative Controls	7
L18	Tubacin	HDAC6 inhibitor	10	Drug	HDAC	7
L19	PTC-209	BMI-1 inhibitor	1	Drug	Miscl.	7
L20	JQ1	BET family inhibitor	1	Drug	BET	7
L21	CPI-360	EZH2 inhibitor	1	Drug	Miscl.	7
L22	EPZ031686	SMYD3 inhibitor	10	Drug	Miscl.	7
L23	ARV-825	BET-targeting PROTAC	0.03	Drug	BET	7
L24	cells	cells	None	cells	Negative Controls	7
M1	cells	cells	None	cells	Negative Controls	7
M2	Tacedinaline	HDAC inhibitor	1	Drug	HDAC	7
M3	Tipifarnib	Farnesyltransferase inhibitor	100	Drug	Miscl.	7
M4	BzCl	BzCl	0	BzCl	Miscl.	7
M5	Pracinostat	HDAC inhibitor	10	Drug	HDAC	7
M6	Tazemetostat	EZH2 inhibitor	10	Drug	Miscl.	7
M7	NVP-LCL161	IAPs, SMAC mimetic	250	Drug	Miscl.	7
M8	Abexinostat	HDAC1-selective inhibitor	10	Drug	HDAC	7
M9	Ivosidenib	IDH1 R132H/R132C inhibitor	10	Drug	Miscl.	7
M10	Tucidinostat	HDAC1/2/3/10 inhibitor	10	Drug	HDAC	7
M11	AR-42	HDAC inhibitor	100	Drug	HDAC	7
M12	Molibresib	BET family inhibitor	10	Drug	BET	7
M13	Mivebresib	BET family inhibitor	100	Drug	BET	7

M14	Tubastatin A	HDAC6 inhibitor	10	Drug	HDAC	7
M15	EPZ-5687	EZH2 inhibitor	10	Drug	Miscl.	7
M16	RGFP966	HDAC3 inhibitor	10	Drug	HDAC	7
M17	SGC0946	DOT1L inhibitor	10	Drug	Miscl.	7
M18	Tubacin	HDAC6 inhibitor	100	Drug	HDAC	7
M19	PTC-209	BMI-1 inhibitor	10	Drug	Miscl.	7
M20	JQ1	BET family inhibitor	10	Drug	BET	7
M21	CPI-360	EZH2 inhibitor	10	Drug	Miscl.	7
M22	EPZ031686	SMYD3 inhibitor	100	Drug	Miscl.	7
M23	ARV-825	BET-targeting PROTAC	0.3	Drug	BET	7
M24	cells	cells	None	cells	Negative Controls	7
N1	cells	cells	None	cells	Negative Controls	7
N2	Tacedinaline	HDAC inhibitor	10	Drug	HDAC	7
N3	Tipifarnib	Farnesyltransferase inhibitor	1000	Drug	Miscl.	7
N4	Entinostat	HDAC inhibitor	100	Drug	HDAC	7
N5	Pracinostat	HDAC inhibitor	100	Drug	HDAC	7
N6	Tazemetostat	EZH2 inhibitor	100	Drug	Miscl.	7
N7	DMSO	DMSO	0	DMSO	Negative Controls	7
N8	Abexinostat	HDAC1-selective inhibitor	100	Drug	HDAC	7
N9	Ivosidenib	IDH1 R132H/R132C inhibitor	100	Drug	Miscl.	7
N10	Tucidinostat	HDAC1/2/3/10 inhibitor	100	Drug	HDAC	7
N11	BzCl	BzCl	0	BzCl	Miscl.	7
N12	Molibresib	BET family inhibitor	100	Drug	BET	7
N13	Mivebresib	BET family inhibitor	1000	Drug	BET	7
N14	Tubastatin A	HDAC6 inhibitor	100	Drug	HDAC	7
N15	EPZ-5687	EZH2 inhibitor	100	Drug	Miscl.	7
N16	RGFP966	HDAC3 inhibitor	100	Drug	HDAC	7
N17	SGC0946	DOT1L inhibitor	100	Drug	Miscl.	7
N18	Tubacin	HDAC6 inhibitor	1000	Drug	HDAC	7
N19	PTC-209	BMI-1 inhibitor	100	Drug	Miscl.	7
N20	JQ1	BET family inhibitor	100	Drug	BET	7
N21	CPI-360	EZH2 inhibitor	100	Drug	Miscl.	7
N22	EPZ031686	SMYD3 inhibitor	1000	Drug	Miscl.	7

N23	ARV-825	BET-targeting PROTAC	3	Drug	BET	7
N24	cells	cells	None	cells	Negative Controls	7
O1	cells	cells	None	cells	Negative Controls	7
O2	Tacedinaline	HDAC inhibitor	100	Drug	HDAC	7
O3	Tipifarnib	Farnesyltransferase inhibitor	10000	Drug	Miscl.	7
O4	Entinostat	HDAC inhibitor	1000	Drug	HDAC	7
O5	Pracinostat	HDAC inhibitor	1000	Drug	HDAC	7
O6	Tazemetostat	EZH2 inhibitor	1000	Drug	Miscl.	7
O7	NVP-LCL161	IAPs, SMAC mimetic	2500	Drug	Miscl.	7
O8	Abexinostat	HDAC1-selective inhibitor	1000	Drug	HDAC	7
O9	Ivosidenib	IDH1 R132H/R132C inhibitor	1000	Drug	Miscl.	7
O10	Tucidinostat	HDAC1/2/3/10 inhibitor	1000	Drug	HDAC	7
O11	AR-42	HDAC inhibitor	1000	Drug	HDAC	7
O12	Molibresib	BET family inhibitor	1000	Drug	BET	7
O13	DMSO	DMSO	0	DMSO	Negative Controls	7
O14	Tubastatin A	HDAC6 inhibitor	1000	Drug	HDAC	7
O15	EPZ-5687	EZH2 inhibitor	1000	Drug	Miscl.	7
O16	RGFP966	HDAC3 inhibitor	1000	Drug	HDAC	7
O17	SGC0946	DOT1L inhibitor	1000	Drug	Miscl.	7
O18	BzCl	BzCl	0	BzCl	Miscl.	7
O19	PTC-209	BMI-1 inhibitor	1000	Drug	Miscl.	7
O20	JQ1	BET family inhibitor	1000	Drug	BET	7
O21	CPI-360	EZH2 inhibitor	1000	Drug	Miscl.	7
O22	EPZ031686	SMYD3 inhibitor	10000	Drug	Miscl.	7
O23	ARV-825	BET-targeting PROTAC	30	Drug	BET	7
O24	cells	cells	None	cells	Negative Controls	7
P1	cells	cells	None	cells	Negative Controls	7
P2	Tacedinaline	HDAC inhibitor	1000	Drug	HDAC	7
P3	DMSO	DMSO	0	DMSO	Negative Controls	7
P4	Entinostat	HDAC inhibitor	10000	Drug	HDAC	7
P5	Pracinostat	HDAC inhibitor	10000	Drug	HDAC	7
P6	Tazemetostat	EZH2 inhibitor	10000	Drug	Miscl.	7
P7	NVP-LCL161	IAPs, SMAC mimetic	25000	Drug	Miscl.	7

P8	Abexinostat	HDAC1-selective inhibitor	10000	Drug	HDAC	7
P9	Ivosidenib	IDH1 R132H/R132C inhibitor	10000	Drug	Miscl.	7
P10	Tucidinostat	HDAC1/2/3/10 inhibitor	10000	Drug	HDAC	7
P11	AR-42	HDAC inhibitor	10000	Drug	HDAC	7
P12	Molibresib	BET family inhibitor	10000	Drug	BET	7
P13	Mivebresib	BET family inhibitor	10000	Drug	BET	7
P14	Tubastatin A	HDAC6 inhibitor	10000	Drug	HDAC	7
P15	EPZ-5687	EZH2 inhibitor	10000	Drug	Miscl.	7
P16	RGFP966	HDAC3 inhibitor	10000	Drug	HDAC	7
P17	SGC0946	DOT1L inhibitor	10000	Drug	Miscl.	7
P18	Tubacin	HDAC6 inhibitor	10000	Drug	HDAC	7
P19	PTC-209	BMI-1 inhibitor	10000	Drug	Miscl.	7
P20	JQ1	BET family inhibitor	10000	Drug	BET	7
P21	CPI-360	EZH2 inhibitor	10000	Drug	Miscl.	7
P22	DMSO	DMSO	0	DMSO	Negative Controls	7
P23	ARV-825	BET-targeting PROTAC	300	Drug	BET	7
P24	BzCl	BzCl	0	BzCl	Miscl.	7
A1	cells	cells	None	cells	Negative Controls	8
A2	BzCl	BzCl	0	BzCl	Miscl.	8
A3	Ganetespib	HSP90 inhibitor	1000	Drug	Miscl.	8
A4	Rigosertib	Ras-Raf interaction inhibitor, contaminated by microtubile depolymerizer…	10000	Drug	Miscl.	8
A5	Varespladib	Secretory phospholipase A2 inhibitor	10000	Drug	Miscl.	8
A6	Luminespib	HSP90 inhibitor	1000	Drug	Miscl.	8
A7	Filanesib	KSP/Eg5 inhibitor	1000	Drug	Miscl.	8
A8	Onalespib	HSP90 inhibitor	2500	Drug	Miscl.	8
A9	Litronesib	Eg5 inhibitor	1000	Drug	Miscl.	8
A10	Taladegib	Smothened (Hh) inhib	10000	Drug	Miscl.	8
A11	APR-246	p53 activator, thioredoxin reductase 1 inhibitor	10000	Drug	Miscl.	8
A12	URB597	FAAH inhibitor	1000	Drug	Miscl.	8
A13	SAR405838	MDM2 inhibitor	10000	Drug	Miscl.	8
A14	NVP-CGM097	p53-MDM2 inhibitor	25000	Drug	Miscl.	8
A15	VER 155008	HSP70 inhibitor	10000	Drug	Miscl.	8
A16	NMS-873	p97/VCP inhibitor	10000	Drug	Miscl.	8

A17	MST-312	Telomerase inhibitor	10000	Drug	Miscl.	8
A18	Sabutoclax	pan-Bcl-2 family inhibitor	25000	Drug	Miscl.	8
A19	GSK2830371	Wip1 inhibitor	5000	Drug	Miscl.	8
A20	A-1155463	BCL-XL inhibitor	10000	Drug	Miscl.	8
A21	NVP-SHP099	SHP2 inhibitor	10000	Drug	Miscl.	8
A22	S-63845	MCL-1 inhibitor	1000	Drug	Miscl.	8
A23	BzCl	BzCl	0	BzCl	Miscl.	8
A24	cells	cells	None	cells	Negative Controls	8
B1	cells	cells	None	cells	Negative Controls	8
B2	Tarenflurbil	Gamma-secretase inhibitor	10000	Drug	Miscl.	8
B3	Ganetespib	HSP90 inhibitor	100	Drug	Miscl.	8
B4	Rigosertib	Ras-Raf interaction inhibitor, contaminated by microtubile depolymerizer…	1000	Drug	Miscl.	8
B5	Varespladib	Secretory phospholipase A2 inhibitor	1000	Drug	Miscl.	8
B6	Luminespib	HSP90 inhibitor	100	Drug	Miscl.	8
B7	Filanesib	KSP/Eg5 inhibitor	100	Drug	Miscl.	8
B8	Onalespib	HSP90 inhibitor	250	Drug	Miscl.	8
B9	DMSO	DMSO	0	DMSO	Negative Controls	8
B10	Taladegib	Smothened (Hh) inhib	1000	Drug	Miscl.	8
B11	APR-246	p53 activator, thioredoxin reductase 1 inhibitor	1000	Drug	Miscl.	8
B12	URB597	FAAH inhibitor	100	Drug	Miscl.	8
B13	SAR405838	MDM2 inhibitor	1000	Drug	Miscl.	8
B14	NVP-CGM097	p53-MDM2 inhibitor	2500	Drug	Miscl.	8
B15	VER 155008	HSP70 inhibitor	1000	Drug	Miscl.	8
B16	BzCl	BzCl	0	BzCl	Miscl.	8
B17	MST-312	Telomerase inhibitor	1000	Drug	Miscl.	8
B18	Sabutoclax	pan-Bcl-2 family inhibitor	2500	Drug	Miscl.	8
B19	GSK2830371	Wip1 inhibitor	500	Drug	Miscl.	8
B20	A-1155463	BCL-XL inhibitor	1000	Drug	Miscl.	8
B21	NVP-SHP099	SHP2 inhibitor	1000	Drug	Miscl.	8
B22	S-63845	MCL-1 inhibitor	100	Drug	Miscl.	8
B23	Disulfiram(+CuCl2)	Antabuse	50000	Drug	Miscl.	8
B24	cells	cells	None	cells	Negative Controls	8
C1	cells	cells	None	cells	Negative Controls	8

C2	Tarenflurbil	Gamma-secretase inhibitor	1000	Drug	Miscl.	8
C3	Ganetespib	HSP90 inhibitor	10	Drug	Miscl.	8
C4	Rigosertib	Ras-Raf interaction inhibitor, contaminated by microtubile depolymerizer…	100	Drug	Miscl.	8
C5	Varespladib	Secretory phospholipase A2 inhibitor	100	Drug	Miscl.	8
C6	Luminespib	HSP90 inhibitor	10	Drug	Miscl.	8
C7	Filanesib	KSP/Eg5 inhibitor	10	Drug	Miscl.	8
C8	Onalespib	HSP90 inhibitor	25	Drug	Miscl.	8
C9	Litronesib	Eg5 inhibitor	100	Drug	Miscl.	8
C10	Taladegib	Smothened (Hh) inhib	100	Drug	Miscl.	8
C11	APR-246	p53 activator, thioredoxin reductase 1 inhibitor	100	Drug	Miscl.	8
C12	DMSO	DMSO	0	DMSO	Negative Controls	8
C13	SAR405838	MDM2 inhibitor	100	Drug	Miscl.	8
C14	NVP-CGM097	p53-MDM2 inhibitor	250	Drug	Miscl.	8
C15	VER 155008	HSP70 inhibitor	100	Drug	Miscl.	8
C16	NMS-873	p97/VCP inhibitor	1000	Drug	Miscl.	8
C17	MST-312	Telomerase inhibitor	100	Drug	Miscl.	8
C18	Sabutoclax	pan-Bcl-2 family inhibitor	250	Drug	Miscl.	8
C19	GSK2830371	Wip1 inhibitor	50	Drug	Miscl.	8
C20	DMSO	DMSO	0	DMSO	Negative Controls	8
C21	NVP-SHP099	SHP2 inhibitor	100	Drug	Miscl.	8
C22	S-63845	MCL-1 inhibitor	10	Drug	Miscl.	8
C23	Disulfiram(+CuCl2)	Antabuse	5000	Drug	Miscl.	8
C24	cells	cells	None	cells	Negative Controls	8
D1	cells	cells	None	cells	Negative Controls	8
D2	Tarenflurbil	Gamma-secretase inhibitor	100	Drug	Miscl.	8
D3	Ganetespib	HSP90 inhibitor	1	Drug	Miscl.	8
D4	Rigosertib	Ras-Raf interaction inhibitor, contaminated by microtubile depolymerizer…	10	Drug	Miscl.	8
D5	DMSO	DMSO	0	DMSO	Negative Controls	8
D6	Luminespib	HSP90 inhibitor	1	Drug	Miscl.	8
D7	Filanesib	KSP/Eg5 inhibitor	1	Drug	Miscl.	8
D8	Onalespib	HSP90 inhibitor	2.5	Drug	Miscl.	8
D9	Litronesib	Eg5 inhibitor	10	Drug	Miscl.	8
D10	Taladegib	Smothened (Hh) inhib	10	Drug	Miscl.	8

D11	APR-246	p53 activator, thioredoxin reductase 1 inhibitor	10	Drug	Miscl.	8
D12	URB597	FAAH inhibitor	10	Drug	Miscl.	8
D13	SAR405838	MDM2 inhibitor	10	Drug	Miscl.	8
D14	NVP-CGM097	p53-MDM2 inhibitor	25	Drug	Miscl.	8
D15	VER 155008	HSP70 inhibitor	10	Drug	Miscl.	8
D16	NMS-873	p97/VCP inhibitor	100	Drug	Miscl.	8
D17	MST-312	Telomerase inhibitor	10	Drug	Miscl.	8
D18	Sabutoclax	pan-Bcl-2 family inhibitor	25	Drug	Miscl.	8
D19	GSK2830371	Wip1 inhibitor	5	Drug	Miscl.	8
D20	A-1155463	BCL-XL inhibitor	100	Drug	Miscl.	8
D21	NVP-SHP099	SHP2 inhibitor	10	Drug	Miscl.	8
D22	S-63845	MCL-1 inhibitor	1	Drug	Miscl.	8
D23	Disulfiram(+CuCl2)	Antabuse	500	Drug	Miscl.	8
D24	cells	cells	None	cells	Negative Controls	8
E1	cells	cells	None	cells	Negative Controls	8
E2	Tarenflurbil	Gamma-secretase inhibitor	10	Drug	Miscl.	8
E3	Ganetespib	HSP90 inhibitor	0.1	Drug	Miscl.	8
E4	Rigosertib	Ras-Raf interaction inhibitor, contaminated by microtubile depolymerizer…	1	Drug	Miscl.	8
E5	Varespladib	Secretory phospholipase A2 inhibitor	10	Drug	Miscl.	8
E6	Luminespib	HSP90 inhibitor	0.1	Drug	Miscl.	8
E7	Filanesib	KSP/Eg5 inhibitor	0.1	Drug	Miscl.	8
E8	Onalespib	HSP90 inhibitor	0.25	Drug	Miscl.	8
E9	Litronesib	Eg5 inhibitor	1	Drug	Miscl.	8
E10	Taladegib	Smothened (Hh) inhib	1	Drug	Miscl.	8
E11	APR-246	p53 activator, thioredoxin reductase 1 inhibitor	1	Drug	Miscl.	8
E12	URB597	FAAH inhibitor	1	Drug	Miscl.	8
E13	SAR405838	MDM2 inhibitor	1	Drug	Miscl.	8
E14	NVP-CGM097	p53-MDM2 inhibitor	2.5	Drug	Miscl.	8
E15	DMSO	DMSO	0	DMSO	Negative Controls	8
E16	NMS-873	p97/VCP inhibitor	10	Drug	Miscl.	8
E17	MST-312	Telomerase inhibitor	1	Drug	Miscl.	8
E18	Sabutoclax	pan-Bcl-2 family inhibitor	2.5	Drug	Miscl.	8
E19	GSK2830371	Wip1 inhibitor	0.5	Drug	Miscl.	8

E20	A-1155463	BCL-XL inhibitor	10	Drug	Miscl.	8
E21	NVP-SHP099	SHP2 inhibitor	1	Drug	Miscl.	8
E22	S-63845	MCL-1 inhibitor	0.1	Drug	Miscl.	8
E23	Disulfiram(+CuCl2)	Antabuse	50	Drug	Miscl.	8
E24	cells	cells	None	cells	Negative Controls	8
F1	cells	cells	None	cells	Negative Controls	8
F2	Tarenflurbil	Gamma-secretase inhibitor	1	Drug	Miscl.	8
F3	Marimastat	MMP-9, MMP-1, MMP-2, MMP-14, MMP-7 inhibitor	10000	Drug	Miscl.	8
F4	Napabucasin	CSC inhibitor, STAT3 mediated	20000	Drug	Miscl.	8
F5	Varespladib	Secretory phospholipase A2 inhibitor	1	Drug	Miscl.	8
F6	Daporinad	NAMPT inhibitor	1000	Drug	Miscl.	8
F7	Triapine	ribonucleotide reductase inhibitor	10000	Drug	Miscl.	8
F8	Glasdegib	Smo inhibitor	1000	Drug	Miscl.	8
F9	Litronesib	Eg5 inhibitor	0.1	Drug	Miscl.	8
F10	BzCl	BzCl	0	BzCl	Miscl.	8
F11	Pevonedistat	NAE inhibitor	10000	Drug	Miscl.	8
F12	URB597	FAAH inhibitor	0.1	Drug	Miscl.	8
F13	Verdinexor	XPO1/CRM1 inhibitor	1000	Drug	Miscl.	8
F14	PAC-1	procaspase-3 activator	10000	Drug	Miscl.	8
F15	VER 155008	HSP70 inhibitor	1	Drug	Miscl.	8
F16	NMS-873	p97/VCP inhibitor	1	Drug	Miscl.	8
F17	ML323	USP1-UAF1 inhibitor	10000	Drug	Miscl.	8
F18	SH-4-54	STAT3 inhibitor	25000	Drug	Miscl.	8
F19	A-1331852	Bcl-XL inhibitor	1000	Drug	Miscl.	8
F20	A-1155463	BCL-XL inhibitor	1	Drug	Miscl.	8
F21	Necrostatin 2	Necroptosis inhibitor	10000	Drug	Miscl.	8
F22	TAK-530	pan-RAF inhibitor	10000	Drug	Miscl.	8
F23	Disulfiram(+CuCl2)	Antabuse	5	Drug	Miscl.	8
F24	cells	cells	None	cells	Negative Controls	8
G1	cells	cells	None	cells	Negative Controls	8
G2	Tosedostat	Aminopeptidase inhibitor	10000	Drug	Miscl.	8
G3	Marimastat	MMP-9, MMP-1, MMP-2, MMP-14, MMP-7 inhibitor	1000	Drug	Miscl.	8
G4	Napabucasin	CSC inhibitor, STAT3 mediated	2000	Drug	Miscl.	8

G5	CPI-613	pyruvate dehydrogenase, alpha-ketoglutarate dehydrogenase inhibitor	10000	Drug	Miscl.	8
G6	BzCl	BzCl	0	BzCl	Miscl.	8
G7	Triapine	ribonucleotide reductase inhibitor	1000	Drug	Miscl.	8
G8	Glasdegib	Smo inhibitor	100	Drug	Miscl.	8
G9	Omaveloxolone	Nrf2 activator	10000	Drug	Miscl.	8
G10	Saridegib	Smothened (Hh) inhib	10000	Drug	Miscl.	8
G11	Pevonedistat	NAE inhibitor	1000	Drug	Miscl.	8
G12	NVP-LGK974	PORCN inhibitor	10000	Drug	Miscl.	8
G13	Verdinexor	XPO1/CRM1 inhibitor	100	Drug	Miscl.	8
G14	PAC-1	procaspase-3 activator	1000	Drug	Miscl.	8
G15	WEHI-539	Bcl-XL inhibitor	2500	Drug	Miscl.	8
G16	PF-3845	FAAH inhibitor	10000	Drug	Miscl.	8
G17	ML323	USP1-UAF1 inhibitor	1000	Drug	Miscl.	8
G18	SH-4-54	STAT3 inhibitor	2500	Drug	Miscl.	8
G19	A-1331852	Bcl-XL inhibitor	100	Drug	Miscl.	8
G20	TH588	MTH1 inhibitor	25000	Drug	Miscl.	8
G21	Necrostatin 2	Necroptosis inhibitor	1000	Drug	Miscl.	8
G22	TAK-530	pan-RAF inhibitor	1000	Drug	Miscl.	8
G23	Erastin	VDAC inhibitor, induces ferroptosis	10000	Drug	Miscl.	8
G24	cells	cells	None	cells	Negative Controls	8
H1	cells	cells	None	cells	Negative Controls	8
H2	Tosedostat	Aminopeptidase inhibitor	1000	Drug	Miscl.	8
H3	Marimastat	MMP-9, MMP-1, MMP-2, MMP-14, MMP-7 inhibitor	100	Drug	Miscl.	8
H4	Napabucasin	CSC inhibitor, STAT3 mediated	200	Drug	Miscl.	8
H5	CPI-613	pyruvate dehydrogenase, alpha-ketoglutarate dehydrogenase inhibitor	1000	Drug	Miscl.	8
H6	Daporinad	NAMPT inhibitor	100	Drug	Miscl.	8
H7	Triapine	ribonucleotide reductase inhibitor	100	Drug	Miscl.	8
H8	Glasdegib	Smo inhibitor	10	Drug	Miscl.	8
H9	Omaveloxolone	Nrf2 activator	1000	Drug	Miscl.	8
H10	Saridegib	Smothened (Hh) inhib	1000	Drug	Miscl.	8
H11	Pevonedistat	NAE inhibitor	100	Drug	Miscl.	8
H12	NVP-LGK974	PORCN inhibitor	1000	Drug	Miscl.	8
H13	Verdinexor	XPO1/CRM1 inhibitor	10	Drug	Miscl.	8

H14	PAC-1	procaspase-3 activator	100	Drug	Miscl.	8
H15	WEHI-539	Bcl-XL inhibitor	250	Drug	Miscl.	8
H16	PF-3845	FAAH inhibitor	1000	Drug	Miscl.	8
H17	ML323	USP1-UAF1 inhibitor	100	Drug	Miscl.	8
H18	SH-4-54	STAT3 inhibitor	250	Drug	Miscl.	8
H19	DMSO	DMSO	0	DMSO	Negative Controls	8
H20	TH588	MTH1 inhibitor	2500	Drug	Miscl.	8
H21	Necrostatin 2	Necroptosis inhibitor	100	Drug	Miscl.	8
H22	TAK-530	pan-RAF inhibitor	100	Drug	Miscl.	8
H23	Erastin	VDAC inhibitor, induces ferroptosis	1000	Drug	Miscl.	8
H24	BzCl	BzCl	0	BzCl	Miscl.	8
I1	cells	cells	None	cells	Negative Controls	8
I2	Tosedostat	Aminopeptidase inhibitor	100	Drug	Miscl.	8
I3	Marimastat	MMP-9, MMP-1, MMP-2, MMP-14, MMP-7 inhibitor	10	Drug	Miscl.	8
I4	Napabucasin	CSC inhibitor, STAT3 mediated	20	Drug	Miscl.	8
I5	CPI-613	pyruvate dehydrogenase, alpha-ketoglutarate dehydrogenase inhibitor	100	Drug	Miscl.	8
I6	Daporinad	NAMPT inhibitor	10	Drug	Miscl.	8
I7	Triapine	ribonucleotide reductase inhibitor	10	Drug	Miscl.	8
I8	DMSO	DMSO	0	DMSO	Negative Controls	8
I9	Omaveloxolone	Nrf2 activator	100	Drug	Miscl.	8
I10	Saridegib	Smothened (Hh) inhib	100	Drug	Miscl.	8
I11	Pevonedistat	NAE inhibitor	10	Drug	Miscl.	8
I12	NVP-LGK974	PORCN inhibitor	100	Drug	Miscl.	8
I13	Verdinexor	XPO1/CRM1 inhibitor	1	Drug	Miscl.	8
I14	PAC-1	procaspase-3 activator	10	Drug	Miscl.	8
I15	WEHI-539	Bcl-XL inhibitor	25	Drug	Miscl.	8
I16	PF-3845	FAAH inhibitor	100	Drug	Miscl.	8
I17	ML323	USP1-UAF1 inhibitor	10	Drug	Miscl.	8
I18	SH-4-54	STAT3 inhibitor	25	Drug	Miscl.	8
I19	A-1331852	Bcl-XL inhibitor	10	Drug	Miscl.	8
I20	TH588	MTH1 inhibitor	250	Drug	Miscl.	8
I21	Necrostatin 2	Necroptosis inhibitor	10	Drug	Miscl.	8
I22	TAK-530	pan-RAF inhibitor	10	Drug	Miscl.	8

I23	Erastin	VDAC inhibitor, induces ferroptosis	100	Drug	Miscl.	8
I24	cells	cells	None	cells	Negative Controls	8
J1	cells	cells	None	cells	Negative Controls	8
J2	Tosedostat	Aminopeptidase inhibitor	10	Drug	Miscl.	8
J3	Marimastat	MMP-9, MMP-1, MMP-2, MMP-14, MMP-7 inhibitor	1	Drug	Miscl.	8
J4	Napabucasin	CSC inhibitor, STAT3 mediated	2	Drug	Miscl.	8
J5	CPI-613	pyruvate dehydrogenase, alpha-ketoglutarate dehydrogenase inhibitor	10	Drug	Miscl.	8
J6	Daporinad	NAMPT inhibitor	1	Drug	Miscl.	8
J7	Triapine	ribonucleotide reductase inhibitor	1	Drug	Miscl.	8
J8	Glasdegib	Smo inhibitor	1	Drug	Miscl.	8
J9	Omaveloxolone	Nrf2 activator	10	Drug	Miscl.	8
J10	Saridegib	Smothened (Hh) inhib	10	Drug	Miscl.	8
J11	Pevonedistat	NAE inhibitor	1	Drug	Miscl.	8
J12	NVP-LGK974	PORCN inhibitor	10	Drug	Miscl.	8
J13	Verdinexor	XPO1/CRM1 inhibitor	0.1	Drug	Miscl.	8
J14	BzCl	BzCl	0	BzCl	Miscl.	8
J15	WEHI-539	Bcl-XL inhibitor	2.5	Drug	Miscl.	8
J16	PF-3845	FAAH inhibitor	10	Drug	Miscl.	8
J17	ML323	USP1-UAF1 inhibitor	1	Drug	Miscl.	8
J18	SH-4-54	STAT3 inhibitor	2.5	Drug	Miscl.	8
J19	A-1331852	Bcl-XL inhibitor	1	Drug	Miscl.	8
J20	TH588	MTH1 inhibitor	25	Drug	Miscl.	8
J21	Necrostatin 2	Necroptosis inhibitor	1	Drug	Miscl.	8
J22	TAK-530	pan-RAF inhibitor	1	Drug	Miscl.	8
J23	Erastin	VDAC inhibitor, induces ferroptosis	10	Drug	Miscl.	8
J24	cells	cells	None	cells	Negative Controls	8
K1	cells	cells	None	cells	Negative Controls	8
K2	Tosedostat	Aminopeptidase inhibitor	1	Drug	Miscl.	8
K3	Darapladib	lipoprotein-associated phospholipase A2 inhibitor	0.1	Drug	Miscl.	8
K4	BIIB021	HSP90 inhibitor	1	Drug	Miscl.	8
K5	CPI-613	pyruvate dehydrogenase, alpha-ketoglutarate dehydrogenase inhibitor	1	Drug	Miscl.	8
K6	Daporinad	NAMPT inhibitor	0.1	Drug	Miscl.	8
K7	SB 743921	Mitotic inhibitor. Eg5/KSP inhibitor	0.01	Drug	Miscl.	8

K8	Glasdegib	Smo inhibitor	0.1	Drug	Miscl.	8
K9	Omaveloxolone	Nrf2 activator	1	Drug	Miscl.	8
K10	Saridegib	Smothened (Hh) inhib	1	Drug	Miscl.	8
K11	CUDC-305	HSP90 inhibitor	1	Drug	Miscl.	8
K12	NVP-LGK974	PORCN inhibitor	1	Drug	Miscl.	8
K13	AZD3965	MCT1 inhibitor	0.1	Drug	Miscl.	8
K14	PAC-1	procaspase-3 activator	1	Drug	Miscl.	8
K15	WEHI-539	Bcl-XL inhibitor	0.25	Drug	Miscl.	8
K16	PF-3845	FAAH inhibitor	1	Drug	Miscl.	8
K17	BCI	Dusp6 inhibitor	5	Drug	Miscl.	8
K18	A-1210477	MCL-1 inhibitor	5	Drug	Miscl.	8
K19	A-1331852	Bcl-XL inhibitor	0.1	Drug	Miscl.	8
K20	TH588	MTH1 inhibitor	2.5	Drug	Miscl.	8
K21	BzCl	BzCl	0	BzCl	Miscl.	8
K22	CPI-0610	BET family inhibitor	1	Drug	BET	8
K23	Erastin	VDAC inhibitor, induces ferroptosis	1	Drug	Miscl.	8
K24	cells	cells	None	cells	Negative Controls	8
L1	cells	cells	None	cells	Negative Controls	8
L2	Cilengitide	alphaVbeta3 integrin inhibitor	1	Drug	Miscl.	8
L3	Darapladib	lipoprotein-associated phospholipase A2 inhibitor	1	Drug	Miscl.	8
L4	BIIB021	HSP90 inhibitor	10	Drug	Miscl.	8
L5	AVN944	IMPDH inhibitor	1	Drug	Miscl.	8
L6	MK-0752	gamma-secretase/notch inhibitor	0.1	Drug	Miscl.	8
L7	SB 743921	Mitotic inhibitor. Eg5/KSP inhibitor	0.1	Drug	Miscl.	8
L8	TIC10	ERK & AKT inhibitor, TRAIL inducer	2.5	Drug	Miscl.	8
L9	VLX1570	proteasome deubiquitinase inhibitor	1	Drug	Miscl.	8
L10	E7820	Integrin alpha2 expression inhibitor	5	Drug	Miscl.	8
L11	CUDC-305	HSP90 inhibitor	10	Drug	Miscl.	8
L12	GSK923295	CENP-E inhibitor	1	Drug	Miscl.	8
L13	AZD3965	MCT1 inhibitor	1	Drug	Miscl.	8
L14	AT-406	XIAP, cIAP1, cIAP2 inhibitor	1	Drug	Miscl.	8
L15	ONX-0914	LMP7 (immunoproteasome)	1	Drug	Miscl.	8
L16	TRAM-34	intermediate-conductance Ca2+-activated K+ channel inh.	0.1	Drug	Miscl.	8

L17	DMSO	DMSO	0	DMSO	Negative Controls	8
L18	A-1210477	MCL-1 inhibitor	50	Drug	Miscl.	8
L19	Galiellalactone	STAT3-DNA interaction inhibitor	2.5	Drug	Miscl.	8
L20	BRD7116	Leukemic stem cell inhibitor	1	Drug	Miscl.	8
L21	JPH203	LAT1 inhibitor	0.5	Drug	Miscl.	8
L22	CPI-0610	BET family inhibitor	10	Drug	BET	8
L23	RSL3	GPX4 inhibitor, induces ferroptosis	1	Drug	Miscl.	8
L24	cells	cells	None	cells	Negative Controls	8
M1	cells	cells	None	cells	Negative Controls	8
M2	Cilengitide	alphaVbeta3 integrin inhibitor	10	Drug	Miscl.	8
M3	Darapladib	lipoprotein-associated phospholipase A2 inhibitor	10	Drug	Miscl.	8
M4	BzCl	BzCl	0	BzCl	Miscl.	8
M5	AVN944	IMPDH inhibitor	10	Drug	Miscl.	8
M6	MK-0752	gamma-secretase/notch inhibitor	1	Drug	Miscl.	8
M7	SB 743921	Mitotic inhibitor. Eg5/KSP inhibitor	1	Drug	Miscl.	8
M8	TIC10	ERK & AKT inhibitor, TRAIL inducer	25	Drug	Miscl.	8
M9	VLX1570	proteasome deubiquitinase inhibitor	10	Drug	Miscl.	8
M10	E7820	Integrin alpha2 expression inhibitor	50	Drug	Miscl.	8
M11	CUDC-305	HSP90 inhibitor	100	Drug	Miscl.	8
M12	GSK923295	CENP-E inhibitor	10	Drug	Miscl.	8
M13	AZD3965	MCT1 inhibitor	10	Drug	Miscl.	8
M14	AT-406	XIAP, cIAP1, cIAP2 inhibitor	10	Drug	Miscl.	8
M15	ONX-0914	LMP7 (immunoproteasome)	10	Drug	Miscl.	8
M16	TRAM-34	intermediate-conductance Ca2+-activated K+ channel inh.	1	Drug	Miscl.	8
M17	BCI	Dusp6 inhibitor	50	Drug	Miscl.	8
M18	A-1210477	MCL-1 inhibitor	500	Drug	Miscl.	8
M19	Galiellalactone	STAT3-DNA interaction inhibitor	25	Drug	Miscl.	8
M20	BRD7116	Leukemic stem cell inhibitor	10	Drug	Miscl.	8
M21	JPH203	LAT1 inhibitor	5	Drug	Miscl.	8
M22	CPI-0610	BET family inhibitor	100	Drug	BET	8
M23	RSL3	GPX4 inhibitor, induces ferroptosis	10	Drug	Miscl.	8
M24	cells	cells	None	cells	Negative Controls	8
N1	cells	cells	None	cells	Negative Controls	8

N2	Cilengitide	alphaVbeta3 integrin inhibitor	100	Drug	Miscl.	8
N3	Darapladib	lipoprotein-associated phospholipase A2 inhibitor	100	Drug	Miscl.	8
N4	BIIB021	HSP90 inhibitor	100	Drug	Miscl.	8
N5	AVN944	IMPDH inhibitor	100	Drug	Miscl.	8
N6	MK-0752	gamma-secretase/notch inhibitor	10	Drug	Miscl.	8
N7	DMSO	DMSO	0	DMSO	Negative Controls	8
N8	TIC10	ERK & AKT inhibitor, TRAIL inducer	250	Drug	Miscl.	8
N9	VLX1570	proteasome deubiquitinase inhibitor	100	Drug	Miscl.	8
N10	E7820	Integrin alpha2 expression inhibitor	500	Drug	Miscl.	8
N11	BzCl	BzCl	0	BzCl	Miscl.	8
N12	GSK923295	CENP-E inhibitor	100	Drug	Miscl.	8
N13	AZD3965	MCT1 inhibitor	100	Drug	Miscl.	8
N14	AT-406	XIAP, cIAP1, cIAP2 inhibitor	100	Drug	Miscl.	8
N15	ONX-0914	LMP7 (immunoproteasome)	100	Drug	Miscl.	8
N16	TRAM-34	intermediate-conductance Ca2+-activated K+ channel inh.	10	Drug	Miscl.	8
N17	BCI	Dusp6 inhibitor	500	Drug	Miscl.	8
N18	A-1210477	MCL-1 inhibitor	5000	Drug	Miscl.	8
N19	Galiellalactone	STAT3-DNA interaction inhibitor	250	Drug	Miscl.	8
N20	BRD7116	Leukemic stem cell inhibitor	100	Drug	Miscl.	8
N21	JPH203	LAT1 inhibitor	50	Drug	Miscl.	8
N22	CPI-0610	BET family inhibitor	1000	Drug	BET	8
N23	RSL3	GPX4 inhibitor, induces ferroptosis	100	Drug	Miscl.	8
N24	cells	cells	None	cells	Negative Controls	8
O1	cells	cells	None	cells	Negative Controls	8
O2	Cilengitide	alphaVbeta3 integrin inhibitor	1000	Drug	Miscl.	8
O3	Darapladib	lipoprotein-associated phospholipase A2 inhibitor	1000	Drug	Miscl.	8
O4	BIIB021	HSP90 inhibitor	1000	Drug	Miscl.	8
O5	AVN944	IMPDH inhibitor	1000	Drug	Miscl.	8
O6	MK-0752	gamma-secretase/notch inhibitor	100	Drug	Miscl.	8
O7	SB 743921	Mitotic inhibitor. Eg5/KSP inhibitor	10	Drug	Miscl.	8
O8	TIC10	ERK & AKT inhibitor, TRAIL inducer	2500	Drug	Miscl.	8
O9	VLX1570	proteasome deubiquitinase inhibitor	1000	Drug	Miscl.	8
O10	E7820	Integrin alpha2 expression inhibitor	5000	Drug	Miscl.	8

O11	CUDC-305	HSP90 inhibitor	1000	Drug	Miscl.	8
O12	GSK923295	CENP-E inhibitor	1000	Drug	Miscl.	8
O13	DMSO	DMSO	0	DMSO	Negative Controls	8
O14	AT-406	XIAP, cIAP1, cIAP2 inhibitor	1000	Drug	Miscl.	8
O15	ONX-0914	LMP7 (immunoproteasome)	1000	Drug	Miscl.	8
O16	TRAM-34	intermediate-conductance Ca2+-activated K+ channel inh.	100	Drug	Miscl.	8
O17	BCI	Dusp6 inhibitor	5000	Drug	Miscl.	8
O18	BzCl	BzCl	0	BzCl	Miscl.	8
O19	Galiellalactone	STAT3-DNA interaction inhibitor	2500	Drug	Miscl.	8
O20	BRD7116	Leukemic stem cell inhibitor	1000	Drug	Miscl.	8
O21	JPH203	LAT1 inhibitor	500	Drug	Miscl.	8
O22	CPI-0610	BET family inhibitor	10000	Drug	BET	8
O23	RSL3	GPX4 inhibitor, induces ferroptosis	1000	Drug	Miscl.	8
O24	cells	cells	None	cells	Negative Controls	8
P1	cells	cells	None	cells	Negative Controls	8
P2	Cilengitide	alphaVbeta3 integrin inhibitor	10000	Drug	Miscl.	8
P3	DMSO	DMSO	0	DMSO	Negative Controls	8
P4	BIIB021	HSP90 inhibitor	10000	Drug	Miscl.	8
P5	AVN944	IMPDH inhibitor	10000	Drug	Miscl.	8
P6	MK-0752	gamma-secretase/notch inhibitor	1000	Drug	Miscl.	8
P7	SB 743921	Mitotic inhibitor. Eg5/KSP inhibitor	100	Drug	Miscl.	8
P8	TIC10	ERK & AKT inhibitor, TRAIL inducer	25000	Drug	Miscl.	8
P9	VLX1570	proteasome deubiquitinase inhibitor	10000	Drug	Miscl.	8
P10	E7820	Integrin alpha2 expression inhibitor	50000	Drug	Miscl.	8
P11	CUDC-305	HSP90 inhibitor	10000	Drug	Miscl.	8
P12	GSK923295	CENP-E inhibitor	10000	Drug	Miscl.	8
P13	AZD3965	MCT1 inhibitor	1000	Drug	Miscl.	8
P14	AT-406	XIAP, cIAP1, cIAP2 inhibitor	10000	Drug	Miscl.	8
P15	ONX-0914	LMP7 (immunoproteasome)	10000	Drug	Miscl.	8
P16	TRAM-34	intermediate-conductance Ca2+-activated K+ channel inh.	1000	Drug	Miscl.	8
P17	BCI	Dusp6 inhibitor	50000	Drug	Miscl.	8
P18	A-1210477	MCL-1 inhibitor	50000	Drug	Miscl.	8
P19	Galiellalactone	STAT3-DNA interaction inhibitor	25000	Drug	Miscl.	8

P20	BRD7116	Leukemic stem cell inhibitor	10000	Drug	Miscl.	8
P21	JPH203	LAT1 inhibitor	5000	Drug	Miscl.	8
P22	DMSO	DMSO	0	DMSO	Negative Controls	8
P23	RSL3	GPX4 inhibitor, induces ferroptosis	10000	Drug	Miscl.	8
P24	BzCl	BzCl	0	BzCl	Miscl.	8

Supplementary Table 2

Plate number	Coculture combination				
	KB	KW	O3B	O8W	MHB
1	0.68	0.57	0.63	0.64	0.58
2	0.57	0.75	0.78	0.39	0.59
3	0.7	0.72	0.82	0.54	0.48
4	0.73	0.4	0.79	0.45	0.51
5	0.65	0.81	0.83	0.5	0.68
6	0.64	0.68	0.73	0.4	0.52
7	0.63	0.81	0.75	0.52	0.71
8	0.58	0.8	0.8	0.58	0.77

Supplementary Table 3

Drug class	Mechanims of Action (MOA)
Conventional chemotherapy	Mitotic<br>Topoisomerase
Kinase inhibitor	Cyclin dependant kinase (CDK)<br>Epidermal growth factor receptor (EGFR)<br>Mitogen-activated protein kinase ½ (MEK1/2)<br>Phosphoinositide 3-kinase (PI3K)<br>Vascular endothelial growth factor receptor (VEGFR)
Differentiating/epigenetic inhibitor	Bromodomain and extra-terminal motif (BET)<br>Histone deacetylase (HDAC)<br>Poly adenosine diphosphate-ribose polymerase (PARP)

Supplementary Table 4

Mechanism of Action (MOA)	*es* from CellProfiler (CP) features					average CP per target	*es* from MobileNetV2 features with unmasked bounding box					average NN per target	*es* from MobileNetV2 features with masked bounding box					average NN per target (masked)
	KB	KW	MHB	O3B	O8W		KB	KW	MHB	O3B	O8W		KB	KW	MHB	O3B	O8W	
BET	80.0	86.7	75.6	55.6	75.6	74.7	71.1	71.1	66.7	62.2	62.2	66.7	84.4	55.6	62.2	51.1	64.4	63.6
CDK	70.0	73.3	26.7	36.7	41.7	49.7	53.3	33.3	10.0	61.7	20.0	35.7	58.3	66.7	23.3	26.7	65.0	48.0
EGFR	71.3	48.8	81.3	76.3	13.8	58.3	30.0	47.5	85.0	85.0	30.0	55.5	63.8	55.0	77.5	71.3	28.8	59.3
HDAC	69.5	77.1	72.1	50.5	74.3	68.7	71.4	83.8	51.9	77.1	77.1	72.3	61.0	77.1	65.4	56.2	74.3	66.8
MEK1/2	73.3	33.3	93.3	86.7	76.7	72.7	73.3	43.3	90.0	90.0	63.3	72.0	63.3	60.0	83.3	86.7	76.7	74.0
Mitotic	73.3	91.1	73.3	71.1	75.6	76.9	75.6	82.2	66.7	80.0	73.3	75.6	62.2	77.8	75.6	57.8	73.3	69.3
PARP	60.0	76.0	52.0	88.0	68.0	68.8	84.0	84.0	60.0	72.0	44.0	68.8	52.0	60.0	88.0	96.0	80.0	75.2
PI3K	66.2	25.4	38.8	62.3	34.6	45.4	13.1	29.2	18.6	72.3	65.4	39.7	56.2	36.9	41.9	77.7	29.2	48.4
Topoisomerase	70.0	90.0	75.0	58.3	76.7	74.0	53.3	93.3	85.0	66.7	81.7	76.0	75.0	68.3	68.3	58.3	76.7	69.3
VEGFR	28.4	32.6	52.6	35.8	35.8	37.1	44.2	44.2	36.8	54.7	40.0	44.0	65.3	24.2	45.3	57.9	35.8	45.7
Average	66.2	63.4	64.1	62.1	57.3	62.6	56.9	61.2	57.1	72.2	55.7	60.6	64.1	58.2	63.1	64.0	60.4	62.0
					es > 80	7.0					*es* > 80	11.0					*es* > 80	5.0
					es > 75	16.0					*es* > 75	15.0					*es* > 75	13.0
					es > 70	25.0					*es* > 70	22.0					*es* > 70	17.0
					es > 65	30.0					*es* > 65	26.0					*es* > 65	22.0
					es > 60	31.0					*es* > 60	30.0					*es* > 60	29.0

Mechanism of Action (MOA)	*es* from ResNet50 features with unmasked bounding box					average NN per target	*es* from ResNet50 features with masked bounding box					average NN per target (masked)
	KB	KW	MHB	O3B	O8W		KB	KW	MHB	O3B	O8W	
BET	100.0	77.8	66.7	73.3	46.7	72.9	44.4	51.1	53.3	82.2	62.2	58.7
CDK	36.7	33.3	10.0	60.0	18.3	31.7	46.7	61.7	31.7	28.3	31.7	40.0
EGFR	40.0	40.0	83.8	80.0	26.3	54.0	42.5	48.8	76.3	82.5	16.3	53.3
HDAC	82.9	81.0	61.5	75.2	63.8	72.9	34.3	73.3	58.7	59.0	68.6	58.8
MEK1/2	26.7	46.7	93.3	90.0	66.7	64.7	50.0	66.7	90.0	96.7	76.7	76.0
Mitotic	42.2	80.0	62.2	73.3	71.1	65.8	40.0	68.9	66.7	75.6	80.0	66.2
PARP	64.0	92.0	72.0	72.0	56.0	71.2	32.0	60.0	80.0	88.0	84.0	68.8
PI3K	100.0	41.5	25.6	66.2	63.1	59.3	28.5	51.5	41.1	71.5	44.6	47.4
Topoisomerase	36.7	73.3	80.0	68.3	78.3	67.3	43.3	76.7	68.3	58.3	66.7	62.7
VEGFR	3.2	34.7	33.7	57.9	33.7	32.6	64.2	35.8	43.2	49.5	42.1	46.9
Average	53.2	60.0	58.9	71.6	52.4	59.2	42.6	59.4	60.9	69.2	57.3	57.9
					es > 80	8.0					*es* > 80	6.0
					es > 75	14.0					*es* > 75	12.0
					es > 70	20.0					*es* > 70	14.0
					es > 65	24.0					*es* > 65	20.0
					es > 60	29.0					*es* > 60	23.0

Supplementary Table 5

Mechanism of Action (MOA)	*es* from CellProfiler (CP) features					average CP per target	*es* from EfficientNetB0 features with unmasked bounding box					average NN per target	*es* from EfficientNetB0 features with masked bounding box					average NN per target (masked)
	KB	KW	MHB	O3B	O8W		KB	KW	MHB	O3B	O8W		KB	KW	MHB	O3B	O8W	
BET	77.8	80.0	73.3	51.1	73.3	71.1	86.7	46.7	64.4	51.1	53.3	60.4	86.7	46.7	60.0	51.1	53.3	59.6
CDK	68.3	68.3	21.7	26.7	28.3	42.7	53.3	63.3	21.7	20.0	36.7	39.0	53.3	63.3	13.3	20.0	36.7	37.3
EGFR	66.3	37.5	77.5	76.3	7.5	53.0	68.8	52.5	85.0	81.3	22.5	62.0	68.8	52.5	76.3	81.3	22.5	60.3
HDAC	67.6	76.2	70.2	46.7	74.3	67.0	53.3	61.0	50.0	50.5	65.7	56.1	53.3	61.0	51.0	50.5	65.7	56.3
MEK1/2	56.7	23.3	93.3	83.3	66.7	64.7	63.3	53.3	96.7	83.3	76.7	74.7	63.3	53.3	73.3	83.3	76.7	70.0
Mitotic	73.3	88.9	77.8	71.1	71.1	76.4	68.9	64.4	68.9	57.8	60.0	64.0	68.9	64.4	55.6	57.8	60.0	61.3
PARP	48.0	76.0	40.0	60.0	68.0	58.4	56.0	60.0	52.0	80.0	64.0	62.4	56.0	60.0	88.0	80.0	64.0	69.6
PI3K	63.8	18.5	35.7	60.0	31.5	41.9	60.8	39.2	20.2	73.8	31.5	45.1	60.8	39.2	31.8	73.8	31.5	47.4
Topoisomeras	68.3	80.0	75.0	50.0	75.0	69.7	65.0	65.0	58.3	60.0	71.7	64.0	65.0	65.0	66.7	60.0	71.7	65.7
VEGFR	22.1	23.2	49.5	31.6	30.5	31.4	62.1	29.5	27.4	50.5	46.3	43.2	62.1	29.5	34.7	50.5	46.3	44.6
Average	61.2	57.2	61.4	55.7	52.6	57.6	63.8	53.5	54.5	60.8	52.8	57.1	63.8	53.5	55.1	60.8	52.8	57.2
					es > 80	3.0					*es* > 80	5.0					*es* > 80	4.0
					es > 75	11.0					*es* > 75	7.0					*es* > 75	7.0
					es > 70	20.0					*es* > 70	9.0					*es* > 70	10.0
					es > 65	27.0					*es* > 65	13.0					*es* > 65	14.0
					es > 60	28.0					*es* > 60	23.0					*es* > 60	23.0

Mechanism of Action (MOA)	*es* from MobileNetV2 features with unmasked bounding box					average NN per target	*es* from MobileNetV2 features with masked bounding box					average NN per target (masked)
	KB	KW	MHB	O3B	O8W		KB	KW	MHB	O3B	O8W	
BET	71.1	71.1	64.4	60.0	62.2	65.8	80.0	53.3	57.8	53.3	60.0	60.9
CDK	40.0	26.7	6.7	58.3	15.0	29.3	58.3	61.7	16.7	21.7	51.7	42.0
EGFR	27.5	25.0	85.0	85.0	26.3	49.8	61.3	47.5	76.3	70.0	25.0	56.0
HDAC	69.5	80.0	45.2	76.2	77.1	69.6	59.0	75.2	64.4	54.3	72.4	65.1
MEK1/2	73.3	26.7	90.0	90.0	63.3	68.7	53.3	40.0	83.3	83.3	73.3	66.7
Mitotic	75.6	77.8	60.0	80.0	68.9	72.4	60.0	77.8	66.7	53.3	68.9	65.3
PARP	56.0	68.0	52.0	68.0	36.0	56.0	44.0	40.0	76.0	96.0	76.0	66.4
PI3K	7.7	22.3	15.5	69.2	64.6	35.9	53.8	28.5	37.2	77.7	25.4	44.5
Topoisomeras	45.0	93.3	81.7	65.0	81.7	73.3	73.3	68.3	66.7	58.3	75.0	68.3
VEGFR	37.9	43.2	31.6	52.6	31.6	39.4	62.1	14.7	40.0	53.7	32.6	40.6
Average	50.4	53.4	53.2	70.4	52.7	56.0	60.5	50.7	58.5	62.2	56.0	57.6
					es > 80	7.0					*es* > 80	3.0
					es > 75	13.0					*es* > 75	10.0
					es > 70	16.0					*es* > 70	14.0
					es > 65	21.0					*es* > 65	19.0
					es > 60	26.0					*es* > 60	23.0

Mechanism of Action	*es* from ResNet50 features with unmasked bounding box	average NN per target	*es* from ResNet50 features with masked bounding box	average NN per target

(MOA)	KB	KW	MHB	O3B	O8W	per target	KB	KW	MHB	O3B	O8W	(masked)
BET	100.0	77.8	64.4	68.9	51.1	72.4	42.2	51.1	51.1	82.2	57.8	56.9
CDK	36.7	26.7	5.0	46.7	13.3	25.7	41.7	56.7	25.0	28.3	25.0	35.3
EGFR	32.5	31.3	85.0	77.5	18.8	49.0	41.3	43.8	75.0	82.5	15.0	51.5
HDAC	79.0	81.0	55.8	72.4	62.9	70.2	32.4	68.6	57.7	60.0	66.7	57.1
MEK1/2	20.0	30.0	93.3	83.3	66.7	58.7	53.3	50.0	90.0	96.7	76.7	73.3
Mitotic	40.0	80.0	62.2	75.6	66.7	64.9	42.2	64.4	53.3	75.6	71.1	61.3
PARP	40.0	88.0	40.0	64.0	48.0	56.0	24.0	56.0	68.0	84.0	80.0	62.4
PI3K	100.0	31.5	20.9	63.8	60.0	55.3	27.7	50.8	37.2	70.0	43.1	45.7
Topoisomerase	36.7	70.0	76.7	65.0	78.3	65.3	41.7	71.7	65.0	60.0	68.3	61.3
VEGFR	1.1	27.4	29.5	55.8	25.3	27.8	54.7	32.6	38.9	47.4	38.9	42.5
Average	48.6	54.4	53.3	67.3	49.1	54.5	40.1	54.6	56.1	68.7	54.3	54.7
					es > 80	7.0					*es* > 80	5.0
					es > 75	14.0					*es* > 75	8.0
					es > 70	15.0					*es* > 70	11.0
					es > 65	19.0					*es* > 65	16.0
					es > 60	25.0					*es* > 60	18.0

Supplementary Table 6

Mechanism of Action (MOA)	lowest 2 concentrations es from CellProfiler features					per target average
	KB	KW	MHB	O3B	O8W	
BET	61.1	77.8	50.0	27.8	61.1	55.6
CDK	45.8	50.0	12.5	16.7	33.3	31.7
EGFR	56.2	34.3	62.5	56.3	15.6	45.0
HDAC	69.0	57.1	57.1	45.2	54.8	56.7
MEK1/2	58.3	25.0	83.3	66.7	41.7	55.0
Mitotic	66.7	77.7	55.6	55.6	50.0	61.1
PARP	50.0	60.0	50.0	80.0	30.0	54.0
PI3K	38.5	17.3	23.1	46.2	30.8	31.1
Topoisomeras	66.7	91.7	70.8	45.8	62.5	67.5
VEGFR	26.3	39.5	71.1	36.8	42.1	43.2
Average	53.9	53.0	53.6	47.7	42.2	50.1

Mechanism of Action (MOA)	lowest 2 concentrations *es* from unmasked EfficientNetB0 features					per target average
	KB	KW	MHB	O3B	O8W	
BET	44.4	55.5	50.0	27.7	38.8	43.3
CDK	12.5	33.3	16.6	41.6	4.2	21.6
EGFR	40.6	34.7	68.8	68.8	15.6	45.7
HDAC	57.1	52.4	57.1	57.1	50.0	54.8
MEK1/2	41.6	0.0	91.7	75.0	66.6	55.0
Mitotic	55.5	33.3	38.9	72.2	50.0	50.0
PARP	70.0	50.0	60.0	60.0	40.0	56.0
PI3K	19.2	36.5	38.5	57.6	63.4	43.0
Topoisomeras	54.1	70.8	70.8	54.1	66.6	63.3
VEGFR	44.7	47.3	55.3	50.0	15.7	42.6
Average	44.0	41.4	54.8	56.4	41.1	47.5

Mechanism of Action (MOA)	lowest 2 concentrations *es* from masked EfficientNetB0 features					per target average
	KB	KW	MHB	O3B	O8W	
BET	77.7	5.5	38.8	22.2	27.7	34.4
CDK	33.3	33.3	0.0	0.0	33.3	20.0
EGFR	75.0	50.0	53.1	62.5	34.3	55.0
HDAC	45.2	35.7	61.9	45.2	50.0	47.6
MEK1/2	50.0	41.6	50.0	58.3	50.0	50.0
Mitotic	66.6	55.5	22.2	33.3	44.4	44.4
PARP	80.0	70.0	80.0	70.0	50.0	70.0
PI3K	51.9	25.0	19.2	65.3	40.0	40.3
Topoisomeras	70.8	58.3	70.8	50.0	58.3	61.6
VEGFR	68.4	28.9	52.6	60.5	50.0	52.1
Average	61.9	40.4	44.9	46.7	43.8	47.5

Mechanism of Action (MOA)	highest 3 concentrations es from CellProfiler features					per target average
	KB	KW	MHB	O3B	O8W	
BET	92.6	92.6	92.6	74.1	85.2	87.4
CDK	86.1	88.9	36.1	50.0	47.2	61.7
EGFR	81.3	58.3	93.8	89.6	12.5	67.1
HDAC	69.8	90.5	82.3	54.0	87.3	76.8
MEK1/2	83.3	38.9	100.0	100.0	100.0	84.4
Mitotic	77.8	100.0	85.2	81.5	92.6	87.4
PARP	66.7	86.7	53.3	93.3	93.3	78.7
PI3K	84.6	30.8	50.0	73.1	37.2	55.1
Topoisomeras	72.2	88.9	77.8	66.7	86.1	78.3
VEGFR	29.8	28.1	40.4	35.1	31.6	33.0
Average	74.4	70.4	71.1	71.7	67.3	71.0

Mechanism of Action (MOA)	highest 3 concentrations *es* from unmasked EfficientNetB0 features					per target average
	KB	KW	MHB	O3B	O8W	
BET	85.1	92.5	85.2	81.4	81.4	85.1
CDK	77.7	36.1	47.2	94.4	8.3	52.8
EGFR	35.4	45.8	95.8	95.8	16.6	57.9
HDAC	68.2	92.0	46.8	87.3	80.9	75.0
MEK1/2	88.8	38.8	100.0	100.0	88.8	83.3
Mitotic	92.5	88.8	85.2	88.8	81.4	87.3
PARP	66.6	86.6	73.3	93.3	93.3	82.6
PI3K	39.7	65.3	19.5	83.3	52.5	52.1
Topoisomeras	55.5	86.1	50.0	86.1	86.1	72.8
VEGFR	40.3	29.8	19.3	47.3	12.2	29.8
Average	65.0	66.2	62.2	85.8	60.2	67.9

Mechanism of Action (MOA)	highest 3 concentrations *es* from masked EfficientNetB0 features					per target average
	KB	KW	MHB	O3B	O8W	
BET	96.2	77.7	85.2	70.3	74.0	80.7
CDK	80.5	91.6	38.8	38.8	50.0	59.9
EGFR	64.5	66.6	95.8	95.8	22.9	69.1
HDAC	60.3	77.7	51.6	58.7	73.0	64.3
MEK1/2	77.7	61.1	94.4	100.0	94.4	85.5
Mitotic	88.8	85.1	81.5	81.4	81.4	83.6
PARP	53.3	93.3	93.3	100.0	93.3	86.6
PI3K	69.2	53.8	42.9	79.4	30.8	55.2
Topoisomeras	66.6	77.7	55.6	69.4	80.6	70.0
VEGFR	61.4	35.1	28.1	52.6	45.6	44.6
Average	71.9	72.0	66.7	74.6	64.6	70.0

Supplementary Table 7

Mechanism of Action (MOA)	CellProfiler *es* for MHB	EfficientNetB0 *es* from MOA trained MHB	EfficientNetB0 *es* from binary trained MHB
BET	75.6	48.9	88.9
CDK	26.7	26.7	8.3
EGFR	81.3	76.3	70.0
HDAC	72.1	62.5	53.8
MEK1/2	93.3	100.0	76.7
Mitotic	73.3	80.0	71.1
PARP	52.0	80.0	36.0
PI3K	38.8	24.8	6.2
Topoisomerase	75.0	65.0	70.0
VEGFR	52.6	62.1	62.1
Average	64.1	62.6	54.3

Supplementary Table 8

Mechanism of Action (MOA)	*es* from fine-tuned Neural Network (NN) ResNet50					average trained NN per target (filtered)
	KB	KW	MHB	O3B	O8W	
BET	82.2	51.1	48.9	77.8	60.0	64.0
CDK	73.3	45.0	33.3	23.3	21.7	39.3
EGFR	80.0	51.3	78.8	83.8	16.3	62.0
HDAC	54.3	73.3	60.0	58.1	60.0	61.1
MEK1/2	83.3	66.7	93.3	96.7	73.3	82.7
Mitotic-i	77.8	64.4	55.6	80.0	82.2	72.0
PARP	60.0	68.0	72.0	88.0	76.0	72.8
PI3K	66.2	42.3	40.8	71.5	39.2	52.0
Topoisomerase	81.7	70.0	66.7	53.3	71.7	68.7
VEGFR	61.1	33.7	48.4	43.2	35.8	44.4
average	72.0	56.6	59.8	67.6	53.6	61.9
					es > 80	8
					es > 75	14
					es > 70	20
					es > 65	25
					es > 60	27

Evaluating Feature Extraction in Ovarian Cancer Cell Line Co-Cultures Using Deep Neural Networks

Osheen Sharma[1*], Greta Gudoityte[1], Rezan Minozada[1], Olli P. Kallioniemi[1,2], Riku Turkki[2], Lassi Paavolainen[2,3], Brinton Seashore-Ludlow[1**]

[1]Department of Oncology-Pathology, Karolinska Institutet, Science for Life Laboratory, Stockholm, Sweden

[2]Institute for Molecular Medicine Finland (FIMM), HiLIFE, University of Helsinki, Helsinki, Finland

[3]iCAN Digital Precision Cancer Medicine Flagship, University of Helsinki, Helsinki, Finland

[*]Correspondence: osheen.sharma@ki.se
[**]Correspondence: brinton.seashore-ludlow@ki.se
