# Supplementary material for: Evaluating feature extraction in ovarian cancer cell line co-cultures using deep neural networks
Source: Commun Biol. 2025 Feb 25;8:303. doi: 10.1038/s42003-025-07766-w (PMC11862010; doi:10.1038/s42003-025-07766-w)
Supplement: Supplementary file 2 — Description of Additional Supplementary Files [file 42003_2025_7766_MOESM2_ESM.docx]

Description of Additional Supplementary Files

**File name:** Supplementary Data 1

**Description:** Enrichment Score and p-value acquired from CellProfiler Features for all coculture combinations.

**File name:** Supplementary Data 2

**Description:** Enrichment Score and p-value acquired from EWicientNetB0 model with bounding box size of 50 by 50 for all co-culture combinations.

**File name:** Supplementary Data 3

**Description:** Enrichment Score and p-value acquired from EWicientNetB0 model with bounding box size of 90 by 90 for all co-culture combinations.

**File name:** Supplementary Data 4

**Description:** Enrichment Score and p-value acquired from MobileNetV2 model with bounding box size of 50 by 50 for all co-culture combinations.

**File name:** Supplementary Data 5

**Description:** Enrichment Score and p-value acquired from MobileNetV2 model with bounding box size of 90 by 90 for all co-culture combinations.

**File name:** Supplementary Data 6

**Description:** Enrichment Score and p-value acquired from ResNet50 model with bounding box size of 50 by 50 for all co-culture combinations.

**File name:** Supplementary Data 7

**Description:** Enrichment Score and p-value acquired from ResNet50 model with bounding box size of 90 by 90 for all co-culture combinations.
