# Supplementary material for: Evaluating feature extraction in ovarian cancer cell line co-cultures using deep neural networks
Source: Commun Biol. 2025 Feb 25;8:303. doi: 10.1038/s42003-025-07766-w (PMC11862010; doi:10.1038/s42003-025-07766-w)
Supplement: Supplementary file 3 — Supplementary Data 1 [file 42003_2025_7766_MOESM3_ESM.pdf]

	Well_annotation	l_concentra	ell_Catego	Highest_ES	Pvalue
0	2-KB-A16-Gefitinib-EGFR inhibitor	10000	EGFR	0.376517	0.047
1	2-KB-A19-Erlotinib-EGFR inhibitor	10000	EGFR	0.389831	0.055
2	2-KB-B19-Erlotinib-EGFR inhibitor	1000	EGFR	0.448026	0.005
3	2-KB-C16-Gefitinib-EGFR inhibitor	1000	EGFR	0.561269	0
4	2-KB-C19-Erlotinib-EGFR inhibitor	100	EGFR	0.563605	0
5	2-KB-D16-Gefitinib-EGFR inhibitor	100	EGFR	0.237315	0.439
6	2-KB-D19-Erlotinib-EGFR inhibitor	10	EGFR	0.226425	0.546
7	2-KB-E16-Gefitinib-EGFR inhibitor	10	EGFR	0.507193	0.001
8	2-KB-E19-Erlotinib-EGFR inhibitor	1	EGFR	0.521175	0
9	2-KB-F16-Gefitinib-EGFR inhibitor	1	EGFR	0.36395	0.078
10	2-KB-K11-Afatinib-EGFR inhibitor	0.1	EGFR	0.372007	0.002
11	2-KB-L11-Afatinib-EGFR inhibitor	1	EGFR	0.574213	0
12	2-KB-L16-Osimertinib-EGFR(L858R/T790M) inhibitor	0.25	EGFR	0.255112	0.521
13	2-KB-L19-Lapatinib-HER2, EGFR inhibitor	0.1	EGFR	0.491646	0
14	2-KB-M11-Afatinib-EGFR inhibitor	10	EGFR	0.554224	0
15	2-KB-M16-Osimertinib-EGFR(L858R/T790M) inhibitor	2.5	EGFR	0.292218	0.121
16	2-KB-M19-Lapatinib-HER2, EGFR inhibitor	1	EGFR	0.365503	0.024
17	2-KB-N16-Osimertinib-EGFR(L858R/T790M) inhibitor	25	EGFR	0.268254	0.051
18	2-KB-N19-Lapatinib-HER2, EGFR inhibitor	10	EGFR	0.493973	0
19	2-KB-O11-Afatinib-EGFR inhibitor	100	EGFR	0.48537	0.001
20	2-KB-O16-Osimertinib-EGFR(L858R/T790M) inhibitor	250	EGFR	0.460204	0.002
21	2-KB-O19-Lapatinib-HER2, EGFR inhibitor	100	EGFR	0.463903	0
22	2-KB-P11-Afatinib-EGFR inhibitor	1000	EGFR	0.432622	0
23	2-KB-P16-Osimertinib-EGFR(L858R/T790M) inhibitor	2500	EGFR	0.432153	0.013
24	2-KB-P19-Lapatinib-HER2, EGFR inhibitor	1000	EGFR	0.587485	0
25	3-KB-F21-Rociletinib-EGFR(L858R/T790M) inhibitor	10000	EGFR	0.43784	0.036
26	3-KB-G20-Neratinib-EGFR inhibitor	1000	EGFR	0.456124	0.005
27	3-KB-G21-Rociletinib-EGFR(L858R/T790M) inhibitor	1000	EGFR	0.498824	0
28	3-KB-H20-Neratinib-EGFR inhibitor	100	EGFR	0.492401	0
29	3-KB-H21-Rociletinib-EGFR(L858R/T790M) inhibitor	100	EGFR	0.521829	0
30	3-KB-I20-Neratinib-EGFR inhibitor	10	EGFR	0.483096	0
31	3-KB-I21-Rociletinib-EGFR(L858R/T790M) inhibitor	10	EGFR	0.403276	0.044

32	3-KB-J20-Neratinib-EGFR inhibitor	1	EGFR	0.298771	0.25
33	3-KB-J21-Rocileti nib-EGFR(L858R/T790M) inhibitor	1	EGFR	0.463402	0.001
34	3-KB-K4-Canertinib-pan-HER inhibitor	1	EGFR	0.302678	0.311
35	3-KB-K18-Dacomitinib-pan-HER inhibitor	0.1	EGFR	0.410651	0.054
36	3-KB-K20-Neratinib-EGFR inhibitor	0.1	EGFR	0.355491	0.064
37	3-KB-L4-Canertinib-pan-HER inhibitor	10	EGFR	0.396787	0.009
38	3-KB-L18-Dacomitinib-pan-HER inhibitor	1	EGFR	0.447151	0
39	3-KB-M18-Dacomitinib-pan-HER inhibitor	10	EGFR	0.548793	0
40	3-KB-N4-Canertinib-pan-HER inhibitor	100	EGFR	0.435713	0
41	3-KB-N18-Dacomitinib-pan-HER inhibitor	100	EGFR	0.56615	0
42	3-KB-O4-Canertinib-pan-HER inhibitor	1000	EGFR	0.430166	0.002
43	3-KB-P4-Canertinib-pan-HER inhibitor	10000	EGFR	0.07067	1
44	3-KB-P18-Dacomitinib-pan-HER inhibitor	1000	EGFR	0.498455	0
45	4-KB-F13-Sapitinib-Pan-HER inhibitor	1000	EGFR	0.427467	0.001
46	4-KB-G13-Sapitinib-Pan-HER inhibitor	100	EGFR	0.467832	0.019
47	4-KB-G16-Varlitinib-EGFR HER2 inhibitor	10000	EGFR	0.421948	0.018
48	4-KB-H13-Sapitinib-Pan-HER inhibitor	10	EGFR	0.599708	0
49	4-KB-H16-Varlitinib-EGFR HER2 inhibitor	1000	EGFR	0.346769	0.025
50	4-KB-I13-Sapitinib-Pan-HER inhibitor	1	EGFR	0.321168	0.049
51	4-KB-I16-Varlitinib-EGFR HER2 inhibitor	100	EGFR	0.389592	0.004
52	4-KB-J13-Sapitinib-Pan-HER inhibitor	0.1	EGFR	0.209458	0.416
53	4-KB-J16-Varlitinib-EGFR HER2 inhibitor	10	EGFR	0.207045	0.823
54	4-KB-K7-Icotinib-EGFR inhibitor	1	EGFR	0.365991	0.014
55	4-KB-K13-Tesevatinib-EGFR, ERBB2, VEGFR, EPHB4	0.1	EGFR	0.290379	0.411
56	4-KB-K16-Varlitinib-EGFR HER2 inhibitor	1	EGFR	0.493878	0.002
57	4-KB-L7-Icotinib-EGFR inhibitor	10	EGFR	0.444861	0.014
58	4-KB-L13-Tesevatinib-EGFR, ERBB2, VEGFR, EPHB4	1	EGFR	0.515829	0.005
59	4-KB-M7-Icotinib-EGFR inhibitor	100	EGFR	0.208008	0.627
60	4-KB-M13-Tesevatinib-EGFR, ERBB2, VEGFR, EPHB4	10	EGFR	0.45989	0
61	4-KB-N13-Tesevatinib-EGFR, ERBB2, VEGFR, EPHB4	100	EGFR	0.455449	0.003
62	4-KB-O7-Icotinib-EGFR inhibitor	1000	EGFR	0.323829	0.024
63	4-KB-P7-Icotinib-EGFR inhibitor	10000	EGFR	0.08354	0.955
64	4-KB-P13-Tesevatinib-EGFR, ERBB2, VEGFR, EPHB4	1000	EGFR	0.42523	0.005

65	5-KB-F4-Poziotinib-pan-HER inhibitor	1000	EGFR	0.464608	0.005
66	5-KB-F7-AZD3759-EGFR inhibitor, BBB penetrable	1000	EGFR	0.363726	0.132
67	5-KB-G4-Poziotinib-pan-HER inhibitor	100	EGFR	0.446675	0.002
68	5-KB-G7-AZD3759-EGFR inhibitor, BBB penetrable	100	EGFR	0.337221	0.012
69	5-KB-H4-Poziotinib-pan-HER inhibitor	10	EGFR	0.52492	0
70	5-KB-H7-AZD3759-EGFR inhibitor, BBB penetrable	10	EGFR	0.219717	0.303
71	5-KB-I4-Poziotinib-pan-HER inhibitor	1	EGFR	0.434652	0.006
72	5-KB-I7-AZD3759-EGFR inhibitor, BBB penetrable	1	EGFR	0.359079	0.012
73	5-KB-J4-Poziotinib-pan-HER inhibitor	0.1	EGFR	0.448729	0.008
74	5-KB-J7-AZD3759-EGFR inhibitor, BBB penetrable	0.1	EGFR	0.071203	0.968
75	5-KB-K7-Olmutinib-EGFR(L858R/T790M) inhibitor	0.1	EGFR	0.372295	0.087
76	5-KB-L7-Olmutinib-EGFR(L858R/T790M) inhibitor	1	EGFR	0.264522	0.161
77	5-KB-M7-Olmutinib-EGFR(L858R/T790M) inhibitor	10	EGFR	0.362988	0.007
78	5-KB-O7-Olmutinib-EGFR(L858R/T790M) inhibitor	100	EGFR	0.4167	0
79	5-KB-P7-Olmutinib-EGFR(L858R/T790M) inhibitor	1000	EGFR	0.249637	0.348
80	2-KB-A15-Lenvatinib-VEGFR inhibitor	2500	VEGFR	0.348094	0.333
81	2-KB-A17-Nintedanib-VEGFR, PDGFR, FGFR inhibitor	10000	VEGFR	0.272572	0.662
82	2-KB-A20-Tivozanib-VEGFR1, 2, 3, c-Kit, PDGFRB inhibitor	10000	VEGFR	0.300723	0.426
83	2-KB-B15-Lenvatinib-VEGFR inhibitor	250	VEGFR	0.297196	0.067
84	2-KB-B17-Nintedanib-VEGFR, PDGFR, FGFR inhibitor	1000	VEGFR	0.463492	0.002
85	2-KB-B20-Tivozanib-VEGFR1, 2, 3, c-Kit, PDGFRB inhibitor	1000	VEGFR	0.343957	0.111
86	2-KB-C15-Lenvatinib-VEGFR inhibitor	25	VEGFR	0.37059	0.01
87	2-KB-C17-Nintedanib-VEGFR, PDGFR, FGFR inhibitor	100	VEGFR	0.4135	0.008
88	2-KB-D15-Lenvatinib-VEGFR inhibitor	2.5	VEGFR	0.426045	0.001
89	2-KB-D17-Nintedanib-VEGFR, PDGFR, FGFR inhibitor	10	VEGFR	0.15658	0.899
90	2-KB-D20-Tivozanib-VEGFR1, 2, 3, c-Kit, PDGFRB inhibitor	100	VEGFR	0.298936	0.291
91	2-KB-E17-Nintedanib-VEGFR, PDGFR, FGFR inhibitor	1	VEGFR	0.360791	0.005
92	2-KB-E20-Tivozanib-VEGFR1, 2, 3, c-Kit, PDGFRB inhibitor	10	VEGFR	0.337531	0.051
93	2-KB-F13-Axitinib-VEGFR, PDGFR, KIT inhibitor	10000	VEGFR	0.198918	0.838
94	2-KB-F15-Lenvatinib-VEGFR inhibitor	0.25	VEGFR	0.261079	0.035
95	2-KB-F19-Regorafenib-B-Raf, c-Kit, VEGFR2 inhibitor	10000	VEGFR	0.240461	0.925
96	2-KB-F20-Tivozanib-VEGFR1, 2, 3, c-Kit, PDGFRB inhibitor	1	VEGFR	0.361066	0.124
97	2-KB-F21-Vatalanib-VEGFR-1 & -2 inhibitor	10000	VEGFR	0.438828	0.013

98	2-KB-G10-Apatinib-VEGFR inhibitor	10000	VEGFR	0.320751	0.037
99	2-KB-G13-Axitinib-VEGFR, PDGFR, KIT inhibitor	1000	VEGFR	0.226311	0.606
100	2-KB-G19-Regorafenib-B-Raf, c-Kit, VEGFR2 inhibitor	1000	VEGFR	0.298509	0.275
101	2-KB-G21-Vatalanib-VEGFR-1 & -2 inhibitor	1000	VEGFR	0.313664	0.27
102	2-KB-H10-Apatinib-VEGFR inhibitor	1000	VEGFR	0.353218	0.002
103	2-KB-H13-Axitinib-VEGFR, PDGFR, KIT inhibitor	100	VEGFR	0.244003	0.595
104	2-KB-H21-Vatalanib-VEGFR-1 & -2 inhibitor	100	VEGFR	0.360396	0.095
105	2-KB-I10-Apatinib-VEGFR inhibitor	100	VEGFR	0.113924	0.463
106	2-KB-I13-Axitinib-VEGFR, PDGFR, KIT inhibitor	10	VEGFR	0.372208	0.003
107	2-KB-I19-Regorafenib-B-Raf, c-Kit, VEGFR2 inhibitor	100	VEGFR	0.295916	0.174
108	2-KB-I21-Vatalanib-VEGFR-1 & -2 inhibitor	10	VEGFR	0.337285	0.163
109	2-KB-J10-Apatinib-VEGFR inhibitor	10	VEGFR	0.215824	0.796
110	2-KB-J13-Axitinib-VEGFR, PDGFR, KIT inhibitor	1	VEGFR	0.23813	0.303
111	2-KB-J19-Regorafenib-B-Raf, c-Kit, VEGFR2 inhibitor	10	VEGFR	0.410098	0.016
112	2-KB-J21-Vatalanib-VEGFR-1 & -2 inhibitor	1	VEGFR	0.362149	0.04
113	2-KB-K10-Apatinib-VEGFR inhibitor	1	VEGFR	0.294271	0.323
114	2-KB-K13-Vandetanib-VEGFR,EGFR, RET inhibitor	0.1	VEGFR	0.240274	0.563
115	2-KB-K17-Pazopanib-VEGFR inhibitor	1	VEGFR	0.466463	0
116	2-KB-K19-Regorafenib-B-Raf, c-Kit, VEGFR2 inhibitor	1	VEGFR	0.299238	0.323
117	2-KB-L12-Sorafenib-B-Raf, FGFR-1, VEGFR-2 & -3, PDGFR-beta, KIT, and FLT3 inhib	0.1	VEGFR	0.246413	0.298
118	2-KB-L13-Vandetanib-VEGFR,EGFR, RET inhibitor	1	VEGFR	0.309745	0.073
119	2-KB-L21-Cediranib-KDR/Flt/VEGFR inhibitor	0.1	VEGFR	0.334301	0.107
120	2-KB-M12-Sorafenib-B-Raf, FGFR-1, VEGFR-2 & -3, PDGFR-beta, KIT, and FLT3 inhib	1	VEGFR	0.299787	0.057
121	2-KB-M13-Vandetanib-VEGFR,EGFR, RET inhibitor	10	VEGFR	0.385128	0.005
122	2-KB-M17-Pazopanib-VEGFR inhibitor	10	VEGFR	0.385532	0.01
123	2-KB-M21-Cediranib-KDR/Flt/VEGFR inhibitor	1	VEGFR	0.242719	0.847
124	2-KB-N12-Sorafenib-B-Raf, FGFR-1, VEGFR-2 & -3, PDGFR-beta, KIT, and FLT3 inhib	10	VEGFR	0.254151	0.1
125	2-KB-N13-Vandetanib-VEGFR,EGFR, RET inhibitor	100	VEGFR	0.350859	0.022
126	2-KB-N17-Pazopanib-VEGFR inhibitor	100	VEGFR	0.446742	0
127	2-KB-N21-Cediranib-KDR/Flt/VEGFR inhibitor	10	VEGFR	0.365861	0.007
128	2-KB-O12-Sorafenib-B-Raf, FGFR-1, VEGFR-2 & -3, PDGFR-beta, KIT, and FLT3 inhib	100	VEGFR	0.276119	0.125
129	2-KB-O17-Pazopanib-VEGFR inhibitor	1000	VEGFR	0.401784	0.016
130	2-KB-O21-Cediranib-KDR/Flt/VEGFR inhibitor	100	VEGFR	0.332511	0.117

131	2-KB-P12-Sorafenib-B-Raf, FGFR-1, VEGFR-2 & -3, PDGFR-beta, KIT, and FLT3 inhib	1000	VEGFR	0.266437	0.113
132	2-KB-P13-Vandetanib-VEGFR,EGFR, RET inhibitor	1000	VEGFR	0.404259	0.001
133	2-KB-P17-Pazopanib-VEGFR inhibitor	10000	VEGFR	0.388218	0.049
134	2-KB-P21-Cediranib-KDR/Flt/VEGFR inhibitor	1000	VEGFR	0.383815	0.047
135	3-KB-A3-Cabozantinib-VEGFR2, Met, FLT3, Tie2, Kit and Ret inhibitor	1000	VEGFR	0.279807	0.451
136	3-KB-A6-Foretinib-MET, VEGFR2 inhibitor	1000	VEGFR	0.31719	0.373
137	3-KB-A18-Linifanib-VEGFR, PDGFR, CSF-1R, FLT3 inhibitor	1000	VEGFR	0.243317	0.246
138	3-KB-B3-Cabozantinib-VEGFR2, Met, FLT3, Tie2, Kit and Ret inhibitor	100	VEGFR	0.271487	0.082
139	3-KB-B6-Foretinib-MET, VEGFR2 inhibitor	100	VEGFR	0.27468	0.185
140	3-KB-B18-Linifanib-VEGFR, PDGFR, CSF-1R, FLT3 inhibitor	100	VEGFR	0.218224	0.235
141	3-KB-C3-Cabozantinib-VEGFR2, Met, FLT3, Tie2, Kit and Ret inhibitor	10	VEGFR	0.236012	0.251
142	3-KB-C6-Foretinib-MET, VEGFR2 inhibitor	10	VEGFR	0.144389	0.789
143	3-KB-C18-Linifanib-VEGFR, PDGFR, CSF-1R, FLT3 inhibitor	10	VEGFR	0.232239	0.417
144	3-KB-D3-Cabozantinib-VEGFR2, Met, FLT3, Tie2, Kit and Ret inhibitor	1	VEGFR	0.184561	0.57
145	3-KB-D6-Foretinib-MET, VEGFR2 inhibitor	1	VEGFR	0.101267	0.915
146	3-KB-D18-Linifanib-VEGFR, PDGFR, CSF-1R, FLT3 inhibitor	1	VEGFR	0.331059	0.059
147	3-KB-E3-Cabozantinib-VEGFR2, Met, FLT3, Tie2, Kit and Ret inhibitor	0.1	VEGFR	0.176365	0.711
148	3-KB-E6-Foretinib-MET, VEGFR2 inhibitor	0.1	VEGFR	0.223085	0.731
149	3-KB-E18-Linifanib-VEGFR, PDGFR, CSF-1R, FLT3 inhibitor	0.1	VEGFR	0.33375	0.152
150	3-KB-F18-Brivanib-VEGFR inhibitor	1000	VEGFR	0.395317	0.001
151	3-KB-G18-Brivanib-VEGFR inhibitor	100	VEGFR	0.508456	0
152	3-KB-H18-Brivanib-VEGFR inhibitor	10	VEGFR	0.198461	0.557
153	3-KB-I18-Brivanib-VEGFR inhibitor	1	VEGFR	0.238133	0.111
154	3-KB-J18-Brivanib-VEGFR inhibitor	0.1	VEGFR	0.067986	0.989
155	4-KB-A12-ENMD-2076-pan-Aurora, VEGFR inhibitor	10000	VEGFR	0.2471	0.929
156	4-KB-A15-Golvatinib-MET, VEGFR2 inhibitor	2500	VEGFR	0.215431	0.236
157	4-KB-A20-Motesanib-VEGFR, PDGFR, Ret, Kit inhibitor	10000	VEGFR	0.259498	0.286
158	4-KB-B12-ENMD-2076-pan-Aurora, VEGFR inhibitor	1000	VEGFR	0.324731	0.394
159	4-KB-B15-Golvatinib-MET, VEGFR2 inhibitor	250	VEGFR	0.221062	0.366
160	4-KB-B20-Motesanib-VEGFR, PDGFR, Ret, Kit inhibitor	1000	VEGFR	0.318755	0.194
161	4-KB-C15-Golvatinib-MET, VEGFR2 inhibitor	25	VEGFR	0.297285	0.311
162	4-KB-D12-ENMD-2076-pan-Aurora, VEGFR inhibitor	100	VEGFR	0.349231	0.238
163	4-KB-D15-Golvatinib-MET, VEGFR2 inhibitor	2.5	VEGFR	0.289142	0.801

**164**	4-KB-D20-Motesanib-VEGFR, PDGFR, Ret, Kit inhibitor	100	VEGFR	0.26155	0.64
**165**	4-KB-E12-ENMD-2076-pan-Aurora, VEGFR inhibitor	10	VEGFR	0.363251	0
**166**	4-KB-E20-Motesanib-VEGFR, PDGFR, Ret, Kit inhibitor	10	VEGFR	0.266433	0.507
**167**	4-KB-F12-ENMD-2076-pan-Aurora, VEGFR inhibitor	1	VEGFR	0.056016	0.983
**168**	4-KB-F15-Golvatinib-MET, VEGFR2 inhibitor	0.25	VEGFR	0.305451	0.787
**169**	4-KB-F20-Motesanib-VEGFR, PDGFR, Ret, Kit inhibitor	1	VEGFR	0.330835	0.213
**170**	4-KB-L16-Telatinib-VEGFR, KIT, PDGFR inhibitor	1	VEGFR	0.170936	0.591
**171**	4-KB-M16-Telatinib-VEGFR, KIT, PDGFR inhibitor	10	VEGFR	0.418746	0
**172**	4-KB-N16-Telatinib-VEGFR, KIT, PDGFR inhibitor	100	VEGFR	0.302094	0.004
**173**	4-KB-O16-Telatinib-VEGFR, KIT, PDGFR inhibitor	1000	VEGFR	0.366713	0.142
**174**	4-KB-P16-Telatinib-VEGFR, KIT, PDGFR inhibitor	10000	VEGFR	0.265627	0.132
**175**	2-KB-L10-Idelalisib-PI3K inhibitor, p110δ-selective	1	PI3K	0.253412	0.461
**176**	2-KB-M10-Idelalisib-PI3K inhibitor, p110δ-selective	10	PI3K	0.173682	0.73
**177**	2-KB-N10-Idelalisib-PI3K inhibitor, p110δ-selective	100	PI3K	0.379793	0
**178**	2-KB-O10-Idelalisib-PI3K inhibitor, p110δ-selective	1000	PI3K	0.461284	0
**179**	2-KB-P10-Idelalisib-PI3K inhibitor, p110δ-selective	10000	PI3K	0.409203	0.001
**180**	3-KB-A16-Perifosine-AKT/PI3K inhibitor	2500	PI3K	0.05354	0.997
**181**	3-KB-C16-Perifosine-AKT/PI3K inhibitor	250	PI3K	0.096704	0.83
**182**	3-KB-D16-Perifosine-AKT/PI3K inhibitor	25	PI3K	0.211424	0.508
**183**	3-KB-E16-Perifosine-AKT/PI3K inhibitor	2.5	PI3K	0.115176	0.874
**184**	3-KB-F16-Perifosine-AKT/PI3K inhibitor	0.25	PI3K	0.147947	0.829
**185**	3-KB-F17-Miltefosine-Antimicrobial, inhibits PI3K/AKT	100000	PI3K	0.44317	0.003
**186**	3-KB-F19-Duvelisib-PI3K inhibitor	500	PI3K	0.402141	0.002
**187**	3-KB-G17-Miltefosine-Antimicrobial, inhibits PI3K/AKT	10000	PI3K	0.263936	0.358
**188**	3-KB-G19-Duvelisib-PI3K inhibitor	50	PI3K	0.404204	0.002
**189**	3-KB-H17-Miltefosine-Antimicrobial, inhibits PI3K/AKT	1000	PI3K	0.095536	0.95
**190**	3-KB-I17-Miltefosine-Antimicrobial, inhibits PI3K/AKT	100	PI3K	0.082578	0.985
**191**	3-KB-I19-Duvelisib-PI3K inhibitor	5	PI3K	0.28818	0.162
**192**	3-KB-J17-Miltefosine-Antimicrobial, inhibits PI3K/AKT	10	PI3K	0.188181	0.546
**193**	3-KB-J19-Duvelisib-PI3K inhibitor	0.5	PI3K	0.260908	0.371
**194**	3-KB-K19-Duvelisib-PI3K inhibitor	0.05	PI3K	0.374696	0.02
**195**	3-KB-L8-Pictilisib-PI3K inhibitor, pan-class I	1	PI3K	0.113368	0.813
**196**	3-KB-L21-Taselisib-PI3K alpha, delta, (gamma) selective inhibitor	0.1	PI3K	0.302898	0.3

197	3-KB-M8-Pictilisib-PI3K inhibitor, pan-class I	10	PI3K	0.077217	0.933
198	3-KB-M21-Taselisib-PI3K alpha, delta, (gamma) selective inhibitor	1	PI3K	0.313905	0.195
199	3-KB-N8-Pictilisib-PI3K inhibitor, pan-class I	100	PI3K	0.427246	0
200	3-KB-N21-Taselisib-PI3K alpha, delta, (gamma) selective inhibitor	10	PI3K	0.388499	0.003
201	3-KB-O8-Pictilisib-PI3K inhibitor, pan-class I	1000	PI3K	0.515187	0
202	3-KB-O21-Taselisib-PI3K alpha, delta, (gamma) selective inhibitor	100	PI3K	0.390013	0.026
203	3-KB-P8-Pictilisib-PI3K inhibitor, pan-class I	10000	PI3K	0.515347	0
204	3-KB-P21-Taselisib-PI3K alpha, delta, (gamma) selective inhibitor	1000	PI3K	0.508524	0
205	4-KB-A19-Alpelisib-PI3Kalpha inhibitor	2500	PI3K	0.212439	0.487
206	4-KB-B19-Alpelisib-PI3Kalpha inhibitor	250	PI3K	0.183317	0.633
207	4-KB-C19-Alpelisib-PI3Kalpha inhibitor	25	PI3K	0.426263	0
208	4-KB-D19-Alpelisib-PI3Kalpha inhibitor	2.5	PI3K	0.536808	0
209	4-KB-E19-Alpelisib-PI3Kalpha inhibitor	0.25	PI3K	0.524938	0
210	4-KB-F14-NVP-BGT226-PI3K/mTOR inhibitor	1000	PI3K	0.466761	0
211	4-KB-G2-TGR-1202-PI3Kdelta inhibitor	2500	PI3K	0.24342	0.486
212	4-KB-G5-Sonolisib-PI3K inhibitor, pan-class I. Irreversible	10000	PI3K	0.511247	0
213	4-KB-G14-NVP-BGT226-PI3K/mTOR inhibitor	100	PI3K	0.49144	0
214	4-KB-G20-Buparlisib-PI3K inhibitor, pan-class I	10000	PI3K	0.474294	0
215	4-KB-H2-TGR-1202-PI3Kdelta inhibitor	250	PI3K	0.20411	0.656
216	4-KB-H5-Sonolisib-PI3K inhibitor, pan-class I. Irreversible	1000	PI3K	0.451636	0.002
217	4-KB-H14-NVP-BGT226-PI3K/mTOR inhibitor	10	PI3K	0.494376	0
218	4-KB-H20-Buparlisib-PI3K inhibitor, pan-class I	1000	PI3K	0.511252	0
219	4-KB-I2-TGR-1202-PI3Kdelta inhibitor	25	PI3K	0.324643	0.086
220	4-KB-I5-Sonolisib-PI3K inhibitor, pan-class I. Irreversible	100	PI3K	0.303237	0.292
221	4-KB-I14-NVP-BGT226-PI3K/mTOR inhibitor	1	PI3K	0.424743	0.023
222	4-KB-I20-Buparlisib-PI3K inhibitor, pan-class I	100	PI3K	0.375516	0.033
223	4-KB-J2-TGR-1202-PI3Kdelta inhibitor	2.5	PI3K	0.099093	0.973
224	4-KB-J5-Sonolisib-PI3K inhibitor, pan-class I. Irreversible	10	PI3K	0.089127	0.886
225	4-KB-J20-Buparlisib-PI3K inhibitor, pan-class I	10	PI3K	0.337926	0.048
226	4-KB-K2-TGR-1202-PI3Kdelta inhibitor	0.25	PI3K	0.232701	0.762
227	4-KB-K4-Dactolisib-mTOR/(PI3K) inhibitor	0.1	PI3K	0.363359	0.055
228	4-KB-K5-Sonolisib-PI3K inhibitor, pan-class I. Irreversible	1	PI3K	0.380845	0.045
229	4-KB-K14-NVP-BGT226-PI3K/mTOR inhibitor	0.1	PI3K	0.310723	0.034

230	4-KB-K20-Buparlisib-PI3K inhibitor, pan-class I	1	PI3K	0.380803	0.005
231	4-KB-L4-Dactolisib-mTOR/(PI3K) inhibitor	1	PI3K	0.391553	0.026
232	4-KB-L14-Gedatolisib-PI3K/mTOR inhibitor	0.1	PI3K	0.062603	0.967
233	4-KB-L15-TG100-115-PI3K gamma/delta inhibitor	1	PI3K	0.370763	0
234	4-KB-L21-Copanlisib-PI3K alpha, delta selective inhibitor	0.1	PI3K	0.448882	0
235	4-KB-M14-Gedatolisib-PI3K/mTOR inhibitor	1	PI3K	0.284765	0.063
236	4-KB-M15-TG100-115-PI3K gamma/delta inhibitor	10	PI3K	0.586529	0
237	4-KB-M21-Copanlisib-PI3K alpha, delta selective inhibitor	1	PI3K	0.419026	0
238	4-KB-N4-Dactolisib-mTOR/(PI3K) inhibitor	10	PI3K	0.539622	0
239	4-KB-N14-Gedatolisib-PI3K/mTOR inhibitor	10	PI3K	0.444822	0
240	4-KB-N15-TG100-115-PI3K gamma/delta inhibitor	100	PI3K	0.538415	0.001
241	4-KB-N21-Copanlisib-PI3K alpha, delta selective inhibitor	10	PI3K	0.413483	0.002
242	4-KB-O4-Dactolisib-mTOR/(PI3K) inhibitor	100	PI3K	0.438187	0.001
243	4-KB-O14-Gedatolisib-PI3K/mTOR inhibitor	100	PI3K	0.430508	0.003
244	4-KB-O15-TG100-115-PI3K gamma/delta inhibitor	1000	PI3K	0.380116	0.004
245	4-KB-O21-Copanlisib-PI3K alpha, delta selective inhibitor	100	PI3K	0.491676	0
246	4-KB-P4-Dactolisib-mTOR/(PI3K) inhibitor	1000	PI3K	0.479778	0
247	4-KB-P14-Gedatolisib-PI3K/mTOR inhibitor	1000	PI3K	0.53203	0
248	4-KB-P15-TG100-115-PI3K gamma/delta inhibitor	10000	PI3K	0.410993	0
249	4-KB-P21-Copanlisib-PI3K alpha, delta selective inhibitor	1000	PI3K	0.495928	0
250	5-KB-A6-LY3023414-PI3K/mTOR/DNA-PK inhibitor	2500	PI3K	0.530094	0
251	5-KB-A7-AMG319-PI3Kdelta inhibitor	1000	PI3K	0.35365	0.003
252	5-KB-A16-AZD-6482-PI3Kbeta-selective inhibitor	2500	PI3K	0.51203	0
253	5-KB-A17-Palomid-529-AKT, MTOR, PI3K inhibitor	10000	PI3K	0.394982	0
254	5-KB-B6-LY3023414-PI3K/mTOR/DNA-PK inhibitor	250	PI3K	0.430651	0
255	5-KB-B7-AMG319-PI3Kdelta inhibitor	100	PI3K	0.424713	0
256	5-KB-B17-Palomid-529-AKT, MTOR, PI3K inhibitor	1000	PI3K	0.391068	0.006
257	5-KB-C6-LY3023414-PI3K/mTOR/DNA-PK inhibitor	25	PI3K	0.427532	0.001
258	5-KB-C7-AMG319-PI3Kdelta inhibitor	10	PI3K	0.456173	0
259	5-KB-C16-AZD-6482-PI3Kbeta-selective inhibitor	250	PI3K	0.532824	0
260	5-KB-C17-Palomid-529-AKT, MTOR, PI3K inhibitor	100	PI3K	0.516164	0
261	5-KB-D6-LY3023414-PI3K/mTOR/DNA-PK inhibitor	2.5	PI3K	0.237701	0.036
262	5-KB-D7-AMG319-PI3Kdelta inhibitor	1	PI3K	0.455937	0

263	5-KB-D16-AZD-6482-PI3Kbeta-selective inhibitor	25	PI3K	0.440495	0
264	5-KB-D17-Palomid-529-AKT, MTOR, PI3K inhibitor	10	PI3K	0.166273	0.716
265	5-KB-E6-LY3023414-PI3K/mTOR/DNA-PK inhibitor	0.25	PI3K	0.214309	0.557
266	5-KB-E7-AMG319-PI3Kdelta inhibitor	0.1	PI3K	0.039724	1
267	5-KB-E16-AZD-6482-PI3Kbeta-selective inhibitor	2.5	PI3K	0.605216	0
268	5-KB-E17-Palomid-529-AKT, MTOR, PI3K inhibitor	1	PI3K	0.361457	0.002
269	5-KB-F11-GSK2636771-PI3K beta selective inhibitor	10000	PI3K	0.450405	0
270	5-KB-F16-AZD-6482-PI3Kbeta-selective inhibitor	0.25	PI3K	0.466864	0
271	5-KB-G9-Serabelisib-PI3Kalpha selective inhibitor	10000	PI3K	0.535778	0
272	5-KB-G11-GSK2636771-PI3K beta selective inhibitor	1000	PI3K	0.432233	0
273	5-KB-H9-Serabelisib-PI3Kalpha selective inhibitor	1000	PI3K	0.447519	0
274	5-KB-H11-GSK2636771-PI3K beta selective inhibitor	100	PI3K	0.555707	0
275	5-KB-I9-Serabelisib-PI3Kalpha selective inhibitor	100	PI3K	0.507248	0
276	5-KB-I11-GSK2636771-PI3K beta selective inhibitor	10	PI3K	0.099638	0.597
277	5-KB-J9-Serabelisib-PI3Kalpha selective inhibitor	10	PI3K	0.233781	0.074
278	5-KB-J11-GSK2636771-PI3K beta selective inhibitor	1	PI3K	0.089172	0.799
279	5-KB-K9-Serabelisib-PI3Kalpha selective inhibitor	1	PI3K	0.0725	0.967
280	5-KB-L14-AZD-8186-PI3Kbeta inhibitor	0.1	PI3K	0.238689	0.053
281	5-KB-L20-ZSTK474-PI3K gamma selective inhibitor	1	PI3K	0.440134	0.001
282	5-KB-L23-Omipalisib-PI3K/mTOR inhibitor	0.1	PI3K	0.242475	0.532
283	5-KB-M14-AZD-8186-PI3Kbeta inhibitor	1	PI3K	0.38529	0
284	5-KB-M20-ZSTK474-PI3K gamma selective inhibitor	10	PI3K	0.307785	0.084
285	5-KB-M23-Omipalisib-PI3K/mTOR inhibitor	1	PI3K	0.277998	0.415
286	5-KB-N14-AZD-8186-PI3Kbeta inhibitor	10	PI3K	0.378798	0
287	5-KB-N20-ZSTK474-PI3K gamma selective inhibitor	100	PI3K	0.382293	0.009
288	5-KB-N23-Omipalisib-PI3K/mTOR inhibitor	10	PI3K	0.489789	0
289	5-KB-O14-AZD-8186-PI3Kbeta inhibitor	100	PI3K	0.474664	0
290	5-KB-O20-ZSTK474-PI3K gamma selective inhibitor	1000	PI3K	0.506772	0
291	5-KB-O23-Omipalisib-PI3K/mTOR inhibitor	100	PI3K	0.513007	0
292	5-KB-P14-AZD-8186-PI3Kbeta inhibitor	1000	PI3K	0.41039	0.001
293	5-KB-P20-ZSTK474-PI3K gamma selective inhibitor	10000	PI3K	0.534357	0
294	5-KB-P23-Omipalisib-PI3K/mTOR inhibitor	1000	PI3K	0.517412	0
295	6-KB-A8-TGX-221-PI3K beta selective inhibitor	10000	PI3K	0.491664	0

296	6-KB-B8-TGX-221-PI3K beta selective inhibitor	1000	PI3K	0.400607	0.004
297	6-KB-C8-TGX-221-PI3K beta selective inhibitor	100	PI3K	0.493631	0
298	6-KB-D8-TGX-221-PI3K beta selective inhibitor	10	PI3K	0.267275	0.18
299	6-KB-E8-TGX-221-PI3K beta selective inhibitor	1	PI3K	0.097957	1
300	6-KB-L6-GDC-0084-PI3K/mTOR inhibitor	1	PI3K	0.211896	0.939
301	6-KB-M6-GDC-0084-PI3K/mTOR inhibitor	10	PI3K	0.043534	1
302	6-KB-N6-GDC-0084-PI3K/mTOR inhibitor	100	PI3K	0.504062	0
303	6-KB-O6-GDC-0084-PI3K/mTOR inhibitor	1000	PI3K	0.502995	0
304	6-KB-P6-GDC-0084-PI3K/mTOR inhibitor	10000	PI3K	0.444452	0.007
305	1-KB-F11-Amsacrine-DNA intercalation, Topo II inhibitor	10000	Topoisome	0.555514	0
306	1-KB-G11-Amsacrine-DNA intercalation, Topo II inhibitor	1000	Topoisome	0.594795	0
307	1-KB-G20-Epirubicin-Topoisomerase II inhibitor	1000	Topoisome	0.460753	0.069
308	1-KB-H11-Amsacrine-DNA intercalation, Topo II inhibitor	100	Topoisome	0.599593	0
309	1-KB-H20-Epirubicin-Topoisomerase II inhibitor	100	Topoisome	0.560043	0
310	1-KB-I11-Amsacrine-DNA intercalation, Topo II inhibitor	10	Topoisome	0.114917	1
311	1-KB-I20-Epirubicin-Topoisomerase II inhibitor	10	Topoisome	0.471427	0.002
312	1-KB-J11-Amsacrine-DNA intercalation, Topo II inhibitor	1	Topoisome	0.581872	0
313	1-KB-J20-Epirubicin-Topoisomerase II inhibitor	1	Topoisome	0.168978	0.996
314	1-KB-K11-SN-38-Active metabolite of irinotecan. Topoisomerase I inhibitor	1	Topoisome	0.598284	0
315	1-KB-K20-Epirubicin-Topoisomerase II inhibitor	0.1	Topoisome	0.058169	1
316	1-KB-L11-SN-38-Active metabolite of irinotecan. Topoisomerase I inhibitor	10	Topoisome	0.566368	0
317	1-KB-L14-Topotecan-Topoisomerase I inhibitor. Camptothecin analog	1	Topoisome	0.519946	0
318	1-KB-M11-SN-38-Active metabolite of irinotecan. Topoisomerase I inhibitor	100	Topoisome	0.593553	0
319	1-KB-M14-Topotecan-Topoisomerase I inhibitor. Camptothecin analog	10	Topoisome	0.297037	0.667
320	1-KB-N14-Topotecan-Topoisomerase I inhibitor. Camptothecin analog	100	Topoisome	0.575479	0
321	1-KB-O11-SN-38-Active metabolite of irinotecan. Topoisomerase I inhibitor	1000	Topoisome	0.444337	0.109
322	1-KB-O14-Topotecan-Topoisomerase I inhibitor. Camptothecin analog	1000	Topoisome	0.55199	0
323	1-KB-P11-SN-38-Active metabolite of irinotecan. Topoisomerase I inhibitor	10000	Topoisome	0.415097	0.213
324	1-KB-P14-Topotecan-Topoisomerase I inhibitor. Camptothecin analog	10000	Topoisome	0.446169	0.119
325	3-KB-A11-Etoposide-Topoisomerase II inhibitor	10000	Topoisome	0.581638	0
326	3-KB-B11-Etoposide-Topoisomerase II inhibitor	1000	Topoisome	0.579378	0
327	3-KB-C11-Etoposide-Topoisomerase II inhibitor	100	Topoisome	0.543262	0
328	3-KB-D11-Etoposide-Topoisomerase II inhibitor	10	Topoisome	0.324432	0.097

329	3-KB-E11-Etoposide-Topoisomerase II inhibitor	1	Topoisome	0.488024	0.013
330	3-KB-G9-Daunorubicin-Topoisomerase II inhibitor	1000	Topoisome	0.433208	0.2
331	3-KB-G10-Teniposide-Topoisomerase II inhibitor	10000	Topoisome	0.365295	0.007
332	3-KB-H9-Daunorubicin-Topoisomerase II inhibitor	100	Topoisome	0.592831	0
333	3-KB-H10-Teniposide-Topoisomerase II inhibitor	1000	Topoisome	0.379642	0.061
334	3-KB-I9-Daunorubicin-Topoisomerase II inhibitor	10	Topoisome	0.563417	0.001
335	3-KB-I10-Teniposide-Topoisomerase II inhibitor	100	Topoisome	0.556928	0
336	3-KB-J9-Daunorubicin-Topoisomerase II inhibitor	1	Topoisome	0.451992	0
337	3-KB-J10-Teniposide-Topoisomerase II inhibitor	10	Topoisome	0.259095	0.078
338	3-KB-K7-Idarubicin-Topoisomerase II inhibitor	0.1	Topoisome	0.347775	0.008
339	3-KB-K9-Daunorubicin-Topoisomerase II inhibitor	0.1	Topoisome	0.572181	0
340	3-KB-K10-Teniposide-Topoisomerase II inhibitor	1	Topoisome	0.644161	0
341	3-KB-L6-Doxorubicin-Topoisomerase II inhibitor	0.1	Topoisome	0.379951	0.001
342	3-KB-L7-Idarubicin-Topoisomerase II inhibitor	1	Topoisome	0.526533	0
343	3-KB-L9-Valrubicin-Topoisomerase II inhibitor	0.5	Topoisome	0.410007	0.012
344	3-KB-L10-Mitoxantrone-Topoisomerase II inhibitor	0.1	Topoisome	0.066188	0.998
345	3-KB-L16-Pixantrone-topoisomerase II inhibitor	1	Topoisome	0.589915	0
346	3-KB-M6-Doxorubicin-Topoisomerase II inhibitor	1	Topoisome	0.157036	0.78
347	3-KB-M7-Idarubicin-Topoisomerase II inhibitor	10	Topoisome	0.553312	0
348	3-KB-M9-Valrubicin-Topoisomerase II inhibitor	5	Topoisome	0.500742	0
349	3-KB-M10-Mitoxantrone-Topoisomerase II inhibitor	1	Topoisome	0.495833	0
350	3-KB-M16-Pixantrone-topoisomerase II inhibitor	10	Topoisome	0.431883	0.031
351	3-KB-N6-Doxorubicin-Topoisomerase II inhibitor	10	Topoisome	0.59348	0
352	3-KB-N9-Valrubicin-Topoisomerase II inhibitor	50	Topoisome	0.569309	0
353	3-KB-N10-Mitoxantrone-Topoisomerase II inhibitor	10	Topoisome	0.575447	0
354	3-KB-N16-Pixantrone-topoisomerase II inhibitor	100	Topoisome	0.40721	0.008
355	3-KB-O6-Doxorubicin-Topoisomerase II inhibitor	100	Topoisome	0.562655	0
356	3-KB-O7-Idarubicin-Topoisomerase II inhibitor	100	Topoisome	0.585644	0
357	3-KB-O9-Valrubicin-Topoisomerase II inhibitor	500	Topoisome	0.576624	0
358	3-KB-O10-Mitoxantrone-Topoisomerase II inhibitor	100	Topoisome	0.606289	0
359	3-KB-O16-Pixantrone-topoisomerase II inhibitor	1000	Topoisome	0.101151	0.995
360	3-KB-P6-Doxorubicin-Topoisomerase II inhibitor	1000	Topoisome	0.464945	0.08
361	3-KB-P7-Idarubicin-Topoisomerase II inhibitor	1000	Topoisome	0.412781	0.27

362	3-KB-P9-Valrubicin-Topoisomerase II inhibitor	5000	Topoisome	0.513817	0.013
363	3-KB-P10-Mitoxantrone-Topoisomerase II inhibitor	1000	Topoisome	0.448398	0.104
364	3-KB-P16-Pixantrone-topoisomerase II inhibitor	10000	Topoisome	0.544823	0
365	1-KB-A10-Vinorelbine-Mitotic inhibitor. Vinca alkaloid microtubule depolymerizer	10000	Mitotic	0.71578	0
366	1-KB-A13-Ixabepilone-Mitotic inhibitor. Epothilone microtubule stabilizer.	1000	Mitotic	0.746863	0
367	1-KB-A18-Paclitaxel-Mitotic inhibitor, taxane microtubule stabilizer	1000	Mitotic	0.715367	0
368	1-KB-B10-Vinorelbine-Mitotic inhibitor. Vinca alkaloid microtubule depolymerizer	1000	Mitotic	0.7117	0
369	1-KB-B13-Ixabepilone-Mitotic inhibitor. Epothilone microtubule stabilizer.	100	Mitotic	0.190474	0.832
370	1-KB-B18-Paclitaxel-Mitotic inhibitor, taxane microtubule stabilizer	100	Mitotic	0.722762	0
371	1-KB-C10-Vinorelbine-Mitotic inhibitor. Vinca alkaloid microtubule depolymerizer	100	Mitotic	0.720924	0
372	1-KB-C13-Ixabepilone-Mitotic inhibitor. Epothilone microtubule stabilizer.	10	Mitotic	0.195806	0.529
373	1-KB-C18-Paclitaxel-Mitotic inhibitor, taxane microtubule stabilizer	10	Mitotic	0.757775	0
374	1-KB-D10-Vinorelbine-Mitotic inhibitor. Vinca alkaloid microtubule depolymerizer	10	Mitotic	0.730141	0
375	1-KB-D13-Ixabepilone-Mitotic inhibitor. Epothilone microtubule stabilizer.	1	Mitotic	0.432692	0.001
376	1-KB-D18-Paclitaxel-Mitotic inhibitor, taxane microtubule stabilizer	1	Mitotic	0.689696	0
377	1-KB-E10-Vinorelbine-Mitotic inhibitor. Vinca alkaloid microtubule depolymerizer	1	Mitotic	0.531243	0
378	1-KB-E13-Ixabepilone-Mitotic inhibitor. Epothilone microtubule stabilizer.	0.1	Mitotic	0.525497	0.001
379	1-KB-E18-Paclitaxel-Mitotic inhibitor, taxane microtubule stabilizer	0.1	Mitotic	0.421354	0.035
380	1-KB-F13-Vinblastine-Mitotic inhibitor. Vinca alkaloid microtubule depolymerizer	1000	Mitotic	0.720081	0
381	1-KB-G13-Vinblastine-Mitotic inhibitor. Vinca alkaloid microtubule depolymerizer	100	Mitotic	0.467762	0.003
382	1-KB-G15-Eribulin-Mitotic inhibitor, microtubule depolymerizer.	1000	Mitotic	0.717778	0
383	1-KB-H13-Vinblastine-Mitotic inhibitor. Vinca alkaloid microtubule depolymerizer	10	Mitotic	0.289242	0.422
384	1-KB-H15-Eribulin-Mitotic inhibitor, microtubule depolymerizer.	100	Mitotic	0.709613	0
385	1-KB-I13-Vinblastine-Mitotic inhibitor. Vinca alkaloid microtubule depolymerizer	1	Mitotic	0.245988	0.609
386	1-KB-I15-Eribulin-Mitotic inhibitor, microtubule depolymerizer.	10	Mitotic	0.71808	0
387	1-KB-J13-Vinblastine-Mitotic inhibitor. Vinca alkaloid microtubule depolymerizer	0.1	Mitotic	0.257401	0.411
388	1-KB-J15-Eribulin-Mitotic inhibitor, microtubule depolymerizer.	1	Mitotic	0.75353	0
389	1-KB-K7-Vincristine-Mitotic inhibitor. Vinca alkaloid microtubule depolymerizer	0.1	Mitotic	0.497503	0
390	1-KB-K15-Eribulin-Mitotic inhibitor, microtubule depolymerizer.	0.1	Mitotic	0.67029	0
391	1-KB-L7-Vincristine-Mitotic inhibitor. Vinca alkaloid microtubule depolymerizer	1	Mitotic	0.383494	0.23
392	1-KB-L20-Vinflunine-Mitotic inhibitor. Vinca alkaloid microtubule depolymerizer	0.1	Mitotic	0.232785	0.758
393	1-KB-M7-Vincristine-Mitotic inhibitor. Vinca alkaloid microtubule depolymerizer	10	Mitotic	0.544591	0
394	1-KB-M20-Vinflunine-Mitotic inhibitor. Vinca alkaloid microtubule depolymerizer	1	Mitotic	0.365881	0.104

395	1-KB-N20-Vinflunine-Mitotic inhibitor. Vinca alkaloid microtubule depolymerizer	10	Mitotic	0.177094	0.896
396	1-KB-O7-Vincristine-Mitotic inhibitor. Vinca alkaloid microtubule depolymerizer	100	Mitotic	0.730836	0
397	1-KB-O20-Vinflunine-Mitotic inhibitor. Vinca alkaloid microtubule depolymerizer	100	Mitotic	0.340359	0.186
398	1-KB-P7-Vincristine-Mitotic inhibitor. Vinca alkaloid microtubule depolymerizer	1000	Mitotic	0.727047	0
399	1-KB-P20-Vinflunine-Mitotic inhibitor. Vinca alkaloid microtubule depolymerizer	1000	Mitotic	0.713236	0
400	3-KB-A7-Docetaxel-Mitotic inhibitor, taxane microtubule stabilizer	1000	Mitotic	0.733933	0
401	3-KB-B7-Docetaxel-Mitotic inhibitor, taxane microtubule stabilizer	100	Mitotic	0.755627	0
402	3-KB-C7-Docetaxel-Mitotic inhibitor, taxane microtubule stabilizer	10	Mitotic	0.767499	0
403	3-KB-D7-Docetaxel-Mitotic inhibitor, taxane microtubule stabilizer	1	Mitotic	0.646376	0
404	3-KB-E7-Docetaxel-Mitotic inhibitor, taxane microtubule stabilizer	0.1	Mitotic	0.480604	0.026
405	6-KB-L19-ABT-751-Mitotic inhibitor. Colchicine site binding microtubule depolymerizer.	1	Mitotic	0.148106	0.992
406	6-KB-M19-ABT-751-Mitotic inhibitor. Colchicine site binding microtubule depolymerizer.	10	Mitotic	0.620512	0
407	6-KB-N19-ABT-751-Mitotic inhibitor. Colchicine site binding microtubule depolymerizer.	100	Mitotic	0.389753	0.954
408	6-KB-O19-ABT-751-Mitotic inhibitor. Colchicine site binding microtubule depolymerizer.	1000	Mitotic	0.740145	0
409	6-KB-P19-ABT-751-Mitotic inhibitor. Colchicine site binding microtubule depolymerizer.	10000	Mitotic	0.735522	0
410	2-KB-A12-Trametinib-MEK1/2 inhibitor	250	MEK1/2	0.637991	0.001
411	2-KB-B12-Trametinib-MEK1/2 inhibitor	25	MEK1/2	0.777684	0
412	2-KB-D12-Trametinib-MEK1/2 inhibitor	2.5	MEK1/2	0.609677	0
413	2-KB-E12-Trametinib-MEK1/2 inhibitor	0.25	MEK1/2	0.568209	0.001
414	2-KB-F12-Trametinib-MEK1/2 inhibitor	2.5000000	MEK1/2	0.519545	0.007
415	2-KB-F14-Cobimetinib-MEK1/2 inhibitor	1000	MEK1/2	0.563623	0.01
416	2-KB-G14-Cobimetinib-MEK1/2 inhibitor	100	MEK1/2	0.680961	0
417	2-KB-H14-Cobimetinib-MEK1/2 inhibitor	10	MEK1/2	0.662927	0
418	2-KB-I14-Cobimetinib-MEK1/2 inhibitor	1	MEK1/2	0.376158	0.233
419	2-KB-K14-Cobimetinib-MEK1/2 inhibitor	0.1	MEK1/2	0.370566	0.486
420	2-KB-L20-Selumetinib-MEK1/2 inhibitor	1	MEK1/2	0.540531	0.005
421	2-KB-M20-Selumetinib-MEK1/2 inhibitor	10	MEK1/2	0.602849	0.002
422	2-KB-N20-Selumetinib-MEK1/2 inhibitor	100	MEK1/2	0.645224	0
423	2-KB-O20-Selumetinib-MEK1/2 inhibitor	1000	MEK1/2	0.667714	0
424	2-KB-P20-Selumetinib-MEK1/2 inhibitor	10000	MEK1/2	0.634442	0
425	4-KB-A10-Binimetinib-MEK1/2 inhibitor	1000	MEK1/2	0.490322	0.038
426	4-KB-A13-PD0325901-MEK1/2 inhibitor	1000	MEK1/2	0.640187	0
427	4-KB-B10-Binimetinib-MEK1/2 inhibitor	100	MEK1/2	0.512008	0.01

428	4-KB-B13-PD0325901-MEK1/2 inhibitor	100	MEK1/2	0.581798	0.003
429	4-KB-C10-Binimetinib-MEK1/2 inhibitor	10	MEK1/2	0.212513	0.899
430	4-KB-C13-PD0325901-MEK1/2 inhibitor	10	MEK1/2	0.587599	0
431	4-KB-D10-Binimetinib-MEK1/2 inhibitor	1	MEK1/2	0.328606	0.238
432	4-KB-D13-PD0325901-MEK1/2 inhibitor	1	MEK1/2	0.686571	0
433	4-KB-E10-Binimetinib-MEK1/2 inhibitor	0.1	MEK1/2	0.45343	0.047
434	4-KB-E13-PD0325901-MEK1/2 inhibitor	0.1	MEK1/2	0.321492	0.516
435	4-KB-L19-GDC-0623-MEK1/2 inhibitor	0.25	MEK1/2	0.545577	0.009
436	4-KB-M19-GDC-0623-MEK1/2 inhibitor	2.5	MEK1/2	0.38353	0.281
437	4-KB-N19-GDC-0623-MEK1/2 inhibitor	25	MEK1/2	0.45131	0.364
438	4-KB-O19-GDC-0623-MEK1/2 inhibitor	250	MEK1/2	0.402688	0.207
439	4-KB-P19-GDC-0623-MEK1/2 inhibitor	2500	MEK1/2	0.61294	0.002
440	1-KB-L2-Olaparib-PARP inhibitor	1	PARP	0.461537	0.077
441	1-KB-L6-Rucaparib-PARP inhibitor	1	PARP	0.388852	0.268
442	1-KB-M2-Olaparib-PARP inhibitor	10	PARP	0.325495	0.47
443	1-KB-M6-Rucaparib-PARP inhibitor	10	PARP	0.481562	0.065
444	1-KB-N2-Olaparib-PARP inhibitor	100	PARP	0.635038	0.001
445	1-KB-N6-Rucaparib-PARP inhibitor	100	PARP	0.326392	0.65
446	1-KB-O2-Olaparib-PARP inhibitor	1000	PARP	0.560752	0.002
447	1-KB-O6-Rucaparib-PARP inhibitor	1000	PARP	0.629249	0.001
448	1-KB-P2-Olaparib-PARP inhibitor	10000	PARP	0.393265	0.223
449	1-KB-P6-Rucaparib-PARP inhibitor	10000	PARP	0.460855	0.089
450	7-KB-A3-Talazoparib-PARP1/2 inhibitor	1000	PARP	0.45235	0.086
451	7-KB-B2-Veliparib-PARP inhibitor	10000	PARP	0.567505	0.008
452	7-KB-B3-Talazoparib-PARP1/2 inhibitor	100	PARP	0.557348	0.009
453	7-KB-C2-Veliparib-PARP inhibitor	1000	PARP	0.613617	0.007
454	7-KB-C3-Talazoparib-PARP1/2 inhibitor	10	PARP	0.323129	0.373
455	7-KB-D2-Veliparib-PARP inhibitor	100	PARP	0.483624	0.042
456	7-KB-D3-Talazoparib-PARP1/2 inhibitor	1	PARP	0.677189	0.003
457	7-KB-E2-Veliparib-PARP inhibitor	10	PARP	0.130892	0.994
458	7-KB-E3-Talazoparib-PARP1/2 inhibitor	0.1	PARP	0.616198	0.002
459	7-KB-F2-Veliparib-PARP inhibitor	1	PARP	0.551276	0.007
460	7-KB-G2-Niraparib-PARP inhibitor	10000	PARP	0.50979	0.037

461	7-KB-H2-Niraparib-PARP inhibitor	1000	PARP	0.57271	0.003
462	7-KB-I2-Niraparib-PARP inhibitor	100	PARP	0.622803	0.005
463	7-KB-J2-Niraparib-PARP inhibitor	10	PARP	0.577096	0.008
464	7-KB-K2-Niraparib-PARP inhibitor	1	PARP	0.61087	0
465	3-KB-A19-Dinaciclib-CDK inhibitor	1000	CDK	0.595396	0
466	3-KB-B19-Dinaciclib-CDK inhibitor	100	CDK	0.599791	0
467	3-KB-B23-Abemaciclib-CDK4/6 inhibitor	2500	CDK	0.468715	0.001
468	3-KB-C19-Dinaciclib-CDK inhibitor	10	CDK	0.528489	0.001
469	3-KB-C23-Abemaciclib-CDK4/6 inhibitor	250	CDK	0.478514	0.004
470	3-KB-D19-Dinaciclib-CDK inhibitor	1	CDK	0.468718	0.012
471	3-KB-D23-Abemaciclib-CDK4/6 inhibitor	25	CDK	0.496603	0.003
472	3-KB-E19-Dinaciclib-CDK inhibitor	0.1	CDK	0.290474	0.463
473	3-KB-E23-Abemaciclib-CDK4/6 inhibitor	2.5	CDK	0.460765	0.009
474	3-KB-F23-Abemaciclib-CDK4/6 inhibitor	0.25	CDK	0.382539	0.02
475	3-KB-K17-Palbociclib-CDK4/6 inhibitor	1	CDK	0.500489	0
476	3-KB-L19-Ribociclib-CDK4/6 inhibitor	1	CDK	0.508861	0.001
477	3-KB-M17-Palbociclib-CDK4/6 inhibitor	10	CDK	0.575159	0
478	3-KB-M19-Ribociclib-CDK4/6 inhibitor	10	CDK	0.4961	0
479	3-KB-N17-Palbociclib-CDK4/6 inhibitor	100	CDK	0.535316	0
480	3-KB-N19-Ribociclib-CDK4/6 inhibitor	100	CDK	0.547259	0
481	3-KB-O17-Palbociclib-CDK4/6 inhibitor	1000	CDK	0.552952	0
482	3-KB-O19-Ribociclib-CDK4/6 inhibitor	1000	CDK	0.581084	0
483	3-KB-P17-Palbociclib-CDK4/6 inhibitor	10000	CDK	0.432712	0.012
484	3-KB-P19-Ribociclib-CDK4/6 inhibitor	10000	CDK	0.46721	0.011
485	4-KB-A4-SNS-032-CDK inhibitor	10000	CDK	0.612599	0
486	4-KB-A8-Milciclib-CDK2 inhibitor	10000	CDK	0.621064	0
487	4-KB-B4-SNS-032-CDK inhibitor	1000	CDK	0.602667	0
488	4-KB-B8-Milciclib-CDK2 inhibitor	1000	CDK	0.52622	0
489	4-KB-C4-SNS-032-CDK inhibitor	100	CDK	0.526849	0
490	4-KB-C8-Milciclib-CDK2 inhibitor	100	CDK	0.443571	0.077
491	4-KB-D4-SNS-032-CDK inhibitor	10	CDK	0.350999	0.492
492	4-KB-D8-Milciclib-CDK2 inhibitor	10	CDK	0.32716	0.449
493	4-KB-E4-SNS-032-CDK inhibitor	1	CDK	0.274146	0.556

494	4-KB-E8-Milciclib-CDK2 inhibitor	1	CDK	0.430498	0.045
495	4-KB-F4-Seliciclib-CDK2/7/9 inhibitor	10000	CDK	0.473336	0.002
496	4-KB-F22-Alvocidib-CDK inhibitor	10000	CDK	0.603969	0
497	4-KB-G4-Seliciclib-CDK2/7/9 inhibitor	1000	CDK	0.435094	0.012
498	4-KB-G22-Alvocidib-CDK inhibitor	1000	CDK	0.606694	0
499	4-KB-H4-Seliciclib-CDK2/7/9 inhibitor	100	CDK	0.36025	0.166
500	4-KB-H22-Alvocidib-CDK inhibitor	100	CDK	0.468308	0.001
501	4-KB-I4-Seliciclib-CDK2/7/9 inhibitor	10	CDK	0.187269	0.615
502	4-KB-I22-Alvocidib-CDK inhibitor	10	CDK	0.440881	0.015
503	4-KB-J4-Seliciclib-CDK2/7/9 inhibitor	1	CDK	0.096209	0.869
504	4-KB-J22-Alvocidib-CDK inhibitor	1	CDK	0.492256	0.047
505	5-KB-A19-AZD-5438-CDK1,2,9 inhibitor	10000	CDK	0.53494	0.002
506	5-KB-B19-AZD-5438-CDK1,2,9 inhibitor	1000	CDK	0.447952	0.006
507	5-KB-C19-AZD-5438-CDK1,2,9 inhibitor	100	CDK	0.443162	0.001
508	5-KB-D19-AZD-5438-CDK1,2,9 inhibitor	10	CDK	0.349325	0.133
509	5-KB-E19-AZD-5438-CDK1,2,9 inhibitor	1	CDK	0.417282	0.008
510	5-KB-K17-AT7519-CDK1, 2, 4, 6 and 9 inhibitor	1	CDK	0.345569	0.178
511	5-KB-M17-AT7519-CDK1, 2, 4, 6 and 9 inhibitor	10	CDK	0.216356	0.883
512	5-KB-N17-AT7519-CDK1, 2, 4, 6 and 9 inhibitor	100	CDK	0.453747	0.483
513	5-KB-O17-AT7519-CDK1, 2, 4, 6 and 9 inhibitor	1000	CDK	0.567697	0
514	5-KB-P17-AT7519-CDK1, 2, 4, 6 and 9 inhibitor	10000	CDK	0.577379	0
515	6-KB-A17-Senexin B-CDK8/19 inhibitor	1000	CDK	0.457076	0.005
516	6-KB-B17-Senexin B-CDK8/19 inhibitor	100	CDK	0.50087	0.416
517	6-KB-C17-Senexin B-CDK8/19 inhibitor	10	CDK	0.525803	0.236
518	6-KB-D17-Senexin B-CDK8/19 inhibitor	1	CDK	0.265273	0.477
519	6-KB-E17-Senexin B-CDK8/19 inhibitor	0.1	CDK	0.373574	0.058
520	6-KB-L15-THZ2-CDK7 inhibitor	1	CDK	0.182881	0.628
521	6-KB-M15-THZ2-CDK7 inhibitor	10	CDK	0.476611	0.173
522	6-KB-N15-THZ2-CDK7 inhibitor	100	CDK	0.514217	0.004
523	6-KB-O15-THZ2-CDK7 inhibitor	1000	CDK	0.612629	0
524	6-KB-P15-THZ2-CDK7 inhibitor	10000	CDK	0.622909	0
525	7-KB-A21-dBET1-BET-targeting PROTAC	10000	BET	0.639139	0
526	7-KB-A22-PFI-1-BET family inhibitor	30000	BET	0.762648	0

527	7-KB-B21-dBET1-BET-targeting PROTAC	1000	BET	0.667395	0
528	7-KB-B22-PFI-1-BET family inhibitor	3000	BET	0.751796	0
529	7-KB-C21-dBET1-BET-targeting PROTAC	100	BET	0.740247	0
530	7-KB-C22-PFI-1-BET family inhibitor	300	BET	0.73035	0
531	7-KB-D21-dBET1-BET-targeting PROTAC	10	BET	0.323172	0.373
532	7-KB-D22-PFI-1-BET family inhibitor	30	BET	0.25885	0.556
533	7-KB-E21-dBET1-BET-targeting PROTAC	1	BET	0.515519	0.001
534	7-KB-E22-PFI-1-BET family inhibitor	3	BET	0.392177	0.181
535	7-KB-G10-Birabresib-BET family inhibitor	10000	BET	0.745831	0
536	7-KB-G15-I-BET151-BET family inhibitor	10000	BET	0.765871	0
537	7-KB-H10-Birabresib-BET family inhibitor	1000	BET	0.769465	0
538	7-KB-H15-I-BET151-BET family inhibitor	1000	BET	0.744413	0
539	7-KB-I10-Birabresib-BET family inhibitor	100	BET	0.737437	0
540	7-KB-I15-I-BET151-BET family inhibitor	100	BET	0.628311	0
541	7-KB-J10-Birabresib-BET family inhibitor	10	BET	0.426057	0.003
542	7-KB-J15-I-BET151-BET family inhibitor	10	BET	0.610494	0
543	7-KB-K10-Birabresib-BET family inhibitor	1	BET	0.101519	1
544	7-KB-K13-Mivebresib-BET family inhibitor	1	BET	0.7071	0
545	7-KB-K15-I-BET151-BET family inhibitor	1	BET	0.56655	0
546	7-KB-L12-Molibresib-BET family inhibitor	1	BET	0.561032	0
547	7-KB-L13-Mivebresib-BET family inhibitor	10	BET	0.727586	0
548	7-KB-L20-JQ1-BET family inhibitor	1	BET	0.405023	0.157
549	7-KB-L23-ARV-825-BET-targeting PROTAC	0.03	BET	0.524121	0.003
550	7-KB-M12-Molibresib-BET family inhibitor	10	BET	0.732975	0
551	7-KB-M13-Mivebresib-BET family inhibitor	100	BET	0.756318	0
552	7-KB-M20-JQ1-BET family inhibitor	10	BET	0.168874	0.95
553	7-KB-M23-ARV-825-BET-targeting PROTAC	0.3	BET	0.496385	0.002
554	7-KB-N12-Molibresib-BET family inhibitor	100	BET	0.510188	0
555	7-KB-N13-Mivebresib-BET family inhibitor	1000	BET	0.718052	0
556	7-KB-N20-JQ1-BET family inhibitor	100	BET	0.719253	0
557	7-KB-N23-ARV-825-BET-targeting PROTAC	3	BET	0.652711	0
558	7-KB-O12-Molibresib-BET family inhibitor	1000	BET	0.739286	0
559	7-KB-O20-JQ1-BET family inhibitor	1000	BET	0.739292	0

560	7-KB-O23-ARV-825-BET-targeting PROTAC	30	BET	0.683159	0
561	7-KB-P12-Molibresib-BET family inhibitor	10000	BET	0.73196	0
562	7-KB-P13-Mivebresib-BET family inhibitor	10000	BET	0.662095	0
563	7-KB-P20-JQ1-BET family inhibitor	10000	BET	0.702951	0
564	7-KB-P23-ARV-825-BET-targeting PROTAC	300	BET	0.74161	0
565	8-KB-K22-CPI-0610-BET family inhibitor	1	BET	0.186237	0.946
566	8-KB-L22-CPI-0610-BET family inhibitor	10	BET	0.467296	0.047
567	8-KB-M22-CPI-0610-BET family inhibitor	100	BET	0.537154	0
568	8-KB-N22-CPI-0610-BET family inhibitor	1000	BET	0.243112	0.905
569	8-KB-O22-CPI-0610-BET family inhibitor	10000	BET	0.356215	0.682
570	1-KB-A3-Vorinostat-HDAC inhibitor	10000	HDAC	0.522788	0
571	1-KB-B3-Vorinostat-HDAC inhibitor	1000	HDAC	0.50183	0
572	1-KB-C3-Vorinostat-HDAC inhibitor	100	HDAC	0.394226	0.001
573	1-KB-D3-Vorinostat-HDAC inhibitor	10	HDAC	0.142569	0.92
574	1-KB-E3-Vorinostat-HDAC inhibitor	1	HDAC	0.33834	0.027
575	1-KB-L12-Romidepsin-HDAC inhibitor	0.1	HDAC	0.34365	0
576	1-KB-M12-Romidepsin-HDAC inhibitor	1	HDAC	0.445381	0
577	1-KB-N12-Romidepsin-HDAC inhibitor	10	HDAC	0.502741	0.001
578	1-KB-O12-Romidepsin-HDAC inhibitor	100	HDAC	0.462134	0.01
579	1-KB-P12-Romidepsin-HDAC inhibitor	1000	HDAC	0.410908	0.122
580	3-KB-A4-Panobinostat-HDAC inhibitor	1000	HDAC	0.507933	0.003
581	3-KB-B4-Panobinostat-HDAC inhibitor	100	HDAC	0.58023	0
582	3-KB-C4-Panobinostat-HDAC inhibitor	10	HDAC	0.521521	0
583	3-KB-D4-Panobinostat-HDAC inhibitor	1	HDAC	0.408896	0.002
584	3-KB-E4-Panobinostat-HDAC inhibitor	0.1	HDAC	0.143786	0.905
585	3-KB-F7-Quisinostat-HDAC inhibitor	1000	HDAC	0.493509	0.003
586	3-KB-G7-Quisinostat-HDAC inhibitor	100	HDAC	0.610409	0
587	3-KB-G12-Valproic acid-HDAC inhibitor	1000000	HDAC	0.327315	0
588	3-KB-H7-Quisinostat-HDAC inhibitor	10	HDAC	0.482262	0
589	3-KB-H12-Valproic acid-HDAC inhibitor	100000	HDAC	0.357784	0.008
590	3-KB-I7-Quisinostat-HDAC inhibitor	1	HDAC	0.069254	0.987
591	3-KB-I12-Valproic acid-HDAC inhibitor	10000	HDAC	0.195178	0.351
592	3-KB-J7-Quisinostat-HDAC inhibitor	0.1	HDAC	0.357288	0.014

593	3-KB-J12-Valproic acid-HDAC inhibitor	1000	HDAC	0.297992	0.001
594	3-KB-K3-Belinostat-HDAC inhibitor	1	HDAC	0.23095	0.741
595	3-KB-K12-Valproic acid-HDAC inhibitor	100	HDAC	0.103374	0.862
596	3-KB-L3-Belinostat-HDAC inhibitor	10	HDAC	0.27548	0.153
597	3-KB-M3-Belinostat-HDAC inhibitor	100	HDAC	0.464129	0
598	3-KB-N3-Belinostat-HDAC inhibitor	1000	HDAC	0.646913	0
599	3-KB-O3-Belinostat-HDAC inhibitor	10000	HDAC	0.343812	0.444
600	7-KB-A5-Mocetinostat-HDAC inhibitor (HDAC1 & 2-selective)	10000	HDAC	0.357554	0.385
601	7-KB-A7-CUDC-907-HDAC1/2/3/10, PI3Kalpha inhibitor	10000	HDAC	0.32572	0.469
602	7-KB-A9-Givinostat-HDAC inhibitor	1000	HDAC	0.679678	0
603	7-KB-A12-Rocilinostat-HDAC-6 selective inhibitor	10000	HDAC	0.653241	0
604	7-KB-B5-Mocetinostat-HDAC inhibitor (HDAC1 & 2-selective)	1000	HDAC	0.519915	0
605	7-KB-B7-CUDC-907-HDAC1/2/3/10, PI3Kalpha inhibitor	1000	HDAC	0.362097	0.343
606	7-KB-B12-Rocilinostat-HDAC-6 selective inhibitor	1000	HDAC	0.465176	0
607	7-KB-C5-Mocetinostat-HDAC inhibitor (HDAC1 & 2-selective)	100	HDAC	0.464104	0
608	7-KB-C7-CUDC-907-HDAC1/2/3/10, PI3Kalpha inhibitor	100	HDAC	0.537672	0
609	7-KB-C9-Givinostat-HDAC inhibitor	100	HDAC	0.474164	0
610	7-KB-D7-CUDC-907-HDAC1/2/3/10, PI3Kalpha inhibitor	10	HDAC	0.51286	0
611	7-KB-D9-Givinostat-HDAC inhibitor	10	HDAC	0.439635	0
612	7-KB-D12-Rocilinostat-HDAC-6 selective inhibitor	100	HDAC	0.342818	0
613	7-KB-E5-Mocetinostat-HDAC inhibitor (HDAC1 & 2-selective)	10	HDAC	0.286284	0.353
614	7-KB-E7-CUDC-907-HDAC1/2/3/10, PI3Kalpha inhibitor	1	HDAC	0.448305	0
615	7-KB-E9-Givinostat-HDAC inhibitor	1	HDAC	0.449508	0
616	7-KB-E12-Rocilinostat-HDAC-6 selective inhibitor	10	HDAC	0.53936	0
617	7-KB-F5-Mocetinostat-HDAC inhibitor (HDAC1 & 2-selective)	1	HDAC	0.101738	0.999
618	7-KB-F7-Resminostat-HDAC1, 3, 6 inhibitor	10000	HDAC	0.533255	0
619	7-KB-F9-Givinostat-HDAC inhibitor	0.1	HDAC	0.379446	0
620	7-KB-F12-Rocilinostat-HDAC-6 selective inhibitor	1	HDAC	0.269754	0.019
621	7-KB-F19-PCI-34051-HDAC8 inhibitor	10000	HDAC	0.332804	0.388
622	7-KB-G7-Resminostat-HDAC1, 3, 6 inhibitor	1000	HDAC	0.507901	0
623	7-KB-G19-PCI-34051-HDAC8 inhibitor	1000	HDAC	0.238887	0.853
624	7-KB-H7-Resminostat-HDAC1, 3, 6 inhibitor	100	HDAC	0.435982	0
625	7-KB-I7-Resminostat-HDAC1, 3, 6 inhibitor	10	HDAC	0.490084	0

626	7-KB-I19-PCI-34051-HDAC8 inhibitor	100	HDAC	0.466789	0
627	7-KB-J7-Resminostat-HDAC1, 3, 6 inhibitor	1	HDAC	0.362301	0.036
628	7-KB-J19-PCI-34051-HDAC8 inhibitor	10	HDAC	0.507356	0
629	7-KB-K4-Entinostat-HDAC inhibitor	1	HDAC	0.363049	0.002
630	7-KB-K11-AR-42-HDAC inhibitor	1	HDAC	0.298732	0.049
631	7-KB-K18-Tubacin-HDAC6 inhibitor	1	HDAC	0.524854	0
632	7-KB-K19-PCI-34051-HDAC8 inhibitor	1	HDAC	0.485967	0
633	7-KB-L2-Tacedinaline-HDAC inhibitor	0.1	HDAC	0.142243	0.985
634	7-KB-L4-Entinostat-HDAC inhibitor	10	HDAC	0.280476	0.158
635	7-KB-L5-Pracinostat-HDAC inhibitor	1	HDAC	0.295395	0.356
636	7-KB-L8-Abexinostat-HDAC1-selective inhibitor	1	HDAC	0.46473	0
637	7-KB-L10-Tucidinostat-HDAC1/2/3/10 inhibitor	1	HDAC	0.377737	0
638	7-KB-L11-AR-42-HDAC inhibitor	10	HDAC	0.515428	0
639	7-KB-L14-Tubastatin A-HDAC6 inhibitor	1	HDAC	0.448139	0
640	7-KB-L16-RGFP966-HDAC3 inhibitor	1	HDAC	0.375409	0
641	7-KB-L18-Tubacin-HDAC6 inhibitor	10	HDAC	0.197977	0.915
642	7-KB-M2-Tacedinaline-HDAC inhibitor	1	HDAC	0.189571	0.862
643	7-KB-M5-Pracinostat-HDAC inhibitor	10	HDAC	0.503623	0
644	7-KB-M8-Abexinostat-HDAC1-selective inhibitor	10	HDAC	0.390136	0
645	7-KB-M10-Tucidinostat-HDAC1/2/3/10 inhibitor	10	HDAC	0.400435	0.018
646	7-KB-M11-AR-42-HDAC inhibitor	100	HDAC	0.448563	0
647	7-KB-M14-Tubastatin A-HDAC6 inhibitor	10	HDAC	0.444199	0.003
648	7-KB-M16-RGFP966-HDAC3 inhibitor	10	HDAC	0.388631	0.035
649	7-KB-M18-Tubacin-HDAC6 inhibitor	100	HDAC	0.271517	0.568
650	7-KB-N2-Tacedinaline-HDAC inhibitor	10	HDAC	0.115606	0.986
651	7-KB-N4-Entinostat-HDAC inhibitor	100	HDAC	0.314533	0.088
652	7-KB-N5-Pracinostat-HDAC inhibitor	100	HDAC	0.462603	0
653	7-KB-N8-Abexinostat-HDAC1-selective inhibitor	100	HDAC	0.481962	0
654	7-KB-N10-Tucidinostat-HDAC1/2/3/10 inhibitor	100	HDAC	0.42928	0
655	7-KB-N14-Tubastatin A-HDAC6 inhibitor	100	HDAC	0.246726	0.088
656	7-KB-N16-RGFP966-HDAC3 inhibitor	100	HDAC	0.178999	0.223
657	7-KB-N18-Tubacin-HDAC6 inhibitor	1000	HDAC	0.256211	0.021
658	7-KB-O2-Tacedinaline-HDAC inhibitor	100	HDAC	0.237251	0.679

**659**	7-KB-O4-Entinostat-HDAC inhibitor	1000	HDAC	0.48855	0
**660**	7-KB-O5-Pracinostat-HDAC inhibitor	1000	HDAC	0.649782	0
**661**	7-KB-O8-Abexinostat-HDAC1-selective inhibitor	1000	HDAC	0.684585	0
**662**	7-KB-O10-Tucidinostat-HDAC1/2/3/10 inhibitor	1000	HDAC	0.498036	0
**663**	7-KB-O11-AR-42-HDAC inhibitor	1000	HDAC	0.567787	0
**664**	7-KB-O14-Tubastatin A-HDAC6 inhibitor	1000	HDAC	0.156764	0.429
**665**	7-KB-O16-RGFP966-HDAC3 inhibitor	1000	HDAC	0.468966	0
**666**	7-KB-P2-Tacedinaline-HDAC inhibitor	1000	HDAC	0.127271	0.756
**667**	7-KB-P4-Entinostat-HDAC inhibitor	10000	HDAC	0.540984	0
**668**	7-KB-P5-Pracinostat-HDAC inhibitor	10000	HDAC	0.391739	0.195
**669**	7-KB-P8-Abexinostat-HDAC1-selective inhibitor	10000	HDAC	0.30196	0.539
**670**	7-KB-P10-Tucidinostat-HDAC1/2/3/10 inhibitor	10000	HDAC	0.559569	0
**671**	7-KB-P11-AR-42-HDAC inhibitor	10000	HDAC	0.44618	0.028
**672**	7-KB-P14-Tubastatin A-HDAC6 inhibitor	10000	HDAC	0.081318	0.961
**673**	7-KB-P16-RGFP966-HDAC3 inhibitor	10000	HDAC	0.442915	0
**674**	7-KB-P18-Tubacin-HDAC6 inhibitor	10000	HDAC	0.440881	0
**0**	2-KW-A16-Gefitinib-EGFR inhibitor	10000	EGFR	0.316468	0.467
**1**	2-KW-A19-Erlotinib-EGFR inhibitor	10000	EGFR	0.497448	0.002
**2**	2-KW-B19-Erlotinib-EGFR inhibitor	1000	EGFR	0.417556	0.013
**3**	2-KW-C16-Gefitinib-EGFR inhibitor	1000	EGFR	0.482408	0.879
**4**	2-KW-C19-Erlotinib-EGFR inhibitor	100	EGFR	0.348943	0.442
**5**	2-KW-D16-Gefitinib-EGFR inhibitor	100	EGFR	0.310364	0.212
**6**	2-KW-D19-Erlotinib-EGFR inhibitor	10	EGFR	0.22919	0.522
**7**	2-KW-E16-Gefitinib-EGFR inhibitor	10	EGFR	0.320638	0.009
**8**	2-KW-E19-Erlotinib-EGFR inhibitor	1	EGFR	0.366803	0.06
**9**	2-KW-F16-Gefitinib-EGFR inhibitor	1	EGFR	0.325479	0.028
**10**	2-KW-K11-Afatinib-EGFR inhibitor	0.1	EGFR	0.112133	0.865
**11**	2-KW-L11-Afatinib-EGFR inhibitor	1	EGFR	0.434799	0.008
**12**	2-KW-L16-Osimertinib-EGFR(L858R/T790M) inhibitor	0.25	EGFR	0.335435	0.384
**13**	2-KW-L19-Lapatinib-HER2, EGFR inhibitor	0.1	EGFR	0.439172	0.004
**14**	2-KW-M11-Afatinib-EGFR inhibitor	10	EGFR	0.301202	0.932
**15**	2-KW-M16-Osimertinib-EGFR(L858R/T790M) inhibitor	2.5	EGFR	0.472742	0.002
**16**	2-KW-M19-Lapatinib-HER2, EGFR inhibitor	1	EGFR	0.42851	0.421

17	2-KW-N16-Osimertinib-EGFR(L858R/T790M) inhibitor	25	EGFR	0.406673	0.023
18	2-KW-N19-Lapatinib-HER2, EGFR inhibitor	10	EGFR	0.277489	0.73
19	2-KW-O11-Afatinib-EGFR inhibitor	100	EGFR	0.50568	0
20	2-KW-O16-Osimertinib-EGFR(L858R/T790M) inhibitor	250	EGFR	0.404121	0.139
21	2-KW-O19-Lapatinib-HER2, EGFR inhibitor	100	EGFR	0.406309	0.099
22	2-KW-P11-Afatinib-EGFR inhibitor	1000	EGFR	0.462641	0.012
23	2-KW-P16-Osimertinib-EGFR(L858R/T790M) inhibitor	2500	EGFR	0.479079	0.007
24	2-KW-P19-Lapatinib-HER2, EGFR inhibitor	1000	EGFR	0.469111	0
25	3-KW-F21-Rociletinib-EGFR(L858R/T790M) inhibitor	10000	EGFR	0.511322	0.002
26	3-KW-G20-Neratinib-EGFR inhibitor	1000	EGFR	0.48452	0.012
27	3-KW-G21-Rociletinib-EGFR(L858R/T790M) inhibitor	1000	EGFR	0.37892	0.088
28	3-KW-H20-Neratinib-EGFR inhibitor	100	EGFR	0.47577	0.001
29	3-KW-H21-Rociletinib-EGFR(L858R/T790M) inhibitor	100	EGFR	0.501521	0
30	3-KW-I20-Neratinib-EGFR inhibitor	10	EGFR	0.482531	0
31	3-KW-I21-Rociletinib-EGFR(L858R/T790M) inhibitor	10	EGFR	0.43573	0.026
32	3-KW-J20-Neratinib-EGFR inhibitor	1	EGFR	0.536244	0.007
33	3-KW-J21-Rociletinib-EGFR(L858R/T790M) inhibitor	1	EGFR	0.38715	0.101
34	3-KW-K4-Canertinib-pan-HER inhibitor	1	EGFR	0.318353	0.404
35	3-KW-K18-Dacomitinib-pan-HER inhibitor	0.1	EGFR	0.425052	0.001
36	3-KW-K20-Neratinib-EGFR inhibitor	0.1	EGFR	0.535169	0
37	3-KW-L4-Canertinib-pan-HER inhibitor	10	EGFR	0.164376	0.935
38	3-KW-L18-Dacomitinib-pan-HER inhibitor	1	EGFR	0.526767	0.007
39	3-KW-M18-Dacomitinib-pan-HER inhibitor	10	EGFR	0.300934	0.101
40	3-KW-N4-Canertinib-pan-HER inhibitor	100	EGFR	0.436984	0.025
41	3-KW-N18-Dacomitinib-pan-HER inhibitor	100	EGFR	0.49937	0
42	3-KW-O4-Canertinib-pan-HER inhibitor	1000	EGFR	0.316132	0.973
43	3-KW-P4-Canertinib-pan-HER inhibitor	10000	EGFR	0.438335	0.137
44	3-KW-P18-Dacomitinib-pan-HER inhibitor	1000	EGFR	0.489476	0.003
45	4-KW-F13-Sapitinib-Pan-HER inhibitor	1000	EGFR	0.377755	0.059
46	4-KW-G13-Sapitinib-Pan-HER inhibitor	100	EGFR	0.510609	0
47	4-KW-G16-Varlitinib-EGFR HER2 inhibitor	10000	EGFR	0.460508	0.008
48	4-KW-H13-Sapitinib-Pan-HER inhibitor	10	EGFR	0.387896	0.023
49	4-KW-H16-Varlitinib-EGFR HER2 inhibitor	1000	EGFR	0.366428	0.014

50	4-KW-I13-Sapitinib-Pan-HER inhibitor	1	EGFR	0.367271	0.105
51	4-KW-I16-Varlitinib-EGFR HER2 inhibitor	100	EGFR	0.241649	0.475
52	4-KW-J13-Sapitinib-Pan-HER inhibitor	0.1	EGFR	0.341531	0.162
53	4-KW-J16-Varlitinib-EGFR HER2 inhibitor	10	EGFR	0.225958	0.328
54	4-KW-K7-Icotinib-EGFR inhibitor	1	EGFR	0.161752	0.853
55	4-KW-K13-Tesevatinib-EGFR, ERBB2, VEGFR, EPHB4	0.1	EGFR	0.172467	0.299
56	4-KW-K16-Varlitinib-EGFR HER2 inhibitor	1	EGFR	0.421557	0.054
57	4-KW-L7-Icotinib-EGFR inhibitor	10	EGFR	0.259986	0.492
58	4-KW-L13-Tesevatinib-EGFR, ERBB2, VEGFR, EPHB4	1	EGFR	0.178178	0.667
59	4-KW-M7-Icotinib-EGFR inhibitor	100	EGFR	0.377432	0.041
60	4-KW-M13-Tesevatinib-EGFR, ERBB2, VEGFR, EPHB4	10	EGFR	0.416171	0.037
61	4-KW-N13-Tesevatinib-EGFR, ERBB2, VEGFR, EPHB4	100	EGFR	0.501639	0.001
62	4-KW-O7-Icotinib-EGFR inhibitor	1000	EGFR	0.236793	0.595
63	4-KW-P7-Icotinib-EGFR inhibitor	10000	EGFR	0.469011	0.004
64	4-KW-P13-Tesevatinib-EGFR, ERBB2, VEGFR, EPHB4	1000	EGFR	0.438894	0.021
65	5-KW-F4-Poziotinib-pan-HER inhibitor	1000	EGFR	0.423204	0.012
66	5-KW-F7-AZD3759-EGFR inhibitor, BBB penetrable	1000	EGFR	0.15606	0.776
67	5-KW-G4-Poziotinib-pan-HER inhibitor	100	EGFR	0.373838	0.087
68	5-KW-G7-AZD3759-EGFR inhibitor, BBB penetrable	100	EGFR	0.328214	0.109
69	5-KW-H4-Poziotinib-pan-HER inhibitor	10	EGFR	0.400821	0.024
70	5-KW-H7-AZD3759-EGFR inhibitor, BBB penetrable	10	EGFR	0.102474	0.916
71	5-KW-I4-Poziotinib-pan-HER inhibitor	1	EGFR	0.12602	0.991
72	5-KW-I7-AZD3759-EGFR inhibitor, BBB penetrable	1	EGFR	0.115545	0.989
73	5-KW-J4-Poziotinib-pan-HER inhibitor	0.1	EGFR	0.339559	0.128
74	5-KW-J7-AZD3759-EGFR inhibitor, BBB penetrable	0.1	EGFR	0.421868	0.019
75	5-KW-K7-Olmutinib-EGFR(L858R/T790M) inhibitor	0.1	EGFR	0.299575	0.544
76	5-KW-L7-Olmutinib-EGFR(L858R/T790M) inhibitor	1	EGFR	0.365095	0.117
77	5-KW-M7-Olmutinib-EGFR(L858R/T790M) inhibitor	10	EGFR	0.402684	0.014
78	5-KW-O7-Olmutinib-EGFR(L858R/T790M) inhibitor	100	EGFR	0.416475	0.015
79	5-KW-P7-Olmutinib-EGFR(L858R/T790M) inhibitor	1000	EGFR	0.301858	0.23
80	2-KW-A15-Lenvatinib-VEGFR inhibitor	2500	VEGFR	0.30019	0.157
81	2-KW-A17-Nintedanib-VEGFR, PDGFR, FGFR inhibitor	10000	VEGFR	0.371654	0.074
82	2-KW-A20-Tivozanib-VEGFR1, 2, 3, c-Kit, PDGFRB inhibitor	10000	VEGFR	0.273846	0.81

83	2-KW-B15-Lenvatinib-VEGFR inhibitor	250	VEGFR	0.430901	0.104
84	2-KW-B17-Nintedanib-VEGFR, PDGFR, FGFR inhibitor	1000	VEGFR	0.480572	0.06
85	2-KW-B20-Tivozanib-VEGFR1, 2, 3, c-Kit, PDGFRB inhibitor	1000	VEGFR	0.180355	0.932
86	2-KW-C15-Lenvatinib-VEGFR inhibitor	25	VEGFR	0.515998	0.206
87	2-KW-C17-Nintedanib-VEGFR, PDGFR, FGFR inhibitor	100	VEGFR	0.398461	0.003
88	2-KW-D15-Lenvatinib-VEGFR inhibitor	2.5	VEGFR	0.341856	0.302
89	2-KW-D17-Nintedanib-VEGFR, PDGFR, FGFR inhibitor	10	VEGFR	0.510311	0.218
90	2-KW-D20-Tivozanib-VEGFR1, 2, 3, c-Kit, PDGFRB inhibitor	100	VEGFR	0.278606	0.79
91	2-KW-E17-Nintedanib-VEGFR, PDGFR, FGFR inhibitor	1	VEGFR	0.481244	0.291
92	2-KW-E20-Tivozanib-VEGFR1, 2, 3, c-Kit, PDGFRB inhibitor	10	VEGFR	0.258499	0.801
93	2-KW-F13-Axitinib-VEGFR, PDGFR, KIT inhibitor	10000	VEGFR	0.213588	0.818
94	2-KW-F15-Lenvatinib-VEGFR inhibitor	0.25	VEGFR	0.336528	0.008
95	2-KW-F19-Regorafenib-B-Raf, c-Kit, VEGFR2 inhibitor	10000	VEGFR	0.198051	0.897
96	2-KW-F20-Tivozanib-VEGFR1, 2, 3, c-Kit, PDGFRB inhibitor	1	VEGFR	0.35998	0.016
97	2-KW-F21-Vatalanib-VEGFR-1 & -2 inhibitor	10000	VEGFR	0.223371	0.901
98	2-KW-G10-Apatinib-VEGFR inhibitor	10000	VEGFR	0.354317	0.001
99	2-KW-G13-Axitinib-VEGFR, PDGFR, KIT inhibitor	1000	VEGFR	0.222155	0.949
100	2-KW-G19-Regorafenib-B-Raf, c-Kit, VEGFR2 inhibitor	1000	VEGFR	0.299327	0.397
101	2-KW-G21-Vatalanib-VEGFR-1 & -2 inhibitor	1000	VEGFR	0.405325	0.182
102	2-KW-H10-Apatinib-VEGFR inhibitor	1000	VEGFR	0.331839	0.048
103	2-KW-H13-Axitinib-VEGFR, PDGFR, KIT inhibitor	100	VEGFR	0.541205	0.248
104	2-KW-H21-Vatalanib-VEGFR-1 & -2 inhibitor	100	VEGFR	0.315546	0.04
105	2-KW-I10-Apatinib-VEGFR inhibitor	100	VEGFR	0.488985	0.278
106	2-KW-I13-Axitinib-VEGFR, PDGFR, KIT inhibitor	10	VEGFR	0.270295	0.486
107	2-KW-I19-Regorafenib-B-Raf, c-Kit, VEGFR2 inhibitor	100	VEGFR	0.299323	0.453
108	2-KW-I21-Vatalanib-VEGFR-1 & -2 inhibitor	10	VEGFR	0.226077	0.909
109	2-KW-J10-Apatinib-VEGFR inhibitor	10	VEGFR	0.34506	0.001
110	2-KW-J13-Axitinib-VEGFR, PDGFR, KIT inhibitor	1	VEGFR	0.307826	0.023
111	2-KW-J19-Regorafenib-B-Raf, c-Kit, VEGFR2 inhibitor	10	VEGFR	0.252455	0.91
112	2-KW-J21-Vatalanib-VEGFR-1 & -2 inhibitor	1	VEGFR	0.351023	0.128
113	2-KW-K10-Apatinib-VEGFR inhibitor	1	VEGFR	0.261531	0.733
114	2-KW-K13-Vandetanib-VEGFR,EGFR, RET inhibitor	0.1	VEGFR	0.402975	0.071
115	2-KW-K17-Pazopanib-VEGFR inhibitor	1	VEGFR	0.396878	0

116	2-KW-K19-Regorafenib-B-Raf, c-Kit, VEGFR2 inhibitor	1	VEGFR	0.408061	0.043
117	2-KW-L12-Sorafenib-B-Raf, FGFR-1, VEGFR-2 & -3, PDGFR-beta, KIT, and FLT3 inhib	0.1	VEGFR	0.50398	0.388
118	2-KW-L13-Vandetanib-VEGFR,EGFR, RET inhibitor	1	VEGFR	0.327207	0.016
119	2-KW-L21-Cediranib-KDR/Flt/VEGFR inhibitor	0.1	VEGFR	0.292192	0.397
120	2-KW-M12-Sorafenib-B-Raf, FGFR-1, VEGFR-2 & -3, PDGFR-beta, KIT, and FLT3 inhib	1	VEGFR	0.369963	0.001
121	2-KW-M13-Vandetanib-VEGFR,EGFR, RET inhibitor	10	VEGFR	0.421006	0
122	2-KW-M17-Pazopanib-VEGFR inhibitor	10	VEGFR	0.317861	0.23
123	2-KW-M21-Cediranib-KDR/Flt/VEGFR inhibitor	1	VEGFR	0.341261	0.656
124	2-KW-N12-Sorafenib-B-Raf, FGFR-1, VEGFR-2 & -3, PDGFR-beta, KIT, and FLT3 inhib	10	VEGFR	0.32534	0.086
125	2-KW-N13-Vandetanib-VEGFR,EGFR, RET inhibitor	100	VEGFR	0.345692	0.289
126	2-KW-N17-Pazopanib-VEGFR inhibitor	100	VEGFR	0.347162	0.01
127	2-KW-N21-Cediranib-KDR/Flt/VEGFR inhibitor	10	VEGFR	0.457355	0.001
128	2-KW-O12-Sorafenib-B-Raf, FGFR-1, VEGFR-2 & -3, PDGFR-beta, KIT, and FLT3 inhib	100	VEGFR	0.223374	0.923
129	2-KW-O17-Pazopanib-VEGFR inhibitor	1000	VEGFR	0.226001	0.681
130	2-KW-O21-Cediranib-KDR/Flt/VEGFR inhibitor	100	VEGFR	0.250022	0.893
131	2-KW-P12-Sorafenib-B-Raf, FGFR-1, VEGFR-2 & -3, PDGFR-beta, KIT, and FLT3 inhib	1000	VEGFR	0.364448	0.033
132	2-KW-P13-Vandetanib-VEGFR,EGFR, RET inhibitor	1000	VEGFR	0.324373	0.688
133	2-KW-P17-Pazopanib-VEGFR inhibitor	10000	VEGFR	0.27216	0.927
134	2-KW-P21-Cediranib-KDR/Flt/VEGFR inhibitor	1000	VEGFR	0.411793	0.012
135	3-KW-A3-Cabozantinib-VEGFR2, Met, FLT3, Tie2, Kit and Ret inhibitor	1000	VEGFR	0.346538	0.005
136	3-KW-A6-Foretinib-MET, VEGFR2 inhibitor	1000	VEGFR	0.250045	0.737
137	3-KW-A18-Linifanib-VEGFR, PDGFR, CSF-1R, FLT3 inhibitor	1000	VEGFR	0.354326	0.02
138	3-KW-B3-Cabozantinib-VEGFR2, Met, FLT3, Tie2, Kit and Ret inhibitor	100	VEGFR	0.2816	0.082
139	3-KW-B6-Foretinib-MET, VEGFR2 inhibitor	100	VEGFR	0.3558	0
140	3-KW-B18-Linifanib-VEGFR, PDGFR, CSF-1R, FLT3 inhibitor	100	VEGFR	0.402788	0.001
141	3-KW-C3-Cabozantinib-VEGFR2, Met, FLT3, Tie2, Kit and Ret inhibitor	10	VEGFR	0.395406	0.058
142	3-KW-C6-Foretinib-MET, VEGFR2 inhibitor	10	VEGFR	0.287134	0.759
143	3-KW-C18-Linifanib-VEGFR, PDGFR, CSF-1R, FLT3 inhibitor	10	VEGFR	0.346232	0.577
144	3-KW-D3-Cabozantinib-VEGFR2, Met, FLT3, Tie2, Kit and Ret inhibitor	1	VEGFR	0.237061	0.926
145	3-KW-D6-Foretinib-MET, VEGFR2 inhibitor	1	VEGFR	0.306708	0.045
146	3-KW-D18-Linifanib-VEGFR, PDGFR, CSF-1R, FLT3 inhibitor	1	VEGFR	0.346636	0.29
147	3-KW-E3-Cabozantinib-VEGFR2, Met, FLT3, Tie2, Kit and Ret inhibitor	0.1	VEGFR	0.22634	0.397
148	3-KW-E6-Foretinib-MET, VEGFR2 inhibitor	0.1	VEGFR	0.440723	0.001

149	3-KW-E18-Linifanib-VEGFR, PDGFR, CSF-1R, FLT3 inhibitor	0.1	VEGFR	0.453078	0.011
150	3-KW-F18-Brivanib-VEGFR inhibitor	1000	VEGFR	0.462415	0
151	3-KW-G18-Brivanib-VEGFR inhibitor	100	VEGFR	0.295923	0.122
152	3-KW-H18-Brivanib-VEGFR inhibitor	10	VEGFR	0.293388	0.589
153	3-KW-I18-Brivanib-VEGFR inhibitor	1	VEGFR	0.541112	0
154	3-KW-J18-Brivanib-VEGFR inhibitor	0.1	VEGFR	0.431738	0.004
155	4-KW-A12-ENMD-2076-pan-Aurora, VEGFR inhibitor	10000	VEGFR	0.328864	0.601
156	4-KW-A15-Golvatinib-MET, VEGFR2 inhibitor	2500	VEGFR	0.193669	0.746
157	4-KW-A20-Motesanib-VEGFR, PDGFR, Ret, Kit inhibitor	10000	VEGFR	0.143593	0.988
158	4-KW-B12-ENMD-2076-pan-Aurora, VEGFR inhibitor	1000	VEGFR	0.309995	0.539
159	4-KW-B15-Golvatinib-MET, VEGFR2 inhibitor	250	VEGFR	0.26517	0.027
160	4-KW-B20-Motesanib-VEGFR, PDGFR, Ret, Kit inhibitor	1000	VEGFR	0.19358	0.606
161	4-KW-C15-Golvatinib-MET, VEGFR2 inhibitor	25	VEGFR	0.196697	0.491
162	4-KW-D12-ENMD-2076-pan-Aurora, VEGFR inhibitor	100	VEGFR	0.304765	0.55
163	4-KW-D15-Golvatinib-MET, VEGFR2 inhibitor	2.5	VEGFR	0.180897	0.325
164	4-KW-D20-Motesanib-VEGFR, PDGFR, Ret, Kit inhibitor	100	VEGFR	0.238126	0.223
165	4-KW-E12-ENMD-2076-pan-Aurora, VEGFR inhibitor	10	VEGFR	0.226423	0.029
166	4-KW-E20-Motesanib-VEGFR, PDGFR, Ret, Kit inhibitor	10	VEGFR	0.207842	0.414
167	4-KW-F12-ENMD-2076-pan-Aurora, VEGFR inhibitor	1	VEGFR	0.230715	0.18
168	4-KW-F15-Golvatinib-MET, VEGFR2 inhibitor	0.25	VEGFR	0.209749	0.922
169	4-KW-F20-Motesanib-VEGFR, PDGFR, Ret, Kit inhibitor	1	VEGFR	0.246027	0.13
170	4-KW-L16-Telatinib-VEGFR, KIT, PDGFR inhibitor	1	VEGFR	0.252633	0.439
171	4-KW-M16-Telatinib-VEGFR, KIT, PDGFR inhibitor	10	VEGFR	0.324678	0.026
172	4-KW-N16-Telatinib-VEGFR, KIT, PDGFR inhibitor	100	VEGFR	0.336184	0.004
173	4-KW-O16-Telatinib-VEGFR, KIT, PDGFR inhibitor	1000	VEGFR	0.179574	0.954
174	4-KW-P16-Telatinib-VEGFR, KIT, PDGFR inhibitor	10000	VEGFR	0.191356	0.939
175	2-KW-L10-Idelalisib-PI3K inhibitor, p110δ-selective	1	PI3K	0.387494	0.003
176	2-KW-M10-Idelalisib-PI3K inhibitor, p110δ-selective	10	PI3K	0.277667	0.034
177	2-KW-N10-Idelalisib-PI3K inhibitor, p110δ-selective	100	PI3K	0.251508	0.5
178	2-KW-O10-Idelalisib-PI3K inhibitor, p110δ-selective	1000	PI3K	0.393289	0.004
179	2-KW-P10-Idelalisib-PI3K inhibitor, p110δ-selective	10000	PI3K	0.37509	0.005
180	3-KW-A16-Perifosine-AKT/PI3K inhibitor	2500	PI3K	0.191788	0.394
181	3-KW-C16-Perifosine-AKT/PI3K inhibitor	250	PI3K	0.277839	0.218

182	3-KW-D16-Perifosine-AKT/PI3K inhibitor	25	PI3K	0.244789	0.047
183	3-KW-E16-Perifosine-AKT/PI3K inhibitor	2.5	PI3K	0.301432	0.019
184	3-KW-F16-Perifosine-AKT/PI3K inhibitor	0.25	PI3K	0.170659	0.903
185	3-KW-F17-Miltefosine-Antimicrobial, inhibits PI3K/AKT	100000	PI3K	0.352012	0.521
186	3-KW-F19-Duvelisib-PI3K inhibitor	500	PI3K	0.401161	0.003
187	3-KW-G17-Miltefosine-Antimicrobial, inhibits PI3K/AKT	10000	PI3K	0.262	0.857
188	3-KW-G19-Duvelisib-PI3K inhibitor	50	PI3K	0.340424	0.013
189	3-KW-H17-Miltefosine-Antimicrobial, inhibits PI3K/AKT	1000	PI3K	0.263863	0.2
190	3-KW-I17-Miltefosine-Antimicrobial, inhibits PI3K/AKT	100	PI3K	0.359988	0.091
191	3-KW-I19-Duvelisib-PI3K inhibitor	5	PI3K	0.365277	0.089
192	3-KW-J17-Miltefosine-Antimicrobial, inhibits PI3K/AKT	10	PI3K	0.33283	0.401
193	3-KW-J19-Duvelisib-PI3K inhibitor	0.5	PI3K	0.513732	0.001
194	3-KW-K19-Duvelisib-PI3K inhibitor	0.05	PI3K	0.416361	0.001
195	3-KW-L8-Pictilisib-PI3K inhibitor, pan-class I	1	PI3K	0.257223	0.368
196	3-KW-L21-Taselisib-PI3K alpha, delta, (gamma) selective inhibitor	0.1	PI3K	0.2765	0.464
197	3-KW-M8-Pictilisib-PI3K inhibitor, pan-class I	10	PI3K	0.280991	0.257
198	3-KW-M21-Taselisib-PI3K alpha, delta, (gamma) selective inhibitor	1	PI3K	0.286543	0.42
199	3-KW-N8-Pictilisib-PI3K inhibitor, pan-class I	100	PI3K	0.571195	0.012
200	3-KW-N21-Taselisib-PI3K alpha, delta, (gamma) selective inhibitor	10	PI3K	0.349466	0.152
201	3-KW-O8-Pictilisib-PI3K inhibitor, pan-class I	1000	PI3K	0.442023	0.008
202	3-KW-O21-Taselisib-PI3K alpha, delta, (gamma) selective inhibitor	100	PI3K	0.373332	0.018
203	3-KW-P8-Pictilisib-PI3K inhibitor, pan-class I	10000	PI3K	0.348645	0.4
204	3-KW-P21-Taselisib-PI3K alpha, delta, (gamma) selective inhibitor	1000	PI3K	0.404034	0.037
205	4-KW-A19-Alpelisib-PI3Kalpha inhibitor	2500	PI3K	0.350886	0.1
206	4-KW-B19-Alpelisib-PI3Kalpha inhibitor	250	PI3K	0.222073	0.756
207	4-KW-C19-Alpelisib-PI3Kalpha inhibitor	25	PI3K	0.135543	0.773
208	4-KW-D19-Alpelisib-PI3Kalpha inhibitor	2.5	PI3K	0.157358	0.642
209	4-KW-E19-Alpelisib-PI3Kalpha inhibitor	0.25	PI3K	0.242362	0.48
210	4-KW-F14-NVP-BGT226-PI3K/mTOR inhibitor	1000	PI3K	0.293574	0.895
211	4-KW-G2-TGR-1202-PI3Kdelta inhibitor	2500	PI3K	0.360422	0.333
212	4-KW-G5-Sonolisib-PI3K inhibitor, pan-class I. Irreversible	10000	PI3K	0.387971	0.062
213	4-KW-G14-NVP-BGT226-PI3K/mTOR inhibitor	100	PI3K	0.271641	0.77
214	4-KW-G20-Buparlisib-PI3K inhibitor, pan-class I	10000	PI3K	0.378786	0.237

215	4-KW-H2-TGR-1202-PI3Kdelta inhibitor	250	PI3K	0.382654	0.18
216	4-KW-H5-Sonolisib-PI3K inhibitor, pan-class I. Irreversible	1000	PI3K	0.198239	0.066
217	4-KW-H14-NVP-BGT226-PI3K/mTOR inhibitor	10	PI3K	0.342407	0.273
218	4-KW-H20-Buparlisib-PI3K inhibitor, pan-class I	1000	PI3K	0.407473	0.009
219	4-KW-I2-TGR-1202-PI3Kdelta inhibitor	25	PI3K	0.249427	0.318
220	4-KW-I5-Sonolisib-PI3K inhibitor, pan-class I. Irreversible	100	PI3K	0.273852	0.262
221	4-KW-I14-NVP-BGT226-PI3K/mTOR inhibitor	1	PI3K	0.121646	0.885
222	4-KW-I20-Buparlisib-PI3K inhibitor, pan-class I	100	PI3K	0.232699	0.295
223	4-KW-J2-TGR-1202-PI3Kdelta inhibitor	2.5	PI3K	0.261197	0.19
224	4-KW-J5-Sonolisib-PI3K inhibitor, pan-class I. Irreversible	10	PI3K	0.339941	0.164
225	4-KW-J20-Buparlisib-PI3K inhibitor, pan-class I	10	PI3K	0.360058	0.147
226	4-KW-K2-TGR-1202-PI3Kdelta inhibitor	0.25	PI3K	0.326712	0.189
227	4-KW-K4-Dactolisib-mTOR/(PI3K) inhibitor	0.1	PI3K	0.312305	0.551
228	4-KW-K5-Sonolisib-PI3K inhibitor, pan-class I. Irreversible	1	PI3K	0.36633	0.14
229	4-KW-K14-NVP-BGT226-PI3K/mTOR inhibitor	0.1	PI3K	0.191267	0.778
230	4-KW-K20-Buparlisib-PI3K inhibitor, pan-class I	1	PI3K	0.309266	0.371
231	4-KW-L4-Dactolisib-mTOR/(PI3K) inhibitor	1	PI3K	0.332481	0.762
232	4-KW-L14-Gedatolisib-PI3K/mTOR inhibitor	0.1	PI3K	0.190165	0.828
233	4-KW-L15-TG100-115-PI3K gamma/delta inhibitor	1	PI3K	0.187691	0.235
234	4-KW-L21-Copanlisib-PI3K alpha, delta selective inhibitor	0.1	PI3K	0.228205	0.068
235	4-KW-M14-Gedatolisib-PI3K/mTOR inhibitor	1	PI3K	0.199872	0.188
236	4-KW-M15-TG100-115-PI3K gamma/delta inhibitor	10	PI3K	0.107812	0.904
237	4-KW-M21-Copanlisib-PI3K alpha, delta selective inhibitor	1	PI3K	0.299638	0.435
238	4-KW-N4-Dactolisib-mTOR/(PI3K) inhibitor	10	PI3K	0.275706	0.57
239	4-KW-N14-Gedatolisib-PI3K/mTOR inhibitor	10	PI3K	0.307619	0.397
240	4-KW-N15-TG100-115-PI3K gamma/delta inhibitor	100	PI3K	0.179028	0.67
241	4-KW-N21-Copanlisib-PI3K alpha, delta selective inhibitor	10	PI3K	0.432651	0
242	4-KW-O4-Dactolisib-mTOR/(PI3K) inhibitor	100	PI3K	0.382879	0.698
243	4-KW-O14-Gedatolisib-PI3K/mTOR inhibitor	100	PI3K	0.283385	0.688
244	4-KW-O15-TG100-115-PI3K gamma/delta inhibitor	1000	PI3K	0.215241	0.692
245	4-KW-O21-Copanlisib-PI3K alpha, delta selective inhibitor	100	PI3K	0.334493	0.118
246	4-KW-P4-Dactolisib-mTOR/(PI3K) inhibitor	1000	PI3K	0.279835	0.732
247	4-KW-P14-Gedatolisib-PI3K/mTOR inhibitor	1000	PI3K	0.342405	0.394

248	4-KW-P15-TG100-115-PI3K gamma/delta inhibitor	10000	PI3K	0.251747	0.067
249	4-KW-P21-Copanlisib-PI3K alpha, delta selective inhibitor	1000	PI3K	0.343282	0.396
250	5-KW-A6-LY3023414-PI3K/mTOR/DNA-PK inhibitor	2500	PI3K	0.228997	0.886
251	5-KW-A7-AMG319-PI3Kdelta inhibitor	1000	PI3K	0.171488	0.04
252	5-KW-A16-AZD-6482-PI3Kbeta-selective inhibitor	2500	PI3K	0.36079	0.005
253	5-KW-A17-Palomid-529-AKT, MTOR, PI3K inhibitor	10000	PI3K	0.247722	0.194
254	5-KW-B6-LY3023414-PI3K/mTOR/DNA-PK inhibitor	250	PI3K	0.242154	0.81
255	5-KW-B7-AMG319-PI3Kdelta inhibitor	100	PI3K	0.106432	0.298
256	5-KW-B17-Palomid-529-AKT, MTOR, PI3K inhibitor	1000	PI3K	0.202372	0.054
257	5-KW-C6-LY3023414-PI3K/mTOR/DNA-PK inhibitor	25	PI3K	0.233166	0.628
258	5-KW-C7-AMG319-PI3Kdelta inhibitor	10	PI3K	0.173837	0.061
259	5-KW-C16-AZD-6482-PI3Kbeta-selective inhibitor	250	PI3K	0.159379	0.257
260	5-KW-C17-Palomid-529-AKT, MTOR, PI3K inhibitor	100	PI3K	0.302023	0
261	5-KW-D6-LY3023414-PI3K/mTOR/DNA-PK inhibitor	2.5	PI3K	0.363079	0.078
262	5-KW-D7-AMG319-PI3Kdelta inhibitor	1	PI3K	0.222393	0.006
263	5-KW-D16-AZD-6482-PI3Kbeta-selective inhibitor	25	PI3K	0.269257	0.003
264	5-KW-D17-Palomid-529-AKT, MTOR, PI3K inhibitor	10	PI3K	0.147723	0.802
265	5-KW-E6-LY3023414-PI3K/mTOR/DNA-PK inhibitor	0.25	PI3K	0.250091	0.376
266	5-KW-E7-AMG319-PI3Kdelta inhibitor	0.1	PI3K	0.197245	0.066
267	5-KW-E16-AZD-6482-PI3Kbeta-selective inhibitor	2.5	PI3K	0.185568	0.947
268	5-KW-E17-Palomid-529-AKT, MTOR, PI3K inhibitor	1	PI3K	0.386722	0.017
269	5-KW-F11-GSK2636771-PI3K beta selective inhibitor	10000	PI3K	0.445492	0
270	5-KW-F16-AZD-6482-PI3Kbeta-selective inhibitor	0.25	PI3K	0.194498	0.042
271	5-KW-G9-Serabelisib-PI3Kalpha selective inhibitor	10000	PI3K	0.357869	0.017
272	5-KW-G11-GSK2636771-PI3K beta selective inhibitor	1000	PI3K	0.233746	0.004
273	5-KW-H9-Serabelisib-PI3Kalpha selective inhibitor	1000	PI3K	0.249306	0.03
274	5-KW-H11-GSK2636771-PI3K beta selective inhibitor	100	PI3K	0.336202	0
275	5-KW-I9-Serabelisib-PI3Kalpha selective inhibitor	100	PI3K	0.23974	0.291
276	5-KW-I11-GSK2636771-PI3K beta selective inhibitor	10	PI3K	0.177651	0.82
277	5-KW-J9-Serabelisib-PI3Kalpha selective inhibitor	10	PI3K	0.329011	0.111
278	5-KW-J11-GSK2636771-PI3K beta selective inhibitor	1	PI3K	0.238184	0.031
279	5-KW-K9-Serabelisib-PI3Kalpha selective inhibitor	1	PI3K	0.293893	0.128
280	5-KW-L14-AZD-8186-PI3Kbeta inhibitor	0.1	PI3K	0.193498	0.706

281	5-KW-L20-ZSTK474-PI3K gamma selective inhibitor	1	PI3K	0.228062	0.607
282	5-KW-L23-Omipalisib-PI3K/mTOR inhibitor	0.1	PI3K	0.189772	0.487
283	5-KW-M14-AZD-8186-PI3Kbeta inhibitor	1	PI3K	0.27464	0.245
284	5-KW-M20-ZSTK474-PI3K gamma selective inhibitor	10	PI3K	0.206049	0.736
285	5-KW-M23-Omipalisib-PI3K/mTOR inhibitor	1	PI3K	0.353554	0.693
286	5-KW-N14-AZD-8186-PI3Kbeta inhibitor	10	PI3K	0.358926	0.03
287	5-KW-N20-ZSTK474-PI3K gamma selective inhibitor	100	PI3K	0.283743	0.262
288	5-KW-N23-Omipalisib-PI3K/mTOR inhibitor	10	PI3K	0.313511	0.121
289	5-KW-O14-AZD-8186-PI3Kbeta inhibitor	100	PI3K	0.479309	0
290	5-KW-O20-ZSTK474-PI3K gamma selective inhibitor	1000	PI3K	0.344416	0.109
291	5-KW-O23-Omipalisib-PI3K/mTOR inhibitor	100	PI3K	0.283754	0.568
292	5-KW-P14-AZD-8186-PI3Kbeta inhibitor	1000	PI3K	0.368721	0.005
293	5-KW-P20-ZSTK474-PI3K gamma selective inhibitor	10000	PI3K	0.289698	0.538
294	5-KW-P23-Omipalisib-PI3K/mTOR inhibitor	1000	PI3K	0.326961	0.293
295	6-KW-A8-TGX-221-PI3K beta selective inhibitor	10000	PI3K	0.213502	0.172
296	6-KW-B8-TGX-221-PI3K beta selective inhibitor	1000	PI3K	0.187137	0.164
297	6-KW-C8-TGX-221-PI3K beta selective inhibitor	100	PI3K	0.268521	0.01
298	6-KW-D8-TGX-221-PI3K beta selective inhibitor	10	PI3K	0.237754	0.783
299	6-KW-E8-TGX-221-PI3K beta selective inhibitor	1	PI3K	0.239667	0.849
300	6-KW-L6-GDC-0084-PI3K/mTOR inhibitor	1	PI3K	0.221208	0.762
301	6-KW-M6-GDC-0084-PI3K/mTOR inhibitor	10	PI3K	0.202388	0.865
302	6-KW-N6-GDC-0084-PI3K/mTOR inhibitor	100	PI3K	0.21716	0.795
303	6-KW-O6-GDC-0084-PI3K/mTOR inhibitor	1000	PI3K	0.300451	0.399
304	6-KW-P6-GDC-0084-PI3K/mTOR inhibitor	10000	PI3K	0.306192	0.572
305	1-KW-F11-Amsacrine-DNA intercalation, Topo II inhibitor	10000	Topoisome	0.709814	0
306	1-KW-G11-Amsacrine-DNA intercalation, Topo II inhibitor	1000	Topoisome	0.619539	0.005
307	1-KW-G20-Epirubicin-Topoisomerase II inhibitor	1000	Topoisome	0.536464	0.02
308	1-KW-H11-Amsacrine-DNA intercalation, Topo II inhibitor	100	Topoisome	0.698272	0
309	1-KW-H20-Epirubicin-Topoisomerase II inhibitor	100	Topoisome	0.694123	0
310	1-KW-I11-Amsacrine-DNA intercalation, Topo II inhibitor	10	Topoisome	0.757012	0
311	1-KW-I20-Epirubicin-Topoisomerase II inhibitor	10	Topoisome	0.579565	0.009
312	1-KW-J11-Amsacrine-DNA intercalation, Topo II inhibitor	1	Topoisome	0.450837	0.001
313	1-KW-J20-Epirubicin-Topoisomerase II inhibitor	1	Topoisome	0.741624	0

314	1-KW-K11-SN-38-Active metabolite of irinotecan. Topoisomerase I inhibitor	1	Topoisome	0.752014	0
315	1-KW-K20-Epirubicin-Topoisomerase II inhibitor	0.1	Topoisome	0.756177	0
316	1-KW-L11-SN-38-Active metabolite of irinotecan. Topoisomerase I inhibitor	10	Topoisome	0.670158	0.001
317	1-KW-L14-Topotecan-Topoisomerase I inhibitor. Camptothecin analog	1	Topoisome	0.755734	0
318	1-KW-M11-SN-38-Active metabolite of irinotecan. Topoisomerase I inhibitor	100	Topoisome	0.53353	0.023
319	1-KW-M14-Topotecan-Topoisomerase I inhibitor. Camptothecin analog	10	Topoisome	0.451211	0.004
320	1-KW-N14-Topotecan-Topoisomerase I inhibitor. Camptothecin analog	100	Topoisome	0.613782	0.006
321	1-KW-O11-SN-38-Active metabolite of irinotecan. Topoisomerase I inhibitor	1000	Topoisome	0.463988	0.038
322	1-KW-O14-Topotecan-Topoisomerase I inhibitor. Camptothecin analog	1000	Topoisome	0.541478	0.013
323	1-KW-P11-SN-38-Active metabolite of irinotecan. Topoisomerase I inhibitor	10000	Topoisome	0.309532	0.842
324	1-KW-P14-Topotecan-Topoisomerase I inhibitor. Camptothecin analog	10000	Topoisome	0.493129	0.009
325	3-KW-A11-Etoposide-Topoisomerase II inhibitor	10000	Topoisome	0.563301	0.001
326	3-KW-B11-Etoposide-Topoisomerase II inhibitor	1000	Topoisome	0.55653	0.001
327	3-KW-C11-Etoposide-Topoisomerase II inhibitor	100	Topoisome	0.526418	0
328	3-KW-D11-Etoposide-Topoisomerase II inhibitor	10	Topoisome	0.515986	0.012
329	3-KW-E11-Etoposide-Topoisomerase II inhibitor	1	Topoisome	0.260632	0.483
330	3-KW-G9-Daunorubicin-Topoisomerase II inhibitor	1000	Topoisome	0.46841	0.044
331	3-KW-G10-Teniposide-Topoisomerase II inhibitor	10000	Topoisome	0.294759	0.008
332	3-KW-H9-Daunorubicin-Topoisomerase II inhibitor	100	Topoisome	0.543537	0
333	3-KW-H10-Teniposide-Topoisomerase II inhibitor	1000	Topoisome	0.366963	0.008
334	3-KW-I9-Daunorubicin-Topoisomerase II inhibitor	10	Topoisome	0.57927	0
335	3-KW-I10-Teniposide-Topoisomerase II inhibitor	100	Topoisome	0.258818	0.112
336	3-KW-J9-Daunorubicin-Topoisomerase II inhibitor	1	Topoisome	0.521785	0
337	3-KW-J10-Teniposide-Topoisomerase II inhibitor	10	Topoisome	0.553585	0
338	3-KW-K7-Idarubicin-Topoisomerase II inhibitor	0.1	Topoisome	0.553717	0
339	3-KW-K9-Daunorubicin-Topoisomerase II inhibitor	0.1	Topoisome	0.586928	0
340	3-KW-K10-Teniposide-Topoisomerase II inhibitor	1	Topoisome	0.546182	0
341	3-KW-L6-Doxorubicin-Topoisomerase II inhibitor	0.1	Topoisome	0.130991	0.929
342	3-KW-L7-Idarubicin-Topoisomerase II inhibitor	1	Topoisome	0.32795	0.026
343	3-KW-L9-Valrubicin-Topoisomerase II inhibitor	0.5	Topoisome	0.591277	0
344	3-KW-L10-Mitoxantrone-Topoisomerase II inhibitor	0.1	Topoisome	0.456924	0
345	3-KW-L16-Pixantrone-topoisomerase II inhibitor	1	Topoisome	0.426334	0
346	3-KW-M6-Doxorubicin-Topoisomerase II inhibitor	1	Topoisome	0.531908	0

347	3-KW-M7-Idarubicin-Topoisomerase II inhibitor	10	Topoisome	0.599409	0
348	3-KW-M9-Valrubicin-Topoisomerase II inhibitor	5	Topoisome	0.61656	0
349	3-KW-M10-Mitoxantrone-Topoisomerase II inhibitor	1	Topoisome	0.458401	0.001
350	3-KW-M16-Pixantrone-topoisomerase II inhibitor	10	Topoisome	0.573401	0
351	3-KW-N6-Doxorubicin-Topoisomerase II inhibitor	10	Topoisome	0.637959	0
352	3-KW-N9-Valrubicin-Topoisomerase II inhibitor	50	Topoisome	0.559821	0
353	3-KW-N10-Mitoxantrone-Topoisomerase II inhibitor	10	Topoisome	0.460967	0.028
354	3-KW-N16-Pixantrone-topoisomerase II inhibitor	100	Topoisome	0.468862	0.043
355	3-KW-O6-Doxorubicin-Topoisomerase II inhibitor	100	Topoisome	0.538184	0.002
356	3-KW-O7-Idarubicin-Topoisomerase II inhibitor	100	Topoisome	0.536196	0.001
357	3-KW-O9-Valrubicin-Topoisomerase II inhibitor	500	Topoisome	0.54754	0
358	3-KW-O10-Mitoxantrone-Topoisomerase II inhibitor	100	Topoisome	0.560576	0.001
359	3-KW-O16-Pixantrone-topoisomerase II inhibitor	1000	Topoisome	0.500156	0.009
360	3-KW-P6-Doxorubicin-Topoisomerase II inhibitor	1000	Topoisome	0.477988	0.028
361	3-KW-P7-Idarubicin-Topoisomerase II inhibitor	1000	Topoisome	0.354726	0.532
362	3-KW-P9-Valrubicin-Topoisomerase II inhibitor	5000	Topoisome	0.520518	0
363	3-KW-P10-Mitoxantrone-Topoisomerase II inhibitor	1000	Topoisome	0.507811	0.004
364	3-KW-P16-Pixantrone-topoisomerase II inhibitor	10000	Topoisome	0.203785	0.963
365	1-KW-A10-Vinorelbine-Mitotic inhibitor. Vinca alkaloid microtubule depolymerizer	10000	Mitotic	0.62914	0
366	1-KW-A13-Ixabepilone-Mitotic inhibitor. Epothilone microtubule stabilizer.	1000	Mitotic	0.636451	0
367	1-KW-A18-Paclitaxel-Mitotic inhibitor, taxane microtubule stabilizer	1000	Mitotic	0.663086	0.02
368	1-KW-B10-Vinorelbine-Mitotic inhibitor. Vinca alkaloid microtubule depolymerizer	1000	Mitotic	0.693287	0
369	1-KW-B13-Ixabepilone-Mitotic inhibitor. Epothilone microtubule stabilizer.	100	Mitotic	0.752762	0.001
370	1-KW-B18-Paclitaxel-Mitotic inhibitor, taxane microtubule stabilizer	100	Mitotic	0.606822	0.001
371	1-KW-C10-Vinorelbine-Mitotic inhibitor. Vinca alkaloid microtubule depolymerizer	100	Mitotic	0.730602	0
372	1-KW-C13-Ixabepilone-Mitotic inhibitor. Epothilone microtubule stabilizer.	10	Mitotic	0.640401	0
373	1-KW-C18-Paclitaxel-Mitotic inhibitor, taxane microtubule stabilizer	10	Mitotic	0.61667	0.015
374	1-KW-D10-Vinorelbine-Mitotic inhibitor. Vinca alkaloid microtubule depolymerizer	10	Mitotic	0.613052	0
375	1-KW-D13-Ixabepilone-Mitotic inhibitor. Epothilone microtubule stabilizer.	1	Mitotic	0.721973	0.002
376	1-KW-D18-Paclitaxel-Mitotic inhibitor, taxane microtubule stabilizer	1	Mitotic	0.753955	0
377	1-KW-E10-Vinorelbine-Mitotic inhibitor. Vinca alkaloid microtubule depolymerizer	1	Mitotic	0.395656	0.02
378	1-KW-E13-Ixabepilone-Mitotic inhibitor. Epothilone microtubule stabilizer.	0.1	Mitotic	0.315474	0.25
379	1-KW-E18-Paclitaxel-Mitotic inhibitor, taxane microtubule stabilizer	0.1	Mitotic	0.773694	0

380	1-KW-F13-Vinblastine-Mitotic inhibitor. Vinca alkaloid microtubule depolymerizer	1000	Mitotic	0.642516	0
381	1-KW-G13-Vinblastine-Mitotic inhibitor. Vinca alkaloid microtubule depolymerizer	100	Mitotic	0.702565	0
382	1-KW-G15-Eribulin-Mitotic inhibitor, microtubule depolymerizer.	1000	Mitotic	0.7085	0
383	1-KW-H13-Vinblastine-Mitotic inhibitor. Vinca alkaloid microtubule depolymerizer	10	Mitotic	0.73138	0.001
384	1-KW-H15-Eribulin-Mitotic inhibitor, microtubule depolymerizer.	100	Mitotic	0.682954	0
385	1-KW-I13-Vinblastine-Mitotic inhibitor. Vinca alkaloid microtubule depolymerizer	1	Mitotic	0.570188	0
386	1-KW-I15-Eribulin-Mitotic inhibitor, microtubule depolymerizer.	10	Mitotic	0.760272	0
387	1-KW-J13-Vinblastine-Mitotic inhibitor. Vinca alkaloid microtubule depolymerizer	0.1	Mitotic	0.339301	0.12
388	1-KW-J15-Eribulin-Mitotic inhibitor, microtubule depolymerizer.	1	Mitotic	0.54428	0.002
389	1-KW-K7-Vincristine-Mitotic inhibitor. Vinca alkaloid microtubule depolymerizer	0.1	Mitotic	0.61883	0
390	1-KW-K15-Eribulin-Mitotic inhibitor, microtubule depolymerizer.	0.1	Mitotic	0.699736	0
391	1-KW-L7-Vincristine-Mitotic inhibitor. Vinca alkaloid microtubule depolymerizer	1	Mitotic	0.727765	0.001
392	1-KW-L20-Vinflunine-Mitotic inhibitor. Vinca alkaloid microtubule depolymerizer	0.1	Mitotic	0.773044	0
393	1-KW-M7-Vincristine-Mitotic inhibitor. Vinca alkaloid microtubule depolymerizer	10	Mitotic	0.726925	0
394	1-KW-M20-Vinflunine-Mitotic inhibitor. Vinca alkaloid microtubule depolymerizer	1	Mitotic	0.783503	0
395	1-KW-N20-Vinflunine-Mitotic inhibitor. Vinca alkaloid microtubule depolymerizer	10	Mitotic	0.740001	0.002
396	1-KW-O7-Vincristine-Mitotic inhibitor. Vinca alkaloid microtubule depolymerizer	100	Mitotic	0.769007	0
397	1-KW-O20-Vinflunine-Mitotic inhibitor. Vinca alkaloid microtubule depolymerizer	100	Mitotic	0.65994	0
398	1-KW-P7-Vincristine-Mitotic inhibitor. Vinca alkaloid microtubule depolymerizer	1000	Mitotic	0.747339	0
399	1-KW-P20-Vinflunine-Mitotic inhibitor. Vinca alkaloid microtubule depolymerizer	1000	Mitotic	0.61133	0
400	3-KW-A7-Docetaxel-Mitotic inhibitor, taxane microtubule stabilizer	1000	Mitotic	0.724173	0
401	3-KW-B7-Docetaxel-Mitotic inhibitor, taxane microtubule stabilizer	100	Mitotic	0.677667	0
402	3-KW-C7-Docetaxel-Mitotic inhibitor, taxane microtubule stabilizer	10	Mitotic	0.634863	0
403	3-KW-D7-Docetaxel-Mitotic inhibitor, taxane microtubule stabilizer	1	Mitotic	0.235574	0.882
404	3-KW-E7-Docetaxel-Mitotic inhibitor, taxane microtubule stabilizer	0.1	Mitotic	0.426457	0.212
405	6-KW-L19-ABT-751-Mitotic inhibitor. Colchicine site binding microtubule depolymerizer.	1	Mitotic	0.48575	0.006
406	6-KW-M19-ABT-751-Mitotic inhibitor. Colchicine site binding microtubule depolymerizer.	10	Mitotic	0.499272	0.03
407	6-KW-N19-ABT-751-Mitotic inhibitor. Colchicine site binding microtubule depolymerizer.	100	Mitotic	0.499088	0.008
408	6-KW-O19-ABT-751-Mitotic inhibitor. Colchicine site binding microtubule depolymerizer.	1000	Mitotic	0.499177	0.02
409	6-KW-P19-ABT-751-Mitotic inhibitor. Colchicine site binding microtubule depolymerizer.	10000	Mitotic	0.629376	0
410	2-KW-A12-Trametinib-MEK1/2 inhibitor	250	MEK1/2	0.406142	0.508
411	2-KW-B12-Trametinib-MEK1/2 inhibitor	25	MEK1/2	0.407664	0.902
412	2-KW-D12-Trametinib-MEK1/2 inhibitor	2.5	MEK1/2	0.588321	0.065

413	2-KW-E12-Trametinib-MEK1/2 inhibitor	0.25	MEK1/2	0.525007	0.001
414	2-KW-F12-Trametinib-MEK1/2 inhibitor	2.5000000	MEK1/2	0.471228	0.055
415	2-KW-F14-Cobimetinib-MEK1/2 inhibitor	1000	MEK1/2	0.381586	0.658
416	2-KW-G14-Cobimetinib-MEK1/2 inhibitor	100	MEK1/2	0.596299	0.001
417	2-KW-H14-Cobimetinib-MEK1/2 inhibitor	10	MEK1/2	0.56491	0
418	2-KW-I14-Cobimetinib-MEK1/2 inhibitor	1	MEK1/2	0.390377	0.221
419	2-KW-K14-Cobimetinib-MEK1/2 inhibitor	0.1	MEK1/2	0.445755	0.734
420	2-KW-L20-Selumetinib-MEK1/2 inhibitor	1	MEK1/2	0.302117	0.573
421	2-KW-M20-Selumetinib-MEK1/2 inhibitor	10	MEK1/2	0.42112	0.36
422	2-KW-N20-Selumetinib-MEK1/2 inhibitor	100	MEK1/2	0.363393	0.494
423	2-KW-O20-Selumetinib-MEK1/2 inhibitor	1000	MEK1/2	0.412726	0.333
424	2-KW-P20-Selumetinib-MEK1/2 inhibitor	10000	MEK1/2	0.280278	0.782
425	4-KW-A10-Binimetinib-MEK1/2 inhibitor	1000	MEK1/2	0.482252	0.005
426	4-KW-A13-PD0325901-MEK1/2 inhibitor	1000	MEK1/2	0.507995	0.021
427	4-KW-B10-Binimetinib-MEK1/2 inhibitor	100	MEK1/2	0.42899	0.003
428	4-KW-B13-PD0325901-MEK1/2 inhibitor	100	MEK1/2	0.336081	0.996
429	4-KW-C10-Binimetinib-MEK1/2 inhibitor	10	MEK1/2	0.496717	0.043
430	4-KW-C13-PD0325901-MEK1/2 inhibitor	10	MEK1/2	0.66959	0
431	4-KW-D10-Binimetinib-MEK1/2 inhibitor	1	MEK1/2	0.418673	0.174
432	4-KW-D13-PD0325901-MEK1/2 inhibitor	1	MEK1/2	0.486367	0.037
433	4-KW-E10-Binimetinib-MEK1/2 inhibitor	0.1	MEK1/2	0.431145	0.057
434	4-KW-E13-PD0325901-MEK1/2 inhibitor	0.1	MEK1/2	0.519157	0.037
435	4-KW-L19-GDC-0623-MEK1/2 inhibitor	0.25	MEK1/2	0.212934	0.964
436	4-KW-M19-GDC-0623-MEK1/2 inhibitor	2.5	MEK1/2	0.183855	0.999
437	4-KW-N19-GDC-0623-MEK1/2 inhibitor	25	MEK1/2	0.583794	0.126
438	4-KW-O19-GDC-0623-MEK1/2 inhibitor	250	MEK1/2	0.35232	0.661
439	4-KW-P19-GDC-0623-MEK1/2 inhibitor	2500	MEK1/2	0.376087	0.467
440	1-KW-L2-Olaparib-PARP inhibitor	1	PARP	0.825349	0
441	1-KW-L6-Rucaparib-PARP inhibitor	1	PARP	0.40227	0.688
442	1-KW-M2-Olaparib-PARP inhibitor	10	PARP	0.828086	0
443	1-KW-M6-Rucaparib-PARP inhibitor	10	PARP	0.426986	0.607
444	1-KW-N2-Olaparib-PARP inhibitor	100	PARP	0.624528	0.008
445	1-KW-N6-Rucaparib-PARP inhibitor	100	PARP	0.820713	0

446	1-KW-O2-Olaparib-PARP inhibitor	1000	PARP	0.796948	0
447	1-KW-O6-Rucaparib-PARP inhibitor	1000	PARP	0.791916	0
448	1-KW-P2-Olaparib-PARP inhibitor	10000	PARP	0.714269	0.002
449	1-KW-P6-Rucaparib-PARP inhibitor	10000	PARP	0.778445	0
450	7-KW-A3-Talazoparib-PARP1/2 inhibitor	1000	PARP	0.565091	0.01
451	7-KW-B2-Veliparib-PARP inhibitor	10000	PARP	0.824414	0
452	7-KW-B3-Talazoparib-PARP1/2 inhibitor	100	PARP	0.786143	0
453	7-KW-C2-Veliparib-PARP inhibitor	1000	PARP	0.457528	0.108
454	7-KW-C3-Talazoparib-PARP1/2 inhibitor	10	PARP	0.789691	0
455	7-KW-D2-Veliparib-PARP inhibitor	100	PARP	0.574064	0.014
456	7-KW-D3-Talazoparib-PARP1/2 inhibitor	1	PARP	0.417449	0.169
457	7-KW-E2-Veliparib-PARP inhibitor	10	PARP	0.73571	0
458	7-KW-E3-Talazoparib-PARP1/2 inhibitor	0.1	PARP	0.296963	0.284
459	7-KW-F2-Veliparib-PARP inhibitor	1	PARP	0.730059	0
460	7-KW-G2-Niraparib-PARP inhibitor	10000	PARP	0.753675	0
461	7-KW-H2-Niraparib-PARP inhibitor	1000	PARP	0.371487	0.621
462	7-KW-I2-Niraparib-PARP inhibitor	100	PARP	0.818367	0
463	7-KW-J2-Niraparib-PARP inhibitor	10	PARP	0.722338	0.021
464	7-KW-K2-Niraparib-PARP inhibitor	1	PARP	0.672511	0
465	3-KW-A19-Dinaciclib-CDK inhibitor	1000	CDK	0.635293	0
466	3-KW-B19-Dinaciclib-CDK inhibitor	100	CDK	0.583455	0
467	3-KW-B23-Abemaciclib-CDK4/6 inhibitor	2500	CDK	0.525586	0
468	3-KW-C19-Dinaciclib-CDK inhibitor	10	CDK	0.501306	0.003
469	3-KW-C23-Abemaciclib-CDK4/6 inhibitor	250	CDK	0.552228	0
470	3-KW-D19-Dinaciclib-CDK inhibitor	1	CDK	0.522349	0.001
471	3-KW-D23-Abemaciclib-CDK4/6 inhibitor	25	CDK	0.590665	0
472	3-KW-E19-Dinaciclib-CDK inhibitor	0.1	CDK	0.548866	0.012
473	3-KW-E23-Abemaciclib-CDK4/6 inhibitor	2.5	CDK	0.519819	0.002
474	3-KW-F23-Abemaciclib-CDK4/6 inhibitor	0.25	CDK	0.397471	0.104
475	3-KW-K17-Palbociclib-CDK4/6 inhibitor	1	CDK	0.519832	0
476	3-KW-L19-Ribociclib-CDK4/6 inhibitor	1	CDK	0.200787	0.816
477	3-KW-M17-Palbociclib-CDK4/6 inhibitor	10	CDK	0.529069	0.001
478	3-KW-M19-Ribociclib-CDK4/6 inhibitor	10	CDK	0.509895	0

479	3-KW-N17-Palbociclib-CDK4/6 inhibitor	100	CDK	0.582955	0
480	3-KW-N19-Ribociclib-CDK4/6 inhibitor	100	CDK	0.573554	0
481	3-KW-O17-Palbociclib-CDK4/6 inhibitor	1000	CDK	0.54894	0
482	3-KW-O19-Ribociclib-CDK4/6 inhibitor	1000	CDK	0.543445	0
483	3-KW-P17-Palbociclib-CDK4/6 inhibitor	10000	CDK	0.521951	0.001
484	3-KW-P19-Ribociclib-CDK4/6 inhibitor	10000	CDK	0.55335	0
485	4-KW-A4-SNS-032-CDK inhibitor	10000	CDK	0.596675	0
486	4-KW-A8-Milciclib-CDK2 inhibitor	10000	CDK	0.517656	0.006
487	4-KW-B4-SNS-032-CDK inhibitor	1000	CDK	0.618624	0
488	4-KW-B8-Milciclib-CDK2 inhibitor	1000	CDK	0.541825	0
489	4-KW-C4-SNS-032-CDK inhibitor	100	CDK	0.550945	0
490	4-KW-C8-Milciclib-CDK2 inhibitor	100	CDK	0.466361	0.013
491	4-KW-D4-SNS-032-CDK inhibitor	10	CDK	0.368117	0.112
492	4-KW-D8-Milciclib-CDK2 inhibitor	10	CDK	0.18412	0.622
493	4-KW-E4-SNS-032-CDK inhibitor	1	CDK	0.493878	0.007
494	4-KW-E8-Milciclib-CDK2 inhibitor	1	CDK	0.12961	0.9
495	4-KW-F4-Seliciclib-CDK2/7/9 inhibitor	10000	CDK	0.527451	0.001
496	4-KW-F22-Alvocidib-CDK inhibitor	10000	CDK	0.663058	0
497	4-KW-G4-Seliciclib-CDK2/7/9 inhibitor	1000	CDK	0.458094	0.004
498	4-KW-G22-Alvocidib-CDK inhibitor	1000	CDK	0.664295	0
499	4-KW-H4-Seliciclib-CDK2/7/9 inhibitor	100	CDK	0.339699	0.052
500	4-KW-H22-Alvocidib-CDK inhibitor	100	CDK	0.53904	0
501	4-KW-I4-Seliciclib-CDK2/7/9 inhibitor	10	CDK	0.376961	0.132
502	4-KW-I22-Alvocidib-CDK inhibitor	10	CDK	0.466406	0.034
503	4-KW-J4-Seliciclib-CDK2/7/9 inhibitor	1	CDK	0.316647	0.212
504	4-KW-J22-Alvocidib-CDK inhibitor	1	CDK	0.459764	0.018
505	5-KW-A19-AZD-5438-CDK1,2,9 inhibitor	10000	CDK	0.614369	0
506	5-KW-B19-AZD-5438-CDK1,2,9 inhibitor	1000	CDK	0.607562	0
507	5-KW-C19-AZD-5438-CDK1,2,9 inhibitor	100	CDK	0.460742	0.009
508	5-KW-D19-AZD-5438-CDK1,2,9 inhibitor	10	CDK	0.430637	0.008
509	5-KW-E19-AZD-5438-CDK1,2,9 inhibitor	1	CDK	0.51088	0.001
510	5-KW-K17-AT7519-CDK1, 2, 4, 6 and 9 inhibitor	1	CDK	0.379738	0.15
511	5-KW-M17-AT7519-CDK1, 2, 4, 6 and 9 inhibitor	10	CDK	0.379226	0.05

512	5-KW-N17-AT7519-CDK1, 2, 4, 6 and 9 inhibitor	100	CDK	0.405001	0.065
513	5-KW-O17-AT7519-CDK1, 2, 4, 6 and 9 inhibitor	1000	CDK	0.473941	0.023
514	5-KW-P17-AT7519-CDK1, 2, 4, 6 and 9 inhibitor	10000	CDK	0.600174	0
515	6-KW-A17-Senexin B-CDK8/19 inhibitor	1000	CDK	0.409759	0.091
516	6-KW-B17-Senexin B-CDK8/19 inhibitor	100	CDK	0.34522	0.538
517	6-KW-C17-Senexin B-CDK8/19 inhibitor	10	CDK	0.47136	0.004
518	6-KW-D17-Senexin B-CDK8/19 inhibitor	1	CDK	0.44115	0.008
519	6-KW-E17-Senexin B-CDK8/19 inhibitor	0.1	CDK	0.171496	0.978
520	6-KW-L15-THZ2-CDK7 inhibitor	1	CDK	0.073508	0.998
521	6-KW-M15-THZ2-CDK7 inhibitor	10	CDK	0.326354	0.157
522	6-KW-N15-THZ2-CDK7 inhibitor	100	CDK	0.435356	0.023
523	6-KW-O15-THZ2-CDK7 inhibitor	1000	CDK	0.554388	0
524	6-KW-P15-THZ2-CDK7 inhibitor	10000	CDK	0.488726	0
525	7-KW-A21-dBET1-BET-targeting PROTAC	10000	BET	0.655565	0
526	7-KW-A22-PFI-1-BET family inhibitor	30000	BET	0.800748	0
527	7-KW-B21-dBET1-BET-targeting PROTAC	1000	BET	0.412766	0.111
528	7-KW-B22-PFI-1-BET family inhibitor	3000	BET	0.67922	0
529	7-KW-C21-dBET1-BET-targeting PROTAC	100	BET	0.736195	0.003
530	7-KW-C22-PFI-1-BET family inhibitor	300	BET	0.692177	0
531	7-KW-D21-dBET1-BET-targeting PROTAC	10	BET	0.684731	0.025
532	7-KW-D22-PFI-1-BET family inhibitor	30	BET	0.725569	0.008
533	7-KW-E21-dBET1-BET-targeting PROTAC	1	BET	0.634827	0
534	7-KW-E22-PFI-1-BET family inhibitor	3	BET	0.601187	0.001
535	7-KW-G10-Birabresib-BET family inhibitor	10000	BET	0.785724	0
536	7-KW-G15-I-BET151-BET family inhibitor	10000	BET	0.808989	0
537	7-KW-H10-Birabresib-BET family inhibitor	1000	BET	0.742112	0
538	7-KW-H15-I-BET151-BET family inhibitor	1000	BET	0.782974	0
539	7-KW-I10-Birabresib-BET family inhibitor	100	BET	0.758992	0
540	7-KW-I15-I-BET151-BET family inhibitor	100	BET	0.770851	0
541	7-KW-J10-Birabresib-BET family inhibitor	10	BET	0.740251	0
542	7-KW-J15-I-BET151-BET family inhibitor	10	BET	0.677497	0.026
543	7-KW-K10-Birabresib-BET family inhibitor	1	BET	0.487245	0.012
544	7-KW-K13-Mivebresib-BET family inhibitor	1	BET	0.304374	0.312

545	7-KW-K15-I-BET151-BET family inhibitor	1	BET	0.719673	0.013
546	7-KW-L12-Molibresib-BET family inhibitor	1	BET	0.291053	0.125
547	7-KW-L13-Mivebresib-BET family inhibitor	10	BET	0.6703	0.002
548	7-KW-L20-JQ1-BET family inhibitor	1	BET	0.591908	0
549	7-KW-L23-ARV-825-BET-targeting PROTAC	0.03	BET	0.472104	0.025
550	7-KW-M12-Molibresib-BET family inhibitor	10	BET	0.378848	0.046
551	7-KW-M13-Mivebresib-BET family inhibitor	100	BET	0.800327	0
552	7-KW-M20-JQ1-BET family inhibitor	10	BET	0.636099	0
553	7-KW-M23-ARV-825-BET-targeting PROTAC	0.3	BET	0.630113	0.016
554	7-KW-N12-Molibresib-BET family inhibitor	100	BET	0.585135	0.003
555	7-KW-N13-Mivebresib-BET family inhibitor	1000	BET	0.797318	0
556	7-KW-N20-JQ1-BET family inhibitor	100	BET	0.753147	0
557	7-KW-N23-ARV-825-BET-targeting PROTAC	3	BET	0.593037	0.192
558	7-KW-O12-Molibresib-BET family inhibitor	1000	BET	0.721437	0
559	7-KW-O20-JQ1-BET family inhibitor	1000	BET	0.712507	0
560	7-KW-O23-ARV-825-BET-targeting PROTAC	30	BET	0.720814	0
561	7-KW-P12-Molibresib-BET family inhibitor	10000	BET	0.798074	0
562	7-KW-P13-Mivebresib-BET family inhibitor	10000	BET	0.793592	0
563	7-KW-P20-JQ1-BET family inhibitor	10000	BET	0.802077	0
564	7-KW-P23-ARV-825-BET-targeting PROTAC	300	BET	0.815511	0
565	8-KW-K22-CPI-0610-BET family inhibitor	1	BET	0.139864	0.998
566	8-KW-L22-CPI-0610-BET family inhibitor	10	BET	0.251208	0.464
567	8-KW-M22-CPI-0610-BET family inhibitor	100	BET	0.613726	0
568	8-KW-N22-CPI-0610-BET family inhibitor	1000	BET	0.788992	0
569	8-KW-O22-CPI-0610-BET family inhibitor	10000	BET	0.766346	0
570	1-KW-A3-Vorinostat-HDAC inhibitor	10000	HDAC	0.551543	0
571	1-KW-B3-Vorinostat-HDAC inhibitor	1000	HDAC	0.3169	0.936
572	1-KW-C3-Vorinostat-HDAC inhibitor	100	HDAC	0.802773	0
573	1-KW-D3-Vorinostat-HDAC inhibitor	10	HDAC	0.173269	0.931
574	1-KW-E3-Vorinostat-HDAC inhibitor	1	HDAC	0.06495	0.99
575	1-KW-L12-Romidepsin-HDAC inhibitor	0.1	HDAC	0.812804	0
576	1-KW-M12-Romidepsin-HDAC inhibitor	1	HDAC	0.153317	0.582
577	1-KW-N12-Romidepsin-HDAC inhibitor	10	HDAC	0.526287	0

**578**	1-KW-O12-Romidepsin-HDAC inhibitor	100	HDAC	0.53565	0
**579**	1-KW-P12-Romidepsin-HDAC inhibitor	1000	HDAC	0.539177	0
**580**	3-KW-A4-Panobinostat-HDAC inhibitor	1000	HDAC	0.513906	0
**581**	3-KW-B4-Panobinostat-HDAC inhibitor	100	HDAC	0.4874	0
**582**	3-KW-C4-Panobinostat-HDAC inhibitor	10	HDAC	0.328209	0.227
**583**	3-KW-D4-Panobinostat-HDAC inhibitor	1	HDAC	0.070968	1
**584**	3-KW-E4-Panobinostat-HDAC inhibitor	0.1	HDAC	0.290767	0.667
**585**	3-KW-F7-Quisinostat-HDAC inhibitor	1000	HDAC	0.543566	0
**586**	3-KW-G7-Quisinostat-HDAC inhibitor	100	HDAC	0.417158	0.027
**587**	3-KW-G12-Valproic acid-HDAC inhibitor	1000000	HDAC	0.144877	0.793
**588**	3-KW-H7-Quisinostat-HDAC inhibitor	10	HDAC	0.396151	0.026
**589**	3-KW-H12-Valproic acid-HDAC inhibitor	100000	HDAC	0.450941	0
**590**	3-KW-I7-Quisinostat-HDAC inhibitor	1	HDAC	0.114072	0.952
**591**	3-KW-I12-Valproic acid-HDAC inhibitor	10000	HDAC	0.189074	0.575
**592**	3-KW-J7-Quisinostat-HDAC inhibitor	0.1	HDAC	0.390301	0.626
**593**	3-KW-J12-Valproic acid-HDAC inhibitor	1000	HDAC	0.307432	0.463
**594**	3-KW-K3-Belinostat-HDAC inhibitor	1	HDAC	0.215797	0.982
**595**	3-KW-K12-Valproic acid-HDAC inhibitor	100	HDAC	0.395475	0.153
**596**	3-KW-L3-Belinostat-HDAC inhibitor	10	HDAC	0.216787	0.96
**597**	3-KW-M3-Belinostat-HDAC inhibitor	100	HDAC	0.396776	0.056
**598**	3-KW-N3-Belinostat-HDAC inhibitor	1000	HDAC	0.448724	0.009
**599**	3-KW-O3-Belinostat-HDAC inhibitor	10000	HDAC	0.54013	0
**600**	7-KW-A5-Mocetinostat-HDAC inhibitor (HDAC1 & 2-selective)	10000	HDAC	0.504054	0
**601**	7-KW-A7-CUDC-907-HDAC1/2/3/10, PI3Kalpha inhibitor	10000	HDAC	0.528306	0
**602**	7-KW-A9-Givinostat-HDAC inhibitor	1000	HDAC	0.41124	0.024
**603**	7-KW-A12-Rocilinostat-HDAC-6 selective inhibitor	10000	HDAC	0.670818	0
**604**	7-KW-B5-Mocetinostat-HDAC inhibitor (HDAC1 & 2-selective)	1000	HDAC	0.390068	0.012
**605**	7-KW-B7-CUDC-907-HDAC1/2/3/10, PI3Kalpha inhibitor	1000	HDAC	0.561844	0
**606**	7-KW-B12-Rocilinostat-HDAC-6 selective inhibitor	1000	HDAC	0.448739	0.004
**607**	7-KW-C5-Mocetinostat-HDAC inhibitor (HDAC1 & 2-selective)	100	HDAC	0.417243	0.015
**608**	7-KW-C7-CUDC-907-HDAC1/2/3/10, PI3Kalpha inhibitor	100	HDAC	0.528865	0
**609**	7-KW-C9-Givinostat-HDAC inhibitor	100	HDAC	0.764979	0
**610**	7-KW-D7-CUDC-907-HDAC1/2/3/10, PI3Kalpha inhibitor	10	HDAC	0.814152	0

611	7-KW-D9-Givinostat-HDAC inhibitor	10	HDAC	0.357166	0.003
612	7-KW-D12-Rocilinostat-HDAC-6 selective inhibitor	100	HDAC	0.815572	0
613	7-KW-E5-Mocetinostat-HDAC inhibitor (HDAC1 & 2-selective)	10	HDAC	0.73351	0
614	7-KW-E7-CUDC-907-HDAC1/2/3/10, PI3Kalpha inhibitor	1	HDAC	0.622766	0
615	7-KW-E9-Givinostat-HDAC inhibitor	1	HDAC	0.093361	0.676
616	7-KW-E12-Rocilinostat-HDAC-6 selective inhibitor	10	HDAC	0.810986	0
617	7-KW-F5-Mocetinostat-HDAC inhibitor (HDAC1 & 2-selective)	1	HDAC	0.811348	0
618	7-KW-F7-Resminostat-HDAC1, 3, 6 inhibitor	10000	HDAC	0.551379	0
619	7-KW-F9-Givinostat-HDAC inhibitor	0.1	HDAC	0.068606	0.875
620	7-KW-F12-Rocilinostat-HDAC-6 selective inhibitor	1	HDAC	0.271959	0.074
621	7-KW-F19-PCI-34051-HDAC8 inhibitor	10000	HDAC	0.699711	0
622	7-KW-G7-Resminostat-HDAC1, 3, 6 inhibitor	1000	HDAC	0.77633	0
623	7-KW-G19-PCI-34051-HDAC8 inhibitor	1000	HDAC	0.396849	0.04
624	7-KW-H7-Resminostat-HDAC1, 3, 6 inhibitor	100	HDAC	0.512094	0
625	7-KW-I7-Resminostat-HDAC1, 3, 6 inhibitor	10	HDAC	0.462381	0
626	7-KW-I19-PCI-34051-HDAC8 inhibitor	100	HDAC	0.442476	0.003
627	7-KW-J7-Resminostat-HDAC1, 3, 6 inhibitor	1	HDAC	0.256399	0.246
628	7-KW-J19-PCI-34051-HDAC8 inhibitor	10	HDAC	0.800395	0
629	7-KW-K4-Entinostat-HDAC inhibitor	1	HDAC	0.803113	0
630	7-KW-K11-AR-42-HDAC inhibitor	1	HDAC	0.439439	0.001
631	7-KW-K18-Tubacin-HDAC6 inhibitor	1	HDAC	0.383059	0.07
632	7-KW-K19-PCI-34051-HDAC8 inhibitor	1	HDAC	0.790339	0
633	7-KW-L2-Tacedinaline-HDAC inhibitor	0.1	HDAC	0.802047	0
634	7-KW-L4-Entinostat-HDAC inhibitor	10	HDAC	0.74592	0
635	7-KW-L5-Pracinostat-HDAC inhibitor	1	HDAC	0.631362	0
636	7-KW-L8-Abexinostat-HDAC1-selective inhibitor	1	HDAC	0.809434	0
637	7-KW-L10-Tucidinostat-HDAC1/2/3/10 inhibitor	1	HDAC	0.813504	0
638	7-KW-L11-AR-42-HDAC inhibitor	10	HDAC	0.8038	0
639	7-KW-L14-Tubastatin A-HDAC6 inhibitor	1	HDAC	0.810024	0
640	7-KW-L16-RGFP966-HDAC3 inhibitor	1	HDAC	0.389938	0.017
641	7-KW-L18-Tubacin-HDAC6 inhibitor	10	HDAC	0.814507	0
642	7-KW-M2-Tacedinaline-HDAC inhibitor	1	HDAC	0.613215	0
643	7-KW-M5-Pracinostat-HDAC inhibitor	10	HDAC	0.134235	0.972

644	7-KW-M8-Abexinostat-HDAC1-selective inhibitor	10	HDAC	0.815963	0
645	7-KW-M10-Tucidinostat-HDAC1/2/3/10 inhibitor	10	HDAC	0.814637	0
646	7-KW-M11-AR-42-HDAC inhibitor	100	HDAC	0.767992	0
647	7-KW-M14-Tubastatin A-HDAC6 inhibitor	10	HDAC	0.791003	0
648	7-KW-M16-RGFP966-HDAC3 inhibitor	10	HDAC	0.354186	0.175
649	7-KW-M18-Tubacin-HDAC6 inhibitor	100	HDAC	0.449743	0.005
650	7-KW-N2-Tacedinaline-HDAC inhibitor	10	HDAC	0.737514	0
651	7-KW-N4-Entinostat-HDAC inhibitor	100	HDAC	0.698642	0
652	7-KW-N5-Pracinostat-HDAC inhibitor	100	HDAC	0.747187	0
653	7-KW-N8-Abexinostat-HDAC1-selective inhibitor	100	HDAC	0.768701	0
654	7-KW-N10-Tucidinostat-HDAC1/2/3/10 inhibitor	100	HDAC	0.79357	0
655	7-KW-N14-Tubastatin A-HDAC6 inhibitor	100	HDAC	0.790524	0
656	7-KW-N16-RGFP966-HDAC3 inhibitor	100	HDAC	0.469233	0.006
657	7-KW-N18-Tubacin-HDAC6 inhibitor	1000	HDAC	0.794864	0
658	7-KW-O2-Tacedinaline-HDAC inhibitor	100	HDAC	0.169398	0.948
659	7-KW-O4-Entinostat-HDAC inhibitor	1000	HDAC	0.54649	0
660	7-KW-O5-Pracinostat-HDAC inhibitor	1000	HDAC	0.431599	0.008
661	7-KW-O8-Abexinostat-HDAC1-selective inhibitor	1000	HDAC	0.463264	0
662	7-KW-O10-Tucidinostat-HDAC1/2/3/10 inhibitor	1000	HDAC	0.759876	0
663	7-KW-O11-AR-42-HDAC inhibitor	1000	HDAC	0.767597	0
664	7-KW-O14-Tubastatin A-HDAC6 inhibitor	1000	HDAC	0.714703	0
665	7-KW-O16-RGFP966-HDAC3 inhibitor	1000	HDAC	0.768663	0
666	7-KW-P2-Tacedinaline-HDAC inhibitor	1000	HDAC	0.395118	0.041
667	7-KW-P4-Entinostat-HDAC inhibitor	10000	HDAC	0.73106	0
668	7-KW-P5-Pracinostat-HDAC inhibitor	10000	HDAC	0.563978	0
669	7-KW-P8-Abexinostat-HDAC1-selective inhibitor	10000	HDAC	0.549474	0
670	7-KW-P10-Tucidinostat-HDAC1/2/3/10 inhibitor	10000	HDAC	0.695869	0
671	7-KW-P11-AR-42-HDAC inhibitor	10000	HDAC	0.566729	0
672	7-KW-P14-Tubastatin A-HDAC6 inhibitor	10000	HDAC	0.771195	0
673	7-KW-P16-RGFP966-HDAC3 inhibitor	10000	HDAC	0.728285	0
674	7-KW-P18-Tubacin-HDAC6 inhibitor	10000	HDAC	0.700093	0
0	2-MHB-A16-Gefitinib-EGFR inhibitor	10000	EGFR	0.64314	0
1	2-MHB-A19-Erlotinib-EGFR inhibitor	10000	EGFR	0.616671	0

**2**	2-MHB-B19-Erlotinib-EGFR inhibitor	1000	EGFR	0.675736	0
**3**	2-MHB-C16-Gefitinib-EGFR inhibitor	1000	EGFR	0.68597	0
**4**	2-MHB-C19-Erlotinib-EGFR inhibitor	100	EGFR	0.648226	0
**5**	2-MHB-D16-Gefitinib-EGFR inhibitor	100	EGFR	0.691619	0
**6**	2-MHB-D19-Erlotinib-EGFR inhibitor	10	EGFR	0.131571	0.91
**7**	2-MHB-E16-Gefitinib-EGFR inhibitor	10	EGFR	0.641952	0
**8**	2-MHB-E19-Erlotinib-EGFR inhibitor	1	EGFR	0.040156	1
**9**	2-MHB-F16-Gefitinib-EGFR inhibitor	1	EGFR	0.542343	0
**10**	2-MHB-K11-Afatinib-EGFR inhibitor	0.1	EGFR	0.451064	0
**11**	2-MHB-L11-Afatinib-EGFR inhibitor	1	EGFR	0.514177	0
**12**	2-MHB-L16-Osimertinib-EGFR(L858R/T790M) inhibitor	0.25	EGFR	0.284136	0.04
**13**	2-MHB-L19-Lapatinib-HER2, EGFR inhibitor	0.1	EGFR	0.525192	0
**14**	2-MHB-M11-Afatinib-EGFR inhibitor	10	EGFR	0.62947	0
**15**	2-MHB-M16-Osimertinib-EGFR(L858R/T790M) inhibitor	2.5	EGFR	0.596474	0
**16**	2-MHB-M19-Lapatinib-HER2, EGFR inhibitor	1	EGFR	0.640918	0
**17**	2-MHB-N16-Osimertinib-EGFR(L858R/T790M) inhibitor	25	EGFR	0.661826	0
**18**	2-MHB-N19-Lapatinib-HER2, EGFR inhibitor	10	EGFR	0.571019	0
**19**	2-MHB-O11-Afatinib-EGFR inhibitor	100	EGFR	0.672029	0
**20**	2-MHB-O16-Osimertinib-EGFR(L858R/T790M) inhibitor	250	EGFR	0.64375	0
**21**	2-MHB-O19-Lapatinib-HER2, EGFR inhibitor	100	EGFR	0.705694	0
**22**	2-MHB-P11-Afatinib-EGFR inhibitor	1000	EGFR	0.610666	0
**23**	2-MHB-P16-Osimertinib-EGFR(L858R/T790M) inhibitor	2500	EGFR	0.61752	0
**24**	2-MHB-P19-Lapatinib-HER2, EGFR inhibitor	1000	EGFR	0.616921	0
**25**	3-MHB-F21-Rociletinib-EGFR(L858R/T790M) inhibitor	10000	EGFR	0.549167	0
**26**	3-MHB-G20-Neratinib-EGFR inhibitor	1000	EGFR	0.649662	0
**27**	3-MHB-G21-Rociletinib-EGFR(L858R/T790M) inhibitor	1000	EGFR	0.617733	0
**28**	3-MHB-H20-Neratinib-EGFR inhibitor	100	EGFR	0.695903	0
**29**	3-MHB-H21-Rociletinib-EGFR(L858R/T790M) inhibitor	100	EGFR	0.122953	0.966
**30**	3-MHB-I20-Neratinib-EGFR inhibitor	10	EGFR	0.662489	0
**31**	3-MHB-I21-Rociletinib-EGFR(L858R/T790M) inhibitor	10	EGFR	0.080961	0.993
**32**	3-MHB-J20-Neratinib-EGFR inhibitor	1	EGFR	0.183363	0.968
**33**	3-MHB-J21-Rociletinib-EGFR(L858R/T790M) inhibitor	1	EGFR	0.338081	0.413
**34**	3-MHB-K4-Canertinib-pan-HER inhibitor	1	EGFR	0.291222	0.146

35	3-MHB-K18-Dacomitinib-pan-HER inhibitor	0.1	EGFR	0.380285	0.033
36	3-MHB-K20-Neratinib-EGFR inhibitor	0.1	EGFR	0.446751	0.002
37	3-MHB-L4-Canertinib-pan-HER inhibitor	10	EGFR	0.660561	0
38	3-MHB-L18-Dacomitinib-pan-HER inhibitor	1	EGFR	0.667279	0
39	3-MHB-M18-Dacomitinib-pan-HER inhibitor	10	EGFR	0.692206	0
40	3-MHB-N4-Canertinib-pan-HER inhibitor	100	EGFR	0.689039	0
41	3-MHB-N18-Dacomitinib-pan-HER inhibitor	100	EGFR	0.702925	0
42	3-MHB-O4-Canertinib-pan-HER inhibitor	1000	EGFR	0.629504	0
43	3-MHB-P4-Canertinib-pan-HER inhibitor	10000	EGFR	0.453676	0.048
44	3-MHB-P18-Dacomitinib-pan-HER inhibitor	1000	EGFR	0.566855	0
45	4-MHB-F13-Sapitinib-Pan-HER inhibitor	1000	EGFR	0.656287	0
46	4-MHB-G13-Sapitinib-Pan-HER inhibitor	100	EGFR	0.688002	0
47	4-MHB-G16-Varlitinib-EGFR HER2 inhibitor	10000	EGFR	0.665233	0
48	4-MHB-H13-Sapitinib-Pan-HER inhibitor	10	EGFR	0.654633	0
49	4-MHB-H16-Varlitinib-EGFR HER2 inhibitor	1000	EGFR	0.665747	0
50	4-MHB-I13-Sapitinib-Pan-HER inhibitor	1	EGFR	0.654212	0
51	4-MHB-I16-Varlitinib-EGFR HER2 inhibitor	100	EGFR	0.614413	0
52	4-MHB-J13-Sapitinib-Pan-HER inhibitor	0.1	EGFR	0.497819	0
53	4-MHB-J16-Varlitinib-EGFR HER2 inhibitor	10	EGFR	0.0686	0.998
54	4-MHB-K7-Icotinib-EGFR inhibitor	1	EGFR	0.490432	0.533
55	4-MHB-K13-Tesevatinib-EGFR, ERBB2, VEGFR, EPHB4	0.1	EGFR	0.378889	0.032
56	4-MHB-K16-Varlitinib-EGFR HER2 inhibitor	1	EGFR	0.041113	0.999
57	4-MHB-L7-Icotinib-EGFR inhibitor	10	EGFR	0.433607	0.002
58	4-MHB-L13-Tesevatinib-EGFR, ERBB2, VEGFR, EPHB4	1	EGFR	0.566891	0
59	4-MHB-M7-Icotinib-EGFR inhibitor	100	EGFR	0.626231	0
60	4-MHB-M13-Tesevatinib-EGFR, ERBB2, VEGFR, EPHB4	10	EGFR	0.67134	0
61	4-MHB-N13-Tesevatinib-EGFR, ERBB2, VEGFR, EPHB4	100	EGFR	0.676043	0
62	4-MHB-O7-Icotinib-EGFR inhibitor	1000	EGFR	0.699354	0
63	4-MHB-P7-Icotinib-EGFR inhibitor	10000	EGFR	0.682261	0
64	4-MHB-P13-Tesevatinib-EGFR, ERBB2, VEGFR, EPHB4	1000	EGFR	0.674883	0
65	5-MHB-F4-Poziotinib-pan-HER inhibitor	1000	EGFR	0.588595	0
66	5-MHB-F7-AZD3759-EGFR inhibitor, BBB penetrable	1000	EGFR	0.656869	0
67	5-MHB-G4-Poziotinib-pan-HER inhibitor	100	EGFR	0.661827	0

68	5-MHB-G7-AZD3759-EGFR inhibitor, BBB penetrable	100	EGFR	0.625923	0
69	5-MHB-H4-Poziotinib-pan-HER inhibitor	10	EGFR	0.636877	0
70	5-MHB-H7-AZD3759-EGFR inhibitor, BBB penetrable	10	EGFR	0.496651	0
71	5-MHB-I4-Poziotinib-pan-HER inhibitor	1	EGFR	0.639197	0
72	5-MHB-I7-AZD3759-EGFR inhibitor, BBB penetrable	1	EGFR	0.28005	0.21
73	5-MHB-J4-Poziotinib-pan-HER inhibitor	0.1	EGFR	0.459848	0
74	5-MHB-J7-AZD3759-EGFR inhibitor, BBB penetrable	0.1	EGFR	0.067619	0.985
75	5-MHB-K7-Olmutinib-EGFR(L858R/T790M) inhibitor	0.1	EGFR	0.47891	0.001
76	5-MHB-L7-Olmutinib-EGFR(L858R/T790M) inhibitor	1	EGFR	0.039756	0.995
77	5-MHB-M7-Olmutinib-EGFR(L858R/T790M) inhibitor	10	EGFR	0.276969	0.641
78	5-MHB-O7-Olmutinib-EGFR(L858R/T790M) inhibitor	100	EGFR	0.357384	0.086
79	5-MHB-P7-Olmutinib-EGFR(L858R/T790M) inhibitor	1000	EGFR	0.562423	0
80	2-MHB-A15-Lenvatinib-VEGFR inhibitor	2500	VEGFR	0.180022	0.942
81	2-MHB-A17-Nintedanib-VEGFR, PDGFR, FGFR inhibitor	10000	VEGFR	0.216009	0.788
82	2-MHB-A20-Tivozanib-VEGFR1, 2, 3, c-Kit, PDGFRB inhibitor	10000	VEGFR	0.170134	0.994
83	2-MHB-B15-Lenvatinib-VEGFR inhibitor	250	VEGFR	0.252231	0.653
84	2-MHB-B17-Nintedanib-VEGFR, PDGFR, FGFR inhibitor	1000	VEGFR	0.279604	0.679
85	2-MHB-B20-Tivozanib-VEGFR1, 2, 3, c-Kit, PDGFRB inhibitor	1000	VEGFR	0.378669	0
86	2-MHB-C15-Lenvatinib-VEGFR inhibitor	25	VEGFR	0.375884	0
87	2-MHB-C17-Nintedanib-VEGFR, PDGFR, FGFR inhibitor	100	VEGFR	0.530984	0
88	2-MHB-D15-Lenvatinib-VEGFR inhibitor	2.5	VEGFR	0.485046	0
89	2-MHB-D17-Nintedanib-VEGFR, PDGFR, FGFR inhibitor	10	VEGFR	0.426036	0.006
90	2-MHB-D20-Tivozanib-VEGFR1, 2, 3, c-Kit, PDGFRB inhibitor	100	VEGFR	0.329994	0
91	2-MHB-E17-Nintedanib-VEGFR, PDGFR, FGFR inhibitor	1	VEGFR	0.454884	0
92	2-MHB-E20-Tivozanib-VEGFR1, 2, 3, c-Kit, PDGFRB inhibitor	10	VEGFR	0.278401	0
93	2-MHB-F13-Axitinib-VEGFR, PDGFR, KIT inhibitor	10000	VEGFR	0.380876	0.218
94	2-MHB-F15-Lenvatinib-VEGFR inhibitor	0.25	VEGFR	0.300559	0
95	2-MHB-F19-Regorafenib-B-Raf, c-Kit, VEGFR2 inhibitor	10000	VEGFR	0.121793	0.999
96	2-MHB-F20-Tivozanib-VEGFR1, 2, 3, c-Kit, PDGFRB inhibitor	1	VEGFR	0.400257	0
97	2-MHB-F21-Vatalanib-VEGFR-1 & -2 inhibitor	10000	VEGFR	0.439204	0
98	2-MHB-G10-Apatinib-VEGFR inhibitor	10000	VEGFR	0.16414	0.272
99	2-MHB-G13-Axitinib-VEGFR, PDGFR, KIT inhibitor	1000	VEGFR	0.33765	0.271
100	2-MHB-G19-Regorafenib-B-Raf, c-Kit, VEGFR2 inhibitor	1000	VEGFR	0.283123	0.511

101	2-MHB-G21-Vatalanib-VEGFR-1 & -2 inhibitor	1000	VEGFR	0.466825	0
102	2-MHB-H10-Apatinib-VEGFR inhibitor	1000	VEGFR	0.344017	0.074
103	2-MHB-H13-Axitinib-VEGFR, PDGFR, KIT inhibitor	100	VEGFR	0.442785	0
104	2-MHB-H21-Vatalanib-VEGFR-1 & -2 inhibitor	100	VEGFR	0.490193	0
105	2-MHB-I10-Apatinib-VEGFR inhibitor	100	VEGFR	0.514487	0.002
106	2-MHB-I13-Axitinib-VEGFR, PDGFR, KIT inhibitor	10	VEGFR	0.295986	0.263
107	2-MHB-I19-Regorafenib-B-Raf, c-Kit, VEGFR2 inhibitor	100	VEGFR	0.115131	0.999
108	2-MHB-I21-Vatalanib-VEGFR-1 & -2 inhibitor	10	VEGFR	0.573932	0
109	2-MHB-J10-Apatinib-VEGFR inhibitor	10	VEGFR	0.430104	0
110	2-MHB-J13-Axitinib-VEGFR, PDGFR, KIT inhibitor	1	VEGFR	0.361269	0.045
111	2-MHB-J19-Regorafenib-B-Raf, c-Kit, VEGFR2 inhibitor	10	VEGFR	0.444713	0
112	2-MHB-J21-Vatalanib-VEGFR-1 & -2 inhibitor	1	VEGFR	0.329208	0
113	2-MHB-K10-Apatinib-VEGFR inhibitor	1	VEGFR	0.331529	0
114	2-MHB-K13-Vandetanib-VEGFR,EGFR, RET inhibitor	0.1	VEGFR	0.335774	0.006
115	2-MHB-K17-Pazopanib-VEGFR inhibitor	1	VEGFR	0.449584	0
116	2-MHB-K19-Regorafenib-B-Raf, c-Kit, VEGFR2 inhibitor	1	VEGFR	0.356913	0.019
117	2-MHB-L12-Sorafenib-B-Raf, FGFR-1, VEGFR-2 & -3, PDGFR-beta, KIT, and FLT3 inhib	0.1	VEGFR	0.438318	0
118	2-MHB-L13-Vandetanib-VEGFR,EGFR, RET inhibitor	1	VEGFR	0.244055	0.644
119	2-MHB-L21-Cediranib-KDR/Flt/VEGFR inhibitor	0.1	VEGFR	0.358951	0.052
120	2-MHB-M12-Sorafenib-B-Raf, FGFR-1, VEGFR-2 & -3, PDGFR-beta, KIT, and FLT3 inhib	1	VEGFR	0.25011	0.707
121	2-MHB-M13-Vandetanib-VEGFR,EGFR, RET inhibitor	10	VEGFR	0.439083	0
122	2-MHB-M17-Pazopanib-VEGFR inhibitor	10	VEGFR	0.547918	0
123	2-MHB-M21-Cediranib-KDR/Flt/VEGFR inhibitor	1	VEGFR	0.419237	0
124	2-MHB-N12-Sorafenib-B-Raf, FGFR-1, VEGFR-2 & -3, PDGFR-beta, KIT, and FLT3 inhib	10	VEGFR	0.485974	0
125	2-MHB-N13-Vandetanib-VEGFR,EGFR, RET inhibitor	100	VEGFR	0.064389	1
126	2-MHB-N17-Pazopanib-VEGFR inhibitor	100	VEGFR	0.321179	0.014
127	2-MHB-N21-Cediranib-KDR/Flt/VEGFR inhibitor	10	VEGFR	0.146979	0.99
128	2-MHB-O12-Sorafenib-B-Raf, FGFR-1, VEGFR-2 & -3, PDGFR-beta, KIT, and FLT3 inhib	100	VEGFR	0.207021	0.412
129	2-MHB-O17-Pazopanib-VEGFR inhibitor	1000	VEGFR	0.079083	0.919
130	2-MHB-O21-Cediranib-KDR/Flt/VEGFR inhibitor	100	VEGFR	0.588645	0
131	2-MHB-P12-Sorafenib-B-Raf, FGFR-1, VEGFR-2 & -3, PDGFR-beta, KIT, and FLT3 inhib	1000	VEGFR	0.090434	0.966
132	2-MHB-P13-Vandetanib-VEGFR,EGFR, RET inhibitor	1000	VEGFR	0.120804	0.998
133	2-MHB-P17-Pazopanib-VEGFR inhibitor	10000	VEGFR	0.11337	0.959

134	2-MHB-P21-Cediranib-KDR/Flt/VEGFR inhibitor	1000	VEGFR	0.152722	0.951
135	3-MHB-A3-Cabozantinib-VEGFR2, Met, FLT3, Tie2, Kit and Ret inhibitor	1000	VEGFR	0.523751	0
136	3-MHB-A6-Foretinib-MET, VEGFR2 inhibitor	1000	VEGFR	0.219866	0.953
137	3-MHB-A18-Linifanib-VEGFR, PDGFR, CSF-1R, FLT3 inhibitor	1000	VEGFR	0.533694	0
138	3-MHB-B3-Cabozantinib-VEGFR2, Met, FLT3, Tie2, Kit and Ret inhibitor	100	VEGFR	0.45349	0.001
139	3-MHB-B6-Foretinib-MET, VEGFR2 inhibitor	100	VEGFR	0.1893	0.873
140	3-MHB-B18-Linifanib-VEGFR, PDGFR, CSF-1R, FLT3 inhibitor	100	VEGFR	0.195958	0.959
141	3-MHB-C3-Cabozantinib-VEGFR2, Met, FLT3, Tie2, Kit and Ret inhibitor	10	VEGFR	0.411303	0.016
142	3-MHB-C6-Foretinib-MET, VEGFR2 inhibitor	10	VEGFR	0.512343	0
143	3-MHB-C18-Linifanib-VEGFR, PDGFR, CSF-1R, FLT3 inhibitor	10	VEGFR	0.233405	0.792
144	3-MHB-D3-Cabozantinib-VEGFR2, Met, FLT3, Tie2, Kit and Ret inhibitor	1	VEGFR	0.356406	0.011
145	3-MHB-D6-Foretinib-MET, VEGFR2 inhibitor	1	VEGFR	0.400531	0.014
146	3-MHB-D18-Linifanib-VEGFR, PDGFR, CSF-1R, FLT3 inhibitor	1	VEGFR	0.352312	0.009
147	3-MHB-E3-Cabozantinib-VEGFR2, Met, FLT3, Tie2, Kit and Ret inhibitor	0.1	VEGFR	0.440857	0
148	3-MHB-E6-Foretinib-MET, VEGFR2 inhibitor	0.1	VEGFR	0.349758	0.003
149	3-MHB-E18-Linifanib-VEGFR, PDGFR, CSF-1R, FLT3 inhibitor	0.1	VEGFR	0.251748	0.668
150	3-MHB-F18-Brivanib-VEGFR inhibitor	1000	VEGFR	0.548425	0
151	3-MHB-G18-Brivanib-VEGFR inhibitor	100	VEGFR	0.481295	0
152	3-MHB-H18-Brivanib-VEGFR inhibitor	10	VEGFR	0.237922	0.01
153	3-MHB-I18-Brivanib-VEGFR inhibitor	1	VEGFR	0.18613	0.079
154	3-MHB-J18-Brivanib-VEGFR inhibitor	0.1	VEGFR	0.281417	0.095
155	4-MHB-A12-ENMD-2076-pan-Aurora, VEGFR inhibitor	10000	VEGFR	0.074832	1
156	4-MHB-A15-Golvatinib-MET, VEGFR2 inhibitor	2500	VEGFR	0.089256	0.998
157	4-MHB-A20-Motesanib-VEGFR, PDGFR, Ret, Kit inhibitor	10000	VEGFR	0.358545	0.201
158	4-MHB-B12-ENMD-2076-pan-Aurora, VEGFR inhibitor	1000	VEGFR	0.189671	0.982
159	4-MHB-B15-Golvatinib-MET, VEGFR2 inhibitor	250	VEGFR	0.313793	0.141
160	4-MHB-B20-Motesanib-VEGFR, PDGFR, Ret, Kit inhibitor	1000	VEGFR	0.170982	0.745
161	4-MHB-C15-Golvatinib-MET, VEGFR2 inhibitor	25	VEGFR	0.24501	0.618
162	4-MHB-D12-ENMD-2076-pan-Aurora, VEGFR inhibitor	100	VEGFR	0.142485	0.975
163	4-MHB-D15-Golvatinib-MET, VEGFR2 inhibitor	2.5	VEGFR	0.307227	0.134
164	4-MHB-D20-Motesanib-VEGFR, PDGFR, Ret, Kit inhibitor	100	VEGFR	0.422336	0.003
165	4-MHB-E12-ENMD-2076-pan-Aurora, VEGFR inhibitor	10	VEGFR	0.386445	0
166	4-MHB-E20-Motesanib-VEGFR, PDGFR, Ret, Kit inhibitor	10	VEGFR	0.191125	0.982

167	4-MHB-F12-ENMD-2076-pan-Aurora, VEGFR inhibitor	1	VEGFR	0.323937	0.01
168	4-MHB-F15-Golvatinib-MET, VEGFR2 inhibitor	0.25	VEGFR	0.411191	0.005
169	4-MHB-F20-Motesanib-VEGFR, PDGFR, Ret, Kit inhibitor	1	VEGFR	0.234517	0.609
170	4-MHB-L16-Telatinib-VEGFR, KIT, PDGFR inhibitor	1	VEGFR	0.283457	0.477
171	4-MHB-M16-Telatinib-VEGFR, KIT, PDGFR inhibitor	10	VEGFR	0.399596	0.014
172	4-MHB-N16-Telatinib-VEGFR, KIT, PDGFR inhibitor	100	VEGFR	0.310518	0.444
173	4-MHB-O16-Telatinib-VEGFR, KIT, PDGFR inhibitor	1000	VEGFR	0.347361	0.015
174	4-MHB-P16-Telatinib-VEGFR, KIT, PDGFR inhibitor	10000	VEGFR	0.104843	0.989
175	2-MHB-L10-Idelalisib-PI3K inhibitor, p110δ-selective	1	PI3K	0.208556	0.798
176	2-MHB-M10-Idelalisib-PI3K inhibitor, p110δ-selective	10	PI3K	0.28198	0.431
177	2-MHB-N10-Idelalisib-PI3K inhibitor, p110δ-selective	100	PI3K	0.323493	0.011
178	2-MHB-O10-Idelalisib-PI3K inhibitor, p110δ-selective	1000	PI3K	0.193948	0.045
179	2-MHB-P10-Idelalisib-PI3K inhibitor, p110δ-selective	10000	PI3K	0.285649	0.135
180	3-MHB-A16-Perifosine-AKT/PI3K inhibitor	2500	PI3K	0.307816	0.042
181	3-MHB-C16-Perifosine-AKT/PI3K inhibitor	250	PI3K	0.184912	0.935
182	3-MHB-D16-Perifosine-AKT/PI3K inhibitor	25	PI3K	0.140325	0.121
183	3-MHB-E16-Perifosine-AKT/PI3K inhibitor	2.5	PI3K	0.254247	0.324
184	3-MHB-F16-Perifosine-AKT/PI3K inhibitor	0.25	PI3K	0.148785	0.838
185	3-MHB-F19-Duvelisib-PI3K inhibitor	500	PI3K	0.374195	0.004
186	3-MHB-G17-Miltefosine-Antimicrobial, inhibits PI3K/AKT	10000	PI3K	0.184683	0.719
187	3-MHB-G19-Duvelisib-PI3K inhibitor	50	PI3K	0.290195	0.007
188	3-MHB-H17-Miltefosine-Antimicrobial, inhibits PI3K/AKT	1000	PI3K	0.205328	0.626
189	3-MHB-I17-Miltefosine-Antimicrobial, inhibits PI3K/AKT	100	PI3K	0.119227	0.321
190	3-MHB-I19-Duvelisib-PI3K inhibitor	5	PI3K	0.346282	0.009
191	3-MHB-J17-Miltefosine-Antimicrobial, inhibits PI3K/AKT	10	PI3K	0.237252	0.256
192	3-MHB-J19-Duvelisib-PI3K inhibitor	0.5	PI3K	0.244996	0.554
193	3-MHB-K19-Duvelisib-PI3K inhibitor	0.05	PI3K	0.134169	0.866
194	3-MHB-L8-Pictilisib-PI3K inhibitor, pan-class I	1	PI3K	0.396786	0.003
195	3-MHB-L21-Taselisib-PI3K alpha, delta, (gamma) selective inhibitor	0.1	PI3K	0.299109	0.284
196	3-MHB-M8-Pictilisib-PI3K inhibitor, pan-class I	10	PI3K	0.370112	0.001
197	3-MHB-M21-Taselisib-PI3K alpha, delta, (gamma) selective inhibitor	1	PI3K	0.191761	0.884
198	3-MHB-N8-Pictilisib-PI3K inhibitor, pan-class I	100	PI3K	0.382392	0.002
199	3-MHB-N21-Taselisib-PI3K alpha, delta, (gamma) selective inhibitor	10	PI3K	0.428636	0

200	3-MHB-O8-Pictilisib-PI3K inhibitor, pan-class I	1000	PI3K	0.367516	0.038
201	3-MHB-O21-Taselisib-PI3K alpha, delta, (gamma) selective inhibitor	100	PI3K	0.403228	0.001
202	3-MHB-P8-Pictilisib-PI3K inhibitor, pan-class I	10000	PI3K	0.317513	0.698
203	3-MHB-P21-Taselisib-PI3K alpha, delta, (gamma) selective inhibitor	1000	PI3K	0.33825	0.18
204	4-MHB-A19-Alpelisib-PI3Kalpha inhibitor	2500	PI3K	0.414145	0.001
205	4-MHB-B19-Alpelisib-PI3Kalpha inhibitor	250	PI3K	0.401379	0
206	4-MHB-C19-Alpelisib-PI3Kalpha inhibitor	25	PI3K	0.244421	0.112
207	4-MHB-D19-Alpelisib-PI3Kalpha inhibitor	2.5	PI3K	0.318016	0.049
208	4-MHB-E19-Alpelisib-PI3Kalpha inhibitor	0.25	PI3K	0.163677	0.205
209	4-MHB-G2-TGR-1202-PI3Kdelta inhibitor	2500	PI3K	0.181462	0.957
210	4-MHB-G5-Sonolisib-PI3K inhibitor, pan-class I. Irreversible	10000	PI3K	0.221633	0.85
211	4-MHB-G14-NVP-BGT226-PI3K/mTOR inhibitor	100	PI3K	0.344313	0.122
212	4-MHB-G20-Buparlisib-PI3K inhibitor, pan-class I	10000	PI3K	0.343437	0.511
213	4-MHB-H2-TGR-1202-PI3Kdelta inhibitor	250	PI3K	0.194041	0.627
214	4-MHB-H5-Sonolisib-PI3K inhibitor, pan-class I. Irreversible	1000	PI3K	0.206258	0.919
215	4-MHB-H14-NVP-BGT226-PI3K/mTOR inhibitor	10	PI3K	0.395911	0.007
216	4-MHB-H20-Buparlisib-PI3K inhibitor, pan-class I	1000	PI3K	0.379764	0.009
217	4-MHB-I2-TGR-1202-PI3Kdelta inhibitor	25	PI3K	0.095262	0.964
218	4-MHB-I5-Sonolisib-PI3K inhibitor, pan-class I. Irreversible	100	PI3K	0.21046	0.853
219	4-MHB-I14-NVP-BGT226-PI3K/mTOR inhibitor	1	PI3K	0.193785	0.045
220	4-MHB-I20-Buparlisib-PI3K inhibitor, pan-class I	100	PI3K	0.374126	0
221	4-MHB-J2-TGR-1202-PI3Kdelta inhibitor	2.5	PI3K	0.150299	0.976
222	4-MHB-J5-Sonolisib-PI3K inhibitor, pan-class I. Irreversible	10	PI3K	0.303155	0.735
223	4-MHB-J20-Buparlisib-PI3K inhibitor, pan-class I	10	PI3K	0.266069	0.287
224	4-MHB-K2-TGR-1202-PI3Kdelta inhibitor	0.25	PI3K	0.085901	0.993
225	4-MHB-K4-Dactolisib-mTOR/(PI3K) inhibitor	0.1	PI3K	0.136161	0.982
226	4-MHB-K5-Sonolisib-PI3K inhibitor, pan-class I. Irreversible	1	PI3K	0.160291	0.716
227	4-MHB-K14-NVP-BGT226-PI3K/mTOR inhibitor	0.1	PI3K	0.234198	0.306
228	4-MHB-K20-Buparlisib-PI3K inhibitor, pan-class I	1	PI3K	0.251791	0.344
229	4-MHB-L4-Dactolisib-mTOR/(PI3K) inhibitor	1	PI3K	0.118512	0.778
230	4-MHB-L14-Gedatolisib-PI3K/mTOR inhibitor	0.1	PI3K	0.30035	0.043
231	4-MHB-L15-TG100-115-PI3K gamma/delta inhibitor	1	PI3K	0.16357	0.688
232	4-MHB-L21-Copanlisib-PI3K alpha, delta selective inhibitor	0.1	PI3K	0.30596	0.413

233	4-MHB-M14-Gedatolisib-PI3K/mTOR inhibitor	1	PI3K	0.367551	0
234	4-MHB-M15-TG100-115-PI3K gamma/delta inhibitor	10	PI3K	0.205558	0.492
235	4-MHB-M21-Copanlisib-PI3K alpha, delta selective inhibitor	1	PI3K	0.434874	0
236	4-MHB-N4-Dactolisib-mTOR/(PI3K) inhibitor	10	PI3K	0.424204	0.002
237	4-MHB-N14-Gedatolisib-PI3K/mTOR inhibitor	10	PI3K	0.346975	0.063
238	4-MHB-N15-TG100-115-PI3K gamma/delta inhibitor	100	PI3K	0.309374	0.001
239	4-MHB-N21-Copanlisib-PI3K alpha, delta selective inhibitor	10	PI3K	0.400017	0.001
240	4-MHB-O4-Dactolisib-mTOR/(PI3K) inhibitor	100	PI3K	0.37268	0.014
241	4-MHB-O14-Gedatolisib-PI3K/mTOR inhibitor	100	PI3K	0.376636	0.008
242	4-MHB-O15-TG100-115-PI3K gamma/delta inhibitor	1000	PI3K	0.231568	0.154
243	4-MHB-O21-Copanlisib-PI3K alpha, delta selective inhibitor	100	PI3K	0.363732	0.061
244	4-MHB-P4-Dactolisib-mTOR/(PI3K) inhibitor	1000	PI3K	0.405081	0
245	4-MHB-P14-Gedatolisib-PI3K/mTOR inhibitor	1000	PI3K	0.415312	0.005
246	4-MHB-P15-TG100-115-PI3K gamma/delta inhibitor	10000	PI3K	0.354002	0.037
247	4-MHB-P21-Copanlisib-PI3K alpha, delta selective inhibitor	1000	PI3K	0.477994	0
248	5-MHB-A6-LY3023414-PI3K/mTOR/DNA-PK inhibitor	2500	PI3K	0.45446	0
249	5-MHB-A7-AMG319-PI3Kdelta inhibitor	1000	PI3K	0.19621	0.977
250	5-MHB-A16-AZD-6482-PI3Kbeta-selective inhibitor	2500	PI3K	0.373084	0.007
251	5-MHB-A17-Palomid-529-AKT, MTOR, PI3K inhibitor	10000	PI3K	0.191911	0.935
252	5-MHB-B6-LY3023414-PI3K/mTOR/DNA-PK inhibitor	250	PI3K	0.312071	0.261
253	5-MHB-B7-AMG319-PI3Kdelta inhibitor	100	PI3K	0.26348	0.76
254	5-MHB-B17-Palomid-529-AKT, MTOR, PI3K inhibitor	1000	PI3K	0.402202	0.025
255	5-MHB-C6-LY3023414-PI3K/mTOR/DNA-PK inhibitor	25	PI3K	0.155573	0.805
256	5-MHB-C7-AMG319-PI3Kdelta inhibitor	10	PI3K	0.165969	0.58
257	5-MHB-C16-AZD-6482-PI3Kbeta-selective inhibitor	250	PI3K	0.284294	0.354
258	5-MHB-C17-Palomid-529-AKT, MTOR, PI3K inhibitor	100	PI3K	0.15831	0.222
259	5-MHB-D6-LY3023414-PI3K/mTOR/DNA-PK inhibitor	2.5	PI3K	0.133884	0.582
260	5-MHB-D7-AMG319-PI3Kdelta inhibitor	1	PI3K	0.218813	0.855
261	5-MHB-D16-AZD-6482-PI3Kbeta-selective inhibitor	25	PI3K	0.303334	0.656
262	5-MHB-D17-Palomid-529-AKT, MTOR, PI3K inhibitor	10	PI3K	0.138757	0.249
263	5-MHB-E6-LY3023414-PI3K/mTOR/DNA-PK inhibitor	0.25	PI3K	0.209176	0.083
264	5-MHB-E7-AMG319-PI3Kdelta inhibitor	0.1	PI3K	0.221409	0.29
265	5-MHB-E16-AZD-6482-PI3Kbeta-selective inhibitor	2.5	PI3K	0.157587	0.151

266	5-MHB-E17-Palomid-529-AKT, MTOR, PI3K inhibitor	1	PI3K	0.264202	0.118
267	5-MHB-F11-GSK2636771-PI3K beta selective inhibitor	10000	PI3K	0.391352	0.004
268	5-MHB-F16-AZD-6482-PI3Kbeta-selective inhibitor	0.25	PI3K	0.251422	0.148
269	5-MHB-G9-Serabelisib-PI3Kalpha selective inhibitor	10000	PI3K	0.33188	0.162
270	5-MHB-G11-GSK2636771-PI3K beta selective inhibitor	1000	PI3K	0.254916	0.014
271	5-MHB-H9-Serabelisib-PI3Kalpha selective inhibitor	1000	PI3K	0.376905	0.005
272	5-MHB-H11-GSK2636771-PI3K beta selective inhibitor	100	PI3K	0.280646	0.031
273	5-MHB-I9-Serabelisib-PI3Kalpha selective inhibitor	100	PI3K	0.16071	0.484
274	5-MHB-I11-GSK2636771-PI3K beta selective inhibitor	10	PI3K	0.128827	0.731
275	5-MHB-J9-Serabelisib-PI3Kalpha selective inhibitor	10	PI3K	0.112649	0.505
276	5-MHB-J11-GSK2636771-PI3K beta selective inhibitor	1	PI3K	0.201681	0.064
277	5-MHB-K9-Serabelisib-PI3Kalpha selective inhibitor	1	PI3K	0.360404	0.012
278	5-MHB-L14-AZD-8186-PI3Kbeta inhibitor	0.1	PI3K	0.278891	0.004
279	5-MHB-L20-ZSTK474-PI3K gamma selective inhibitor	1	PI3K	0.288672	0.661
280	5-MHB-L23-Omipalisib-PI3K/mTOR inhibitor	0.1	PI3K	0.345222	0
281	5-MHB-M14-AZD-8186-PI3Kbeta inhibitor	1	PI3K	0.340188	0.069
282	5-MHB-M20-ZSTK474-PI3K gamma selective inhibitor	10	PI3K	0.197293	0.041
283	5-MHB-M23-Omipalisib-PI3K/mTOR inhibitor	1	PI3K	0.416907	0
284	5-MHB-N14-AZD-8186-PI3Kbeta inhibitor	10	PI3K	0.230256	0.407
285	5-MHB-N20-ZSTK474-PI3K gamma selective inhibitor	100	PI3K	0.361257	0.002
286	5-MHB-N23-Omipalisib-PI3K/mTOR inhibitor	10	PI3K	0.396371	0.002
287	5-MHB-O14-AZD-8186-PI3Kbeta inhibitor	100	PI3K	0.325881	0.095
288	5-MHB-O20-ZSTK474-PI3K gamma selective inhibitor	1000	PI3K	0.358661	0.029
289	5-MHB-O23-Omipalisib-PI3K/mTOR inhibitor	100	PI3K	0.349663	0.089
290	5-MHB-P14-AZD-8186-PI3Kbeta inhibitor	1000	PI3K	0.387902	0.002
291	5-MHB-P20-ZSTK474-PI3K gamma selective inhibitor	10000	PI3K	0.351766	0.047
292	5-MHB-P23-Omipalisib-PI3K/mTOR inhibitor	1000	PI3K	0.289452	0.689
293	6-MHB-A8-TGX-221-PI3K beta selective inhibitor	10000	PI3K	0.321285	0.039
294	6-MHB-B8-TGX-221-PI3K beta selective inhibitor	1000	PI3K	0.160577	0.991
295	6-MHB-C8-TGX-221-PI3K beta selective inhibitor	100	PI3K	0.134364	0.859
296	6-MHB-D8-TGX-221-PI3K beta selective inhibitor	10	PI3K	0.26485	0.753
297	6-MHB-E8-TGX-221-PI3K beta selective inhibitor	1	PI3K	0.278821	0.601
298	6-MHB-L6-GDC-0084-PI3K/mTOR inhibitor	1	PI3K	0.174172	0.992

**299**	6-MHB-M6-GDC-0084-PI3K/mTOR inhibitor	10	PI3K	0.232902	0.082
**300**	6-MHB-N6-GDC-0084-PI3K/mTOR inhibitor	100	PI3K	0.376254	0.007
**301**	6-MHB-O6-GDC-0084-PI3K/mTOR inhibitor	1000	PI3K	0.32195	0.376
**302**	6-MHB-P6-GDC-0084-PI3K/mTOR inhibitor	10000	PI3K	0.30349	0.857
**303**	1-MHB-F11-Amsacrine-DNA intercalation, Topo II inhibitor	10000	Topoisome	0.703278	0
**304**	1-MHB-G11-Amsacrine-DNA intercalation, Topo II inhibitor	1000	Topoisome	0.693763	0
**305**	1-MHB-G20-Epirubicin-Topoisomerase II inhibitor	1000	Topoisome	0.597354	0
**306**	1-MHB-H11-Amsacrine-DNA intercalation, Topo II inhibitor	100	Topoisome	0.701416	0
**307**	1-MHB-H20-Epirubicin-Topoisomerase II inhibitor	100	Topoisome	0.680449	0
**308**	1-MHB-I11-Amsacrine-DNA intercalation, Topo II inhibitor	10	Topoisome	0.195337	0.22
**309**	1-MHB-I20-Epirubicin-Topoisomerase II inhibitor	10	Topoisome	0.540761	0
**310**	1-MHB-J11-Amsacrine-DNA intercalation, Topo II inhibitor	1	Topoisome	0.341694	0.327
**311**	1-MHB-J20-Epirubicin-Topoisomerase II inhibitor	1	Topoisome	0.465862	0
**312**	1-MHB-K11-SN-38-Active metabolite of irinotecan. Topoisomerase I inhibitor	1	Topoisome	0.694683	0
**313**	1-MHB-K20-Epirubicin-Topoisomerase II inhibitor	0.1	Topoisome	0.697683	0
**314**	1-MHB-L11-SN-38-Active metabolite of irinotecan. Topoisomerase I inhibitor	10	Topoisome	0.676607	0
**315**	1-MHB-L14-Topotecan-Topoisomerase I inhibitor. Camptothecin analog	1	Topoisome	0.611669	0
**316**	1-MHB-M11-SN-38-Active metabolite of irinotecan. Topoisomerase I inhibitor	100	Topoisome	0.620021	0
**317**	1-MHB-M14-Topotecan-Topoisomerase I inhibitor. Camptothecin analog	10	Topoisome	0.674334	0
**318**	1-MHB-N14-Topotecan-Topoisomerase I inhibitor. Camptothecin analog	100	Topoisome	0.670908	0
**319**	1-MHB-O11-SN-38-Active metabolite of irinotecan. Topoisomerase I inhibitor	1000	Topoisome	0.399638	0.281
**320**	1-MHB-O14-Topotecan-Topoisomerase I inhibitor. Camptothecin analog	1000	Topoisome	0.689978	0
**321**	1-MHB-P11-SN-38-Active metabolite of irinotecan. Topoisomerase I inhibitor	10000	Topoisome	0.391203	0.202
**322**	1-MHB-P14-Topotecan-Topoisomerase I inhibitor. Camptothecin analog	10000	Topoisome	0.388443	0.36
**323**	3-MHB-A11-Etoposide-Topoisomerase II inhibitor	10000	Topoisome	0.64886	0
**324**	3-MHB-B11-Etoposide-Topoisomerase II inhibitor	1000	Topoisome	0.607181	0
**325**	3-MHB-C11-Etoposide-Topoisomerase II inhibitor	100	Topoisome	0.589541	0
**326**	3-MHB-D11-Etoposide-Topoisomerase II inhibitor	10	Topoisome	0.642925	0
**327**	3-MHB-E11-Etoposide-Topoisomerase II inhibitor	1	Topoisome	0.539661	0
**328**	3-MHB-G9-Daunorubicin-Topoisomerase II inhibitor	1000	Topoisome	0.495719	0.009
**329**	3-MHB-G10-Teniposide-Topoisomerase II inhibitor	10000	Topoisome	0.567507	0
**330**	3-MHB-H9-Daunorubicin-Topoisomerase II inhibitor	100	Topoisome	0.62262	0
**331**	3-MHB-H10-Teniposide-Topoisomerase II inhibitor	1000	Topoisome	0.485383	0

332	3-MHB-I9-Daunorubicin-Topoisomerase II inhibitor	10	Topoisome	0.505706	0.001
333	3-MHB-I10-Teniposide-Topoisomerase II inhibitor	100	Topoisome	0.297245	0.356
334	3-MHB-J9-Daunorubicin-Topoisomerase II inhibitor	1	Topoisome	0.311119	0.013
335	3-MHB-J10-Teniposide-Topoisomerase II inhibitor	10	Topoisome	0.574974	0
336	3-MHB-K7-Idarubicin-Topoisomerase II inhibitor	0.1	Topoisome	0.204331	0.463
337	3-MHB-K9-Daunorubicin-Topoisomerase II inhibitor	0.1	Topoisome	0.677026	0
338	3-MHB-K10-Teniposide-Topoisomerase II inhibitor	1	Topoisome	0.533212	0
339	3-MHB-L6-Doxorubicin-Topoisomerase II inhibitor	0.1	Topoisome	0.121387	0.999
340	3-MHB-L7-Idarubicin-Topoisomerase II inhibitor	1	Topoisome	0.090453	1
341	3-MHB-L9-Valrubicin-Topoisomerase II inhibitor	0.5	Topoisome	0.570121	0
342	3-MHB-L10-Mitoxantrone-Topoisomerase II inhibitor	0.1	Topoisome	0.565582	0
343	3-MHB-L16-Pixantrone-topoisomerase II inhibitor	1	Topoisome	0.27925	0.579
344	3-MHB-M6-Doxorubicin-Topoisomerase II inhibitor	1	Topoisome	0.699641	0
345	3-MHB-M7-Idarubicin-Topoisomerase II inhibitor	10	Topoisome	0.57803	0
346	3-MHB-M9-Valrubicin-Topoisomerase II inhibitor	5	Topoisome	0.602218	0
347	3-MHB-M10-Mitoxantrone-Topoisomerase II inhibitor	1	Topoisome	0.296801	0.12
348	3-MHB-M16-Pixantrone-topoisomerase II inhibitor	10	Topoisome	0.508611	0
349	3-MHB-N6-Doxorubicin-Topoisomerase II inhibitor	10	Topoisome	0.26003	0.715
350	3-MHB-N9-Valrubicin-Topoisomerase II inhibitor	50	Topoisome	0.631249	0
351	3-MHB-N10-Mitoxantrone-Topoisomerase II inhibitor	10	Topoisome	0.644819	0
352	3-MHB-N16-Pixantrone-topoisomerase II inhibitor	100	Topoisome	0.498876	0.007
353	3-MHB-O6-Doxorubicin-Topoisomerase II inhibitor	100	Topoisome	0.635874	0
354	3-MHB-O7-Idarubicin-Topoisomerase II inhibitor	100	Topoisome	0.646007	0
355	3-MHB-O9-Valrubicin-Topoisomerase II inhibitor	500	Topoisome	0.646781	0
356	3-MHB-O10-Mitoxantrone-Topoisomerase II inhibitor	100	Topoisome	0.653696	0
357	3-MHB-O16-Pixantrone-topoisomerase II inhibitor	1000	Topoisome	0.30071	0.318
358	3-MHB-P6-Doxorubicin-Topoisomerase II inhibitor	1000	Topoisome	0.607979	0
359	3-MHB-P7-Idarubicin-Topoisomerase II inhibitor	1000	Topoisome	0.473992	0.021
360	3-MHB-P9-Valrubicin-Topoisomerase II inhibitor	5000	Topoisome	0.351983	0.488
361	3-MHB-P10-Mitoxantrone-Topoisomerase II inhibitor	1000	Topoisome	0.6094	0
362	3-MHB-P16-Pixantrone-topoisomerase II inhibitor	10000	Topoisome	0.274174	0.452
363	1-MHB-A10-Vinorelbine-Mitotic inhibitor. Vinca alkaloid microtubule depolymerizer	10000	Mitotic	0.631105	0.001
364	1-MHB-A13-Ixabepilone-Mitotic inhibitor. Epothilone microtubule stabilizer.	1000	Mitotic	0.608809	0.001

365	1-MHB-A18-Paclitaxel-Mitotic inhibitor, taxane microtubule stabilizer	1000	Mitotic	0.620835	0
366	1-MHB-B10-Vinorelbine-Mitotic inhibitor. Vinca alkaloid microtubule depolymerizer	1000	Mitotic	0.606963	0
367	1-MHB-B13-Ixabepilone-Mitotic inhibitor. Epothilone microtubule stabilizer.	100	Mitotic	0.097417	0.999
368	1-MHB-B18-Paclitaxel-Mitotic inhibitor, taxane microtubule stabilizer	100	Mitotic	0.608313	0
369	1-MHB-C10-Vinorelbine-Mitotic inhibitor. Vinca alkaloid microtubule depolymerizer	100	Mitotic	0.600754	0
370	1-MHB-C13-Ixabepilone-Mitotic inhibitor. Epothilone microtubule stabilizer.	10	Mitotic	0.576079	0
371	1-MHB-C18-Paclitaxel-Mitotic inhibitor, taxane microtubule stabilizer	10	Mitotic	0.613066	0
372	1-MHB-D10-Vinorelbine-Mitotic inhibitor. Vinca alkaloid microtubule depolymerizer	10	Mitotic	0.491792	0.002
373	1-MHB-D13-Ixabepilone-Mitotic inhibitor. Epothilone microtubule stabilizer.	1	Mitotic	0.621629	0
374	1-MHB-D18-Paclitaxel-Mitotic inhibitor, taxane microtubule stabilizer	1	Mitotic	0.602597	0
375	1-MHB-E10-Vinorelbine-Mitotic inhibitor. Vinca alkaloid microtubule depolymerizer	1	Mitotic	0.687661	0
376	1-MHB-E13-Ixabepilone-Mitotic inhibitor. Epothilone microtubule stabilizer.	0.1	Mitotic	0.165132	0.596
377	1-MHB-E18-Paclitaxel-Mitotic inhibitor, taxane microtubule stabilizer	0.1	Mitotic	0.535301	0
378	1-MHB-F13-Vinblastine-Mitotic inhibitor. Vinca alkaloid microtubule depolymerizer	1000	Mitotic	0.614457	0
379	1-MHB-G13-Vinblastine-Mitotic inhibitor. Vinca alkaloid microtubule depolymerizer	100	Mitotic	0.604081	0.001
380	1-MHB-G15-Eribulin-Mitotic inhibitor, microtubule depolymerizer.	1000	Mitotic	0.620609	0.001
381	1-MHB-H13-Vinblastine-Mitotic inhibitor. Vinca alkaloid microtubule depolymerizer	10	Mitotic	0.559525	0
382	1-MHB-H15-Eribulin-Mitotic inhibitor, microtubule depolymerizer.	100	Mitotic	0.628341	0
383	1-MHB-I13-Vinblastine-Mitotic inhibitor. Vinca alkaloid microtubule depolymerizer	1	Mitotic	0.409935	0.082
384	1-MHB-I15-Eribulin-Mitotic inhibitor, microtubule depolymerizer.	10	Mitotic	0.582356	0.002
385	1-MHB-J13-Vinblastine-Mitotic inhibitor. Vinca alkaloid microtubule depolymerizer	0.1	Mitotic	0.222238	0.912
386	1-MHB-J15-Eribulin-Mitotic inhibitor, microtubule depolymerizer.	1	Mitotic	0.348701	0.056
387	1-MHB-K7-Vincristine-Mitotic inhibitor. Vinca alkaloid microtubule depolymerizer	0.1	Mitotic	0.504165	0.001
388	1-MHB-K15-Eribulin-Mitotic inhibitor, microtubule depolymerizer.	0.1	Mitotic	0.301748	0.014
389	1-MHB-L7-Vincristine-Mitotic inhibitor. Vinca alkaloid microtubule depolymerizer	1	Mitotic	0.596092	0
390	1-MHB-L20-Vinflunine-Mitotic inhibitor. Vinca alkaloid microtubule depolymerizer	0.1	Mitotic	0.353596	0.051
391	1-MHB-M7-Vincristine-Mitotic inhibitor. Vinca alkaloid microtubule depolymerizer	10	Mitotic	0.505184	0
392	1-MHB-M20-Vinflunine-Mitotic inhibitor. Vinca alkaloid microtubule depolymerizer	1	Mitotic	0.57589	0
393	1-MHB-N20-Vinflunine-Mitotic inhibitor. Vinca alkaloid microtubule depolymerizer	10	Mitotic	0.49663	0.005
394	1-MHB-O7-Vincristine-Mitotic inhibitor. Vinca alkaloid microtubule depolymerizer	100	Mitotic	0.55972	0
395	1-MHB-O20-Vinflunine-Mitotic inhibitor. Vinca alkaloid microtubule depolymerizer	100	Mitotic	0.431813	0.222
396	1-MHB-P7-Vincristine-Mitotic inhibitor. Vinca alkaloid microtubule depolymerizer	1000	Mitotic	0.564952	0
397	1-MHB-P20-Vinflunine-Mitotic inhibitor. Vinca alkaloid microtubule depolymerizer	1000	Mitotic	0.556534	0

398	3-MHB-A7-Docetaxel-Mitotic inhibitor, taxane microtubule stabilizer	1000	Mitotic	0.531378	0.008
399	3-MHB-B7-Docetaxel-Mitotic inhibitor, taxane microtubule stabilizer	100	Mitotic	0.573395	0.005
400	3-MHB-C7-Docetaxel-Mitotic inhibitor, taxane microtubule stabilizer	10	Mitotic	0.43197	0.076
401	3-MHB-D7-Docetaxel-Mitotic inhibitor, taxane microtubule stabilizer	1	Mitotic	0.358805	0.309
402	3-MHB-E7-Docetaxel-Mitotic inhibitor, taxane microtubule stabilizer	0.1	Mitotic	0.246778	0.493
403	6-MHB-L19-ABT-751-Mitotic inhibitor. Colchicine site binding microtubule depolymerizer.	1	Mitotic	0.171386	0.706
404	6-MHB-M19-ABT-751-Mitotic inhibitor. Colchicine site binding microtubule depolymerizer.	10	Mitotic	0.553998	0.001
405	6-MHB-N19-ABT-751-Mitotic inhibitor. Colchicine site binding microtubule depolymerizer.	100	Mitotic	0.266759	0.716
406	6-MHB-O19-ABT-751-Mitotic inhibitor. Colchicine site binding microtubule depolymerizer.	1000	Mitotic	0.546751	0.003
407	6-MHB-P19-ABT-751-Mitotic inhibitor. Colchicine site binding microtubule depolymerizer.	10000	Mitotic	0.611121	0
408	2-MHB-A12-Trametinib-MEK1/2 inhibitor	250	MEK1/2	0.757626	0
409	2-MHB-B12-Trametinib-MEK1/2 inhibitor	25	MEK1/2	0.754409	0
410	2-MHB-D12-Trametinib-MEK1/2 inhibitor	2.5	MEK1/2	0.78788	0
411	2-MHB-E12-Trametinib-MEK1/2 inhibitor	0.25	MEK1/2	0.734061	0
412	2-MHB-F12-Trametinib-MEK1/2 inhibitor	2.5000000	MEK1/2	0.578468	0
413	2-MHB-F14-Cobimetinib-MEK1/2 inhibitor	1000	MEK1/2	0.692047	0
414	2-MHB-G14-Cobimetinib-MEK1/2 inhibitor	100	MEK1/2	0.713974	0
415	2-MHB-H14-Cobimetinib-MEK1/2 inhibitor	10	MEK1/2	0.743877	0
416	2-MHB-I14-Cobimetinib-MEK1/2 inhibitor	1	MEK1/2	0.701624	0
417	2-MHB-K14-Cobimetinib-MEK1/2 inhibitor	0.1	MEK1/2	0.660367	0
418	2-MHB-L20-Selumetinib-MEK1/2 inhibitor	1	MEK1/2	0.682634	0
419	2-MHB-M20-Selumetinib-MEK1/2 inhibitor	10	MEK1/2	0.783405	0
420	2-MHB-N20-Selumetinib-MEK1/2 inhibitor	100	MEK1/2	0.771249	0
421	2-MHB-O20-Selumetinib-MEK1/2 inhibitor	1000	MEK1/2	0.753938	0
422	2-MHB-P20-Selumetinib-MEK1/2 inhibitor	10000	MEK1/2	0.767152	0
423	4-MHB-A10-Binimetinib-MEK1/2 inhibitor	1000	MEK1/2	0.720703	0
424	4-MHB-A13-PD0325901-MEK1/2 inhibitor	1000	MEK1/2	0.763977	0
425	4-MHB-B10-Binimetinib-MEK1/2 inhibitor	100	MEK1/2	0.805308	0
426	4-MHB-B13-PD0325901-MEK1/2 inhibitor	100	MEK1/2	0.798139	0
427	4-MHB-C10-Binimetinib-MEK1/2 inhibitor	10	MEK1/2	0.707846	0
428	4-MHB-C13-PD0325901-MEK1/2 inhibitor	10	MEK1/2	0.829412	0
429	4-MHB-D10-Binimetinib-MEK1/2 inhibitor	1	MEK1/2	0.748262	0
430	4-MHB-D13-PD0325901-MEK1/2 inhibitor	1	MEK1/2	0.738953	0

431	4-MHB-E10-Binimetinib-MEK1/2 inhibitor	0.1	MEK1/2	0.578846	0.001
432	4-MHB-E13-PD0325901-MEK1/2 inhibitor	0.1	MEK1/2	0.699172	0
433	4-MHB-L19-GDC-0623-MEK1/2 inhibitor	0.25	MEK1/2	0.385544	0.159
434	4-MHB-M19-GDC-0623-MEK1/2 inhibitor	2.5	MEK1/2	0.259862	0.547
435	4-MHB-N19-GDC-0623-MEK1/2 inhibitor	25	MEK1/2	0.770953	0
436	4-MHB-O19-GDC-0623-MEK1/2 inhibitor	250	MEK1/2	0.742218	0
437	4-MHB-P19-GDC-0623-MEK1/2 inhibitor	2500	MEK1/2	0.704277	0
438	1-MHB-L2-Olaparib-PARP inhibitor	1	PARP	0.754451	0
439	1-MHB-L6-Rucaparib-PARP inhibitor	1	PARP	0.595868	0
440	1-MHB-M2-Olaparib-PARP inhibitor	10	PARP	0.669786	0.001
441	1-MHB-M6-Rucaparib-PARP inhibitor	10	PARP	0.27802	0.387
442	1-MHB-N2-Olaparib-PARP inhibitor	100	PARP	0.673237	0
443	1-MHB-N6-Rucaparib-PARP inhibitor	100	PARP	0.492445	0.019
444	1-MHB-O2-Olaparib-PARP inhibitor	1000	PARP	0.636216	0.002
445	1-MHB-O6-Rucaparib-PARP inhibitor	1000	PARP	0.40213	0.162
446	1-MHB-P2-Olaparib-PARP inhibitor	10000	PARP	0.595889	0.003
447	1-MHB-P6-Rucaparib-PARP inhibitor	10000	PARP	0.533114	0.037
448	7-MHB-A3-Talazoparib-PARP1/2 inhibitor	1000	PARP	0.5012	0.052
449	7-MHB-B2-Veliparib-PARP inhibitor	10000	PARP	0.438621	0.174
450	7-MHB-B3-Talazoparib-PARP1/2 inhibitor	100	PARP	0.529009	0.021
451	7-MHB-C2-Veliparib-PARP inhibitor	1000	PARP	0.307735	0.165
452	7-MHB-C3-Talazoparib-PARP1/2 inhibitor	10	PARP	0.51711	0.055
453	7-MHB-D2-Veliparib-PARP inhibitor	100	PARP	0.445214	0.108
454	7-MHB-D3-Talazoparib-PARP1/2 inhibitor	1	PARP	0.446453	0.126
455	7-MHB-E2-Veliparib-PARP inhibitor	10	PARP	0.557994	0.008
456	7-MHB-E3-Talazoparib-PARP1/2 inhibitor	0.1	PARP	0.457195	0.102
457	7-MHB-F2-Veliparib-PARP inhibitor	1	PARP	0.517358	0.026
458	7-MHB-G2-Niraparib-PARP inhibitor	10000	PARP	0.448513	0.188
459	7-MHB-H2-Niraparib-PARP inhibitor	1000	PARP	0.57245	0.005
460	7-MHB-I2-Niraparib-PARP inhibitor	100	PARP	0.426593	0.028
461	7-MHB-J2-Niraparib-PARP inhibitor	10	PARP	0.472297	0.075
462	7-MHB-K2-Niraparib-PARP inhibitor	1	PARP	0.304534	0.101
463	3-MHB-A19-Dinaciclib-CDK inhibitor	1000	CDK	0.462004	0.015

464	3-MHB-B19-Dinaciclib-CDK inhibitor	100	CDK	0.492407	0.006
465	3-MHB-B23-Abemaciclib-CDK4/6 inhibitor	2500	CDK	0.373903	0.201
466	3-MHB-C19-Dinaciclib-CDK inhibitor	10	CDK	0.364698	0.229
467	3-MHB-C23-Abemaciclib-CDK4/6 inhibitor	250	CDK	0.249424	0.559
468	3-MHB-D19-Dinaciclib-CDK inhibitor	1	CDK	0.228276	0.424
469	3-MHB-D23-Abemaciclib-CDK4/6 inhibitor	25	CDK	0.282948	0.562
470	3-MHB-E19-Dinaciclib-CDK inhibitor	0.1	CDK	0.438093	0.008
471	3-MHB-E23-Abemaciclib-CDK4/6 inhibitor	2.5	CDK	0.233664	0.248
472	3-MHB-F23-Abemaciclib-CDK4/6 inhibitor	0.25	CDK	0.381075	0.247
473	3-MHB-K17-Palbociclib-CDK4/6 inhibitor	1	CDK	0.285836	0.267
474	3-MHB-L19-Ribociclib-CDK4/6 inhibitor	1	CDK	0.237739	0.734
475	3-MHB-M17-Palbociclib-CDK4/6 inhibitor	10	CDK	0.356691	0.208
476	3-MHB-M19-Ribociclib-CDK4/6 inhibitor	10	CDK	0.131092	0.522
477	3-MHB-N17-Palbociclib-CDK4/6 inhibitor	100	CDK	0.440077	0.014
478	3-MHB-N19-Ribociclib-CDK4/6 inhibitor	100	CDK	0.31898	0.308
479	3-MHB-O17-Palbociclib-CDK4/6 inhibitor	1000	CDK	0.362585	0.185
480	3-MHB-O19-Ribociclib-CDK4/6 inhibitor	1000	CDK	0.17394	0.821
481	3-MHB-P17-Palbociclib-CDK4/6 inhibitor	10000	CDK	0.355114	0.138
482	3-MHB-P19-Ribociclib-CDK4/6 inhibitor	10000	CDK	0.466088	0.01
483	4-MHB-A4-SNS-032-CDK inhibitor	10000	CDK	0.475974	0.036
484	4-MHB-A8-Milciclib-CDK2 inhibitor	10000	CDK	0.481918	0.024
485	4-MHB-B4-SNS-032-CDK inhibitor	1000	CDK	0.474381	0.074
486	4-MHB-B8-Milciclib-CDK2 inhibitor	1000	CDK	0.343111	0.199
487	4-MHB-C4-SNS-032-CDK inhibitor	100	CDK	0.29859	0.453
488	4-MHB-C8-Milciclib-CDK2 inhibitor	100	CDK	0.211105	0.804
489	4-MHB-D4-SNS-032-CDK inhibitor	10	CDK	0.384658	0.234
490	4-MHB-D8-Milciclib-CDK2 inhibitor	10	CDK	0.417693	0.034
491	4-MHB-E4-SNS-032-CDK inhibitor	1	CDK	0.169616	0.517
492	4-MHB-E8-Milciclib-CDK2 inhibitor	1	CDK	0.393114	0.186
493	4-MHB-F4-Seliciclib-CDK2/7/9 inhibitor	10000	CDK	0.397311	0.08
494	4-MHB-F22-Alvocidib-CDK inhibitor	10000	CDK	0.483843	0.058
495	4-MHB-G4-Seliciclib-CDK2/7/9 inhibitor	1000	CDK	0.334665	0.322
496	4-MHB-G22-Alvocidib-CDK inhibitor	1000	CDK	0.469883	0.049

497	4-MHB-H4-Seliciclib-CDK2/7/9 inhibitor	100	CDK	0.286618	0.207
498	4-MHB-H22-Alvocidib-CDK inhibitor	100	CDK	0.352939	0.232
499	4-MHB-I4-Seliciclib-CDK2/7/9 inhibitor	10	CDK	0.263513	0.573
500	4-MHB-I22-Alvocidib-CDK inhibitor	10	CDK	0.298953	0.09
501	4-MHB-J4-Seliciclib-CDK2/7/9 inhibitor	1	CDK	0.226903	0.612
502	4-MHB-J22-Alvocidib-CDK inhibitor	1	CDK	0.209839	0.191
503	5-MHB-A19-AZD-5438-CDK1,2,9 inhibitor	10000	CDK	0.422088	0.027
504	5-MHB-B19-AZD-5438-CDK1,2,9 inhibitor	1000	CDK	0.343945	0.301
505	5-MHB-C19-AZD-5438-CDK1,2,9 inhibitor	100	CDK	0.46616	0.001
506	5-MHB-D19-AZD-5438-CDK1,2,9 inhibitor	10	CDK	0.186476	0.273
507	5-MHB-E19-AZD-5438-CDK1,2,9 inhibitor	1	CDK	0.341308	0.07
508	5-MHB-K17-AT7519-CDK1, 2, 4, 6 and 9 inhibitor	1	CDK	0.308808	0.336
509	5-MHB-M17-AT7519-CDK1, 2, 4, 6 and 9 inhibitor	10	CDK	0.31229	0.483
510	5-MHB-N17-AT7519-CDK1, 2, 4, 6 and 9 inhibitor	100	CDK	0.389841	0.023
511	5-MHB-O17-AT7519-CDK1, 2, 4, 6 and 9 inhibitor	1000	CDK	0.321441	0.404
512	5-MHB-P17-AT7519-CDK1, 2, 4, 6 and 9 inhibitor	10000	CDK	0.412076	0.118
513	6-MHB-A17-Senexin B-CDK8/19 inhibitor	1000	CDK	0.423364	0.047
514	6-MHB-B17-Senexin B-CDK8/19 inhibitor	100	CDK	0.423434	0.023
515	6-MHB-C17-Senexin B-CDK8/19 inhibitor	10	CDK	0.343325	0.164
516	6-MHB-D17-Senexin B-CDK8/19 inhibitor	1	CDK	0.113029	0.763
517	6-MHB-E17-Senexin B-CDK8/19 inhibitor	0.1	CDK	0.428552	0.017
518	6-MHB-L15-THZ2-CDK7 inhibitor	1	CDK	0.30277	0.325
519	6-MHB-M15-THZ2-CDK7 inhibitor	10	CDK	0.256444	0.492
520	6-MHB-N15-THZ2-CDK7 inhibitor	100	CDK	0.373262	0.098
521	6-MHB-O15-THZ2-CDK7 inhibitor	1000	CDK	0.486	0.041
522	6-MHB-P15-THZ2-CDK7 inhibitor	10000	CDK	0.474689	0.064
523	7-MHB-A21-dBET1-BET-targeting PROTAC	10000	BET	0.611068	0
524	7-MHB-A22-PFI-1-BET family inhibitor	30000	BET	0.667588	0
525	7-MHB-B21-dBET1-BET-targeting PROTAC	1000	BET	0.479999	0.03
526	7-MHB-B22-PFI-1-BET family inhibitor	3000	BET	0.58825	0
527	7-MHB-C21-dBET1-BET-targeting PROTAC	100	BET	0.172237	0.414
528	7-MHB-C22-PFI-1-BET family inhibitor	300	BET	0.152748	0.478
529	7-MHB-D21-dBET1-BET-targeting PROTAC	10	BET	0.176227	0.458

530	7-MHB-D22-PFI-1-BET family inhibitor	30	BET	0.365864	0.035
531	7-MHB-E21-dBET1-BET-targeting PROTAC	1	BET	0.559114	0
532	7-MHB-E22-PFI-1-BET family inhibitor	3	BET	0.486623	0.01
533	7-MHB-G10-Birabresib-BET family inhibitor	10000	BET	0.637334	0
534	7-MHB-G15-I-BET151-BET family inhibitor	10000	BET	0.626441	0
535	7-MHB-H10-Birabresib-BET family inhibitor	1000	BET	0.633219	0
536	7-MHB-H15-I-BET151-BET family inhibitor	1000	BET	0.607704	0
537	7-MHB-I10-Birabresib-BET family inhibitor	100	BET	0.592784	0
538	7-MHB-I15-I-BET151-BET family inhibitor	100	BET	0.523992	0
539	7-MHB-J10-Birabresib-BET family inhibitor	10	BET	0.229696	0.297
540	7-MHB-J15-I-BET151-BET family inhibitor	10	BET	0.20625	0.789
541	7-MHB-K10-Birabresib-BET family inhibitor	1	BET	0.135897	0.643
542	7-MHB-K13-Mivebresib-BET family inhibitor	1	BET	0.415969	0.087
543	7-MHB-K15-I-BET151-BET family inhibitor	1	BET	0.283187	0.165
544	7-MHB-L12-Molibresib-BET family inhibitor	1	BET	0.594876	0
545	7-MHB-L13-Mivebresib-BET family inhibitor	10	BET	0.55557	0
546	7-MHB-L20-JQ1-BET family inhibitor	1	BET	0.644639	0
547	7-MHB-L23-ARV-825-BET-targeting PROTAC	0.03	BET	0.647869	0
548	7-MHB-M12-Molibresib-BET family inhibitor	10	BET	0.426235	0.01
549	7-MHB-M13-Mivebresib-BET family inhibitor	100	BET	0.630007	0
550	7-MHB-M20-JQ1-BET family inhibitor	10	BET	0.312628	0.243
551	7-MHB-M23-ARV-825-BET-targeting PROTAC	0.3	BET	0.18905	0.414
552	7-MHB-N12-Molibresib-BET family inhibitor	100	BET	0.50203	0.001
553	7-MHB-N13-Mivebresib-BET family inhibitor	1000	BET	0.570942	0
554	7-MHB-N20-JQ1-BET family inhibitor	100	BET	0.5642	0
555	7-MHB-N23-ARV-825-BET-targeting PROTAC	3	BET	0.484302	0.001
556	7-MHB-O12-Molibresib-BET family inhibitor	1000	BET	0.641837	0
557	7-MHB-O20-JQ1-BET family inhibitor	1000	BET	0.614645	0
558	7-MHB-O23-ARV-825-BET-targeting PROTAC	30	BET	0.574042	0
559	7-MHB-P12-Molibresib-BET family inhibitor	10000	BET	0.625606	0
560	7-MHB-P13-Mivebresib-BET family inhibitor	10000	BET	0.591833	0
561	7-MHB-P20-JQ1-BET family inhibitor	10000	BET	0.619403	0
562	7-MHB-P23-ARV-825-BET-targeting PROTAC	300	BET	0.597059	0

**563**	8-MHB-K22-CPI-0610-BET family inhibitor	1	BET	0.215929	0.838
**564**	8-MHB-L22-CPI-0610-BET family inhibitor	10	BET	0.546325	0
**565**	8-MHB-M22-CPI-0610-BET family inhibitor	100	BET	0.504767	0
**566**	8-MHB-N22-CPI-0610-BET family inhibitor	1000	BET	0.586395	0
**567**	8-MHB-O22-CPI-0610-BET family inhibitor	10000	BET	0.581693	0.001
**568**	1-MHB-A3-Vorinostat-HDAC inhibitor	10000	HDAC	0.36695	0.292
**569**	1-MHB-B3-Vorinostat-HDAC inhibitor	1000	HDAC	0.461062	0
**570**	1-MHB-C3-Vorinostat-HDAC inhibitor	100	HDAC	0.167496	0.116
**571**	1-MHB-D3-Vorinostat-HDAC inhibitor	10	HDAC	0.162487	0.472
**572**	1-MHB-E3-Vorinostat-HDAC inhibitor	1	HDAC	0.148196	0.621
**573**	1-MHB-L12-Romidepsin-HDAC inhibitor	0.1	HDAC	0.158724	0.863
**574**	1-MHB-M12-Romidepsin-HDAC inhibitor	1	HDAC	0.543064	0
**575**	1-MHB-N12-Romidepsin-HDAC inhibitor	10	HDAC	0.510769	0
**576**	1-MHB-O12-Romidepsin-HDAC inhibitor	100	HDAC	0.496858	0.002
**577**	1-MHB-P12-Romidepsin-HDAC inhibitor	1000	HDAC	0.224826	0.912
**578**	3-MHB-A4-Panobinostat-HDAC inhibitor	1000	HDAC	0.415322	0.098
**579**	3-MHB-B4-Panobinostat-HDAC inhibitor	100	HDAC	0.473842	0
**580**	3-MHB-C4-Panobinostat-HDAC inhibitor	10	HDAC	0.540808	0
**581**	3-MHB-D4-Panobinostat-HDAC inhibitor	1	HDAC	0.098941	0.838
**582**	3-MHB-E4-Panobinostat-HDAC inhibitor	0.1	HDAC	0.318764	0.02
**583**	3-MHB-F7-Quisinostat-HDAC inhibitor	1000	HDAC	0.433932	0.04
**584**	3-MHB-G7-Quisinostat-HDAC inhibitor	100	HDAC	0.475463	0
**585**	3-MHB-G12-Valproic acid-HDAC inhibitor	1000000	HDAC	0.153005	0.142
**586**	3-MHB-H7-Quisinostat-HDAC inhibitor	10	HDAC	0.567006	0
**587**	3-MHB-H12-Valproic acid-HDAC inhibitor	100000	HDAC	0.138349	0.906
**588**	3-MHB-I7-Quisinostat-HDAC inhibitor	1	HDAC	0.198139	0.26
**589**	3-MHB-I12-Valproic acid-HDAC inhibitor	10000	HDAC	0.119891	0.969
**590**	3-MHB-J7-Quisinostat-HDAC inhibitor	0.1	HDAC	0.130169	0.523
**591**	3-MHB-J12-Valproic acid-HDAC inhibitor	1000	HDAC	0.204474	0.12
**592**	3-MHB-K3-Belinostat-HDAC inhibitor	1	HDAC	0.168957	0.824
**593**	3-MHB-K12-Valproic acid-HDAC inhibitor	100	HDAC	0.407337	0
**594**	3-MHB-L3-Belinostat-HDAC inhibitor	10	HDAC	0.130661	0.792
**595**	3-MHB-M3-Belinostat-HDAC inhibitor	100	HDAC	0.415653	0

596	3-MHB-N3-Belinostat-HDAC inhibitor	1000	HDAC	0.435361	0.001
597	3-MHB-O3-Belinostat-HDAC inhibitor	10000	HDAC	0.50679	0
598	7-MHB-A5-Mocetinostat-HDAC inhibitor (HDAC1 & 2-selective)	10000	HDAC	0.485648	0
599	7-MHB-A7-CUDC-907-HDAC1/2/3/10, PI3Kalpha inhibitor	10000	HDAC	0.493781	0
600	7-MHB-A9-Givinostat-HDAC inhibitor	1000	HDAC	0.47013	0
601	7-MHB-A12-Rocilinostat-HDAC-6 selective inhibitor	10000	HDAC	0.530699	0
602	7-MHB-B5-Mocetinostat-HDAC inhibitor (HDAC1 & 2-selective)	1000	HDAC	0.504397	0
603	7-MHB-B12-Rocilinostat-HDAC-6 selective inhibitor	1000	HDAC	0.492797	0
604	7-MHB-C5-Mocetinostat-HDAC inhibitor (HDAC1 & 2-selective)	100	HDAC	0.569917	0
605	7-MHB-C7-CUDC-907-HDAC1/2/3/10, PI3Kalpha inhibitor	100	HDAC	0.513796	0
606	7-MHB-C9-Givinostat-HDAC inhibitor	100	HDAC	0.538142	0
607	7-MHB-D7-CUDC-907-HDAC1/2/3/10, PI3Kalpha inhibitor	10	HDAC	0.531154	0
608	7-MHB-D9-Givinostat-HDAC inhibitor	10	HDAC	0.594245	0
609	7-MHB-D12-Rocilinostat-HDAC-6 selective inhibitor	100	HDAC	0.478107	0
610	7-MHB-E5-Mocetinostat-HDAC inhibitor (HDAC1 & 2-selective)	10	HDAC	0.56511	0
611	7-MHB-E7-CUDC-907-HDAC1/2/3/10, PI3Kalpha inhibitor	1	HDAC	0.549866	0
612	7-MHB-E9-Givinostat-HDAC inhibitor	1	HDAC	0.227804	0.04
613	7-MHB-E12-Rocilinostat-HDAC-6 selective inhibitor	10	HDAC	0.421394	0
614	7-MHB-F5-Mocetinostat-HDAC inhibitor (HDAC1 & 2-selective)	1	HDAC	0.497109	0
615	7-MHB-F7-Resminostat-HDAC1, 3, 6 inhibitor	10000	HDAC	0.500801	0
616	7-MHB-F9-Givinostat-HDAC inhibitor	0.1	HDAC	0.492371	0
617	7-MHB-F12-Rocilinostat-HDAC-6 selective inhibitor	1	HDAC	0.440945	0
618	7-MHB-F19-PCI-34051-HDAC8 inhibitor	10000	HDAC	0.404861	0.007
619	7-MHB-G7-Resminostat-HDAC1, 3, 6 inhibitor	1000	HDAC	0.53165	0
620	7-MHB-G19-PCI-34051-HDAC8 inhibitor	1000	HDAC	0.093513	0.689
621	7-MHB-H7-Resminostat-HDAC1, 3, 6 inhibitor	100	HDAC	0.583315	0
622	7-MHB-I7-Resminostat-HDAC1, 3, 6 inhibitor	10	HDAC	0.452979	0
623	7-MHB-I19-PCI-34051-HDAC8 inhibitor	100	HDAC	0.230719	0.17
624	7-MHB-J7-Resminostat-HDAC1, 3, 6 inhibitor	1	HDAC	0.075667	0.937
625	7-MHB-J19-PCI-34051-HDAC8 inhibitor	10	HDAC	0.380972	0.005
626	7-MHB-K4-Entinostat-HDAC inhibitor	1	HDAC	0.232189	0.645
627	7-MHB-K11-AR-42-HDAC inhibitor	1	HDAC	0.337529	0.051
628	7-MHB-K18-Tubacin-HDAC6 inhibitor	1	HDAC	0.464116	0

629	7-MHB-K19-PCI-34051-HDAC8 inhibitor	1	HDAC	0.111946	0.487
630	7-MHB-L2-Tacedinaline-HDAC inhibitor	0.1	HDAC	0.081169	0.953
631	7-MHB-L4-Entinostat-HDAC inhibitor	10	HDAC	0.163605	0.975
632	7-MHB-L5-Pracinostat-HDAC inhibitor	1	HDAC	0.370607	0.001
633	7-MHB-L8-Abexinostat-HDAC1-selective inhibitor	1	HDAC	0.47964	0
634	7-MHB-L10-Tucidinostat-HDAC1/2/3/10 inhibitor	1	HDAC	0.47194	0
635	7-MHB-L11-AR-42-HDAC inhibitor	10	HDAC	0.480181	0
636	7-MHB-L14-Tubastatin A-HDAC6 inhibitor	1	HDAC	0.528429	0
637	7-MHB-L16-RGFP966-HDAC3 inhibitor	1	HDAC	0.486048	0
638	7-MHB-L18-Tubacin-HDAC6 inhibitor	10	HDAC	0.081141	0.553
639	7-MHB-M2-Tacedinaline-HDAC inhibitor	1	HDAC	0.234098	0.628
640	7-MHB-M5-Pracinostat-HDAC inhibitor	10	HDAC	0.312947	0.218
641	7-MHB-M8-Abexinostat-HDAC1-selective inhibitor	10	HDAC	0.462298	0
642	7-MHB-M10-Tucidinostat-HDAC1/2/3/10 inhibitor	10	HDAC	0.41774	0
643	7-MHB-M11-AR-42-HDAC inhibitor	100	HDAC	0.565374	0
644	7-MHB-M14-Tubastatin A-HDAC6 inhibitor	10	HDAC	0.564242	0
645	7-MHB-M16-RGFP966-HDAC3 inhibitor	10	HDAC	0.543162	0
646	7-MHB-M18-Tubacin-HDAC6 inhibitor	100	HDAC	0.500993	0
647	7-MHB-N2-Tacedinaline-HDAC inhibitor	10	HDAC	0.099337	0.987
648	7-MHB-N4-Entinostat-HDAC inhibitor	100	HDAC	0.514464	0
649	7-MHB-N5-Pracinostat-HDAC inhibitor	100	HDAC	0.587419	0
650	7-MHB-N8-Abexinostat-HDAC1-selective inhibitor	100	HDAC	0.591598	0
651	7-MHB-N10-Tucidinostat-HDAC1/2/3/10 inhibitor	100	HDAC	0.602665	0
652	7-MHB-N14-Tubastatin A-HDAC6 inhibitor	100	HDAC	0.503643	0
653	7-MHB-N16-RGFP966-HDAC3 inhibitor	100	HDAC	0.520095	0
654	7-MHB-N18-Tubacin-HDAC6 inhibitor	1000	HDAC	0.4607	0
655	7-MHB-O2-Tacedinaline-HDAC inhibitor	100	HDAC	0.051035	0.953
656	7-MHB-O4-Entinostat-HDAC inhibitor	1000	HDAC	0.534333	0
657	7-MHB-O5-Pracinostat-HDAC inhibitor	1000	HDAC	0.519107	0
658	7-MHB-O8-Abexinostat-HDAC1-selective inhibitor	1000	HDAC	0.514547	0
659	7-MHB-O10-Tucidinostat-HDAC1/2/3/10 inhibitor	1000	HDAC	0.545711	0
660	7-MHB-O11-AR-42-HDAC inhibitor	1000	HDAC	0.463149	0
661	7-MHB-O14-Tubastatin A-HDAC6 inhibitor	1000	HDAC	0.307164	0.002

662	7-MHB-O16-RGFP966-HDAC3 inhibitor	1000	HDAC	0.543427	0
663	7-MHB-P2-Tacedinaline-HDAC inhibitor	1000	HDAC	0.553957	0
664	7-MHB-P4-Entinostat-HDAC inhibitor	10000	HDAC	0.541164	0
665	7-MHB-P5-Pracinostat-HDAC inhibitor	10000	HDAC	0.423925	0.006
666	7-MHB-P8-Abexinostat-HDAC1-selective inhibitor	10000	HDAC	0.529519	0
667	7-MHB-P10-Tucidinostat-HDAC1/2/3/10 inhibitor	10000	HDAC	0.491295	0
668	7-MHB-P11-AR-42-HDAC inhibitor	10000	HDAC	0.497566	0
669	7-MHB-P14-Tubastatin A-HDAC6 inhibitor	10000	HDAC	0.456541	0.001
670	7-MHB-P16-RGFP966-HDAC3 inhibitor	10000	HDAC	0.556934	0
671	7-MHB-P18-Tubacin-HDAC6 inhibitor	10000	HDAC	0.51336	0
0	2-O3B-A16-Gefitinib-EGFR inhibitor	10000	EGFR	0.64988	0
1	2-O3B-A19-Erlotinib-EGFR inhibitor	10000	EGFR	0.621798	0
2	2-O3B-B19-Erlotinib-EGFR inhibitor	1000	EGFR	0.646722	0
3	2-O3B-C16-Gefitinib-EGFR inhibitor	1000	EGFR	0.681161	0
4	2-O3B-C19-Erlotinib-EGFR inhibitor	100	EGFR	0.696818	0
5	2-O3B-D16-Gefitinib-EGFR inhibitor	100	EGFR	0.677941	0
6	2-O3B-D19-Erlotinib-EGFR inhibitor	10	EGFR	0.314779	0.353
7	2-O3B-E16-Gefitinib-EGFR inhibitor	10	EGFR	0.643187	0
8	2-O3B-E19-Erlotinib-EGFR inhibitor	1	EGFR	0.313896	0.234
9	2-O3B-F16-Gefitinib-EGFR inhibitor	1	EGFR	0.539322	0
10	2-O3B-K11-Afatinib-EGFR inhibitor	0.1	EGFR	0.401061	0
11	2-O3B-L11-Afatinib-EGFR inhibitor	1	EGFR	0.480269	0.002
12	2-O3B-L16-Osimertinib-EGFR(L858R/T790M) inhibitor	0.25	EGFR	0.29174	0.312
13	2-O3B-L19-Lapatinib-HER2, EGFR inhibitor	0.1	EGFR	0.524403	0
14	2-O3B-M11-Afatinib-EGFR inhibitor	10	EGFR	0.709799	0
15	2-O3B-M16-Osimertinib-EGFR(L858R/T790M) inhibitor	2.5	EGFR	0.670498	0
16	2-O3B-M19-Lapatinib-HER2, EGFR inhibitor	1	EGFR	0.55564	0
17	2-O3B-N16-Osimertinib-EGFR(L858R/T790M) inhibitor	25	EGFR	0.471501	0
18	2-O3B-N19-Lapatinib-HER2, EGFR inhibitor	10	EGFR	0.505793	0
19	2-O3B-O11-Afatinib-EGFR inhibitor	100	EGFR	0.709635	0
20	2-O3B-O16-Osimertinib-EGFR(L858R/T790M) inhibitor	250	EGFR	0.650748	0
21	2-O3B-O19-Lapatinib-HER2, EGFR inhibitor	100	EGFR	0.456518	0
22	2-O3B-P11-Afatinib-EGFR inhibitor	1000	EGFR	0.606772	0

23	2-O3B-P16-Osimertinib-EGFR(L858R/T790M) inhibitor	2500	EGFR	0.626197	0
24	2-O3B-P19-Lapatinib-HER2, EGFR inhibitor	1000	EGFR	0.645162	0
25	3-O3B-F21-Rociletinib-EGFR(L858R/T790M) inhibitor	10000	EGFR	0.62216	0
26	3-O3B-G20-Neratinib-EGFR inhibitor	1000	EGFR	0.595282	0
27	3-O3B-G21-Rociletinib-EGFR(L858R/T790M) inhibitor	1000	EGFR	0.531532	0
28	3-O3B-H20-Neratinib-EGFR inhibitor	100	EGFR	0.656817	0
29	3-O3B-H21-Rociletinib-EGFR(L858R/T790M) inhibitor	100	EGFR	0.304878	0.311
30	3-O3B-I20-Neratinib-EGFR inhibitor	10	EGFR	0.526513	0
31	3-O3B-I21-Rociletinib-EGFR(L858R/T790M) inhibitor	10	EGFR	0.486793	0
32	3-O3B-J20-Neratinib-EGFR inhibitor	1	EGFR	0.298966	0.667
33	3-O3B-J21-Rociletinib-EGFR(L858R/T790M) inhibitor	1	EGFR	0.034505	1
34	3-O3B-K4-Canertinib-pan-HER inhibitor	1	EGFR	0.094446	0.991
35	3-O3B-K18-Dacomitinib-pan-HER inhibitor	0.1	EGFR	0.646858	0
36	3-O3B-K20-Neratinib-EGFR inhibitor	0.1	EGFR	0.431303	0.002
37	3-O3B-L4-Canertinib-pan-HER inhibitor	10	EGFR	0.47411	0.001
38	3-O3B-L18-Dacomitinib-pan-HER inhibitor	1	EGFR	0.092287	0.958
39	3-O3B-M18-Dacomitinib-pan-HER inhibitor	10	EGFR	0.693724	0
40	3-O3B-N4-Canertinib-pan-HER inhibitor	100	EGFR	0.645487	0
41	3-O3B-N18-Dacomitinib-pan-HER inhibitor	100	EGFR	0.654817	0
42	3-O3B-O4-Canertinib-pan-HER inhibitor	1000	EGFR	0.651533	0
43	3-O3B-P4-Canertinib-pan-HER inhibitor	10000	EGFR	0.540313	0.001
44	3-O3B-P18-Dacomitinib-pan-HER inhibitor	1000	EGFR	0.643355	0
45	4-O3B-F13-Sapitinib-Pan-HER inhibitor	1000	EGFR	0.659097	0
46	4-O3B-G13-Sapitinib-Pan-HER inhibitor	100	EGFR	0.652738	0
47	4-O3B-G16-Varlitinib-EGFR HER2 inhibitor	10000	EGFR	0.599437	0
48	4-O3B-H13-Sapitinib-Pan-HER inhibitor	10	EGFR	0.528662	0
49	4-O3B-H16-Varlitinib-EGFR HER2 inhibitor	1000	EGFR	0.649295	0
50	4-O3B-I13-Sapitinib-Pan-HER inhibitor	1	EGFR	0.568601	0
51	4-O3B-I16-Varlitinib-EGFR HER2 inhibitor	100	EGFR	0.57479	0
52	4-O3B-J13-Sapitinib-Pan-HER inhibitor	0.1	EGFR	0.483336	0
53	4-O3B-J16-Varlitinib-EGFR HER2 inhibitor	10	EGFR	0.122171	0.705
54	4-O3B-K7-Icotinib-EGFR inhibitor	1	EGFR	0.432455	0.132
55	4-O3B-K13-Tesevatinib-EGFR, ERBB2, VEGFR, EPHB4	0.1	EGFR	0.476859	0

**56**	4-O3B-K16-Varlitinib-EGFR HER2 inhibitor	1	EGFR	0.577374	0
**57**	4-O3B-L7-Icotinib-EGFR inhibitor	10	EGFR	0.533397	0
**58**	4-O3B-L13-Tesevatinib-EGFR, ERBB2, VEGFR, EPHB4	1	EGFR	0.480345	0
**59**	4-O3B-M7-Icotinib-EGFR inhibitor	100	EGFR	0.600502	0
**60**	4-O3B-M13-Tesevatinib-EGFR, ERBB2, VEGFR, EPHB4	10	EGFR	0.674373	0
**61**	4-O3B-N13-Tesevatinib-EGFR, ERBB2, VEGFR, EPHB4	100	EGFR	0.630752	0
**62**	4-O3B-O7-Icotinib-EGFR inhibitor	1000	EGFR	0.639879	0
**63**	4-O3B-P7-Icotinib-EGFR inhibitor	10000	EGFR	0.624257	0
**64**	4-O3B-P13-Tesevatinib-EGFR, ERBB2, VEGFR, EPHB4	1000	EGFR	0.579439	0
**65**	5-O3B-F4-Poziotinib-pan-HER inhibitor	1000	EGFR	0.624983	0
**66**	5-O3B-F7-AZD3759-EGFR inhibitor, BBB penetrable	1000	EGFR	0.64826	0
**67**	5-O3B-G4-Poziotinib-pan-HER inhibitor	100	EGFR	0.682245	0
**68**	5-O3B-G7-AZD3759-EGFR inhibitor, BBB penetrable	100	EGFR	0.317644	0.193
**69**	5-O3B-H4-Poziotinib-pan-HER inhibitor	10	EGFR	0.613519	0
**70**	5-O3B-H7-AZD3759-EGFR inhibitor, BBB penetrable	10	EGFR	0.126499	0.969
**71**	5-O3B-I4-Poziotinib-pan-HER inhibitor	1	EGFR	0.445221	0.001
**72**	5-O3B-I7-AZD3759-EGFR inhibitor, BBB penetrable	1	EGFR	0.234064	0.863
**73**	5-O3B-J4-Poziotinib-pan-HER inhibitor	0.1	EGFR	0.302692	0.326
**74**	5-O3B-J7-AZD3759-EGFR inhibitor, BBB penetrable	0.1	EGFR	0.216547	0.858
**75**	5-O3B-K7-Olmutinib-EGFR(L858R/T790M) inhibitor	0.1	EGFR	0.28804	0.623
**76**	5-O3B-L7-Olmutinib-EGFR(L858R/T790M) inhibitor	1	EGFR	0.293249	0.53
**77**	5-O3B-M7-Olmutinib-EGFR(L858R/T790M) inhibitor	10	EGFR	0.22652	0.905
**78**	5-O3B-O7-Olmutinib-EGFR(L858R/T790M) inhibitor	100	EGFR	0.140156	0.997
**79**	5-O3B-P7-Olmutinib-EGFR(L858R/T790M) inhibitor	1000	EGFR	0.59299	0
**80**	2-O3B-A15-Lenvatinib-VEGFR inhibitor	2500	VEGFR	0.239064	0.575
**81**	2-O3B-A17-Nintedanib-VEGFR, PDGFR, FGFR inhibitor	10000	VEGFR	0.399103	0.062
**82**	2-O3B-A20-Tivozanib-VEGFR1, 2, 3, c-Kit, PDGFRB inhibitor	10000	VEGFR	0.222074	0.88
**83**	2-O3B-B15-Lenvatinib-VEGFR inhibitor	250	VEGFR	0.357192	0
**84**	2-O3B-B17-Nintedanib-VEGFR, PDGFR, FGFR inhibitor	1000	VEGFR	0.419773	0.012
**85**	2-O3B-B20-Tivozanib-VEGFR1, 2, 3, c-Kit, PDGFRB inhibitor	1000	VEGFR	0.473781	0
**86**	2-O3B-C15-Lenvatinib-VEGFR inhibitor	25	VEGFR	0.25214	0.079
**87**	2-O3B-C17-Nintedanib-VEGFR, PDGFR, FGFR inhibitor	100	VEGFR	0.563394	0
**88**	2-O3B-D15-Lenvatinib-VEGFR inhibitor	2.5	VEGFR	0.251889	0.128

**89**	2-O3B-D17-Nintedanib-VEGFR, PDGFR, FGFR inhibitor	10	VEGFR	0.380755	0.004
**90**	2-O3B-D20-Tivozanib-VEGFR1, 2, 3, c-Kit, PDGFRB inhibitor	100	VEGFR	0.277287	0.003
**91**	2-O3B-E17-Nintedanib-VEGFR, PDGFR, FGFR inhibitor	1	VEGFR	0.279428	0.375
**92**	2-O3B-E20-Tivozanib-VEGFR1, 2, 3, c-Kit, PDGFRB inhibitor	10	VEGFR	0.324006	0.197
**93**	2-O3B-F13-Axitinib-VEGFR, PDGFR, KIT inhibitor	10000	VEGFR	0.143227	0.995
**94**	2-O3B-F15-Lenvatinib-VEGFR inhibitor	0.25	VEGFR	0.373928	0
**95**	2-O3B-F19-Regorafenib-B-Raf, c-Kit, VEGFR2 inhibitor	10000	VEGFR	0.24197	0.902
**96**	2-O3B-F20-Tivozanib-VEGFR1, 2, 3, c-Kit, PDGFRB inhibitor	1	VEGFR	0.32838	0.008
**97**	2-O3B-F21-Vatalanib-VEGFR-1 & -2 inhibitor	10000	VEGFR	0.294027	0.419
**98**	2-O3B-G10-Apatinib-VEGFR inhibitor	10000	VEGFR	0.306682	0.387
**99**	2-O3B-G13-Axitinib-VEGFR, PDGFR, KIT inhibitor	1000	VEGFR	0.114462	0.991
**100**	2-O3B-G19-Regorafenib-B-Raf, c-Kit, VEGFR2 inhibitor	1000	VEGFR	0.260402	0.541
**101**	2-O3B-G21-Vatalanib-VEGFR-1 & -2 inhibitor	1000	VEGFR	0.439209	0.001
**102**	2-O3B-H10-Apatinib-VEGFR inhibitor	1000	VEGFR	0.370833	0.021
**103**	2-O3B-H13-Axitinib-VEGFR, PDGFR, KIT inhibitor	100	VEGFR	0.327793	0.24
**104**	2-O3B-H21-Vatalanib-VEGFR-1 & -2 inhibitor	100	VEGFR	0.410818	0.001
**105**	2-O3B-I10-Apatinib-VEGFR inhibitor	100	VEGFR	0.405324	0.023
**106**	2-O3B-I13-Axitinib-VEGFR, PDGFR, KIT inhibitor	10	VEGFR	0.428811	0.011
**107**	2-O3B-I19-Regorafenib-B-Raf, c-Kit, VEGFR2 inhibitor	100	VEGFR	0.288039	0.393
**108**	2-O3B-I21-Vatalanib-VEGFR-1 & -2 inhibitor	10	VEGFR	0.384744	0.016
**109**	2-O3B-J10-Apatinib-VEGFR inhibitor	10	VEGFR	0.511873	0
**110**	2-O3B-J13-Axitinib-VEGFR, PDGFR, KIT inhibitor	1	VEGFR	0.370077	0.007
**111**	2-O3B-J19-Regorafenib-B-Raf, c-Kit, VEGFR2 inhibitor	10	VEGFR	0.23343	0.724
**112**	2-O3B-J21-Vatalanib-VEGFR-1 & -2 inhibitor	1	VEGFR	0.425254	0.001
**113**	2-O3B-K10-Apatinib-VEGFR inhibitor	1	VEGFR	0.418428	0
**114**	2-O3B-K13-Vandetanib-VEGFR,EGFR, RET inhibitor	0.1	VEGFR	0.370995	0.044
**115**	2-O3B-K17-Pazopanib-VEGFR inhibitor	1	VEGFR	0.317652	0.03
**116**	2-O3B-K19-Regorafenib-B-Raf, c-Kit, VEGFR2 inhibitor	1	VEGFR	0.326692	0.189
**117**	2-O3B-L12-Sorafenib-B-Raf, FGFR-1, VEGFR-2 & -3, PDGFR-beta, KIT, and FLT3 inhib	0.1	VEGFR	0.530846	0
**118**	2-O3B-L13-Vandetanib-VEGFR,EGFR, RET inhibitor	1	VEGFR	0.28196	0.365
**119**	2-O3B-L21-Cediranib-KDR/Flt/VEGFR inhibitor	0.1	VEGFR	0.346171	0.096
**120**	2-O3B-M12-Sorafenib-B-Raf, FGFR-1, VEGFR-2 & -3, PDGFR-beta, KIT, and FLT3 inhib	1	VEGFR	0.306976	0.204
**121**	2-O3B-M13-Vandetanib-VEGFR,EGFR, RET inhibitor	10	VEGFR	0.386361	0.042

122	2-O3B-M17-Pazopanib-VEGFR inhibitor	10	VEGFR	0.421779	0.004
123	2-O3B-M21-Cediranib-KDR/Flt/VEGFR inhibitor	1	VEGFR	0.184211	0.944
124	2-O3B-N12-Sorafenib-B-Raf, FGFR-1, VEGFR-2 & -3, PDGFR-beta, KIT, and FLT3 inhib	10	VEGFR	0.256714	0.053
125	2-O3B-N13-Vandetanib-VEGFR,EGFR, RET inhibitor	100	VEGFR	0.365866	0.097
126	2-O3B-N17-Pazopanib-VEGFR inhibitor	100	VEGFR	0.180915	0.298
127	2-O3B-N21-Cediranib-KDR/Flt/VEGFR inhibitor	10	VEGFR	0.259041	0.056
128	2-O3B-O12-Sorafenib-B-Raf, FGFR-1, VEGFR-2 & -3, PDGFR-beta, KIT, and FLT3 inhib	100	VEGFR	0.244458	0.196
129	2-O3B-O17-Pazopanib-VEGFR inhibitor	1000	VEGFR	0.424845	0.003
130	2-O3B-O21-Cediranib-KDR/Flt/VEGFR inhibitor	100	VEGFR	0.425822	0.002
131	2-O3B-P12-Sorafenib-B-Raf, FGFR-1, VEGFR-2 & -3, PDGFR-beta, KIT, and FLT3 inhib	1000	VEGFR	0.293727	0.049
132	2-O3B-P13-Vandetanib-VEGFR,EGFR, RET inhibitor	1000	VEGFR	0.374533	0.102
133	2-O3B-P17-Pazopanib-VEGFR inhibitor	10000	VEGFR	0.419907	0
134	2-O3B-P21-Cediranib-KDR/Flt/VEGFR inhibitor	1000	VEGFR	0.28228	0.369
135	3-O3B-A3-Cabozantinib-VEGFR2, Met, FLT3, Tie2, Kit and Ret inhibitor	1000	VEGFR	0.387637	0.007
136	3-O3B-A6-Foretinib-MET, VEGFR2 inhibitor	1000	VEGFR	0.161761	0.986
137	3-O3B-A18-Linifanib-VEGFR, PDGFR, CSF-1R, FLT3 inhibitor	1000	VEGFR	0.341618	0.15
138	3-O3B-B3-Cabozantinib-VEGFR2, Met, FLT3, Tie2, Kit and Ret inhibitor	100	VEGFR	0.27531	0.241
139	3-O3B-B6-Foretinib-MET, VEGFR2 inhibitor	100	VEGFR	0.19441	0.049
140	3-O3B-B18-Linifanib-VEGFR, PDGFR, CSF-1R, FLT3 inhibitor	100	VEGFR	0.306086	0.218
141	3-O3B-C3-Cabozantinib-VEGFR2, Met, FLT3, Tie2, Kit and Ret inhibitor	10	VEGFR	0.274616	0.506
142	3-O3B-C6-Foretinib-MET, VEGFR2 inhibitor	10	VEGFR	0.521115	0
143	3-O3B-C18-Linifanib-VEGFR, PDGFR, CSF-1R, FLT3 inhibitor	10	VEGFR	0.36903	0
144	3-O3B-D3-Cabozantinib-VEGFR2, Met, FLT3, Tie2, Kit and Ret inhibitor	1	VEGFR	0.238277	0.491
145	3-O3B-D6-Foretinib-MET, VEGFR2 inhibitor	1	VEGFR	0.164227	0.113
146	3-O3B-D18-Linifanib-VEGFR, PDGFR, CSF-1R, FLT3 inhibitor	1	VEGFR	0.21499	0.043
147	3-O3B-E3-Cabozantinib-VEGFR2, Met, FLT3, Tie2, Kit and Ret inhibitor	0.1	VEGFR	0.232291	0.325
148	3-O3B-E6-Foretinib-MET, VEGFR2 inhibitor	0.1	VEGFR	0.246729	0.264
149	3-O3B-E18-Linifanib-VEGFR, PDGFR, CSF-1R, FLT3 inhibitor	0.1	VEGFR	0.185502	0.97
150	3-O3B-F18-Brivanib-VEGFR inhibitor	1000	VEGFR	0.269694	0.578
151	3-O3B-G18-Brivanib-VEGFR inhibitor	100	VEGFR	0.206457	0.926
152	3-O3B-H18-Brivanib-VEGFR inhibitor	10	VEGFR	0.197917	0.047
153	3-O3B-I18-Brivanib-VEGFR inhibitor	1	VEGFR	0.313157	0.454
154	3-O3B-J18-Brivanib-VEGFR inhibitor	0.1	VEGFR	0.171123	0.978

155	4-O3B-A12-ENMD-2076-pan-Aurora, VEGFR inhibitor	10000	VEGFR	0.205053	0.994
156	4-O3B-A15-Golvatinib-MET, VEGFR2 inhibitor	2500	VEGFR	0.273937	0.226
157	4-O3B-A20-Motesanib-VEGFR, PDGFR, Ret, Kit inhibitor	10000	VEGFR	0.191514	0.752
158	4-O3B-B12-ENMD-2076-pan-Aurora, VEGFR inhibitor	1000	VEGFR	0.215122	0.898
159	4-O3B-B15-Golvatinib-MET, VEGFR2 inhibitor	250	VEGFR	0.194409	0.223
160	4-O3B-B20-Motesanib-VEGFR, PDGFR, Ret, Kit inhibitor	1000	VEGFR	0.196607	0.422
161	4-O3B-C15-Golvatinib-MET, VEGFR2 inhibitor	25	VEGFR	0.233692	0.051
162	4-O3B-D12-ENMD-2076-pan-Aurora, VEGFR inhibitor	100	VEGFR	0.270026	0.999
163	4-O3B-D15-Golvatinib-MET, VEGFR2 inhibitor	2.5	VEGFR	0.187443	0.979
164	4-O3B-D20-Motesanib-VEGFR, PDGFR, Ret, Kit inhibitor	100	VEGFR	0.195427	1
165	4-O3B-E12-ENMD-2076-pan-Aurora, VEGFR inhibitor	10	VEGFR	0.229637	0.803
166	4-O3B-E20-Motesanib-VEGFR, PDGFR, Ret, Kit inhibitor	10	VEGFR	0.151484	0.999
167	4-O3B-F12-ENMD-2076-pan-Aurora, VEGFR inhibitor	1	VEGFR	0.277857	0.99
168	4-O3B-F15-Golvatinib-MET, VEGFR2 inhibitor	0.25	VEGFR	0.202807	0.991
169	4-O3B-F20-Motesanib-VEGFR, PDGFR, Ret, Kit inhibitor	1	VEGFR	0.214349	0.803
170	4-O3B-L16-Telatinib-VEGFR, KIT, PDGFR inhibitor	1	VEGFR	0.290826	0.613
171	4-O3B-M16-Telatinib-VEGFR, KIT, PDGFR inhibitor	10	VEGFR	0.204796	1
172	4-O3B-N16-Telatinib-VEGFR, KIT, PDGFR inhibitor	100	VEGFR	0.324436	0.998
173	4-O3B-O16-Telatinib-VEGFR, KIT, PDGFR inhibitor	1000	VEGFR	0.291573	0.859
174	4-O3B-P16-Telatinib-VEGFR, KIT, PDGFR inhibitor	10000	VEGFR	0.452545	0.004
175	2-O3B-L10-Idelalisib-PI3K inhibitor, p110δ-selective	1	PI3K	0.027904	1
176	2-O3B-M10-Idelalisib-PI3K inhibitor, p110δ-selective	10	PI3K	0.252975	0.34
177	2-O3B-N10-Idelalisib-PI3K inhibitor, p110δ-selective	100	PI3K	0.392773	0.026
178	2-O3B-O10-Idelalisib-PI3K inhibitor, p110δ-selective	1000	PI3K	0.394357	0.025
179	2-O3B-P10-Idelalisib-PI3K inhibitor, p110δ-selective	10000	PI3K	0.47279	0
180	3-O3B-A16-Perifosine-AKT/PI3K inhibitor	2500	PI3K	0.069524	0.913
181	3-O3B-C16-Perifosine-AKT/PI3K inhibitor	250	PI3K	0.333321	0.03
182	3-O3B-D16-Perifosine-AKT/PI3K inhibitor	25	PI3K	0.03843	0.959
183	3-O3B-E16-Perifosine-AKT/PI3K inhibitor	2.5	PI3K	0.169684	0.369
184	3-O3B-F16-Perifosine-AKT/PI3K inhibitor	0.25	PI3K	0.264165	0.37
185	3-O3B-F17-Miltefosine-Antimicrobial, inhibits PI3K/AKT	100000	PI3K	0.483185	0.001
186	3-O3B-F19-Duvelisib-PI3K inhibitor	500	PI3K	0.40699	0.002
187	3-O3B-G17-Miltefosine-Antimicrobial, inhibits PI3K/AKT	10000	PI3K	0.12426	0.811

188	3-O3B-G19-Duvelisib-PI3K inhibitor	50	PI3K	0.401614	0.002
189	3-O3B-H17-Miltefosine-Antimicrobial, inhibits PI3K/AKT	1000	PI3K	0.078532	0.774
190	3-O3B-I17-Miltefosine-Antimicrobial, inhibits PI3K/AKT	100	PI3K	0.155225	0.73
191	3-O3B-I19-Duvelisib-PI3K inhibitor	5	PI3K	0.379305	0.008
192	3-O3B-J17-Miltefosine-Antimicrobial, inhibits PI3K/AKT	10	PI3K	0.151772	0.924
193	3-O3B-J19-Duvelisib-PI3K inhibitor	0.5	PI3K	0.049368	0.964
194	3-O3B-K19-Duvelisib-PI3K inhibitor	0.05	PI3K	0.31714	0.023
195	3-O3B-L8-Pictilisib-PI3K inhibitor, pan-class I	1	PI3K	0.245189	0.593
196	3-O3B-L21-Taselisib-PI3K alpha, delta, (gamma) selective inhibitor	0.1	PI3K	0.094574	0.928
197	3-O3B-M8-Pictilisib-PI3K inhibitor, pan-class I	10	PI3K	0.400272	0.004
198	3-O3B-M21-Taselisib-PI3K alpha, delta, (gamma) selective inhibitor	1	PI3K	0.406672	0.002
199	3-O3B-N8-Pictilisib-PI3K inhibitor, pan-class I	100	PI3K	0.431082	0
200	3-O3B-N21-Taselisib-PI3K alpha, delta, (gamma) selective inhibitor	10	PI3K	0.208595	0.589
201	3-O3B-O8-Pictilisib-PI3K inhibitor, pan-class I	1000	PI3K	0.448545	0
202	3-O3B-O21-Taselisib-PI3K alpha, delta, (gamma) selective inhibitor	100	PI3K	0.349792	0.019
203	3-O3B-P8-Pictilisib-PI3K inhibitor, pan-class I	10000	PI3K	0.467518	0
204	3-O3B-P21-Taselisib-PI3K alpha, delta, (gamma) selective inhibitor	1000	PI3K	0.449084	0
205	4-O3B-A19-Alpelisib-PI3Kalpha inhibitor	2500	PI3K	0.491334	0
206	4-O3B-B19-Alpelisib-PI3Kalpha inhibitor	250	PI3K	0.385577	0.002
207	4-O3B-C19-Alpelisib-PI3Kalpha inhibitor	25	PI3K	0.278748	0.107
208	4-O3B-D19-Alpelisib-PI3Kalpha inhibitor	2.5	PI3K	0.382582	0.714
209	4-O3B-E19-Alpelisib-PI3Kalpha inhibitor	0.25	PI3K	0.40769	0
210	4-O3B-F14-NVP-BGT226-PI3K/mTOR inhibitor	1000	PI3K	0.473942	0.009
211	4-O3B-G2-TGR-1202-PI3Kdelta inhibitor	2500	PI3K	0.30363	0.373
212	4-O3B-G5-Sonolisib-PI3K inhibitor, pan-class I. Irreversible	10000	PI3K	0.362382	0.904
213	4-O3B-G14-NVP-BGT226-PI3K/mTOR inhibitor	100	PI3K	0.446818	0.012
214	4-O3B-G20-Buparlisib-PI3K inhibitor, pan-class I	10000	PI3K	0.445113	0.034
215	4-O3B-H2-TGR-1202-PI3Kdelta inhibitor	250	PI3K	0.291433	0.999
216	4-O3B-H5-Sonolisib-PI3K inhibitor, pan-class I. Irreversible	1000	PI3K	0.274536	0.215
217	4-O3B-H14-NVP-BGT226-PI3K/mTOR inhibitor	10	PI3K	0.446084	0
218	4-O3B-H20-Buparlisib-PI3K inhibitor, pan-class I	1000	PI3K	0.45644	0
219	4-O3B-I2-TGR-1202-PI3Kdelta inhibitor	25	PI3K	0.317965	0.995
220	4-O3B-I5-Sonolisib-PI3K inhibitor, pan-class I. Irreversible	100	PI3K	0.38967	0.02

221	4-O3B-I14-NVP-BGT226-PI3K/mTOR inhibitor	1	PI3K	0.325508	0.043
222	4-O3B-I20-Buparlisib-PI3K inhibitor, pan-class I	100	PI3K	0.356439	0.976
223	4-O3B-J2-TGR-1202-PI3Kdelta inhibitor	2.5	PI3K	0.105607	0.977
224	4-O3B-J5-Sonolisib-PI3K inhibitor, pan-class I. Irreversible	10	PI3K	0.398242	0.995
225	4-O3B-J20-Buparlisib-PI3K inhibitor, pan-class I	10	PI3K	0.432488	0
226	4-O3B-K2-TGR-1202-PI3Kdelta inhibitor	0.25	PI3K	0.233719	0.756
227	4-O3B-K4-Dactolisib-mTOR/(PI3K) inhibitor	0.1	PI3K	0.406139	0.001
228	4-O3B-K5-Sonolisib-PI3K inhibitor, pan-class I. Irreversible	1	PI3K	0.413996	0.001
229	4-O3B-K14-NVP-BGT226-PI3K/mTOR inhibitor	0.1	PI3K	0.280162	0.999
230	4-O3B-K20-Buparlisib-PI3K inhibitor, pan-class I	1	PI3K	0.322872	0.313
231	4-O3B-L4-Dactolisib-mTOR/(PI3K) inhibitor	1	PI3K	0.427104	0.002
232	4-O3B-L14-Gedatolisib-PI3K/mTOR inhibitor	0.1	PI3K	0.407467	0.37
233	4-O3B-L15-TG100-115-PI3K gamma/delta inhibitor	1	PI3K	0.30557	0.91
234	4-O3B-L21-Copanlisib-PI3K alpha, delta selective inhibitor	0.1	PI3K	0.423875	0.002
235	4-O3B-M14-Gedatolisib-PI3K/mTOR inhibitor	1	PI3K	0.431996	0
236	4-O3B-M15-TG100-115-PI3K gamma/delta inhibitor	10	PI3K	0.495529	0
237	4-O3B-M21-Copanlisib-PI3K alpha, delta selective inhibitor	1	PI3K	0.218116	0.583
238	4-O3B-N4-Dactolisib-mTOR/(PI3K) inhibitor	10	PI3K	0.397564	0.001
239	4-O3B-N14-Gedatolisib-PI3K/mTOR inhibitor	10	PI3K	0.420287	0.007
240	4-O3B-N15-TG100-115-PI3K gamma/delta inhibitor	100	PI3K	0.309598	0.531
241	4-O3B-N21-Copanlisib-PI3K alpha, delta selective inhibitor	10	PI3K	0.410782	0.237
242	4-O3B-O4-Dactolisib-mTOR/(PI3K) inhibitor	100	PI3K	0.447763	0.005
243	4-O3B-O14-Gedatolisib-PI3K/mTOR inhibitor	100	PI3K	0.444635	0.015
244	4-O3B-O15-TG100-115-PI3K gamma/delta inhibitor	1000	PI3K	0.511559	0
245	4-O3B-O21-Copanlisib-PI3K alpha, delta selective inhibitor	100	PI3K	0.474134	0
246	4-O3B-P4-Dactolisib-mTOR/(PI3K) inhibitor	1000	PI3K	0.45119	0
247	4-O3B-P14-Gedatolisib-PI3K/mTOR inhibitor	1000	PI3K	0.449723	0.007
248	4-O3B-P15-TG100-115-PI3K gamma/delta inhibitor	10000	PI3K	0.465733	0
249	4-O3B-P21-Copanlisib-PI3K alpha, delta selective inhibitor	1000	PI3K	0.446592	0.019
250	5-O3B-A6-LY3023414-PI3K/mTOR/DNA-PK inhibitor	2500	PI3K	0.442492	0.007
251	5-O3B-A7-AMG319-PI3Kdelta inhibitor	1000	PI3K	0.382675	0.005
252	5-O3B-A16-AZD-6482-PI3Kbeta-selective inhibitor	2500	PI3K	0.408541	0.002
253	5-O3B-A17-Palomid-529-AKT, MTOR, PI3K inhibitor	10000	PI3K	0.371349	0.004

254	5-O3B-B6-LY3023414-PI3K/mTOR/DNA-PK inhibitor	250	PI3K	0.437463	0
255	5-O3B-B7-AMG319-PI3Kdelta inhibitor	100	PI3K	0.367141	0.024
256	5-O3B-B17-Palomid-529-AKT, MTOR, PI3K inhibitor	1000	PI3K	0.362519	0.009
257	5-O3B-C6-LY3023414-PI3K/mTOR/DNA-PK inhibitor	25	PI3K	0.333846	0.154
258	5-O3B-C7-AMG319-PI3Kdelta inhibitor	10	PI3K	0.376533	0.003
259	5-O3B-C16-AZD-6482-PI3Kbeta-selective inhibitor	250	PI3K	0.361945	0.016
260	5-O3B-C17-Palomid-529-AKT, MTOR, PI3K inhibitor	100	PI3K	0.278737	0.424
261	5-O3B-D6-LY3023414-PI3K/mTOR/DNA-PK inhibitor	2.5	PI3K	0.124965	0.785
262	5-O3B-D7-AMG319-PI3Kdelta inhibitor	1	PI3K	0.314378	0.163
263	5-O3B-D16-AZD-6482-PI3Kbeta-selective inhibitor	25	PI3K	0.436903	0
264	5-O3B-D17-Palomid-529-AKT, MTOR, PI3K inhibitor	10	PI3K	0.407584	0
265	5-O3B-E6-LY3023414-PI3K/mTOR/DNA-PK inhibitor	0.25	PI3K	0.276701	0.385
266	5-O3B-E7-AMG319-PI3Kdelta inhibitor	0.1	PI3K	0.386895	0.005
267	5-O3B-E16-AZD-6482-PI3Kbeta-selective inhibitor	2.5	PI3K	0.383649	0.005
268	5-O3B-E17-Palomid-529-AKT, MTOR, PI3K inhibitor	1	PI3K	0.382798	0.003
269	5-O3B-F11-GSK2636771-PI3K beta selective inhibitor	10000	PI3K	0.321935	0.018
270	5-O3B-F16-AZD-6482-PI3Kbeta-selective inhibitor	0.25	PI3K	0.337699	0.023
271	5-O3B-G9-Serabelisib-PI3Kalpha selective inhibitor	10000	PI3K	0.450186	0
272	5-O3B-G11-GSK2636771-PI3K beta selective inhibitor	1000	PI3K	0.171096	0.372
273	5-O3B-H9-Serabelisib-PI3Kalpha selective inhibitor	1000	PI3K	0.367404	0.052
274	5-O3B-H11-GSK2636771-PI3K beta selective inhibitor	100	PI3K	0.315652	0.159
275	5-O3B-I9-Serabelisib-PI3Kalpha selective inhibitor	100	PI3K	0.137551	0.692
276	5-O3B-I11-GSK2636771-PI3K beta selective inhibitor	10	PI3K	0.343769	0.003
277	5-O3B-J9-Serabelisib-PI3Kalpha selective inhibitor	10	PI3K	0.349907	0.083
278	5-O3B-J11-GSK2636771-PI3K beta selective inhibitor	1	PI3K	0.3095	0.006
279	5-O3B-K9-Serabelisib-PI3Kalpha selective inhibitor	1	PI3K	0.454444	0
280	5-O3B-L14-AZD-8186-PI3Kbeta inhibitor	0.1	PI3K	0.281128	0.347
281	5-O3B-L20-ZSTK474-PI3K gamma selective inhibitor	1	PI3K	0.388814	0.002
282	5-O3B-L23-Omipalisib-PI3K/mTOR inhibitor	0.1	PI3K	0.310672	0.107
283	5-O3B-M14-AZD-8186-PI3Kbeta inhibitor	1	PI3K	0.401677	0
284	5-O3B-M20-ZSTK474-PI3K gamma selective inhibitor	10	PI3K	0.381277	0.016
285	5-O3B-M23-Omipalisib-PI3K/mTOR inhibitor	1	PI3K	0.434378	0
286	5-O3B-N14-AZD-8186-PI3Kbeta inhibitor	10	PI3K	0.397434	0

287	5-O3B-N20-ZSTK474-PI3K gamma selective inhibitor	100	PI3K	0.496257	0
288	5-O3B-N23-Omipalisib-PI3K/mTOR inhibitor	10	PI3K	0.433013	0
289	5-O3B-O14-AZD-8186-PI3Kbeta inhibitor	100	PI3K	0.473118	0
290	5-O3B-O20-ZSTK474-PI3K gamma selective inhibitor	1000	PI3K	0.446261	0.001
291	5-O3B-O23-Omipalisib-PI3K/mTOR inhibitor	100	PI3K	0.447397	0.001
292	5-O3B-P14-AZD-8186-PI3Kbeta inhibitor	1000	PI3K	0.459166	0
293	5-O3B-P20-ZSTK474-PI3K gamma selective inhibitor	10000	PI3K	0.434319	0.001
294	5-O3B-P23-Omipalisib-PI3K/mTOR inhibitor	1000	PI3K	0.450857	0
295	6-O3B-A8-TGX-221-PI3K beta selective inhibitor	10000	PI3K	0.476792	0
296	6-O3B-B8-TGX-221-PI3K beta selective inhibitor	1000	PI3K	0.458756	0
297	6-O3B-C8-TGX-221-PI3K beta selective inhibitor	100	PI3K	0.417514	0.005
298	6-O3B-D8-TGX-221-PI3K beta selective inhibitor	10	PI3K	0.251993	0.392
299	6-O3B-E8-TGX-221-PI3K beta selective inhibitor	1	PI3K	0.052886	0.971
300	6-O3B-L6-GDC-0084-PI3K/mTOR inhibitor	1	PI3K	0.106207	0.809
301	6-O3B-M6-GDC-0084-PI3K/mTOR inhibitor	10	PI3K	0.058646	0.997
302	6-O3B-N6-GDC-0084-PI3K/mTOR inhibitor	100	PI3K	0.35432	0.051
303	6-O3B-O6-GDC-0084-PI3K/mTOR inhibitor	1000	PI3K	0.483837	0
304	6-O3B-P6-GDC-0084-PI3K/mTOR inhibitor	10000	PI3K	0.472855	0.022
305	1-O3B-F11-Amsacrine-DNA intercalation, Topo II inhibitor	10000	Topoisome	0.571709	0.042
306	1-O3B-G11-Amsacrine-DNA intercalation, Topo II inhibitor	1000	Topoisome	0.620847	0.002
307	1-O3B-G20-Epirubicin-Topoisomerase II inhibitor	1000	Topoisome	0.506913	0.022
308	1-O3B-H11-Amsacrine-DNA intercalation, Topo II inhibitor	100	Topoisome	0.618096	0.004
309	1-O3B-H20-Epirubicin-Topoisomerase II inhibitor	100	Topoisome	0.619448	0.007
310	1-O3B-I11-Amsacrine-DNA intercalation, Topo II inhibitor	10	Topoisome	0.562796	0.086
311	1-O3B-I20-Epirubicin-Topoisomerase II inhibitor	10	Topoisome	0.522615	0.365
312	1-O3B-J11-Amsacrine-DNA intercalation, Topo II inhibitor	1	Topoisome	0.60663	0.14
313	1-O3B-J20-Epirubicin-Topoisomerase II inhibitor	1	Topoisome	0.15355	0.931
314	1-O3B-K11-SN-38-Active metabolite of irinotecan. Topoisomerase I inhibitor	1	Topoisome	0.619699	0.007
315	1-O3B-K20-Epirubicin-Topoisomerase II inhibitor	0.1	Topoisome	0.161478	0.989
316	1-O3B-L11-SN-38-Active metabolite of irinotecan. Topoisomerase I inhibitor	10	Topoisome	0.223013	0.913
317	1-O3B-L14-Topotecan-Topoisomerase I inhibitor. Camptothecin analog	1	Topoisome	0.179001	0.807
318	1-O3B-M11-SN-38-Active metabolite of irinotecan. Topoisomerase I inhibitor	100	Topoisome	0.577859	0.052
319	1-O3B-M14-Topotecan-Topoisomerase I inhibitor. Camptothecin analog	10	Topoisome	0.595425	0.019

320	1-O3B-N14-Topotecan-Topoisomerase I inhibitor. Camptothecin analog	100	Topoisome	0.611021	0.007
321	1-O3B-O11-SN-38-Active metabolite of irinotecan. Topoisomerase I inhibitor	1000	Topoisome	0.410294	0.143
322	1-O3B-O14-Topotecan-Topoisomerase I inhibitor. Camptothecin analog	1000	Topoisome	0.583982	0.039
323	1-O3B-P11-SN-38-Active metabolite of irinotecan. Topoisomerase I inhibitor	10000	Topoisome	0.40367	0.202
324	1-O3B-P14-Topotecan-Topoisomerase I inhibitor. Camptothecin analog	10000	Topoisome	0.406501	0.24
325	3-O3B-A11-Etoposide-Topoisomerase II inhibitor	10000	Topoisome	0.601063	0
326	3-O3B-B11-Etoposide-Topoisomerase II inhibitor	1000	Topoisome	0.632423	0
327	3-O3B-C11-Etoposide-Topoisomerase II inhibitor	100	Topoisome	0.62486	0
328	3-O3B-D11-Etoposide-Topoisomerase II inhibitor	10	Topoisome	0.268899	0.046
329	3-O3B-E11-Etoposide-Topoisomerase II inhibitor	1	Topoisome	0.283619	0.01
330	3-O3B-G9-Daunorubicin-Topoisomerase II inhibitor	1000	Topoisome	0.42456	0.174
331	3-O3B-G10-Teniposide-Topoisomerase II inhibitor	10000	Topoisome	0.207514	0.878
332	3-O3B-H9-Daunorubicin-Topoisomerase II inhibitor	100	Topoisome	0.595187	0
333	3-O3B-H10-Teniposide-Topoisomerase II inhibitor	1000	Topoisome	0.188408	0.234
334	3-O3B-I9-Daunorubicin-Topoisomerase II inhibitor	10	Topoisome	0.358884	0.009
335	3-O3B-I10-Teniposide-Topoisomerase II inhibitor	100	Topoisome	0.418658	0.001
336	3-O3B-J9-Daunorubicin-Topoisomerase II inhibitor	1	Topoisome	0.527372	0
337	3-O3B-J10-Teniposide-Topoisomerase II inhibitor	10	Topoisome	0.418994	0
338	3-O3B-K7-Idarubicin-Topoisomerase II inhibitor	0.1	Topoisome	0.309146	0.773
339	3-O3B-K9-Daunorubicin-Topoisomerase II inhibitor	0.1	Topoisome	0.44223	0
340	3-O3B-K10-Teniposide-Topoisomerase II inhibitor	1	Topoisome	0.332071	0
341	3-O3B-L6-Doxorubicin-Topoisomerase II inhibitor	0.1	Topoisome	0.1262	0.725
342	3-O3B-L7-Idarubicin-Topoisomerase II inhibitor	1	Topoisome	0.266877	0.944
343	3-O3B-L9-Valrubicin-Topoisomerase II inhibitor	0.5	Topoisome	0.530137	0
344	3-O3B-L10-Mitoxantrone-Topoisomerase II inhibitor	0.1	Topoisome	0.177885	0.167
345	3-O3B-L16-Pixantrone-topoisomerase II inhibitor	1	Topoisome	0.2384	0.272
346	3-O3B-M6-Doxorubicin-Topoisomerase II inhibitor	1	Topoisome	0.161852	0.4
347	3-O3B-M7-Idarubicin-Topoisomerase II inhibitor	10	Topoisome	0.561048	0
348	3-O3B-M9-Valrubicin-Topoisomerase II inhibitor	5	Topoisome	0.443695	0
349	3-O3B-M10-Mitoxantrone-Topoisomerase II inhibitor	1	Topoisome	0.57575	0
350	3-O3B-M16-Pixantrone-topoisomerase II inhibitor	10	Topoisome	0.198859	0.958
351	3-O3B-N6-Doxorubicin-Topoisomerase II inhibitor	10	Topoisome	0.489814	0.001
352	3-O3B-N9-Valrubicin-Topoisomerase II inhibitor	50	Topoisome	0.613406	0

353	3-O3B-N10-Mitoxantrone-Topoisomerase II inhibitor	10	Topoisome	0.616015	0
354	3-O3B-N16-Pixantrone-topoisomerase II inhibitor	100	Topoisome	0.356085	0
355	3-O3B-O6-Doxorubicin-Topoisomerase II inhibitor	100	Topoisome	0.622681	0
356	3-O3B-O7-Idarubicin-Topoisomerase II inhibitor	100	Topoisome	0.609792	0
357	3-O3B-O9-Valrubicin-Topoisomerase II inhibitor	500	Topoisome	0.599717	0
358	3-O3B-O10-Mitoxantrone-Topoisomerase II inhibitor	100	Topoisome	0.62123	0
359	3-O3B-O16-Pixantrone-topoisomerase II inhibitor	1000	Topoisome	0.424242	0.038
360	3-O3B-P6-Doxorubicin-Topoisomerase II inhibitor	1000	Topoisome	0.282427	0.745
361	3-O3B-P7-Idarubicin-Topoisomerase II inhibitor	1000	Topoisome	0.357734	0.35
362	3-O3B-P9-Valrubicin-Topoisomerase II inhibitor	5000	Topoisome	0.378995	0.273
363	3-O3B-P10-Mitoxantrone-Topoisomerase II inhibitor	1000	Topoisome	0.383671	0.102
364	3-O3B-P16-Pixantrone-topoisomerase II inhibitor	10000	Topoisome	0.46612	0.006
365	1-O3B-A10-Vinorelbine-Mitotic inhibitor. Vinca alkaloid microtubule depolymerizer	10000	Mitotic	0.801992	0
366	1-O3B-A13-Ixabepilone-Mitotic inhibitor. Epothilone microtubule stabilizer.	1000	Mitotic	0.718799	0
367	1-O3B-A18-Paclitaxel-Mitotic inhibitor, taxane microtubule stabilizer	1000	Mitotic	0.806544	0
368	1-O3B-B10-Vinorelbine-Mitotic inhibitor. Vinca alkaloid microtubule depolymerizer	1000	Mitotic	0.805453	0
369	1-O3B-B13-Ixabepilone-Mitotic inhibitor. Epothilone microtubule stabilizer.	100	Mitotic	0.32786	0.007
370	1-O3B-B18-Paclitaxel-Mitotic inhibitor, taxane microtubule stabilizer	100	Mitotic	0.818154	0
371	1-O3B-C10-Vinorelbine-Mitotic inhibitor. Vinca alkaloid microtubule depolymerizer	100	Mitotic	0.818038	0
372	1-O3B-C13-Ixabepilone-Mitotic inhibitor. Epothilone microtubule stabilizer.	10	Mitotic	0.696187	0
373	1-O3B-C18-Paclitaxel-Mitotic inhibitor, taxane microtubule stabilizer	10	Mitotic	0.710826	0
374	1-O3B-D10-Vinorelbine-Mitotic inhibitor. Vinca alkaloid microtubule depolymerizer	10	Mitotic	0.709948	0
375	1-O3B-D13-Ixabepilone-Mitotic inhibitor. Epothilone microtubule stabilizer.	1	Mitotic	0.573833	0
376	1-O3B-D18-Paclitaxel-Mitotic inhibitor, taxane microtubule stabilizer	1	Mitotic	0.342524	0.503
377	1-O3B-E10-Vinorelbine-Mitotic inhibitor. Vinca alkaloid microtubule depolymerizer	1	Mitotic	0.529265	0.002
378	1-O3B-E13-Ixabepilone-Mitotic inhibitor. Epothilone microtubule stabilizer.	0.1	Mitotic	0.676011	0.005
379	1-O3B-E18-Paclitaxel-Mitotic inhibitor, taxane microtubule stabilizer	0.1	Mitotic	0.368327	0.51
380	1-O3B-F13-Vinblastine-Mitotic inhibitor. Vinca alkaloid microtubule depolymerizer	1000	Mitotic	0.801654	0
381	1-O3B-G13-Vinblastine-Mitotic inhibitor. Vinca alkaloid microtubule depolymerizer	100	Mitotic	0.397714	0.29
382	1-O3B-G15-Eribulin-Mitotic inhibitor, microtubule depolymerizer.	1000	Mitotic	0.810598	0
383	1-O3B-H13-Vinblastine-Mitotic inhibitor. Vinca alkaloid microtubule depolymerizer	10	Mitotic	0.113161	0.978
384	1-O3B-H15-Eribulin-Mitotic inhibitor, microtubule depolymerizer.	100	Mitotic	0.733619	0
385	1-O3B-I13-Vinblastine-Mitotic inhibitor. Vinca alkaloid microtubule depolymerizer	1	Mitotic	0.492148	0.003

386	1-O3B-I15-Eribulin-Mitotic inhibitor, microtubule depolymerizer.	10	Mitotic	0.820771	0
387	1-O3B-J13-Vinblastine-Mitotic inhibitor. Vinca alkaloid microtubule depolymerizer	0.1	Mitotic	0.738123	0.001
388	1-O3B-J15-Eribulin-Mitotic inhibitor, microtubule depolymerizer.	1	Mitotic	0.689284	0
389	1-O3B-K7-Vincristine-Mitotic inhibitor. Vinca alkaloid microtubule depolymerizer	0.1	Mitotic	0.654838	0.006
390	1-O3B-K15-Eribulin-Mitotic inhibitor, microtubule depolymerizer.	0.1	Mitotic	0.379598	0.159
391	1-O3B-L7-Vincristine-Mitotic inhibitor. Vinca alkaloid microtubule depolymerizer	1	Mitotic	0.286684	0.906
392	1-O3B-L20-Vinflunine-Mitotic inhibitor. Vinca alkaloid microtubule depolymerizer	0.1	Mitotic	0.344527	0.288
393	1-O3B-M7-Vincristine-Mitotic inhibitor. Vinca alkaloid microtubule depolymerizer	10	Mitotic	0.416965	0.149
394	1-O3B-M20-Vinflunine-Mitotic inhibitor. Vinca alkaloid microtubule depolymerizer	1	Mitotic	0.718149	0
395	1-O3B-N20-Vinflunine-Mitotic inhibitor. Vinca alkaloid microtubule depolymerizer	10	Mitotic	0.292034	0.266
396	1-O3B-O7-Vincristine-Mitotic inhibitor. Vinca alkaloid microtubule depolymerizer	100	Mitotic	0.629893	0.007
397	1-O3B-O20-Vinflunine-Mitotic inhibitor. Vinca alkaloid microtubule depolymerizer	100	Mitotic	0.254444	0.815
398	1-O3B-P7-Vincristine-Mitotic inhibitor. Vinca alkaloid microtubule depolymerizer	1000	Mitotic	0.695672	0
399	1-O3B-P20-Vinflunine-Mitotic inhibitor. Vinca alkaloid microtubule depolymerizer	1000	Mitotic	0.821167	0
400	3-O3B-A7-Docetaxel-Mitotic inhibitor, taxane microtubule stabilizer	1000	Mitotic	0.773539	0
401	3-O3B-B7-Docetaxel-Mitotic inhibitor, taxane microtubule stabilizer	100	Mitotic	0.779272	0
402	3-O3B-C7-Docetaxel-Mitotic inhibitor, taxane microtubule stabilizer	10	Mitotic	0.665329	0
403	3-O3B-D7-Docetaxel-Mitotic inhibitor, taxane microtubule stabilizer	1	Mitotic	0.254774	0.769
404	3-O3B-E7-Docetaxel-Mitotic inhibitor, taxane microtubule stabilizer	0.1	Mitotic	0.174829	0.798
405	6-O3B-L19-ABT-751-Mitotic inhibitor. Colchicine site binding microtubule depolymerizer.	1	Mitotic	0.615559	0
406	6-O3B-M19-ABT-751-Mitotic inhibitor. Colchicine site binding microtubule depolymerizer.	10	Mitotic	0.265342	0.924
407	6-O3B-N19-ABT-751-Mitotic inhibitor. Colchicine site binding microtubule depolymerizer.	100	Mitotic	0.439007	0.034
408	6-O3B-O19-ABT-751-Mitotic inhibitor. Colchicine site binding microtubule depolymerizer.	1000	Mitotic	0.826371	0
409	6-O3B-P19-ABT-751-Mitotic inhibitor. Colchicine site binding microtubule depolymerizer.	10000	Mitotic	0.819124	0
410	2-O3B-A12-Trametinib-MEK1/2 inhibitor	250	MEK1/2	0.690321	0
411	2-O3B-B12-Trametinib-MEK1/2 inhibitor	25	MEK1/2	0.716935	0
412	2-O3B-D12-Trametinib-MEK1/2 inhibitor	2.5	MEK1/2	0.70341	0
413	2-O3B-E12-Trametinib-MEK1/2 inhibitor	0.25	MEK1/2	0.710614	0
414	2-O3B-F12-Trametinib-MEK1/2 inhibitor	2.5000000	MEK1/2	0.676876	0
415	2-O3B-F14-Cobimetinib-MEK1/2 inhibitor	1000	MEK1/2	0.70958	0
416	2-O3B-G14-Cobimetinib-MEK1/2 inhibitor	100	MEK1/2	0.714033	0
417	2-O3B-H14-Cobimetinib-MEK1/2 inhibitor	10	MEK1/2	0.735952	0
418	2-O3B-I14-Cobimetinib-MEK1/2 inhibitor	1	MEK1/2	0.657594	0

**419**	2-O3B-K14-Cobimetinib-MEK1/2 inhibitor	0.1	MEK1/2	0.584187	0
**420**	2-O3B-L20-Selumetinib-MEK1/2 inhibitor	1	MEK1/2	0.208698	0.943
**421**	2-O3B-M20-Selumetinib-MEK1/2 inhibitor	10	MEK1/2	0.637398	0
**422**	2-O3B-N20-Selumetinib-MEK1/2 inhibitor	100	MEK1/2	0.745519	0
**423**	2-O3B-O20-Selumetinib-MEK1/2 inhibitor	1000	MEK1/2	0.727903	0
**424**	2-O3B-P20-Selumetinib-MEK1/2 inhibitor	10000	MEK1/2	0.714827	0
**425**	4-O3B-A10-Binimetinib-MEK1/2 inhibitor	1000	MEK1/2	0.755669	0
**426**	4-O3B-A13-PD0325901-MEK1/2 inhibitor	1000	MEK1/2	0.761235	0
**427**	4-O3B-B10-Binimetinib-MEK1/2 inhibitor	100	MEK1/2	0.783629	0
**428**	4-O3B-B13-PD0325901-MEK1/2 inhibitor	100	MEK1/2	0.757669	0
**429**	4-O3B-C10-Binimetinib-MEK1/2 inhibitor	10	MEK1/2	0.758989	0
**430**	4-O3B-C13-PD0325901-MEK1/2 inhibitor	10	MEK1/2	0.665825	0
**431**	4-O3B-D10-Binimetinib-MEK1/2 inhibitor	1	MEK1/2	0.506621	0.298
**432**	4-O3B-D13-PD0325901-MEK1/2 inhibitor	1	MEK1/2	0.666011	0
**433**	4-O3B-E10-Binimetinib-MEK1/2 inhibitor	0.1	MEK1/2	0.432317	0.091
**434**	4-O3B-E13-PD0325901-MEK1/2 inhibitor	0.1	MEK1/2	0.487413	0.549
**435**	4-O3B-L19-GDC-0623-MEK1/2 inhibitor	0.25	MEK1/2	0.629141	0
**436**	4-O3B-M19-GDC-0623-MEK1/2 inhibitor	2.5	MEK1/2	0.546786	0.026
**437**	4-O3B-N19-GDC-0623-MEK1/2 inhibitor	25	MEK1/2	0.75053	0
**438**	4-O3B-O19-GDC-0623-MEK1/2 inhibitor	250	MEK1/2	0.613851	0
**439**	4-O3B-P19-GDC-0623-MEK1/2 inhibitor	2500	MEK1/2	0.785765	0
**440**	1-O3B-L2-Olaparib-PARP inhibitor	1	PARP	0.533975	0.063
**441**	1-O3B-L6-Rucaparib-PARP inhibitor	1	PARP	0.721338	0.034
**442**	1-O3B-M2-Olaparib-PARP inhibitor	10	PARP	0.697085	0.022
**443**	1-O3B-M6-Rucaparib-PARP inhibitor	10	PARP	0.575086	0.025
**444**	1-O3B-N2-Olaparib-PARP inhibitor	100	PARP	0.674113	0.021
**445**	1-O3B-N6-Rucaparib-PARP inhibitor	100	PARP	0.680676	0.018
**446**	1-O3B-O2-Olaparib-PARP inhibitor	1000	PARP	0.675097	0.021
**447**	1-O3B-O6-Rucaparib-PARP inhibitor	1000	PARP	0.670831	0.028
**448**	1-O3B-P2-Olaparib-PARP inhibitor	10000	PARP	0.672798	0.021
**449**	1-O3B-P6-Rucaparib-PARP inhibitor	10000	PARP	0.671487	0.026
**450**	7-O3B-A3-Talazoparib-PARP1/2 inhibitor	1000	PARP	0.633849	0
**451**	7-O3B-B2-Veliparib-PARP inhibitor	10000	PARP	0.686889	0

452	7-O3B-B3-Talazoparib-PARP1/2 inhibitor	100	PARP	0.669302	0
453	7-O3B-C2-Veliparib-PARP inhibitor	1000	PARP	0.792725	0
454	7-O3B-C3-Talazoparib-PARP1/2 inhibitor	10	PARP	0.677854	0.001
455	7-O3B-D2-Veliparib-PARP inhibitor	100	PARP	0.624554	0.167
456	7-O3B-D3-Talazoparib-PARP1/2 inhibitor	1	PARP	0.735452	0
457	7-O3B-E2-Veliparib-PARP inhibitor	10	PARP	0.680896	0.076
458	7-O3B-E3-Talazoparib-PARP1/2 inhibitor	0.1	PARP	0.59714	0.004
459	7-O3B-F2-Veliparib-PARP inhibitor	1	PARP	0.67925	0.002
460	7-O3B-G2-Niraparib-PARP inhibitor	10000	PARP	0.70729	0
461	7-O3B-H2-Niraparib-PARP inhibitor	1000	PARP	0.732042	0
462	7-O3B-I2-Niraparib-PARP inhibitor	100	PARP	0.628999	0
463	7-O3B-J2-Niraparib-PARP inhibitor	10	PARP	0.670083	0.02
464	7-O3B-K2-Niraparib-PARP inhibitor	1	PARP	0.624175	0.002
465	3-O3B-A19-Dinaciclib-CDK inhibitor	1000	CDK	0.551825	0
466	3-O3B-B19-Dinaciclib-CDK inhibitor	100	CDK	0.519344	0.005
467	3-O3B-B23-Abemaciclib-CDK4/6 inhibitor	2500	CDK	0.339184	0.228
468	3-O3B-C19-Dinaciclib-CDK inhibitor	10	CDK	0.38898	0.056
469	3-O3B-C23-Abemaciclib-CDK4/6 inhibitor	250	CDK	0.366005	0.115
470	3-O3B-D19-Dinaciclib-CDK inhibitor	1	CDK	0.133191	0.745
471	3-O3B-D23-Abemaciclib-CDK4/6 inhibitor	25	CDK	0.215144	0.704
472	3-O3B-E19-Dinaciclib-CDK inhibitor	0.1	CDK	0.074385	0.991
473	3-O3B-E23-Abemaciclib-CDK4/6 inhibitor	2.5	CDK	0.344976	0.396
474	3-O3B-F23-Abemaciclib-CDK4/6 inhibitor	0.25	CDK	0.244293	0.457
475	3-O3B-K17-Palbociclib-CDK4/6 inhibitor	1	CDK	0.410734	0.04
476	3-O3B-L19-Ribociclib-CDK4/6 inhibitor	1	CDK	0.14321	0.564
477	3-O3B-M17-Palbociclib-CDK4/6 inhibitor	10	CDK	0.424156	0.034
478	3-O3B-M19-Ribociclib-CDK4/6 inhibitor	10	CDK	0.446914	0.001
479	3-O3B-N17-Palbociclib-CDK4/6 inhibitor	100	CDK	0.283103	0.184
480	3-O3B-N19-Ribociclib-CDK4/6 inhibitor	100	CDK	0.474505	0.002
481	3-O3B-O17-Palbociclib-CDK4/6 inhibitor	1000	CDK	0.423449	0.035
482	3-O3B-O19-Ribociclib-CDK4/6 inhibitor	1000	CDK	0.369892	0.077
483	3-O3B-P17-Palbociclib-CDK4/6 inhibitor	10000	CDK	0.275507	0.696
484	3-O3B-P19-Ribociclib-CDK4/6 inhibitor	10000	CDK	0.339006	0.336

485	4-O3B-A4-SNS-032-CDK inhibitor	10000	CDK	0.572411	0
486	4-O3B-A8-Milciclib-CDK2 inhibitor	10000	CDK	0.566464	0.002
487	4-O3B-B4-SNS-032-CDK inhibitor	1000	CDK	0.580539	0
488	4-O3B-B8-Milciclib-CDK2 inhibitor	1000	CDK	0.482089	0.037
489	4-O3B-C4-SNS-032-CDK inhibitor	100	CDK	0.402714	0.11
490	4-O3B-C8-Milciclib-CDK2 inhibitor	100	CDK	0.333336	0.321
491	4-O3B-D4-SNS-032-CDK inhibitor	10	CDK	0.39379	0.149
492	4-O3B-D8-Milciclib-CDK2 inhibitor	10	CDK	0.257957	0.987
493	4-O3B-E4-SNS-032-CDK inhibitor	1	CDK	0.401634	0.334
494	4-O3B-E8-Milciclib-CDK2 inhibitor	1	CDK	0.382171	0.633
495	4-O3B-F4-Seliciclib-CDK2/7/9 inhibitor	10000	CDK	0.427195	0.072
496	4-O3B-F22-Alvocidib-CDK inhibitor	10000	CDK	0.602502	0
497	4-O3B-G4-Seliciclib-CDK2/7/9 inhibitor	1000	CDK	0.37024	0.204
498	4-O3B-G22-Alvocidib-CDK inhibitor	1000	CDK	0.598461	0.001
499	4-O3B-H4-Seliciclib-CDK2/7/9 inhibitor	100	CDK	0.454178	0.019
500	4-O3B-H22-Alvocidib-CDK inhibitor	100	CDK	0.385929	0.113
501	4-O3B-I4-Seliciclib-CDK2/7/9 inhibitor	10	CDK	0.375908	0.191
502	4-O3B-I22-Alvocidib-CDK inhibitor	10	CDK	0.408461	0.155
503	4-O3B-J4-Seliciclib-CDK2/7/9 inhibitor	1	CDK	0.462921	0.023
504	4-O3B-J22-Alvocidib-CDK inhibitor	1	CDK	0.203507	0.894
505	5-O3B-A19-AZD-5438-CDK1,2,9 inhibitor	10000	CDK	0.43698	0.398
506	5-O3B-B19-AZD-5438-CDK1,2,9 inhibitor	1000	CDK	0.371632	0.19
507	5-O3B-C19-AZD-5438-CDK1,2,9 inhibitor	100	CDK	0.430216	0.034
508	5-O3B-D19-AZD-5438-CDK1,2,9 inhibitor	10	CDK	0.303721	0.305
509	5-O3B-E19-AZD-5438-CDK1,2,9 inhibitor	1	CDK	0.390743	0.104
510	5-O3B-K17-AT7519-CDK1, 2, 4, 6 and 9 inhibitor	1	CDK	0.386194	0.059
511	5-O3B-M17-AT7519-CDK1, 2, 4, 6 and 9 inhibitor	10	CDK	0.355166	0.325
512	5-O3B-N17-AT7519-CDK1, 2, 4, 6 and 9 inhibitor	100	CDK	0.392478	0.138
513	5-O3B-O17-AT7519-CDK1, 2, 4, 6 and 9 inhibitor	1000	CDK	0.580057	0
514	5-O3B-P17-AT7519-CDK1, 2, 4, 6 and 9 inhibitor	10000	CDK	0.584938	0
515	6-O3B-A17-Senexin B-CDK8/19 inhibitor	1000	CDK	0.394845	0.107
516	6-O3B-B17-Senexin B-CDK8/19 inhibitor	100	CDK	0.428834	0.037
517	6-O3B-C17-Senexin B-CDK8/19 inhibitor	10	CDK	0.407326	0.866

518	6-O3B-D17-Senexin B-CDK8/19 inhibitor	1	CDK	0.066908	0.905
519	6-O3B-E17-Senexin B-CDK8/19 inhibitor	0.1	CDK	0.275293	0.994
520	6-O3B-L15-THZ2-CDK7 inhibitor	1	CDK	0.332628	0.995
521	6-O3B-M15-THZ2-CDK7 inhibitor	10	CDK	0.250053	0.565
522	6-O3B-N15-THZ2-CDK7 inhibitor	100	CDK	0.55381	0.008
523	6-O3B-O15-THZ2-CDK7 inhibitor	1000	CDK	0.636774	0
524	6-O3B-P15-THZ2-CDK7 inhibitor	10000	CDK	0.6469	0
525	7-O3B-A21-dBET1-BET-targeting PROTAC	10000	BET	0.404575	0.151
526	7-O3B-A22-PFI-1-BET family inhibitor	30000	BET	0.685684	0
527	7-O3B-B21-dBET1-BET-targeting PROTAC	1000	BET	0.549959	0
528	7-O3B-B22-PFI-1-BET family inhibitor	3000	BET	0.583888	0
529	7-O3B-C21-dBET1-BET-targeting PROTAC	100	BET	0.356272	0.474
530	7-O3B-C22-PFI-1-BET family inhibitor	300	BET	0.347155	0.257
531	7-O3B-D21-dBET1-BET-targeting PROTAC	10	BET	0.232794	0.994
532	7-O3B-D22-PFI-1-BET family inhibitor	30	BET	0.56223	0.001
533	7-O3B-E21-dBET1-BET-targeting PROTAC	1	BET	0.408379	0.18
534	7-O3B-E22-PFI-1-BET family inhibitor	3	BET	0.494411	0.09
535	7-O3B-G10-Birabresib-BET family inhibitor	10000	BET	0.636684	0
536	7-O3B-G15-I-BET151-BET family inhibitor	10000	BET	0.669636	0
537	7-O3B-H10-Birabresib-BET family inhibitor	1000	BET	0.68948	0
538	7-O3B-H15-I-BET151-BET family inhibitor	1000	BET	0.694905	0
539	7-O3B-I10-Birabresib-BET family inhibitor	100	BET	0.668997	0
540	7-O3B-I15-I-BET151-BET family inhibitor	100	BET	0.418314	0.175
541	7-O3B-J10-Birabresib-BET family inhibitor	10	BET	0.348646	0.016
542	7-O3B-J15-I-BET151-BET family inhibitor	10	BET	0.323231	0.147
543	7-O3B-K10-Birabresib-BET family inhibitor	1	BET	0.141022	0.514
544	7-O3B-K13-Mivebresib-BET family inhibitor	1	BET	0.261237	0.191
545	7-O3B-K15-I-BET151-BET family inhibitor	1	BET	0.350586	0.828
546	7-O3B-L12-Molibresib-BET family inhibitor	1	BET	0.284913	0.701
547	7-O3B-L13-Mivebresib-BET family inhibitor	10	BET	0.640502	0
548	7-O3B-L20-JQ1-BET family inhibitor	1	BET	0.192866	0.968
549	7-O3B-L23-ARV-825-BET-targeting PROTAC	0.03	BET	0.430252	0.892
550	7-O3B-M12-Molibresib-BET family inhibitor	10	BET	0.390146	0.847

551	7-O3B-M13-Mivebresib-BET family inhibitor	100	BET	0.69418	0
552	7-O3B-M20-JQ1-BET family inhibitor	10	BET	0.455247	0.013
553	7-O3B-M23-ARV-825-BET-targeting PROTAC	0.3	BET	0.485466	0.004
554	7-O3B-N12-Molibresib-BET family inhibitor	100	BET	0.192864	0.373
555	7-O3B-N13-Mivebresib-BET family inhibitor	1000	BET	0.661757	0
556	7-O3B-N20-JQ1-BET family inhibitor	100	BET	0.646814	0
557	7-O3B-N23-ARV-825-BET-targeting PROTAC	3	BET	0.471537	0.028
558	7-O3B-O12-Molibresib-BET family inhibitor	1000	BET	0.64321	0
559	7-O3B-O20-JQ1-BET family inhibitor	1000	BET	0.702098	0
560	7-O3B-O23-ARV-825-BET-targeting PROTAC	30	BET	0.638625	0
561	7-O3B-P12-Molibresib-BET family inhibitor	10000	BET	0.69852	0
562	7-O3B-P13-Mivebresib-BET family inhibitor	10000	BET	0.661216	0
563	7-O3B-P20-JQ1-BET family inhibitor	10000	BET	0.720039	0
564	7-O3B-P23-ARV-825-BET-targeting PROTAC	300	BET	0.689846	0
565	8-O3B-K22-CPI-0610-BET family inhibitor	1	BET	0.218716	0.889
566	8-O3B-L22-CPI-0610-BET family inhibitor	10	BET	0.423865	0.712
567	8-O3B-M22-CPI-0610-BET family inhibitor	100	BET	0.372222	0.222
568	8-O3B-N22-CPI-0610-BET family inhibitor	1000	BET	0.564248	0
569	8-O3B-O22-CPI-0610-BET family inhibitor	10000	BET	0.230632	0.825
570	1-O3B-A3-Vorinostat-HDAC inhibitor	10000	HDAC	0.381234	0.18
571	1-O3B-B3-Vorinostat-HDAC inhibitor	1000	HDAC	0.29407	0.606
572	1-O3B-C3-Vorinostat-HDAC inhibitor	100	HDAC	0.274385	0.775
573	1-O3B-D3-Vorinostat-HDAC inhibitor	10	HDAC	0.533798	0.368
574	1-O3B-E3-Vorinostat-HDAC inhibitor	1	HDAC	0.480354	0.517
575	1-O3B-L12-Romidepsin-HDAC inhibitor	0.1	HDAC	0.414944	0.002
576	1-O3B-M12-Romidepsin-HDAC inhibitor	1	HDAC	0.532176	0.301
577	1-O3B-N12-Romidepsin-HDAC inhibitor	10	HDAC	0.379617	0.405
578	1-O3B-O12-Romidepsin-HDAC inhibitor	100	HDAC	0.372414	0.338
579	1-O3B-P12-Romidepsin-HDAC inhibitor	1000	HDAC	0.365907	0.381
580	3-O3B-A4-Panobinostat-HDAC inhibitor	1000	HDAC	0.380378	0.028
581	3-O3B-B4-Panobinostat-HDAC inhibitor	100	HDAC	0.341309	0.064
582	3-O3B-C4-Panobinostat-HDAC inhibitor	10	HDAC	0.63961	0
583	3-O3B-D4-Panobinostat-HDAC inhibitor	1	HDAC	0.209165	0.901

584	3-O3B-E4-Panobinostat-HDAC inhibitor	0.1	HDAC	0.209275	0.803
585	3-O3B-F7-Quisinostat-HDAC inhibitor	1000	HDAC	0.309707	0.336
586	3-O3B-G7-Quisinostat-HDAC inhibitor	100	HDAC	0.337142	0.094
587	3-O3B-G12-Valproic acid-HDAC inhibitor	1000000	HDAC	0.09189	0.947
588	3-O3B-H7-Quisinostat-HDAC inhibitor	10	HDAC	0.355335	0
589	3-O3B-H12-Valproic acid-HDAC inhibitor	100000	HDAC	0.230003	0.015
590	3-O3B-I7-Quisinostat-HDAC inhibitor	1	HDAC	0.349278	0.005
591	3-O3B-I12-Valproic acid-HDAC inhibitor	10000	HDAC	0.481674	0
592	3-O3B-J7-Quisinostat-HDAC inhibitor	0.1	HDAC	0.09655	0.786
593	3-O3B-J12-Valproic acid-HDAC inhibitor	1000	HDAC	0.083725	0.723
594	3-O3B-K3-Belinostat-HDAC inhibitor	1	HDAC	0.24462	0.52
595	3-O3B-K12-Valproic acid-HDAC inhibitor	100	HDAC	0.302732	0.069
596	3-O3B-L3-Belinostat-HDAC inhibitor	10	HDAC	0.087381	0.9
597	3-O3B-M3-Belinostat-HDAC inhibitor	100	HDAC	0.317075	0.299
598	3-O3B-N3-Belinostat-HDAC inhibitor	1000	HDAC	0.355438	0.105
599	3-O3B-O3-Belinostat-HDAC inhibitor	10000	HDAC	0.360757	0.135
600	7-O3B-A5-Mocetinostat-HDAC inhibitor (HDAC1 & 2-selective)	10000	HDAC	0.37935	0.057
601	7-O3B-A7-CUDC-907-HDAC1/2/3/10, PI3Kalpha inhibitor	10000	HDAC	0.406252	0.047
602	7-O3B-A9-Givinostat-HDAC inhibitor	1000	HDAC	0.380859	0.046
603	7-O3B-A12-Rocilinostat-HDAC-6 selective inhibitor	10000	HDAC	0.371611	0.021
604	7-O3B-B5-Mocetinostat-HDAC inhibitor (HDAC1 & 2-selective)	1000	HDAC	0.359614	0.82
605	7-O3B-B7-CUDC-907-HDAC1/2/3/10, PI3Kalpha inhibitor	1000	HDAC	0.409981	0.066
606	7-O3B-B12-Rocilinostat-HDAC-6 selective inhibitor	1000	HDAC	0.519834	0
607	7-O3B-C5-Mocetinostat-HDAC inhibitor (HDAC1 & 2-selective)	100	HDAC	0.637475	0
608	7-O3B-C7-CUDC-907-HDAC1/2/3/10, PI3Kalpha inhibitor	100	HDAC	0.439868	0.002
609	7-O3B-C9-Givinostat-HDAC inhibitor	100	HDAC	0.586772	0
610	7-O3B-D7-CUDC-907-HDAC1/2/3/10, PI3Kalpha inhibitor	10	HDAC	0.365915	0.617
611	7-O3B-D9-Givinostat-HDAC inhibitor	10	HDAC	0.606	0
612	7-O3B-D12-Rocilinostat-HDAC-6 selective inhibitor	100	HDAC	0.558603	0
613	7-O3B-E5-Mocetinostat-HDAC inhibitor (HDAC1 & 2-selective)	10	HDAC	0.461926	0.762
614	7-O3B-E7-CUDC-907-HDAC1/2/3/10, PI3Kalpha inhibitor	1	HDAC	0.39518	0.045
615	7-O3B-E9-Givinostat-HDAC inhibitor	1	HDAC	0.358372	0.866
616	7-O3B-E12-Rocilinostat-HDAC-6 selective inhibitor	10	HDAC	0.318282	0.001

617	7-O3B-F5-Mocetinostat-HDAC inhibitor (HDAC1 & 2-selective)	1	HDAC	0.32359	0.058
618	7-O3B-F7-Resminostat-HDAC1, 3, 6 inhibitor	10000	HDAC	0.417888	0.016
619	7-O3B-F9-Givinostat-HDAC inhibitor	0.1	HDAC	0.207921	0.045
620	7-O3B-F12-Rocilinostat-HDAC-6 selective inhibitor	1	HDAC	0.466026	0.153
621	7-O3B-F19-PCI-34051-HDAC8 inhibitor	10000	HDAC	0.43353	0.007
622	7-O3B-G7-Resminostat-HDAC1, 3, 6 inhibitor	1000	HDAC	0.536335	0
623	7-O3B-G19-PCI-34051-HDAC8 inhibitor	1000	HDAC	0.388906	0.733
624	7-O3B-H7-Resminostat-HDAC1, 3, 6 inhibitor	100	HDAC	0.523448	0
625	7-O3B-I7-Resminostat-HDAC1, 3, 6 inhibitor	10	HDAC	0.466641	0.001
626	7-O3B-I19-PCI-34051-HDAC8 inhibitor	100	HDAC	0.480222	0
627	7-O3B-J7-Resminostat-HDAC1, 3, 6 inhibitor	1	HDAC	0.50013	0.156
628	7-O3B-J19-PCI-34051-HDAC8 inhibitor	10	HDAC	0.276033	0.001
629	7-O3B-K4-Entinostat-HDAC inhibitor	1	HDAC	0.530661	0
630	7-O3B-K11-AR-42-HDAC inhibitor	1	HDAC	0.272239	0.061
631	7-O3B-K18-Tubacin-HDAC6 inhibitor	1	HDAC	0.412634	0
632	7-O3B-K19-PCI-34051-HDAC8 inhibitor	1	HDAC	0.385828	0.083
633	7-O3B-L2-Tacedinaline-HDAC inhibitor	0.1	HDAC	0.324023	0.258
634	7-O3B-L4-Entinostat-HDAC inhibitor	10	HDAC	0.349968	0.001
635	7-O3B-L5-Pracinostat-HDAC inhibitor	1	HDAC	0.479342	0
636	7-O3B-L8-Abexinostat-HDAC1-selective inhibitor	1	HDAC	0.466364	0.001
637	7-O3B-L10-Tucidinostat-HDAC1/2/3/10 inhibitor	1	HDAC	0.428398	0.362
638	7-O3B-L11-AR-42-HDAC inhibitor	10	HDAC	0.565568	0
639	7-O3B-L14-Tubastatin A-HDAC6 inhibitor	1	HDAC	0.365118	0
640	7-O3B-L16-RGFP966-HDAC3 inhibitor	1	HDAC	0.653923	0
641	7-O3B-L18-Tubacin-HDAC6 inhibitor	10	HDAC	0.453675	0
642	7-O3B-M2-Tacedinaline-HDAC inhibitor	1	HDAC	0.103097	0.99
643	7-O3B-M5-Pracinostat-HDAC inhibitor	10	HDAC	0.522048	0
644	7-O3B-M8-Abexinostat-HDAC1-selective inhibitor	10	HDAC	0.415414	0.088
645	7-O3B-M10-Tucidinostat-HDAC1/2/3/10 inhibitor	10	HDAC	0.464566	0.556
646	7-O3B-M11-AR-42-HDAC inhibitor	100	HDAC	0.522081	0
647	7-O3B-M14-Tubastatin A-HDAC6 inhibitor	10	HDAC	0.341383	0.007
648	7-O3B-M16-RGFP966-HDAC3 inhibitor	10	HDAC	0.572791	0
649	7-O3B-M18-Tubacin-HDAC6 inhibitor	100	HDAC	0.526547	0

650	7-O3B-N2-Tacedinaline-HDAC inhibitor	10	HDAC	0.220712	0.409
651	7-O3B-N4-Entinostat-HDAC inhibitor	100	HDAC	0.465721	0.002
652	7-O3B-N5-Pracinostat-HDAC inhibitor	100	HDAC	0.645763	0
653	7-O3B-N8-Abexinostat-HDAC1-selective inhibitor	100	HDAC	0.47069	0.226
654	7-O3B-N10-Tucidinostat-HDAC1/2/3/10 inhibitor	100	HDAC	0.637753	0
655	7-O3B-N14-Tubastatin A-HDAC6 inhibitor	100	HDAC	0.423797	0
656	7-O3B-N16-RGFP966-HDAC3 inhibitor	100	HDAC	0.395281	0.332
657	7-O3B-N18-Tubacin-HDAC6 inhibitor	1000	HDAC	0.510451	0.001
658	7-O3B-O2-Tacedinaline-HDAC inhibitor	100	HDAC	0.371937	0.503
659	7-O3B-O4-Entinostat-HDAC inhibitor	1000	HDAC	0.626837	0
660	7-O3B-O5-Pracinostat-HDAC inhibitor	1000	HDAC	0.350004	0.033
661	7-O3B-O8-Abexinostat-HDAC1-selective inhibitor	1000	HDAC	0.373691	0.102
662	7-O3B-O10-Tucidinostat-HDAC1/2/3/10 inhibitor	1000	HDAC	0.51539	0.153
663	7-O3B-O11-AR-42-HDAC inhibitor	1000	HDAC	0.411961	0.013
664	7-O3B-O14-Tubastatin A-HDAC6 inhibitor	1000	HDAC	0.242728	0.017
665	7-O3B-O16-RGFP966-HDAC3 inhibitor	1000	HDAC	0.48711	0
666	7-O3B-P2-Tacedinaline-HDAC inhibitor	1000	HDAC	0.446856	0
667	7-O3B-P4-Entinostat-HDAC inhibitor	10000	HDAC	0.33605	0.259
668	7-O3B-P5-Pracinostat-HDAC inhibitor	10000	HDAC	0.386922	0.149
669	7-O3B-P8-Abexinostat-HDAC1-selective inhibitor	10000	HDAC	0.409108	0.05
670	7-O3B-P10-Tucidinostat-HDAC1/2/3/10 inhibitor	10000	HDAC	0.315549	0.372
671	7-O3B-P11-AR-42-HDAC inhibitor	10000	HDAC	0.399639	0.129
672	7-O3B-P14-Tubastatin A-HDAC6 inhibitor	10000	HDAC	0.634027	0
673	7-O3B-P16-RGFP966-HDAC3 inhibitor	10000	HDAC	0.520432	0
674	7-O3B-P18-Tubacin-HDAC6 inhibitor	10000	HDAC	0.422813	0.078
0	2-O8W-A16-Gefitinib-EGFR inhibitor	10000	EGFR	0.283876	0.215
1	2-O8W-A19-Erlotinib-EGFR inhibitor	10000	EGFR	0.23925	0.49
2	2-O8W-B19-Erlotinib-EGFR inhibitor	1000	EGFR	0.204161	0.384
3	2-O8W-C16-Gefitinib-EGFR inhibitor	1000	EGFR	0.181497	0.324
4	2-O8W-C19-Erlotinib-EGFR inhibitor	100	EGFR	0.186361	0.737
5	2-O8W-D16-Gefitinib-EGFR inhibitor	100	EGFR	0.192326	0.26
6	2-O8W-D19-Erlotinib-EGFR inhibitor	10	EGFR	0.205776	0.693
7	2-O8W-E16-Gefitinib-EGFR inhibitor	10	EGFR	0.374748	0.074

8	2-O8W-E19-Erlotinib-EGFR inhibitor	1	EGFR	0.338438	0.14
9	2-O8W-F16-Gefitinib-EGFR inhibitor	1	EGFR	0.227486	0.682
10	2-O8W-K11-Afatinib-EGFR inhibitor	0.1	EGFR	0.39298	0.002
11	2-O8W-L11-Afatinib-EGFR inhibitor	1	EGFR	0.262203	0.442
12	2-O8W-L16-Osimertinib-EGFR(L858R/T790M) inhibitor	0.25	EGFR	0.292519	0.278
13	2-O8W-L19-Lapatinib-HER2, EGFR inhibitor	0.1	EGFR	0.327062	0.047
14	2-O8W-M11-Afatinib-EGFR inhibitor	10	EGFR	0.175913	0.34
15	2-O8W-M16-Osimertinib-EGFR(L858R/T790M) inhibitor	2.5	EGFR	0.283535	0.222
16	2-O8W-M19-Lapatinib-HER2, EGFR inhibitor	1	EGFR	0.293215	0.166
17	2-O8W-N16-Osimertinib-EGFR(L858R/T790M) inhibitor	25	EGFR	0.379538	0.011
18	2-O8W-N19-Lapatinib-HER2, EGFR inhibitor	10	EGFR	0.292123	0.04
19	2-O8W-O11-Afatinib-EGFR inhibitor	100	EGFR	0.43852	0.003
20	2-O8W-O16-Osimertinib-EGFR(L858R/T790M) inhibitor	250	EGFR	0.253154	0.541
21	2-O8W-O19-Lapatinib-HER2, EGFR inhibitor	100	EGFR	0.295197	0.29
22	2-O8W-P11-Afatinib-EGFR inhibitor	1000	EGFR	0.102266	0.785
23	2-O8W-P16-Osimertinib-EGFR(L858R/T790M) inhibitor	2500	EGFR	0.26022	0.443
24	2-O8W-P19-Lapatinib-HER2, EGFR inhibitor	1000	EGFR	0.273144	0.315
25	3-O8W-F21-Rociletinib-EGFR(L858R/T790M) inhibitor	10000	EGFR	0.118881	0.99
26	3-O8W-G20-Neratinib-EGFR inhibitor	1000	EGFR	0.225788	0.801
27	3-O8W-G21-Rociletinib-EGFR(L858R/T790M) inhibitor	1000	EGFR	0.185729	0.966
28	3-O8W-H20-Neratinib-EGFR inhibitor	100	EGFR	0.159629	0.566
29	3-O8W-H21-Rociletinib-EGFR(L858R/T790M) inhibitor	100	EGFR	0.176189	0.704
30	3-O8W-I20-Neratinib-EGFR inhibitor	10	EGFR	0.217467	0.696
31	3-O8W-I21-Rociletinib-EGFR(L858R/T790M) inhibitor	10	EGFR	0.288396	0.243
32	3-O8W-J20-Neratinib-EGFR inhibitor	1	EGFR	0.326226	0.095
33	3-O8W-J21-Rociletinib-EGFR(L858R/T790M) inhibitor	1	EGFR	0.415273	0.012
34	3-O8W-K4-Canertinib-pan-HER inhibitor	1	EGFR	0.204774	0.878
35	3-O8W-K18-Dacomitinib-pan-HER inhibitor	0.1	EGFR	0.397702	0.021
36	3-O8W-K20-Neratinib-EGFR inhibitor	0.1	EGFR	0.175136	0.731
37	3-O8W-L4-Canertinib-pan-HER inhibitor	10	EGFR	0.193037	0.514
38	3-O8W-L18-Dacomitinib-pan-HER inhibitor	1	EGFR	0.147104	0.745
39	3-O8W-M18-Dacomitinib-pan-HER inhibitor	10	EGFR	0.313905	0.079
40	3-O8W-N4-Canertinib-pan-HER inhibitor	100	EGFR	0.271215	0.186

41	3-O8W-N18-Dacomitinib-pan-HER inhibitor	100	EGFR	0.348828	0.028
42	3-O8W-O4-Canertinib-pan-HER inhibitor	1000	EGFR	0.195427	0.68
43	3-O8W-P4-Canertinib-pan-HER inhibitor	10000	EGFR	0.102658	0.994
44	3-O8W-P18-Dacomitinib-pan-HER inhibitor	1000	EGFR	0.23593	0.509
45	4-O8W-F13-Sapitinib-Pan-HER inhibitor	1000	EGFR	0.291931	0.187
46	4-O8W-G13-Sapitinib-Pan-HER inhibitor	100	EGFR	0.341792	0.074
47	4-O8W-G16-Varlitinib-EGFR HER2 inhibitor	10000	EGFR	0.262249	0.388
48	4-O8W-H13-Sapitinib-Pan-HER inhibitor	10	EGFR	0.169863	0.396
49	4-O8W-H16-Varlitinib-EGFR HER2 inhibitor	1000	EGFR	0.215481	0.364
50	4-O8W-I13-Sapitinib-Pan-HER inhibitor	1	EGFR	0.235984	0.377
51	4-O8W-I16-Varlitinib-EGFR HER2 inhibitor	100	EGFR	0.265415	0.337
52	4-O8W-J13-Sapitinib-Pan-HER inhibitor	0.1	EGFR	0.294595	0.117
53	4-O8W-J16-Varlitinib-EGFR HER2 inhibitor	10	EGFR	0.310192	0.396
54	4-O8W-K7-Icotinib-EGFR inhibitor	1	EGFR	0.259966	0.443
55	4-O8W-K13-Tesevatinib-EGFR, ERBB2, VEGFR, EPHB4	0.1	EGFR	0.275018	0.135
56	4-O8W-K16-Varlitinib-EGFR HER2 inhibitor	1	EGFR	0.315389	0.188
57	4-O8W-L7-Icotinib-EGFR inhibitor	10	EGFR	0.174548	0.926
58	4-O8W-L13-Tesevatinib-EGFR, ERBB2, VEGFR, EPHB4	1	EGFR	0.287962	0.352
59	4-O8W-M7-Icotinib-EGFR inhibitor	100	EGFR	0.210611	0.778
60	4-O8W-M13-Tesevatinib-EGFR, ERBB2, VEGFR, EPHB4	10	EGFR	0.393526	0.002
61	4-O8W-N13-Tesevatinib-EGFR, ERBB2, VEGFR, EPHB4	100	EGFR	0.314482	0.042
62	4-O8W-O7-Icotinib-EGFR inhibitor	1000	EGFR	0.124948	0.592
63	4-O8W-P7-Icotinib-EGFR inhibitor	10000	EGFR	0.308566	0.161
64	4-O8W-P13-Tesevatinib-EGFR, ERBB2, VEGFR, EPHB4	1000	EGFR	0.213005	0.545
65	5-O8W-F4-Poziotinib-pan-HER inhibitor	1000	EGFR	0.335164	0.15
66	5-O8W-F7-AZD3759-EGFR inhibitor, BBB penetrable	1000	EGFR	0.274986	0.371
67	5-O8W-G4-Poziotinib-pan-HER inhibitor	100	EGFR	0.216415	0.272
68	5-O8W-G7-AZD3759-EGFR inhibitor, BBB penetrable	100	EGFR	0.168238	0.709
69	5-O8W-H4-Poziotinib-pan-HER inhibitor	10	EGFR	0.213209	0.342
70	5-O8W-H7-AZD3759-EGFR inhibitor, BBB penetrable	10	EGFR	0.251131	0.396
71	5-O8W-I4-Poziotinib-pan-HER inhibitor	1	EGFR	0.146841	0.558
72	5-O8W-I7-AZD3759-EGFR inhibitor, BBB penetrable	1	EGFR	0.219356	0.655
73	5-O8W-J4-Poziotinib-pan-HER inhibitor	0.1	EGFR	0.281756	0.018

**74**	5-O8W-J7-AZD3759-EGFR inhibitor, BBB penetrable	0.1	EGFR	0.260931	0.222
**75**	5-O8W-K7-Olmutinib-EGFR(L858R/T790M) inhibitor	0.1	EGFR	0.295722	0.176
**76**	5-O8W-L7-Olmutinib-EGFR(L858R/T790M) inhibitor	1	EGFR	0.190646	0.706
**77**	5-O8W-M7-Olmutinib-EGFR(L858R/T790M) inhibitor	10	EGFR	0.187697	0.773
**78**	5-O8W-O7-Olmutinib-EGFR(L858R/T790M) inhibitor	100	EGFR	0.299151	0.135
**79**	5-O8W-P7-Olmutinib-EGFR(L858R/T790M) inhibitor	1000	EGFR	0.202782	0.744
**80**	2-O8W-A15-Lenvatinib-VEGFR inhibitor	2500	VEGFR	0.318892	0.065
**81**	2-O8W-A17-Nintedanib-VEGFR, PDGFR, FGFR inhibitor	10000	VEGFR	0.395479	0.018
**82**	2-O8W-A20-Tivozanib-VEGFR1, 2, 3, c-Kit, PDGFRB inhibitor	10000	VEGFR	0.335768	0.196
**83**	2-O8W-B15-Lenvatinib-VEGFR inhibitor	250	VEGFR	0.328384	0.039
**84**	2-O8W-B17-Nintedanib-VEGFR, PDGFR, FGFR inhibitor	1000	VEGFR	0.258393	0.726
**85**	2-O8W-B20-Tivozanib-VEGFR1, 2, 3, c-Kit, PDGFRB inhibitor	1000	VEGFR	0.184987	0.399
**86**	2-O8W-C15-Lenvatinib-VEGFR inhibitor	25	VEGFR	0.366494	0.007
**87**	2-O8W-C17-Nintedanib-VEGFR, PDGFR, FGFR inhibitor	100	VEGFR	0.363129	0.006
**88**	2-O8W-D15-Lenvatinib-VEGFR inhibitor	2.5	VEGFR	0.405984	0.001
**89**	2-O8W-D17-Nintedanib-VEGFR, PDGFR, FGFR inhibitor	10	VEGFR	0.353003	0.064
**90**	2-O8W-D20-Tivozanib-VEGFR1, 2, 3, c-Kit, PDGFRB inhibitor	100	VEGFR	0.40919	0
**91**	2-O8W-E17-Nintedanib-VEGFR, PDGFR, FGFR inhibitor	1	VEGFR	0.293843	0.122
**92**	2-O8W-E20-Tivozanib-VEGFR1, 2, 3, c-Kit, PDGFRB inhibitor	10	VEGFR	0.291164	0.206
**93**	2-O8W-F13-Axitinib-VEGFR, PDGFR, KIT inhibitor	10000	VEGFR	0.334076	0.087
**94**	2-O8W-F15-Lenvatinib-VEGFR inhibitor	0.25	VEGFR	0.461311	0
**95**	2-O8W-F19-Regorafenib-B-Raf, c-Kit, VEGFR2 inhibitor	10000	VEGFR	0.179219	0.915
**96**	2-O8W-F20-Tivozanib-VEGFR1, 2, 3, c-Kit, PDGFRB inhibitor	1	VEGFR	0.306984	0.106
**97**	2-O8W-F21-Vatalanib-VEGFR-1 & -2 inhibitor	10000	VEGFR	0.099808	0.944
**98**	2-O8W-G10-Apatinib-VEGFR inhibitor	10000	VEGFR	0.340923	0.025
**99**	2-O8W-G13-Axitinib-VEGFR, PDGFR, KIT inhibitor	1000	VEGFR	0.324751	0.042
**100**	2-O8W-G19-Regorafenib-B-Raf, c-Kit, VEGFR2 inhibitor	1000	VEGFR	0.128248	0.759
**101**	2-O8W-G21-Vatalanib-VEGFR-1 & -2 inhibitor	1000	VEGFR	0.337283	0.032
**102**	2-O8W-H10-Apatinib-VEGFR inhibitor	1000	VEGFR	0.216891	0.323
**103**	2-O8W-H13-Axitinib-VEGFR, PDGFR, KIT inhibitor	100	VEGFR	0.253802	0.322
**104**	2-O8W-H21-Vatalanib-VEGFR-1 & -2 inhibitor	100	VEGFR	0.085251	0.91
**105**	2-O8W-I10-Apatinib-VEGFR inhibitor	100	VEGFR	0.396072	0.003
**106**	2-O8W-I13-Axitinib-VEGFR, PDGFR, KIT inhibitor	10	VEGFR	0.33037	0.022

107	2-O8W-I19-Regorafenib-B-Raf, c-Kit, VEGFR2 inhibitor	100	VEGFR	0.351248	0.01
108	2-O8W-I21-Vatalanib-VEGFR-1 & -2 inhibitor	10	VEGFR	0.392921	0.014
109	2-O8W-J10-Apatinib-VEGFR inhibitor	10	VEGFR	0.252294	0.237
110	2-O8W-J13-Axitinib-VEGFR, PDGFR, KIT inhibitor	1	VEGFR	0.356341	0.032
111	2-O8W-J19-Regorafenib-B-Raf, c-Kit, VEGFR2 inhibitor	10	VEGFR	0.387483	0.005
112	2-O8W-J21-Vatalanib-VEGFR-1 & -2 inhibitor	1	VEGFR	0.377742	0.004
113	2-O8W-K10-Apatinib-VEGFR inhibitor	1	VEGFR	0.259397	0.182
114	2-O8W-K13-Vandetanib-VEGFR,EGFR, RET inhibitor	0.1	VEGFR	0.133503	0.587
115	2-O8W-K17-Pazopanib-VEGFR inhibitor	1	VEGFR	0.44165	0
116	2-O8W-K19-Regorafenib-B-Raf, c-Kit, VEGFR2 inhibitor	1	VEGFR	0.357687	0.008
117	2-O8W-L12-Sorafenib-B-Raf, FGFR-1, VEGFR-2 & -3, PDGFR-beta, KIT, and FLT3 inhib	0.1	VEGFR	0.475915	0
118	2-O8W-L13-Vandetanib-VEGFR,EGFR, RET inhibitor	1	VEGFR	0.432882	0.001
119	2-O8W-L21-Cediranib-KDR/Flt/VEGFR inhibitor	0.1	VEGFR	0.389739	0.009
120	2-O8W-M12-Sorafenib-B-Raf, FGFR-1, VEGFR-2 & -3, PDGFR-beta, KIT, and FLT3 inhib	1	VEGFR	0.302221	0.083
121	2-O8W-M13-Vandetanib-VEGFR,EGFR, RET inhibitor	10	VEGFR	0.439143	0
122	2-O8W-M17-Pazopanib-VEGFR inhibitor	10	VEGFR	0.364579	0.009
123	2-O8W-M21-Cediranib-KDR/Flt/VEGFR inhibitor	1	VEGFR	0.352654	0.019
124	2-O8W-N12-Sorafenib-B-Raf, FGFR-1, VEGFR-2 & -3, PDGFR-beta, KIT, and FLT3 inhib	10	VEGFR	0.386794	0.002
125	2-O8W-N13-Vandetanib-VEGFR,EGFR, RET inhibitor	100	VEGFR	0.210979	0.211
126	2-O8W-N17-Pazopanib-VEGFR inhibitor	100	VEGFR	0.359799	0.01
127	2-O8W-N21-Cediranib-KDR/Flt/VEGFR inhibitor	10	VEGFR	0.147273	0.849
128	2-O8W-O12-Sorafenib-B-Raf, FGFR-1, VEGFR-2 & -3, PDGFR-beta, KIT, and FLT3 inhib	100	VEGFR	0.340073	0.134
129	2-O8W-O17-Pazopanib-VEGFR inhibitor	1000	VEGFR	0.434877	0
130	2-O8W-O21-Cediranib-KDR/Flt/VEGFR inhibitor	100	VEGFR	0.380687	0.004
131	2-O8W-P12-Sorafenib-B-Raf, FGFR-1, VEGFR-2 & -3, PDGFR-beta, KIT, and FLT3 inhib	1000	VEGFR	0.163493	0.437
132	2-O8W-P13-Vandetanib-VEGFR,EGFR, RET inhibitor	1000	VEGFR	0.173376	0.617
133	2-O8W-P17-Pazopanib-VEGFR inhibitor	10000	VEGFR	0.161687	0.577
134	2-O8W-P21-Cediranib-KDR/Flt/VEGFR inhibitor	1000	VEGFR	0.393338	0.002
135	3-O8W-A3-Cabozantinib-VEGFR2, Met, FLT3, Tie2, Kit and Ret inhibitor	1000	VEGFR	0.231509	0.276
136	3-O8W-A6-Foretinib-MET, VEGFR2 inhibitor	1000	VEGFR	0.335788	0.177
137	3-O8W-A18-Linifanib-VEGFR, PDGFR, CSF-1R, FLT3 inhibitor	1000	VEGFR	0.209452	0.858
138	3-O8W-B3-Cabozantinib-VEGFR2, Met, FLT3, Tie2, Kit and Ret inhibitor	100	VEGFR	0.187653	0.376
139	3-O8W-B6-Foretinib-MET, VEGFR2 inhibitor	100	VEGFR	0.286384	0.227

140	3-O8W-B18-Linifanib-VEGFR, PDGFR, CSF-1R, FLT3 inhibitor	100	VEGFR	0.169605	0.541
141	3-O8W-C3-Cabozantinib-VEGFR2, Met, FLT3, Tie2, Kit and Ret inhibitor	10	VEGFR	0.126394	0.828
142	3-O8W-C6-Foretinib-MET, VEGFR2 inhibitor	10	VEGFR	0.111645	0.943
143	3-O8W-C18-Linifanib-VEGFR, PDGFR, CSF-1R, FLT3 inhibitor	10	VEGFR	0.229929	0.372
144	3-O8W-D3-Cabozantinib-VEGFR2, Met, FLT3, Tie2, Kit and Ret inhibitor	1	VEGFR	0.228016	0.611
145	3-O8W-D6-Foretinib-MET, VEGFR2 inhibitor	1	VEGFR	0.166227	0.87
146	3-O8W-D18-Linifanib-VEGFR, PDGFR, CSF-1R, FLT3 inhibitor	1	VEGFR	0.139539	0.657
147	3-O8W-E3-Cabozantinib-VEGFR2, Met, FLT3, Tie2, Kit and Ret inhibitor	0.1	VEGFR	0.180345	0.907
148	3-O8W-E6-Foretinib-MET, VEGFR2 inhibitor	0.1	VEGFR	0.202026	0.926
149	3-O8W-E18-Linifanib-VEGFR, PDGFR, CSF-1R, FLT3 inhibitor	0.1	VEGFR	0.264191	0.307
150	3-O8W-F18-Brivanib-VEGFR inhibitor	1000	VEGFR	0.269109	0.251
151	3-O8W-G18-Brivanib-VEGFR inhibitor	100	VEGFR	0.277946	0.261
152	3-O8W-H18-Brivanib-VEGFR inhibitor	10	VEGFR	0.468883	0.011
153	3-O8W-I18-Brivanib-VEGFR inhibitor	1	VEGFR	0.168852	0.645
154	3-O8W-J18-Brivanib-VEGFR inhibitor	0.1	VEGFR	0.286922	0.721
155	4-O8W-A12-ENMD-2076-pan-Aurora, VEGFR inhibitor	10000	VEGFR	0.183086	0.87
156	4-O8W-A15-Golvatinib-MET, VEGFR2 inhibitor	2500	VEGFR	0.113136	0.696
157	4-O8W-A20-Motesanib-VEGFR, PDGFR, Ret, Kit inhibitor	10000	VEGFR	0.261769	0.311
158	4-O8W-B12-ENMD-2076-pan-Aurora, VEGFR inhibitor	1000	VEGFR	0.327976	0.076
159	4-O8W-B15-Golvatinib-MET, VEGFR2 inhibitor	250	VEGFR	0.097905	0.816
160	4-O8W-B20-Motesanib-VEGFR, PDGFR, Ret, Kit inhibitor	1000	VEGFR	0.27852	0.378
161	4-O8W-C15-Golvatinib-MET, VEGFR2 inhibitor	25	VEGFR	0.320432	0.246
162	4-O8W-D12-ENMD-2076-pan-Aurora, VEGFR inhibitor	100	VEGFR	0.45213	0
163	4-O8W-D15-Golvatinib-MET, VEGFR2 inhibitor	2.5	VEGFR	0.263774	0.254
164	4-O8W-D20-Motesanib-VEGFR, PDGFR, Ret, Kit inhibitor	100	VEGFR	0.20142	0.365
165	4-O8W-E12-ENMD-2076-pan-Aurora, VEGFR inhibitor	10	VEGFR	0.383642	0.004
166	4-O8W-E20-Motesanib-VEGFR, PDGFR, Ret, Kit inhibitor	10	VEGFR	0.293997	0.212
167	4-O8W-F12-ENMD-2076-pan-Aurora, VEGFR inhibitor	1	VEGFR	0.283983	0.31
168	4-O8W-F15-Golvatinib-MET, VEGFR2 inhibitor	0.25	VEGFR	0.202366	0.755
169	4-O8W-F20-Motesanib-VEGFR, PDGFR, Ret, Kit inhibitor	1	VEGFR	0.350909	0.037
170	4-O8W-L16-Telatinib-VEGFR, KIT, PDGFR inhibitor	1	VEGFR	0.127056	0.889
171	4-O8W-M16-Telatinib-VEGFR, KIT, PDGFR inhibitor	10	VEGFR	0.245161	0.189
172	4-O8W-N16-Telatinib-VEGFR, KIT, PDGFR inhibitor	100	VEGFR	0.261098	0.402

173	4-O8W-O16-Telatinib-VEGFR, KIT, PDGFR inhibitor	1000	VEGFR	0.232047	0.591
174	4-O8W-P16-Telatinib-VEGFR, KIT, PDGFR inhibitor	10000	VEGFR	0.259592	0.437
175	2-O8W-L10-Idelalisib-PI3K inhibitor, p110δ-selective	1	PI3K	0.361023	0.007
176	2-O8W-M10-Idelalisib-PI3K inhibitor, p110δ-selective	10	PI3K	0.240433	0.247
177	2-O8W-N10-Idelalisib-PI3K inhibitor, p110δ-selective	100	PI3K	0.264788	0.4
178	2-O8W-O10-Idelalisib-PI3K inhibitor, p110δ-selective	1000	PI3K	0.45435	0
179	2-O8W-P10-Idelalisib-PI3K inhibitor, p110δ-selective	10000	PI3K	0.343765	0.019
180	3-O8W-A16-Perifosine-AKT/PI3K inhibitor	2500	PI3K	0.159761	0.502
181	3-O8W-C16-Perifosine-AKT/PI3K inhibitor	250	PI3K	0.288761	0.014
182	3-O8W-D16-Perifosine-AKT/PI3K inhibitor	25	PI3K	0.132431	0.955
183	3-O8W-E16-Perifosine-AKT/PI3K inhibitor	2.5	PI3K	0.233706	0.68
184	3-O8W-F16-Perifosine-AKT/PI3K inhibitor	0.25	PI3K	0.194372	0.734
185	3-O8W-F17-Miltefosine-Antimicrobial, inhibits PI3K/AKT	100000	PI3K	0.14032	0.997
186	3-O8W-F19-Duvelisib-PI3K inhibitor	500	PI3K	0.343279	0.003
187	3-O8W-G17-Miltefosine-Antimicrobial, inhibits PI3K/AKT	10000	PI3K	0.071293	0.989
188	3-O8W-G19-Duvelisib-PI3K inhibitor	50	PI3K	0.291739	0.032
189	3-O8W-H17-Miltefosine-Antimicrobial, inhibits PI3K/AKT	1000	PI3K	0.456317	0
190	3-O8W-I17-Miltefosine-Antimicrobial, inhibits PI3K/AKT	100	PI3K	0.146261	0.98
191	3-O8W-I19-Duvelisib-PI3K inhibitor	5	PI3K	0.324512	0.051
192	3-O8W-J17-Miltefosine-Antimicrobial, inhibits PI3K/AKT	10	PI3K	0.214386	0.082
193	3-O8W-J19-Duvelisib-PI3K inhibitor	0.5	PI3K	0.308682	0.005
194	3-O8W-K19-Duvelisib-PI3K inhibitor	0.05	PI3K	0.300938	0.037
195	3-O8W-L8-Pictilisib-PI3K inhibitor, pan-class I	1	PI3K	0.170455	0.949
196	3-O8W-L21-Taselisib-PI3K alpha, delta, (gamma) selective inhibitor	0.1	PI3K	0.193928	0.391
197	3-O8W-M8-Pictilisib-PI3K inhibitor, pan-class I	10	PI3K	0.249847	0.604
198	3-O8W-M21-Taselisib-PI3K alpha, delta, (gamma) selective inhibitor	1	PI3K	0.405706	0
199	3-O8W-N8-Pictilisib-PI3K inhibitor, pan-class I	100	PI3K	0.334102	0
200	3-O8W-N21-Taselisib-PI3K alpha, delta, (gamma) selective inhibitor	10	PI3K	0.263896	0.071
201	3-O8W-O8-Pictilisib-PI3K inhibitor, pan-class I	1000	PI3K	0.323949	0.303
202	3-O8W-O21-Taselisib-PI3K alpha, delta, (gamma) selective inhibitor	100	PI3K	0.347609	0.008
203	3-O8W-P8-Pictilisib-PI3K inhibitor, pan-class I	10000	PI3K	0.193254	0.855
204	3-O8W-P21-Taselisib-PI3K alpha, delta, (gamma) selective inhibitor	1000	PI3K	0.49357	0
205	4-O8W-A19-Alpelisib-PI3Kalpha inhibitor	2500	PI3K	0.311126	0.005

206	4-O8W-B19-Alpelisib-PI3Kalpha inhibitor	250	PI3K	0.199147	0.057
207	4-O8W-C19-Alpelisib-PI3Kalpha inhibitor	25	PI3K	0.378566	0
208	4-O8W-D19-Alpelisib-PI3Kalpha inhibitor	2.5	PI3K	0.231119	0.663
209	4-O8W-E19-Alpelisib-PI3Kalpha inhibitor	0.25	PI3K	0.292497	0.186
210	4-O8W-F14-NVP-BGT226-PI3K/mTOR inhibitor	1000	PI3K	0.111156	0.999
211	4-O8W-G2-TGR-1202-PI3Kdelta inhibitor	2500	PI3K	0.262763	0.215
212	4-O8W-G5-Sonolisib-PI3K inhibitor, pan-class I. Irreversible	10000	PI3K	0.305148	0.053
213	4-O8W-G14-NVP-BGT226-PI3K/mTOR inhibitor	100	PI3K	0.237354	0.569
214	4-O8W-G20-Buparlisib-PI3K inhibitor, pan-class I	10000	PI3K	0.06699	1
215	4-O8W-H2-TGR-1202-PI3Kdelta inhibitor	250	PI3K	0.287465	0.311
216	4-O8W-H5-Sonolisib-PI3K inhibitor, pan-class I. Irreversible	1000	PI3K	0.131048	0.897
217	4-O8W-H14-NVP-BGT226-PI3K/mTOR inhibitor	10	PI3K	0.292847	0.11
218	4-O8W-H20-Buparlisib-PI3K inhibitor, pan-class I	1000	PI3K	0.500272	0
219	4-O8W-I2-TGR-1202-PI3Kdelta inhibitor	25	PI3K	0.256052	0.584
220	4-O8W-I5-Sonolisib-PI3K inhibitor, pan-class I. Irreversible	100	PI3K	0.245794	0.445
221	4-O8W-I14-NVP-BGT226-PI3K/mTOR inhibitor	1	PI3K	0.289202	0.007
222	4-O8W-I20-Buparlisib-PI3K inhibitor, pan-class I	100	PI3K	0.501953	0
223	4-O8W-J2-TGR-1202-PI3Kdelta inhibitor	2.5	PI3K	0.118804	0.774
224	4-O8W-J5-Sonolisib-PI3K inhibitor, pan-class I. Irreversible	10	PI3K	0.150256	0.973
225	4-O8W-J20-Buparlisib-PI3K inhibitor, pan-class I	10	PI3K	0.360905	0.066
226	4-O8W-K2-TGR-1202-PI3Kdelta inhibitor	0.25	PI3K	0.249115	0.292
227	4-O8W-K4-Dactolisib-mTOR/(PI3K) inhibitor	0.1	PI3K	0.277077	0.168
228	4-O8W-K5-Sonolisib-PI3K inhibitor, pan-class I. Irreversible	1	PI3K	0.2386	0.707
229	4-O8W-K14-NVP-BGT226-PI3K/mTOR inhibitor	0.1	PI3K	0.280247	0.099
230	4-O8W-K20-Buparlisib-PI3K inhibitor, pan-class I	1	PI3K	0.465912	0
231	4-O8W-L4-Dactolisib-mTOR/(PI3K) inhibitor	1	PI3K	0.405458	0
232	4-O8W-L14-Gedatolisib-PI3K/mTOR inhibitor	0.1	PI3K	0.143136	0.176
233	4-O8W-L15-TG100-115-PI3K gamma/delta inhibitor	1	PI3K	0.262817	0.068
234	4-O8W-L21-Copanlisib-PI3K alpha, delta selective inhibitor	0.1	PI3K	0.282776	0.005
235	4-O8W-M14-Gedatolisib-PI3K/mTOR inhibitor	1	PI3K	0.274815	0.175
236	4-O8W-M15-TG100-115-PI3K gamma/delta inhibitor	10	PI3K	0.215707	0.486
237	4-O8W-M21-Copanlisib-PI3K alpha, delta selective inhibitor	1	PI3K	0.326601	0.007
238	4-O8W-N4-Dactolisib-mTOR/(PI3K) inhibitor	10	PI3K	0.272292	0.222

239	4-O8W-N14-Gedatolisib-PI3K/mTOR inhibitor	10	PI3K	0.206395	0.164
240	4-O8W-N15-TG100-115-PI3K gamma/delta inhibitor	100	PI3K	0.147932	0.183
241	4-O8W-N21-Copanlisib-PI3K alpha, delta selective inhibitor	10	PI3K	0.369134	0.003
242	4-O8W-O4-Dactolisib-mTOR/(PI3K) inhibitor	100	PI3K	0.248409	0.377
243	4-O8W-O14-Gedatolisib-PI3K/mTOR inhibitor	100	PI3K	0.284215	0.139
244	4-O8W-O15-TG100-115-PI3K gamma/delta inhibitor	1000	PI3K	0.277331	0.166
245	4-O8W-O21-Copanlisib-PI3K alpha, delta selective inhibitor	100	PI3K	0.383947	0.001
246	4-O8W-P4-Dactolisib-mTOR/(PI3K) inhibitor	1000	PI3K	0.274311	0.418
247	4-O8W-P14-Gedatolisib-PI3K/mTOR inhibitor	1000	PI3K	0.261467	0.532
248	4-O8W-P15-TG100-115-PI3K gamma/delta inhibitor	10000	PI3K	0.323963	0.005
249	4-O8W-P21-Copanlisib-PI3K alpha, delta selective inhibitor	1000	PI3K	0.323868	0.039
250	5-O8W-A6-LY3023414-PI3K/mTOR/DNA-PK inhibitor	2500	PI3K	0.260476	0.268
251	5-O8W-A7-AMG319-PI3Kdelta inhibitor	1000	PI3K	0.394169	0.001
252	5-O8W-A16-AZD-6482-PI3Kbeta-selective inhibitor	2500	PI3K	0.35365	0.008
253	5-O8W-A17-Palomid-529-AKT, MTOR, PI3K inhibitor	10000	PI3K	0.185698	0.614
254	5-O8W-B6-LY3023414-PI3K/mTOR/DNA-PK inhibitor	250	PI3K	0.272102	0.129
255	5-O8W-B7-AMG319-PI3Kdelta inhibitor	100	PI3K	0.188393	0.848
256	5-O8W-B17-Palomid-529-AKT, MTOR, PI3K inhibitor	1000	PI3K	0.097528	0.995
257	5-O8W-C6-LY3023414-PI3K/mTOR/DNA-PK inhibitor	25	PI3K	0.067678	0.976
258	5-O8W-C7-AMG319-PI3Kdelta inhibitor	10	PI3K	0.258007	0.226
259	5-O8W-C16-AZD-6482-PI3Kbeta-selective inhibitor	250	PI3K	0.10124	0.948
260	5-O8W-C17-Palomid-529-AKT, MTOR, PI3K inhibitor	100	PI3K	0.092405	0.993
261	5-O8W-D6-LY3023414-PI3K/mTOR/DNA-PK inhibitor	2.5	PI3K	0.240147	0.614
262	5-O8W-D7-AMG319-PI3Kdelta inhibitor	1	PI3K	0.269064	0.148
263	5-O8W-D16-AZD-6482-PI3Kbeta-selective inhibitor	25	PI3K	0.324525	0.009
264	5-O8W-D17-Palomid-529-AKT, MTOR, PI3K inhibitor	10	PI3K	0.165871	0.609
265	5-O8W-E6-LY3023414-PI3K/mTOR/DNA-PK inhibitor	0.25	PI3K	0.195528	0.8
266	5-O8W-E7-AMG319-PI3Kdelta inhibitor	0.1	PI3K	0.155573	0.883
267	5-O8W-E16-AZD-6482-PI3Kbeta-selective inhibitor	2.5	PI3K	0.198986	0.288
268	5-O8W-E17-Palomid-529-AKT, MTOR, PI3K inhibitor	1	PI3K	0.407823	0
269	5-O8W-F11-GSK2636771-PI3K beta selective inhibitor	10000	PI3K	0.126449	0.682
270	5-O8W-F16-AZD-6482-PI3Kbeta-selective inhibitor	0.25	PI3K	0.094533	0.889
271	5-O8W-G9-Serabelisib-PI3Kalpha selective inhibitor	10000	PI3K	0.378378	0.002

272	5-O8W-G11-GSK2636771-PI3K beta selective inhibitor	1000	PI3K	0.225505	0.49
273	5-O8W-H9-Serabelisib-PI3Kalpha selective inhibitor	1000	PI3K	0.216712	0.029
274	5-O8W-H11-GSK2636771-PI3K beta selective inhibitor	100	PI3K	0.23508	0.467
275	5-O8W-I9-Serabelisib-PI3Kalpha selective inhibitor	100	PI3K	0.275892	0.176
276	5-O8W-I11-GSK2636771-PI3K beta selective inhibitor	10	PI3K	0.360925	0.002
277	5-O8W-J9-Serabelisib-PI3Kalpha selective inhibitor	10	PI3K	0.114484	0.981
278	5-O8W-J11-GSK2636771-PI3K beta selective inhibitor	1	PI3K	0.119126	0.682
279	5-O8W-K9-Serabelisib-PI3Kalpha selective inhibitor	1	PI3K	0.108265	0.873
280	5-O8W-L14-AZD-8186-PI3Kbeta inhibitor	0.1	PI3K	0.400816	0.006
281	5-O8W-L20-ZSTK474-PI3K gamma selective inhibitor	1	PI3K	0.410951	0
282	5-O8W-L23-Omipalisib-PI3K/mTOR inhibitor	0.1	PI3K	0.210536	0.162
283	5-O8W-M14-AZD-8186-PI3Kbeta inhibitor	1	PI3K	0.31033	0.058
284	5-O8W-M20-ZSTK474-PI3K gamma selective inhibitor	10	PI3K	0.411309	0.001
285	5-O8W-M23-Omipalisib-PI3K/mTOR inhibitor	1	PI3K	0.338066	0.003
286	5-O8W-N14-AZD-8186-PI3Kbeta inhibitor	10	PI3K	0.390814	0.001
287	5-O8W-N20-ZSTK474-PI3K gamma selective inhibitor	100	PI3K	0.193029	0.114
288	5-O8W-N23-Omipalisib-PI3K/mTOR inhibitor	10	PI3K	0.459559	0
289	5-O8W-O14-AZD-8186-PI3Kbeta inhibitor	100	PI3K	0.379387	0.007
290	5-O8W-O20-ZSTK474-PI3K gamma selective inhibitor	1000	PI3K	0.413191	0
291	5-O8W-O23-Omipalisib-PI3K/mTOR inhibitor	100	PI3K	0.297671	0.07
292	5-O8W-P14-AZD-8186-PI3Kbeta inhibitor	1000	PI3K	0.196401	0.076
293	5-O8W-P20-ZSTK474-PI3K gamma selective inhibitor	10000	PI3K	0.465131	0
294	5-O8W-P23-Omipalisib-PI3K/mTOR inhibitor	1000	PI3K	0.237045	0.506
295	6-O8W-A8-TGX-221-PI3K beta selective inhibitor	10000	PI3K	0.178152	0.852
296	6-O8W-B8-TGX-221-PI3K beta selective inhibitor	1000	PI3K	0.116538	0.991
297	6-O8W-C8-TGX-221-PI3K beta selective inhibitor	100	PI3K	0.176164	0.896
298	6-O8W-D8-TGX-221-PI3K beta selective inhibitor	10	PI3K	0.234792	0.441
299	6-O8W-E8-TGX-221-PI3K beta selective inhibitor	1	PI3K	0.228064	0.443
300	6-O8W-L6-GDC-0084-PI3K/mTOR inhibitor	1	PI3K	0.330698	0.019
301	6-O8W-M6-GDC-0084-PI3K/mTOR inhibitor	10	PI3K	0.18668	0.606
302	6-O8W-N6-GDC-0084-PI3K/mTOR inhibitor	100	PI3K	0.277695	0
303	6-O8W-O6-GDC-0084-PI3K/mTOR inhibitor	1000	PI3K	0.34505	0.008
304	6-O8W-P6-GDC-0084-PI3K/mTOR inhibitor	10000	PI3K	0.197986	0.886

305	1-O8W-F11-Amsacrine-DNA intercalation, Topo II inhibitor	10000	Topoisome	0.565872	0
306	1-O8W-G11-Amsacrine-DNA intercalation, Topo II inhibitor	1000	Topoisome	0.568588	0
307	1-O8W-G20-Epirubicin-Topoisomerase II inhibitor	1000	Topoisome	0.610532	0
308	1-O8W-H11-Amsacrine-DNA intercalation, Topo II inhibitor	100	Topoisome	0.546429	0
309	1-O8W-H20-Epirubicin-Topoisomerase II inhibitor	100	Topoisome	0.551403	0
310	1-O8W-I11-Amsacrine-DNA intercalation, Topo II inhibitor	10	Topoisome	0.587002	0
311	1-O8W-I20-Epirubicin-Topoisomerase II inhibitor	10	Topoisome	0.565604	0
312	1-O8W-J11-Amsacrine-DNA intercalation, Topo II inhibitor	1	Topoisome	0.076872	0.96
313	1-O8W-J20-Epirubicin-Topoisomerase II inhibitor	1	Topoisome	0.463576	0
314	1-O8W-K11-SN-38-Active metabolite of irinotecan. Topoisomerase I inhibitor	1	Topoisome	0.543613	0
315	1-O8W-K20-Epirubicin-Topoisomerase II inhibitor	0.1	Topoisome	0.124413	0.976
316	1-O8W-L11-SN-38-Active metabolite of irinotecan. Topoisomerase I inhibitor	10	Topoisome	0.585782	0
317	1-O8W-L14-Topotecan-Topoisomerase I inhibitor. Camptothecin analog	1	Topoisome	0.408282	0.028
318	1-O8W-M11-SN-38-Active metabolite of irinotecan. Topoisomerase I inhibitor	100	Topoisome	0.573839	0
319	1-O8W-M14-Topotecan-Topoisomerase I inhibitor. Camptothecin analog	10	Topoisome	0.556022	0
320	1-O8W-N14-Topotecan-Topoisomerase I inhibitor. Camptothecin analog	100	Topoisome	0.575065	0
321	1-O8W-O11-SN-38-Active metabolite of irinotecan. Topoisomerase I inhibitor	1000	Topoisome	0.196587	0.641
322	1-O8W-O14-Topotecan-Topoisomerase I inhibitor. Camptothecin analog	1000	Topoisome	0.567082	0
323	1-O8W-P11-SN-38-Active metabolite of irinotecan. Topoisomerase I inhibitor	10000	Topoisome	0.560584	0
324	1-O8W-P14-Topotecan-Topoisomerase I inhibitor. Camptothecin analog	10000	Topoisome	0.535986	0
325	3-O8W-A11-Etoposide-Topoisomerase II inhibitor	10000	Topoisome	0.591008	0
326	3-O8W-B11-Etoposide-Topoisomerase II inhibitor	1000	Topoisome	0.552775	0
327	3-O8W-C11-Etoposide-Topoisomerase II inhibitor	100	Topoisome	0.581163	0
328	3-O8W-D11-Etoposide-Topoisomerase II inhibitor	10	Topoisome	0.104709	0.873
329	3-O8W-E11-Etoposide-Topoisomerase II inhibitor	1	Topoisome	0.342116	0.168
330	3-O8W-G9-Daunorubicin-Topoisomerase II inhibitor	1000	Topoisome	0.42956	0.009
331	3-O8W-G10-Teniposide-Topoisomerase II inhibitor	10000	Topoisome	0.633883	0
332	3-O8W-H9-Daunorubicin-Topoisomerase II inhibitor	100	Topoisome	0.433738	0.218
333	3-O8W-H10-Teniposide-Topoisomerase II inhibitor	1000	Topoisome	0.501699	0.005
334	3-O8W-I9-Daunorubicin-Topoisomerase II inhibitor	10	Topoisome	0.525502	0
335	3-O8W-I10-Teniposide-Topoisomerase II inhibitor	100	Topoisome	0.068572	0.992
336	3-O8W-J9-Daunorubicin-Topoisomerase II inhibitor	1	Topoisome	0.495088	0
337	3-O8W-J10-Teniposide-Topoisomerase II inhibitor	10	Topoisome	0.313392	0.097

338	3-O8W-K7-Idarubicin-Topoisomerase II inhibitor	0.1	Topoisome	0.300713	0.12
339	3-O8W-K9-Daunorubicin-Topoisomerase II inhibitor	0.1	Topoisome	0.202843	0.468
340	3-O8W-K10-Teniposide-Topoisomerase II inhibitor	1	Topoisome	0.197243	0.556
341	3-O8W-L6-Doxorubicin-Topoisomerase II inhibitor	0.1	Topoisome	0.471315	0.003
342	3-O8W-L7-Idarubicin-Topoisomerase II inhibitor	1	Topoisome	0.451275	0.002
343	3-O8W-L9-Valrubicin-Topoisomerase II inhibitor	0.5	Topoisome	0.627631	0
344	3-O8W-L10-Mitoxantrone-Topoisomerase II inhibitor	0.1	Topoisome	0.448126	0.004
345	3-O8W-L16-Pixantrone-topoisomerase II inhibitor	1	Topoisome	0.28151	0.633
346	3-O8W-M6-Doxorubicin-Topoisomerase II inhibitor	1	Topoisome	0.53525	0
347	3-O8W-M7-Idarubicin-Topoisomerase II inhibitor	10	Topoisome	0.537129	0
348	3-O8W-M9-Valrubicin-Topoisomerase II inhibitor	5	Topoisome	0.49826	0
349	3-O8W-M10-Mitoxantrone-Topoisomerase II inhibitor	1	Topoisome	0.510023	0
350	3-O8W-M16-Pixantrone-topoisomerase II inhibitor	10	Topoisome	0.495303	0
351	3-O8W-N6-Doxorubicin-Topoisomerase II inhibitor	10	Topoisome	0.472841	0
352	3-O8W-N9-Valrubicin-Topoisomerase II inhibitor	50	Topoisome	0.515495	0
353	3-O8W-N10-Mitoxantrone-Topoisomerase II inhibitor	10	Topoisome	0.539932	0
354	3-O8W-N16-Pixantrone-topoisomerase II inhibitor	100	Topoisome	0.052054	1
355	3-O8W-O6-Doxorubicin-Topoisomerase II inhibitor	100	Topoisome	0.565439	0
356	3-O8W-O7-Idarubicin-Topoisomerase II inhibitor	100	Topoisome	0.598878	0
357	3-O8W-O9-Valrubicin-Topoisomerase II inhibitor	500	Topoisome	0.615293	0
358	3-O8W-O10-Mitoxantrone-Topoisomerase II inhibitor	100	Topoisome	0.598326	0
359	3-O8W-O16-Pixantrone-topoisomerase II inhibitor	1000	Topoisome	0.12558	0.865
360	3-O8W-P6-Doxorubicin-Topoisomerase II inhibitor	1000	Topoisome	0.578889	0
361	3-O8W-P7-Idarubicin-Topoisomerase II inhibitor	1000	Topoisome	0.499045	0
362	3-O8W-P9-Valrubicin-Topoisomerase II inhibitor	5000	Topoisome	0.507103	0.002
363	3-O8W-P10-Mitoxantrone-Topoisomerase II inhibitor	1000	Topoisome	0.52101	0
364	3-O8W-P16-Pixantrone-topoisomerase II inhibitor	10000	Topoisome	0.539627	0
365	1-O8W-A10-Vinorelbine-Mitotic inhibitor. Vinca alkaloid microtubule depolymerizer	10000	Mitotic	0.731953	0
366	1-O8W-A13-Ixabepilone-Mitotic inhibitor. Epothilone microtubule stabilizer.	1000	Mitotic	0.632394	0
367	1-O8W-A18-Paclitaxel-Mitotic inhibitor, taxane microtubule stabilizer	1000	Mitotic	0.708218	0
368	1-O8W-B10-Vinorelbine-Mitotic inhibitor. Vinca alkaloid microtubule depolymerizer	1000	Mitotic	0.680982	0
369	1-O8W-B13-Ixabepilone-Mitotic inhibitor. Epothilone microtubule stabilizer.	100	Mitotic	0.38654	0.035
370	1-O8W-B18-Paclitaxel-Mitotic inhibitor, taxane microtubule stabilizer	100	Mitotic	0.655939	0

371	1-O8W-C10-Vinorelbine-Mitotic inhibitor. Vinca alkaloid microtubule depolymerizer	100	Mitotic	0.618087	0
372	1-O8W-C13-Ixabepilone-Mitotic inhibitor. Epothilone microtubule stabilizer.	10	Mitotic	0.59062	0
373	1-O8W-C18-Paclitaxel-Mitotic inhibitor, taxane microtubule stabilizer	10	Mitotic	0.645578	0
374	1-O8W-D10-Vinorelbine-Mitotic inhibitor. Vinca alkaloid microtubule depolymerizer	10	Mitotic	0.692171	0
375	1-O8W-D13-Ixabepilone-Mitotic inhibitor. Epothilone microtubule stabilizer.	1	Mitotic	0.253483	0.184
376	1-O8W-D18-Paclitaxel-Mitotic inhibitor, taxane microtubule stabilizer	1	Mitotic	0.483415	0
377	1-O8W-E10-Vinorelbine-Mitotic inhibitor. Vinca alkaloid microtubule depolymerizer	1	Mitotic	0.219999	0.522
378	1-O8W-E13-Ixabepilone-Mitotic inhibitor. Epothilone microtubule stabilizer.	0.1	Mitotic	0.134974	0.677
379	1-O8W-E18-Paclitaxel-Mitotic inhibitor, taxane microtubule stabilizer	0.1	Mitotic	0.441761	0.009
380	1-O8W-F13-Vinblastine-Mitotic inhibitor. Vinca alkaloid microtubule depolymerizer	1000	Mitotic	0.685818	0
381	1-O8W-G13-Vinblastine-Mitotic inhibitor. Vinca alkaloid microtubule depolymerizer	100	Mitotic	0.638977	0
382	1-O8W-G15-Eribulin-Mitotic inhibitor, microtubule depolymerizer.	1000	Mitotic	0.741301	0
383	1-O8W-H13-Vinblastine-Mitotic inhibitor. Vinca alkaloid microtubule depolymerizer	10	Mitotic	0.535411	0
384	1-O8W-H15-Eribulin-Mitotic inhibitor, microtubule depolymerizer.	100	Mitotic	0.607813	0
385	1-O8W-I13-Vinblastine-Mitotic inhibitor. Vinca alkaloid microtubule depolymerizer	1	Mitotic	0.641026	0
386	1-O8W-I15-Eribulin-Mitotic inhibitor, microtubule depolymerizer.	10	Mitotic	0.614666	0
387	1-O8W-J13-Vinblastine-Mitotic inhibitor. Vinca alkaloid microtubule depolymerizer	0.1	Mitotic	0.14926	0.709
388	1-O8W-J15-Eribulin-Mitotic inhibitor, microtubule depolymerizer.	1	Mitotic	0.627747	0
389	1-O8W-K7-Vincristine-Mitotic inhibitor. Vinca alkaloid microtubule depolymerizer	0.1	Mitotic	0.541498	0.001
390	1-O8W-K15-Eribulin-Mitotic inhibitor, microtubule depolymerizer.	0.1	Mitotic	0.160299	0.91
391	1-O8W-L7-Vincristine-Mitotic inhibitor. Vinca alkaloid microtubule depolymerizer	1	Mitotic	0.207136	0.53
392	1-O8W-L20-Vinflunine-Mitotic inhibitor. Vinca alkaloid microtubule depolymerizer	0.1	Mitotic	0.145928	0.95
393	1-O8W-M7-Vincristine-Mitotic inhibitor. Vinca alkaloid microtubule depolymerizer	10	Mitotic	0.395861	0.03
394	1-O8W-M20-Vinflunine-Mitotic inhibitor. Vinca alkaloid microtubule depolymerizer	1	Mitotic	0.509744	0.002
395	1-O8W-N20-Vinflunine-Mitotic inhibitor. Vinca alkaloid microtubule depolymerizer	10	Mitotic	0.381341	0.083
396	1-O8W-O7-Vincristine-Mitotic inhibitor. Vinca alkaloid microtubule depolymerizer	100	Mitotic	0.572277	0
397	1-O8W-O20-Vinflunine-Mitotic inhibitor. Vinca alkaloid microtubule depolymerizer	100	Mitotic	0.419758	0.032
398	1-O8W-P7-Vincristine-Mitotic inhibitor. Vinca alkaloid microtubule depolymerizer	1000	Mitotic	0.688266	0
399	1-O8W-P20-Vinflunine-Mitotic inhibitor. Vinca alkaloid microtubule depolymerizer	1000	Mitotic	0.645536	0
400	3-O8W-A7-Docetaxel-Mitotic inhibitor, taxane microtubule stabilizer	1000	Mitotic	0.678057	0
401	3-O8W-B7-Docetaxel-Mitotic inhibitor, taxane microtubule stabilizer	100	Mitotic	0.643932	0
402	3-O8W-C7-Docetaxel-Mitotic inhibitor, taxane microtubule stabilizer	10	Mitotic	0.633611	0
403	3-O8W-D7-Docetaxel-Mitotic inhibitor, taxane microtubule stabilizer	1	Mitotic	0.63247	0

404	3-O8W-E7-Docetaxel-Mitotic inhibitor, taxane microtubule stabilizer	0.1	Mitotic	0.525659	0
405	6-O8W-L19-ABT-751-Mitotic inhibitor. Colchicine site binding microtubule depolymerizer.	1	Mitotic	0.220894	0.838
406	6-O8W-M19-ABT-751-Mitotic inhibitor. Colchicine site binding microtubule depolymerizer.	10	Mitotic	0.320668	0.342
407	6-O8W-N19-ABT-751-Mitotic inhibitor. Colchicine site binding microtubule depolymerizer.	100	Mitotic	0.158717	0.965
408	6-O8W-O19-ABT-751-Mitotic inhibitor. Colchicine site binding microtubule depolymerizer.	1000	Mitotic	0.622159	0
409	6-O8W-P19-ABT-751-Mitotic inhibitor. Colchicine site binding microtubule depolymerizer.	10000	Mitotic	0.626635	0
410	2-O8W-A12-Trametinib-MEK1/2 inhibitor	250	MEK1/2	0.568653	0.001
411	2-O8W-B12-Trametinib-MEK1/2 inhibitor	25	MEK1/2	0.762942	0
412	2-O8W-D12-Trametinib-MEK1/2 inhibitor	2.5	MEK1/2	0.874249	0
413	2-O8W-E12-Trametinib-MEK1/2 inhibitor	0.25	MEK1/2	0.739676	0
414	2-O8W-F12-Trametinib-MEK1/2 inhibitor	2.5000000	MEK1/2	0.376692	0.092
415	2-O8W-F14-Cobimetinib-MEK1/2 inhibitor	1000	MEK1/2	0.637856	0
416	2-O8W-G14-Cobimetinib-MEK1/2 inhibitor	100	MEK1/2	0.76737	0
417	2-O8W-H14-Cobimetinib-MEK1/2 inhibitor	10	MEK1/2	0.761251	0
418	2-O8W-I14-Cobimetinib-MEK1/2 inhibitor	1	MEK1/2	0.559418	0
419	2-O8W-K14-Cobimetinib-MEK1/2 inhibitor	0.1	MEK1/2	0.711773	0
420	2-O8W-L20-Selumetinib-MEK1/2 inhibitor	1	MEK1/2	0.245975	0.815
421	2-O8W-M20-Selumetinib-MEK1/2 inhibitor	10	MEK1/2	0.28632	0.58
422	2-O8W-N20-Selumetinib-MEK1/2 inhibitor	100	MEK1/2	0.587436	0
423	2-O8W-O20-Selumetinib-MEK1/2 inhibitor	1000	MEK1/2	0.679589	0
424	2-O8W-P20-Selumetinib-MEK1/2 inhibitor	10000	MEK1/2	0.773904	0
425	4-O8W-A10-Binimetinib-MEK1/2 inhibitor	1000	MEK1/2	0.748077	0
426	4-O8W-A13-PD0325901-MEK1/2 inhibitor	1000	MEK1/2	0.766241	0
427	4-O8W-B10-Binimetinib-MEK1/2 inhibitor	100	MEK1/2	0.727853	0
428	4-O8W-B13-PD0325901-MEK1/2 inhibitor	100	MEK1/2	0.763821	0
429	4-O8W-C10-Binimetinib-MEK1/2 inhibitor	10	MEK1/2	0.453335	0.035
430	4-O8W-C13-PD0325901-MEK1/2 inhibitor	10	MEK1/2	0.449835	0.003
431	4-O8W-D10-Binimetinib-MEK1/2 inhibitor	1	MEK1/2	0.60006	0
432	4-O8W-D13-PD0325901-MEK1/2 inhibitor	1	MEK1/2	0.288631	0.195
433	4-O8W-E10-Binimetinib-MEK1/2 inhibitor	0.1	MEK1/2	0.316908	0.29
434	4-O8W-E13-PD0325901-MEK1/2 inhibitor	0.1	MEK1/2	0.483767	0.017
435	4-O8W-L19-GDC-0623-MEK1/2 inhibitor	0.25	MEK1/2	0.439656	0.102
436	4-O8W-M19-GDC-0623-MEK1/2 inhibitor	2.5	MEK1/2	0.378429	0.236

437	4-O8W-N19-GDC-0623-MEK1/2 inhibitor	25	MEK1/2	0.486088	0.036
438	4-O8W-O19-GDC-0623-MEK1/2 inhibitor	250	MEK1/2	0.504913	0.007
439	4-O8W-P19-GDC-0623-MEK1/2 inhibitor	2500	MEK1/2	0.640649	0
440	1-O8W-L2-Olaparib-PARP inhibitor	1	PARP	0.557138	0.006
441	1-O8W-L6-Rucaparib-PARP inhibitor	1	PARP	0.341363	0.46
442	1-O8W-M2-Olaparib-PARP inhibitor	10	PARP	0.722778	0
443	1-O8W-M6-Rucaparib-PARP inhibitor	10	PARP	0.401817	0.236
444	1-O8W-N2-Olaparib-PARP inhibitor	100	PARP	0.602182	0
445	1-O8W-N6-Rucaparib-PARP inhibitor	100	PARP	0.440775	0.163
446	1-O8W-O2-Olaparib-PARP inhibitor	1000	PARP	0.559612	0.011
447	1-O8W-O6-Rucaparib-PARP inhibitor	1000	PARP	0.596637	0.002
448	1-O8W-P2-Olaparib-PARP inhibitor	10000	PARP	0.616211	0
449	1-O8W-P6-Rucaparib-PARP inhibitor	10000	PARP	0.580486	0.001
450	7-O8W-A3-Talazoparib-PARP1/2 inhibitor	1000	PARP	0.606807	0
451	7-O8W-B2-Veliparib-PARP inhibitor	10000	PARP	0.581313	0.002
452	7-O8W-B3-Talazoparib-PARP1/2 inhibitor	100	PARP	0.593615	0.001
453	7-O8W-C2-Veliparib-PARP inhibitor	1000	PARP	0.810511	0
454	7-O8W-C3-Talazoparib-PARP1/2 inhibitor	10	PARP	0.567577	0.003
455	7-O8W-D2-Veliparib-PARP inhibitor	100	PARP	0.576092	0.009
456	7-O8W-D3-Talazoparib-PARP1/2 inhibitor	1	PARP	0.538043	0.004
457	7-O8W-E2-Veliparib-PARP inhibitor	10	PARP	0.198396	0.95
458	7-O8W-E3-Talazoparib-PARP1/2 inhibitor	0.1	PARP	0.435287	0.222
459	7-O8W-F2-Veliparib-PARP inhibitor	1	PARP	0.082766	1
460	7-O8W-G2-Niraparib-PARP inhibitor	10000	PARP	0.577939	0.002
461	7-O8W-H2-Niraparib-PARP inhibitor	1000	PARP	0.615128	0.002
462	7-O8W-I2-Niraparib-PARP inhibitor	100	PARP	0.584273	0
463	7-O8W-J2-Niraparib-PARP inhibitor	10	PARP	0.265021	0.718
464	7-O8W-K2-Niraparib-PARP inhibitor	1	PARP	0.194861	0.983
465	3-O8W-A19-Dinaciclib-CDK inhibitor	1000	CDK	0.329194	0.233
466	3-O8W-B19-Dinaciclib-CDK inhibitor	100	CDK	0.373307	0.196
467	3-O8W-B23-Abemaciclib-CDK4/6 inhibitor	2500	CDK	0.443259	0
468	3-O8W-C19-Dinaciclib-CDK inhibitor	10	CDK	0.444284	0.003
469	3-O8W-C23-Abemaciclib-CDK4/6 inhibitor	250	CDK	0.430287	0.019

**470**	3-O8W-D19-Dinaciclib-CDK inhibitor	1	CDK	0.204483	0.491
**471**	3-O8W-D23-Abemaciclib-CDK4/6 inhibitor	25	CDK	0.164566	0.711
**472**	3-O8W-E19-Dinaciclib-CDK inhibitor	0.1	CDK	0.441004	0.04
**473**	3-O8W-E23-Abemaciclib-CDK4/6 inhibitor	2.5	CDK	0.220208	0.537
**474**	3-O8W-F23-Abemaciclib-CDK4/6 inhibitor	0.25	CDK	0.102389	0.924
**475**	3-O8W-K17-Palbociclib-CDK4/6 inhibitor	1	CDK	0.468516	0
**476**	3-O8W-L19-Ribociclib-CDK4/6 inhibitor	1	CDK	0.367231	0.07
**477**	3-O8W-M17-Palbociclib-CDK4/6 inhibitor	10	CDK	0.404944	0.045
**478**	3-O8W-M19-Ribociclib-CDK4/6 inhibitor	10	CDK	0.432559	0.034
**479**	3-O8W-N17-Palbociclib-CDK4/6 inhibitor	100	CDK	0.456151	0.001
**480**	3-O8W-N19-Ribociclib-CDK4/6 inhibitor	100	CDK	0.416683	0.01
**481**	3-O8W-O17-Palbociclib-CDK4/6 inhibitor	1000	CDK	0.426505	0.014
**482**	3-O8W-O19-Ribociclib-CDK4/6 inhibitor	1000	CDK	0.48037	0
**483**	3-O8W-P17-Palbociclib-CDK4/6 inhibitor	10000	CDK	0.407725	0.013
**484**	3-O8W-P19-Ribociclib-CDK4/6 inhibitor	10000	CDK	0.398959	0.014
**485**	4-O8W-A4-SNS-032-CDK inhibitor	10000	CDK	0.386331	0.107
**486**	4-O8W-A8-Milciclib-CDK2 inhibitor	10000	CDK	0.374385	0.133
**487**	4-O8W-B4-SNS-032-CDK inhibitor	1000	CDK	0.394941	0.069
**488**	4-O8W-B8-Milciclib-CDK2 inhibitor	1000	CDK	0.081244	0.961
**489**	4-O8W-C4-SNS-032-CDK inhibitor	100	CDK	0.409918	0.006
**490**	4-O8W-C8-Milciclib-CDK2 inhibitor	100	CDK	0.148535	0.81
**491**	4-O8W-D4-SNS-032-CDK inhibitor	10	CDK	0.35936	0.079
**492**	4-O8W-D8-Milciclib-CDK2 inhibitor	10	CDK	0.096451	0.934
**493**	4-O8W-E4-SNS-032-CDK inhibitor	1	CDK	0.414799	0.005
**494**	4-O8W-E8-Milciclib-CDK2 inhibitor	1	CDK	0.280366	0.415
**495**	4-O8W-F4-Seliciclib-CDK2/7/9 inhibitor	10000	CDK	0.417264	0.014
**496**	4-O8W-F22-Alvocidib-CDK inhibitor	10000	CDK	0.380774	0.075
**497**	4-O8W-G4-Seliciclib-CDK2/7/9 inhibitor	1000	CDK	0.408327	0.003
**498**	4-O8W-G22-Alvocidib-CDK inhibitor	1000	CDK	0.357621	0.429
**499**	4-O8W-H4-Seliciclib-CDK2/7/9 inhibitor	100	CDK	0.375292	0.119
**500**	4-O8W-H22-Alvocidib-CDK inhibitor	100	CDK	0.404842	0.059
**501**	4-O8W-I4-Seliciclib-CDK2/7/9 inhibitor	10	CDK	0.404111	0.028
**502**	4-O8W-I22-Alvocidib-CDK inhibitor	10	CDK	0.383069	0.064

503	4-O8W-J4-Seliciclib-CDK2/7/9 inhibitor	1	CDK	0.236117	0.83
504	4-O8W-J22-Alvocidib-CDK inhibitor	1	CDK	0.410915	0.018
505	5-O8W-A19-AZD-5438-CDK1,2,9 inhibitor	10000	CDK	0.15824	0.946
506	5-O8W-B19-AZD-5438-CDK1,2,9 inhibitor	1000	CDK	0.370126	0.065
507	5-O8W-C19-AZD-5438-CDK1,2,9 inhibitor	100	CDK	0.391002	0.021
508	5-O8W-D19-AZD-5438-CDK1,2,9 inhibitor	10	CDK	0.390299	0.019
509	5-O8W-E19-AZD-5438-CDK1,2,9 inhibitor	1	CDK	0.349182	0.103
510	5-O8W-K17-AT7519-CDK1, 2, 4, 6 and 9 inhibitor	1	CDK	0.16357	0.691
511	5-O8W-M17-AT7519-CDK1, 2, 4, 6 and 9 inhibitor	10	CDK	0.312723	0.057
512	5-O8W-N17-AT7519-CDK1, 2, 4, 6 and 9 inhibitor	100	CDK	0.40873	0.011
513	5-O8W-O17-AT7519-CDK1, 2, 4, 6 and 9 inhibitor	1000	CDK	0.281129	0.424
514	5-O8W-P17-AT7519-CDK1, 2, 4, 6 and 9 inhibitor	10000	CDK	0.408587	0.072
515	6-O8W-A17-Senexin B-CDK8/19 inhibitor	1000	CDK	0.386714	0.031
516	6-O8W-B17-Senexin B-CDK8/19 inhibitor	100	CDK	0.410524	0.17
517	6-O8W-C17-Senexin B-CDK8/19 inhibitor	10	CDK	0.346923	0.082
518	6-O8W-D17-Senexin B-CDK8/19 inhibitor	1	CDK	0.318164	0.254
519	6-O8W-E17-Senexin B-CDK8/19 inhibitor	0.1	CDK	0.160902	0.682
520	6-O8W-L15-THZ2-CDK7 inhibitor	1	CDK	0.203188	0.917
521	6-O8W-M15-THZ2-CDK7 inhibitor	10	CDK	0.346839	0.082
522	6-O8W-N15-THZ2-CDK7 inhibitor	100	CDK	0.387929	0.008
523	6-O8W-O15-THZ2-CDK7 inhibitor	1000	CDK	0.420671	0.114
524	6-O8W-P15-THZ2-CDK7 inhibitor	10000	CDK	0.473914	0.023
525	7-O8W-A21-dBET1-BET-targeting PROTAC	10000	BET	0.473993	0.005
526	7-O8W-A22-PFI-1-BET family inhibitor	30000	BET	0.607344	0
527	7-O8W-B21-dBET1-BET-targeting PROTAC	1000	BET	0.475535	0.001
528	7-O8W-B22-PFI-1-BET family inhibitor	3000	BET	0.621011	0
529	7-O8W-C21-dBET1-BET-targeting PROTAC	100	BET	0.52079	0
530	7-O8W-C22-PFI-1-BET family inhibitor	300	BET	0.5027	0.001
531	7-O8W-D21-dBET1-BET-targeting PROTAC	10	BET	0.238798	0.421
532	7-O8W-D22-PFI-1-BET family inhibitor	30	BET	0.555164	0
533	7-O8W-E21-dBET1-BET-targeting PROTAC	1	BET	0.358894	0.035
534	7-O8W-E22-PFI-1-BET family inhibitor	3	BET	0.455024	0.002
535	7-O8W-G10-Birabresib-BET family inhibitor	10000	BET	0.521047	0

536	7-O8W-G15-I-BET151-BET family inhibitor	10000	BET	0.522055	0
537	7-O8W-H10-Birabresib-BET family inhibitor	1000	BET	0.632987	0
538	7-O8W-H15-I-BET151-BET family inhibitor	1000	BET	0.603932	0
539	7-O8W-I10-Birabresib-BET family inhibitor	100	BET	0.618424	0
540	7-O8W-I15-I-BET151-BET family inhibitor	100	BET	0.638493	0
541	7-O8W-J10-Birabresib-BET family inhibitor	10	BET	0.43895	0.052
542	7-O8W-J15-I-BET151-BET family inhibitor	10	BET	0.335891	0.124
543	7-O8W-K10-Birabresib-BET family inhibitor	1	BET	0.313503	0.627
544	7-O8W-K13-Mivebresib-BET family inhibitor	1	BET	0.217611	0.443
545	7-O8W-K15-I-BET151-BET family inhibitor	1	BET	0.456847	0.003
546	7-O8W-L12-Molibresib-BET family inhibitor	1	BET	0.447448	0.03
547	7-O8W-L13-Mivebresib-BET family inhibitor	10	BET	0.638096	0
548	7-O8W-L20-JQ1-BET family inhibitor	1	BET	0.424291	0.014
549	7-O8W-L23-ARV-825-BET-targeting PROTAC	0.03	BET	0.444724	0.013
550	7-O8W-M12-Molibresib-BET family inhibitor	10	BET	0.181489	0.654
551	7-O8W-M13-Mivebresib-BET family inhibitor	100	BET	0.518358	0
552	7-O8W-M20-JQ1-BET family inhibitor	10	BET	0.377463	0.094
553	7-O8W-M23-ARV-825-BET-targeting PROTAC	0.3	BET	0.457347	0.004
554	7-O8W-N12-Molibresib-BET family inhibitor	100	BET	0.120375	0.936
555	7-O8W-N13-Mivebresib-BET family inhibitor	1000	BET	0.518429	0
556	7-O8W-N20-JQ1-BET family inhibitor	100	BET	0.696858	0
557	7-O8W-N23-ARV-825-BET-targeting PROTAC	3	BET	0.607611	0
558	7-O8W-O12-Molibresib-BET family inhibitor	1000	BET	0.649961	0
559	7-O8W-O20-JQ1-BET family inhibitor	1000	BET	0.549667	0
560	7-O8W-O23-ARV-825-BET-targeting PROTAC	30	BET	0.466062	0.003
561	7-O8W-P12-Molibresib-BET family inhibitor	10000	BET	0.498508	0.001
562	7-O8W-P13-Mivebresib-BET family inhibitor	10000	BET	0.223115	0.979
563	7-O8W-P20-JQ1-BET family inhibitor	10000	BET	0.541758	0
564	7-O8W-P23-ARV-825-BET-targeting PROTAC	300	BET	0.38645	0.137
565	8-O8W-K22-CPI-0610-BET family inhibitor	1	BET	0.538829	0.001
566	8-O8W-L22-CPI-0610-BET family inhibitor	10	BET	0.431016	0.01
567	8-O8W-M22-CPI-0610-BET family inhibitor	100	BET	0.32988	0.097
568	8-O8W-N22-CPI-0610-BET family inhibitor	1000	BET	0.563707	0

569	8-O8W-O22-CPI-0610-BET family inhibitor	10000	BET	0.541456	0
570	1-O8W-A3-Vorinostat-HDAC inhibitor	10000	HDAC	0.44457	0.002
571	1-O8W-B3-Vorinostat-HDAC inhibitor	1000	HDAC	0.438646	0
572	1-O8W-C3-Vorinostat-HDAC inhibitor	100	HDAC	0.42509	0
573	1-O8W-D3-Vorinostat-HDAC inhibitor	10	HDAC	0.432983	0.001
574	1-O8W-E3-Vorinostat-HDAC inhibitor	1	HDAC	0.434783	0.003
575	1-O8W-L12-Romidepsin-HDAC inhibitor	0.1	HDAC	0.501139	0.01
576	1-O8W-M12-Romidepsin-HDAC inhibitor	1	HDAC	0.345236	0.014
577	1-O8W-N12-Romidepsin-HDAC inhibitor	10	HDAC	0.429324	0
578	1-O8W-O12-Romidepsin-HDAC inhibitor	100	HDAC	0.350161	0.01
579	1-O8W-P12-Romidepsin-HDAC inhibitor	1000	HDAC	0.386216	0.017
580	3-O8W-A4-Panobinostat-HDAC inhibitor	1000	HDAC	0.485515	0.003
581	3-O8W-B4-Panobinostat-HDAC inhibitor	100	HDAC	0.375728	0.008
582	3-O8W-C4-Panobinostat-HDAC inhibitor	10	HDAC	0.41598	0
583	3-O8W-D4-Panobinostat-HDAC inhibitor	1	HDAC	0.469799	0
584	3-O8W-E4-Panobinostat-HDAC inhibitor	0.1	HDAC	0.469868	0
585	3-O8W-F7-Quisinostat-HDAC inhibitor	1000	HDAC	0.429491	0.005
586	3-O8W-G7-Quisinostat-HDAC inhibitor	100	HDAC	0.421533	0
587	3-O8W-G12-Valproic acid-HDAC inhibitor	1000000	HDAC	0.085317	0.928
588	3-O8W-H7-Quisinostat-HDAC inhibitor	10	HDAC	0.41762	0
589	3-O8W-H12-Valproic acid-HDAC inhibitor	100000	HDAC	0.348493	0.015
590	3-O8W-I7-Quisinostat-HDAC inhibitor	1	HDAC	0.356459	0.004
591	3-O8W-I12-Valproic acid-HDAC inhibitor	10000	HDAC	0.134103	0.738
592	3-O8W-J7-Quisinostat-HDAC inhibitor	0.1	HDAC	0.401643	0
593	3-O8W-J12-Valproic acid-HDAC inhibitor	1000	HDAC	0.100028	0.853
594	3-O8W-K3-Belinostat-HDAC inhibitor	1	HDAC	0.414083	0.005
595	3-O8W-K12-Valproic acid-HDAC inhibitor	100	HDAC	0.207566	0.206
596	3-O8W-L3-Belinostat-HDAC inhibitor	10	HDAC	0.204119	0.419
597	3-O8W-M3-Belinostat-HDAC inhibitor	100	HDAC	0.417604	0
598	3-O8W-N3-Belinostat-HDAC inhibitor	1000	HDAC	0.423261	0
599	3-O8W-O3-Belinostat-HDAC inhibitor	10000	HDAC	0.430444	0.009
600	7-O8W-A5-Mocetinostat-HDAC inhibitor (HDAC1 & 2-selective)	10000	HDAC	0.420293	0
601	7-O8W-A7-CUDC-907-HDAC1/2/3/10, PI3Kalpha inhibitor	10000	HDAC	0.441411	0.001

**602**	7-O8W-A9-Givinostat-HDAC inhibitor	1000	HDAC	0.390979	0.004
**603**	7-O8W-A12-Rocilinostat-HDAC-6 selective inhibitor	10000	HDAC	0.441028	0
**604**	7-O8W-B5-Mocetinostat-HDAC inhibitor (HDAC1 & 2-selective)	1000	HDAC	0.460983	0
**605**	7-O8W-B7-CUDC-907-HDAC1/2/3/10, PI3Kalpha inhibitor	1000	HDAC	0.533032	0
**606**	7-O8W-B12-Rocilinostat-HDAC-6 selective inhibitor	1000	HDAC	0.422145	0
**607**	7-O8W-C5-Mocetinostat-HDAC inhibitor (HDAC1 & 2-selective)	100	HDAC	0.482971	0
**608**	7-O8W-C7-CUDC-907-HDAC1/2/3/10, PI3Kalpha inhibitor	100	HDAC	0.419109	0.015
**609**	7-O8W-C9-Givinostat-HDAC inhibitor	100	HDAC	0.423548	0
**610**	7-O8W-D7-CUDC-907-HDAC1/2/3/10, PI3Kalpha inhibitor	10	HDAC	0.448382	0
**611**	7-O8W-D9-Givinostat-HDAC inhibitor	10	HDAC	0.511004	0
**612**	7-O8W-D12-Rocilinostat-HDAC-6 selective inhibitor	100	HDAC	0.058761	0.959
**613**	7-O8W-E5-Mocetinostat-HDAC inhibitor (HDAC1 & 2-selective)	10	HDAC	0.449305	0
**614**	7-O8W-E7-CUDC-907-HDAC1/2/3/10, PI3Kalpha inhibitor	1	HDAC	0.46449	0
**615**	7-O8W-E9-Givinostat-HDAC inhibitor	1	HDAC	0.479255	0
**616**	7-O8W-E12-Rocilinostat-HDAC-6 selective inhibitor	10	HDAC	0.114405	0.749
**617**	7-O8W-F5-Mocetinostat-HDAC inhibitor (HDAC1 & 2-selective)	1	HDAC	0.500388	0
**618**	7-O8W-F7-Resminostat-HDAC1, 3, 6 inhibitor	10000	HDAC	0.432918	0
**619**	7-O8W-F9-Givinostat-HDAC inhibitor	0.1	HDAC	0.472122	0
**620**	7-O8W-F12-Rocilinostat-HDAC-6 selective inhibitor	1	HDAC	0.406381	0.001
**621**	7-O8W-F19-PCI-34051-HDAC8 inhibitor	10000	HDAC	0.463708	0
**622**	7-O8W-G7-Resminostat-HDAC1, 3, 6 inhibitor	1000	HDAC	0.463743	0
**623**	7-O8W-G19-PCI-34051-HDAC8 inhibitor	1000	HDAC	0.261383	0.507
**624**	7-O8W-H7-Resminostat-HDAC1, 3, 6 inhibitor	100	HDAC	0.48068	0
**625**	7-O8W-I7-Resminostat-HDAC1, 3, 6 inhibitor	10	HDAC	0.397578	0
**626**	7-O8W-I19-PCI-34051-HDAC8 inhibitor	100	HDAC	0.23309	0.44
**627**	7-O8W-J7-Resminostat-HDAC1, 3, 6 inhibitor	1	HDAC	0.124655	0.647
**628**	7-O8W-J19-PCI-34051-HDAC8 inhibitor	10	HDAC	0.144562	0.772
**629**	7-O8W-K4-Entinostat-HDAC inhibitor	1	HDAC	0.180902	0.471
**630**	7-O8W-K11-AR-42-HDAC inhibitor	1	HDAC	0.056573	0.935
**631**	7-O8W-K18-Tubacin-HDAC6 inhibitor	1	HDAC	0.108824	0.919
**632**	7-O8W-K19-PCI-34051-HDAC8 inhibitor	1	HDAC	0.115537	0.895
**633**	7-O8W-L2-Tacedinaline-HDAC inhibitor	0.1	HDAC	0.09436	0.997
**634**	7-O8W-L4-Entinostat-HDAC inhibitor	10	HDAC	0.084582	0.992

635	7-O8W-L5-Pracinostat-HDAC inhibitor	1	HDAC	0.125629	0.947
636	7-O8W-L8-Abexinostat-HDAC1-selective inhibitor	1	HDAC	0.440427	0
637	7-O8W-L10-Tucidinostat-HDAC1/2/3/10 inhibitor	1	HDAC	0.318523	0.087
638	7-O8W-L11-AR-42-HDAC inhibitor	10	HDAC	0.405878	0
639	7-O8W-L14-Tubastatin A-HDAC6 inhibitor	1	HDAC	0.094857	0.911
640	7-O8W-L16-RGFP966-HDAC3 inhibitor	1	HDAC	0.085147	0.971
641	7-O8W-L18-Tubacin-HDAC6 inhibitor	10	HDAC	0.102267	0.914
642	7-O8W-M2-Tacedinaline-HDAC inhibitor	1	HDAC	0.260522	0.083
643	7-O8W-M5-Pracinostat-HDAC inhibitor	10	HDAC	0.432393	0
644	7-O8W-M8-Abexinostat-HDAC1-selective inhibitor	10	HDAC	0.424412	0
645	7-O8W-M10-Tucidinostat-HDAC1/2/3/10 inhibitor	10	HDAC	0.448956	0
646	7-O8W-M11-AR-42-HDAC inhibitor	100	HDAC	0.397536	0
647	7-O8W-M14-Tubastatin A-HDAC6 inhibitor	10	HDAC	0.495988	0
648	7-O8W-M16-RGFP966-HDAC3 inhibitor	10	HDAC	0.106859	0.812
649	7-O8W-M18-Tubacin-HDAC6 inhibitor	100	HDAC	0.183836	0.599
650	7-O8W-N2-Tacedinaline-HDAC inhibitor	10	HDAC	0.472931	0
651	7-O8W-N4-Entinostat-HDAC inhibitor	100	HDAC	0.446682	0.001
652	7-O8W-N5-Pracinostat-HDAC inhibitor	100	HDAC	0.436449	0
653	7-O8W-N8-Abexinostat-HDAC1-selective inhibitor	100	HDAC	0.433594	0
654	7-O8W-N10-Tucidinostat-HDAC1/2/3/10 inhibitor	100	HDAC	0.445201	0
655	7-O8W-N14-Tubastatin A-HDAC6 inhibitor	100	HDAC	0.391842	0.001
656	7-O8W-N16-RGFP966-HDAC3 inhibitor	100	HDAC	0.070649	0.996
657	7-O8W-N18-Tubacin-HDAC6 inhibitor	1000	HDAC	0.261445	0.406
658	7-O8W-O2-Tacedinaline-HDAC inhibitor	100	HDAC	0.473269	0
659	7-O8W-O4-Entinostat-HDAC inhibitor	1000	HDAC	0.455717	0
660	7-O8W-O5-Pracinostat-HDAC inhibitor	1000	HDAC	0.388306	0.002
661	7-O8W-O8-Abexinostat-HDAC1-selective inhibitor	1000	HDAC	0.380821	0.001
662	7-O8W-O10-Tucidinostat-HDAC1/2/3/10 inhibitor	1000	HDAC	0.452865	0
663	7-O8W-O11-AR-42-HDAC inhibitor	1000	HDAC	0.446158	0
664	7-O8W-O14-Tubastatin A-HDAC6 inhibitor	1000	HDAC	0.453687	0
665	7-O8W-O16-RGFP966-HDAC3 inhibitor	1000	HDAC	0.482336	0
666	7-O8W-P2-Tacedinaline-HDAC inhibitor	1000	HDAC	0.394825	0
667	7-O8W-P4-Entinostat-HDAC inhibitor	10000	HDAC	0.446107	0

**668**	7-O8W-P5-Pracinostat-HDAC inhibitor	10000	HDAC	0.44447	0.004
**669**	7-O8W-P8-Abexinostat-HDAC1-selective inhibitor	10000	HDAC	0.479177	0
**670**	7-O8W-P10-Tucidinostat-HDAC1/2/3/10 inhibitor	10000	HDAC	0.442314	0
**671**	7-O8W-P11-AR-42-HDAC inhibitor	10000	HDAC	0.463724	0
**672**	7-O8W-P14-Tubastatin A-HDAC6 inhibitor	10000	HDAC	0.449527	0
**673**	7-O8W-P16-RGFP966-HDAC3 inhibitor	10000	HDAC	0.460462	0
**674**	7-O8W-P18-Tubacin-HDAC6 inhibitor	10000	HDAC	0.431614	0
