# Supplementary material for: Evaluating feature extraction in ovarian cancer cell line co-cultures using deep neural networks
Source: Commun Biol. 2025 Feb 25;8:303. doi: 10.1038/s42003-025-07766-w (PMC11862010; doi:10.1038/s42003-025-07766-w)
Supplement: Supplementary file 4 — Supplementary Data 2 [file 42003_2025_7766_MOESM4_ESM.pdf]

	Well_annotati	_concentra	ell_Catego	Highest_ES	Pvalue
**0**	2-KB-A16-G	10000	EGFR	0.302603	0.12
**1**	2-KB-A19-E	10000	EGFR	0.312596	0.067
**2**	2-KB-B19-E	1000	EGFR	0.33745	0.03
**3**	2-KB-C16-G	1000	EGFR	0.326119	0.019
**4**	2-KB-C19-E	100	EGFR	0.456013	0
**5**	2-KB-D16-G	100	EGFR	0.274036	0.195
**6**	2-KB-D19-E	10	EGFR	0.447235	0
**7**	2-KB-E16-G	10	EGFR	0.349625	0.009
**8**	2-KB-E19-E	1	EGFR	0.301001	0.089
**9**	2-KB-F16-G	1	EGFR	0.394769	0
**10**	2-KB-K11-A	0.1	EGFR	0.410376	0.003
**11**	2-KB-L11-A	1	EGFR	0.461668	0
**12**	2-KB-L16-O	0.25	EGFR	0.272643	0.229
**13**	2-KB-L19-L	0.1	EGFR	0.350826	0.007
**14**	2-KB-M11-A	10	EGFR	0.372895	0.003
**15**	2-KB-M16-(	2.5	EGFR	0.386266	0.001
**16**	2-KB-M19-I	1	EGFR	0.34277	0.027
**17**	2-KB-N16-C	25	EGFR	0.314068	0.061
**18**	2-KB-N19-L	10	EGFR	0.178195	0.362
**19**	2-KB-O11-A	100	EGFR	0.381549	0
**20**	2-KB-O16-C	250	EGFR	0.33914	0.027
**21**	2-KB-O19-L	100	EGFR	0.428676	0
**22**	2-KB-P11-A	1000	EGFR	0.308471	0.095
**23**	2-KB-P16-C	2500	EGFR	0.268429	0.283
**24**	2-KB-P19-L	1000	EGFR	0.399531	0.005
**25**	3-KB-F21-R	10000	EGFR	0.162167	0.816
**26**	3-KB-G20-N	1000	EGFR	0.312888	0.046
**27**	3-KB-G21-R	1000	EGFR	0.171171	0.466
**28**	3-KB-H20-N	100	EGFR	0.080838	0.781
**29**	3-KB-H21-R	100	EGFR	0.159686	0.475
**30**	3-KB-I20-N	10	EGFR	0.09409	0.696
**31**	3-KB-I21-R	10	EGFR	0.331332	0.145
**32**	3-KB-J20-N	1	EGFR	0.15811	0.35
**33**	3-KB-J21-R	1	EGFR	0.144451	0.7

**34**	3-KB-K4-Ca	1	EGFR	0.282337	0.022
**35**	3-KB-K18-D	0.1	EGFR	0.122297	0.481
**36**	3-KB-K20-N	0.1	EGFR	0.209264	0.662
**37**	3-KB-L4-Ca	10	EGFR	0.13909	0.546
**38**	3-KB-L18-D	1	EGFR	0.212577	0.076
**39**	3-KB-M18-I	10	EGFR	0.456671	0
**40**	3-KB-N4-Ca	100	EGFR	0.208605	0.622
**41**	3-KB-N18-D	100	EGFR	0.171144	0.328
**42**	3-KB-O4-Ca	1000	EGFR	0.234991	0.506
**43**	3-KB-P4-Ca	10000	EGFR	0.193665	0.715
**44**	3-KB-P18-D	1000	EGFR	0.272317	0.184
**45**	4-KB-F13-Sa	1000	EGFR	0.249089	0.257
**46**	4-KB-G13-S	100	EGFR	0.27621	0.172
**47**	4-KB-G16-V	10000	EGFR	0.280601	0.176
**48**	4-KB-H13-S	10	EGFR	0.340405	0.008
**49**	4-KB-H16-V	1000	EGFR	0.30464	0.093
**50**	4-KB-I13-Sa	1	EGFR	0.413986	0.002
**51**	4-KB-I16-Va	100	EGFR	0.184193	0.291
**52**	4-KB-J13-Sa	0.1	EGFR	0.048607	0.964
**53**	4-KB-J16-Va	10	EGFR	0.163413	0.289
**54**	4-KB-K7-Ico	1	EGFR	0.174799	0.529
**55**	4-KB-K13-T	0.1	EGFR	0.32308	0.105
**56**	4-KB-K16-V	1	EGFR	0.334084	0.022
**57**	4-KB-L7-Ico	10	EGFR	0.197794	0.154
**58**	4-KB-L13-Te	1	EGFR	0.526353	0
**59**	4-KB-M7-Ic	100	EGFR	0.379055	0.03
**60**	4-KB-M13-T	10	EGFR	0.402334	0.001
**61**	4-KB-N13-T	100	EGFR	0.248998	0.39
**62**	4-KB-O7-Ico	1000	EGFR	0.174392	0.401
**63**	4-KB-P7-Ico	10000	EGFR	0.453576	0
**64**	4-KB-P13-T	1000	EGFR	0.285426	0.165
**65**	5-KB-F4-Po	1000	EGFR	0.291355	0.137
**66**	5-KB-F7-AZI	1000	EGFR	0.284946	0.117
**67**	5-KB-G4-Po	100	EGFR	0.337308	0.03
**68**	5-KB-G7-AZ	100	EGFR	0.358565	0.001

**69**	5-KB-H4-Pc	10	EGFR	0.312631	0.049
**70**	5-KB-H7-AZ	10	EGFR	0.089539	0.698
**71**	5-KB-I4-Po:	1	EGFR	0.298905	0.103
**72**	5-KB-I7-AZ[	1	EGFR	0.12833	0.456
**73**	5-KB-J4-Po:	0.1	EGFR	0.365758	0.009
**74**	5-KB-J7-AZI	0.1	EGFR	0.076859	0.911
**75**	5-KB-K7-Ol	0.1	EGFR	0.197547	0.783
**76**	5-KB-L7-Ol	1	EGFR	0.123113	0.808
**77**	5-KB-M7-Ol	10	EGFR	0.267913	0.267
**78**	5-KB-O7-Ol	100	EGFR	0.24886	0.355
**79**	5-KB-P7-Ol	1000	EGFR	0.234918	0.508
**80**	2-KB-A15-L	2500	VEGFR	0.322375	0.028
**81**	2-KB-A17-N	10000	VEGFR	0.281103	0.175
**82**	2-KB-A20-T	10000	VEGFR	0.362897	0.004
**83**	2-KB-B15-L	250	VEGFR	0.287264	0.074
**84**	2-KB-B17-N	1000	VEGFR	0.350475	0.007
**85**	2-KB-B20-T	1000	VEGFR	0.324597	0.034
**86**	2-KB-C15-L	25	VEGFR	0.370086	0.002
**87**	2-KB-C17-N	100	VEGFR	0.317291	0.037
**88**	2-KB-D15-L	2.5	VEGFR	0.437256	0
**89**	2-KB-D17-N	10	VEGFR	0.423337	0
**90**	2-KB-D20-T	100	VEGFR	0.336783	0.006
**91**	2-KB-E17-N	1	VEGFR	0.317826	0.025
**92**	2-KB-E20-Ti	10	VEGFR	0.301042	0.071
**93**	2-KB-F13-A	10000	VEGFR	0.277833	0.129
**94**	2-KB-F15-L	0.25	VEGFR	0.488861	0
**95**	2-KB-F19-R	10000	VEGFR	0.249456	0.338
**96**	2-KB-F20-Ti	1	VEGFR	0.449186	0
**97**	2-KB-F21-V	10000	VEGFR	0.243894	0.354
**98**	2-KB-G10-A	10000	VEGFR	0.330194	0.016
**99**	2-KB-G13-A	1000	VEGFR	0.375966	0.001
**100**	2-KB-G19-R	1000	VEGFR	0.329311	0.021
**101**	2-KB-G21-V	1000	VEGFR	0.243449	0.338
**102**	2-KB-H10-A	1000	VEGFR	0.402421	0.002
**103**	2-KB-H13-A	100	VEGFR	0.310337	0.03

**104**	2-KB-H21-V	100	VEGFR	0.352296	0.014
**105**	2-KB-I10-Ap	100	VEGFR	0.244951	0.023
**106**	2-KB-I13-Ax	10	VEGFR	0.409921	0
**107**	2-KB-I19-Re	100	VEGFR	0.388918	0.039
**108**	2-KB-I21-Va	10	VEGFR	0.152646	0.955
**109**	2-KB-J10-Ap	10	VEGFR	0.425581	0
**110**	2-KB-J13-A	1	VEGFR	0.444238	0
**111**	2-KB-J19-R	10	VEGFR	0.117869	0.608
**112**	2-KB-J21-V	1	VEGFR	0.257918	0.49
**113**	2-KB-K10-A	1	VEGFR	0.109992	0.506
**114**	2-KB-K13-V	0.1	VEGFR	0.300447	0.012
**115**	2-KB-K17-P	1	VEGFR	0.302027	0.001
**116**	2-KB-K19-R	1	VEGFR	0.375853	0.001
**117**	2-KB-L12-S	0.1	VEGFR	0.202408	0.04
**118**	2-KB-L13-V	1	VEGFR	0.174715	0.072
**119**	2-KB-L21-C	0.1	VEGFR	0.304429	0.093
**120**	2-KB-M12-S	1	VEGFR	0.232672	0.098
**121**	2-KB-M13-V	10	VEGFR	0.417203	0
**122**	2-KB-M17-P	10	VEGFR	0.35704	0.004
**123**	2-KB-M21-C	1	VEGFR	0.355456	0.006
**124**	2-KB-N12-S	10	VEGFR	0.351674	0.001
**125**	2-KB-N13-V	100	VEGFR	0.339453	0.014
**126**	2-KB-N17-P	100	VEGFR	0.378642	0.001
**127**	2-KB-N21-C	10	VEGFR	0.175769	0.585
**128**	2-KB-O12-S	100	VEGFR	0.40156	0.011
**129**	2-KB-O17-P	1000	VEGFR	0.357955	0.003
**130**	2-KB-O21-C	100	VEGFR	0.304262	0.073
**131**	2-KB-P12-S	1000	VEGFR	0.311517	0.052
**132**	2-KB-P13-V	1000	VEGFR	0.239554	0.369
**133**	2-KB-P17-P	10000	VEGFR	0.27148	0.222
**134**	2-KB-P21-C	1000	VEGFR	0.313431	0.061
**135**	3-KB-A3-Ca	1000	VEGFR	0.290242	0.155
**136**	3-KB-A6-Fo	1000	VEGFR	0.211093	0.643
**137**	3-KB-A18-L	1000	VEGFR	0.212452	0.058
**138**	3-KB-B3-Ca	100	VEGFR	0.201095	0.169

**139**	3-KB-B6-Fo	100	VEGFR	0.259305	0.337
**140**	3-KB-B18-L	100	VEGFR	0.204584	0.431
**141**	3-KB-C3-Ca	10	VEGFR	0.17655	0.145
**142**	3-KB-C6-Fo	10	VEGFR	0.228982	0.163
**143**	3-KB-C18-L	10	VEGFR	0.126643	0.424
**144**	3-KB-D3-Ca	1	VEGFR	0.165212	0.287
**145**	3-KB-D6-Fo	1	VEGFR	0.104666	0.523
**146**	3-KB-D18-L	1	VEGFR	0.214568	0.408
**147**	3-KB-E3-Cal	0.1	VEGFR	0.141454	0.312
**148**	3-KB-E6-For	0.1	VEGFR	0.13583	0.306
**149**	3-KB-E18-Li	0.1	VEGFR	0.164183	0.836
**150**	3-KB-F18-B	1000	VEGFR	0.246422	0.237
**151**	3-KB-G18-E	100	VEGFR	0.399554	0.011
**152**	3-KB-H18-E	10	VEGFR	0.11005	0.618
**153**	3-KB-I18-Br	1	VEGFR	0.166729	0.542
**154**	3-KB-J18-Br	0.1	VEGFR	0.16639	0.278
**155**	4-KB-A12-E	10000	VEGFR	0.216233	0.584
**156**	4-KB-A15-G	2500	VEGFR	0.273104	0.312
**157**	4-KB-A20-N	10000	VEGFR	0.239641	0.152
**158**	4-KB-B12-E	1000	VEGFR	0.21367	0.575
**159**	4-KB-B15-G	250	VEGFR	0.305679	0.131
**160**	4-KB-B20-N	1000	VEGFR	0.27369	0.231
**161**	4-KB-C15-G	25	VEGFR	0.051479	0.897
**162**	4-KB-D12-E	100	VEGFR	0.365457	0.001
**163**	4-KB-D15-G	2.5	VEGFR	0.226414	0.154
**164**	4-KB-D20-N	100	VEGFR	0.305789	0.094
**165**	4-KB-E12-El	10	VEGFR	0.357682	0
**166**	4-KB-E20-M	10	VEGFR	0.252093	0.353
**167**	4-KB-F12-El	1	VEGFR	0.121732	0.384
**168**	4-KB-F15-G	0.25	VEGFR	0.295259	0.03
**169**	4-KB-F20-M	1	VEGFR	0.304554	0.426
**170**	4-KB-L16-To	1	VEGFR	0.148012	0.307
**171**	4-KB-M16-T	10	VEGFR	0.386554	0.01
**172**	4-KB-N16-T	100	VEGFR	0.142673	0.961
**173**	4-KB-O16-T	1000	VEGFR	0.187635	0.68

**174**	4-KB-P16-T	10000	VEGFR	0.213387	0.555
**175**	2-KB-L10-Ic	1	PI3K	0.15962	0.815
**176**	2-KB-M10-I	10	PI3K	0.235025	0.047
**177**	2-KB-N10-I	100	PI3K	0.270044	0.062
**178**	2-KB-O10-I	1000	PI3K	0.18785	0.656
**179**	2-KB-P10-Ic	10000	PI3K	0.237465	0.339
**180**	3-KB-A16-P	2500	PI3K	0.104152	0.431
**181**	3-KB-C16-P	250	PI3K	0.153326	0.141
**182**	3-KB-D16-F	25	PI3K	0.293584	0.055
**183**	3-KB-E16-P	2.5	PI3K	0.224495	0.071
**184**	3-KB-F16-P	0.25	PI3K	0.259907	0.192
**185**	3-KB-F17-N	100000	PI3K	0.108576	0.988
**186**	3-KB-F19-D	500	PI3K	0.143361	0.984
**187**	3-KB-G17-N	10000	PI3K	0.119199	0.483
**188**	3-KB-G19-D	50	PI3K	0.206634	0.515
**189**	3-KB-H17-N	1000	PI3K	0.114648	0.332
**190**	3-KB-I17-M	100	PI3K	0.085153	0.751
**191**	3-KB-I19-D	5	PI3K	0.128568	0.343
**192**	3-KB-J17-N	10	PI3K	0.137002	0.167
**193**	3-KB-J19-D	0.5	PI3K	0.150822	0.182
**194**	3-KB-K19-D	0.05	PI3K	0.126149	0.269
**195**	3-KB-L8-Pic	1	PI3K	0.128634	0.28
**196**	3-KB-L21-T	0.1	PI3K	0.118697	0.983
**197**	3-KB-M8-Pi	10	PI3K	0.133342	0.964
**198**	3-KB-M21-T	1	PI3K	0.081721	0.814
**199**	3-KB-N8-Pi	100	PI3K	0.198447	0.478
**200**	3-KB-N21-T	10	PI3K	0.099928	0.731
**201**	3-KB-O8-Pi	1000	PI3K	0.242474	0.242
**202**	3-KB-O21-T	100	PI3K	0.083611	0.978
**203**	3-KB-P8-Pic	10000	PI3K	0.217512	0.489
**204**	3-KB-P21-T	1000	PI3K	0.134222	0.223
**205**	4-KB-A19-A	2500	PI3K	0.270275	0.008
**206**	4-KB-B19-A	250	PI3K	0.326282	0
**207**	4-KB-C19-A	25	PI3K	0.151174	0.191
**208**	4-KB-D19-A	2.5	PI3K	0.220758	0.021

**209**	4-KB-E19-A	0.25	PI3K	0.309451	0.034
**210**	4-KB-F14-N	1000	PI3K	0.210699	0.542
**211**	4-KB-G2-TG	2500	PI3K	0.075786	0.946
**212**	4-KB-G5-So	10000	PI3K	0.383942	0
**213**	4-KB-G14-N	100	PI3K	0.326977	0.016
**214**	4-KB-G20-E	10000	PI3K	0.258783	0.194
**215**	4-KB-H2-TG	250	PI3K	0.284432	0.204
**216**	4-KB-H5-So	1000	PI3K	0.103336	0.49
**217**	4-KB-H14-N	10	PI3K	0.341761	0.002
**218**	4-KB-H20-E	1000	PI3K	0.239407	0.294
**219**	4-KB-I2-TGI	25	PI3K	0.251596	0.289
**220**	4-KB-I5-Sor	100	PI3K	0.090671	0.864
**221**	4-KB-I14-N'	1	PI3K	0.169281	0.14
**222**	4-KB-I20-Bu	100	PI3K	0.311903	0.014
**223**	4-KB-J2-TGI	2.5	PI3K	0.162936	0.845
**224**	4-KB-J5-Sor	10	PI3K	0.126153	0.397
**225**	4-KB-J20-Bu	10	PI3K	0.087305	0.945
**226**	4-KB-K2-TG	0.25	PI3K	0.191142	0.176
**227**	4-KB-K4-Da	0.1	PI3K	0.104471	0.796
**228**	4-KB-K5-So	1	PI3K	0.233357	0.21
**229**	4-KB-K14-N	0.1	PI3K	0.14255	0.167
**230**	4-KB-K20-B	1	PI3K	0.363082	0.001
**231**	4-KB-L4-Da	1	PI3K	0.221342	0.499
**232**	4-KB-L14-G	0.1	PI3K	0.132764	0.185
**233**	4-KB-L15-T	1	PI3K	0.335956	0.001
**234**	4-KB-L21-C	0.1	PI3K	0.239587	0.324
**235**	4-KB-M14-(	1	PI3K	0.164652	0.112
**236**	4-KB-M15-T	10	PI3K	0.12144	0.285
**237**	4-KB-M21-(	1	PI3K	0.322971	0.007
**238**	4-KB-N4-Da	10	PI3K	0.331098	0.011
**239**	4-KB-N14-G	10	PI3K	0.385196	0
**240**	4-KB-N15-T	100	PI3K	0.272407	0.21
**241**	4-KB-N21-C	10	PI3K	0.348932	0.008
**242**	4-KB-O4-Da	100	PI3K	0.288489	0.062
**243**	4-KB-O14-G	100	PI3K	0.31383	0.012

**244**	4-KB-O15-T	1000	PI3K	0.295879	0.031
**245**	4-KB-O21-C	100	PI3K	0.292978	0.058
**246**	4-KB-P4-Da	1000	PI3K	0.313641	0.018
**247**	4-KB-P14-G	1000	PI3K	0.297019	0.043
**248**	4-KB-P15-T	10000	PI3K	0.364012	0
**249**	4-KB-P21-C	1000	PI3K	0.265056	0.155
**250**	5-KB-A6-LY	2500	PI3K	0.326014	0.012
**251**	5-KB-A7-AN	1000	PI3K	0.339808	0.003
**252**	5-KB-A16-A	2500	PI3K	0.292229	0.022
**253**	5-KB-A17-P	10000	PI3K	0.19547	0.025
**254**	5-KB-B6-LY	250	PI3K	0.275842	0.128
**255**	5-KB-B7-AN	100	PI3K	0.26707	0.039
**256**	5-KB-B17-P	1000	PI3K	0.309794	0.001
**257**	5-KB-C6-LY	25	PI3K	0.225353	0.527
**258**	5-KB-C7-AN	10	PI3K	0.091821	0.994
**259**	5-KB-C16-A	250	PI3K	0.253988	0.174
**260**	5-KB-C17-P	100	PI3K	0.204451	0.552
**261**	5-KB-D6-LY	2.5	PI3K	0.205016	0.603
**262**	5-KB-D7-AN	1	PI3K	0.104315	0.278
**263**	5-KB-D16-A	25	PI3K	0.312857	0.006
**264**	5-KB-D17-P	10	PI3K	0.202302	0.164
**265**	5-KB-E6-LY	0.25	PI3K	0.065647	0.772
**266**	5-KB-E7-AN	0.1	PI3K	0.120325	0.743
**267**	5-KB-E16-A	2.5	PI3K	0.299056	0.122
**268**	5-KB-E17-P	1	PI3K	0.140439	0.859
**269**	5-KB-F11-G	10000	PI3K	0.336761	0.001
**270**	5-KB-F16-A	0.25	PI3K	0.141127	0.137
**271**	5-KB-G9-Se	10000	PI3K	0.323795	0.006
**272**	5-KB-G11-C	1000	PI3K	0.32439	0.003
**273**	5-KB-H9-Se	1000	PI3K	0.353719	0
**274**	5-KB-H11-C	100	PI3K	0.322688	0.001
**275**	5-KB-I9-Ser	100	PI3K	0.300963	0.003
**276**	5-KB-I11-G	10	PI3K	0.261627	0.006
**277**	5-KB-J9-Ser	10	PI3K	0.277368	0.023
**278**	5-KB-J11-G	1	PI3K	0.158092	0.1

**279**	5-KB-K9-Sei	1	PI3K	0.175215	0.09
**280**	5-KB-L14-A	0.1	PI3K	0.306072	0.042
**281**	5-KB-L20-Z	1	PI3K	0.08303	0.672
**282**	5-KB-L23-O	0.1	PI3K	0.063884	0.956
**283**	5-KB-M14-/	1	PI3K	0.326766	0.003
**284**	5-KB-M20-Z	10	PI3K	0.176411	0.786
**285**	5-KB-M23-(	1	PI3K	0.088696	0.985
**286**	5-KB-N14-A	10	PI3K	0.289416	0.064
**287**	5-KB-N20-Z	100	PI3K	0.309522	0.052
**288**	5-KB-N23-C	10	PI3K	0.296984	0.052
**289**	5-KB-O14-A	100	PI3K	0.265533	0.134
**290**	5-KB-O20-Z	1000	PI3K	0.259542	0.198
**291**	5-KB-O23-C	100	PI3K	0.302053	0.051
**292**	5-KB-P14-A	1000	PI3K	0.240781	0.313
**293**	5-KB-P20-Z	10000	PI3K	0.290171	0.056
**294**	5-KB-P23-C	1000	PI3K	0.226647	0.467
**295**	6-KB-A8-TG	10000	PI3K	0.328643	0.004
**296**	6-KB-B8-TG	1000	PI3K	0.28194	0.02
**297**	6-KB-C8-TG	100	PI3K	0.327139	0.018
**298**	6-KB-D8-TG	10	PI3K	0.116154	0.296
**299**	6-KB-E8-TG	1	PI3K	0.132333	0.551
**300**	6-KB-L6-GD	1	PI3K	0.202511	0.612
**301**	6-KB-M6-GI	10	PI3K	0.251374	0.252
**302**	6-KB-N6-GI	100	PI3K	0.233577	0.387
**303**	6-KB-O6-GI	1000	PI3K	0.288743	0.042
**304**	6-KB-P6-GD	10000	PI3K	0.245333	0.303
**305**	1-KB-F11-A	10000	Topoisome	0.336447	0.063
**306**	1-KB-G11-A	1000	Topoisome	0.368397	0.008
**307**	1-KB-G20-E	1000	Topoisome	0.305594	0.176
**308**	1-KB-H11-A	100	Topoisome	0.400417	0.002
**309**	1-KB-H20-E	100	Topoisome	0.385375	0.004
**310**	1-KB-I11-Ar	10	Topoisome	0.331826	0.051
**311**	1-KB-I20-Ep	10	Topoisome	0.380515	0.009
**312**	1-KB-J11-Ar	1	Topoisome	0.369647	0.023
**313**	1-KB-J20-Ep	1	Topoisome	0.30793	0.144

**314**	1-KB-K11-S	1	Topoisome	0.344291	0.046
**315**	1-KB-K20-E	0.1	Topoisome	0.248579	0.449
**316**	1-KB-L11-SI	10	Topoisome	0.34276	0.073
**317**	1-KB-L14-T	1	Topoisome	0.398546	0.001
**318**	1-KB-M11-S	100	Topoisome	0.330111	0.081
**319**	1-KB-M14-T	10	Topoisome	0.4127	0.002
**320**	1-KB-N14-T	100	Topoisome	0.394546	0.009
**321**	1-KB-O11-S	1000	Topoisome	0.307001	0.175
**322**	1-KB-O14-T	1000	Topoisome	0.314528	0.124
**323**	1-KB-P11-S	10000	Topoisome	0.311818	0.144
**324**	1-KB-P14-T	10000	Topoisome	0.304968	0.165
**325**	3-KB-A11-E	10000	Topoisome	0.42368	0.001
**326**	3-KB-B11-E	1000	Topoisome	0.584814	0
**327**	3-KB-C11-E	100	Topoisome	0.364601	0.017
**328**	3-KB-D11-E	10	Topoisome	0.267658	0.013
**329**	3-KB-E11-E	1	Topoisome	0.582007	0
**330**	3-KB-G9-Da	1000	Topoisome	0.173342	0.237
**331**	3-KB-G10-T	10000	Topoisome	0.304207	0.02
**332**	3-KB-H9-Da	100	Topoisome	0.329639	0.044
**333**	3-KB-H10-T	1000	Topoisome	0.183826	0.149
**334**	3-KB-I9-Dau	10	Topoisome	0.282791	0.008
**335**	3-KB-I10-Te	100	Topoisome	0.291091	0.009
**336**	3-KB-J9-Dau	1	Topoisome	0.209268	0.113
**337**	3-KB-J10-Te	10	Topoisome	0.258213	0.02
**338**	3-KB-K7-Ida	0.1	Topoisome	0.200072	0.171
**339**	3-KB-K9-Da	0.1	Topoisome	0.342715	0.003
**340**	3-KB-K10-T	1	Topoisome	0.182599	0.214
**341**	3-KB-L6-Do	0.1	Topoisome	0.24518	0.019
**342**	3-KB-L7-Ida	1	Topoisome	0.281948	0.087
**343**	3-KB-L9-Val	0.5	Topoisome	0.284039	0.409
**344**	3-KB-L10-M	0.1	Topoisome	0.320822	0.016
**345**	3-KB-L16-P	1	Topoisome	0.21255	0.074
**346**	3-KB-M6-Do	1	Topoisome	0.494041	0
**347**	3-KB-M7-Id	10	Topoisome	0.259881	0.036
**348**	3-KB-M9-Va	5	Topoisome	0.354559	0.002

349	3-KB-M10-I	1	Topoisome	0.329445	0.306
350	3-KB-M16-I	10	Topoisome	0.585157	0
351	3-KB-N6-Dc	10	Topoisome	0.42274	0.006
352	3-KB-N9-Va	50	Topoisome	0.438632	0
353	3-KB-N10-N	10	Topoisome	0.21754	0.652
354	3-KB-N16-P	100	Topoisome	0.454876	0
355	3-KB-O6-Dc	100	Topoisome	0.488692	0
356	3-KB-O7-Ida	100	Topoisome	0.30245	0.16
357	3-KB-O9-Va	500	Topoisome	0.418946	0
358	3-KB-O10-N	100	Topoisome	0.216341	0.714
359	3-KB-O16-P	1000	Topoisome	0.497399	0
360	3-KB-P6-Dc	1000	Topoisome	0.26963	0.378
361	3-KB-P7-Ida	1000	Topoisome	0.280332	0.304
362	3-KB-P9-Va	5000	Topoisome	0.285188	0.291
363	3-KB-P10-N	1000	Topoisome	0.295498	0.216
364	3-KB-P16-P	10000	Topoisome	0.497967	0
365	1-KB-A10-V	10000	Mitotic	0.60708	0
366	1-KB-A13-Ix	1000	Mitotic	0.609055	0
367	1-KB-A18-P	1000	Mitotic	0.572921	0
368	1-KB-B10-V	1000	Mitotic	0.6052	0
369	1-KB-B13-Ix	100	Mitotic	0.600553	0
370	1-KB-B18-P	100	Mitotic	0.578868	0
371	1-KB-C10-V	100	Mitotic	0.590109	0
372	1-KB-C13-Ix	10	Mitotic	0.276927	0.59
373	1-KB-C18-P	10	Mitotic	0.591834	0
374	1-KB-D10-V	10	Mitotic	0.658698	0
375	1-KB-D13-I:	1	Mitotic	0.128354	0.644
376	1-KB-D18-P	1	Mitotic	0.658803	0
377	1-KB-E10-V	1	Mitotic	0.501462	0.001
378	1-KB-E13-Ix	0.1	Mitotic	0.177042	0.341
379	1-KB-E18-P	0.1	Mitotic	0.6188	0
380	1-KB-F13-V	1000	Mitotic	0.59971	0
381	1-KB-G13-V	100	Mitotic	0.582537	0
382	1-KB-G15-E	1000	Mitotic	0.585029	0
383	1-KB-H13-V	10	Mitotic	0.674812	0

**384**	1-KB-H15-E	100	Mitotic	0.594852	0
**385**	1-KB-I13-Vi	1	Mitotic	0.637209	0
**386**	1-KB-I15-Er	10	Mitotic	0.579191	0
**387**	1-KB-J13-Vi	0.1	Mitotic	0.535113	0
**388**	1-KB-J15-Er	1	Mitotic	0.598364	0
**389**	1-KB-K7-Vir	0.1	Mitotic	0.07052	0.926
**390**	1-KB-K15-E	0.1	Mitotic	0.67761	0
**391**	1-KB-L7-Vir	1	Mitotic	0.199166	0.767
**392**	1-KB-L20-V	0.1	Mitotic	0.168896	0.96
**393**	1-KB-M7-Vi	10	Mitotic	0.652172	0
**394**	1-KB-M20-V	1	Mitotic	0.513426	0
**395**	1-KB-N20-V	10	Mitotic	0.63156	0
**396**	1-KB-O7-Vi	100	Mitotic	0.652075	0
**397**	1-KB-O20-V	100	Mitotic	0.561533	0
**398**	1-KB-P7-Vir	1000	Mitotic	0.589522	0
**399**	1-KB-P20-V	1000	Mitotic	0.597174	0
**400**	3-KB-A7-Dc	1000	Mitotic	0.619421	0
**401**	3-KB-B7-Dc	100	Mitotic	0.575272	0
**402**	3-KB-C7-Dc	10	Mitotic	0.569338	0
**403**	3-KB-D7-Dc	1	Mitotic	0.617832	0
**404**	3-KB-E7-Do	0.1	Mitotic	0.043792	1
**405**	6-KB-L19-A	1	Mitotic	0.345791	0.493
**406**	6-KB-M19-A	10	Mitotic	0.32753	0.193
**407**	6-KB-N19-A	100	Mitotic	0.069021	1
**408**	6-KB-O19-A	1000	Mitotic	0.524607	0
**409**	6-KB-P19-A	10000	Mitotic	0.527349	0
**410**	2-KB-A12-T	250	MEK1/2	0.550405	0
**411**	2-KB-B12-T	25	MEK1/2	0.523783	0
**412**	2-KB-D12-T	2.5	MEK1/2	0.605404	0
**413**	2-KB-E12-T	0.25	MEK1/2	0.59536	0
**414**	2-KB-F12-T	2.5000000	MEK1/2	0.446428	0.049
**415**	2-KB-F14-C	1000	MEK1/2	0.539456	0
**416**	2-KB-G14-C	100	MEK1/2	0.544339	0
**417**	2-KB-H14-C	10	MEK1/2	0.590449	0
**418**	2-KB-I14-C	1	MEK1/2	0.568902	0.001

**419**	2-KB-K14-C	0.1	MEK1/2	0.208418	0.605
**420**	2-KB-L20-S	1	MEK1/2	0.077594	1
**421**	2-KB-M20-S	10	MEK1/2	0.641095	0
**422**	2-KB-N20-S	100	MEK1/2	0.651833	0
**423**	2-KB-O20-S	1000	MEK1/2	0.590051	0
**424**	2-KB-P20-S	10000	MEK1/2	0.524945	0.004
**425**	4-KB-A10-B	1000	MEK1/2	0.477329	0.003
**426**	4-KB-A13-P	1000	MEK1/2	0.516357	0.001
**427**	4-KB-B10-B	100	MEK1/2	0.513489	0.001
**428**	4-KB-B13-P	100	MEK1/2	0.491576	0.007
**429**	4-KB-C10-B	10	MEK1/2	0.500036	0
**430**	4-KB-C13-P	10	MEK1/2	0.445684	0.015
**431**	4-KB-D10-E	1	MEK1/2	0.156987	0.634
**432**	4-KB-D13-F	1	MEK1/2	0.545408	0.001
**433**	4-KB-E10-B	0.1	MEK1/2	0.222055	0.362
**434**	4-KB-E13-P	0.1	MEK1/2	0.202583	0.427
**435**	4-KB-L19-G	0.25	MEK1/2	0.387412	0.26
**436**	4-KB-M19-(	2.5	MEK1/2	0.145868	0.699
**437**	4-KB-N19-G	25	MEK1/2	0.205444	0.547
**438**	4-KB-O19-G	250	MEK1/2	0.246528	0.936
**439**	4-KB-P19-G	2500	MEK1/2	0.414622	0.038
**440**	1-KB-L2-Ola	1	PARP	0.321044	0.453
**441**	1-KB-L6-Ru	1	PARP	0.622655	0.002
**442**	1-KB-M2-Ol	10	PARP	0.419812	0.2
**443**	1-KB-M6-Ru	10	PARP	0.446611	0.034
**444**	1-KB-N2-Ol	100	PARP	0.484747	0.157
**445**	1-KB-N6-Ru	100	PARP	0.409805	0.071
**446**	1-KB-O2-Ol	1000	PARP	0.444845	0.036
**447**	1-KB-O6-Ru	1000	PARP	0.438507	0.045
**448**	1-KB-P2-Ola	10000	PARP	0.454334	0.018
**449**	1-KB-P6-Ru	10000	PARP	0.422965	0.072
**450**	7-KB-A3-Tal	1000	PARP	0.555981	0.006
**451**	7-KB-B2-Ve	10000	PARP	0.652248	0
**452**	7-KB-B3-Tal	100	PARP	0.668396	0
**453**	7-KB-C2-Ve	1000	PARP	0.597615	0.004

**454**	7-KB-C3-Tal	10	PARP	0.257377	0.626
**455**	7-KB-D2-Ve	100	PARP	0.477717	0.124
**456**	7-KB-D3-Ta	1	PARP	0.618628	0.004
**457**	7-KB-E2-Vel	10	PARP	0.135998	1
**458**	7-KB-E3-Tal	0.1	PARP	0.629831	0.001
**459**	7-KB-F2-Vel	1	PARP	0.677383	0
**460**	7-KB-G2-Ni	10000	PARP	0.561487	0.002
**461**	7-KB-H2-Ni	1000	PARP	0.649465	0
**462**	7-KB-I2-Nir	100	PARP	0.66236	0
**463**	7-KB-J2-Nir	10	PARP	0.539476	0.002
**464**	7-KB-K2-Ni	1	PARP	0.515996	0.009
**465**	3-KB-A19-D	1000	CDK	0.41895	0.005
**466**	3-KB-B19-D	100	CDK	0.408769	0.003
**467**	3-KB-B23-A	2500	CDK	0.364058	0.006
**468**	3-KB-C19-D	10	CDK	0.386585	0.016
**469**	3-KB-C23-A	250	CDK	0.374543	0.008
**470**	3-KB-D19-D	1	CDK	0.374253	0.041
**471**	3-KB-D23-A	25	CDK	0.328157	0.24
**472**	3-KB-E19-D	0.1	CDK	0.322933	0.162
**473**	3-KB-E23-A	2.5	CDK	0.345446	0.043
**474**	3-KB-F23-A	0.25	CDK	0.265788	0.318
**475**	3-KB-K17-P	1	CDK	0.162541	0.625
**476**	3-KB-L19-R	1	CDK	0.116992	0.68
**477**	3-KB-M17-I	10	CDK	0.444235	0.047
**478**	3-KB-M19-I	10	CDK	0.107872	0.771
**479**	3-KB-N17-F	100	CDK	0.411585	0.005
**480**	3-KB-N19-F	100	CDK	0.163801	0.516
**481**	3-KB-O17-F	1000	CDK	0.420524	0.002
**482**	3-KB-O19-F	1000	CDK	0.493513	0
**483**	3-KB-P17-P	10000	CDK	0.48297	0
**484**	3-KB-P19-R	10000	CDK	0.393769	0.056
**485**	4-KB-A4-SN	10000	CDK	0.42123	0.002
**486**	4-KB-A8-Mi	10000	CDK	0.364624	0.023
**487**	4-KB-B4-SN	1000	CDK	0.381833	0.016
**488**	4-KB-B8-Mi	1000	CDK	0.381113	0.008

**489**	4-KB-C4-SN	100	CDK	0.367326	0.024
**490**	4-KB-C8-Mi	100	CDK	0.28091	0.188
**491**	4-KB-D4-SN	10	CDK	0.320104	0.423
**492**	4-KB-D8-Mi	10	CDK	0.135758	0.555
**493**	4-KB-E4-SN	1	CDK	0.207144	0.542
**494**	4-KB-E8-Mi	1	CDK	0.298456	0.328
**495**	4-KB-F4-Sel	10000	CDK	0.339341	0.036
**496**	4-KB-F22-A	10000	CDK	0.362708	0.024
**497**	4-KB-G4-Se	1000	CDK	0.144716	0.794
**498**	4-KB-G22-A	1000	CDK	0.370961	0.015
**499**	4-KB-H4-Se	100	CDK	0.11605	0.613
**500**	4-KB-H22-A	100	CDK	0.391584	0.006
**501**	4-KB-I4-Seli	10	CDK	0.183994	0.761
**502**	4-KB-I22-Al	10	CDK	0.33358	0.334
**503**	4-KB-J4-Sel	1	CDK	0.142719	0.512
**504**	4-KB-J22-Al	1	CDK	0.158621	0.443
**505**	5-KB-A19-A	10000	CDK	0.367863	0.017
**506**	5-KB-B19-A	1000	CDK	0.419202	0
**507**	5-KB-C19-A	100	CDK	0.363621	0.026
**508**	5-KB-D19-A	10	CDK	0.313423	0.143
**509**	5-KB-E19-A	1	CDK	0.142209	0.921
**510**	5-KB-K17-A	1	CDK	0.271163	0.217
**511**	5-KB-M17-A	10	CDK	0.233058	0.677
**512**	5-KB-N17-A	100	CDK	0.339529	0.062
**513**	5-KB-O17-A	1000	CDK	0.377293	0.014
**514**	5-KB-P17-A	10000	CDK	0.369067	0.031
**515**	6-KB-A17-S	1000	CDK	0.338921	0.021
**516**	6-KB-B17-S	100	CDK	0.382025	0.004
**517**	6-KB-C17-S	10	CDK	0.108945	0.761
**518**	6-KB-D17-S	1	CDK	0.097377	0.747
**519**	6-KB-E17-S	0.1	CDK	0.2601	0.62
**520**	6-KB-L15-T	1	CDK	0.076808	0.854
**521**	6-KB-M15-T	10	CDK	0.11386	0.754
**522**	6-KB-N15-T	100	CDK	0.382394	0.011
**523**	6-KB-O15-T	1000	CDK	0.35908	0.036

**524**	6-KB-P15-T	10000	CDK	0.349498	0.049
**525**	7-KB-A21-d	10000	BET	0.604147	0
**526**	7-KB-A22-P	30000	BET	0.601998	0
**527**	7-KB-B21-d	1000	BET	0.627688	0
**528**	7-KB-B22-P	3000	BET	0.672736	0
**529**	7-KB-C21-d	100	BET	0.625045	0
**530**	7-KB-C22-P	300	BET	0.642345	0
**531**	7-KB-D21-c	10	BET	0.528032	0.002
**532**	7-KB-D22-F	30	BET	0.643883	0
**533**	7-KB-E21-d	1	BET	0.531847	0
**534**	7-KB-E22-P	3	BET	0.663811	0
**535**	7-KB-G10-E	10000	BET	0.615923	0
**536**	7-KB-G15-I-	10000	BET	0.622411	0
**537**	7-KB-H10-E	1000	BET	0.614709	0
**538**	7-KB-H15-I-	1000	BET	0.633072	0
**539**	7-KB-I10-Bi	100	BET	0.67828	0
**540**	7-KB-I15-I-I	100	BET	0.583332	0
**541**	7-KB-J10-Bi	10	BET	0.803796	0
**542**	7-KB-J15-I-I	10	BET	0.186369	0.551
**543**	7-KB-K10-B	1	BET	0.506753	0
**544**	7-KB-K13-N	1	BET	0.671519	0
**545**	7-KB-K15-I-	1	BET	0.13376	0.781
**546**	7-KB-L12-N	1	BET	0.180728	0.382
**547**	7-KB-L13-N	10	BET	0.705526	0
**548**	7-KB-L20-J(	1	BET	0.091771	0.917
**549**	7-KB-L23-A	0.03	BET	0.208942	0.619
**550**	7-KB-M12-I	10	BET	0.128177	0.997
**551**	7-KB-M13-I	100	BET	0.621475	0
**552**	7-KB-M20-J	10	BET	0.355332	0.076
**553**	7-KB-M23-/	0.3	BET	0.159573	0.731
**554**	7-KB-N12-N	100	BET	0.764926	0
**555**	7-KB-N13-N	1000	BET	0.596551	0
**556**	7-KB-N20-J	100	BET	0.755668	0
**557**	7-KB-N23-A	3	BET	0.115746	0.668
**558**	7-KB-O12-N	1000	BET	0.60814	0

**559**	7-KB-O20-J	1000	BET	0.674019	0
**560**	7-KB-O23-A	30	BET	0.673625	0
**561**	7-KB-P12-N	10000	BET	0.630854	0
**562**	7-KB-P13-N	10000	BET	0.620445	0
**563**	7-KB-P20-J	10000	BET	0.655108	0
**564**	7-KB-P23-A	300	BET	0.58915	0
**565**	8-KB-K22-C	1	BET	0.266109	0.584
**566**	8-KB-L22-C	10	BET	0.403689	0.182
**567**	8-KB-M22-(	100	BET	0.221099	0.582
**568**	8-KB-N22-C	1000	BET	0.106215	0.78
**569**	8-KB-O22-C	10000	BET	0.134385	0.611
**570**	1-KB-A3-Vo	10000	HDAC	0.322202	0.029
**571**	1-KB-B3-Vo	1000	HDAC	0.374316	0.002
**572**	1-KB-C3-Vo	100	HDAC	0.286045	0.088
**573**	1-KB-D3-Vc	10	HDAC	0.336479	0.198
**574**	1-KB-E3-Vo	1	HDAC	0.064889	0.968
**575**	1-KB-L12-R	0.1	HDAC	0.043413	0.955
**576**	1-KB-M12-I	1	HDAC	0.43803	0
**577**	1-KB-N12-F	10	HDAC	0.29461	0.082
**578**	1-KB-O12-F	100	HDAC	0.322559	0.021
**579**	1-KB-P12-R	1000	HDAC	0.318869	0.038
**580**	3-KB-A4-Pa	1000	HDAC	0.293188	0.096
**581**	3-KB-B4-Pa	100	HDAC	0.315499	0.065
**582**	3-KB-C4-Pa	10	HDAC	0.123885	0.679
**583**	3-KB-D4-Pa	1	HDAC	0.077405	0.743
**584**	3-KB-E4-Pa	0.1	HDAC	0.073181	0.762
**585**	3-KB-F7-Qu	1000	HDAC	0.289926	0.097
**586**	3-KB-G7-Qu	100	HDAC	0.062315	0.864
**587**	3-KB-G12-V	1000000	HDAC	0.143965	0.411
**588**	3-KB-H7-Qu	10	HDAC	0.075442	0.888
**589**	3-KB-H12-V	100000	HDAC	0.205107	0.146
**590**	3-KB-I7-Qui	1	HDAC	0.145843	0.27
**591**	3-KB-I12-Va	10000	HDAC	0.120899	0.345
**592**	3-KB-J7-Qu	0.1	HDAC	0.080453	0.794
**593**	3-KB-J12-Va	1000	HDAC	0.079479	0.77

**594**	3-KB-K3-Be	1	HDAC	0.122662	0.712
**595**	3-KB-K12-V	100	HDAC	0.126799	0.367
**596**	3-KB-L3-Bel	10	HDAC	0.160008	0.565
**597**	3-KB-M3-Be	100	HDAC	0.3335	0.016
**598**	3-KB-N3-Be	1000	HDAC	0.290745	0.057
**599**	3-KB-O3-Be	10000	HDAC	0.372172	0.002
**600**	7-KB-A5-Mo	10000	HDAC	0.354127	0.005
**601**	7-KB-A7-CU	10000	HDAC	0.384088	0
**602**	7-KB-A9-Gi	1000	HDAC	0.355352	0.006
**603**	7-KB-A12-R	10000	HDAC	0.35081	0.007
**604**	7-KB-B5-Mo	1000	HDAC	0.459774	0
**605**	7-KB-B7-CU	1000	HDAC	0.386688	0
**606**	7-KB-B12-R	1000	HDAC	0.283803	0.01
**607**	7-KB-C5-Mo	100	HDAC	0.407694	0
**608**	7-KB-C7-CU	100	HDAC	0.387663	0
**609**	7-KB-C9-Gi	100	HDAC	0.508586	0
**610**	7-KB-D7-CU	10	HDAC	0.538869	0
**611**	7-KB-D9-Gi	10	HDAC	0.338115	0.108
**612**	7-KB-D12-R	100	HDAC	0.410221	0.001
**613**	7-KB-E5-Mo	10	HDAC	0.23868	0.347
**614**	7-KB-E7-CU	1	HDAC	0.522559	0
**615**	7-KB-E9-Gi	1	HDAC	0.377283	0.005
**616**	7-KB-E12-R	10	HDAC	0.466578	0
**617**	7-KB-F5-Mo	1	HDAC	0.112462	0.797
**618**	7-KB-F7-Re	10000	HDAC	0.390833	0.001
**619**	7-KB-F9-Gi	0.1	HDAC	0.379214	0.001
**620**	7-KB-F12-R	1	HDAC	0.423233	0.003
**621**	7-KB-F19-P	10000	HDAC	0.375521	0.001
**622**	7-KB-G7-Re	1000	HDAC	0.56724	0
**623**	7-KB-G19-P	1000	HDAC	0.46592	0
**624**	7-KB-H7-Re	100	HDAC	0.120708	0.468
**625**	7-KB-I7-Res	10	HDAC	0.531649	0
**626**	7-KB-I19-P	100	HDAC	0.455992	0
**627**	7-KB-J7-Res	1	HDAC	0.424701	0
**628**	7-KB-J19-P	10	HDAC	0.097436	0.647

**629**	7-KB-K4-En	1	HDAC	0.505934	0
**630**	7-KB-K11-A	1	HDAC	0.184454	0.051
**631**	7-KB-K18-T	1	HDAC	0.070965	0.705
**632**	7-KB-K19-P	1	HDAC	0.484368	0
**633**	7-KB-L2-Ta	0.1	HDAC	0.497032	0
**634**	7-KB-L4-En	10	HDAC	0.503217	0
**635**	7-KB-L5-Pra	1	HDAC	0.527229	0
**636**	7-KB-L8-Ab	1	HDAC	0.466584	0
**637**	7-KB-L10-T	1	HDAC	0.317002	0.011
**638**	7-KB-L11-A	10	HDAC	0.565275	0
**639**	7-KB-L14-T	1	HDAC	0.4926	0
**640**	7-KB-L16-R	1	HDAC	0.051396	0.865
**641**	7-KB-L18-T	10	HDAC	0.353826	0.024
**642**	7-KB-M2-Ta	1	HDAC	0.506772	0
**643**	7-KB-M5-Pr	10	HDAC	0.522844	0
**644**	7-KB-M8-Al	10	HDAC	0.55099	0
**645**	7-KB-M10-	10	HDAC	0.349759	0
**646**	7-KB-M11-	100	HDAC	0.47786	0
**647**	7-KB-M14-	10	HDAC	0.500921	0
**648**	7-KB-M16-	10	HDAC	0.158942	0.774
**649**	7-KB-M18-	100	HDAC	0.484625	0
**650**	7-KB-N2-Ta	10	HDAC	0.490357	0
**651**	7-KB-N4-En	100	HDAC	0.546066	0
**652**	7-KB-N5-Pr	100	HDAC	0.269829	0.156
**653**	7-KB-N8-Ab	100	HDAC	0.47602	0
**654**	7-KB-N10-T	100	HDAC	0.450378	0
**655**	7-KB-N14-T	100	HDAC	0.106728	0.709
**656**	7-KB-N16-R	100	HDAC	0.077652	0.705
**657**	7-KB-N18-T	1000	HDAC	0.45299	0
**658**	7-KB-O2-Ta	100	HDAC	0.464478	0
**659**	7-KB-O4-En	1000	HDAC	0.494755	0
**660**	7-KB-O5-Pr	1000	HDAC	0.409032	0
**661**	7-KB-O8-Ab	1000	HDAC	0.375067	0
**662**	7-KB-O10-T	1000	HDAC	0.58914	0
**663**	7-KB-O11-A	1000	HDAC	0.354359	0.007

**664**	7-KB-O14-T	1000	HDAC	0.124402	0.832
**665**	7-KB-O16-F	1000	HDAC	0.390018	0.002
**666**	7-KB-P2-Ta	1000	HDAC	0.258875	0.13
**667**	7-KB-P4-En	10000	HDAC	0.407111	0
**668**	7-KB-P5-Pr	10000	HDAC	0.399364	0
**669**	7-KB-P8-Ab	10000	HDAC	0.381123	0.001
**670**	7-KB-P10-T	10000	HDAC	0.415488	0
**671**	7-KB-P11-A	10000	HDAC	0.398576	0
**672**	7-KB-P14-T	10000	HDAC	0.260627	0.172
**673**	7-KB-P16-R	10000	HDAC	0.446616	0
**674**	7-KB-P18-T	10000	HDAC	0.445926	0
**0**	2-KW-A16-	10000	EGFR	0.391938	0.061
**1**	2-KW-A19-	10000	EGFR	0.42993	0.001
**2**	2-KW-B19-	1000	EGFR	0.393743	0.049
**3**	2-KW-C16-	1000	EGFR	0.448915	0.003
**4**	2-KW-C19-	100	EGFR	0.201153	0.096
**5**	2-KW-D16-	100	EGFR	0.180648	0.592
**6**	2-KW-D19-	10	EGFR	0.079576	0.705
**7**	2-KW-E16-	10	EGFR	0.090525	0.699
**8**	2-KW-E19-	1	EGFR	0.437924	0
**9**	2-KW-F16-	1	EGFR	0.192173	0.737
**10**	2-KW-K11-	0.1	EGFR	0.28992	0.269
**11**	2-KW-L11-	1	EGFR	0.401875	0
**12**	2-KW-L16-	0.25	EGFR	0.377659	0.005
**13**	2-KW-L19-	0.1	EGFR	0.283481	0.36
**14**	2-KW-M11-	10	EGFR	0.378252	0.052
**15**	2-KW-M16-	2.5	EGFR	0.422311	0
**16**	2-KW-M19-	1	EGFR	0.432056	0
**17**	2-KW-N16-	25	EGFR	0.405648	0.001
**18**	2-KW-N19-	10	EGFR	0.29704	0.208
**19**	2-KW-O11-	100	EGFR	0.448898	0
**20**	2-KW-O16-	250	EGFR	0.354234	0.064
**21**	2-KW-O19-	100	EGFR	0.340834	0.117
**22**	2-KW-P11-	1000	EGFR	0.381661	0.077
**23**	2-KW-P16-	2500	EGFR	0.389564	0.067

**24**	2-KW-P19-I	1000	EGFR	0.414294	0.008
**25**	3-KW-F21-I	10000	EGFR	0.369761	0.129
**26**	3-KW-G20-	1000	EGFR	0.37838	0.071
**27**	3-KW-G21-	1000	EGFR	0.453795	0.003
**28**	3-KW-H20-	100	EGFR	0.389314	0.004
**29**	3-KW-H21-	100	EGFR	0.223407	0.387
**30**	3-KW-I20-N	10	EGFR	0.407897	0.004
**31**	3-KW-I21-F	10	EGFR	0.258428	0.316
**32**	3-KW-J20-N	1	EGFR	0.087563	0.666
**33**	3-KW-J21-F	1	EGFR	0.149923	0.64
**34**	3-KW-K4-Ca	1	EGFR	0.268262	0.276
**35**	3-KW-K18-I	0.1	EGFR	0.362454	0.042
**36**	3-KW-K20-I	0.1	EGFR	0.39546	0.003
**37**	3-KW-L4-Ca	10	EGFR	0.284641	0.257
**38**	3-KW-L18-I	1	EGFR	0.298292	0.258
**39**	3-KW-M18-	10	EGFR	0.315919	0.218
**40**	3-KW-N4-C	100	EGFR	0.360359	0.055
**41**	3-KW-N18-	100	EGFR	0.37971	0.048
**42**	3-KW-O4-C	1000	EGFR	0.361616	0.069
**43**	3-KW-P4-Ca	10000	EGFR	0.357131	0.204
**44**	3-KW-P18-I	1000	EGFR	0.394741	0.005
**45**	4-KW-F13-S	1000	EGFR	0.446108	0
**46**	4-KW-G13-	100	EGFR	0.427145	0.001
**47**	4-KW-G16-	10000	EGFR	0.363871	0.085
**48**	4-KW-H13-	10	EGFR	0.16388	0.609
**49**	4-KW-H16-	1000	EGFR	0.298194	0.091
**50**	4-KW-I13-S	1	EGFR	0.385987	0.021
**51**	4-KW-I16-V	100	EGFR	0.243805	0.223
**52**	4-KW-J13-S	0.1	EGFR	0.370746	0.133
**53**	4-KW-J16-V	10	EGFR	0.050237	0.999
**54**	4-KW-K7-Ic	1	EGFR	0.430627	0.001
**55**	4-KW-K13-T	0.1	EGFR	0.208463	0.55
**56**	4-KW-K16-V	1	EGFR	0.228329	0.203
**57**	4-KW-L7-Ic	10	EGFR	0.347443	0.037
**58**	4-KW-L13-T	1	EGFR	0.267683	0.392

**59**	4-KW-M7-I	100	EGFR	0.381113	0.02
**60**	4-KW-M13-	10	EGFR	0.411726	0.008
**61**	4-KW-N13-	100	EGFR	0.404805	0.01
**62**	4-KW-O7-Ic	1000	EGFR	0.412086	0.038
**63**	4-KW-P7-Ic	10000	EGFR	0.278543	0.183
**64**	4-KW-P13-	1000	EGFR	0.339896	0.131
**65**	5-KW-F4-Pc	1000	EGFR	0.361959	0.049
**66**	5-KW-F7-AZ	1000	EGFR	0.371125	0.018
**67**	5-KW-G4-P	100	EGFR	0.36711	0.053
**68**	5-KW-G7-A	100	EGFR	0.424313	0
**69**	5-KW-H4-P	10	EGFR	0.332799	0.142
**70**	5-KW-H7-A	10	EGFR	0.075294	0.916
**71**	5-KW-I4-Pc	1	EGFR	0.316782	0.23
**72**	5-KW-I7-AZ	1	EGFR	0.349919	0.072
**73**	5-KW-J4-Pc	0.1	EGFR	0.344407	0.171
**74**	5-KW-J7-AZ	0.1	EGFR	0.302576	0.131
**75**	5-KW-K7-O	0.1	EGFR	0.286062	0.307
**76**	5-KW-L7-OI	1	EGFR	0.364691	0.026
**77**	5-KW-M7-C	10	EGFR	0.370324	0.05
**78**	5-KW-O7-O	100	EGFR	0.365547	0.037
**79**	5-KW-P7-O	1000	EGFR	0.425071	0.001
**80**	2-KW-A15-I	2500	VEGFR	0.333227	0.239
**81**	2-KW-A17-I	10000	VEGFR	0.337912	0.305
**82**	2-KW-A20-	10000	VEGFR	0.337994	0.323
**83**	2-KW-B15-I	250	VEGFR	0.308975	0.059
**84**	2-KW-B17-I	1000	VEGFR	0.456342	0
**85**	2-KW-B20-	1000	VEGFR	0.356185	0.027
**86**	2-KW-C15-I	25	VEGFR	0.267079	0.074
**87**	2-KW-C17-I	100	VEGFR	0.444604	0
**88**	2-KW-D15-	2.5	VEGFR	0.170084	0.037
**89**	2-KW-D17-	10	VEGFR	0.309082	0.003
**90**	2-KW-D20-	100	VEGFR	0.312689	0.066
**91**	2-KW-E17-I	1	VEGFR	0.16982	0.05
**92**	2-KW-E20-	10	VEGFR	0.13555	0.912
**93**	2-KW-F13-/	10000	VEGFR	0.219271	0.853

**94**	2-KW-F15-I	0.25	VEGFR	0.065131	0.979
**95**	2-KW-F19-I	10000	VEGFR	0.256748	0.785
**96**	2-KW-F20-T	1	VEGFR	0.254338	0.278
**97**	2-KW-F21-V	10000	VEGFR	0.288173	0.392
**98**	2-KW-G10-	10000	VEGFR	0.334315	0.001
**99**	2-KW-G13-	1000	VEGFR	0.308284	0.125
**100**	2-KW-G19-	1000	VEGFR	0.306547	0.282
**101**	2-KW-G21-	1000	VEGFR	0.398893	0.001
**102**	2-KW-H10-	1000	VEGFR	0.399958	0
**103**	2-KW-H13-	100	VEGFR	0.460872	0
**104**	2-KW-H21-	100	VEGFR	0.262234	0.015
**105**	2-KW-I10-A	100	VEGFR	0.306867	0.17
**106**	2-KW-I13-A	10	VEGFR	0.45206	0
**107**	2-KW-I19-F	100	VEGFR	0.225385	0.526
**108**	2-KW-I21-V	10	VEGFR	0.146558	0.988
**109**	2-KW-J10-A	10	VEGFR	0.222832	0.012
**110**	2-KW-J13-A	1	VEGFR	0.283381	0.187
**111**	2-KW-J19-F	10	VEGFR	0.149553	0.834
**112**	2-KW-J21-V	1	VEGFR	0.330377	0.184
**113**	2-KW-K10-A	1	VEGFR	0.259382	0.42
**114**	2-KW-K13-Y	0.1	VEGFR	0.290969	0.461
**115**	2-KW-K17-I	1	VEGFR	0.371089	0.001
**116**	2-KW-K19-I	1	VEGFR	0.374215	0.002
**117**	2-KW-L12-S	0.1	VEGFR	0.319458	0.094
**118**	2-KW-L13-Y	1	VEGFR	0.160801	0.591
**119**	2-KW-L21-(	0.1	VEGFR	0.179163	0.915
**120**	2-KW-M12-	1	VEGFR	0.309107	0.271
**121**	2-KW-M13-	10	VEGFR	0.438958	0.001
**122**	2-KW-M17-	10	VEGFR	0.362939	0.013
**123**	2-KW-M21-	1	VEGFR	0.199589	0.788
**124**	2-KW-N12-	10	VEGFR	0.298871	0.446
**125**	2-KW-N13-	100	VEGFR	0.264545	0.489
**126**	2-KW-N17-	100	VEGFR	0.328611	0.122
**127**	2-KW-N21-	10	VEGFR	0.294473	0.122
**128**	2-KW-O12-	100	VEGFR	0.243471	0.767

**129**	2-KW-O17-	1000	VEGFR	0.285269	0.26
**130**	2-KW-O21-	100	VEGFR	0.20316	0.895
**131**	2-KW-P12-	1000	VEGFR	0.393002	0.01
**132**	2-KW-P13-	1000	VEGFR	0.26304	0.697
**133**	2-KW-P17-	10000	VEGFR	0.333433	0.296
**134**	2-KW-P21-	1000	VEGFR	0.315323	0.472
**135**	3-KW-A3-C	1000	VEGFR	0.317511	0.366
**136**	3-KW-A6-F	1000	VEGFR	0.296674	0.521
**137**	3-KW-A18-	1000	VEGFR	0.465764	0
**138**	3-KW-B3-C	100	VEGFR	0.298798	0.486
**139**	3-KW-B6-F	100	VEGFR	0.300461	0.297
**140**	3-KW-B18-	100	VEGFR	0.189555	0.028
**141**	3-KW-C3-C	10	VEGFR	0.167264	0.08
**142**	3-KW-C6-F	10	VEGFR	0.2647	0.562
**143**	3-KW-C18-	10	VEGFR	0.144281	0.958
**144**	3-KW-D3-C	1	VEGFR	0.188657	0.906
**145**	3-KW-D6-F	1	VEGFR	0.214465	0.178
**146**	3-KW-D18-	1	VEGFR	0.35417	0.046
**147**	3-KW-E3-C	0.1	VEGFR	0.327109	0.029
**148**	3-KW-E6-F	0.1	VEGFR	0.522718	0
**149**	3-KW-E18-	0.1	VEGFR	0.28643	0.015
**150**	3-KW-F18-	1000	VEGFR	0.25224	0
**151**	3-KW-G18-	100	VEGFR	0.417301	0
**152**	3-KW-H18-	10	VEGFR	0.3291	0.054
**153**	3-KW-I18-E	1	VEGFR	0.412479	0
**154**	3-KW-J18-E	0.1	VEGFR	0.288934	0.003
**155**	4-KW-A12-	10000	VEGFR	0.283872	0.652
**156**	4-KW-A15-	2500	VEGFR	0.266508	0.715
**157**	4-KW-A20-	10000	VEGFR	0.275799	0.431
**158**	4-KW-B12-	1000	VEGFR	0.244341	0.811
**159**	4-KW-B15-	250	VEGFR	0.298311	0.049
**160**	4-KW-B20-	1000	VEGFR	0.229358	0.404
**161**	4-KW-C15-	25	VEGFR	0.22553	0.012
**162**	4-KW-D12-	100	VEGFR	0.317945	0.562
**163**	4-KW-D15-	2.5	VEGFR	0.170247	0.245

**164**	4-KW-D20-	100	VEGFR	0.167393	0.016
**165**	4-KW-E12-I	10	VEGFR	0.333724	0
**166**	4-KW-E20-I	10	VEGFR	0.194186	0.031
**167**	4-KW-F12-I	1	VEGFR	0.37384	0.01
**168**	4-KW-F15-(	0.25	VEGFR	0.289235	0.222
**169**	4-KW-F20-I	1	VEGFR	0.16797	0.043
**170**	4-KW-L16-T	1	VEGFR	0.378108	0.007
**171**	4-KW-M16-	10	VEGFR	0.193482	0.803
**172**	4-KW-N16-	100	VEGFR	0.275365	0.262
**173**	4-KW-O16-	1000	VEGFR	0.223383	0.892
**174**	4-KW-P16-T	10000	VEGFR	0.261896	0.79
**175**	2-KW-L10-I	1	PI3K	0.241889	0.662
**176**	2-KW-M10-	10	PI3K	0.380624	0.002
**177**	2-KW-N10-	100	PI3K	0.309166	0.058
**178**	2-KW-O10-	1000	PI3K	0.411753	0.004
**179**	2-KW-P10-I	10000	PI3K	0.407673	0.004
**180**	3-KW-A16-I	2500	PI3K	0.088043	0.509
**181**	3-KW-C16-I	250	PI3K	0.239955	0.405
**182**	3-KW-D16-	25	PI3K	0.259095	0.365
**183**	3-KW-E16-I	2.5	PI3K	0.071629	0.687
**184**	3-KW-F16-I	0.25	PI3K	0.075087	0.481
**185**	3-KW-F17-I	100000	PI3K	0.363546	0.07
**186**	3-KW-F19-I	500	PI3K	0.388654	0
**187**	3-KW-G17-	10000	PI3K	0.191736	0.686
**188**	3-KW-G19-	50	PI3K	0.3024	0.126
**189**	3-KW-H17-	1000	PI3K	0.181882	0.586
**190**	3-KW-I17-N	100	PI3K	0.270626	0.339
**191**	3-KW-I19-D	5	PI3K	0.083112	0.652
**192**	3-KW-J17-N	10	PI3K	0.232239	0.55
**193**	3-KW-J19-D	0.5	PI3K	0.179336	0.977
**194**	3-KW-K19-I	0.05	PI3K	0.064741	0.823
**195**	3-KW-L8-Pi	1	PI3K	0.245675	0.067
**196**	3-KW-L21-T	0.1	PI3K	0.128577	0.742
**197**	3-KW-M8-P	10	PI3K	0.34639	0.009
**198**	3-KW-M21-	1	PI3K	0.159343	0.784

**199**	3-KW-N8-P	100	PI3K	0.369193	0.011
**200**	3-KW-N21-	10	PI3K	0.182932	0.83
**201**	3-KW-O8-P	1000	PI3K	0.394295	0.009
**202**	3-KW-O21-	100	PI3K	0.375073	0.015
**203**	3-KW-P8-Pi	10000	PI3K	0.38651	0.023
**204**	3-KW-P21-T	1000	PI3K	0.416354	0.004
**205**	4-KW-A19-A	2500	PI3K	0.498222	0
**206**	4-KW-B19-A	250	PI3K	0.468841	0
**207**	4-KW-C19-A	25	PI3K	0.044337	0.847
**208**	4-KW-D19-	2.5	PI3K	0.053966	0.667
**209**	4-KW-E19-A	0.25	PI3K	0.414792	0
**210**	4-KW-F14-I	1000	PI3K	0.437043	0
**211**	4-KW-G2-T	2500	PI3K	0.324979	0.058
**212**	4-KW-G5-S	10000	PI3K	0.370914	0.003
**213**	4-KW-G14-	100	PI3K	0.447611	0
**214**	4-KW-G20-	10000	PI3K	0.443475	0
**215**	4-KW-H2-T	250	PI3K	0.10052	0.487
**216**	4-KW-H5-S	1000	PI3K	0.17631	0.195
**217**	4-KW-H14-	10	PI3K	0.450532	0
**218**	4-KW-H20-	1000	PI3K	0.456981	0
**219**	4-KW-I2-TG	25	PI3K	0.252658	0.187
**220**	4-KW-I5-So	100	PI3K	0.2409	0.337
**221**	4-KW-I14-N	1	PI3K	0.524433	0
**222**	4-KW-I20-E	100	PI3K	0.430492	0
**223**	4-KW-J2-TG	2.5	PI3K	0.186789	0.544
**224**	4-KW-J5-Sc	10	PI3K	0.37262	0.027
**225**	4-KW-J20-E	10	PI3K	0.473463	0
**226**	4-KW-K2-T	0.25	PI3K	0.166438	0.985
**227**	4-KW-K4-D	0.1	PI3K	0.432249	0
**228**	4-KW-K5-S	1	PI3K	0.498772	0
**229**	4-KW-K14-I	0.1	PI3K	0.481451	0
**230**	4-KW-K20-I	1	PI3K	0.414104	0
**231**	4-KW-L4-D	1	PI3K	0.408441	0
**232**	4-KW-L14-	0.1	PI3K	0.415972	0
**233**	4-KW-L15-T	1	PI3K	0.301502	0.031

**234**	4-KW-L21-(	0.1	PI3K	0.282323	0.03
**235**	4-KW-M14-	1	PI3K	0.472834	0
**236**	4-KW-M15-	10	PI3K	0.492889	0
**237**	4-KW-M21-	1	PI3K	0.460497	0
**238**	4-KW-N4-D	10	PI3K	0.376719	0.006
**239**	4-KW-N14-	10	PI3K	0.459581	0
**240**	4-KW-N15-	100	PI3K	0.476847	0
**241**	4-KW-N21-	10	PI3K	0.340067	0.076
**242**	4-KW-O4-D	100	PI3K	0.412402	0
**243**	4-KW-O14-	100	PI3K	0.433736	0
**244**	4-KW-O15-	1000	PI3K	0.378595	0
**245**	4-KW-O21-	100	PI3K	0.445382	0
**246**	4-KW-P4-D	1000	PI3K	0.414733	0.001
**247**	4-KW-P14-(	1000	PI3K	0.436569	0.001
**248**	4-KW-P15-T	10000	PI3K	0.418286	0.001
**249**	4-KW-P21-(	1000	PI3K	0.449156	0
**250**	5-KW-A6-LY	2500	PI3K	0.413432	0.002
**251**	5-KW-A7-Al	1000	PI3K	0.321157	0.019
**252**	5-KW-A16-,	2500	PI3K	0.456886	0
**253**	5-KW-A17-I	10000	PI3K	0.268617	0.244
**254**	5-KW-B6-LY	250	PI3K	0.320575	0.138
**255**	5-KW-B7-Al	100	PI3K	0.055967	0.701
**256**	5-KW-B17-I	1000	PI3K	0.232556	0.022
**257**	5-KW-C6-LY	25	PI3K	0.237428	0.449
**258**	5-KW-C7-Al	10	PI3K	0.046038	0.945
**259**	5-KW-C16-,	250	PI3K	0.412934	0
**260**	5-KW-C17-I	100	PI3K	0.28545	0.103
**261**	5-KW-D6-LY	2.5	PI3K	0.161315	0.782
**262**	5-KW-D7-A	1	PI3K	0.07323	0.456
**263**	5-KW-D16-	25	PI3K	0.366599	0.007
**264**	5-KW-D17-	10	PI3K	0.118419	0.386
**265**	5-KW-E6-LY	0.25	PI3K	0.287404	0.121
**266**	5-KW-E7-Al	0.1	PI3K	0.175296	0.644
**267**	5-KW-E16-/	2.5	PI3K	0.282897	0.079
**268**	5-KW-E17-I	1	PI3K	0.320042	0.058

**269**	5-KW-F11-(	10000	PI3K	0.432852	0
**270**	5-KW-F16-/	0.25	PI3K	0.164201	0.395
**271**	5-KW-G9-S	10000	PI3K	0.361951	0.026
**272**	5-KW-G11-	1000	PI3K	0.448077	0
**273**	5-KW-H9-S	1000	PI3K	0.378106	0.001
**274**	5-KW-H11-	100	PI3K	0.363566	0.004
**275**	5-KW-I9-Se	100	PI3K	0.094495	0.817
**276**	5-KW-I11-C	10	PI3K	0.311327	0.02
**277**	5-KW-J9-Se	10	PI3K	0.431207	0
**278**	5-KW-J11-(	1	PI3K	0.114015	0.552
**279**	5-KW-K9-S	1	PI3K	0.313911	0.072
**280**	5-KW-L14-/	0.1	PI3K	0.308649	0.053
**281**	5-KW-L20-7	1	PI3K	0.300932	0.052
**282**	5-KW-L23-(	0.1	PI3K	0.053196	0.99
**283**	5-KW-M14-	1	PI3K	0.240054	0.061
**284**	5-KW-M20-	10	PI3K	0.098554	0.736
**285**	5-KW-M23-	1	PI3K	0.187085	0.806
**286**	5-KW-N14-	10	PI3K	0.329726	0.079
**287**	5-KW-N20-	100	PI3K	0.357916	0.026
**288**	5-KW-N23-	10	PI3K	0.390349	0.007
**289**	5-KW-O14-	100	PI3K	0.373612	0.004
**290**	5-KW-O20-	1000	PI3K	0.386699	0.006
**291**	5-KW-O23-	100	PI3K	0.390558	0.011
**292**	5-KW-P14-/	1000	PI3K	0.351017	0.041
**293**	5-KW-P20-7	10000	PI3K	0.394659	0.005
**294**	5-KW-P23-(	1000	PI3K	0.385425	0.014
**295**	6-KW-A8-T(	10000	PI3K	0.429074	0
**296**	6-KW-B8-T(	1000	PI3K	0.155158	0.841
**297**	6-KW-C8-T(	100	PI3K	0.363778	0.018
**298**	6-KW-D8-T(	10	PI3K	0.120927	0.718
**299**	6-KW-E8-T(	1	PI3K	0.13598	0.439
**300**	6-KW-L6-G	1	PI3K	0.214095	0.445
**301**	6-KW-M6-C	10	PI3K	0.264934	0.236
**302**	6-KW-N6-G	100	PI3K	0.182375	0.92
**303**	6-KW-O6-G	1000	PI3K	0.385313	0.007

**304**	6-KW-P6-G	10000	PI3K	0.387906	0.017
**305**	1-KW-F11-/	10000	Topoisome	0.596247	0
**306**	1-KW-G11-	1000	Topoisome	0.569388	0
**307**	1-KW-G20-	1000	Topoisome	0.440101	0.011
**308**	1-KW-H11-	100	Topoisome	0.56365	0
**309**	1-KW-H20-	100	Topoisome	0.598034	0
**310**	1-KW-I11-A	10	Topoisome	0.109689	0.598
**311**	1-KW-I20-E	10	Topoisome	0.44288	0.003
**312**	1-KW-J11-/	1	Topoisome	0.450417	0
**313**	1-KW-J20-E	1	Topoisome	0.06159	0.972
**314**	1-KW-K11-:	1	Topoisome	0.598676	0
**315**	1-KW-K20-I	0.1	Topoisome	0.412752	0.018
**316**	1-KW-L11-S	10	Topoisome	0.605966	0
**317**	1-KW-L14-T	1	Topoisome	0.448758	0.002
**318**	1-KW-M11-	100	Topoisome	0.434417	0.015
**319**	1-KW-M14-	10	Topoisome	0.524455	0
**320**	1-KW-N14-	100	Topoisome	0.607491	0
**321**	1-KW-O11-	1000	Topoisome	0.401634	0.063
**322**	1-KW-O14-	1000	Topoisome	0.539018	0
**323**	1-KW-P11-:	10000	Topoisome	0.401563	0.091
**324**	1-KW-P14-T	10000	Topoisome	0.431717	0.042
**325**	3-KW-A11-I	10000	Topoisome	0.599678	0
**326**	3-KW-B11-I	1000	Topoisome	0.60058	0
**327**	3-KW-C11-I	100	Topoisome	0.461279	0
**328**	3-KW-D11-	10	Topoisome	0.495178	0
**329**	3-KW-E11-I	1	Topoisome	0.468579	0
**330**	3-KW-G9-D	1000	Topoisome	0.427878	0.029
**331**	3-KW-G10-	10000	Topoisome	0.360678	0.002
**332**	3-KW-H9-D	100	Topoisome	0.63196	0
**333**	3-KW-H10-	1000	Topoisome	0.3468	0.02
**334**	3-KW-I9-Da	10	Topoisome	0.628326	0
**335**	3-KW-I10-T	100	Topoisome	0.444076	0.012
**336**	3-KW-J9-Da	1	Topoisome	0.23731	0.157
**337**	3-KW-J10-T	10	Topoisome	0.486534	0.001
**338**	3-KW-K7-Ic	0.1	Topoisome	0.36228	0.092

339	3-KW-K9-D	0.1	Topoisome	0.372378	0.001
340	3-KW-K10-	1	Topoisome	0.550365	0
341	3-KW-L6-D	0.1	Topoisome	0.302345	0.45
342	3-KW-L7-Id	1	Topoisome	0.198831	0.338
343	3-KW-L9-Va	0.5	Topoisome	0.559646	0
344	3-KW-L10-I	0.1	Topoisome	0.490127	0.002
345	3-KW-L16-I	1	Topoisome	0.306899	0.244
346	3-KW-M6-D	1	Topoisome	0.583688	0
347	3-KW-M7-I	10	Topoisome	0.380549	0.06
348	3-KW-M9-V	5	Topoisome	0.537556	0
349	3-KW-M10-	1	Topoisome	0.486242	0
350	3-KW-M16-	10	Topoisome	0.422011	0.008
351	3-KW-N6-D	10	Topoisome	0.414263	0.004
352	3-KW-N9-V	50	Topoisome	0.628256	0
353	3-KW-N10-	10	Topoisome	0.448528	0.001
354	3-KW-N16-	100	Topoisome	0.421907	0.009
355	3-KW-O6-D	100	Topoisome	0.555618	0
356	3-KW-O7-I	100	Topoisome	0.567123	0
357	3-KW-O9-V	500	Topoisome	0.621882	0
358	3-KW-O10-	100	Topoisome	0.592531	0
359	3-KW-O16-	1000	Topoisome	0.439983	0.003
360	3-KW-P6-D	1000	Topoisome	0.482499	0.001
361	3-KW-P7-I	1000	Topoisome	0.407363	0.073
362	3-KW-P9-Va	5000	Topoisome	0.500836	0
363	3-KW-P10-I	1000	Topoisome	0.461206	0.006
364	3-KW-P16-I	10000	Topoisome	0.23584	0.813
365	1-KW-A10-	10000	Mitotic	0.56034	0
366	1-KW-A13-I	1000	Mitotic	0.609994	0
367	1-KW-A18-I	1000	Mitotic	0.579539	0
368	1-KW-B10-	1000	Mitotic	0.569909	0
369	1-KW-B13-I	100	Mitotic	0.53957	0
370	1-KW-B18-I	100	Mitotic	0.582783	0
371	1-KW-C10-	100	Mitotic	0.567646	0
372	1-KW-C13-I	10	Mitotic	0.266512	0.078
373	1-KW-C18-I	10	Mitotic	0.571121	0

**374**	1-KW-D10-	10	Mitotic	0.210551	0.111
**375**	1-KW-D13-	1	Mitotic	0.154455	0.411
**376**	1-KW-D18-	1	Mitotic	0.269661	0.213
**377**	1-KW-E10-\	1	Mitotic	0.190061	0.145
**378**	1-KW-E13-I	0.1	Mitotic	0.211116	0.202
**379**	1-KW-E18-I	0.1	Mitotic	0.244081	0.489
**380**	1-KW-F13-\	1000	Mitotic	0.566626	0
**381**	1-KW-G13-	100	Mitotic	0.353981	0.018
**382**	1-KW-G15-	1000	Mitotic	0.56773	0
**383**	1-KW-H13-	10	Mitotic	0.249928	0.101
**384**	1-KW-H15-	100	Mitotic	0.571753	0.001
**385**	1-KW-I13-\	1	Mitotic	0.453714	0.002
**386**	1-KW-I15-E	10	Mitotic	0.577293	0
**387**	1-KW-J13-\	0.1	Mitotic	0.392502	0.028
**388**	1-KW-J15-E	1	Mitotic	0.449166	0.004
**389**	1-KW-K7-Vi	0.1	Mitotic	0.558966	0
**390**	1-KW-K15-I	0.1	Mitotic	0.543812	0
**391**	1-KW-L7-Vi	1	Mitotic	0.462848	0.056
**392**	1-KW-L20-\	0.1	Mitotic	0.553643	0
**393**	1-KW-M7-\	10	Mitotic	0.468442	0.002
**394**	1-KW-M20-	1	Mitotic	0.240387	0.397
**395**	1-KW-N20-	10	Mitotic	0.612999	0
**396**	1-KW-O7-V	100	Mitotic	0.634234	0
**397**	1-KW-O20-	100	Mitotic	0.573849	0
**398**	1-KW-P7-Vi	1000	Mitotic	0.564131	0
**399**	1-KW-P20-\	1000	Mitotic	0.619976	0
**400**	3-KW-A7-D	1000	Mitotic	0.516347	0
**401**	3-KW-B7-D	100	Mitotic	0.570414	0
**402**	3-KW-C7-D	10	Mitotic	0.580145	0
**403**	3-KW-D7-D	1	Mitotic	0.113863	0.845
**404**	3-KW-E7-D	0.1	Mitotic	0.175004	0.543
**405**	6-KW-L19-/	1	Mitotic	0.249532	0.699
**406**	6-KW-M19-	10	Mitotic	0.224564	0.829
**407**	6-KW-N19-	100	Mitotic	0.28895	0.645
**408**	6-KW-O19-	1000	Mitotic	0.554817	0.001

**409**	6-KW-P19-/	10000	Mitotic	0.521522	0
**410**	2-KW-A12-ˉ	250	MEK1/2	0.390974	0.41
**411**	2-KW-B12-ˉ	25	MEK1/2	0.394982	0.274
**412**	2-KW-D12-	2.5	MEK1/2	0.440957	0.1
**413**	2-KW-E12-ˉ	0.25	MEK1/2	0.291315	0.252
**414**	2-KW-F12-ˉ	2.5000000	MEK1/2	0.190692	0.796
**415**	2-KW-F14-(	1000	MEK1/2	0.416622	0.195
**416**	2-KW-G14-	100	MEK1/2	0.452199	0.072
**417**	2-KW-H14-	10	MEK1/2	0.468592	0.05
**418**	2-KW-I14-C	1	MEK1/2	0.392272	0.174
**419**	2-KW-K14-(	0.1	MEK1/2	0.196901	0.905
**420**	2-KW-L20-S	1	MEK1/2	0.199726	0.898
**421**	2-KW-M20-	10	MEK1/2	0.188372	0.817
**422**	2-KW-N20-	100	MEK1/2	0.352706	0.366
**423**	2-KW-O20-	1000	MEK1/2	0.405651	0.243
**424**	2-KW-P20-S	10000	MEK1/2	0.421179	0.208
**425**	4-KW-A10-I	1000	MEK1/2	0.606907	0
**426**	4-KW-A13-I	1000	MEK1/2	0.52608	0.006
**427**	4-KW-B10-I	100	MEK1/2	0.55851	0.003
**428**	4-KW-B13-I	100	MEK1/2	0.47536	0.014
**429**	4-KW-C10-I	10	MEK1/2	0.280804	0.453
**430**	4-KW-C13-I	10	MEK1/2	0.552805	0
**431**	4-KW-D10-	1	MEK1/2	0.196484	0.728
**432**	4-KW-D13-	1	MEK1/2	0.32285	0.084
**433**	4-KW-E10-I	0.1	MEK1/2	0.279874	0.065
**434**	4-KW-E13-I	0.1	MEK1/2	0.239901	0.178
**435**	4-KW-L19-(	0.25	MEK1/2	0.24849	0.776
**436**	4-KW-M19-	2.5	MEK1/2	0.384214	0.233
**437**	4-KW-N19-	25	MEK1/2	0.490249	0.02
**438**	4-KW-O19-	250	MEK1/2	0.433131	0.113
**439**	4-KW-P19-(	2500	MEK1/2	0.494563	0.02
**440**	1-KW-L2-OI	1	PARP	0.470108	0.068
**441**	1-KW-L6-Rı	1	PARP	0.632414	0
**442**	1-KW-M2-C	10	PARP	0.426769	0.141
**443**	1-KW-M6-F	10	PARP	0.461388	0.053

**444**	1-KW-N2-O	100	PARP	0.472619	0.049
**445**	1-KW-N6-R	100	PARP	0.586747	0.001
**446**	1-KW-O2-O	1000	PARP	0.601337	0
**447**	1-KW-O6-R	1000	PARP	0.531757	0.008
**448**	1-KW-P2-O	10000	PARP	0.641734	0
**449**	1-KW-P6-R	10000	PARP	0.465459	0.08
**450**	7-KW-A3-Ta	1000	PARP	0.621563	0.001
**451**	7-KW-B2-V	10000	PARP	0.648214	0
**452**	7-KW-B3-Ta	100	PARP	0.62389	0
**453**	7-KW-C2-V	1000	PARP	0.666298	0.001
**454**	7-KW-C3-Ta	10	PARP	0.554408	0.001
**455**	7-KW-D2-V	100	PARP	0.571009	0.004
**456**	7-KW-D3-T	1	PARP	0.505138	0.015
**457**	7-KW-E2-Ve	10	PARP	0.513871	0.007
**458**	7-KW-E3-Ta	0.1	PARP	0.235046	0.425
**459**	7-KW-F2-Ve	1	PARP	0.57355	0.003
**460**	7-KW-G2-N	10000	PARP	0.587946	0.001
**461**	7-KW-H2-N	1000	PARP	0.526057	0.005
**462**	7-KW-I2-Ni	100	PARP	0.383163	0.268
**463**	7-KW-J2-Ni	10	PARP	0.433127	0.03
**464**	7-KW-K2-N	1	PARP	0.380308	0.269
**465**	3-KW-A19-	1000	CDK	0.346625	0.342
**466**	3-KW-B19-	100	CDK	0.346522	0.362
**467**	3-KW-B23-	2500	CDK	0.380226	0.033
**468**	3-KW-C19-	10	CDK	0.440534	0.013
**469**	3-KW-C23-	250	CDK	0.391104	0.014
**470**	3-KW-D19-	1	CDK	0.529395	0
**471**	3-KW-D23-	25	CDK	0.316702	0.229
**472**	3-KW-E19-	0.1	CDK	0.123257	0.376
**473**	3-KW-E23-	2.5	CDK	0.358546	0.076
**474**	3-KW-F23-	0.25	CDK	0.072296	0.911
**475**	3-KW-K17-	1	CDK	0.421252	0.009
**476**	3-KW-L19-	1	CDK	0.169565	0.697
**477**	3-KW-M17-	10	CDK	0.421055	0.007
**478**	3-KW-M19-	10	CDK	0.336322	0.186

**479**	3-KW-N17-	100	CDK	0.44739	0.004
**480**	3-KW-N19-	100	CDK	0.459285	0.001
**481**	3-KW-O17-	1000	CDK	0.363432	0.047
**482**	3-KW-O19-	1000	CDK	0.473746	0.001
**483**	3-KW-P17-I	10000	CDK	0.321406	0.197
**484**	3-KW-P19-I	10000	CDK	0.530912	0.001
**485**	4-KW-A4-SI	10000	CDK	0.371666	0.165
**486**	4-KW-A8-M	10000	CDK	0.366372	0.194
**487**	4-KW-B4-SI	1000	CDK	0.378124	0.165
**488**	4-KW-B8-M	1000	CDK	0.389879	0.021
**489**	4-KW-C4-SI	100	CDK	0.42797	0.011
**490**	4-KW-C8-M	100	CDK	0.458898	0
**491**	4-KW-D4-SI	10	CDK	0.250093	0.41
**492**	4-KW-D8-M	10	CDK	0.100803	0.868
**493**	4-KW-E4-SI	1	CDK	0.354637	0.041
**494**	4-KW-E8-M	1	CDK	0.100933	0.461
**495**	4-KW-F4-Se	10000	CDK	0.39192	0.02
**496**	4-KW-F22-/	10000	CDK	0.376294	0.171
**497**	4-KW-G4-S	1000	CDK	0.203057	0.722
**498**	4-KW-G22-	1000	CDK	0.378822	0.144
**499**	4-KW-H4-S	100	CDK	0.182408	0.167
**500**	4-KW-H22-	100	CDK	0.403981	0.014
**501**	4-KW-I4-Se	10	CDK	0.29298	0.421
**502**	4-KW-I22-A	10	CDK	0.395014	0.004
**503**	4-KW-J4-Se	1	CDK	0.290416	0.32
**504**	4-KW-J22-/	1	CDK	0.18332	0.165
**505**	5-KW-A19-,	10000	CDK	0.377205	0.158
**506**	5-KW-B19-,	1000	CDK	0.377019	0.126
**507**	5-KW-C19-,	100	CDK	0.336208	0.136
**508**	5-KW-D19-	10	CDK	0.283046	0.449
**509**	5-KW-E19-/	1	CDK	0.406399	0.01
**510**	5-KW-K17-,	1	CDK	0.344891	0.163
**511**	5-KW-M17-	10	CDK	0.365349	0.064
**512**	5-KW-N17-	100	CDK	0.374772	0.094
**513**	5-KW-O17-	1000	CDK	0.372128	0.176

**514**	5-KW-P17-	10000	CDK	0.374726	0.185
**515**	6-KW-A17-	1000	CDK	0.345923	0.261
**516**	6-KW-B17-	100	CDK	0.176985	0.895
**517**	6-KW-C17-	10	CDK	0.284014	0.095
**518**	6-KW-D17-	1	CDK	0.157432	0.62
**519**	6-KW-E17-	0.1	CDK	0.402532	0.018
**520**	6-KW-L15-	1	CDK	0.192003	0.792
**521**	6-KW-M15-	10	CDK	0.40971	0.017
**522**	6-KW-N15-	100	CDK	0.389753	0.06
**523**	6-KW-O15-	1000	CDK	0.385329	0.157
**524**	6-KW-P15-	10000	CDK	0.372479	0.24
**525**	7-KW-A21-	10000	BET	0.650895	0
**526**	7-KW-A22-	30000	BET	0.679335	0
**527**	7-KW-B21-	1000	BET	0.215558	0.685
**528**	7-KW-B22-	3000	BET	0.679033	0
**529**	7-KW-C21-	100	BET	0.476144	0.002
**530**	7-KW-C22-	300	BET	0.095682	0.935
**531**	7-KW-D21-	10	BET	0.139531	0.905
**532**	7-KW-D22-	30	BET	0.389222	0.127
**533**	7-KW-E21-	1	BET	0.570069	0
**534**	7-KW-E22-	3	BET	0.568581	0
**535**	7-KW-G10-	10000	BET	0.593308	0
**536**	7-KW-G15-	10000	BET	0.596953	0
**537**	7-KW-H10-	1000	BET	0.654389	0
**538**	7-KW-H15-	1000	BET	0.673103	0
**539**	7-KW-I10-	100	BET	0.675848	0
**540**	7-KW-I15-	100	BET	0.580264	0
**541**	7-KW-J10-	10	BET	0.461176	0.018
**542**	7-KW-J15-	10	BET	0.310685	0.329
**543**	7-KW-K10-	1	BET	0.494074	0
**544**	7-KW-K13-	1	BET	0.426117	0.022
**545**	7-KW-K15-	1	BET	0.447339	0.01
**546**	7-KW-L12-	1	BET	0.244677	0.292
**547**	7-KW-L13-	10	BET	0.615232	0
**548**	7-KW-L20-	1	BET	0.313963	0.508

**549**	7-KW-L23-/	0.03	BET	0.39851	0.044
**550**	7-KW-M12-	10	BET	0.586047	0
**551**	7-KW-M13-	100	BET	0.58799	0
**552**	7-KW-M20-	10	BET	0.436034	0.019
**553**	7-KW-M23-	0.3	BET	0.399035	0.08
**554**	7-KW-N12-	100	BET	0.574301	0
**555**	7-KW-N13-	1000	BET	0.574105	0
**556**	7-KW-N20-	100	BET	0.675239	0
**557**	7-KW-N23-	3	BET	0.496406	0.001
**558**	7-KW-O12-	1000	BET	0.669549	0
**559**	7-KW-O20-	1000	BET	0.658631	0
**560**	7-KW-O23-	30	BET	0.647656	0
**561**	7-KW-P12-|	10000	BET	0.629953	0
**562**	7-KW-P13-|	10000	BET	0.57654	0
**563**	7-KW-P20-.	10000	BET	0.580172	0
**564**	7-KW-P23-,	300	BET	0.596893	0
**565**	8-KW-K22-(	1	BET	0.130054	0.987
**566**	8-KW-L22-(	10	BET	0.151518	0.775
**567**	8-KW-M22-	100	BET	0.395822	0.046
**568**	8-KW-N22-	1000	BET	0.583817	0
**569**	8-KW-O22-	10000	BET	0.545309	0
**570**	1-KW-A3-V(	10000	HDAC	0.403405	0.007
**571**	1-KW-B3-V(	1000	HDAC	0.448695	0
**572**	1-KW-C3-V(	100	HDAC	0.046488	0.968
**573**	1-KW-D3-V	10	HDAC	0.070594	0.95
**574**	1-KW-E3-V(	1	HDAC	0.067115	0.729
**575**	1-KW-L12-I	0.1	HDAC	0.327166	0.029
**576**	1-KW-M12-	1	HDAC	0.303626	0.127
**577**	1-KW-N12-	10	HDAC	0.421098	0.005
**578**	1-KW-O12-	100	HDAC	0.425723	0.007
**579**	1-KW-P12-|	1000	HDAC	0.426448	0.005
**580**	3-KW-A4-P;	1000	HDAC	0.433983	0
**581**	3-KW-B4-P;	100	HDAC	0.426187	0.002
**582**	3-KW-C4-P;	10	HDAC	0.455652	0
**583**	3-KW-D4-P	1	HDAC	0.176558	0.856

**584**	3-KW-E4-Pa	0.1	HDAC	0.033622	1
**585**	3-KW-F7-Q	1000	HDAC	0.425784	0
**586**	3-KW-G7-Q	100	HDAC	0.414515	0.001
**587**	3-KW-G12-	1000000	HDAC	0.120041	0.912
**588**	3-KW-H7-Q	10	HDAC	0.435958	0
**589**	3-KW-H12-	100000	HDAC	0.358704	0.022
**590**	3-KW-I7-Qu	1	HDAC	0.500789	0
**591**	3-KW-I12-V	10000	HDAC	0.110889	0.512
**592**	3-KW-J7-Qu	0.1	HDAC	0.036747	0.962
**593**	3-KW-J12-V	1000	HDAC	0.313978	0.019
**594**	3-KW-K3-Be	1	HDAC	0.04923	1
**595**	3-KW-K12-	100	HDAC	0.340011	0.05
**596**	3-KW-L3-Be	10	HDAC	0.292049	0.283
**597**	3-KW-M3-B	100	HDAC	0.369538	0.005
**598**	3-KW-N3-B	1000	HDAC	0.408643	0.003
**599**	3-KW-O3-B	10000	HDAC	0.434455	0.001
**600**	7-KW-A5-M	10000	HDAC	0.448872	0.001
**601**	7-KW-A7-C	10000	HDAC	0.476619	0
**602**	7-KW-A9-G	1000	HDAC	0.469324	0
**603**	7-KW-A12-I	10000	HDAC	0.498674	0
**604**	7-KW-B5-M	1000	HDAC	0.559034	0
**605**	7-KW-B7-C	1000	HDAC	0.466141	0.001
**606**	7-KW-B12-I	1000	HDAC	0.616805	0
**607**	7-KW-C5-M	100	HDAC	0.440313	0
**608**	7-KW-C7-C	100	HDAC	0.452085	0.002
**609**	7-KW-C9-G	100	HDAC	0.614212	0
**610**	7-KW-D7-C	10	HDAC	0.069063	0.994
**611**	7-KW-D9-G	10	HDAC	0.517781	0
**612**	7-KW-D12-	100	HDAC	0.471466	0
**613**	7-KW-E5-M	10	HDAC	0.257645	0.18
**614**	7-KW-E7-Cl	1	HDAC	0.402815	0.001
**615**	7-KW-E9-Gl	1	HDAC	0.14909	0.363
**616**	7-KW-E12-I	10	HDAC	0.447288	0
**617**	7-KW-F5-M	1	HDAC	0.068975	0.616
**618**	7-KW-F7-Re	10000	HDAC	0.45866	0

**619**	7-KW-F9-Gi	0.1	HDAC	0.079691	0.573
**620**	7-KW-F12-I	1	HDAC	0.542478	0
**621**	7-KW-F19-I	10000	HDAC	0.414654	0
**622**	7-KW-G7-R	1000	HDAC	0.467527	0
**623**	7-KW-G19-	1000	HDAC	0.315375	0.279
**624**	7-KW-H7-R	100	HDAC	0.482695	0
**625**	7-KW-I7-Re	10	HDAC	0.415842	0
**626**	7-KW-I19-F	100	HDAC	0.44243	0
**627**	7-KW-J7-Re	1	HDAC	0.163129	0.167
**628**	7-KW-J19-F	10	HDAC	0.054571	0.751
**629**	7-KW-K4-Er	1	HDAC	0.428193	0
**630**	7-KW-K11-,	1	HDAC	0.524959	0
**631**	7-KW-K18-'	1	HDAC	0.448109	0
**632**	7-KW-K19-I	1	HDAC	0.474988	0
**633**	7-KW-L2-Ta	0.1	HDAC	0.235701	0.707
**634**	7-KW-L4-Er	10	HDAC	0.271829	0.235
**635**	7-KW-L5-Pr	1	HDAC	0.452581	0
**636**	7-KW-L8-Al	1	HDAC	0.397334	0.003
**637**	7-KW-L10-'	1	HDAC	0.512189	0
**638**	7-KW-L11-/	10	HDAC	0.629349	0
**639**	7-KW-L14-'	1	HDAC	0.187291	0.261
**640**	7-KW-L16-I	1	HDAC	0.454304	0
**641**	7-KW-L18-'	10	HDAC	0.496565	0
**642**	7-KW-M2-T	1	HDAC	0.42692	0.001
**643**	7-KW-M5-F	10	HDAC	0.422607	0.001
**644**	7-KW-M8-A	10	HDAC	0.417622	0
**645**	7-KW-M10-	10	HDAC	0.088573	0.659
**646**	7-KW-M11-	100	HDAC	0.534778	0
**647**	7-KW-M14-	10	HDAC	0.468275	0
**648**	7-KW-M16-	10	HDAC	0.418866	0
**649**	7-KW-M18-	100	HDAC	0.375815	0.004
**650**	7-KW-N2-Ti	10	HDAC	0.276451	0.164
**651**	7-KW-N4-Er	100	HDAC	0.506313	0
**652**	7-KW-N5-P	100	HDAC	0.503949	0
**653**	7-KW-N8-A	100	HDAC	0.511319	0

**654**	7-KW-N10-	100	HDAC	0.516509	0
**655**	7-KW-N14-	100	HDAC	0.378903	0
**656**	7-KW-N16-	100	HDAC	0.443922	0
**657**	7-KW-N18-	1000	HDAC	0.458115	0
**658**	7-KW-O2-T	100	HDAC	0.444593	0
**659**	7-KW-O4-E	1000	HDAC	0.523823	0
**660**	7-KW-O5-P	1000	HDAC	0.460384	0.001
**661**	7-KW-O8-A	1000	HDAC	0.467988	0
**662**	7-KW-O10-	1000	HDAC	0.507326	0
**663**	7-KW-O11-	1000	HDAC	0.466032	0
**664**	7-KW-O14-	1000	HDAC	0.378842	0.003
**665**	7-KW-O16-	1000	HDAC	0.467399	0
**666**	7-KW-P2-T	1000	HDAC	0.448145	0
**667**	7-KW-P4-E	10000	HDAC	0.44723	0
**668**	7-KW-P5-P	10000	HDAC	0.462621	0.003
**669**	7-KW-P8-A	10000	HDAC	0.454833	0
**670**	7-KW-P10-	10000	HDAC	0.467412	0
**671**	7-KW-P11-	10000	HDAC	0.450917	0
**672**	7-KW-P14-	10000	HDAC	0.542844	0
**673**	7-KW-P16-	10000	HDAC	0.455535	0
**674**	7-KW-P18-	10000	HDAC	0.445872	0.002
**0**	2-MHB-A16	10000	EGFR	0.467657	0
**1**	2-MHB-A19	10000	EGFR	0.461521	0
**2**	2-MHB-B19	1000	EGFR	0.497501	0
**3**	2-MHB-C16	1000	EGFR	0.530169	0
**4**	2-MHB-C19	100	EGFR	0.49251	0
**5**	2-MHB-D16	100	EGFR	0.626435	0
**6**	2-MHB-D19	10	EGFR	0.079041	0.963
**7**	2-MHB-E16	10	EGFR	0.537825	0
**8**	2-MHB-E19	1	EGFR	0.053634	0.982
**9**	2-MHB-F16	1	EGFR	0.514985	0
**10**	2-MHB-K11	0.1	EGFR	0.505711	0
**11**	2-MHB-L11	1	EGFR	0.591624	0
**12**	2-MHB-L16	0.25	EGFR	0.040257	0.996
**13**	2-MHB-L19	0.1	EGFR	0.616301	0

**14**	2-MHB-M1	10	EGFR	0.565814	0
**15**	2-MHB-M1	2.5	EGFR	0.61465	0
**16**	2-MHB-M1	1	EGFR	0.64446	0
**17**	2-MHB-N1	25	EGFR	0.562601	0
**18**	2-MHB-N1	10	EGFR	0.631942	0
**19**	2-MHB-O11	100	EGFR	0.543452	0
**20**	2-MHB-O1	250	EGFR	0.4834	0
**21**	2-MHB-O1	100	EGFR	0.520933	0
**22**	2-MHB-P11	1000	EGFR	0.433622	0
**23**	2-MHB-P1	2500	EGFR	0.510554	0
**24**	2-MHB-P1	1000	EGFR	0.459125	0
**25**	3-MHB-F21	10000	EGFR	0.393246	0.003
**26**	3-MHB-G2	1000	EGFR	0.426196	0
**27**	3-MHB-G21	1000	EGFR	0.418967	0
**28**	3-MHB-H2	100	EGFR	0.466141	0
**29**	3-MHB-H21	100	EGFR	0.141292	0.959
**30**	3-MHB-I20	10	EGFR	0.482711	0
**31**	3-MHB-I21	10	EGFR	0.123375	0.918
**32**	3-MHB-J20	1	EGFR	0.029235	1
**33**	3-MHB-J21	1	EGFR	0.413677	0.015
**34**	3-MHB-K4-	1	EGFR	0.457237	0
**35**	3-MHB-K18	0.1	EGFR	0.489264	0
**36**	3-MHB-K2	0.1	EGFR	0.494402	0
**37**	3-MHB-L4-	10	EGFR	0.474195	0
**38**	3-MHB-L18	1	EGFR	0.499192	0
**39**	3-MHB-M1	10	EGFR	0.458149	0
**40**	3-MHB-N4-	100	EGFR	0.458415	0
**41**	3-MHB-N1	100	EGFR	0.484617	0
**42**	3-MHB-O4-	1000	EGFR	0.452834	0
**43**	3-MHB-P4-	10000	EGFR	0.392657	0.007
**44**	3-MHB-P1	1000	EGFR	0.410491	0.001
**45**	4-MHB-F13	1000	EGFR	0.491071	0
**46**	4-MHB-G1	100	EGFR	0.49362	0
**47**	4-MHB-G1	10000	EGFR	0.478003	0
**48**	4-MHB-H1	10	EGFR	0.587322	0

49	4-MHB-H16	1000	EGFR	0.478654	0
50	4-MHB-I13-	1	EGFR	0.508989	0
51	4-MHB-I16-	100	EGFR	0.607822	0
52	4-MHB-J13	0.1	EGFR	0.190776	0.214
53	4-MHB-J16	10	EGFR	0.050096	0.93
54	4-MHB-K7-I	1	EGFR	0.500419	0
55	4-MHB-K13	0.1	EGFR	0.495345	0
56	4-MHB-K16	1	EGFR	0.055317	0.93
57	4-MHB-L7-I	10	EGFR	0.498259	0
58	4-MHB-L13	1	EGFR	0.516397	0
59	4-MHB-M7-	100	EGFR	0.496006	0
60	4-MHB-M1	10	EGFR	0.527129	0
61	4-MHB-N13	100	EGFR	0.523572	0
62	4-MHB-O7-	1000	EGFR	0.611496	0
63	4-MHB-P7-I	10000	EGFR	0.552843	0
64	4-MHB-P13	1000	EGFR	0.449964	0
65	5-MHB-F4-I	1000	EGFR	0.550679	0
66	5-MHB-F7-/	1000	EGFR	0.507812	0
67	5-MHB-G4-	100	EGFR	0.578163	0
68	5-MHB-G7-	100	EGFR	0.505365	0
69	5-MHB-H4-	10	EGFR	0.484268	0
70	5-MHB-H7-	10	EGFR	0.530777	0
71	5-MHB-I4-F	1	EGFR	0.456501	0
72	5-MHB-I7-A	1	EGFR	0.04116	0.965
73	5-MHB-J4-F	0.1	EGFR	0.501444	0
74	5-MHB-J7-/	0.1	EGFR	0.523062	0
75	5-MHB-K7-(	0.1	EGFR	0.571996	0
76	5-MHB-L7-(	1	EGFR	0.023246	1
77	5-MHB-M7-	10	EGFR	0.048388	0.996
78	5-MHB-O7-	100	EGFR	0.433756	0.001
79	5-MHB-P7-(	1000	EGFR	0.430311	0
80	2-MHB-A15	2500	VEGFR	0.077849	1
81	2-MHB-A17	10000	VEGFR	0.079037	0.999
82	2-MHB-A20	10000	VEGFR	0.068939	1
83	2-MHB-B15	250	VEGFR	0.117264	0.966

**84**	2-MHB-B17	1000	VEGFR	0.139562	0.934
**85**	2-MHB-B20	1000	VEGFR	0.28837	0.144
**86**	2-MHB-C15	25	VEGFR	0.151072	0.946
**87**	2-MHB-C17	100	VEGFR	0.188121	0.646
**88**	2-MHB-D15	2.5	VEGFR	0.38804	0
**89**	2-MHB-D17	10	VEGFR	0.231232	0.365
**90**	2-MHB-D20	100	VEGFR	0.278661	0.003
**91**	2-MHB-E17	1	VEGFR	0.318565	0.004
**92**	2-MHB-E20	10	VEGFR	0.383147	0.002
**93**	2-MHB-F13	10000	VEGFR	0.237004	0.371
**94**	2-MHB-F15	0.25	VEGFR	0.239764	0
**95**	2-MHB-F19	10000	VEGFR	0.076897	0.999
**96**	2-MHB-F20	1	VEGFR	0.282099	0
**97**	2-MHB-F21	10000	VEGFR	0.254321	0.005
**98**	2-MHB-G10	10000	VEGFR	0.17462	0.049
**99**	2-MHB-G13	1000	VEGFR	0.28954	0.222
**100**	2-MHB-G19	1000	VEGFR	0.119709	0.989
**101**	2-MHB-G21	1000	VEGFR	0.300569	0.051
**102**	2-MHB-H10	1000	VEGFR	0.247764	0.001
**103**	2-MHB-H13	100	VEGFR	0.331022	0.019
**104**	2-MHB-H21	100	VEGFR	0.389317	0.009
**105**	2-MHB-I10-	100	VEGFR	0.244241	0
**106**	2-MHB-I13-	10	VEGFR	0.301497	0.021
**107**	2-MHB-I19-	100	VEGFR	0.208255	0.71
**108**	2-MHB-I21-	10	VEGFR	0.300428	0.001
**109**	2-MHB-J10	10	VEGFR	0.21404	0.002
**110**	2-MHB-J13	1	VEGFR	0.264607	0.001
**111**	2-MHB-J19	10	VEGFR	0.402374	0
**112**	2-MHB-J21	1	VEGFR	0.26671	0.001
**113**	2-MHB-K10	1	VEGFR	0.237247	0.008
**114**	2-MHB-K13	0.1	VEGFR	0.351872	0.01
**115**	2-MHB-K17	1	VEGFR	0.404139	0
**116**	2-MHB-K19	1	VEGFR	0.236668	0.001
**117**	2-MHB-L12	0.1	VEGFR	0.244988	0.002
**118**	2-MHB-L13	1	VEGFR	0.245734	0.32

**119**	2-MHB-L21	0.1	VEGFR	0.371498	0.004
**120**	2-MHB-M1[illegible]	1	VEGFR	0.20934	0.702
**121**	2-MHB-M1[illegible]	10	VEGFR	0.24426	0.003
**122**	2-MHB-M1[illegible]	10	VEGFR	0.173202	0.875
**123**	2-MHB-M2[illegible]	1	VEGFR	0.256461	0.001
**124**	2-MHB-N12	10	VEGFR	0.238107	0
**125**	2-MHB-N13	100	VEGFR	0.184021	0.72
**126**	2-MHB-N17	100	VEGFR	0.296958	0.07
**127**	2-MHB-N21	10	VEGFR	0.302943	0.001
**128**	2-MHB-O12	100	VEGFR	0.181328	0.492
**129**	2-MHB-O17	1000	VEGFR	0.24057	0.394
**130**	2-MHB-O21	100	VEGFR	0.302219	0
**131**	2-MHB-P12	1000	VEGFR	0.201536	0.647
**132**	2-MHB-P13	1000	VEGFR	0.122611	0.972
**133**	2-MHB-P17	10000	VEGFR	0.182089	0.252
**134**	2-MHB-P21	1000	VEGFR	0.151525	0.911
**135**	3-MHB-A3-[illegible]	1000	VEGFR	0.151246	0.937
**136**	3-MHB-A6-[illegible]	1000	VEGFR	0.088181	0.999
**137**	3-MHB-A18	1000	VEGFR	0.168521	0.884
**138**	3-MHB-B3-[illegible]	100	VEGFR	0.157843	0.917
**139**	3-MHB-B6-[illegible]	100	VEGFR	0.181113	0.722
**140**	3-MHB-B18	100	VEGFR	0.112351	0.978
**141**	3-MHB-C3-[illegible]	10	VEGFR	0.192626	0.688
**142**	3-MHB-C6-[illegible]	10	VEGFR	0.211631	0.48
**143**	3-MHB-C18	10	VEGFR	0.117469	0.997
**144**	3-MHB-D3-[illegible]	1	VEGFR	0.217615	0.466
**145**	3-MHB-D6-[illegible]	1	VEGFR	0.22081	0.698
**146**	3-MHB-D18	1	VEGFR	0.169162	0.893
**147**	3-MHB-E3-[illegible]	0.1	VEGFR	0.20031	0.158
**148**	3-MHB-E6-[illegible]	0.1	VEGFR	0.173183	0.487
**149**	3-MHB-E18	0.1	VEGFR	0.166064	0.831
**150**	3-MHB-F18	1000	VEGFR	0.197331	0.739
**151**	3-MHB-G18	100	VEGFR	0.203255	0.05
**152**	3-MHB-H18	10	VEGFR	0.170873	0.082
**153**	3-MHB-I18-[illegible]	1	VEGFR	0.159729	0.041

**154**	3-MHB-J18	0.1	VEGFR	0.188925	0.039
**155**	4-MHB-A12	10000	VEGFR	0.091992	1
**156**	4-MHB-A15	2500	VEGFR	0.181493	0.675
**157**	4-MHB-A20	10000	VEGFR	0.129386	0.917
**158**	4-MHB-B12	1000	VEGFR	0.075327	0.998
**159**	4-MHB-B15	250	VEGFR	0.12118	0.968
**160**	4-MHB-B20	1000	VEGFR	0.099217	0.995
**161**	4-MHB-C15	25	VEGFR	0.112371	0.994
**162**	4-MHB-D12	100	VEGFR	0.252681	0.328
**163**	4-MHB-D15	2.5	VEGFR	0.281034	0.155
**164**	4-MHB-D20	100	VEGFR	0.16622	0.864
**165**	4-MHB-E12	10	VEGFR	0.244048	0
**166**	4-MHB-E20	10	VEGFR	0.120161	0.994
**167**	4-MHB-F12	1	VEGFR	0.275119	0.222
**168**	4-MHB-F15	0.25	VEGFR	0.24794	0.575
**169**	4-MHB-F20	1	VEGFR	0.139971	0.956
**170**	4-MHB-L16	1	VEGFR	0.264111	0.215
**171**	4-MHB-M1	10	VEGFR	0.330224	0.085
**172**	4-MHB-N16	100	VEGFR	0.106284	0.994
**173**	4-MHB-O16	1000	VEGFR	0.091163	0.993
**174**	4-MHB-P16	10000	VEGFR	0.203246	0.262
**175**	2-MHB-L10	1	PI3K	0.204253	0.586
**176**	2-MHB-M1	10	PI3K	0.098922	0.635
**177**	2-MHB-N10	100	PI3K	0.11272	0.995
**178**	2-MHB-O10	1000	PI3K	0.162148	0.865
**179**	2-MHB-P10	10000	PI3K	0.187455	0.842
**180**	3-MHB-A16	2500	PI3K	0.148227	0.959
**181**	3-MHB-C16	250	PI3K	0.272255	0.118
**182**	3-MHB-D16	25	PI3K	0.111288	0.844
**183**	3-MHB-E16	2.5	PI3K	0.337726	0.018
**184**	3-MHB-F16	0.25	PI3K	0.279496	0.077
**185**	3-MHB-F17	100000	PI3K	0.187864	0.805
**186**	3-MHB-F19	500	PI3K	0.232116	0.282
**187**	3-MHB-G17	10000	PI3K	0.156681	0.009
**188**	3-MHB-G19	50	PI3K	0.243608	0.197

**189**	3-MHB-H17	1000	PI3K	0.146833	0.107
**190**	3-MHB-I17-	100	PI3K	0.138557	0.197
**191**	3-MHB-I19-	5	PI3K	0.316685	0.004
**192**	3-MHB-J17	10	PI3K	0.157053	0.059
**193**	3-MHB-J19	0.5	PI3K	0.271666	0.009
**194**	3-MHB-K19	0.05	PI3K	0.187357	0.012
**195**	3-MHB-L8-I	1	PI3K	0.329765	0.057
**196**	3-MHB-L21	0.1	PI3K	0.331105	0.003
**197**	3-MHB-M8-	10	PI3K	0.175812	0.236
**198**	3-MHB-M2	1	PI3K	0.202282	0.025
**199**	3-MHB-N8-	100	PI3K	0.243789	0.219
**200**	3-MHB-N21	10	PI3K	0.277247	0.08
**201**	3-MHB-O8-	1000	PI3K	0.223122	0.532
**202**	3-MHB-O21	100	PI3K	0.207074	0.694
**203**	3-MHB-P8-I	10000	PI3K	0.184837	0.892
**204**	3-MHB-P21	1000	PI3K	0.190718	0.815
**205**	4-MHB-A19	2500	PI3K	0.198651	0.782
**206**	4-MHB-B19	250	PI3K	0.19811	0.788
**207**	4-MHB-C19	25	PI3K	0.207827	0.645
**208**	4-MHB-D19	2.5	PI3K	0.205925	0.673
**209**	4-MHB-E19	0.25	PI3K	0.28728	0.095
**210**	4-MHB-G2-	2500	PI3K	0.231032	0.25
**211**	4-MHB-G5-	10000	PI3K	0.293251	0.026
**212**	4-MHB-G14	100	PI3K	0.254696	0.283
**213**	4-MHB-G20	10000	PI3K	0.213923	0.726
**214**	4-MHB-H2-	250	PI3K	0.260283	0.168
**215**	4-MHB-H5-	1000	PI3K	0.338352	0.002
**216**	4-MHB-H14	10	PI3K	0.270467	0.065
**217**	4-MHB-H20	1000	PI3K	0.250139	0.298
**218**	4-MHB-I2-T	25	PI3K	0.144409	0.505
**219**	4-MHB-I5-S	100	PI3K	0.290507	0.114
**220**	4-MHB-I14-	1	PI3K	0.130148	0.113
**221**	4-MHB-I20-	100	PI3K	0.275609	0.072
**222**	4-MHB-J2-T	2.5	PI3K	0.290657	0.121
**223**	4-MHB-J5-S	10	PI3K	0.212748	0.054

**224**	4-MHB-J20	10	PI3K	0.369937	0.002
**225**	4-MHB-K2-[illegible]	0.25	PI3K	0.265521	0.073
**226**	4-MHB-K4-[illegible]	0.1	PI3K	0.35823	0.015
**227**	4-MHB-K5-[illegible]	1	PI3K	0.433812	0.001
**228**	4-MHB-K14	0.1	PI3K	0.128601	0.273
**229**	4-MHB-K20	1	PI3K	0.338653	0.009
**230**	4-MHB-L4-[illegible]	1	PI3K	0.160197	0.184
**231**	4-MHB-L14	0.1	PI3K	0.283065	0.138
**232**	4-MHB-L15	1	PI3K	0.088215	0.631
**233**	4-MHB-L21	0.1	PI3K	0.315936	0.015
**234**	4-MHB-M1[illegible]	1	PI3K	0.19193	0.065
**235**	4-MHB-M1[illegible]	10	PI3K	0.272192	0.032
**236**	4-MHB-M2[illegible]	1	PI3K	0.356057	0
**237**	4-MHB-N4-	10	PI3K	0.310233	0.007
**238**	4-MHB-N1[illegible]	10	PI3K	0.392977	0
**239**	4-MHB-N1[illegible]	100	PI3K	0.287447	0.06
**240**	4-MHB-N21	10	PI3K	0.24258	0.314
**241**	4-MHB-O4-	100	PI3K	0.212679	0.653
**242**	4-MHB-O1[illegible]	100	PI3K	0.253319	0.184
**243**	4-MHB-O1[illegible]	1000	PI3K	0.233051	0.222
**244**	4-MHB-O21	100	PI3K	0.236957	0.477
**245**	4-MHB-P4-[illegible]	1000	PI3K	0.202123	0.753
**246**	4-MHB-P14	1000	PI3K	0.223315	0.74
**247**	4-MHB-P15	10000	PI3K	0.149283	0.956
**248**	4-MHB-P21	1000	PI3K	0.196009	0.89
**249**	5-MHB-A6-[illegible]	2500	PI3K	0.341546	0.002
**250**	5-MHB-A7-[illegible]	1000	PI3K	0.185409	0.574
**251**	5-MHB-A16	2500	PI3K	0.17125	0.933
**252**	5-MHB-A17	10000	PI3K	0.161338	0.917
**253**	5-MHB-B6-[illegible]	250	PI3K	0.236218	0.297
**254**	5-MHB-B7-[illegible]	100	PI3K	0.190753	0.727
**255**	5-MHB-B17	1000	PI3K	0.167599	0.892
**256**	5-MHB-C6-[illegible]	25	PI3K	0.20113	0.318
**257**	5-MHB-C7-[illegible]	10	PI3K	0.114899	0.692
**258**	5-MHB-C16	250	PI3K	0.260472	0.008

**259**	5-MHB-C17	100	PI3K	0.16846	0.043
**260**	5-MHB-D6-	2.5	PI3K	0.229266	0.096
**261**	5-MHB-D7-	1	PI3K	0.387698	0.006
**262**	5-MHB-D16	25	PI3K	0.183868	0.494
**263**	5-MHB-D17	10	PI3K	0.076692	0.687
**264**	5-MHB-E6-I	0.25	PI3K	0.166932	0.041
**265**	5-MHB-E7-	0.1	PI3K	0.236749	0.001
**266**	5-MHB-E16	2.5	PI3K	0.123357	0.18
**267**	5-MHB-E17	1	PI3K	0.187976	0
**268**	5-MHB-F11	10000	PI3K	0.2402	0.004
**269**	5-MHB-F16	0.25	PI3K	0.151315	0.031
**270**	5-MHB-G9-	10000	PI3K	0.197252	0.78
**271**	5-MHB-G11	1000	PI3K	0.1873	0.012
**272**	5-MHB-H9-	1000	PI3K	0.361831	0
**273**	5-MHB-H11	100	PI3K	0.336714	0.007
**274**	5-MHB-I9-S	100	PI3K	0.265092	0.098
**275**	5-MHB-I11-	10	PI3K	0.103436	0.975
**276**	5-MHB-J9-S	10	PI3K	0.102879	0.345
**277**	5-MHB-J11	1	PI3K	0.145261	0.382
**278**	5-MHB-K9-	1	PI3K	0.331767	0.003
**279**	5-MHB-L14	0.1	PI3K	0.117251	0.258
**280**	5-MHB-L20	1	PI3K	0.268432	0.046
**281**	5-MHB-L23	0.1	PI3K	0.118581	0.835
**282**	5-MHB-M1	1	PI3K	0.275467	0.197
**283**	5-MHB-M2	10	PI3K	0.155803	0.043
**284**	5-MHB-M2	1	PI3K	0.253503	0.182
**285**	5-MHB-N1	10	PI3K	0.313003	0.047
**286**	5-MHB-N2	100	PI3K	0.401546	0.002
**287**	5-MHB-N2	10	PI3K	0.202881	0.766
**288**	5-MHB-O1	100	PI3K	0.223212	0.268
**289**	5-MHB-O2	1000	PI3K	0.218341	0.602
**290**	5-MHB-O2	100	PI3K	0.188016	0.872
**291**	5-MHB-P14	1000	PI3K	0.202082	0.594
**292**	5-MHB-P2	10000	PI3K	0.193145	0.818
**293**	5-MHB-P23	1000	PI3K	0.197489	0.853

**294**	6-MHB-A8-	10000	PI3K	0.180831	0.791
**295**	6-MHB-B8-	1000	PI3K	0.154438	0.908
**296**	6-MHB-C8-	100	PI3K	0.099516	0.998
**297**	6-MHB-D8-	10	PI3K	0.20306	0.725
**298**	6-MHB-E8-	1	PI3K	0.070948	0.769
**299**	6-MHB-L6-	1	PI3K	0.213771	0.575
**300**	6-MHB-M6-	10	PI3K	0.328038	0.194
**301**	6-MHB-N6-	100	PI3K	0.226602	0.368
**302**	6-MHB-O6-	1000	PI3K	0.197532	0.738
**303**	6-MHB-P6-	10000	PI3K	0.221721	0.547
**304**	1-MHB-F11	10000	Topoisome	0.314631	0.22
**305**	1-MHB-G11	1000	Topoisome	0.421335	0.002
**306**	1-MHB-G20	1000	Topoisome	0.331181	0.122
**307**	1-MHB-H11	100	Topoisome	0.482796	0
**308**	1-MHB-H20	100	Topoisome	0.376186	0.021
**309**	1-MHB-I11-	10	Topoisome	0.122229	0.617
**310**	1-MHB-I20-	10	Topoisome	0.114627	0.614
**311**	1-MHB-J11	1	Topoisome	0.438085	0.003
**312**	1-MHB-J20	1	Topoisome	0.075082	0.761
**313**	1-MHB-K11	1	Topoisome	0.425337	0.006
**314**	1-MHB-K20	0.1	Topoisome	0.184343	0.831
**315**	1-MHB-L11	10	Topoisome	0.37364	0.022
**316**	1-MHB-L14	1	Topoisome	0.462679	0.002
**317**	1-MHB-M1	100	Topoisome	0.334111	0.103
**318**	1-MHB-M1	10	Topoisome	0.446581	0.001
**319**	1-MHB-N14	100	Topoisome	0.505804	0
**320**	1-MHB-O11	1000	Topoisome	0.3569	0.063
**321**	1-MHB-O14	1000	Topoisome	0.355024	0.058
**322**	1-MHB-P11	10000	Topoisome	0.322998	0.198
**323**	1-MHB-P14	10000	Topoisome	0.314264	0.171
**324**	3-MHB-A11	10000	Topoisome	0.337168	0.084
**325**	3-MHB-B11	1000	Topoisome	0.371948	0.027
**326**	3-MHB-C11	100	Topoisome	0.286287	0.237
**327**	3-MHB-D11	10	Topoisome	0.492183	0
**328**	3-MHB-E11	1	Topoisome	0.417506	0

**329**	3-MHB-G9-	1000	Topoisome	0.335095	0.161
**330**	3-MHB-G1(	10000	Topoisome	0.117053	0.606
**331**	3-MHB-H9-	100	Topoisome	0.392	0.014
**332**	3-MHB-H1(	1000	Topoisome	0.60856	0
**333**	3-MHB-I9-D	10	Topoisome	0.531975	0
**334**	3-MHB-I10-	100	Topoisome	0.22353	0.205
**335**	3-MHB-J9-D	1	Topoisome	0.1504	0.552
**336**	3-MHB-J10	10	Topoisome	0.112867	0.496
**337**	3-MHB-K7-I	0.1	Topoisome	0.344481	0.03
**338**	3-MHB-K9-I	0.1	Topoisome	0.409147	0.002
**339**	3-MHB-K10	1	Topoisome	0.454278	0.009
**340**	3-MHB-L6-I	0.1	Topoisome	0.468932	0
**341**	3-MHB-L7-I	1	Topoisome	0.323964	0.017
**342**	3-MHB-L9-V	0.5	Topoisome	0.223334	0.271
**343**	3-MHB-L10	0.1	Topoisome	0.562426	0
**344**	3-MHB-L16	1	Topoisome	0.444327	0
**345**	3-MHB-M6-	1	Topoisome	0.421817	0.023
**346**	3-MHB-M7-	10	Topoisome	0.444264	0.001
**347**	3-MHB-M9-	5	Topoisome	0.441114	0
**348**	3-MHB-M1(	1	Topoisome	0.236241	0.108
**349**	3-MHB-M1(	10	Topoisome	0.443219	0
**350**	3-MHB-N6-	10	Topoisome	0.367894	0.059
**351**	3-MHB-N9-	50	Topoisome	0.447493	0
**352**	3-MHB-N1(	10	Topoisome	0.421282	0.001
**353**	3-MHB-N16	100	Topoisome	0.083755	0.91
**354**	3-MHB-O6-	100	Topoisome	0.443225	0
**355**	3-MHB-O7-	100	Topoisome	0.392829	0.009
**356**	3-MHB-O9-	500	Topoisome	0.423609	0.002
**357**	3-MHB-O1(	100	Topoisome	0.39293	0.006
**358**	3-MHB-O16	1000	Topoisome	0.490108	0
**359**	3-MHB-P6-I	1000	Topoisome	0.349974	0.068
**360**	3-MHB-P7-I	1000	Topoisome	0.33693	0.139
**361**	3-MHB-P9-V	5000	Topoisome	0.346731	0.047
**362**	3-MHB-P1(	1000	Topoisome	0.341358	0.094
**363**	3-MHB-P16	10000	Topoisome	0.386572	0.006

**364**	1-MHB-A1(	10000	Mitotic	0.539118	0
**365**	1-MHB-A13	1000	Mitotic	0.540038	0
**366**	1-MHB-A18	1000	Mitotic	0.539211	0
**367**	1-MHB-B1(	1000	Mitotic	0.487672	0.002
**368**	1-MHB-B13	100	Mitotic	0.292369	0.023
**369**	1-MHB-B18	100	Mitotic	0.535447	0
**370**	1-MHB-C1(	100	Mitotic	0.543994	0
**371**	1-MHB-C13	10	Mitotic	0.131887	0.436
**372**	1-MHB-C18	10	Mitotic	0.499022	0
**373**	1-MHB-D1(	10	Mitotic	0.259902	0.694
**374**	1-MHB-D13	1	Mitotic	0.186151	0.882
**375**	1-MHB-D18	1	Mitotic	0.556952	0
**376**	1-MHB-E10	1	Mitotic	0.25876	0.05
**377**	1-MHB-E13	0.1	Mitotic	0.173111	0.382
**378**	1-MHB-E18	0.1	Mitotic	0.26133	0.047
**379**	1-MHB-F13	1000	Mitotic	0.479402	0
**380**	1-MHB-G13	100	Mitotic	0.543663	0
**381**	1-MHB-G15	1000	Mitotic	0.500502	0
**382**	1-MHB-H13	10	Mitotic	0.515577	0
**383**	1-MHB-H15	100	Mitotic	0.561874	0
**384**	1-MHB-I13-	1	Mitotic	0.269359	0.05
**385**	1-MHB-I15-	10	Mitotic	0.515399	0
**386**	1-MHB-J13	0.1	Mitotic	0.415562	0.044
**387**	1-MHB-J15	1	Mitotic	0.429208	0.003
**388**	1-MHB-K7-'	0.1	Mitotic	0.149815	0.494
**389**	1-MHB-K15	0.1	Mitotic	0.314575	0.011
**390**	1-MHB-L7-\	1	Mitotic	0.470947	0
**391**	1-MHB-L20	0.1	Mitotic	0.06224	0.924
**392**	1-MHB-M7-	10	Mitotic	0.404778	0.028
**393**	1-MHB-M2(	1	Mitotic	0.212871	0.124
**394**	1-MHB-N2(	10	Mitotic	0.306408	0.064
**395**	1-MHB-O7-	100	Mitotic	0.513606	0
**396**	1-MHB-O2(	100	Mitotic	0.306752	0.053
**397**	1-MHB-P7-'	1000	Mitotic	0.549444	0
**398**	1-MHB-P2(	1000	Mitotic	0.489361	0

**399**	3-MHB-A7-[illegible]	1000	Mitotic	0.464178	0.003
**400**	3-MHB-B7-[illegible]	100	Mitotic	0.437937	0.009
**401**	3-MHB-C7-[illegible]	10	Mitotic	0.458711	0.001
**402**	3-MHB-D7-[illegible]	1	Mitotic	0.459049	0.001
**403**	3-MHB-E7-[illegible]	0.1	Mitotic	0.088914	0.998
**404**	6-MHB-L19	1	Mitotic	0.245206	0.445
**405**	6-MHB-M1[illegible]	10	Mitotic	0.125838	0.576
**406**	6-MHB-N1[illegible]	100	Mitotic	0.257086	0.496
**407**	6-MHB-O1[illegible]	1000	Mitotic	0.464344	0
**408**	6-MHB-P1[illegible]	10000	Mitotic	0.438415	0.009
**409**	2-MHB-A12	250	MEK1/2	0.578395	0
**410**	2-MHB-B12	25	MEK1/2	0.617219	0
**411**	2-MHB-D1[illegible]	2.5	MEK1/2	0.615115	0
**412**	2-MHB-E12	0.25	MEK1/2	0.753884	0
**413**	2-MHB-F12	0.025	MEK1/2	0.718049	0
**414**	2-MHB-F14	1000	MEK1/2	0.583345	0
**415**	2-MHB-G1[illegible]	100	MEK1/2	0.566904	0
**416**	2-MHB-H1[illegible]	10	MEK1/2	0.63389	0
**417**	2-MHB-I14[illegible]	1	MEK1/2	0.682028	0
**418**	2-MHB-K14	0.1	MEK1/2	0.684942	0
**419**	2-MHB-L20	1	MEK1/2	0.379287	0.024
**420**	2-MHB-M2[illegible]	10	MEK1/2	0.627377	0
**421**	2-MHB-N2[illegible]	100	MEK1/2	0.54847	0
**422**	2-MHB-O2[illegible]	1000	MEK1/2	0.575336	0
**423**	2-MHB-P2[illegible]	10000	MEK1/2	0.518894	0
**424**	4-MHB-A1[illegible]	1000	MEK1/2	0.528613	0.002
**425**	4-MHB-A13	1000	MEK1/2	0.563328	0
**426**	4-MHB-B1[illegible]	100	MEK1/2	0.611104	0
**427**	4-MHB-B13	100	MEK1/2	0.596479	0
**428**	4-MHB-C1[illegible]	10	MEK1/2	0.696768	0
**429**	4-MHB-C13	10	MEK1/2	0.577797	0
**430**	4-MHB-D1[illegible]	1	MEK1/2	0.632975	0
**431**	4-MHB-D1[illegible]	1	MEK1/2	0.580985	0
**432**	4-MHB-E10	0.1	MEK1/2	0.700143	0
**433**	4-MHB-E13	0.1	MEK1/2	0.593135	0

**434**	4-MHB-L19	0.25	MEK1/2	0.557458	0.006
**435**	4-MHB-M1	2.5	MEK1/2	0.077264	0.999
**436**	4-MHB-N1	25	MEK1/2	0.582192	0.001
**437**	4-MHB-O1	250	MEK1/2	0.694156	0
**438**	4-MHB-P1	2500	MEK1/2	0.600386	0
**439**	1-MHB-L2-	1	PARP	0.59352	0.006
**440**	1-MHB-L6-	1	PARP	0.150392	0.879
**441**	1-MHB-M2-	10	PARP	0.128897	0.842
**442**	1-MHB-M6-	10	PARP	0.447899	0.128
**443**	1-MHB-N2-	100	PARP	0.463144	0.043
**444**	1-MHB-N6-	100	PARP	0.59133	0
**445**	1-MHB-O2-	1000	PARP	0.439942	0.044
**446**	1-MHB-O6-	1000	PARP	0.510362	0.03
**447**	1-MHB-P2-	10000	PARP	0.38171	0.191
**448**	1-MHB-P6-	10000	PARP	0.395141	0.168
**449**	7-MHB-A3-	1000	PARP	0.472022	0.028
**450**	7-MHB-B2-	10000	PARP	0.558504	0.002
**451**	7-MHB-B3-	100	PARP	0.471294	0.044
**452**	7-MHB-C2-	1000	PARP	0.262983	0.3
**453**	7-MHB-C3-	10	PARP	0.472608	0.06
**454**	7-MHB-D2-	100	PARP	0.58379	0.001
**455**	7-MHB-D3-	1	PARP	0.506906	0.007
**456**	7-MHB-E2-	10	PARP	0.512877	0.012
**457**	7-MHB-E3-	0.1	PARP	0.429778	0.06
**458**	7-MHB-F2-	1	PARP	0.557176	0
**459**	7-MHB-G2-	10000	PARP	0.514511	0.005
**460**	7-MHB-H2-	1000	PARP	0.558676	0.001
**461**	7-MHB-I2-	100	PARP	0.593015	0.001
**462**	7-MHB-J2-	10	PARP	0.523596	0.001
**463**	7-MHB-K2-	1	PARP	0.626818	0
**464**	3-MHB-A1	1000	CDK	0.362183	0.064
**465**	3-MHB-B1	100	CDK	0.379357	0.047
**466**	3-MHB-B2	2500	CDK	0.426425	0.003
**467**	3-MHB-C1	10	CDK	0.373216	0.029
**468**	3-MHB-C2	250	CDK	0.363792	0.034

**469**	3-MHB-D19	1	CDK	0.364706	0.022
**470**	3-MHB-D23	25	CDK	0.375126	0.035
**471**	3-MHB-E19	0.1	CDK	0.237801	0.483
**472**	3-MHB-E23	2.5	CDK	0.331647	0.145
**473**	3-MHB-F23	0.25	CDK	0.439551	0
**474**	3-MHB-K17	1	CDK	0.309949	0.246
**475**	3-MHB-L19	1	CDK	0.346945	0.06
**476**	3-MHB-M17	10	CDK	0.245242	0.483
**477**	3-MHB-M19	10	CDK	0.040398	0.965
**478**	3-MHB-N17	100	CDK	0.369569	0.044
**479**	3-MHB-N19	100	CDK	0.458457	0.011
**480**	3-MHB-O17	1000	CDK	0.380835	0.024
**481**	3-MHB-O19	1000	CDK	0.333868	0.034
**482**	3-MHB-P17	10000	CDK	0.195276	0.735
**483**	3-MHB-P19	10000	CDK	0.430917	0.003
**484**	4-MHB-A4-S	10000	CDK	0.35149	0.061
**485**	4-MHB-A8-	10000	CDK	0.388498	0.023
**486**	4-MHB-B4-S	1000	CDK	0.37699	0.04
**487**	4-MHB-B8-	1000	CDK	0.292043	0.23
**488**	4-MHB-C4-S	100	CDK	0.432046	0.016
**489**	4-MHB-C8-	100	CDK	0.290711	0.17
**490**	4-MHB-D4-	10	CDK	0.255571	0.091
**491**	4-MHB-D8-	10	CDK	0.341675	0.174
**492**	4-MHB-E4-S	1	CDK	0.189432	0.116
**493**	4-MHB-E8-	1	CDK	0.225189	0.342
**494**	4-MHB-F4-S	10000	CDK	0.219782	0.582
**495**	4-MHB-F22	10000	CDK	0.399431	0.012
**496**	4-MHB-G4-	1000	CDK	0.289815	0.191
**497**	4-MHB-G22	1000	CDK	0.359794	0.086
**498**	4-MHB-H4-	100	CDK	0.253618	0.257
**499**	4-MHB-H22	100	CDK	0.46036	0
**500**	4-MHB-I4-S	10	CDK	0.391491	0.048
**501**	4-MHB-I22-	10	CDK	0.295007	0.124
**502**	4-MHB-J4-S	1	CDK	0.289323	0.22
**503**	4-MHB-J22	1	CDK	0.407337	0.047

**504**	5-MHB-A19	10000	CDK	0.337498	0.125
**505**	5-MHB-B19	1000	CDK	0.331391	0.105
**506**	5-MHB-C19	100	CDK	0.458797	0.001
**507**	5-MHB-D19	10	CDK	0.086802	0.759
**508**	5-MHB-E19	1	CDK	0.108671	0.729
**509**	5-MHB-K17	1	CDK	0.162317	0.843
**510**	5-MHB-M1	10	CDK	0.100818	0.997
**511**	5-MHB-N17	100	CDK	0.175781	0.396
**512**	5-MHB-O17	1000	CDK	0.332082	0.154
**513**	5-MHB-P17	10000	CDK	0.400626	0.008
**514**	6-MHB-A17	1000	CDK	0.308068	0.156
**515**	6-MHB-B17	100	CDK	0.280396	0.307
**516**	6-MHB-C17	10	CDK	0.11575	0.62
**517**	6-MHB-D17	1	CDK	0.320419	0.115
**518**	6-MHB-E17	0.1	CDK	0.299427	0.474
**519**	6-MHB-L15	1	CDK	0.087256	0.993
**520**	6-MHB-M15	10	CDK	0.056842	0.936
**521**	6-MHB-N15	100	CDK	0.289972	0.258
**522**	6-MHB-O15	1000	CDK	0.340297	0.106
**523**	6-MHB-P15	10000	CDK	0.353369	0.075
**524**	7-MHB-A21	10000	BET	0.578976	0
**525**	7-MHB-A22	30000	BET	0.576724	0
**526**	7-MHB-B21	1000	BET	0.53158	0
**527**	7-MHB-B22	3000	BET	0.560326	0
**528**	7-MHB-C21	100	BET	0.165209	0.347
**529**	7-MHB-C22	300	BET	0.198988	0.287
**530**	7-MHB-D21	10	BET	0.404522	0.027
**531**	7-MHB-D22	30	BET	0.292569	0.253
**532**	7-MHB-E21	1	BET	0.34989	0.099
**533**	7-MHB-E22	3	BET	0.248062	0.558
**534**	7-MHB-G10	10000	BET	0.579244	0
**535**	7-MHB-G15	10000	BET	0.620389	0
**536**	7-MHB-H10	1000	BET	0.632766	0
**537**	7-MHB-H15	1000	BET	0.600825	0
**538**	7-MHB-I10-	100	BET	0.604685	0

**539**	7-MHB-I15-	100	BET	0.274002	0.059
**540**	7-MHB-J10	10	BET	0.124846	0.418
**541**	7-MHB-J15	10	BET	0.413343	0
**542**	7-MHB-K10	1	BET	0.083006	0.787
**543**	7-MHB-K13	1	BET	0.583141	0
**544**	7-MHB-K15	1	BET	0.582485	0
**545**	7-MHB-L12	1	BET	0.443607	0.002
**546**	7-MHB-L13	10	BET	0.693941	0
**547**	7-MHB-L20	1	BET	0.475027	0.001
**548**	7-MHB-L23	0.03	BET	0.594066	0
**549**	7-MHB-M1:	10	BET	0.237438	0.723
**550**	7-MHB-M1:	100	BET	0.603929	0
**551**	7-MHB-M2(	10	BET	0.267905	0.215
**552**	7-MHB-M2:	0.3	BET	0.201358	0.177
**553**	7-MHB-N12	100	BET	0.200181	0.212
**554**	7-MHB-N13	1000	BET	0.575129	0
**555**	7-MHB-N2(	100	BET	0.61443	0
**556**	7-MHB-N23	3	BET	0.592723	0
**557**	7-MHB-O12	1000	BET	0.618601	0
**558**	7-MHB-O2(	1000	BET	0.554528	0
**559**	7-MHB-O23	30	BET	0.670713	0
**560**	7-MHB-P12	10000	BET	0.515724	0.001
**561**	7-MHB-P13	10000	BET	0.511936	0
**562**	7-MHB-P2(	10000	BET	0.552689	0
**563**	7-MHB-P23	300	BET	0.622706	0
**564**	8-MHB-K22	1	BET	0.339776	0.236
**565**	8-MHB-L22	10	BET	0.406971	0.018
**566**	8-MHB-M2:	100	BET	0.399522	0.041
**567**	8-MHB-N22	1000	BET	0.512743	0
**568**	8-MHB-O22	10000	BET	0.458648	0.005
**569**	1-MHB-A3-'	10000	HDAC	0.207092	0.694
**570**	1-MHB-B3-'	1000	HDAC	0.272033	0.131
**571**	1-MHB-C3-'	100	HDAC	0.149028	0.071
**572**	1-MHB-D3-	10	HDAC	0.367515	0
**573**	1-MHB-E3-\	1	HDAC	0.352441	0.012

**574**	1-MHB-L12	0.1	HDAC	0.12881	0.188
**575**	1-MHB-M1:	1	HDAC	0.18562	0.068
**576**	1-MHB-N12	10	HDAC	0.217831	0.697
**577**	1-MHB-O12	100	HDAC	0.254841	0.205
**578**	1-MHB-P12	1000	HDAC	0.217331	0.785
**579**	3-MHB-A4-I	1000	HDAC	0.172684	0.87
**580**	3-MHB-B4-I	100	HDAC	0.206903	0.81
**581**	3-MHB-C4-I	10	HDAC	0.455584	0.001
**582**	3-MHB-D4-	1	HDAC	0.172128	0.064
**583**	3-MHB-E4-I	0.1	HDAC	0.36291	0.011
**584**	3-MHB-F7-(	1000	HDAC	0.219191	0.734
**585**	3-MHB-G7-	100	HDAC	0.213035	0.693
**586**	3-MHB-G12	1000000	HDAC	0.120782	0.22
**587**	3-MHB-H7-	10	HDAC	0.313863	0.001
**588**	3-MHB-H12	100000	HDAC	0.351021	0.02
**589**	3-MHB-I7-C	1	HDAC	0.105303	0.504
**590**	3-MHB-I12-	10000	HDAC	0.191862	0.396
**591**	3-MHB-J7-(	0.1	HDAC	0.224143	0.114
**592**	3-MHB-J12	1000	HDAC	0.115341	0.385
**593**	3-MHB-K3-I	1	HDAC	0.266883	0.493
**594**	3-MHB-K12	100	HDAC	0.21384	0.018
**595**	3-MHB-L3-I	10	HDAC	0.25762	0.327
**596**	3-MHB-M3-	100	HDAC	0.15783	0.873
**597**	3-MHB-N3-	1000	HDAC	0.211366	0.658
**598**	3-MHB-O3-	10000	HDAC	0.211019	0.782
**599**	7-MHB-A5-I	10000	HDAC	0.238637	0.708
**600**	7-MHB-A7-	10000	HDAC	0.246608	0.688
**601**	7-MHB-A9-	1000	HDAC	0.265695	0.329
**602**	7-MHB-A12	10000	HDAC	0.317122	0.064
**603**	7-MHB-B5-I	1000	HDAC	0.338549	0.009
**604**	7-MHB-B12	1000	HDAC	0.409015	0
**605**	7-MHB-C5-I	100	HDAC	0.164333	0.769
**606**	7-MHB-C7-	100	HDAC	0.335079	0.039
**607**	7-MHB-C9-	100	HDAC	0.39144	0
**608**	7-MHB-D7-	10	HDAC	0.357606	0.003

**609**	7-MHB-D9-	10	HDAC	0.220771	0.002
**610**	7-MHB-D12	100	HDAC	0.291565	0.02
**611**	7-MHB-E5-I	10	HDAC	0.280606	0.008
**612**	7-MHB-E7-(	1	HDAC	0.204428	0.005
**613**	7-MHB-E9-(	1	HDAC	0.450551	0
**614**	7-MHB-E12	10	HDAC	0.140785	0.077
**615**	7-MHB-F5-I	1	HDAC	0.389584	0.012
**616**	7-MHB-F7-I	10000	HDAC	0.322135	0.052
**617**	7-MHB-F9-(	0.1	HDAC	0.40811	0
**618**	7-MHB-F12	1	HDAC	0.366707	0
**619**	7-MHB-F19	10000	HDAC	0.327208	0.014
**620**	7-MHB-G7-	1000	HDAC	0.367215	0
**621**	7-MHB-G19	1000	HDAC	0.143508	0.093
**622**	7-MHB-H7-	100	HDAC	0.447385	0
**623**	7-MHB-I7-F	10	HDAC	0.304081	0.109
**624**	7-MHB-I19-	100	HDAC	0.230579	0.002
**625**	7-MHB-J7-F	1	HDAC	0.126262	0.135
**626**	7-MHB-J19	10	HDAC	0.341231	0.007
**627**	7-MHB-K4-I	1	HDAC	0.374822	0.004
**628**	7-MHB-K11	1	HDAC	0.3785	0.001
**629**	7-MHB-K18	1	HDAC	0.37593	0.001
**630**	7-MHB-K19	1	HDAC	0.124472	0.222
**631**	7-MHB-L2-T	0.1	HDAC	0.311294	0.05
**632**	7-MHB-L4-I	10	HDAC	0.275584	0.191
**633**	7-MHB-L5-I	1	HDAC	0.33279	0.001
**634**	7-MHB-L8-A	1	HDAC	0.396335	0
**635**	7-MHB-L10	1	HDAC	0.295183	0.002
**636**	7-MHB-L11	10	HDAC	0.398606	0
**637**	7-MHB-L14	1	HDAC	0.240564	0.061
**638**	7-MHB-L16	1	HDAC	0.152718	0.085
**639**	7-MHB-L18	10	HDAC	0.144108	0.097
**640**	7-MHB-M2-	1	HDAC	0.278705	0.15
**641**	7-MHB-M5-	10	HDAC	0.393028	0.002
**642**	7-MHB-M8-	10	HDAC	0.419732	0
**643**	7-MHB-M1(	10	HDAC	0.554934	0

**644**	7-MHB-M1[illegible]	100	HDAC	0.416792	0
**645**	7-MHB-M1[illegible]	10	HDAC	0.267568	0.001
**646**	7-MHB-M1[illegible]	10	HDAC	0.341428	0.006
**647**	7-MHB-M1[illegible]	100	HDAC	0.343985	0.01
**648**	7-MHB-N2-	10	HDAC	0.264935	0.144
**649**	7-MHB-N4-	100	HDAC	0.414642	0.004
**650**	7-MHB-N5-	100	HDAC	0.435661	0
**651**	7-MHB-N8-	100	HDAC	0.324795	0.013
**652**	7-MHB-N1[illegible]	100	HDAC	0.389912	0
**653**	7-MHB-N1[illegible]	100	HDAC	0.387256	0
**654**	7-MHB-N1[illegible]	100	HDAC	0.221001	0.116
**655**	7-MHB-N1[illegible]	1000	HDAC	0.146619	0.082
**656**	7-MHB-O2-	100	HDAC	0.307056	0.136
**657**	7-MHB-O4-	1000	HDAC	0.324713	0.014
**658**	7-MHB-O5-	1000	HDAC	0.345083	0.015
**659**	7-MHB-O8-	1000	HDAC	0.324352	0.038
**660**	7-MHB-O1[illegible]	1000	HDAC	0.437183	0
**661**	7-MHB-O11	1000	HDAC	0.348975	0.008
**662**	7-MHB-O1[illegible]	1000	HDAC	0.312393	0.002
**663**	7-MHB-O1[illegible]	1000	HDAC	0.141836	0.668
**664**	7-MHB-P2-[illegible]	1000	HDAC	0.341159	0.011
**665**	7-MHB-P4-[illegible]	10000	HDAC	0.304456	0.061
**666**	7-MHB-P5-[illegible]	10000	HDAC	0.27927	0.312
**667**	7-MHB-P8-[illegible]	10000	HDAC	0.294676	0.205
**668**	7-MHB-P1[illegible]	10000	HDAC	0.321176	0.029
**669**	7-MHB-P11	10000	HDAC	0.320445	0.075
**670**	7-MHB-P14	10000	HDAC	0.313982	0.063
**671**	7-MHB-P1[illegible]	10000	HDAC	0.261478	0.418
**672**	7-MHB-P1[illegible]	10000	HDAC	0.419898	0
**0**	2-O3B-A16[illegible]	10000	EGFR	0.524783	0
**1**	2-O3B-A19[illegible]	10000	EGFR	0.521988	0
**2**	2-O3B-B19[illegible]	1000	EGFR	0.524117	0
**3**	2-O3B-C16[illegible]	1000	EGFR	0.532434	0
**4**	2-O3B-C19[illegible]	100	EGFR	0.587887	0
**5**	2-O3B-D16	100	EGFR	0.555661	0

**6**	2-O3B-D19	10	EGFR	0.400062	0
**7**	2-O3B-E16-	10	EGFR	0.50034	0
**8**	2-O3B-E19-	1	EGFR	0.121884	0.951
**9**	2-O3B-F16-	1	EGFR	0.393839	0.002
**10**	2-O3B-K11-	0.1	EGFR	0.575023	0
**11**	2-O3B-L11-	1	EGFR	0.558027	0
**12**	2-O3B-L16-	0.25	EGFR	0.242743	0.377
**13**	2-O3B-L19-	0.1	EGFR	0.464281	0
**14**	2-O3B-M11	10	EGFR	0.601051	0
**15**	2-O3B-M16	2.5	EGFR	0.299601	0.413
**16**	2-O3B-M19	1	EGFR	0.505713	0
**17**	2-O3B-N16	25	EGFR	0.525573	0
**18**	2-O3B-N19	10	EGFR	0.587783	0
**19**	2-O3B-O11	100	EGFR	0.521455	0
**20**	2-O3B-O16	250	EGFR	0.526829	0
**21**	2-O3B-O19	100	EGFR	0.573695	0
**22**	2-O3B-P11-	1000	EGFR	0.516758	0
**23**	2-O3B-P16-	2500	EGFR	0.502085	0
**24**	2-O3B-P19-	1000	EGFR	0.549804	0
**25**	3-O3B-F21-	10000	EGFR	0.476702	0
**26**	3-O3B-G20	1000	EGFR	0.495153	0
**27**	3-O3B-G21	1000	EGFR	0.50066	0
**28**	3-O3B-H20	100	EGFR	0.54189	0
**29**	3-O3B-H21	100	EGFR	0.283942	0.218
**30**	3-O3B-I20-	10	EGFR	0.587074	0
**31**	3-O3B-I21-	10	EGFR	0.597543	0
**32**	3-O3B-J20-	1	EGFR	0.136489	0.808
**33**	3-O3B-J21-	1	EGFR	0.053481	0.975
**34**	3-O3B-K4-(	1	EGFR	0.069663	1
**35**	3-O3B-K18-	0.1	EGFR	0.498127	0
**36**	3-O3B-K20-	0.1	EGFR	0.475341	0
**37**	3-O3B-L4-C	10	EGFR	0.50087	0
**38**	3-O3B-L18-	1	EGFR	0.052452	0.999
**39**	3-O3B-M18	10	EGFR	0.525348	0
**40**	3-O3B-N4-(	100	EGFR	0.424682	0

**41**	3-O3B-N18	100	EGFR	0.522237	0
**42**	3-O3B-O4-(	1000	EGFR	0.507027	0
**43**	3-O3B-P4-(	10000	EGFR	0.4748	0
**44**	3-O3B-P18	1000	EGFR	0.507907	0
**45**	4-O3B-F13-	1000	EGFR	0.498644	0
**46**	4-O3B-G13	100	EGFR	0.560017	0
**47**	4-O3B-G16	10000	EGFR	0.492023	0
**48**	4-O3B-H13	10	EGFR	0.554339	0
**49**	4-O3B-H16	1000	EGFR	0.561085	0
**50**	4-O3B-I13-!	1	EGFR	0.069907	0.985
**51**	4-O3B-I16-'	100	EGFR	0.602354	0
**52**	4-O3B-J13-	0.1	EGFR	0.503862	0
**53**	4-O3B-J16-	10	EGFR	0.025776	1
**54**	4-O3B-K7-I	1	EGFR	0.478671	0
**55**	4-O3B-K13	0.1	EGFR	0.524461	0
**56**	4-O3B-K16	1	EGFR	0.561189	0
**57**	4-O3B-L7-I	10	EGFR	0.527318	0
**58**	4-O3B-L13-	1	EGFR	0.552128	0
**59**	4-O3B-M7-I	100	EGFR	0.513666	0
**60**	4-O3B-M13	10	EGFR	0.599308	0
**61**	4-O3B-N13	100	EGFR	0.519308	0
**62**	4-O3B-O7-I	1000	EGFR	0.513815	0
**63**	4-O3B-P7-I	10000	EGFR	0.565476	0
**64**	4-O3B-P13	1000	EGFR	0.499777	0
**65**	5-O3B-F4-P	1000	EGFR	0.495555	0
**66**	5-O3B-F7-A	1000	EGFR	0.55417	0
**67**	5-O3B-G4-F	100	EGFR	0.501017	0
**68**	5-O3B-G7-/	100	EGFR	0.58107	0
**69**	5-O3B-H4-F	10	EGFR	0.582119	0
**70**	5-O3B-H7-/	10	EGFR	0.056386	0.99
**71**	5-O3B-I4-P	1	EGFR	0.519776	0
**72**	5-O3B-I7-A	1	EGFR	0.486413	0
**73**	5-O3B-J4-P	0.1	EGFR	0.434702	0.018
**74**	5-O3B-J7-A	0.1	EGFR	0.431983	0
**75**	5-O3B-K7-(	0.1	EGFR	0.455473	0

**76**	5-O3B-L7-C	1	EGFR	0.296165	0.154
**77**	5-O3B-M7-(	10	EGFR	0.554143	0
**78**	5-O3B-O7-(	100	EGFR	0.544515	0
**79**	5-O3B-P7-(	1000	EGFR	0.51692	0
**80**	2-O3B-A15-	2500	VEGFR	0.547401	0
**81**	2-O3B-A17-	10000	VEGFR	0.272893	0.294
**82**	2-O3B-A20-	10000	VEGFR	0.262831	0.344
**83**	2-O3B-B15-	250	VEGFR	0.540992	0
**84**	2-O3B-B17-	1000	VEGFR	0.283788	0.217
**85**	2-O3B-B20-	1000	VEGFR	0.558839	0
**86**	2-O3B-C15-	25	VEGFR	0.297297	0.088
**87**	2-O3B-C17-	100	VEGFR	0.467877	0
**88**	2-O3B-D15	2.5	VEGFR	0.232097	0.053
**89**	2-O3B-D17	10	VEGFR	0.506689	0
**90**	2-O3B-D20	100	VEGFR	0.356717	0.009
**91**	2-O3B-E17-	1	VEGFR	0.391575	0.001
**92**	2-O3B-E20-	10	VEGFR	0.515181	0
**93**	2-O3B-F13-	10000	VEGFR	0.281716	0.199
**94**	2-O3B-F15-	0.25	VEGFR	0.586361	0
**95**	2-O3B-F19-	10000	VEGFR	0.257427	0.383
**96**	2-O3B-F20-	1	VEGFR	0.466468	0
**97**	2-O3B-F21-	10000	VEGFR	0.255996	0.321
**98**	2-O3B-G10	10000	VEGFR	0.33919	0.01
**99**	2-O3B-G13	1000	VEGFR	0.515951	0
**100**	2-O3B-G19	1000	VEGFR	0.509884	0
**101**	2-O3B-G21	1000	VEGFR	0.380051	0.001
**102**	2-O3B-H10	1000	VEGFR	0.440799	0
**103**	2-O3B-H13	100	VEGFR	0.455806	0
**104**	2-O3B-H21	100	VEGFR	0.261911	0.218
**105**	2-O3B-I10-,	100	VEGFR	0.326497	0.028
**106**	2-O3B-I13-,	10	VEGFR	0.588331	0
**107**	2-O3B-I19-I	100	VEGFR	0.372054	0.003
**108**	2-O3B-I21-'	10	VEGFR	0.343313	0.001
**109**	2-O3B-J10-	10	VEGFR	0.366632	0.002
**110**	2-O3B-J13-	1	VEGFR	0.286225	0.088

**111**	2-O3B-J19-	10	VEGFR	0.252503	0.164
**112**	2-O3B-J21-	1	VEGFR	0.401035	0
**113**	2-O3B-K10-	1	VEGFR	0.464395	0
**114**	2-O3B-K13-	0.1	VEGFR	0.538079	0
**115**	2-O3B-K17-	1	VEGFR	0.191637	0.582
**116**	2-O3B-K19-	1	VEGFR	0.340898	0.01
**117**	2-O3B-L12-	0.1	VEGFR	0.252349	0.226
**118**	2-O3B-L13-	1	VEGFR	0.417486	0
**119**	2-O3B-L21-	0.1	VEGFR	0.508649	0
**120**	2-O3B-M12	1	VEGFR	0.481408	0
**121**	2-O3B-M13	10	VEGFR	0.246923	0.017
**122**	2-O3B-M17	10	VEGFR	0.508116	0
**123**	2-O3B-M21	1	VEGFR	0.299506	0.119
**124**	2-O3B-N12	10	VEGFR	0.052333	0.964
**125**	2-O3B-N13	100	VEGFR	0.287682	0.133
**126**	2-O3B-N17	100	VEGFR	0.265971	0.304
**127**	2-O3B-N21	10	VEGFR	0.041189	0.939
**128**	2-O3B-O12	100	VEGFR	0.08095	0.846
**129**	2-O3B-O17	1000	VEGFR	0.384866	0
**130**	2-O3B-O21	100	VEGFR	0.372861	0.001
**131**	2-O3B-P12-	1000	VEGFR	0.397624	0.001
**132**	2-O3B-P13-	1000	VEGFR	0.241657	0.502
**133**	2-O3B-P17-	10000	VEGFR	0.287175	0.212
**134**	2-O3B-P21-	1000	VEGFR	0.406577	0
**135**	3-O3B-A3-(	1000	VEGFR	0.356674	0.012
**136**	3-O3B-A6-F	1000	VEGFR	0.218882	0.73
**137**	3-O3B-A18-	1000	VEGFR	0.486857	0
**138**	3-O3B-B3-(	100	VEGFR	0.399415	0.003
**139**	3-O3B-B6-F	100	VEGFR	0.421268	0
**140**	3-O3B-B18-	100	VEGFR	0.347449	0.05
**141**	3-O3B-C3-(	10	VEGFR	0.256711	0.397
**142**	3-O3B-C6-F	10	VEGFR	0.502897	0
**143**	3-O3B-C18-	10	VEGFR	0.236668	0.194
**144**	3-O3B-D3-(	1	VEGFR	0.11165	0.454
**145**	3-O3B-D6-F	1	VEGFR	0.082311	0.742

**146**	3-O3B-D18	1	VEGFR	0.126105	0.265
**147**	3-O3B-E3-C	0.1	VEGFR	0.148967	0.444
**148**	3-O3B-E6-F	0.1	VEGFR	0.117486	0.644
**149**	3-O3B-E18-	0.1	VEGFR	0.174604	0.78
**150**	3-O3B-F18-	1000	VEGFR	0.407072	0.008
**151**	3-O3B-G18	100	VEGFR	0.164174	0.214
**152**	3-O3B-H18	10	VEGFR	0.16459	0.348
**153**	3-O3B-I18-I	1	VEGFR	0.176302	0.59
**154**	3-O3B-J18-	0.1	VEGFR	0.214275	0.672
**155**	4-O3B-A12-	10000	VEGFR	0.238434	0.566
**156**	4-O3B-A15-	2500	VEGFR	0.468521	0
**157**	4-O3B-A20-	10000	VEGFR	0.249989	0.407
**158**	4-O3B-B12-	1000	VEGFR	0.228208	0.601
**159**	4-O3B-B15-	250	VEGFR	0.120363	0.417
**160**	4-O3B-B20-	1000	VEGFR	0.096603	0.937
**161**	4-O3B-C15-	25	VEGFR	0.230488	0.304
**162**	4-O3B-D12	100	VEGFR	0.298875	0.09
**163**	4-O3B-D15	2.5	VEGFR	0.200354	0.74
**164**	4-O3B-D20	100	VEGFR	0.341201	0.014
**165**	4-O3B-E12-	10	VEGFR	0.387926	0.005
**166**	4-O3B-E20-	10	VEGFR	0.464556	0
**167**	4-O3B-F12-	1	VEGFR	0.121799	0.421
**168**	4-O3B-F15-	0.25	VEGFR	0.256528	0.19
**169**	4-O3B-F20-	1	VEGFR	0.065192	0.721
**170**	4-O3B-L16-	1	VEGFR	0.459227	0
**171**	4-O3B-M16	10	VEGFR	0.236577	0.585
**172**	4-O3B-N16	100	VEGFR	0.138036	0.951
**173**	4-O3B-O16	1000	VEGFR	0.488873	0
**174**	4-O3B-P16-	10000	VEGFR	0.312909	0.059
**175**	2-O3B-L10-	1	PI3K	0.049999	0.987
**176**	2-O3B-M10	10	PI3K	0.363985	0.002
**177**	2-O3B-N10	100	PI3K	0.371954	0.001
**178**	2-O3B-O10	1000	PI3K	0.389465	0
**179**	2-O3B-P10-	10000	PI3K	0.389688	0
**180**	3-O3B-A16-	2500	PI3K	0.037437	0.979

**181**	3-O3B-C16·	250	PI3K	0.279394	0.198
**182**	3-O3B-D16	25	PI3K	0.023621	0.987
**183**	3-O3B-E16·	2.5	PI3K	0.04614	0.957
**184**	3-O3B-F16·	0.25	PI3K	0.342778	0.021
**185**	3-O3B-F17·	100000	PI3K	0.370105	0.003
**186**	3-O3B-F19·	500	PI3K	0.427593	0
**187**	3-O3B-G17	10000	PI3K	0.025538	0.986
**188**	3-O3B-G19	50	PI3K	0.396413	0.001
**189**	3-O3B-H17	1000	PI3K	0.131573	0.815
**190**	3-O3B-I17-I	100	PI3K	0.285035	0.038
**191**	3-O3B-I19-I	5	PI3K	0.395971	0
**192**	3-O3B-J17-	10	PI3K	0.318826	0.038
**193**	3-O3B-J19-	0.5	PI3K	0.171287	0.439
**194**	3-O3B-K19·	0.05	PI3K	0.07656	0.797
**195**	3-O3B-L8-P	1	PI3K	0.142129	0.467
**196**	3-O3B-L21-	0.1	PI3K	0.065925	0.917
**197**	3-O3B-M8-I	10	PI3K	0.432899	0
**198**	3-O3B-M21	1	PI3K	0.316267	0.005
**199**	3-O3B-N8-F	100	PI3K	0.377707	0
**200**	3-O3B-N21	10	PI3K	0.078006	0.976
**201**	3-O3B-O8-F	1000	PI3K	0.366219	0.001
**202**	3-O3B-O21	100	PI3K	0.360055	0
**203**	3-O3B-P8-F	10000	PI3K	0.362845	0.005
**204**	3-O3B-P21·	1000	PI3K	0.367826	0.002
**205**	4-O3B-A19·	2500	PI3K	0.401026	0
**206**	4-O3B-B19·	250	PI3K	0.411972	0
**207**	4-O3B-C19·	25	PI3K	0.411659	0
**208**	4-O3B-D19	2.5	PI3K	0.367565	0
**209**	4-O3B-E19·	0.25	PI3K	0.432775	0
**210**	4-O3B-F14·	1000	PI3K	0.375335	0
**211**	4-O3B-G2-1	2500	PI3K	0.327606	0.115
**212**	4-O3B-G5-S	10000	PI3K	0.506661	0
**213**	4-O3B-G14	100	PI3K	0.391858	0
**214**	4-O3B-G20	10000	PI3K	0.375848	0.003
**215**	4-O3B-H2-1	250	PI3K	0.103402	0.714

**216**	4-O3B-H5-S	1000	PI3K	0.434563	0
**217**	4-O3B-H14	10	PI3K	0.404437	0
**218**	4-O3B-H20	1000	PI3K	0.395015	0
**219**	4-O3B-I2-T(	25	PI3K	0.298705	0.467
**220**	4-O3B-I5-S(	100	PI3K	0.473882	0
**221**	4-O3B-I14-I	1	PI3K	0.410521	0
**222**	4-O3B-I20-I	100	PI3K	0.040158	0.963
**223**	4-O3B-J2-T(	2.5	PI3K	0.018973	1
**224**	4-O3B-J5-S(	10	PI3K	0.281293	0.038
**225**	4-O3B-J20-	10	PI3K	0.054304	0.882
**226**	4-O3B-K2-T	0.25	PI3K	0.233162	0.843
**227**	4-O3B-K4-D	0.1	PI3K	0.454531	0
**228**	4-O3B-K5-S	1	PI3K	0.439071	0
**229**	4-O3B-K14-	0.1	PI3K	0.204338	0.643
**230**	4-O3B-K20-	1	PI3K	0.418641	0
**231**	4-O3B-L4-D	1	PI3K	0.433469	0
**232**	4-O3B-L14-	0.1	PI3K	0.429192	0
**233**	4-O3B-L15-	1	PI3K	0.028306	0.986
**234**	4-O3B-L21-	0.1	PI3K	0.3206	0.039
**235**	4-O3B-M14	1	PI3K	0.277306	0.034
**236**	4-O3B-M15	10	PI3K	0.407343	0
**237**	4-O3B-M21	1	PI3K	0.138769	0.426
**238**	4-O3B-N4-D	10	PI3K	0.399504	0
**239**	4-O3B-N14	10	PI3K	0.437874	0
**240**	4-O3B-N15	100	PI3K	0.37846	0
**241**	4-O3B-N21	10	PI3K	0.388578	0
**242**	4-O3B-O4-D	100	PI3K	0.390497	0
**243**	4-O3B-O14	100	PI3K	0.378981	0
**244**	4-O3B-O15	1000	PI3K	0.423534	0
**245**	4-O3B-O21	100	PI3K	0.397225	0
**246**	4-O3B-P4-D	1000	PI3K	0.401433	0
**247**	4-O3B-P14-	1000	PI3K	0.385364	0
**248**	4-O3B-P15-	10000	PI3K	0.402292	0
**249**	4-O3B-P21-	1000	PI3K	0.391771	0.001
**250**	5-O3B-A6-L	2500	PI3K	0.376303	0.001

**251**	5-O3B-A7-A	1000	PI3K	0.392048	0
**252**	5-O3B-A16-	2500	PI3K	0.440324	0
**253**	5-O3B-A17-	10000	PI3K	0.127448	0.742
**254**	5-O3B-B6-L	250	PI3K	0.38904	0
**255**	5-O3B-B7-A	100	PI3K	0.053329	0.907
**256**	5-O3B-B17-	1000	PI3K	0.42956	0
**257**	5-O3B-C6-L	25	PI3K	0.313986	0.018
**258**	5-O3B-C7-A	10	PI3K	0.456396	0
**259**	5-O3B-C16-	250	PI3K	0.443642	0
**260**	5-O3B-C17-	100	PI3K	0.406605	0
**261**	5-O3B-D6-L	2.5	PI3K	0.077855	0.936
**262**	5-O3B-D7-A	1	PI3K	0.451642	0
**263**	5-O3B-D16	25	PI3K	0.401296	0
**264**	5-O3B-D17	10	PI3K	0.430517	0
**265**	5-O3B-E6-L	0.25	PI3K	0.05481	0.907
**266**	5-O3B-E7-A	0.1	PI3K	0.171395	0.226
**267**	5-O3B-E16-	2.5	PI3K	0.424775	0
**268**	5-O3B-E17-	1	PI3K	0.401367	0
**269**	5-O3B-F11-	10000	PI3K	0.443322	0
**270**	5-O3B-F16-	0.25	PI3K	0.416191	0
**271**	5-O3B-G9-S	10000	PI3K	0.46302	0
**272**	5-O3B-G11	1000	PI3K	0.439488	0
**273**	5-O3B-H9-S	1000	PI3K	0.422898	0
**274**	5-O3B-H11	100	PI3K	0.393774	0
**275**	5-O3B-I9-Se	100	PI3K	0.080802	0.956
**276**	5-O3B-I11-	10	PI3K	0.246323	0.184
**277**	5-O3B-J9-S	10	PI3K	0.406757	0
**278**	5-O3B-J11-	1	PI3K	0.139414	0.594
**279**	5-O3B-K9-S	1	PI3K	0.37367	0.001
**280**	5-O3B-L14-	0.1	PI3K	0.394379	0
**281**	5-O3B-L20-	1	PI3K	0.445109	0.001
**282**	5-O3B-L23-	0.1	PI3K	0.139758	0.945
**283**	5-O3B-M14	1	PI3K	0.404181	0
**284**	5-O3B-M20	10	PI3K	0.429144	0
**285**	5-O3B-M23	1	PI3K	0.419804	0

**286**	5-O3B-N14	10	PI3K	0.418096	0
**287**	5-O3B-N20	100	PI3K	0.416489	0
**288**	5-O3B-N23	10	PI3K	0.447982	0
**289**	5-O3B-O14	100	PI3K	0.36978	0
**290**	5-O3B-O20	1000	PI3K	0.384202	0.002
**291**	5-O3B-O23	100	PI3K	0.3826	0
**292**	5-O3B-P14-	1000	PI3K	0.414866	0
**293**	5-O3B-P20-	10000	PI3K	0.383082	0
**294**	5-O3B-P23-	1000	PI3K	0.379554	0
**295**	6-O3B-A8-T	10000	PI3K	0.380405	0.001
**296**	6-O3B-B8-T	1000	PI3K	0.379729	0
**297**	6-O3B-C8-T	100	PI3K	0.388018	0
**298**	6-O3B-D8-T	10	PI3K	0.275659	0.057
**299**	6-O3B-E8-T	1	PI3K	0.027594	0.995
**300**	6-O3B-L6-G	1	PI3K	0.083431	0.977
**301**	6-O3B-M6-	10	PI3K	0.172102	0.806
**302**	6-O3B-N6-(	100	PI3K	0.321115	0.01
**303**	6-O3B-O6-(	1000	PI3K	0.376469	0.001
**304**	6-O3B-P6-G	10000	PI3K	0.367629	0.002
**305**	1-O3B-F11-	10000	Topoisome	0.39872	0.013
**306**	1-O3B-G11	1000	Topoisome	0.64557	0
**307**	1-O3B-G20	1000	Topoisome	0.405946	0.005
**308**	1-O3B-H11	100	Topoisome	0.618641	0
**309**	1-O3B-H20	100	Topoisome	0.549212	0
**310**	1-O3B-I11-,	10	Topoisome	0.337622	0.07
**311**	1-O3B-I20-I	10	Topoisome	0.351156	0.043
**312**	1-O3B-J11-	1	Topoisome	0.151994	0.736
**313**	1-O3B-J20-	1	Topoisome	0.310888	0.146
**314**	1-O3B-K11-	1	Topoisome	0.593678	0
**315**	1-O3B-K20-	0.1	Topoisome	0.250626	0.508
**316**	1-O3B-L11-	10	Topoisome	0.571308	0
**317**	1-O3B-L14-	1	Topoisome	0.243917	0.273
**318**	1-O3B-M11	100	Topoisome	0.452614	0
**319**	1-O3B-M14	10	Topoisome	0.478013	0
**320**	1-O3B-N14	100	Topoisome	0.619178	0

**321**	1-O3B-O11	1000	Topoisome	0.411122	0.01
**322**	1-O3B-O14	1000	Topoisome	0.480457	0.001
**323**	1-O3B-P11-	10000	Topoisome	0.414418	0.005
**324**	1-O3B-P14-	10000	Topoisome	0.416032	0.006
**325**	3-O3B-A11-	10000	Topoisome	0.515112	0
**326**	3-O3B-B11-	1000	Topoisome	0.649692	0
**327**	3-O3B-C11-	100	Topoisome	0.533522	0
**328**	3-O3B-D11	10	Topoisome	0.425526	0.006
**329**	3-O3B-E11-	1	Topoisome	0.427073	0
**330**	3-O3B-G9-D	1000	Topoisome	0.406652	0.011
**331**	3-O3B-G10	10000	Topoisome	0.517711	0
**332**	3-O3B-H9-D	100	Topoisome	0.558739	0
**333**	3-O3B-H10	1000	Topoisome	0.154237	0.573
**334**	3-O3B-I9-D	10	Topoisome	0.340136	0.324
**335**	3-O3B-I10-	100	Topoisome	0.190236	0.541
**336**	3-O3B-J9-D	1	Topoisome	0.198354	0.557
**337**	3-O3B-J10-	10	Topoisome	0.17239	0.552
**338**	3-O3B-K7-	0.1	Topoisome	0.342522	0.036
**339**	3-O3B-K9-D	0.1	Topoisome	0.406933	0.003
**340**	3-O3B-K10-	1	Topoisome	0.064562	0.855
**341**	3-O3B-L6-D	0.1	Topoisome	0.153664	0.468
**342**	3-O3B-L7-	1	Topoisome	0.540634	0.001
**343**	3-O3B-L9-V	0.5	Topoisome	0.416631	0.005
**344**	3-O3B-L10-	0.1	Topoisome	0.070648	0.859
**345**	3-O3B-L16-	1	Topoisome	0.382655	0.01
**346**	3-O3B-M6-	1	Topoisome	0.123368	0.735
**347**	3-O3B-M7-	10	Topoisome	0.583703	0
**348**	3-O3B-M9-	5	Topoisome	0.428017	0.005
**349**	3-O3B-M10	1	Topoisome	0.296096	0.032
**350**	3-O3B-M16	10	Topoisome	0.44995	0
**351**	3-O3B-N6-D	10	Topoisome	0.490457	0
**352**	3-O3B-N9-	50	Topoisome	0.627174	0
**353**	3-O3B-N10	10	Topoisome	0.55565	0
**354**	3-O3B-N16	100	Topoisome	0.279741	0.135
**355**	3-O3B-O6-D	100	Topoisome	0.595101	0

**356**	3-O3B-O7-I	100	Topoisome	0.652413	0
**357**	3-O3B-O9-\	500	Topoisome	0.613061	0
**358**	3-O3B-O10	100	Topoisome	0.669857	0
**359**	3-O3B-O16	1000	Topoisome	0.416104	0.004
**360**	3-O3B-P6-D	1000	Topoisome	0.425983	0.005
**361**	3-O3B-P7-I	1000	Topoisome	0.398776	0.019
**362**	3-O3B-P9-V	5000	Topoisome	0.411379	0.005
**363**	3-O3B-P10-	1000	Topoisome	0.407253	0.012
**364**	3-O3B-P16-	10000	Topoisome	0.220655	0.645
**365**	1-O3B-A10-	10000	Mitotic	0.600279	0
**366**	1-O3B-A13-	1000	Mitotic	0.613608	0
**367**	1-O3B-A18-	1000	Mitotic	0.614741	0
**368**	1-O3B-B10-	1000	Mitotic	0.60555	0
**369**	1-O3B-B13-	100	Mitotic	0.048955	1
**370**	1-O3B-B18-	100	Mitotic	0.582853	0
**371**	1-O3B-C10-	100	Mitotic	0.59994	0
**372**	1-O3B-C13-	10	Mitotic	0.57275	0
**373**	1-O3B-C18-	10	Mitotic	0.609641	0
**374**	1-O3B-D10	10	Mitotic	0.584391	0
**375**	1-O3B-D13	1	Mitotic	0.287992	0.209
**376**	1-O3B-D18	1	Mitotic	0.607765	0
**377**	1-O3B-E10-	1	Mitotic	0.558115	0
**378**	1-O3B-E13-	0.1	Mitotic	0.455144	0.02
**379**	1-O3B-E18-	0.1	Mitotic	0.528803	0
**380**	1-O3B-F13-	1000	Mitotic	0.608426	0
**381**	1-O3B-G13	100	Mitotic	0.60435	0
**382**	1-O3B-G15	1000	Mitotic	0.610963	0
**383**	1-O3B-H13	10	Mitotic	0.219984	0.832
**384**	1-O3B-H15	100	Mitotic	0.605754	0
**385**	1-O3B-I13-'	1	Mitotic	0.548613	0
**386**	1-O3B-I15-I	10	Mitotic	0.595217	0
**387**	1-O3B-J13-	0.1	Mitotic	0.286945	0.131
**388**	1-O3B-J15-	1	Mitotic	0.582626	0
**389**	1-O3B-K7-V	0.1	Mitotic	0.072052	1
**390**	1-O3B-K15-	0.1	Mitotic	0.627707	0

**391**	1-O3B-L7-V	1	Mitotic	0.198708	0.959
**392**	1-O3B-L20-	0.1	Mitotic	0.370325	0.047
**393**	1-O3B-M7-'	10	Mitotic	0.468261	0.005
**394**	1-O3B-M2C	1	Mitotic	0.659965	0
**395**	1-O3B-N20	10	Mitotic	0.511095	0.002
**396**	1-O3B-O7-\	100	Mitotic	0.590794	0
**397**	1-O3B-O20	100	Mitotic	0.582085	0
**398**	1-O3B-P7-V	1000	Mitotic	0.59044	0
**399**	1-O3B-P20-	1000	Mitotic	0.580364	0
**400**	3-O3B-A7-D	1000	Mitotic	0.546207	0
**401**	3-O3B-B7-D	100	Mitotic	0.552611	0
**402**	3-O3B-C7-D	10	Mitotic	0.565026	0
**403**	3-O3B-D7-D	1	Mitotic	0.560538	0
**404**	3-O3B-E7-D	0.1	Mitotic	0.045367	1
**405**	6-O3B-L19-	1	Mitotic	0.604208	0
**406**	6-O3B-M19	10	Mitotic	0.571528	0
**407**	6-O3B-N19	100	Mitotic	0.050387	1
**408**	6-O3B-O19	1000	Mitotic	0.569855	0
**409**	6-O3B-P19-	10000	Mitotic	0.566826	0
**410**	2-O3B-A12-	250	MEK1/2	0.645754	0
**411**	2-O3B-B12-	25	MEK1/2	0.661792	0
**412**	2-O3B-D12	2.5	MEK1/2	0.747561	0
**413**	2-O3B-E12-	0.25	MEK1/2	0.622342	0
**414**	2-O3B-F12-	2.5000000	MEK1/2	0.700587	0
**415**	2-O3B-F14-	1000	MEK1/2	0.649878	0
**416**	2-O3B-G14	100	MEK1/2	0.67182	0
**417**	2-O3B-H14	10	MEK1/2	0.715651	0
**418**	2-O3B-I14-	1	MEK1/2	0.69298	0
**419**	2-O3B-K14-	0.1	MEK1/2	0.609262	0
**420**	2-O3B-L20-	1	MEK1/2	0.173967	0.992
**421**	2-O3B-M2C	10	MEK1/2	0.678425	0
**422**	2-O3B-N20	100	MEK1/2	0.667647	0
**423**	2-O3B-O20	1000	MEK1/2	0.61494	0
**424**	2-O3B-P20-	10000	MEK1/2	0.681177	0
**425**	4-O3B-A10-	1000	MEK1/2	0.753356	0

**426**	4-O3B-A13-	1000	MEK1/2	0.698393	0
**427**	4-O3B-B10-	100	MEK1/2	0.774761	0
**428**	4-O3B-B13-	100	MEK1/2	0.72602	0
**429**	4-O3B-C10-	10	MEK1/2	0.826325	0
**430**	4-O3B-C13-	10	MEK1/2	0.689183	0
**431**	4-O3B-D10	1	MEK1/2	0.217997	0.694
**432**	4-O3B-D13	1	MEK1/2	0.787494	0
**433**	4-O3B-E10-	0.1	MEK1/2	0.102608	0.973
**434**	4-O3B-E13-	0.1	MEK1/2	0.475385	0.047
**435**	4-O3B-L19-	0.25	MEK1/2	0.670597	0
**436**	4-O3B-M19	2.5	MEK1/2	0.628022	0
**437**	4-O3B-N19	25	MEK1/2	0.562004	0
**438**	4-O3B-O19	250	MEK1/2	0.670021	0
**439**	4-O3B-P19-	2500	MEK1/2	0.762172	0
**440**	1-O3B-L2-C	1	PARP	0.461689	0.091
**441**	1-O3B-L6-R	1	PARP	0.442396	0.037
**442**	1-O3B-M2-	10	PARP	0.414166	0.097
**443**	1-O3B-M6-	10	PARP	0.351286	0.275
**444**	1-O3B-N2-(	100	PARP	0.740776	0
**445**	1-O3B-N6-F	100	PARP	0.775936	0
**446**	1-O3B-O2-(	1000	PARP	0.722412	0
**447**	1-O3B-O6-F	1000	PARP	0.702132	0
**448**	1-O3B-P2-C	10000	PARP	0.647745	0
**449**	1-O3B-P6-F	10000	PARP	0.650684	0
**450**	7-O3B-A3-T	1000	PARP	0.616754	0
**451**	7-O3B-B2-V	10000	PARP	0.646902	0
**452**	7-O3B-B3-T	100	PARP	0.640032	0
**453**	7-O3B-C2-V	1000	PARP	0.546201	0.019
**454**	7-O3B-C3-T	10	PARP	0.771819	0
**455**	7-O3B-D2-V	100	PARP	0.167623	0.676
**456**	7-O3B-D3-T	1	PARP	0.765853	0
**457**	7-O3B-E2-V	10	PARP	0.458202	0.006
**458**	7-O3B-E3-T	0.1	PARP	0.491516	0.017
**459**	7-O3B-F2-V	1	PARP	0.177372	0.495
**460**	7-O3B-G2-N	10000	PARP	0.73584	0

**461**	7-O3B-H2-N	1000	PARP	0.683916	0
**462**	7-O3B-I2-N	100	PARP	0.665224	0
**463**	7-O3B-J2-N	10	PARP	0.59735	0.001
**464**	7-O3B-K2-N	1	PARP	0.652029	0
**465**	3-O3B-A19-	1000	CDK	0.465538	0
**466**	3-O3B-B19-	100	CDK	0.441496	0.001
**467**	3-O3B-B23-	2500	CDK	0.397576	0.008
**468**	3-O3B-C19-	10	CDK	0.459813	0.001
**469**	3-O3B-C23-	250	CDK	0.410294	0.006
**470**	3-O3B-D19	1	CDK	0.074233	0.957
**471**	3-O3B-D23	25	CDK	0.4878	0
**472**	3-O3B-E19-	0.1	CDK	0.106241	0.759
**473**	3-O3B-E23-	2.5	CDK	0.352359	0.135
**474**	3-O3B-F23-	0.25	CDK	0.409067	0.032
**475**	3-O3B-K17-	1	CDK	0.111551	0.933
**476**	3-O3B-L19-	1	CDK	0.497683	0
**477**	3-O3B-M17	10	CDK	0.359408	0.044
**478**	3-O3B-M19	10	CDK	0.410054	0.001
**479**	3-O3B-N17	100	CDK	0.400342	0.003
**480**	3-O3B-N19	100	CDK	0.494791	0.002
**481**	3-O3B-O17	1000	CDK	0.439752	0.001
**482**	3-O3B-O19	1000	CDK	0.45227	0.003
**483**	3-O3B-P17-	10000	CDK	0.426054	0
**484**	3-O3B-P19-	10000	CDK	0.395728	0.01
**485**	4-O3B-A4-S	10000	CDK	0.430933	0
**486**	4-O3B-A8-N	10000	CDK	0.433919	0.001
**487**	4-O3B-B4-S	1000	CDK	0.445332	0.001
**488**	4-O3B-B8-N	1000	CDK	0.411158	0.007
**489**	4-O3B-C4-S	100	CDK	0.476629	0
**490**	4-O3B-C8-N	100	CDK	0.386063	0.012
**491**	4-O3B-D4-S	10	CDK	0.160532	0.957
**492**	4-O3B-D8-N	10	CDK	0.09875	0.932
**493**	4-O3B-E4-S	1	CDK	0.049421	1
**494**	4-O3B-E8-N	1	CDK	0.347237	0.048
**495**	4-O3B-F4-S	10000	CDK	0.442198	0.001

**496**	4-O3B-F22-	10000	CDK	0.433736	0.006
**497**	4-O3B-G4-S	1000	CDK	0.33716	0.093
**498**	4-O3B-G22	1000	CDK	0.440872	0.002
**499**	4-O3B-H4-S	100	CDK	0.296303	0.253
**500**	4-O3B-H22	100	CDK	0.433036	0.001
**501**	4-O3B-I4-Se	10	CDK	0.369264	0.012
**502**	4-O3B-I22-,	10	CDK	0.430656	0.059
**503**	4-O3B-J4-S	1	CDK	0.280371	0.567
**504**	4-O3B-J22-	1	CDK	0.328416	0.341
**505**	5-O3B-A19-	10000	CDK	0.421225	0.01
**506**	5-O3B-B19-	1000	CDK	0.437462	0.001
**507**	5-O3B-C19-	100	CDK	0.390765	0.008
**508**	5-O3B-D19	10	CDK	0.036339	0.998
**509**	5-O3B-E19-	1	CDK	0.445008	0.002
**510**	5-O3B-K17-	1	CDK	0.421644	0.002
**511**	5-O3B-M17	10	CDK	0.367962	0.11
**512**	5-O3B-N17	100	CDK	0.501286	0
**513**	5-O3B-O17	1000	CDK	0.459235	0
**514**	5-O3B-P17-	10000	CDK	0.472836	0
**515**	6-O3B-A17-	1000	CDK	0.406195	0.006
**516**	6-O3B-B17-	100	CDK	0.421447	0.003
**517**	6-O3B-C17-	10	CDK	0.421484	0.003
**518**	6-O3B-D17	1	CDK	0.047707	0.979
**519**	6-O3B-E17-	0.1	CDK	0.452251	0.002
**520**	6-O3B-L15-	1	CDK	0.085401	0.991
**521**	6-O3B-M15	10	CDK	0.405231	0.003
**522**	6-O3B-N15	100	CDK	0.448861	0.001
**523**	6-O3B-O15	1000	CDK	0.450721	0
**524**	6-O3B-P15-	10000	CDK	0.43969	0.002
**525**	7-O3B-A21-	10000	BET	0.398559	0.047
**526**	7-O3B-A22-	30000	BET	0.502166	0.002
**527**	7-O3B-B21-	1000	BET	0.430286	0.012
**528**	7-O3B-B22-	3000	BET	0.303967	0.051
**529**	7-O3B-C21-	100	BET	0.177113	0.514
**530**	7-O3B-C22-	300	BET	0.589067	0

**531**	7-O3B-D21	10	BET	0.079813	0.829
**532**	7-O3B-D22	30	BET	0.354717	0.114
**533**	7-O3B-E21-	1	BET	0.088809	0.894
**534**	7-O3B-E22-	3	BET	0.230175	0.239
**535**	7-O3B-G10	10000	BET	0.509049	0.001
**536**	7-O3B-G15	10000	BET	0.49707	0.001
**537**	7-O3B-H10	1000	BET	0.505697	0
**538**	7-O3B-H15	1000	BET	0.517286	0
**539**	7-O3B-I10-	100	BET	0.614659	0
**540**	7-O3B-I15-	100	BET	0.499722	0.001
**541**	7-O3B-J10-	10	BET	0.59897	0
**542**	7-O3B-J15-	10	BET	0.201807	0.668
**543**	7-O3B-K10-	1	BET	0.128929	0.559
**544**	7-O3B-K13-	1	BET	0.195534	0.24
**545**	7-O3B-K15-	1	BET	0.153271	0.584
**546**	7-O3B-L12-	1	BET	0.330092	0.163
**547**	7-O3B-L13-	10	BET	0.577717	0
**548**	7-O3B-L20-	1	BET	0.44462	0.012
**549**	7-O3B-L23-	0.03	BET	0.139943	0.574
**550**	7-O3B-M12	10	BET	0.535624	0
**551**	7-O3B-M13	100	BET	0.536635	0
**552**	7-O3B-M20	10	BET	0.527453	0.002
**553**	7-O3B-M23	0.3	BET	0.322315	0.223
**554**	7-O3B-N12	100	BET	0.22923	0.16
**555**	7-O3B-N13	1000	BET	0.456496	0.007
**556**	7-O3B-N20	100	BET	0.554951	0
**557**	7-O3B-N23	3	BET	0.68069	0
**558**	7-O3B-O12	1000	BET	0.509957	0
**559**	7-O3B-O20	1000	BET	0.472377	0.004
**560**	7-O3B-O23	30	BET	0.36889	0.062
**561**	7-O3B-P12-	10000	BET	0.467848	0.003
**562**	7-O3B-P13-	10000	BET	0.398146	0.055
**563**	7-O3B-P20-	10000	BET	0.453674	0
**564**	7-O3B-P23-	300	BET	0.477803	0
**565**	8-O3B-K22-	1	BET	0.179484	0.855

**566**	8-O3B-L22-	10	BET	0.315731	0.247
**567**	8-O3B-M22	100	BET	0.447024	0.006
**568**	8-O3B-N22	1000	BET	0.406344	0.021
**569**	8-O3B-O22	10000	BET	0.404707	0.009
**570**	1-O3B-A3-\	10000	HDAC	0.378543	0.002
**571**	1-O3B-B3-\	1000	HDAC	0.385785	0.001
**572**	1-O3B-C3-\	100	HDAC	0.371073	0.002
**573**	1-O3B-D3-\	10	HDAC	0.132485	0.955
**574**	1-O3B-E3-V	1	HDAC	0.253773	0.284
**575**	1-O3B-L12-	0.1	HDAC	0.435737	0
**576**	1-O3B-M12	1	HDAC	0.5249	0
**577**	1-O3B-N12	10	HDAC	0.395538	0.001
**578**	1-O3B-O12	100	HDAC	0.385408	0.002
**579**	1-O3B-P12-	1000	HDAC	0.399285	0
**580**	3-O3B-A4-F	1000	HDAC	0.381966	0.003
**581**	3-O3B-B4-F	100	HDAC	0.404979	0
**582**	3-O3B-C4-F	10	HDAC	0.506528	0
**583**	3-O3B-D4-F	1	HDAC	0.42261	0
**584**	3-O3B-E4-P	0.1	HDAC	0.290698	0.509
**585**	3-O3B-F7-C	1000	HDAC	0.40739	0
**586**	3-O3B-G7-(	100	HDAC	0.384212	0.001
**587**	3-O3B-G12	1000000	HDAC	0.332372	0.004
**588**	3-O3B-H7-(	10	HDAC	0.462476	0.002
**589**	3-O3B-H12	100000	HDAC	0.108178	0.94
**590**	3-O3B-I7-Q	1	HDAC	0.184456	0.843
**591**	3-O3B-I12-'	10000	HDAC	0.201928	0.521
**592**	3-O3B-J7-Q	0.1	HDAC	0.070869	0.955
**593**	3-O3B-J12-	1000	HDAC	0.131538	0.956
**594**	3-O3B-K3-E	1	HDAC	0.370086	0.005
**595**	3-O3B-K12-	100	HDAC	0.312159	0.004
**596**	3-O3B-L3-B	10	HDAC	0.027625	0.991
**597**	3-O3B-M3-I	100	HDAC	0.402251	0.001
**598**	3-O3B-N3-E	1000	HDAC	0.372275	0.004
**599**	3-O3B-O3-E	10000	HDAC	0.378022	0.005
**600**	7-O3B-A5-N	10000	HDAC	0.427719	0

**601**	7-O3B-A7-C	10000	HDAC	0.434385	0
**602**	7-O3B-A9-G	1000	HDAC	0.416364	0
**603**	7-O3B-A12-	10000	HDAC	0.416031	0.001
**604**	7-O3B-B5-M	1000	HDAC	0.443498	0
**605**	7-O3B-B7-C	1000	HDAC	0.421319	0
**606**	7-O3B-B12-	1000	HDAC	0.422295	0
**607**	7-O3B-C5-M	100	HDAC	0.284908	0.111
**608**	7-O3B-C7-C	100	HDAC	0.421592	0
**609**	7-O3B-C9-G	100	HDAC	0.44089	0
**610**	7-O3B-D7-C	10	HDAC	0.435163	0.001
**611**	7-O3B-D9-C	10	HDAC	0.288622	0.061
**612**	7-O3B-D12	100	HDAC	0.110869	0.314
**613**	7-O3B-E5-M	10	HDAC	0.099913	0.77
**614**	7-O3B-E7-C	1	HDAC	0.447217	0.001
**615**	7-O3B-E9-G	1	HDAC	0.098669	0.983
**616**	7-O3B-E12-	10	HDAC	0.089231	0.796
**617**	7-O3B-F5-M	1	HDAC	0.08439	0.66
**618**	7-O3B-F7-R	10000	HDAC	0.421145	0
**619**	7-O3B-F9-G	0.1	HDAC	0.303957	0
**620**	7-O3B-F12-	1	HDAC	0.444805	0
**621**	7-O3B-F19-	10000	HDAC	0.409966	0
**622**	7-O3B-G7-F	1000	HDAC	0.428078	0.001
**623**	7-O3B-G19	1000	HDAC	0.367904	0.036
**624**	7-O3B-H7-F	100	HDAC	0.437704	0
**625**	7-O3B-I7-R	10	HDAC	0.497296	0
**626**	7-O3B-I19-I	100	HDAC	0.486246	0
**627**	7-O3B-J7-R	1	HDAC	0.511706	0
**628**	7-O3B-J19-	10	HDAC	0.067738	0.809
**629**	7-O3B-K4-E	1	HDAC	0.393728	0.001
**630**	7-O3B-K11-	1	HDAC	0.455482	0
**631**	7-O3B-K18-	1	HDAC	0.189448	0.094
**632**	7-O3B-K19-	1	HDAC	0.504946	0
**633**	7-O3B-L2-T	0.1	HDAC	0.229817	0.82
**634**	7-O3B-L4-E	10	HDAC	0.180559	0.512
**635**	7-O3B-L5-P	1	HDAC	0.411962	0

**636**	7-O3B-L8-A	1	HDAC	0.392416	0
**637**	7-O3B-L10-	1	HDAC	0.152796	0.195
**638**	7-O3B-L11-	10	HDAC	0.440626	0
**639**	7-O3B-L14-	1	HDAC	0.498038	0
**640**	7-O3B-L16-	1	HDAC	0.442625	0
**641**	7-O3B-L18-	10	HDAC	0.165658	0.882
**642**	7-O3B-M2-1	1	HDAC	0.325438	0.043
**643**	7-O3B-M5-I	10	HDAC	0.4109	0
**644**	7-O3B-M8-,	10	HDAC	0.519076	0
**645**	7-O3B-M10	10	HDAC	0.502665	0
**646**	7-O3B-M11	100	HDAC	0.458227	0
**647**	7-O3B-M14	10	HDAC	0.051878	0.928
**648**	7-O3B-M16	10	HDAC	0.497743	0
**649**	7-O3B-M18	100	HDAC	0.386932	0.001
**650**	7-O3B-N2-1	10	HDAC	0.314146	0.02
**651**	7-O3B-N4-E	100	HDAC	0.495261	0
**652**	7-O3B-N5-F	100	HDAC	0.576404	0
**653**	7-O3B-N8-/	100	HDAC	0.565697	0
**654**	7-O3B-N10	100	HDAC	0.528837	0
**655**	7-O3B-N14	100	HDAC	0.298276	0.021
**656**	7-O3B-N16	100	HDAC	0.22315	0.317
**657**	7-O3B-N18	1000	HDAC	0.416666	0
**658**	7-O3B-O2-1	100	HDAC	0.023253	1
**659**	7-O3B-O4-E	1000	HDAC	0.547758	0
**660**	7-O3B-O5-F	1000	HDAC	0.416506	0
**661**	7-O3B-O8-/	1000	HDAC	0.413167	0
**662**	7-O3B-O10	1000	HDAC	0.511488	0
**663**	7-O3B-O11	1000	HDAC	0.406411	0
**664**	7-O3B-O14	1000	HDAC	0.051461	0.848
**665**	7-O3B-O16	1000	HDAC	0.402239	0
**666**	7-O3B-P2-T	1000	HDAC	0.472349	0
**667**	7-O3B-P4-E	10000	HDAC	0.446333	0
**668**	7-O3B-P5-F	10000	HDAC	0.434446	0
**669**	7-O3B-P8-A	10000	HDAC	0.416154	0
**670**	7-O3B-P10-	10000	HDAC	0.427246	0

**671**	7-O3B-P11	10000	HDAC	0.443886	0
**672**	7-O3B-P14	10000	HDAC	0.465299	0
**673**	7-O3B-P16	10000	HDAC	0.436259	0
**674**	7-O3B-P18	10000	HDAC	0.427115	0
**0**	2-O8W-A16	10000	EGFR	0.250498	0.192
**1**	2-O8W-A19	10000	EGFR	0.292628	0.241
**2**	2-O8W-B19	1000	EGFR	0.075242	0.987
**3**	2-O8W-C16	1000	EGFR	0.302559	0.006
**4**	2-O8W-C19	100	EGFR	0.164352	0.588
**5**	2-O8W-D16	100	EGFR	0.173535	0.692
**6**	2-O8W-D19	10	EGFR	0.173118	0.656
**7**	2-O8W-E16	10	EGFR	0.126721	0.904
**8**	2-O8W-E19	1	EGFR	0.156623	0.734
**9**	2-O8W-F16	1	EGFR	0.177549	0.689
**10**	2-O8W-K11	0.1	EGFR	0.24431	0.036
**11**	2-O8W-L11	1	EGFR	0.17372	0.643
**12**	2-O8W-L16	0.25	EGFR	0.294473	0.187
**13**	2-O8W-L19	0.1	EGFR	0.212037	0.229
**14**	2-O8W-M1	10	EGFR	0.299325	0.122
**15**	2-O8W-M1	2.5	EGFR	0.159006	0.317
**16**	2-O8W-M1	1	EGFR	0.21041	0.461
**17**	2-O8W-N16	25	EGFR	0.403491	0
**18**	2-O8W-N19	10	EGFR	0.402701	0.003
**19**	2-O8W-O1	100	EGFR	0.334424	0.01
**20**	2-O8W-O16	250	EGFR	0.303986	0.15
**21**	2-O8W-O19	100	EGFR	0.146003	0.901
**22**	2-O8W-P11	1000	EGFR	0.259964	0.262
**23**	2-O8W-P16	2500	EGFR	0.257966	0.196
**24**	2-O8W-P19	1000	EGFR	0.303026	0.216
**25**	3-O8W-F21	10000	EGFR	0.098676	0.967
**26**	3-O8W-G20	1000	EGFR	0.217882	0.442
**27**	3-O8W-G2	1000	EGFR	0.31256	0.12
**28**	3-O8W-H20	100	EGFR	0.216239	0.198
**29**	3-O8W-H2	100	EGFR	0.199132	0.503
**30**	3-O8W-I20	10	EGFR	0.112584	0.937

**31**	3-O8W-I21	10	EGFR	0.134633	0.451
**32**	3-O8W-J20	1	EGFR	0.356034	0.055
**33**	3-O8W-J21	1	EGFR	0.259959	0.233
**34**	3-O8W-K4-	1	EGFR	0.295832	0.301
**35**	3-O8W-K18	0.1	EGFR	0.146241	0.919
**36**	3-O8W-K20	0.1	EGFR	0.135313	0.46
**37**	3-O8W-L4-	10	EGFR	0.190936	0.595
**38**	3-O8W-L18	1	EGFR	0.147598	0.356
**39**	3-O8W-M1	10	EGFR	0.341265	0.098
**40**	3-O8W-N4-	100	EGFR	0.295705	0.067
**41**	3-O8W-N18	100	EGFR	0.445854	0.001
**42**	3-O8W-O4-	1000	EGFR	0.170924	0.618
**43**	3-O8W-P4-	10000	EGFR	0.103243	0.963
**44**	3-O8W-P18	1000	EGFR	0.272365	0.284
**45**	4-O8W-F13	1000	EGFR	0.232808	0.222
**46**	4-O8W-G13	100	EGFR	0.315156	0.127
**47**	4-O8W-G16	10000	EGFR	0.176414	0.684
**48**	4-O8W-H13	10	EGFR	0.212105	0.238
**49**	4-O8W-H16	1000	EGFR	0.156933	0.348
**50**	4-O8W-I13	1	EGFR	0.206782	0.067
**51**	4-O8W-I16	100	EGFR	0.219436	0.124
**52**	4-O8W-J13	0.1	EGFR	0.282596	0.03
**53**	4-O8W-J16	10	EGFR	0.465148	0
**54**	4-O8W-K7-	1	EGFR	0.242481	0.062
**55**	4-O8W-K13	0.1	EGFR	0.22487	0.051
**56**	4-O8W-K16	1	EGFR	0.246411	0.029
**57**	4-O8W-L7-	10	EGFR	0.186577	0.259
**58**	4-O8W-L13	1	EGFR	0.288642	0.056
**59**	4-O8W-M7	100	EGFR	0.194608	0.248
**60**	4-O8W-M1	10	EGFR	0.347966	0.037
**61**	4-O8W-N13	100	EGFR	0.224085	0.049
**62**	4-O8W-O7-	1000	EGFR	0.191118	0.236
**63**	4-O8W-P7-	10000	EGFR	0.177366	0.31
**64**	4-O8W-P13	1000	EGFR	0.100954	0.934
**65**	5-O8W-F4-	1000	EGFR	0.254904	0.31

**66**	5-O8W-F7-	1000	EGFR	0.288147	0.068
**67**	5-O8W-G4-	100	EGFR	0.245918	0.274
**68**	5-O8W-G7-	100	EGFR	0.204325	0.461
**69**	5-O8W-H4-	10	EGFR	0.289679	0.122
**70**	5-O8W-H7-	10	EGFR	0.129312	0.878
**71**	5-O8W-I4-	1	EGFR	0.26734	0.146
**72**	5-O8W-I7-	1	EGFR	0.343294	0.018
**73**	5-O8W-J4-	0.1	EGFR	0.195738	0.367
**74**	5-O8W-J7-	0.1	EGFR	0.139646	0.852
**75**	5-O8W-K7-	0.1	EGFR	0.236683	0.288
**76**	5-O8W-L7-	1	EGFR	0.207367	0.093
**77**	5-O8W-M7	10	EGFR	0.360325	0.006
**78**	5-O8W-O7-	100	EGFR	0.198428	0.147
**79**	5-O8W-P7-	1000	EGFR	0.231365	0.45
**80**	2-O8W-A1	2500	VEGFR	0.182837	0.379
**81**	2-O8W-A1	10000	VEGFR	0.223791	0.393
**82**	2-O8W-A2	10000	VEGFR	0.228685	0.332
**83**	2-O8W-B1	250	VEGFR	0.406335	0
**84**	2-O8W-B1	1000	VEGFR	0.191592	0.218
**85**	2-O8W-B2	1000	VEGFR	0.260105	0.189
**86**	2-O8W-C1	25	VEGFR	0.228724	0.251
**87**	2-O8W-C1	100	VEGFR	0.276141	0.054
**88**	2-O8W-D1	2.5	VEGFR	0.252122	0.152
**89**	2-O8W-D1	10	VEGFR	0.173101	0.498
**90**	2-O8W-D2	100	VEGFR	0.192031	0.513
**91**	2-O8W-E17	1	VEGFR	0.263285	0.122
**92**	2-O8W-E2	10	VEGFR	0.239105	0.264
**93**	2-O8W-F13	10000	VEGFR	0.273272	0.172
**94**	2-O8W-F15	0.25	VEGFR	0.215242	0.095
**95**	2-O8W-F1	10000	VEGFR	0.224375	0.373
**96**	2-O8W-F2	1	VEGFR	0.112646	0.671
**97**	2-O8W-F21	10000	VEGFR	0.148253	0.51
**98**	2-O8W-G1	10000	VEGFR	0.289184	0.038
**99**	2-O8W-G1	1000	VEGFR	0.363401	0.069
**100**	2-O8W-G1	1000	VEGFR	0.30758	0.031

**101**	2-O8W-G2	1000	VEGFR	0.228321	0.32
**102**	2-O8W-H1(	1000	VEGFR	0.25533	0.134
**103**	2-O8W-H1	100	VEGFR	0.30658	0.09
**104**	2-O8W-H2	100	VEGFR	0.147396	0.803
**105**	2-O8W-I10	100	VEGFR	0.24305	0.154
**106**	2-O8W-I13	10	VEGFR	0.351086	0.001
**107**	2-O8W-I19	100	VEGFR	0.236426	0.198
**108**	2-O8W-I21	10	VEGFR	0.269083	0.101
**109**	2-O8W-J10	10	VEGFR	0.223696	0.196
**110**	2-O8W-J13	1	VEGFR	0.132841	0.46
**111**	2-O8W-J19	10	VEGFR	0.309262	0.018
**112**	2-O8W-J21	1	VEGFR	0.261843	0.096
**113**	2-O8W-K1(	1	VEGFR	0.242971	0.187
**114**	2-O8W-K1	0.1	VEGFR	0.181701	0.591
**115**	2-O8W-K1	1	VEGFR	0.391029	0
**116**	2-O8W-K1	1	VEGFR	0.275125	0.03
**117**	2-O8W-L12	0.1	VEGFR	0.335059	0.002
**118**	2-O8W-L13	1	VEGFR	0.241256	0.185
**119**	2-O8W-L21	0.1	VEGFR	0.266354	0.13
**120**	2-O8W-M1	1	VEGFR	0.261363	0.106
**121**	2-O8W-M1	10	VEGFR	0.1306	0.536
**122**	2-O8W-M1	10	VEGFR	0.371682	0
**123**	2-O8W-M2	1	VEGFR	0.136836	0.567
**124**	2-O8W-N1	10	VEGFR	0.365888	0.02
**125**	2-O8W-N1	100	VEGFR	0.212806	0.237
**126**	2-O8W-N1	100	VEGFR	0.32681	0.009
**127**	2-O8W-N2	10	VEGFR	0.112339	0.623
**128**	2-O8W-O1	100	VEGFR	0.243639	0.195
**129**	2-O8W-O1	1000	VEGFR	0.301637	0.029
**130**	2-O8W-O2	100	VEGFR	0.254802	0.481
**131**	2-O8W-P1	1000	VEGFR	0.335566	0.019
**132**	2-O8W-P1	1000	VEGFR	0.261727	0.309
**133**	2-O8W-P1	10000	VEGFR	0.240987	0.218
**134**	2-O8W-P21	1000	VEGFR	0.18747	0.742
**135**	3-O8W-A3-	1000	VEGFR	0.281902	0.055

**136**	3-O8W-A6-	1000	VEGFR	0.200236	0.533
**137**	3-O8W-A18	1000	VEGFR	0.226609	0.295
**138**	3-O8W-B3-	100	VEGFR	0.109553	0.689
**139**	3-O8W-B6-	100	VEGFR	0.218981	0.267
**140**	3-O8W-B18	100	VEGFR	0.230174	0.315
**141**	3-O8W-C3-	10	VEGFR	0.135559	0.647
**142**	3-O8W-C6-	10	VEGFR	0.198972	0.243
**143**	3-O8W-C18	10	VEGFR	0.156274	0.744
**144**	3-O8W-D3-	1	VEGFR	0.142278	0.914
**145**	3-O8W-D6-	1	VEGFR	0.083643	0.885
**146**	3-O8W-D18	1	VEGFR	0.232719	0.324
**147**	3-O8W-E3-	0.1	VEGFR	0.135605	0.987
**148**	3-O8W-E6-	0.1	VEGFR	0.161466	0.96
**149**	3-O8W-E18	0.1	VEGFR	0.224877	0.295
**150**	3-O8W-F18	1000	VEGFR	0.277208	0.097
**151**	3-O8W-G18	100	VEGFR	0.281206	0.056
**152**	3-O8W-H18	10	VEGFR	0.196974	0.269
**153**	3-O8W-I18	1	VEGFR	0.219343	0.523
**154**	3-O8W-J18	0.1	VEGFR	0.236906	0.272
**155**	4-O8W-A12	10000	VEGFR	0.173401	0.66
**156**	4-O8W-A15	2500	VEGFR	0.151116	0.423
**157**	4-O8W-A20	10000	VEGFR	0.206471	0.243
**158**	4-O8W-B12	1000	VEGFR	0.15931	0.792
**159**	4-O8W-B15	250	VEGFR	0.163498	0.331
**160**	4-O8W-B20	1000	VEGFR	0.134656	0.557
**161**	4-O8W-C15	25	VEGFR	0.17494	0.288
**162**	4-O8W-D12	100	VEGFR	0.147299	0.516
**163**	4-O8W-D15	2.5	VEGFR	0.222952	0.287
**164**	4-O8W-D20	100	VEGFR	0.155187	0.674
**165**	4-O8W-E12	10	VEGFR	0.240921	0.437
**166**	4-O8W-E20	10	VEGFR	0.177669	0.317
**167**	4-O8W-F12	1	VEGFR	0.151357	0.459
**168**	4-O8W-F15	0.25	VEGFR	0.156659	0.664
**169**	4-O8W-F20	1	VEGFR	0.104958	0.762
**170**	4-O8W-L16	1	VEGFR	0.114826	0.599

**171**	4-O8W-M1	10	VEGFR	0.184125	0.429
**172**	4-O8W-N1(	100	VEGFR	0.114038	0.658
**173**	4-O8W-O1(	1000	VEGFR	0.153008	0.388
**174**	4-O8W-P1(	10000	VEGFR	0.182922	0.555
**175**	2-O8W-L1C	1	PI3K	0.287662	0.047
**176**	2-O8W-M1	10	PI3K	0.346365	0
**177**	2-O8W-N1(	100	PI3K	0.023674	1
**178**	2-O8W-O1(	1000	PI3K	0.322817	0.024
**179**	2-O8W-P1(	10000	PI3K	0.151441	0.753
**180**	3-O8W-A1(	2500	PI3K	0.255986	0.017
**181**	3-O8W-C1(	250	PI3K	0.169779	0.088
**182**	3-O8W-D1(	25	PI3K	0.373412	0.002
**183**	3-O8W-E1(	2.5	PI3K	0.286067	0.115
**184**	3-O8W-F1(	0.25	PI3K	0.20061	0.055
**185**	3-O8W-F17	100000	PI3K	0.024397	1
**186**	3-O8W-F1!	500	PI3K	0.343943	0
**187**	3-O8W-G1.	10000	PI3K	0.091361	0.984
**188**	3-O8W-G1!	50	PI3K	0.244277	0.386
**189**	3-O8W-H1.	1000	PI3K	0.120106	0.927
**190**	3-O8W-I17	100	PI3K	0.060542	0.998
**191**	3-O8W-I19	5	PI3K	0.213597	0.018
**192**	3-O8W-J17	10	PI3K	0.176049	0.048
**193**	3-O8W-J19	0.5	PI3K	0.187911	0.132
**194**	3-O8W-K1!	0.05	PI3K	0.20383	0.742
**195**	3-O8W-L8-I	1	PI3K	0.110969	0.966
**196**	3-O8W-L21	0.1	PI3K	0.180483	0.689
**197**	3-O8W-M8	10	PI3K	0.283305	0.085
**198**	3-O8W-M2	1	PI3K	0.15035	0.962
**199**	3-O8W-N8-	100	PI3K	0.378205	0
**200**	3-O8W-N2:	10	PI3K	0.137877	0.848
**201**	3-O8W-O8-	1000	PI3K	0.164113	0.73
**202**	3-O8W-O2:	100	PI3K	0.386476	0
**203**	3-O8W-P8-	10000	PI3K	0.11943	0.933
**204**	3-O8W-P21	1000	PI3K	0.251015	0.208
**205**	4-O8W-A1!	2500	PI3K	0.310321	0

**206**	4-O8W-B19	250	PI3K	0.281633	0.001
**207**	4-O8W-C19	25	PI3K	0.343017	0
**208**	4-O8W-D19	2.5	PI3K	0.432264	0
**209**	4-O8W-E19	0.25	PI3K	0.030238	1
**210**	4-O8W-F14	1000	PI3K	0.033224	1
**211**	4-O8W-G2-	2500	PI3K	0.38524	0
**212**	4-O8W-G5-	10000	PI3K	0.26303	0.063
**213**	4-O8W-G14	100	PI3K	0.135054	0.899
**214**	4-O8W-G20	10000	PI3K	0.032386	1
**215**	4-O8W-H2-	250	PI3K	0.280854	0
**216**	4-O8W-H5-	1000	PI3K	0.117157	0.937
**217**	4-O8W-H14	10	PI3K	0.363038	0
**218**	4-O8W-H20	1000	PI3K	0.341901	0
**219**	4-O8W-I2-T	25	PI3K	0.384755	0.002
**220**	4-O8W-I5-S	100	PI3K	0.421013	0
**221**	4-O8W-I14	1	PI3K	0.332501	0
**222**	4-O8W-I20	100	PI3K	0.356944	0
**223**	4-O8W-J2-T	2.5	PI3K	0.314269	0
**224**	4-O8W-J5-S	10	PI3K	0.217908	0.201
**225**	4-O8W-J20	10	PI3K	0.182185	0.278
**226**	4-O8W-K2-	0.25	PI3K	0.387827	0
**227**	4-O8W-K4-	0.1	PI3K	0.407534	0
**228**	4-O8W-K5-	1	PI3K	0.344724	0.006
**229**	4-O8W-K14	0.1	PI3K	0.400935	0
**230**	4-O8W-K20	1	PI3K	0.339659	0
**231**	4-O8W-L4-I	1	PI3K	0.315032	0
**232**	4-O8W-L14	0.1	PI3K	0.302648	0
**233**	4-O8W-L15	1	PI3K	0.41051	0
**234**	4-O8W-L21	0.1	PI3K	0.349731	0
**235**	4-O8W-M1	1	PI3K	0.28557	0.11
**236**	4-O8W-M1	10	PI3K	0.374891	0
**237**	4-O8W-M2	1	PI3K	0.359117	0
**238**	4-O8W-N4-	10	PI3K	0.387067	0
**239**	4-O8W-N14	10	PI3K	0.344601	0
**240**	4-O8W-N15	100	PI3K	0.308304	0

**241**	4-O8W-N2:	10	PI3K	0.298061	0
**242**	4-O8W-O4-	100	PI3K	0.220201	0.417
**243**	4-O8W-O1/	100	PI3K	0.416903	0
**244**	4-O8W-O1!	1000	PI3K	0.412039	0
**245**	4-O8W-O2:	100	PI3K	0.452733	0
**246**	4-O8W-P4-	1000	PI3K	0.154684	0.85
**247**	4-O8W-P1/	1000	PI3K	0.142057	0.853
**248**	4-O8W-P1!	10000	PI3K	0.409638	0
**249**	4-O8W-P21	1000	PI3K	0.174325	0.591
**250**	5-O8W-A6-	2500	PI3K	0.147444	0.863
**251**	5-O8W-A7-	1000	PI3K	0.319603	0
**252**	5-O8W-A1(	2500	PI3K	0.358639	0.007
**253**	5-O8W-A1:	10000	PI3K	0.282006	0.422
**254**	5-O8W-B6-	250	PI3K	0.260141	0.162
**255**	5-O8W-B7-	100	PI3K	0.35769	0.027
**256**	5-O8W-B1:	1000	PI3K	0.06489	0.999
**257**	5-O8W-C6-	25	PI3K	0.30736	0.002
**258**	5-O8W-C7-	10	PI3K	0.127604	0.923
**259**	5-O8W-C1(	250	PI3K	0.294913	0
**260**	5-O8W-C1:	100	PI3K	0.098613	0.952
**261**	5-O8W-D6-	2.5	PI3K	0.356291	0
**262**	5-O8W-D7-	1	PI3K	0.356315	0
**263**	5-O8W-D1(	25	PI3K	0.218778	0.461
**264**	5-O8W-D1:	10	PI3K	0.362987	0
**265**	5-O8W-E6-I	0.25	PI3K	0.1276	0.904
**266**	5-O8W-E7-,	0.1	PI3K	0.158951	0.754
**267**	5-O8W-E1(	2.5	PI3K	0.103259	0.936
**268**	5-O8W-E17	1	PI3K	0.054033	0.998
**269**	5-O8W-F11	10000	PI3K	0.175005	0.52
**270**	5-O8W-F1(	0.25	PI3K	0.30268	0.042
**271**	5-O8W-G9-	10000	PI3K	0.355536	0.006
**272**	5-O8W-G1:	1000	PI3K	0.208601	0.201
**273**	5-O8W-H9-	1000	PI3K	0.250986	0.001
**274**	5-O8W-H1:	100	PI3K	0.296759	0.044
**275**	5-O8W-I9-S	100	PI3K	0.285861	0

**276**	5-O8W-I11	10	PI3K	0.234799	0.006
**277**	5-O8W-J9-!	10	PI3K	0.314331	0
**278**	5-O8W-J11	1	PI3K	0.275091	0
**279**	5-O8W-K9-	1	PI3K	0.276988	0
**280**	5-O8W-L14	0.1	PI3K	0.15133	0.609
**281**	5-O8W-L2C	1	PI3K	0.331841	0
**282**	5-O8W-L23	0.1	PI3K	0.216122	0.023
**283**	5-O8W-M1	1	PI3K	0.365002	0
**284**	5-O8W-M2	10	PI3K	0.457414	0
**285**	5-O8W-M2	1	PI3K	0.284106	0
**286**	5-O8W-N1	10	PI3K	0.403777	0
**287**	5-O8W-N2(	100	PI3K	0.227782	0.008
**288**	5-O8W-N2:	10	PI3K	0.401568	0
**289**	5-O8W-O1	100	PI3K	0.473442	0
**290**	5-O8W-O2(	1000	PI3K	0.276608	0.053
**291**	5-O8W-O2:	100	PI3K	0.13146	0.853
**292**	5-O8W-P1	1000	PI3K	0.239639	0.004
**293**	5-O8W-P2(	10000	PI3K	0.146288	0.795
**294**	5-O8W-P2:	1000	PI3K	0.120202	0.936
**295**	6-O8W-A8-	10000	PI3K	0.286104	0.127
**296**	6-O8W-B8-	1000	PI3K	0.044199	0.998
**297**	6-O8W-C8-	100	PI3K	0.113796	0.922
**298**	6-O8W-D8-	10	PI3K	0.263717	0.097
**299**	6-O8W-E8-	1	PI3K	0.261598	0.016
**300**	6-O8W-L6-	1	PI3K	0.265032	0
**301**	6-O8W-M6	10	PI3K	0.317773	0
**302**	6-O8W-N6-	100	PI3K	0.251235	0
**303**	6-O8W-O6-	1000	PI3K	0.427582	0
**304**	6-O8W-P6-	10000	PI3K	0.032855	1
**305**	1-O8W-F11	10000	Topoisome	0.498081	0
**306**	1-O8W-G1:	1000	Topoisome	0.502355	0
**307**	1-O8W-G2(	1000	Topoisome	0.450627	0
**308**	1-O8W-H1:	100	Topoisome	0.431649	0.006
**309**	1-O8W-H2(	100	Topoisome	0.46722	0
**310**	1-O8W-I11	10	Topoisome	0.45997	0

**311**	1-O8W-I20	10	Topoisome	0.233796	0.725
**312**	1-O8W-J11	1	Topoisome	0.172105	0.54
**313**	1-O8W-J20	1	Topoisome	0.315867	0.068
**314**	1-O8W-K11	1	Topoisome	0.221476	0.489
**315**	1-O8W-K2(	0.1	Topoisome	0.033412	1
**316**	1-O8W-L11	10	Topoisome	0.503568	0
**317**	1-O8W-L14	1	Topoisome	0.135073	0.794
**318**	1-O8W-M1	100	Topoisome	0.49997	0
**319**	1-O8W-M1	10	Topoisome	0.266186	0.235
**320**	1-O8W-N1·	100	Topoisome	0.488649	0
**321**	1-O8W-O1:	1000	Topoisome	0.473249	0
**322**	1-O8W-O1·	1000	Topoisome	0.539454	0
**323**	1-O8W-P11	10000	Topoisome	0.466457	0
**324**	1-O8W-P1·	10000	Topoisome	0.457466	0
**325**	3-O8W-A11	10000	Topoisome	0.491221	0
**326**	3-O8W-B11	1000	Topoisome	0.531673	0
**327**	3-O8W-C11	100	Topoisome	0.464886	0
**328**	3-O8W-D1:	10	Topoisome	0.511971	0
**329**	3-O8W-E11	1	Topoisome	0.079984	0.957
**330**	3-O8W-G9-	1000	Topoisome	0.47121	0
**331**	3-O8W-G1(	10000	Topoisome	0.489972	0
**332**	3-O8W-H9-	100	Topoisome	0.476054	0
**333**	3-O8W-H1(	1000	Topoisome	0.494379	0
**334**	3-O8W-I9-[	10	Topoisome	0.328256	0.337
**335**	3-O8W-I10	100	Topoisome	0.438033	0
**336**	3-O8W-J9-I	1	Topoisome	0.568434	0
**337**	3-O8W-J10	10	Topoisome	0.56904	0
**338**	3-O8W-K7-	0.1	Topoisome	0.49911	0
**339**	3-O8W-K9-	0.1	Topoisome	0.036273	1
**340**	3-O8W-K1(	1	Topoisome	0.54539	0
**341**	3-O8W-L6-I	0.1	Topoisome	0.509206	0
**342**	3-O8W-L7-I	1	Topoisome	0.518733	0.001
**343**	3-O8W-L9-'	0.5	Topoisome	0.560376	0
**344**	3-O8W-L1C	0.1	Topoisome	0.586219	0
**345**	3-O8W-L16	1	Topoisome	0.412922	0.005

346	3-O8W-M6	1	Topoisome	0.458593	0
347	3-O8W-M7	10	Topoisome	0.514134	0
348	3-O8W-M9	5	Topoisome	0.516333	0
349	3-O8W-M1	1	Topoisome	0.62788	0
350	3-O8W-M1	10	Topoisome	0.584026	0
351	3-O8W-N6-	10	Topoisome	0.059802	0.978
352	3-O8W-N9-	50	Topoisome	0.383784	0.034
353	3-O8W-N1(	10	Topoisome	0.597449	0
354	3-O8W-N1(	100	Topoisome	0.070447	0.986
355	3-O8W-O6-	100	Topoisome	0.514506	0
356	3-O8W-O7-	100	Topoisome	0.479194	0
357	3-O8W-O9-	500	Topoisome	0.509611	0
358	3-O8W-O1(	100	Topoisome	0.523385	0
359	3-O8W-O1(	1000	Topoisome	0.099133	0.966
360	3-O8W-P6-	1000	Topoisome	0.480326	0
361	3-O8W-P7-	1000	Topoisome	0.496621	0
362	3-O8W-P9-	5000	Topoisome	0.516015	0
363	3-O8W-P1(	1000	Topoisome	0.521447	0
364	3-O8W-P1(	10000	Topoisome	0.526217	0
365	1-O8W-A1(	10000	Mitotic	0.569736	0
366	1-O8W-A1:	1000	Mitotic	0.560985	0
367	1-O8W-A1{	1000	Mitotic	0.568315	0
368	1-O8W-B1(	1000	Mitotic	0.557203	0
369	1-O8W-B1:	100	Mitotic	0.108165	0.968
370	1-O8W-B1{	100	Mitotic	0.574438	0
371	1-O8W-C1(	100	Mitotic	0.560573	0
372	1-O8W-C1:	10	Mitotic	0.377848	0.06
373	1-O8W-C1{	10	Mitotic	0.549419	0
374	1-O8W-D1(	10	Mitotic	0.503277	0
375	1-O8W-D1:	1	Mitotic	0.060846	1
376	1-O8W-D1{	1	Mitotic	0.396409	0.01
377	1-O8W-E1(	1	Mitotic	0.455794	0.001
378	1-O8W-E13	0.1	Mitotic	0.536643	0
379	1-O8W-E1{	0.1	Mitotic	0.454628	0
380	1-O8W-F13	1000	Mitotic	0.574487	0

**381**	1-O8W-G1:	100	Mitotic	0.572154	0
**382**	1-O8W-G1!	1000	Mitotic	0.549814	0
**383**	1-O8W-H1:	10	Mitotic	0.400281	0.014
**384**	1-O8W-H1!	100	Mitotic	0.553538	0
**385**	1-O8W-I13	1	Mitotic	0.169771	0.966
**386**	1-O8W-I15	10	Mitotic	0.556105	0
**387**	1-O8W-J13	0.1	Mitotic	0.233608	0.268
**388**	1-O8W-J15	1	Mitotic	0.506309	0
**389**	1-O8W-K7-	0.1	Mitotic	0.405881	0.009
**390**	1-O8W-K1!	0.1	Mitotic	0.082936	0.927
**391**	1-O8W-L7-'	1	Mitotic	0.126439	0.908
**392**	1-O8W-L2(	0.1	Mitotic	0.039325	1
**393**	1-O8W-M7	10	Mitotic	0.099722	0.896
**394**	1-O8W-M2	1	Mitotic	0.463366	0.007
**395**	1-O8W-N2(	10	Mitotic	0.385109	0.039
**396**	1-O8W-O7-	100	Mitotic	0.552624	0
**397**	1-O8W-O2(	100	Mitotic	0.177645	0.85
**398**	1-O8W-P7-	1000	Mitotic	0.547704	0
**399**	1-O8W-P2(	1000	Mitotic	0.544482	0
**400**	3-O8W-A7-	1000	Mitotic	0.513953	0
**401**	3-O8W-B7-	100	Mitotic	0.514942	0
**402**	3-O8W-C7-	10	Mitotic	0.50152	0
**403**	3-O8W-D7-	1	Mitotic	0.342747	0.157
**404**	3-O8W-E7-I	0.1	Mitotic	0.401517	0.014
**405**	6-O8W-L1(	1	Mitotic	0.227497	0.38
**406**	6-O8W-M1	10	Mitotic	0.145103	0.756
**407**	6-O8W-N1(	100	Mitotic	0.135149	0.678
**408**	6-O8W-O1(	1000	Mitotic	0.546734	0
**409**	6-O8W-P1(	10000	Mitotic	0.524693	0
**410**	2-O8W-A1:	250	MEK1/2	0.366506	0.129
**411**	2-O8W-B1:	25	MEK1/2	0.528163	0.003
**412**	2-O8W-D1:	2.5	MEK1/2	0.768852	0
**413**	2-O8W-E12	0.25	MEK1/2	0.662431	0
**414**	2-O8W-F12	2.5000000	MEK1/2	0.20874	0.806
**415**	2-O8W-F14	1000	MEK1/2	0.439891	0.031

**416**	2-O8W-G1‹	100	MEK1/2	0.744933	0
**417**	2-O8W-H1‹	10	MEK1/2	0.526824	0
**418**	2-O8W-I14	1	MEK1/2	0.377496	0.189
**419**	2-O8W-K1‹	0.1	MEK1/2	0.540061	0.003
**420**	2-O8W-L2(	1	MEK1/2	0.163374	0.957
**421**	2-O8W-M2	10	MEK1/2	0.460643	0.009
**422**	2-O8W-N2(	100	MEK1/2	0.67526	0
**423**	2-O8W-O2(	1000	MEK1/2	0.686656	0
**424**	2-O8W-P2(	10000	MEK1/2	0.451345	0.029
**425**	4-O8W-A1(	1000	MEK1/2	0.724714	0
**426**	4-O8W-A1:	1000	MEK1/2	0.693597	0
**427**	4-O8W-B1(	100	MEK1/2	0.737253	0
**428**	4-O8W-B1:	100	MEK1/2	0.755722	0
**429**	4-O8W-C1(	10	MEK1/2	0.722074	0
**430**	4-O8W-C1:	10	MEK1/2	0.701649	0
**431**	4-O8W-D1(	1	MEK1/2	0.686295	0
**432**	4-O8W-D1:	1	MEK1/2	0.643191	0
**433**	4-O8W-E1(	0.1	MEK1/2	0.108359	0.982
**434**	4-O8W-E13	0.1	MEK1/2	0.528466	0.013
**435**	4-O8W-L1(	0.25	MEK1/2	0.647212	0
**436**	4-O8W-M1	2.5	MEK1/2	0.57213	0.004
**437**	4-O8W-N1(	25	MEK1/2	0.226795	0.413
**438**	4-O8W-O1(	250	MEK1/2	0.633633	0
**439**	4-O8W-P1(	2500	MEK1/2	0.71452	0
**440**	1-O8W-L2-(	1	PARP	0.209586	0.563
**441**	1-O8W-L6-I	1	PARP	0.224219	0.912
**442**	1-O8W-M2	10	PARP	0.417835	0.078
**443**	1-O8W-M6	10	PARP	0.102716	1
**444**	1-O8W-N2-	100	PARP	0.527951	0.008
**445**	1-O8W-N6-	100	PARP	0.202192	0.569
**446**	1-O8W-O2-	1000	PARP	0.651993	0
**447**	1-O8W-O6-	1000	PARP	0.489419	0.007
**448**	1-O8W-P2-	10000	PARP	0.58299	0
**449**	1-O8W-P6-	10000	PARP	0.565019	0
**450**	7-O8W-A3-	1000	PARP	0.694655	0

**451**	7-O8W-B2- 10000	PARP	0.665937	0
**452**	7-O8W-B3- 100	PARP	0.658935	0
**453**	7-O8W-C2- 1000	PARP	0.461958	0.013
**454**	7-O8W-C3- 10	PARP	0.669517	0
**455**	7-O8W-D2- 100	PARP	0.583869	0
**456**	7-O8W-D3- 1	PARP	0.545748	0
**457**	7-O8W-E2- 10	PARP	0.43157	0.039
**458**	7-O8W-E3- 0.1	PARP	0.439561	0.039
**459**	7-O8W-F2- 1	PARP	0.24523	0.533
**460**	7-O8W-G2- 10000	PARP	0.670598	0
**461**	7-O8W-H2- 1000	PARP	0.641978	0
**462**	7-O8W-I2- 100	PARP	0.657883	0
**463**	7-O8W-J2- 10	PARP	0.254132	0.668
**464**	7-O8W-K2- 1	PARP	0.667625	0
**465**	3-O8W-A1 1000	CDK	0.234632	0.39
**466**	3-O8W-B1 100	CDK	0.246155	0.314
**467**	3-O8W-B2 2500	CDK	0.339891	0.084
**468**	3-O8W-C1 10	CDK	0.301909	0.074
**469**	3-O8W-C2 250	CDK	0.316938	0.281
**470**	3-O8W-D1 1	CDK	0.258743	0.084
**471**	3-O8W-D2 25	CDK	0.301533	0.102
**472**	3-O8W-E1 0.1	CDK	0.456305	0.003
**473**	3-O8W-E23 2.5	CDK	0.25074	0.707
**474**	3-O8W-F23 0.25	CDK	0.254028	0.117
**475**	3-O8W-K1 1	CDK	0.116213	0.938
**476**	3-O8W-L1 1	CDK	0.257358	0.301
**477**	3-O8W-M1 10	CDK	0.319228	0.097
**478**	3-O8W-M1 10	CDK	0.246229	0.092
**479**	3-O8W-N1 100	CDK	0.183517	0.277
**480**	3-O8W-N1 100	CDK	0.172757	0.246
**481**	3-O8W-O1 1000	CDK	0.208184	0.353
**482**	3-O8W-O1 1000	CDK	0.355826	0.105
**483**	3-O8W-P1 10000	CDK	0.225836	0.564
**484**	3-O8W-P1 10000	CDK	0.381538	0.053
**485**	4-O8W-A4- 10000	CDK	0.318738	0.064

**486**	4-O8W-A8-	10000	CDK	0.288331	0.134
**487**	4-O8W-B4-	1000	CDK	0.285145	0.129
**488**	4-O8W-B8-	1000	CDK	0.125716	0.912
**489**	4-O8W-C4-	100	CDK	0.149152	0.772
**490**	4-O8W-C8-	100	CDK	0.128365	0.959
**491**	4-O8W-D4-	10	CDK	0.217206	0.652
**492**	4-O8W-D8-	10	CDK	0.23266	0.554
**493**	4-O8W-E4-	1	CDK	0.115194	0.876
**494**	4-O8W-E8-	1	CDK	0.218229	0.555
**495**	4-O8W-F4-	10000	CDK	0.310637	0.338
**496**	4-O8W-F22	10000	CDK	0.330837	0.048
**497**	4-O8W-G4-	1000	CDK	0.358967	0.057
**498**	4-O8W-G2	1000	CDK	0.339167	0.045
**499**	4-O8W-H4-	100	CDK	0.342598	0.025
**500**	4-O8W-H2	100	CDK	0.288517	0.136
**501**	4-O8W-I4-	10	CDK	0.126023	0.859
**502**	4-O8W-I22	10	CDK	0.334224	0.098
**503**	4-O8W-J4-	1	CDK	0.321926	0.339
**504**	4-O8W-J22	1	CDK	0.211606	0.243
**505**	5-O8W-A1	10000	CDK	0.286484	0.174
**506**	5-O8W-B1	1000	CDK	0.189714	0.688
**507**	5-O8W-C1	100	CDK	0.376743	0.052
**508**	5-O8W-D1	10	CDK	0.309495	0.45
**509**	5-O8W-E1	1	CDK	0.145811	0.93
**510**	5-O8W-K1	1	CDK	0.089736	0.803
**511**	5-O8W-M1	10	CDK	0.108947	0.755
**512**	5-O8W-N1	100	CDK	0.256597	0.34
**513**	5-O8W-O1	1000	CDK	0.243231	0.356
**514**	5-O8W-P1	10000	CDK	0.26107	0.257
**515**	6-O8W-A1	1000	CDK	0.159747	0.503
**516**	6-O8W-B1	100	CDK	0.132256	0.962
**517**	6-O8W-C1	10	CDK	0.229785	0.288
**518**	6-O8W-D1	1	CDK	0.296113	0.155
**519**	6-O8W-E17	0.1	CDK	0.150421	0.684
**520**	6-O8W-L1	1	CDK	0.133064	0.708

**521**	6-O8W-M1	10	CDK	0.237269	0.238
**522**	6-O8W-N1!	100	CDK	0.130425	0.665
**523**	6-O8W-O1!	1000	CDK	0.2661	0.208
**524**	6-O8W-P1!	10000	CDK	0.28248	0.184
**525**	7-O8W-A21	10000	BET	0.388285	0.02
**526**	7-O8W-A22	30000	BET	0.585032	0
**527**	7-O8W-B21	1000	BET	0.438292	0.027
**528**	7-O8W-B22	3000	BET	0.205872	0.283
**529**	7-O8W-C21	100	BET	0.35822	0.059
**530**	7-O8W-C22	300	BET	0.352516	0.049
**531**	7-O8W-D2:	10	BET	0.365322	0.047
**532**	7-O8W-D2:	30	BET	0.398439	0.068
**533**	7-O8W-E21	1	BET	0.148311	0.58
**534**	7-O8W-E22	3	BET	0.176	0.405
**535**	7-O8W-G1(	10000	BET	0.437982	0.005
**536**	7-O8W-G1!	10000	BET	0.532009	0
**537**	7-O8W-H1(	1000	BET	0.433543	0.011
**538**	7-O8W-H1!	1000	BET	0.480507	0.001
**539**	7-O8W-I10	100	BET	0.313004	0.091
**540**	7-O8W-I15	100	BET	0.336031	0.039
**541**	7-O8W-J10	10	BET	0.372383	0.02
**542**	7-O8W-J15	10	BET	0.410651	0.014
**543**	7-O8W-K1(	1	BET	0.260201	0.387
**544**	7-O8W-K1:	1	BET	0.502785	0
**545**	7-O8W-K1!	1	BET	0.159269	0.701
**546**	7-O8W-L12	1	BET	0.493476	0.002
**547**	7-O8W-L13	10	BET	0.653048	0
**548**	7-O8W-L2C	1	BET	0.175313	0.409
**549**	7-O8W-L23	0.03	BET	0.236814	0.409
**550**	7-O8W-M1	10	BET	0.159918	0.687
**551**	7-O8W-M1	100	BET	0.514527	0
**552**	7-O8W-M2	10	BET	0.544386	0
**553**	7-O8W-M2	0.3	BET	0.332085	0.09
**554**	7-O8W-N1:	100	BET	0.712893	0
**555**	7-O8W-N1:	1000	BET	0.460785	0

**556**	7-O8W-N2(	100	BET	0.4829	0
**557**	7-O8W-N2:	3	BET	0.375114	0.037
**558**	7-O8W-O1:	1000	BET	0.518483	0.001
**559**	7-O8W-O2(	1000	BET	0.490565	0.001
**560**	7-O8W-O2:	30	BET	0.495916	0.001
**561**	7-O8W-P1:	10000	BET	0.516955	0
**562**	7-O8W-P1:	10000	BET	0.342178	0.061
**563**	7-O8W-P2(	10000	BET	0.449381	0.003
**564**	7-O8W-P2:	300	BET	0.412464	0.008
**565**	8-O8W-K2:	1	BET	0.111425	0.738
**566**	8-O8W-L22	10	BET	0.291515	0.225
**567**	8-O8W-M2	100	BET	0.384561	0.102
**568**	8-O8W-N2:	1000	BET	0.517432	0.002
**569**	8-O8W-O2:	10000	BET	0.458038	0.003
**570**	1-O8W-A3-	10000	HDAC	0.46773	0
**571**	1-O8W-B3-	1000	HDAC	0.392237	0
**572**	1-O8W-C3-	100	HDAC	0.259899	0.318
**573**	1-O8W-D3-	10	HDAC	0.366036	0
**574**	1-O8W-E3-'	1	HDAC	0.389544	0
**575**	1-O8W-L12	0.1	HDAC	0.051112	0.968
**576**	1-O8W-M1	1	HDAC	0.100674	0.783
**577**	1-O8W-N1:	10	HDAC	0.403063	0
**578**	1-O8W-O1:	100	HDAC	0.381101	0
**579**	1-O8W-P1:	1000	HDAC	0.367799	0
**580**	3-O8W-A4-	1000	HDAC	0.347367	0
**581**	3-O8W-B4-	100	HDAC	0.404901	0
**582**	3-O8W-C4-	10	HDAC	0.412031	0.001
**583**	3-O8W-D4-	1	HDAC	0.328904	0.024
**584**	3-O8W-E4-I	0.1	HDAC	0.329073	0.004
**585**	3-O8W-F7-(	1000	HDAC	0.364089	0
**586**	3-O8W-G7-	100	HDAC	0.397578	0
**587**	3-O8W-G1:	1000000	HDAC	0.332945	0.005
**588**	3-O8W-H7-	10	HDAC	0.331751	0.018
**589**	3-O8W-H1:	100000	HDAC	0.164453	0.594
**590**	3-O8W-I7-(	1	HDAC	0.033252	0.999

**591**	3-O8W-I12	10000	HDAC	0.148742	0.564
**592**	3-O8W-J7-(	0.1	HDAC	0.437703	0
**593**	3-O8W-J12	1000	HDAC	0.142902	0.688
**594**	3-O8W-K3-	1	HDAC	0.03856	1
**595**	3-O8W-K12	100	HDAC	0.156029	0.6
**596**	3-O8W-L3-I	10	HDAC	0.249632	0.127
**597**	3-O8W-M3	100	HDAC	0.258823	0.323
**598**	3-O8W-N3-	1000	HDAC	0.4057	0
**599**	3-O8W-O3-	10000	HDAC	0.362643	0.001
**600**	7-O8W-A5-	10000	HDAC	0.354186	0
**601**	7-O8W-A7-	10000	HDAC	0.36876	0
**602**	7-O8W-A9-	1000	HDAC	0.448529	0
**603**	7-O8W-A12	10000	HDAC	0.464721	0
**604**	7-O8W-B5-	1000	HDAC	0.43709	0
**605**	7-O8W-B7-	1000	HDAC	0.38383	0
**606**	7-O8W-B12	1000	HDAC	0.412362	0
**607**	7-O8W-C5-	100	HDAC	0.443164	0
**608**	7-O8W-C7-	100	HDAC	0.353248	0.002
**609**	7-O8W-C9-	100	HDAC	0.368097	0.002
**610**	7-O8W-D7-	10	HDAC	0.459597	0
**611**	7-O8W-D9-	10	HDAC	0.17315	0.526
**612**	7-O8W-D12	100	HDAC	0.292823	0.106
**613**	7-O8W-E5-I	10	HDAC	0.411379	0
**614**	7-O8W-E7-(	1	HDAC	0.34234	0
**615**	7-O8W-E9-(	1	HDAC	0.125856	0.593
**616**	7-O8W-E12	10	HDAC	0.105389	0.937
**617**	7-O8W-F5-I	1	HDAC	0.383409	0
**618**	7-O8W-F7-I	10000	HDAC	0.46581	0
**619**	7-O8W-F9-(	0.1	HDAC	0.410296	0.001
**620**	7-O8W-F12	1	HDAC	0.382241	0
**621**	7-O8W-F19	10000	HDAC	0.386584	0
**622**	7-O8W-G7-	1000	HDAC	0.449092	0
**623**	7-O8W-G19	1000	HDAC	0.369092	0
**624**	7-O8W-H7-	100	HDAC	0.099453	0.911
**625**	7-O8W-I7-F	10	HDAC	0.419699	0

**626**	7-O8W-I19	100	HDAC	0.055492	0.974
**627**	7-O8W-J7-	1	HDAC	0.4923	0
**628**	7-O8W-J19	10	HDAC	0.120905	0.885
**629**	7-O8W-K4-	1	HDAC	0.378794	0.001
**630**	7-O8W-K11	1	HDAC	0.089885	0.864
**631**	7-O8W-K18	1	HDAC	0.023841	0.998
**632**	7-O8W-K19	1	HDAC	0.137424	0.523
**633**	7-O8W-L2-	0.1	HDAC	0.140488	0.836
**634**	7-O8W-L4-	10	HDAC	0.188898	0.204
**635**	7-O8W-L5-	1	HDAC	0.160074	0.405
**636**	7-O8W-L8-	1	HDAC	0.41525	0
**637**	7-O8W-L10	1	HDAC	0.264457	0.115
**638**	7-O8W-L11	10	HDAC	0.388871	0.003
**639**	7-O8W-L14	1	HDAC	0.14088	0.665
**640**	7-O8W-L16	1	HDAC	0.056342	0.992
**641**	7-O8W-L18	10	HDAC	0.104946	0.921
**642**	7-O8W-M2	1	HDAC	0.299544	0.118
**643**	7-O8W-M5	10	HDAC	0.441195	0
**644**	7-O8W-M8	10	HDAC	0.439611	0
**645**	7-O8W-M1	10	HDAC	0.458961	0
**646**	7-O8W-M1	100	HDAC	0.242933	0.059
**647**	7-O8W-M1	10	HDAC	0.425225	0
**648**	7-O8W-M1	10	HDAC	0.377034	0.001
**649**	7-O8W-M1	100	HDAC	0.432972	0
**650**	7-O8W-N2-	10	HDAC	0.442132	0
**651**	7-O8W-N4-	100	HDAC	0.440554	0
**652**	7-O8W-N5-	100	HDAC	0.451601	0
**653**	7-O8W-N8-	100	HDAC	0.308881	0.01
**654**	7-O8W-N10	100	HDAC	0.45706	0
**655**	7-O8W-N14	100	HDAC	0.132412	0.632
**656**	7-O8W-N16	100	HDAC	0.222173	0.1
**657**	7-O8W-N18	1000	HDAC	0.366351	0.008
**658**	7-O8W-O2-	100	HDAC	0.431535	0
**659**	7-O8W-O4-	1000	HDAC	0.515346	0
**660**	7-O8W-O5-	1000	HDAC	0.426142	0

**661**	7-O8W-O8-	1000	HDAC	0.490781	0
**662**	7-O8W-O1(	1000	HDAC	0.349101	0.004
**663**	7-O8W-O1:	1000	HDAC	0.4526	0
**664**	7-O8W-O1‹	1000	HDAC	0.544238	0
**665**	7-O8W-O1€	1000	HDAC	0.429614	0.001
**666**	7-O8W-P2-	1000	HDAC	0.135096	0.499
**667**	7-O8W-P4-	10000	HDAC	0.473383	0
**668**	7-O8W-P5-	10000	HDAC	0.366678	0
**669**	7-O8W-P8-	10000	HDAC	0.349609	0
**670**	7-O8W-P1(	10000	HDAC	0.4543	0
**671**	7-O8W-P1:	10000	HDAC	0.358866	0
**672**	7-O8W-P1‹	10000	HDAC	0.416733	0
**673**	7-O8W-P1€	10000	HDAC	0.497262	0
**674**	7-O8W-P1ᵹ	10000	HDAC	0.383683	0
