# Supplementary material for: Evaluating feature extraction in ovarian cancer cell line co-cultures using deep neural networks
Source: Commun Biol. 2025 Feb 25;8:303. doi: 10.1038/s42003-025-07766-w (PMC11862010; doi:10.1038/s42003-025-07766-w)
Supplement: Supplementary file 5 — Supplementary Data 3 [file 42003_2025_7766_MOESM5_ESM.pdf]

	Well_annotati	_concentra	ell_Catego	Highest_ES	Pvalue
**0**	2-KB-A16-G	10000	EGFR	0.371434	0.019
**1**	2-KB-A19-E	10000	EGFR	0.554818	0
**2**	2-KB-B19-E	1000	EGFR	0.182817	0.922
**3**	2-KB-C16-G	1000	EGFR	0.559866	0
**4**	2-KB-C19-E	100	EGFR	0.560381	0
**5**	2-KB-D16-G	100	EGFR	0.213268	0.335
**6**	2-KB-D19-E	10	EGFR	0.454705	0
**7**	2-KB-E16-G	10	EGFR	0.50186	0
**8**	2-KB-E19-E	1	EGFR	0.529522	0
**9**	2-KB-F16-G	1	EGFR	0.458124	0
**10**	2-KB-K11-A	0.1	EGFR	0.417556	0
**11**	2-KB-L11-A	1	EGFR	0.490293	0
**12**	2-KB-L16-O	0.25	EGFR	0.559801	0
**13**	2-KB-L19-L	0.1	EGFR	0.523638	0
**14**	2-KB-M11-A	10	EGFR	0.472248	0
**15**	2-KB-M16-O	2.5	EGFR	0.440895	0
**16**	2-KB-M19-L	1	EGFR	0.506358	0
**17**	2-KB-N16-O	25	EGFR	0.481277	0
**18**	2-KB-N19-L	10	EGFR	0.499524	0
**19**	2-KB-O11-A	100	EGFR	0.527323	0
**20**	2-KB-O16-O	250	EGFR	0.472361	0
**21**	2-KB-O19-L	100	EGFR	0.479151	0
**22**	2-KB-P11-A	1000	EGFR	0.307172	0.24
**23**	2-KB-P16-O	2500	EGFR	0.411317	0.02
**24**	2-KB-P19-L	1000	EGFR	0.525212	0
**25**	3-KB-F21-R	10000	EGFR	0.093805	0.991
**26**	3-KB-G20-N	1000	EGFR	0.533567	0
**27**	3-KB-G21-R	1000	EGFR	0.184169	0.794
**28**	3-KB-H20-N	100	EGFR	0.529357	0
**29**	3-KB-H21-R	100	EGFR	0.415804	0
**30**	3-KB-I20-N	10	EGFR	0.481125	0
**31**	3-KB-I21-R	10	EGFR	0.39245	0.008
**32**	3-KB-J20-N	1	EGFR	0.46116	0
**33**	3-KB-J21-R	1	EGFR	0.494191	0

**34**	3-KB-K4-Ca	1	EGFR	0.282686	0.011
**35**	3-KB-K18-D	0.1	EGFR	0.507462	0
**36**	3-KB-K20-N	0.1	EGFR	0.067209	1
**37**	3-KB-L4-Ca	10	EGFR	0.479035	0
**38**	3-KB-L18-D	1	EGFR	0.44867	0
**39**	3-KB-M18-I	10	EGFR	0.528717	0
**40**	3-KB-N4-Ca	100	EGFR	0.136542	0.953
**41**	3-KB-N18-D	100	EGFR	0.403564	0
**42**	3-KB-O4-Ca	1000	EGFR	0.27832	0.471
**43**	3-KB-P4-Ca	10000	EGFR	0.118713	1
**44**	3-KB-P18-D	1000	EGFR	0.368907	0.076
**45**	4-KB-F13-Sa	1000	EGFR	0.402014	0.009
**46**	4-KB-G13-S	100	EGFR	0.44605	0
**47**	4-KB-G16-V	10000	EGFR	0.295292	0.414
**48**	4-KB-H13-S	10	EGFR	0.521484	0
**49**	4-KB-H16-V	1000	EGFR	0.34745	0.082
**50**	4-KB-I13-Sa	1	EGFR	0.397385	0
**51**	4-KB-I16-Va	100	EGFR	0.404877	0.001
**52**	4-KB-J13-Sa	0.1	EGFR	0.476206	0
**53**	4-KB-J16-Va	10	EGFR	0.408569	0.001
**54**	4-KB-K7-Ico	1	EGFR	0.495045	0
**55**	4-KB-K13-T	0.1	EGFR	0.062507	0.92
**56**	4-KB-K16-V	1	EGFR	0.514947	0
**57**	4-KB-L7-Ico	10	EGFR	0.516787	0
**58**	4-KB-L13-To	1	EGFR	0.468685	0
**59**	4-KB-M7-Ic	100	EGFR	0.284135	0.079
**60**	4-KB-M13-T	10	EGFR	0.401307	0.009
**61**	4-KB-N13-T	100	EGFR	0.385498	0.007
**62**	4-KB-O7-Ico	1000	EGFR	0.421523	0
**63**	4-KB-P7-Ico	10000	EGFR	0.155813	0.828
**64**	4-KB-P13-T	1000	EGFR	0.271924	0.391
**65**	5-KB-F4-Po	1000	EGFR	0.39912	0.009
**66**	5-KB-F7-AZ	1000	EGFR	0.471865	0
**67**	5-KB-G4-Po	100	EGFR	0.394278	0.006
**68**	5-KB-G7-AZ	100	EGFR	0.366235	0.003

**69**	5-KB-H4-Pc	10	EGFR	0.56235	0
**70**	5-KB-H7-AZ	10	EGFR	0.32256	0.002
**71**	5-KB-I4-Po:	1	EGFR	0.24436	0.761
**72**	5-KB-I7-AZI	1	EGFR	0.269581	0.063
**73**	5-KB-J4-Po:	0.1	EGFR	0.279413	0.402
**74**	5-KB-J7-AZI	0.1	EGFR	0.07817	0.962
**75**	5-KB-K7-Ol	0.1	EGFR	0.083856	0.866
**76**	5-KB-L7-Ol	1	EGFR	0.066948	0.998
**77**	5-KB-M7-Ol	10	EGFR	0.188651	0.163
**78**	5-KB-O7-Ol	100	EGFR	0.181859	0.84
**79**	5-KB-P7-Ol	1000	EGFR	0.036486	1
**80**	2-KB-A15-L	2500	VEGFR	0.471037	0
**81**	2-KB-A17-N	10000	VEGFR	0.224207	0.838
**82**	2-KB-A20-T	10000	VEGFR	0.43895	0
**83**	2-KB-B15-L	250	VEGFR	0.246693	0.596
**84**	2-KB-B17-N	1000	VEGFR	0.475185	0
**85**	2-KB-B20-T	1000	VEGFR	0.402061	0.004
**86**	2-KB-C15-L	25	VEGFR	0.504206	0
**87**	2-KB-C17-N	100	VEGFR	0.508904	0
**88**	2-KB-D15-L	2.5	VEGFR	0.486605	0
**89**	2-KB-D17-N	10	VEGFR	0.51912	0
**90**	2-KB-D20-T	100	VEGFR	0.412381	0
**91**	2-KB-E17-N	1	VEGFR	0.38261	0.017
**92**	2-KB-E20-Ti	10	VEGFR	0.399678	0.005
**93**	2-KB-F13-A	10000	VEGFR	0.441579	0
**94**	2-KB-F15-L	0.25	VEGFR	0.429929	0
**95**	2-KB-F19-R	10000	VEGFR	0.22852	0.891
**96**	2-KB-F20-Ti	1	VEGFR	0.331119	0
**97**	2-KB-F21-V	10000	VEGFR	0.468096	0
**98**	2-KB-G10-A	10000	VEGFR	0.252847	0.333
**99**	2-KB-G13-A	1000	VEGFR	0.435604	0.005
**100**	2-KB-G19-R	1000	VEGFR	0.49694	0
**101**	2-KB-G21-V	1000	VEGFR	0.436542	0.001
**102**	2-KB-H10-A	1000	VEGFR	0.454179	0
**103**	2-KB-H13-A	100	VEGFR	0.517889	0

**104**	2-KB-H21-V	100	VEGFR	0.465173	0
**105**	2-KB-I10-Ap	100	VEGFR	0.030126	1
**106**	2-KB-I13-Ax	10	VEGFR	0.468253	0
**107**	2-KB-I19-Re	100	VEGFR	0.489277	0
**108**	2-KB-I21-Va	10	VEGFR	0.531237	0
**109**	2-KB-J10-Ap	10	VEGFR	0.462219	0
**110**	2-KB-J13-A:	1	VEGFR	0.392069	0
**111**	2-KB-J19-Re	10	VEGFR	0.478918	0
**112**	2-KB-J21-Va	1	VEGFR	0.4877	0
**113**	2-KB-K10-A	1	VEGFR	0.41101	0
**114**	2-KB-K13-V	0.1	VEGFR	0.445036	0
**115**	2-KB-K17-P	1	VEGFR	0.463505	0
**116**	2-KB-K19-R	1	VEGFR	0.508314	0
**117**	2-KB-L12-S	0.1	VEGFR	0.389677	0
**118**	2-KB-L13-V	1	VEGFR	0.387733	0
**119**	2-KB-L21-C	0.1	VEGFR	0.490825	0
**120**	2-KB-M12-S	1	VEGFR	0.420344	0
**121**	2-KB-M13-V	10	VEGFR	0.473486	0
**122**	2-KB-M17-P	10	VEGFR	0.512171	0
**123**	2-KB-M21-C	1	VEGFR	0.52653	0
**124**	2-KB-N12-S	10	VEGFR	0.208062	0.045
**125**	2-KB-N13-V	100	VEGFR	0.432641	0
**126**	2-KB-N17-P	100	VEGFR	0.476766	0
**127**	2-KB-N21-C	10	VEGFR	0.502315	0
**128**	2-KB-O12-S	100	VEGFR	0.504405	0
**129**	2-KB-O17-P	1000	VEGFR	0.490793	0
**130**	2-KB-O21-C	100	VEGFR	0.528038	0
**131**	2-KB-P12-S	1000	VEGFR	0.316249	0.042
**132**	2-KB-P13-V	1000	VEGFR	0.34265	0.024
**133**	2-KB-P17-P	10000	VEGFR	0.488309	0
**134**	2-KB-P21-C	1000	VEGFR	0.465337	0
**135**	3-KB-A3-Ca	1000	VEGFR	0.411577	0
**136**	3-KB-A6-Fo	1000	VEGFR	0.341569	0.276
**137**	3-KB-A18-L	1000	VEGFR	0.180283	0.681
**138**	3-KB-B3-Ca	100	VEGFR	0.132156	0.841

**139**	3-KB-B6-Fo	100	VEGFR	0.230294	0.428
**140**	3-KB-B18-L	100	VEGFR	0.22529	0.414
**141**	3-KB-C3-Ca	10	VEGFR	0.208191	0.495
**142**	3-KB-C6-Fo	10	VEGFR	0.097725	0.892
**143**	3-KB-C18-L	10	VEGFR	0.286378	0.121
**144**	3-KB-D3-Ca	1	VEGFR	0.156931	0.823
**145**	3-KB-D6-Fo	1	VEGFR	0.080644	0.963
**146**	3-KB-D18-L	1	VEGFR	0.182033	0.797
**147**	3-KB-E3-Cal	0.1	VEGFR	0.085605	0.907
**148**	3-KB-E6-For	0.1	VEGFR	0.066374	0.97
**149**	3-KB-E18-Li	0.1	VEGFR	0.346496	0.002
**150**	3-KB-F18-B	1000	VEGFR	0.320709	0.129
**151**	3-KB-G18-E	100	VEGFR	0.427811	0
**152**	3-KB-H18-E	10	VEGFR	0.334415	0.001
**153**	3-KB-I18-Br	1	VEGFR	0.410875	0
**154**	3-KB-J18-Br	0.1	VEGFR	0.427536	0
**155**	4-KB-A12-E	10000	VEGFR	0.205803	0.653
**156**	4-KB-A15-G	2500	VEGFR	0.213163	0.441
**157**	4-KB-A20-N	10000	VEGFR	0.215667	0.409
**158**	4-KB-B12-E	1000	VEGFR	0.196022	0.853
**159**	4-KB-B15-G	250	VEGFR	0.263361	0.289
**160**	4-KB-B20-N	1000	VEGFR	0.209844	0.562
**161**	4-KB-C15-G	25	VEGFR	0.163635	0.608
**162**	4-KB-D12-E	100	VEGFR	0.400549	0
**163**	4-KB-D15-G	2.5	VEGFR	0.308129	0.127
**164**	4-KB-D20-N	100	VEGFR	0.46645	0.002
**165**	4-KB-E12-El	10	VEGFR	0.067312	0.915
**166**	4-KB-E20-M	10	VEGFR	0.242665	0.402
**167**	4-KB-F12-El	1	VEGFR	0.089162	0.924
**168**	4-KB-F15-G	0.25	VEGFR	0.40176	0
**169**	4-KB-F20-M	1	VEGFR	0.196689	0.336
**170**	4-KB-L16-T	1	VEGFR	0.240635	0.178
**171**	4-KB-M16-T	10	VEGFR	0.183451	0.218
**172**	4-KB-N16-T	100	VEGFR	0.28703	0.023
**173**	4-KB-O16-T	1000	VEGFR	0.359226	0.029

**174**	4-KB-P16-T	10000	VEGFR	0.318225	0.07
**175**	2-KB-L10-Ic	1	PI3K	0.22883	0.094
**176**	2-KB-M10-I	10	PI3K	0.253551	0.163
**177**	2-KB-N10-I	100	PI3K	0.421212	0
**178**	2-KB-O10-I	1000	PI3K	0.458069	0
**179**	2-KB-P10-Ic	10000	PI3K	0.422018	0
**180**	3-KB-A16-P	2500	PI3K	0.045905	0.996
**181**	3-KB-C16-P	250	PI3K	0.128721	0.443
**182**	3-KB-D16-F	25	PI3K	0.088098	0.995
**183**	3-KB-E16-P	2.5	PI3K	0.060835	0.978
**184**	3-KB-F16-P	0.25	PI3K	0.366654	0.127
**185**	3-KB-F17-M	100000	PI3K	0.048441	0.945
**186**	3-KB-F19-D	500	PI3K	0.056135	0.962
**187**	3-KB-G17-N	10000	PI3K	0.366931	0
**188**	3-KB-G19-D	50	PI3K	0.461671	0
**189**	3-KB-H17-N	1000	PI3K	0.257335	0.512
**190**	3-KB-I17-M	100	PI3K	0.074403	0.865
**191**	3-KB-I19-D	5	PI3K	0.31531	0.001
**192**	3-KB-J17-M	10	PI3K	0.416544	0
**193**	3-KB-J19-D	0.5	PI3K	0.402815	0
**194**	3-KB-K19-D	0.05	PI3K	0.302979	0.013
**195**	3-KB-L8-Pic	1	PI3K	0.24215	0.016
**196**	3-KB-L21-T	0.1	PI3K	0.061521	0.985
**197**	3-KB-M8-Pi	10	PI3K	0.346489	0
**198**	3-KB-M21-T	1	PI3K	0.182351	0.703
**199**	3-KB-N8-Pi	100	PI3K	0.342122	0.062
**200**	3-KB-N21-T	10	PI3K	0.207971	0.665
**201**	3-KB-O8-Pi	1000	PI3K	0.394767	0.003
**202**	3-KB-O21-T	100	PI3K	0.181799	0.895
**203**	3-KB-P8-Pic	10000	PI3K	0.37939	0.069
**204**	3-KB-P21-T	1000	PI3K	0.171067	0.213
**205**	4-KB-A19-A	2500	PI3K	0.102503	0.979
**206**	4-KB-B19-A	250	PI3K	0.136721	0.925
**207**	4-KB-C19-A	25	PI3K	0.135891	0.995
**208**	4-KB-D19-A	2.5	PI3K	0.173051	0.916

**209**	4-KB-E19-A	0.25	PI3K	0.283833	0.01
**210**	4-KB-F14-N	1000	PI3K	0.061461	1
**211**	4-KB-G2-TG	2500	PI3K	0.144864	0.956
**212**	4-KB-G5-So	10000	PI3K	0.439026	0
**213**	4-KB-G14-N	100	PI3K	0.524328	0
**214**	4-KB-G20-E	10000	PI3K	0.275719	0.385
**215**	4-KB-H2-TG	250	PI3K	0.084941	0.981
**216**	4-KB-H5-So	1000	PI3K	0.431463	0
**217**	4-KB-H14-N	10	PI3K	0.419711	0
**218**	4-KB-H20-E	1000	PI3K	0.508852	0
**219**	4-KB-I2-TGI	25	PI3K	0.288008	0.23
**220**	4-KB-I5-Sor	100	PI3K	0.345968	0.001
**221**	4-KB-I14-N'	1	PI3K	0.382342	0
**222**	4-KB-I20-Bu	100	PI3K	0.417624	0
**223**	4-KB-J2-TGI	2.5	PI3K	0.058756	0.988
**224**	4-KB-J5-Sor	10	PI3K	0.056906	0.945
**225**	4-KB-J20-Bu	10	PI3K	0.266493	0.013
**226**	4-KB-K2-TG	0.25	PI3K	0.130495	0.957
**227**	4-KB-K4-Da	0.1	PI3K	0.074045	0.983
**228**	4-KB-K5-So	1	PI3K	0.275118	0.029
**229**	4-KB-K14-N	0.1	PI3K	0.132875	0.734
**230**	4-KB-K20-B	1	PI3K	0.369514	0
**231**	4-KB-L4-Da	1	PI3K	0.287401	0.21
**232**	4-KB-L14-G	0.1	PI3K	0.115297	0.748
**233**	4-KB-L15-T	1	PI3K	0.365664	0
**234**	4-KB-L21-C	0.1	PI3K	0.434662	0
**235**	4-KB-M14-(	1	PI3K	0.276045	0.183
**236**	4-KB-M15-T	10	PI3K	0.319332	0
**237**	4-KB-M21-(	1	PI3K	0.403257	0
**238**	4-KB-N4-Da	10	PI3K	0.516511	0
**239**	4-KB-N14-G	10	PI3K	0.494213	0
**240**	4-KB-N15-T	100	PI3K	0.083344	0.962
**241**	4-KB-N21-C	10	PI3K	0.369933	0.001
**242**	4-KB-O4-Da	100	PI3K	0.44482	0
**243**	4-KB-O14-G	100	PI3K	0.43798	0

**244**	4-KB-O15-T	1000	PI3K	0.407836	0
**245**	4-KB-O21-C	100	PI3K	0.546582	0
**246**	4-KB-P4-Da	1000	PI3K	0.432293	0
**247**	4-KB-P14-G	1000	PI3K	0.488851	0
**248**	4-KB-P15-T	10000	PI3K	0.371644	0.002
**249**	4-KB-P21-C	1000	PI3K	0.502871	0
**250**	5-KB-A6-LY	2500	PI3K	0.525417	0
**251**	5-KB-A7-AN	1000	PI3K	0.30192	0.107
**252**	5-KB-A16-A	2500	PI3K	0.457776	0
**253**	5-KB-A17-P	10000	PI3K	0.426192	0
**254**	5-KB-B6-LY	250	PI3K	0.435093	0
**255**	5-KB-B7-AN	100	PI3K	0.108768	0.972
**256**	5-KB-B17-P	1000	PI3K	0.195969	0.219
**257**	5-KB-C6-LY	25	PI3K	0.473138	0
**258**	5-KB-C7-AN	10	PI3K	0.126487	0.717
**259**	5-KB-C16-A	250	PI3K	0.468645	0
**260**	5-KB-C17-P	100	PI3K	0.422201	0
**261**	5-KB-D6-LY	2.5	PI3K	0.16898	0.942
**262**	5-KB-D7-AN	1	PI3K	0.353244	0.001
**263**	5-KB-D16-A	25	PI3K	0.470915	0
**264**	5-KB-D17-P	10	PI3K	0.305075	0.007
**265**	5-KB-E6-LY	0.25	PI3K	0.176862	0.873
**266**	5-KB-E7-AN	0.1	PI3K	0.136918	0.667
**267**	5-KB-E16-A	2.5	PI3K	0.286871	0
**268**	5-KB-E17-P	1	PI3K	0.363577	0
**269**	5-KB-F11-G	10000	PI3K	0.475993	0
**270**	5-KB-F16-A	0.25	PI3K	0.297777	0
**271**	5-KB-G9-Se	10000	PI3K	0.51688	0
**272**	5-KB-G11-G	1000	PI3K	0.405882	0
**273**	5-KB-H9-Se	1000	PI3K	0.444031	0
**274**	5-KB-H11-G	100	PI3K	0.46753	0
**275**	5-KB-I9-Ser	100	PI3K	0.323665	0
**276**	5-KB-I11-G	10	PI3K	0.168619	0.112
**277**	5-KB-J9-Ser	10	PI3K	0.295246	0
**278**	5-KB-J11-G	1	PI3K	0.246699	0.006

**279**	5-KB-K9-Se	1	PI3K	0.239402	0.007
**280**	5-KB-L14-A	0.1	PI3K	0.099283	0.582
**281**	5-KB-L20-Z	1	PI3K	0.343889	0.018
**282**	5-KB-L23-O	0.1	PI3K	0.101395	0.981
**283**	5-KB-M14-/	1	PI3K	0.458275	0
**284**	5-KB-M20-Z	10	PI3K	0.395202	0
**285**	5-KB-M23-(	1	PI3K	0.402177	0
**286**	5-KB-N14-A	10	PI3K	0.379167	0.002
**287**	5-KB-N20-Z	100	PI3K	0.359556	0.003
**288**	5-KB-N23-C	10	PI3K	0.569619	0
**289**	5-KB-O14-A	100	PI3K	0.439019	0
**290**	5-KB-O20-Z	1000	PI3K	0.477641	0
**291**	5-KB-O23-C	100	PI3K	0.53139	0
**292**	5-KB-P14-A	1000	PI3K	0.383311	0
**293**	5-KB-P20-Z	10000	PI3K	0.546298	0
**294**	5-KB-P23-C	1000	PI3K	0.465081	0.002
**295**	6-KB-A8-TG	10000	PI3K	0.523432	0
**296**	6-KB-B8-TG	1000	PI3K	0.379729	0
**297**	6-KB-C8-TG	100	PI3K	0.466335	0
**298**	6-KB-D8-TG	10	PI3K	0.384311	0
**299**	6-KB-E8-TG	1	PI3K	0.146936	0.702
**300**	6-KB-L6-GD	1	PI3K	0.220789	0.568
**301**	6-KB-M6-GI	10	PI3K	0.06846	0.834
**302**	6-KB-N6-GI	100	PI3K	0.409087	0
**303**	6-KB-O6-GI	1000	PI3K	0.532672	0
**304**	6-KB-P6-GD	10000	PI3K	0.343236	0.046
**305**	1-KB-F11-A	10000	Topoisome	0.504824	0
**306**	1-KB-G11-A	1000	Topoisome	0.452213	0.019
**307**	1-KB-G20-E	1000	Topoisome	0.350448	0.294
**308**	1-KB-H11-A	100	Topoisome	0.483012	0.002
**309**	1-KB-H20-E	100	Topoisome	0.462817	0.008
**310**	1-KB-I11-Ar	10	Topoisome	0.27622	0.381
**311**	1-KB-I20-Ep	10	Topoisome	0.491065	0.003
**312**	1-KB-J11-Ar	1	Topoisome	0.406267	0.009
**313**	1-KB-J20-Ep	1	Topoisome	0.297542	0.167

**314**	1-KB-K11-S	1	Topoisome	0.594817	0
**315**	1-KB-K20-E	0.1	Topoisome	0.061942	1
**316**	1-KB-L11-Sl	10	Topoisome	0.486044	0.002
**317**	1-KB-L14-T	1	Topoisome	0.448692	0
**318**	1-KB-M11-S	100	Topoisome	0.427237	0.076
**319**	1-KB-M14-T	10	Topoisome	0.398076	0.072
**320**	1-KB-N14-T	100	Topoisome	0.444349	0.011
**321**	1-KB-O11-S	1000	Topoisome	0.344367	0.334
**322**	1-KB-O14-T	1000	Topoisome	0.443649	0.042
**323**	1-KB-P11-S	10000	Topoisome	0.34996	0.307
**324**	1-KB-P14-T	10000	Topoisome	0.352511	0.299
**325**	3-KB-A11-E	10000	Topoisome	0.512402	0
**326**	3-KB-B11-E	1000	Topoisome	0.547566	0
**327**	3-KB-C11-E	100	Topoisome	0.181065	0.909
**328**	3-KB-D11-E	10	Topoisome	0.39298	0.001
**329**	3-KB-E11-E	1	Topoisome	0.467613	0
**330**	3-KB-G9-Da	1000	Topoisome	0.447876	0
**331**	3-KB-G10-T	10000	Topoisome	0.466511	0
**332**	3-KB-H9-Da	100	Topoisome	0.417299	0
**333**	3-KB-H10-T	1000	Topoisome	0.466114	0
**334**	3-KB-I9-Dau	10	Topoisome	0.532173	0
**335**	3-KB-I10-Te	100	Topoisome	0.428588	0
**336**	3-KB-J9-Dau	1	Topoisome	0.292241	0.243
**337**	3-KB-J10-Te	10	Topoisome	0.452228	0.001
**338**	3-KB-K7-Ida	0.1	Topoisome	0.476877	0
**339**	3-KB-K9-Da	0.1	Topoisome	0.52852	0
**340**	3-KB-K10-T	1	Topoisome	0.331975	0.029
**341**	3-KB-L6-Do	0.1	Topoisome	0.475062	0
**342**	3-KB-L7-Ida	1	Topoisome	0.517649	0
**343**	3-KB-L9-Val	0.5	Topoisome	0.076999	0.903
**344**	3-KB-L10-M	0.1	Topoisome	0.502295	0
**345**	3-KB-L16-P	1	Topoisome	0.471344	0
**346**	3-KB-M6-Do	1	Topoisome	0.617565	0
**347**	3-KB-M7-Id	10	Topoisome	0.207963	0.201
**348**	3-KB-M9-Va	5	Topoisome	0.495854	0

**349**	3-KB-M10-I	1	Topoisome	0.153813	0.404
**350**	3-KB-M16-I	10	Topoisome	0.556099	0
**351**	3-KB-N6-Dc	10	Topoisome	0.40771	0.042
**352**	3-KB-N9-Va	50	Topoisome	0.517754	0
**353**	3-KB-N10-N	10	Topoisome	0.212805	0.916
**354**	3-KB-N16-F	100	Topoisome	0.405059	0.008
**355**	3-KB-O6-Dc	100	Topoisome	0.541283	0
**356**	3-KB-O7-Ida	100	Topoisome	0.319829	0.372
**357**	3-KB-O9-Va	500	Topoisome	0.542629	0
**358**	3-KB-O10-N	100	Topoisome	0.278866	0.012
**359**	3-KB-O16-F	1000	Topoisome	0.511184	0
**360**	3-KB-P6-Dc	1000	Topoisome	0.327188	0.493
**361**	3-KB-P7-Ida	1000	Topoisome	0.341886	0.448
**362**	3-KB-P9-Va	5000	Topoisome	0.374568	0.312
**363**	3-KB-P10-N	1000	Topoisome	0.520342	0.007
**364**	3-KB-P16-P	10000	Topoisome	0.548352	0
**365**	1-KB-A10-V	10000	Mitotic	0.708422	0
**366**	1-KB-A13-I	1000	Mitotic	0.71694	0
**367**	1-KB-A18-P	1000	Mitotic	0.708179	0
**368**	1-KB-B10-V	1000	Mitotic	0.705834	0
**369**	1-KB-B13-I	100	Mitotic	0.494226	0.01
**370**	1-KB-B18-P	100	Mitotic	0.714467	0
**371**	1-KB-C10-V	100	Mitotic	0.70977	0
**372**	1-KB-C13-I	10	Mitotic	0.239437	0.552
**373**	1-KB-C18-P	10	Mitotic	0.698536	0
**374**	1-KB-D10-V	10	Mitotic	0.648276	0
**375**	1-KB-D13-I	1	Mitotic	0.617524	0
**376**	1-KB-D18-F	1	Mitotic	0.570552	0
**377**	1-KB-E10-V	1	Mitotic	0.648348	0
**378**	1-KB-E13-I	0.1	Mitotic	0.717659	0
**379**	1-KB-E18-P	0.1	Mitotic	0.262941	0.242
**380**	1-KB-F13-V	1000	Mitotic	0.705938	0
**381**	1-KB-G13-V	100	Mitotic	0.531961	0
**382**	1-KB-G15-E	1000	Mitotic	0.708154	0
**383**	1-KB-H13-V	10	Mitotic	0.484091	0.005

**384**	1-KB-H15-E	100	Mitotic	0.70898	0
**385**	1-KB-I13-Vi	1	Mitotic	0.447814	0.006
**386**	1-KB-I15-Er	10	Mitotic	0.721809	0
**387**	1-KB-J13-Vi	0.1	Mitotic	0.3698	0.182
**388**	1-KB-J15-Er	1	Mitotic	0.698966	0
**389**	1-KB-K7-Vir	0.1	Mitotic	0.455161	0.001
**390**	1-KB-K15-E	0.1	Mitotic	0.504262	0.001
**391**	1-KB-L7-Vir	1	Mitotic	0.41591	0.021
**392**	1-KB-L20-V	0.1	Mitotic	0.237906	0.263
**393**	1-KB-M7-Vi	10	Mitotic	0.548079	0
**394**	1-KB-M20-\	1	Mitotic	0.44774	0.016
**395**	1-KB-N20-\	10	Mitotic	0.251887	0.441
**396**	1-KB-O7-Vi	100	Mitotic	0.619379	0
**397**	1-KB-O20-\	100	Mitotic	0.340469	0.022
**398**	1-KB-P7-Vir	1000	Mitotic	0.713083	0
**399**	1-KB-P20-V	1000	Mitotic	0.707438	0
**400**	3-KB-A7-Dc	1000	Mitotic	0.697183	0
**401**	3-KB-B7-Dc	100	Mitotic	0.690877	0
**402**	3-KB-C7-Dc	10	Mitotic	0.688423	0
**403**	3-KB-D7-Dc	1	Mitotic	0.365125	0.106
**404**	3-KB-E7-Do	0.1	Mitotic	0.110075	0.876
**405**	6-KB-L19-A	1	Mitotic	0.204458	0.815
**406**	6-KB-M19-/	10	Mitotic	0.413826	0.03
**407**	6-KB-N19-A	100	Mitotic	0.307764	0.428
**408**	6-KB-O19-A	1000	Mitotic	0.678639	0
**409**	6-KB-P19-A	10000	Mitotic	0.668527	0
**410**	2-KB-A12-T	250	MEK1/2	0.598239	0
**411**	2-KB-B12-T	25	MEK1/2	0.668638	0
**412**	2-KB-D12-T	2.5	MEK1/2	0.52574	0.002
**413**	2-KB-E12-Tı	0.25	MEK1/2	0.245787	0.393
**414**	2-KB-F12-Tı	2.5000000	MEK1/2	0.510929	0.017
**415**	2-KB-F14-C	1000	MEK1/2	0.605413	0
**416**	2-KB-G14-C	100	MEK1/2	0.587974	0
**417**	2-KB-H14-C	10	MEK1/2	0.593995	0
**418**	2-KB-I14-Cc	1	MEK1/2	0.224921	0.38

**419**	2-KB-K14-C	0.1	MEK1/2	0.258858	0.274
**420**	2-KB-L20-S	1	MEK1/2	0.583772	0
**421**	2-KB-M20-S	10	MEK1/2	0.587336	0.001
**422**	2-KB-N20-S	100	MEK1/2	0.595535	0
**423**	2-KB-O20-S	1000	MEK1/2	0.64177	0
**424**	2-KB-P20-S	10000	MEK1/2	0.653073	0
**425**	4-KB-A10-B	1000	MEK1/2	0.416269	0.091
**426**	4-KB-A13-P	1000	MEK1/2	0.503431	0.015
**427**	4-KB-B10-B	100	MEK1/2	0.42428	0.104
**428**	4-KB-B13-P	100	MEK1/2	0.510928	0.011
**429**	4-KB-C10-B	10	MEK1/2	0.386677	0.188
**430**	4-KB-C13-P	10	MEK1/2	0.471025	0.029
**431**	4-KB-D10-E	1	MEK1/2	0.180559	0.909
**432**	4-KB-D13-F	1	MEK1/2	0.329367	0.293
**433**	4-KB-E10-B	0.1	MEK1/2	0.526074	0.009
**434**	4-KB-E13-P	0.1	MEK1/2	0.520783	0.003
**435**	4-KB-L19-G	0.25	MEK1/2	0.458538	0.029
**436**	4-KB-M19-C	2.5	MEK1/2	0.201651	0.72
**437**	4-KB-N19-C	25	MEK1/2	0.503025	0.01
**438**	4-KB-O19-C	250	MEK1/2	0.084852	0.979
**439**	4-KB-P19-G	2500	MEK1/2	0.501242	0.009
**440**	1-KB-L2-Ola	1	PARP	0.524	0.003
**441**	1-KB-L6-Ru	1	PARP	0.382471	0.435
**442**	1-KB-M2-Ol	10	PARP	0.447933	0.044
**443**	1-KB-M6-Ru	10	PARP	0.466361	0.043
**444**	1-KB-N2-Ol	100	PARP	0.536712	0.001
**445**	1-KB-N6-Ru	100	PARP	0.200734	0.827
**446**	1-KB-O2-Ol	1000	PARP	0.328356	0.424
**447**	1-KB-O6-Ru	1000	PARP	0.392306	0.284
**448**	1-KB-P2-Ola	10000	PARP	0.583359	0.006
**449**	1-KB-P6-Ru	10000	PARP	0.549447	0.011
**450**	7-KB-A3-Tal	1000	PARP	0.54779	0.008
**451**	7-KB-B2-Ve	10000	PARP	0.368456	0.321
**452**	7-KB-B3-Tal	100	PARP	0.464263	0.054
**453**	7-KB-C2-Ve	1000	PARP	0.530702	0.008

**454**	7-KB-C3-Tal	10	PARP	0.342795	0.284
**455**	7-KB-D2-Ve	100	PARP	0.540881	0.001
**456**	7-KB-D3-Ta	1	PARP	0.539426	0.004
**457**	7-KB-E2-Vel	10	PARP	0.354957	0.122
**458**	7-KB-E3-Tal	0.1	PARP	0.515992	0.037
**459**	7-KB-F2-Vel	1	PARP	0.464434	0.021
**460**	7-KB-G2-Ni	10000	PARP	0.530067	0.02
**461**	7-KB-H2-Ni	1000	PARP	0.386186	0.209
**462**	7-KB-I2-Nir	100	PARP	0.578192	0.001
**463**	7-KB-J2-Nir	10	PARP	0.521236	0.002
**464**	7-KB-K2-Ni	1	PARP	0.554969	0
**465**	3-KB-A19-D	1000	CDK	0.581723	0
**466**	3-KB-B19-D	100	CDK	0.581397	0
**467**	3-KB-B23-A	2500	CDK	0.317264	0.318
**468**	3-KB-C19-D	10	CDK	0.478224	0.002
**469**	3-KB-C23-A	250	CDK	0.441444	0.005
**470**	3-KB-D19-D	1	CDK	0.281666	0.219
**471**	3-KB-D23-A	25	CDK	0.380029	0.019
**472**	3-KB-E19-D	0.1	CDK	0.443876	0.006
**473**	3-KB-E23-A	2.5	CDK	0.344597	0.127
**474**	3-KB-F23-A	0.25	CDK	0.052484	0.99
**475**	3-KB-K17-P	1	CDK	0.360429	0.048
**476**	3-KB-L19-R	1	CDK	0.453902	0
**477**	3-KB-M17-I	10	CDK	0.423027	0.009
**478**	3-KB-M19-I	10	CDK	0.423768	0.008
**479**	3-KB-N17-F	100	CDK	0.37568	0.033
**480**	3-KB-N19-R	100	CDK	0.327979	0.027
**481**	3-KB-O17-F	1000	CDK	0.422553	0.04
**482**	3-KB-O19-R	1000	CDK	0.391775	0.014
**483**	3-KB-P17-P	10000	CDK	0.48078	0
**484**	3-KB-P19-R	10000	CDK	0.408268	0.012
**485**	4-KB-A4-SN	10000	CDK	0.526358	0
**486**	4-KB-A8-Mi	10000	CDK	0.526291	0.001
**487**	4-KB-B4-SN	1000	CDK	0.540206	0
**488**	4-KB-B8-Mi	1000	CDK	0.51274	0.001

**489**	4-KB-C4-SN	100	CDK	0.508938	0
**490**	4-KB-C8-Mi	100	CDK	0.246197	0.536
**491**	4-KB-D4-SN	10	CDK	0.323032	0.115
**492**	4-KB-D8-Mi	10	CDK	0.265218	0.251
**493**	4-KB-E4-SN	1	CDK	0.071629	0.994
**494**	4-KB-E8-Mi	1	CDK	0.247808	0.185
**495**	4-KB-F4-Sel	10000	CDK	0.403385	0.045
**496**	4-KB-F22-A	10000	CDK	0.541389	0
**497**	4-KB-G4-Se	1000	CDK	0.315461	0.35
**498**	4-KB-G22-A	1000	CDK	0.550631	0
**499**	4-KB-H4-Se	100	CDK	0.167088	0.816
**500**	4-KB-H22-A	100	CDK	0.375062	0.057
**501**	4-KB-I4-Seli	10	CDK	0.088239	0.884
**502**	4-KB-I22-Al	10	CDK	0.426671	0.014
**503**	4-KB-J4-Sel	1	CDK	0.115289	0.802
**504**	4-KB-J22-Al	1	CDK	0.404213	0.03
**505**	5-KB-A19-A	10000	CDK	0.562295	0
**506**	5-KB-B19-A	1000	CDK	0.507658	0.001
**507**	5-KB-C19-A	100	CDK	0.334537	0.286
**508**	5-KB-D19-A	10	CDK	0.126556	0.765
**509**	5-KB-E19-A	1	CDK	0.218024	0.605
**510**	5-KB-K17-A	1	CDK	0.250098	0.267
**511**	5-KB-M17-A	10	CDK	0.134694	0.965
**512**	5-KB-N17-A	100	CDK	0.359113	0.104
**513**	5-KB-O17-A	1000	CDK	0.561514	0
**514**	5-KB-P17-A	10000	CDK	0.558986	0
**515**	6-KB-A17-S	1000	CDK	0.425605	0.006
**516**	6-KB-B17-S	100	CDK	0.397499	0.025
**517**	6-KB-C17-S	10	CDK	0.413702	0.028
**518**	6-KB-D17-S	1	CDK	0.06958	0.965
**519**	6-KB-E17-S	0.1	CDK	0.285126	0.153
**520**	6-KB-L15-T	1	CDK	0.140685	0.904
**521**	6-KB-M15-	10	CDK	0.402002	0.008
**522**	6-KB-N15-T	100	CDK	0.479288	0.008
**523**	6-KB-O15-T	1000	CDK	0.55324	0

**524**	6-KB-P15-T 10000	CDK	0.544646	0
**525**	7-KB-A21-d 10000	BET	0.649498	0
**526**	7-KB-A22-P 30000	BET	0.703647	0
**527**	7-KB-B21-d 1000	BET	0.753413	0
**528**	7-KB-B22-P 3000	BET	0.732272	0
**529**	7-KB-C21-d 100	BET	0.689149	0
**530**	7-KB-C22-P 300	BET	0.744921	0
**531**	7-KB-D21-c 10	BET	0.687303	0
**532**	7-KB-D22-F 30	BET	0.403182	0.013
**533**	7-KB-E21-d 1	BET	0.624274	0
**534**	7-KB-E22-P 3	BET	0.464081	0.003
**535**	7-KB-G10-E 10000	BET	0.696056	0
**536**	7-KB-G15-I- 10000	BET	0.660922	0
**537**	7-KB-H10-E 1000	BET	0.698998	0
**538**	7-KB-H15-I- 1000	BET	0.691075	0
**539**	7-KB-I10-Bi 100	BET	0.678007	0
**540**	7-KB-I15-I-I 100	BET	0.404339	0.003
**541**	7-KB-J10-Bi 10	BET	0.481801	0
**542**	7-KB-J15-I-I 10	BET	0.643766	0
**543**	7-KB-K10-B 1	BET	0.187432	0.422
**544**	7-KB-K13-N 1	BET	0.650643	0
**545**	7-KB-K15-I- 1	BET	0.240201	0.471
**546**	7-KB-L12-N 1	BET	0.802999	0
**547**	7-KB-L13-N 10	BET	0.708692	0
**548**	7-KB-L20-J(1	BET	0.441246	0.011
**549**	7-KB-L23-A 0.03	BET	0.499029	0.001
**550**	7-KB-M12-I 10	BET	0.564724	0
**551**	7-KB-M13-I 100	BET	0.699553	0
**552**	7-KB-M20-J 10	BET	0.472852	0.019
**553**	7-KB-M23-/ 0.3	BET	0.457136	0.005
**554**	7-KB-N12-N 100	BET	0.65734	0
**555**	7-KB-N13-N 1000	BET	0.665135	0
**556**	7-KB-N20-J 100	BET	0.736805	0
**557**	7-KB-N23-A 3	BET	0.702436	0
**558**	7-KB-O12-N 1000	BET	0.705551	0

**559**	7-KB-O20-J	1000	BET	0.694394	0
**560**	7-KB-O23-A	30	BET	0.766179	0
**561**	7-KB-P12-N	10000	BET	0.700858	0
**562**	7-KB-P13-N	10000	BET	0.675716	0
**563**	7-KB-P20-J	10000	BET	0.702555	0
**564**	7-KB-P23-A	300	BET	0.693726	0
**565**	8-KB-K22-C	1	BET	0.128304	0.952
**566**	8-KB-L22-C	10	BET	0.217025	0.852
**567**	8-KB-M22-(	100	BET	0.53289	0
**568**	8-KB-N22-C	1000	BET	0.305345	0.342
**569**	8-KB-O22-C	10000	BET	0.430795	0.014
**570**	1-KB-A3-Vo	10000	HDAC	0.31927	0.278
**571**	1-KB-B3-Vo	1000	HDAC	0.37816	0.019
**572**	1-KB-C3-Vo	100	HDAC	0.15369	0.449
**573**	1-KB-D3-Vc	10	HDAC	0.15659	0.767
**574**	1-KB-E3-Vo	1	HDAC	0.34901	0.086
**575**	1-KB-L12-R	0.1	HDAC	0.302543	0.059
**576**	1-KB-M12-I	1	HDAC	0.481347	0
**577**	1-KB-N12-F	10	HDAC	0.465077	0.001
**578**	1-KB-O12-F	100	HDAC	0.355301	0.082
**579**	1-KB-P12-R	1000	HDAC	0.341843	0.116
**580**	3-KB-A4-Pa	1000	HDAC	0.438598	0.005
**581**	3-KB-B4-Pa	100	HDAC	0.531866	0
**582**	3-KB-C4-Pa	10	HDAC	0.427284	0.001
**583**	3-KB-D4-Pa	1	HDAC	0.388259	0.001
**584**	3-KB-E4-Pa	0.1	HDAC	0.061818	0.989
**585**	3-KB-F7-Qu	1000	HDAC	0.396008	0.021
**586**	3-KB-G7-Qu	100	HDAC	0.204553	0.151
**587**	3-KB-G12-V	1000000	HDAC	0.140109	0.794
**588**	3-KB-H7-Qu	10	HDAC	0.1192	0.691
**589**	3-KB-H12-V	100000	HDAC	0.112054	0.606
**590**	3-KB-I7-Qui	1	HDAC	0.202536	0.235
**591**	3-KB-I12-Va	10000	HDAC	0.288935	0.039
**592**	3-KB-J7-Qu	0.1	HDAC	0.065564	0.999
**593**	3-KB-J12-Va	1000	HDAC	0.130879	0.909

**594**	3-KB-K3-Be	1	HDAC	0.150338	0.68
**595**	3-KB-K12-V	100	HDAC	0.249909	0.006
**596**	3-KB-L3-Bel	10	HDAC	0.260572	0.021
**597**	3-KB-M3-Be	100	HDAC	0.281355	0.253
**598**	3-KB-N3-Be	1000	HDAC	0.076535	0.996
**599**	3-KB-O3-Be	10000	HDAC	0.192466	0.861
**600**	7-KB-A5-Mc	10000	HDAC	0.454357	0.006
**601**	7-KB-A7-CU	10000	HDAC	0.2807	0.29
**602**	7-KB-A9-Gi	1000	HDAC	0.552568	0
**603**	7-KB-A12-R	10000	HDAC	0.533008	0
**604**	7-KB-B5-Mc	1000	HDAC	0.547367	0
**605**	7-KB-B7-CU	1000	HDAC	0.355521	0.065
**606**	7-KB-B12-R	1000	HDAC	0.471932	0
**607**	7-KB-C5-Mc	100	HDAC	0.398395	0
**608**	7-KB-C7-CU	100	HDAC	0.464257	0.001
**609**	7-KB-C9-Gi	100	HDAC	0.414214	0.002
**610**	7-KB-D7-CU	10	HDAC	0.43681	0
**611**	7-KB-D9-Gi	10	HDAC	0.373107	0
**612**	7-KB-D12-F	100	HDAC	0.376177	0
**613**	7-KB-E5-Mc	10	HDAC	0.367168	0.044
**614**	7-KB-E7-CU	1	HDAC	0.431316	0.001
**615**	7-KB-E9-Giv	1	HDAC	0.19539	0.654
**616**	7-KB-E12-R	10	HDAC	0.287403	0.04
**617**	7-KB-F5-Mc	1	HDAC	0.129808	0.995
**618**	7-KB-F7-Res	10000	HDAC	0.439462	0.003
**619**	7-KB-F9-Giv	0.1	HDAC	0.226214	0.111
**620**	7-KB-F12-R	1	HDAC	0.141664	0.559
**621**	7-KB-F19-P	10000	HDAC	0.337292	0.194
**622**	7-KB-G7-Re	1000	HDAC	0.422953	0
**623**	7-KB-G19-F	1000	HDAC	0.167041	0.942
**624**	7-KB-H7-Re	100	HDAC	0.471607	0
**625**	7-KB-I7-Res	10	HDAC	0.334747	0
**626**	7-KB-I19-P(	100	HDAC	0.157105	0.663
**627**	7-KB-J7-Res	1	HDAC	0.463621	0
**628**	7-KB-J19-P	10	HDAC	0.237851	0.408

**629**	7-KB-K4-En	1	HDAC	0.381854	0
**630**	7-KB-K11-A	1	HDAC	0.328773	0
**631**	7-KB-K18-T	1	HDAC	0.19959	0.731
**632**	7-KB-K19-P	1	HDAC	0.206057	0.381
**633**	7-KB-L2-Ta	0.1	HDAC	0.115872	0.983
**634**	7-KB-L4-En	10	HDAC	0.182163	0.551
**635**	7-KB-L5-Pra	1	HDAC	0.332415	0
**636**	7-KB-L8-Ab	1	HDAC	0.222411	0.89
**637**	7-KB-L10-T	1	HDAC	0.462364	0
**638**	7-KB-L11-A	10	HDAC	0.398644	0
**639**	7-KB-L14-T	1	HDAC	0.421301	0
**640**	7-KB-L16-R	1	HDAC	0.204169	0.051
**641**	7-KB-L18-T	10	HDAC	0.089458	0.999
**642**	7-KB-M2-Ta	1	HDAC	0.139376	0.89
**643**	7-KB-M5-Pr	10	HDAC	0.546454	0
**644**	7-KB-M8-Al	10	HDAC	0.439805	0
**645**	7-KB-M10-	10	HDAC	0.158211	0.945
**646**	7-KB-M11-	100	HDAC	0.488612	0
**647**	7-KB-M14-	10	HDAC	0.419233	0.003
**648**	7-KB-M16-I	10	HDAC	0.347465	0.096
**649**	7-KB-M18-	100	HDAC	0.144245	0.929
**650**	7-KB-N2-Ta	10	HDAC	0.111485	0.938
**651**	7-KB-N4-En	100	HDAC	0.311299	0.008
**652**	7-KB-N5-Pr	100	HDAC	0.485373	0
**653**	7-KB-N8-Ab	100	HDAC	0.517439	0
**654**	7-KB-N10-T	100	HDAC	0.300163	0.007
**655**	7-KB-N14-T	100	HDAC	0.179076	0.342
**656**	7-KB-N16-R	100	HDAC	0.152402	0.349
**657**	7-KB-N18-T	1000	HDAC	0.240412	0.021
**658**	7-KB-O2-Ta	100	HDAC	0.14807	0.942
**659**	7-KB-O4-En	1000	HDAC	0.549106	0
**660**	7-KB-O5-Pr	1000	HDAC	0.569296	0
**661**	7-KB-O8-Ab	1000	HDAC	0.542919	0
**662**	7-KB-O10-T	1000	HDAC	0.513037	0
**663**	7-KB-O11-A	1000	HDAC	0.50373	0

**664**	7-KB-O14-T	1000	HDAC	0.223708	0.332
**665**	7-KB-O16-F	1000	HDAC	0.390682	0.001
**666**	7-KB-P2-Ta	1000	HDAC	0.171986	0.196
**667**	7-KB-P4-En	10000	HDAC	0.54004	0
**668**	7-KB-P5-Pr	10000	HDAC	0.402548	0.015
**669**	7-KB-P8-Ab	10000	HDAC	0.180486	0.82
**670**	7-KB-P10-T	10000	HDAC	0.556625	0
**671**	7-KB-P11-A	10000	HDAC	0.426254	0.006
**672**	7-KB-P14-T	10000	HDAC	0.086259	0.885
**673**	7-KB-P16-R	10000	HDAC	0.401579	0.001
**674**	7-KB-P18-T	10000	HDAC	0.364979	0.003
**0**	2-KW-A16-	10000	EGFR	0.299176	0.091
**1**	2-KW-A19-	10000	EGFR	0.392632	0.001
**2**	2-KW-B19-	1000	EGFR	0.383442	0.004
**3**	2-KW-C16-	1000	EGFR	0.241055	0.348
**4**	2-KW-C19-	100	EGFR	0.486237	0
**5**	2-KW-D16-	100	EGFR	0.288413	0.16
**6**	2-KW-D19-	10	EGFR	0.378702	0.001
**7**	2-KW-E16-	10	EGFR	0.38225	0.002
**8**	2-KW-E19-	1	EGFR	0.402977	0.002
**9**	2-KW-F16-	1	EGFR	0.128533	0.877
**10**	2-KW-K11-	0.1	EGFR	0.204261	0.533
**11**	2-KW-L11-	1	EGFR	0.366965	0.002
**12**	2-KW-L16-	0.25	EGFR	0.330556	0.068
**13**	2-KW-L19-	0.1	EGFR	0.455464	0
**14**	2-KW-M11-	10	EGFR	0.451509	0
**15**	2-KW-M16-	2.5	EGFR	0.47067	0
**16**	2-KW-M19-	1	EGFR	0.445568	0
**17**	2-KW-N16-	25	EGFR	0.441097	0.001
**18**	2-KW-N19-	10	EGFR	0.097697	0.888
**19**	2-KW-O11-	100	EGFR	0.413731	0.001
**20**	2-KW-O16-	250	EGFR	0.121768	0.826
**21**	2-KW-O19-	100	EGFR	0.422947	0
**22**	2-KW-P11-	1000	EGFR	0.273794	0.072
**23**	2-KW-P16-	2500	EGFR	0.404399	0.002

**24**	2-KW-P19-I	1000	EGFR	0.392891	0.001
**25**	3-KW-F21-I	10000	EGFR	0.342506	0.039
**26**	3-KW-G20-	1000	EGFR	0.343874	0.002
**27**	3-KW-G21-	1000	EGFR	0.397211	0
**28**	3-KW-H20-	100	EGFR	0.359848	0
**29**	3-KW-H21-	100	EGFR	0.27669	0.082
**30**	3-KW-I20-N	10	EGFR	0.372105	0.002
**31**	3-KW-I21-F	10	EGFR	0.211933	0.629
**32**	3-KW-J20-N	1	EGFR	0.408206	0.001
**33**	3-KW-J21-F	1	EGFR	0.351471	0.049
**34**	3-KW-K4-C	1	EGFR	0.10079	0.947
**35**	3-KW-K18-I	0.1	EGFR	0.465382	0
**36**	3-KW-K20-I	0.1	EGFR	0.41087	0
**37**	3-KW-L4-C	10	EGFR	0.107162	0.914
**38**	3-KW-L18-I	1	EGFR	0.363876	0.031
**39**	3-KW-M18-	10	EGFR	0.423487	0
**40**	3-KW-N4-C	100	EGFR	0.490775	0
**41**	3-KW-N18-	100	EGFR	0.410927	0
**42**	3-KW-O4-C	1000	EGFR	0.358038	0.007
**43**	3-KW-P4-C	10000	EGFR	0.058152	1
**44**	3-KW-P18-I	1000	EGFR	0.318337	0.084
**45**	4-KW-F13-S	1000	EGFR	0.255618	0.08
**46**	4-KW-G13-	100	EGFR	0.329566	0.002
**47**	4-KW-G16-	10000	EGFR	0.341638	0.006
**48**	4-KW-H13-	10	EGFR	0.281511	0.055
**49**	4-KW-H16-	1000	EGFR	0.280097	0.036
**50**	4-KW-I13-S	1	EGFR	0.141154	0.635
**51**	4-KW-I16-V	100	EGFR	0.125619	0.754
**52**	4-KW-J13-S	0.1	EGFR	0.115084	0.828
**53**	4-KW-J16-V	10	EGFR	0.120251	0.722
**54**	4-KW-K7-Ic	1	EGFR	0.09881	0.908
**55**	4-KW-K13-	0.1	EGFR	0.17676	0.455
**56**	4-KW-K16-	1	EGFR	0.255741	0.048
**57**	4-KW-L7-Ic	10	EGFR	0.217227	0.384
**58**	4-KW-L13-	1	EGFR	0.07183	0.937

**59**	4-KW-M7-I	100	EGFR	0.291362	0.025
**60**	4-KW-M13-	10	EGFR	0.313559	0.006
**61**	4-KW-N13-	100	EGFR	0.337849	0.001
**62**	4-KW-O7-Ic	1000	EGFR	0.159256	0.787
**63**	4-KW-P7-Ic	10000	EGFR	0.277263	0.098
**64**	4-KW-P13-'	1000	EGFR	0.299845	0.026
**65**	5-KW-F4-Pc	1000	EGFR	0.448698	0
**66**	5-KW-F7-AZ	1000	EGFR	0.17688	0.552
**67**	5-KW-G4-P	100	EGFR	0.441186	0.001
**68**	5-KW-G7-A	100	EGFR	0.439155	0
**69**	5-KW-H4-P	10	EGFR	0.256954	0.351
**70**	5-KW-H7-A	10	EGFR	0.345107	0.039
**71**	5-KW-I4-Pc	1	EGFR	0.312599	0.328
**72**	5-KW-I7-AZ	1	EGFR	0.344303	0.021
**73**	5-KW-J4-Pc	0.1	EGFR	0.199816	0.674
**74**	5-KW-J7-AZ	0.1	EGFR	0.368657	0.013
**75**	5-KW-K7-O	0.1	EGFR	0.120629	0.894
**76**	5-KW-L7-OI	1	EGFR	0.433669	0.001
**77**	5-KW-M7-C	10	EGFR	0.404493	0
**78**	5-KW-O7-O	100	EGFR	0.472248	0
**79**	5-KW-P7-O	1000	EGFR	0.394756	0.004
**80**	2-KW-A15-I	2500	VEGFR	0.268227	0.033
**81**	2-KW-A17-I	10000	VEGFR	0.261322	0.096
**82**	2-KW-A20-'	10000	VEGFR	0.171824	0.662
**83**	2-KW-B15-I	250	VEGFR	0.292615	0.012
**84**	2-KW-B17-I	1000	VEGFR	0.310895	0
**85**	2-KW-B20-'	1000	VEGFR	0.234457	0.596
**86**	2-KW-C15-I	25	VEGFR	0.142908	0.592
**87**	2-KW-C17-I	100	VEGFR	0.238786	0.085
**88**	2-KW-D15-	2.5	VEGFR	0.241684	0.064
**89**	2-KW-D17-	10	VEGFR	0.229056	0.353
**90**	2-KW-D20-	100	VEGFR	0.311575	0.09
**91**	2-KW-E17-I	1	VEGFR	0.158812	0.746
**92**	2-KW-E20-'	10	VEGFR	0.139042	0.869
**93**	2-KW-F13-/	10000	VEGFR	0.176451	0.943

**94**	2-KW-F15-I 0.25	VEGFR	0.193626	0.217
**95**	2-KW-F19-I 10000	VEGFR	0.129888	0.949
**96**	2-KW-F20-T 1	VEGFR	0.218621	0.262
**97**	2-KW-F21-\ 10000	VEGFR	0.138182	0.993
**98**	2-KW-G10- 10000	VEGFR	0.36782	0
**99**	2-KW-G13- 1000	VEGFR	0.1831	0.816
**100**	2-KW-G19- 1000	VEGFR	0.272391	0.087
**101**	2-KW-G21- 1000	VEGFR	0.317004	0.065
**102**	2-KW-H10- 1000	VEGFR	0.362971	0.001
**103**	2-KW-H13- 100	VEGFR	0.386433	0.003
**104**	2-KW-H21- 100	VEGFR	0.206147	0.392
**105**	2-KW-I10-A 100	VEGFR	0.262574	0.06
**106**	2-KW-I13-A 10	VEGFR	0.352441	0.002
**107**	2-KW-I19-F 100	VEGFR	0.148349	0.759
**108**	2-KW-I21-\ 10	VEGFR	0.243671	0.481
**109**	2-KW-J10-A 10	VEGFR	0.374729	0.005
**110**	2-KW-J13-A 1	VEGFR	0.440046	0
**111**	2-KW-J19-F 10	VEGFR	0.15435	0.873
**112**	2-KW-J21-\ 1	VEGFR	0.279618	0.389
**113**	2-KW-K10-, 1	VEGFR	0.34705	0.008
**114**	2-KW-K13-' 0.1	VEGFR	0.415672	0
**115**	2-KW-K17-I 1	VEGFR	0.267074	0.081
**116**	2-KW-K19-I 1	VEGFR	0.30462	0.139
**117**	2-KW-L12-S 0.1	VEGFR	0.437942	0
**118**	2-KW-L13-\ 1	VEGFR	0.180577	0.423
**119**	2-KW-L21-(0.1	VEGFR	0.134471	0.955
**120**	2-KW-M12- 1	VEGFR	0.243316	0.197
**121**	2-KW-M13- 10	VEGFR	0.477056	0
**122**	2-KW-M17- 10	VEGFR	0.344734	0.003
**123**	2-KW-M21- 1	VEGFR	0.283216	0.228
**124**	2-KW-N12- 10	VEGFR	0.358147	0.001
**125**	2-KW-N13- 100	VEGFR	0.183592	0.255
**126**	2-KW-N17- 100	VEGFR	0.325531	0.021
**127**	2-KW-N21- 10	VEGFR	0.287992	0.144
**128**	2-KW-O12- 100	VEGFR	0.381264	0.002

**129**	2-KW-O17-	1000	VEGFR	0.153139	0.887
**130**	2-KW-O21-	100	VEGFR	0.269649	0.315
**131**	2-KW-P12-	1000	VEGFR	0.265695	0.113
**132**	2-KW-P13-	1000	VEGFR	0.256867	0.042
**133**	2-KW-P17-	10000	VEGFR	0.174719	0.639
**134**	2-KW-P21-	1000	VEGFR	0.302749	0.026
**135**	3-KW-A3-C	1000	VEGFR	0.253716	0.064
**136**	3-KW-A6-F	1000	VEGFR	0.128636	0.989
**137**	3-KW-A18-	1000	VEGFR	0.261493	0.018
**138**	3-KW-B3-C	100	VEGFR	0.233462	0.222
**139**	3-KW-B6-F	100	VEGFR	0.293218	0.007
**140**	3-KW-B18-	100	VEGFR	0.241414	0.037
**141**	3-KW-C3-C	10	VEGFR	0.185239	0.422
**142**	3-KW-C6-F	10	VEGFR	0.18622	0.637
**143**	3-KW-C18-	10	VEGFR	0.244699	0.394
**144**	3-KW-D3-C	1	VEGFR	0.119515	0.886
**145**	3-KW-D6-F	1	VEGFR	0.146902	0.5
**146**	3-KW-D18-	1	VEGFR	0.24376	0.365
**147**	3-KW-E3-C	0.1	VEGFR	0.164382	0.46
**148**	3-KW-E6-F	0.1	VEGFR	0.276745	0.371
**149**	3-KW-E18-	0.1	VEGFR	0.203183	0.268
**150**	3-KW-F18-	1000	VEGFR	0.241511	0.074
**151**	3-KW-G18-	100	VEGFR	0.27221	0.285
**152**	3-KW-H18-	10	VEGFR	0.212401	0.613
**153**	3-KW-I18-	1	VEGFR	0.484727	0
**154**	3-KW-J18-	0.1	VEGFR	0.238699	0.058
**155**	4-KW-A12-	10000	VEGFR	0.25028	0.902
**156**	4-KW-A15-	2500	VEGFR	0.305226	0.004
**157**	4-KW-A20-	10000	VEGFR	0.111542	0.987
**158**	4-KW-B12-	1000	VEGFR	0.136884	0.788
**159**	4-KW-B15-	250	VEGFR	0.2651	0.009
**160**	4-KW-B20-	1000	VEGFR	0.171871	0.424
**161**	4-KW-C15-	25	VEGFR	0.298675	0.001
**162**	4-KW-D12-	100	VEGFR	0.218927	0.411
**163**	4-KW-D15-	2.5	VEGFR	0.226391	0.085

**164**	4-KW-D20-	100	VEGFR	0.16037	0.539
**165**	4-KW-E12-	10	VEGFR	0.239998	0.039
**166**	4-KW-E20-	10	VEGFR	0.182075	0.447
**167**	4-KW-F12-	1	VEGFR	0.279037	0.008
**168**	4-KW-F15-	0.25	VEGFR	0.228861	0.098
**169**	4-KW-F20-	1	VEGFR	0.191253	0.251
**170**	4-KW-L16-	1	VEGFR	0.274549	0.051
**171**	4-KW-M16	10	VEGFR	0.281354	0.004
**172**	4-KW-N16-	100	VEGFR	0.176063	0.435
**173**	4-KW-O16-	1000	VEGFR	0.289424	0.029
**174**	4-KW-P16-	10000	VEGFR	0.324049	0.04
**175**	2-KW-L10-	1	PI3K	0.185588	0.278
**176**	2-KW-M10	10	PI3K	0.215523	0.203
**177**	2-KW-N10-	100	PI3K	0.273141	0.065
**178**	2-KW-O10-	1000	PI3K	0.158738	0.895
**179**	2-KW-P10-	10000	PI3K	0.349502	0
**180**	3-KW-A16-	2500	PI3K	0.111749	0.8
**181**	3-KW-C16-	250	PI3K	0.147522	0.644
**182**	3-KW-D16-	25	PI3K	0.144352	0.618
**183**	3-KW-E16-	2.5	PI3K	0.225245	0.214
**184**	3-KW-F16-	0.25	PI3K	0.183243	0.1
**185**	3-KW-F17-	100000	PI3K	0.150817	0.999
**186**	3-KW-F19-	500	PI3K	0.322507	0.002
**187**	3-KW-G17-	10000	PI3K	0.233062	0.093
**188**	3-KW-G19-	50	PI3K	0.329466	0.013
**189**	3-KW-H17-	1000	PI3K	0.316785	0.001
**190**	3-KW-I17-	100	PI3K	0.114266	0.919
**191**	3-KW-I19-	5	PI3K	0.325956	0.006
**192**	3-KW-J17-	10	PI3K	0.162995	0.608
**193**	3-KW-J19-	0.5	PI3K	0.075724	0.976
**194**	3-KW-K19-	0.05	PI3K	0.106638	0.969
**195**	3-KW-L8-Pi	1	PI3K	0.16044	0.795
**196**	3-KW-L21-	0.1	PI3K	0.230434	0.118
**197**	3-KW-M8-	10	PI3K	0.215935	0.284
**198**	3-KW-M21	1	PI3K	0.049178	1

**199**	3-KW-N8-P 100	PI3K	0.46005	0
**200**	3-KW-N21- 10	PI3K	0.200092	0.231
**201**	3-KW-O8-P 1000	PI3K	0.415265	0
**202**	3-KW-O21- 100	PI3K	0.299851	0.044
**203**	3-KW-P8-Pi 10000	PI3K	0.389053	0.004
**204**	3-KW-P21-1 1000	PI3K	0.390587	0
**205**	4-KW-A19-, 2500	PI3K	0.380862	0
**206**	4-KW-B19-, 250	PI3K	0.279545	0.003
**207**	4-KW-C19-, 25	PI3K	0.214573	0.078
**208**	4-KW-D19- 2.5	PI3K	0.221832	0.125
**209**	4-KW-E19-/ 0.25	PI3K	0.336673	0
**210**	4-KW-F14-I 1000	PI3K	0.162028	0.977
**211**	4-KW-G2-T 2500	PI3K	0.301907	0.023
**212**	4-KW-G5-S 10000	PI3K	0.266777	0.168
**213**	4-KW-G14- 100	PI3K	0.281283	0.055
**214**	4-KW-G20- 10000	PI3K	0.363407	0.137
**215**	4-KW-H2-T 250	PI3K	0.316273	0.003
**216**	4-KW-H5-S 1000	PI3K	0.287856	0.011
**217**	4-KW-H14- 10	PI3K	0.344391	0
**218**	4-KW-H20- 1000	PI3K	0.373445	0
**219**	4-KW-I2-TG 25	PI3K	0.162319	0.41
**220**	4-KW-I5-So 100	PI3K	0.110689	0.931
**221**	4-KW-I14-N 1	PI3K	0.317914	0.005
**222**	4-KW-I20-E 100	PI3K	0.255425	0.005
**223**	4-KW-J2-TG 2.5	PI3K	0.079702	0.966
**224**	4-KW-J5-Sc 10	PI3K	0.294617	0.037
**225**	4-KW-J20-E 10	PI3K	0.31944	0.021
**226**	4-KW-K2-T 0.25	PI3K	0.197611	0.304
**227**	4-KW-K4-D 0.1	PI3K	0.136462	0.767
**228**	4-KW-K5-S 1	PI3K	0.200687	0.65
**229**	4-KW-K14-I 0.1	PI3K	0.365199	0
**230**	4-KW-K20-I 1	PI3K	0.309275	0.01
**231**	4-KW-L4-D 1	PI3K	0.323026	0.065
**232**	4-KW-L14-(0.1	PI3K	0.320204	0
**233**	4-KW-L15-1 1	PI3K	0.228148	0.014

**234**	4-KW-L21-(0.1	PI3K	0.33539	0
**235**	4-KW-M14- 1	PI3K	0.316346	0
**236**	4-KW-M15- 10	PI3K	0.297964	0.002
**237**	4-KW-M21- 1	PI3K	0.257542	0.237
**238**	4-KW-N4-D 10	PI3K	0.182904	0.696
**239**	4-KW-N14- 10	PI3K	0.209939	0.295
**240**	4-KW-N15- 100	PI3K	0.332717	0
**241**	4-KW-N21- 10	PI3K	0.370544	0
**242**	4-KW-O4-D 100	PI3K	0.203668	0.526
**243**	4-KW-O14- 100	PI3K	0.253807	0.084
**244**	4-KW-O15- 1000	PI3K	0.328786	0
**245**	4-KW-O21- 100	PI3K	0.340405	0
**246**	4-KW-P4-D 1000	PI3K	0.221895	0.334
**247**	4-KW-P14-(1000	PI3K	0.370447	0
**248**	4-KW-P15-1 10000	PI3K	0.300772	0.001
**249**	4-KW-P21-(1000	PI3K	0.363294	0.076
**250**	5-KW-A6-LY 2500	PI3K	0.226168	0.521
**251**	5-KW-A7-Al 1000	PI3K	0.264352	0.003
**252**	5-KW-A16-, 2500	PI3K	0.360777	0
**253**	5-KW-A17-I 10000	PI3K	0.175063	0.185
**254**	5-KW-B6-LY 250	PI3K	0.196762	0.75
**255**	5-KW-B7-Al 100	PI3K	0.160308	0.213
**256**	5-KW-B17-I 1000	PI3K	0.265965	0.004
**257**	5-KW-C6-LY 25	PI3K	0.195058	0.588
**258**	5-KW-C7-Al 10	PI3K	0.187752	0.136
**259**	5-KW-C16-, 250	PI3K	0.196311	0.058
**260**	5-KW-C17-I 100	PI3K	0.260407	0.003
**261**	5-KW-D6-L' 2.5	PI3K	0.15575	0.862
**262**	5-KW-D7-A 1	PI3K	0.227525	0.086
**263**	5-KW-D16- 25	PI3K	0.183505	0.139
**264**	5-KW-D17- 10	PI3K	0.224722	0.241
**265**	5-KW-E6-LY 0.25	PI3K	0.193027	0.11
**266**	5-KW-E7-Al 0.1	PI3K	0.280081	0.009
**267**	5-KW-E16-/ 2.5	PI3K	0.148524	0.869
**268**	5-KW-E17-I 1	PI3K	0.144062	0.808

**269**	5-KW-F11-(	10000	PI3K	0.39233	0
**270**	5-KW-F16-/	0.25	PI3K	0.167644	0.711
**271**	5-KW-G9-S	10000	PI3K	0.352854	0
**272**	5-KW-G11-	1000	PI3K	0.303038	0
**273**	5-KW-H9-S	1000	PI3K	0.363152	0
**274**	5-KW-H11-	100	PI3K	0.300044	0.001
**275**	5-KW-I9-Se	100	PI3K	0.247535	0.021
**276**	5-KW-I11-(	10	PI3K	0.21782	0.344
**277**	5-KW-J9-Se	10	PI3K	0.253071	0.15
**278**	5-KW-J11-(	1	PI3K	0.229347	0.209
**279**	5-KW-K9-S	1	PI3K	0.156721	0.503
**280**	5-KW-L14-/	0.1	PI3K	0.169348	0.846
**281**	5-KW-L20-:	1	PI3K	0.203001	0.687
**282**	5-KW-L23-(	0.1	PI3K	0.060837	1
**283**	5-KW-M14-	1	PI3K	0.193647	0.594
**284**	5-KW-M20-	10	PI3K	0.173463	0.851
**285**	5-KW-M23-	1	PI3K	0.167016	0.816
**286**	5-KW-N14-	10	PI3K	0.146896	0.922
**287**	5-KW-N20-	100	PI3K	0.243762	0.401
**288**	5-KW-N23-	10	PI3K	0.297705	0.06
**289**	5-KW-O14-	100	PI3K	0.206511	0.219
**290**	5-KW-O20-	1000	PI3K	0.342579	0.004
**291**	5-KW-O23-	100	PI3K	0.38109	0.001
**292**	5-KW-P14-/	1000	PI3K	0.255386	0.303
**293**	5-KW-P20-:	10000	PI3K	0.400776	0
**294**	5-KW-P23-(	1000	PI3K	0.400612	0.017
**295**	6-KW-A8-T(	10000	PI3K	0.276282	0.005
**296**	6-KW-B8-T(	1000	PI3K	0.252631	0.035
**297**	6-KW-C8-T(	100	PI3K	0.056293	0.996
**298**	6-KW-D8-T(	10	PI3K	0.09258	0.992
**299**	6-KW-E8-T(	1	PI3K	0.26157	0.021
**300**	6-KW-L6-G	1	PI3K	0.042969	1
**301**	6-KW-M6-(	10	PI3K	0.205459	0.495
**302**	6-KW-N6-G	100	PI3K	0.205439	0.639
**303**	6-KW-O6-G	1000	PI3K	0.343651	0.001

**304**	6-KW-P6-G	10000	PI3K	0.419575	0
**305**	1-KW-F11-/	10000	Topoisome	0.570818	0
**306**	1-KW-G11-	1000	Topoisome	0.654011	0
**307**	1-KW-G20-	1000	Topoisome	0.487658	0
**308**	1-KW-H11-	100	Topoisome	0.545577	0
**309**	1-KW-H20-	100	Topoisome	0.517428	0
**310**	1-KW-I11-/	10	Topoisome	0.466331	0
**311**	1-KW-I20-E	10	Topoisome	0.493028	0
**312**	1-KW-J11-/	1	Topoisome	0.248555	0.124
**313**	1-KW-J20-E	1	Topoisome	0.365016	0.011
**314**	1-KW-K11-:	1	Topoisome	0.544474	0
**315**	1-KW-K20-I	0.1	Topoisome	0.314783	0.179
**316**	1-KW-L11-S	10	Topoisome	0.582549	0
**317**	1-KW-L14-T	1	Topoisome	0.37327	0.006
**318**	1-KW-M11-	100	Topoisome	0.566281	0
**319**	1-KW-M14-	10	Topoisome	0.363593	0.008
**320**	1-KW-N14-	100	Topoisome	0.540814	0
**321**	1-KW-O11-	1000	Topoisome	0.568696	0.003
**322**	1-KW-O14-	1000	Topoisome	0.508906	0
**323**	1-KW-P11-:	10000	Topoisome	0.416375	0.305
**324**	1-KW-P14-T	10000	Topoisome	0.564991	0
**325**	3-KW-A11-I	10000	Topoisome	0.588107	0
**326**	3-KW-B11-I	1000	Topoisome	0.585133	0
**327**	3-KW-C11-I	100	Topoisome	0.372575	0.008
**328**	3-KW-D11-	10	Topoisome	0.17253	0.581
**329**	3-KW-E11-I	1	Topoisome	0.198953	0.346
**330**	3-KW-G9-D	1000	Topoisome	0.575684	0
**331**	3-KW-G10-	10000	Topoisome	0.236748	0.232
**332**	3-KW-H9-D	100	Topoisome	0.558468	0
**333**	3-KW-H10-	1000	Topoisome	0.258622	0.112
**334**	3-KW-I9-Da	10	Topoisome	0.460202	0
**335**	3-KW-I10-T	100	Topoisome	0.173063	0.468
**336**	3-KW-J9-Da	1	Topoisome	0.406675	0.005
**337**	3-KW-J10-T	10	Topoisome	0.361005	0.045
**338**	3-KW-K7-Ic	0.1	Topoisome	0.31722	0.03

**339**	3-KW-K9-D	0.1	Topoisome	0.498076	0
**340**	3-KW-K10-`	1	Topoisome	0.258852	0.26
**341**	3-KW-L6-D	0.1	Topoisome	0.036751	1
**342**	3-KW-L7-Id	1	Topoisome	0.280455	0.257
**343**	3-KW-L9-Va	0.5	Topoisome	0.359186	0.012
**344**	3-KW-L10-I	0.1	Topoisome	0.323319	0.062
**345**	3-KW-L16-I	1	Topoisome	0.300995	0.146
**346**	3-KW-M6-D	1	Topoisome	0.46236	0
**347**	3-KW-M7-I	10	Topoisome	0.271083	0.395
**348**	3-KW-M9-V	5	Topoisome	0.383132	0.009
**349**	3-KW-M10-	1	Topoisome	0.079908	0.963
**350**	3-KW-M16-	10	Topoisome	0.436334	0.002
**351**	3-KW-N6-D	10	Topoisome	0.380976	0.037
**352**	3-KW-N9-V	50	Topoisome	0.439853	0.003
**353**	3-KW-N10-	10	Topoisome	0.390346	0.021
**354**	3-KW-N16-	100	Topoisome	0.259554	0.204
**355**	3-KW-O6-D	100	Topoisome	0.56315	0
**356**	3-KW-O7-Ic	100	Topoisome	0.61696	0
**357**	3-KW-O9-V	500	Topoisome	0.594807	0
**358**	3-KW-O10-	100	Topoisome	0.59776	0
**359**	3-KW-O16-	1000	Topoisome	0.387686	0.038
**360**	3-KW-P6-D	1000	Topoisome	0.470948	0
**361**	3-KW-P7-Ic	1000	Topoisome	0.449932	0.21
**362**	3-KW-P9-Va	5000	Topoisome	0.553778	0
**363**	3-KW-P10-I	1000	Topoisome	0.489568	0
**364**	3-KW-P16-I	10000	Topoisome	0.291706	0.328
**365**	1-KW-A10-`	10000	Mitotic	0.601986	0.002
**366**	1-KW-A13-I	1000	Mitotic	0.586937	0
**367**	1-KW-A18-I	1000	Mitotic	0.754035	0
**368**	1-KW-B10-`	1000	Mitotic	0.629586	0
**369**	1-KW-B13-I	100	Mitotic	0.099347	0.954
**370**	1-KW-B18-I	100	Mitotic	0.620086	0
**371**	1-KW-C10-`	100	Mitotic	0.673177	0
**372**	1-KW-C13-I	10	Mitotic	0.425556	0.003
**373**	1-KW-C18-I	10	Mitotic	0.563386	0

**374**	1-KW-D10-	10	Mitotic	0.447431	0.003
**375**	1-KW-D13-	1	Mitotic	0.17368	0.651
**376**	1-KW-D18-	1	Mitotic	0.434018	0.001
**377**	1-KW-E10-\	1	Mitotic	0.477274	0.001
**378**	1-KW-E13-I	0.1	Mitotic	0.291382	0.316
**379**	1-KW-E18-I	0.1	Mitotic	0.366888	0.027
**380**	1-KW-F13-\	1000	Mitotic	0.654892	0
**381**	1-KW-G13-	100	Mitotic	0.163413	0.618
**382**	1-KW-G15-	1000	Mitotic	0.605741	0.002
**383**	1-KW-H13-	10	Mitotic	0.442568	0
**384**	1-KW-H15-	100	Mitotic	0.637459	0
**385**	1-KW-I13-\	1	Mitotic	0.460274	0
**386**	1-KW-I15-E	10	Mitotic	0.702457	0
**387**	1-KW-J13-\	0.1	Mitotic	0.133228	0.759
**388**	1-KW-J15-E	1	Mitotic	0.527974	0
**389**	1-KW-K7-Vi	0.1	Mitotic	0.284821	0.353
**390**	1-KW-K15-I	0.1	Mitotic	0.537907	0
**391**	1-KW-L7-Vi	1	Mitotic	0.382648	0.037
**392**	1-KW-L20-\	0.1	Mitotic	0.343543	0.208
**393**	1-KW-M7-\	10	Mitotic	0.457214	0
**394**	1-KW-M20-	1	Mitotic	0.455846	0.001
**395**	1-KW-N20-	10	Mitotic	0.391924	0.021
**396**	1-KW-O7-V	100	Mitotic	0.17164	0.688
**397**	1-KW-O20-	100	Mitotic	0.403922	0.03
**398**	1-KW-P7-Vi	1000	Mitotic	0.70711	0
**399**	1-KW-P20-\	1000	Mitotic	0.536002	0
**400**	3-KW-A7-D	1000	Mitotic	0.74865	0
**401**	3-KW-B7-D	100	Mitotic	0.769984	0
**402**	3-KW-C7-D	10	Mitotic	0.726914	0
**403**	3-KW-D7-D	1	Mitotic	0.241945	0.231
**404**	3-KW-E7-D	0.1	Mitotic	0.313926	0.249
**405**	6-KW-L19-/	1	Mitotic	0.368013	0.037
**406**	6-KW-M19-	10	Mitotic	0.301692	0.286
**407**	6-KW-N19-	100	Mitotic	0.284234	0.542
**408**	6-KW-O19-	1000	Mitotic	0.705848	0

**409**	6-KW-P19-/	10000	Mitotic	0.545017	0.002
**410**	2-KW-A12-	250	MEK1/2	0.460325	0.016
**411**	2-KW-B12-	25	MEK1/2	0.41371	0.069
**412**	2-KW-D12-	2.5	MEK1/2	0.349475	0.142
**413**	2-KW-E12-	0.25	MEK1/2	0.459613	0.002
**414**	2-KW-F12-	2.5000000	MEK1/2	0.2865	0.449
**415**	2-KW-F14-(	1000	MEK1/2	0.460921	0.02
**416**	2-KW-G14-	100	MEK1/2	0.546611	0.002
**417**	2-KW-H14-	10	MEK1/2	0.414551	0.008
**418**	2-KW-I14-C	1	MEK1/2	0.248115	0.436
**419**	2-KW-K14-	0.1	MEK1/2	0.205257	0.821
**420**	2-KW-L20-S	1	MEK1/2	0.155922	0.918
**421**	2-KW-M20-	10	MEK1/2	0.191852	0.886
**422**	2-KW-N20-	100	MEK1/2	0.183249	0.904
**423**	2-KW-O20-	1000	MEK1/2	0.259831	0.767
**424**	2-KW-P20-	10000	MEK1/2	0.260188	0.608
**425**	4-KW-A10-	1000	MEK1/2	0.44373	0.002
**426**	4-KW-A13-	1000	MEK1/2	0.600916	0
**427**	4-KW-B10-	100	MEK1/2	0.494025	0.001
**428**	4-KW-B13-	100	MEK1/2	0.443586	0.007
**429**	4-KW-C10-	10	MEK1/2	0.49533	0
**430**	4-KW-C13-	10	MEK1/2	0.42519	0.01
**431**	4-KW-D10-	1	MEK1/2	0.494438	0.002
**432**	4-KW-D13-	1	MEK1/2	0.475343	0.009
**433**	4-KW-E10-I	0.1	MEK1/2	0.485126	0.002
**434**	4-KW-E13-I	0.1	MEK1/2	0.474168	0.001
**435**	4-KW-L19-(	0.25	MEK1/2	0.197551	0.931
**436**	4-KW-M19-	2.5	MEK1/2	0.156533	0.996
**437**	4-KW-N19-	25	MEK1/2	0.381145	0.086
**438**	4-KW-O19-	250	MEK1/2	0.362014	0.274
**439**	4-KW-P19-	2500	MEK1/2	0.5601	0
**440**	1-KW-L2-OI	1	PARP	0.62854	0
**441**	1-KW-L6-Ru	1	PARP	0.528308	0.012
**442**	1-KW-M2-C	10	PARP	0.609635	0.002
**443**	1-KW-M6-F	10	PARP	0.308289	0.422

**444**	1-KW-N2-O	100	PARP	0.566105	0.001
**445**	1-KW-N6-R	100	PARP	0.556053	0.006
**446**	1-KW-O2-O	1000	PARP	0.604926	0.001
**447**	1-KW-O6-R	1000	PARP	0.540743	0.005
**448**	1-KW-P2-O	10000	PARP	0.547954	0.029
**449**	1-KW-P6-R	10000	PARP	0.545716	0.004
**450**	7-KW-A3-Ta	1000	PARP	0.573261	0.016
**451**	7-KW-B2-Ve	10000	PARP	0.664557	0
**452**	7-KW-B3-Ta	100	PARP	0.546159	0.012
**453**	7-KW-C2-Ve	1000	PARP	0.571143	0.003
**454**	7-KW-C3-Ta	10	PARP	0.607416	0.001
**455**	7-KW-D2-V	100	PARP	0.557401	0.002
**456**	7-KW-D3-Ta	1	PARP	0.317401	0.157
**457**	7-KW-E2-Ve	10	PARP	0.606781	0.006
**458**	7-KW-E3-Ta	0.1	PARP	0.283864	0.273
**459**	7-KW-F2-Ve	1	PARP	0.478242	0.006
**460**	7-KW-G2-N	10000	PARP	0.59113	0.008
**461**	7-KW-H2-N	1000	PARP	0.56112	0.001
**462**	7-KW-I2-Ni	100	PARP	0.356541	0.315
**463**	7-KW-J2-Ni	10	PARP	0.453576	0.025
**464**	7-KW-K2-N	1	PARP	0.503966	0.007
**465**	3-KW-A19-I	1000	CDK	0.577997	0
**466**	3-KW-B19-I	100	CDK	0.582443	0
**467**	3-KW-B23-A	2500	CDK	0.494488	0
**468**	3-KW-C19-I	10	CDK	0.430511	0.001
**469**	3-KW-C23-A	250	CDK	0.497467	0
**470**	3-KW-D19-	1	CDK	0.463571	0
**471**	3-KW-D23-	25	CDK	0.488263	0
**472**	3-KW-E19-I	0.1	CDK	0.300677	0.028
**473**	3-KW-E23-A	2.5	CDK	0.337896	0.108
**474**	3-KW-F23-A	0.25	CDK	0.221345	0.708
**475**	3-KW-K17-I	1	CDK	0.380447	0.014
**476**	3-KW-L19-I	1	CDK	0.375308	0.063
**477**	3-KW-M17-	10	CDK	0.459915	0
**478**	3-KW-M19-	10	CDK	0.448276	0.002

**479**	3-KW-N17-	100	CDK	0.452822	0
**480**	3-KW-N19-	100	CDK	0.479922	0
**481**	3-KW-O17-	1000	CDK	0.49591	0
**482**	3-KW-O19-	1000	CDK	0.458552	0
**483**	3-KW-P17-I	10000	CDK	0.456414	0
**484**	3-KW-P19-I	10000	CDK	0.451307	0
**485**	4-KW-A4-SI	10000	CDK	0.579043	0
**486**	4-KW-A8-M	10000	CDK	0.5871	0
**487**	4-KW-B4-SI	1000	CDK	0.57964	0
**488**	4-KW-B8-M	1000	CDK	0.411981	0.001
**489**	4-KW-C4-SI	100	CDK	0.389221	0.031
**490**	4-KW-C8-M	100	CDK	0.234675	0.264
**491**	4-KW-D4-SI	10	CDK	0.144146	0.769
**492**	4-KW-D8-M	10	CDK	0.089123	0.909
**493**	4-KW-E4-SI	1	CDK	0.270695	0.309
**494**	4-KW-E8-M	1	CDK	0.14111	0.71
**495**	4-KW-F4-Se	10000	CDK	0.431073	0.009
**496**	4-KW-F22-A	10000	CDK	0.574144	0
**497**	4-KW-G4-S	1000	CDK	0.073032	0.995
**498**	4-KW-G22-	1000	CDK	0.577681	0
**499**	4-KW-H4-S	100	CDK	0.291409	0.084
**500**	4-KW-H22-	100	CDK	0.395351	0.005
**501**	4-KW-I4-Se	10	CDK	0.103967	0.919
**502**	4-KW-I22-A	10	CDK	0.364721	0.007
**503**	4-KW-J4-Se	1	CDK	0.087895	0.956
**504**	4-KW-J22-A	1	CDK	0.262166	0.178
**505**	5-KW-A19-A	10000	CDK	0.598037	0
**506**	5-KW-B19-A	1000	CDK	0.421176	0.002
**507**	5-KW-C19-A	100	CDK	0.451113	0.001
**508**	5-KW-D19-	10	CDK	0.159909	0.88
**509**	5-KW-E19-A	1	CDK	0.415921	0.009
**510**	5-KW-K17-A	1	CDK	0.334621	0.137
**511**	5-KW-M17-	10	CDK	0.423179	0.005
**512**	5-KW-N17-	100	CDK	0.44328	0.004
**513**	5-KW-O17-	1000	CDK	0.584007	0

514	5-KW-P17-	10000	CDK	0.589781	0
515	6-KW-A17-	1000	CDK	0.448988	0
516	6-KW-B17-	100	CDK	0.366447	0.02
517	6-KW-C17-	10	CDK	0.33972	0.016
518	6-KW-D17-	1	CDK	0.230971	0.223
519	6-KW-E17-	0.1	CDK	0.142744	0.806
520	6-KW-L15-	1	CDK	0.097373	0.896
521	6-KW-M15-	10	CDK	0.253963	0.424
522	6-KW-N15-	100	CDK	0.421589	0.007
523	6-KW-O15-	1000	CDK	0.583225	0.001
524	6-KW-P15-	10000	CDK	0.488314	0.002
525	7-KW-A21-	10000	BET	0.350825	0.15
526	7-KW-A22-	30000	BET	0.630568	0
527	7-KW-B21-	1000	BET	0.404865	0.014
528	7-KW-B22-	3000	BET	0.480805	0
529	7-KW-C21-	100	BET	0.244896	0.28
530	7-KW-C22-	300	BET	0.414498	0.001
531	7-KW-D21-	10	BET	0.460914	0.001
532	7-KW-D22-	30	BET	0.27108	0.532
533	7-KW-E21-	1	BET	0.27277	0.461
534	7-KW-E22-	3	BET	0.225219	0.74
535	7-KW-G10-	10000	BET	0.660963	0
536	7-KW-G15-	10000	BET	0.646597	0
537	7-KW-H10-	1000	BET	0.646124	0
538	7-KW-H15-	1000	BET	0.628901	0
539	7-KW-I10-	100	BET	0.505406	0
540	7-KW-I15-	100	BET	0.274355	0.17
541	7-KW-J10-	10	BET	0.266744	0.246
542	7-KW-J15-	10	BET	0.236205	0.356
543	7-KW-K10-	1	BET	0.24918	0.357
544	7-KW-K13-	1	BET	0.202765	0.662
545	7-KW-K15-	1	BET	0.264386	0.401
546	7-KW-L12-	1	BET	0.218748	0.571
547	7-KW-L13-	10	BET	0.257353	0.241
548	7-KW-L20-	1	BET	0.178502	0.856

**549**	7-KW-L23-	0.03	BET	0.250518	0.445
**550**	7-KW-M12-	10	BET	0.224863	0.755
**551**	7-KW-M13-	100	BET	0.634975	0
**552**	7-KW-M20-	10	BET	0.211398	0.769
**553**	7-KW-M23-	0.3	BET	0.284194	0.401
**554**	7-KW-N12-	100	BET	0.186175	0.855
**555**	7-KW-N13-	1000	BET	0.637939	0.001
**556**	7-KW-N20-	100	BET	0.630486	0
**557**	7-KW-N23-	3	BET	0.385236	0.064
**558**	7-KW-O12-	1000	BET	0.574894	0
**559**	7-KW-O20-	1000	BET	0.657553	0
**560**	7-KW-O23-	30	BET	0.686027	0
**561**	7-KW-P12-	10000	BET	0.644116	0
**562**	7-KW-P13-	10000	BET	0.595803	0.003
**563**	7-KW-P20-	10000	BET	0.643239	0
**564**	7-KW-P23-	300	BET	0.643994	0
**565**	8-KW-K22-	1	BET	0.287527	0.163
**566**	8-KW-L22-	10	BET	0.216784	0.316
**567**	8-KW-M22-	100	BET	0.183542	0.705
**568**	8-KW-N22-	1000	BET	0.483259	0
**569**	8-KW-O22-	10000	BET	0.537863	0
**570**	1-KW-A3-V	10000	HDAC	0.51674	0
**571**	1-KW-B3-V	1000	HDAC	0.362423	0.012
**572**	1-KW-C3-V	100	HDAC	0.308072	0.062
**573**	1-KW-D3-V	10	HDAC	0.164631	0.727
**574**	1-KW-E3-V	1	HDAC	0.137028	0.931
**575**	1-KW-L12-	0.1	HDAC	0.182893	0.491
**576**	1-KW-M12-	1	HDAC	0.321136	0.007
**577**	1-KW-N12-	10	HDAC	0.454005	0.02
**578**	1-KW-O12-	100	HDAC	0.455906	0.01
**579**	1-KW-P12-	1000	HDAC	0.362063	0.111
**580**	3-KW-A4-P	1000	HDAC	0.373625	0.081
**581**	3-KW-B4-P	100	HDAC	0.532768	0
**582**	3-KW-C4-P	10	HDAC	0.300639	0.116
**583**	3-KW-D4-P	1	HDAC	0.080645	0.961

**584**	3-KW-E4-Pa	0.1	HDAC	0.265783	0.155
**585**	3-KW-F7-Q	1000	HDAC	0.478656	0.008
**586**	3-KW-G7-Q	100	HDAC	0.326454	0.049
**587**	3-KW-G12-	1000000	HDAC	0.12207	0.754
**588**	3-KW-H7-Q	10	HDAC	0.357387	0.008
**589**	3-KW-H12-	100000	HDAC	0.064605	0.97
**590**	3-KW-I7-Qu	1	HDAC	0.198199	0.594
**591**	3-KW-I12-V	10000	HDAC	0.094941	0.84
**592**	3-KW-J7-Qu	0.1	HDAC	0.127739	0.77
**593**	3-KW-J12-V	1000	HDAC	0.153559	0.533
**594**	3-KW-K3-B	1	HDAC	0.063206	1
**595**	3-KW-K12-	100	HDAC	0.290921	0.066
**596**	3-KW-L3-Be	10	HDAC	0.051943	0.997
**597**	3-KW-M3-E	100	HDAC	0.334082	0.031
**598**	3-KW-N3-B	1000	HDAC	0.387531	0.003
**599**	3-KW-O3-B	10000	HDAC	0.386709	0.037
**600**	7-KW-A5-M	10000	HDAC	0.527822	0
**601**	7-KW-A7-C	10000	HDAC	0.437916	0.005
**602**	7-KW-A9-G	1000	HDAC	0.481325	0
**603**	7-KW-A12-	10000	HDAC	0.499894	0
**604**	7-KW-B5-M	1000	HDAC	0.5272	0
**605**	7-KW-B7-C	1000	HDAC	0.43965	0.011
**606**	7-KW-B12-	1000	HDAC	0.546949	0
**607**	7-KW-C5-M	100	HDAC	0.489592	0
**608**	7-KW-C7-C	100	HDAC	0.551417	0
**609**	7-KW-C9-G	100	HDAC	0.462399	0
**610**	7-KW-D7-C	10	HDAC	0.091735	0.795
**611**	7-KW-D9-G	10	HDAC	0.458822	0
**612**	7-KW-D12-	100	HDAC	0.268131	0.019
**613**	7-KW-E5-M	10	HDAC	0.2787	0.059
**614**	7-KW-E7-C	1	HDAC	0.215626	0.186
**615**	7-KW-E9-G	1	HDAC	0.182868	0.184
**616**	7-KW-E12-	10	HDAC	0.162086	0.467
**617**	7-KW-F5-M	1	HDAC	0.439981	0
**618**	7-KW-F7-R	10000	HDAC	0.523483	0

619	7-KW-F9-Gi	0.1	HDAC	0.224947	0.054
620	7-KW-F12-I	1	HDAC	0.365529	0
621	7-KW-F19-I	10000	HDAC	0.234488	0.302
622	7-KW-G7-R	1000	HDAC	0.460098	0
623	7-KW-G19-	1000	HDAC	0.163299	0.7
624	7-KW-H7-R	100	HDAC	0.414401	0
625	7-KW-I7-Re	10	HDAC	0.196837	0.306
626	7-KW-I19-F	100	HDAC	0.183031	0.521
627	7-KW-J7-Re	1	HDAC	0.411032	0
628	7-KW-J19-F	10	HDAC	0.050184	0.987
629	7-KW-K4-Er	1	HDAC	0.266005	0.181
630	7-KW-K11-/	1	HDAC	0.351324	0
631	7-KW-K18-1	1	HDAC	0.320633	0.038
632	7-KW-K19-I	1	HDAC	0.415755	0.001
633	7-KW-L2-Ta	0.1	HDAC	0.279427	0.05
634	7-KW-L4-Er	10	HDAC	0.434625	0
635	7-KW-L5-Pr	1	HDAC	0.136182	0.834
636	7-KW-L8-Al	1	HDAC	0.114307	0.826
637	7-KW-L10-1	1	HDAC	0.493898	0
638	7-KW-L11-/	10	HDAC	0.388819	0
639	7-KW-L14-1	1	HDAC	0.260266	0.065
640	7-KW-L16-I	1	HDAC	0.310889	0.02
641	7-KW-L18-1	10	HDAC	0.439105	0
642	7-KW-M2-T	1	HDAC	0.167765	0.725
643	7-KW-M5-P	10	HDAC	0.166117	0.65
644	7-KW-M8-A	10	HDAC	0.260482	0.029
645	7-KW-M10-	10	HDAC	0.497037	0
646	7-KW-M11-	100	HDAC	0.412708	0
647	7-KW-M14-	10	HDAC	0.283342	0.063
648	7-KW-M16-	10	HDAC	0.451403	0
649	7-KW-M18-	100	HDAC	0.607313	0
650	7-KW-N2-Ta	10	HDAC	0.240814	0.161
651	7-KW-N4-Er	100	HDAC	0.409739	0
652	7-KW-N5-P	100	HDAC	0.410515	0
653	7-KW-N8-A	100	HDAC	0.43918	0

**654**	7-KW-N10-	100	HDAC	0.397193	0.001
**655**	7-KW-N14-	100	HDAC	0.128372	0.712
**656**	7-KW-N16-	100	HDAC	0.22835	0.16
**657**	7-KW-N18-	1000	HDAC	0.287136	0.107
**658**	7-KW-O2-Ta	100	HDAC	0.3329	0.028
**659**	7-KW-O4-Er	1000	HDAC	0.488914	0
**660**	7-KW-O5-P	1000	HDAC	0.500547	0
**661**	7-KW-O8-A	1000	HDAC	0.507194	0
**662**	7-KW-O10-	1000	HDAC	0.424577	0
**663**	7-KW-O11-	1000	HDAC	0.466346	0
**664**	7-KW-O14-	1000	HDAC	0.378244	0.001
**665**	7-KW-O16-	1000	HDAC	0.412644	0.001
**666**	7-KW-P2-Ta	1000	HDAC	0.392792	0.001
**667**	7-KW-P4-Er	10000	HDAC	0.474465	0
**668**	7-KW-P5-Pı	10000	HDAC	0.431316	0.006
**669**	7-KW-P8-Al	10000	HDAC	0.397807	0.035
**670**	7-KW-P10-	10000	HDAC	0.527227	0
**671**	7-KW-P11-	10000	HDAC	0.4839	0.002
**672**	7-KW-P14-	10000	HDAC	0.417345	0.001
**673**	7-KW-P16-	10000	HDAC	0.369348	0.009
**674**	7-KW-P18-	10000	HDAC	0.447657	0
**0**	2-MHB-A16	10000	EGFR	0.665107	0
**1**	2-MHB-A19	10000	EGFR	0.665234	0
**2**	2-MHB-B19	1000	EGFR	0.588841	0
**3**	2-MHB-C16	1000	EGFR	0.698621	0
**4**	2-MHB-C19	100	EGFR	0.578222	0
**5**	2-MHB-D16	100	EGFR	0.672425	0
**6**	2-MHB-D19	10	EGFR	0.059092	0.992
**7**	2-MHB-E16	10	EGFR	0.189802	0.823
**8**	2-MHB-E19	1	EGFR	0.029024	1
**9**	2-MHB-F16	1	EGFR	0.382997	0.015
**10**	2-MHB-K11	0.1	EGFR	0.118348	0.972
**11**	2-MHB-L11	1	EGFR	0.390537	0.004
**12**	2-MHB-L16	0.25	EGFR	0.055749	0.992
**13**	2-MHB-L19	0.1	EGFR	0.628982	0

**14**	2-MHB-M1	10	EGFR	0.683394	0
**15**	2-MHB-M1	2.5	EGFR	0.49585	0
**16**	2-MHB-M1	1	EGFR	0.341685	0.01
**17**	2-MHB-N1	25	EGFR	0.292626	0.015
**18**	2-MHB-N1	10	EGFR	0.638026	0
**19**	2-MHB-O11	100	EGFR	0.661786	0
**20**	2-MHB-O1	250	EGFR	0.685214	0
**21**	2-MHB-O1	100	EGFR	0.65973	0
**22**	2-MHB-P11	1000	EGFR	0.672607	0
**23**	2-MHB-P1	2500	EGFR	0.676354	0
**24**	2-MHB-P1	1000	EGFR	0.664896	0
**25**	3-MHB-F21	10000	EGFR	0.559854	0
**26**	3-MHB-G2	1000	EGFR	0.68309	0
**27**	3-MHB-G21	1000	EGFR	0.624805	0
**28**	3-MHB-H2	100	EGFR	0.677859	0
**29**	3-MHB-H21	100	EGFR	0.288081	0.045
**30**	3-MHB-I20	10	EGFR	0.681907	0
**31**	3-MHB-I21	10	EGFR	0.130857	0.782
**32**	3-MHB-J20	1	EGFR	0.223575	0.175
**33**	3-MHB-J21	1	EGFR	0.220762	0.662
**34**	3-MHB-K4-	1	EGFR	0.245124	0.441
**35**	3-MHB-K18	0.1	EGFR	0.626594	0
**36**	3-MHB-K2	0.1	EGFR	0.503576	0
**37**	3-MHB-L4-	10	EGFR	0.649094	0
**38**	3-MHB-L18	1	EGFR	0.585978	0
**39**	3-MHB-M1	10	EGFR	0.686959	0
**40**	3-MHB-N4-	100	EGFR	0.636641	0
**41**	3-MHB-N1	100	EGFR	0.682563	0
**42**	3-MHB-O4-	1000	EGFR	0.651538	0
**43**	3-MHB-P4-	10000	EGFR	0.603243	0
**44**	3-MHB-P18	1000	EGFR	0.670492	0
**45**	4-MHB-F13	1000	EGFR	0.673819	0
**46**	4-MHB-G1	100	EGFR	0.671047	0
**47**	4-MHB-G1	10000	EGFR	0.68236	0
**48**	4-MHB-H1	10	EGFR	0.708349	0

**49**	4-MHB-H16	1000	EGFR	0.699482	0
**50**	4-MHB-I13-	1	EGFR	0.519352	0
**51**	4-MHB-I16-	100	EGFR	0.59437	0
**52**	4-MHB-J13	0.1	EGFR	0.311202	0.025
**53**	4-MHB-J16	10	EGFR	0.119343	0.729
**54**	4-MHB-K7-	1	EGFR	0.55255	0
**55**	4-MHB-K13	0.1	EGFR	0.105193	0.797
**56**	4-MHB-K16	1	EGFR	0.109061	0.687
**57**	4-MHB-L7-	10	EGFR	0.37583	0.001
**58**	4-MHB-L13	1	EGFR	0.728305	0
**59**	4-MHB-M7-	100	EGFR	0.599512	0
**60**	4-MHB-M1	10	EGFR	0.694375	0
**61**	4-MHB-N13	100	EGFR	0.670799	0
**62**	4-MHB-O7-	1000	EGFR	0.676825	0
**63**	4-MHB-P7-	10000	EGFR	0.668704	0
**64**	4-MHB-P13	1000	EGFR	0.671137	0
**65**	5-MHB-F4-	1000	EGFR	0.652557	0
**66**	5-MHB-F7-	1000	EGFR	0.686624	0
**67**	5-MHB-G4-	100	EGFR	0.668119	0
**68**	5-MHB-G7-	100	EGFR	0.672706	0
**69**	5-MHB-H4-	10	EGFR	0.65306	0
**70**	5-MHB-H7-	10	EGFR	0.657396	0
**71**	5-MHB-I4-	1	EGFR	0.648328	0
**72**	5-MHB-I7-	1	EGFR	0.41392	0
**73**	5-MHB-J4-	0.1	EGFR	0.416995	0
**74**	5-MHB-J7-	0.1	EGFR	0.11159	0.914
**75**	5-MHB-K7-	0.1	EGFR	0.163538	0.892
**76**	5-MHB-L7-	1	EGFR	0.106107	0.732
**77**	5-MHB-M7-	10	EGFR	0.081039	0.995
**78**	5-MHB-O7-	100	EGFR	0.049281	0.994
**79**	5-MHB-P7-	1000	EGFR	0.46979	0
**80**	2-MHB-A15	2500	VEGFR	0.197874	0.74
**81**	2-MHB-A17	10000	VEGFR	0.129589	0.981
**82**	2-MHB-A20	10000	VEGFR	0.13218	0.996
**83**	2-MHB-B15	250	VEGFR	0.256155	0.448

**84**	2-MHB-B17	1000	VEGFR	0.152764	0.915
**85**	2-MHB-B20	1000	VEGFR	0.331512	0.004
**86**	2-MHB-C15	25	VEGFR	0.343251	0.04
**87**	2-MHB-C17	100	VEGFR	0.357897	0.007
**88**	2-MHB-D15	2.5	VEGFR	0.377306	0
**89**	2-MHB-D17	10	VEGFR	0.319838	0.034
**90**	2-MHB-D20	100	VEGFR	0.333571	0.001
**91**	2-MHB-E17	1	VEGFR	0.344349	0.001
**92**	2-MHB-E20	10	VEGFR	0.371352	0
**93**	2-MHB-F13	10000	VEGFR	0.260485	0.472
**94**	2-MHB-F15	0.25	VEGFR	0.399991	0
**95**	2-MHB-F19	10000	VEGFR	0.089581	1
**96**	2-MHB-F20	1	VEGFR	0.333949	0
**97**	2-MHB-F21	10000	VEGFR	0.329367	0
**98**	2-MHB-G10	10000	VEGFR	0.315325	0.004
**99**	2-MHB-G13	1000	VEGFR	0.230274	0.488
**100**	2-MHB-G19	1000	VEGFR	0.110587	0.99
**101**	2-MHB-G21	1000	VEGFR	0.437902	0
**102**	2-MHB-H10	1000	VEGFR	0.370237	0.001
**103**	2-MHB-H13	100	VEGFR	0.364728	0
**104**	2-MHB-H21	100	VEGFR	0.40573	0
**105**	2-MHB-I10-	100	VEGFR	0.307173	0.052
**106**	2-MHB-I13-	10	VEGFR	0.29318	0.093
**107**	2-MHB-I19-	100	VEGFR	0.051299	1
**108**	2-MHB-I21-	10	VEGFR	0.375167	0
**109**	2-MHB-J10	10	VEGFR	0.373275	0
**110**	2-MHB-J13	1	VEGFR	0.234014	0.421
**111**	2-MHB-J19	10	VEGFR	0.286521	0.125
**112**	2-MHB-J21	1	VEGFR	0.351681	0
**113**	2-MHB-K10	1	VEGFR	0.337721	0
**114**	2-MHB-K13	0.1	VEGFR	0.335085	0.001
**115**	2-MHB-K17	1	VEGFR	0.438264	0
**116**	2-MHB-K19	1	VEGFR	0.052891	0.997
**117**	2-MHB-L12	0.1	VEGFR	0.464316	0
**118**	2-MHB-L13	1	VEGFR	0.237745	0.287

**119**	2-MHB-L21	0.1	VEGFR	0.288752	0.101
**120**	2-MHB-M1	1	VEGFR	0.349981	0.033
**121**	2-MHB-M1	10	VEGFR	0.314273	0.109
**122**	2-MHB-M1	10	VEGFR	0.398108	0.004
**123**	2-MHB-M2	1	VEGFR	0.363617	0
**124**	2-MHB-N12	10	VEGFR	0.385317	0
**125**	2-MHB-N13	100	VEGFR	0.141854	0.934
**126**	2-MHB-N17	100	VEGFR	0.218275	0.119
**127**	2-MHB-N21	10	VEGFR	0.183529	0.351
**128**	2-MHB-O12	100	VEGFR	0.156386	0.938
**129**	2-MHB-O17	1000	VEGFR	0.182143	0.409
**130**	2-MHB-O21	100	VEGFR	0.237157	0.008
**131**	2-MHB-P12	1000	VEGFR	0.240337	0.655
**132**	2-MHB-P13	1000	VEGFR	0.052358	0.999
**133**	2-MHB-P17	10000	VEGFR	0.245043	0.517
**134**	2-MHB-P21	1000	VEGFR	0.047276	0.998
**135**	3-MHB-A3-	1000	VEGFR	0.132224	0.836
**136**	3-MHB-A6-	1000	VEGFR	0.201127	0.926
**137**	3-MHB-A18	1000	VEGFR	0.061585	1
**138**	3-MHB-B3-	100	VEGFR	0.157615	0.897
**139**	3-MHB-B6-	100	VEGFR	0.110149	0.975
**140**	3-MHB-B18	100	VEGFR	0.164391	0.838
**141**	3-MHB-C3-	10	VEGFR	0.256218	0.439
**142**	3-MHB-C6-	10	VEGFR	0.230087	0.44
**143**	3-MHB-C18	10	VEGFR	0.270212	0.274
**144**	3-MHB-D3-	1	VEGFR	0.245711	0.15
**145**	3-MHB-D6-	1	VEGFR	0.166688	0.763
**146**	3-MHB-D18	1	VEGFR	0.317427	0.004
**147**	3-MHB-E3-	0.1	VEGFR	0.289792	0.018
**148**	3-MHB-E6-	0.1	VEGFR	0.237117	0.151
**149**	3-MHB-E18	0.1	VEGFR	0.247339	0.28
**150**	3-MHB-F18	1000	VEGFR	0.170844	0.862
**151**	3-MHB-G18	100	VEGFR	0.335491	0.001
**152**	3-MHB-H18	10	VEGFR	0.266494	0.003
**153**	3-MHB-I18-	1	VEGFR	0.169383	0.442

**154**	3-MHB-J18	0.1	VEGFR	0.13926	0.8
**155**	4-MHB-A12	10000	VEGFR	0.069149	1
**156**	4-MHB-A15	2500	VEGFR	0.194746	0.845
**157**	4-MHB-A20	10000	VEGFR	0.378112	0
**158**	4-MHB-B12	1000	VEGFR	0.084992	0.999
**159**	4-MHB-B15	250	VEGFR	0.170814	0.448
**160**	4-MHB-B20	1000	VEGFR	0.216252	0.133
**161**	4-MHB-C15	25	VEGFR	0.18605	0.362
**162**	4-MHB-D12	100	VEGFR	0.174174	0.569
**163**	4-MHB-D15	2.5	VEGFR	0.240758	0.255
**164**	4-MHB-D20	100	VEGFR	0.220317	0.142
**165**	4-MHB-E12	10	VEGFR	0.213086	0.262
**166**	4-MHB-E20	10	VEGFR	0.185346	0.607
**167**	4-MHB-F12	1	VEGFR	0.339986	0.004
**168**	4-MHB-F15	0.25	VEGFR	0.265573	0.251
**169**	4-MHB-F20	1	VEGFR	0.32142	0.011
**170**	4-MHB-L16	1	VEGFR	0.118347	0.979
**171**	4-MHB-M1[illegible]	10	VEGFR	0.228406	0.378
**172**	4-MHB-N16	100	VEGFR	0.310634	0.037
**173**	4-MHB-O16	1000	VEGFR	0.174018	0.361
**174**	4-MHB-P16	10000	VEGFR	0.138252	0.859
**175**	2-MHB-L10	1	PI3K	0.148759	0.907
**176**	2-MHB-M1[illegible]	10	PI3K	0.205024	0.504
**177**	2-MHB-N10	100	PI3K	0.147464	0.553
**178**	2-MHB-O10	1000	PI3K	0.292421	0.003
**179**	2-MHB-P10	10000	PI3K	0.360364	0.005
**180**	3-MHB-A16	2500	PI3K	0.202814	0.661
**181**	3-MHB-C16	250	PI3K	0.133739	0.787
**182**	3-MHB-D16	25	PI3K	0.146807	0.25
**183**	3-MHB-E16	2.5	PI3K	0.212039	0.475
**184**	3-MHB-F16	0.25	PI3K	0.407546	0
**185**	3-MHB-F17	100000	PI3K	0.197318	0.808
**186**	3-MHB-F19	500	PI3K	0.19842	0.696
**187**	3-MHB-G17	10000	PI3K	0.157063	0.864
**188**	3-MHB-G19	50	PI3K	0.049271	0.994

**189**	3-MHB-H17	1000	PI3K	0.12944	0.859
**190**	3-MHB-I17-	100	PI3K	0.233292	0.05
**191**	3-MHB-I19-	5	PI3K	0.163339	0.854
**192**	3-MHB-J17	10	PI3K	0.313662	0.002
**193**	3-MHB-J19	0.5	PI3K	0.265748	0.21
**194**	3-MHB-K19	0.05	PI3K	0.291273	0.015
**195**	3-MHB-L8-	1	PI3K	0.15065	0.943
**196**	3-MHB-L21	0.1	PI3K	0.35193	0
**197**	3-MHB-M8-	10	PI3K	0.161788	0.38
**198**	3-MHB-M2	1	PI3K	0.241085	0.12
**199**	3-MHB-N8-	100	PI3K	0.301952	0.07
**200**	3-MHB-N21	10	PI3K	0.3416	0.001
**201**	3-MHB-O8-	1000	PI3K	0.287158	0.194
**202**	3-MHB-O21	100	PI3K	0.324796	0.017
**203**	3-MHB-P8-	10000	PI3K	0.319428	0.026
**204**	3-MHB-P21	1000	PI3K	0.319384	0.016
**205**	4-MHB-A19	2500	PI3K	0.378538	0
**206**	4-MHB-B19	250	PI3K	0.366994	0.004
**207**	4-MHB-C19	25	PI3K	0.165892	0.27
**208**	4-MHB-D19	2.5	PI3K	0.174402	0.192
**209**	4-MHB-E19	0.25	PI3K	0.173898	0.167
**210**	4-MHB-G2-	2500	PI3K	0.103737	0.995
**211**	4-MHB-G5-	10000	PI3K	0.265324	0.286
**212**	4-MHB-G14	100	PI3K	0.320248	0.09
**213**	4-MHB-G20	10000	PI3K	0.265439	0.621
**214**	4-MHB-H2-	250	PI3K	0.076028	0.95
**215**	4-MHB-H5-	1000	PI3K	0.171087	0.889
**216**	4-MHB-H14	10	PI3K	0.303036	0.073
**217**	4-MHB-H20	1000	PI3K	0.330488	0.043
**218**	4-MHB-I2-T	25	PI3K	0.088247	0.999
**219**	4-MHB-I5-S	100	PI3K	0.119075	0.991
**220**	4-MHB-I14-	1	PI3K	0.158542	0.149
**221**	4-MHB-I20-	100	PI3K	0.29317	0.033
**222**	4-MHB-J2-T	2.5	PI3K	0.149317	0.653
**223**	4-MHB-J5-S	10	PI3K	0.270168	0.151

**224**	4-MHB-J20	10	PI3K	0.283854	0.012
**225**	4-MHB-K2-	0.25	PI3K	0.122776	0.948
**226**	4-MHB-K4-	0.1	PI3K	0.175649	0.272
**227**	4-MHB-K5-	1	PI3K	0.112915	0.614
**228**	4-MHB-K14	0.1	PI3K	0.192955	0.056
**229**	4-MHB-K20	1	PI3K	0.280416	0.004
**230**	4-MHB-L4-	1	PI3K	0.122562	0.6
**231**	4-MHB-L14	0.1	PI3K	0.270948	0.104
**232**	4-MHB-L15	1	PI3K	0.185648	0.129
**233**	4-MHB-L21	0.1	PI3K	0.339488	0.007
**234**	4-MHB-M1	1	PI3K	0.245244	0.005
**235**	4-MHB-M1	10	PI3K	0.105416	0.661
**236**	4-MHB-M2	1	PI3K	0.233907	0.01
**237**	4-MHB-N4-	10	PI3K	0.20032	0.661
**238**	4-MHB-N1	10	PI3K	0.358354	0
**239**	4-MHB-N1	100	PI3K	0.201377	0.047
**240**	4-MHB-N21	10	PI3K	0.388176	0
**241**	4-MHB-O4-	100	PI3K	0.302652	0.227
**242**	4-MHB-O1	100	PI3K	0.32025	0.036
**243**	4-MHB-O1	1000	PI3K	0.285034	0.007
**244**	4-MHB-O21	100	PI3K	0.316332	0.085
**245**	4-MHB-P4-	1000	PI3K	0.32236	0.12
**246**	4-MHB-P14	1000	PI3K	0.199086	0.708
**247**	4-MHB-P15	10000	PI3K	0.30188	0.028
**248**	4-MHB-P21	1000	PI3K	0.316089	0.019
**249**	5-MHB-A6-	2500	PI3K	0.298596	0.15
**250**	5-MHB-A7-	1000	PI3K	0.256202	0.304
**251**	5-MHB-A16	2500	PI3K	0.354726	0.003
**252**	5-MHB-A17	10000	PI3K	0.200974	0.697
**253**	5-MHB-B6-	250	PI3K	0.315198	0.088
**254**	5-MHB-B7-	100	PI3K	0.099829	0.971
**255**	5-MHB-B17	1000	PI3K	0.279259	0.022
**256**	5-MHB-C6-	25	PI3K	0.133071	0.841
**257**	5-MHB-C7-	10	PI3K	0.116812	0.639
**258**	5-MHB-C16	250	PI3K	0.277202	0.028

**259**	5-MHB-C17	100	PI3K	0.235609	0.088
**260**	5-MHB-D6-	2.5	PI3K	0.111014	0.817
**261**	5-MHB-D7-	1	PI3K	0.250567	0.29
**262**	5-MHB-D16	25	PI3K	0.303628	0.011
**263**	5-MHB-D17	10	PI3K	0.166572	0.231
**264**	5-MHB-E6-I	0.25	PI3K	0.239384	0.254
**265**	5-MHB-E7-/	0.1	PI3K	0.327023	0.078
**266**	5-MHB-E16	2.5	PI3K	0.116454	0.643
**267**	5-MHB-E17	1	PI3K	0.234388	0.191
**268**	5-MHB-F11	10000	PI3K	0.394714	0.001
**269**	5-MHB-F16	0.25	PI3K	0.154233	0.64
**270**	5-MHB-G9-	10000	PI3K	0.379547	0
**271**	5-MHB-G11	1000	PI3K	0.286744	0.039
**272**	5-MHB-H9-	1000	PI3K	0.368208	0
**273**	5-MHB-H11	100	PI3K	0.172866	0.503
**274**	5-MHB-I9-S	100	PI3K	0.372198	0
**275**	5-MHB-I11-	10	PI3K	0.157151	0.476
**276**	5-MHB-J9-S	10	PI3K	0.11202	0.718
**277**	5-MHB-J11	1	PI3K	0.136138	0.929
**278**	5-MHB-K9-S	1	PI3K	0.24325	0.496
**279**	5-MHB-L14	0.1	PI3K	0.295619	0.003
**280**	5-MHB-L20	1	PI3K	0.159515	0.645
**281**	5-MHB-L23	0.1	PI3K	0.182601	0.189
**282**	5-MHB-M14	1	PI3K	0.193838	0.623
**283**	5-MHB-M20	10	PI3K	0.178441	0.098
**284**	5-MHB-M23	1	PI3K	0.264472	0.088
**285**	5-MHB-N14	10	PI3K	0.203429	0.516
**286**	5-MHB-N20	100	PI3K	0.178216	0.375
**287**	5-MHB-N23	10	PI3K	0.363344	0
**288**	5-MHB-O14	100	PI3K	0.297863	0.012
**289**	5-MHB-O20	1000	PI3K	0.344881	0.019
**290**	5-MHB-O23	100	PI3K	0.343134	0.005
**291**	5-MHB-P14	1000	PI3K	0.342293	0.006
**292**	5-MHB-P20	10000	PI3K	0.331229	0.027
**293**	5-MHB-P23	1000	PI3K	0.217646	0.791

**294**	6-MHB-A8-[illegible]	10000	PI3K	0.329091	0.029
**295**	6-MHB-B8-[illegible]	1000	PI3K	0.073018	0.98
**296**	6-MHB-C8-[illegible]	100	PI3K	0.095432	0.995
**297**	6-MHB-D8-	10	PI3K	0.186662	0.846
**298**	6-MHB-E8-[illegible]	1	PI3K	0.085788	0.982
**299**	6-MHB-L6-[illegible]	1	PI3K	0.149171	0.958
**300**	6-MHB-M6-	10	PI3K	0.133181	0.916
**301**	6-MHB-N6-	100	PI3K	0.181377	0.839
**302**	6-MHB-O6-	1000	PI3K	0.329583	0.052
**303**	6-MHB-P6-[illegible]	10000	PI3K	0.222162	0.978
**304**	1-MHB-F11	10000	Topoisome	0.5254	0.007
**305**	1-MHB-G1[illegible]	1000	Topoisome	0.525712	0
**306**	1-MHB-G2[illegible]	1000	Topoisome	0.320122	0.275
**307**	1-MHB-H1[illegible]	100	Topoisome	0.516307	0
**308**	1-MHB-H2[illegible]	100	Topoisome	0.454779	0.002
**309**	1-MHB-I11-	10	Topoisome	0.125575	0.751
**310**	1-MHB-I20-	10	Topoisome	0.12117	0.721
**311**	1-MHB-J11	1	Topoisome	0.283736	0.39
**312**	1-MHB-J20	1	Topoisome	0.116338	0.708
**313**	1-MHB-K11	1	Topoisome	0.462273	0
**314**	1-MHB-K2[illegible]	0.1	Topoisome	0.445046	0.001
**315**	1-MHB-L11	10	Topoisome	0.521536	0.001
**316**	1-MHB-L14	1	Topoisome	0.456158	0.001
**317**	1-MHB-M1[illegible]	100	Topoisome	0.469285	0.016
**318**	1-MHB-M1[illegible]	10	Topoisome	0.403377	0.005
**319**	1-MHB-N1[illegible]	100	Topoisome	0.510276	0
**320**	1-MHB-O1[illegible]	1000	Topoisome	0.384575	0.073
**321**	1-MHB-O1[illegible]	1000	Topoisome	0.532604	0
**322**	1-MHB-P11	10000	Topoisome	0.285252	0.522
**323**	1-MHB-P14	10000	Topoisome	0.356018	0.099
**324**	3-MHB-A11	10000	Topoisome	0.526486	0
**325**	3-MHB-B11	1000	Topoisome	0.538544	0
**326**	3-MHB-C11	100	Topoisome	0.397115	0.012
**327**	3-MHB-D1[illegible]	10	Topoisome	0.393294	0.003
**328**	3-MHB-E11	1	Topoisome	0.482929	0

**329**	3-MHB-G9-	1000	Topoisome	0.345473	0.181
**330**	3-MHB-G10	10000	Topoisome	0.373989	0.055
**331**	3-MHB-H9-	100	Topoisome	0.536957	0
**332**	3-MHB-H10	1000	Topoisome	0.504345	0
**333**	3-MHB-I9-D	10	Topoisome	0.419446	0.009
**334**	3-MHB-I10-	100	Topoisome	0.179659	0.627
**335**	3-MHB-J9-D	1	Topoisome	0.292013	0.021
**336**	3-MHB-J10	10	Topoisome	0.254487	0.082
**337**	3-MHB-K7-	0.1	Topoisome	0.160793	0.389
**338**	3-MHB-K9-	0.1	Topoisome	0.441896	0.001
**339**	3-MHB-K10	1	Topoisome	0.439746	0.001
**340**	3-MHB-L6-	0.1	Topoisome	0.369202	0.027
**341**	3-MHB-L7-	1	Topoisome	0.358753	0.02
**342**	3-MHB-L9-	0.5	Topoisome	0.255782	0.207
**343**	3-MHB-L10	0.1	Topoisome	0.406814	0.009
**344**	3-MHB-L16	1	Topoisome	0.130239	0.849
**345**	3-MHB-M6-	1	Topoisome	0.333368	0.013
**346**	3-MHB-M7-	10	Topoisome	0.46096	0.003
**347**	3-MHB-M9-	5	Topoisome	0.429523	0.001
**348**	3-MHB-M10	1	Topoisome	0.370216	0.008
**349**	3-MHB-M10	10	Topoisome	0.450055	0.002
**350**	3-MHB-N6-	10	Topoisome	0.237298	0.264
**351**	3-MHB-N9-	50	Topoisome	0.448469	0.002
**352**	3-MHB-N10	10	Topoisome	0.493837	0
**353**	3-MHB-N16	100	Topoisome	0.089692	0.943
**354**	3-MHB-O6-	100	Topoisome	0.5213	0
**355**	3-MHB-O7-	100	Topoisome	0.567589	0
**356**	3-MHB-O9-	500	Topoisome	0.583458	0
**357**	3-MHB-O10	100	Topoisome	0.511568	0
**358**	3-MHB-O16	1000	Topoisome	0.130324	0.895
**359**	3-MHB-P6-	1000	Topoisome	0.358264	0.077
**360**	3-MHB-P7-	1000	Topoisome	0.337366	0.226
**361**	3-MHB-P9-	5000	Topoisome	0.384729	0.205
**362**	3-MHB-P10	1000	Topoisome	0.435499	0.068
**363**	3-MHB-P16	10000	Topoisome	0.287221	0.247

**364**	1-MHB-A1(	10000	Mitotic	0.645202	0
**365**	1-MHB-A13	1000	Mitotic	0.665155	0
**366**	1-MHB-A18	1000	Mitotic	0.661117	0
**367**	1-MHB-B1(	1000	Mitotic	0.623986	0
**368**	1-MHB-B13	100	Mitotic	0.221975	0.499
**369**	1-MHB-B18	100	Mitotic	0.679426	0
**370**	1-MHB-C1(	100	Mitotic	0.666376	0
**371**	1-MHB-C13	10	Mitotic	0.601881	0
**372**	1-MHB-C18	10	Mitotic	0.548273	0
**373**	1-MHB-D1(	10	Mitotic	0.26894	0.497
**374**	1-MHB-D1:	1	Mitotic	0.324522	0.167
**375**	1-MHB-D18	1	Mitotic	0.405803	0.028
**376**	1-MHB-E10	1	Mitotic	0.375009	0.028
**377**	1-MHB-E13	0.1	Mitotic	0.238599	0.212
**378**	1-MHB-E18	0.1	Mitotic	0.279894	0.245
**379**	1-MHB-F13	1000	Mitotic	0.625361	0
**380**	1-MHB-G1:	100	Mitotic	0.59676	0
**381**	1-MHB-G15	1000	Mitotic	0.655756	0
**382**	1-MHB-H1:	10	Mitotic	0.415952	0.013
**383**	1-MHB-H15	100	Mitotic	0.623428	0
**384**	1-MHB-I13-	1	Mitotic	0.200587	0.441
**385**	1-MHB-I15-	10	Mitotic	0.62208	0
**386**	1-MHB-J13	0.1	Mitotic	0.225326	0.379
**387**	1-MHB-J15	1	Mitotic	0.504093	0
**388**	1-MHB-K7-'	0.1	Mitotic	0.356062	0.131
**389**	1-MHB-K15	0.1	Mitotic	0.282127	0.107
**390**	1-MHB-L7-\	1	Mitotic	0.40178	0.02
**391**	1-MHB-L20	0.1	Mitotic	0.17313	0.48
**392**	1-MHB-M7-	10	Mitotic	0.331854	0.099
**393**	1-MHB-M2(	1	Mitotic	0.29666	0.055
**394**	1-MHB-N2(	10	Mitotic	0.256087	0.136
**395**	1-MHB-O7-	100	Mitotic	0.577028	0
**396**	1-MHB-O2(	100	Mitotic	0.155744	0.58
**397**	1-MHB-P7-'	1000	Mitotic	0.59274	0
**398**	1-MHB-P2(	1000	Mitotic	0.548013	0

**399**	3-MHB-A7-l	1000	Mitotic	0.570176	0.001
**400**	3-MHB-B7-l	100	Mitotic	0.664082	0
**401**	3-MHB-C7-l	10	Mitotic	0.562049	0.002
**402**	3-MHB-D7-	1	Mitotic	0.23549	0.648
**403**	3-MHB-E7-l	0.1	Mitotic	0.305501	0.182
**404**	6-MHB-L19	1	Mitotic	0.153624	0.829
**405**	6-MHB-M1!	10	Mitotic	0.316418	0.094
**406**	6-MHB-N1!	100	Mitotic	0.140415	0.975
**407**	6-MHB-O1!	1000	Mitotic	0.603863	0.001
**408**	6-MHB-P1!	10000	Mitotic	0.664867	0
**409**	2-MHB-A12	250	MEK1/2	0.779207	0
**410**	2-MHB-B12	25	MEK1/2	0.762211	0
**411**	2-MHB-D12	2.5	MEK1/2	0.737536	0
**412**	2-MHB-E12	0.25	MEK1/2	0.709556	0
**413**	2-MHB-F12	0.025	MEK1/2	0.587325	0
**414**	2-MHB-F14	1000	MEK1/2	0.716329	0
**415**	2-MHB-G14	100	MEK1/2	0.744702	0
**416**	2-MHB-H14	10	MEK1/2	0.769848	0
**417**	2-MHB-I14-	1	MEK1/2	0.397775	0.238
**418**	2-MHB-K14	0.1	MEK1/2	0.375327	0.172
**419**	2-MHB-L20	1	MEK1/2	0.108851	0.998
**420**	2-MHB-M2(	10	MEK1/2	0.788241	0
**421**	2-MHB-N2(	100	MEK1/2	0.743354	0
**422**	2-MHB-O2(	1000	MEK1/2	0.734846	0
**423**	2-MHB-P2(	10000	MEK1/2	0.702326	0
**424**	4-MHB-A1(	1000	MEK1/2	0.720377	0
**425**	4-MHB-A13	1000	MEK1/2	0.72141	0
**426**	4-MHB-B1(	100	MEK1/2	0.771902	0
**427**	4-MHB-B13	100	MEK1/2	0.770275	0
**428**	4-MHB-C1(	10	MEK1/2	0.780819	0
**429**	4-MHB-C13	10	MEK1/2	0.789553	0
**430**	4-MHB-D1(	1	MEK1/2	0.437974	0.025
**431**	4-MHB-D13	1	MEK1/2	0.745769	0
**432**	4-MHB-E10	0.1	MEK1/2	0.572531	0
**433**	4-MHB-E13	0.1	MEK1/2	0.320684	0.241

**434**	4-MHB-L19	0.25	MEK1/2	0.244833	0.561
**435**	4-MHB-M1!	2.5	MEK1/2	0.179681	0.718
**436**	4-MHB-N1!	25	MEK1/2	0.363442	0.224
**437**	4-MHB-O1!	250	MEK1/2	0.710132	0
**438**	4-MHB-P19	2500	MEK1/2	0.711362	0
**439**	1-MHB-L2-(	1	PARP	0.743892	0
**440**	1-MHB-L6-I	1	PARP	0.656378	0
**441**	1-MHB-M2-	10	PARP	0.217397	0.554
**442**	1-MHB-M6-	10	PARP	0.677508	0
**443**	1-MHB-N2-	100	PARP	0.668737	0
**444**	1-MHB-N6-	100	PARP	0.56473	0
**445**	1-MHB-O2-	1000	PARP	0.36345	0.307
**446**	1-MHB-O6-	1000	PARP	0.710832	0
**447**	1-MHB-P2-(	10000	PARP	0.635332	0
**448**	1-MHB-P6-I	10000	PARP	0.598062	0.001
**449**	7-MHB-A3-'	1000	PARP	0.605532	0
**450**	7-MHB-B2-'	10000	PARP	0.712199	0
**451**	7-MHB-B3-'	100	PARP	0.564711	0.002
**452**	7-MHB-C2-'	1000	PARP	0.482076	0.009
**453**	7-MHB-C3-'	10	PARP	0.540167	0.002
**454**	7-MHB-D2-	100	PARP	0.662887	0
**455**	7-MHB-D3-	1	PARP	0.427812	0.136
**456**	7-MHB-E2-\	10	PARP	0.705033	0
**457**	7-MHB-E3-'	0.1	PARP	0.559932	0.002
**458**	7-MHB-F2-\	1	PARP	0.694569	0
**459**	7-MHB-G2-	10000	PARP	0.609758	0
**460**	7-MHB-H2-	1000	PARP	0.715358	0
**461**	7-MHB-I2-N	100	PARP	0.630505	0
**462**	7-MHB-J2-N	10	PARP	0.515876	0.01
**463**	7-MHB-K2-I	1	PARP	0.678411	0
**464**	3-MHB-A19	1000	CDK	0.479975	0.007
**465**	3-MHB-B19	100	CDK	0.489638	0.001
**466**	3-MHB-B23	2500	CDK	0.223041	0.346
**467**	3-MHB-C19	10	CDK	0.284434	0.323
**468**	3-MHB-C23	250	CDK	0.287397	0.291

**469**	3-MHB-D19	1	CDK	0.186648	0.48
**470**	3-MHB-D23	25	CDK	0.157655	0.691
**471**	3-MHB-E19	0.1	CDK	0.263978	0.449
**472**	3-MHB-E23	2.5	CDK	0.162184	0.615
**473**	3-MHB-F23	0.25	CDK	0.174342	0.65
**474**	3-MHB-K17	1	CDK	0.136953	0.913
**475**	3-MHB-L19	1	CDK	0.158539	0.92
**476**	3-MHB-M1	10	CDK	0.322091	0.315
**477**	3-MHB-M19	10	CDK	0.194529	0.258
**478**	3-MHB-N17	100	CDK	0.358976	0.048
**479**	3-MHB-N19	100	CDK	0.229651	0.578
**480**	3-MHB-O17	1000	CDK	0.420554	0.002
**481**	3-MHB-O19	1000	CDK	0.292967	0.088
**482**	3-MHB-P17	10000	CDK	0.192673	0.857
**483**	3-MHB-P19	10000	CDK	0.382545	0.08
**484**	4-MHB-A4-	10000	CDK	0.4478	0.044
**485**	4-MHB-A8-	10000	CDK	0.461738	0.003
**486**	4-MHB-B4-	1000	CDK	0.44908	0.048
**487**	4-MHB-B8-	1000	CDK	0.185215	0.928
**488**	4-MHB-C4-	100	CDK	0.295416	0.145
**489**	4-MHB-C8-	100	CDK	0.297149	0.348
**490**	4-MHB-D4-	10	CDK	0.319332	0.219
**491**	4-MHB-D8-	10	CDK	0.301664	0.285
**492**	4-MHB-E4-	1	CDK	0.217476	0.175
**493**	4-MHB-E8-	1	CDK	0.230929	0.556
**494**	4-MHB-F4-	10000	CDK	0.224071	0.696
**495**	4-MHB-F22	10000	CDK	0.488584	0.003
**496**	4-MHB-G4-	1000	CDK	0.390655	0.027
**497**	4-MHB-G22	1000	CDK	0.502758	0.007
**498**	4-MHB-H4-	100	CDK	0.210014	0.37
**499**	4-MHB-H22	100	CDK	0.359369	0.123
**500**	4-MHB-I4-S	10	CDK	0.324183	0.149
**501**	4-MHB-I22-	10	CDK	0.204747	0.241
**502**	4-MHB-J4-	1	CDK	0.328489	0.077
**503**	4-MHB-J22	1	CDK	0.180352	0.438

504	5-MHB-A19	10000	CDK	0.181315	0.956
505	5-MHB-B19	1000	CDK	0.187277	0.93
506	5-MHB-C19	100	CDK	0.372384	0.007
507	5-MHB-D19	10	CDK	0.205799	0.121
508	5-MHB-E19	1	CDK	0.196128	0.468
509	5-MHB-K17	1	CDK	0.176526	0.816
510	5-MHB-M17	10	CDK	0.232486	0.568
511	5-MHB-N17	100	CDK	0.353386	0.009
512	5-MHB-O17	1000	CDK	0.157825	0.976
513	5-MHB-P17	10000	CDK	0.183277	0.801
514	6-MHB-A17	1000	CDK	0.301707	0.246
515	6-MHB-B17	100	CDK	0.347783	0.054
516	6-MHB-C17	10	CDK	0.296102	0.214
517	6-MHB-D17	1	CDK	0.18537	0.461
518	6-MHB-E17	0.1	CDK	0.20024	0.798
519	6-MHB-L15	1	CDK	0.207568	0.718
520	6-MHB-M15	10	CDK	0.235965	0.307
521	6-MHB-N15	100	CDK	0.203417	0.827
522	6-MHB-O15	1000	CDK	0.471702	0.028
523	6-MHB-P15	10000	CDK	0.417041	0.048
524	7-MHB-A21	10000	BET	0.57854	0
525	7-MHB-A22	30000	BET	0.636404	0
526	7-MHB-B21	1000	BET	0.30493	0.167
527	7-MHB-B22	3000	BET	0.523304	0
528	7-MHB-C21	100	BET	0.172674	0.569
529	7-MHB-C22	300	BET	0.231819	0.318
530	7-MHB-D21	10	BET	0.156128	0.606
531	7-MHB-D22	30	BET	0.18427	0.639
532	7-MHB-E21	1	BET	0.266619	0.451
533	7-MHB-E22	3	BET	0.275677	0.401
534	7-MHB-G10	10000	BET	0.664363	0
535	7-MHB-G15	10000	BET	0.645199	0
536	7-MHB-H10	1000	BET	0.639593	0
537	7-MHB-H15	1000	BET	0.616937	0
538	7-MHB-I10-	100	BET	0.59533	0

**539**	7-MHB-I15-	100	BET	0.62839	0
**540**	7-MHB-J10	10	BET	0.139728	0.756
**541**	7-MHB-J15	10	BET	0.24159	0.299
**542**	7-MHB-K10	1	BET	0.214444	0.555
**543**	7-MHB-K13	1	BET	0.435871	0.017
**544**	7-MHB-K15	1	BET	0.208436	0.727
**545**	7-MHB-L12	1	BET	0.25173	0.55
**546**	7-MHB-L13	10	BET	0.618095	0
**547**	7-MHB-L20	1	BET	0.477658	0.003
**548**	7-MHB-L23	0.03	BET	0.540346	0
**549**	7-MHB-M1:	10	BET	0.411239	0.029
**550**	7-MHB-M1:	100	BET	0.649796	0
**551**	7-MHB-M2(	10	BET	0.354182	0.298
**552**	7-MHB-M2:	0.3	BET	0.123823	0.743
**553**	7-MHB-N12	100	BET	0.12682	0.933
**554**	7-MHB-N13	1000	BET	0.653241	0
**555**	7-MHB-N2(	100	BET	0.555554	0
**556**	7-MHB-N23	3	BET	0.428395	0.002
**557**	7-MHB-O12	1000	BET	0.592058	0
**558**	7-MHB-O2(	1000	BET	0.666213	0
**559**	7-MHB-O23	30	BET	0.527867	0
**560**	7-MHB-P12	10000	BET	0.671461	0
**561**	7-MHB-P13	10000	BET	0.602694	0
**562**	7-MHB-P2(	10000	BET	0.62552	0
**563**	7-MHB-P23	300	BET	0.593792	0
**564**	8-MHB-K22	1	BET	0.398642	0.014
**565**	8-MHB-L22	10	BET	0.61144	0
**566**	8-MHB-M2:	100	BET	0.541271	0
**567**	8-MHB-N22	1000	BET	0.581793	0
**568**	8-MHB-O22	10000	BET	0.615259	0
**569**	1-MHB-A3-'	10000	HDAC	0.264472	0.451
**570**	1-MHB-B3-'	1000	HDAC	0.378566	0.003
**571**	1-MHB-C3-'	100	HDAC	0.275005	0.001
**572**	1-MHB-D3-	10	HDAC	0.230235	0.548
**573**	1-MHB-E3-\	1	HDAC	0.143547	0.913

**574**	1-MHB-L12	0.1	HDAC	0.325181	0.001
**575**	1-MHB-M1:	1	HDAC	0.407049	0
**576**	1-MHB-N12	10	HDAC	0.261236	0.407
**577**	1-MHB-O12	100	HDAC	0.254321	0.528
**578**	1-MHB-P12	1000	HDAC	0.273078	0.398
**579**	3-MHB-A4-I	1000	HDAC	0.279003	0.571
**580**	3-MHB-B4-I	100	HDAC	0.208461	0.838
**581**	3-MHB-C4-I	10	HDAC	0.38612	0.001
**582**	3-MHB-D4-	1	HDAC	0.196036	0.18
**583**	3-MHB-E4-I	0.1	HDAC	0.395759	0
**584**	3-MHB-F7-(	1000	HDAC	0.291009	0.617
**585**	3-MHB-G7-	100	HDAC	0.235172	0.762
**586**	3-MHB-G12	1000000	HDAC	0.092784	0.702
**587**	3-MHB-H7-	10	HDAC	0.477363	0
**588**	3-MHB-H12	100000	HDAC	0.513247	0
**589**	3-MHB-I7-(	1	HDAC	0.339046	0
**590**	3-MHB-I12-	10000	HDAC	0.232259	0.114
**591**	3-MHB-J7-(	0.1	HDAC	0.36253	0.002
**592**	3-MHB-J12	1000	HDAC	0.362861	0
**593**	3-MHB-K3-I	1	HDAC	0.192942	0.825
**594**	3-MHB-K12	100	HDAC	0.431993	0
**595**	3-MHB-L3-I	10	HDAC	0.256922	0.232
**596**	3-MHB-M3-	100	HDAC	0.394248	0
**597**	3-MHB-N3-	1000	HDAC	0.227825	0.838
**598**	3-MHB-O3-	10000	HDAC	0.079117	0.984
**599**	7-MHB-A5-I	10000	HDAC	0.342234	0.182
**600**	7-MHB-A7-(	10000	HDAC	0.341808	0.215
**601**	7-MHB-A9-(	1000	HDAC	0.331167	0.109
**602**	7-MHB-A12	10000	HDAC	0.260419	0.692
**603**	7-MHB-B5-I	1000	HDAC	0.331614	0.107
**604**	7-MHB-B12	1000	HDAC	0.371859	0.003
**605**	7-MHB-C5-I	100	HDAC	0.048589	0.994
**606**	7-MHB-C7-(	100	HDAC	0.343948	0.041
**607**	7-MHB-C9-(	100	HDAC	0.495999	0
**608**	7-MHB-D7-	10	HDAC	0.311054	0.208

**609**	7-MHB-D9-	10	HDAC	0.490233	0
**610**	7-MHB-D12	100	HDAC	0.132268	0.809
**611**	7-MHB-E5-I	10	HDAC	0.5111	0
**612**	7-MHB-E7-(	1	HDAC	0.534264	0
**613**	7-MHB-E9-(	1	HDAC	0.341703	0
**614**	7-MHB-E12	10	HDAC	0.28011	0.011
**615**	7-MHB-F5-I	1	HDAC	0.468609	0
**616**	7-MHB-F7-I	10000	HDAC	0.365766	0.024
**617**	7-MHB-F9-(	0.1	HDAC	0.486601	0
**618**	7-MHB-F12	1	HDAC	0.154893	0.804
**619**	7-MHB-F19	10000	HDAC	0.397293	0
**620**	7-MHB-G7-	1000	HDAC	0.377429	0.009
**621**	7-MHB-G19	1000	HDAC	0.143357	0.218
**622**	7-MHB-H7-	100	HDAC	0.519109	0
**623**	7-MHB-I7-F	10	HDAC	0.374375	0.004
**624**	7-MHB-I19-	100	HDAC	0.213197	0.034
**625**	7-MHB-J7-F	1	HDAC	0.291812	0.041
**626**	7-MHB-J19	10	HDAC	0.26596	0.147
**627**	7-MHB-K4-I	1	HDAC	0.146039	0.937
**628**	7-MHB-K11	1	HDAC	0.196481	0.63
**629**	7-MHB-K18	1	HDAC	0.419773	0.001
**630**	7-MHB-K19	1	HDAC	0.136843	0.356
**631**	7-MHB-L2-T	0.1	HDAC	0.151285	0.541
**632**	7-MHB-L4-I	10	HDAC	0.136134	0.691
**633**	7-MHB-L5-I	1	HDAC	0.199703	0.065
**634**	7-MHB-L8-A	1	HDAC	0.430107	0
**635**	7-MHB-L10	1	HDAC	0.446839	0
**636**	7-MHB-L11	10	HDAC	0.394514	0
**637**	7-MHB-L14	1	HDAC	0.352042	0.011
**638**	7-MHB-L16	1	HDAC	0.469106	0
**639**	7-MHB-L18	10	HDAC	0.047485	0.991
**640**	7-MHB-M2-	1	HDAC	0.311458	0.11
**641**	7-MHB-M5-	10	HDAC	0.380152	0
**642**	7-MHB-M8-	10	HDAC	0.52879	0
**643**	7-MHB-M1(	10	HDAC	0.473259	0

**644**	7-MHB-M1[illegible]	100	HDAC	0.53479	0
**645**	7-MHB-M1[illegible]	10	HDAC	0.459241	0
**646**	7-MHB-M1[illegible]	10	HDAC	0.392692	0.001
**647**	7-MHB-M1[illegible]	100	HDAC	0.454409	0
**648**	7-MHB-N2-	10	HDAC	0.204609	0.697
**649**	7-MHB-N4-	100	HDAC	0.377067	0
**650**	7-MHB-N5-	100	HDAC	0.419823	0
**651**	7-MHB-N8-	100	HDAC	0.531486	0
**652**	7-MHB-N1[illegible]	100	HDAC	0.467114	0
**653**	7-MHB-N1[illegible]	100	HDAC	0.497141	0
**654**	7-MHB-N1[illegible]	100	HDAC	0.421231	0
**655**	7-MHB-N1[illegible]	1000	HDAC	0.385646	0.003
**656**	7-MHB-O2-	100	HDAC	0.127468	0.605
**657**	7-MHB-O4-	1000	HDAC	0.407454	0
**658**	7-MHB-O5-	1000	HDAC	0.263465	0.699
**659**	7-MHB-O8-	1000	HDAC	0.300692	0.522
**660**	7-MHB-O1[illegible]	1000	HDAC	0.522847	0
**661**	7-MHB-O11	1000	HDAC	0.265253	0.604
**662**	7-MHB-O1[illegible]	1000	HDAC	0.523296	0
**663**	7-MHB-O1[illegible]	1000	HDAC	0.319509	0
**664**	7-MHB-P2-[illegible]	1000	HDAC	0.418163	0
**665**	7-MHB-P4-[illegible]	10000	HDAC	0.27144	0.59
**666**	7-MHB-P5-[illegible]	10000	HDAC	0.321283	0.1
**667**	7-MHB-P8-[illegible]	10000	HDAC	0.267363	0.272
**668**	7-MHB-P1[illegible]	10000	HDAC	0.26923	0.629
**669**	7-MHB-P11	10000	HDAC	0.264221	0.294
**670**	7-MHB-P14	10000	HDAC	0.357984	0.01
**671**	7-MHB-P1[illegible]	10000	HDAC	0.481448	0
**672**	7-MHB-P1[illegible]	10000	HDAC	0.429992	0
**0**	2-O3B-A16-	10000	EGFR	0.555658	0
**1**	2-O3B-A19-	10000	EGFR	0.562443	0
**2**	2-O3B-B19-	1000	EGFR	0.5686	0
**3**	2-O3B-C16-	1000	EGFR	0.547195	0
**4**	2-O3B-C19-	100	EGFR	0.517166	0
**5**	2-O3B-D16	100	EGFR	0.586169	0

**6**	2-O3B-D19	10	EGFR	0.040714	0.994
**7**	2-O3B-E16-	10	EGFR	0.595502	0
**8**	2-O3B-E19-	1	EGFR	0.309483	0.023
**9**	2-O3B-F16-	1	EGFR	0.452169	0
**10**	2-O3B-K11-	0.1	EGFR	0.488646	0
**11**	2-O3B-L11-	1	EGFR	0.149303	0.806
**12**	2-O3B-L16-	0.25	EGFR	0.078074	0.998
**13**	2-O3B-L19-	0.1	EGFR	0.462456	0
**14**	2-O3B-M11	10	EGFR	0.5803	0
**15**	2-O3B-M16	2.5	EGFR	0.178081	0.366
**16**	2-O3B-M19	1	EGFR	0.462497	0
**17**	2-O3B-N16	25	EGFR	0.386302	0.021
**18**	2-O3B-N19	10	EGFR	0.400529	0.001
**19**	2-O3B-O11	100	EGFR	0.568991	0
**20**	2-O3B-O16	250	EGFR	0.563797	0
**21**	2-O3B-O19	100	EGFR	0.447001	0.001
**22**	2-O3B-P11-	1000	EGFR	0.559823	0
**23**	2-O3B-P16-	2500	EGFR	0.522546	0
**24**	2-O3B-P19-	1000	EGFR	0.607651	0
**25**	3-O3B-F21-	10000	EGFR	0.520544	0
**26**	3-O3B-G20	1000	EGFR	0.536805	0
**27**	3-O3B-G21	1000	EGFR	0.459613	0
**28**	3-O3B-H20	100	EGFR	0.535717	0
**29**	3-O3B-H21	100	EGFR	0.461668	0
**30**	3-O3B-I20-	10	EGFR	0.470567	0
**31**	3-O3B-I21-	10	EGFR	0.417454	0.002
**32**	3-O3B-J20-	1	EGFR	0.477504	0
**33**	3-O3B-J21-	1	EGFR	0.495277	0
**34**	3-O3B-K4-C	1	EGFR	0.062334	0.93
**35**	3-O3B-K18-	0.1	EGFR	0.498671	0
**36**	3-O3B-K20-	0.1	EGFR	0.170524	0.544
**37**	3-O3B-L4-C	10	EGFR	0.470112	0
**38**	3-O3B-L18-	1	EGFR	0.05794	0.943
**39**	3-O3B-M18	10	EGFR	0.623843	0
**40**	3-O3B-N4-C	100	EGFR	0.048804	0.944

**41**	3-O3B-N18	100	EGFR	0.606373	0
**42**	3-O3B-O4-(	1000	EGFR	0.580075	0
**43**	3-O3B-P4-(	10000	EGFR	0.299378	0.352
**44**	3-O3B-P18-	1000	EGFR	0.569908	0
**45**	4-O3B-F13-	1000	EGFR	0.53636	0
**46**	4-O3B-G13	100	EGFR	0.591985	0
**47**	4-O3B-G16	10000	EGFR	0.512215	0.005
**48**	4-O3B-H13	10	EGFR	0.575572	0
**49**	4-O3B-H16	1000	EGFR	0.594582	0
**50**	4-O3B-I13-	1	EGFR	0.302468	0.082
**51**	4-O3B-I16-	100	EGFR	0.471673	0
**52**	4-O3B-J13-	0.1	EGFR	0.319501	0.008
**53**	4-O3B-J16-	10	EGFR	0.099365	0.874
**54**	4-O3B-K7-I	1	EGFR	0.496868	0
**55**	4-O3B-K13-	0.1	EGFR	0.042865	0.967
**56**	4-O3B-K16-	1	EGFR	0.522831	0
**57**	4-O3B-L7-I	10	EGFR	0.501982	0
**58**	4-O3B-L13-	1	EGFR	0.5291	0
**59**	4-O3B-M7-	100	EGFR	0.48611	0
**60**	4-O3B-M13	10	EGFR	0.522348	0
**61**	4-O3B-N13	100	EGFR	0.542798	0
**62**	4-O3B-O7-I	1000	EGFR	0.51037	0
**63**	4-O3B-P7-I	10000	EGFR	0.501559	0
**64**	4-O3B-P13-	1000	EGFR	0.542412	0
**65**	5-O3B-F4-P	1000	EGFR	0.512839	0.001
**66**	5-O3B-F7-A	1000	EGFR	0.519699	0
**67**	5-O3B-G4-F	100	EGFR	0.533151	0
**68**	5-O3B-G7-A	100	EGFR	0.456653	0
**69**	5-O3B-H4-F	10	EGFR	0.54831	0
**70**	5-O3B-H7-A	10	EGFR	0.456243	0
**71**	5-O3B-I4-P	1	EGFR	0.434853	0.001
**72**	5-O3B-I7-A	1	EGFR	0.351814	0.037
**73**	5-O3B-J4-P	0.1	EGFR	0.427104	0
**74**	5-O3B-J7-A	0.1	EGFR	0.300028	0.22
**75**	5-O3B-K7-(	0.1	EGFR	0.319531	0.054

**76**	5-O3B-L7-C	1	EGFR	0.437249	0
**77**	5-O3B-M7-(	10	EGFR	0.380522	0.004
**78**	5-O3B-O7-(	100	EGFR	0.401351	0.002
**79**	5-O3B-P7-(	1000	EGFR	0.477895	0
**80**	2-O3B-A15-	2500	VEGFR	0.378899	0.001
**81**	2-O3B-A17-	10000	VEGFR	0.156471	0.969
**82**	2-O3B-A20-	10000	VEGFR	0.143929	0.973
**83**	2-O3B-B15-	250	VEGFR	0.41125	0
**84**	2-O3B-B17-	1000	VEGFR	0.175237	0.881
**85**	2-O3B-B20-	1000	VEGFR	0.445267	0
**86**	2-O3B-C15-	25	VEGFR	0.349308	0.003
**87**	2-O3B-C17-	100	VEGFR	0.569584	0
**88**	2-O3B-D15	2.5	VEGFR	0.497769	0
**89**	2-O3B-D17	10	VEGFR	0.507671	0
**90**	2-O3B-D20	100	VEGFR	0.232862	0.048
**91**	2-O3B-E17-	1	VEGFR	0.29644	0.051
**92**	2-O3B-E20-	10	VEGFR	0.493008	0
**93**	2-O3B-F13-	10000	VEGFR	0.090499	1
**94**	2-O3B-F15-	0.25	VEGFR	0.310425	0
**95**	2-O3B-F19-	10000	VEGFR	0.124213	1
**96**	2-O3B-F20-	1	VEGFR	0.368276	0
**97**	2-O3B-F21-	10000	VEGFR	0.257393	0.326
**98**	2-O3B-G10	10000	VEGFR	0.239403	0.32
**99**	2-O3B-G13	1000	VEGFR	0.273349	0.246
**100**	2-O3B-G19	1000	VEGFR	0.449313	0
**101**	2-O3B-G21	1000	VEGFR	0.468099	0
**102**	2-O3B-H10	1000	VEGFR	0.510938	0
**103**	2-O3B-H13	100	VEGFR	0.459933	0
**104**	2-O3B-H21	100	VEGFR	0.388879	0.003
**105**	2-O3B-I10-,	100	VEGFR	0.229184	0.558
**106**	2-O3B-I13-,	10	VEGFR	0.55685	0
**107**	2-O3B-I19-I	100	VEGFR	0.314149	0.029
**108**	2-O3B-I21-'	10	VEGFR	0.375248	0.005
**109**	2-O3B-J10-	10	VEGFR	0.344508	0.054
**110**	2-O3B-J13-	1	VEGFR	0.273299	0.008

**111**	2-O3B-J19-	10	VEGFR	0.341084	0.012
**112**	2-O3B-J21-	1	VEGFR	0.281372	0.02
**113**	2-O3B-K10·	1	VEGFR	0.250224	0.007
**114**	2-O3B-K13·	0.1	VEGFR	0.468115	0
**115**	2-O3B-K17·	1	VEGFR	0.304691	0.026
**116**	2-O3B-K19·	1	VEGFR	0.273473	0.151
**117**	2-O3B-L12-	0.1	VEGFR	0.456418	0
**118**	2-O3B-L13-	1	VEGFR	0.448347	0.001
**119**	2-O3B-L21-	0.1	VEGFR	0.301691	0.281
**120**	2-O3B-M12	1	VEGFR	0.476774	0
**121**	2-O3B-M13	10	VEGFR	0.592736	0
**122**	2-O3B-M17	10	VEGFR	0.295372	0.036
**123**	2-O3B-M21	1	VEGFR	0.155391	0.903
**124**	2-O3B-N12	10	VEGFR	0.138056	0.239
**125**	2-O3B-N13	100	VEGFR	0.309847	0.041
**126**	2-O3B-N17	100	VEGFR	0.305432	0.002
**127**	2-O3B-N21	10	VEGFR	0.309945	0.017
**128**	2-O3B-O12	100	VEGFR	0.271313	0.004
**129**	2-O3B-O17	1000	VEGFR	0.410134	0.003
**130**	2-O3B-O21	100	VEGFR	0.179868	0.809
**131**	2-O3B-P12·	1000	VEGFR	0.467849	0
**132**	2-O3B-P13·	1000	VEGFR	0.171852	0.891
**133**	2-O3B-P17·	10000	VEGFR	0.395899	0.003
**134**	2-O3B-P21·	1000	VEGFR	0.36961	0.001
**135**	3-O3B-A3-(	1000	VEGFR	0.154657	0.909
**136**	3-O3B-A6-F	1000	VEGFR	0.096798	0.996
**137**	3-O3B-A18·	1000	VEGFR	0.451171	0
**138**	3-O3B-B3-(	100	VEGFR	0.248486	0.609
**139**	3-O3B-B6-F	100	VEGFR	0.314272	0.001
**140**	3-O3B-B18·	100	VEGFR	0.121507	0.526
**141**	3-O3B-C3-(	10	VEGFR	0.133094	0.923
**142**	3-O3B-C6-F	10	VEGFR	0.186006	0.678
**143**	3-O3B-C18·	10	VEGFR	0.329048	0.009
**144**	3-O3B-D3-(	1	VEGFR	0.164641	0.579
**145**	3-O3B-D6-F	1	VEGFR	0.180829	0.126

**146**	3-O3B-D18	1	VEGFR	0.25576	0.008
**147**	3-O3B-E3-C	0.1	VEGFR	0.229766	0.039
**148**	3-O3B-E6-F	0.1	VEGFR	0.405478	0
**149**	3-O3B-E18-	0.1	VEGFR	0.264329	0.041
**150**	3-O3B-F18-	1000	VEGFR	0.373512	0.007
**151**	3-O3B-G18	100	VEGFR	0.158387	0.468
**152**	3-O3B-H18	10	VEGFR	0.159451	0.868
**153**	3-O3B-I18-	1	VEGFR	0.154484	0.93
**154**	3-O3B-J18-	0.1	VEGFR	0.11975	0.98
**155**	4-O3B-A12-	10000	VEGFR	0.108088	1
**156**	4-O3B-A15-	2500	VEGFR	0.288715	0.03
**157**	4-O3B-A20-	10000	VEGFR	0.157961	0.809
**158**	4-O3B-B12-	1000	VEGFR	0.100713	0.999
**159**	4-O3B-B15-	250	VEGFR	0.265539	0.009
**160**	4-O3B-B20-	1000	VEGFR	0.082566	0.815
**161**	4-O3B-C15-	25	VEGFR	0.108171	0.983
**162**	4-O3B-D12	100	VEGFR	0.328307	0.04
**163**	4-O3B-D15	2.5	VEGFR	0.203372	0.525
**164**	4-O3B-D20	100	VEGFR	0.136829	0.956
**165**	4-O3B-E12-	10	VEGFR	0.161789	0.725
**166**	4-O3B-E20-	10	VEGFR	0.38968	0.001
**167**	4-O3B-F12-	1	VEGFR	0.259501	0.131
**168**	4-O3B-F15-	0.25	VEGFR	0.177935	0.579
**169**	4-O3B-F20-	1	VEGFR	0.199411	0.101
**170**	4-O3B-L16-	1	VEGFR	0.458207	0
**171**	4-O3B-M16	10	VEGFR	0.100296	0.997
**172**	4-O3B-N16	100	VEGFR	0.125544	0.462
**173**	4-O3B-O16	1000	VEGFR	0.460558	0
**174**	4-O3B-P16-	10000	VEGFR	0.375424	0.02
**175**	2-O3B-L10-	1	PI3K	0.053908	0.913
**176**	2-O3B-M10	10	PI3K	0.220089	0.403
**177**	2-O3B-N10	100	PI3K	0.240685	0.412
**178**	2-O3B-O10	1000	PI3K	0.485938	0
**179**	2-O3B-P10-	10000	PI3K	0.389976	0.001
**180**	3-O3B-A16-	2500	PI3K	0.032931	0.989

**181**	3-O3B-C16-	250	PI3K	0.238302	0.076
**182**	3-O3B-D16	25	PI3K	0.045188	0.94
**183**	3-O3B-E16-	2.5	PI3K	0.066114	0.803
**184**	3-O3B-F16-	0.25	PI3K	0.323304	0.003
**185**	3-O3B-F17-	100000	PI3K	0.170321	0.922
**186**	3-O3B-F19-	500	PI3K	0.499939	0
**187**	3-O3B-G17	10000	PI3K	0.049171	0.978
**188**	3-O3B-G19	50	PI3K	0.420601	0
**189**	3-O3B-H17	1000	PI3K	0.409518	0
**190**	3-O3B-I17-	100	PI3K	0.418896	0
**191**	3-O3B-I19-	5	PI3K	0.435334	0
**192**	3-O3B-J17-	10	PI3K	0.40559	0
**193**	3-O3B-J19-	0.5	PI3K	0.440435	0
**194**	3-O3B-K19-	0.05	PI3K	0.068054	0.918
**195**	3-O3B-L8-P	1	PI3K	0.431409	0
**196**	3-O3B-L21-	0.1	PI3K	0.070398	0.866
**197**	3-O3B-M8-	10	PI3K	0.33549	0
**198**	3-O3B-M21	1	PI3K	0.036623	0.994
**199**	3-O3B-N8-F	100	PI3K	0.477173	0
**200**	3-O3B-N21	10	PI3K	0.053373	0.952
**201**	3-O3B-O8-F	1000	PI3K	0.421696	0.011
**202**	3-O3B-O21	100	PI3K	0.251764	0.34
**203**	3-O3B-P8-F	10000	PI3K	0.36402	0.12
**204**	3-O3B-P21-	1000	PI3K	0.378867	0
**205**	4-O3B-A19-	2500	PI3K	0.441895	0
**206**	4-O3B-B19-	250	PI3K	0.220611	0.567
**207**	4-O3B-C19-	25	PI3K	0.381144	0.001
**208**	4-O3B-D19	2.5	PI3K	0.095599	0.74
**209**	4-O3B-E19-	0.25	PI3K	0.072853	0.982
**210**	4-O3B-F14-	1000	PI3K	0.435759	0.027
**211**	4-O3B-G2-T	2500	PI3K	0.45166	0
**212**	4-O3B-G5-S	10000	PI3K	0.434919	0
**213**	4-O3B-G14	100	PI3K	0.456867	0.016
**214**	4-O3B-G20	10000	PI3K	0.426029	0.028
**215**	4-O3B-H2-T	250	PI3K	0.503591	0

**216**	4-O3B-H5-S	1000	PI3K	0.407332	0
**217**	4-O3B-H14	10	PI3K	0.402445	0
**218**	4-O3B-H20	1000	PI3K	0.400002	0
**219**	4-O3B-I2-T	25	PI3K	0.164186	0.904
**220**	4-O3B-I5-S	100	PI3K	0.404164	0
**221**	4-O3B-I14-	1	PI3K	0.49104	0
**222**	4-O3B-I20-	100	PI3K	0.197536	0.684
**223**	4-O3B-J2-T	2.5	PI3K	0.026371	0.998
**224**	4-O3B-J5-S	10	PI3K	0.399859	0
**225**	4-O3B-J20-	10	PI3K	0.106291	0.934
**226**	4-O3B-K2-T	0.25	PI3K	0.076013	0.992
**227**	4-O3B-K4-D	0.1	PI3K	0.402428	0
**228**	4-O3B-K5-S	1	PI3K	0.431382	0
**229**	4-O3B-K14-	0.1	PI3K	0.082931	0.878
**230**	4-O3B-K20-	1	PI3K	0.33027	0.005
**231**	4-O3B-L4-D	1	PI3K	0.418626	0
**232**	4-O3B-L14-	0.1	PI3K	0.358534	0
**233**	4-O3B-L15-	1	PI3K	0.197391	0.178
**234**	4-O3B-L21-	0.1	PI3K	0.38446	0
**235**	4-O3B-M14	1	PI3K	0.436527	0
**236**	4-O3B-M15	10	PI3K	0.424886	0
**237**	4-O3B-M21	1	PI3K	0.413093	0
**238**	4-O3B-N4-D	10	PI3K	0.422524	0
**239**	4-O3B-N14	10	PI3K	0.422487	0
**240**	4-O3B-N15	100	PI3K	0.323748	0
**241**	4-O3B-N21	10	PI3K	0.50069	0
**242**	4-O3B-O4-D	100	PI3K	0.434112	0.002
**243**	4-O3B-O14	100	PI3K	0.460043	0.005
**244**	4-O3B-O15	1000	PI3K	0.407206	0
**245**	4-O3B-O21	100	PI3K	0.415317	0
**246**	4-O3B-P4-D	1000	PI3K	0.42021	0
**247**	4-O3B-P14-	1000	PI3K	0.438339	0.029
**248**	4-O3B-P15-	10000	PI3K	0.462668	0
**249**	4-O3B-P21-	1000	PI3K	0.438364	0.025
**250**	5-O3B-A6-L	2500	PI3K	0.431449	0.035

**251**	5-O3B-A7-A	1000	PI3K	0.441243	0
**252**	5-O3B-A16-	2500	PI3K	0.508504	0
**253**	5-O3B-A17-	10000	PI3K	0.382829	0
**254**	5-O3B-B6-L	250	PI3K	0.410944	0.006
**255**	5-O3B-B7-A	100	PI3K	0.428839	0
**256**	5-O3B-B17-	1000	PI3K	0.419593	0
**257**	5-O3B-C6-L	25	PI3K	0.410306	0
**258**	5-O3B-C7-A	10	PI3K	0.501704	0
**259**	5-O3B-C16-	250	PI3K	0.509984	0
**260**	5-O3B-C17-	100	PI3K	0.352997	0.019
**261**	5-O3B-D6-L	2.5	PI3K	0.34737	0.002
**262**	5-O3B-D7-A	1	PI3K	0.403018	0
**263**	5-O3B-D16	25	PI3K	0.435671	0
**264**	5-O3B-D17	10	PI3K	0.375648	0
**265**	5-O3B-E6-L	0.25	PI3K	0.37697	0
**266**	5-O3B-E7-A	0.1	PI3K	0.420081	0
**267**	5-O3B-E16-	2.5	PI3K	0.442362	0
**268**	5-O3B-E17-	1	PI3K	0.312186	0.005
**269**	5-O3B-F11-	10000	PI3K	0.443019	0
**270**	5-O3B-F16-	0.25	PI3K	0.432601	0
**271**	5-O3B-G9-S	10000	PI3K	0.492274	0
**272**	5-O3B-G11	1000	PI3K	0.451728	0
**273**	5-O3B-H9-S	1000	PI3K	0.388212	0
**274**	5-O3B-H11	100	PI3K	0.446541	0
**275**	5-O3B-I9-Se	100	PI3K	0.10535	0.854
**276**	5-O3B-I11-	10	PI3K	0.465204	0
**277**	5-O3B-J9-S	10	PI3K	0.396159	0
**278**	5-O3B-J11-	1	PI3K	0.456544	0
**279**	5-O3B-K9-S	1	PI3K	0.435182	0
**280**	5-O3B-L14-	0.1	PI3K	0.373856	0.003
**281**	5-O3B-L20-	1	PI3K	0.480656	0
**282**	5-O3B-L23-	0.1	PI3K	0.22992	0.565
**283**	5-O3B-M14	1	PI3K	0.455104	0
**284**	5-O3B-M2C	10	PI3K	0.405724	0
**285**	5-O3B-M23	1	PI3K	0.411016	0

**286**	5-O3B-N14	10	PI3K	0.459363	0
**287**	5-O3B-N20	100	PI3K	0.479696	0
**288**	5-O3B-N23	10	PI3K	0.476467	0
**289**	5-O3B-O14	100	PI3K	0.466023	0
**290**	5-O3B-O20	1000	PI3K	0.450034	0.009
**291**	5-O3B-O23	100	PI3K	0.456777	0.006
**292**	5-O3B-P14-	1000	PI3K	0.436128	0
**293**	5-O3B-P20-	10000	PI3K	0.456658	0.007
**294**	5-O3B-P23-	1000	PI3K	0.308728	0.339
**295**	6-O3B-A8-T	10000	PI3K	0.407683	0
**296**	6-O3B-B8-T	1000	PI3K	0.447557	0
**297**	6-O3B-C8-T	100	PI3K	0.359788	0.001
**298**	6-O3B-D8-T	10	PI3K	0.066913	0.99
**299**	6-O3B-E8-T	1	PI3K	0.08291	0.84
**300**	6-O3B-L6-G	1	PI3K	0.072554	0.913
**301**	6-O3B-M6-	10	PI3K	0.051803	0.97
**302**	6-O3B-N6-	100	PI3K	0.133033	0.895
**303**	6-O3B-O6-	1000	PI3K	0.400646	0
**304**	6-O3B-P6-G	10000	PI3K	0.409068	0.083
**305**	1-O3B-F11-	10000	Topoisome	0.580965	0
**306**	1-O3B-G11	1000	Topoisome	0.593547	0
**307**	1-O3B-G20	1000	Topoisome	0.437754	0.136
**308**	1-O3B-H11	100	Topoisome	0.568882	0
**309**	1-O3B-H20	100	Topoisome	0.583423	0
**310**	1-O3B-I11-	10	Topoisome	0.380346	0.032
**311**	1-O3B-I20-	10	Topoisome	0.39112	0.006
**312**	1-O3B-J11-	1	Topoisome	0.206085	0.173
**313**	1-O3B-J20-	1	Topoisome	0.249176	0.045
**314**	1-O3B-K11-	1	Topoisome	0.535884	0
**315**	1-O3B-K20-	0.1	Topoisome	0.276501	0.133
**316**	1-O3B-L11-	10	Topoisome	0.555376	0
**317**	1-O3B-L14-	1	Topoisome	0.197759	0.23
**318**	1-O3B-M11	100	Topoisome	0.498237	0
**319**	1-O3B-M14	10	Topoisome	0.5422	0
**320**	1-O3B-N14	100	Topoisome	0.58983	0

**321**	1-O3B-O11	1000	Topoisome	0.578717	0.001
**322**	1-O3B-O14	1000	Topoisome	0.582618	0
**323**	1-O3B-P11-	10000	Topoisome	0.546305	0.004
**324**	1-O3B-P14-	10000	Topoisome	0.565331	0
**325**	3-O3B-A11-	10000	Topoisome	0.545485	0
**326**	3-O3B-B11-	1000	Topoisome	0.557544	0
**327**	3-O3B-C11-	100	Topoisome	0.596486	0
**328**	3-O3B-D11	10	Topoisome	0.28307	0.02
**329**	3-O3B-E11-	1	Topoisome	0.270061	0.026
**330**	3-O3B-G9-	1000	Topoisome	0.292276	0.401
**331**	3-O3B-G10	10000	Topoisome	0.139258	0.781
**332**	3-O3B-H9-	100	Topoisome	0.53926	0
**333**	3-O3B-H10	1000	Topoisome	0.160221	0.869
**334**	3-O3B-I9-D	10	Topoisome	0.203327	0.767
**335**	3-O3B-I10-	100	Topoisome	0.164523	0.853
**336**	3-O3B-J9-D	1	Topoisome	0.166279	0.872
**337**	3-O3B-J10-	10	Topoisome	0.16334	0.901
**338**	3-O3B-K7-	0.1	Topoisome	0.297018	0.178
**339**	3-O3B-K9-	0.1	Topoisome	0.347118	0.014
**340**	3-O3B-K10-	1	Topoisome	0.251143	0.031
**341**	3-O3B-L6-	0.1	Topoisome	0.125636	0.586
**342**	3-O3B-L7-	1	Topoisome	0.189195	0.751
**343**	3-O3B-L9-V	0.5	Topoisome	0.281958	0.011
**344**	3-O3B-L10-	0.1	Topoisome	0.232297	0.059
**345**	3-O3B-L16-	1	Topoisome	0.201298	0.153
**346**	3-O3B-M6-	1	Topoisome	0.216182	0.079
**347**	3-O3B-M7-	10	Topoisome	0.398221	0.023
**348**	3-O3B-M9-	5	Topoisome	0.301346	0.017
**349**	3-O3B-M10	1	Topoisome	0.267238	0.183
**350**	3-O3B-M16	10	Topoisome	0.267963	0.028
**351**	3-O3B-N6-	10	Topoisome	0.348329	0.004
**352**	3-O3B-N9-	50	Topoisome	0.558718	0
**353**	3-O3B-N10	10	Topoisome	0.605038	0
**354**	3-O3B-N16	100	Topoisome	0.246354	0.037
**355**	3-O3B-O6-	100	Topoisome	0.584256	0

**356**	3-O3B-O7-I	100	Topoisome	0.55292	0
**357**	3-O3B-O9-\	500	Topoisome	0.585362	0
**358**	3-O3B-O10	100	Topoisome	0.59252	0
**359**	3-O3B-O16	1000	Topoisome	0.362731	0.124
**360**	3-O3B-P6-D	1000	Topoisome	0.44549	0.101
**361**	3-O3B-P7-I	1000	Topoisome	0.375875	0.335
**362**	3-O3B-P9-V	5000	Topoisome	0.36961	0.306
**363**	3-O3B-P10-	1000	Topoisome	0.483829	0.009
**364**	3-O3B-P16-	10000	Topoisome	0.255959	0.434
**365**	1-O3B-A10-	10000	Mitotic	0.723827	0
**366**	1-O3B-A13-	1000	Mitotic	0.729534	0
**367**	1-O3B-A18-	1000	Mitotic	0.727287	0
**368**	1-O3B-B10-	1000	Mitotic	0.730189	0
**369**	1-O3B-B13-	100	Mitotic	0.306483	0.027
**370**	1-O3B-B18-	100	Mitotic	0.734241	0
**371**	1-O3B-C10-	100	Mitotic	0.733174	0
**372**	1-O3B-C13-	10	Mitotic	0.216969	0.794
**373**	1-O3B-C18-	10	Mitotic	0.734127	0
**374**	1-O3B-D10	10	Mitotic	0.73296	0
**375**	1-O3B-D13	1	Mitotic	0.312776	0.02
**376**	1-O3B-D18	1	Mitotic	0.362624	0.099
**377**	1-O3B-E10-	1	Mitotic	0.453153	0.005
**378**	1-O3B-E13-	0.1	Mitotic	0.543811	0
**379**	1-O3B-E18-	0.1	Mitotic	0.219113	0.803
**380**	1-O3B-F13-	1000	Mitotic	0.732733	0
**381**	1-O3B-G13	100	Mitotic	0.373918	0.046
**382**	1-O3B-G15	1000	Mitotic	0.720237	0
**383**	1-O3B-H13	10	Mitotic	0.230066	0.373
**384**	1-O3B-H15	100	Mitotic	0.738175	0
**385**	1-O3B-I13-'	1	Mitotic	0.234183	0.562
**386**	1-O3B-I15-I	10	Mitotic	0.729252	0
**387**	1-O3B-J13-	0.1	Mitotic	0.188166	0.23
**388**	1-O3B-J15-	1	Mitotic	0.723125	0
**389**	1-O3B-K7-V	0.1	Mitotic	0.080159	0.99
**390**	1-O3B-K15-	0.1	Mitotic	0.325502	0.338

**391**	1-O3B-L7-V	1	Mitotic	0.148708	0.958
**392**	1-O3B-L20-	0.1	Mitotic	0.446746	0
**393**	1-O3B-M7-'	10	Mitotic	0.238653	0.21
**394**	1-O3B-M2C	1	Mitotic	0.380864	0.111
**395**	1-O3B-N20	10	Mitotic	0.329703	0.009
**396**	1-O3B-O7-\	100	Mitotic	0.386749	0.027
**397**	1-O3B-O20	100	Mitotic	0.249017	0.551
**398**	1-O3B-P7-\	1000	Mitotic	0.748858	0
**399**	1-O3B-P20-	1000	Mitotic	0.732418	0
**400**	3-O3B-A7-D	1000	Mitotic	0.735253	0
**401**	3-O3B-B7-D	100	Mitotic	0.710323	0
**402**	3-O3B-C7-D	10	Mitotic	0.733021	0
**403**	3-O3B-D7-D	1	Mitotic	0.17461	0.896
**404**	3-O3B-E7-D	0.1	Mitotic	0.162455	0.913
**405**	6-O3B-L19-	1	Mitotic	0.154363	0.887
**406**	6-O3B-M19	10	Mitotic	0.242675	0.541
**407**	6-O3B-N19	100	Mitotic	0.165101	0.628
**408**	6-O3B-O19	1000	Mitotic	0.725387	0
**409**	6-O3B-P19-	10000	Mitotic	0.722317	0
**410**	2-O3B-A12-	250	MEK1/2	0.610702	0
**411**	2-O3B-B12-	25	MEK1/2	0.712035	0
**412**	2-O3B-D12	2.5	MEK1/2	0.761405	0
**413**	2-O3B-E12-	0.25	MEK1/2	0.796596	0
**414**	2-O3B-F12-	2.5000000	MEK1/2	0.292442	0.35
**415**	2-O3B-F14-	1000	MEK1/2	0.603943	0
**416**	2-O3B-G14	100	MEK1/2	0.722834	0
**417**	2-O3B-H14	10	MEK1/2	0.68635	0
**418**	2-O3B-I14-	1	MEK1/2	0.162531	0.803
**419**	2-O3B-K14-	0.1	MEK1/2	0.493079	0.017
**420**	2-O3B-L20-	1	MEK1/2	0.121568	0.996
**421**	2-O3B-M2C	10	MEK1/2	0.398106	0.144
**422**	2-O3B-N20	100	MEK1/2	0.784952	0
**423**	2-O3B-O20	1000	MEK1/2	0.744783	0
**424**	2-O3B-P20-	10000	MEK1/2	0.669897	0
**425**	4-O3B-A10-	1000	MEK1/2	0.66338	0

**426**	4-O3B-A13-	1000	MEK1/2	0.695533	0
**427**	4-O3B-B10-	100	MEK1/2	0.66098	0
**428**	4-O3B-B13-	100	MEK1/2	0.768962	0
**429**	4-O3B-C10-	10	MEK1/2	0.659173	0
**430**	4-O3B-C13-	10	MEK1/2	0.572238	0
**431**	4-O3B-D10	1	MEK1/2	0.580434	0
**432**	4-O3B-D13	1	MEK1/2	0.718901	0
**433**	4-O3B-E10-	0.1	MEK1/2	0.611727	0
**434**	4-O3B-E13-	0.1	MEK1/2	0.133834	0.996
**435**	4-O3B-L19-	0.25	MEK1/2	0.602708	0
**436**	4-O3B-M19	2.5	MEK1/2	0.456327	0.016
**437**	4-O3B-N19	25	MEK1/2	0.744186	0
**438**	4-O3B-O19	250	MEK1/2	0.656833	0
**439**	4-O3B-P19-	2500	MEK1/2	0.684742	0
**440**	1-O3B-L2-C	1	PARP	0.434736	0.014
**441**	1-O3B-L6-R	1	PARP	0.618424	0.001
**442**	1-O3B-M2-	10	PARP	0.679611	0
**443**	1-O3B-M6-	10	PARP	0.667018	0
**444**	1-O3B-N2-(	100	PARP	0.778252	0
**445**	1-O3B-N6-F	100	PARP	0.728886	0
**446**	1-O3B-O2-(	1000	PARP	0.77539	0
**447**	1-O3B-O6-F	1000	PARP	0.738026	0
**448**	1-O3B-P2-C	10000	PARP	0.745062	0
**449**	1-O3B-P6-R	10000	PARP	0.734611	0
**450**	7-O3B-A3-T	1000	PARP	0.747126	0
**451**	7-O3B-B2-V	10000	PARP	0.813897	0
**452**	7-O3B-B3-T	100	PARP	0.748631	0
**453**	7-O3B-C2-V	1000	PARP	0.685295	0
**454**	7-O3B-C3-T	10	PARP	0.738731	0
**455**	7-O3B-D2-V	100	PARP	0.410671	0.018
**456**	7-O3B-D3-T	1	PARP	0.825245	0
**457**	7-O3B-E2-V	10	PARP	0.386972	0.279
**458**	7-O3B-E3-T	0.1	PARP	0.323214	0.422
**459**	7-O3B-F2-V	1	PARP	0.504085	0.006
**460**	7-O3B-G2-N	10000	PARP	0.766864	0

**461**	7-O3B-H2-N	1000	PARP	0.754386	0
**462**	7-O3B-I2-N	100	PARP	0.823933	0
**463**	7-O3B-J2-N	10	PARP	0.457117	0.054
**464**	7-O3B-K2-N	1	PARP	0.484049	0.025
**465**	3-O3B-A19-	1000	CDK	0.400578	0.016
**466**	3-O3B-B19-	100	CDK	0.38555	0.023
**467**	3-O3B-B23-	2500	CDK	0.247339	0.593
**468**	3-O3B-C19-	10	CDK	0.255025	0.387
**469**	3-O3B-C23-	250	CDK	0.215209	0.717
**470**	3-O3B-D19	1	CDK	0.162762	0.286
**471**	3-O3B-D23	25	CDK	0.171773	0.318
**472**	3-O3B-E19-	0.1	CDK	0.202726	0.32
**473**	3-O3B-E23-	2.5	CDK	0.193525	0.373
**474**	3-O3B-F23-	0.25	CDK	0.187479	0.288
**475**	3-O3B-K17-	1	CDK	0.274876	0.286
**476**	3-O3B-L19-	1	CDK	0.200703	0.154
**477**	3-O3B-M17	10	CDK	0.189043	0.45
**478**	3-O3B-M19	10	CDK	0.16846	0.648
**479**	3-O3B-N17	100	CDK	0.222081	0.189
**480**	3-O3B-N19	100	CDK	0.182964	0.242
**481**	3-O3B-O17	1000	CDK	0.18063	0.583
**482**	3-O3B-O19	1000	CDK	0.232805	0.141
**483**	3-O3B-P17-	10000	CDK	0.168713	0.897
**484**	3-O3B-P19-	10000	CDK	0.217386	0.546
**485**	4-O3B-A4-S	10000	CDK	0.494125	0.001
**486**	4-O3B-A8-N	10000	CDK	0.562005	0.001
**487**	4-O3B-B4-S	1000	CDK	0.45829	0.007
**488**	4-O3B-B8-N	1000	CDK	0.449926	0.04
**489**	4-O3B-C4-S	100	CDK	0.29625	0.363
**490**	4-O3B-C8-N	100	CDK	0.244567	0.472
**491**	4-O3B-D4-S	10	CDK	0.158167	0.866
**492**	4-O3B-D8-N	10	CDK	0.143333	0.961
**493**	4-O3B-E4-S	1	CDK	0.188392	0.875
**494**	4-O3B-E8-N	1	CDK	0.24541	0.432
**495**	4-O3B-F4-S	10000	CDK	0.375173	0.14

**496**	4-O3B-F22-	10000	CDK	0.476042	0.001
**497**	4-O3B-G4-S	1000	CDK	0.287125	0.427
**498**	4-O3B-G22	1000	CDK	0.449917	0.005
**499**	4-O3B-H4-S	100	CDK	0.263341	0.351
**500**	4-O3B-H22	100	CDK	0.200364	0.292
**501**	4-O3B-I4-Se	10	CDK	0.236118	0.532
**502**	4-O3B-I22-,	10	CDK	0.187684	0.591
**503**	4-O3B-J4-S	1	CDK	0.232583	0.557
**504**	4-O3B-J22-	1	CDK	0.163323	0.405
**505**	5-O3B-A19-	10000	CDK	0.486348	0.005
**506**	5-O3B-B19-	1000	CDK	0.325504	0.412
**507**	5-O3B-C19-	100	CDK	0.285499	0.326
**508**	5-O3B-D19	10	CDK	0.191673	0.54
**509**	5-O3B-E19-	1	CDK	0.286799	0.326
**510**	5-O3B-K17-	1	CDK	0.251898	0.491
**511**	5-O3B-M17	10	CDK	0.282225	0.343
**512**	5-O3B-N17	100	CDK	0.263591	0.476
**513**	5-O3B-O17	1000	CDK	0.465214	0.008
**514**	5-O3B-P17-	10000	CDK	0.465812	0.011
**515**	6-O3B-A17-	1000	CDK	0.228854	0.611
**516**	6-O3B-B17-	100	CDK	0.27873	0.306
**517**	6-O3B-C17-	10	CDK	0.306396	0.188
**518**	6-O3B-D17	1	CDK	0.121348	0.609
**519**	6-O3B-E17-	0.1	CDK	0.241858	0.425
**520**	6-O3B-L15-	1	CDK	0.166537	0.774
**521**	6-O3B-M15	10	CDK	0.175366	0.716
**522**	6-O3B-N15	100	CDK	0.571742	0.001
**523**	6-O3B-O15	1000	CDK	0.446579	0.003
**524**	6-O3B-P15-	10000	CDK	0.45726	0.002
**525**	7-O3B-A21-	10000	BET	0.401031	0.03
**526**	7-O3B-A22-	30000	BET	0.650683	0
**527**	7-O3B-B21-	1000	BET	0.301555	0.143
**528**	7-O3B-B22-	3000	BET	0.47343	0.001
**529**	7-O3B-C21-	100	BET	0.257889	0.539
**530**	7-O3B-C22-	300	BET	0.547224	0

**531**	7-O3B-D21	10	BET	0.235508	0.774
**532**	7-O3B-D22	30	BET	0.495212	0
**533**	7-O3B-E21-	1	BET	0.248784	0.564
**534**	7-O3B-E22-	3	BET	0.307661	0.616
**535**	7-O3B-G10	10000	BET	0.626981	0
**536**	7-O3B-G15	10000	BET	0.647391	0
**537**	7-O3B-H10	1000	BET	0.633976	0
**538**	7-O3B-H15	1000	BET	0.652517	0
**539**	7-O3B-I10-I	100	BET	0.621946	0
**540**	7-O3B-I15-I	100	BET	0.329837	0.228
**541**	7-O3B-J10-	10	BET	0.33352	0.012
**542**	7-O3B-J15-	10	BET	0.30227	0.222
**543**	7-O3B-K10-	1	BET	0.19801	0.342
**544**	7-O3B-K13-	1	BET	0.312694	0.01
**545**	7-O3B-K15-	1	BET	0.348271	0.062
**546**	7-O3B-L12-	1	BET	0.172958	0.893
**547**	7-O3B-L13-	10	BET	0.600728	0
**548**	7-O3B-L20-	1	BET	0.195156	0.567
**549**	7-O3B-L23-	0.03	BET	0.173114	0.67
**550**	7-O3B-M12	10	BET	0.243646	0.183
**551**	7-O3B-M13	100	BET	0.638745	0
**552**	7-O3B-M2C	10	BET	0.246505	0.154
**553**	7-O3B-M23	0.3	BET	0.282108	0.472
**554**	7-O3B-N12	100	BET	0.242034	0.123
**555**	7-O3B-N13	1000	BET	0.644504	0
**556**	7-O3B-N20	100	BET	0.482465	0
**557**	7-O3B-N23	3	BET	0.280327	0.051
**558**	7-O3B-O12	1000	BET	0.60236	0
**559**	7-O3B-O20	1000	BET	0.634649	0.001
**560**	7-O3B-O23	30	BET	0.432295	0.008
**561**	7-O3B-P12-	10000	BET	0.637163	0
**562**	7-O3B-P13-	10000	BET	0.63146	0
**563**	7-O3B-P20-	10000	BET	0.642822	0.001
**564**	7-O3B-P23-	300	BET	0.642659	0
**565**	8-O3B-K22-	1	BET	0.164133	0.976

566	8-O3B-L22-	10	BET	0.139927	0.661
567	8-O3B-M22	100	BET	0.312531	0.229
568	8-O3B-N22	1000	BET	0.332625	0.09
569	8-O3B-O22	10000	BET	0.232633	0.286
570	1-O3B-A3-\	10000	HDAC	0.525906	0
571	1-O3B-B3-\	1000	HDAC	0.402302	0
572	1-O3B-C3-\	100	HDAC	0.170046	0.887
573	1-O3B-D3-\	10	HDAC	0.33317	0.182
574	1-O3B-E3-V	1	HDAC	0.123073	0.975
575	1-O3B-L12-	0.1	HDAC	0.19531	0.117
576	1-O3B-M12	1	HDAC	0.243122	0.042
577	1-O3B-N12	10	HDAC	0.521297	0
578	1-O3B-O12	100	HDAC	0.31399	0.016
579	1-O3B-P12-	1000	HDAC	0.316648	0.022
580	3-O3B-A4-F	1000	HDAC	0.303536	0.042
581	3-O3B-B4-F	100	HDAC	0.323793	0.229
582	3-O3B-C4-F	10	HDAC	0.385216	0.002
583	3-O3B-D4-F	1	HDAC	0.159134	0.782
584	3-O3B-E4-P	0.1	HDAC	0.144887	0.875
585	3-O3B-F7-C	1000	HDAC	0.306175	0.074
586	3-O3B-G7-(	100	HDAC	0.214583	0.643
587	3-O3B-G12	1000000	HDAC	0.135941	0.263
588	3-O3B-H7-(	10	HDAC	0.132157	0.963
589	3-O3B-H12	100000	HDAC	0.154418	0.879
590	3-O3B-I7-Q	1	HDAC	0.126358	0.965
591	3-O3B-I12-'	10000	HDAC	0.123942	0.96
592	3-O3B-J7-Q	0.1	HDAC	0.116467	0.988
593	3-O3B-J12-	1000	HDAC	0.128245	0.957
594	3-O3B-K3-E	1	HDAC	0.219481	0.378
595	3-O3B-K12-	100	HDAC	0.292293	0.003
596	3-O3B-L3-B	10	HDAC	0.183434	0.282
597	3-O3B-M3-I	100	HDAC	0.277525	0.197
598	3-O3B-N3-E	1000	HDAC	0.36523	0.114
599	3-O3B-O3-E	10000	HDAC	0.345898	0.024
600	7-O3B-A5-N	10000	HDAC	0.457854	0.009

**601**	7-O3B-A7-C	10000	HDAC	0.341478	0.008
**602**	7-O3B-A9-G	1000	HDAC	0.429534	0.023
**603**	7-O3B-A12-	10000	HDAC	0.407578	0.037
**604**	7-O3B-B5-M	1000	HDAC	0.358679	0.017
**605**	7-O3B-B7-C	1000	HDAC	0.378439	0.029
**606**	7-O3B-B12-	1000	HDAC	0.288064	0.127
**607**	7-O3B-C5-M	100	HDAC	0.372899	0.005
**608**	7-O3B-C7-C	100	HDAC	0.497133	0.001
**609**	7-O3B-C9-G	100	HDAC	0.352086	0.004
**610**	7-O3B-D7-C	10	HDAC	0.312877	0.157
**611**	7-O3B-D9-C	10	HDAC	0.216321	0.552
**612**	7-O3B-D12	100	HDAC	0.276162	0.001
**613**	7-O3B-E5-M	10	HDAC	0.294523	0.025
**614**	7-O3B-E7-C	1	HDAC	0.180254	0.822
**615**	7-O3B-E9-G	1	HDAC	0.183426	0.762
**616**	7-O3B-E12-	10	HDAC	0.289229	0.058
**617**	7-O3B-F5-M	1	HDAC	0.294906	0.011
**618**	7-O3B-F7-R	10000	HDAC	0.482585	0.001
**619**	7-O3B-F9-G	0.1	HDAC	0.317243	0
**620**	7-O3B-F12-	1	HDAC	0.31179	0
**621**	7-O3B-F19-	10000	HDAC	0.27601	0.167
**622**	7-O3B-G7-F	1000	HDAC	0.278963	0.129
**623**	7-O3B-G19	1000	HDAC	0.234063	0.494
**624**	7-O3B-H7-F	100	HDAC	0.371935	0.015
**625**	7-O3B-I7-R	10	HDAC	0.287269	0.054
**626**	7-O3B-I19-	100	HDAC	0.239625	0.468
**627**	7-O3B-J7-R	1	HDAC	0.467555	0
**628**	7-O3B-J19-	10	HDAC	0.348191	0
**629**	7-O3B-K4-E	1	HDAC	0.304259	0.009
**630**	7-O3B-K11-	1	HDAC	0.32431	0.006
**631**	7-O3B-K18-	1	HDAC	0.276775	0.005
**632**	7-O3B-K19-	1	HDAC	0.201435	0.791
**633**	7-O3B-L2-T	0.1	HDAC	0.06205	0.998
**634**	7-O3B-L4-E	10	HDAC	0.241442	0.014
**635**	7-O3B-L5-P	1	HDAC	0.204469	0.647

**636**	7-O3B-L8-A	1	HDAC	0.167483	0.835
**637**	7-O3B-L10-	1	HDAC	0.327206	0.001
**638**	7-O3B-L11-	10	HDAC	0.270297	0.078
**639**	7-O3B-L14-	1	HDAC	0.340717	0
**640**	7-O3B-L16-	1	HDAC	0.359791	0.019
**641**	7-O3B-L18-	10	HDAC	0.30192	0.089
**642**	7-O3B-M2-T	1	HDAC	0.145295	0.252
**643**	7-O3B-M5-I	10	HDAC	0.285942	0.003
**644**	7-O3B-M8-,	10	HDAC	0.23723	0.009
**645**	7-O3B-M10	10	HDAC	0.342248	0
**646**	7-O3B-M11	100	HDAC	0.33189	0.027
**647**	7-O3B-M14	10	HDAC	0.217893	0.263
**648**	7-O3B-M16	10	HDAC	0.429758	0
**649**	7-O3B-M18	100	HDAC	0.27017	0.121
**650**	7-O3B-N2-T	10	HDAC	0.236973	0.039
**651**	7-O3B-N4-E	100	HDAC	0.351827	0
**652**	7-O3B-N5-F	100	HDAC	0.582475	0
**653**	7-O3B-N8-A	100	HDAC	0.361218	0
**654**	7-O3B-N10	100	HDAC	0.405648	0
**655**	7-O3B-N14	100	HDAC	0.120887	0.815
**656**	7-O3B-N16	100	HDAC	0.351519	0
**657**	7-O3B-N18	1000	HDAC	0.404022	0
**658**	7-O3B-O2-T	100	HDAC	0.210764	0.115
**659**	7-O3B-O4-E	1000	HDAC	0.403375	0
**660**	7-O3B-O5-F	1000	HDAC	0.300542	0.152
**661**	7-O3B-O8-A	1000	HDAC	0.324865	0.066
**662**	7-O3B-O10	1000	HDAC	0.275567	0.017
**663**	7-O3B-O11	1000	HDAC	0.313955	0.076
**664**	7-O3B-O14	1000	HDAC	0.223499	0.005
**665**	7-O3B-O16	1000	HDAC	0.307918	0.056
**666**	7-O3B-P2-T	1000	HDAC	0.318568	0.001
**667**	7-O3B-P4-E	10000	HDAC	0.354687	0.03
**668**	7-O3B-P5-F	10000	HDAC	0.371109	0.035
**669**	7-O3B-P8-A	10000	HDAC	0.356528	0.053
**670**	7-O3B-P10-	10000	HDAC	0.298507	0.203

**671**	7-O3B-P11-	10000	HDAC	0.367198	0.056
**672**	7-O3B-P14-	10000	HDAC	0.254578	0.004
**673**	7-O3B-P16-	10000	HDAC	0.398013	0
**674**	7-O3B-P18-	10000	HDAC	0.466161	0.012
**0**	2-O8W-A16	10000	EGFR	0.310455	0.018
**1**	2-O8W-A19	10000	EGFR	0.326757	0.026
**2**	2-O8W-B19	1000	EGFR	0.158829	0.678
**3**	2-O8W-C16	1000	EGFR	0.144611	0.324
**4**	2-O8W-C19	100	EGFR	0.09203	0.844
**5**	2-O8W-D16	100	EGFR	0.141376	0.771
**6**	2-O8W-D19	10	EGFR	0.100422	0.998
**7**	2-O8W-E16	10	EGFR	0.321374	0.046
**8**	2-O8W-E19	1	EGFR	0.204262	0.656
**9**	2-O8W-F16	1	EGFR	0.271983	0.456
**10**	2-O8W-K11	0.1	EGFR	0.318283	0.144
**11**	2-O8W-L11	1	EGFR	0.306372	0.02
**12**	2-O8W-L16	0.25	EGFR	0.355535	0.017
**13**	2-O8W-L19	0.1	EGFR	0.142566	0.881
**14**	2-O8W-M1	10	EGFR	0.144382	0.798
**15**	2-O8W-M1	2.5	EGFR	0.324647	0.02
**16**	2-O8W-M1	1	EGFR	0.286612	0.177
**17**	2-O8W-N16	25	EGFR	0.346508	0.119
**18**	2-O8W-N19	10	EGFR	0.358853	0
**19**	2-O8W-O1	100	EGFR	0.354037	0.004
**20**	2-O8W-O16	250	EGFR	0.319455	0.052
**21**	2-O8W-O19	100	EGFR	0.294158	0.149
**22**	2-O8W-P11	1000	EGFR	0.282614	0.259
**23**	2-O8W-P16	2500	EGFR	0.296953	0.216
**24**	2-O8W-P19	1000	EGFR	0.296501	0.119
**25**	3-O8W-F21	10000	EGFR	0.059944	1
**26**	3-O8W-G20	1000	EGFR	0.220794	0.597
**27**	3-O8W-G2	1000	EGFR	0.254462	0.327
**28**	3-O8W-H20	100	EGFR	0.121243	0.827
**29**	3-O8W-H2	100	EGFR	0.213896	0.723
**30**	3-O8W-I20	10	EGFR	0.190031	0.597

**31**	3-O8W-I21	10	EGFR	0.147164	0.614
**32**	3-O8W-J20	1	EGFR	0.311173	0.02
**33**	3-O8W-J21	1	EGFR	0.233297	0.846
**34**	3-O8W-K4-	1	EGFR	0.278573	0.282
**35**	3-O8W-K18	0.1	EGFR	0.360735	0
**36**	3-O8W-K20	0.1	EGFR	0.162186	0.721
**37**	3-O8W-L4-	10	EGFR	0.107601	0.873
**38**	3-O8W-L18	1	EGFR	0.148689	0.669
**39**	3-O8W-M1	10	EGFR	0.318497	0.003
**40**	3-O8W-N4-	100	EGFR	0.339708	0.002
**41**	3-O8W-N18	100	EGFR	0.342757	0.003
**42**	3-O8W-O4-	1000	EGFR	0.090543	0.929
**43**	3-O8W-P4-	10000	EGFR	0.217291	0.969
**44**	3-O8W-P18	1000	EGFR	0.34547	0.001
**45**	4-O8W-F13	1000	EGFR	0.28425	0.064
**46**	4-O8W-G13	100	EGFR	0.314923	0.039
**47**	4-O8W-G16	10000	EGFR	0.174004	0.924
**48**	4-O8W-H13	10	EGFR	0.152153	0.796
**49**	4-O8W-H16	1000	EGFR	0.105061	0.906
**50**	4-O8W-I13	1	EGFR	0.1958	0.501
**51**	4-O8W-I16	100	EGFR	0.219992	0.224
**52**	4-O8W-J13	0.1	EGFR	0.298703	0.014
**53**	4-O8W-J16	10	EGFR	0.313028	0.019
**54**	4-O8W-K7-	1	EGFR	0.329564	0.023
**55**	4-O8W-K13	0.1	EGFR	0.192092	0.169
**56**	4-O8W-K16	1	EGFR	0.273632	0.416
**57**	4-O8W-L7-	10	EGFR	0.327483	0.035
**58**	4-O8W-L13	1	EGFR	0.368611	0.019
**59**	4-O8W-M7	100	EGFR	0.310975	0.112
**60**	4-O8W-M1	10	EGFR	0.301438	0.058
**61**	4-O8W-N13	100	EGFR	0.189856	0.115
**62**	4-O8W-O7-	1000	EGFR	0.185343	0.615
**63**	4-O8W-P7-	10000	EGFR	0.283889	0.175
**64**	4-O8W-P13	1000	EGFR	0.268725	0.282
**65**	5-O8W-F4-	1000	EGFR	0.309844	0.128

**66**	5-O8W-F7-/	1000	EGFR	0.325339	0.011
**67**	5-O8W-G4-	100	EGFR	0.163775	0.69
**68**	5-O8W-G7-	100	EGFR	0.210759	0.719
**69**	5-O8W-H4-	10	EGFR	0.13169	0.836
**70**	5-O8W-H7-	10	EGFR	0.322168	0.008
**71**	5-O8W-I4-F	1	EGFR	0.186054	0.672
**72**	5-O8W-I7-/	1	EGFR	0.220805	0.638
**73**	5-O8W-J4-I	0.1	EGFR	0.166889	0.727
**74**	5-O8W-J7-/	0.1	EGFR	0.317215	0.101
**75**	5-O8W-K7-	0.1	EGFR	0.213179	0.878
**76**	5-O8W-L7-	1	EGFR	0.296035	0.083
**77**	5-O8W-M7	10	EGFR	0.152815	0.987
**78**	5-O8W-O7-	100	EGFR	0.181216	0.617
**79**	5-O8W-P7-	1000	EGFR	0.27636	0.268
**80**	2-O8W-A1!	2500	VEGFR	0.345936	0.006
**81**	2-O8W-A1	10000	VEGFR	0.356641	0.027
**82**	2-O8W-A2(	10000	VEGFR	0.156757	0.999
**83**	2-O8W-B1!	250	VEGFR	0.441377	0
**84**	2-O8W-B1	1000	VEGFR	0.417953	0
**85**	2-O8W-B2(	1000	VEGFR	0.193285	0.541
**86**	2-O8W-C1!	25	VEGFR	0.323348	0.014
**87**	2-O8W-C1	100	VEGFR	0.385278	0.001
**88**	2-O8W-D1!	2.5	VEGFR	0.403032	0
**89**	2-O8W-D1	10	VEGFR	0.183494	0.203
**90**	2-O8W-D2(	100	VEGFR	0.464541	0
**91**	2-O8W-E17	1	VEGFR	0.450134	0
**92**	2-O8W-E2C	10	VEGFR	0.242725	0.69
**93**	2-O8W-F13	10000	VEGFR	0.286085	0.269
**94**	2-O8W-F15	0.25	VEGFR	0.457177	0
**95**	2-O8W-F1!	10000	VEGFR	0.107202	1
**96**	2-O8W-F2C	1	VEGFR	0.435179	0
**97**	2-O8W-F21	10000	VEGFR	0.174043	0.878
**98**	2-O8W-G1(	10000	VEGFR	0.373553	0.003
**99**	2-O8W-G1:	1000	VEGFR	0.396993	0.003
**100**	2-O8W-G1!	1000	VEGFR	0.184025	0.187

**101**	2-O8W-G2	1000	VEGFR	0.413153	0
**102**	2-O8W-H1	1000	VEGFR	0.219966	0.502
**103**	2-O8W-H1	100	VEGFR	0.395067	0.001
**104**	2-O8W-H2	100	VEGFR	0.096516	0.946
**105**	2-O8W-I10	100	VEGFR	0.517991	0
**106**	2-O8W-I13	10	VEGFR	0.510616	0
**107**	2-O8W-I19	100	VEGFR	0.277134	0.036
**108**	2-O8W-I21	10	VEGFR	0.12943	0.987
**109**	2-O8W-J10	10	VEGFR	0.446304	0
**110**	2-O8W-J13	1	VEGFR	0.456166	0
**111**	2-O8W-J19	10	VEGFR	0.418526	0
**112**	2-O8W-J21	1	VEGFR	0.369691	0
**113**	2-O8W-K1	1	VEGFR	0.240223	0.159
**114**	2-O8W-K1	0.1	VEGFR	0.219547	0.48
**115**	2-O8W-K1	1	VEGFR	0.408939	0.001
**116**	2-O8W-K1	1	VEGFR	0.41886	0
**117**	2-O8W-L12	0.1	VEGFR	0.343521	0.001
**118**	2-O8W-L13	1	VEGFR	0.500072	0
**119**	2-O8W-L21	0.1	VEGFR	0.330508	0.11
**120**	2-O8W-M1	1	VEGFR	0.342618	0.009
**121**	2-O8W-M1	10	VEGFR	0.387551	0
**122**	2-O8W-M1	10	VEGFR	0.401906	0
**123**	2-O8W-M2	1	VEGFR	0.270965	0.053
**124**	2-O8W-N1	10	VEGFR	0.428753	0
**125**	2-O8W-N1	100	VEGFR	0.206877	0.257
**126**	2-O8W-N1	100	VEGFR	0.362612	0
**127**	2-O8W-N2	10	VEGFR	0.182671	0.628
**128**	2-O8W-O1	100	VEGFR	0.392154	0.002
**129**	2-O8W-O1	1000	VEGFR	0.260628	0.439
**130**	2-O8W-O2	100	VEGFR	0.284888	0.122
**131**	2-O8W-P1	1000	VEGFR	0.203436	0.452
**132**	2-O8W-P1	1000	VEGFR	0.365408	0.016
**133**	2-O8W-P1	10000	VEGFR	0.173532	0.924
**134**	2-O8W-P2	1000	VEGFR	0.300461	0.03
**135**	3-O8W-A3-	1000	VEGFR	0.18638	0.691

**136**	3-O8W-A6-	1000	VEGFR	0.239428	0.618
**137**	3-O8W-A1ε	1000	VEGFR	0.118281	0.998
**138**	3-O8W-B3-	100	VEGFR	0.127714	0.824
**139**	3-O8W-B6-	100	VEGFR	0.340436	0.05
**140**	3-O8W-B1ε	100	VEGFR	0.116001	0.874
**141**	3-O8W-C3-	10	VEGFR	0.11019	0.903
**142**	3-O8W-C6-	10	VEGFR	0.169897	0.603
**143**	3-O8W-C1ε	10	VEGFR	0.189357	0.578
**144**	3-O8W-D3-	1	VEGFR	0.105186	0.966
**145**	3-O8W-D6-	1	VEGFR	0.110567	0.868
**146**	3-O8W-D1ε	1	VEGFR	0.102832	0.918
**147**	3-O8W-E3-(	0.1	VEGFR	0.261516	0.353
**148**	3-O8W-E6-I	0.1	VEGFR	0.182165	0.924
**149**	3-O8W-E18	0.1	VEGFR	0.345463	0.004
**150**	3-O8W-F18	1000	VEGFR	0.34661	0.031
**151**	3-O8W-G1ε	100	VEGFR	0.285746	0.029
**152**	3-O8W-H1ε	10	VEGFR	0.156849	0.467
**153**	3-O8W-I18	1	VEGFR	0.130491	0.815
**154**	3-O8W-J18	0.1	VEGFR	0.213608	0.194
**155**	4-O8W-A1⁊	10000	VEGFR	0.053861	1
**156**	4-O8W-A1ξ	2500	VEGFR	0.076241	0.948
**157**	4-O8W-A2(	10000	VEGFR	0.329128	0.026
**158**	4-O8W-B1⁊	1000	VEGFR	0.275339	0.184
**159**	4-O8W-B1ξ	250	VEGFR	0.080165	0.986
**160**	4-O8W-B2(	1000	VEGFR	0.426416	0
**161**	4-O8W-C1ξ	25	VEGFR	0.398401	0
**162**	4-O8W-D1⁊	100	VEGFR	0.378633	0.002
**163**	4-O8W-D1ξ	2.5	VEGFR	0.289229	0.006
**164**	4-O8W-D2(	100	VEGFR	0.062636	0.982
**165**	4-O8W-E12	10	VEGFR	0.32441	0.004
**166**	4-O8W-E2C	10	VEGFR	0.291782	0.091
**167**	4-O8W-F12	1	VEGFR	0.325072	0.009
**168**	4-O8W-F1ξ	0.25	VEGFR	0.091957	0.871
**169**	4-O8W-F2C	1	VEGFR	0.291153	0.067
**170**	4-O8W-L1ε	1	VEGFR	0.189895	0.498

**171**	4-O8W-M1	10	VEGFR	0.104467	0.951
**172**	4-O8W-N1(	100	VEGFR	0.287905	0.218
**173**	4-O8W-O1(	1000	VEGFR	0.350481	0.011
**174**	4-O8W-P16	10000	VEGFR	0.249309	0.362
**175**	2-O8W-L1C	1	PI3K	0.343065	0.008
**176**	2-O8W-M1	10	PI3K	0.290426	0.035
**177**	2-O8W-N1(	100	PI3K	0.252404	0.155
**178**	2-O8W-O1(	1000	PI3K	0.35364	0.001
**179**	2-O8W-P1C	10000	PI3K	0.369356	0.001
**180**	3-O8W-A16	2500	PI3K	0.088396	0.953
**181**	3-O8W-C16	250	PI3K	0.090506	0.799
**182**	3-O8W-D1(	25	PI3K	0.262168	0.385
**183**	3-O8W-E16	2.5	PI3K	0.334153	0.009
**184**	3-O8W-F16	0.25	PI3K	0.314879	0.026
**185**	3-O8W-F17	100000	PI3K	0.189716	0.995
**186**	3-O8W-F19	500	PI3K	0.209534	0.198
**187**	3-O8W-G17	10000	PI3K	0.176117	0.807
**188**	3-O8W-G19	50	PI3K	0.25141	0.162
**189**	3-O8W-H17	1000	PI3K	0.328602	0.026
**190**	3-O8W-I17	100	PI3K	0.111423	0.801
**191**	3-O8W-I19	5	PI3K	0.063288	0.981
**192**	3-O8W-J17	10	PI3K	0.150035	0.619
**193**	3-O8W-J19	0.5	PI3K	0.181678	0.804
**194**	3-O8W-K19	0.05	PI3K	0.286582	0.076
**195**	3-O8W-L8-I	1	PI3K	0.304033	0.125
**196**	3-O8W-L21	0.1	PI3K	0.199611	0.646
**197**	3-O8W-M8	10	PI3K	0.35451	0.001
**198**	3-O8W-M2	1	PI3K	0.369044	0.003
**199**	3-O8W-N8-	100	PI3K	0.279817	0.082
**200**	3-O8W-N2:	10	PI3K	0.141638	0.875
**201**	3-O8W-O8-	1000	PI3K	0.28371	0.061
**202**	3-O8W-O2:	100	PI3K	0.222218	0.086
**203**	3-O8W-P8-	10000	PI3K	0.317577	0.529
**204**	3-O8W-P21	1000	PI3K	0.341651	0.013
**205**	4-O8W-A19	2500	PI3K	0.154095	0.751

**206**	4-O8W-B1S	250	PI3K	0.161975	0.552
**207**	4-O8W-C1S	25	PI3K	0.252901	0.082
**208**	4-O8W-D1S	2.5	PI3K	0.202779	0.269
**209**	4-O8W-E1S	0.25	PI3K	0.335388	0.037
**210**	4-O8W-F14	1000	PI3K	0.126858	0.998
**211**	4-O8W-G2-	2500	PI3K	0.348508	0.011
**212**	4-O8W-G5-	10000	PI3K	0.327193	0.006
**213**	4-O8W-G14	100	PI3K	0.286883	0.754
**214**	4-O8W-G2(	10000	PI3K	0.212087	0.938
**215**	4-O8W-H2-	250	PI3K	0.176422	0.866
**216**	4-O8W-H5-	1000	PI3K	0.321971	0.061
**217**	4-O8W-H14	10	PI3K	0.292645	0.076
**218**	4-O8W-H2(	1000	PI3K	0.375527	0
**219**	4-O8W-I2-1	25	PI3K	0.226636	0.484
**220**	4-O8W-I5-S	100	PI3K	0.347594	0.007
**221**	4-O8W-I14	1	PI3K	0.388727	0
**222**	4-O8W-I20	100	PI3K	0.375183	0.004
**223**	4-O8W-J2-1	2.5	PI3K	0.343175	0.073
**224**	4-O8W-J5-S	10	PI3K	0.332504	0.031
**225**	4-O8W-J20	10	PI3K	0.04847	0.997
**226**	4-O8W-K2-	0.25	PI3K	0.300112	0.022
**227**	4-O8W-K4-	0.1	PI3K	0.293338	0.042
**228**	4-O8W-K5-	1	PI3K	0.32881	0.01
**229**	4-O8W-K14	0.1	PI3K	0.289559	0.021
**230**	4-O8W-K2(	1	PI3K	0.331484	0.053
**231**	4-O8W-L4-I	1	PI3K	0.313793	0.022
**232**	4-O8W-L14	0.1	PI3K	0.191832	0.352
**233**	4-O8W-L15	1	PI3K	0.415646	0
**234**	4-O8W-L21	0.1	PI3K	0.312257	0.115
**235**	4-O8W-M1	1	PI3K	0.29359	0.017
**236**	4-O8W-M1	10	PI3K	0.297046	0.045
**237**	4-O8W-M2	1	PI3K	0.198702	0.246
**238**	4-O8W-N4-	10	PI3K	0.276706	0.09
**239**	4-O8W-N14	10	PI3K	0.173085	0.783
**240**	4-O8W-N15	100	PI3K	0.175133	0.233

**241**	4-O8W-N2	10	PI3K	0.163466	0.715
**242**	4-O8W-O4-	100	PI3K	0.324597	0.142
**243**	4-O8W-O14	100	PI3K	0.316565	0.029
**244**	4-O8W-O15	1000	PI3K	0.38075	0
**245**	4-O8W-O2	100	PI3K	0.323375	0.006
**246**	4-O8W-P4-	1000	PI3K	0.284862	0.752
**247**	4-O8W-P14	1000	PI3K	0.275824	0.831
**248**	4-O8W-P15	10000	PI3K	0.411489	0
**249**	4-O8W-P21	1000	PI3K	0.369817	0.001
**250**	5-O8W-A6-	2500	PI3K	0.301286	0.706
**251**	5-O8W-A7-	1000	PI3K	0.372305	0.003
**252**	5-O8W-A16	2500	PI3K	0.340856	0.003
**253**	5-O8W-A17	10000	PI3K	0.31248	0.133
**254**	5-O8W-B6-	250	PI3K	0.286612	0.214
**255**	5-O8W-B7-	100	PI3K	0.246526	0.612
**256**	5-O8W-B17	1000	PI3K	0.328527	0.067
**257**	5-O8W-C6-	25	PI3K	0.285971	0.067
**258**	5-O8W-C7-	10	PI3K	0.19876	0.466
**259**	5-O8W-C16	250	PI3K	0.157923	0.625
**260**	5-O8W-C17	100	PI3K	0.254022	0.199
**261**	5-O8W-D6-	2.5	PI3K	0.192214	0.592
**262**	5-O8W-D7-	1	PI3K	0.222678	0.372
**263**	5-O8W-D16	25	PI3K	0.167947	0.272
**264**	5-O8W-D17	10	PI3K	0.225929	0.655
**265**	5-O8W-E6-	0.25	PI3K	0.328958	0.012
**266**	5-O8W-E7-	0.1	PI3K	0.210918	0.245
**267**	5-O8W-E16	2.5	PI3K	0.187781	0.312
**268**	5-O8W-E17	1	PI3K	0.202474	0.3
**269**	5-O8W-F11	10000	PI3K	0.199938	0.382
**270**	5-O8W-F16	0.25	PI3K	0.17235	0.773
**271**	5-O8W-G9-	10000	PI3K	0.38266	0.002
**272**	5-O8W-G1	1000	PI3K	0.213995	0.087
**273**	5-O8W-H9-	1000	PI3K	0.155023	0.354
**274**	5-O8W-H1	100	PI3K	0.289637	0.09
**275**	5-O8W-I9-	100	PI3K	0.254528	0.051

**276**	5-O8W-I11	10	PI3K	0.078187	0.794
**277**	5-O8W-J9-S	10	PI3K	0.235243	0.162
**278**	5-O8W-J11	1	PI3K	0.220164	0.074
**279**	5-O8W-K9-	1	PI3K	0.186908	0.617
**280**	5-O8W-L14	0.1	PI3K	0.139353	0.731
**281**	5-O8W-L2(	1	PI3K	0.425343	0
**282**	5-O8W-L23	0.1	PI3K	0.156404	0.68
**283**	5-O8W-M1	1	PI3K	0.298585	0.034
**284**	5-O8W-M2	10	PI3K	0.37865	0.002
**285**	5-O8W-M2	1	PI3K	0.202231	0.451
**286**	5-O8W-N14	10	PI3K	0.396245	0.002
**287**	5-O8W-N2(	100	PI3K	0.144565	0.698
**288**	5-O8W-N2:	10	PI3K	0.364085	0.002
**289**	5-O8W-O14	100	PI3K	0.401384	0
**290**	5-O8W-O2(	1000	PI3K	0.343825	0.011
**291**	5-O8W-O2:	100	PI3K	0.346401	0.006
**292**	5-O8W-P14	1000	PI3K	0.185457	0.316
**293**	5-O8W-P2(	10000	PI3K	0.364613	0.003
**294**	5-O8W-P2:	1000	PI3K	0.288954	0.707
**295**	6-O8W-A8-	10000	PI3K	0.333449	0.336
**296**	6-O8W-B8-	1000	PI3K	0.085135	0.962
**297**	6-O8W-C8-	100	PI3K	0.125975	0.636
**298**	6-O8W-D8-	10	PI3K	0.125351	0.872
**299**	6-O8W-E8-1	1	PI3K	0.11113	0.86
**300**	6-O8W-L6-(	1	PI3K	0.089253	0.826
**301**	6-O8W-M6	10	PI3K	0.151787	0.448
**302**	6-O8W-N6-	100	PI3K	0.122729	0.736
**303**	6-O8W-O6-	1000	PI3K	0.282443	0.028
**304**	6-O8W-P6-	10000	PI3K	0.206618	0.959
**305**	1-O8W-F11	10000	Topoisome	0.593339	0
**306**	1-O8W-G1:	1000	Topoisome	0.560649	0
**307**	1-O8W-G2(	1000	Topoisome	0.539671	0.003
**308**	1-O8W-H1:	100	Topoisome	0.393812	0.018
**309**	1-O8W-H2(	100	Topoisome	0.40485	0.021
**310**	1-O8W-I11	10	Topoisome	0.431356	0.002

**311**	1-O8W-I20	10	Topoisome	0.415097	0.016
**312**	1-O8W-J11	1	Topoisome	0.068234	0.96
**313**	1-O8W-J20	1	Topoisome	0.168438	0.593
**314**	1-O8W-K11	1	Topoisome	0.396452	0.007
**315**	1-O8W-K2(	0.1	Topoisome	0.288047	0.154
**316**	1-O8W-L11	10	Topoisome	0.566855	0
**317**	1-O8W-L14	1	Topoisome	0.323256	0.049
**318**	1-O8W-M1	100	Topoisome	0.599144	0
**319**	1-O8W-M1	10	Topoisome	0.386048	0.006
**320**	1-O8W-N1	100	Topoisome	0.434992	0.003
**321**	1-O8W-O1	1000	Topoisome	0.218959	0.557
**322**	1-O8W-O1	1000	Topoisome	0.569612	0
**323**	1-O8W-P11	10000	Topoisome	0.250868	0.821
**324**	1-O8W-P1	10000	Topoisome	0.466166	0.034
**325**	3-O8W-A11	10000	Topoisome	0.546271	0.001
**326**	3-O8W-B11	1000	Topoisome	0.448854	0.002
**327**	3-O8W-C11	100	Topoisome	0.489798	0
**328**	3-O8W-D1	10	Topoisome	0.131607	0.853
**329**	3-O8W-E11	1	Topoisome	0.100294	0.91
**330**	3-O8W-G9-	1000	Topoisome	0.325565	0.182
**331**	3-O8W-G1(	10000	Topoisome	0.477031	0
**332**	3-O8W-H9-	100	Topoisome	0.589783	0
**333**	3-O8W-H1(	1000	Topoisome	0.464921	0
**334**	3-O8W-I9-[	10	Topoisome	0.408423	0.008
**335**	3-O8W-I10	100	Topoisome	0.126772	0.783
**336**	3-O8W-J9-I	1	Topoisome	0.422638	0
**337**	3-O8W-J10	10	Topoisome	0.262176	0.18
**338**	3-O8W-K7-	0.1	Topoisome	0.269993	0.154
**339**	3-O8W-K9-	0.1	Topoisome	0.232175	0.504
**340**	3-O8W-K1(	1	Topoisome	0.272737	0.409
**341**	3-O8W-L6-I	0.1	Topoisome	0.272335	0.211
**342**	3-O8W-L7-I	1	Topoisome	0.375344	0.007
**343**	3-O8W-L9-'	0.5	Topoisome	0.479076	0
**344**	3-O8W-L1C	0.1	Topoisome	0.491747	0
**345**	3-O8W-L16	1	Topoisome	0.421205	0.02

346	3-O8W-M6	1	Topoisome	0.405744	0.016
347	3-O8W-M7	10	Topoisome	0.412528	0.002
348	3-O8W-M9	5	Topoisome	0.418702	0.003
349	3-O8W-M1	1	Topoisome	0.44545	0.001
350	3-O8W-M1	10	Topoisome	0.382288	0.008
351	3-O8W-N6-	10	Topoisome	0.443404	0.001
352	3-O8W-N9-	50	Topoisome	0.406645	0.002
353	3-O8W-N1(	10	Topoisome	0.38763	0.022
354	3-O8W-N1(	100	Topoisome	0.06251	0.986
355	3-O8W-O6-	100	Topoisome	0.48185	0.001
356	3-O8W-O7-	100	Topoisome	0.618326	0
357	3-O8W-O9-	500	Topoisome	0.5814	0
358	3-O8W-O1(	100	Topoisome	0.609063	0
359	3-O8W-O1(	1000	Topoisome	0.102475	0.883
360	3-O8W-P6-	1000	Topoisome	0.544198	0.001
361	3-O8W-P7-	1000	Topoisome	0.310108	0.237
362	3-O8W-P9-	5000	Topoisome	0.52898	0.001
363	3-O8W-P1(	1000	Topoisome	0.522835	0.007
364	3-O8W-P1(	10000	Topoisome	0.381139	0.021
365	1-O8W-A1(	10000	Mitotic	0.63832	0
366	1-O8W-A1:	1000	Mitotic	0.738819	0
367	1-O8W-A1ε	1000	Mitotic	0.748891	0
368	1-O8W-B1(	1000	Mitotic	0.728631	0
369	1-O8W-B1:	100	Mitotic	0.438959	0.005
370	1-O8W-B1ε	100	Mitotic	0.731498	0
371	1-O8W-C1(	100	Mitotic	0.737645	0
372	1-O8W-C1:	10	Mitotic	0.506059	0
373	1-O8W-C1ε	10	Mitotic	0.70512	0
374	1-O8W-D1(	10	Mitotic	0.526745	0.006
375	1-O8W-D1:	1	Mitotic	0.22557	0.434
376	1-O8W-D1ε	1	Mitotic	0.40128	0.006
377	1-O8W-E1C	1	Mitotic	0.509458	0
378	1-O8W-E13	0.1	Mitotic	0.110937	0.896
379	1-O8W-E1ε	0.1	Mitotic	0.383391	0.009
380	1-O8W-F13	1000	Mitotic	0.746256	0

**381**	1-O8W-G1:	100	Mitotic	0.421272	0.005
**382**	1-O8W-G1!	1000	Mitotic	0.656387	0
**383**	1-O8W-H1:	10	Mitotic	0.391213	0.01
**384**	1-O8W-H1!	100	Mitotic	0.739505	0
**385**	1-O8W-I13	1	Mitotic	0.329743	0.171
**386**	1-O8W-I15	10	Mitotic	0.72099	0
**387**	1-O8W-J13	0.1	Mitotic	0.119401	0.85
**388**	1-O8W-J15	1	Mitotic	0.39628	0.029
**389**	1-O8W-K7-	0.1	Mitotic	0.344719	0.103
**390**	1-O8W-K1!	0.1	Mitotic	0.426257	0.003
**391**	1-O8W-L7-'	1	Mitotic	0.37251	0.141
**392**	1-O8W-L2(	0.1	Mitotic	0.16583	0.947
**393**	1-O8W-M7	10	Mitotic	0.29899	0.215
**394**	1-O8W-M2	1	Mitotic	0.355219	0.054
**395**	1-O8W-N2(	10	Mitotic	0.273066	0.416
**396**	1-O8W-O7-	100	Mitotic	0.336969	0.254
**397**	1-O8W-O2(	100	Mitotic	0.265738	0.373
**398**	1-O8W-P7-	1000	Mitotic	0.66296	0
**399**	1-O8W-P2(	1000	Mitotic	0.66654	0
**400**	3-O8W-A7-	1000	Mitotic	0.706023	0
**401**	3-O8W-B7-	100	Mitotic	0.723335	0
**402**	3-O8W-C7-	10	Mitotic	0.673836	0
**403**	3-O8W-D7-	1	Mitotic	0.368717	0.039
**404**	3-O8W-E7-I	0.1	Mitotic	0.344567	0.043
**405**	6-O8W-L1S	1	Mitotic	0.122529	0.874
**406**	6-O8W-M1	10	Mitotic	0.125921	0.974
**407**	6-O8W-N1S	100	Mitotic	0.158754	0.569
**408**	6-O8W-O1S	1000	Mitotic	0.676255	0
**409**	6-O8W-P1S	10000	Mitotic	0.711738	0
**410**	2-O8W-A1:	250	MEK1/2	0.568052	0.003
**411**	2-O8W-B1:	25	MEK1/2	0.71008	0
**412**	2-O8W-D1:	2.5	MEK1/2	0.69627	0
**413**	2-O8W-E12	0.25	MEK1/2	0.515023	0.001
**414**	2-O8W-F12	2.5000000	MEK1/2	0.122071	0.978
**415**	2-O8W-F14	1000	MEK1/2	0.525781	0.01

**416**	2-O8W-G1‹	100	MEK1/2	0.720697	0
**417**	2-O8W-H1‹	10	MEK1/2	0.646836	0
**418**	2-O8W-I14	1	MEK1/2	0.175258	0.973
**419**	2-O8W-K1‹	0.1	MEK1/2	0.514383	0.009
**420**	2-O8W-L2(	1	MEK1/2	0.514078	0.007
**421**	2-O8W-M2	10	MEK1/2	0.536099	0.001
**422**	2-O8W-N2(	100	MEK1/2	0.656645	0
**423**	2-O8W-O2(	1000	MEK1/2	0.688951	0
**424**	2-O8W-P2(	10000	MEK1/2	0.717321	0
**425**	4-O8W-A1(	1000	MEK1/2	0.74395	0
**426**	4-O8W-A1:	1000	MEK1/2	0.720985	0
**427**	4-O8W-B1(	100	MEK1/2	0.73788	0
**428**	4-O8W-B1:	100	MEK1/2	0.785811	0
**429**	4-O8W-C1(	10	MEK1/2	0.657575	0
**430**	4-O8W-C1:	10	MEK1/2	0.770504	0
**431**	4-O8W-D1(	1	MEK1/2	0.198428	0.892
**432**	4-O8W-D1:	1	MEK1/2	0.221252	0.608
**433**	4-O8W-E1(	0.1	MEK1/2	0.171968	0.889
**434**	4-O8W-E13	0.1	MEK1/2	0.524582	0.001
**435**	4-O8W-L1(	0.25	MEK1/2	0.498402	0.009
**436**	4-O8W-M1	2.5	MEK1/2	0.216091	0.893
**437**	4-O8W-N1(	25	MEK1/2	0.271339	0.71
**438**	4-O8W-O1(	250	MEK1/2	0.621824	0
**439**	4-O8W-P1(	2500	MEK1/2	0.727857	0
**440**	1-O8W-L2-(	1	PARP	0.50717	0.019
**441**	1-O8W-L6-(	1	PARP	0.375439	0.491
**442**	1-O8W-M2	10	PARP	0.53507	0.006
**443**	1-O8W-M6	10	PARP	0.099874	0.996
**444**	1-O8W-N2-	100	PARP	0.572131	0
**445**	1-O8W-N6-	100	PARP	0.506226	0.026
**446**	1-O8W-O2-	1000	PARP	0.618636	0.001
**447**	1-O8W-O6-	1000	PARP	0.576154	0.001
**448**	1-O8W-P2-	10000	PARP	0.578472	0.008
**449**	1-O8W-P6-	10000	PARP	0.59533	0
**450**	7-O8W-A3-	1000	PARP	0.607264	0.001

**451**	7-O8W-B2-	10000	PARP	0.546701	0.005
**452**	7-O8W-B3-	100	PARP	0.627513	0.001
**453**	7-O8W-C2-	1000	PARP	0.505725	0.026
**454**	7-O8W-C3-	10	PARP	0.599333	0
**455**	7-O8W-D2-	100	PARP	0.437211	0.128
**456**	7-O8W-D3-	1	PARP	0.663631	0
**457**	7-O8W-E2-'	10	PARP	0.575454	0.006
**458**	7-O8W-E3-'	0.1	PARP	0.552883	0.003
**459**	7-O8W-F2-'	1	PARP	0.083066	1
**460**	7-O8W-G2-	10000	PARP	0.629705	0.006
**461**	7-O8W-H2-	1000	PARP	0.637875	0
**462**	7-O8W-I2-N	100	PARP	0.520801	0.004
**463**	7-O8W-J2-I	10	PARP	0.307155	0.583
**464**	7-O8W-K2-	1	PARP	0.203201	0.761
**465**	3-O8W-A19	1000	CDK	0.434871	0.045
**466**	3-O8W-B19	100	CDK	0.456466	0.021
**467**	3-O8W-B23	2500	CDK	0.367826	0.027
**468**	3-O8W-C19	10	CDK	0.405483	0
**469**	3-O8W-C23	250	CDK	0.294503	0.094
**470**	3-O8W-D19	1	CDK	0.282707	0.211
**471**	3-O8W-D23	25	CDK	0.117387	0.912
**472**	3-O8W-E19	0.1	CDK	0.367155	0.112
**473**	3-O8W-E23	2.5	CDK	0.139159	0.94
**474**	3-O8W-F23	0.25	CDK	0.085163	0.972
**475**	3-O8W-K17	1	CDK	0.380842	0.017
**476**	3-O8W-L19	1	CDK	0.279008	0.142
**477**	3-O8W-M1	10	CDK	0.351731	0.126
**478**	3-O8W-M1	10	CDK	0.5067	0
**479**	3-O8W-N17	100	CDK	0.370239	0.009
**480**	3-O8W-N19	100	CDK	0.277839	0.198
**481**	3-O8W-O17	1000	CDK	0.339907	0.028
**482**	3-O8W-O19	1000	CDK	0.373567	0.014
**483**	3-O8W-P17	10000	CDK	0.429936	0.002
**484**	3-O8W-P19	10000	CDK	0.398715	0.011
**485**	4-O8W-A4-	10000	CDK	0.403898	0.055

**486**	4-O8W-A8-	10000	CDK	0.415175	0.013
**487**	4-O8W-B4-	1000	CDK	0.521184	0.005
**488**	4-O8W-B8-	1000	CDK	0.127372	0.981
**489**	4-O8W-C4-	100	CDK	0.319888	0.11
**490**	4-O8W-C8-	100	CDK	0.143509	0.975
**491**	4-O8W-D4-	10	CDK	0.338938	0.036
**492**	4-O8W-D8-	10	CDK	0.122444	0.841
**493**	4-O8W-E4-	1	CDK	0.412556	0.005
**494**	4-O8W-E8-	1	CDK	0.119624	0.977
**495**	4-O8W-F4-	10000	CDK	0.398776	0.036
**496**	4-O8W-F22	10000	CDK	0.535017	0.004
**497**	4-O8W-G4-	1000	CDK	0.448238	0.003
**498**	4-O8W-G2	1000	CDK	0.423714	0.057
**499**	4-O8W-H4-	100	CDK	0.331557	0.159
**500**	4-O8W-H2	100	CDK	0.282911	0.497
**501**	4-O8W-I4-	10	CDK	0.340805	0.075
**502**	4-O8W-I22	10	CDK	0.425716	0.005
**503**	4-O8W-J4-	1	CDK	0.189284	0.89
**504**	4-O8W-J22	1	CDK	0.437222	0.019
**505**	5-O8W-A1	10000	CDK	0.449273	0.158
**506**	5-O8W-B1	1000	CDK	0.351265	0.46
**507**	5-O8W-C1	100	CDK	0.409312	0.009
**508**	5-O8W-D1	10	CDK	0.410085	0.001
**509**	5-O8W-E1	1	CDK	0.355948	0.07
**510**	5-O8W-K1	1	CDK	0.274959	0.373
**511**	5-O8W-M1	10	CDK	0.332358	0.055
**512**	5-O8W-N1	100	CDK	0.411171	0.012
**513**	5-O8W-O1	1000	CDK	0.433999	0.174
**514**	5-O8W-P1	10000	CDK	0.436592	0.04
**515**	6-O8W-A1	1000	CDK	0.417865	0.013
**516**	6-O8W-B1	100	CDK	0.316377	0.13
**517**	6-O8W-C1	10	CDK	0.267735	0.2
**518**	6-O8W-D1	1	CDK	0.421107	0.004
**519**	6-O8W-E17	0.1	CDK	0.080163	0.917
**520**	6-O8W-L1	1	CDK	0.106463	0.994

521	6-O8W-M1	10	CDK	0.160898	0.599
522	6-O8W-N1!	100	CDK	0.28908	0.05
523	6-O8W-O1!	1000	CDK	0.383159	0.06
524	6-O8W-P1!	10000	CDK	0.439918	0.141
525	7-O8W-A21	10000	BET	0.551844	0.002
526	7-O8W-A2:	30000	BET	0.489936	0
527	7-O8W-B21	1000	BET	0.248009	0.609
528	7-O8W-B2:	3000	BET	0.491227	0
529	7-O8W-C21	100	BET	0.287527	0.367
530	7-O8W-C2:	300	BET	0.445155	0.003
531	7-O8W-D2:	10	BET	0.299559	0.486
532	7-O8W-D2:	30	BET	0.390469	0.086
533	7-O8W-E21	1	BET	0.134609	0.843
534	7-O8W-E22	3	BET	0.281818	0.154
535	7-O8W-G1(	10000	BET	0.552102	0.003
536	7-O8W-G1!	10000	BET	0.517119	0.013
537	7-O8W-H1(	1000	BET	0.516456	0
538	7-O8W-H1!	1000	BET	0.393986	0.001
539	7-O8W-I10	100	BET	0.580577	0
540	7-O8W-I15	100	BET	0.539709	0
541	7-O8W-J10	10	BET	0.625234	0
542	7-O8W-J15	10	BET	0.376246	0.039
543	7-O8W-K1(	1	BET	0.123388	0.987
544	7-O8W-K1:	1	BET	0.573765	0
545	7-O8W-K1!	1	BET	0.488125	0
546	7-O8W-L12	1	BET	0.088308	0.999
547	7-O8W-L13	10	BET	0.458705	0
548	7-O8W-L2C	1	BET	0.259396	0.256
549	7-O8W-L23	0.03	BET	0.302335	0.111
550	7-O8W-M1	10	BET	0.211414	0.633
551	7-O8W-M1	100	BET	0.569386	0
552	7-O8W-M2	10	BET	0.205906	0.803
553	7-O8W-M2	0.3	BET	0.30906	0.054
554	7-O8W-N1:	100	BET	0.329258	0.174
555	7-O8W-N1:	1000	BET	0.541708	0.017

**556**	7-O8W-N2(	100	BET	0.545696	0
**557**	7-O8W-N2:	3	BET	0.471656	0
**558**	7-O8W-O1:	1000	BET	0.402493	0.019
**559**	7-O8W-O2(	1000	BET	0.530881	0.008
**560**	7-O8W-O2:	30	BET	0.563212	0
**561**	7-O8W-P1:	10000	BET	0.572963	0.002
**562**	7-O8W-P1:	10000	BET	0.409547	0.382
**563**	7-O8W-P2(	10000	BET	0.548451	0.009
**564**	7-O8W-P2:	300	BET	0.317272	0.647
**565**	8-O8W-K2:	1	BET	0.272239	0.132
**566**	8-O8W-L22	10	BET	0.227521	0.652
**567**	8-O8W-M2	100	BET	0.231706	0.369
**568**	8-O8W-N2:	1000	BET	0.287601	0.184
**569**	8-O8W-O2:	10000	BET	0.503495	0.026
**570**	1-O8W-A3-	10000	HDAC	0.501131	0.008
**571**	1-O8W-B3-	1000	HDAC	0.425235	0
**572**	1-O8W-C3-	100	HDAC	0.362267	0.014
**573**	1-O8W-D3-	10	HDAC	0.398973	0.001
**574**	1-O8W-E3-'	1	HDAC	0.397596	0
**575**	1-O8W-L12	0.1	HDAC	0.098685	0.861
**576**	1-O8W-M1	1	HDAC	0.299355	0.051
**577**	1-O8W-N1:	10	HDAC	0.478317	0
**578**	1-O8W-O1:	100	HDAC	0.501905	0.001
**579**	1-O8W-P1:	1000	HDAC	0.297096	0.193
**580**	3-O8W-A4-	1000	HDAC	0.412481	0.085
**581**	3-O8W-B4-	100	HDAC	0.519087	0
**582**	3-O8W-C4-	10	HDAC	0.448532	0
**583**	3-O8W-D4-	1	HDAC	0.387273	0.003
**584**	3-O8W-E4-I	0.1	HDAC	0.396404	0
**585**	3-O8W-F7-(	1000	HDAC	0.397136	0.019
**586**	3-O8W-G7-	100	HDAC	0.47775	0
**587**	3-O8W-G1:	1000000	HDAC	0.108351	0.932
**588**	3-O8W-H7-	10	HDAC	0.392445	0.002
**589**	3-O8W-H1:	100000	HDAC	0.220746	0.115
**590**	3-O8W-I7-(	1	HDAC	0.080942	0.858

**591**	3-O8W-I12	10000	HDAC	0.080634	0.91
**592**	3-O8W-J7-(	0.1	HDAC	0.180355	0.317
**593**	3-O8W-J12	1000	HDAC	0.083482	0.929
**594**	3-O8W-K3-	1	HDAC	0.259619	0.584
**595**	3-O8W-K12	100	HDAC	0.082318	0.941
**596**	3-O8W-L3-I	10	HDAC	0.063996	0.988
**597**	3-O8W-M3	100	HDAC	0.194014	0.596
**598**	3-O8W-N3-	1000	HDAC	0.48322	0.002
**599**	3-O8W-O3-	10000	HDAC	0.423767	0.06
**600**	7-O8W-A5-	10000	HDAC	0.418715	0.007
**601**	7-O8W-A7-	10000	HDAC	0.359161	0.226
**602**	7-O8W-A9-	1000	HDAC	0.528323	0
**603**	7-O8W-A12	10000	HDAC	0.536737	0
**604**	7-O8W-B5-	1000	HDAC	0.509964	0
**605**	7-O8W-B7-	1000	HDAC	0.401758	0.128
**606**	7-O8W-B12	1000	HDAC	0.470154	0
**607**	7-O8W-C5-	100	HDAC	0.432691	0
**608**	7-O8W-C7-	100	HDAC	0.412947	0.078
**609**	7-O8W-C9-	100	HDAC	0.472534	0
**610**	7-O8W-D7-	10	HDAC	0.531584	0
**611**	7-O8W-D9-	10	HDAC	0.541274	0
**612**	7-O8W-D12	100	HDAC	0.064613	0.993
**613**	7-O8W-E5-I	10	HDAC	0.441054	0
**614**	7-O8W-E7-(	1	HDAC	0.428388	0.001
**615**	7-O8W-E9-(	1	HDAC	0.447019	0
**616**	7-O8W-E12	10	HDAC	0.157468	0.835
**617**	7-O8W-F5-I	1	HDAC	0.395459	0
**618**	7-O8W-F7-I	10000	HDAC	0.332614	0.051
**619**	7-O8W-F9-(	0.1	HDAC	0.333954	0
**620**	7-O8W-F12	1	HDAC	0.237684	0.025
**621**	7-O8W-F19	10000	HDAC	0.440864	0
**622**	7-O8W-G7-	1000	HDAC	0.498436	0
**623**	7-O8W-G19	1000	HDAC	0.439735	0
**624**	7-O8W-H7-	100	HDAC	0.459739	0
**625**	7-O8W-I7-F	10	HDAC	0.361657	0.013

**626**	7-O8W-I19	100	HDAC	0.403136	0
**627**	7-O8W-J7-I	1	HDAC	0.177016	0.765
**628**	7-O8W-J19	10	HDAC	0.456805	0.002
**629**	7-O8W-K4-	1	HDAC	0.269087	0.29
**630**	7-O8W-K11	1	HDAC	0.059094	0.98
**631**	7-O8W-K18	1	HDAC	0.129355	0.875
**632**	7-O8W-K19	1	HDAC	0.072126	0.959
**633**	7-O8W-L2-[illegible]	0.1	HDAC	0.110351	0.955
**634**	7-O8W-L4-I	10	HDAC	0.065328	0.956
**635**	7-O8W-L5-I	1	HDAC	0.248482	0.176
**636**	7-O8W-L8-,	1	HDAC	0.454722	0
**637**	7-O8W-L10	1	HDAC	0.390828	0
**638**	7-O8W-L11	10	HDAC	0.453474	0
**639**	7-O8W-L14	1	HDAC	0.101637	0.776
**640**	7-O8W-L16	1	HDAC	0.071483	0.961
**641**	7-O8W-L18	10	HDAC	0.093666	0.858
**642**	7-O8W-M2	1	HDAC	0.457924	0.001
**643**	7-O8W-M5	10	HDAC	0.27198	0.033
**644**	7-O8W-M8	10	HDAC	0.504354	0
**645**	7-O8W-M1	10	HDAC	0.441808	0
**646**	7-O8W-M1	100	HDAC	0.380941	0.001
**647**	7-O8W-M1	10	HDAC	0.399342	0.001
**648**	7-O8W-M1	10	HDAC	0.26305	0.3
**649**	7-O8W-M1	100	HDAC	0.334658	0.061
**650**	7-O8W-N2-	10	HDAC	0.430151	0
**651**	7-O8W-N4-	100	HDAC	0.46555	0
**652**	7-O8W-N5-	100	HDAC	0.458185	0
**653**	7-O8W-N8-	100	HDAC	0.453196	0
**654**	7-O8W-N1(	100	HDAC	0.377612	0
**655**	7-O8W-N14	100	HDAC	0.370794	0.004
**656**	7-O8W-N16	100	HDAC	0.186785	0.454
**657**	7-O8W-N18	1000	HDAC	0.040258	1
**658**	7-O8W-O2-	100	HDAC	0.381735	0
**659**	7-O8W-O4-	1000	HDAC	0.543558	0
**660**	7-O8W-O5-	1000	HDAC	0.549505	0

**661**	7-O8W-O8-	1000	HDAC	0.529672	0
**662**	7-O8W-O1(	1000	HDAC	0.459936	0
**663**	7-O8W-O1:	1000	HDAC	0.558	0
**664**	7-O8W-O14	1000	HDAC	0.395726	0.001
**665**	7-O8W-O1(	1000	HDAC	0.528034	0
**666**	7-O8W-P2-	1000	HDAC	0.356176	0
**667**	7-O8W-P4-	10000	HDAC	0.521317	0
**668**	7-O8W-P5-	10000	HDAC	0.439124	0.037
**669**	7-O8W-P8-	10000	HDAC	0.431781	0.077
**670**	7-O8W-P1(	10000	HDAC	0.535635	0
**671**	7-O8W-P1:	10000	HDAC	0.333565	0.058
**672**	7-O8W-P14	10000	HDAC	0.457679	0
**673**	7-O8W-P1(	10000	HDAC	0.460732	0
**674**	7-O8W-P18	10000	HDAC	0.470925	0
