# Supplementary material for: Evaluating feature extraction in ovarian cancer cell line co-cultures using deep neural networks
Source: Commun Biol. 2025 Feb 25;8:303. doi: 10.1038/s42003-025-07766-w (PMC11862010; doi:10.1038/s42003-025-07766-w)
Supplement: Supplementary file 6 — Supplementary Data 4 [file 42003_2025_7766_MOESM6_ESM.pdf]

	Well_annotati	_concentra	ell_Catego	Highest_ES	Pvalue
0	2-KB-A16-G	10000	EGFR	0.276325	0.181
1	2-KB-A19-E	10000	EGFR	0.295893	0.071
2	2-KB-B19-E	1000	EGFR	0.323256	0.038
3	2-KB-C16-G	1000	EGFR	0.329233	0.012
4	2-KB-C19-E	100	EGFR	0.336621	0.032
5	2-KB-D16-G	100	EGFR	0.309978	0.022
6	2-KB-D19-E	10	EGFR	0.449125	0.001
7	2-KB-E16-G	10	EGFR	0.364859	0.005
8	2-KB-E19-E	1	EGFR	0.291297	0.111
9	2-KB-F16-G	1	EGFR	0.33682	0.02
10	2-KB-K11-A	0.1	EGFR	0.348252	0.023
11	2-KB-L11-A	1	EGFR	0.388812	0.009
12	2-KB-L16-O	0.25	EGFR	0.294932	0.093
13	2-KB-L19-L	0.1	EGFR	0.353279	0.002
14	2-KB-M11-	10	EGFR	0.303587	0.043
15	2-KB-M16-	2.5	EGFR	0.372317	0.002
16	2-KB-M19-	1	EGFR	0.264489	0.171
17	2-KB-N16-C	25	EGFR	0.288852	0.078
18	2-KB-N19-L	10	EGFR	0.091746	0.71
19	2-KB-O11-A	100	EGFR	0.311384	0.041
20	2-KB-O16-C	250	EGFR	0.295005	0.108
21	2-KB-O19-L	100	EGFR	0.341751	0.024
22	2-KB-P11-A	1000	EGFR	0.318133	0.157
23	2-KB-P16-C	2500	EGFR	0.282899	0.116
24	2-KB-P19-L	1000	EGFR	0.219964	0.505
25	3-KB-F21-R	10000	EGFR	0.214488	0.663
26	3-KB-G20-N	1000	EGFR	0.255139	0.224
27	3-KB-G21-R	1000	EGFR	0.291633	0.053
28	3-KB-H20-N	100	EGFR	0.118519	0.53
29	3-KB-H21-R	100	EGFR	0.108608	0.814
30	3-KB-I20-N	10	EGFR	0.09281	0.685
31	3-KB-I21-R	10	EGFR	0.293979	0.444
32	3-KB-J20-N	1	EGFR	0.126334	0.583
33	3-KB-J21-R	1	EGFR	0.138052	0.741

**34**	3-KB-K4-Ca	1	EGFR	0.204637	0.221
**35**	3-KB-K18-D	0.1	EGFR	0.122479	0.491
**36**	3-KB-K20-N	0.1	EGFR	0.197348	0.654
**37**	3-KB-L4-Ca	10	EGFR	0.124146	0.514
**38**	3-KB-L18-D	1	EGFR	0.158647	0.347
**39**	3-KB-M18-I	10	EGFR	0.317935	0.021
**40**	3-KB-N4-Ca	100	EGFR	0.13676	0.924
**41**	3-KB-N18-D	100	EGFR	0.354912	0
**42**	3-KB-O4-Ca	1000	EGFR	0.234759	0.369
**43**	3-KB-P4-Ca	10000	EGFR	0.223768	0.438
**44**	3-KB-P18-D	1000	EGFR	0.248481	0.237
**45**	4-KB-F13-Sa	1000	EGFR	0.288352	0.151
**46**	4-KB-G13-S	100	EGFR	0.296608	0.113
**47**	4-KB-G16-V	10000	EGFR	0.271961	0.217
**48**	4-KB-H13-S	10	EGFR	0.340642	0.005
**49**	4-KB-H16-V	1000	EGFR	0.257287	0.242
**50**	4-KB-I13-Sa	1	EGFR	0.414777	0.001
**51**	4-KB-I16-Va	100	EGFR	0.079503	0.875
**52**	4-KB-J13-Sa	0.1	EGFR	0.061248	0.886
**53**	4-KB-J16-Va	10	EGFR	0.103753	0.569
**54**	4-KB-K7-Ico	1	EGFR	0.123131	0.623
**55**	4-KB-K13-T	0.1	EGFR	0.237837	0.249
**56**	4-KB-K16-V	1	EGFR	0.287511	0.162
**57**	4-KB-L7-Ico	10	EGFR	0.160619	0.257
**58**	4-KB-L13-Te	1	EGFR	0.414827	0
**59**	4-KB-M7-Ic	100	EGFR	0.31301	0.038
**60**	4-KB-M13-T	10	EGFR	0.315319	0.035
**61**	4-KB-N13-T	100	EGFR	0.282352	0.143
**62**	4-KB-O7-Ico	1000	EGFR	0.240997	0.168
**63**	4-KB-P7-Ico	10000	EGFR	0.348576	0.008
**64**	4-KB-P13-T	1000	EGFR	0.282866	0.189
**65**	5-KB-F4-Po	1000	EGFR	0.252425	0.294
**66**	5-KB-F7-AZ	1000	EGFR	0.298596	0.058
**67**	5-KB-G4-Po	100	EGFR	0.309787	0.234
**68**	5-KB-G7-AZ	100	EGFR	0.301571	0.016

**69**	5-KB-H4-Pc	10	EGFR	0.259688	0.248
**70**	5-KB-H7-AZ	10	EGFR	0.12018	0.396
**71**	5-KB-I4-Po:	1	EGFR	0.256997	0.329
**72**	5-KB-I7-AZI	1	EGFR	0.093467	0.761
**73**	5-KB-J4-Po:	0.1	EGFR	0.359817	0.001
**74**	5-KB-J7-AZI	0.1	EGFR	0.143469	0.508
**75**	5-KB-K7-Ol	0.1	EGFR	0.274764	0.287
**76**	5-KB-L7-Ol	1	EGFR	0.074533	0.926
**77**	5-KB-M7-Ol	10	EGFR	0.262916	0.268
**78**	5-KB-O7-Ol	100	EGFR	0.253201	0.252
**79**	5-KB-P7-Ol	1000	EGFR	0.231158	0.358
**80**	2-KB-A15-L	2500	VEGFR	0.342682	0.011
**81**	2-KB-A17-N	10000	VEGFR	0.32733	0.024
**82**	2-KB-A20-T	10000	VEGFR	0.359415	0.003
**83**	2-KB-B15-L	250	VEGFR	0.371334	0.002
**84**	2-KB-B17-N	1000	VEGFR	0.350353	0.006
**85**	2-KB-B20-T	1000	VEGFR	0.372587	0.002
**86**	2-KB-C15-L	25	VEGFR	0.38996	0.001
**87**	2-KB-C17-N	100	VEGFR	0.338576	0.004
**88**	2-KB-D15-L	2.5	VEGFR	0.246086	0.081
**89**	2-KB-D17-N	10	VEGFR	0.276798	0.154
**90**	2-KB-D20-T	100	VEGFR	0.357859	0.001
**91**	2-KB-E17-N	1	VEGFR	0.33852	0.006
**92**	2-KB-E20-Ti	10	VEGFR	0.332051	0.003
**93**	2-KB-F13-A	10000	VEGFR	0.275338	0.147
**94**	2-KB-F15-L	0.25	VEGFR	0.467457	0
**95**	2-KB-F19-R	10000	VEGFR	0.361101	0.003
**96**	2-KB-F20-Ti	1	VEGFR	0.311714	0.096
**97**	2-KB-F21-V	10000	VEGFR	0.233394	0.436
**98**	2-KB-G10-A	10000	VEGFR	0.306837	0.014
**99**	2-KB-G13-A	1000	VEGFR	0.341756	0.004
**100**	2-KB-G19-R	1000	VEGFR	0.288229	0.109
**101**	2-KB-G21-V	1000	VEGFR	0.267102	0.18
**102**	2-KB-H10-A	1000	VEGFR	0.389156	0.01
**103**	2-KB-H13-A	100	VEGFR	0.257748	0.211

**104**	2-KB-H21-V	100	VEGFR	0.166345	0.563
**105**	2-KB-I10-Ap	100	VEGFR	0.203839	0.048
**106**	2-KB-I13-Ax	10	VEGFR	0.382281	0.001
**107**	2-KB-I19-Re	100	VEGFR	0.267999	0.176
**108**	2-KB-I21-Va	10	VEGFR	0.1889	0.758
**109**	2-KB-J10-Ap	10	VEGFR	0.40132	0.002
**110**	2-KB-J13-A:	1	VEGFR	0.366845	0.003
**111**	2-KB-J19-Re	10	VEGFR	0.125046	0.492
**112**	2-KB-J21-Va	1	VEGFR	0.275346	0.144
**113**	2-KB-K10-A	1	VEGFR	0.129035	0.258
**114**	2-KB-K13-V	0.1	VEGFR	0.306681	0
**115**	2-KB-K17-P	1	VEGFR	0.177848	0.129
**116**	2-KB-K19-R	1	VEGFR	0.165773	0.347
**117**	2-KB-L12-S	0.1	VEGFR	0.180449	0.056
**118**	2-KB-L13-V	1	VEGFR	0.137065	0.196
**119**	2-KB-L21-C	0.1	VEGFR	0.335594	0.012
**120**	2-KB-M12-S	1	VEGFR	0.288723	0.018
**121**	2-KB-M13-V	10	VEGFR	0.335737	0
**122**	2-KB-M17-I	10	VEGFR	0.30473	0.022
**123**	2-KB-M21-C	1	VEGFR	0.384156	0.002
**124**	2-KB-N12-S	10	VEGFR	0.378415	0
**125**	2-KB-N13-V	100	VEGFR	0.356208	0.003
**126**	2-KB-N17-F	100	VEGFR	0.416886	0
**127**	2-KB-N21-C	10	VEGFR	0.273103	0.073
**128**	2-KB-O12-S	100	VEGFR	0.269841	0.233
**129**	2-KB-O17-F	1000	VEGFR	0.334578	0.02
**130**	2-KB-O21-C	100	VEGFR	0.28947	0.121
**131**	2-KB-P12-S	1000	VEGFR	0.16768	0.866
**132**	2-KB-P13-V	1000	VEGFR	0.302002	0.059
**133**	2-KB-P17-P	10000	VEGFR	0.300196	0.123
**134**	2-KB-P21-C	1000	VEGFR	0.35195	0.006
**135**	3-KB-A3-Ca	1000	VEGFR	0.362665	0.029
**136**	3-KB-A6-Fo	1000	VEGFR	0.250095	0.295
**137**	3-KB-A18-L	1000	VEGFR	0.125391	0.512
**138**	3-KB-B3-Ca	100	VEGFR	0.219923	0.299

**139**	3-KB-B6-Fo	100	VEGFR	0.327904	0.198
**140**	3-KB-B18-L	100	VEGFR	0.173375	0.36
**141**	3-KB-C3-Ca	10	VEGFR	0.157712	0.304
**142**	3-KB-C6-Fo	10	VEGFR	0.240726	0.449
**143**	3-KB-C18-L	10	VEGFR	0.116074	0.411
**144**	3-KB-D3-Ca	1	VEGFR	0.168609	0.304
**145**	3-KB-D6-Fo	1	VEGFR	0.107724	0.483
**146**	3-KB-D18-L	1	VEGFR	0.299128	0.002
**147**	3-KB-E3-Cal	0.1	VEGFR	0.151416	0.323
**148**	3-KB-E6-Fo	0.1	VEGFR	0.130018	0.431
**149**	3-KB-E18-Li	0.1	VEGFR	0.12205	0.928
**150**	3-KB-F18-B	1000	VEGFR	0.273335	0.303
**151**	3-KB-G18-E	100	VEGFR	0.349254	0
**152**	3-KB-H18-E	10	VEGFR	0.068935	0.882
**153**	3-KB-I18-Br	1	VEGFR	0.212986	0.535
**154**	3-KB-J18-B	0.1	VEGFR	0.097975	0.788
**155**	4-KB-A12-E	10000	VEGFR	0.276489	0.151
**156**	4-KB-A15-G	2500	VEGFR	0.316486	0.048
**157**	4-KB-A20-N	10000	VEGFR	0.334392	0
**158**	4-KB-B12-E	1000	VEGFR	0.271664	0.117
**159**	4-KB-B15-G	250	VEGFR	0.296887	0.077
**160**	4-KB-B20-N	1000	VEGFR	0.313381	0.022
**161**	4-KB-C15-G	25	VEGFR	0.073781	0.712
**162**	4-KB-D12-E	100	VEGFR	0.222274	0.208
**163**	4-KB-D15-G	2.5	VEGFR	0.218472	0.03
**164**	4-KB-D20-N	100	VEGFR	0.244709	0.742
**165**	4-KB-E12-El	10	VEGFR	0.334705	0.173
**166**	4-KB-E20-M	10	VEGFR	0.323111	0.022
**167**	4-KB-F12-El	1	VEGFR	0.164199	0.154
**168**	4-KB-F15-G	0.25	VEGFR	0.111674	0.719
**169**	4-KB-F20-M	1	VEGFR	0.304724	0.024
**170**	4-KB-L16-T	1	VEGFR	0.124101	0.37
**171**	4-KB-M16-T	10	VEGFR	0.266338	0.071
**172**	4-KB-N16-T	100	VEGFR	0.302076	0.019
**173**	4-KB-O16-T	1000	VEGFR	0.25341	0.27

**174**	4-KB-P16-T	10000	VEGFR	0.317074	0.032
**175**	2-KB-L10-I(	1	PI3K	0.157555	0.735
**176**	2-KB-M10-I	10	PI3K	0.281769	0.014
**177**	2-KB-N10-I	100	PI3K	0.247908	0.146
**178**	2-KB-O10-I	1000	PI3K	0.206215	0.321
**179**	2-KB-P10-I(	10000	PI3K	0.27034	0.088
**180**	3-KB-A16-P	2500	PI3K	0.11246	0.351
**181**	3-KB-C16-P	250	PI3K	0.152737	0.124
**182**	3-KB-D16-F	25	PI3K	0.313779	0.012
**183**	3-KB-E16-P	2.5	PI3K	0.188267	0.112
**184**	3-KB-F16-P	0.25	PI3K	0.232875	0.371
**185**	3-KB-F17-N	100000	PI3K	0.105095	0.984
**186**	3-KB-F19-D	500	PI3K	0.203837	0.867
**187**	3-KB-G17-N	10000	PI3K	0.176373	0.047
**188**	3-KB-G19-D	50	PI3K	0.212218	0.378
**189**	3-KB-H17-N	1000	PI3K	0.114794	0.428
**190**	3-KB-I17-M	100	PI3K	0.108796	0.449
**191**	3-KB-I19-D	5	PI3K	0.138011	0.249
**192**	3-KB-J17-N	10	PI3K	0.133814	0.292
**193**	3-KB-J19-D	0.5	PI3K	0.152356	0.322
**194**	3-KB-K19-D	0.05	PI3K	0.124326	0.346
**195**	3-KB-L8-Pic	1	PI3K	0.13092	0.236
**196**	3-KB-L21-T	0.1	PI3K	0.119989	0.982
**197**	3-KB-M8-Pi	10	PI3K	0.215671	0.133
**198**	3-KB-M21-T	1	PI3K	0.121034	0.343
**199**	3-KB-N8-Pi	100	PI3K	0.157923	0.706
**200**	3-KB-N21-T	10	PI3K	0.066325	0.811
**201**	3-KB-O8-Pi	1000	PI3K	0.20503	0.411
**202**	3-KB-O21-T	100	PI3K	0.147319	0.895
**203**	3-KB-P8-Pic	10000	PI3K	0.228673	0.239
**204**	3-KB-P21-T	1000	PI3K	0.164377	0.062
**205**	4-KB-A19-A	2500	PI3K	0.143787	0.482
**206**	4-KB-B19-A	250	PI3K	0.22178	0.143
**207**	4-KB-C19-A	25	PI3K	0.089938	0.732
**208**	4-KB-D19-A	2.5	PI3K	0.128216	0.339

**209**	4-KB-E19-A	0.25	PI3K	0.247468	0.464
**210**	4-KB-F14-N	1000	PI3K	0.253322	0.152
**211**	4-KB-G2-TG	2500	PI3K	0.134605	0.852
**212**	4-KB-G5-So	10000	PI3K	0.188747	0.248
**213**	4-KB-G14-N	100	PI3K	0.259146	0.103
**214**	4-KB-G20-E	10000	PI3K	0.264256	0.127
**215**	4-KB-H2-TG	250	PI3K	0.261522	0.668
**216**	4-KB-H5-So	1000	PI3K	0.117427	0.505
**217**	4-KB-H14-N	10	PI3K	0.272252	0.038
**218**	4-KB-H20-E	1000	PI3K	0.237907	0.281
**219**	4-KB-I2-TGI	25	PI3K	0.208783	0.607
**220**	4-KB-I5-Sor	100	PI3K	0.121005	0.911
**221**	4-KB-I14-N	1	PI3K	0.124933	0.542
**222**	4-KB-I20-Bu	100	PI3K	0.154248	0.96
**223**	4-KB-J2-TGI	2.5	PI3K	0.146648	0.939
**224**	4-KB-J5-Sor	10	PI3K	0.144941	0.18
**225**	4-KB-J20-Bu	10	PI3K	0.198676	0.367
**226**	4-KB-K2-TG	0.25	PI3K	0.08575	0.995
**227**	4-KB-K4-Da	0.1	PI3K	0.099975	0.777
**228**	4-KB-K5-So	1	PI3K	0.192747	0.204
**229**	4-KB-K14-N	0.1	PI3K	0.113261	0.373
**230**	4-KB-K20-B	1	PI3K	0.245864	0.379
**231**	4-KB-L4-Da	1	PI3K	0.219679	0.41
**232**	4-KB-L14-G	0.1	PI3K	0.089377	0.482
**233**	4-KB-L15-T	1	PI3K	0.311644	0.004
**234**	4-KB-L21-C	0.1	PI3K	0.252369	0.063
**235**	4-KB-M14-(	1	PI3K	0.091891	0.624
**236**	4-KB-M15-T	10	PI3K	0.093263	0.574
**237**	4-KB-M21-(	1	PI3K	0.184831	0.876
**238**	4-KB-N4-Da	10	PI3K	0.26172	0.124
**239**	4-KB-N14-G	10	PI3K	0.283103	0.033
**240**	4-KB-N15-T	100	PI3K	0.297415	0.318
**241**	4-KB-N21-C	10	PI3K	0.326379	0.03
**242**	4-KB-O4-Da	100	PI3K	0.259076	0.132
**243**	4-KB-O14-G	100	PI3K	0.275607	0.07

**244**	4-KB-O15-T	1000	PI3K	0.221976	0.299
**245**	4-KB-O21-C	100	PI3K	0.274782	0.105
**246**	4-KB-P4-Da	1000	PI3K	0.246082	0.162
**247**	4-KB-P14-G	1000	PI3K	0.272617	0.087
**248**	4-KB-P15-T	10000	PI3K	0.350338	0.003
**249**	4-KB-P21-C	1000	PI3K	0.257775	0.129
**250**	5-KB-A6-LY	2500	PI3K	0.238462	0.179
**251**	5-KB-A7-AN	1000	PI3K	0.328384	0.007
**252**	5-KB-A16-A	2500	PI3K	0.247297	0.08
**253**	5-KB-A17-P	10000	PI3K	0.131563	0.208
**254**	5-KB-B6-LY	250	PI3K	0.229678	0.247
**255**	5-KB-B7-AN	100	PI3K	0.241156	0.152
**256**	5-KB-B17-P	1000	PI3K	0.268434	0.197
**257**	5-KB-C6-LY	25	PI3K	0.250368	0.112
**258**	5-KB-C7-AN	10	PI3K	0.114786	0.934
**259**	5-KB-C16-A	250	PI3K	0.247407	0.142
**260**	5-KB-C17-P	100	PI3K	0.229663	0.335
**261**	5-KB-D6-LY	2.5	PI3K	0.202423	0.543
**262**	5-KB-D7-AN	1	PI3K	0.140133	0.118
**263**	5-KB-D16-A	25	PI3K	0.280127	0.028
**264**	5-KB-D17-P	10	PI3K	0.19287	0.041
**265**	5-KB-E6-LY	0.25	PI3K	0.101692	0.481
**266**	5-KB-E7-AN	0.1	PI3K	0.1803	0.112
**267**	5-KB-E16-A	2.5	PI3K	0.27165	0.056
**268**	5-KB-E17-P	1	PI3K	0.200997	0.032
**269**	5-KB-F11-G	10000	PI3K	0.268346	0.051
**270**	5-KB-F16-A	0.25	PI3K	0.125201	0.238
**271**	5-KB-G9-Se	10000	PI3K	0.24381	0.174
**272**	5-KB-G11-G	1000	PI3K	0.242543	0.086
**273**	5-KB-H9-Se	1000	PI3K	0.321566	0.005
**274**	5-KB-H11-G	100	PI3K	0.317634	0.001
**275**	5-KB-I9-Ser	100	PI3K	0.375031	0.003
**276**	5-KB-I11-G	10	PI3K	0.122587	0.5
**277**	5-KB-J9-Ser	10	PI3K	0.257329	0.065
**278**	5-KB-J11-G	1	PI3K	0.247221	0.001

**279**	5-KB-K9-Se	1	PI3K	0.169612	0.197
**280**	5-KB-L14-A	0.1	PI3K	0.195252	0.655
**281**	5-KB-L20-Z	1	PI3K	0.09963	0.635
**282**	5-KB-L23-O	0.1	PI3K	0.119039	0.768
**283**	5-KB-M14-/	1	PI3K	0.276669	0.008
**284**	5-KB-M20-Z	10	PI3K	0.257884	0.184
**285**	5-KB-M23-(	1	PI3K	0.068107	0.99
**286**	5-KB-N14-A	10	PI3K	0.259591	0.093
**287**	5-KB-N20-Z	100	PI3K	0.270403	0.156
**288**	5-KB-N23-C	10	PI3K	0.285438	0.06
**289**	5-KB-O14-A	100	PI3K	0.250509	0.154
**290**	5-KB-O20-Z	1000	PI3K	0.250951	0.162
**291**	5-KB-O23-C	100	PI3K	0.232474	0.396
**292**	5-KB-P14-A	1000	PI3K	0.245451	0.17
**293**	5-KB-P20-Z	10000	PI3K	0.257324	0.11
**294**	5-KB-P23-C	1000	PI3K	0.236053	0.273
**295**	6-KB-A8-TG	10000	PI3K	0.255792	0.153
**296**	6-KB-B8-TG	1000	PI3K	0.24624	0.386
**297**	6-KB-C8-TG	100	PI3K	0.247702	0.346
**298**	6-KB-D8-TG	10	PI3K	0.083852	0.728
**299**	6-KB-E8-TG	1	PI3K	0.09793	0.793
**300**	6-KB-L6-GD	1	PI3K	0.166765	0.746
**301**	6-KB-M6-GI	10	PI3K	0.248006	0.339
**302**	6-KB-N6-GI	100	PI3K	0.21082	0.418
**303**	6-KB-O6-GI	1000	PI3K	0.240252	0.248
**304**	6-KB-P6-GD	10000	PI3K	0.249091	0.215
**305**	1-KB-F11-A	10000	Topoisome	0.371899	0.017
**306**	1-KB-G11-A	1000	Topoisome	0.42965	0
**307**	1-KB-G20-E	1000	Topoisome	0.33067	0.05
**308**	1-KB-H11-A	100	Topoisome	0.454442	0
**309**	1-KB-H20-E	100	Topoisome	0.425779	0
**310**	1-KB-I11-Ar	10	Topoisome	0.407473	0.004
**311**	1-KB-I20-Ep	10	Topoisome	0.343344	0.03
**312**	1-KB-J11-Ar	1	Topoisome	0.134471	0.673
**313**	1-KB-J20-Ep	1	Topoisome	0.266359	0.374

**314**	1-KB-K11-S	1	Topoisome	0.414658	0.002
**315**	1-KB-K20-E	0.1	Topoisome	0.180977	0.853
**316**	1-KB-L11-Sl	10	Topoisome	0.401912	0
**317**	1-KB-L14-T	1	Topoisome	0.452915	0
**318**	1-KB-M11-S	100	Topoisome	0.347778	0.036
**319**	1-KB-M14-T	10	Topoisome	0.354729	0.015
**320**	1-KB-N14-T	100	Topoisome	0.429337	0.001
**321**	1-KB-O11-S	1000	Topoisome	0.326715	0.058
**322**	1-KB-O14-T	1000	Topoisome	0.378654	0.008
**323**	1-KB-P11-S	10000	Topoisome	0.325301	0.072
**324**	1-KB-P14-T	10000	Topoisome	0.351823	0.032
**325**	3-KB-A11-E	10000	Topoisome	0.384502	0.011
**326**	3-KB-B11-E	1000	Topoisome	0.561157	0
**327**	3-KB-C11-E	100	Topoisome	0.396104	0.006
**328**	3-KB-D11-E	10	Topoisome	0.157274	0.349
**329**	3-KB-E11-E	1	Topoisome	0.367528	0.004
**330**	3-KB-G9-Da	1000	Topoisome	0.20462	0.193
**331**	3-KB-G10-T	10000	Topoisome	0.334	0
**332**	3-KB-H9-Da	100	Topoisome	0.348935	0.057
**333**	3-KB-H10-T	1000	Topoisome	0.1771	0.19
**334**	3-KB-I9-Dau	10	Topoisome	0.331008	0
**335**	3-KB-I10-Te	100	Topoisome	0.294435	0.021
**336**	3-KB-J9-Dau	1	Topoisome	0.187641	0.2
**337**	3-KB-J10-Te	10	Topoisome	0.219924	0.055
**338**	3-KB-K7-Ida	0.1	Topoisome	0.248672	0.062
**339**	3-KB-K9-Da	0.1	Topoisome	0.245959	0.034
**340**	3-KB-K10-T	1	Topoisome	0.222016	0.066
**341**	3-KB-L6-Do	0.1	Topoisome	0.141392	0.423
**342**	3-KB-L7-Ida	1	Topoisome	0.305757	0.01
**343**	3-KB-L9-Val	0.5	Topoisome	0.382159	0.088
**344**	3-KB-L10-M	0.1	Topoisome	0.258468	0.06
**345**	3-KB-L16-P	1	Topoisome	0.186507	0.133
**346**	3-KB-M6-Do	1	Topoisome	0.38956	0.004
**347**	3-KB-M7-Id	10	Topoisome	0.218711	0.088
**348**	3-KB-M9-Va	5	Topoisome	0.21189	0.092

**349**	3-KB-M10-I	1	Topoisome	0.381611	0.079
**350**	3-KB-M16-I	10	Topoisome	0.384488	0.053
**351**	3-KB-N6-Dc	10	Topoisome	0.439751	0.016
**352**	3-KB-N9-Va	50	Topoisome	0.448004	0
**353**	3-KB-N10-M	10	Topoisome	0.298258	0.149
**354**	3-KB-N16-P	100	Topoisome	0.529105	0
**355**	3-KB-O6-Dc	100	Topoisome	0.42119	0
**356**	3-KB-O7-Ida	100	Topoisome	0.305306	0.119
**357**	3-KB-O9-Va	500	Topoisome	0.439829	0
**358**	3-KB-O10-M	100	Topoisome	0.309774	0.089
**359**	3-KB-O16-P	1000	Topoisome	0.387678	0.017
**360**	3-KB-P6-Dc	1000	Topoisome	0.268006	0.298
**361**	3-KB-P7-Ida	1000	Topoisome	0.300563	0.104
**362**	3-KB-P9-Va	5000	Topoisome	0.266049	0.273
**363**	3-KB-P10-M	1000	Topoisome	0.345371	0.042
**364**	3-KB-P16-P	10000	Topoisome	0.438659	0
**365**	1-KB-A10-V	10000	Mitotic	0.563393	0
**366**	1-KB-A13-Ix	1000	Mitotic	0.561852	0
**367**	1-KB-A18-P	1000	Mitotic	0.543823	0
**368**	1-KB-B10-V	1000	Mitotic	0.551275	0
**369**	1-KB-B13-Ix	100	Mitotic	0.509672	0
**370**	1-KB-B18-P	100	Mitotic	0.553581	0
**371**	1-KB-C10-V	100	Mitotic	0.554988	0
**372**	1-KB-C13-Ix	10	Mitotic	0.191249	0.59
**373**	1-KB-C18-P	10	Mitotic	0.544815	0
**374**	1-KB-D10-V	10	Mitotic	0.609157	0
**375**	1-KB-D13-Ix	1	Mitotic	0.095644	0.785
**376**	1-KB-D18-P	1	Mitotic	0.569946	0
**377**	1-KB-E10-V	1	Mitotic	0.179448	0.779
**378**	1-KB-E13-Ix	0.1	Mitotic	0.096861	0.75
**379**	1-KB-E18-P	0.1	Mitotic	0.599059	0
**380**	1-KB-F13-V	1000	Mitotic	0.561346	0
**381**	1-KB-G13-V	100	Mitotic	0.501694	0
**382**	1-KB-G15-E	1000	Mitotic	0.530364	0
**383**	1-KB-H13-V	10	Mitotic	0.463953	0.001

**384**	1-KB-H15-E	100	Mitotic	0.51626	0
**385**	1-KB-I13-Vi	1	Mitotic	0.516132	0
**386**	1-KB-I15-Er	10	Mitotic	0.544739	0
**387**	1-KB-J13-Vi	0.1	Mitotic	0.286135	0.129
**388**	1-KB-J15-Er	1	Mitotic	0.563586	0
**389**	1-KB-K7-Vir	0.1	Mitotic	0.064819	0.922
**390**	1-KB-K15-E	0.1	Mitotic	0.541574	0
**391**	1-KB-L7-Vir	1	Mitotic	0.189892	0.577
**392**	1-KB-L20-V	0.1	Mitotic	0.507563	0
**393**	1-KB-M7-Vi	10	Mitotic	0.54035	0
**394**	1-KB-M20-\	1	Mitotic	0.466739	0
**395**	1-KB-N20-\	10	Mitotic	0.580308	0
**396**	1-KB-O7-Vi	100	Mitotic	0.573782	0
**397**	1-KB-O20-\	100	Mitotic	0.551246	0
**398**	1-KB-P7-Vir	1000	Mitotic	0.52159	0
**399**	1-KB-P20-V	1000	Mitotic	0.561975	0
**400**	3-KB-A7-Dc	1000	Mitotic	0.567184	0
**401**	3-KB-B7-Dc	100	Mitotic	0.518495	0
**402**	3-KB-C7-Dc	10	Mitotic	0.459585	0
**403**	3-KB-D7-Dc	1	Mitotic	0.54609	0
**404**	3-KB-E7-Do	0.1	Mitotic	0.045973	1
**405**	6-KB-L19-A	1	Mitotic	0.195754	0.97
**406**	6-KB-M19-/	10	Mitotic	0.092932	0.984
**407**	6-KB-N19-A	100	Mitotic	0.069959	1
**408**	6-KB-O19-A	1000	Mitotic	0.451938	0.001
**409**	6-KB-P19-A	10000	Mitotic	0.447493	0.003
**410**	2-KB-A12-T	250	MEK1/2	0.558788	0
**411**	2-KB-B12-T	25	MEK1/2	0.516031	0.001
**412**	2-KB-D12-T	2.5	MEK1/2	0.583866	0
**413**	2-KB-E12-Ti	0.25	MEK1/2	0.616531	0
**414**	2-KB-F12-Ti	2.5000000	MEK1/2	0.26659	0.59
**415**	2-KB-F14-C	1000	MEK1/2	0.571321	0.001
**416**	2-KB-G14-C	100	MEK1/2	0.553569	0
**417**	2-KB-H14-C	10	MEK1/2	0.577912	0
**418**	2-KB-I14-Cc	1	MEK1/2	0.494765	0.005

**419**	2-KB-K14-C	0.1	MEK1/2	0.142993	0.807
**420**	2-KB-L20-S	1	MEK1/2	0.067682	1
**421**	2-KB-M20-S	10	MEK1/2	0.580904	0
**422**	2-KB-N20-S	100	MEK1/2	0.57634	0
**423**	2-KB-O20-S	1000	MEK1/2	0.560118	0
**424**	2-KB-P20-S	10000	MEK1/2	0.532049	0.001
**425**	4-KB-A10-B	1000	MEK1/2	0.487947	0.001
**426**	4-KB-A13-P	1000	MEK1/2	0.528866	0
**427**	4-KB-B10-B	100	MEK1/2	0.501302	0.001
**428**	4-KB-B13-P	100	MEK1/2	0.50721	0.001
**429**	4-KB-C10-B	10	MEK1/2	0.521267	0
**430**	4-KB-C13-P	10	MEK1/2	0.498182	0.003
**431**	4-KB-D10-E	1	MEK1/2	0.185401	0.517
**432**	4-KB-D13-F	1	MEK1/2	0.597178	0.002
**433**	4-KB-E10-B	0.1	MEK1/2	0.146486	0.882
**434**	4-KB-E13-P	0.1	MEK1/2	0.105272	0.835
**435**	4-KB-L19-G	0.25	MEK1/2	0.485828	0.01
**436**	4-KB-M19-(	2.5	MEK1/2	0.219583	0.447
**437**	4-KB-N19-G	25	MEK1/2	0.10141	0.863
**438**	4-KB-O19-G	250	MEK1/2	0.43962	0.016
**439**	4-KB-P19-G	2500	MEK1/2	0.480755	0.004
**440**	1-KB-L2-Ola	1	PARP	0.247069	0.604
**441**	1-KB-L6-Ru	1	PARP	0.494057	0.011
**442**	1-KB-M2-Ol	10	PARP	0.533234	0.042
**443**	1-KB-M6-Ru	10	PARP	0.460643	0.012
**444**	1-KB-N2-Ol	100	PARP	0.479444	0.169
**445**	1-KB-N6-Ru	100	PARP	0.44585	0.033
**446**	1-KB-O2-Ol	1000	PARP	0.494522	0.008
**447**	1-KB-O6-Ru	1000	PARP	0.451237	0.024
**448**	1-KB-P2-Ola	10000	PARP	0.515588	0.004
**449**	1-KB-P6-Ru	10000	PARP	0.442179	0.037
**450**	7-KB-A3-Tal	1000	PARP	0.512799	0.003
**451**	7-KB-B2-Ve	10000	PARP	0.6107	0
**452**	7-KB-B3-Tal	100	PARP	0.691355	0
**453**	7-KB-C2-Ve	1000	PARP	0.520306	0.018

**454**	7-KB-C3-Tal	10	PARP	0.405401	0.077
**455**	7-KB-D2-Ve	100	PARP	0.51875	0.029
**456**	7-KB-D3-Ta	1	PARP	0.72356	0
**457**	7-KB-E2-Vel	10	PARP	0.133847	1
**458**	7-KB-E3-Tal	0.1	PARP	0.671067	0.002
**459**	7-KB-F2-Vel	1	PARP	0.608148	0
**460**	7-KB-G2-Ni	10000	PARP	0.60415	0
**461**	7-KB-H2-Ni	1000	PARP	0.619276	0
**462**	7-KB-I2-Nir	100	PARP	0.619298	0
**463**	7-KB-J2-Nir	10	PARP	0.601307	0
**464**	7-KB-K2-Ni	1	PARP	0.542424	0.001
**465**	3-KB-A19-D	1000	CDK	0.474547	0
**466**	3-KB-B19-D	100	CDK	0.486828	0
**467**	3-KB-B23-A	2500	CDK	0.3662	0.013
**468**	3-KB-C19-D	10	CDK	0.379539	0.009
**469**	3-KB-C23-A	250	CDK	0.403284	0.005
**470**	3-KB-D19-D	1	CDK	0.412982	0.004
**471**	3-KB-D23-A	25	CDK	0.503523	0
**472**	3-KB-E19-D	0.1	CDK	0.21424	0.337
**473**	3-KB-E23-A	2.5	CDK	0.374979	0.026
**474**	3-KB-F23-A	0.25	CDK	0.156439	0.807
**475**	3-KB-K17-P	1	CDK	0.157913	0.46
**476**	3-KB-L19-R	1	CDK	0.116779	0.577
**477**	3-KB-M17-I	10	CDK	0.333577	0.071
**478**	3-KB-M19-I	10	CDK	0.115644	0.713
**479**	3-KB-N17-F	100	CDK	0.476085	0.001
**480**	3-KB-N19-F	100	CDK	0.131175	0.678
**481**	3-KB-O17-F	1000	CDK	0.435332	0
**482**	3-KB-O19-F	1000	CDK	0.539162	0
**483**	3-KB-P17-P	10000	CDK	0.403372	0.007
**484**	3-KB-P19-R	10000	CDK	0.478318	0
**485**	4-KB-A4-SN	10000	CDK	0.438523	0
**486**	4-KB-A8-Mi	10000	CDK	0.368374	0.016
**487**	4-KB-B4-SN	1000	CDK	0.382228	0.008
**488**	4-KB-B8-Mi	1000	CDK	0.377019	0.01

**489**	4-KB-C4-SN	100	CDK	0.358705	0.021
**490**	4-KB-C8-Mi	100	CDK	0.268307	0.221
**491**	4-KB-D4-SN	10	CDK	0.369581	0.173
**492**	4-KB-D8-Mi	10	CDK	0.132784	0.61
**493**	4-KB-E4-SN	1	CDK	0.203706	0.779
**494**	4-KB-E8-Mi	1	CDK	0.145748	0.788
**495**	4-KB-F4-Sel	10000	CDK	0.375723	0.011
**496**	4-KB-F22-A	10000	CDK	0.357871	0.027
**497**	4-KB-G4-Se	1000	CDK	0.255201	0.701
**498**	4-KB-G22-A	1000	CDK	0.348696	0.031
**499**	4-KB-H4-Se	100	CDK	0.091362	0.808
**500**	4-KB-H22-A	100	CDK	0.447638	0.001
**501**	4-KB-I4-Seli	10	CDK	0.212094	0.851
**502**	4-KB-I22-Al	10	CDK	0.495187	0
**503**	4-KB-J4-Sel	1	CDK	0.092812	0.83
**504**	4-KB-J22-Al	1	CDK	0.258127	0.142
**505**	5-KB-A19-A	10000	CDK	0.359041	0.023
**506**	5-KB-B19-A	1000	CDK	0.347203	0.035
**507**	5-KB-C19-A	100	CDK	0.338753	0.04
**508**	5-KB-D19-A	10	CDK	0.296663	0.211
**509**	5-KB-E19-A	1	CDK	0.105448	0.951
**510**	5-KB-K17-A	1	CDK	0.228079	0.409
**511**	5-KB-M17-A	10	CDK	0.285017	0.193
**512**	5-KB-N17-A	100	CDK	0.418898	0.007
**513**	5-KB-O17-A	1000	CDK	0.354115	0.025
**514**	5-KB-P17-A	10000	CDK	0.369857	0.026
**515**	6-KB-A17-S	1000	CDK	0.376967	0.006
**516**	6-KB-B17-S	100	CDK	0.526856	0
**517**	6-KB-C17-S	10	CDK	0.220852	0.277
**518**	6-KB-D17-S	1	CDK	0.146047	0.398
**519**	6-KB-E17-S	0.1	CDK	0.319247	0.169
**520**	6-KB-L15-T	1	CDK	0.094736	0.732
**521**	6-KB-M15-	10	CDK	0.102922	0.71
**522**	6-KB-N15-T	100	CDK	0.436647	0.003
**523**	6-KB-O15-T	1000	CDK	0.324023	0.075

**524**	6-KB-P15-T	10000	CDK	0.321456	0.102
**525**	7-KB-A21-d	10000	BET	0.575465	0
**526**	7-KB-A22-P	30000	BET	0.58938	0
**527**	7-KB-B21-d	1000	BET	0.57355	0
**528**	7-KB-B22-P	3000	BET	0.57743	0
**529**	7-KB-C21-d	100	BET	0.626886	0
**530**	7-KB-C22-P	300	BET	0.62141	0
**531**	7-KB-D21-c	10	BET	0.563538	0
**532**	7-KB-D22-F	30	BET	0.553067	0
**533**	7-KB-E21-d	1	BET	0.663061	0
**534**	7-KB-E22-P	3	BET	0.739656	0
**535**	7-KB-G10-E	10000	BET	0.584006	0
**536**	7-KB-G15-I-	10000	BET	0.671635	0
**537**	7-KB-H10-E	1000	BET	0.597483	0
**538**	7-KB-H15-I-	1000	BET	0.649375	0
**539**	7-KB-I10-Bi	100	BET	0.533515	0
**540**	7-KB-I15-I-I	100	BET	0.607778	0
**541**	7-KB-J10-Bi	10	BET	0.649712	0
**542**	7-KB-J15-I-I	10	BET	0.122033	0.67
**543**	7-KB-K10-B	1	BET	0.29039	0.25
**544**	7-KB-K13-N	1	BET	0.668562	0
**545**	7-KB-K15-I-	1	BET	0.104	0.916
**546**	7-KB-L12-N	1	BET	0.148214	0.549
**547**	7-KB-L13-N	10	BET	0.607847	0
**548**	7-KB-L20-J(	1	BET	0.230374	0.322
**549**	7-KB-L23-A	0.03	BET	0.255622	0.427
**550**	7-KB-M12-I	10	BET	0.133434	0.805
**551**	7-KB-M13-I	100	BET	0.609668	0
**552**	7-KB-M20-J	10	BET	0.579692	0
**553**	7-KB-M23-/	0.3	BET	0.205444	0.51
**554**	7-KB-N12-N	100	BET	0.742263	0
**555**	7-KB-N13-N	1000	BET	0.655169	0
**556**	7-KB-N20-J	100	BET	0.776306	0
**557**	7-KB-N23-A	3	BET	0.185482	0.352
**558**	7-KB-O12-N	1000	BET	0.534165	0

**559**	7-KB-O20-J	1000	BET	0.691148	0
**560**	7-KB-O23-A	30	BET	0.612253	0
**561**	7-KB-P12-N	10000	BET	0.587147	0
**562**	7-KB-P13-N	10000	BET	0.606553	0
**563**	7-KB-P20-J	10000	BET	0.611467	0
**564**	7-KB-P23-A	300	BET	0.6416	0
**565**	8-KB-K22-C	1	BET	0.515396	0.001
**566**	8-KB-L22-C	10	BET	0.369105	0.436
**567**	8-KB-M22-(	100	BET	0.262619	0.302
**568**	8-KB-N22-C	1000	BET	0.120099	0.751
**569**	8-KB-O22-C	10000	BET	0.123472	0.721
**570**	1-KB-A3-Vo	10000	HDAC	0.337673	0.006
**571**	1-KB-B3-Vo	1000	HDAC	0.438661	0
**572**	1-KB-C3-Vo	100	HDAC	0.338359	0.004
**573**	1-KB-D3-Vc	10	HDAC	0.371093	0.082
**574**	1-KB-E3-Vo	1	HDAC	0.084332	0.819
**575**	1-KB-L12-R	0.1	HDAC	0.049182	0.88
**576**	1-KB-M12-I	1	HDAC	0.478701	0
**577**	1-KB-N12-R	10	HDAC	0.322098	0.015
**578**	1-KB-O12-R	100	HDAC	0.318343	0.016
**579**	1-KB-P12-R	1000	HDAC	0.328485	0.012
**580**	3-KB-A4-Pa	1000	HDAC	0.301271	0.049
**581**	3-KB-B4-Pa	100	HDAC	0.352908	0.003
**582**	3-KB-C4-Pa	10	HDAC	0.152486	0.592
**583**	3-KB-D4-Pa	1	HDAC	0.052794	0.941
**584**	3-KB-E4-Pa	0.1	HDAC	0.072924	0.839
**585**	3-KB-F7-Qu	1000	HDAC	0.299565	0.031
**586**	3-KB-G7-Qu	100	HDAC	0.074623	0.798
**587**	3-KB-G12-V	1000000	HDAC	0.058844	0.927
**588**	3-KB-H7-Qu	10	HDAC	0.105609	0.821
**589**	3-KB-H12-V	100000	HDAC	0.190624	0.25
**590**	3-KB-I7-Qui	1	HDAC	0.134	0.392
**591**	3-KB-I12-Va	10000	HDAC	0.112553	0.395
**592**	3-KB-J7-Qu	0.1	HDAC	0.032711	0.985
**593**	3-KB-J12-Va	1000	HDAC	0.078848	0.748

**594**	3-KB-K3-Be	1	HDAC	0.168672	0.372
**595**	3-KB-K12-V	100	HDAC	0.147339	0.224
**596**	3-KB-L3-Bel	10	HDAC	0.196736	0.286
**597**	3-KB-M3-Be	100	HDAC	0.29624	0.048
**598**	3-KB-N3-Be	1000	HDAC	0.310871	0.02
**599**	3-KB-O3-Be	10000	HDAC	0.301903	0.034
**600**	7-KB-A5-Mc	10000	HDAC	0.375376	0.001
**601**	7-KB-A7-CL	10000	HDAC	0.383725	0
**602**	7-KB-A9-Gi	1000	HDAC	0.412355	0
**603**	7-KB-A12-R	10000	HDAC	0.4114	0
**604**	7-KB-B5-Mc	1000	HDAC	0.566987	0
**605**	7-KB-B7-CL	1000	HDAC	0.378538	0
**606**	7-KB-B12-R	1000	HDAC	0.460089	0
**607**	7-KB-C5-Mc	100	HDAC	0.396361	0
**608**	7-KB-C7-CL	100	HDAC	0.397501	0
**609**	7-KB-C9-Gi	100	HDAC	0.520159	0
**610**	7-KB-D7-CL	10	HDAC	0.543554	0
**611**	7-KB-D9-Gi	10	HDAC	0.371253	0.001
**612**	7-KB-D12-R	100	HDAC	0.216176	0.217
**613**	7-KB-E5-Mc	10	HDAC	0.458867	0.001
**614**	7-KB-E7-CU	1	HDAC	0.559686	0
**615**	7-KB-E9-Giv	1	HDAC	0.297675	0.113
**616**	7-KB-E12-R	10	HDAC	0.459341	0
**617**	7-KB-F5-Mc	1	HDAC	0.119905	0.652
**618**	7-KB-F7-Res	10000	HDAC	0.395737	0.001
**619**	7-KB-F9-Giv	0.1	HDAC	0.521665	0
**620**	7-KB-F12-R	1	HDAC	0.387828	0.003
**621**	7-KB-F19-P	10000	HDAC	0.388666	0
**622**	7-KB-G7-Re	1000	HDAC	0.514053	0
**623**	7-KB-G19-P	1000	HDAC	0.468874	0
**624**	7-KB-H7-Re	100	HDAC	0.18606	0.045
**625**	7-KB-I7-Res	10	HDAC	0.592548	0
**626**	7-KB-I19-P(	100	HDAC	0.455496	0
**627**	7-KB-J7-Res	1	HDAC	0.532782	0
**628**	7-KB-J19-P	10	HDAC	0.09942	0.792

**629**	7-KB-K4-En	1	HDAC	0.514197	0
**630**	7-KB-K11-A	1	HDAC	0.289688	0.001
**631**	7-KB-K18-T	1	HDAC	0.103358	0.381
**632**	7-KB-K19-P	1	HDAC	0.456575	0
**633**	7-KB-L2-Ta	0.1	HDAC	0.388731	0
**634**	7-KB-L4-En	10	HDAC	0.490904	0
**635**	7-KB-L5-Pra	1	HDAC	0.539574	0
**636**	7-KB-L8-Ab	1	HDAC	0.449284	0
**637**	7-KB-L10-T	1	HDAC	0.069483	0.844
**638**	7-KB-L11-A	10	HDAC	0.573846	0
**639**	7-KB-L14-T	1	HDAC	0.584202	0
**640**	7-KB-L16-R	1	HDAC	0.06752	0.815
**641**	7-KB-L18-T	10	HDAC	0.392921	0.002
**642**	7-KB-M2-Ta	1	HDAC	0.442719	0
**643**	7-KB-M5-Pr	10	HDAC	0.476695	0
**644**	7-KB-M8-Al	10	HDAC	0.552216	0
**645**	7-KB-M10-	10	HDAC	0.278174	0.227
**646**	7-KB-M11-	100	HDAC	0.503695	0
**647**	7-KB-M14-	10	HDAC	0.4447	0
**648**	7-KB-M16-I	10	HDAC	0.122839	0.64
**649**	7-KB-M18-	100	HDAC	0.464053	0
**650**	7-KB-N2-Ta	10	HDAC	0.429497	0
**651**	7-KB-N4-En	100	HDAC	0.469596	0
**652**	7-KB-N5-Pr	100	HDAC	0.400331	0
**653**	7-KB-N8-Ab	100	HDAC	0.499468	0
**654**	7-KB-N10-T	100	HDAC	0.381246	0
**655**	7-KB-N14-T	100	HDAC	0.093948	0.563
**656**	7-KB-N16-R	100	HDAC	0.057999	0.875
**657**	7-KB-N18-T	1000	HDAC	0.447967	0
**658**	7-KB-O2-Ta	100	HDAC	0.423649	0
**659**	7-KB-O4-En	1000	HDAC	0.427491	0
**660**	7-KB-O5-Pr	1000	HDAC	0.450102	0
**661**	7-KB-O8-Ab	1000	HDAC	0.400967	0
**662**	7-KB-O10-T	1000	HDAC	0.556284	0
**663**	7-KB-O11-A	1000	HDAC	0.423119	0

**664**	7-KB-O14-T	1000	HDAC	0.074626	0.983
**665**	7-KB-O16-F	1000	HDAC	0.365337	0.003
**666**	7-KB-P2-Ta	1000	HDAC	0.325086	0.003
**667**	7-KB-P4-En	10000	HDAC	0.435379	0
**668**	7-KB-P5-Pr	10000	HDAC	0.394077	0.001
**669**	7-KB-P8-Ab	10000	HDAC	0.392851	0.001
**670**	7-KB-P10-T	10000	HDAC	0.441805	0
**671**	7-KB-P11-A	10000	HDAC	0.384991	0
**672**	7-KB-P14-T	10000	HDAC	0.274687	0.178
**673**	7-KB-P16-R	10000	HDAC	0.423917	0
**674**	7-KB-P18-T	10000	HDAC	0.407317	0
**0**	2-KW-A16-	10000	EGFR	0.355229	0.138
**1**	2-KW-A19-	10000	EGFR	0.369579	0.011
**2**	2-KW-B19-	1000	EGFR	0.399553	0.016
**3**	2-KW-C16-	1000	EGFR	0.363885	0.005
**4**	2-KW-C19-	100	EGFR	0.238141	0.289
**5**	2-KW-D16-	100	EGFR	0.302386	0.116
**6**	2-KW-D19-	10	EGFR	0.064102	0.903
**7**	2-KW-E16-	10	EGFR	0.107867	0.67
**8**	2-KW-E19-	1	EGFR	0.258452	0.516
**9**	2-KW-F16-	1	EGFR	0.358926	0.022
**10**	2-KW-K11-	0.1	EGFR	0.256915	0.133
**11**	2-KW-L11-	1	EGFR	0.278049	0.1
**12**	2-KW-L16-	0.25	EGFR	0.341359	0.018
**13**	2-KW-L19-	0.1	EGFR	0.330671	0.174
**14**	2-KW-M11-	10	EGFR	0.351723	0.032
**15**	2-KW-M16-	2.5	EGFR	0.358884	0.003
**16**	2-KW-M19-	1	EGFR	0.422177	0
**17**	2-KW-N16-	25	EGFR	0.334246	0.012
**18**	2-KW-N19-	10	EGFR	0.316792	0.1
**19**	2-KW-O11-	100	EGFR	0.347614	0.088
**20**	2-KW-O16-	250	EGFR	0.347698	0.046
**21**	2-KW-O19-	100	EGFR	0.326231	0.096
**22**	2-KW-P11-	1000	EGFR	0.386103	0.074
**23**	2-KW-P16-	2500	EGFR	0.360848	0.09

**24**	2-KW-P19-I	1000	EGFR	0.376898	0.032
**25**	3-KW-F21-I	10000	EGFR	0.341949	0.158
**26**	3-KW-G20-	1000	EGFR	0.359844	0.046
**27**	3-KW-G21-	1000	EGFR	0.417316	0
**28**	3-KW-H20-	100	EGFR	0.392089	0.001
**29**	3-KW-H21-	100	EGFR	0.151109	0.726
**30**	3-KW-I20-N	10	EGFR	0.333334	0.043
**31**	3-KW-I21-F	10	EGFR	0.375564	0.015
**32**	3-KW-J20-N	1	EGFR	0.049016	0.926
**33**	3-KW-J21-F	1	EGFR	0.220576	0.383
**34**	3-KW-K4-C	1	EGFR	0.314503	0.156
**35**	3-KW-K18-I	0.1	EGFR	0.37474	0.006
**36**	3-KW-K20-I	0.1	EGFR	0.413908	0.001
**37**	3-KW-L4-C	10	EGFR	0.314498	0.133
**38**	3-KW-L18-I	1	EGFR	0.344704	0.02
**39**	3-KW-M18-	10	EGFR	0.348029	0.056
**40**	3-KW-N4-C	100	EGFR	0.349053	0.04
**41**	3-KW-N18-	100	EGFR	0.400577	0.006
**42**	3-KW-O4-C	1000	EGFR	0.348981	0.094
**43**	3-KW-P4-C	10000	EGFR	0.340692	0.248
**44**	3-KW-P18-I	1000	EGFR	0.353984	0.013
**45**	4-KW-F13-S	1000	EGFR	0.335215	0.047
**46**	4-KW-G13-	100	EGFR	0.362929	0.032
**47**	4-KW-G16-	10000	EGFR	0.350226	0.172
**48**	4-KW-H13-	10	EGFR	0.117346	0.718
**49**	4-KW-H16-	1000	EGFR	0.15668	0.524
**50**	4-KW-I13-S	1	EGFR	0.439153	0.001
**51**	4-KW-I16-V	100	EGFR	0.155715	0.684
**52**	4-KW-J13-S	0.1	EGFR	0.358992	0.078
**53**	4-KW-J16-V	10	EGFR	0.039731	0.98
**54**	4-KW-K7-Ic	1	EGFR	0.201039	0.592
**55**	4-KW-K13-T	0.1	EGFR	0.264641	0.311
**56**	4-KW-K16-Y	1	EGFR	0.362295	0.046
**57**	4-KW-L7-Ic	10	EGFR	0.183407	0.57
**58**	4-KW-L13-T	1	EGFR	0.223279	0.465

**59**	4-KW-M7-I	100	EGFR	0.374035	0.017
**60**	4-KW-M13-	10	EGFR	0.399711	0.006
**61**	4-KW-N13-	100	EGFR	0.413611	0.003
**62**	4-KW-O7-I	1000	EGFR	0.220628	0.728
**63**	4-KW-P7-I	10000	EGFR	0.381218	0.02
**64**	4-KW-P13-	1000	EGFR	0.29019	0.323
**65**	5-KW-F4-P	1000	EGFR	0.358346	0.039
**66**	5-KW-F7-A	1000	EGFR	0.304621	0.094
**67**	5-KW-G4-P	100	EGFR	0.347434	0.063
**68**	5-KW-G7-A	100	EGFR	0.350801	0.009
**69**	5-KW-H4-P	10	EGFR	0.352861	0.022
**70**	5-KW-H7-A	10	EGFR	0.173199	0.652
**71**	5-KW-I4-P	1	EGFR	0.238125	0.645
**72**	5-KW-I7-A	1	EGFR	0.285505	0.16
**73**	5-KW-J4-P	0.1	EGFR	0.352581	0.015
**74**	5-KW-J7-A	0.1	EGFR	0.374716	0.035
**75**	5-KW-K7-O	0.1	EGFR	0.311746	0.074
**76**	5-KW-L7-O	1	EGFR	0.366276	0.018
**77**	5-KW-M7-O	10	EGFR	0.363363	0.041
**78**	5-KW-O7-O	100	EGFR	0.353616	0.028
**79**	5-KW-P7-O	1000	EGFR	0.335748	0.053
**80**	2-KW-A15-	2500	VEGFR	0.297825	0.289
**81**	2-KW-A17-	10000	VEGFR	0.267039	0.655
**82**	2-KW-A20-	10000	VEGFR	0.298541	0.564
**83**	2-KW-B15-	250	VEGFR	0.532232	0
**84**	2-KW-B17-	1000	VEGFR	0.531046	0
**85**	2-KW-B20-	1000	VEGFR	0.32611	0.028
**86**	2-KW-C15-	25	VEGFR	0.309139	0.016
**87**	2-KW-C17-	100	VEGFR	0.521208	0
**88**	2-KW-D15-	2.5	VEGFR	0.296196	0
**89**	2-KW-D17-	10	VEGFR	0.450056	0
**90**	2-KW-D20-	100	VEGFR	0.35169	0.001
**91**	2-KW-E17-	1	VEGFR	0.266348	0.002
**92**	2-KW-E20-	10	VEGFR	0.065486	1
**93**	2-KW-F13-	10000	VEGFR	0.201294	0.868

**94**	2-KW-F15-I	0.25	VEGFR	0.070936	0.924
**95**	2-KW-F19-I	10000	VEGFR	0.184926	0.955
**96**	2-KW-F20-T	1	VEGFR	0.341172	0.054
**97**	2-KW-F21-V	10000	VEGFR	0.240604	0.221
**98**	2-KW-G10-	10000	VEGFR	0.53636	0
**99**	2-KW-G13-	1000	VEGFR	0.30993	0.087
**100**	2-KW-G19-	1000	VEGFR	0.298155	0.163
**101**	2-KW-G21-	1000	VEGFR	0.389611	0.006
**102**	2-KW-H10-	1000	VEGFR	0.461979	0
**103**	2-KW-H13-	100	VEGFR	0.500259	0
**104**	2-KW-H21-	100	VEGFR	0.252253	0.002
**105**	2-KW-I10-A	100	VEGFR	0.384122	0
**106**	2-KW-I13-A	10	VEGFR	0.378481	0
**107**	2-KW-I19-F	100	VEGFR	0.130537	0.917
**108**	2-KW-I21-V	10	VEGFR	0.096605	0.996
**109**	2-KW-J10-A	10	VEGFR	0.383946	0
**110**	2-KW-J13-A	1	VEGFR	0.322307	0.051
**111**	2-KW-J19-F	10	VEGFR	0.24074	0.477
**112**	2-KW-J21-V	1	VEGFR	0.305174	0.148
**113**	2-KW-K10-A	1	VEGFR	0.211632	0.641
**114**	2-KW-K13-V	0.1	VEGFR	0.318779	0.107
**115**	2-KW-K17-I	1	VEGFR	0.531439	0
**116**	2-KW-K19-I	1	VEGFR	0.41288	0.001
**117**	2-KW-L12-S	0.1	VEGFR	0.351452	0.005
**118**	2-KW-L13-V	1	VEGFR	0.093686	0.806
**119**	2-KW-L21-C	0.1	VEGFR	0.14347	0.706
**120**	2-KW-M12-	1	VEGFR	0.251198	0.538
**121**	2-KW-M13-	10	VEGFR	0.459942	0
**122**	2-KW-M17-	10	VEGFR	0.322742	0.017
**123**	2-KW-M21-	1	VEGFR	0.204669	0.756
**124**	2-KW-N12-	10	VEGFR	0.231929	0.68
**125**	2-KW-N13-	100	VEGFR	0.177036	0.837
**126**	2-KW-N17-	100	VEGFR	0.340767	0.007
**127**	2-KW-N21-	10	VEGFR	0.428025	0
**128**	2-KW-O12-	100	VEGFR	0.194531	0.96

**129**	2-KW-O17-	1000	VEGFR	0.294146	0.091
**130**	2-KW-O21-	100	VEGFR	0.169232	0.928
**131**	2-KW-P12-!	1000	VEGFR	0.331357	0.098
**132**	2-KW-P13-'	1000	VEGFR	0.180723	0.98
**133**	2-KW-P17-l	10000	VEGFR	0.324546	0.375
**134**	2-KW-P21-(	1000	VEGFR	0.314455	0.618
**135**	3-KW-A3-Ca	1000	VEGFR	0.33698	0.113
**136**	3-KW-A6-Fc	1000	VEGFR	0.219662	0.818
**137**	3-KW-A18-l	1000	VEGFR	0.399875	0
**138**	3-KW-B3-Ca	100	VEGFR	0.43166	0.001
**139**	3-KW-B6-Fc	100	VEGFR	0.384141	0.003
**140**	3-KW-B18-l	100	VEGFR	0.192006	0.01
**141**	3-KW-C3-Ca	10	VEGFR	0.146363	0.122
**142**	3-KW-C6-Fc	10	VEGFR	0.217199	0.777
**143**	3-KW-C18-l	10	VEGFR	0.152652	0.914
**144**	3-KW-D3-C	1	VEGFR	0.159809	0.938
**145**	3-KW-D6-Fc	1	VEGFR	0.189963	0.079
**146**	3-KW-D18-	1	VEGFR	0.328507	0.039
**147**	3-KW-E3-Ca	0.1	VEGFR	0.392393	0.001
**148**	3-KW-E6-Fc	0.1	VEGFR	0.362697	0
**149**	3-KW-E18-l	0.1	VEGFR	0.225921	0
**150**	3-KW-F18-l	1000	VEGFR	0.306775	0
**151**	3-KW-G18-	100	VEGFR	0.34015	0.01
**152**	3-KW-H18-	10	VEGFR	0.337175	0.072
**153**	3-KW-I18-E	1	VEGFR	0.438369	0.001
**154**	3-KW-J18-E	0.1	VEGFR	0.367109	0.014
**155**	4-KW-A12-l	10000	VEGFR	0.291714	0.764
**156**	4-KW-A15-(	2500	VEGFR	0.279719	0.616
**157**	4-KW-A20-l	10000	VEGFR	0.24292	0.917
**158**	4-KW-B12-l	1000	VEGFR	0.355843	0.111
**159**	4-KW-B15-(	250	VEGFR	0.339088	0
**160**	4-KW-B20-l	1000	VEGFR	0.071939	0.947
**161**	4-KW-C15-(	25	VEGFR	0.398765	0
**162**	4-KW-D12-	100	VEGFR	0.237381	0.961
**163**	4-KW-D15-	2.5	VEGFR	0.095786	0.557

**164**	4-KW-D20-	100	VEGFR	0.094106	0.381
**165**	4-KW-E12-I	10	VEGFR	0.248956	0
**166**	4-KW-E20-I	10	VEGFR	0.123252	0.34
**167**	4-KW-F12-I	1	VEGFR	0.2257	0.007
**168**	4-KW-F15-(	0.25	VEGFR	0.13665	0.796
**169**	4-KW-F20-I	1	VEGFR	0.112392	0.197
**170**	4-KW-L16-'	1	VEGFR	0.401256	0.005
**171**	4-KW-M16-	10	VEGFR	0.088693	0.835
**172**	4-KW-N16-	100	VEGFR	0.407405	0.002
**173**	4-KW-O16-	1000	VEGFR	0.243561	0.91
**174**	4-KW-P16-'	10000	VEGFR	0.232703	0.914
**175**	2-KW-L10-I	1	PI3K	0.197851	0.684
**176**	2-KW-M10-	10	PI3K	0.363023	0.005
**177**	2-KW-N10-	100	PI3K	0.329522	0.015
**178**	2-KW-O10-	1000	PI3K	0.357909	0.033
**179**	2-KW-P10-I	10000	PI3K	0.321508	0.094
**180**	3-KW-A16-I	2500	PI3K	0.061229	0.786
**181**	3-KW-C16-I	250	PI3K	0.238691	0.453
**182**	3-KW-D16-	25	PI3K	0.215591	0.461
**183**	3-KW-E16-I	2.5	PI3K	0.082195	0.531
**184**	3-KW-F16-I	0.25	PI3K	0.105092	0.284
**185**	3-KW-F17-I	100000	PI3K	0.301433	0.269
**186**	3-KW-F19-I	500	PI3K	0.330557	0.05
**187**	3-KW-G17-	10000	PI3K	0.255302	0.206
**188**	3-KW-G19-	50	PI3K	0.265012	0.107
**189**	3-KW-H17-	1000	PI3K	0.243249	0.213
**190**	3-KW-I17-N	100	PI3K	0.280642	0.073
**191**	3-KW-I19-D	5	PI3K	0.151977	0.695
**192**	3-KW-J17-N	10	PI3K	0.246275	0.127
**193**	3-KW-J19-D	0.5	PI3K	0.216349	0.711
**194**	3-KW-K19-I	0.05	PI3K	0.105027	0.386
**195**	3-KW-L8-Pi	1	PI3K	0.29868	0.066
**196**	3-KW-L21-'	0.1	PI3K	0.123765	0.531
**197**	3-KW-M8-F	10	PI3K	0.184947	0.627
**198**	3-KW-M21-	1	PI3K	0.13827	0.844

**199**	3-KW-N8-P	100	PI3K	0.305842	0.019
**200**	3-KW-N21-	10	PI3K	0.214382	0.628
**201**	3-KW-O8-P	1000	PI3K	0.322803	0.148
**202**	3-KW-O21-	100	PI3K	0.316531	0.219
**203**	3-KW-P8-Pi	10000	PI3K	0.313251	0.262
**204**	3-KW-P21-1	1000	PI3K	0.344449	0.172
**205**	4-KW-A19-,	2500	PI3K	0.429452	0
**206**	4-KW-B19-,	250	PI3K	0.309702	0.092
**207**	4-KW-C19-,	25	PI3K	0.09171	0.506
**208**	4-KW-D19-	2.5	PI3K	0.074166	0.499
**209**	4-KW-E19-/	0.25	PI3K	0.217277	0.207
**210**	4-KW-F14-I	1000	PI3K	0.38723	0.039
**211**	4-KW-G2-T	2500	PI3K	0.170394	0.363
**212**	4-KW-G5-S	10000	PI3K	0.274428	0.197
**213**	4-KW-G14-	100	PI3K	0.356574	0.048
**214**	4-KW-G20-	10000	PI3K	0.361797	0.132
**215**	4-KW-H2-T	250	PI3K	0.123787	0.122
**216**	4-KW-H5-S	1000	PI3K	0.104595	0.369
**217**	4-KW-H14-	10	PI3K	0.423284	0
**218**	4-KW-H20-	1000	PI3K	0.388381	0.009
**219**	4-KW-I2-TG	25	PI3K	0.084069	0.731
**220**	4-KW-I5-So	100	PI3K	0.117398	0.708
**221**	4-KW-I14-N	1	PI3K	0.420865	0.001
**222**	4-KW-I20-E	100	PI3K	0.181476	0.503
**223**	4-KW-J2-TG	2.5	PI3K	0.129985	0.715
**224**	4-KW-J5-Sc	10	PI3K	0.289908	0.344
**225**	4-KW-J20-E	10	PI3K	0.164195	0.468
**226**	4-KW-K2-T	0.25	PI3K	0.249878	0.536
**227**	4-KW-K4-D	0.1	PI3K	0.326992	0.122
**228**	4-KW-K5-S	1	PI3K	0.318443	0.022
**229**	4-KW-K14-I	0.1	PI3K	0.406152	0
**230**	4-KW-K20-I	1	PI3K	0.344594	0.069
**231**	4-KW-L4-D	1	PI3K	0.296779	0.384
**232**	4-KW-L14-(	0.1	PI3K	0.415447	0
**233**	4-KW-L15-1	1	PI3K	0.164786	0.481

**234**	4-KW-L21-	0.1	PI3K	0.136325	0.41
**235**	4-KW-M14	1	PI3K	0.342282	0.007
**236**	4-KW-M15	10	PI3K	0.399095	0
**237**	4-KW-M21	1	PI3K	0.317651	0.061
**238**	4-KW-N4-D	10	PI3K	0.279098	0.374
**239**	4-KW-N14-	10	PI3K	0.30479	0.048
**240**	4-KW-N15-	100	PI3K	0.449448	0
**241**	4-KW-N21-	10	PI3K	0.311536	0.253
**242**	4-KW-O4-D	100	PI3K	0.294981	0.212
**243**	4-KW-O14-	100	PI3K	0.36071	0.047
**244**	4-KW-O15-	1000	PI3K	0.192485	0.615
**245**	4-KW-O21-	100	PI3K	0.38061	0.028
**246**	4-KW-P4-D	1000	PI3K	0.329594	0.177
**247**	4-KW-P14-	1000	PI3K	0.379843	0.052
**248**	4-KW-P15-	10000	PI3K	0.270128	0.191
**249**	4-KW-P21-	1000	PI3K	0.377411	0.11
**250**	5-KW-A6-L	2500	PI3K	0.35977	0.03
**251**	5-KW-A7-A	1000	PI3K	0.258744	0.114
**252**	5-KW-A16-	2500	PI3K	0.386147	0.012
**253**	5-KW-A17-	10000	PI3K	0.291237	0.045
**254**	5-KW-B6-L	250	PI3K	0.304846	0.072
**255**	5-KW-B7-A	100	PI3K	0.077719	0.487
**256**	5-KW-B17-	1000	PI3K	0.204607	0.054
**257**	5-KW-C6-L	25	PI3K	0.253428	0.151
**258**	5-KW-C7-A	10	PI3K	0.126908	0.907
**259**	5-KW-C16-	250	PI3K	0.351079	0.007
**260**	5-KW-C17-	100	PI3K	0.265071	0.046
**261**	5-KW-D6-L	2.5	PI3K	0.225466	0.497
**262**	5-KW-D7-A	1	PI3K	0.098929	0.256
**263**	5-KW-D16-	25	PI3K	0.305191	0.026
**264**	5-KW-D17-	10	PI3K	0.115855	0.18
**265**	5-KW-E6-L	0.25	PI3K	0.262104	0.157
**266**	5-KW-E7-A	0.1	PI3K	0.170315	0.405
**267**	5-KW-E16-	2.5	PI3K	0.28742	0.05
**268**	5-KW-E17-	1	PI3K	0.299408	0.036

**269**	5-KW-F11-(	10000	PI3K	0.356324	0.004
**270**	5-KW-F16-/	0.25	PI3K	0.274088	0.085
**271**	5-KW-G9-S	10000	PI3K	0.326906	0.005
**272**	5-KW-G11-	1000	PI3K	0.347787	0
**273**	5-KW-H9-S	1000	PI3K	0.311957	0.007
**274**	5-KW-H11-	100	PI3K	0.290464	0.01
**275**	5-KW-I9-Se	100	PI3K	0.145551	0.666
**276**	5-KW-I11-G	10	PI3K	0.273832	0.025
**277**	5-KW-J9-Se	10	PI3K	0.332635	0.006
**278**	5-KW-J11-(	1	PI3K	0.245156	0.049
**279**	5-KW-K9-S	1	PI3K	0.327083	0.005
**280**	5-KW-L14-/	0.1	PI3K	0.292953	0.03
**281**	5-KW-L20-Z	1	PI3K	0.265156	0.433
**282**	5-KW-L23-(	0.1	PI3K	0.117006	0.854
**283**	5-KW-M14-	1	PI3K	0.316031	0.075
**284**	5-KW-M20-	10	PI3K	0.156459	0.826
**285**	5-KW-M23-	1	PI3K	0.19733	0.817
**286**	5-KW-N14-	10	PI3K	0.295798	0.087
**287**	5-KW-N20-	100	PI3K	0.305985	0.094
**288**	5-KW-N23-	10	PI3K	0.342653	0.072
**289**	5-KW-O14-	100	PI3K	0.307696	0.061
**290**	5-KW-O20-	1000	PI3K	0.344639	0.063
**291**	5-KW-O23-	100	PI3K	0.337806	0.068
**292**	5-KW-P14-/	1000	PI3K	0.314661	0.025
**293**	5-KW-P20-Z	10000	PI3K	0.336707	0.073
**294**	5-KW-P23-(	1000	PI3K	0.348873	0.094
**295**	6-KW-A8-T(	10000	PI3K	0.410435	0.002
**296**	6-KW-B8-T(	1000	PI3K	0.197167	0.34
**297**	6-KW-C8-T(	100	PI3K	0.278459	0.319
**298**	6-KW-D8-T(	10	PI3K	0.237393	0.441
**299**	6-KW-E8-T(	1	PI3K	0.118577	0.388
**300**	6-KW-L6-GI	1	PI3K	0.161393	0.926
**301**	6-KW-M6-G	10	PI3K	0.116868	0.741
**302**	6-KW-N6-G	100	PI3K	0.248191	0.329
**303**	6-KW-O6-G	1000	PI3K	0.289373	0.356

**304**	6-KW-P6-G	10000	PI3K	0.327327	0.22
**305**	1-KW-F11-/	10000	Topoisome	0.584508	0
**306**	1-KW-G11-	1000	Topoisome	0.605102	0
**307**	1-KW-G20-	1000	Topoisome	0.542758	0
**308**	1-KW-H11-	100	Topoisome	0.593698	0
**309**	1-KW-H20-	100	Topoisome	0.617611	0
**310**	1-KW-I11-A	10	Topoisome	0.618762	0
**311**	1-KW-I20-E	10	Topoisome	0.521156	0
**312**	1-KW-J11-/	1	Topoisome	0.575958	0
**313**	1-KW-J20-E	1	Topoisome	0.138978	0.896
**314**	1-KW-K11-:	1	Topoisome	0.623626	0
**315**	1-KW-K20-l	0.1	Topoisome	0.499087	0
**316**	1-KW-L11-S	10	Topoisome	0.608172	0
**317**	1-KW-L14-T	1	Topoisome	0.592813	0
**318**	1-KW-M11-	100	Topoisome	0.54454	0
**319**	1-KW-M14-	10	Topoisome	0.620878	0
**320**	1-KW-N14-	100	Topoisome	0.592624	0
**321**	1-KW-O11-	1000	Topoisome	0.535635	0
**322**	1-KW-O14-	1000	Topoisome	0.575421	0
**323**	1-KW-P11-:	10000	Topoisome	0.51822	0
**324**	1-KW-P14-T	10000	Topoisome	0.515191	0
**325**	3-KW-A11-l	10000	Topoisome	0.651584	0
**326**	3-KW-B11-l	1000	Topoisome	0.623853	0
**327**	3-KW-C11-l	100	Topoisome	0.522865	0
**328**	3-KW-D11-	10	Topoisome	0.072026	0.966
**329**	3-KW-E11-l	1	Topoisome	0.532	0
**330**	3-KW-G9-D	1000	Topoisome	0.532544	0
**331**	3-KW-G10-	10000	Topoisome	0.549692	0
**332**	3-KW-H9-D	100	Topoisome	0.653311	0
**333**	3-KW-H10-	1000	Topoisome	0.476975	0
**334**	3-KW-I9-Da	10	Topoisome	0.638224	0
**335**	3-KW-I10-T	100	Topoisome	0.465044	0
**336**	3-KW-J9-Da	1	Topoisome	0.309506	0.03
**337**	3-KW-J10-T	10	Topoisome	0.472061	0
**338**	3-KW-K7-Ic	0.1	Topoisome	0.519672	0

**339**	3-KW-K9-D	0.1	Topoisome	0.508922	0
**340**	3-KW-K10-	1	Topoisome	0.5382	0
**341**	3-KW-L6-D	0.1	Topoisome	0.500433	0.001
**342**	3-KW-L7-Id	1	Topoisome	0.581795	0
**343**	3-KW-L9-V	0.5	Topoisome	0.662447	0
**344**	3-KW-L10-I	0.1	Topoisome	0.515097	0.001
**345**	3-KW-L16-I	1	Topoisome	0.452434	0.003
**346**	3-KW-M6-D	1	Topoisome	0.616213	0
**347**	3-KW-M7-I	10	Topoisome	0.494127	0
**348**	3-KW-M9-V	5	Topoisome	0.555052	0
**349**	3-KW-M10-	1	Topoisome	0.583339	0
**350**	3-KW-M16-	10	Topoisome	0.540525	0
**351**	3-KW-N6-D	10	Topoisome	0.47579	0
**352**	3-KW-N9-V	50	Topoisome	0.584135	0
**353**	3-KW-N10-	10	Topoisome	0.450483	0
**354**	3-KW-N16-	100	Topoisome	0.521804	0
**355**	3-KW-O6-D	100	Topoisome	0.631614	0
**356**	3-KW-O7-I	100	Topoisome	0.604497	0
**357**	3-KW-O9-V	500	Topoisome	0.636098	0
**358**	3-KW-O10-	100	Topoisome	0.640259	0
**359**	3-KW-O16-	1000	Topoisome	0.199926	0.781
**360**	3-KW-P6-D	1000	Topoisome	0.551371	0
**361**	3-KW-P7-Id	1000	Topoisome	0.532015	0
**362**	3-KW-P9-V	5000	Topoisome	0.563962	0
**363**	3-KW-P10-I	1000	Topoisome	0.525401	0
**364**	3-KW-P16-I	10000	Topoisome	0.419821	0.072
**365**	1-KW-A10-	10000	Mitotic	0.675008	0
**366**	1-KW-A13-I	1000	Mitotic	0.681058	0
**367**	1-KW-A18-I	1000	Mitotic	0.674388	0
**368**	1-KW-B10-	1000	Mitotic	0.678041	0
**369**	1-KW-B13-I	100	Mitotic	0.382947	0.033
**370**	1-KW-B18-I	100	Mitotic	0.674987	0
**371**	1-KW-C10-	100	Mitotic	0.676247	0
**372**	1-KW-C13-I	10	Mitotic	0.501612	0.002
**373**	1-KW-C18-I	10	Mitotic	0.675728	0

**374**	1-KW-D10-	10	Mitotic	0.328583	0.055
**375**	1-KW-D13-	1	Mitotic	0.549569	0
**376**	1-KW-D18-	1	Mitotic	0.340556	0.212
**377**	1-KW-E10-\	1	Mitotic	0.424638	0.003
**378**	1-KW-E13-I	0.1	Mitotic	0.468362	0.003
**379**	1-KW-E18-I	0.1	Mitotic	0.333903	0.169
**380**	1-KW-F13-\	1000	Mitotic	0.68918	0
**381**	1-KW-G13-	100	Mitotic	0.410992	0.004
**382**	1-KW-G15-	1000	Mitotic	0.669005	0
**383**	1-KW-H13-	10	Mitotic	0.499761	0
**384**	1-KW-H15-	100	Mitotic	0.67392	0
**385**	1-KW-I13-\	1	Mitotic	0.543784	0
**386**	1-KW-I15-E	10	Mitotic	0.680462	0
**387**	1-KW-J13-\	0.1	Mitotic	0.505506	0
**388**	1-KW-J15-E	1	Mitotic	0.580447	0
**389**	1-KW-K7-Vi	0.1	Mitotic	0.605819	0
**390**	1-KW-K15-I	0.1	Mitotic	0.618615	0
**391**	1-KW-L7-Vi	1	Mitotic	0.499496	0
**392**	1-KW-L20-\	0.1	Mitotic	0.627557	0
**393**	1-KW-M7-\	10	Mitotic	0.38714	0.056
**394**	1-KW-M20-	1	Mitotic	0.442156	0.034
**395**	1-KW-N20-	10	Mitotic	0.640448	0
**396**	1-KW-O7-V	100	Mitotic	0.666636	0
**397**	1-KW-O20-	100	Mitotic	0.556494	0
**398**	1-KW-P7-Vi	1000	Mitotic	0.673303	0
**399**	1-KW-P20-\	1000	Mitotic	0.697788	0
**400**	3-KW-A7-D	1000	Mitotic	0.596386	0
**401**	3-KW-B7-D	100	Mitotic	0.618292	0
**402**	3-KW-C7-D	10	Mitotic	0.622613	0
**403**	3-KW-D7-D	1	Mitotic	0.063281	0.947
**404**	3-KW-E7-D	0.1	Mitotic	0.304653	0.438
**405**	6-KW-L19-/	1	Mitotic	0.476294	0.001
**406**	6-KW-M19-	10	Mitotic	0.300226	0.252
**407**	6-KW-N19-	100	Mitotic	0.317737	0.261
**408**	6-KW-O19-	1000	Mitotic	0.606966	0

**409**	6-KW-P19-/	10000	Mitotic	0.594053	0
**410**	2-KW-A12-'	250	MEK1/2	0.338104	0.744
**411**	2-KW-B12-'	25	MEK1/2	0.442587	0.055
**412**	2-KW-D12-	2.5	MEK1/2	0.456197	0.016
**413**	2-KW-E12-'	0.25	MEK1/2	0.427628	0.011
**414**	2-KW-F12-'	2.5000000	MEK1/2	0.335876	0.061
**415**	2-KW-F14-(	1000	MEK1/2	0.333811	0.405
**416**	2-KW-G14-	100	MEK1/2	0.442673	0.056
**417**	2-KW-H14-	10	MEK1/2	0.559564	0
**418**	2-KW-I14-C	1	MEK1/2	0.317217	0.402
**419**	2-KW-K14-	0.1	MEK1/2	0.215693	0.735
**420**	2-KW-L20-S	1	MEK1/2	0.177338	0.986
**421**	2-KW-M20-	10	MEK1/2	0.167482	0.953
**422**	2-KW-N20-	100	MEK1/2	0.389675	0.172
**423**	2-KW-O20-	1000	MEK1/2	0.327808	0.533
**424**	2-KW-P20-S	10000	MEK1/2	0.351463	0.417
**425**	4-KW-A10-I	1000	MEK1/2	0.612205	0
**426**	4-KW-A13-I	1000	MEK1/2	0.510552	0.028
**427**	4-KW-B10-I	100	MEK1/2	0.452226	0
**428**	4-KW-B13-I	100	MEK1/2	0.500448	0.004
**429**	4-KW-C10-I	10	MEK1/2	0.447112	0.038
**430**	4-KW-C13-I	10	MEK1/2	0.633089	0
**431**	4-KW-D10-	1	MEK1/2	0.491074	0.004
**432**	4-KW-D13-	1	MEK1/2	0.369346	0.024
**433**	4-KW-E10-I	0.1	MEK1/2	0.490418	0
**434**	4-KW-E13-I	0.1	MEK1/2	0.350606	0.005
**435**	4-KW-L19-(	0.25	MEK1/2	0.337003	0.621
**436**	4-KW-M19-	2.5	MEK1/2	0.247225	0.796
**437**	4-KW-N19-	25	MEK1/2	0.218654	0.399
**438**	4-KW-O19-	250	MEK1/2	0.315859	0.582
**439**	4-KW-P19-	2500	MEK1/2	0.456192	0.13
**440**	1-KW-L2-OI	1	PARP	0.567708	0
**441**	1-KW-L6-Ru	1	PARP	0.551819	0.001
**442**	1-KW-M2-C	10	PARP	0.598849	0.002
**443**	1-KW-M6-F	10	PARP	0.503907	0.013

**444**	1-KW-N2-O	100	PARP	0.598009	0.001
**445**	1-KW-N6-R	100	PARP	0.751761	0
**446**	1-KW-O2-O	1000	PARP	0.565152	0.002
**447**	1-KW-O6-R	1000	PARP	0.543637	0.002
**448**	1-KW-P2-O	10000	PARP	0.588113	0
**449**	1-KW-P6-R	10000	PARP	0.498018	0.023
**450**	7-KW-A3-Ta	1000	PARP	0.507923	0.017
**451**	7-KW-B2-Ve	10000	PARP	0.704764	0
**452**	7-KW-B3-Ta	100	PARP	0.564306	0.003
**453**	7-KW-C2-Ve	1000	PARP	0.542131	0.004
**454**	7-KW-C3-Ta	10	PARP	0.539047	0.005
**455**	7-KW-D2-V	100	PARP	0.632446	0
**456**	7-KW-D3-Ta	1	PARP	0.478618	0.045
**457**	7-KW-E2-Ve	10	PARP	0.381545	0.098
**458**	7-KW-E3-Ta	0.1	PARP	0.125521	0.845
**459**	7-KW-F2-Ve	1	PARP	0.36049	0.09
**460**	7-KW-G2-N	10000	PARP	0.559431	0.002
**461**	7-KW-H2-N	1000	PARP	0.621309	0
**462**	7-KW-I2-Ni	100	PARP	0.390449	0.223
**463**	7-KW-J2-Ni	10	PARP	0.45705	0.029
**464**	7-KW-K2-N	1	PARP	0.565442	0
**465**	3-KW-A19-I	1000	CDK	0.38658	0.113
**466**	3-KW-B19-I	100	CDK	0.385387	0.102
**467**	3-KW-B23-A	2500	CDK	0.393264	0.017
**468**	3-KW-C19-I	10	CDK	0.471468	0.001
**469**	3-KW-C23-A	250	CDK	0.423763	0.002
**470**	3-KW-D19-	1	CDK	0.414838	0.001
**471**	3-KW-D23-	25	CDK	0.242897	0.499
**472**	3-KW-E19-I	0.1	CDK	0.102738	0.723
**473**	3-KW-E23-A	2.5	CDK	0.311816	0.392
**474**	3-KW-F23-A	0.25	CDK	0.110228	0.892
**475**	3-KW-K17-I	1	CDK	0.260617	0.261
**476**	3-KW-L19-I	1	CDK	0.278781	0.693
**477**	3-KW-M17-	10	CDK	0.445127	0
**478**	3-KW-M19-	10	CDK	0.277893	0.386

**479**	3-KW-N17-	100	CDK	0.325079	0.088
**480**	3-KW-N19-	100	CDK	0.486975	0
**481**	3-KW-O17-	1000	CDK	0.333362	0.121
**482**	3-KW-O19-	1000	CDK	0.422631	0.002
**483**	3-KW-P17-l	10000	CDK	0.28261	0.301
**484**	3-KW-P19-l	10000	CDK	0.454476	0.001
**485**	4-KW-A4-Sl	10000	CDK	0.382734	0.221
**486**	4-KW-A8-M	10000	CDK	0.398718	0.186
**487**	4-KW-B4-Sl	1000	CDK	0.385024	0.253
**488**	4-KW-B8-M	1000	CDK	0.434129	0.002
**489**	4-KW-C4-Sl	100	CDK	0.432752	0.002
**490**	4-KW-C8-M	100	CDK	0.269705	0.062
**491**	4-KW-D4-Sl	10	CDK	0.195066	0.175
**492**	4-KW-D8-M	10	CDK	0.14586	0.576
**493**	4-KW-E4-SI	1	CDK	0.237743	0.133
**494**	4-KW-E8-M	1	CDK	0.093614	0.542
**495**	4-KW-F4-Se	10000	CDK	0.4293	0.008
**496**	4-KW-F22-/	10000	CDK	0.405856	0.116
**497**	4-KW-G4-S	1000	CDK	0.328301	0.329
**498**	4-KW-G22-	1000	CDK	0.378833	0.319
**499**	4-KW-H4-S	100	CDK	0.16497	0.203
**500**	4-KW-H22-	100	CDK	0.430374	0.001
**501**	4-KW-I4-Se	10	CDK	0.158664	0.461
**502**	4-KW-I22-A	10	CDK	0.290913	0.034
**503**	4-KW-J4-Se	1	CDK	0.180777	0.627
**504**	4-KW-J22-/	1	CDK	0.194165	0.092
**505**	5-KW-A19-,	10000	CDK	0.356306	0.222
**506**	5-KW-B19-,	1000	CDK	0.36626	0.226
**507**	5-KW-C19-,	100	CDK	0.294943	0.266
**508**	5-KW-D19-	10	CDK	0.165107	0.937
**509**	5-KW-E19-/	1	CDK	0.474518	0.001
**510**	5-KW-K17-,	1	CDK	0.254463	0.446
**511**	5-KW-M17-	10	CDK	0.368641	0.037
**512**	5-KW-N17-	100	CDK	0.463801	0
**513**	5-KW-O17-	1000	CDK	0.355331	0.252

**514**	5-KW-P17-/	10000	CDK	0.358847	0.194
**515**	6-KW-A17-:	1000	CDK	0.291503	0.44
**516**	6-KW-B17-:	100	CDK	0.265503	0.201
**517**	6-KW-C17-:	10	CDK	0.259988	0.047
**518**	6-KW-D17-	1	CDK	0.250023	0.19
**519**	6-KW-E17-:	0.1	CDK	0.363464	0.038
**520**	6-KW-L15-7	1	CDK	0.150233	0.896
**521**	6-KW-M15-	10	CDK	0.423419	0
**522**	6-KW-N15-	100	CDK	0.420188	0.01
**523**	6-KW-O15-	1000	CDK	0.344052	0.361
**524**	6-KW-P15-7	10000	CDK	0.321221	0.416
**525**	7-KW-A21-	10000	BET	0.512274	0.001
**526**	7-KW-A22-I	30000	BET	0.693045	0
**527**	7-KW-B21-	1000	BET	0.171263	0.573
**528**	7-KW-B22-I	3000	BET	0.734542	0
**529**	7-KW-C21-	100	BET	0.425806	0.036
**530**	7-KW-C22-I	300	BET	0.173766	0.452
**531**	7-KW-D21-	10	BET	0.164867	0.571
**532**	7-KW-D22-	30	BET	0.34901	0.099
**533**	7-KW-E21-(	1	BET	0.515086	0
**534**	7-KW-E22-I	3	BET	0.526697	0
**535**	7-KW-G10-	10000	BET	0.566337	0
**536**	7-KW-G15-	10000	BET	0.596185	0
**537**	7-KW-H10-	1000	BET	0.608873	0
**538**	7-KW-H15-	1000	BET	0.719072	0
**539**	7-KW-I10-E	100	BET	0.564105	0
**540**	7-KW-I15-I	100	BET	0.516246	0.001
**541**	7-KW-J10-E	10	BET	0.346971	0.125
**542**	7-KW-J15-I	10	BET	0.349994	0.059
**543**	7-KW-K10-I	1	BET	0.310582	0.162
**544**	7-KW-K13-I	1	BET	0.379831	0.038
**545**	7-KW-K15-I	1	BET	0.47672	0
**546**	7-KW-L12-I	1	BET	0.547829	0
**547**	7-KW-L13-I	10	BET	0.605725	0
**548**	7-KW-L20-J	1	BET	0.492259	0.011

549	7-KW-L23-/	0.03	BET	0.37612	0.084
550	7-KW-M12-	10	BET	0.473777	0.002
551	7-KW-M13-	100	BET	0.548119	0
552	7-KW-M20-	10	BET	0.378584	0.078
553	7-KW-M23-	0.3	BET	0.376752	0.077
554	7-KW-N12-	100	BET	0.487892	0
555	7-KW-N13-	1000	BET	0.529486	0.003
556	7-KW-N20-	100	BET	0.643913	0
557	7-KW-N23-	3	BET	0.556803	0
558	7-KW-O12-	1000	BET	0.689962	0
559	7-KW-O20-	1000	BET	0.637079	0
560	7-KW-O23-	30	BET	0.550917	0
561	7-KW-P12-I	10000	BET	0.650577	0
562	7-KW-P13-I	10000	BET	0.471453	0.021
563	7-KW-P20-.	10000	BET	0.528382	0.002
564	7-KW-P23-,	300	BET	0.525532	0
565	8-KW-K22-(	1	BET	0.198869	0.859
566	8-KW-L22-(	10	BET	0.132351	0.925
567	8-KW-M22-	100	BET	0.347041	0.091
568	8-KW-N22-	1000	BET	0.528075	0
569	8-KW-O22-	10000	BET	0.470037	0.012
570	1-KW-A3-V	10000	HDAC	0.438574	0
571	1-KW-B3-V	1000	HDAC	0.482386	0
572	1-KW-C3-V	100	HDAC	0.043996	0.998
573	1-KW-D3-V	10	HDAC	0.036373	0.982
574	1-KW-E3-V	1	HDAC	0.055436	0.882
575	1-KW-L12-I	0.1	HDAC	0.495356	0
576	1-KW-M12-	1	HDAC	0.51061	0
577	1-KW-N12-	10	HDAC	0.464903	0
578	1-KW-O12-	100	HDAC	0.46849	0
579	1-KW-P12-I	1000	HDAC	0.471701	0
580	3-KW-A4-P	1000	HDAC	0.453121	0
581	3-KW-B4-P	100	HDAC	0.454958	0
582	3-KW-C4-P	10	HDAC	0.380389	0
583	3-KW-D4-P	1	HDAC	0.2052	0.699

**584**	3-KW-E4-Pa	0.1	HDAC	0.020984	1
**585**	3-KW-F7-Q	1000	HDAC	0.457237	0
**586**	3-KW-G7-Q	100	HDAC	0.450783	0
**587**	3-KW-G12-	1000000	HDAC	0.272211	0.228
**588**	3-KW-H7-Q	10	HDAC	0.3901	0
**589**	3-KW-H12-	100000	HDAC	0.316034	0.022
**590**	3-KW-I7-Qu	1	HDAC	0.493607	0
**591**	3-KW-I12-V	10000	HDAC	0.342399	0.015
**592**	3-KW-J7-Qu	0.1	HDAC	0.034683	0.938
**593**	3-KW-J12-V	1000	HDAC	0.335604	0.002
**594**	3-KW-K3-Be	1	HDAC	0.02897	1
**595**	3-KW-K12-V	100	HDAC	0.274229	0.113
**596**	3-KW-L3-Be	10	HDAC	0.267847	0.453
**597**	3-KW-M3-B	100	HDAC	0.47455	0
**598**	3-KW-N3-B	1000	HDAC	0.453638	0
**599**	3-KW-O3-B	10000	HDAC	0.456224	0
**600**	7-KW-A5-M	10000	HDAC	0.451541	0
**601**	7-KW-A7-C	10000	HDAC	0.474232	0
**602**	7-KW-A9-G	1000	HDAC	0.478922	0
**603**	7-KW-A12-I	10000	HDAC	0.502521	0
**604**	7-KW-B5-M	1000	HDAC	0.500129	0
**605**	7-KW-B7-C	1000	HDAC	0.486966	0
**606**	7-KW-B12-I	1000	HDAC	0.567047	0
**607**	7-KW-C5-M	100	HDAC	0.465869	0
**608**	7-KW-C7-C	100	HDAC	0.473783	0
**609**	7-KW-C9-G	100	HDAC	0.51496	0
**610**	7-KW-D7-C	10	HDAC	0.113815	0.938
**611**	7-KW-D9-G	10	HDAC	0.47451	0
**612**	7-KW-D12-	100	HDAC	0.419084	0
**613**	7-KW-E5-M	10	HDAC	0.147707	0.844
**614**	7-KW-E7-C	1	HDAC	0.102391	0.947
**615**	7-KW-E9-G	1	HDAC	0.486515	0
**616**	7-KW-E12-I	10	HDAC	0.486818	0
**617**	7-KW-F5-M	1	HDAC	0.027692	0.98
**618**	7-KW-F7-Re	10000	HDAC	0.47923	0

**619**	7-KW-F9-Gi	0.1	HDAC	0.065976	0.905
**620**	7-KW-F12-I	1	HDAC	0.48128	0
**621**	7-KW-F19-I	10000	HDAC	0.468989	0
**622**	7-KW-G7-R	1000	HDAC	0.50347	0
**623**	7-KW-G19-	1000	HDAC	0.441477	0
**624**	7-KW-H7-R	100	HDAC	0.46328	0
**625**	7-KW-I7-Re	10	HDAC	0.464899	0
**626**	7-KW-I19-F	100	HDAC	0.497328	0
**627**	7-KW-J7-Re	1	HDAC	0.331962	0.015
**628**	7-KW-J19-F	10	HDAC	0.084401	0.744
**629**	7-KW-K4-Er	1	HDAC	0.415777	0
**630**	7-KW-K11-/	1	HDAC	0.458669	0
**631**	7-KW-K18-T	1	HDAC	0.47991	0
**632**	7-KW-K19-I	1	HDAC	0.492749	0
**633**	7-KW-L2-Ta	0.1	HDAC	0.485847	0
**634**	7-KW-L4-Er	10	HDAC	0.349783	0.049
**635**	7-KW-L5-Pr	1	HDAC	0.449595	0
**636**	7-KW-L8-Al	1	HDAC	0.309519	0.037
**637**	7-KW-L10-T	1	HDAC	0.487329	0
**638**	7-KW-L11-/	10	HDAC	0.515736	0
**639**	7-KW-L14-T	1	HDAC	0.457591	0
**640**	7-KW-L16-I	1	HDAC	0.484154	0
**641**	7-KW-L18-T	10	HDAC	0.490866	0
**642**	7-KW-M2-T	1	HDAC	0.434903	0
**643**	7-KW-M5-F	10	HDAC	0.458789	0
**644**	7-KW-M8-A	10	HDAC	0.394651	0
**645**	7-KW-M10-	10	HDAC	0.087803	0.771
**646**	7-KW-M11-	100	HDAC	0.472826	0
**647**	7-KW-M14-	10	HDAC	0.491411	0
**648**	7-KW-M16-	10	HDAC	0.4457	0
**649**	7-KW-M18-	100	HDAC	0.476685	0
**650**	7-KW-N2-Ta	10	HDAC	0.422603	0
**651**	7-KW-N4-Er	100	HDAC	0.473486	0
**652**	7-KW-N5-P	100	HDAC	0.496902	0
**653**	7-KW-N8-A	100	HDAC	0.510528	0

**654**	7-KW-N10-	100	HDAC	0.485525	0
**655**	7-KW-N14-	100	HDAC	0.480675	0
**656**	7-KW-N16-	100	HDAC	0.460443	0
**657**	7-KW-N18-	1000	HDAC	0.441981	0
**658**	7-KW-O2-Ta	100	HDAC	0.510791	0
**659**	7-KW-O4-Er	1000	HDAC	0.494945	0
**660**	7-KW-O5-P	1000	HDAC	0.478768	0
**661**	7-KW-O8-A	1000	HDAC	0.468008	0
**662**	7-KW-O10-	1000	HDAC	0.464187	0
**663**	7-KW-O11-	1000	HDAC	0.478141	0
**664**	7-KW-O14-	1000	HDAC	0.402609	0
**665**	7-KW-O16-	1000	HDAC	0.521653	0
**666**	7-KW-P2-Ta	1000	HDAC	0.471652	0
**667**	7-KW-P4-Er	10000	HDAC	0.475035	0
**668**	7-KW-P5-Pr	10000	HDAC	0.492508	0
**669**	7-KW-P8-Al	10000	HDAC	0.479934	0
**670**	7-KW-P10-T	10000	HDAC	0.497442	0
**671**	7-KW-P11-A	10000	HDAC	0.481746	0
**672**	7-KW-P14-T	10000	HDAC	0.473539	0
**673**	7-KW-P16-I	10000	HDAC	0.465439	0
**674**	7-KW-P18-T	10000	HDAC	0.471071	0
**0**	2-MHB-A16	10000	EGFR	0.459718	0
**1**	2-MHB-A19	10000	EGFR	0.464763	0
**2**	2-MHB-B19	1000	EGFR	0.484034	0
**3**	2-MHB-C16	1000	EGFR	0.539445	0
**4**	2-MHB-C19	100	EGFR	0.473815	0
**5**	2-MHB-D16	100	EGFR	0.531436	0
**6**	2-MHB-D19	10	EGFR	0.04209	0.964
**7**	2-MHB-E16	10	EGFR	0.562406	0
**8**	2-MHB-E19	1	EGFR	0.041232	0.969
**9**	2-MHB-F16	1	EGFR	0.503678	0
**10**	2-MHB-K11	0.1	EGFR	0.524044	0
**11**	2-MHB-L11	1	EGFR	0.527706	0
**12**	2-MHB-L16	0.25	EGFR	0.032107	0.977
**13**	2-MHB-L19	0.1	EGFR	0.617539	0

**14**	2-MHB-M1[illegible]	10	EGFR	0.504633	0
**15**	2-MHB-M1[illegible]	2.5	EGFR	0.559256	0
**16**	2-MHB-M1[illegible]	1	EGFR	0.594313	0
**17**	2-MHB-N1[illegible]	25	EGFR	0.53712	0
**18**	2-MHB-N1[illegible]	10	EGFR	0.547988	0
**19**	2-MHB-O11	100	EGFR	0.535792	0
**20**	2-MHB-O1[illegible]	250	EGFR	0.469915	0
**21**	2-MHB-O1[illegible]	100	EGFR	0.534809	0
**22**	2-MHB-P11	1000	EGFR	0.455022	0
**23**	2-MHB-P1[illegible]	2500	EGFR	0.51539	0
**24**	2-MHB-P1[illegible]	1000	EGFR	0.466187	0
**25**	3-MHB-F21	10000	EGFR	0.411353	0
**26**	3-MHB-G2[illegible]	1000	EGFR	0.458486	0
**27**	3-MHB-G21	1000	EGFR	0.425776	0
**28**	3-MHB-H2[illegible]	100	EGFR	0.472329	0
**29**	3-MHB-H21	100	EGFR	0.06595	1
**30**	3-MHB-I20[illegible]	10	EGFR	0.470712	0
**31**	3-MHB-I21[illegible]	10	EGFR	0.03979	1
**32**	3-MHB-J20	1	EGFR	0.035079	0.955
**33**	3-MHB-J21	1	EGFR	0.454721	0
**34**	3-MHB-K4-[illegible]	1	EGFR	0.416579	0
**35**	3-MHB-K18	0.1	EGFR	0.490876	0
**36**	3-MHB-K2[illegible]	0.1	EGFR	0.533077	0
**37**	3-MHB-L4-[illegible]	10	EGFR	0.451568	0
**38**	3-MHB-L18	1	EGFR	0.515917	0
**39**	3-MHB-M1[illegible]	10	EGFR	0.465585	0
**40**	3-MHB-N4-	100	EGFR	0.466874	0
**41**	3-MHB-N1[illegible]	100	EGFR	0.466447	0
**42**	3-MHB-O4-	1000	EGFR	0.451179	0
**43**	3-MHB-P4-[illegible]	10000	EGFR	0.429726	0
**44**	3-MHB-P18	1000	EGFR	0.423266	0
**45**	4-MHB-F13	1000	EGFR	0.516564	0
**46**	4-MHB-G1[illegible]	100	EGFR	0.512573	0
**47**	4-MHB-G1[illegible]	10000	EGFR	0.499982	0
**48**	4-MHB-H1[illegible]	10	EGFR	0.526386	0

**49**	4-MHB-H16	1000	EGFR	0.43529	0
**50**	4-MHB-I13-	1	EGFR	0.511921	0
**51**	4-MHB-I16-	100	EGFR	0.549648	0
**52**	4-MHB-J13	0.1	EGFR	0.154441	0.419
**53**	4-MHB-J16	10	EGFR	0.060977	0.848
**54**	4-MHB-K7-I	1	EGFR	0.520461	0
**55**	4-MHB-K13	0.1	EGFR	0.490327	0
**56**	4-MHB-K16	1	EGFR	0.052071	0.878
**57**	4-MHB-L7-I	10	EGFR	0.493033	0
**58**	4-MHB-L13	1	EGFR	0.517468	0
**59**	4-MHB-M7-	100	EGFR	0.5059	0
**60**	4-MHB-M1:	10	EGFR	0.512115	0
**61**	4-MHB-N13	100	EGFR	0.54689	0
**62**	4-MHB-O7-	1000	EGFR	0.583915	0
**63**	4-MHB-P7-I	10000	EGFR	0.527552	0
**64**	4-MHB-P13	1000	EGFR	0.476723	0
**65**	5-MHB-F4-I	1000	EGFR	0.481986	0
**66**	5-MHB-F7-/	1000	EGFR	0.490662	0
**67**	5-MHB-G4-	100	EGFR	0.483841	0
**68**	5-MHB-G7-	100	EGFR	0.479479	0
**69**	5-MHB-H4-	10	EGFR	0.462177	0
**70**	5-MHB-H7-	10	EGFR	0.499205	0
**71**	5-MHB-I4-F	1	EGFR	0.468574	0
**72**	5-MHB-I7-A	1	EGFR	0.037953	0.944
**73**	5-MHB-J4-F	0.1	EGFR	0.420157	0.001
**74**	5-MHB-J7-/	0.1	EGFR	0.527153	0
**75**	5-MHB-K7-(	0.1	EGFR	0.518297	0
**76**	5-MHB-L7-(	1	EGFR	0.046224	0.903
**77**	5-MHB-M7-	10	EGFR	0.178179	0.727
**78**	5-MHB-O7-	100	EGFR	0.413317	0.005
**79**	5-MHB-P7-(	1000	EGFR	0.455986	0
**80**	2-MHB-A15	2500	VEGFR	0.074167	0.998
**81**	2-MHB-A17	10000	VEGFR	0.070187	0.998
**82**	2-MHB-A2C	10000	VEGFR	0.0764	0.997
**83**	2-MHB-B15	250	VEGFR	0.097288	0.979

**84**	2-MHB-B17	1000	VEGFR	0.10424	0.976
**85**	2-MHB-B20	1000	VEGFR	0.263474	0.175
**86**	2-MHB-C15	25	VEGFR	0.125665	0.938
**87**	2-MHB-C17	100	VEGFR	0.20356	0.792
**88**	2-MHB-D15	2.5	VEGFR	0.284928	0.03
**89**	2-MHB-D17	10	VEGFR	0.240661	0.226
**90**	2-MHB-D20	100	VEGFR	0.240887	0.006
**91**	2-MHB-E17	1	VEGFR	0.223371	0.021
**92**	2-MHB-E20	10	VEGFR	0.262936	0.059
**93**	2-MHB-F13	10000	VEGFR	0.14717	0.827
**94**	2-MHB-F15	0.25	VEGFR	0.286934	0
**95**	2-MHB-F19	10000	VEGFR	0.104303	0.982
**96**	2-MHB-F20	1	VEGFR	0.251514	0
**97**	2-MHB-F21	10000	VEGFR	0.214513	0.008
**98**	2-MHB-G10	10000	VEGFR	0.21151	0.004
**99**	2-MHB-G13	1000	VEGFR	0.197257	0.539
**100**	2-MHB-G19	1000	VEGFR	0.172932	0.918
**101**	2-MHB-G21	1000	VEGFR	0.287252	0.005
**102**	2-MHB-H10	1000	VEGFR	0.320041	0
**103**	2-MHB-H13	100	VEGFR	0.259907	0.034
**104**	2-MHB-H21	100	VEGFR	0.349335	0.072
**105**	2-MHB-I10-	100	VEGFR	0.261402	0
**106**	2-MHB-I13-	10	VEGFR	0.36253	0
**107**	2-MHB-I19-	100	VEGFR	0.169646	0.893
**108**	2-MHB-I21-	10	VEGFR	0.287098	0
**109**	2-MHB-J10	10	VEGFR	0.302261	0
**110**	2-MHB-J13	1	VEGFR	0.331602	0
**111**	2-MHB-J19	10	VEGFR	0.401129	0.002
**112**	2-MHB-J21	1	VEGFR	0.244822	0.002
**113**	2-MHB-K10	1	VEGFR	0.253665	0
**114**	2-MHB-K13	0.1	VEGFR	0.333267	0.003
**115**	2-MHB-K17	1	VEGFR	0.376516	0.004
**116**	2-MHB-K19	1	VEGFR	0.231435	0.003
**117**	2-MHB-L12	0.1	VEGFR	0.351666	0
**118**	2-MHB-L13	1	VEGFR	0.108157	0.974

119	2-MHB-L21	0.1	VEGFR	0.371457	0
120	2-MHB-M1	1	VEGFR	0.154538	0.694
121	2-MHB-M1	10	VEGFR	0.281954	0
122	2-MHB-M1	10	VEGFR	0.114676	0.961
123	2-MHB-M2	1	VEGFR	0.27836	0
124	2-MHB-N1	10	VEGFR	0.264912	0
125	2-MHB-N1	100	VEGFR	0.11373	0.962
126	2-MHB-N1	100	VEGFR	0.256446	0.114
127	2-MHB-N21	10	VEGFR	0.213885	0.166
128	2-MHB-O1	100	VEGFR	0.170102	0.089
129	2-MHB-O1	1000	VEGFR	0.213319	0.499
130	2-MHB-O21	100	VEGFR	0.284742	0
131	2-MHB-P12	1000	VEGFR	0.208419	0.013
132	2-MHB-P13	1000	VEGFR	0.050635	1
133	2-MHB-P17	10000	VEGFR	0.219948	0.016
134	2-MHB-P21	1000	VEGFR	0.236915	0.51
135	3-MHB-A3-	1000	VEGFR	0.181639	0.687
136	3-MHB-A6-	1000	VEGFR	0.10003	0.986
137	3-MHB-A18	1000	VEGFR	0.174985	0.736
138	3-MHB-B3-	100	VEGFR	0.106327	0.974
139	3-MHB-B6-	100	VEGFR	0.190578	0.841
140	3-MHB-B18	100	VEGFR	0.094885	0.991
141	3-MHB-C3-	10	VEGFR	0.192321	0.749
142	3-MHB-C6-	10	VEGFR	0.173877	0.72
143	3-MHB-C18	10	VEGFR	0.099262	0.996
144	3-MHB-D3-	1	VEGFR	0.166573	0.953
145	3-MHB-D6-	1	VEGFR	0.185434	0.826
146	3-MHB-D1	1	VEGFR	0.113952	0.968
147	3-MHB-E3-	0.1	VEGFR	0.139805	0.383
148	3-MHB-E6-	0.1	VEGFR	0.184082	0.666
149	3-MHB-E18	0.1	VEGFR	0.165262	0.803
150	3-MHB-F18	1000	VEGFR	0.20168	0.875
151	3-MHB-G1	100	VEGFR	0.188529	0.024
152	3-MHB-H1	10	VEGFR	0.162303	0.066
153	3-MHB-I18	1	VEGFR	0.194827	0.006

**154**	3-MHB-J18	0.1	VEGFR	0.193875	0.022
**155**	4-MHB-A12	10000	VEGFR	0.069472	1
**156**	4-MHB-A15	2500	VEGFR	0.189699	0.929
**157**	4-MHB-A20	10000	VEGFR	0.140248	0.928
**158**	4-MHB-B12	1000	VEGFR	0.116925	0.976
**159**	4-MHB-B15	250	VEGFR	0.125611	0.962
**160**	4-MHB-B20	1000	VEGFR	0.068156	0.998
**161**	4-MHB-C15	25	VEGFR	0.114419	0.952
**162**	4-MHB-D12	100	VEGFR	0.249996	0.101
**163**	4-MHB-D15	2.5	VEGFR	0.255702	0.143
**164**	4-MHB-D20	100	VEGFR	0.136925	0.969
**165**	4-MHB-E12	10	VEGFR	0.296908	0
**166**	4-MHB-E20	10	VEGFR	0.046747	1
**167**	4-MHB-F12	1	VEGFR	0.274411	0.053
**168**	4-MHB-F15	0.25	VEGFR	0.353153	0.035
**169**	4-MHB-F20	1	VEGFR	0.053192	0.999
**170**	4-MHB-L16	1	VEGFR	0.199872	0.432
**171**	4-MHB-M16	10	VEGFR	0.333349	0.013
**172**	4-MHB-N16	100	VEGFR	0.085279	0.995
**173**	4-MHB-O16	1000	VEGFR	0.081985	0.997
**174**	4-MHB-P16	10000	VEGFR	0.182681	0.269
**175**	2-MHB-L10	1	PI3K	0.199546	0.352
**176**	2-MHB-M10	10	PI3K	0.07359	0.904
**177**	2-MHB-N10	100	PI3K	0.15333	0.988
**178**	2-MHB-O10	1000	PI3K	0.166897	0.794
**179**	2-MHB-P10	10000	PI3K	0.167338	0.894
**180**	3-MHB-A16	2500	PI3K	0.197477	0.82
**181**	3-MHB-C16	250	PI3K	0.147069	0.906
**182**	3-MHB-D16	25	PI3K	0.093681	0.862
**183**	3-MHB-E16	2.5	PI3K	0.292149	0.019
**184**	3-MHB-F16	0.25	PI3K	0.217514	0.211
**185**	3-MHB-F17	100000	PI3K	0.190182	0.766
**186**	3-MHB-F19	500	PI3K	0.178514	0.72
**187**	3-MHB-G17	10000	PI3K	0.110544	0.201
**188**	3-MHB-G19	50	PI3K	0.230724	0.095

**189**	3-MHB-H17	1000	PI3K	0.083572	0.39
**190**	3-MHB-I17-	100	PI3K	0.072826	0.685
**191**	3-MHB-I19-	5	PI3K	0.233442	0.348
**192**	3-MHB-J17	10	PI3K	0.107764	0.492
**193**	3-MHB-J19	0.5	PI3K	0.322294	0.002
**194**	3-MHB-K19	0.05	PI3K	0.124475	0.213
**195**	3-MHB-L8-I	1	PI3K	0.285473	0.082
**196**	3-MHB-L21	0.1	PI3K	0.240442	0.062
**197**	3-MHB-M8-	10	PI3K	0.193276	0.182
**198**	3-MHB-M21	1	PI3K	0.092502	0.655
**199**	3-MHB-N8-	100	PI3K	0.1844	0.683
**200**	3-MHB-N21	10	PI3K	0.21428	0.477
**201**	3-MHB-O8-	1000	PI3K	0.192241	0.672
**202**	3-MHB-O21	100	PI3K	0.17796	0.77
**203**	3-MHB-P8-I	10000	PI3K	0.190542	0.527
**204**	3-MHB-P21	1000	PI3K	0.184686	0.706
**205**	4-MHB-A19	2500	PI3K	0.182	0.815
**206**	4-MHB-B19	250	PI3K	0.168347	0.864
**207**	4-MHB-C19	25	PI3K	0.153504	0.513
**208**	4-MHB-D19	2.5	PI3K	0.190294	0.739
**209**	4-MHB-E19	0.25	PI3K	0.239042	0.341
**210**	4-MHB-G2-	2500	PI3K	0.209293	0.354
**211**	4-MHB-G5-	10000	PI3K	0.238211	0.217
**212**	4-MHB-G14	100	PI3K	0.261386	0.092
**213**	4-MHB-G20	10000	PI3K	0.193515	0.678
**214**	4-MHB-H2-	250	PI3K	0.275049	0.434
**215**	4-MHB-H5-	1000	PI3K	0.289599	0.037
**216**	4-MHB-H14	10	PI3K	0.267775	0.097
**217**	4-MHB-H20	1000	PI3K	0.244608	0.2
**218**	4-MHB-I2-T	25	PI3K	0.123257	0.577
**219**	4-MHB-I5-S	100	PI3K	0.293187	0.032
**220**	4-MHB-I14-	1	PI3K	0.078165	0.529
**221**	4-MHB-I20-	100	PI3K	0.242584	0.158
**222**	4-MHB-J2-T	2.5	PI3K	0.368074	0.034
**223**	4-MHB-J5-S	10	PI3K	0.264845	0.005

**224**	4-MHB-J20	10	PI3K	0.294561	0.007
**225**	4-MHB-K2-[illegible]	0.25	PI3K	0.291693	0.125
**226**	4-MHB-K4-[illegible]	0.1	PI3K	0.304521	0.051
**227**	4-MHB-K5-[illegible]	1	PI3K	0.345541	0.053
**228**	4-MHB-K14	0.1	PI3K	0.121052	0.747
**229**	4-MHB-K20	1	PI3K	0.279583	0.006
**230**	4-MHB-L4-[illegible]	1	PI3K	0.113639	0.414
**231**	4-MHB-L14	0.1	PI3K	0.279746	0.008
**232**	4-MHB-L15	1	PI3K	0.065994	0.852
**233**	4-MHB-L21	0.1	PI3K	0.300762	0.006
**234**	4-MHB-M1[illegible]	1	PI3K	0.179661	0.169
**235**	4-MHB-M1[illegible]	10	PI3K	0.269865	0.013
**236**	4-MHB-M2[illegible]	1	PI3K	0.210666	0.019
**237**	4-MHB-N4-	10	PI3K	0.293626	0.073
**238**	4-MHB-N1[illegible]	10	PI3K	0.370973	0
**239**	4-MHB-N15	100	PI3K	0.231095	0.228
**240**	4-MHB-N21	10	PI3K	0.2494	0.152
**241**	4-MHB-O4-	100	PI3K	0.205799	0.561
**242**	4-MHB-O1[illegible]	100	PI3K	0.233508	0.234
**243**	4-MHB-O15	1000	PI3K	0.228403	0.174
**244**	4-MHB-O21	100	PI3K	0.208917	0.535
**245**	4-MHB-P4-[illegible]	1000	PI3K	0.195676	0.679
**246**	4-MHB-P14	1000	PI3K	0.202032	0.643
**247**	4-MHB-P15	10000	PI3K	0.182016	0.74
**248**	4-MHB-P21	1000	PI3K	0.182996	0.725
**249**	5-MHB-A6-[illegible]	2500	PI3K	0.422636	0
**250**	5-MHB-A7-[illegible]	1000	PI3K	0.15339	0.504
**251**	5-MHB-A16	2500	PI3K	0.146782	0.972
**252**	5-MHB-A17	10000	PI3K	0.194532	0.582
**253**	5-MHB-B6-[illegible]	250	PI3K	0.227248	0.275
**254**	5-MHB-B7-[illegible]	100	PI3K	0.20927	0.3
**255**	5-MHB-B17	1000	PI3K	0.20988	0.45
**256**	5-MHB-C6-[illegible]	25	PI3K	0.190399	0.212
**257**	5-MHB-C7-[illegible]	10	PI3K	0.152668	0.189
**258**	5-MHB-C16	250	PI3K	0.229046	0.006

**259**	5-MHB-C17	100	PI3K	0.192691	0.025
**260**	5-MHB-D6-	2.5	PI3K	0.266498	0.083
**261**	5-MHB-D7-	1	PI3K	0.306265	0.041
**262**	5-MHB-D16	25	PI3K	0.150738	0.627
**263**	5-MHB-D17	10	PI3K	0.060222	0.734
**264**	5-MHB-E6-I	0.25	PI3K	0.172501	0.052
**265**	5-MHB-E7-/	0.1	PI3K	0.239581	0.009
**266**	5-MHB-E16	2.5	PI3K	0.143421	0.069
**267**	5-MHB-E17	1	PI3K	0.194139	0.008
**268**	5-MHB-F11	10000	PI3K	0.43184	0
**269**	5-MHB-F16	0.25	PI3K	0.186143	0.013
**270**	5-MHB-G9-	10000	PI3K	0.194801	0.625
**271**	5-MHB-G11	1000	PI3K	0.191075	0.022
**272**	5-MHB-H9-	1000	PI3K	0.354515	0.054
**273**	5-MHB-H11	100	PI3K	0.268498	0.143
**274**	5-MHB-I9-S	100	PI3K	0.226326	0.158
**275**	5-MHB-I11-	10	PI3K	0.167057	0.502
**276**	5-MHB-J9-S	10	PI3K	0.162291	0.049
**277**	5-MHB-J11	1	PI3K	0.133876	0.775
**278**	5-MHB-K9-S	1	PI3K	0.238483	0.14
**279**	5-MHB-L14	0.1	PI3K	0.130248	0.305
**280**	5-MHB-L20	1	PI3K	0.231477	0.281
**281**	5-MHB-L23	0.1	PI3K	0.180118	0.434
**282**	5-MHB-M14	1	PI3K	0.253572	0.099
**283**	5-MHB-M20	10	PI3K	0.136204	0.132
**284**	5-MHB-M23	1	PI3K	0.196083	0.839
**285**	5-MHB-N14	10	PI3K	0.227904	0.163
**286**	5-MHB-N20	100	PI3K	0.426175	0.001
**287**	5-MHB-N23	10	PI3K	0.171662	0.807
**288**	5-MHB-O14	100	PI3K	0.268047	0.058
**289**	5-MHB-O20	1000	PI3K	0.182602	0.675
**290**	5-MHB-O23	100	PI3K	0.176285	0.787
**291**	5-MHB-P14	1000	PI3K	0.211027	0.749
**292**	5-MHB-P20	10000	PI3K	0.19785	0.626
**293**	5-MHB-P23	1000	PI3K	0.18238	0.666

**294**	6-MHB-A8-7	10000	PI3K	0.181478	0.792
**295**	6-MHB-B8-7	1000	PI3K	0.163218	0.841
**296**	6-MHB-C8-7	100	PI3K	0.104006	0.998
**297**	6-MHB-D8-	10	PI3K	0.262096	0.533
**298**	6-MHB-E8-7	1	PI3K	0.042335	0.878
**299**	6-MHB-L6-(	1	PI3K	0.181998	0.796
**300**	6-MHB-M6-	10	PI3K	0.256326	0.161
**301**	6-MHB-N6-	100	PI3K	0.183986	0.698
**302**	6-MHB-O6-	1000	PI3K	0.202281	0.581
**303**	6-MHB-P6-(	10000	PI3K	0.2111	0.462
**304**	1-MHB-F11	10000	Topoisome	0.378034	0.017
**305**	1-MHB-G11	1000	Topoisome	0.44418	0
**306**	1-MHB-G2(	1000	Topoisome	0.36586	0.02
**307**	1-MHB-H11	100	Topoisome	0.467785	0
**308**	1-MHB-H2(	100	Topoisome	0.400723	0.001
**309**	1-MHB-I11-	10	Topoisome	0.088	0.787
**310**	1-MHB-I20-	10	Topoisome	0.123476	0.636
**311**	1-MHB-J11	1	Topoisome	0.448683	0.001
**312**	1-MHB-J20	1	Topoisome	0.066891	0.792
**313**	1-MHB-K11	1	Topoisome	0.544449	0
**314**	1-MHB-K2(	0.1	Topoisome	0.173978	0.745
**315**	1-MHB-L11	10	Topoisome	0.40776	0.001
**316**	1-MHB-L14	1	Topoisome	0.438928	0
**317**	1-MHB-M1:	100	Topoisome	0.366575	0.019
**318**	1-MHB-M1.	10	Topoisome	0.427116	0.001
**319**	1-MHB-N14	100	Topoisome	0.519563	0
**320**	1-MHB-O11	1000	Topoisome	0.364903	0.022
**321**	1-MHB-O14	1000	Topoisome	0.395142	0.005
**322**	1-MHB-P11	10000	Topoisome	0.400409	0.004
**323**	1-MHB-P14	10000	Topoisome	0.364192	0.015
**324**	3-MHB-A11	10000	Topoisome	0.426232	0
**325**	3-MHB-B11	1000	Topoisome	0.401354	0.003
**326**	3-MHB-C11	100	Topoisome	0.406425	0
**327**	3-MHB-D11	10	Topoisome	0.45585	0
**328**	3-MHB-E11	1	Topoisome	0.438481	0

**329**	3-MHB-G9-	1000	Topoisome	0.383395	0.016
**330**	3-MHB-G1(	10000	Topoisome	0.265032	0.038
**331**	3-MHB-H9-	100	Topoisome	0.467573	0
**332**	3-MHB-H1(	1000	Topoisome	0.574512	0
**333**	3-MHB-I9-[	10	Topoisome	0.540497	0
**334**	3-MHB-I10-	100	Topoisome	0.412736	0.009
**335**	3-MHB-J9-[	1	Topoisome	0.113788	0.678
**336**	3-MHB-J10	10	Topoisome	0.101787	0.603
**337**	3-MHB-K7-I	0.1	Topoisome	0.440393	0
**338**	3-MHB-K9-I	0.1	Topoisome	0.433721	0
**339**	3-MHB-K1(	1	Topoisome	0.456682	0.001
**340**	3-MHB-L6-I	0.1	Topoisome	0.404179	0.023
**341**	3-MHB-L7-I	1	Topoisome	0.13691	0.577
**342**	3-MHB-L9-\	0.5	Topoisome	0.205884	0.652
**343**	3-MHB-L10	0.1	Topoisome	0.496634	0
**344**	3-MHB-L16	1	Topoisome	0.51567	0
**345**	3-MHB-M6-	1	Topoisome	0.579091	0
**346**	3-MHB-M7-	10	Topoisome	0.494248	0
**347**	3-MHB-M9-	5	Topoisome	0.46116	0
**348**	3-MHB-M1(	1	Topoisome	0.316726	0.019
**349**	3-MHB-M1(	10	Topoisome	0.515408	0
**350**	3-MHB-N6-	10	Topoisome	0.440092	0.002
**351**	3-MHB-N9-	50	Topoisome	0.516874	0
**352**	3-MHB-N1(	10	Topoisome	0.488196	0
**353**	3-MHB-N16	100	Topoisome	0.116035	0.807
**354**	3-MHB-O6-	100	Topoisome	0.486531	0
**355**	3-MHB-O7-	100	Topoisome	0.475467	0
**356**	3-MHB-O9-	500	Topoisome	0.457446	0
**357**	3-MHB-O1(	100	Topoisome	0.507959	0
**358**	3-MHB-O16	1000	Topoisome	0.43997	0
**359**	3-MHB-P6-I	1000	Topoisome	0.338848	0.039
**360**	3-MHB-P7-I	1000	Topoisome	0.391388	0.009
**361**	3-MHB-P9-\	5000	Topoisome	0.414486	0
**362**	3-MHB-P1(	1000	Topoisome	0.376024	0.015
**363**	3-MHB-P16	10000	Topoisome	0.415275	0.001

**364**	1-MHB-A1C	10000	Mitotic	0.533971	0
**365**	1-MHB-A13	1000	Mitotic	0.529935	0
**366**	1-MHB-A18	1000	Mitotic	0.531363	0
**367**	1-MHB-B1C	1000	Mitotic	0.538579	0
**368**	1-MHB-B13	100	Mitotic	0.223902	0.156
**369**	1-MHB-B18	100	Mitotic	0.540443	0
**370**	1-MHB-C1C	100	Mitotic	0.518412	0
**371**	1-MHB-C13	10	Mitotic	0.100711	0.586
**372**	1-MHB-C18	10	Mitotic	0.50619	0
**373**	1-MHB-D1(	10	Mitotic	0.10316	0.993
**374**	1-MHB-D13	1	Mitotic	0.248954	0.354
**375**	1-MHB-D18	1	Mitotic	0.644078	0
**376**	1-MHB-E10	1	Mitotic	0.367971	0.005
**377**	1-MHB-E13	0.1	Mitotic	0.213	0.248
**378**	1-MHB-E18	0.1	Mitotic	0.14344	0.549
**379**	1-MHB-F13	1000	Mitotic	0.511592	0
**380**	1-MHB-G13	100	Mitotic	0.529583	0
**381**	1-MHB-G15	1000	Mitotic	0.518361	0
**382**	1-MHB-H13	10	Mitotic	0.497149	0
**383**	1-MHB-H15	100	Mitotic	0.511763	0
**384**	1-MHB-I13-	1	Mitotic	0.224036	0.165
**385**	1-MHB-I15-	10	Mitotic	0.534398	0
**386**	1-MHB-J13	0.1	Mitotic	0.489441	0
**387**	1-MHB-J15	1	Mitotic	0.499933	0
**388**	1-MHB-K7-	0.1	Mitotic	0.071585	0.861
**389**	1-MHB-K15	0.1	Mitotic	0.289953	0.046
**390**	1-MHB-L7-	1	Mitotic	0.555691	0
**391**	1-MHB-L20	0.1	Mitotic	0.052711	0.996
**392**	1-MHB-M7-	10	Mitotic	0.523472	0
**393**	1-MHB-M2(	1	Mitotic	0.158565	0.35
**394**	1-MHB-N2(	10	Mitotic	0.408842	0.018
**395**	1-MHB-O7-	100	Mitotic	0.53069	0
**396**	1-MHB-O2(	100	Mitotic	0.167423	0.37
**397**	1-MHB-P7-	1000	Mitotic	0.558654	0
**398**	1-MHB-P2C	1000	Mitotic	0.559225	0

**399**	3-MHB-A7-I	1000	Mitotic	0.412496	0.016
**400**	3-MHB-B7-I	100	Mitotic	0.455772	0.002
**401**	3-MHB-C7-I	10	Mitotic	0.470053	0.001
**402**	3-MHB-D7-	1	Mitotic	0.456652	0
**403**	3-MHB-E7-I	0.1	Mitotic	0.082258	0.994
**404**	6-MHB-L19	1	Mitotic	0.091076	0.773
**405**	6-MHB-M1	10	Mitotic	0.086665	0.803
**406**	6-MHB-N1	100	Mitotic	0.27598	0.789
**407**	6-MHB-O1	1000	Mitotic	0.461626	0
**408**	6-MHB-P1	10000	Mitotic	0.473091	0.001
**409**	2-MHB-A12	250	MEK1/2	0.658437	0
**410**	2-MHB-B12	25	MEK1/2	0.666425	0
**411**	2-MHB-D1	2.5	MEK1/2	0.687077	0
**412**	2-MHB-E12	0.25	MEK1/2	0.737951	0
**413**	2-MHB-F12	0.025	MEK1/2	0.692131	0
**414**	2-MHB-F14	1000	MEK1/2	0.607177	0
**415**	2-MHB-G1	100	MEK1/2	0.63386	0
**416**	2-MHB-H1	10	MEK1/2	0.64706	0
**417**	2-MHB-I14	1	MEK1/2	0.45734	0.057
**418**	2-MHB-K14	0.1	MEK1/2	0.692866	0
**419**	2-MHB-L20	1	MEK1/2	0.330486	0.093
**420**	2-MHB-M2	10	MEK1/2	0.658146	0
**421**	2-MHB-N2	100	MEK1/2	0.556445	0
**422**	2-MHB-O2	1000	MEK1/2	0.617323	0
**423**	2-MHB-P2	10000	MEK1/2	0.594888	0
**424**	4-MHB-A1	1000	MEK1/2	0.581864	0
**425**	4-MHB-A13	1000	MEK1/2	0.601694	0
**426**	4-MHB-B1	100	MEK1/2	0.694326	0
**427**	4-MHB-B13	100	MEK1/2	0.62545	0
**428**	4-MHB-C1	10	MEK1/2	0.779829	0
**429**	4-MHB-C13	10	MEK1/2	0.636331	0
**430**	4-MHB-D1	1	MEK1/2	0.661796	0
**431**	4-MHB-D13	1	MEK1/2	0.668647	0
**432**	4-MHB-E10	0.1	MEK1/2	0.669355	0
**433**	4-MHB-E13	0.1	MEK1/2	0.549688	0.001

**434**	4-MHB-L19	0.25	MEK1/2	0.602204	0
**435**	4-MHB-M1!	2.5	MEK1/2	0.08315	0.995
**436**	4-MHB-N1!	25	MEK1/2	0.574228	0
**437**	4-MHB-O1!	250	MEK1/2	0.670737	0
**438**	4-MHB-P1!	2500	MEK1/2	0.634501	0
**439**	1-MHB-L2-(	1	PARP	0.496601	0.061
**440**	1-MHB-L6-I	1	PARP	0.259731	0.417
**441**	1-MHB-M2-	10	PARP	0.101534	0.973
**442**	1-MHB-M6-	10	PARP	0.538555	0.004
**443**	1-MHB-N2-	100	PARP	0.566678	0
**444**	1-MHB-N6-	100	PARP	0.599757	0.007
**445**	1-MHB-O2-	1000	PARP	0.476214	0.014
**446**	1-MHB-O6-	1000	PARP	0.494924	0.073
**447**	1-MHB-P2-(	10000	PARP	0.384182	0.143
**448**	1-MHB-P6-I	10000	PARP	0.360273	0.319
**449**	7-MHB-A3-'	1000	PARP	0.487106	0.006
**450**	7-MHB-B2-'	10000	PARP	0.334096	0.097
**451**	7-MHB-B3-'	100	PARP	0.458507	0.027
**452**	7-MHB-C2-'	1000	PARP	0.162453	0.61
**453**	7-MHB-C3-'	10	PARP	0.350534	0.36
**454**	7-MHB-D2-	100	PARP	0.607553	0
**455**	7-MHB-D3-	1	PARP	0.652175	0
**456**	7-MHB-E2-\	10	PARP	0.561429	0.01
**457**	7-MHB-E3-'	0.1	PARP	0.610443	0
**458**	7-MHB-F2-\	1	PARP	0.530723	0.002
**459**	7-MHB-G2-	10000	PARP	0.487749	0.005
**460**	7-MHB-H2-	1000	PARP	0.595417	0
**461**	7-MHB-I2-N	100	PARP	0.444704	0.01
**462**	7-MHB-J2-N	10	PARP	0.628328	0
**463**	7-MHB-K2-I	1	PARP	0.371562	0.069
**464**	3-MHB-A1!	1000	CDK	0.271377	0.308
**465**	3-MHB-B1!	100	CDK	0.30759	0.164
**466**	3-MHB-B23	2500	CDK	0.293577	0.204
**467**	3-MHB-C1!	10	CDK	0.277232	0.297
**468**	3-MHB-C23	250	CDK	0.304816	0.186

**469**	3-MHB-D19	1	CDK	0.144584	0.459
**470**	3-MHB-D23	25	CDK	0.350058	0.054
**471**	3-MHB-E19	0.1	CDK	0.223628	0.55
**472**	3-MHB-E23	2.5	CDK	0.364387	0.076
**473**	3-MHB-F23	0.25	CDK	0.428045	0.003
**474**	3-MHB-K17	1	CDK	0.329258	0.095
**475**	3-MHB-L19	1	CDK	0.259204	0.258
**476**	3-MHB-M1	10	CDK	0.250101	0.298
**477**	3-MHB-M1	10	CDK	0.039393	0.939
**478**	3-MHB-N17	100	CDK	0.17713	0.687
**479**	3-MHB-N1	100	CDK	0.343705	0.037
**480**	3-MHB-O17	1000	CDK	0.331584	0.077
**481**	3-MHB-O1	1000	CDK	0.40759	0.009
**482**	3-MHB-P17	10000	CDK	0.236873	0.493
**483**	3-MHB-P1	10000	CDK	0.428063	0.014
**484**	4-MHB-A4-	10000	CDK	0.257808	0.388
**485**	4-MHB-A8-	10000	CDK	0.339944	0.058
**486**	4-MHB-B4-	1000	CDK	0.292005	0.215
**487**	4-MHB-B8-	1000	CDK	0.311744	0.201
**488**	4-MHB-C4-	100	CDK	0.238867	0.188
**489**	4-MHB-C8-	100	CDK	0.286654	0.172
**490**	4-MHB-D4-	10	CDK	0.252842	0.07
**491**	4-MHB-D8-	10	CDK	0.158894	0.869
**492**	4-MHB-E4-	1	CDK	0.198005	0.083
**493**	4-MHB-E8-	1	CDK	0.240616	0.104
**494**	4-MHB-F4-	10000	CDK	0.231369	0.482
**495**	4-MHB-F22	10000	CDK	0.327188	0.078
**496**	4-MHB-G4-	1000	CDK	0.256899	0.15
**497**	4-MHB-G2	1000	CDK	0.345987	0.04
**498**	4-MHB-H4-	100	CDK	0.240108	0.173
**499**	4-MHB-H2	100	CDK	0.313482	0.12
**500**	4-MHB-I4-S	10	CDK	0.26584	0.301
**501**	4-MHB-I22	10	CDK	0.087474	0.942
**502**	4-MHB-J4-	1	CDK	0.234678	0.357
**503**	4-MHB-J22	1	CDK	0.250188	0.52

**504**	5-MHB-A19	10000	CDK	0.310026	0.141
**505**	5-MHB-B19	1000	CDK	0.268131	0.314
**506**	5-MHB-C19	100	CDK	0.45407	0.015
**507**	5-MHB-D19	10	CDK	0.110408	0.519
**508**	5-MHB-E19	1	CDK	0.168921	0.42
**509**	5-MHB-K17	1	CDK	0.146131	0.799
**510**	5-MHB-M17	10	CDK	0.308136	0.141
**511**	5-MHB-N17	100	CDK	0.140851	0.564
**512**	5-MHB-O17	1000	CDK	0.280404	0.275
**513**	5-MHB-P17	10000	CDK	0.282972	0.256
**514**	6-MHB-A17	1000	CDK	0.261164	0.376
**515**	6-MHB-B17	100	CDK	0.25851	0.378
**516**	6-MHB-C17	10	CDK	0.147097	0.266
**517**	6-MHB-D17	1	CDK	0.138939	0.404
**518**	6-MHB-E17	0.1	CDK	0.149044	0.655
**519**	6-MHB-L15	1	CDK	0.143452	0.838
**520**	6-MHB-M15	10	CDK	0.070555	0.922
**521**	6-MHB-N15	100	CDK	0.246955	0.411
**522**	6-MHB-O15	1000	CDK	0.259115	0.439
**523**	6-MHB-P15	10000	CDK	0.290433	0.275
**524**	7-MHB-A21	10000	BET	0.567878	0
**525**	7-MHB-A22	30000	BET	0.601095	0
**526**	7-MHB-B21	1000	BET	0.559007	0
**527**	7-MHB-B22	3000	BET	0.529989	0
**528**	7-MHB-C21	100	BET	0.160555	0.263
**529**	7-MHB-C22	300	BET	0.212945	0.125
**530**	7-MHB-D21	10	BET	0.367881	0.04
**531**	7-MHB-D22	30	BET	0.164998	0.456
**532**	7-MHB-E21	1	BET	0.44163	0.005
**533**	7-MHB-E22	3	BET	0.177698	0.91
**534**	7-MHB-G10	10000	BET	0.577345	0
**535**	7-MHB-G15	10000	BET	0.581456	0
**536**	7-MHB-H10	1000	BET	0.575827	0
**537**	7-MHB-H15	1000	BET	0.589934	0
**538**	7-MHB-I10-	100	BET	0.518288	0

**539**	7-MHB-I15-	100	BET	0.213629	0.54
**540**	7-MHB-J10	10	BET	0.150201	0.228
**541**	7-MHB-J15	10	BET	0.440685	0.002
**542**	7-MHB-K10	1	BET	0.109361	0.58
**543**	7-MHB-K13	1	BET	0.588277	0
**544**	7-MHB-K15	1	BET	0.529015	0
**545**	7-MHB-L12	1	BET	0.401767	0.003
**546**	7-MHB-L13	10	BET	0.616131	0
**547**	7-MHB-L20	1	BET	0.473939	0
**548**	7-MHB-L23	0.03	BET	0.565962	0
**549**	7-MHB-M1	10	BET	0.240345	0.469
**550**	7-MHB-M1	100	BET	0.601389	0
**551**	7-MHB-M2	10	BET	0.147491	0.695
**552**	7-MHB-M2	0.3	BET	0.197891	0.149
**553**	7-MHB-N12	100	BET	0.095712	0.882
**554**	7-MHB-N13	1000	BET	0.51121	0
**555**	7-MHB-N20	100	BET	0.553944	0
**556**	7-MHB-N23	3	BET	0.222821	0.139
**557**	7-MHB-O12	1000	BET	0.572684	0
**558**	7-MHB-O20	1000	BET	0.56461	0
**559**	7-MHB-O23	30	BET	0.58787	0
**560**	7-MHB-P12	10000	BET	0.546064	0
**561**	7-MHB-P13	10000	BET	0.536998	0
**562**	7-MHB-P20	10000	BET	0.574178	0
**563**	7-MHB-P23	300	BET	0.615455	0
**564**	8-MHB-K22	1	BET	0.226626	0.447
**565**	8-MHB-L22	10	BET	0.209629	0.733
**566**	8-MHB-M2	100	BET	0.193814	0.846
**567**	8-MHB-N22	1000	BET	0.42524	0.004
**568**	8-MHB-O22	10000	BET	0.383032	0.031
**569**	1-MHB-A3-'	10000	HDAC	0.205428	0.581
**570**	1-MHB-B3-'	1000	HDAC	0.21797	0.339
**571**	1-MHB-C3-'	100	HDAC	0.107374	0.233
**572**	1-MHB-D3-	10	HDAC	0.219688	0.292
**573**	1-MHB-E3-\	1	HDAC	0.249581	0.293

**574**	1-MHB-L12	0.1	HDAC	0.156413	0.09
**575**	1-MHB-M1[illegible]	1	HDAC	0.149399	0.633
**576**	1-MHB-N12	10	HDAC	0.206037	0.609
**577**	1-MHB-O12	100	HDAC	0.102846	0.969
**578**	1-MHB-P12	1000	HDAC	0.228033	0.547
**579**	3-MHB-A4-	1000	HDAC	0.242259	0.583
**580**	3-MHB-B4-	100	HDAC	0.207135	0.641
**581**	3-MHB-C4-	10	HDAC	0.361215	0.01
**582**	3-MHB-D4-	1	HDAC	0.153361	0.122
**583**	3-MHB-E4-	0.1	HDAC	0.30051	0.026
**584**	3-MHB-F7-[illegible]	1000	HDAC	0.213304	0.566
**585**	3-MHB-G7-	100	HDAC	0.202664	0.61
**586**	3-MHB-G12	1000000	HDAC	0.088843	0.469
**587**	3-MHB-H7-	10	HDAC	0.300462	0.062
**588**	3-MHB-H12	100000	HDAC	0.250942	0.064
**589**	3-MHB-I7-[illegible]	1	HDAC	0.064375	0.655
**590**	3-MHB-I12-	10000	HDAC	0.125391	0.621
**591**	3-MHB-J7-[illegible]	0.1	HDAC	0.151074	0.304
**592**	3-MHB-J12	1000	HDAC	0.09387	0.562
**593**	3-MHB-K3-	1	HDAC	0.346612	0.019
**594**	3-MHB-K12	100	HDAC	0.19153	0.48
**595**	3-MHB-L3-	10	HDAC	0.200753	0.118
**596**	3-MHB-M3-	100	HDAC	0.191217	0.602
**597**	3-MHB-N3-	1000	HDAC	0.238409	0.335
**598**	3-MHB-O3-	10000	HDAC	0.217192	0.62
**599**	7-MHB-A5-	10000	HDAC	0.256664	0.296
**600**	7-MHB-A7-	10000	HDAC	0.273655	0.187
**601**	7-MHB-A9-	1000	HDAC	0.296814	0.082
**602**	7-MHB-A12	10000	HDAC	0.298034	0.049
**603**	7-MHB-B5-	1000	HDAC	0.325325	0.009
**604**	7-MHB-B12	1000	HDAC	0.379337	0
**605**	7-MHB-C5-	100	HDAC	0.044291	1
**606**	7-MHB-C7-	100	HDAC	0.335422	0.008
**607**	7-MHB-C9-	100	HDAC	0.388102	0
**608**	7-MHB-D7-	10	HDAC	0.325726	0.009

**609**	7-MHB-D9-	10	HDAC	0.158348	0.081
**610**	7-MHB-D12	100	HDAC	0.153154	0.672
**611**	7-MHB-E5-I	10	HDAC	0.267102	0
**612**	7-MHB-E7-(	1	HDAC	0.215146	0.007
**613**	7-MHB-E9-(	1	HDAC	0.423324	0
**614**	7-MHB-E12	10	HDAC	0.109759	0.213
**615**	7-MHB-F5-I	1	HDAC	0.312295	0.003
**616**	7-MHB-F7-I	10000	HDAC	0.331062	0.013
**617**	7-MHB-F9-(	0.1	HDAC	0.362185	0.001
**618**	7-MHB-F12	1	HDAC	0.332706	0.008
**619**	7-MHB-F19	10000	HDAC	0.344827	0.004
**620**	7-MHB-G7-	1000	HDAC	0.332436	0.012
**621**	7-MHB-G19	1000	HDAC	0.121715	0.237
**622**	7-MHB-H7-	100	HDAC	0.374859	0
**623**	7-MHB-I7-F	10	HDAC	0.359551	0.017
**624**	7-MHB-I19-	100	HDAC	0.122306	0.267
**625**	7-MHB-J7-F	1	HDAC	0.141317	0.124
**626**	7-MHB-J19	10	HDAC	0.270355	0.08
**627**	7-MHB-K4-I	1	HDAC	0.323895	0.014
**628**	7-MHB-K11	1	HDAC	0.317193	0.01
**629**	7-MHB-K18	1	HDAC	0.317642	0.01
**630**	7-MHB-K19	1	HDAC	0.106379	0.307
**631**	7-MHB-L2-T	0.1	HDAC	0.283182	0.009
**632**	7-MHB-L4-I	10	HDAC	0.347503	0.007
**633**	7-MHB-L5-I	1	HDAC	0.189965	0.035
**634**	7-MHB-L8-A	1	HDAC	0.408376	0
**635**	7-MHB-L10	1	HDAC	0.191051	0.463
**636**	7-MHB-L11	10	HDAC	0.356775	0.001
**637**	7-MHB-L14	1	HDAC	0.288842	0.028
**638**	7-MHB-L16	1	HDAC	0.174054	0.119
**639**	7-MHB-L18	10	HDAC	0.133908	0.097
**640**	7-MHB-M2-	1	HDAC	0.423079	0
**641**	7-MHB-M5-	10	HDAC	0.230916	0.045
**642**	7-MHB-M8-	10	HDAC	0.484774	0
**643**	7-MHB-M1(	10	HDAC	0.384719	0

**644**	7-MHB-M1	100	HDAC	0.411199	0
**645**	7-MHB-M1	10	HDAC	0.234026	0.008
**646**	7-MHB-M1	10	HDAC	0.297479	0.014
**647**	7-MHB-M1	100	HDAC	0.424908	0
**648**	7-MHB-N2-	10	HDAC	0.327383	0.036
**649**	7-MHB-N4-	100	HDAC	0.394393	0
**650**	7-MHB-N5-	100	HDAC	0.394362	0
**651**	7-MHB-N8-	100	HDAC	0.269073	0.083
**652**	7-MHB-N1	100	HDAC	0.364525	0.002
**653**	7-MHB-N1	100	HDAC	0.366169	0
**654**	7-MHB-N1	100	HDAC	0.324823	0.023
**655**	7-MHB-N1	1000	HDAC	0.125826	0.191
**656**	7-MHB-O2-	100	HDAC	0.394272	0.001
**657**	7-MHB-O4-	1000	HDAC	0.299272	0.054
**658**	7-MHB-O5-	1000	HDAC	0.349912	0.003
**659**	7-MHB-O8-	1000	HDAC	0.337066	0.006
**660**	7-MHB-O1	1000	HDAC	0.379349	0.001
**661**	7-MHB-O1	1000	HDAC	0.32104	0.014
**662**	7-MHB-O1	1000	HDAC	0.315091	0
**663**	7-MHB-O1	1000	HDAC	0.223377	0.288
**664**	7-MHB-P2-	1000	HDAC	0.376127	0.002
**665**	7-MHB-P4-	10000	HDAC	0.317658	0.018
**666**	7-MHB-P5-	10000	HDAC	0.283251	0.135
**667**	7-MHB-P8-	10000	HDAC	0.28407	0.159
**668**	7-MHB-P1	10000	HDAC	0.329486	0.017
**669**	7-MHB-P1	10000	HDAC	0.301808	0.075
**670**	7-MHB-P1	10000	HDAC	0.374555	0.02
**671**	7-MHB-P1	10000	HDAC	0.330873	0.054
**672**	7-MHB-P1	10000	HDAC	0.461576	0
**0**	2-O3B-A16	10000	EGFR	0.498809	0
**1**	2-O3B-A19	10000	EGFR	0.491526	0
**2**	2-O3B-B19	1000	EGFR	0.529767	0
**3**	2-O3B-C16	1000	EGFR	0.533453	0
**4**	2-O3B-C19	100	EGFR	0.508309	0
**5**	2-O3B-D16	100	EGFR	0.541172	0

**6**	2-O3B-D19	10	EGFR	0.286882	0.224
**7**	2-O3B-E16-	10	EGFR	0.48023	0
**8**	2-O3B-E19-	1	EGFR	0.072723	0.982
**9**	2-O3B-F16-	1	EGFR	0.425269	0.001
**10**	2-O3B-K11-	0.1	EGFR	0.534487	0
**11**	2-O3B-L11-	1	EGFR	0.443163	0
**12**	2-O3B-L16-	0.25	EGFR	0.353219	0.11
**13**	2-O3B-L19-	0.1	EGFR	0.475028	0
**14**	2-O3B-M11	10	EGFR	0.55591	0
**15**	2-O3B-M16	2.5	EGFR	0.337088	0.082
**16**	2-O3B-M19	1	EGFR	0.449501	0
**17**	2-O3B-N16	25	EGFR	0.50449	0
**18**	2-O3B-N19	10	EGFR	0.572298	0
**19**	2-O3B-O11	100	EGFR	0.529588	0
**20**	2-O3B-O16	250	EGFR	0.499148	0
**21**	2-O3B-O19	100	EGFR	0.556455	0
**22**	2-O3B-P11-	1000	EGFR	0.524897	0
**23**	2-O3B-P16-	2500	EGFR	0.496844	0
**24**	2-O3B-P19-	1000	EGFR	0.50606	0
**25**	3-O3B-F21-	10000	EGFR	0.537369	0
**26**	3-O3B-G20	1000	EGFR	0.488003	0
**27**	3-O3B-G21	1000	EGFR	0.471852	0
**28**	3-O3B-H20	100	EGFR	0.517097	0
**29**	3-O3B-H21	100	EGFR	0.12034	0.719
**30**	3-O3B-I20-I	10	EGFR	0.585246	0
**31**	3-O3B-I21-I	10	EGFR	0.595895	0
**32**	3-O3B-J20-	1	EGFR	0.111636	0.764
**33**	3-O3B-J21-	1	EGFR	0.067049	0.849
**34**	3-O3B-K4-C	1	EGFR	0.158379	0.91
**35**	3-O3B-K18-	0.1	EGFR	0.485156	0
**36**	3-O3B-K20-	0.1	EGFR	0.459792	0
**37**	3-O3B-L4-C	10	EGFR	0.444946	0
**38**	3-O3B-L18-	1	EGFR	0.037291	1
**39**	3-O3B-M18	10	EGFR	0.47876	0
**40**	3-O3B-N4-C	100	EGFR	0.394255	0

**41**	3-O3B-N18	100	EGFR	0.497622	0
**42**	3-O3B-O4-(	1000	EGFR	0.484083	0
**43**	3-O3B-P4-(	10000	EGFR	0.464056	0
**44**	3-O3B-P18·	1000	EGFR	0.471581	0
**45**	4-O3B-F13-	1000	EGFR	0.54731	0
**46**	4-O3B-G13	100	EGFR	0.579162	0
**47**	4-O3B-G16	10000	EGFR	0.531479	0
**48**	4-O3B-H13	10	EGFR	0.452342	0
**49**	4-O3B-H16	1000	EGFR	0.583952	0
**50**	4-O3B-I13-!	1	EGFR	0.062488	0.926
**51**	4-O3B-I16-'	100	EGFR	0.612776	0
**52**	4-O3B-J13-	0.1	EGFR	0.451685	0
**53**	4-O3B-J16-	10	EGFR	0.035926	0.979
**54**	4-O3B-K7-I	1	EGFR	0.547387	0
**55**	4-O3B-K13·	0.1	EGFR	0.370333	0.003
**56**	4-O3B-K16·	1	EGFR	0.487232	0
**57**	4-O3B-L7-I(	10	EGFR	0.51226	0
**58**	4-O3B-L13-	1	EGFR	0.518001	0
**59**	4-O3B-M7-I	100	EGFR	0.598083	0
**60**	4-O3B-M13	10	EGFR	0.58466	0
**61**	4-O3B-N13	100	EGFR	0.574692	0
**62**	4-O3B-O7-I	1000	EGFR	0.523209	0
**63**	4-O3B-P7-I(	10000	EGFR	0.581775	0
**64**	4-O3B-P13·	1000	EGFR	0.525322	0
**65**	5-O3B-F4-P	1000	EGFR	0.5019	0
**66**	5-O3B-F7-A	1000	EGFR	0.542035	0
**67**	5-O3B-G4-F	100	EGFR	0.515623	0
**68**	5-O3B-G7-/	100	EGFR	0.576745	0
**69**	5-O3B-H4-F	10	EGFR	0.556227	0
**70**	5-O3B-H7-/	10	EGFR	0.082837	0.798
**71**	5-O3B-I4-P(	1	EGFR	0.503885	0
**72**	5-O3B-I7-A:	1	EGFR	0.435097	0
**73**	5-O3B-J4-P	0.1	EGFR	0.447834	0
**74**	5-O3B-J7-A	0.1	EGFR	0.378352	0.005
**75**	5-O3B-K7-(	0.1	EGFR	0.402742	0

**76**	5-O3B-L7-C	1	EGFR	0.448446	0
**77**	5-O3B-M7-(	10	EGFR	0.475095	0
**78**	5-O3B-O7-(	100	EGFR	0.445621	0
**79**	5-O3B-P7-C	1000	EGFR	0.498928	0
**80**	2-O3B-A15-	2500	VEGFR	0.524138	0
**81**	2-O3B-A17-	10000	VEGFR	0.305202	0.097
**82**	2-O3B-A20-	10000	VEGFR	0.284273	0.167
**83**	2-O3B-B15-	250	VEGFR	0.624469	0
**84**	2-O3B-B17-	1000	VEGFR	0.339654	0.013
**85**	2-O3B-B20-	1000	VEGFR	0.541936	0
**86**	2-O3B-C15-	25	VEGFR	0.15555	0.669
**87**	2-O3B-C17-	100	VEGFR	0.446947	0
**88**	2-O3B-D15	2.5	VEGFR	0.409731	0
**89**	2-O3B-D17	10	VEGFR	0.444031	0
**90**	2-O3B-D20	100	VEGFR	0.478902	0
**91**	2-O3B-E17-	1	VEGFR	0.384267	0
**92**	2-O3B-E20-	10	VEGFR	0.446082	0
**93**	2-O3B-F13-	10000	VEGFR	0.277023	0.163
**94**	2-O3B-F15-	0.25	VEGFR	0.558611	0
**95**	2-O3B-F19-	10000	VEGFR	0.270716	0.294
**96**	2-O3B-F20-	1	VEGFR	0.468837	0
**97**	2-O3B-F21-	10000	VEGFR	0.280037	0.178
**98**	2-O3B-G10	10000	VEGFR	0.395602	0
**99**	2-O3B-G13	1000	VEGFR	0.49098	0
**100**	2-O3B-G19	1000	VEGFR	0.451117	0
**101**	2-O3B-G21	1000	VEGFR	0.434012	0
**102**	2-O3B-H10	1000	VEGFR	0.220711	0.074
**103**	2-O3B-H13	100	VEGFR	0.494319	0
**104**	2-O3B-H21	100	VEGFR	0.464118	0
**105**	2-O3B-I10-,	100	VEGFR	0.345851	0.008
**106**	2-O3B-I13-,	10	VEGFR	0.536767	0
**107**	2-O3B-I19-I	100	VEGFR	0.318555	0.054
**108**	2-O3B-I21-'	10	VEGFR	0.268531	0.008
**109**	2-O3B-J10-	10	VEGFR	0.416226	0
**110**	2-O3B-J13-	1	VEGFR	0.476173	0

**111**	2-O3B-J19-	10	VEGFR	0.225139	0.409
**112**	2-O3B-J21-	1	VEGFR	0.468366	0
**113**	2-O3B-K10·	1	VEGFR	0.479531	0
**114**	2-O3B-K13·	0.1	VEGFR	0.535247	0
**115**	2-O3B-K17·	1	VEGFR	0.230511	0.457
**116**	2-O3B-K19·	1	VEGFR	0.318826	0.022
**117**	2-O3B-L12-	0.1	VEGFR	0.500774	0
**118**	2-O3B-L13-	1	VEGFR	0.471576	0
**119**	2-O3B-L21-	0.1	VEGFR	0.379332	0.001
**120**	2-O3B-M12	1	VEGFR	0.446729	0
**121**	2-O3B-M13	10	VEGFR	0.384876	0
**122**	2-O3B-M17	10	VEGFR	0.39992	0
**123**	2-O3B-M21	1	VEGFR	0.288591	0.184
**124**	2-O3B-N12	10	VEGFR	0.06034	0.764
**125**	2-O3B-N13	100	VEGFR	0.378777	0
**126**	2-O3B-N17	100	VEGFR	0.365066	0
**127**	2-O3B-N21	10	VEGFR	0.131898	0.23
**128**	2-O3B-O12	100	VEGFR	0.135127	0.294
**129**	2-O3B-O17	1000	VEGFR	0.400285	0
**130**	2-O3B-O21	100	VEGFR	0.343112	0.015
**131**	2-O3B-P12·	1000	VEGFR	0.506255	0
**132**	2-O3B-P13·	1000	VEGFR	0.25381	0.339
**133**	2-O3B-P17·	10000	VEGFR	0.360679	0.003
**134**	2-O3B-P21·	1000	VEGFR	0.34364	0.002
**135**	3-O3B-A3-(	1000	VEGFR	0.322436	0.03
**136**	3-O3B-A6-F	1000	VEGFR	0.253136	0.329
**137**	3-O3B-A18·	1000	VEGFR	0.415773	0.001
**138**	3-O3B-B3-(	100	VEGFR	0.397182	0.001
**139**	3-O3B-B6-F	100	VEGFR	0.466873	0
**140**	3-O3B-B18·	100	VEGFR	0.41895	0
**141**	3-O3B-C3-(	10	VEGFR	0.178226	0.838
**142**	3-O3B-C6-F	10	VEGFR	0.439276	0
**143**	3-O3B-C18·	10	VEGFR	0.307929	0.08
**144**	3-O3B-D3-(	1	VEGFR	0.079762	0.691
**145**	3-O3B-D6-F	1	VEGFR	0.110643	0.403

**146**	3-O3B-D18	1	VEGFR	0.108339	0.323
**147**	3-O3B-E3-C	0.1	VEGFR	0.125338	0.617
**148**	3-O3B-E6-F	0.1	VEGFR	0.167638	0.156
**149**	3-O3B-E18-	0.1	VEGFR	0.278826	0.189
**150**	3-O3B-F18-	1000	VEGFR	0.117697	0.981
**151**	3-O3B-G18	100	VEGFR	0.15421	0.232
**152**	3-O3B-H18	10	VEGFR	0.092969	0.656
**153**	3-O3B-I18-I	1	VEGFR	0.112166	0.794
**154**	3-O3B-J18-	0.1	VEGFR	0.167911	0.841
**155**	4-O3B-A12-	10000	VEGFR	0.252012	0.438
**156**	4-O3B-A15-	2500	VEGFR	0.399828	0.002
**157**	4-O3B-A20-	10000	VEGFR	0.21213	0.624
**158**	4-O3B-B12-	1000	VEGFR	0.250568	0.38
**159**	4-O3B-B15-	250	VEGFR	0.162994	0.198
**160**	4-O3B-B20-	1000	VEGFR	0.352377	0.002
**161**	4-O3B-C15-	25	VEGFR	0.170954	0.661
**162**	4-O3B-D12	100	VEGFR	0.281971	0.089
**163**	4-O3B-D15	2.5	VEGFR	0.096165	0.946
**164**	4-O3B-D20	100	VEGFR	0.319374	0.105
**165**	4-O3B-E12-	10	VEGFR	0.188491	0.7
**166**	4-O3B-E20-	10	VEGFR	0.281124	0.179
**167**	4-O3B-F12-	1	VEGFR	0.042013	0.966
**168**	4-O3B-F15-	0.25	VEGFR	0.105082	0.97
**169**	4-O3B-F20-	1	VEGFR	0.051025	0.84
**170**	4-O3B-L16-	1	VEGFR	0.320931	0.011
**171**	4-O3B-M16	10	VEGFR	0.166287	0.714
**172**	4-O3B-N16	100	VEGFR	0.338332	0.003
**173**	4-O3B-O16	1000	VEGFR	0.450795	0
**174**	4-O3B-P16-	10000	VEGFR	0.337087	0.012
**175**	2-O3B-L10-	1	PI3K	0.045183	0.989
**176**	2-O3B-M10	10	PI3K	0.332698	0.005
**177**	2-O3B-N10	100	PI3K	0.378452	0
**178**	2-O3B-O10	1000	PI3K	0.370205	0
**179**	2-O3B-P10-	10000	PI3K	0.37606	0.002
**180**	3-O3B-A16-	2500	PI3K	0.04423	0.959

**181**	3-O3B-C16-	250	PI3K	0.366219	0.005
**182**	3-O3B-D16	25	PI3K	0.023161	0.98
**183**	3-O3B-E16-	2.5	PI3K	0.024423	0.976
**184**	3-O3B-F16-	0.25	PI3K	0.361386	0
**185**	3-O3B-F17-	100000	PI3K	0.366093	0
**186**	3-O3B-F19-	500	PI3K	0.396077	0
**187**	3-O3B-G17	10000	PI3K	0.028727	0.976
**188**	3-O3B-G19	50	PI3K	0.364316	0
**189**	3-O3B-H17	1000	PI3K	0.087933	0.894
**190**	3-O3B-I17-I	100	PI3K	0.155283	0.49
**191**	3-O3B-I19-I	5	PI3K	0.417108	0
**192**	3-O3B-J17-	10	PI3K	0.315437	0.015
**193**	3-O3B-J19-	0.5	PI3K	0.064268	0.892
**194**	3-O3B-K19-	0.05	PI3K	0.112582	0.899
**195**	3-O3B-L8-P	1	PI3K	0.043485	0.931
**196**	3-O3B-L21-	0.1	PI3K	0.040441	0.956
**197**	3-O3B-M8-I	10	PI3K	0.428986	0
**198**	3-O3B-M21	1	PI3K	0.325402	0.007
**199**	3-O3B-N8-F	100	PI3K	0.336335	0.007
**200**	3-O3B-N21	10	PI3K	0.07387	0.999
**201**	3-O3B-O8-F	1000	PI3K	0.382819	0
**202**	3-O3B-O21	100	PI3K	0.34606	0.002
**203**	3-O3B-P8-F	10000	PI3K	0.371458	0.001
**204**	3-O3B-P21-	1000	PI3K	0.337491	0.005
**205**	4-O3B-A19-	2500	PI3K	0.381602	0
**206**	4-O3B-B19-	250	PI3K	0.279907	0.074
**207**	4-O3B-C19-	25	PI3K	0.381072	0
**208**	4-O3B-D19	2.5	PI3K	0.346499	0.006
**209**	4-O3B-E19-	0.25	PI3K	0.349625	0.011
**210**	4-O3B-F14-	1000	PI3K	0.380803	0
**211**	4-O3B-G2-T	2500	PI3K	0.255951	0.304
**212**	4-O3B-G5-S	10000	PI3K	0.298353	0.043
**213**	4-O3B-G14	100	PI3K	0.380449	0
**214**	4-O3B-G20	10000	PI3K	0.37789	0.001
**215**	4-O3B-H2-T	250	PI3K	0.069647	0.699

**216**	4-O3B-H5-S	1000	PI3K	0.383554	0
**217**	4-O3B-H14	10	PI3K	0.394277	0
**218**	4-O3B-H20	1000	PI3K	0.374269	0.001
**219**	4-O3B-I2-T(	25	PI3K	0.266744	0.486
**220**	4-O3B-I5-S(	100	PI3K	0.29996	0.059
**221**	4-O3B-I14-I	1	PI3K	0.356363	0.001
**222**	4-O3B-I20-I	100	PI3K	0.146893	0.301
**223**	4-O3B-J2-T(	2.5	PI3K	0.016874	1
**224**	4-O3B-J5-S(	10	PI3K	0.279553	0.195
**225**	4-O3B-J20-	10	PI3K	0.067704	0.772
**226**	4-O3B-K2-T	0.25	PI3K	0.2227	0.845
**227**	4-O3B-K4-D	0.1	PI3K	0.376809	0
**228**	4-O3B-K5-S	1	PI3K	0.389799	0
**229**	4-O3B-K14-	0.1	PI3K	0.241659	0.311
**230**	4-O3B-K20-	1	PI3K	0.305903	0.013
**231**	4-O3B-L4-D	1	PI3K	0.386791	0
**232**	4-O3B-L14-	0.1	PI3K	0.378906	0.001
**233**	4-O3B-L15-	1	PI3K	0.049702	0.95
**234**	4-O3B-L21-	0.1	PI3K	0.298273	0.031
**235**	4-O3B-M14	1	PI3K	0.204958	0.278
**236**	4-O3B-M15	10	PI3K	0.421572	0
**237**	4-O3B-M21	1	PI3K	0.103542	0.712
**238**	4-O3B-N4-D	10	PI3K	0.39094	0
**239**	4-O3B-N14	10	PI3K	0.40041	0
**240**	4-O3B-N15	100	PI3K	0.259911	0.094
**241**	4-O3B-N21	10	PI3K	0.365081	0
**242**	4-O3B-O4-D	100	PI3K	0.380939	0
**243**	4-O3B-O14	100	PI3K	0.382894	0
**244**	4-O3B-O15	1000	PI3K	0.391817	0
**245**	4-O3B-O21	100	PI3K	0.394522	0
**246**	4-O3B-P4-D	1000	PI3K	0.374288	0.001
**247**	4-O3B-P14-	1000	PI3K	0.379832	0
**248**	4-O3B-P15-	10000	PI3K	0.38634	0
**249**	4-O3B-P21-	1000	PI3K	0.372203	0
**250**	5-O3B-A6-L	2500	PI3K	0.384531	0.001

**251**	5-O3B-A7-A	1000	PI3K	0.370535	0.002
**252**	5-O3B-A16-	2500	PI3K	0.410408	0
**253**	5-O3B-A17-	10000	PI3K	0.413932	0
**254**	5-O3B-B6-L	250	PI3K	0.404996	0
**255**	5-O3B-B7-A	100	PI3K	0.035547	0.985
**256**	5-O3B-B17-	1000	PI3K	0.434928	0
**257**	5-O3B-C6-L	25	PI3K	0.375674	0.002
**258**	5-O3B-C7-A	10	PI3K	0.426259	0
**259**	5-O3B-C16-	250	PI3K	0.361592	0
**260**	5-O3B-C17-	100	PI3K	0.404716	0
**261**	5-O3B-D6-L	2.5	PI3K	0.398321	0.001
**262**	5-O3B-D7-A	1	PI3K	0.409599	0
**263**	5-O3B-D16	25	PI3K	0.417814	0
**264**	5-O3B-D17	10	PI3K	0.412653	0
**265**	5-O3B-E6-L	0.25	PI3K	0.049768	0.88
**266**	5-O3B-E7-A	0.1	PI3K	0.136423	0.465
**267**	5-O3B-E16-	2.5	PI3K	0.349575	0.001
**268**	5-O3B-E17-	1	PI3K	0.408682	0
**269**	5-O3B-F11-	10000	PI3K	0.406253	0
**270**	5-O3B-F16-	0.25	PI3K	0.254092	0.082
**271**	5-O3B-G9-S	10000	PI3K	0.396951	0
**272**	5-O3B-G11	1000	PI3K	0.405908	0
**273**	5-O3B-H9-S	1000	PI3K	0.401485	0
**274**	5-O3B-H11	100	PI3K	0.418262	0
**275**	5-O3B-I9-Se	100	PI3K	0.094206	0.947
**276**	5-O3B-I11-	10	PI3K	0.311117	0.023
**277**	5-O3B-J9-S	10	PI3K	0.405677	0
**278**	5-O3B-J11-	1	PI3K	0.08747	0.919
**279**	5-O3B-K9-S	1	PI3K	0.349596	0
**280**	5-O3B-L14-	0.1	PI3K	0.4042	0
**281**	5-O3B-L20-	1	PI3K	0.415426	0
**282**	5-O3B-L23-	0.1	PI3K	0.267575	0.145
**283**	5-O3B-M14	1	PI3K	0.410677	0
**284**	5-O3B-M2C	10	PI3K	0.437085	0
**285**	5-O3B-M23	1	PI3K	0.403937	0

286	5-O3B-N14	10	PI3K	0.394527	0
287	5-O3B-N20	100	PI3K	0.415205	0
288	5-O3B-N23	10	PI3K	0.419129	0
289	5-O3B-O14	100	PI3K	0.399626	0
290	5-O3B-O20	1000	PI3K	0.402695	0
291	5-O3B-O23	100	PI3K	0.381574	0
292	5-O3B-P14	1000	PI3K	0.412272	0
293	5-O3B-P20	10000	PI3K	0.381675	0
294	5-O3B-P23	1000	PI3K	0.40272	0
295	6-O3B-A8-T	10000	PI3K	0.37167	0
296	6-O3B-B8-T	1000	PI3K	0.371838	0.001
297	6-O3B-C8-T	100	PI3K	0.382626	0
298	6-O3B-D8-T	10	PI3K	0.348693	0.003
299	6-O3B-E8-T	1	PI3K	0.019934	1
300	6-O3B-L6-G	1	PI3K	0.164503	0.818
301	6-O3B-M6-	10	PI3K	0.306229	0.041
302	6-O3B-N6-(	100	PI3K	0.291467	0.038
303	6-O3B-O6-(	1000	PI3K	0.377396	0
304	6-O3B-P6-G	10000	PI3K	0.370223	0
305	1-O3B-F11-	10000	Topoisome	0.435109	0.002
306	1-O3B-G11	1000	Topoisome	0.625128	0
307	1-O3B-G20	1000	Topoisome	0.435857	0.003
308	1-O3B-H11	100	Topoisome	0.64795	0
309	1-O3B-H20	100	Topoisome	0.617426	0
310	1-O3B-I11-	10	Topoisome	0.340746	0.1
311	1-O3B-I20-	10	Topoisome	0.365363	0.097
312	1-O3B-J11-	1	Topoisome	0.166774	0.394
313	1-O3B-J20-	1	Topoisome	0.145704	0.739
314	1-O3B-K11	1	Topoisome	0.587355	0
315	1-O3B-K20	0.1	Topoisome	0.231557	0.717
316	1-O3B-L11-	10	Topoisome	0.577204	0
317	1-O3B-L14-	1	Topoisome	0.313475	0.107
318	1-O3B-M11	100	Topoisome	0.57983	0
319	1-O3B-M14	10	Topoisome	0.588716	0
320	1-O3B-N14	100	Topoisome	0.612753	0

**321**	1-O3B-O11	1000	Topoisome	0.426948	0.002
**322**	1-O3B-O14	1000	Topoisome	0.564325	0
**323**	1-O3B-P11-	10000	Topoisome	0.431132	0.001
**324**	1-O3B-P14-	10000	Topoisome	0.423674	0.002
**325**	3-O3B-A11-	10000	Topoisome	0.571898	0
**326**	3-O3B-B11-	1000	Topoisome	0.61761	0
**327**	3-O3B-C11-	100	Topoisome	0.566724	0
**328**	3-O3B-D11	10	Topoisome	0.45812	0.003
**329**	3-O3B-E11-	1	Topoisome	0.264114	0.053
**330**	3-O3B-G9-D	1000	Topoisome	0.444838	0.001
**331**	3-O3B-G10	10000	Topoisome	0.487634	0.004
**332**	3-O3B-H9-D	100	Topoisome	0.576604	0
**333**	3-O3B-H10	1000	Topoisome	0.115607	0.585
**334**	3-O3B-I9-D	10	Topoisome	0.486435	0
**335**	3-O3B-I10-	100	Topoisome	0.139336	0.5
**336**	3-O3B-J9-D	1	Topoisome	0.170554	0.622
**337**	3-O3B-J10-	10	Topoisome	0.138835	0.536
**338**	3-O3B-K7-I	0.1	Topoisome	0.165678	0.481
**339**	3-O3B-K9-D	0.1	Topoisome	0.470882	0.001
**340**	3-O3B-K10-	1	Topoisome	0.072183	0.761
**341**	3-O3B-L6-D	0.1	Topoisome	0.074349	0.782
**342**	3-O3B-L7-I	1	Topoisome	0.408777	0.059
**343**	3-O3B-L9-V	0.5	Topoisome	0.361825	0.042
**344**	3-O3B-L10-	0.1	Topoisome	0.075114	0.767
**345**	3-O3B-L16-	1	Topoisome	0.35097	0.066
**346**	3-O3B-M6-I	1	Topoisome	0.205864	0.305
**347**	3-O3B-M7-I	10	Topoisome	0.558897	0
**348**	3-O3B-M9-	5	Topoisome	0.335721	0.144
**349**	3-O3B-M10	1	Topoisome	0.431545	0.011
**350**	3-O3B-M16	10	Topoisome	0.413781	0
**351**	3-O3B-N6-D	10	Topoisome	0.413308	0.001
**352**	3-O3B-N9-V	50	Topoisome	0.578662	0
**353**	3-O3B-N10	10	Topoisome	0.527477	0
**354**	3-O3B-N16	100	Topoisome	0.221019	0.38
**355**	3-O3B-O6-D	100	Topoisome	0.584889	0

**356**	3-O3B-O7-I	100	Topoisome	0.633328	0
**357**	3-O3B-O9-\	500	Topoisome	0.600775	0
**358**	3-O3B-O10	100	Topoisome	0.624153	0
**359**	3-O3B-O16	1000	Topoisome	0.452873	0
**360**	3-O3B-P6-D	1000	Topoisome	0.439712	0.003
**361**	3-O3B-P7-I	1000	Topoisome	0.439156	0
**362**	3-O3B-P9-V	5000	Topoisome	0.443452	0
**363**	3-O3B-P10-	1000	Topoisome	0.429922	0.002
**364**	3-O3B-P16-	10000	Topoisome	0.40229	0.001
**365**	1-O3B-A10-	10000	Mitotic	0.599819	0
**366**	1-O3B-A13-	1000	Mitotic	0.602566	0
**367**	1-O3B-A18-	1000	Mitotic	0.59323	0
**368**	1-O3B-B10-	1000	Mitotic	0.59095	0
**369**	1-O3B-B13-	100	Mitotic	0.037254	1
**370**	1-O3B-B18-	100	Mitotic	0.581408	0
**371**	1-O3B-C10-	100	Mitotic	0.594252	0
**372**	1-O3B-C13-	10	Mitotic	0.430659	0.01
**373**	1-O3B-C18-	10	Mitotic	0.59639	0
**374**	1-O3B-D10	10	Mitotic	0.620087	0
**375**	1-O3B-D13	1	Mitotic	0.167737	0.533
**376**	1-O3B-D18	1	Mitotic	0.598062	0
**377**	1-O3B-E10-	1	Mitotic	0.383005	0.045
**378**	1-O3B-E13-	0.1	Mitotic	0.491883	0.007
**379**	1-O3B-E18-	0.1	Mitotic	0.52598	0
**380**	1-O3B-F13-	1000	Mitotic	0.596304	0
**381**	1-O3B-G13	100	Mitotic	0.555194	0
**382**	1-O3B-G15	1000	Mitotic	0.602855	0
**383**	1-O3B-H13	10	Mitotic	0.215566	0.823
**384**	1-O3B-H15	100	Mitotic	0.597221	0
**385**	1-O3B-I13-'	1	Mitotic	0.521329	0
**386**	1-O3B-I15-I	10	Mitotic	0.580903	0
**387**	1-O3B-J13-	0.1	Mitotic	0.321607	0.282
**388**	1-O3B-J15-	1	Mitotic	0.596158	0
**389**	1-O3B-K7-V	0.1	Mitotic	0.116799	0.912
**390**	1-O3B-K15-	0.1	Mitotic	0.593175	0

**391**	1-O3B-L7-V	1	Mitotic	0.377074	0.033
**392**	1-O3B-L20-	0.1	Mitotic	0.112116	0.814
**393**	1-O3B-M7-'	10	Mitotic	0.564411	0
**394**	1-O3B-M2C	1	Mitotic	0.552268	0
**395**	1-O3B-N20	10	Mitotic	0.288083	0.084
**396**	1-O3B-O7-\	100	Mitotic	0.596739	0
**397**	1-O3B-O20	100	Mitotic	0.613388	0
**398**	1-O3B-P7-\	1000	Mitotic	0.593051	0
**399**	1-O3B-P20-	1000	Mitotic	0.58212	0
**400**	3-O3B-A7-D	1000	Mitotic	0.50567	0
**401**	3-O3B-B7-D	100	Mitotic	0.507322	0
**402**	3-O3B-C7-D	10	Mitotic	0.491088	0
**403**	3-O3B-D7-D	1	Mitotic	0.576383	0
**404**	3-O3B-E7-D	0.1	Mitotic	0.044958	1
**405**	6-O3B-L19-	1	Mitotic	0.580659	0
**406**	6-O3B-M19	10	Mitotic	0.620261	0
**407**	6-O3B-N19	100	Mitotic	0.056215	1
**408**	6-O3B-O19	1000	Mitotic	0.546355	0
**409**	6-O3B-P19-	10000	Mitotic	0.576376	0
**410**	2-O3B-A12-	250	MEK1/2	0.596501	0
**411**	2-O3B-B12-	25	MEK1/2	0.647857	0
**412**	2-O3B-D12	2.5	MEK1/2	0.659039	0
**413**	2-O3B-E12-	0.25	MEK1/2	0.727481	0
**414**	2-O3B-F12-	2.5000000	MEK1/2	0.640898	0
**415**	2-O3B-F14-	1000	MEK1/2	0.583106	0
**416**	2-O3B-G14	100	MEK1/2	0.675977	0
**417**	2-O3B-H14	10	MEK1/2	0.656966	0
**418**	2-O3B-I14-	1	MEK1/2	0.602327	0
**419**	2-O3B-K14-	0.1	MEK1/2	0.568738	0
**420**	2-O3B-L20-	1	MEK1/2	0.436894	0.204
**421**	2-O3B-M2C	10	MEK1/2	0.649392	0
**422**	2-O3B-N20	100	MEK1/2	0.63106	0
**423**	2-O3B-O20	1000	MEK1/2	0.606059	0
**424**	2-O3B-P20-	10000	MEK1/2	0.627728	0
**425**	4-O3B-A10-	1000	MEK1/2	0.622247	0

**426**	4-O3B-A13-	1000	MEK1/2	0.62981	0
**427**	4-O3B-B10-	100	MEK1/2	0.739049	0
**428**	4-O3B-B13-	100	MEK1/2	0.596912	0
**429**	4-O3B-C10-	10	MEK1/2	0.721497	0
**430**	4-O3B-C13-	10	MEK1/2	0.613331	0
**431**	4-O3B-D10	1	MEK1/2	0.254256	0.512
**432**	4-O3B-D13	1	MEK1/2	0.721852	0
**433**	4-O3B-E10-	0.1	MEK1/2	0.151856	0.733
**434**	4-O3B-E13-	0.1	MEK1/2	0.557704	0
**435**	4-O3B-L19-	0.25	MEK1/2	0.520492	0.002
**436**	4-O3B-M19	2.5	MEK1/2	0.679979	0
**437**	4-O3B-N19	25	MEK1/2	0.604173	0
**438**	4-O3B-O19	250	MEK1/2	0.62645	0
**439**	4-O3B-P19-	2500	MEK1/2	0.71847	0
**440**	1-O3B-L2-C	1	PARP	0.504776	0.057
**441**	1-O3B-L6-R	1	PARP	0.417415	0.077
**442**	1-O3B-M2-	10	PARP	0.489536	0.01
**443**	1-O3B-M6-	10	PARP	0.407384	0.098
**444**	1-O3B-N2-(	100	PARP	0.723817	0
**445**	1-O3B-N6-F	100	PARP	0.787382	0
**446**	1-O3B-O2-(	1000	PARP	0.536052	0.004
**447**	1-O3B-O6-F	1000	PARP	0.577757	0
**448**	1-O3B-P2-C	10000	PARP	0.504994	0.005
**449**	1-O3B-P6-F	10000	PARP	0.551872	0
**450**	7-O3B-A3-T	1000	PARP	0.512833	0.002
**451**	7-O3B-B2-V	10000	PARP	0.592145	0
**452**	7-O3B-B3-T	100	PARP	0.499862	0.009
**453**	7-O3B-C2-V	1000	PARP	0.560411	0.006
**454**	7-O3B-C3-T	10	PARP	0.676204	0
**455**	7-O3B-D2-V	100	PARP	0.162377	0.758
**456**	7-O3B-D3-T	1	PARP	0.768171	0
**457**	7-O3B-E2-V	10	PARP	0.598213	0.001
**458**	7-O3B-E3-T	0.1	PARP	0.482965	0.066
**459**	7-O3B-F2-V	1	PARP	0.159257	0.553
**460**	7-O3B-G2-N	10000	PARP	0.600673	0

**461**	7-O3B-H2-N	1000	PARP	0.516869	0.005
**462**	7-O3B-I2-N	100	PARP	0.758694	0
**463**	7-O3B-J2-N	10	PARP	0.545957	0.003
**464**	7-O3B-K2-N	1	PARP	0.451522	0.086
**465**	3-O3B-A19-	1000	CDK	0.429146	0.002
**466**	3-O3B-B19-	100	CDK	0.418482	0.001
**467**	3-O3B-B23-	2500	CDK	0.408691	0.003
**468**	3-O3B-C19-	10	CDK	0.416774	0.001
**469**	3-O3B-C23-	250	CDK	0.418673	0.001
**470**	3-O3B-D19	1	CDK	0.09816	0.798
**471**	3-O3B-D23	25	CDK	0.412592	0.002
**472**	3-O3B-E19-	0.1	CDK	0.083597	0.861
**473**	3-O3B-E23-	2.5	CDK	0.136625	0.834
**474**	3-O3B-F23-	0.25	CDK	0.3643	0.113
**475**	3-O3B-K17-	1	CDK	0.09008	0.934
**476**	3-O3B-L19-	1	CDK	0.154595	0.754
**477**	3-O3B-M17	10	CDK	0.252073	0.292
**478**	3-O3B-M19	10	CDK	0.189583	0.811
**479**	3-O3B-N17	100	CDK	0.355107	0.022
**480**	3-O3B-N19	100	CDK	0.392728	0.031
**481**	3-O3B-O17	1000	CDK	0.419117	0.002
**482**	3-O3B-O19	1000	CDK	0.41644	0
**483**	3-O3B-P17-	10000	CDK	0.356673	0.022
**484**	3-O3B-P19-	10000	CDK	0.394055	0.011
**485**	4-O3B-A4-S	10000	CDK	0.443505	0
**486**	4-O3B-A8-N	10000	CDK	0.438356	0.001
**487**	4-O3B-B4-S	1000	CDK	0.426214	0.002
**488**	4-O3B-B8-N	1000	CDK	0.42797	0.003
**489**	4-O3B-C4-S	100	CDK	0.445748	0
**490**	4-O3B-C8-N	100	CDK	0.322936	0.05
**491**	4-O3B-D4-S	10	CDK	0.395255	0.082
**492**	4-O3B-D8-N	10	CDK	0.357089	0.066
**493**	4-O3B-E4-S	1	CDK	0.211585	0.523
**494**	4-O3B-E8-N	1	CDK	0.371229	0.007
**495**	4-O3B-F4-S	10000	CDK	0.420687	0.002

**496**	4-O3B-F22-	10000	CDK	0.44491	0.001
**497**	4-O3B-G4-S	1000	CDK	0.317256	0.08
**498**	4-O3B-G22	1000	CDK	0.426637	0.004
**499**	4-O3B-H4-S	100	CDK	0.314155	0.144
**500**	4-O3B-H22	100	CDK	0.439008	0
**501**	4-O3B-I4-Se	10	CDK	0.399421	0.007
**502**	4-O3B-I22-,	10	CDK	0.410023	0.073
**503**	4-O3B-J4-S	1	CDK	0.224589	0.681
**504**	4-O3B-J22-	1	CDK	0.392523	0.066
**505**	5-O3B-A19-	10000	CDK	0.417128	0.001
**506**	5-O3B-B19-	1000	CDK	0.423532	0.001
**507**	5-O3B-C19-	100	CDK	0.400702	0.009
**508**	5-O3B-D19	10	CDK	0.037716	0.995
**509**	5-O3B-E19-	1	CDK	0.424732	0.002
**510**	5-O3B-K17-	1	CDK	0.418722	0.004
**511**	5-O3B-M17	10	CDK	0.179075	0.963
**512**	5-O3B-N17	100	CDK	0.403181	0.003
**513**	5-O3B-O17	1000	CDK	0.431092	0
**514**	5-O3B-P17-	10000	CDK	0.431715	0.002
**515**	6-O3B-A17-	1000	CDK	0.269455	0.197
**516**	6-O3B-B17-	100	CDK	0.450228	0.001
**517**	6-O3B-C17-	10	CDK	0.437788	0.001
**518**	6-O3B-D17	1	CDK	0.038844	0.984
**519**	6-O3B-E17-	0.1	CDK	0.396153	0.019
**520**	6-O3B-L15-	1	CDK	0.077922	0.989
**521**	6-O3B-M15	10	CDK	0.363516	0.093
**522**	6-O3B-N15	100	CDK	0.38887	0.016
**523**	6-O3B-O15	1000	CDK	0.418912	0.005
**524**	6-O3B-P15-	10000	CDK	0.414833	0.005
**525**	7-O3B-A21-	10000	BET	0.549606	0
**526**	7-O3B-A22-	30000	BET	0.63154	0
**527**	7-O3B-B21-	1000	BET	0.599755	0
**528**	7-O3B-B22-	3000	BET	0.488163	0
**529**	7-O3B-C21-	100	BET	0.32377	0.072
**530**	7-O3B-C22-	300	BET	0.222613	0.406

**531**	7-O3B-D21	10	BET	0.092728	0.706
**532**	7-O3B-D22	30	BET	0.291311	0.25
**533**	7-O3B-E21-	1	BET	0.090743	0.818
**534**	7-O3B-E22-	3	BET	0.15028	0.653
**535**	7-O3B-G10	10000	BET	0.560103	0
**536**	7-O3B-G15	10000	BET	0.57737	0
**537**	7-O3B-H10	1000	BET	0.60441	0
**538**	7-O3B-H15	1000	BET	0.614305	0
**539**	7-O3B-I10-I	100	BET	0.642241	0
**540**	7-O3B-I15-I	100	BET	0.584004	0
**541**	7-O3B-J10-	10	BET	0.506654	0.002
**542**	7-O3B-J15-	10	BET	0.361574	0.15
**543**	7-O3B-K10-	1	BET	0.094397	0.806
**544**	7-O3B-K13-	1	BET	0.400808	0.02
**545**	7-O3B-K15-	1	BET	0.351308	0.056
**546**	7-O3B-L12-	1	BET	0.492733	0
**547**	7-O3B-L13-	10	BET	0.685981	0
**548**	7-O3B-L20-	1	BET	0.390679	0.033
**549**	7-O3B-L23-	0.03	BET	0.147014	0.591
**550**	7-O3B-M12	10	BET	0.641024	0
**551**	7-O3B-M13	100	BET	0.674938	0
**552**	7-O3B-M2C	10	BET	0.609888	0
**553**	7-O3B-M23	0.3	BET	0.331547	0.199
**554**	7-O3B-N12	100	BET	0.337145	0.058
**555**	7-O3B-N13	1000	BET	0.561377	0
**556**	7-O3B-N20	100	BET	0.701322	0
**557**	7-O3B-N23	3	BET	0.496552	0.006
**558**	7-O3B-O12	1000	BET	0.645654	0
**559**	7-O3B-O20	1000	BET	0.511808	0.001
**560**	7-O3B-O23	30	BET	0.668191	0
**561**	7-O3B-P12-	10000	BET	0.595161	0
**562**	7-O3B-P13-	10000	BET	0.498808	0
**563**	7-O3B-P20-	10000	BET	0.563334	0
**564**	7-O3B-P23-	300	BET	0.6294	0
**565**	8-O3B-K22-	1	BET	0.185597	0.853

**566**	8-O3B-L22-	10	BET	0.297702	0.27
**567**	8-O3B-M22	100	BET	0.303131	0.277
**568**	8-O3B-N22	1000	BET	0.321579	0.125
**569**	8-O3B-O22	10000	BET	0.248007	0.195
**570**	1-O3B-A3-\	10000	HDAC	0.348179	0.003
**571**	1-O3B-B3-\	1000	HDAC	0.351012	0.006
**572**	1-O3B-C3-\	100	HDAC	0.320241	0.035
**573**	1-O3B-D3-\	10	HDAC	0.081412	0.992
**574**	1-O3B-E3-V	1	HDAC	0.252144	0.36
**575**	1-O3B-L12-	0.1	HDAC	0.440478	0
**576**	1-O3B-M12	1	HDAC	0.517661	0
**577**	1-O3B-N12	10	HDAC	0.35752	0.004
**578**	1-O3B-O12	100	HDAC	0.341797	0.015
**579**	1-O3B-P12-	1000	HDAC	0.362747	0.001
**580**	3-O3B-A4-F	1000	HDAC	0.342792	0.014
**581**	3-O3B-B4-F	100	HDAC	0.336819	0.017
**582**	3-O3B-C4-F	10	HDAC	0.465924	0
**583**	3-O3B-D4-F	1	HDAC	0.315289	0.031
**584**	3-O3B-E4-P	0.1	HDAC	0.358259	0.03
**585**	3-O3B-F7-C	1000	HDAC	0.336127	0.017
**586**	3-O3B-G7-(	100	HDAC	0.38339	0
**587**	3-O3B-G12	1000000	HDAC	0.511549	0
**588**	3-O3B-H7-(	10	HDAC	0.478492	0
**589**	3-O3B-H12	100000	HDAC	0.072381	0.963
**590**	3-O3B-I7-Q	1	HDAC	0.217688	0.615
**591**	3-O3B-I12-'	10000	HDAC	0.213274	0.309
**592**	3-O3B-J7-Q	0.1	HDAC	0.064781	0.893
**593**	3-O3B-J12-	1000	HDAC	0.104712	0.894
**594**	3-O3B-K3-E	1	HDAC	0.359159	0.001
**595**	3-O3B-K12-	100	HDAC	0.395159	0
**596**	3-O3B-L3-B	10	HDAC	0.02369	1
**597**	3-O3B-M3-I	100	HDAC	0.341409	0.006
**598**	3-O3B-N3-E	1000	HDAC	0.329112	0.018
**599**	3-O3B-O3-E	10000	HDAC	0.327648	0.017
**600**	7-O3B-A5-N	10000	HDAC	0.421615	0

**601**	7-O3B-A7-C	10000	HDAC	0.431716	0
**602**	7-O3B-A9-G	1000	HDAC	0.416026	0
**603**	7-O3B-A12-	10000	HDAC	0.431361	0
**604**	7-O3B-B5-M	1000	HDAC	0.551571	0
**605**	7-O3B-B7-C	1000	HDAC	0.429853	0
**606**	7-O3B-B12-	1000	HDAC	0.486685	0
**607**	7-O3B-C5-M	100	HDAC	0.204155	0.699
**608**	7-O3B-C7-C	100	HDAC	0.43016	0
**609**	7-O3B-C9-G	100	HDAC	0.501441	0
**610**	7-O3B-D7-C	10	HDAC	0.536676	0
**611**	7-O3B-D9-C	10	HDAC	0.550817	0
**612**	7-O3B-D12	100	HDAC	0.043252	0.891
**613**	7-O3B-E5-N	10	HDAC	0.249502	0.051
**614**	7-O3B-E7-C	1	HDAC	0.575826	0
**615**	7-O3B-E9-G	1	HDAC	0.264487	0.149
**616**	7-O3B-E12-	10	HDAC	0.087366	0.802
**617**	7-O3B-F5-N	1	HDAC	0.099137	0.502
**618**	7-O3B-F7-R	10000	HDAC	0.452506	0
**619**	7-O3B-F9-G	0.1	HDAC	0.49895	0
**620**	7-O3B-F12-	1	HDAC	0.532934	0
**621**	7-O3B-F19-	10000	HDAC	0.46588	0
**622**	7-O3B-G7-F	1000	HDAC	0.525556	0
**623**	7-O3B-G19	1000	HDAC	0.552444	0
**624**	7-O3B-H7-F	100	HDAC	0.540618	0
**625**	7-O3B-I7-R	10	HDAC	0.561972	0
**626**	7-O3B-I19-I	100	HDAC	0.554257	0
**627**	7-O3B-J7-R	1	HDAC	0.468241	0
**628**	7-O3B-J19-	10	HDAC	0.128997	0.52
**629**	7-O3B-K4-E	1	HDAC	0.481365	0
**630**	7-O3B-K11-	1	HDAC	0.334521	0.013
**631**	7-O3B-K18-	1	HDAC	0.130536	0.672
**632**	7-O3B-K19-	1	HDAC	0.452489	0
**633**	7-O3B-L2-T	0.1	HDAC	0.149317	0.912
**634**	7-O3B-L4-E	10	HDAC	0.360534	0.001
**635**	7-O3B-L5-P	1	HDAC	0.460449	0

**636**	7-O3B-L8-A	1	HDAC	0.396761	0
**637**	7-O3B-L10-	1	HDAC	0.232473	0.059
**638**	7-O3B-L11-	10	HDAC	0.54165	0
**639**	7-O3B-L14-	1	HDAC	0.540759	0
**640**	7-O3B-L16-	1	HDAC	0.553527	0
**641**	7-O3B-L18-	10	HDAC	0.319774	0.017
**642**	7-O3B-M2-T	1	HDAC	0.148734	0.86
**643**	7-O3B-M5-I	10	HDAC	0.619563	0
**644**	7-O3B-M8-,	10	HDAC	0.575283	0
**645**	7-O3B-M10	10	HDAC	0.604244	0
**646**	7-O3B-M11	100	HDAC	0.485999	0
**647**	7-O3B-M14	10	HDAC	0.085319	0.804
**648**	7-O3B-M16	10	HDAC	0.549364	0
**649**	7-O3B-M18	100	HDAC	0.540162	0
**650**	7-O3B-N2-T	10	HDAC	0.067662	0.993
**651**	7-O3B-N4-E	100	HDAC	0.491406	0
**652**	7-O3B-N5-F	100	HDAC	0.600808	0
**653**	7-O3B-N8-A	100	HDAC	0.590498	0
**654**	7-O3B-N10	100	HDAC	0.595226	0
**655**	7-O3B-N14	100	HDAC	0.38362	0
**656**	7-O3B-N16	100	HDAC	0.132458	0.423
**657**	7-O3B-N18	1000	HDAC	0.539464	0
**658**	7-O3B-O2-T	100	HDAC	0.021682	1
**659**	7-O3B-O4-E	1000	HDAC	0.607908	0
**660**	7-O3B-O5-F	1000	HDAC	0.452239	0
**661**	7-O3B-O8-A	1000	HDAC	0.455388	0
**662**	7-O3B-O10	1000	HDAC	0.621557	0
**663**	7-O3B-O11	1000	HDAC	0.490057	0
**664**	7-O3B-O14	1000	HDAC	0.048014	0.872
**665**	7-O3B-O16	1000	HDAC	0.473938	0
**666**	7-O3B-P2-T	1000	HDAC	0.524136	0
**667**	7-O3B-P4-E	10000	HDAC	0.521671	0
**668**	7-O3B-P5-F	10000	HDAC	0.431158	0
**669**	7-O3B-P8-A	10000	HDAC	0.442068	0
**670**	7-O3B-P10-	10000	HDAC	0.530362	0

**671**	7-O3B-P11·	10000	HDAC	0.470799	0
**672**	7-O3B-P14·	10000	HDAC	0.545302	0
**673**	7-O3B-P16·	10000	HDAC	0.451823	0
**674**	7-O3B-P18·	10000	HDAC	0.431223	0
**0**	2-O8W-A16	10000	EGFR	0.279372	0.083
**1**	2-O8W-A19	10000	EGFR	0.299439	0.105
**2**	2-O8W-B19	1000	EGFR	0.08056	0.969
**3**	2-O8W-C16	1000	EGFR	0.346803	0.005
**4**	2-O8W-C19	100	EGFR	0.117093	0.845
**5**	2-O8W-D16	100	EGFR	0.134845	0.784
**6**	2-O8W-D19	10	EGFR	0.094084	0.979
**7**	2-O8W-E16	10	EGFR	0.097751	0.923
**8**	2-O8W-E19	1	EGFR	0.049759	0.999
**9**	2-O8W-F16	1	EGFR	0.15774	0.714
**10**	2-O8W-K11	0.1	EGFR	0.254041	0.015
**11**	2-O8W-L11	1	EGFR	0.212631	0.307
**12**	2-O8W-L16	0.25	EGFR	0.353794	0.01
**13**	2-O8W-L19	0.1	EGFR	0.157967	0.659
**14**	2-O8W-M1	10	EGFR	0.337617	0.015
**15**	2-O8W-M1	2.5	EGFR	0.195646	0.118
**16**	2-O8W-M1	1	EGFR	0.163393	0.712
**17**	2-O8W-N1(	25	EGFR	0.297448	0.008
**18**	2-O8W-N19	10	EGFR	0.27206	0.042
**19**	2-O8W-O1:	100	EGFR	0.325034	0.005
**20**	2-O8W-O1(	250	EGFR	0.370351	0.019
**21**	2-O8W-O19	100	EGFR	0.13028	0.853
**22**	2-O8W-P11	1000	EGFR	0.403357	0
**23**	2-O8W-P16	2500	EGFR	0.207847	0.44
**24**	2-O8W-P19	1000	EGFR	0.176662	0.919
**25**	3-O8W-F21	10000	EGFR	0.127927	0.899
**26**	3-O8W-G2(	1000	EGFR	0.196361	0.536
**27**	3-O8W-G2:	1000	EGFR	0.26368	0.433
**28**	3-O8W-H2(	100	EGFR	0.113153	0.75
**29**	3-O8W-H2:	100	EGFR	0.137972	0.607
**30**	3-O8W-I20	10	EGFR	0.119705	0.874

**31**	3-O8W-I21	10	EGFR	0.173452	0.301
**32**	3-O8W-J20	1	EGFR	0.228127	0.535
**33**	3-O8W-J21	1	EGFR	0.269393	0.219
**34**	3-O8W-K4-	1	EGFR	0.201266	0.834
**35**	3-O8W-K1{	0.1	EGFR	0.095451	0.948
**36**	3-O8W-K2(	0.1	EGFR	0.185314	0.221
**37**	3-O8W-L4-(	10	EGFR	0.18992	0.935
**38**	3-O8W-L1{	1	EGFR	0.188264	0.198
**39**	3-O8W-M1	10	EGFR	0.343376	0.168
**40**	3-O8W-N4-	100	EGFR	0.360808	0.004
**41**	3-O8W-N1{	100	EGFR	0.451941	0
**42**	3-O8W-O4-	1000	EGFR	0.112026	0.993
**43**	3-O8W-P4-	10000	EGFR	0.098322	0.943
**44**	3-O8W-P1{	1000	EGFR	0.399552	0.005
**45**	4-O8W-F13	1000	EGFR	0.195528	0.347
**46**	4-O8W-G1:	100	EGFR	0.451503	0
**47**	4-O8W-G1(	10000	EGFR	0.160192	0.774
**48**	4-O8W-H1:	10	EGFR	0.166062	0.476
**49**	4-O8W-H1(	1000	EGFR	0.189203	0.332
**50**	4-O8W-I13	1	EGFR	0.260017	0.027
**51**	4-O8W-I16	100	EGFR	0.239348	0.105
**52**	4-O8W-J13	0.1	EGFR	0.323071	0.002
**53**	4-O8W-J16	10	EGFR	0.423121	0
**54**	4-O8W-K7-	1	EGFR	0.278584	0.016
**55**	4-O8W-K1:	0.1	EGFR	0.343377	0.002
**56**	4-O8W-K1(	1	EGFR	0.218463	0.115
**57**	4-O8W-L7-I	10	EGFR	0.189782	0.269
**58**	4-O8W-L13	1	EGFR	0.302186	0.023
**59**	4-O8W-M7	100	EGFR	0.22837	0.067
**60**	4-O8W-M1	10	EGFR	0.443616	0
**61**	4-O8W-N1:	100	EGFR	0.239765	0.032
**62**	4-O8W-O7-	1000	EGFR	0.213205	0.101
**63**	4-O8W-P7-	10000	EGFR	0.19121	0.25
**64**	4-O8W-P1:	1000	EGFR	0.135027	0.743
**65**	5-O8W-F4-I	1000	EGFR	0.284977	0.319

**66**	5-O8W-F7-,	1000	EGFR	0.142616	0.582
**67**	5-O8W-G4-	100	EGFR	0.144314	0.566
**68**	5-O8W-G7-	100	EGFR	0.253344	0.12
**69**	5-O8W-H4-	10	EGFR	0.137693	0.892
**70**	5-O8W-H7-	10	EGFR	0.143333	0.749
**71**	5-O8W-I4-F	1	EGFR	0.18677	0.359
**72**	5-O8W-I7-/	1	EGFR	0.214816	0.307
**73**	5-O8W-J4-I	0.1	EGFR	0.219746	0.228
**74**	5-O8W-J7-/	0.1	EGFR	0.148181	0.798
**75**	5-O8W-K7-	0.1	EGFR	0.226824	0.284
**76**	5-O8W-L7-	1	EGFR	0.252165	0.017
**77**	5-O8W-M7	10	EGFR	0.317777	0.004
**78**	5-O8W-O7-	100	EGFR	0.183662	0.26
**79**	5-O8W-P7-	1000	EGFR	0.407669	0.001
**80**	2-O8W-A15	2500	VEGFR	0.129641	0.689
**81**	2-O8W-A17	10000	VEGFR	0.307896	0.031
**82**	2-O8W-A2(	10000	VEGFR	0.242459	0.144
**83**	2-O8W-B15	250	VEGFR	0.342698	0.058
**84**	2-O8W-B17	1000	VEGFR	0.356607	0.005
**85**	2-O8W-B2(	1000	VEGFR	0.326903	0.014
**86**	2-O8W-C15	25	VEGFR	0.264363	0.048
**87**	2-O8W-C17	100	VEGFR	0.293913	0.024
**88**	2-O8W-D1!	2.5	VEGFR	0.259931	0.074
**89**	2-O8W-D1:	10	VEGFR	0.198971	0.392
**90**	2-O8W-D2(	100	VEGFR	0.30486	0.02
**91**	2-O8W-E17	1	VEGFR	0.310882	0.027
**92**	2-O8W-E2C	10	VEGFR	0.266581	0.104
**93**	2-O8W-F13	10000	VEGFR	0.351261	0.002
**94**	2-O8W-F15	0.25	VEGFR	0.225652	0.121
**95**	2-O8W-F19	10000	VEGFR	0.218017	0.344
**96**	2-O8W-F2C	1	VEGFR	0.191666	0.522
**97**	2-O8W-F21	10000	VEGFR	0.126252	0.526
**98**	2-O8W-G1(	10000	VEGFR	0.327028	0
**99**	2-O8W-G1:	1000	VEGFR	0.419528	0
**100**	2-O8W-G1!	1000	VEGFR	0.309244	0.011

**101**	2-O8W-G2	1000	VEGFR	0.247481	0.165
**102**	2-O8W-H1	1000	VEGFR	0.329089	0.002
**103**	2-O8W-H1	100	VEGFR	0.190922	0.828
**104**	2-O8W-H2	100	VEGFR	0.154619	0.546
**105**	2-O8W-I10	100	VEGFR	0.276253	0.022
**106**	2-O8W-I13	10	VEGFR	0.284179	0.021
**107**	2-O8W-I19	100	VEGFR	0.298957	0.026
**108**	2-O8W-I21	10	VEGFR	0.29525	0.083
**109**	2-O8W-J10	10	VEGFR	0.291996	0.014
**110**	2-O8W-J13	1	VEGFR	0.139178	0.62
**111**	2-O8W-J19	10	VEGFR	0.318103	0.007
**112**	2-O8W-J21	1	VEGFR	0.240877	0.148
**113**	2-O8W-K1	1	VEGFR	0.28447	0.011
**114**	2-O8W-K1	0.1	VEGFR	0.275915	0.027
**115**	2-O8W-K1	1	VEGFR	0.397298	0
**116**	2-O8W-K1	1	VEGFR	0.393183	0
**117**	2-O8W-L12	0.1	VEGFR	0.349641	0.001
**118**	2-O8W-L13	1	VEGFR	0.266818	0.036
**119**	2-O8W-L21	0.1	VEGFR	0.188367	0.787
**120**	2-O8W-M1	1	VEGFR	0.278652	0.028
**121**	2-O8W-M1	10	VEGFR	0.374386	0
**122**	2-O8W-M1	10	VEGFR	0.36531	0.001
**123**	2-O8W-M2	1	VEGFR	0.114386	0.735
**124**	2-O8W-N1	10	VEGFR	0.388658	0
**125**	2-O8W-N1	100	VEGFR	0.345529	0.001
**126**	2-O8W-N1	100	VEGFR	0.233703	0.208
**127**	2-O8W-N2	10	VEGFR	0.07576	0.892
**128**	2-O8W-O1	100	VEGFR	0.221534	0.466
**129**	2-O8W-O1	1000	VEGFR	0.27385	0.04
**130**	2-O8W-O2	100	VEGFR	0.377488	0.011
**131**	2-O8W-P1	1000	VEGFR	0.404539	0
**132**	2-O8W-P1	1000	VEGFR	0.278056	0.303
**133**	2-O8W-P1	10000	VEGFR	0.238587	0.151
**134**	2-O8W-P21	1000	VEGFR	0.104263	0.977
**135**	3-O8W-A3-	1000	VEGFR	0.18268	0.388

**136**	3-O8W-A6-	1000	VEGFR	0.217712	0.313
**137**	3-O8W-A1ε	1000	VEGFR	0.191101	0.579
**138**	3-O8W-B3-	100	VEGFR	0.129779	0.63
**139**	3-O8W-B6-	100	VEGFR	0.103468	0.884
**140**	3-O8W-B1ε	100	VEGFR	0.275277	0.032
**141**	3-O8W-C3-	10	VEGFR	0.073135	0.94
**142**	3-O8W-C6-	10	VEGFR	0.123649	0.645
**143**	3-O8W-C1ε	10	VEGFR	0.157364	0.762
**144**	3-O8W-D3-	1	VEGFR	0.0945	0.926
**145**	3-O8W-D6-	1	VEGFR	0.102595	0.9
**146**	3-O8W-D1ε	1	VEGFR	0.24424	0.12
**147**	3-O8W-E3-(	0.1	VEGFR	0.122044	0.968
**148**	3-O8W-E6-I	0.1	VEGFR	0.140901	0.965
**149**	3-O8W-E18	0.1	VEGFR	0.250741	0.11
**150**	3-O8W-F18	1000	VEGFR	0.20967	0.458
**151**	3-O8W-G1ε	100	VEGFR	0.211676	0.462
**152**	3-O8W-H1ε	10	VEGFR	0.302941	0.003
**153**	3-O8W-I18	1	VEGFR	0.177734	0.737
**154**	3-O8W-J18	0.1	VEGFR	0.282903	0.024
**155**	4-O8W-A1ℤ	10000	VEGFR	0.222109	0.213
**156**	4-O8W-A1ξ	2500	VEGFR	0.146192	0.54
**157**	4-O8W-A2(	10000	VEGFR	0.397586	0.002
**158**	4-O8W-B1ℤ	1000	VEGFR	0.243437	0.164
**159**	4-O8W-B1ξ	250	VEGFR	0.065395	0.95
**160**	4-O8W-B2(	1000	VEGFR	0.128698	0.719
**161**	4-O8W-C1ξ	25	VEGFR	0.10486	0.78
**162**	4-O8W-D1ℤ	100	VEGFR	0.101667	0.811
**163**	4-O8W-D1ξ	2.5	VEGFR	0.261768	0.021
**164**	4-O8W-D2(	100	VEGFR	0.302079	0.046
**165**	4-O8W-E12	10	VEGFR	0.344757	0.002
**166**	4-O8W-E2C	10	VEGFR	0.138437	0.747
**167**	4-O8W-F12	1	VEGFR	0.167587	0.355
**168**	4-O8W-F1ξ	0.25	VEGFR	0.250121	0.276
**169**	4-O8W-F2C	1	VEGFR	0.069908	0.934
**170**	4-O8W-L1€	1	VEGFR	0.08931	0.813

**171**	4-O8W-M1	10	VEGFR	0.25655	0.258
**172**	4-O8W-N1(	100	VEGFR	0.117427	0.779
**173**	4-O8W-O1(	1000	VEGFR	0.112774	0.862
**174**	4-O8W-P1(	10000	VEGFR	0.259013	0.096
**175**	2-O8W-L1C	1	PI3K	0.135944	0.867
**176**	2-O8W-M1	10	PI3K	0.333763	0.001
**177**	2-O8W-N1(	100	PI3K	0.019436	1
**178**	2-O8W-O1(	1000	PI3K	0.171556	0.736
**179**	2-O8W-P1(	10000	PI3K	0.113493	0.895
**180**	3-O8W-A1(	2500	PI3K	0.280264	0
**181**	3-O8W-C1(	250	PI3K	0.292975	0
**182**	3-O8W-D1(	25	PI3K	0.39356	0
**183**	3-O8W-E1(	2.5	PI3K	0.464567	0
**184**	3-O8W-F1(	0.25	PI3K	0.307463	0
**185**	3-O8W-F17	100000	PI3K	0.01577	1
**186**	3-O8W-F1(	500	PI3K	0.329345	0
**187**	3-O8W-G1	10000	PI3K	0.059237	0.998
**188**	3-O8W-G1(	50	PI3K	0.108699	0.938
**189**	3-O8W-H1	1000	PI3K	0.372829	0.009
**190**	3-O8W-I17	100	PI3K	0.033513	1
**191**	3-O8W-I19	5	PI3K	0.357272	0
**192**	3-O8W-J17	10	PI3K	0.291645	0.002
**193**	3-O8W-J19	0.5	PI3K	0.317354	0
**194**	3-O8W-K1(	0.05	PI3K	0.07406	0.994
**195**	3-O8W-L8-	1	PI3K	0.115436	0.935
**196**	3-O8W-L21	0.1	PI3K	0.269887	0.005
**197**	3-O8W-M8	10	PI3K	0.290797	0.12
**198**	3-O8W-M2	1	PI3K	0.219858	0.753
**199**	3-O8W-N8-	100	PI3K	0.324353	0
**200**	3-O8W-N2	10	PI3K	0.054502	0.991
**201**	3-O8W-O8-	1000	PI3K	0.141593	0.806
**202**	3-O8W-O2	100	PI3K	0.306109	0.002
**203**	3-O8W-P8-	10000	PI3K	0.085372	0.973
**204**	3-O8W-P21	1000	PI3K	0.432439	0
**205**	4-O8W-A1(	2500	PI3K	0.333114	0

**206**	4-O8W-B1[illegible]	250	PI3K	0.254252	0.005
**207**	4-O8W-C1[illegible]	25	PI3K	0.366731	0
**208**	4-O8W-D1[illegible]	2.5	PI3K	0.291372	0.178
**209**	4-O8W-E1[illegible]	0.25	PI3K	0.05498	1
**210**	4-O8W-F14	1000	PI3K	0.036174	0.999
**211**	4-O8W-G2-	2500	PI3K	0.37491	0
**212**	4-O8W-G5-	10000	PI3K	0.101585	0.947
**213**	4-O8W-G1[illegible]	100	PI3K	0.119166	0.952
**214**	4-O8W-G2[illegible]	10000	PI3K	0.032349	0.999
**215**	4-O8W-H2-	250	PI3K	0.252511	0.01
**216**	4-O8W-H5-	1000	PI3K	0.043233	0.996
**217**	4-O8W-H1[illegible]	10	PI3K	0.386005	0
**218**	4-O8W-H2[illegible]	1000	PI3K	0.339907	0
**219**	4-O8W-I2-[illegible]	25	PI3K	0.368205	0
**220**	4-O8W-I5-[illegible]	100	PI3K	0.428887	0
**221**	4-O8W-I14	1	PI3K	0.359168	0
**222**	4-O8W-I20	100	PI3K	0.327762	0
**223**	4-O8W-J2-[illegible]	2.5	PI3K	0.273383	0.003
**224**	4-O8W-J5-[illegible]	10	PI3K	0.233565	0.389
**225**	4-O8W-J20	10	PI3K	0.303798	0.006
**226**	4-O8W-K2-	0.25	PI3K	0.37239	0
**227**	4-O8W-K4-	0.1	PI3K	0.435326	0
**228**	4-O8W-K5-	1	PI3K	0.29856	0.002
**229**	4-O8W-K1[illegible]	0.1	PI3K	0.435668	0
**230**	4-O8W-K2[illegible]	1	PI3K	0.3645	0
**231**	4-O8W-L4-[illegible]	1	PI3K	0.338634	0
**232**	4-O8W-L14	0.1	PI3K	0.339467	0
**233**	4-O8W-L1[illegible]	1	PI3K	0.369421	0
**234**	4-O8W-L21	0.1	PI3K	0.324385	0
**235**	4-O8W-M1	1	PI3K	0.412796	0
**236**	4-O8W-M1	10	PI3K	0.389814	0
**237**	4-O8W-M2	1	PI3K	0.330375	0
**238**	4-O8W-N4-	10	PI3K	0.373074	0
**239**	4-O8W-N1[illegible]	10	PI3K	0.371488	0
**240**	4-O8W-N1[illegible]	100	PI3K	0.339814	0

**241**	4-O8W-N2:	10	PI3K	0.308522	0
**242**	4-O8W-O4-	100	PI3K	0.266821	0.377
**243**	4-O8W-O14	100	PI3K	0.438722	0
**244**	4-O8W-O1!	1000	PI3K	0.459209	0
**245**	4-O8W-O2:	100	PI3K	0.489459	0
**246**	4-O8W-P4-	1000	PI3K	0.20614	0.715
**247**	4-O8W-P14	1000	PI3K	0.163235	0.852
**248**	4-O8W-P15	10000	PI3K	0.437886	0
**249**	4-O8W-P21	1000	PI3K	0.14679	0.843
**250**	5-O8W-A6-	2500	PI3K	0.155132	0.81
**251**	5-O8W-A7-	1000	PI3K	0.318458	0
**252**	5-O8W-A16	2500	PI3K	0.439405	0
**253**	5-O8W-A17	10000	PI3K	0.402208	0.003
**254**	5-O8W-B6-	250	PI3K	0.341501	0.042
**255**	5-O8W-B7-	100	PI3K	0.367972	0.017
**256**	5-O8W-B17	1000	PI3K	0.047303	0.997
**257**	5-O8W-C6-	25	PI3K	0.178942	0.222
**258**	5-O8W-C7-	10	PI3K	0.094326	0.961
**259**	5-O8W-C16	250	PI3K	0.340116	0
**260**	5-O8W-C17	100	PI3K	0.111108	0.931
**261**	5-O8W-D6-	2.5	PI3K	0.32567	0
**262**	5-O8W-D7-	1	PI3K	0.279431	0.049
**263**	5-O8W-D16	25	PI3K	0.308466	0.019
**264**	5-O8W-D17	10	PI3K	0.359806	0
**265**	5-O8W-E6-I	0.25	PI3K	0.070688	0.993
**266**	5-O8W-E7-,	0.1	PI3K	0.086034	0.968
**267**	5-O8W-E16	2.5	PI3K	0.103084	0.931
**268**	5-O8W-E17	1	PI3K	0.07489	0.987
**269**	5-O8W-F11	10000	PI3K	0.208433	0.26
**270**	5-O8W-F16	0.25	PI3K	0.233931	0.125
**271**	5-O8W-G9-	10000	PI3K	0.448535	0
**272**	5-O8W-G1:	1000	PI3K	0.16809	0.539
**273**	5-O8W-H9-	1000	PI3K	0.272556	0
**274**	5-O8W-H1:	100	PI3K	0.377271	0
**275**	5-O8W-I9-S	100	PI3K	0.32532	0

**276**	5-O8W-I11	10	PI3K	0.357179	0
**277**	5-O8W-J9-S	10	PI3K	0.340493	0
**278**	5-O8W-J11	1	PI3K	0.300027	0
**279**	5-O8W-K9-	1	PI3K	0.346769	0
**280**	5-O8W-L14	0.1	PI3K	0.366086	0
**281**	5-O8W-L20	1	PI3K	0.330605	0
**282**	5-O8W-L23	0.1	PI3K	0.237893	0.015
**283**	5-O8W-M1	1	PI3K	0.406128	0
**284**	5-O8W-M2	10	PI3K	0.46396	0
**285**	5-O8W-M2	1	PI3K	0.291477	0.002
**286**	5-O8W-N14	10	PI3K	0.452263	0
**287**	5-O8W-N20	100	PI3K	0.314573	0
**288**	5-O8W-N23	10	PI3K	0.376868	0
**289**	5-O8W-O14	100	PI3K	0.501073	0
**290**	5-O8W-O20	1000	PI3K	0.479153	0
**291**	5-O8W-O23	100	PI3K	0.110548	0.916
**292**	5-O8W-P14	1000	PI3K	0.377532	0
**293**	5-O8W-P20	10000	PI3K	0.133264	0.856
**294**	5-O8W-P23	1000	PI3K	0.118969	0.9
**295**	6-O8W-A8-	10000	PI3K	0.371768	0.001
**296**	6-O8W-B8-	1000	PI3K	0.028545	1
**297**	6-O8W-C8-	100	PI3K	0.361694	0.001
**298**	6-O8W-D8-	10	PI3K	0.228647	0.268
**299**	6-O8W-E8-T	1	PI3K	0.231169	0.03
**300**	6-O8W-L6-(	1	PI3K	0.294822	0
**301**	6-O8W-M6	10	PI3K	0.401166	0
**302**	6-O8W-N6-	100	PI3K	0.302121	0
**303**	6-O8W-O6-	1000	PI3K	0.440887	0
**304**	6-O8W-P6-	10000	PI3K	0.021096	1
**305**	1-O8W-F11	10000	Topoisome	0.577957	0
**306**	1-O8W-G11	1000	Topoisome	0.5375	0
**307**	1-O8W-G20	1000	Topoisome	0.469348	0
**308**	1-O8W-H11	100	Topoisome	0.58069	0
**309**	1-O8W-H20	100	Topoisome	0.614075	0
**310**	1-O8W-I11	10	Topoisome	0.60436	0

311	1-O8W-I20	10	Topoisome	0.085935	0.999
312	1-O8W-J11	1	Topoisome	0.389254	0.006
313	1-O8W-J20	1	Topoisome	0.209399	0.545
314	1-O8W-K1	1	Topoisome	0.549573	0
315	1-O8W-K2(	0.1	Topoisome	0.036109	1
316	1-O8W-L11	10	Topoisome	0.571904	0
317	1-O8W-L14	1	Topoisome	0.239839	0.235
318	1-O8W-M1	100	Topoisome	0.510601	0
319	1-O8W-M1	10	Topoisome	0.361101	0.009
320	1-O8W-N1	100	Topoisome	0.638823	0
321	1-O8W-O1	1000	Topoisome	0.486807	0
322	1-O8W-O1	1000	Topoisome	0.568504	0
323	1-O8W-P1	10000	Topoisome	0.420115	0
324	1-O8W-P1	10000	Topoisome	0.477192	0
325	3-O8W-A1	10000	Topoisome	0.487028	0
326	3-O8W-B1	1000	Topoisome	0.531734	0
327	3-O8W-C1	100	Topoisome	0.488737	0
328	3-O8W-D1	10	Topoisome	0.533381	0
329	3-O8W-E11	1	Topoisome	0.218863	0.623
330	3-O8W-G9-	1000	Topoisome	0.453943	0
331	3-O8W-G1(	10000	Topoisome	0.650362	0
332	3-O8W-H9-	100	Topoisome	0.514634	0
333	3-O8W-H1(	1000	Topoisome	0.509517	0
334	3-O8W-I9-[	10	Topoisome	0.101977	0.949
335	3-O8W-I10	100	Topoisome	0.480533	0
336	3-O8W-J9-I	1	Topoisome	0.516638	0
337	3-O8W-J10	10	Topoisome	0.606845	0
338	3-O8W-K7-	0.1	Topoisome	0.485892	0
339	3-O8W-K9-	0.1	Topoisome	0.039009	0.993
340	3-O8W-K1(	1	Topoisome	0.601667	0
341	3-O8W-L6-	0.1	Topoisome	0.443638	0
342	3-O8W-L7-	1	Topoisome	0.193893	0.604
343	3-O8W-L9-	0.5	Topoisome	0.639136	0
344	3-O8W-L1C	0.1	Topoisome	0.519026	0
345	3-O8W-L16	1	Topoisome	0.458261	0

**346**	3-O8W-M6	1	Topoisome	0.500879	0
**347**	3-O8W-M7	10	Topoisome	0.578891	0
**348**	3-O8W-M9	5	Topoisome	0.574193	0
**349**	3-O8W-M1	1	Topoisome	0.516683	0
**350**	3-O8W-M1	10	Topoisome	0.686121	0
**351**	3-O8W-N6-	10	Topoisome	0.070619	0.945
**352**	3-O8W-N9-	50	Topoisome	0.506033	0
**353**	3-O8W-N1(	10	Topoisome	0.659523	0
**354**	3-O8W-N1(	100	Topoisome	0.103597	0.941
**355**	3-O8W-O6-	100	Topoisome	0.48388	0
**356**	3-O8W-O7-	100	Topoisome	0.507657	0
**357**	3-O8W-O9-	500	Topoisome	0.517066	0
**358**	3-O8W-O1(	100	Topoisome	0.542506	0
**359**	3-O8W-O1(	1000	Topoisome	0.199995	0.857
**360**	3-O8W-P6-	1000	Topoisome	0.47232	0
**361**	3-O8W-P7-	1000	Topoisome	0.45442	0
**362**	3-O8W-P9-	5000	Topoisome	0.475871	0
**363**	3-O8W-P1(	1000	Topoisome	0.468246	0
**364**	3-O8W-P1(	10000	Topoisome	0.692409	0
**365**	1-O8W-A1(	10000	Mitotic	0.578259	0
**366**	1-O8W-A1:	1000	Mitotic	0.519252	0
**367**	1-O8W-A1{	1000	Mitotic	0.485644	0
**368**	1-O8W-B1(	1000	Mitotic	0.555306	0
**369**	1-O8W-B1:	100	Mitotic	0.092608	0.963
**370**	1-O8W-B1{	100	Mitotic	0.512461	0
**371**	1-O8W-C1(	100	Mitotic	0.539039	0
**372**	1-O8W-C1:	10	Mitotic	0.50178	0.002
**373**	1-O8W-C1{	10	Mitotic	0.499178	0
**374**	1-O8W-D1(	10	Mitotic	0.556774	0
**375**	1-O8W-D1:	1	Mitotic	0.048579	0.997
**376**	1-O8W-D1{	1	Mitotic	0.545276	0.001
**377**	1-O8W-E1C	1	Mitotic	0.421748	0
**378**	1-O8W-E13	0.1	Mitotic	0.531554	0
**379**	1-O8W-E1{	0.1	Mitotic	0.480771	0.001
**380**	1-O8W-F13	1000	Mitotic	0.510131	0

**381**	1-O8W-G1:	100	Mitotic	0.601629	0
**382**	1-O8W-G1!	1000	Mitotic	0.536895	0
**383**	1-O8W-H1:	10	Mitotic	0.558448	0
**384**	1-O8W-H1!	100	Mitotic	0.494893	0
**385**	1-O8W-I13	1	Mitotic	0.416153	0.028
**386**	1-O8W-I15	10	Mitotic	0.487664	0
**387**	1-O8W-J13	0.1	Mitotic	0.478682	0
**388**	1-O8W-J15	1	Mitotic	0.605178	0
**389**	1-O8W-K7-	0.1	Mitotic	0.572395	0
**390**	1-O8W-K1!	0.1	Mitotic	0.133124	0.808
**391**	1-O8W-L7-'	1	Mitotic	0.093869	0.959
**392**	1-O8W-L2(	0.1	Mitotic	0.041513	1
**393**	1-O8W-M7	10	Mitotic	0.066593	0.995
**394**	1-O8W-M2	1	Mitotic	0.381991	0.102
**395**	1-O8W-N2(	10	Mitotic	0.382573	0.043
**396**	1-O8W-O7-	100	Mitotic	0.607524	0
**397**	1-O8W-O2(	100	Mitotic	0.106369	0.95
**398**	1-O8W-P7-	1000	Mitotic	0.498172	0
**399**	1-O8W-P2(	1000	Mitotic	0.486574	0
**400**	3-O8W-A7-	1000	Mitotic	0.476294	0.001
**401**	3-O8W-B7-	100	Mitotic	0.441567	0
**402**	3-O8W-C7-	10	Mitotic	0.482336	0.001
**403**	3-O8W-D7-	1	Mitotic	0.354022	0.129
**404**	3-O8W-E7-I	0.1	Mitotic	0.470111	0.001
**405**	6-O8W-L19	1	Mitotic	0.193003	0.663
**406**	6-O8W-M1	10	Mitotic	0.174639	0.789
**407**	6-O8W-N1!	100	Mitotic	0.100309	0.88
**408**	6-O8W-O1!	1000	Mitotic	0.522018	0
**409**	6-O8W-P19	10000	Mitotic	0.548431	0
**410**	2-O8W-A12	250	MEK1/2	0.305684	0.389
**411**	2-O8W-B12	25	MEK1/2	0.64585	0
**412**	2-O8W-D1:	2.5	MEK1/2	0.793659	0
**413**	2-O8W-E12	0.25	MEK1/2	0.621756	0
**414**	2-O8W-F12	2.5000000	MEK1/2	0.271035	0.55
**415**	2-O8W-F14	1000	MEK1/2	0.435463	0.091

**416**	2-O8W-G14	100	MEK1/2	0.729797	0
**417**	2-O8W-H14	10	MEK1/2	0.269789	0.31
**418**	2-O8W-I14	1	MEK1/2	0.425837	0.075
**419**	2-O8W-K14	0.1	MEK1/2	0.375046	0.131
**420**	2-O8W-L20	1	MEK1/2	0.088555	0.992
**421**	2-O8W-M2	10	MEK1/2	0.270387	0.53
**422**	2-O8W-N20	100	MEK1/2	0.502409	0
**423**	2-O8W-O20	1000	MEK1/2	0.510524	0.001
**424**	2-O8W-P20	10000	MEK1/2	0.540324	0.007
**425**	4-O8W-A10	1000	MEK1/2	0.56788	0
**426**	4-O8W-A13	1000	MEK1/2	0.762376	0
**427**	4-O8W-B10	100	MEK1/2	0.718351	0
**428**	4-O8W-B13	100	MEK1/2	0.670548	0
**429**	4-O8W-C10	10	MEK1/2	0.723976	0
**430**	4-O8W-C13	10	MEK1/2	0.552945	0
**431**	4-O8W-D10	1	MEK1/2	0.528041	0.002
**432**	4-O8W-D13	1	MEK1/2	0.644867	0
**433**	4-O8W-E10	0.1	MEK1/2	0.163944	0.88
**434**	4-O8W-E13	0.1	MEK1/2	0.232173	0.598
**435**	4-O8W-L19	0.25	MEK1/2	0.500225	0.001
**436**	4-O8W-M1	2.5	MEK1/2	0.494942	0.012
**437**	4-O8W-N19	25	MEK1/2	0.382485	0.053
**438**	4-O8W-O19	250	MEK1/2	0.553304	0
**439**	4-O8W-P19	2500	MEK1/2	0.675511	0
**440**	1-O8W-L2-	1	PARP	0.182192	0.693
**441**	1-O8W-L6-	1	PARP	0.23889	0.707
**442**	1-O8W-M2	10	PARP	0.43246	0.086
**443**	1-O8W-M6	10	PARP	0.235961	0.974
**444**	1-O8W-N2-	100	PARP	0.325406	0.306
**445**	1-O8W-N6-	100	PARP	0.176712	0.673
**446**	1-O8W-O2-	1000	PARP	0.662842	0
**447**	1-O8W-O6-	1000	PARP	0.297776	0.294
**448**	1-O8W-P2-	10000	PARP	0.600956	0
**449**	1-O8W-P6-	10000	PARP	0.499325	0.009
**450**	7-O8W-A3-	1000	PARP	0.525915	0.002

**451**	7-O8W-B2-	10000	PARP	0.595309	0
**452**	7-O8W-B3-	100	PARP	0.638627	0
**453**	7-O8W-C2-	1000	PARP	0.364348	0.134
**454**	7-O8W-C3-	10	PARP	0.593842	0
**455**	7-O8W-D2-	100	PARP	0.429582	0.053
**456**	7-O8W-D3-	1	PARP	0.49308	0.007
**457**	7-O8W-E2-'	10	PARP	0.201074	0.656
**458**	7-O8W-E3-'	0.1	PARP	0.328985	0.205
**459**	7-O8W-F2-'	1	PARP	0.158318	0.971
**460**	7-O8W-G2-	10000	PARP	0.575458	0.001
**461**	7-O8W-H2-	1000	PARP	0.646007	0
**462**	7-O8W-I2-I	100	PARP	0.690629	0
**463**	7-O8W-J2-I	10	PARP	0.294188	0.547
**464**	7-O8W-K2-	1	PARP	0.395778	0.173
**465**	3-O8W-A1!	1000	CDK	0.294028	0.067
**466**	3-O8W-B1!	100	CDK	0.265141	0.177
**467**	3-O8W-B2:	2500	CDK	0.356976	0.112
**468**	3-O8W-C1!	10	CDK	0.326853	0.086
**469**	3-O8W-C2:	250	CDK	0.30813	0.183
**470**	3-O8W-D1!	1	CDK	0.324719	0.046
**471**	3-O8W-D2:	25	CDK	0.355339	0.096
**472**	3-O8W-E1!	0.1	CDK	0.504096	0
**473**	3-O8W-E23	2.5	CDK	0.278166	0.285
**474**	3-O8W-F23	0.25	CDK	0.15891	0.574
**475**	3-O8W-K17	1	CDK	0.232552	0.245
**476**	3-O8W-L1!	1	CDK	0.285948	0.099
**477**	3-O8W-M1	10	CDK	0.462117	0.02
**478**	3-O8W-M1	10	CDK	0.195626	0.429
**479**	3-O8W-N1	100	CDK	0.216658	0.187
**480**	3-O8W-N1!	100	CDK	0.179715	0.372
**481**	3-O8W-O1	1000	CDK	0.308557	0.047
**482**	3-O8W-O1!	1000	CDK	0.351874	0.085
**483**	3-O8W-P17	10000	CDK	0.250139	0.448
**484**	3-O8W-P1!	10000	CDK	0.486068	0.003
**485**	4-O8W-A4-	10000	CDK	0.282603	0.094

**486**	4-O8W-A8-	10000	CDK	0.26863	0.134
**487**	4-O8W-B4-	1000	CDK	0.265263	0.149
**488**	4-O8W-B8-	1000	CDK	0.249342	0.206
**489**	4-O8W-C4-	100	CDK	0.192929	0.574
**490**	4-O8W-C8-	100	CDK	0.195052	0.883
**491**	4-O8W-D4-	10	CDK	0.306138	0.181
**492**	4-O8W-D8-	10	CDK	0.18516	0.617
**493**	4-O8W-E4-	1	CDK	0.143014	0.767
**494**	4-O8W-E8-	1	CDK	0.329274	0.131
**495**	4-O8W-F4-	10000	CDK	0.408309	0.074
**496**	4-O8W-F22	10000	CDK	0.348356	0.014
**497**	4-O8W-G4-	1000	CDK	0.313692	0.097
**498**	4-O8W-G22	1000	CDK	0.335971	0.044
**499**	4-O8W-H4-	100	CDK	0.357467	0.028
**500**	4-O8W-H22	100	CDK	0.273148	0.153
**501**	4-O8W-I4-	10	CDK	0.124953	0.849
**502**	4-O8W-I22	10	CDK	0.371623	0.035
**503**	4-O8W-J4-	1	CDK	0.231967	0.389
**504**	4-O8W-J22	1	CDK	0.171301	0.607
**505**	5-O8W-A19	10000	CDK	0.267218	0.151
**506**	5-O8W-B19	1000	CDK	0.24006	0.314
**507**	5-O8W-C19	100	CDK	0.385511	0.03
**508**	5-O8W-D19	10	CDK	0.355062	0.219
**509**	5-O8W-E19	1	CDK	0.256706	0.654
**510**	5-O8W-K17	1	CDK	0.062851	0.949
**511**	5-O8W-M1	10	CDK	0.143286	0.528
**512**	5-O8W-N17	100	CDK	0.178109	0.571
**513**	5-O8W-O17	1000	CDK	0.248328	0.253
**514**	5-O8W-P17	10000	CDK	0.23014	0.318
**515**	6-O8W-A17	1000	CDK	0.110059	0.848
**516**	6-O8W-B17	100	CDK	0.140358	0.959
**517**	6-O8W-C17	10	CDK	0.341521	0.02
**518**	6-O8W-D17	1	CDK	0.390714	0.012
**519**	6-O8W-E17	0.1	CDK	0.27614	0.297
**520**	6-O8W-L15	1	CDK	0.090525	0.894

521	6-O8W-M1	10	CDK	0.255346	0.139
522	6-O8W-N15	100	CDK	0.189559	0.349
523	6-O8W-O15	1000	CDK	0.227732	0.315
524	6-O8W-P15	10000	CDK	0.300779	0.059
525	7-O8W-A21	10000	BET	0.3366	0.059
526	7-O8W-A22	30000	BET	0.712429	0
527	7-O8W-B21	1000	BET	0.611249	0
528	7-O8W-B22	3000	BET	0.344137	0.011
529	7-O8W-C21	100	BET	0.431248	0.002
530	7-O8W-C22	300	BET	0.215099	0.488
531	7-O8W-D21	10	BET	0.121195	0.963
532	7-O8W-D22	30	BET	0.197215	0.587
533	7-O8W-E21	1	BET	0.273186	0.098
534	7-O8W-E22	3	BET	0.281068	0.069
535	7-O8W-G10	10000	BET	0.456236	0.003
536	7-O8W-G15	10000	BET	0.607479	0
537	7-O8W-H10	1000	BET	0.696151	0
538	7-O8W-H15	1000	BET	0.556143	0
539	7-O8W-I10	100	BET	0.305912	0.042
540	7-O8W-I15	100	BET	0.437554	0
541	7-O8W-J10	10	BET	0.208285	0.544
542	7-O8W-J15	10	BET	0.275943	0.16
543	7-O8W-K10	1	BET	0.20774	0.521
544	7-O8W-K13	1	BET	0.456041	0.002
545	7-O8W-K15	1	BET	0.124518	0.799
546	7-O8W-L12	1	BET	0.47521	0
547	7-O8W-L13	10	BET	0.677303	0
548	7-O8W-L20	1	BET	0.338437	0.013
549	7-O8W-L23	0.03	BET	0.333361	0.027
550	7-O8W-M1	10	BET	0.307762	0.063
551	7-O8W-M1	100	BET	0.626493	0
552	7-O8W-M2	10	BET	0.625605	0
553	7-O8W-M2	0.3	BET	0.516819	0
554	7-O8W-N12	100	BET	0.573253	0
555	7-O8W-N13	1000	BET	0.451833	0

**556**	7-O8W-N2(	100	BET	0.469716	0
**557**	7-O8W-N2:	3	BET	0.28139	0.07
**558**	7-O8W-O1:	1000	BET	0.597792	0
**559**	7-O8W-O2(	1000	BET	0.495976	0.001
**560**	7-O8W-O2:	30	BET	0.466234	0
**561**	7-O8W-P1:	10000	BET	0.622961	0
**562**	7-O8W-P1:	10000	BET	0.284159	0.184
**563**	7-O8W-P2(	10000	BET	0.454792	0
**564**	7-O8W-P2:	300	BET	0.280241	0.189
**565**	8-O8W-K2:	1	BET	0.107533	0.888
**566**	8-O8W-L22	10	BET	0.208218	0.667
**567**	8-O8W-M2	100	BET	0.226773	0.428
**568**	8-O8W-N2:	1000	BET	0.692457	0
**569**	8-O8W-O2:	10000	BET	0.523188	0
**570**	1-O8W-A3-	10000	HDAC	0.480154	0
**571**	1-O8W-B3-	1000	HDAC	0.525484	0
**572**	1-O8W-C3-	100	HDAC	0.096637	0.999
**573**	1-O8W-D3-	10	HDAC	0.380562	0
**574**	1-O8W-E3-'	1	HDAC	0.37195	0
**575**	1-O8W-L12	0.1	HDAC	0.037458	0.998
**576**	1-O8W-M1	1	HDAC	0.102964	0.829
**577**	1-O8W-N1:	10	HDAC	0.462585	0
**578**	1-O8W-O1:	100	HDAC	0.447477	0
**579**	1-O8W-P1:	1000	HDAC	0.380201	0
**580**	3-O8W-A4-	1000	HDAC	0.460311	0
**581**	3-O8W-B4-	100	HDAC	0.502051	0
**582**	3-O8W-C4-	10	HDAC	0.536065	0
**583**	3-O8W-D4-	1	HDAC	0.254127	0.465
**584**	3-O8W-E4-I	0.1	HDAC	0.360999	0
**585**	3-O8W-F7-(	1000	HDAC	0.394802	0
**586**	3-O8W-G7-	100	HDAC	0.520827	0
**587**	3-O8W-G1:	1000000	HDAC	0.426878	0
**588**	3-O8W-H7-	10	HDAC	0.418485	0.001
**589**	3-O8W-H1:	100000	HDAC	0.517453	0
**590**	3-O8W-I7-(	1	HDAC	0.197254	0.46

**591**	3-O8W-I12	10000	HDAC	0.466274	0
**592**	3-O8W-J7-(	0.1	HDAC	0.389049	0
**593**	3-O8W-J12	1000	HDAC	0.449717	0
**594**	3-O8W-K3-	1	HDAC	0.032079	0.998
**595**	3-O8W-K12	100	HDAC	0.504754	0
**596**	3-O8W-L3-I	10	HDAC	0.463984	0
**597**	3-O8W-M3	100	HDAC	0.447304	0
**598**	3-O8W-N3-	1000	HDAC	0.495053	0
**599**	3-O8W-O3-	10000	HDAC	0.357486	0
**600**	7-O8W-A5-	10000	HDAC	0.373685	0
**601**	7-O8W-A7-	10000	HDAC	0.471057	0
**602**	7-O8W-A9-	1000	HDAC	0.490477	0
**603**	7-O8W-A12	10000	HDAC	0.516754	0
**604**	7-O8W-B5-	1000	HDAC	0.487542	0
**605**	7-O8W-B7-	1000	HDAC	0.450226	0
**606**	7-O8W-B12	1000	HDAC	0.554393	0
**607**	7-O8W-C5-	100	HDAC	0.440437	0
**608**	7-O8W-C7-	100	HDAC	0.413714	0
**609**	7-O8W-C9-	100	HDAC	0.514449	0
**610**	7-O8W-D7-	10	HDAC	0.571261	0
**611**	7-O8W-D9-	10	HDAC	0.244401	0.116
**612**	7-O8W-D1:	100	HDAC	0.154034	0.799
**613**	7-O8W-E5-I	10	HDAC	0.349885	0.002
**614**	7-O8W-E7-(	1	HDAC	0.592783	0
**615**	7-O8W-E9-(	1	HDAC	0.305349	0.004
**616**	7-O8W-E12	10	HDAC	0.264145	0.107
**617**	7-O8W-F5-I	1	HDAC	0.492776	0
**618**	7-O8W-F7-I	10000	HDAC	0.474762	0
**619**	7-O8W-F9-(	0.1	HDAC	0.561458	0
**620**	7-O8W-F12	1	HDAC	0.401041	0
**621**	7-O8W-F1¦	10000	HDAC	0.378391	0
**622**	7-O8W-G7-	1000	HDAC	0.569212	0
**623**	7-O8W-G1¦	1000	HDAC	0.473843	0
**624**	7-O8W-H7-	100	HDAC	0.04285	0.994
**625**	7-O8W-I7-F	10	HDAC	0.495637	0

**626**	7-O8W-I19	100	HDAC	0.228357	0.048
**627**	7-O8W-J7-I	1	HDAC	0.470826	0
**628**	7-O8W-J19	10	HDAC	0.03081	0.998
**629**	7-O8W-K4-	1	HDAC	0.259769	0.235
**630**	7-O8W-K11	1	HDAC	0.067559	0.967
**631**	7-O8W-K18	1	HDAC	0.03419	0.997
**632**	7-O8W-K19	1	HDAC	0.321432	0
**633**	7-O8W-L2-7	0.1	HDAC	0.028814	0.999
**634**	7-O8W-L4-I	10	HDAC	0.068353	0.97
**635**	7-O8W-L5-I	1	HDAC	0.22981	0.221
**636**	7-O8W-L8-/	1	HDAC	0.3547	0.002
**637**	7-O8W-L10	1	HDAC	0.434563	0
**638**	7-O8W-L11	10	HDAC	0.465617	0
**639**	7-O8W-L14	1	HDAC	0.211722	0.57
**640**	7-O8W-L16	1	HDAC	0.04014	0.994
**641**	7-O8W-L18	10	HDAC	0.054436	0.994
**642**	7-O8W-M2	1	HDAC	0.176238	0.627
**643**	7-O8W-M5	10	HDAC	0.362393	0
**644**	7-O8W-M8	10	HDAC	0.481491	0
**645**	7-O8W-M1	10	HDAC	0.471121	0
**646**	7-O8W-M1	100	HDAC	0.455701	0
**647**	7-O8W-M1	10	HDAC	0.411295	0.002
**648**	7-O8W-M1	10	HDAC	0.467988	0
**649**	7-O8W-M1	100	HDAC	0.510719	0
**650**	7-O8W-N2-	10	HDAC	0.396897	0
**651**	7-O8W-N4-	100	HDAC	0.449356	0
**652**	7-O8W-N5-	100	HDAC	0.503837	0
**653**	7-O8W-N8-	100	HDAC	0.425638	0
**654**	7-O8W-N1(	100	HDAC	0.46798	0
**655**	7-O8W-N14	100	HDAC	0.238509	0.089
**656**	7-O8W-N1(	100	HDAC	0.536402	0
**657**	7-O8W-N18	1000	HDAC	0.240902	0.35
**658**	7-O8W-O2-	100	HDAC	0.366843	0
**659**	7-O8W-O4-	1000	HDAC	0.523466	0
**660**	7-O8W-O5-	1000	HDAC	0.483166	0

**661**	7-O8W-O8-	1000	HDAC	0.501127	0
**662**	7-O8W-O1(	1000	HDAC	0.633858	0
**663**	7-O8W-O1:	1000	HDAC	0.513873	0
**664**	7-O8W-O1‹	1000	HDAC	0.547144	0
**665**	7-O8W-O1€	1000	HDAC	0.397149	0.002
**666**	7-O8W-P2-	1000	HDAC	0.223589	0.095
**667**	7-O8W-P4-	10000	HDAC	0.518995	0
**668**	7-O8W-P5-	10000	HDAC	0.478234	0
**669**	7-O8W-P8-	10000	HDAC	0.408185	0
**670**	7-O8W-P1(	10000	HDAC	0.476427	0
**671**	7-O8W-P11	10000	HDAC	0.376865	0
**672**	7-O8W-P1‹	10000	HDAC	0.549071	0
**673**	7-O8W-P1€	10000	HDAC	0.509083	0
**674**	7-O8W-P1ξ	10000	HDAC	0.430702	0
