# Supplementary material for: Evaluating feature extraction in ovarian cancer cell line co-cultures using deep neural networks
Source: Commun Biol. 2025 Feb 25;8:303. doi: 10.1038/s42003-025-07766-w (PMC11862010; doi:10.1038/s42003-025-07766-w)
Supplement: Supplementary file 7 — Supplementary Data 5 [file 42003_2025_7766_MOESM7_ESM.pdf]

	Well_annotati	_concentra	ell_Catego	Highest_ES	Pvalue
0	2-KB-A16-G	10000	EGFR	0.303206	0.341
1	2-KB-A19-E	10000	EGFR	0.468074	0
2	2-KB-B19-E	1000	EGFR	0.165787	0.845
3	2-KB-C16-G	1000	EGFR	0.495995	0
4	2-KB-C19-E	100	EGFR	0.491916	0
5	2-KB-D16-G	100	EGFR	0.111636	0.868
6	2-KB-D19-E	10	EGFR	0.381683	0
7	2-KB-E16-G	10	EGFR	0.368123	0.005
8	2-KB-E19-E	1	EGFR	0.431999	0
9	2-KB-F16-G	1	EGFR	0.350401	0.024
10	2-KB-K11-A	0.1	EGFR	0.34706	0.009
11	2-KB-L11-A	1	EGFR	0.39481	0
12	2-KB-L16-O	0.25	EGFR	0.526061	0
13	2-KB-L19-L	0.1	EGFR	0.386344	0.012
14	2-KB-M11-A	10	EGFR	0.43476	0.001
15	2-KB-M16-(	2.5	EGFR	0.39941	0.002
16	2-KB-M19-I	1	EGFR	0.42358	0
17	2-KB-N16-C	25	EGFR	0.446424	0
18	2-KB-N19-L	10	EGFR	0.389494	0.004
19	2-KB-O11-A	100	EGFR	0.405585	0
20	2-KB-O16-C	250	EGFR	0.38353	0.013
21	2-KB-O19-L	100	EGFR	0.35053	0.009
22	2-KB-P11-A	1000	EGFR	0.285764	0.477
23	2-KB-P16-C	2500	EGFR	0.366765	0.144
24	2-KB-P19-L	1000	EGFR	0.468688	0
25	3-KB-F21-R	10000	EGFR	0.260698	0.218
26	3-KB-G20-N	1000	EGFR	0.51083	0
27	3-KB-G21-F	1000	EGFR	0.265715	0.181
28	3-KB-H20-N	100	EGFR	0.525399	0
29	3-KB-H21-F	100	EGFR	0.444127	0
30	3-KB-I20-N	10	EGFR	0.428697	0
31	3-KB-I21-R	10	EGFR	0.381446	0.019
32	3-KB-J20-N	1	EGFR	0.383446	0.002
33	3-KB-J21-R	1	EGFR	0.372895	0.004

**34**	3-KB-K4-Ca	1	EGFR	0.279779	0.045
**35**	3-KB-K18-D	0.1	EGFR	0.47169	0
**36**	3-KB-K20-N	0.1	EGFR	0.103742	0.999
**37**	3-KB-L4-Ca	10	EGFR	0.409309	0.001
**38**	3-KB-L18-D	1	EGFR	0.453342	0
**39**	3-KB-M18-I	10	EGFR	0.44849	0
**40**	3-KB-N4-Ca	100	EGFR	0.243886	0.226
**41**	3-KB-N18-D	100	EGFR	0.402933	0
**42**	3-KB-O4-Ca	1000	EGFR	0.337783	0.207
**43**	3-KB-P4-Ca	10000	EGFR	0.297398	0.583
**44**	3-KB-P18-D	1000	EGFR	0.407254	0.001
**45**	4-KB-F13-Sa	1000	EGFR	0.377016	0.044
**46**	4-KB-G13-S	100	EGFR	0.396478	0.017
**47**	4-KB-G16-V	10000	EGFR	0.30974	0.402
**48**	4-KB-H13-S	10	EGFR	0.499741	0
**49**	4-KB-H16-V	1000	EGFR	0.142867	0.892
**50**	4-KB-I13-Sa	1	EGFR	0.36041	0.001
**51**	4-KB-I16-Va	100	EGFR	0.219592	0.341
**52**	4-KB-J13-Sa	0.1	EGFR	0.309094	0.122
**53**	4-KB-J16-Va	10	EGFR	0.286948	0.105
**54**	4-KB-K7-Ico	1	EGFR	0.407428	0
**55**	4-KB-K13-T	0.1	EGFR	0.114259	0.564
**56**	4-KB-K16-V	1	EGFR	0.356869	0.027
**57**	4-KB-L7-Ico	10	EGFR	0.386502	0.002
**58**	4-KB-L13-Te	1	EGFR	0.302929	0.061
**59**	4-KB-M7-Ic	100	EGFR	0.29455	0.037
**60**	4-KB-M13-T	10	EGFR	0.323829	0.049
**61**	4-KB-N13-T	100	EGFR	0.38041	0.018
**62**	4-KB-O7-Ico	1000	EGFR	0.386822	0
**63**	4-KB-P7-Ico	10000	EGFR	0.097694	0.974
**64**	4-KB-P13-T	1000	EGFR	0.270189	0.438
**65**	5-KB-F4-Po	1000	EGFR	0.366088	0.078
**66**	5-KB-F7-AZ	1000	EGFR	0.479503	0
**67**	5-KB-G4-Po	100	EGFR	0.387873	0.018
**68**	5-KB-G7-AZ	100	EGFR	0.332817	0.012

**69**	5-KB-H4-Pc	10	EGFR	0.486043	0
**70**	5-KB-H7-AZ	10	EGFR	0.308582	0.011
**71**	5-KB-I4-Po:	1	EGFR	0.206522	0.593
**72**	5-KB-I7-AZI	1	EGFR	0.226221	0.247
**73**	5-KB-J4-Po:	0.1	EGFR	0.10354	0.932
**74**	5-KB-J7-AZI	0.1	EGFR	0.158989	0.472
**75**	5-KB-K7-Ol	0.1	EGFR	0.102424	0.884
**76**	5-KB-L7-Ol	1	EGFR	0.080958	1
**77**	5-KB-M7-Ol	10	EGFR	0.114884	0.59
**78**	5-KB-O7-Ol	100	EGFR	0.305991	0.044
**79**	5-KB-P7-Ol	1000	EGFR	0.034085	1
**80**	2-KB-A15-L	2500	VEGFR	0.433826	0.001
**81**	2-KB-A17-N	10000	VEGFR	0.298611	0.426
**82**	2-KB-A20-T	10000	VEGFR	0.3819	0.002
**83**	2-KB-B15-L	250	VEGFR	0.211052	0.607
**84**	2-KB-B17-N	1000	VEGFR	0.444052	0
**85**	2-KB-B20-T	1000	VEGFR	0.312969	0.048
**86**	2-KB-C15-L	25	VEGFR	0.533033	0
**87**	2-KB-C17-N	100	VEGFR	0.34897	0.029
**88**	2-KB-D15-L	2.5	VEGFR	0.425057	0
**89**	2-KB-D17-N	10	VEGFR	0.378288	0
**90**	2-KB-D20-T	100	VEGFR	0.37705	0.001
**91**	2-KB-E17-N	1	VEGFR	0.208787	0.631
**92**	2-KB-E20-Ti	10	VEGFR	0.300494	0.183
**93**	2-KB-F13-A	10000	VEGFR	0.369182	0.016
**94**	2-KB-F15-L	0.25	VEGFR	0.379905	0
**95**	2-KB-F19-R	10000	VEGFR	0.341164	0.22
**96**	2-KB-F20-Ti	1	VEGFR	0.348157	0.002
**97**	2-KB-F21-V	10000	VEGFR	0.460212	0
**98**	2-KB-G10-A	10000	VEGFR	0.179968	0.661
**99**	2-KB-G13-A	1000	VEGFR	0.374861	0.008
**100**	2-KB-G19-R	1000	VEGFR	0.466202	0.001
**101**	2-KB-G21-V	1000	VEGFR	0.428314	0.003
**102**	2-KB-H10-A	1000	VEGFR	0.446417	0
**103**	2-KB-H13-A	100	VEGFR	0.436846	0

**104**	2-KB-H21-\	100	VEGFR	0.513412	0
**105**	2-KB-I10-A[	100	VEGFR	0.027446	1
**106**	2-KB-I13-A›	10	VEGFR	0.417314	0
**107**	2-KB-I19-Re	100	VEGFR	0.410266	0
**108**	2-KB-I21-Va	10	VEGFR	0.398096	0.001
**109**	2-KB-J10-A[	10	VEGFR	0.416098	0
**110**	2-KB-J13-A:	1	VEGFR	0.383093	0
**111**	2-KB-J19-R(	10	VEGFR	0.474741	0
**112**	2-KB-J21-V:	1	VEGFR	0.409014	0
**113**	2-KB-K10-A	1	VEGFR	0.372375	0.002
**114**	2-KB-K13-V	0.1	VEGFR	0.397632	0.001
**115**	2-KB-K17-P	1	VEGFR	0.38793	0
**116**	2-KB-K19-R	1	VEGFR	0.467428	0
**117**	2-KB-L12-S(	0.1	VEGFR	0.308016	0.005
**118**	2-KB-L13-V	1	VEGFR	0.328033	0.01
**119**	2-KB-L21-C	0.1	VEGFR	0.426916	0
**120**	2-KB-M12-S	1	VEGFR	0.370114	0
**121**	2-KB-M13-\	10	VEGFR	0.455716	0
**122**	2-KB-M17-I	10	VEGFR	0.425987	0
**123**	2-KB-M21-(	1	VEGFR	0.414154	0
**124**	2-KB-N12-S	10	VEGFR	0.208322	0.135
**125**	2-KB-N13-\	100	VEGFR	0.397202	0
**126**	2-KB-N17-F	100	VEGFR	0.413174	0
**127**	2-KB-N21-C	10	VEGFR	0.404226	0
**128**	2-KB-O12-S	100	VEGFR	0.429854	0
**129**	2-KB-O17-F	1000	VEGFR	0.43459	0
**130**	2-KB-O21-C	100	VEGFR	0.374451	0
**131**	2-KB-P12-S	1000	VEGFR	0.318571	0.049
**132**	2-KB-P13-V	1000	VEGFR	0.357672	0.051
**133**	2-KB-P17-P	10000	VEGFR	0.418676	0
**134**	2-KB-P21-C	1000	VEGFR	0.182918	0.821
**135**	3-KB-A3-Ca	1000	VEGFR	0.373028	0.001
**136**	3-KB-A6-Fo	1000	VEGFR	0.331384	0.097
**137**	3-KB-A18-L	1000	VEGFR	0.13564	0.764
**138**	3-KB-B3-Ca	100	VEGFR	0.117724	0.748

**139**	3-KB-B6-Fo	100	VEGFR	0.190612	0.503
**140**	3-KB-B18-L	100	VEGFR	0.159336	0.604
**141**	3-KB-C3-Ca	10	VEGFR	0.135086	0.715
**142**	3-KB-C6-Fo	10	VEGFR	0.083873	0.727
**143**	3-KB-C18-L	10	VEGFR	0.210768	0.342
**144**	3-KB-D3-Ca	1	VEGFR	0.111984	0.876
**145**	3-KB-D6-Fo	1	VEGFR	0.047005	0.973
**146**	3-KB-D18-L	1	VEGFR	0.277174	0.051
**147**	3-KB-E3-Cal	0.1	VEGFR	0.053444	0.959
**148**	3-KB-E6-Foı	0.1	VEGFR	0.171073	0.303
**149**	3-KB-E18-Li	0.1	VEGFR	0.289522	0.023
**150**	3-KB-F18-B	1000	VEGFR	0.370787	0.052
**151**	3-KB-G18-E	100	VEGFR	0.423252	0.002
**152**	3-KB-H18-E	10	VEGFR	0.277093	0.042
**153**	3-KB-I18-Bı	1	VEGFR	0.380934	0
**154**	3-KB-J18-Bı	0.1	VEGFR	0.375226	0.002
**155**	4-KB-A12-E	10000	VEGFR	0.189201	0.871
**156**	4-KB-A15-G	2500	VEGFR	0.200213	0.434
**157**	4-KB-A20-N	10000	VEGFR	0.211126	0.428
**158**	4-KB-B12-E	1000	VEGFR	0.375015	0.028
**159**	4-KB-B15-G	250	VEGFR	0.299468	0.091
**160**	4-KB-B20-N	1000	VEGFR	0.170075	0.72
**161**	4-KB-C15-G	25	VEGFR	0.351699	0.003
**162**	4-KB-D12-E	100	VEGFR	0.384441	0.001
**163**	4-KB-D15-G	2.5	VEGFR	0.393838	0
**164**	4-KB-D20-N	100	VEGFR	0.381238	0.002
**165**	4-KB-E12-El	10	VEGFR	0.124013	0.456
**166**	4-KB-E20-M	10	VEGFR	0.205729	0.514
**167**	4-KB-F12-El	1	VEGFR	0.167117	0.592
**168**	4-KB-F15-G	0.25	VEGFR	0.432354	0
**169**	4-KB-F20-M	1	VEGFR	0.277709	0.002
**170**	4-KB-L16-T	1	VEGFR	0.270371	0.046
**171**	4-KB-M16-T	10	VEGFR	0.234719	0.031
**172**	4-KB-N16-T	100	VEGFR	0.303375	0.001
**173**	4-KB-O16-T	1000	VEGFR	0.351569	0.016

**174**	4-KB-P16-T	10000	VEGFR	0.292402	0.086
**175**	2-KB-L10-Ic	1	PI3K	0.233691	0.128
**176**	2-KB-M10-I	10	PI3K	0.276412	0.047
**177**	2-KB-N10-I	100	PI3K	0.369257	0
**178**	2-KB-O10-I	1000	PI3K	0.402218	0
**179**	2-KB-P10-Ic	10000	PI3K	0.439366	0
**180**	3-KB-A16-P	2500	PI3K	0.039934	0.982
**181**	3-KB-C16-P	250	PI3K	0.196206	0.171
**182**	3-KB-D16-F	25	PI3K	0.049468	0.992
**183**	3-KB-E16-P	2.5	PI3K	0.036056	0.991
**184**	3-KB-F16-P	0.25	PI3K	0.419328	0
**185**	3-KB-F17-N	100000	PI3K	0.056252	0.916
**186**	3-KB-F19-D	500	PI3K	0.059317	0.936
**187**	3-KB-G17-N	10000	PI3K	0.227116	0.094
**188**	3-KB-G19-D	50	PI3K	0.4299	0.002
**189**	3-KB-H17-N	1000	PI3K	0.242072	0.256
**190**	3-KB-I17-M	100	PI3K	0.117327	0.539
**191**	3-KB-I19-D	5	PI3K	0.263472	0.057
**192**	3-KB-J17-N	10	PI3K	0.347707	0
**193**	3-KB-J19-D	0.5	PI3K	0.343725	0.003
**194**	3-KB-K19-D	0.05	PI3K	0.2644	0.064
**195**	3-KB-L8-Pic	1	PI3K	0.165571	0.134
**196**	3-KB-L21-T	0.1	PI3K	0.066967	0.937
**197**	3-KB-M8-Pi	10	PI3K	0.263557	0.043
**198**	3-KB-M21-T	1	PI3K	0.171695	0.543
**199**	3-KB-N8-Pi	100	PI3K	0.314534	0.052
**200**	3-KB-N21-T	10	PI3K	0.240946	0.292
**201**	3-KB-O8-Pi	1000	PI3K	0.351638	0.01
**202**	3-KB-O21-T	100	PI3K	0.194769	0.816
**203**	3-KB-P8-Pic	10000	PI3K	0.449655	0
**204**	3-KB-P21-T	1000	PI3K	0.145787	0.432
**205**	4-KB-A19-A	2500	PI3K	0.150168	0.842
**206**	4-KB-B19-A	250	PI3K	0.164268	0.776
**207**	4-KB-C19-A	25	PI3K	0.308774	0.053
**208**	4-KB-D19-A	2.5	PI3K	0.128985	0.943

**209**	4-KB-E19-A	0.25	PI3K	0.270528	0.021
**210**	4-KB-F14-N	1000	PI3K	0.152289	0.975
**211**	4-KB-G2-TG	2500	PI3K	0.102138	0.972
**212**	4-KB-G5-So	10000	PI3K	0.46136	0
**213**	4-KB-G14-N	100	PI3K	0.477298	0
**214**	4-KB-G20-E	10000	PI3K	0.39825	0
**215**	4-KB-H2-TG	250	PI3K	0.082702	0.971
**216**	4-KB-H5-So	1000	PI3K	0.296178	0.032
**217**	4-KB-H14-N	10	PI3K	0.367535	0.001
**218**	4-KB-H20-E	1000	PI3K	0.422312	0
**219**	4-KB-I2-TGI	25	PI3K	0.195887	0.849
**220**	4-KB-I5-Sor	100	PI3K	0.314166	0.003
**221**	4-KB-I14-N	1	PI3K	0.330897	0.003
**222**	4-KB-I20-Bu	100	PI3K	0.344657	0.002
**223**	4-KB-J2-TGI	2.5	PI3K	0.048344	0.995
**224**	4-KB-J5-Sor	10	PI3K	0.07166	0.755
**225**	4-KB-J20-Bu	10	PI3K	0.193538	0.114
**226**	4-KB-K2-TG	0.25	PI3K	0.173856	0.866
**227**	4-KB-K4-Da	0.1	PI3K	0.10593	0.617
**228**	4-KB-K5-So	1	PI3K	0.268805	0.059
**229**	4-KB-K14-N	0.1	PI3K	0.177426	0.236
**230**	4-KB-K20-B	1	PI3K	0.316209	0.008
**231**	4-KB-L4-Da	1	PI3K	0.197338	0.877
**232**	4-KB-L14-G	0.1	PI3K	0.143632	0.405
**233**	4-KB-L15-T	1	PI3K	0.327228	0.002
**234**	4-KB-L21-C	0.1	PI3K	0.349921	0.002
**235**	4-KB-M14-(	1	PI3K	0.263753	0.074
**236**	4-KB-M15-	10	PI3K	0.280218	0.011
**237**	4-KB-M21-(	1	PI3K	0.337621	0.003
**238**	4-KB-N4-Da	10	PI3K	0.457247	0
**239**	4-KB-N14-G	10	PI3K	0.388573	0.001
**240**	4-KB-N15-T	100	PI3K	0.201194	0.525
**241**	4-KB-N21-C	10	PI3K	0.370826	0
**242**	4-KB-O4-Da	100	PI3K	0.377617	0.004
**243**	4-KB-O14-G	100	PI3K	0.390707	0

**244**	4-KB-O15-T	1000	PI3K	0.27023	0.289
**245**	4-KB-O21-C	100	PI3K	0.503325	0
**246**	4-KB-P4-Da	1000	PI3K	0.376568	0.001
**247**	4-KB-P14-G	1000	PI3K	0.514442	0
**248**	4-KB-P15-T	10000	PI3K	0.396563	0
**249**	4-KB-P21-C	1000	PI3K	0.49656	0
**250**	5-KB-A6-LY	2500	PI3K	0.49059	0
**251**	5-KB-A7-AN	1000	PI3K	0.348155	0.008
**252**	5-KB-A16-A	2500	PI3K	0.444937	0
**253**	5-KB-A17-P	10000	PI3K	0.428507	0
**254**	5-KB-B6-LY	250	PI3K	0.395535	0.003
**255**	5-KB-B7-AN	100	PI3K	0.150408	0.725
**256**	5-KB-B17-P	1000	PI3K	0.263324	0.052
**257**	5-KB-C6-LY	25	PI3K	0.410266	0.007
**258**	5-KB-C7-AN	10	PI3K	0.107051	0.93
**259**	5-KB-C16-A	250	PI3K	0.430279	0
**260**	5-KB-C17-P	100	PI3K	0.318397	0.004
**261**	5-KB-D6-LY	2.5	PI3K	0.193711	0.652
**262**	5-KB-D7-AN	1	PI3K	0.256955	0.174
**263**	5-KB-D16-A	25	PI3K	0.442353	0
**264**	5-KB-D17-P	10	PI3K	0.189917	0.599
**265**	5-KB-E6-LY	0.25	PI3K	0.176689	0.791
**266**	5-KB-E7-AN	0.1	PI3K	0.136702	0.766
**267**	5-KB-E16-A	2.5	PI3K	0.21693	0.052
**268**	5-KB-E17-P	1	PI3K	0.30527	0.01
**269**	5-KB-F11-G	10000	PI3K	0.456016	0
**270**	5-KB-F16-A	0.25	PI3K	0.302317	0.003
**271**	5-KB-G9-Se	10000	PI3K	0.483795	0
**272**	5-KB-G11-G	1000	PI3K	0.445226	0
**273**	5-KB-H9-Se	1000	PI3K	0.461549	0
**274**	5-KB-H11-G	100	PI3K	0.475245	0
**275**	5-KB-I9-Ser	100	PI3K	0.262797	0.03
**276**	5-KB-I11-G	10	PI3K	0.060272	0.966
**277**	5-KB-J9-Ser	10	PI3K	0.15421	0.264
**278**	5-KB-J11-G	1	PI3K	0.279317	0.02

279	5-KB-K9-Se	1	PI3K	0.174694	0.126
280	5-KB-L14-A	0.1	PI3K	0.095658	0.912
281	5-KB-L20-Z	1	PI3K	0.318617	0.008
282	5-KB-L23-O	0.1	PI3K	0.107996	0.968
283	5-KB-M14-/	1	PI3K	0.454278	0
284	5-KB-M20-Z	10	PI3K	0.326843	0.051
285	5-KB-M23-(	1	PI3K	0.342788	0.019
286	5-KB-N14-A	10	PI3K	0.440012	0
287	5-KB-N20-Z	100	PI3K	0.412165	0
288	5-KB-N23-C	10	PI3K	0.490213	0
289	5-KB-O14-A	100	PI3K	0.469412	0
290	5-KB-O20-Z	1000	PI3K	0.470415	0
291	5-KB-O23-C	100	PI3K	0.505434	0
292	5-KB-P14-A	1000	PI3K	0.431781	0
293	5-KB-P20-Z	10000	PI3K	0.493164	0
294	5-KB-P23-C	1000	PI3K	0.504128	0
295	6-KB-A8-TG	10000	PI3K	0.465698	0
296	6-KB-B8-TG	1000	PI3K	0.403804	0.001
297	6-KB-C8-TG	100	PI3K	0.398216	0
298	6-KB-D8-TG	10	PI3K	0.377341	0
299	6-KB-E8-TG	1	PI3K	0.246903	0.138
300	6-KB-L6-GD	1	PI3K	0.264686	0.148
301	6-KB-M6-GI	10	PI3K	0.082234	0.845
302	6-KB-N6-GI	100	PI3K	0.363029	0.014
303	6-KB-O6-GI	1000	PI3K	0.490924	0
304	6-KB-P6-GD	10000	PI3K	0.393625	0.001
305	1-KB-F11-A	10000	Topoisome	0.44372	0.008
306	1-KB-G11-A	1000	Topoisome	0.456364	0.008
307	1-KB-G20-E	1000	Topoisome	0.321223	0.5
308	1-KB-H11-A	100	Topoisome	0.472485	0
309	1-KB-H20-E	100	Topoisome	0.44547	0.004
310	1-KB-I11-Ar	10	Topoisome	0.387477	0.026
311	1-KB-I20-Ep	10	Topoisome	0.370607	0.031
312	1-KB-J11-Ar	1	Topoisome	0.498242	0
313	1-KB-J20-Ep	1	Topoisome	0.332907	0.193

314	1-KB-K11-S	1	Topoisome	0.447454	0.001
315	1-KB-K20-E	0.1	Topoisome	0.052017	1
316	1-KB-L11-SI	10	Topoisome	0.445064	0.009
317	1-KB-L14-T	1	Topoisome	0.50643	0
318	1-KB-M11-S	100	Topoisome	0.495991	0
319	1-KB-M14-T	10	Topoisome	0.424252	0.009
320	1-KB-N14-T	100	Topoisome	0.433518	0.009
321	1-KB-O11-S	1000	Topoisome	0.312658	0.548
322	1-KB-O14-T	1000	Topoisome	0.466997	0.001
323	1-KB-P11-S	10000	Topoisome	0.318476	0.5
324	1-KB-P14-T	10000	Topoisome	0.321684	0.451
325	3-KB-A11-E	10000	Topoisome	0.477114	0.001
326	3-KB-B11-E	1000	Topoisome	0.487889	0.001
327	3-KB-C11-E	100	Topoisome	0.612323	0
328	3-KB-D11-E	10	Topoisome	0.564962	0
329	3-KB-E11-E	1	Topoisome	0.600057	0
330	3-KB-G9-Da	1000	Topoisome	0.481119	0
331	3-KB-G10-T	10000	Topoisome	0.517591	0
332	3-KB-H9-Da	100	Topoisome	0.463377	0
333	3-KB-H10-T	1000	Topoisome	0.44702	0
334	3-KB-I9-Dau	10	Topoisome	0.563673	0
335	3-KB-I10-Te	100	Topoisome	0.429096	0
336	3-KB-J9-Dau	1	Topoisome	0.431157	0
337	3-KB-J10-Te	10	Topoisome	0.510811	0
338	3-KB-K7-Ida	0.1	Topoisome	0.399385	0
339	3-KB-K9-Da	0.1	Topoisome	0.51485	0
340	3-KB-K10-T	1	Topoisome	0.1563	0.5
341	3-KB-L6-Do	0.1	Topoisome	0.486781	0
342	3-KB-L7-Ida	1	Topoisome	0.515981	0
343	3-KB-L9-Val	0.5	Topoisome	0.072859	0.93
344	3-KB-L10-M	0.1	Topoisome	0.532847	0
345	3-KB-L16-P	1	Topoisome	0.509458	0
346	3-KB-M6-D	1	Topoisome	0.52841	0
347	3-KB-M7-Id	10	Topoisome	0.091059	0.749
348	3-KB-M9-Va	5	Topoisome	0.552941	0

349	3-KB-M10-I	1	Topoisome	0.169617	0.473
350	3-KB-M16-I	10	Topoisome	0.513168	0
351	3-KB-N6-Dc	10	Topoisome	0.636359	0
352	3-KB-N9-Va	50	Topoisome	0.494533	0.001
353	3-KB-N10-M	10	Topoisome	0.258468	0.682
354	3-KB-N16-F	100	Topoisome	0.504493	0
355	3-KB-O6-Dc	100	Topoisome	0.472429	0.001
356	3-KB-O7-Ida	100	Topoisome	0.393583	0.071
357	3-KB-O9-Va	500	Topoisome	0.476207	0.001
358	3-KB-O10-M	100	Topoisome	0.369302	0.001
359	3-KB-O16-F	1000	Topoisome	0.474452	0
360	3-KB-P6-Dc	1000	Topoisome	0.333061	0.414
361	3-KB-P7-Ida	1000	Topoisome	0.349447	0.477
362	3-KB-P9-Va	5000	Topoisome	0.344074	0.425
363	3-KB-P10-M	1000	Topoisome	0.476545	0.002
364	3-KB-P16-P	10000	Topoisome	0.493024	0
365	1-KB-A10-V	10000	Mitotic	0.703525	0
366	1-KB-A13-Ix	1000	Mitotic	0.68651	0
367	1-KB-A18-P	1000	Mitotic	0.715277	0
368	1-KB-B10-V	1000	Mitotic	0.70095	0
369	1-KB-B13-Ix	100	Mitotic	0.358269	0.263
370	1-KB-B18-P	100	Mitotic	0.705785	0
371	1-KB-C10-V	100	Mitotic	0.721234	0
372	1-KB-C13-Ix	10	Mitotic	0.172853	0.883
373	1-KB-C18-P	10	Mitotic	0.699664	0
374	1-KB-D10-V	10	Mitotic	0.665835	0
375	1-KB-D13-I:	1	Mitotic	0.381378	0.087
376	1-KB-D18-F	1	Mitotic	0.490383	0.002
377	1-KB-E10-V	1	Mitotic	0.581844	0
378	1-KB-E13-Ix	0.1	Mitotic	0.264001	0.462
379	1-KB-E18-P	0.1	Mitotic	0.29151	0.218
380	1-KB-F13-V	1000	Mitotic	0.726412	0
381	1-KB-G13-V	100	Mitotic	0.407571	0.046
382	1-KB-G15-E	1000	Mitotic	0.729925	0
383	1-KB-H13-V	10	Mitotic	0.313285	0.309

**384**	1-KB-H15-E	100	Mitotic	0.738417	0
**385**	1-KB-I13-Vi	1	Mitotic	0.303614	0.238
**386**	1-KB-I15-Er	10	Mitotic	0.733139	0
**387**	1-KB-J13-Vi	0.1	Mitotic	0.313111	0.222
**388**	1-KB-J15-Er	1	Mitotic	0.709581	0
**389**	1-KB-K7-Vir	0.1	Mitotic	0.345202	0.059
**390**	1-KB-K15-E	0.1	Mitotic	0.651122	0
**391**	1-KB-L7-Vir	1	Mitotic	0.406311	0.029
**392**	1-KB-L20-V	0.1	Mitotic	0.107265	0.826
**393**	1-KB-M7-Vi	10	Mitotic	0.239552	0.232
**394**	1-KB-M20-\	1	Mitotic	0.181828	0.762
**395**	1-KB-N20-\	10	Mitotic	0.157423	0.727
**396**	1-KB-O7-Vi	100	Mitotic	0.67034	0
**397**	1-KB-O20-\	100	Mitotic	0.187587	0.389
**398**	1-KB-P7-Vir	1000	Mitotic	0.727272	0
**399**	1-KB-P20-V	1000	Mitotic	0.69598	0
**400**	3-KB-A7-Dc	1000	Mitotic	0.687664	0
**401**	3-KB-B7-Dc	100	Mitotic	0.69025	0
**402**	3-KB-C7-Dc	10	Mitotic	0.640676	0
**403**	3-KB-D7-Dc	1	Mitotic	0.445984	0.005
**404**	3-KB-E7-Do	0.1	Mitotic	0.079391	0.992
**405**	6-KB-L19-A	1	Mitotic	0.130148	0.937
**406**	6-KB-M19-/	10	Mitotic	0.595564	0
**407**	6-KB-N19-A	100	Mitotic	0.302662	0.479
**408**	6-KB-O19-A	1000	Mitotic	0.671693	0
**409**	6-KB-P19-A	10000	Mitotic	0.707979	0
**410**	2-KB-A12-T	250	MEK1/2	0.564792	0.003
**411**	2-KB-B12-T	25	MEK1/2	0.58854	0
**412**	2-KB-D12-T	2.5	MEK1/2	0.511976	0.004
**413**	2-KB-E12-T	0.25	MEK1/2	0.370537	0.122
**414**	2-KB-F12-T	2.5000000	MEK1/2	0.517782	0.009
**415**	2-KB-F14-C	1000	MEK1/2	0.548946	0.007
**416**	2-KB-G14-C	100	MEK1/2	0.613731	0
**417**	2-KB-H14-C	10	MEK1/2	0.543717	0
**418**	2-KB-I14-C	1	MEK1/2	0.218932	0.534

**419**	2-KB-K14-C	0.1	MEK1/2	0.34417	0.132
**420**	2-KB-L20-S	1	MEK1/2	0.532723	0.002
**421**	2-KB-M20-S	10	MEK1/2	0.588554	0.001
**422**	2-KB-N20-S	100	MEK1/2	0.565933	0
**423**	2-KB-O20-S	1000	MEK1/2	0.608461	0
**424**	2-KB-P20-S	10000	MEK1/2	0.586529	0.004
**425**	4-KB-A10-B	1000	MEK1/2	0.389825	0.127
**426**	4-KB-A13-P	1000	MEK1/2	0.516696	0.011
**427**	4-KB-B10-B	100	MEK1/2	0.435485	0.075
**428**	4-KB-B13-P	100	MEK1/2	0.491057	0.025
**429**	4-KB-C10-B	10	MEK1/2	0.290881	0.429
**430**	4-KB-C13-P	10	MEK1/2	0.340318	0.319
**431**	4-KB-D10-E	1	MEK1/2	0.189337	0.715
**432**	4-KB-D13-F	1	MEK1/2	0.584422	0.002
**433**	4-KB-E10-B	0.1	MEK1/2	0.564173	0.001
**434**	4-KB-E13-P	0.1	MEK1/2	0.578576	0
**435**	4-KB-L19-G	0.25	MEK1/2	0.496022	0.005
**436**	4-KB-M19-(	2.5	MEK1/2	0.212754	0.561
**437**	4-KB-N19-G	25	MEK1/2	0.332447	0.12
**438**	4-KB-O19-G	250	MEK1/2	0.14957	0.747
**439**	4-KB-P19-G	2500	MEK1/2	0.460592	0.042
**440**	1-KB-L2-Ola	1	PARP	0.426501	0.021
**441**	1-KB-L6-Ru	1	PARP	0.521172	0.014
**442**	1-KB-M2-Ol	10	PARP	0.429619	0.037
**443**	1-KB-M6-Ru	10	PARP	0.436278	0.045
**444**	1-KB-N2-Ol	100	PARP	0.524248	0.002
**445**	1-KB-N6-Ru	100	PARP	0.204256	1
**446**	1-KB-O2-Ol	1000	PARP	0.248582	0.719
**447**	1-KB-O6-Ru	1000	PARP	0.357986	0.624
**448**	1-KB-P2-Ola	10000	PARP	0.460698	0.109
**449**	1-KB-P6-Ru	10000	PARP	0.397512	0.23
**450**	7-KB-A3-Tal	1000	PARP	0.457379	0.101
**451**	7-KB-B2-Ve	10000	PARP	0.370283	0.246
**452**	7-KB-B3-Tal	100	PARP	0.499915	0.024
**453**	7-KB-C2-Ve	1000	PARP	0.708476	0

**454**	7-KB-C3-Tal	10	PARP	0.305913	0.462
**455**	7-KB-D2-Ve	100	PARP	0.572986	0
**456**	7-KB-D3-Ta	1	PARP	0.626913	0.002
**457**	7-KB-E2-Vel	10	PARP	0.258224	0.523
**458**	7-KB-E3-Tal	0.1	PARP	0.428613	0.199
**459**	7-KB-F2-Vel	1	PARP	0.496558	0.011
**460**	7-KB-G2-Ni	10000	PARP	0.471696	0.056
**461**	7-KB-H2-Ni	1000	PARP	0.258549	0.688
**462**	7-KB-I2-Nir	100	PARP	0.610925	0.001
**463**	7-KB-J2-Nir	10	PARP	0.544877	0.006
**464**	7-KB-K2-Ni	1	PARP	0.499196	0.014
**465**	3-KB-A19-D	1000	CDK	0.554191	0
**466**	3-KB-B19-D	100	CDK	0.55233	0.001
**467**	3-KB-B23-A	2500	CDK	0.356447	0.072
**468**	3-KB-C19-D	10	CDK	0.527853	0
**469**	3-KB-C23-A	250	CDK	0.443896	0.002
**470**	3-KB-D19-D	1	CDK	0.297814	0.136
**471**	3-KB-D23-A	25	CDK	0.346084	0.028
**472**	3-KB-E19-D	0.1	CDK	0.480842	0
**473**	3-KB-E23-A	2.5	CDK	0.362703	0.104
**474**	3-KB-F23-A	0.25	CDK	0.052074	0.976
**475**	3-KB-K17-P	1	CDK	0.429237	0.006
**476**	3-KB-L19-R	1	CDK	0.402042	0.006
**477**	3-KB-M17-I	10	CDK	0.473371	0
**478**	3-KB-M19-I	10	CDK	0.382123	0.054
**479**	3-KB-N17-F	100	CDK	0.466605	0.003
**480**	3-KB-N19-F	100	CDK	0.509013	0
**481**	3-KB-O17-F	1000	CDK	0.293403	0.543
**482**	3-KB-O19-F	1000	CDK	0.451902	0.002
**483**	3-KB-P17-P	10000	CDK	0.526208	0
**484**	3-KB-P19-R	10000	CDK	0.358774	0.03
**485**	4-KB-A4-SN	10000	CDK	0.54933	0
**486**	4-KB-A8-Mi	10000	CDK	0.514799	0
**487**	4-KB-B4-SN	1000	CDK	0.539519	0
**488**	4-KB-B8-Mi	1000	CDK	0.523486	0

**489**	4-KB-C4-SN	100	CDK	0.462296	0.005
**490**	4-KB-C8-Mi	100	CDK	0.426681	0.012
**491**	4-KB-D4-SN	10	CDK	0.319551	0.158
**492**	4-KB-D8-Mi	10	CDK	0.2421	0.351
**493**	4-KB-E4-SN	1	CDK	0.071693	0.987
**494**	4-KB-E8-Mi	1	CDK	0.233858	0.332
**495**	4-KB-F4-Sel	10000	CDK	0.362424	0.074
**496**	4-KB-F22-A	10000	CDK	0.54607	0
**497**	4-KB-G4-Se	1000	CDK	0.320962	0.158
**498**	4-KB-G22-A	1000	CDK	0.548748	0
**499**	4-KB-H4-Se	100	CDK	0.200261	0.579
**500**	4-KB-H22-A	100	CDK	0.373678	0.04
**501**	4-KB-I4-Seli	10	CDK	0.114386	0.762
**502**	4-KB-I22-Al	10	CDK	0.400224	0.007
**503**	4-KB-J4-Sel	1	CDK	0.090014	0.854
**504**	4-KB-J22-Al	1	CDK	0.310157	0.138
**505**	5-KB-A19-A	10000	CDK	0.527275	0
**506**	5-KB-B19-A	1000	CDK	0.428334	0.007
**507**	5-KB-C19-A	100	CDK	0.349608	0.09
**508**	5-KB-D19-A	10	CDK	0.319442	0.095
**509**	5-KB-E19-A	1	CDK	0.325721	0.096
**510**	5-KB-K17-A	1	CDK	0.341243	0.055
**511**	5-KB-M17-A	10	CDK	0.351255	0.21
**512**	5-KB-N17-A	100	CDK	0.438122	0.007
**513**	5-KB-O17-A	1000	CDK	0.534111	0
**514**	5-KB-P17-A	10000	CDK	0.535168	0
**515**	6-KB-A17-S	1000	CDK	0.465526	0.001
**516**	6-KB-B17-S	100	CDK	0.416598	0.032
**517**	6-KB-C17-S	10	CDK	0.381178	0.058
**518**	6-KB-D17-S	1	CDK	0.073794	0.95
**519**	6-KB-E17-S	0.1	CDK	0.404718	0.006
**520**	6-KB-L15-T	1	CDK	0.193056	0.662
**521**	6-KB-M15-T	10	CDK	0.335016	0.054
**522**	6-KB-N15-T	100	CDK	0.463732	0.006
**523**	6-KB-O15-T	1000	CDK	0.532992	0

**524**	6-KB-P15-T	10000	CDK	0.525895	0.001
**525**	7-KB-A21-d	10000	BET	0.516029	0
**526**	7-KB-A22-P	30000	BET	0.71894	0
**527**	7-KB-B21-d	1000	BET	0.729327	0
**528**	7-KB-B22-P	3000	BET	0.715226	0
**529**	7-KB-C21-d	100	BET	0.679245	0
**530**	7-KB-C22-P	300	BET	0.728183	0
**531**	7-KB-D21-c	10	BET	0.687731	0
**532**	7-KB-D22-F	30	BET	0.278002	0.396
**533**	7-KB-E21-d	1	BET	0.648339	0
**534**	7-KB-E22-P	3	BET	0.409748	0.036
**535**	7-KB-G10-E	10000	BET	0.701399	0
**536**	7-KB-G15-I-	10000	BET	0.671019	0
**537**	7-KB-H10-E	1000	BET	0.694781	0
**538**	7-KB-H15-I-	1000	BET	0.712853	0
**539**	7-KB-I10-Bi	100	BET	0.795375	0
**540**	7-KB-I15-I-I	100	BET	0.811404	0
**541**	7-KB-J10-Bi	10	BET	0.421209	0
**542**	7-KB-J15-I-I	10	BET	0.529667	0
**543**	7-KB-K10-B	1	BET	0.072094	1
**544**	7-KB-K13-N	1	BET	0.718979	0
**545**	7-KB-K15-I-	1	BET	0.393146	0.004
**546**	7-KB-L12-M	1	BET	0.751005	0
**547**	7-KB-L13-M	10	BET	0.689601	0
**548**	7-KB-L20-J(	1	BET	0.284171	0.384
**549**	7-KB-L23-A	0.03	BET	0.588258	0
**550**	7-KB-M12-I	10	BET	0.601873	0
**551**	7-KB-M13-I	100	BET	0.715358	0
**552**	7-KB-M20-J	10	BET	0.264821	0.237
**553**	7-KB-M23-/	0.3	BET	0.602021	0
**554**	7-KB-N12-N	100	BET	0.610564	0
**555**	7-KB-N13-N	1000	BET	0.661851	0
**556**	7-KB-N20-J	100	BET	0.743812	0
**557**	7-KB-N23-A	3	BET	0.741301	0
**558**	7-KB-O12-N	1000	BET	0.71	0

**559**	7-KB-O20-J	1000	BET	0.726248	0
**560**	7-KB-O23-A	30	BET	0.73001	0
**561**	7-KB-P12-N	10000	BET	0.732032	0
**562**	7-KB-P13-N	10000	BET	0.658832	0
**563**	7-KB-P20-J	10000	BET	0.732677	0
**564**	7-KB-P23-A	300	BET	0.701113	0
**565**	8-KB-K22-C	1	BET	0.106011	0.984
**566**	8-KB-L22-C	10	BET	0.24384	0.573
**567**	8-KB-M22-(	100	BET	0.644398	0
**568**	8-KB-N22-C	1000	BET	0.239491	0.611
**569**	8-KB-O22-C	10000	BET	0.438157	0.024
**570**	1-KB-A3-Vo	10000	HDAC	0.435659	0.026
**571**	1-KB-B3-Vo	1000	HDAC	0.411933	0.001
**572**	1-KB-C3-Vo	100	HDAC	0.204639	0.594
**573**	1-KB-D3-Vc	10	HDAC	0.151497	0.512
**574**	1-KB-E3-Vo	1	HDAC	0.190731	0.566
**575**	1-KB-L12-R	0.1	HDAC	0.271265	0.282
**576**	1-KB-M12-I	1	HDAC	0.430188	0
**577**	1-KB-N12-R	10	HDAC	0.443232	0.005
**578**	1-KB-O12-R	100	HDAC	0.407266	0.019
**579**	1-KB-P12-R	1000	HDAC	0.378588	0.067
**580**	3-KB-A4-Pa	1000	HDAC	0.440959	0.012
**581**	3-KB-B4-Pa	100	HDAC	0.509403	0
**582**	3-KB-C4-Pa	10	HDAC	0.514496	0
**583**	3-KB-D4-Pa	1	HDAC	0.363339	0
**584**	3-KB-E4-Pa	0.1	HDAC	0.057329	0.969
**585**	3-KB-F7-Qu	1000	HDAC	0.451073	0.004
**586**	3-KB-G7-Qu	100	HDAC	0.139388	0.575
**587**	3-KB-G12-V	1000000	HDAC	0.09568	0.962
**588**	3-KB-H7-Qu	10	HDAC	0.175537	0.381
**589**	3-KB-H12-V	100000	HDAC	0.144161	0.378
**590**	3-KB-I7-Qui	1	HDAC	0.309546	0.011
**591**	3-KB-I12-Va	10000	HDAC	0.251617	0.128
**592**	3-KB-J7-Qu	0.1	HDAC	0.153979	0.874
**593**	3-KB-J12-Va	1000	HDAC	0.244689	0.241

**594**	3-KB-K3-Be	1	HDAC	0.125044	0.462
**595**	3-KB-K12-V	100	HDAC	0.088047	0.581
**596**	3-KB-L3-Bel	10	HDAC	0.380243	0
**597**	3-KB-M3-Be	100	HDAC	0.356456	0.05
**598**	3-KB-N3-Be	1000	HDAC	0.06359	0.995
**599**	3-KB-O3-Be	10000	HDAC	0.149765	0.968
**600**	7-KB-A5-Mc	10000	HDAC	0.351088	0.185
**601**	7-KB-A7-CL	10000	HDAC	0.306105	0.386
**602**	7-KB-A9-Gi	1000	HDAC	0.508738	0
**603**	7-KB-A12-R	10000	HDAC	0.466147	0
**604**	7-KB-B5-Mc	1000	HDAC	0.482134	0
**605**	7-KB-B7-CL	1000	HDAC	0.429547	0.011
**606**	7-KB-B12-R	1000	HDAC	0.426553	0
**607**	7-KB-C5-Mc	100	HDAC	0.329185	0.004
**608**	7-KB-C7-CL	100	HDAC	0.493895	0
**609**	7-KB-C9-Gi	100	HDAC	0.384097	0.001
**610**	7-KB-D7-CL	10	HDAC	0.452348	0
**611**	7-KB-D9-Gi	10	HDAC	0.370609	0
**612**	7-KB-D12-F	100	HDAC	0.294849	0
**613**	7-KB-E5-Mc	10	HDAC	0.365834	0.008
**614**	7-KB-E7-CU	1	HDAC	0.408553	0.001
**615**	7-KB-E9-Gi	1	HDAC	0.277891	0.06
**616**	7-KB-E12-R	10	HDAC	0.360339	0.001
**617**	7-KB-F5-Mc	1	HDAC	0.117762	0.997
**618**	7-KB-F7-Re	10000	HDAC	0.495203	0
**619**	7-KB-F9-Gi	0.1	HDAC	0.221717	0.147
**620**	7-KB-F12-R	1	HDAC	0.229234	0.047
**621**	7-KB-F19-P	10000	HDAC	0.286003	0.393
**622**	7-KB-G7-Re	1000	HDAC	0.403388	0.001
**623**	7-KB-G19-F	1000	HDAC	0.127417	0.993
**624**	7-KB-H7-Re	100	HDAC	0.397358	0
**625**	7-KB-I7-Res	10	HDAC	0.330685	0.002
**626**	7-KB-I19-P(	100	HDAC	0.227003	0.281
**627**	7-KB-J7-Res	1	HDAC	0.413397	0.001
**628**	7-KB-J19-P	10	HDAC	0.364461	0.003

**629**	7-KB-K4-En	1	HDAC	0.347264	0
**630**	7-KB-K11-A	1	HDAC	0.278559	0.005
**631**	7-KB-K18-T	1	HDAC	0.345058	0.015
**632**	7-KB-K19-P	1	HDAC	0.41747	0
**633**	7-KB-L2-Ta	0.1	HDAC	0.111484	0.982
**634**	7-KB-L4-En	10	HDAC	0.238373	0.153
**635**	7-KB-L5-Pra	1	HDAC	0.387429	0
**636**	7-KB-L8-Ab	1	HDAC	0.418768	0.003
**637**	7-KB-L10-T	1	HDAC	0.346843	0
**638**	7-KB-L11-A	10	HDAC	0.32737	0.002
**639**	7-KB-L14-T	1	HDAC	0.350575	0.002
**640**	7-KB-L16-R	1	HDAC	0.263491	0.003
**641**	7-KB-L18-T	10	HDAC	0.071361	0.999
**642**	7-KB-M2-Ta	1	HDAC	0.156092	0.783
**643**	7-KB-M5-Pr	10	HDAC	0.386701	0.001
**644**	7-KB-M8-Al	10	HDAC	0.350457	0
**645**	7-KB-M10-	10	HDAC	0.322928	0.265
**646**	7-KB-M11-	100	HDAC	0.3675	0.011
**647**	7-KB-M14-	10	HDAC	0.269716	0.37
**648**	7-KB-M16-	10	HDAC	0.476698	0
**649**	7-KB-M18-	100	HDAC	0.257581	0.297
**650**	7-KB-N2-Ta	10	HDAC	0.114248	0.952
**651**	7-KB-N4-En	100	HDAC	0.420141	0
**652**	7-KB-N5-Pr	100	HDAC	0.371807	0.004
**653**	7-KB-N8-Ab	100	HDAC	0.497135	0
**654**	7-KB-N10-T	100	HDAC	0.273037	0.006
**655**	7-KB-N14-T	100	HDAC	0.290422	0.061
**656**	7-KB-N16-R	100	HDAC	0.173742	0.367
**657**	7-KB-N18-T	1000	HDAC	0.247823	0.012
**658**	7-KB-O2-Ta	100	HDAC	0.136429	0.967
**659**	7-KB-O4-En	1000	HDAC	0.533207	0
**660**	7-KB-O5-Pr	1000	HDAC	0.528616	0
**661**	7-KB-O8-Ab	1000	HDAC	0.510839	0
**662**	7-KB-O10-T	1000	HDAC	0.432836	0
**663**	7-KB-O11-A	1000	HDAC	0.465572	0

**664**	7-KB-O14-T	1000	HDAC	0.175834	0.525
**665**	7-KB-O16-F	1000	HDAC	0.456696	0
**666**	7-KB-P2-Ta	1000	HDAC	0.21552	0.168
**667**	7-KB-P4-En	10000	HDAC	0.524479	0
**668**	7-KB-P5-Pr	10000	HDAC	0.440686	0.016
**669**	7-KB-P8-Ab	10000	HDAC	0.224388	0.704
**670**	7-KB-P10-T	10000	HDAC	0.53435	0
**671**	7-KB-P11-A	10000	HDAC	0.479466	0
**672**	7-KB-P14-T	10000	HDAC	0.063541	0.967
**673**	7-KB-P16-R	10000	HDAC	0.382121	0.002
**674**	7-KB-P18-T	10000	HDAC	0.347149	0.014
**0**	2-KW-A16-	10000	EGFR	0.253093	0.437
**1**	2-KW-A19-	10000	EGFR	0.345383	0.007
**2**	2-KW-B19-	1000	EGFR	0.347758	0.023
**3**	2-KW-C16-	1000	EGFR	0.123053	0.777
**4**	2-KW-C19-	100	EGFR	0.405795	0.001
**5**	2-KW-D16-	100	EGFR	0.206725	0.47
**6**	2-KW-D19-	10	EGFR	0.352581	0.005
**7**	2-KW-E16-	10	EGFR	0.323039	0.011
**8**	2-KW-E19-	1	EGFR	0.38957	0.001
**9**	2-KW-F16-	1	EGFR	0.089877	0.926
**10**	2-KW-K11-	0.1	EGFR	0.185132	0.733
**11**	2-KW-L11-	1	EGFR	0.32047	0.023
**12**	2-KW-L16-	0.25	EGFR	0.341515	0.015
**13**	2-KW-L19-	0.1	EGFR	0.385512	0
**14**	2-KW-M11-	10	EGFR	0.371381	0.005
**15**	2-KW-M16-	2.5	EGFR	0.373766	0.002
**16**	2-KW-M19-	1	EGFR	0.346249	0.014
**17**	2-KW-N16-	25	EGFR	0.378794	0.003
**18**	2-KW-N19-	10	EGFR	0.061565	0.97
**19**	2-KW-O11-	100	EGFR	0.379501	0
**20**	2-KW-O16-	250	EGFR	0.07899	0.913
**21**	2-KW-O19-	100	EGFR	0.409321	0.006
**22**	2-KW-P11-	1000	EGFR	0.278154	0.156
**23**	2-KW-P16-	2500	EGFR	0.35528	0.002

**24**	2-KW-P19-I	1000	EGFR	0.348745	0.008
**25**	3-KW-F21-I	10000	EGFR	0.314546	0.106
**26**	3-KW-G20-	1000	EGFR	0.297747	0.039
**27**	3-KW-G21-	1000	EGFR	0.437609	0
**28**	3-KW-H20-	100	EGFR	0.449543	0
**29**	3-KW-H21-	100	EGFR	0.310372	0.031
**30**	3-KW-I20-N	10	EGFR	0.350102	0.004
**31**	3-KW-I21-F	10	EGFR	0.155562	0.764
**32**	3-KW-J20-N	1	EGFR	0.428089	0
**33**	3-KW-J21-F	1	EGFR	0.361918	0.006
**34**	3-KW-K4-C	1	EGFR	0.136316	0.721
**35**	3-KW-K18-I	0.1	EGFR	0.396037	0
**36**	3-KW-K20-I	0.1	EGFR	0.282643	0.272
**37**	3-KW-L4-C	10	EGFR	0.118653	0.853
**38**	3-KW-L18-I	1	EGFR	0.370371	0.006
**39**	3-KW-M18-	10	EGFR	0.248973	0.175
**40**	3-KW-N4-C	100	EGFR	0.424371	0
**41**	3-KW-N18-	100	EGFR	0.370157	0.001
**42**	3-KW-O4-C	1000	EGFR	0.372271	0.014
**43**	3-KW-P4-C	10000	EGFR	0.084134	1
**44**	3-KW-P18-I	1000	EGFR	0.16544	0.704
**45**	4-KW-F13-S	1000	EGFR	0.290667	0.062
**46**	4-KW-G13-	100	EGFR	0.38423	0.001
**47**	4-KW-G16-	10000	EGFR	0.30902	0.034
**48**	4-KW-H13-	10	EGFR	0.29229	0.074
**49**	4-KW-H16-	1000	EGFR	0.287341	0.096
**50**	4-KW-I13-S	1	EGFR	0.180546	0.384
**51**	4-KW-I16-V	100	EGFR	0.100602	0.847
**52**	4-KW-J13-S	0.1	EGFR	0.074009	0.933
**53**	4-KW-J16-V	10	EGFR	0.079426	0.894
**54**	4-KW-K7-Ic	1	EGFR	0.099534	0.831
**55**	4-KW-K13-T	0.1	EGFR	0.2009	0.506
**56**	4-KW-K16-Y	1	EGFR	0.326273	0.009
**57**	4-KW-L7-Ic	10	EGFR	0.341706	0.014
**58**	4-KW-L13-T	1	EGFR	0.10665	0.858

**59**	4-KW-M7-I	100	EGFR	0.255566	0.148
**60**	4-KW-M13-	10	EGFR	0.378224	0
**61**	4-KW-N13-	100	EGFR	0.406119	0
**62**	4-KW-O7-Ic	1000	EGFR	0.255195	0.19
**63**	4-KW-P7-Ic	10000	EGFR	0.276116	0.078
**64**	4-KW-P13-1	1000	EGFR	0.341365	0.017
**65**	5-KW-F4-Pc	1000	EGFR	0.371707	0.006
**66**	5-KW-F7-AZ	1000	EGFR	0.139669	0.721
**67**	5-KW-G4-P	100	EGFR	0.348671	0.012
**68**	5-KW-G7-A	100	EGFR	0.369925	0.001
**69**	5-KW-H4-P	10	EGFR	0.175652	0.642
**70**	5-KW-H7-A	10	EGFR	0.263228	0.236
**71**	5-KW-I4-Pc	1	EGFR	0.314484	0.071
**72**	5-KW-I7-AZ	1	EGFR	0.320148	0.027
**73**	5-KW-J4-Pc	0.1	EGFR	0.140887	0.714
**74**	5-KW-J7-AZ	0.1	EGFR	0.401754	0.003
**75**	5-KW-K7-O	0.1	EGFR	0.121464	0.787
**76**	5-KW-L7-Ol	1	EGFR	0.431694	0
**77**	5-KW-M7-C	10	EGFR	0.287991	0.162
**78**	5-KW-O7-O	100	EGFR	0.332535	0.049
**79**	5-KW-P7-O	1000	EGFR	0.349987	0.011
**80**	2-KW-A15-I	2500	VEGFR	0.262892	0.065
**81**	2-KW-A17-I	10000	VEGFR	0.317342	0.108
**82**	2-KW-A20-1	10000	VEGFR	0.294588	0.082
**83**	2-KW-B15-I	250	VEGFR	0.199144	0.162
**84**	2-KW-B17-I	1000	VEGFR	0.264144	0.018
**85**	2-KW-B20-1	1000	VEGFR	0.278235	0.172
**86**	2-KW-C15-I	25	VEGFR	0.243035	0.083
**87**	2-KW-C17-I	100	VEGFR	0.278087	0.005
**88**	2-KW-D15-	2.5	VEGFR	0.308388	0.009
**89**	2-KW-D17-	10	VEGFR	0.293901	0.071
**90**	2-KW-D20-	100	VEGFR	0.242891	0.269
**91**	2-KW-E17-I	1	VEGFR	0.220537	0.297
**92**	2-KW-E20-1	10	VEGFR	0.180393	0.626
**93**	2-KW-F13-/	10000	VEGFR	0.116401	0.988

**94**	2-KW-F15-I	0.25	VEGFR	0.160919	0.505
**95**	2-KW-F19-I	10000	VEGFR	0.187504	0.629
**96**	2-KW-F20-T	1	VEGFR	0.138045	0.58
**97**	2-KW-F21-\	10000	VEGFR	0.221659	0.893
**98**	2-KW-G10-	10000	VEGFR	0.298743	0.027
**99**	2-KW-G13-	1000	VEGFR	0.162342	0.775
**100**	2-KW-G19-	1000	VEGFR	0.328336	0.036
**101**	2-KW-G21-	1000	VEGFR	0.279068	0.193
**102**	2-KW-H10-	1000	VEGFR	0.339208	0.013
**103**	2-KW-H13-	100	VEGFR	0.270006	0.099
**104**	2-KW-H21-	100	VEGFR	0.298317	0.052
**105**	2-KW-I10-A	100	VEGFR	0.286751	0.034
**106**	2-KW-I13-A	10	VEGFR	0.254207	0.192
**107**	2-KW-I19-F	100	VEGFR	0.221358	0.246
**108**	2-KW-I21-\	10	VEGFR	0.192232	0.594
**109**	2-KW-J10-A	10	VEGFR	0.229164	0.226
**110**	2-KW-J13-A	1	VEGFR	0.32781	0.008
**111**	2-KW-J19-F	10	VEGFR	0.167874	0.747
**112**	2-KW-J21-\	1	VEGFR	0.328758	0.04
**113**	2-KW-K10-,	1	VEGFR	0.18888	0.605
**114**	2-KW-K13-'	0.1	VEGFR	0.278563	0.051
**115**	2-KW-K17-I	1	VEGFR	0.238457	0.207
**116**	2-KW-K19-I	1	VEGFR	0.241497	0.358
**117**	2-KW-L12-S	0.1	VEGFR	0.335331	0.02
**118**	2-KW-L13-\	1	VEGFR	0.203741	0.31
**119**	2-KW-L21-(	0.1	VEGFR	0.185146	0.63
**120**	2-KW-M12-	1	VEGFR	0.176564	0.529
**121**	2-KW-M13-	10	VEGFR	0.417946	0
**122**	2-KW-M17-	10	VEGFR	0.222229	0.388
**123**	2-KW-M21-	1	VEGFR	0.249857	0.402
**124**	2-KW-N12-	10	VEGFR	0.335592	0.004
**125**	2-KW-N13-	100	VEGFR	0.118868	0.744
**126**	2-KW-N17-	100	VEGFR	0.406237	0
**127**	2-KW-N21-	10	VEGFR	0.300007	0.081
**128**	2-KW-O12-	100	VEGFR	0.175456	0.648

**129**	2-KW-O17-	1000	VEGFR	0.215283	0.434
**130**	2-KW-O21-	100	VEGFR	0.207986	0.623
**131**	2-KW-P12-!	1000	VEGFR	0.25146	0.133
**132**	2-KW-P13-'	1000	VEGFR	0.261454	0.064
**133**	2-KW-P17-I	10000	VEGFR	0.249009	0.226
**134**	2-KW-P21-(	1000	VEGFR	0.357007	0.012
**135**	3-KW-A3-C	1000	VEGFR	0.265362	0.031
**136**	3-KW-A6-F	1000	VEGFR	0.128619	0.975
**137**	3-KW-A18-I	1000	VEGFR	0.285428	0.01
**138**	3-KW-B3-C	100	VEGFR	0.248952	0.071
**139**	3-KW-B6-F	100	VEGFR	0.236927	0.09
**140**	3-KW-B18-I	100	VEGFR	0.313346	0
**141**	3-KW-C3-C	10	VEGFR	0.240694	0.351
**142**	3-KW-C6-F	10	VEGFR	0.22292	0.383
**143**	3-KW-C18-I	10	VEGFR	0.211447	0.44
**144**	3-KW-D3-C	1	VEGFR	0.16206	0.869
**145**	3-KW-D6-F	1	VEGFR	0.142815	0.415
**146**	3-KW-D18-	1	VEGFR	0.22036	0.404
**147**	3-KW-E3-C	0.1	VEGFR	0.166517	0.361
**148**	3-KW-E6-F	0.1	VEGFR	0.263053	0.332
**149**	3-KW-E18-I	0.1	VEGFR	0.270669	0.103
**150**	3-KW-F18-I	1000	VEGFR	0.172619	0.776
**151**	3-KW-G18-	100	VEGFR	0.225678	0.427
**152**	3-KW-H18-	10	VEGFR	0.174801	0.642
**153**	3-KW-I18-E	1	VEGFR	0.308562	0.075
**154**	3-KW-J18-E	0.1	VEGFR	0.160659	0.597
**155**	4-KW-A12-I	10000	VEGFR	0.195326	0.916
**156**	4-KW-A15-(	2500	VEGFR	0.318509	0.003
**157**	4-KW-A20-I	10000	VEGFR	0.136684	0.864
**158**	4-KW-B12-I	1000	VEGFR	0.185698	0.732
**159**	4-KW-B15-(	250	VEGFR	0.230928	0.041
**160**	4-KW-B20-I	1000	VEGFR	0.220871	0.124
**161**	4-KW-C15-(	25	VEGFR	0.237996	0.046
**162**	4-KW-D12-	100	VEGFR	0.242718	0.229
**163**	4-KW-D15-	2.5	VEGFR	0.222898	0.132

**164**	4-KW-D20-	100	VEGFR	0.20055	0.233
**165**	4-KW-E12-I	10	VEGFR	0.218022	0.046
**166**	4-KW-E20-I	10	VEGFR	0.181244	0.346
**167**	4-KW-F12-I	1	VEGFR	0.225737	0.175
**168**	4-KW-F15-(	0.25	VEGFR	0.212553	0.181
**169**	4-KW-F20-I	1	VEGFR	0.227127	0.071
**170**	4-KW-L16-⁻	1	VEGFR	0.155833	0.531
**171**	4-KW-M16-	10	VEGFR	0.248518	0.021
**172**	4-KW-N16-	100	VEGFR	0.202676	0.191
**173**	4-KW-O16-	1000	VEGFR	0.32708	0.003
**174**	4-KW-P16-⁻	10000	VEGFR	0.275756	0.124
**175**	2-KW-L10-I	1	PI3K	0.155655	0.738
**176**	2-KW-M10-	10	PI3K	0.275436	0.034
**177**	2-KW-N10-	100	PI3K	0.210258	0.301
**178**	2-KW-O10-	1000	PI3K	0.20031	0.405
**179**	2-KW-P10-I	10000	PI3K	0.310713	0.009
**180**	3-KW-A16-I	2500	PI3K	0.172131	0.246
**181**	3-KW-C16-I	250	PI3K	0.117719	0.644
**182**	3-KW-D16-	25	PI3K	0.139408	0.407
**183**	3-KW-E16-I	2.5	PI3K	0.219221	0.297
**184**	3-KW-F16-I	0.25	PI3K	0.121695	0.558
**185**	3-KW-F17-I	100000	PI3K	0.270925	0.401
**186**	3-KW-F19-I	500	PI3K	0.247923	0.083
**187**	3-KW-G17-	10000	PI3K	0.148241	0.791
**188**	3-KW-G19-	50	PI3K	0.20777	0.433
**189**	3-KW-H17-	1000	PI3K	0.100812	0.975
**190**	3-KW-I17-N	100	PI3K	0.100694	0.914
**191**	3-KW-I19-D	5	PI3K	0.17484	0.344
**192**	3-KW-J17-N	10	PI3K	0.146562	0.813
**193**	3-KW-J19-D	0.5	PI3K	0.17491	0.686
**194**	3-KW-K19-I	0.05	PI3K	0.122912	0.811
**195**	3-KW-L8-Pi	1	PI3K	0.099948	0.959
**196**	3-KW-L21-⁻	0.1	PI3K	0.301596	0.002
**197**	3-KW-M8-P	10	PI3K	0.206168	0.264
**198**	3-KW-M21-	1	PI3K	0.147292	0.736

**199**	3-KW-N8-P	100	PI3K	0.366243	0.003
**200**	3-KW-N21-	10	PI3K	0.172834	0.296
**201**	3-KW-O8-P	1000	PI3K	0.387515	0
**202**	3-KW-O21-	100	PI3K	0.304607	0.009
**203**	3-KW-P8-Pi	10000	PI3K	0.380732	0.001
**204**	3-KW-P21-'	1000	PI3K	0.407161	0
**205**	4-KW-A19-/	2500	PI3K	0.268701	0.012
**206**	4-KW-B19-/	250	PI3K	0.173812	0.1
**207**	4-KW-C19-/	25	PI3K	0.162571	0.184
**208**	4-KW-D19-	2.5	PI3K	0.211646	0.053
**209**	4-KW-E19-/	0.25	PI3K	0.304127	0.002
**210**	4-KW-F14-I	1000	PI3K	0.278392	0.326
**211**	4-KW-G2-T	2500	PI3K	0.312427	0.003
**212**	4-KW-G5-S	10000	PI3K	0.255486	0.152
**213**	4-KW-G14-	100	PI3K	0.27256	0.095
**214**	4-KW-G20-	10000	PI3K	0.368086	0.008
**215**	4-KW-H2-T	250	PI3K	0.386071	0
**216**	4-KW-H5-S	1000	PI3K	0.348464	0
**217**	4-KW-H14-	10	PI3K	0.324496	0.007
**218**	4-KW-H20-	1000	PI3K	0.388851	0
**219**	4-KW-I2-TG	25	PI3K	0.162533	0.362
**220**	4-KW-I5-So	100	PI3K	0.250638	0.048
**221**	4-KW-I14-N	1	PI3K	0.283089	0.004
**222**	4-KW-I20-E	100	PI3K	0.207451	0.064
**223**	4-KW-J2-TG	2.5	PI3K	0.097762	0.883
**224**	4-KW-J5-Sc	10	PI3K	0.304897	0.037
**225**	4-KW-J20-E	10	PI3K	0.273588	0.037
**226**	4-KW-K2-T	0.25	PI3K	0.21331	0.169
**227**	4-KW-K4-D	0.1	PI3K	0.178733	0.221
**228**	4-KW-K5-S	1	PI3K	0.325655	0.03
**229**	4-KW-K14-I	0.1	PI3K	0.133257	0.643
**230**	4-KW-K20-I	1	PI3K	0.370777	0
**231**	4-KW-L4-D	1	PI3K	0.296518	0.039
**232**	4-KW-L14-(	0.1	PI3K	0.231931	0.046
**233**	4-KW-L15-'	1	PI3K	0.180429	0.101

**234**	4-KW-L21-(	0.1	PI3K	0.342068	0.003
**235**	4-KW-M14-	1	PI3K	0.26422	0.002
**236**	4-KW-M15-	10	PI3K	0.209148	0.092
**237**	4-KW-M21-	1	PI3K	0.298775	0.019
**238**	4-KW-N4-D	10	PI3K	0.109818	0.972
**239**	4-KW-N14-	10	PI3K	0.143785	0.827
**240**	4-KW-N15-	100	PI3K	0.222309	0.034
**241**	4-KW-N21-	10	PI3K	0.419154	0
**242**	4-KW-O4-D	100	PI3K	0.188302	0.655
**243**	4-KW-O14-	100	PI3K	0.258581	0.125
**244**	4-KW-O15-	1000	PI3K	0.322783	0
**245**	4-KW-O21-	100	PI3K	0.37557	0.001
**246**	4-KW-P4-D	1000	PI3K	0.230577	0.355
**247**	4-KW-P14-(	1000	PI3K	0.371225	0.005
**248**	4-KW-P15-7	10000	PI3K	0.341633	0
**249**	4-KW-P21-(	1000	PI3K	0.35982	0.008
**250**	5-KW-A6-LY	2500	PI3K	0.207445	0.506
**251**	5-KW-A7-Al	1000	PI3K	0.202667	0.047
**252**	5-KW-A16-,	2500	PI3K	0.288644	0.029
**253**	5-KW-A17-I	10000	PI3K	0.171985	0.263
**254**	5-KW-B6-LY	250	PI3K	0.169292	0.765
**255**	5-KW-B7-Al	100	PI3K	0.159108	0.288
**256**	5-KW-B17-I	1000	PI3K	0.185055	0.159
**257**	5-KW-C6-LY	25	PI3K	0.157513	0.568
**258**	5-KW-C7-Al	10	PI3K	0.20107	0.086
**259**	5-KW-C16-,	250	PI3K	0.179742	0.102
**260**	5-KW-C17-I	100	PI3K	0.216167	0.023
**261**	5-KW-D6-LY	2.5	PI3K	0.098833	0.974
**262**	5-KW-D7-A	1	PI3K	0.259556	0.056
**263**	5-KW-D16-	25	PI3K	0.171954	0.176
**264**	5-KW-D17-	10	PI3K	0.199604	0.353
**265**	5-KW-E6-LY	0.25	PI3K	0.185859	0.161
**266**	5-KW-E7-Al	0.1	PI3K	0.226407	0.252
**267**	5-KW-E16-/	2.5	PI3K	0.080986	0.986
**268**	5-KW-E17-I	1	PI3K	0.141824	0.623

**269**	5-KW-F11-(	10000	PI3K	0.260489	0.05
**270**	5-KW-F16-/	0.25	PI3K	0.147124	0.754
**271**	5-KW-G9-S	10000	PI3K	0.330498	0.009
**272**	5-KW-G11-	1000	PI3K	0.236081	0.008
**273**	5-KW-H9-S	1000	PI3K	0.225276	0.211
**274**	5-KW-H11-	100	PI3K	0.262792	0.068
**275**	5-KW-I9-Se	100	PI3K	0.163996	0.525
**276**	5-KW-I11-(	10	PI3K	0.180872	0.606
**277**	5-KW-J9-Se	10	PI3K	0.213579	0.281
**278**	5-KW-J11-(	1	PI3K	0.199917	0.388
**279**	5-KW-K9-S	1	PI3K	0.173877	0.272
**280**	5-KW-L14-/	0.1	PI3K	0.152646	0.782
**281**	5-KW-L20-:	1	PI3K	0.137711	0.926
**282**	5-KW-L23-(	0.1	PI3K	0.154666	0.739
**283**	5-KW-M14-	1	PI3K	0.188424	0.528
**284**	5-KW-M20-	10	PI3K	0.17428	0.722
**285**	5-KW-M23-	1	PI3K	0.161045	0.631
**286**	5-KW-N14-	10	PI3K	0.16332	0.857
**287**	5-KW-N20-	100	PI3K	0.250524	0.273
**288**	5-KW-N23-	10	PI3K	0.28513	0.046
**289**	5-KW-O14-	100	PI3K	0.197573	0.129
**290**	5-KW-O20-	1000	PI3K	0.300982	0.017
**291**	5-KW-O23-	100	PI3K	0.32004	0.011
**292**	5-KW-P14-/	1000	PI3K	0.238637	0.331
**293**	5-KW-P20-:	10000	PI3K	0.330925	0.005
**294**	5-KW-P23-(	1000	PI3K	0.362152	0.002
**295**	6-KW-A8-T(	10000	PI3K	0.311202	0.005
**296**	6-KW-B8-T(	1000	PI3K	0.334096	0.007
**297**	6-KW-C8-T(	100	PI3K	0.138613	0.719
**298**	6-KW-D8-T(	10	PI3K	0.065749	0.998
**299**	6-KW-E8-T(	1	PI3K	0.237012	0.089
**300**	6-KW-L6-G	1	PI3K	0.074185	0.997
**301**	6-KW-M6-(	10	PI3K	0.164736	0.668
**302**	6-KW-N6-G	100	PI3K	0.158154	0.808
**303**	6-KW-O6-G	1000	PI3K	0.311507	0.011

**304**	6-KW-P6-G	10000	PI3K	0.374557	0
**305**	1-KW-F11-/	10000	Topoisome	0.631307	0
**306**	1-KW-G11-	1000	Topoisome	0.633038	0
**307**	1-KW-G20-	1000	Topoisome	0.474665	0
**308**	1-KW-H11-	100	Topoisome	0.544786	0
**309**	1-KW-H20-	100	Topoisome	0.605505	0
**310**	1-KW-I11-A	10	Topoisome	0.508478	0
**311**	1-KW-I20-E	10	Topoisome	0.470885	0
**312**	1-KW-J11-A	1	Topoisome	0.514996	0
**313**	1-KW-J20-E	1	Topoisome	0.198467	0.751
**314**	1-KW-K11-S	1	Topoisome	0.539361	0
**315**	1-KW-K20-I	0.1	Topoisome	0.129466	0.954
**316**	1-KW-L11-S	10	Topoisome	0.633394	0
**317**	1-KW-L14-T	1	Topoisome	0.478738	0
**318**	1-KW-M11-	100	Topoisome	0.529214	0
**319**	1-KW-M14-	10	Topoisome	0.483578	0
**320**	1-KW-N14-	100	Topoisome	0.62453	0
**321**	1-KW-O11-	1000	Topoisome	0.439807	0
**322**	1-KW-O14-	1000	Topoisome	0.56604	0
**323**	1-KW-P11-S	10000	Topoisome	0.321101	0.29
**324**	1-KW-P14-T	10000	Topoisome	0.401867	0.013
**325**	3-KW-A11-I	10000	Topoisome	0.593849	0
**326**	3-KW-B11-I	1000	Topoisome	0.597289	0
**327**	3-KW-C11-I	100	Topoisome	0.440524	0
**328**	3-KW-D11-	10	Topoisome	0.43988	0
**329**	3-KW-E11-I	1	Topoisome	0.241169	0.214
**330**	3-KW-G9-D	1000	Topoisome	0.393357	0.035
**331**	3-KW-G10-	10000	Topoisome	0.236616	0.247
**332**	3-KW-H9-D	100	Topoisome	0.593759	0
**333**	3-KW-H10-	1000	Topoisome	0.239734	0.283
**334**	3-KW-I9-Da	10	Topoisome	0.528383	0
**335**	3-KW-I10-T	100	Topoisome	0.301226	0.062
**336**	3-KW-J9-Da	1	Topoisome	0.467174	0
**337**	3-KW-J10-T	10	Topoisome	0.333663	0.053
**338**	3-KW-K7-Ic	0.1	Topoisome	0.447952	0.001

**339**	3-KW-K9-D	0.1	Topoisome	0.434371	0.003
**340**	3-KW-K10-‑	1	Topoisome	0.198059	0.445
**341**	3-KW-L6-D	0.1	Topoisome	0.037369	1
**342**	3-KW-L7-Id	1	Topoisome	0.161658	0.781
**343**	3-KW-L9-Va	0.5	Topoisome	0.478926	0
**344**	3-KW-L10-I	0.1	Topoisome	0.294703	0.111
**345**	3-KW-L16-I	1	Topoisome	0.31582	0.06
**346**	3-KW-M6-D	1	Topoisome	0.369051	0.004
**347**	3-KW-M7-I	10	Topoisome	0.200112	0.757
**348**	3-KW-M9-V	5	Topoisome	0.383617	0.014
**349**	3-KW-M10-	1	Topoisome	0.063393	0.987
**350**	3-KW-M16-	10	Topoisome	0.428961	0
**351**	3-KW-N6-D	10	Topoisome	0.425538	0.004
**352**	3-KW-N9-V	50	Topoisome	0.566827	0
**353**	3-KW-N10-	10	Topoisome	0.396867	0.007
**354**	3-KW-N16-	100	Topoisome	0.207761	0.283
**355**	3-KW-O6-D	100	Topoisome	0.610291	0
**356**	3-KW-O7-I	100	Topoisome	0.587713	0
**357**	3-KW-O9-V	500	Topoisome	0.609759	0
**358**	3-KW-O10-	100	Topoisome	0.598573	0
**359**	3-KW-O16-	1000	Topoisome	0.129952	0.789
**360**	3-KW-P6-D	1000	Topoisome	0.521106	0
**361**	3-KW-P7-Id	1000	Topoisome	0.333919	0.184
**362**	3-KW-P9-V	5000	Topoisome	0.604578	0
**363**	3-KW-P10-I	1000	Topoisome	0.513739	0
**364**	3-KW-P16-I	10000	Topoisome	0.13281	0.921
**365**	1-KW-A10-'	10000	Mitotic	0.602864	0
**366**	1-KW-A13-I	1000	Mitotic	0.576963	0
**367**	1-KW-A18-I	1000	Mitotic	0.629868	0
**368**	1-KW-B10-'	1000	Mitotic	0.629787	0
**369**	1-KW-B13-I	100	Mitotic	0.067608	0.997
**370**	1-KW-B18-I	100	Mitotic	0.570086	0
**371**	1-KW-C10-'	100	Mitotic	0.663802	0
**372**	1-KW-C13-I	10	Mitotic	0.487455	0.001
**373**	1-KW-C18-I	10	Mitotic	0.562497	0

**374**	1-KW-D10-	10	Mitotic	0.390248	0.019
**375**	1-KW-D13-	1	Mitotic	0.226778	0.406
**376**	1-KW-D18-	1	Mitotic	0.409676	0.013
**377**	1-KW-E10-\	1	Mitotic	0.490666	0
**378**	1-KW-E13-I	0.1	Mitotic	0.282277	0.35
**379**	1-KW-E18-I	0.1	Mitotic	0.28658	0.214
**380**	1-KW-F13-\	1000	Mitotic	0.595793	0
**381**	1-KW-G13-	100	Mitotic	0.327488	0.084
**382**	1-KW-G15-	1000	Mitotic	0.641027	0
**383**	1-KW-H13-	10	Mitotic	0.472702	0
**384**	1-KW-H15-	100	Mitotic	0.643805	0
**385**	1-KW-I13-\	1	Mitotic	0.504273	0
**386**	1-KW-I15-E	10	Mitotic	0.664123	0
**387**	1-KW-J13-\	0.1	Mitotic	0.116854	0.836
**388**	1-KW-J15-E	1	Mitotic	0.507038	0
**389**	1-KW-K7-Vi	0.1	Mitotic	0.532719	0
**390**	1-KW-K15-I	0.1	Mitotic	0.666515	0
**391**	1-KW-L7-Vi	1	Mitotic	0.432672	0.005
**392**	1-KW-L20-\	0.1	Mitotic	0.511573	0.001
**393**	1-KW-M7-\	10	Mitotic	0.478485	0
**394**	1-KW-M20-	1	Mitotic	0.417113	0.009
**395**	1-KW-N20-	10	Mitotic	0.467394	0
**396**	1-KW-O7-V	100	Mitotic	0.424283	0.005
**397**	1-KW-O20-	100	Mitotic	0.508204	0
**398**	1-KW-P7-Vi	1000	Mitotic	0.663809	0
**399**	1-KW-P20-\	1000	Mitotic	0.53037	0
**400**	3-KW-A7-D	1000	Mitotic	0.659631	0
**401**	3-KW-B7-D	100	Mitotic	0.652411	0
**402**	3-KW-C7-D	10	Mitotic	0.629843	0
**403**	3-KW-D7-D	1	Mitotic	0.128019	0.853
**404**	3-KW-E7-D	0.1	Mitotic	0.235765	0.925
**405**	6-KW-L19-/	1	Mitotic	0.435193	0.003
**406**	6-KW-M19-	10	Mitotic	0.301262	0.188
**407**	6-KW-N19-	100	Mitotic	0.253933	0.586
**408**	6-KW-O19-	1000	Mitotic	0.563806	0

**409**	6-KW-P19-/ 10000	Mitotic	0.574703	0
**410**	2-KW-A12-' 250	MEK1/2	0.518428	0.012
**411**	2-KW-B12-' 25	MEK1/2	0.36968	0.114
**412**	2-KW-D12- 2.5	MEK1/2	0.3516	0.076
**413**	2-KW-E12-' 0.25	MEK1/2	0.400017	0.039
**414**	2-KW-F12-' 2.5000000	MEK1/2	0.275355	0.547
**415**	2-KW-F14-(1000	MEK1/2	0.415202	0.026
**416**	2-KW-G14- 100	MEK1/2	0.516718	0.002
**417**	2-KW-H14- 10	MEK1/2	0.420127	0.01
**418**	2-KW-I14-C 1	MEK1/2	0.273065	0.368
**419**	2-KW-K14-(0.1	MEK1/2	0.165579	0.941
**420**	2-KW-L20-S 1	MEK1/2	0.267654	0.524
**421**	2-KW-M20- 10	MEK1/2	0.19123	0.87
**422**	2-KW-N20- 100	MEK1/2	0.193303	0.831
**423**	2-KW-O20- 1000	MEK1/2	0.304068	0.514
**424**	2-KW-P20-: 10000	MEK1/2	0.31678	0.285
**425**	4-KW-A10-I 1000	MEK1/2	0.487178	0.001
**426**	4-KW-A13-I 1000	MEK1/2	0.549163	0.001
**427**	4-KW-B10-I 100	MEK1/2	0.516457	0
**428**	4-KW-B13-I 100	MEK1/2	0.428523	0.007
**429**	4-KW-C10-I 10	MEK1/2	0.454094	0.004
**430**	4-KW-C13-I 10	MEK1/2	0.423388	0.006
**431**	4-KW-D10- 1	MEK1/2	0.509689	0
**432**	4-KW-D13- 1	MEK1/2	0.503162	0.002
**433**	4-KW-E10-I 0.1	MEK1/2	0.471422	0.005
**434**	4-KW-E13-I 0.1	MEK1/2	0.496389	0
**435**	4-KW-L19-(0.25	MEK1/2	0.326062	0.471
**436**	4-KW-M19- 2.5	MEK1/2	0.413952	0.504
**437**	4-KW-N19- 25	MEK1/2	0.519078	0
**438**	4-KW-O19- 250	MEK1/2	0.485952	0.004
**439**	4-KW-P19-(2500	MEK1/2	0.428633	0.026
**440**	1-KW-L2-OI 1	PARP	0.332697	0.18
**441**	1-KW-L6-Rı 1	PARP	0.537311	0.003
**442**	1-KW-M2-C 10	PARP	0.33389	0.368
**443**	1-KW-M6-F 10	PARP	0.23228	0.646

**444**	1-KW-N2-O	100	PARP	0.508565	0.008
**445**	1-KW-N6-R	100	PARP	0.495433	0.004
**446**	1-KW-O2-O	1000	PARP	0.518209	0.007
**447**	1-KW-O6-R	1000	PARP	0.423546	0.066
**448**	1-KW-P2-O	10000	PARP	0.469063	0.049
**449**	1-KW-P6-R	10000	PARP	0.428756	0.075
**450**	7-KW-A3-Ta	1000	PARP	0.511382	0.027
**451**	7-KW-B2-V	10000	PARP	0.581525	0
**452**	7-KW-B3-Ta	100	PARP	0.519291	0.013
**453**	7-KW-C2-V	1000	PARP	0.584724	0.003
**454**	7-KW-C3-Ta	10	PARP	0.660891	0
**455**	7-KW-D2-V	100	PARP	0.430415	0.027
**456**	7-KW-D3-Ta	1	PARP	0.254757	0.35
**457**	7-KW-E2-Ve	10	PARP	0.482492	0.074
**458**	7-KW-E3-Ta	0.1	PARP	0.229533	0.472
**459**	7-KW-F2-Ve	1	PARP	0.390485	0.055
**460**	7-KW-G2-N	10000	PARP	0.516524	0.022
**461**	7-KW-H2-N	1000	PARP	0.696391	0
**462**	7-KW-I2-Ni	100	PARP	0.457284	0.042
**463**	7-KW-J2-Ni	10	PARP	0.4035	0.038
**464**	7-KW-K2-N	1	PARP	0.346969	0.112
**465**	3-KW-A19-	1000	CDK	0.508659	0.004
**466**	3-KW-B19-	100	CDK	0.498518	0.003
**467**	3-KW-B23-	2500	CDK	0.471855	0.001
**468**	3-KW-C19-	10	CDK	0.454428	0.002
**469**	3-KW-C23-	250	CDK	0.47682	0
**470**	3-KW-D19-	1	CDK	0.41451	0.013
**471**	3-KW-D23-	25	CDK	0.348987	0.034
**472**	3-KW-E19-	0.1	CDK	0.317449	0.05
**473**	3-KW-E23-	2.5	CDK	0.266039	0.48
**474**	3-KW-F23-	0.25	CDK	0.146608	0.875
**475**	3-KW-K17-	1	CDK	0.379466	0.006
**476**	3-KW-L19-	1	CDK	0.479153	0.003
**477**	3-KW-M17-	10	CDK	0.410792	0.001
**478**	3-KW-M19-	10	CDK	0.344872	0.06

**479**	3-KW-N17- 100	CDK	0.391424	0.004
**480**	3-KW-N19- 100	CDK	0.410367	0
**481**	3-KW-O17- 1000	CDK	0.422988	0.002
**482**	3-KW-O19- 1000	CDK	0.427851	0
**483**	3-KW-P17-I 10000	CDK	0.41279	0.002
**484**	3-KW-P19-I 10000	CDK	0.401007	0.003
**485**	4-KW-A4-SI 10000	CDK	0.558496	0
**486**	4-KW-A8-M 10000	CDK	0.57392	0
**487**	4-KW-B4-SI 1000	CDK	0.541069	0
**488**	4-KW-B8-M 1000	CDK	0.422578	0.001
**489**	4-KW-C4-SI 100	CDK	0.435967	0.001
**490**	4-KW-C8-M 100	CDK	0.287605	0.108
**491**	4-KW-D4-SI 10	CDK	0.046213	0.994
**492**	4-KW-D8-M 10	CDK	0.081848	0.951
**493**	4-KW-E4-SI 1	CDK	0.325676	0.085
**494**	4-KW-E8-M 1	CDK	0.255485	0.234
**495**	4-KW-F4-Se 10000	CDK	0.418857	0.007
**496**	4-KW-F22-/ 10000	CDK	0.518755	0
**497**	4-KW-G4-S 1000	CDK	0.11995	0.978
**498**	4-KW-G22- 1000	CDK	0.543072	0
**499**	4-KW-H4-S 100	CDK	0.41143	0.002
**500**	4-KW-H22- 100	CDK	0.4397	0
**501**	4-KW-I4-Se 10	CDK	0.086697	0.92
**502**	4-KW-I22-A 10	CDK	0.430797	0.004
**503**	4-KW-J4-Se 1	CDK	0.086954	0.904
**504**	4-KW-J22-/ 1	CDK	0.390704	0.014
**505**	5-KW-A19-, 10000	CDK	0.581492	0
**506**	5-KW-B19-, 1000	CDK	0.494531	0.001
**507**	5-KW-C19-, 100	CDK	0.386228	0.007
**508**	5-KW-D19- 10	CDK	0.128949	0.972
**509**	5-KW-E19-/ 1	CDK	0.310698	0.336
**510**	5-KW-K17-, 1	CDK	0.145383	0.919
**511**	5-KW-M17- 10	CDK	0.322936	0.076
**512**	5-KW-N17- 100	CDK	0.383845	0.001
**513**	5-KW-O17- 1000	CDK	0.50652	0

**514**	5-KW-P17-/	10000	CDK	0.536493	0
**515**	6-KW-A17-!	1000	CDK	0.396457	0.008
**516**	6-KW-B17-!	100	CDK	0.318999	0.067
**517**	6-KW-C17-!	10	CDK	0.381308	0.009
**518**	6-KW-D17-	1	CDK	0.460638	0
**519**	6-KW-E17-!	0.1	CDK	0.305317	0.078
**520**	6-KW-L15-7	1	CDK	0.108475	0.994
**521**	6-KW-M15-	10	CDK	0.255122	0.341
**522**	6-KW-N15-	100	CDK	0.425879	0.002
**523**	6-KW-O15-	1000	CDK	0.555304	0
**524**	6-KW-P15-7	10000	CDK	0.488626	0.001
**525**	7-KW-A21-	10000	BET	0.251539	0.478
**526**	7-KW-A22-I	30000	BET	0.61872	0
**527**	7-KW-B21-	1000	BET	0.402547	0.004
**528**	7-KW-B22-I	3000	BET	0.585624	0
**529**	7-KW-C21-	100	BET	0.260215	0.154
**530**	7-KW-C22-I	300	BET	0.392626	0.002
**531**	7-KW-D21-	10	BET	0.635284	0
**532**	7-KW-D22-	30	BET	0.44957	0.007
**533**	7-KW-E21-	1	BET	0.293331	0.232
**534**	7-KW-E22-I	3	BET	0.266747	0.528
**535**	7-KW-G10-	10000	BET	0.646759	0
**536**	7-KW-G15-	10000	BET	0.643318	0
**537**	7-KW-H10-	1000	BET	0.649477	0
**538**	7-KW-H15-	1000	BET	0.628863	0
**539**	7-KW-I10-E	100	BET	0.570189	0
**540**	7-KW-I15-I	100	BET	0.302251	0.154
**541**	7-KW-J10-E	10	BET	0.206887	0.451
**542**	7-KW-J15-I	10	BET	0.234815	0.56
**543**	7-KW-K10-I	1	BET	0.160909	0.827
**544**	7-KW-K13-I	1	BET	0.200986	0.599
**545**	7-KW-K15-I	1	BET	0.253601	0.496
**546**	7-KW-L12-I	1	BET	0.17204	0.796
**547**	7-KW-L13-I	10	BET	0.198971	0.576
**548**	7-KW-L20-J	1	BET	0.443191	0.02

**549**	7-KW-L23-/	0.03	BET	0.243707	0.261
**550**	7-KW-M12-	10	BET	0.147309	0.877
**551**	7-KW-M13-	100	BET	0.63244	0
**552**	7-KW-M20-	10	BET	0.27044	0.455
**553**	7-KW-M23-	0.3	BET	0.236981	0.668
**554**	7-KW-N12-	100	BET	0.150485	0.901
**555**	7-KW-N13-	1000	BET	0.634961	0
**556**	7-KW-N20-	100	BET	0.527605	0
**557**	7-KW-N23-	3	BET	0.406709	0.001
**558**	7-KW-O12-	1000	BET	0.610501	0
**559**	7-KW-O20-	1000	BET	0.651076	0
**560**	7-KW-O23-	30	BET	0.582441	0
**561**	7-KW-P12-I	10000	BET	0.645475	0
**562**	7-KW-P13-I	10000	BET	0.591071	0
**563**	7-KW-P20-.	10000	BET	0.670524	0
**564**	7-KW-P23-,	300	BET	0.657612	0
**565**	8-KW-K22-(	1	BET	0.26696	0.136
**566**	8-KW-L22-(	10	BET	0.201072	0.397
**567**	8-KW-M22-	100	BET	0.2318	0.503
**568**	8-KW-N22-	1000	BET	0.605826	0
**569**	8-KW-O22-	10000	BET	0.614565	0
**570**	1-KW-A3-V(	10000	HDAC	0.385313	0.007
**571**	1-KW-B3-V(	1000	HDAC	0.404119	0
**572**	1-KW-C3-V(	100	HDAC	0.258163	0.175
**573**	1-KW-D3-V	10	HDAC	0.044593	0.995
**574**	1-KW-E3-V(	1	HDAC	0.037456	0.994
**575**	1-KW-L12-I	0.1	HDAC	0.357059	0.001
**576**	1-KW-M12-	1	HDAC	0.423333	0
**577**	1-KW-N12-	10	HDAC	0.434274	0
**578**	1-KW-O12-	100	HDAC	0.473963	0.001
**579**	1-KW-P12-I	1000	HDAC	0.465275	0.007
**580**	3-KW-A4-P;	1000	HDAC	0.409058	0.004
**581**	3-KW-B4-P;	100	HDAC	0.388397	0.004
**582**	3-KW-C4-P;	10	HDAC	0.352107	0.003
**583**	3-KW-D4-P	1	HDAC	0.027159	0.998

**584**	3-KW-E4-P‹	0.1	HDAC	0.231277	0.253
**585**	3-KW-F7-Q	1000	HDAC	0.464262	0
**586**	3-KW-G7-Q	100	HDAC	0.405671	0.001
**587**	3-KW-G12-	1000000	HDAC	0.114015	0.808
**588**	3-KW-H7-Q	10	HDAC	0.44031	0
**589**	3-KW-H12-	100000	HDAC	0.102484	0.852
**590**	3-KW-I7-Qu	1	HDAC	0.184025	0.375
**591**	3-KW-I12-V	10000	HDAC	0.343635	0
**592**	3-KW-J7-Qu	0.1	HDAC	0.239831	0.111
**593**	3-KW-J12-V	1000	HDAC	0.452652	0
**594**	3-KW-K3-B	1	HDAC	0.065979	0.997
**595**	3-KW-K12-	100	HDAC	0.397384	0
**596**	3-KW-L3-Be	10	HDAC	0.061105	0.999
**597**	3-KW-M3-E	100	HDAC	0.390483	0.001
**598**	3-KW-N3-B	1000	HDAC	0.362994	0.01
**599**	3-KW-O3-B	10000	HDAC	0.47009	0
**600**	7-KW-A5-M	10000	HDAC	0.415274	0.006
**601**	7-KW-A7-C	10000	HDAC	0.585985	0
**602**	7-KW-A9-G	1000	HDAC	0.436099	0
**603**	7-KW-A12-	10000	HDAC	0.446808	0
**604**	7-KW-B5-M	1000	HDAC	0.462723	0
**605**	7-KW-B7-C	1000	HDAC	0.452988	0.001
**606**	7-KW-B12-	1000	HDAC	0.47147	0
**607**	7-KW-C5-M	100	HDAC	0.515399	0
**608**	7-KW-C7-C	100	HDAC	0.431843	0
**609**	7-KW-C9-G	100	HDAC	0.513419	0
**610**	7-KW-D7-C	10	HDAC	0.062474	0.952
**611**	7-KW-D9-G	10	HDAC	0.481706	0
**612**	7-KW-D12-	100	HDAC	0.514534	0
**613**	7-KW-E5-M	10	HDAC	0.498914	0
**614**	7-KW-E7-Cl	1	HDAC	0.287799	0.021
**615**	7-KW-E9-Gi	1	HDAC	0.278946	0.006
**616**	7-KW-E12-I	10	HDAC	0.349878	0
**617**	7-KW-F5-M	1	HDAC	0.328654	0.006
**618**	7-KW-F7-Re	10000	HDAC	0.504845	0

**619**	7-KW-F9-Gi	0.1	HDAC	0.44322	0
**620**	7-KW-F12-I	1	HDAC	0.543819	0
**621**	7-KW-F19-I	10000	HDAC	0.328859	0.014
**622**	7-KW-G7-R	1000	HDAC	0.471744	0
**623**	7-KW-G19-	1000	HDAC	0.1488	0.86
**624**	7-KW-H7-R	100	HDAC	0.426252	0
**625**	7-KW-I7-Re	10	HDAC	0.359728	0.001
**626**	7-KW-I19-F	100	HDAC	0.345781	0
**627**	7-KW-J7-Re	1	HDAC	0.441576	0
**628**	7-KW-J19-F	10	HDAC	0.031594	0.997
**629**	7-KW-K4-Er	1	HDAC	0.212762	0.507
**630**	7-KW-K11-,	1	HDAC	0.416899	0
**631**	7-KW-K18-'	1	HDAC	0.311779	0.024
**632**	7-KW-K19-I	1	HDAC	0.414143	0
**633**	7-KW-L2-Ta	0.1	HDAC	0.084595	0.957
**634**	7-KW-L4-Er	10	HDAC	0.513713	0
**635**	7-KW-L5-Pr	1	HDAC	0.10667	0.821
**636**	7-KW-L8-Al	1	HDAC	0.127496	0.713
**637**	7-KW-L10-'	1	HDAC	0.41473	0
**638**	7-KW-L11-/	10	HDAC	0.526885	0
**639**	7-KW-L14-'	1	HDAC	0.363257	0
**640**	7-KW-L16-I	1	HDAC	0.330283	0.004
**641**	7-KW-L18-'	10	HDAC	0.484372	0
**642**	7-KW-M2-T	1	HDAC	0.054454	0.994
**643**	7-KW-M5-F	10	HDAC	0.087054	0.945
**644**	7-KW-M8-A	10	HDAC	0.249936	0.073
**645**	7-KW-M10-	10	HDAC	0.456775	0
**646**	7-KW-M11-	100	HDAC	0.488775	0
**647**	7-KW-M14-	10	HDAC	0.442097	0
**648**	7-KW-M16-	10	HDAC	0.415544	0
**649**	7-KW-M18-	100	HDAC	0.564762	0
**650**	7-KW-N2-Ti	10	HDAC	0.095241	0.895
**651**	7-KW-N4-Er	100	HDAC	0.493265	0
**652**	7-KW-N5-P	100	HDAC	0.445828	0
**653**	7-KW-N8-A	100	HDAC	0.492459	0

**654**	7-KW-N10-	100	HDAC	0.479468	0
**655**	7-KW-N14-	100	HDAC	0.078435	0.913
**656**	7-KW-N16-	100	HDAC	0.192233	0.295
**657**	7-KW-N18-	1000	HDAC	0.29562	0.04
**658**	7-KW-O2-Ta	100	HDAC	0.348874	0.007
**659**	7-KW-O4-Er	1000	HDAC	0.469045	0
**660**	7-KW-O5-P	1000	HDAC	0.451041	0
**661**	7-KW-O8-A	1000	HDAC	0.433976	0
**662**	7-KW-O10-	1000	HDAC	0.502149	0
**663**	7-KW-O11-	1000	HDAC	0.446079	0
**664**	7-KW-O14-	1000	HDAC	0.419021	0
**665**	7-KW-O16-	1000	HDAC	0.461831	0
**666**	7-KW-P2-Ta	1000	HDAC	0.502691	0
**667**	7-KW-P4-Er	10000	HDAC	0.459643	0
**668**	7-KW-P5-Pr	10000	HDAC	0.455229	0.001
**669**	7-KW-P8-Al	10000	HDAC	0.444573	0.003
**670**	7-KW-P10-T	10000	HDAC	0.462277	0
**671**	7-KW-P11-A	10000	HDAC	0.449254	0
**672**	7-KW-P14-T	10000	HDAC	0.492818	0
**673**	7-KW-P16-I	10000	HDAC	0.445	0
**674**	7-KW-P18-T	10000	HDAC	0.39032	0
**0**	2-MHB-A16	10000	EGFR	0.675982	0
**1**	2-MHB-A19	10000	EGFR	0.633356	0
**2**	2-MHB-B19	1000	EGFR	0.551352	0
**3**	2-MHB-C16	1000	EGFR	0.620176	0
**4**	2-MHB-C19	100	EGFR	0.510386	0
**5**	2-MHB-D16	100	EGFR	0.548726	0
**6**	2-MHB-D19	10	EGFR	0.111912	0.913
**7**	2-MHB-E16	10	EGFR	0.310267	0.195
**8**	2-MHB-E19	1	EGFR	0.027214	0.967
**9**	2-MHB-F16	1	EGFR	0.385302	0.009
**10**	2-MHB-K11	0.1	EGFR	0.146973	0.837
**11**	2-MHB-L11	1	EGFR	0.266147	0.398
**12**	2-MHB-L16	0.25	EGFR	0.048665	0.971
**13**	2-MHB-L19	0.1	EGFR	0.495916	0

**14**	2-MHB-M1	10	EGFR	0.67268	0
**15**	2-MHB-M16	2.5	EGFR	0.332698	0.045
**16**	2-MHB-M19	1	EGFR	0.401639	0
**17**	2-MHB-N16	25	EGFR	0.421397	0
**18**	2-MHB-N19	10	EGFR	0.591458	0
**19**	2-MHB-O11	100	EGFR	0.664695	0
**20**	2-MHB-O16	250	EGFR	0.606571	0
**21**	2-MHB-O19	100	EGFR	0.567124	0
**22**	2-MHB-P11	1000	EGFR	0.580681	0
**23**	2-MHB-P16	2500	EGFR	0.683114	0
**24**	2-MHB-P19	1000	EGFR	0.556679	0
**25**	3-MHB-F21	10000	EGFR	0.540336	0
**26**	3-MHB-G20	1000	EGFR	0.676316	0
**27**	3-MHB-G21	1000	EGFR	0.525316	0
**28**	3-MHB-H20	100	EGFR	0.66938	0
**29**	3-MHB-H21	100	EGFR	0.343777	0.009
**30**	3-MHB-I20-	10	EGFR	0.663761	0
**31**	3-MHB-I21-	10	EGFR	0.064838	0.958
**32**	3-MHB-J20	1	EGFR	0.315668	0.001
**33**	3-MHB-J21	1	EGFR	0.167753	0.96
**34**	3-MHB-K4-	1	EGFR	0.322429	0.076
**35**	3-MHB-K18	0.1	EGFR	0.206284	0.608
**36**	3-MHB-K20	0.1	EGFR	0.182947	0.765
**37**	3-MHB-L4-	10	EGFR	0.515178	0
**38**	3-MHB-L18	1	EGFR	0.6488	0
**39**	3-MHB-M1	10	EGFR	0.663603	0
**40**	3-MHB-N4-	100	EGFR	0.616505	0
**41**	3-MHB-N18	100	EGFR	0.65577	0
**42**	3-MHB-O4-	1000	EGFR	0.682266	0
**43**	3-MHB-P4-	10000	EGFR	0.63388	0
**44**	3-MHB-P18	1000	EGFR	0.680245	0
**45**	4-MHB-F13	1000	EGFR	0.643979	0
**46**	4-MHB-G13	100	EGFR	0.607766	0
**47**	4-MHB-G16	10000	EGFR	0.682171	0
**48**	4-MHB-H13	10	EGFR	0.602444	0

**49**	4-MHB-H16	1000	EGFR	0.702626	0
**50**	4-MHB-I13-	1	EGFR	0.504721	0
**51**	4-MHB-I16-	100	EGFR	0.616095	0
**52**	4-MHB-J13	0.1	EGFR	0.346704	0.003
**53**	4-MHB-J16	10	EGFR	0.125527	0.589
**54**	4-MHB-K7-	1	EGFR	0.17167	0.75
**55**	4-MHB-K13	0.1	EGFR	0.126173	0.624
**56**	4-MHB-K16	1	EGFR	0.109448	0.668
**57**	4-MHB-L7-I	10	EGFR	0.43839	0
**58**	4-MHB-L13	1	EGFR	0.696673	0
**59**	4-MHB-M7-	100	EGFR	0.622178	0
**60**	4-MHB-M1	10	EGFR	0.67946	0
**61**	4-MHB-N13	100	EGFR	0.648659	0
**62**	4-MHB-O7-	1000	EGFR	0.62353	0
**63**	4-MHB-P7-	10000	EGFR	0.660121	0
**64**	4-MHB-P13	1000	EGFR	0.651917	0
**65**	5-MHB-F4-I	1000	EGFR	0.604741	0
**66**	5-MHB-F7-A	1000	EGFR	0.62997	0
**67**	5-MHB-G4-	100	EGFR	0.646105	0
**68**	5-MHB-G7-	100	EGFR	0.60204	0
**69**	5-MHB-H4-	10	EGFR	0.615997	0
**70**	5-MHB-H7-	10	EGFR	0.610053	0
**71**	5-MHB-I4-F	1	EGFR	0.61588	0
**72**	5-MHB-I7-A	1	EGFR	0.434899	0
**73**	5-MHB-J4-F	0.1	EGFR	0.521637	0
**74**	5-MHB-J7-A	0.1	EGFR	0.12468	0.837
**75**	5-MHB-K7-	0.1	EGFR	0.535813	0
**76**	5-MHB-L7-(	1	EGFR	0.042484	0.948
**77**	5-MHB-M7-	10	EGFR	0.129504	0.936
**78**	5-MHB-O7-	100	EGFR	0.411566	0
**79**	5-MHB-P7-	1000	EGFR	0.421756	0.001
**80**	2-MHB-A15	2500	VEGFR	0.138498	0.939
**81**	2-MHB-A17	10000	VEGFR	0.086313	0.973
**82**	2-MHB-A20	10000	VEGFR	0.137357	0.967
**83**	2-MHB-B15	250	VEGFR	0.175577	0.849

**84**	2-MHB-B17	1000	VEGFR	0.126751	0.95
**85**	2-MHB-B20	1000	VEGFR	0.340142	0
**86**	2-MHB-C15	25	VEGFR	0.192333	0.721
**87**	2-MHB-C17	100	VEGFR	0.24872	0.257
**88**	2-MHB-D15	2.5	VEGFR	0.268805	0.003
**89**	2-MHB-D17	10	VEGFR	0.312631	0.027
**90**	2-MHB-D20	100	VEGFR	0.353516	0
**91**	2-MHB-E17	1	VEGFR	0.299788	0.001
**92**	2-MHB-E20	10	VEGFR	0.351742	0
**93**	2-MHB-F13	10000	VEGFR	0.224158	0.882
**94**	2-MHB-F15	0.25	VEGFR	0.309164	0.002
**95**	2-MHB-F19	10000	VEGFR	0.0499	0.976
**96**	2-MHB-F20	1	VEGFR	0.408002	0
**97**	2-MHB-F21	10000	VEGFR	0.356752	0
**98**	2-MHB-G10	10000	VEGFR	0.187915	0.392
**99**	2-MHB-G13	1000	VEGFR	0.170642	0.878
**100**	2-MHB-G19	1000	VEGFR	0.16866	0.756
**101**	2-MHB-G21	1000	VEGFR	0.365459	0
**102**	2-MHB-H10	1000	VEGFR	0.262869	0.123
**103**	2-MHB-H13	100	VEGFR	0.316744	0.02
**104**	2-MHB-H21	100	VEGFR	0.346807	0
**105**	2-MHB-I10-	100	VEGFR	0.191201	0.542
**106**	2-MHB-I13-	10	VEGFR	0.284977	0.129
**107**	2-MHB-I19-	100	VEGFR	0.099905	0.973
**108**	2-MHB-I21-	10	VEGFR	0.356242	0
**109**	2-MHB-J10	10	VEGFR	0.246774	0.044
**110**	2-MHB-J13	1	VEGFR	0.205768	0.653
**111**	2-MHB-J19	10	VEGFR	0.314544	0.04
**112**	2-MHB-J21	1	VEGFR	0.359199	0
**113**	2-MHB-K10	1	VEGFR	0.237967	0.045
**114**	2-MHB-K13	0.1	VEGFR	0.292401	0.003
**115**	2-MHB-K17	1	VEGFR	0.392098	0
**116**	2-MHB-K19	1	VEGFR	0.135009	0.909
**117**	2-MHB-L12	0.1	VEGFR	0.356936	0
**118**	2-MHB-L13	1	VEGFR	0.240715	0.298

**119**	2-MHB-L21	0.1	VEGFR	0.19778	0.491
**120**	2-MHB-M1[illegible]	1	VEGFR	0.226833	0.36
**121**	2-MHB-M1[illegible]	10	VEGFR	0.371776	0.019
**122**	2-MHB-M1[illegible]	10	VEGFR	0.298822	0.015
**123**	2-MHB-M2[illegible]	1	VEGFR	0.259295	0.002
**124**	2-MHB-N1[illegible]	10	VEGFR	0.316974	0
**125**	2-MHB-N1[illegible]	100	VEGFR	0.136493	0.922
**126**	2-MHB-N1[illegible]	100	VEGFR	0.245944	0.033
**127**	2-MHB-N21	10	VEGFR	0.280824	0.02
**128**	2-MHB-O1[illegible]	100	VEGFR	0.098186	0.958
**129**	2-MHB-O1[illegible]	1000	VEGFR	0.274522	0.021
**130**	2-MHB-O21	100	VEGFR	0.297729	0.001
**131**	2-MHB-P12	1000	VEGFR	0.132097	0.927
**132**	2-MHB-P13	1000	VEGFR	0.095103	0.968
**133**	2-MHB-P17	10000	VEGFR	0.229176	0.709
**134**	2-MHB-P21	1000	VEGFR	0.100826	0.951
**135**	3-MHB-A3-[illegible]	1000	VEGFR	0.22043	0.277
**136**	3-MHB-A6-[illegible]	1000	VEGFR	0.192436	0.946
**137**	3-MHB-A18	1000	VEGFR	0.080379	0.964
**138**	3-MHB-B3-[illegible]	100	VEGFR	0.201695	0.406
**139**	3-MHB-B6-[illegible]	100	VEGFR	0.108536	0.959
**140**	3-MHB-B18	100	VEGFR	0.230774	0.166
**141**	3-MHB-C3-[illegible]	10	VEGFR	0.233384	0.684
**142**	3-MHB-C6-[illegible]	10	VEGFR	0.19302	0.773
**143**	3-MHB-C18	10	VEGFR	0.29761	0.087
**144**	3-MHB-D3-	1	VEGFR	0.249998	0.228
**145**	3-MHB-D6-	1	VEGFR	0.249413	0.328
**146**	3-MHB-D1[illegible]	1	VEGFR	0.335833	0.008
**147**	3-MHB-E3-[illegible]	0.1	VEGFR	0.432195	0
**148**	3-MHB-E6-[illegible]	0.1	VEGFR	0.313449	0.001
**149**	3-MHB-E18	0.1	VEGFR	0.207951	0.722
**150**	3-MHB-F18	1000	VEGFR	0.144223	0.923
**151**	3-MHB-G1[illegible]	100	VEGFR	0.366265	0
**152**	3-MHB-H1[illegible]	10	VEGFR	0.231704	0.052
**153**	3-MHB-I18[illegible]	1	VEGFR	0.286539	0.023

**154**	3-MHB-J18	0.1	VEGFR	0.179559	0.541
**155**	4-MHB-A12	10000	VEGFR	0.142825	0.961
**156**	4-MHB-A15	2500	VEGFR	0.142163	0.946
**157**	4-MHB-A2(	10000	VEGFR	0.39378	0
**158**	4-MHB-B12	1000	VEGFR	0.087087	0.974
**159**	4-MHB-B15	250	VEGFR	0.300705	0.012
**160**	4-MHB-B2(	1000	VEGFR	0.327542	0.001
**161**	4-MHB-C15	25	VEGFR	0.316462	0.003
**162**	4-MHB-D12	100	VEGFR	0.167267	0.851
**163**	4-MHB-D15	2.5	VEGFR	0.169477	0.586
**164**	4-MHB-D2(	100	VEGFR	0.420003	0
**165**	4-MHB-E12	10	VEGFR	0.293121	0.011
**166**	4-MHB-E20	10	VEGFR	0.328501	0.003
**167**	4-MHB-F12	1	VEGFR	0.255999	0.058
**168**	4-MHB-F15	0.25	VEGFR	0.215985	0.387
**169**	4-MHB-F20	1	VEGFR	0.360668	0
**170**	4-MHB-L16	1	VEGFR	0.245034	0.303
**171**	4-MHB-M1(	10	VEGFR	0.152429	0.849
**172**	4-MHB-N16	100	VEGFR	0.255674	0.037
**173**	4-MHB-O16	1000	VEGFR	0.292088	0.007
**174**	4-MHB-P16	10000	VEGFR	0.219533	0.223
**175**	2-MHB-L10	1	PI3K	0.042625	0.956
**176**	2-MHB-M1(	10	PI3K	0.164839	0.703
**177**	2-MHB-N1(	100	PI3K	0.193822	0.143
**178**	2-MHB-O1(	1000	PI3K	0.273718	0.054
**179**	2-MHB-P1(	10000	PI3K	0.303115	0.017
**180**	3-MHB-A16	2500	PI3K	0.185211	0.478
**181**	3-MHB-C16	250	PI3K	0.261078	0.234
**182**	3-MHB-D16	25	PI3K	0.128212	0.28
**183**	3-MHB-E16	2.5	PI3K	0.179495	0.487
**184**	3-MHB-F16	0.25	PI3K	0.225848	0.701
**185**	3-MHB-F17	100000	PI3K	0.207348	0.929
**186**	3-MHB-F19	500	PI3K	0.243384	0.329
**187**	3-MHB-G17	10000	PI3K	0.106917	0.887
**188**	3-MHB-G1(	50	PI3K	0.120199	0.684

**189**	3-MHB-H17	1000	PI3K	0.145282	0.594
**190**	3-MHB-I17-	100	PI3K	0.190017	0.051
**191**	3-MHB-I19-	5	PI3K	0.194122	0.68
**192**	3-MHB-J17	10	PI3K	0.296651	0.036
**193**	3-MHB-J19	0.5	PI3K	0.07831	0.961
**194**	3-MHB-K19	0.05	PI3K	0.165481	0.391
**195**	3-MHB-L8-I	1	PI3K	0.276726	0.03
**196**	3-MHB-L21	0.1	PI3K	0.158216	0.886
**197**	3-MHB-M8-	10	PI3K	0.306848	0
**198**	3-MHB-M2	1	PI3K	0.250572	0.224
**199**	3-MHB-N8-	100	PI3K	0.342475	0.005
**200**	3-MHB-N21	10	PI3K	0.388146	0
**201**	3-MHB-O8-	1000	PI3K	0.354513	0.021
**202**	3-MHB-O21	100	PI3K	0.360832	0.001
**203**	3-MHB-P8-I	10000	PI3K	0.383033	0.011
**204**	3-MHB-P21	1000	PI3K	0.359932	0.005
**205**	4-MHB-A19	2500	PI3K	0.385797	0
**206**	4-MHB-B19	250	PI3K	0.352864	0.001
**207**	4-MHB-C19	25	PI3K	0.222007	0.057
**208**	4-MHB-D19	2.5	PI3K	0.231122	0.036
**209**	4-MHB-E19	0.25	PI3K	0.231506	0.005
**210**	4-MHB-G2-	2500	PI3K	0.072947	0.94
**211**	4-MHB-G5-	10000	PI3K	0.097348	0.959
**212**	4-MHB-G14	100	PI3K	0.312872	0.102
**213**	4-MHB-G20	10000	PI3K	0.321353	0.194
**214**	4-MHB-H2-	250	PI3K	0.150675	0.799
**215**	4-MHB-H5-	1000	PI3K	0.172458	0.767
**216**	4-MHB-H14	10	PI3K	0.204857	0.708
**217**	4-MHB-H20	1000	PI3K	0.415982	0.001
**218**	4-MHB-I2-T	25	PI3K	0.08783	0.955
**219**	4-MHB-I5-S	100	PI3K	0.180315	0.764
**220**	4-MHB-I14-	1	PI3K	0.277514	0.001
**221**	4-MHB-I20-	100	PI3K	0.330842	0
**222**	4-MHB-J2-T	2.5	PI3K	0.078401	0.958
**223**	4-MHB-J5-S	10	PI3K	0.207655	0.581

**224**	4-MHB-J20	10	PI3K	0.217347	0.082
**225**	4-MHB-K2-[illegible]	0.25	PI3K	0.136745	0.914
**226**	4-MHB-K4-[illegible]	0.1	PI3K	0.177945	0.744
**227**	4-MHB-K5-[illegible]	1	PI3K	0.168456	0.211
**228**	4-MHB-K14	0.1	PI3K	0.249535	0.004
**229**	4-MHB-K20	1	PI3K	0.311824	0
**230**	4-MHB-L4-[illegible]	1	PI3K	0.18534	0.513
**231**	4-MHB-L14	0.1	PI3K	0.326749	0
**232**	4-MHB-L15	1	PI3K	0.206808	0.058
**233**	4-MHB-L21	0.1	PI3K	0.272668	0.027
**234**	4-MHB-M1[illegible]	1	PI3K	0.319838	0
**235**	4-MHB-M1[illegible]	10	PI3K	0.187436	0.098
**236**	4-MHB-M2[illegible]	1	PI3K	0.307964	0
**237**	4-MHB-N4-[illegible]	10	PI3K	0.299274	0.19
**238**	4-MHB-N1[illegible]	10	PI3K	0.41257	0
**239**	4-MHB-N1[illegible]	100	PI3K	0.290904	0
**240**	4-MHB-N21	10	PI3K	0.377774	0
**241**	4-MHB-O4-[illegible]	100	PI3K	0.366332	0.012
**242**	4-MHB-O1[illegible]	100	PI3K	0.299985	0.121
**243**	4-MHB-O1[illegible]	1000	PI3K	0.335084	0
**244**	4-MHB-O21	100	PI3K	0.398159	0
**245**	4-MHB-P4-[illegible]	1000	PI3K	0.361404	0.007
**246**	4-MHB-P14	1000	PI3K	0.314364	0.041
**247**	4-MHB-P15	10000	PI3K	0.262253	0.031
**248**	4-MHB-P21	1000	PI3K	0.345441	0.004
**249**	5-MHB-A6-[illegible]	2500	PI3K	0.293384	0.123
**250**	5-MHB-A7-[illegible]	1000	PI3K	0.212048	0.749
**251**	5-MHB-A16	2500	PI3K	0.348764	0.001
**252**	5-MHB-A17	10000	PI3K	0.240457	0.526
**253**	5-MHB-B6-[illegible]	250	PI3K	0.331501	0.046
**254**	5-MHB-B7-[illegible]	100	PI3K	0.147286	0.732
**255**	5-MHB-B17	1000	PI3K	0.201737	0.161
**256**	5-MHB-C6-[illegible]	25	PI3K	0.155822	0.612
**257**	5-MHB-C7-[illegible]	10	PI3K	0.117302	0.484
**258**	5-MHB-C16	250	PI3K	0.219031	0.732

**259**	5-MHB-C17	100	PI3K	0.085279	0.951
**260**	5-MHB-D6-	2.5	PI3K	0.175033	0.435
**261**	5-MHB-D7-	1	PI3K	0.065104	0.961
**262**	5-MHB-D16	25	PI3K	0.108976	0.905
**263**	5-MHB-D17	10	PI3K	0.144819	0.291
**264**	5-MHB-E6-I	0.25	PI3K	0.175126	0.864
**265**	5-MHB-E7-A	0.1	PI3K	0.236883	0.526
**266**	5-MHB-E16	2.5	PI3K	0.174793	0.092
**267**	5-MHB-E17	1	PI3K	0.227081	0.033
**268**	5-MHB-F11	10000	PI3K	0.350664	0
**269**	5-MHB-F16	0.25	PI3K	0.14006	0.686
**270**	5-MHB-G9-	10000	PI3K	0.384002	0.002
**271**	5-MHB-G11	1000	PI3K	0.240438	0.022
**272**	5-MHB-H9-	1000	PI3K	0.397141	0
**273**	5-MHB-H11	100	PI3K	0.170724	0.335
**274**	5-MHB-I9-S	100	PI3K	0.307857	0.074
**275**	5-MHB-I11-	10	PI3K	0.129026	0.554
**276**	5-MHB-J9-S	10	PI3K	0.131801	0.565
**277**	5-MHB-J11	1	PI3K	0.197925	0.635
**278**	5-MHB-K9-S	1	PI3K	0.289219	0.257
**279**	5-MHB-L14	0.1	PI3K	0.231027	0.011
**280**	5-MHB-L20	1	PI3K	0.236418	0.024
**281**	5-MHB-L23	0.1	PI3K	0.230301	0.019
**282**	5-MHB-M14	1	PI3K	0.350638	0
**283**	5-MHB-M20	10	PI3K	0.206755	0.007
**284**	5-MHB-M23	1	PI3K	0.312834	0.005
**285**	5-MHB-N14	10	PI3K	0.27095	0.029
**286**	5-MHB-N20	100	PI3K	0.266891	0
**287**	5-MHB-N23	10	PI3K	0.385647	0
**288**	5-MHB-O14	100	PI3K	0.297987	0.01
**289**	5-MHB-O20	1000	PI3K	0.386524	0
**290**	5-MHB-O23	100	PI3K	0.375497	0
**291**	5-MHB-P14	1000	PI3K	0.285857	0.092
**292**	5-MHB-P20	10000	PI3K	0.38154	0
**293**	5-MHB-P23	1000	PI3K	0.249803	0.394

294	6-MHB-A8-'	10000	PI3K	0.282528	0.085
295	6-MHB-B8-'	1000	PI3K	0.128231	0.789
296	6-MHB-C8-'	100	PI3K	0.174902	0.841
297	6-MHB-D8-	10	PI3K	0.105361	0.966
298	6-MHB-E8-'	1	PI3K	0.189753	0.466
299	6-MHB-L6-(	1	PI3K	0.194369	0.767
300	6-MHB-M6-	10	PI3K	0.143321	0.952
301	6-MHB-N6-	100	PI3K	0.2147	0.544
302	6-MHB-O6-	1000	PI3K	0.336131	0.031
303	6-MHB-P6-(	10000	PI3K	0.096891	0.956
304	1-MHB-F11	10000	Topoisome	0.570455	0
305	1-MHB-G11	1000	Topoisome	0.628414	0
306	1-MHB-G2(	1000	Topoisome	0.338237	0.152
307	1-MHB-H11	100	Topoisome	0.599634	0
308	1-MHB-H2(	100	Topoisome	0.59704	0
309	1-MHB-I11-	10	Topoisome	0.290224	0.027
310	1-MHB-I20-	10	Topoisome	0.175139	0.328
311	1-MHB-J11	1	Topoisome	0.330119	0.11
312	1-MHB-J20	1	Topoisome	0.176378	0.362
313	1-MHB-K11	1	Topoisome	0.526953	0
314	1-MHB-K2(	0.1	Topoisome	0.448636	0
315	1-MHB-L11	10	Topoisome	0.622856	0
316	1-MHB-L14	1	Topoisome	0.544769	0
317	1-MHB-M1:	100	Topoisome	0.491075	0
318	1-MHB-M1	10	Topoisome	0.467034	0
319	1-MHB-N14	100	Topoisome	0.673001	0
320	1-MHB-O11	1000	Topoisome	0.442913	0.02
321	1-MHB-O14	1000	Topoisome	0.607657	0
322	1-MHB-P11	10000	Topoisome	0.371307	0.053
323	1-MHB-P14	10000	Topoisome	0.374152	0.079
324	3-MHB-A11	10000	Topoisome	0.666096	0
325	3-MHB-B11	1000	Topoisome	0.645586	0
326	3-MHB-C11	100	Topoisome	0.59017	0
327	3-MHB-D11	10	Topoisome	0.61893	0
328	3-MHB-E11	1	Topoisome	0.58048	0

**329**	3-MHB-G9-	1000	Topoisome	0.390584	0.099
**330**	3-MHB-G1(	10000	Topoisome	0.392203	0.028
**331**	3-MHB-H9-	100	Topoisome	0.656071	0
**332**	3-MHB-H1(	1000	Topoisome	0.566471	0
**333**	3-MHB-I9-[	10	Topoisome	0.397987	0.023
**334**	3-MHB-I10-	100	Topoisome	0.370919	0.106
**335**	3-MHB-J9-[	1	Topoisome	0.383457	0
**336**	3-MHB-J10	10	Topoisome	0.497283	0
**337**	3-MHB-K7-I	0.1	Topoisome	0.079624	0.86
**338**	3-MHB-K9-I	0.1	Topoisome	0.601907	0
**339**	3-MHB-K1(	1	Topoisome	0.495179	0
**340**	3-MHB-L6-I	0.1	Topoisome	0.312451	0.228
**341**	3-MHB-L7-I	1	Topoisome	0.179579	0.846
**342**	3-MHB-L9-\	0.5	Topoisome	0.184698	0.359
**343**	3-MHB-L10	0.1	Topoisome	0.491208	0
**344**	3-MHB-L16	1	Topoisome	0.196277	0.517
**345**	3-MHB-M6-	1	Topoisome	0.567303	0
**346**	3-MHB-M7-	10	Topoisome	0.449806	0.001
**347**	3-MHB-M9-	5	Topoisome	0.338129	0.121
**348**	3-MHB-M1(	1	Topoisome	0.438417	0.006
**349**	3-MHB-M1(	10	Topoisome	0.403322	0.005
**350**	3-MHB-N6-	10	Topoisome	0.169345	0.765
**351**	3-MHB-N9-	50	Topoisome	0.521101	0
**352**	3-MHB-N1(	10	Topoisome	0.572257	0
**353**	3-MHB-N16	100	Topoisome	0.216566	0.374
**354**	3-MHB-O6-	100	Topoisome	0.65417	0
**355**	3-MHB-O7-	100	Topoisome	0.657271	0
**356**	3-MHB-O9-	500	Topoisome	0.656472	0
**357**	3-MHB-O1(	100	Topoisome	0.656923	0
**358**	3-MHB-O16	1000	Topoisome	0.119227	0.933
**359**	3-MHB-P6-I	1000	Topoisome	0.550989	0
**360**	3-MHB-P7-I	1000	Topoisome	0.308645	0.326
**361**	3-MHB-P9-\	5000	Topoisome	0.409738	0.053
**362**	3-MHB-P1(	1000	Topoisome	0.466107	0.005
**363**	3-MHB-P16	10000	Topoisome	0.422928	0.004

**364**	1-MHB-A1(	10000	Mitotic	0.58722	0.001
**365**	1-MHB-A13	1000	Mitotic	0.622427	0
**366**	1-MHB-A18	1000	Mitotic	0.685338	0
**367**	1-MHB-B1(	1000	Mitotic	0.560246	0.001
**368**	1-MHB-B13	100	Mitotic	0.106845	0.957
**369**	1-MHB-B18	100	Mitotic	0.651441	0
**370**	1-MHB-C1(	100	Mitotic	0.63457	0
**371**	1-MHB-C13	10	Mitotic	0.393203	0.074
**372**	1-MHB-C18	10	Mitotic	0.470999	0.005
**373**	1-MHB-D1(	10	Mitotic	0.373604	0.101
**374**	1-MHB-D1:	1	Mitotic	0.40499	0.027
**375**	1-MHB-D18	1	Mitotic	0.310484	0.104
**376**	1-MHB-E10	1	Mitotic	0.56448	0
**377**	1-MHB-E13	0.1	Mitotic	0.197817	0.298
**378**	1-MHB-E18	0.1	Mitotic	0.321666	0.074
**379**	1-MHB-F13	1000	Mitotic	0.533305	0
**380**	1-MHB-G1:	100	Mitotic	0.657079	0
**381**	1-MHB-G15	1000	Mitotic	0.638972	0
**382**	1-MHB-H1:	10	Mitotic	0.507099	0
**383**	1-MHB-H15	100	Mitotic	0.674177	0
**384**	1-MHB-I13-	1	Mitotic	0.389313	0.002
**385**	1-MHB-I15-	10	Mitotic	0.523644	0.003
**386**	1-MHB-J13	0.1	Mitotic	0.461812	0
**387**	1-MHB-J15	1	Mitotic	0.479109	0
**388**	1-MHB-K7-'	0.1	Mitotic	0.396139	0.037
**389**	1-MHB-K15	0.1	Mitotic	0.349045	0.004
**390**	1-MHB-L7-'	1	Mitotic	0.338499	0.121
**391**	1-MHB-L20	0.1	Mitotic	0.372029	0.006
**392**	1-MHB-M7-	10	Mitotic	0.440608	0.005
**393**	1-MHB-M2(	1	Mitotic	0.375505	0
**394**	1-MHB-N2(	10	Mitotic	0.302828	0.04
**395**	1-MHB-O7-	100	Mitotic	0.515739	0.003
**396**	1-MHB-O2(	100	Mitotic	0.162181	0.595
**397**	1-MHB-P7-'	1000	Mitotic	0.513077	0.001
**398**	1-MHB-P2(	1000	Mitotic	0.476211	0.015

**399**	3-MHB-A7-[illegible]	1000	Mitotic	0.638698	0
**400**	3-MHB-B7-[illegible]	100	Mitotic	0.581999	0
**401**	3-MHB-C7-[illegible]	10	Mitotic	0.56264	0
**402**	3-MHB-D7-	1	Mitotic	0.41314	0.051
**403**	3-MHB-E7-[illegible]	0.1	Mitotic	0.479455	0.003
**404**	6-MHB-L19	1	Mitotic	0.352227	0.03
**405**	6-MHB-M1[illegible]	10	Mitotic	0.26621	0.189
**406**	6-MHB-N1[illegible]	100	Mitotic	0.121069	0.937
**407**	6-MHB-O1[illegible]	1000	Mitotic	0.443976	0.049
**408**	6-MHB-P1[illegible]	10000	Mitotic	0.520398	0.007
**409**	2-MHB-A12	250	MEK1/2	0.580471	0
**410**	2-MHB-B12	25	MEK1/2	0.724086	0
**411**	2-MHB-D12	2.5	MEK1/2	0.738379	0
**412**	2-MHB-E12	0.25	MEK1/2	0.627229	0
**413**	2-MHB-F12	0.025	MEK1/2	0.529259	0.001
**414**	2-MHB-F14	1000	MEK1/2	0.586439	0
**415**	2-MHB-G1[illegible]	100	MEK1/2	0.670756	0
**416**	2-MHB-H1[illegible]	10	MEK1/2	0.723791	0
**417**	2-MHB-I14-	1	MEK1/2	0.35905	0.167
**418**	2-MHB-K14	0.1	MEK1/2	0.463793	0.04
**419**	2-MHB-L20	1	MEK1/2	0.276695	0.672
**420**	2-MHB-M2[illegible]	10	MEK1/2	0.734727	0
**421**	2-MHB-N2[illegible]	100	MEK1/2	0.661731	0
**422**	2-MHB-O2[illegible]	1000	MEK1/2	0.659076	0
**423**	2-MHB-P2[illegible]	10000	MEK1/2	0.658568	0
**424**	4-MHB-A1[illegible]	1000	MEK1/2	0.653652	0
**425**	4-MHB-A13	1000	MEK1/2	0.609035	0
**426**	4-MHB-B1[illegible]	100	MEK1/2	0.702655	0
**427**	4-MHB-B13	100	MEK1/2	0.758607	0
**428**	4-MHB-C1[illegible]	10	MEK1/2	0.666643	0
**429**	4-MHB-C13	10	MEK1/2	0.668862	0
**430**	4-MHB-D1[illegible]	1	MEK1/2	0.527404	0.008
**431**	4-MHB-D1[illegible]	1	MEK1/2	0.506671	0.01
**432**	4-MHB-E10	0.1	MEK1/2	0.615563	0
**433**	4-MHB-E13	0.1	MEK1/2	0.184156	0.786

**434**	4-MHB-L19	0.25	MEK1/2	0.086902	0.992
**435**	4-MHB-M1!	2.5	MEK1/2	0.120315	0.903
**436**	4-MHB-N1!	25	MEK1/2	0.745262	0
**437**	4-MHB-O1!	250	MEK1/2	0.666679	0
**438**	4-MHB-P1!	2500	MEK1/2	0.661787	0
**439**	1-MHB-L2-(	1	PARP	0.708646	0
**440**	1-MHB-L6-I	1	PARP	0.634681	0
**441**	1-MHB-M2-	10	PARP	0.345844	0.122
**442**	1-MHB-M6-	10	PARP	0.533967	0.005
**443**	1-MHB-N2-	100	PARP	0.643096	0
**444**	1-MHB-N6-	100	PARP	0.374623	0.09
**445**	1-MHB-O2-	1000	PARP	0.664715	0
**446**	1-MHB-O6-	1000	PARP	0.690751	0
**447**	1-MHB-P2-(	10000	PARP	0.686301	0
**448**	1-MHB-P6-I	10000	PARP	0.569751	0.005
**449**	7-MHB-A3-`	1000	PARP	0.656415	0.001
**450**	7-MHB-B2-'	10000	PARP	0.624561	0
**451**	7-MHB-B3-`	100	PARP	0.677251	0
**452**	7-MHB-C2-'	1000	PARP	0.585407	0.007
**453**	7-MHB-C3-`	10	PARP	0.61768	0
**454**	7-MHB-D2-	100	PARP	0.640709	0
**455**	7-MHB-D3-	1	PARP	0.52758	0.013
**456**	7-MHB-E2-\	10	PARP	0.497892	0.023
**457**	7-MHB-E3-`	0.1	PARP	0.443533	0.067
**458**	7-MHB-F2-\	1	PARP	0.711358	0
**459**	7-MHB-G2-	10000	PARP	0.670012	0
**460**	7-MHB-H2-	1000	PARP	0.69281	0
**461**	7-MHB-I2-N	100	PARP	0.701356	0
**462**	7-MHB-J2-N	10	PARP	0.584652	0.004
**463**	7-MHB-K2-I	1	PARP	0.436139	0.049
**464**	3-MHB-A1!	1000	CDK	0.504724	0.007
**465**	3-MHB-B1!	100	CDK	0.467727	0.025
**466**	3-MHB-B23	2500	CDK	0.36129	0.003
**467**	3-MHB-C1!	10	CDK	0.205694	0.814
**468**	3-MHB-C23	250	CDK	0.29566	0.146

**469**	3-MHB-D19	1	CDK	0.199691	0.183
**470**	3-MHB-D23	25	CDK	0.300985	0.077
**471**	3-MHB-E19	0.1	CDK	0.137469	0.955
**472**	3-MHB-E23	2.5	CDK	0.290731	0.041
**473**	3-MHB-F23	0.25	CDK	0.270758	0.255
**474**	3-MHB-K17	1	CDK	0.102841	0.935
**475**	3-MHB-L19	1	CDK	0.149328	0.947
**476**	3-MHB-M1	10	CDK	0.353684	0.012
**477**	3-MHB-M1	10	CDK	0.242317	0.041
**478**	3-MHB-N17	100	CDK	0.439055	0.001
**479**	3-MHB-N19	100	CDK	0.329234	0.024
**480**	3-MHB-O17	1000	CDK	0.325619	0.038
**481**	3-MHB-O19	1000	CDK	0.336841	0.009
**482**	3-MHB-P17	10000	CDK	0.249018	0.629
**483**	3-MHB-P19	10000	CDK	0.288326	0.474
**484**	4-MHB-A4-	10000	CDK	0.472801	0.072
**485**	4-MHB-A8-	10000	CDK	0.417694	0.018
**486**	4-MHB-B4-	1000	CDK	0.452087	0.071
**487**	4-MHB-B8-	1000	CDK	0.220276	0.571
**488**	4-MHB-C4-	100	CDK	0.428525	0.016
**489**	4-MHB-C8-	100	CDK	0.209433	0.529
**490**	4-MHB-D4-	10	CDK	0.232163	0.677
**491**	4-MHB-D8-	10	CDK	0.224352	0.663
**492**	4-MHB-E4-	1	CDK	0.171309	0.491
**493**	4-MHB-E8-	1	CDK	0.229757	0.623
**494**	4-MHB-F4-	10000	CDK	0.237583	0.688
**495**	4-MHB-F22	10000	CDK	0.475192	0.057
**496**	4-MHB-G4-	1000	CDK	0.338359	0.129
**497**	4-MHB-G22	1000	CDK	0.445416	0.064
**498**	4-MHB-H4-	100	CDK	0.2757	0.132
**499**	4-MHB-H22	100	CDK	0.402692	0.024
**500**	4-MHB-I4-S	10	CDK	0.224476	0.67
**501**	4-MHB-I22-	10	CDK	0.191687	0.202
**502**	4-MHB-J4-S	1	CDK	0.155438	0.88
**503**	4-MHB-J22	1	CDK	0.245607	0.075

**504**	5-MHB-A19	10000	CDK	0.296237	0.425
**505**	5-MHB-B19	1000	CDK	0.232141	0.776
**506**	5-MHB-C19	100	CDK	0.274682	0.107
**507**	5-MHB-D19	10	CDK	0.200251	0.098
**508**	5-MHB-E19	1	CDK	0.216454	0.616
**509**	5-MHB-K17	1	CDK	0.150788	0.843
**510**	5-MHB-M1[illegible]	10	CDK	0.258457	0.514
**511**	5-MHB-N17	100	CDK	0.295317	0.055
**512**	5-MHB-O17	1000	CDK	0.159286	0.943
**513**	5-MHB-P17	10000	CDK	0.333168	0.067
**514**	6-MHB-A17	1000	CDK	0.229291	0.701
**515**	6-MHB-B17	100	CDK	0.277442	0.216
**516**	6-MHB-C17	10	CDK	0.179248	0.88
**517**	6-MHB-D17	1	CDK	0.176817	0.757
**518**	6-MHB-E17	0.1	CDK	0.257938	0.569
**519**	6-MHB-L15	1	CDK	0.16344	0.724
**520**	6-MHB-M15	10	CDK	0.140185	0.708
**521**	6-MHB-N15	100	CDK	0.137537	0.962
**522**	6-MHB-O15	1000	CDK	0.45759	0.021
**523**	6-MHB-P15	10000	CDK	0.370414	0.261
**524**	7-MHB-A21	10000	BET	0.534781	0
**525**	7-MHB-A22	30000	BET	0.547341	0
**526**	7-MHB-B21	1000	BET	0.289165	0.113
**527**	7-MHB-B22	3000	BET	0.500287	0
**528**	7-MHB-C21	100	BET	0.203738	0.319
**529**	7-MHB-C22	300	BET	0.193976	0.463
**530**	7-MHB-D21	10	BET	0.207024	0.376
**531**	7-MHB-D22	30	BET	0.256097	0.247
**532**	7-MHB-E21	1	BET	0.396454	0.026
**533**	7-MHB-E22	3	BET	0.449043	0.004
**534**	7-MHB-G10	10000	BET	0.572641	0
**535**	7-MHB-G15	10000	BET	0.620006	0
**536**	7-MHB-H10	1000	BET	0.563509	0
**537**	7-MHB-H15	1000	BET	0.562101	0
**538**	7-MHB-I10-	100	BET	0.513585	0.001

**539**	7-MHB-I15-	100	BET	0.456686	0.007
**540**	7-MHB-J10	10	BET	0.197906	0.653
**541**	7-MHB-J15	10	BET	0.114561	0.865
**542**	7-MHB-K1C	1	BET	0.204361	0.54
**543**	7-MHB-K13	1	BET	0.473245	0.014
**544**	7-MHB-K15	1	BET	0.279705	0.527
**545**	7-MHB-L12	1	BET	0.123817	0.953
**546**	7-MHB-L13	10	BET	0.591063	0
**547**	7-MHB-L20	1	BET	0.43993	0.003
**548**	7-MHB-L23	0.03	BET	0.331606	0.253
**549**	7-MHB-M1:	10	BET	0.318027	0.216
**550**	7-MHB-M1:	100	BET	0.548556	0.001
**551**	7-MHB-M2(	10	BET	0.237085	0.467
**552**	7-MHB-M2:	0.3	BET	0.250901	0.342
**553**	7-MHB-N12	100	BET	0.167809	0.806
**554**	7-MHB-N13	1000	BET	0.517692	0.003
**555**	7-MHB-N2(	100	BET	0.537798	0
**556**	7-MHB-N23	3	BET	0.426635	0.009
**557**	7-MHB-O12	1000	BET	0.521375	0
**558**	7-MHB-O2(	1000	BET	0.538549	0
**559**	7-MHB-O23	30	BET	0.414816	0.006
**560**	7-MHB-P12	10000	BET	0.529312	0
**561**	7-MHB-P13	10000	BET	0.504673	0.007
**562**	7-MHB-P2C	10000	BET	0.578143	0
**563**	7-MHB-P23	300	BET	0.545762	0.001
**564**	8-MHB-K22	1	BET	0.371086	0.076
**565**	8-MHB-L22	10	BET	0.447751	0.004
**566**	8-MHB-M2:	100	BET	0.379776	0.034
**567**	8-MHB-N22	1000	BET	0.545318	0
**568**	8-MHB-O22	10000	BET	0.377306	0.057
**569**	1-MHB-A3-'	10000	HDAC	0.406287	0.011
**570**	1-MHB-B3-'	1000	HDAC	0.414637	0
**571**	1-MHB-C3-'	100	HDAC	0.361043	0
**572**	1-MHB-D3-	10	HDAC	0.260899	0.4
**573**	1-MHB-E3-\	1	HDAC	0.309753	0.049

**574**	1-MHB-L12	0.1	HDAC	0.312807	0.007
**575**	1-MHB-M1:	1	HDAC	0.431592	0
**576**	1-MHB-N12	10	HDAC	0.371281	0.024
**577**	1-MHB-O12	100	HDAC	0.412556	0.085
**578**	1-MHB-P12	1000	HDAC	0.304284	0.146
**579**	3-MHB-A4-	1000	HDAC	0.381883	0.135
**580**	3-MHB-B4-	100	HDAC	0.25058	0.697
**581**	3-MHB-C4-	10	HDAC	0.395859	0.002
**582**	3-MHB-D4-	1	HDAC	0.227239	0.236
**583**	3-MHB-E4-	0.1	HDAC	0.373366	0.003
**584**	3-MHB-F7-	1000	HDAC	0.373331	0.237
**585**	3-MHB-G7-	100	HDAC	0.320351	0.118
**586**	3-MHB-G12	1000000	HDAC	0.195286	0.037
**587**	3-MHB-H7-	10	HDAC	0.457616	0
**588**	3-MHB-H12	100000	HDAC	0.435855	0
**589**	3-MHB-I7-C	1	HDAC	0.372039	0
**590**	3-MHB-I12-	10000	HDAC	0.371947	0
**591**	3-MHB-J7-C	0.1	HDAC	0.315608	0.045
**592**	3-MHB-J12	1000	HDAC	0.42336	0
**593**	3-MHB-K3-	1	HDAC	0.219469	0.636
**594**	3-MHB-K12	100	HDAC	0.450035	0
**595**	3-MHB-L3-	10	HDAC	0.289537	0.188
**596**	3-MHB-M3-	100	HDAC	0.391641	0
**597**	3-MHB-N3-	1000	HDAC	0.226246	0.719
**598**	3-MHB-O3-	10000	HDAC	0.055372	0.952
**599**	7-MHB-A5-	10000	HDAC	0.378843	0.012
**600**	7-MHB-A7-	10000	HDAC	0.438143	0.017
**601**	7-MHB-A9-	1000	HDAC	0.343583	0.088
**602**	7-MHB-A12	10000	HDAC	0.350721	0.062
**603**	7-MHB-B5-	1000	HDAC	0.347241	0.033
**604**	7-MHB-B12	1000	HDAC	0.497327	0
**605**	7-MHB-C5-	100	HDAC	0.080318	0.929
**606**	7-MHB-C7-	100	HDAC	0.274964	0.154
**607**	7-MHB-C9-	100	HDAC	0.465377	0
**608**	7-MHB-D7-	10	HDAC	0.340692	0.067

**609**	7-MHB-D9-	10	HDAC	0.467098	0
**610**	7-MHB-D12	100	HDAC	0.43528	0
**611**	7-MHB-E5-I	10	HDAC	0.493793	0
**612**	7-MHB-E7-(	1	HDAC	0.487597	0
**613**	7-MHB-E9-(	1	HDAC	0.415014	0
**614**	7-MHB-E12	10	HDAC	0.379973	0
**615**	7-MHB-F5-I	1	HDAC	0.407843	0
**616**	7-MHB-F7-I	10000	HDAC	0.349618	0.07
**617**	7-MHB-F9-(	0.1	HDAC	0.491796	0
**618**	7-MHB-F12	1	HDAC	0.446305	0
**619**	7-MHB-F19	10000	HDAC	0.299534	0.131
**620**	7-MHB-G7-	1000	HDAC	0.494352	0
**621**	7-MHB-G19	1000	HDAC	0.069075	0.795
**622**	7-MHB-H7-	100	HDAC	0.489264	0
**623**	7-MHB-I7-F	10	HDAC	0.387891	0.002
**624**	7-MHB-I19-	100	HDAC	0.300958	0.002
**625**	7-MHB-J7-F	1	HDAC	0.042261	0.964
**626**	7-MHB-J19	10	HDAC	0.472618	0
**627**	7-MHB-K4-I	1	HDAC	0.279485	0.158
**628**	7-MHB-K11	1	HDAC	0.317585	0.026
**629**	7-MHB-K18	1	HDAC	0.475192	0
**630**	7-MHB-K19	1	HDAC	0.298178	0
**631**	7-MHB-L2-T	0.1	HDAC	0.175072	0.717
**632**	7-MHB-L4-I	10	HDAC	0.280115	0.197
**633**	7-MHB-L5-I	1	HDAC	0.34363	0.001
**634**	7-MHB-L8-A	1	HDAC	0.433472	0
**635**	7-MHB-L10	1	HDAC	0.439652	0
**636**	7-MHB-L11	10	HDAC	0.428479	0
**637**	7-MHB-L14	1	HDAC	0.416084	0.001
**638**	7-MHB-L16	1	HDAC	0.439495	0
**639**	7-MHB-L18	10	HDAC	0.293124	0.001
**640**	7-MHB-M2-	1	HDAC	0.282903	0.146
**641**	7-MHB-M5-	10	HDAC	0.258419	0.31
**642**	7-MHB-M8-	10	HDAC	0.477472	0
**643**	7-MHB-M1(	10	HDAC	0.456853	0

**644**	7-MHB-M1	100	HDAC	0.557005	0
**645**	7-MHB-M14	10	HDAC	0.467867	0
**646**	7-MHB-M1(	10	HDAC	0.434657	0
**647**	7-MHB-M1	100	HDAC	0.285751	0.236
**648**	7-MHB-N2-	10	HDAC	0.201334	0.692
**649**	7-MHB-N4-	100	HDAC	0.395318	0
**650**	7-MHB-N5-	100	HDAC	0.458202	0
**651**	7-MHB-N8-	100	HDAC	0.517375	0
**652**	7-MHB-N1(	100	HDAC	0.472581	0
**653**	7-MHB-N14	100	HDAC	0.476402	0
**654**	7-MHB-N16	100	HDAC	0.250959	0.049
**655**	7-MHB-N18	1000	HDAC	0.198435	0.497
**656**	7-MHB-O2-	100	HDAC	0.078483	0.945
**657**	7-MHB-O4-	1000	HDAC	0.444575	0
**658**	7-MHB-O5-	1000	HDAC	0.336933	0.077
**659**	7-MHB-O8-	1000	HDAC	0.372246	0.018
**660**	7-MHB-O1(	1000	HDAC	0.582829	0
**661**	7-MHB-O11	1000	HDAC	0.356203	0.031
**662**	7-MHB-O14	1000	HDAC	0.506284	0
**663**	7-MHB-O16	1000	HDAC	0.422664	0
**664**	7-MHB-P2-	1000	HDAC	0.460593	0
**665**	7-MHB-P4-	10000	HDAC	0.350825	0.05
**666**	7-MHB-P5-	10000	HDAC	0.285793	0.159
**667**	7-MHB-P8-	10000	HDAC	0.260771	0.292
**668**	7-MHB-P1(	10000	HDAC	0.334054	0.064
**669**	7-MHB-P11	10000	HDAC	0.288581	0.147
**670**	7-MHB-P14	10000	HDAC	0.354801	0.007
**671**	7-MHB-P16	10000	HDAC	0.493014	0
**672**	7-MHB-P18	10000	HDAC	0.43491	0
**0**	2-O3B-A16-	10000	EGFR	0.646318	0
**1**	2-O3B-A19-	10000	EGFR	0.643475	0
**2**	2-O3B-B19-	1000	EGFR	0.617602	0
**3**	2-O3B-C16-	1000	EGFR	0.658241	0
**4**	2-O3B-C19-	100	EGFR	0.592095	0
**5**	2-O3B-D16	100	EGFR	0.648368	0

**6**	2-O3B-D19	10	EGFR	0.157441	0.956
**7**	2-O3B-E16-	10	EGFR	0.554891	0
**8**	2-O3B-E19-	1	EGFR	0.405588	0.004
**9**	2-O3B-F16-	1	EGFR	0.200374	0.836
**10**	2-O3B-K11-	0.1	EGFR	0.45071	0
**11**	2-O3B-L11-	1	EGFR	0.248192	0.244
**12**	2-O3B-L16-	0.25	EGFR	0.201711	0.811
**13**	2-O3B-L19-	0.1	EGFR	0.477337	0
**14**	2-O3B-M11	10	EGFR	0.687385	0
**15**	2-O3B-M16	2.5	EGFR	0.2125	0.218
**16**	2-O3B-M19	1	EGFR	0.290523	0.189
**17**	2-O3B-N16	25	EGFR	0.351583	0.031
**18**	2-O3B-N19	10	EGFR	0.352625	0.006
**19**	2-O3B-O11	100	EGFR	0.662339	0
**20**	2-O3B-O16	250	EGFR	0.635191	0
**21**	2-O3B-O19	100	EGFR	0.476957	0
**22**	2-O3B-P11-	1000	EGFR	0.63311	0
**23**	2-O3B-P16-	2500	EGFR	0.596407	0
**24**	2-O3B-P19-	1000	EGFR	0.613344	0
**25**	3-O3B-F21-	10000	EGFR	0.620598	0
**26**	3-O3B-G20	1000	EGFR	0.611068	0
**27**	3-O3B-G21	1000	EGFR	0.596506	0
**28**	3-O3B-H20	100	EGFR	0.589192	0
**29**	3-O3B-H21	100	EGFR	0.440471	0
**30**	3-O3B-I20-	10	EGFR	0.492871	0
**31**	3-O3B-I21-	10	EGFR	0.369321	0.014
**32**	3-O3B-J20-	1	EGFR	0.389931	0.003
**33**	3-O3B-J21-	1	EGFR	0.358532	0.027
**34**	3-O3B-K4-(	1	EGFR	0.072154	0.956
**35**	3-O3B-K18-	0.1	EGFR	0.22912	0.074
**36**	3-O3B-K20-	0.1	EGFR	0.218565	0.389
**37**	3-O3B-L4-C	10	EGFR	0.569693	0
**38**	3-O3B-L18-	1	EGFR	0.04842	0.972
**39**	3-O3B-M18	10	EGFR	0.47807	0
**40**	3-O3B-N4-(	100	EGFR	0.088317	0.867

**41**	3-O3B-N18	100	EGFR	0.663711	0
**42**	3-O3B-O4-(	1000	EGFR	0.653433	0
**43**	3-O3B-P4-(	10000	EGFR	0.477908	0.022
**44**	3-O3B-P18-	1000	EGFR	0.63496	0
**45**	4-O3B-F13-	1000	EGFR	0.632696	0
**46**	4-O3B-G13	100	EGFR	0.639256	0
**47**	4-O3B-G16	10000	EGFR	0.604222	0
**48**	4-O3B-H13	10	EGFR	0.645719	0
**49**	4-O3B-H16	1000	EGFR	0.638531	0
**50**	4-O3B-I13-	1	EGFR	0.253973	0.438
**51**	4-O3B-I16-	100	EGFR	0.419869	0
**52**	4-O3B-J13-	0.1	EGFR	0.153602	0.417
**53**	4-O3B-J16-	10	EGFR	0.063725	0.992
**54**	4-O3B-K7-I	1	EGFR	0.425749	0
**55**	4-O3B-K13-	0.1	EGFR	0.064553	0.924
**56**	4-O3B-K16-	1	EGFR	0.437893	0
**57**	4-O3B-L7-I	10	EGFR	0.430846	0.001
**58**	4-O3B-L13-	1	EGFR	0.613582	0
**59**	4-O3B-M7-I	100	EGFR	0.471016	0
**60**	4-O3B-M13	10	EGFR	0.567376	0
**61**	4-O3B-N13	100	EGFR	0.655858	0
**62**	4-O3B-O7-I	1000	EGFR	0.6303	0
**63**	4-O3B-P7-I	10000	EGFR	0.542675	0
**64**	4-O3B-P13-	1000	EGFR	0.656357	0
**65**	5-O3B-F4-P	1000	EGFR	0.617858	0
**66**	5-O3B-F7-A	1000	EGFR	0.584756	0
**67**	5-O3B-G4-I	100	EGFR	0.618248	0
**68**	5-O3B-G7-/	100	EGFR	0.397432	0.003
**69**	5-O3B-H4-I	10	EGFR	0.641583	0
**70**	5-O3B-H7-/	10	EGFR	0.322745	0.071
**71**	5-O3B-I4-P	1	EGFR	0.469719	0
**72**	5-O3B-I7-A	1	EGFR	0.260747	0.413
**73**	5-O3B-J4-P	0.1	EGFR	0.334853	0.077
**74**	5-O3B-J7-A	0.1	EGFR	0.240556	0.669
**75**	5-O3B-K7-(	0.1	EGFR	0.226986	0.723

**76**	5-O3B-L7-C	1	EGFR	0.3165	0.071
**77**	5-O3B-M7-(	10	EGFR	0.324608	0.094
**78**	5-O3B-O7-C	100	EGFR	0.379258	0.063
**79**	5-O3B-P7-C	1000	EGFR	0.58408	0
**80**	2-O3B-A15-	2500	VEGFR	0.271683	0.259
**81**	2-O3B-A17-	10000	VEGFR	0.215552	0.91
**82**	2-O3B-A20-	10000	VEGFR	0.176011	0.969
**83**	2-O3B-B15-	250	VEGFR	0.633314	0
**84**	2-O3B-B17-	1000	VEGFR	0.330834	0.377
**85**	2-O3B-B20-	1000	VEGFR	0.366195	0.016
**86**	2-O3B-C15-	25	VEGFR	0.291736	0.005
**87**	2-O3B-C17-	100	VEGFR	0.485747	0
**88**	2-O3B-D15	2.5	VEGFR	0.509377	0
**89**	2-O3B-D17	10	VEGFR	0.442573	0
**90**	2-O3B-D20	100	VEGFR	0.296383	0.003
**91**	2-O3B-E17-	1	VEGFR	0.28875	0.209
**92**	2-O3B-E20-	10	VEGFR	0.449564	0
**93**	2-O3B-F13-	10000	VEGFR	0.136507	0.989
**94**	2-O3B-F15-	0.25	VEGFR	0.384242	0
**95**	2-O3B-F19-	10000	VEGFR	0.171461	0.996
**96**	2-O3B-F20-	1	VEGFR	0.320427	0.001
**97**	2-O3B-F21-	10000	VEGFR	0.16932	0.911
**98**	2-O3B-G10	10000	VEGFR	0.395131	0.049
**99**	2-O3B-G13	1000	VEGFR	0.127219	0.982
**100**	2-O3B-G19	1000	VEGFR	0.576546	0
**101**	2-O3B-G21	1000	VEGFR	0.351606	0
**102**	2-O3B-H10	1000	VEGFR	0.438562	0
**103**	2-O3B-H13	100	VEGFR	0.367402	0.018
**104**	2-O3B-H21	100	VEGFR	0.353188	0.003
**105**	2-O3B-I10-,	100	VEGFR	0.197373	0.839
**106**	2-O3B-I13-,	10	VEGFR	0.560797	0
**107**	2-O3B-I19-I	100	VEGFR	0.387651	0.004
**108**	2-O3B-I21-'	10	VEGFR	0.439191	0.003
**109**	2-O3B-J10-	10	VEGFR	0.386013	0
**110**	2-O3B-J13-	1	VEGFR	0.36392	0.001

**111**	2-O3B-J19-	10	VEGFR	0.274064	0.566
**112**	2-O3B-J21-	1	VEGFR	0.41682	0
**113**	2-O3B-K10-	1	VEGFR	0.324445	0
**114**	2-O3B-K13-	0.1	VEGFR	0.504027	0
**115**	2-O3B-K17-	1	VEGFR	0.247605	0.322
**116**	2-O3B-K19-	1	VEGFR	0.202259	0.67
**117**	2-O3B-L12-	0.1	VEGFR	0.513251	0
**118**	2-O3B-L13-	1	VEGFR	0.446285	0
**119**	2-O3B-L21-	0.1	VEGFR	0.226238	0.565
**120**	2-O3B-M12	1	VEGFR	0.369827	0.002
**121**	2-O3B-M13	10	VEGFR	0.634488	0
**122**	2-O3B-M17	10	VEGFR	0.299875	0.048
**123**	2-O3B-M21	1	VEGFR	0.124379	0.972
**124**	2-O3B-N12	10	VEGFR	0.253061	0.002
**125**	2-O3B-N13	100	VEGFR	0.373414	0.02
**126**	2-O3B-N17	100	VEGFR	0.313547	0.017
**127**	2-O3B-N21	10	VEGFR	0.385048	0.006
**128**	2-O3B-O12	100	VEGFR	0.308941	0
**129**	2-O3B-O17	1000	VEGFR	0.345021	0.089
**130**	2-O3B-O21	100	VEGFR	0.141368	0.973
**131**	2-O3B-P12-	1000	VEGFR	0.305731	0
**132**	2-O3B-P13-	1000	VEGFR	0.235877	0.881
**133**	2-O3B-P17-	10000	VEGFR	0.382602	0.021
**134**	2-O3B-P21-	1000	VEGFR	0.324469	0.031
**135**	3-O3B-A3-C	1000	VEGFR	0.214187	0.894
**136**	3-O3B-A6-F	1000	VEGFR	0.114724	0.995
**137**	3-O3B-A18-	1000	VEGFR	0.392236	0
**138**	3-O3B-B3-C	100	VEGFR	0.403897	0
**139**	3-O3B-B6-F	100	VEGFR	0.358424	0.003
**140**	3-O3B-B18-	100	VEGFR	0.194725	0.115
**141**	3-O3B-C3-C	10	VEGFR	0.129449	0.98
**142**	3-O3B-C6-F	10	VEGFR	0.470601	0.001
**143**	3-O3B-C18-	10	VEGFR	0.309339	0.004
**144**	3-O3B-D3-C	1	VEGFR	0.284188	0.007
**145**	3-O3B-D6-F	1	VEGFR	0.301066	0

**146**	3-O3B-D18	1	VEGFR	0.324935	0
**147**	3-O3B-E3-C	0.1	VEGFR	0.271568	0.01
**148**	3-O3B-E6-F	0.1	VEGFR	0.315846	0.001
**149**	3-O3B-E18-	0.1	VEGFR	0.241819	0.322
**150**	3-O3B-F18-	1000	VEGFR	0.280714	0.671
**151**	3-O3B-G18	100	VEGFR	0.110921	0.792
**152**	3-O3B-H18	10	VEGFR	0.141599	0.966
**153**	3-O3B-I18-I	1	VEGFR	0.116524	0.985
**154**	3-O3B-J18-	0.1	VEGFR	0.113116	0.984
**155**	4-O3B-A12-	10000	VEGFR	0.190158	0.998
**156**	4-O3B-A15-	2500	VEGFR	0.317699	0.002
**157**	4-O3B-A20-	10000	VEGFR	0.127439	0.951
**158**	4-O3B-B12-	1000	VEGFR	0.122296	0.998
**159**	4-O3B-B15-	250	VEGFR	0.259131	0
**160**	4-O3B-B20-	1000	VEGFR	0.276708	0.003
**161**	4-O3B-C15-	25	VEGFR	0.135445	0.977
**162**	4-O3B-D12	100	VEGFR	0.22107	0.67
**163**	4-O3B-D15	2.5	VEGFR	0.067049	0.997
**164**	4-O3B-D20	100	VEGFR	0.140441	0.912
**165**	4-O3B-E12-	10	VEGFR	0.124357	0.934
**166**	4-O3B-E20-	10	VEGFR	0.247022	0.129
**167**	4-O3B-F12-	1	VEGFR	0.32297	0.066
**168**	4-O3B-F15-	0.25	VEGFR	0.078197	0.988
**169**	4-O3B-F20-	1	VEGFR	0.226906	0.022
**170**	4-O3B-L16-	1	VEGFR	0.393321	0
**171**	4-O3B-M16	10	VEGFR	0.08365	0.998
**172**	4-O3B-N16	100	VEGFR	0.219701	0.03
**173**	4-O3B-O16	1000	VEGFR	0.564478	0
**174**	4-O3B-P16-	10000	VEGFR	0.486002	0.001
**175**	2-O3B-L10-	1	PI3K	0.045074	0.931
**176**	2-O3B-M10	10	PI3K	0.172433	0.739
**177**	2-O3B-N10	100	PI3K	0.132079	0.723
**178**	2-O3B-O10	1000	PI3K	0.474861	0
**179**	2-O3B-P10-	10000	PI3K	0.430908	0
**180**	3-O3B-A16-	2500	PI3K	0.070064	0.94

**181**	3-O3B-C16·	250	PI3K	0.126405	0.723
**182**	3-O3B-D16	25	PI3K	0.066595	0.868
**183**	3-O3B-E16·	2.5	PI3K	0.070058	0.798
**184**	3-O3B-F16·	0.25	PI3K	0.313934	0.031
**185**	3-O3B-F17·	100000	PI3K	0.398165	0.146
**186**	3-O3B-F19·	500	PI3K	0.48241	0
**187**	3-O3B-G17	10000	PI3K	0.085457	0.694
**188**	3-O3B-G19	50	PI3K	0.474388	0
**189**	3-O3B-H17	1000	PI3K	0.432535	0
**190**	3-O3B-I17-I	100	PI3K	0.369027	0
**191**	3-O3B-I19-I	5	PI3K	0.442038	0
**192**	3-O3B-J17-	10	PI3K	0.350902	0.004
**193**	3-O3B-J19-	0.5	PI3K	0.403612	0
**194**	3-O3B-K19·	0.05	PI3K	0.076269	0.759
**195**	3-O3B-L8-P	1	PI3K	0.463201	0
**196**	3-O3B-L21-	0.1	PI3K	0.100073	0.589
**197**	3-O3B-M8-I	10	PI3K	0.358868	0.003
**198**	3-O3B-M21	1	PI3K	0.057143	0.929
**199**	3-O3B-N8-F	100	PI3K	0.484324	0
**200**	3-O3B-N21	10	PI3K	0.060672	0.934
**201**	3-O3B-O8-F	1000	PI3K	0.440649	0.002
**202**	3-O3B-O21	100	PI3K	0.35717	0.027
**203**	3-O3B-P8-F	10000	PI3K	0.402903	0.07
**204**	3-O3B-P21·	1000	PI3K	0.468973	0
**205**	4-O3B-A19·	2500	PI3K	0.414952	0
**206**	4-O3B-B19·	250	PI3K	0.379399	0.013
**207**	4-O3B-C19·	25	PI3K	0.382814	0.006
**208**	4-O3B-D19	2.5	PI3K	0.033559	1
**209**	4-O3B-E19·	0.25	PI3K	0.32171	0.086
**210**	4-O3B-F14·	1000	PI3K	0.437466	0.011
**211**	4-O3B-G2-1	2500	PI3K	0.471864	0
**212**	4-O3B-G5-S	10000	PI3K	0.505032	0
**213**	4-O3B-G14	100	PI3K	0.439406	0.005
**214**	4-O3B-G20	10000	PI3K	0.432941	0.024
**215**	4-O3B-H2-1	250	PI3K	0.454254	0

**216**	4-O3B-H5-S	1000	PI3K	0.347326	0
**217**	4-O3B-H14	10	PI3K	0.373629	0
**218**	4-O3B-H20	1000	PI3K	0.392715	0
**219**	4-O3B-I2-T(	25	PI3K	0.308949	0.302
**220**	4-O3B-I5-S(	100	PI3K	0.419682	0
**221**	4-O3B-I14-I	1	PI3K	0.378192	0
**222**	4-O3B-I20-I	100	PI3K	0.229473	0.482
**223**	4-O3B-J2-T(	2.5	PI3K	0.05648	0.938
**224**	4-O3B-J5-S(	10	PI3K	0.451948	0
**225**	4-O3B-J20-	10	PI3K	0.125088	0.695
**226**	4-O3B-K2-T	0.25	PI3K	0.085612	0.935
**227**	4-O3B-K4-D	0.1	PI3K	0.431666	0
**228**	4-O3B-K5-S	1	PI3K	0.417625	0
**229**	4-O3B-K14-	0.1	PI3K	0.152298	0.869
**230**	4-O3B-K20-	1	PI3K	0.330264	0.051
**231**	4-O3B-L4-D	1	PI3K	0.383367	0
**232**	4-O3B-L14-	0.1	PI3K	0.325729	0.006
**233**	4-O3B-L15-	1	PI3K	0.195193	0.135
**234**	4-O3B-L21-	0.1	PI3K	0.387627	0.002
**235**	4-O3B-M14	1	PI3K	0.391374	0.001
**236**	4-O3B-M15	10	PI3K	0.41509	0
**237**	4-O3B-M21	1	PI3K	0.389474	0
**238**	4-O3B-N4-D	10	PI3K	0.354182	0.004
**239**	4-O3B-N14	10	PI3K	0.361451	0.001
**240**	4-O3B-N15	100	PI3K	0.405079	0
**241**	4-O3B-N21	10	PI3K	0.433223	0
**242**	4-O3B-O4-D	100	PI3K	0.44539	0
**243**	4-O3B-O14	100	PI3K	0.443888	0.006
**244**	4-O3B-O15	1000	PI3K	0.509782	0
**245**	4-O3B-O21	100	PI3K	0.421165	0
**246**	4-O3B-P4-D	1000	PI3K	0.395209	0
**247**	4-O3B-P14-	1000	PI3K	0.433597	0.018
**248**	4-O3B-P15-	10000	PI3K	0.46694	0
**249**	4-O3B-P21-	1000	PI3K	0.440088	0.016
**250**	5-O3B-A6-L	2500	PI3K	0.431048	0.011

**251**	5-O3B-A7-A	1000	PI3K	0.425453	0
**252**	5-O3B-A16-	2500	PI3K	0.438744	0
**253**	5-O3B-A17-	10000	PI3K	0.421245	0
**254**	5-O3B-B6-L	250	PI3K	0.397869	0.002
**255**	5-O3B-B7-A	100	PI3K	0.427341	0
**256**	5-O3B-B17-	1000	PI3K	0.394056	0
**257**	5-O3B-C6-L	25	PI3K	0.407619	0
**258**	5-O3B-C7-A	10	PI3K	0.449087	0
**259**	5-O3B-C16-	250	PI3K	0.459782	0
**260**	5-O3B-C17-	100	PI3K	0.337676	0.017
**261**	5-O3B-D6-L	2.5	PI3K	0.339341	0.011
**262**	5-O3B-D7-A	1	PI3K	0.434492	0
**263**	5-O3B-D16	25	PI3K	0.43438	0
**264**	5-O3B-D17	10	PI3K	0.374067	0
**265**	5-O3B-E6-L	0.25	PI3K	0.355832	0.003
**266**	5-O3B-E7-A	0.1	PI3K	0.37616	0.001
**267**	5-O3B-E16-	2.5	PI3K	0.383299	0.001
**268**	5-O3B-E17-	1	PI3K	0.341275	0.001
**269**	5-O3B-F11-	10000	PI3K	0.509487	0
**270**	5-O3B-F16-	0.25	PI3K	0.343057	0.001
**271**	5-O3B-G9-S	10000	PI3K	0.466756	0
**272**	5-O3B-G11	1000	PI3K	0.462802	0
**273**	5-O3B-H9-S	1000	PI3K	0.381011	0.001
**274**	5-O3B-H11	100	PI3K	0.42769	0
**275**	5-O3B-I9-Se	100	PI3K	0.354589	0.016
**276**	5-O3B-I11-	10	PI3K	0.415241	0
**277**	5-O3B-J9-S	10	PI3K	0.405339	0
**278**	5-O3B-J11-	1	PI3K	0.440191	0
**279**	5-O3B-K9-S	1	PI3K	0.441218	0
**280**	5-O3B-L14-	0.1	PI3K	0.384611	0
**281**	5-O3B-L20-	1	PI3K	0.481067	0
**282**	5-O3B-L23-	0.1	PI3K	0.401925	0.003
**283**	5-O3B-M14	1	PI3K	0.360519	0
**284**	5-O3B-M20	10	PI3K	0.41988	0
**285**	5-O3B-M23	1	PI3K	0.427812	0

**286**	5-O3B-N14	10	PI3K	0.372694	0
**287**	5-O3B-N20	100	PI3K	0.441377	0
**288**	5-O3B-N23	10	PI3K	0.390646	0
**289**	5-O3B-O14	100	PI3K	0.432769	0
**290**	5-O3B-O20	1000	PI3K	0.45124	0
**291**	5-O3B-O23	100	PI3K	0.432278	0.011
**292**	5-O3B-P14	1000	PI3K	0.453507	0
**293**	5-O3B-P20	10000	PI3K	0.427657	0.013
**294**	5-O3B-P23	1000	PI3K	0.417888	0.025
**295**	6-O3B-A8-T	10000	PI3K	0.427095	0.001
**296**	6-O3B-B8-T	1000	PI3K	0.440118	0
**297**	6-O3B-C8-T	100	PI3K	0.347817	0.002
**298**	6-O3B-D8-T	10	PI3K	0.183717	0.596
**299**	6-O3B-E8-T	1	PI3K	0.063451	0.938
**300**	6-O3B-L6-G	1	PI3K	0.051553	0.976
**301**	6-O3B-M6-	10	PI3K	0.058392	0.914
**302**	6-O3B-N6-(	100	PI3K	0.305884	0.083
**303**	6-O3B-O6-(	1000	PI3K	0.402433	0
**304**	6-O3B-P6-G	10000	PI3K	0.423356	0.042
**305**	1-O3B-F11-	10000	Topoisome	0.593945	0
**306**	1-O3B-G11	1000	Topoisome	0.57837	0
**307**	1-O3B-G20	1000	Topoisome	0.312102	0.712
**308**	1-O3B-H11	100	Topoisome	0.544213	0
**309**	1-O3B-H20	100	Topoisome	0.551695	0
**310**	1-O3B-I11-	10	Topoisome	0.365703	0.08
**311**	1-O3B-I20-	10	Topoisome	0.333833	0.149
**312**	1-O3B-J11-	1	Topoisome	0.184551	0.357
**313**	1-O3B-J20-	1	Topoisome	0.180097	0.29
**314**	1-O3B-K11	1	Topoisome	0.561366	0
**315**	1-O3B-K20	0.1	Topoisome	0.193148	0.72
**316**	1-O3B-L11-	10	Topoisome	0.618807	0
**317**	1-O3B-L14-	1	Topoisome	0.142327	0.704
**318**	1-O3B-M11	100	Topoisome	0.564692	0
**319**	1-O3B-M14	10	Topoisome	0.54859	0
**320**	1-O3B-N14	100	Topoisome	0.569943	0

321	1-O3B-O11	1000	Topoisome	0.416168	0.05
322	1-O3B-O14	1000	Topoisome	0.610378	0
323	1-O3B-P11·	10000	Topoisome	0.391234	0.15
324	1-O3B-P14·	10000	Topoisome	0.428185	0.041
325	3-O3B-A11·	10000	Topoisome	0.605963	0
326	3-O3B-B11·	1000	Topoisome	0.579008	0
327	3-O3B-C11·	100	Topoisome	0.577222	0
328	3-O3B-D11	10	Topoisome	0.32017	0.017
329	3-O3B-E11-	1	Topoisome	0.340581	0.002
330	3-O3B-G9-[	1000	Topoisome	0.348206	0.518
331	3-O3B-G10	10000	Topoisome	0.454404	0.004
332	3-O3B-H9-[	100	Topoisome	0.58638	0
333	3-O3B-H10	1000	Topoisome	0.224417	0.632
334	3-O3B-I9-D	10	Topoisome	0.269206	0.449
335	3-O3B-I10-	100	Topoisome	0.25341	0.454
336	3-O3B-J9-D	1	Topoisome	0.269605	0.371
337	3-O3B-J10-	10	Topoisome	0.284239	0.296
338	3-O3B-K7-I	0.1	Topoisome	0.325284	0.511
339	3-O3B-K9-[	0.1	Topoisome	0.343868	0.008
340	3-O3B-K10·	1	Topoisome	0.274101	0.011
341	3-O3B-L6-D	0.1	Topoisome	0.182732	0.439
342	3-O3B-L7-I	1	Topoisome	0.403417	0.01
343	3-O3B-L9-V	0.5	Topoisome	0.330419	0.004
344	3-O3B-L10-	0.1	Topoisome	0.17685	0.201
345	3-O3B-L16-	1	Topoisome	0.162109	0.421
346	3-O3B-M6-I	1	Topoisome	0.248988	0.036
347	3-O3B-M7-I	10	Topoisome	0.462169	0.001
348	3-O3B-M9-'	5	Topoisome	0.323409	0.011
349	3-O3B-M1C	1	Topoisome	0.396628	0.013
350	3-O3B-M16	10	Topoisome	0.322523	0.014
351	3-O3B-N6-[	10	Topoisome	0.353845	0.039
352	3-O3B-N9-\	50	Topoisome	0.573982	0
353	3-O3B-N10	10	Topoisome	0.59223	0
354	3-O3B-N16	100	Topoisome	0.301377	0.004
355	3-O3B-O6-[	100	Topoisome	0.566599	0

356	3-O3B-O7-I	100	Topoisome	0.568705	0
357	3-O3B-O9-\	500	Topoisome	0.572302	0
358	3-O3B-O10	100	Topoisome	0.576938	0
359	3-O3B-O16	1000	Topoisome	0.285917	0.417
360	3-O3B-P6-D	1000	Topoisome	0.379204	0.225
361	3-O3B-P7-I	1000	Topoisome	0.349058	0.547
362	3-O3B-P9-\	5000	Topoisome	0.378732	0.409
363	3-O3B-P10-	1000	Topoisome	0.358267	0.205
364	3-O3B-P16-	10000	Topoisome	0.359603	0.074
365	1-O3B-A10-	10000	Mitotic	0.720992	0
366	1-O3B-A13-	1000	Mitotic	0.724296	0
367	1-O3B-A18-	1000	Mitotic	0.735419	0
368	1-O3B-B10-	1000	Mitotic	0.733134	0
369	1-O3B-B13-	100	Mitotic	0.129321	0.588
370	1-O3B-B18-	100	Mitotic	0.735447	0
371	1-O3B-C10-	100	Mitotic	0.739241	0
372	1-O3B-C13-	10	Mitotic	0.294995	0.457
373	1-O3B-C18-	10	Mitotic	0.725048	0
374	1-O3B-D10	10	Mitotic	0.714711	0
375	1-O3B-D13	1	Mitotic	0.346338	0.029
376	1-O3B-D18	1	Mitotic	0.26599	0.495
377	1-O3B-E10-	1	Mitotic	0.484977	0
378	1-O3B-E13-	0.1	Mitotic	0.379616	0.019
379	1-O3B-E18-	0.1	Mitotic	0.309614	0.564
380	1-O3B-F13-	1000	Mitotic	0.737713	0
381	1-O3B-G13	100	Mitotic	0.419006	0.01
382	1-O3B-G15	1000	Mitotic	0.726683	0
383	1-O3B-H13	10	Mitotic	0.132949	0.891
384	1-O3B-H15	100	Mitotic	0.703037	0
385	1-O3B-I13-'	1	Mitotic	0.303072	0.447
386	1-O3B-I15-I	10	Mitotic	0.731653	0
387	1-O3B-J13-	0.1	Mitotic	0.096288	0.869
388	1-O3B-J15-	1	Mitotic	0.700621	0
389	1-O3B-K7-\	0.1	Mitotic	0.226072	0.695
390	1-O3B-K15-	0.1	Mitotic	0.365249	0.079

**391**	1-O3B-L7-V	1	Mitotic	0.348528	0.082
**392**	1-O3B-L20-	0.1	Mitotic	0.282365	0.23
**393**	1-O3B-M7-'	10	Mitotic	0.161687	0.796
**394**	1-O3B-M2C	1	Mitotic	0.342348	0.27
**395**	1-O3B-N20	10	Mitotic	0.134494	0.59
**396**	1-O3B-O7-\	100	Mitotic	0.448702	0.003
**397**	1-O3B-O20	100	Mitotic	0.307091	0.253
**398**	1-O3B-P7-\	1000	Mitotic	0.741519	0
**399**	1-O3B-P20-	1000	Mitotic	0.723326	0
**400**	3-O3B-A7-D	1000	Mitotic	0.738689	0
**401**	3-O3B-B7-D	100	Mitotic	0.733655	0
**402**	3-O3B-C7-D	10	Mitotic	0.734983	0
**403**	3-O3B-D7-D	1	Mitotic	0.409379	0.04
**404**	3-O3B-E7-D	0.1	Mitotic	0.242285	0.763
**405**	6-O3B-L19-	1	Mitotic	0.337396	0.296
**406**	6-O3B-M19	10	Mitotic	0.202391	0.553
**407**	6-O3B-N19	100	Mitotic	0.203167	0.566
**408**	6-O3B-O19	1000	Mitotic	0.695861	0
**409**	6-O3B-P19-	10000	Mitotic	0.672231	0
**410**	2-O3B-A12-	250	MEK1/2	0.679588	0
**411**	2-O3B-B12-	25	MEK1/2	0.748321	0
**412**	2-O3B-D12	2.5	MEK1/2	0.763081	0
**413**	2-O3B-E12-	0.25	MEK1/2	0.775314	0
**414**	2-O3B-F12-	2.5000000	MEK1/2	0.492484	0.032
**415**	2-O3B-F14-	1000	MEK1/2	0.6532	0
**416**	2-O3B-G14	100	MEK1/2	0.770854	0
**417**	2-O3B-H14	10	MEK1/2	0.791764	0
**418**	2-O3B-I14-	1	MEK1/2	0.50841	0.031
**419**	2-O3B-K14-	0.1	MEK1/2	0.448297	0.057
**420**	2-O3B-L20-	1	MEK1/2	0.278478	0.391
**421**	2-O3B-M2C	10	MEK1/2	0.48135	0.01
**422**	2-O3B-N20	100	MEK1/2	0.811004	0
**423**	2-O3B-O20	1000	MEK1/2	0.753872	0
**424**	2-O3B-P20-	10000	MEK1/2	0.68725	0
**425**	4-O3B-A10-	1000	MEK1/2	0.702212	0

**426**	4-O3B-A13-	1000	MEK1/2	0.774446	0
**427**	4-O3B-B10-	100	MEK1/2	0.806567	0
**428**	4-O3B-B13-	100	MEK1/2	0.807469	0
**429**	4-O3B-C10-	10	MEK1/2	0.834562	0
**430**	4-O3B-C13-	10	MEK1/2	0.513447	0
**431**	4-O3B-D10	1	MEK1/2	0.647409	0
**432**	4-O3B-D13	1	MEK1/2	0.819034	0
**433**	4-O3B-E10-	0.1	MEK1/2	0.706793	0
**434**	4-O3B-E13-	0.1	MEK1/2	0.378957	0.394
**435**	4-O3B-L19-	0.25	MEK1/2	0.804805	0
**436**	4-O3B-M19	2.5	MEK1/2	0.393291	0.105
**437**	4-O3B-N19	25	MEK1/2	0.748373	0
**438**	4-O3B-O19	250	MEK1/2	0.847008	0
**439**	4-O3B-P19-	2500	MEK1/2	0.768213	0
**440**	1-O3B-L2-C	1	PARP	0.577477	0.001
**441**	1-O3B-L6-R	1	PARP	0.663673	0
**442**	1-O3B-M2-	10	PARP	0.610625	0
**443**	1-O3B-M6-	10	PARP	0.694469	0
**444**	1-O3B-N2-(	100	PARP	0.683128	0
**445**	1-O3B-N6-F	100	PARP	0.656979	0
**446**	1-O3B-O2-(	1000	PARP	0.735003	0
**447**	1-O3B-O6-F	1000	PARP	0.724105	0
**448**	1-O3B-P2-C	10000	PARP	0.718733	0
**449**	1-O3B-P6-R	10000	PARP	0.727231	0
**450**	7-O3B-A3-T	1000	PARP	0.729421	0
**451**	7-O3B-B2-V	10000	PARP	0.757705	0
**452**	7-O3B-B3-T	100	PARP	0.739799	0
**453**	7-O3B-C2-V	1000	PARP	0.710421	0
**454**	7-O3B-C3-T	10	PARP	0.740548	0
**455**	7-O3B-D2-V	100	PARP	0.324529	0.199
**456**	7-O3B-D3-T	1	PARP	0.780616	0
**457**	7-O3B-E2-V	10	PARP	0.666458	0
**458**	7-O3B-E3-T	0.1	PARP	0.647248	0
**459**	7-O3B-F2-V	1	PARP	0.670556	0
**460**	7-O3B-G2-N	10000	PARP	0.766366	0

**461**	7-O3B-H2-N	1000	PARP	0.784192	0
**462**	7-O3B-I2-N	100	PARP	0.81064	0
**463**	7-O3B-J2-N	10	PARP	0.62991	0
**464**	7-O3B-K2-N	1	PARP	0.671028	0
**465**	3-O3B-A19-	1000	CDK	0.521994	0
**466**	3-O3B-B19-	100	CDK	0.520805	0.002
**467**	3-O3B-B23-	2500	CDK	0.351914	0.079
**468**	3-O3B-C19-	10	CDK	0.261306	0.335
**469**	3-O3B-C23-	250	CDK	0.320801	0.332
**470**	3-O3B-D19	1	CDK	0.180873	0.244
**471**	3-O3B-D23	25	CDK	0.224034	0.157
**472**	3-O3B-E19-	0.1	CDK	0.260788	0.151
**473**	3-O3B-E23-	2.5	CDK	0.295361	0.068
**474**	3-O3B-F23-	0.25	CDK	0.135104	0.622
**475**	3-O3B-K17-	1	CDK	0.44241	0.001
**476**	3-O3B-L19-	1	CDK	0.278099	0.021
**477**	3-O3B-M17	10	CDK	0.185061	0.295
**478**	3-O3B-M19	10	CDK	0.309851	0.062
**479**	3-O3B-N17	100	CDK	0.342132	0.008
**480**	3-O3B-N19	100	CDK	0.321903	0.011
**481**	3-O3B-O17	1000	CDK	0.190129	0.808
**482**	3-O3B-O19	1000	CDK	0.257405	0.156
**483**	3-O3B-P17-	10000	CDK	0.294178	0.555
**484**	3-O3B-P19-	10000	CDK	0.166379	0.854
**485**	4-O3B-A4-S	10000	CDK	0.570676	0.001
**486**	4-O3B-A8-N	10000	CDK	0.56565	0
**487**	4-O3B-B4-S	1000	CDK	0.571846	0
**488**	4-O3B-B8-N	1000	CDK	0.358163	0.27
**489**	4-O3B-C4-S	100	CDK	0.189068	0.939
**490**	4-O3B-C8-N	100	CDK	0.332165	0.14
**491**	4-O3B-D4-S	10	CDK	0.19965	0.649
**492**	4-O3B-D8-N	10	CDK	0.294717	0.354
**493**	4-O3B-E4-S	1	CDK	0.202829	0.865
**494**	4-O3B-E8-N	1	CDK	0.22447	0.663
**495**	4-O3B-F4-S	10000	CDK	0.268599	0.671

**496**	4-O3B-F22-	10000	CDK	0.563324	0
**497**	4-O3B-G4-S	1000	CDK	0.229671	0.688
**498**	4-O3B-G22	1000	CDK	0.553973	0.002
**499**	4-O3B-H4-S	100	CDK	0.205288	0.796
**500**	4-O3B-H22	100	CDK	0.229528	0.196
**501**	4-O3B-I4-Se	10	CDK	0.283517	0.436
**502**	4-O3B-I22-,	10	CDK	0.185795	0.458
**503**	4-O3B-J4-S	1	CDK	0.249384	0.611
**504**	4-O3B-J22-	1	CDK	0.146812	0.546
**505**	5-O3B-A19-	10000	CDK	0.546658	0.004
**506**	5-O3B-B19-	1000	CDK	0.214452	0.868
**507**	5-O3B-C19-	100	CDK	0.257276	0.516
**508**	5-O3B-D19	10	CDK	0.174037	0.761
**509**	5-O3B-E19-	1	CDK	0.301627	0.216
**510**	5-O3B-K17-	1	CDK	0.225876	0.547
**511**	5-O3B-M17	10	CDK	0.245056	0.581
**512**	5-O3B-N17	100	CDK	0.274669	0.412
**513**	5-O3B-O17	1000	CDK	0.568302	0
**514**	5-O3B-P17-	10000	CDK	0.56027	0.002
**515**	6-O3B-A17-	1000	CDK	0.201731	0.815
**516**	6-O3B-B17-	100	CDK	0.261716	0.569
**517**	6-O3B-C17-	10	CDK	0.325349	0.286
**518**	6-O3B-D17	1	CDK	0.098743	0.761
**519**	6-O3B-E17-	0.1	CDK	0.260631	0.507
**520**	6-O3B-L15-	1	CDK	0.289801	0.286
**521**	6-O3B-M15	10	CDK	0.328731	0.08
**522**	6-O3B-N15	100	CDK	0.387484	0.117
**523**	6-O3B-O15	1000	CDK	0.591911	0
**524**	6-O3B-P15-	10000	CDK	0.598625	0
**525**	7-O3B-A21-	10000	BET	0.395353	0.056
**526**	7-O3B-A22-	30000	BET	0.668711	0
**527**	7-O3B-B21-	1000	BET	0.244877	0.559
**528**	7-O3B-B22-	3000	BET	0.675193	0
**529**	7-O3B-C21-	100	BET	0.206853	0.823
**530**	7-O3B-C22-	300	BET	0.365517	0.06

**531**	7-O3B-D21	10	BET	0.217542	0.76
**532**	7-O3B-D22	30	BET	0.571619	0
**533**	7-O3B-E21-	1	BET	0.225302	0.749
**534**	7-O3B-E22-	3	BET	0.439988	0.054
**535**	7-O3B-G10	10000	BET	0.653916	0
**536**	7-O3B-G15	10000	BET	0.676961	0
**537**	7-O3B-H10	1000	BET	0.658112	0
**538**	7-O3B-H15	1000	BET	0.680927	0
**539**	7-O3B-I10-I	100	BET	0.707147	0
**540**	7-O3B-I15-I	100	BET	0.26462	0.574
**541**	7-O3B-J10-	10	BET	0.245363	0.182
**542**	7-O3B-J15-	10	BET	0.33474	0.333
**543**	7-O3B-K10-	1	BET	0.151571	0.493
**544**	7-O3B-K13-	1	BET	0.295881	0.033
**545**	7-O3B-K15-	1	BET	0.202132	0.641
**546**	7-O3B-L12-	1	BET	0.165422	0.897
**547**	7-O3B-L13-	10	BET	0.694203	0
**548**	7-O3B-L20-	1	BET	0.182985	0.702
**549**	7-O3B-L23-	0.03	BET	0.220774	0.814
**550**	7-O3B-M12	10	BET	0.241972	0.272
**551**	7-O3B-M13	100	BET	0.664273	0
**552**	7-O3B-M2C	10	BET	0.182724	0.381
**553**	7-O3B-M23	0.3	BET	0.62913	0
**554**	7-O3B-N12	100	BET	0.284585	0.023
**555**	7-O3B-N13	1000	BET	0.656434	0
**556**	7-O3B-N20	100	BET	0.727087	0
**557**	7-O3B-N23	3	BET	0.24682	0.153
**558**	7-O3B-O12	1000	BET	0.658442	0
**559**	7-O3B-O20	1000	BET	0.680066	0
**560**	7-O3B-O23	30	BET	0.588181	0
**561**	7-O3B-P12-	10000	BET	0.67936	0
**562**	7-O3B-P13-	10000	BET	0.662292	0
**563**	7-O3B-P20-	10000	BET	0.686673	0
**564**	7-O3B-P23-	300	BET	0.669696	0
**565**	8-O3B-K22-	1	BET	0.349391	0.415

**566**	8-O3B-L22-	10	BET	0.178417	0.442
**567**	8-O3B-M22	100	BET	0.269558	0.416
**568**	8-O3B-N22	1000	BET	0.444087	0.005
**569**	8-O3B-O22	10000	BET	0.326981	0.187
**570**	1-O3B-A3-\	10000	HDAC	0.418807	0.018
**571**	1-O3B-B3-\	1000	HDAC	0.402436	0.006
**572**	1-O3B-C3-\	100	HDAC	0.251737	0.403
**573**	1-O3B-D3-\	10	HDAC	0.286765	0.432
**574**	1-O3B-E3-V	1	HDAC	0.191987	0.864
**575**	1-O3B-L12-	0.1	HDAC	0.302575	0.011
**576**	1-O3B-M12	1	HDAC	0.44401	0
**577**	1-O3B-N12	10	HDAC	0.424821	0.022
**578**	1-O3B-O12	100	HDAC	0.41538	0.163
**579**	1-O3B-P12-	1000	HDAC	0.380729	0.105
**580**	3-O3B-A4-F	1000	HDAC	0.414796	0.176
**581**	3-O3B-B4-F	100	HDAC	0.329434	0.091
**582**	3-O3B-C4-F	10	HDAC	0.430728	0
**583**	3-O3B-D4-F	1	HDAC	0.186838	0.294
**584**	3-O3B-E4-P	0.1	HDAC	0.164511	0.731
**585**	3-O3B-F7-C	1000	HDAC	0.464101	0.04
**586**	3-O3B-G7-(	100	HDAC	0.290115	0.171
**587**	3-O3B-G12	1000000	HDAC	0.114994	0.514
**588**	3-O3B-H7-(	10	HDAC	0.293866	0.204
**589**	3-O3B-H12	100000	HDAC	0.253262	0.315
**590**	3-O3B-I7-Q	1	HDAC	0.275082	0.279
**591**	3-O3B-I12-'	10000	HDAC	0.19588	0.743
**592**	3-O3B-J7-Q	0.1	HDAC	0.240978	0.436
**593**	3-O3B-J12-	1000	HDAC	0.203228	0.817
**594**	3-O3B-K3-E	1	HDAC	0.182102	0.356
**595**	3-O3B-K12-	100	HDAC	0.290612	0.017
**596**	3-O3B-L3-B	10	HDAC	0.166397	0.348
**597**	3-O3B-M3-I	100	HDAC	0.415994	0.007
**598**	3-O3B-N3-E	1000	HDAC	0.407383	0.021
**599**	3-O3B-O3-E	10000	HDAC	0.444382	0.086
**600**	7-O3B-A5-N	10000	HDAC	0.350776	0.071

**601**	7-O3B-A7-C	10000	HDAC	0.415414	0.138
**602**	7-O3B-A9-G	1000	HDAC	0.339906	0.1
**603**	7-O3B-A12-	10000	HDAC	0.352006	0.049
**604**	7-O3B-B5-N	1000	HDAC	0.305173	0.12
**605**	7-O3B-B7-C	1000	HDAC	0.499315	0.004
**606**	7-O3B-B12-	1000	HDAC	0.413747	0.018
**607**	7-O3B-C5-N	100	HDAC	0.429232	0
**608**	7-O3B-C7-C	100	HDAC	0.460734	0
**609**	7-O3B-C9-G	100	HDAC	0.427003	0
**610**	7-O3B-D7-C	10	HDAC	0.243846	0.581
**611**	7-O3B-D9-C	10	HDAC	0.35715	0.012
**612**	7-O3B-D12	100	HDAC	0.175215	0.055
**613**	7-O3B-E5-N	10	HDAC	0.295403	0.028
**614**	7-O3B-E7-C	1	HDAC	0.33855	0.041
**615**	7-O3B-E9-G	1	HDAC	0.286159	0.097
**616**	7-O3B-E12-	10	HDAC	0.443497	0
**617**	7-O3B-F5-N	1	HDAC	0.331055	0.003
**618**	7-O3B-F7-R	10000	HDAC	0.408026	0.007
**619**	7-O3B-F9-G	0.1	HDAC	0.38021	0
**620**	7-O3B-F12-	1	HDAC	0.318727	0
**621**	7-O3B-F19-	10000	HDAC	0.415838	0.001
**622**	7-O3B-G7-F	1000	HDAC	0.456626	0
**623**	7-O3B-G19	1000	HDAC	0.393519	0.002
**624**	7-O3B-H7-F	100	HDAC	0.514685	0
**625**	7-O3B-I7-R	10	HDAC	0.384829	0.001
**626**	7-O3B-I19-I	100	HDAC	0.317638	0.135
**627**	7-O3B-J7-R	1	HDAC	0.463726	0
**628**	7-O3B-J19-	10	HDAC	0.398342	0
**629**	7-O3B-K4-E	1	HDAC	0.426206	0
**630**	7-O3B-K11-	1	HDAC	0.26674	0.054
**631**	7-O3B-K18-	1	HDAC	0.418585	0
**632**	7-O3B-K19-	1	HDAC	0.270755	0.271
**633**	7-O3B-L2-T	0.1	HDAC	0.070485	0.996
**634**	7-O3B-L4-E	10	HDAC	0.097023	0.713
**635**	7-O3B-L5-P	1	HDAC	0.252237	0.382

**636**	7-O3B-L8-A	1	HDAC	0.244586	0.334
**637**	7-O3B-L10-	1	HDAC	0.377813	0
**638**	7-O3B-L11-	10	HDAC	0.393691	0
**639**	7-O3B-L14-	1	HDAC	0.235871	0.016
**640**	7-O3B-L16-	1	HDAC	0.344884	0.022
**641**	7-O3B-L18-	10	HDAC	0.314861	0.049
**642**	7-O3B-M2--	1	HDAC	0.087375	0.698
**643**	7-O3B-M5-I	10	HDAC	0.25595	0.076
**644**	7-O3B-M8-,	10	HDAC	0.161765	0.203
**645**	7-O3B-M1C	10	HDAC	0.40572	0
**646**	7-O3B-M11	100	HDAC	0.429518	0
**647**	7-O3B-M14	10	HDAC	0.152262	0.655
**648**	7-O3B-M16	10	HDAC	0.391676	0.002
**649**	7-O3B-M18	100	HDAC	0.288693	0.05
**650**	7-O3B-N2-T	10	HDAC	0.092722	0.753
**651**	7-O3B-N4-E	100	HDAC	0.295871	0.002
**652**	7-O3B-N5-F	100	HDAC	0.565499	0
**653**	7-O3B-N8-A	100	HDAC	0.435062	0
**654**	7-O3B-N10	100	HDAC	0.510577	0
**655**	7-O3B-N14	100	HDAC	0.217323	0.28
**656**	7-O3B-N16	100	HDAC	0.425177	0
**657**	7-O3B-N18	1000	HDAC	0.479775	0
**658**	7-O3B-O2-T	100	HDAC	0.077151	0.859
**659**	7-O3B-O4-E	1000	HDAC	0.554522	0
**660**	7-O3B-O5-F	1000	HDAC	0.337515	0.043
**661**	7-O3B-O8-A	1000	HDAC	0.341316	0.046
**662**	7-O3B-O10	1000	HDAC	0.486077	0
**663**	7-O3B-O11	1000	HDAC	0.415345	0
**664**	7-O3B-O14	1000	HDAC	0.16581	0.095
**665**	7-O3B-O16	1000	HDAC	0.412003	0
**666**	7-O3B-P2-T	1000	HDAC	0.300972	0.011
**667**	7-O3B-P4-E	10000	HDAC	0.327109	0.074
**668**	7-O3B-P5-F	10000	HDAC	0.475299	0.018
**669**	7-O3B-P8-A	10000	HDAC	0.489036	0.003
**670**	7-O3B-P10-	10000	HDAC	0.296627	0.195

**671**	7-O3B-P11-	10000	HDAC	0.503625	0.007
**672**	7-O3B-P14-	10000	HDAC	0.097538	0.47
**673**	7-O3B-P16-	10000	HDAC	0.463937	0
**674**	7-O3B-P18-	10000	HDAC	0.436853	0.007
**0**	2-O8W-A16	10000	EGFR	0.340885	0.012
**1**	2-O8W-A19	10000	EGFR	0.273039	0.273
**2**	2-O8W-B19	1000	EGFR	0.133904	0.899
**3**	2-O8W-C16	1000	EGFR	0.146149	0.481
**4**	2-O8W-C19	100	EGFR	0.144214	0.692
**5**	2-O8W-D16	100	EGFR	0.181072	0.534
**6**	2-O8W-D19	10	EGFR	0.314122	0.081
**7**	2-O8W-E16	10	EGFR	0.315058	0.096
**8**	2-O8W-E19	1	EGFR	0.157758	0.868
**9**	2-O8W-F16	1	EGFR	0.207026	0.692
**10**	2-O8W-K11	0.1	EGFR	0.229943	0.701
**11**	2-O8W-L11	1	EGFR	0.342642	0.019
**12**	2-O8W-L16	0.25	EGFR	0.358218	0.008
**13**	2-O8W-L19	0.1	EGFR	0.189871	0.618
**14**	2-O8W-M1	10	EGFR	0.097524	0.914
**15**	2-O8W-M1	2.5	EGFR	0.364928	0.016
**16**	2-O8W-M1	1	EGFR	0.400586	0.003
**17**	2-O8W-N16	25	EGFR	0.491444	0
**18**	2-O8W-N19	10	EGFR	0.39534	0
**19**	2-O8W-O11	100	EGFR	0.262164	0.179
**20**	2-O8W-O16	250	EGFR	0.296437	0.155
**21**	2-O8W-O19	100	EGFR	0.300707	0.162
**22**	2-O8W-P11	1000	EGFR	0.262854	0.294
**23**	2-O8W-P16	2500	EGFR	0.255592	0.624
**24**	2-O8W-P19	1000	EGFR	0.319374	0.085
**25**	3-O8W-F21	10000	EGFR	0.22164	0.908
**26**	3-O8W-G20	1000	EGFR	0.258864	0.436
**27**	3-O8W-G21	1000	EGFR	0.244487	0.267
**28**	3-O8W-H20	100	EGFR	0.137238	0.853
**29**	3-O8W-H21	100	EGFR	0.129301	0.976
**30**	3-O8W-I20	10	EGFR	0.355927	0.003

**31**	3-O8W-I21	10	EGFR	0.167427	0.469
**32**	3-O8W-J20	1	EGFR	0.315013	0.04
**33**	3-O8W-J21	1	EGFR	0.382372	0.002
**34**	3-O8W-K4-	1	EGFR	0.279876	0.171
**35**	3-O8W-K18	0.1	EGFR	0.351227	0.003
**36**	3-O8W-K2(	0.1	EGFR	0.098223	0.83
**37**	3-O8W-L4-	10	EGFR	0.148474	0.85
**38**	3-O8W-L18	1	EGFR	0.132064	0.694
**39**	3-O8W-M1	10	EGFR	0.389278	0.001
**40**	3-O8W-N4-	100	EGFR	0.389331	0
**41**	3-O8W-N18	100	EGFR	0.384343	0
**42**	3-O8W-O4-	1000	EGFR	0.174506	0.814
**43**	3-O8W-P4-	10000	EGFR	0.142473	1
**44**	3-O8W-P18	1000	EGFR	0.272397	0.141
**45**	4-O8W-F13	1000	EGFR	0.368054	0.003
**46**	4-O8W-G1:	100	EGFR	0.314234	0.034
**47**	4-O8W-G1(	10000	EGFR	0.20936	0.789
**48**	4-O8W-H1:	10	EGFR	0.177918	0.772
**49**	4-O8W-H1(	1000	EGFR	0.145977	0.795
**50**	4-O8W-I13	1	EGFR	0.284496	0.018
**51**	4-O8W-I16	100	EGFR	0.145368	0.607
**52**	4-O8W-J13	0.1	EGFR	0.459962	0
**53**	4-O8W-J16	10	EGFR	0.350705	0.002
**54**	4-O8W-K7-	1	EGFR	0.250518	0.629
**55**	4-O8W-K1:	0.1	EGFR	0.235066	0.066
**56**	4-O8W-K1(	1	EGFR	0.293439	0.221
**57**	4-O8W-L7-	10	EGFR	0.187316	0.871
**58**	4-O8W-L13	1	EGFR	0.268138	0.285
**59**	4-O8W-M7	100	EGFR	0.329533	0.024
**60**	4-O8W-M1	10	EGFR	0.40483	0
**61**	4-O8W-N1:	100	EGFR	0.158906	0.257
**62**	4-O8W-O7-	1000	EGFR	0.131029	0.82
**63**	4-O8W-P7-	10000	EGFR	0.126831	0.886
**64**	4-O8W-P1:	1000	EGFR	0.282246	0.087
**65**	5-O8W-F4-	1000	EGFR	0.348837	0.03

**66**	5-O8W-F7-	1000	EGFR	0.257096	0.302
**67**	5-O8W-G4-	100	EGFR	0.202067	0.616
**68**	5-O8W-G7-	100	EGFR	0.249877	0.212
**69**	5-O8W-H4-	10	EGFR	0.154567	0.745
**70**	5-O8W-H7-	10	EGFR	0.322612	0.015
**71**	5-O8W-I4-	1	EGFR	0.185513	0.756
**72**	5-O8W-I7-	1	EGFR	0.180506	0.357
**73**	5-O8W-J4-	0.1	EGFR	0.135098	0.665
**74**	5-O8W-J7-	0.1	EGFR	0.309432	0.156
**75**	5-O8W-K7-	0.1	EGFR	0.249746	0.538
**76**	5-O8W-L7-	1	EGFR	0.307401	0.062
**77**	5-O8W-M7	10	EGFR	0.310525	0.18
**78**	5-O8W-O7-	100	EGFR	0.114153	0.827
**79**	5-O8W-P7-	1000	EGFR	0.237654	0.496
**80**	2-O8W-A1	2500	VEGFR	0.314306	0.135
**81**	2-O8W-A1	10000	VEGFR	0.296825	0.517
**82**	2-O8W-A2	10000	VEGFR	0.298283	0.506
**83**	2-O8W-B1	250	VEGFR	0.413996	0
**84**	2-O8W-B1	1000	VEGFR	0.33423	0.015
**85**	2-O8W-B2	1000	VEGFR	0.17738	0.721
**86**	2-O8W-C1	25	VEGFR	0.276238	0.127
**87**	2-O8W-C1	100	VEGFR	0.323931	0.007
**88**	2-O8W-D1	2.5	VEGFR	0.405465	0
**89**	2-O8W-D1	10	VEGFR	0.244109	0.447
**90**	2-O8W-D2	100	VEGFR	0.404519	0
**91**	2-O8W-E17	1	VEGFR	0.344992	0.002
**92**	2-O8W-E2	10	VEGFR	0.253449	0.301
**93**	2-O8W-F13	10000	VEGFR	0.30272	0.636
**94**	2-O8W-F15	0.25	VEGFR	0.390786	0.001
**95**	2-O8W-F1	10000	VEGFR	0.190115	0.947
**96**	2-O8W-F2	1	VEGFR	0.318967	0.008
**97**	2-O8W-F21	10000	VEGFR	0.197816	0.761
**98**	2-O8W-G1	10000	VEGFR	0.357016	0.003
**99**	2-O8W-G1	1000	VEGFR	0.329603	0.044
**100**	2-O8W-G1	1000	VEGFR	0.078142	0.932

**101**	2-O8W-G2:	1000	VEGFR	0.380548	0.001
**102**	2-O8W-H1(	1000	VEGFR	0.244109	0.362
**103**	2-O8W-H1:	100	VEGFR	0.332893	0.07
**104**	2-O8W-H2:	100	VEGFR	0.137763	0.844
**105**	2-O8W-I10	100	VEGFR	0.341186	0.092
**106**	2-O8W-I13	10	VEGFR	0.385921	0.011
**107**	2-O8W-I19	100	VEGFR	0.398327	0
**108**	2-O8W-I21	10	VEGFR	0.231424	0.84
**109**	2-O8W-J10	10	VEGFR	0.437758	0
**110**	2-O8W-J13	1	VEGFR	0.374539	0.001
**111**	2-O8W-J19	10	VEGFR	0.366979	0
**112**	2-O8W-J21	1	VEGFR	0.330645	0.024
**113**	2-O8W-K1(	1	VEGFR	0.348223	0.095
**114**	2-O8W-K1:	0.1	VEGFR	0.338147	0.038
**115**	2-O8W-K1:	1	VEGFR	0.33816	0.011
**116**	2-O8W-K1!	1	VEGFR	0.303385	0.081
**117**	2-O8W-L12	0.1	VEGFR	0.29241	0.074
**118**	2-O8W-L13	1	VEGFR	0.358899	0.008
**119**	2-O8W-L21	0.1	VEGFR	0.239451	0.487
**120**	2-O8W-M1	1	VEGFR	0.308833	0.016
**121**	2-O8W-M1	10	VEGFR	0.442777	0
**122**	2-O8W-M1	10	VEGFR	0.340042	0.006
**123**	2-O8W-M2	1	VEGFR	0.22336	0.243
**124**	2-O8W-N1:	10	VEGFR	0.339525	0.022
**125**	2-O8W-N1:	100	VEGFR	0.301667	0.136
**126**	2-O8W-N1:	100	VEGFR	0.363974	0.002
**127**	2-O8W-N2:	10	VEGFR	0.139128	0.805
**128**	2-O8W-O1:	100	VEGFR	0.243204	0.476
**129**	2-O8W-O1:	1000	VEGFR	0.252767	0.42
**130**	2-O8W-O2:	100	VEGFR	0.290879	0.092
**131**	2-O8W-P1:	1000	VEGFR	0.204564	0.6
**132**	2-O8W-P1:	1000	VEGFR	0.273943	0.279
**133**	2-O8W-P1:	10000	VEGFR	0.185602	0.892
**134**	2-O8W-P21	1000	VEGFR	0.284452	0.154
**135**	3-O8W-A3-	1000	VEGFR	0.188749	0.624

**136**	3-O8W-A6-	1000	VEGFR	0.303802	0.48
**137**	3-O8W-A1ẟ	1000	VEGFR	0.232023	0.261
**138**	3-O8W-B3-	100	VEGFR	0.164562	0.548
**139**	3-O8W-B6-	100	VEGFR	0.178078	0.638
**140**	3-O8W-B1ẟ	100	VEGFR	0.162524	0.664
**141**	3-O8W-C3-	10	VEGFR	0.165448	0.776
**142**	3-O8W-C6-	10	VEGFR	0.143003	0.519
**143**	3-O8W-C1ẟ	10	VEGFR	0.238423	0.198
**144**	3-O8W-D3-	1	VEGFR	0.156624	0.805
**145**	3-O8W-D6-	1	VEGFR	0.251874	0.273
**146**	3-O8W-D1ẟ	1	VEGFR	0.16003	0.755
**147**	3-O8W-E3-	0.1	VEGFR	0.250375	0.25
**148**	3-O8W-E6-	0.1	VEGFR	0.193295	0.716
**149**	3-O8W-E18	0.1	VEGFR	0.328639	0.005
**150**	3-O8W-F18	1000	VEGFR	0.292048	0.108
**151**	3-O8W-G1ẟ	100	VEGFR	0.292849	0.036
**152**	3-O8W-H1ẟ	10	VEGFR	0.200432	0.189
**153**	3-O8W-I18	1	VEGFR	0.201415	0.632
**154**	3-O8W-J18	0.1	VEGFR	0.194093	0.13
**155**	4-O8W-A1.	10000	VEGFR	0.213922	0.982
**156**	4-O8W-A1.	2500	VEGFR	0.100493	0.854
**157**	4-O8W-A2(	10000	VEGFR	0.345396	0.004
**158**	4-O8W-B1.	1000	VEGFR	0.291306	0.225
**159**	4-O8W-B1.	250	VEGFR	0.12855	0.854
**160**	4-O8W-B2(	1000	VEGFR	0.387916	0.004
**161**	4-O8W-C1.	25	VEGFR	0.422372	0
**162**	4-O8W-D1.	100	VEGFR	0.343782	0.008
**163**	4-O8W-D1.	2.5	VEGFR	0.400263	0
**164**	4-O8W-D2(	100	VEGFR	0.09876	0.866
**165**	4-O8W-E12	10	VEGFR	0.272691	0.069
**166**	4-O8W-E2C	10	VEGFR	0.303842	0.069
**167**	4-O8W-F12	1	VEGFR	0.352649	0.003
**168**	4-O8W-F15	0.25	VEGFR	0.217278	0.234
**169**	4-O8W-F2C	1	VEGFR	0.297305	0.049
**170**	4-O8W-L16	1	VEGFR	0.096332	0.842

**171**	4-O8W-M1	10	VEGFR	0.159459	0.761
**172**	4-O8W-N1(	100	VEGFR	0.282479	0.169
**173**	4-O8W-O1(	1000	VEGFR	0.267601	0.068
**174**	4-O8W-P1(	10000	VEGFR	0.230954	0.639
**175**	2-O8W-L1C	1	PI3K	0.312583	0.018
**176**	2-O8W-M1	10	PI3K	0.338369	0.001
**177**	2-O8W-N1(	100	PI3K	0.218256	0.484
**178**	2-O8W-O1(	1000	PI3K	0.434589	0
**179**	2-O8W-P1(	10000	PI3K	0.345241	0
**180**	3-O8W-A1(	2500	PI3K	0.055754	0.998
**181**	3-O8W-C1(	250	PI3K	0.100443	0.832
**182**	3-O8W-D1(	25	PI3K	0.145136	0.798
**183**	3-O8W-E1(	2.5	PI3K	0.28	0.059
**184**	3-O8W-F1(	0.25	PI3K	0.327602	0.003
**185**	3-O8W-F17	100000	PI3K	0.219616	0.995
**186**	3-O8W-F1!	500	PI3K	0.265497	0.006
**187**	3-O8W-G1	10000	PI3K	0.131755	1
**188**	3-O8W-G1!	50	PI3K	0.217498	0.273
**189**	3-O8W-H1	1000	PI3K	0.424974	0
**190**	3-O8W-I17	100	PI3K	0.124762	0.549
**191**	3-O8W-I19	5	PI3K	0.100965	0.812
**192**	3-O8W-J17	10	PI3K	0.11908	0.72
**193**	3-O8W-J19	0.5	PI3K	0.197733	0.247
**194**	3-O8W-K1!	0.05	PI3K	0.241331	0.266
**195**	3-O8W-L8-	1	PI3K	0.202426	0.533
**196**	3-O8W-L21	0.1	PI3K	0.20867	0.091
**197**	3-O8W-M8	10	PI3K	0.257082	0.093
**198**	3-O8W-M2	1	PI3K	0.265311	0.153
**199**	3-O8W-N8-	100	PI3K	0.22472	0.169
**200**	3-O8W-N2	10	PI3K	0.159889	0.598
**201**	3-O8W-O8-	1000	PI3K	0.226448	0.357
**202**	3-O8W-O2	100	PI3K	0.181506	0.509
**203**	3-O8W-P8-	10000	PI3K	0.220595	0.749
**204**	3-O8W-P2	1000	PI3K	0.245775	0.25
**205**	4-O8W-A1!	2500	PI3K	0.183257	0.388

**206**	4-O8W-B1‹	250	PI3K	0.109011	0.806
**207**	4-O8W-C1‹	25	PI3K	0.14293	0.53
**208**	4-O8W-D1‹	2.5	PI3K	0.208368	0.288
**209**	4-O8W-E1‹	0.25	PI3K	0.215527	0.615
**210**	4-O8W-F14	1000	PI3K	0.189874	1
**211**	4-O8W-G2-	2500	PI3K	0.288443	0.091
**212**	4-O8W-G5-	10000	PI3K	0.33155	0.021
**213**	4-O8W-G1‹	100	PI3K	0.240941	0.712
**214**	4-O8W-G2(	10000	PI3K	0.26014	0.76
**215**	4-O8W-H2-	250	PI3K	0.151608	0.53
**216**	4-O8W-H5-	1000	PI3K	0.231113	0.353
**217**	4-O8W-H1‹	10	PI3K	0.249083	0.268
**218**	4-O8W-H2(	1000	PI3K	0.22584	0.052
**219**	4-O8W-I2-1	25	PI3K	0.15172	0.923
**220**	4-O8W-I5-‹	100	PI3K	0.323117	0.014
**221**	4-O8W-I14	1	PI3K	0.34646	0.005
**222**	4-O8W-I20	100	PI3K	0.338214	0.014
**223**	4-O8W-J2-1	2.5	PI3K	0.224277	0.469
**224**	4-O8W-J5-‹	10	PI3K	0.230774	0.412
**225**	4-O8W-J20	10	PI3K	0.043034	0.999
**226**	4-O8W-K2-	0.25	PI3K	0.249229	0.187
**227**	4-O8W-K4-	0.1	PI3K	0.289192	0.067
**228**	4-O8W-K5-	1	PI3K	0.23271	0.56
**229**	4-O8W-K1‹	0.1	PI3K	0.367489	0
**230**	4-O8W-K2(	1	PI3K	0.217366	0.529
**231**	4-O8W-L4-I	1	PI3K	0.250194	0.021
**232**	4-O8W-L14	0.1	PI3K	0.147143	0.374
**233**	4-O8W-L1‹	1	PI3K	0.296049	0.014
**234**	4-O8W-L21	0.1	PI3K	0.238165	0.209
**235**	4-O8W-M1	1	PI3K	0.242128	0.128
**236**	4-O8W-M1	10	PI3K	0.249305	0.175
**237**	4-O8W-M2	1	PI3K	0.219891	0.271
**238**	4-O8W-N4-	10	PI3K	0.244359	0.275
**239**	4-O8W-N1‹	10	PI3K	0.178547	0.614
**240**	4-O8W-N1‹	100	PI3K	0.165887	0.198

**241**	4-O8W-N2:	10	PI3K	0.184468	0.25
**242**	4-O8W-O4-	100	PI3K	0.257402	0.425
**243**	4-O8W-O14	100	PI3K	0.29412	0.034
**244**	4-O8W-O15	1000	PI3K	0.349255	0.004
**245**	4-O8W-O2:	100	PI3K	0.295406	0.035
**246**	4-O8W-P4-	1000	PI3K	0.217263	0.854
**247**	4-O8W-P14	1000	PI3K	0.228098	0.838
**248**	4-O8W-P15	10000	PI3K	0.372182	0.001
**249**	4-O8W-P21	1000	PI3K	0.317208	0.026
**250**	5-O8W-A6-	2500	PI3K	0.224921	0.816
**251**	5-O8W-A7-	1000	PI3K	0.434382	0.001
**252**	5-O8W-A16	2500	PI3K	0.358296	0.001
**253**	5-O8W-A17	10000	PI3K	0.306072	0.057
**254**	5-O8W-B6-	250	PI3K	0.225036	0.631
**255**	5-O8W-B7-	100	PI3K	0.250007	0.171
**256**	5-O8W-B17	1000	PI3K	0.25927	0.23
**257**	5-O8W-C6-	25	PI3K	0.148759	0.676
**258**	5-O8W-C7-	10	PI3K	0.263593	0.121
**259**	5-O8W-C16	250	PI3K	0.162629	0.429
**260**	5-O8W-C17	100	PI3K	0.337815	0
**261**	5-O8W-D6-	2.5	PI3K	0.244033	0.085
**262**	5-O8W-D7-	1	PI3K	0.213277	0.48
**263**	5-O8W-D16	25	PI3K	0.290546	0.001
**264**	5-O8W-D17	10	PI3K	0.280992	0.225
**265**	5-O8W-E6-I	0.25	PI3K	0.281538	0.118
**266**	5-O8W-E7-,	0.1	PI3K	0.191223	0.504
**267**	5-O8W-E16	2.5	PI3K	0.168255	0.413
**268**	5-O8W-E17	1	PI3K	0.276116	0.001
**269**	5-O8W-F11	10000	PI3K	0.226434	0.103
**270**	5-O8W-F16	0.25	PI3K	0.254096	0.236
**271**	5-O8W-G9-	10000	PI3K	0.446332	0
**272**	5-O8W-G1:	1000	PI3K	0.269681	0.027
**273**	5-O8W-H9-	1000	PI3K	0.131727	0.297
**274**	5-O8W-H1:	100	PI3K	0.259737	0.002
**275**	5-O8W-I9-S	100	PI3K	0.314571	0.011

**276**	5-O8W-I11	10	PI3K	0.168525	0.033
**277**	5-O8W-J9-S	10	PI3K	0.246423	0.349
**278**	5-O8W-J11	1	PI3K	0.223936	0.111
**279**	5-O8W-K9-	1	PI3K	0.354749	0.002
**280**	5-O8W-L14	0.1	PI3K	0.285983	0
**281**	5-O8W-L2C	1	PI3K	0.37967	0.001
**282**	5-O8W-L23	0.1	PI3K	0.157863	0.542
**283**	5-O8W-M1	1	PI3K	0.252075	0.127
**284**	5-O8W-M2	10	PI3K	0.455997	0
**285**	5-O8W-M2	1	PI3K	0.174755	0.353
**286**	5-O8W-N14	10	PI3K	0.373058	0
**287**	5-O8W-N2(	100	PI3K	0.119896	0.756
**288**	5-O8W-N23	10	PI3K	0.33049	0.024
**289**	5-O8W-O14	100	PI3K	0.326224	0.012
**290**	5-O8W-O2(	1000	PI3K	0.348125	0.006
**291**	5-O8W-O23	100	PI3K	0.274566	0.143
**292**	5-O8W-P14	1000	PI3K	0.185232	0.109
**293**	5-O8W-P2(	10000	PI3K	0.381034	0
**294**	5-O8W-P23	1000	PI3K	0.237224	0.749
**295**	6-O8W-A8-	10000	PI3K	0.298684	0.089
**296**	6-O8W-B8-	1000	PI3K	0.116087	0.97
**297**	6-O8W-C8-	100	PI3K	0.228792	0.102
**298**	6-O8W-D8-	10	PI3K	0.067613	0.998
**299**	6-O8W-E8-T	1	PI3K	0.111237	0.985
**300**	6-O8W-L6-(	1	PI3K	0.189495	0.269
**301**	6-O8W-M6	10	PI3K	0.230865	0.264
**302**	6-O8W-N6-	100	PI3K	0.137091	0.568
**303**	6-O8W-O6-	1000	PI3K	0.269187	0.128
**304**	6-O8W-P6-	10000	PI3K	0.204648	0.984
**305**	1-O8W-F11	10000	Topoisome	0.578584	0
**306**	1-O8W-G11	1000	Topoisome	0.594686	0
**307**	1-O8W-G2(	1000	Topoisome	0.564753	0
**308**	1-O8W-H11	100	Topoisome	0.567911	0
**309**	1-O8W-H2(	100	Topoisome	0.555623	0
**310**	1-O8W-I11	10	Topoisome	0.58042	0

**311**	1-O8W-I20	10	Topoisome	0.481888	0
**312**	1-O8W-J11	1	Topoisome	0.052207	0.99
**313**	1-O8W-J20	1	Topoisome	0.4144	0.037
**314**	1-O8W-K11	1	Topoisome	0.510386	0
**315**	1-O8W-K2(	0.1	Topoisome	0.112164	0.927
**316**	1-O8W-L11	10	Topoisome	0.586812	0
**317**	1-O8W-L14	1	Topoisome	0.230252	0.493
**318**	1-O8W-M1	100	Topoisome	0.586519	0
**319**	1-O8W-M1	10	Topoisome	0.496791	0
**320**	1-O8W-N1	100	Topoisome	0.577565	0
**321**	1-O8W-O1	1000	Topoisome	0.268853	0.811
**322**	1-O8W-O1	1000	Topoisome	0.580998	0
**323**	1-O8W-P11	10000	Topoisome	0.365739	0.566
**324**	1-O8W-P1	10000	Topoisome	0.565976	0
**325**	3-O8W-A11	10000	Topoisome	0.603575	0
**326**	3-O8W-B11	1000	Topoisome	0.602853	0
**327**	3-O8W-C11	100	Topoisome	0.553755	0
**328**	3-O8W-D1	10	Topoisome	0.129366	0.912
**329**	3-O8W-E11	1	Topoisome	0.117934	0.876
**330**	3-O8W-G9-	1000	Topoisome	0.345714	0.452
**331**	3-O8W-G1(	10000	Topoisome	0.497586	0
**332**	3-O8W-H9-	100	Topoisome	0.598601	0
**333**	3-O8W-H1(	1000	Topoisome	0.508009	0
**334**	3-O8W-I9-[	10	Topoisome	0.537597	0
**335**	3-O8W-I10	100	Topoisome	0.158049	0.679
**336**	3-O8W-J9-I	1	Topoisome	0.621809	0
**337**	3-O8W-J10	10	Topoisome	0.376969	0.035
**338**	3-O8W-K7-	0.1	Topoisome	0.198775	0.639
**339**	3-O8W-K9-	0.1	Topoisome	0.07047	0.974
**340**	3-O8W-K1(	1	Topoisome	0.444463	0.011
**341**	3-O8W-L6-I	0.1	Topoisome	0.406627	0.002
**342**	3-O8W-L7-I	1	Topoisome	0.339812	0.033
**343**	3-O8W-L9-'	0.5	Topoisome	0.587564	0
**344**	3-O8W-L1C	0.1	Topoisome	0.586289	0
**345**	3-O8W-L16	1	Topoisome	0.432201	0.014

346	3-O8W-M6	1	Topoisome	0.473694	0.001
347	3-O8W-M7	10	Topoisome	0.574857	0
348	3-O8W-M9	5	Topoisome	0.49268	0.001
349	3-O8W-M1	1	Topoisome	0.478126	0
350	3-O8W-M1	10	Topoisome	0.475259	0
351	3-O8W-N6-	10	Topoisome	0.485384	0
352	3-O8W-N9-	50	Topoisome	0.454651	0
353	3-O8W-N1(	10	Topoisome	0.528967	0
354	3-O8W-N1(	100	Topoisome	0.054511	0.994
355	3-O8W-O6-	100	Topoisome	0.636417	0
356	3-O8W-O7-	100	Topoisome	0.618967	0
357	3-O8W-O9-	500	Topoisome	0.613465	0
358	3-O8W-O1(	100	Topoisome	0.616144	0
359	3-O8W-O1(	1000	Topoisome	0.150537	0.877
360	3-O8W-P6-	1000	Topoisome	0.546858	0
361	3-O8W-P7-	1000	Topoisome	0.347107	0.571
362	3-O8W-P9-	5000	Topoisome	0.545599	0
363	3-O8W-P1(	1000	Topoisome	0.57548	0
364	3-O8W-P1(	10000	Topoisome	0.435555	0.002
365	1-O8W-A1(	10000	Mitotic	0.646186	0
366	1-O8W-A1:	1000	Mitotic	0.652395	0
367	1-O8W-A1(	1000	Mitotic	0.651798	0
368	1-O8W-B1(	1000	Mitotic	0.6617	0
369	1-O8W-B1:	100	Mitotic	0.4869	0.001
370	1-O8W-B1(	100	Mitotic	0.655198	0
371	1-O8W-C1(	100	Mitotic	0.633858	0
372	1-O8W-C1:	10	Mitotic	0.480839	0.002
373	1-O8W-C1(	10	Mitotic	0.644752	0
374	1-O8W-D1(	10	Mitotic	0.576358	0
375	1-O8W-D1:	1	Mitotic	0.259234	0.545
376	1-O8W-D1(	1	Mitotic	0.427692	0.008
377	1-O8W-E1C	1	Mitotic	0.566604	0
378	1-O8W-E13	0.1	Mitotic	0.092547	0.935
379	1-O8W-E1(	0.1	Mitotic	0.392916	0.006
380	1-O8W-F13	1000	Mitotic	0.643461	0

**381**	1-O8W-G1:	100	Mitotic	0.546151	0
**382**	1-O8W-G1!	1000	Mitotic	0.62206	0
**383**	1-O8W-H1:	10	Mitotic	0.400168	0.022
**384**	1-O8W-H1!	100	Mitotic	0.627974	0
**385**	1-O8W-I13	1	Mitotic	0.466241	0.004
**386**	1-O8W-I15	10	Mitotic	0.64549	0
**387**	1-O8W-J13	0.1	Mitotic	0.118807	0.831
**388**	1-O8W-J15	1	Mitotic	0.546558	0
**389**	1-O8W-K7-	0.1	Mitotic	0.493675	0
**390**	1-O8W-K1!	0.1	Mitotic	0.083903	1
**391**	1-O8W-L7-'	1	Mitotic	0.373224	0.162
**392**	1-O8W-L2(	0.1	Mitotic	0.075662	1
**393**	1-O8W-M7	10	Mitotic	0.18187	0.954
**394**	1-O8W-M2	1	Mitotic	0.397287	0.032
**395**	1-O8W-N2(	10	Mitotic	0.387894	0.052
**396**	1-O8W-O7-	100	Mitotic	0.489138	0.002
**397**	1-O8W-O2(	100	Mitotic	0.374556	0.087
**398**	1-O8W-P7-	1000	Mitotic	0.662876	0
**399**	1-O8W-P2(	1000	Mitotic	0.609857	0
**400**	3-O8W-A7-	1000	Mitotic	0.603067	0
**401**	3-O8W-B7-	100	Mitotic	0.609645	0
**402**	3-O8W-C7-	10	Mitotic	0.586792	0
**403**	3-O8W-D7-	1	Mitotic	0.471003	0
**404**	3-O8W-E7-I	0.1	Mitotic	0.36542	0.032
**405**	6-O8W-L1!	1	Mitotic	0.101457	0.919
**406**	6-O8W-M1	10	Mitotic	0.072137	1
**407**	6-O8W-N1!	100	Mitotic	0.061243	1
**408**	6-O8W-O1!	1000	Mitotic	0.582354	0
**409**	6-O8W-P1!	10000	Mitotic	0.628815	0
**410**	2-O8W-A1:	250	MEK1/2	0.544457	0.002
**411**	2-O8W-B1:	25	MEK1/2	0.603966	0
**412**	2-O8W-D1:	2.5	MEK1/2	0.705379	0
**413**	2-O8W-E12	0.25	MEK1/2	0.497731	0.002
**414**	2-O8W-F12	2.5000000	MEK1/2	0.14356	0.981
**415**	2-O8W-F14	1000	MEK1/2	0.507557	0.01

**416**	2-O8W-G14	100	MEK1/2	0.69624	0
**417**	2-O8W-H14	10	MEK1/2	0.755606	0
**418**	2-O8W-I14	1	MEK1/2	0.105534	1
**419**	2-O8W-K14	0.1	MEK1/2	0.59147	0
**420**	2-O8W-L20	1	MEK1/2	0.558957	0.001
**421**	2-O8W-M2	10	MEK1/2	0.544956	0
**422**	2-O8W-N20	100	MEK1/2	0.704459	0
**423**	2-O8W-O20	1000	MEK1/2	0.768435	0
**424**	2-O8W-P20	10000	MEK1/2	0.607867	0
**425**	4-O8W-A10	1000	MEK1/2	0.701402	0
**426**	4-O8W-A13	1000	MEK1/2	0.6657	0
**427**	4-O8W-B10	100	MEK1/2	0.72531	0
**428**	4-O8W-B13	100	MEK1/2	0.783547	0
**429**	4-O8W-C10	10	MEK1/2	0.649847	0
**430**	4-O8W-C13	10	MEK1/2	0.655897	0
**431**	4-O8W-D10	1	MEK1/2	0.288911	0.623
**432**	4-O8W-D13	1	MEK1/2	0.341975	0.105
**433**	4-O8W-E10	0.1	MEK1/2	0.288837	0.403
**434**	4-O8W-E13	0.1	MEK1/2	0.528727	0.003
**435**	4-O8W-L19	0.25	MEK1/2	0.450609	0.045
**436**	4-O8W-M1	2.5	MEK1/2	0.404402	0.195
**437**	4-O8W-N19	25	MEK1/2	0.219048	0.683
**438**	4-O8W-O19	250	MEK1/2	0.614977	0
**439**	4-O8W-P19	2500	MEK1/2	0.80903	0
**440**	1-O8W-L2-	1	PARP	0.645303	0
**441**	1-O8W-L6-	1	PARP	0.453588	0.229
**442**	1-O8W-M2	10	PARP	0.667158	0
**443**	1-O8W-M6	10	PARP	0.14142	0.981
**444**	1-O8W-N2-	100	PARP	0.622073	0.001
**445**	1-O8W-N6-	100	PARP	0.523253	0.017
**446**	1-O8W-O2-	1000	PARP	0.657114	0
**447**	1-O8W-O6-	1000	PARP	0.663418	0
**448**	1-O8W-P2-	10000	PARP	0.72823	0
**449**	1-O8W-P6-	10000	PARP	0.667913	0
**450**	7-O8W-A3-	1000	PARP	0.725857	0

**451**	7-O8W-B2-	10000	PARP	0.586666	0.001
**452**	7-O8W-B3-	100	PARP	0.647682	0
**453**	7-O8W-C2-	1000	PARP	0.694036	0
**454**	7-O8W-C3-	10	PARP	0.640243	0
**455**	7-O8W-D2-	100	PARP	0.631593	0
**456**	7-O8W-D3-	1	PARP	0.689699	0
**457**	7-O8W-E2-'	10	PARP	0.568156	0.008
**458**	7-O8W-E3-'	0.1	PARP	0.388977	0.386
**459**	7-O8W-F2-'	1	PARP	0.110729	0.992
**460**	7-O8W-G2-	10000	PARP	0.721866	0
**461**	7-O8W-H2-	1000	PARP	0.649833	0
**462**	7-O8W-I2-I	100	PARP	0.624269	0
**463**	7-O8W-J2-I	10	PARP	0.509556	0.017
**464**	7-O8W-K2-	1	PARP	0.237064	0.776
**465**	3-O8W-A1!	1000	CDK	0.548108	0.017
**466**	3-O8W-B1!	100	CDK	0.526265	0.024
**467**	3-O8W-B2:	2500	CDK	0.428772	0
**468**	3-O8W-C1!	10	CDK	0.401587	0.006
**469**	3-O8W-C2:	250	CDK	0.33325	0.048
**470**	3-O8W-D1!	1	CDK	0.3162	0.029
**471**	3-O8W-D2:	25	CDK	0.098031	0.959
**472**	3-O8W-E1!	0.1	CDK	0.428598	0.008
**473**	3-O8W-E23	2.5	CDK	0.147173	0.954
**474**	3-O8W-F23	0.25	CDK	0.088474	0.977
**475**	3-O8W-K17	1	CDK	0.463204	0
**476**	3-O8W-L1!	1	CDK	0.307991	0.013
**477**	3-O8W-M1	10	CDK	0.334027	0.218
**478**	3-O8W-M1	10	CDK	0.292713	0.274
**479**	3-O8W-N1:	100	CDK	0.454212	0.001
**480**	3-O8W-N1!	100	CDK	0.292459	0.044
**481**	3-O8W-O1:	1000	CDK	0.389206	0.003
**482**	3-O8W-O1!	1000	CDK	0.459692	0
**483**	3-O8W-P17	10000	CDK	0.498031	0
**484**	3-O8W-P1!	10000	CDK	0.426679	0.005
**485**	4-O8W-A4-	10000	CDK	0.578584	0.003

**486**	4-O8W-A8-	10000	CDK	0.469471	0.033
**487**	4-O8W-B4-	1000	CDK	0.529955	0.031
**488**	4-O8W-B8-	1000	CDK	0.102693	0.997
**489**	4-O8W-C4-	100	CDK	0.378517	0.014
**490**	4-O8W-C8-	100	CDK	0.148013	0.92
**491**	4-O8W-D4-	10	CDK	0.309982	0.111
**492**	4-O8W-D8-	10	CDK	0.11837	0.9
**493**	4-O8W-E4-!	1	CDK	0.47027	0
**494**	4-O8W-E8-I	1	CDK	0.305057	0.149
**495**	4-O8W-F4-!	10000	CDK	0.391103	0.107
**496**	4-O8W-F22	10000	CDK	0.560639	0.01
**497**	4-O8W-G4-	1000	CDK	0.476157	0.001
**498**	4-O8W-G2:	1000	CDK	0.498035	0.02
**499**	4-O8W-H4-	100	CDK	0.247461	0.696
**500**	4-O8W-H2:	100	CDK	0.423601	0.009
**501**	4-O8W-I4-S	10	CDK	0.334468	0.094
**502**	4-O8W-I22	10	CDK	0.511149	0
**503**	4-O8W-J4-!	1	CDK	0.168567	0.738
**504**	4-O8W-J22	1	CDK	0.40328	0.061
**505**	5-O8W-A1!	10000	CDK	0.396678	0.233
**506**	5-O8W-B1!	1000	CDK	0.360265	0.244
**507**	5-O8W-C1!	100	CDK	0.468979	0.001
**508**	5-O8W-D1!	10	CDK	0.439595	0
**509**	5-O8W-E1!	1	CDK	0.414122	0.005
**510**	5-O8W-K17	1	CDK	0.334718	0.022
**511**	5-O8W-M1	10	CDK	0.350641	0.019
**512**	5-O8W-N1:	100	CDK	0.438998	0.002
**513**	5-O8W-O1:	1000	CDK	0.336463	0.486
**514**	5-O8W-P17	10000	CDK	0.52607	0.002
**515**	6-O8W-A17	1000	CDK	0.446006	0.001
**516**	6-O8W-B17	100	CDK	0.372061	0.037
**517**	6-O8W-C17	10	CDK	0.372284	0.025
**518**	6-O8W-D1:	1	CDK	0.39394	0.009
**519**	6-O8W-E17	0.1	CDK	0.050151	0.977
**520**	6-O8W-L1!	1	CDK	0.276517	0.674

**521**	6-O8W-M1	10	CDK	0.312973	0.096
**522**	6-O8W-N1!	100	CDK	0.404117	0.002
**523**	6-O8W-O1!	1000	CDK	0.558035	0.01
**524**	6-O8W-P1!	10000	CDK	0.589294	0
**525**	7-O8W-A21	10000	BET	0.587683	0
**526**	7-O8W-A22	30000	BET	0.653522	0
**527**	7-O8W-B21	1000	BET	0.490605	0
**528**	7-O8W-B22	3000	BET	0.237116	0.098
**529**	7-O8W-C21	100	BET	0.51368	0
**530**	7-O8W-C22	300	BET	0.517256	0
**531**	7-O8W-D2:	10	BET	0.52464	0
**532**	7-O8W-D2:	30	BET	0.404768	0.07
**533**	7-O8W-E21	1	BET	0.057086	1
**534**	7-O8W-E22	3	BET	0.128027	0.857
**535**	7-O8W-G1(	10000	BET	0.560955	0
**536**	7-O8W-G1!	10000	BET	0.565501	0
**537**	7-O8W-H1(	1000	BET	0.601993	0
**538**	7-O8W-H1!	1000	BET	0.614374	0
**539**	7-O8W-I10	100	BET	0.47159	0.001
**540**	7-O8W-I15	100	BET	0.666208	0
**541**	7-O8W-J10	10	BET	0.200849	0.743
**542**	7-O8W-J15	10	BET	0.209041	0.643
**543**	7-O8W-K1(	1	BET	0.129811	0.976
**544**	7-O8W-K1:	1	BET	0.555295	0
**545**	7-O8W-K1!	1	BET	0.395616	0.017
**546**	7-O8W-L12	1	BET	0.532517	0
**547**	7-O8W-L13	10	BET	0.642635	0
**548**	7-O8W-L2C	1	BET	0.25887	0.223
**549**	7-O8W-L23	0.03	BET	0.196671	0.6
**550**	7-O8W-M1	10	BET	0.450448	0.017
**551**	7-O8W-M1	100	BET	0.571797	0
**552**	7-O8W-M2	10	BET	0.296494	0.591
**553**	7-O8W-M2	0.3	BET	0.29946	0.211
**554**	7-O8W-N1:	100	BET	0.445116	0.006
**555**	7-O8W-N1:	1000	BET	0.557798	0

**556**	7-O8W-N2(100	BET	0.610449	0
**557**	7-O8W-N2: 3	BET	0.485231	0
**558**	7-O8W-O1: 1000	BET	0.590208	0
**559**	7-O8W-O2(1000	BET	0.552852	0
**560**	7-O8W-O2: 30	BET	0.54474	0
**561**	7-O8W-P1: 10000	BET	0.579538	0
**562**	7-O8W-P1: 10000	BET	0.241477	0.967
**563**	7-O8W-P2(10000	BET	0.553656	0.001
**564**	7-O8W-P2: 300	BET	0.417332	0.455
**565**	8-O8W-K2: 1	BET	0.150941	0.734
**566**	8-O8W-L22 10	BET	0.169514	0.844
**567**	8-O8W-M2 100	BET	0.184051	0.698
**568**	8-O8W-N2: 1000	BET	0.486102	0.001
**569**	8-O8W-O2: 10000	BET	0.469148	0.007
**570**	1-O8W-A3- 10000	HDAC	0.488741	0.022
**571**	1-O8W-B3- 1000	HDAC	0.423093	0
**572**	1-O8W-C3- 100	HDAC	0.326046	0.016
**573**	1-O8W-D3- 10	HDAC	0.387959	0.001
**574**	1-O8W-E3-' 1	HDAC	0.322391	0.015
**575**	1-O8W-L12 0.1	HDAC	0.35849	0.14
**576**	1-O8W-M1 1	HDAC	0.327598	0.024
**577**	1-O8W-N1: 10	HDAC	0.411849	0.008
**578**	1-O8W-O1: 100	HDAC	0.430183	0.028
**579**	1-O8W-P1: 1000	HDAC	0.449891	0.024
**580**	3-O8W-A4- 1000	HDAC	0.437438	0.037
**581**	3-O8W-B4- 100	HDAC	0.44167	0.002
**582**	3-O8W-C4- 10	HDAC	0.44088	0
**583**	3-O8W-D4- 1	HDAC	0.331198	0.006
**584**	3-O8W-E4-l 0.1	HDAC	0.366346	0.003
**585**	3-O8W-F7-(1000	HDAC	0.53518	0.001
**586**	3-O8W-G7- 100	HDAC	0.391763	0.007
**587**	3-O8W-G1: 1000000	HDAC	0.173993	0.617
**588**	3-O8W-H7- 10	HDAC	0.443342	0
**589**	3-O8W-H1: 100000	HDAC	0.205199	0.341
**590**	3-O8W-I7-(1	HDAC	0.062597	0.919

**591**	3-O8W-I12	10000	HDAC	0.083358	0.909
**592**	3-O8W-J7-(	0.1	HDAC	0.393334	0
**593**	3-O8W-J12	1000	HDAC	0.099949	0.934
**594**	3-O8W-K3-	1	HDAC	0.196619	0.93
**595**	3-O8W-K12	100	HDAC	0.102378	0.938
**596**	3-O8W-L3-I	10	HDAC	0.097279	0.962
**597**	3-O8W-M3	100	HDAC	0.257439	0.474
**598**	3-O8W-N3-	1000	HDAC	0.41931	0.002
**599**	3-O8W-O3-	10000	HDAC	0.412419	0.072
**600**	7-O8W-A5-	10000	HDAC	0.529568	0.004
**601**	7-O8W-A7-	10000	HDAC	0.534673	0.003
**602**	7-O8W-A9-	1000	HDAC	0.478477	0
**603**	7-O8W-A12	10000	HDAC	0.478523	0.001
**604**	7-O8W-B5-	1000	HDAC	0.443073	0.001
**605**	7-O8W-B7-	1000	HDAC	0.498005	0.004
**606**	7-O8W-B12	1000	HDAC	0.488902	0
**607**	7-O8W-C5-	100	HDAC	0.456289	0
**608**	7-O8W-C7-	100	HDAC	0.437164	0.03
**609**	7-O8W-C9-	100	HDAC	0.485038	0
**610**	7-O8W-D7-	10	HDAC	0.438085	0.002
**611**	7-O8W-D9-	10	HDAC	0.481079	0
**612**	7-O8W-D12	100	HDAC	0.059598	0.995
**613**	7-O8W-E5-I	10	HDAC	0.432488	0
**614**	7-O8W-E7-(	1	HDAC	0.445433	0
**615**	7-O8W-E9-(	1	HDAC	0.463817	0
**616**	7-O8W-E12	10	HDAC	0.15587	0.893
**617**	7-O8W-F5-I	1	HDAC	0.368988	0
**618**	7-O8W-F7-I	10000	HDAC	0.453636	0.001
**619**	7-O8W-F9-(	0.1	HDAC	0.404912	0
**620**	7-O8W-F12	1	HDAC	0.36249	0.001
**621**	7-O8W-F19	10000	HDAC	0.392553	0
**622**	7-O8W-G7-	1000	HDAC	0.438975	0
**623**	7-O8W-G19	1000	HDAC	0.387431	0
**624**	7-O8W-H7-	100	HDAC	0.474049	0
**625**	7-O8W-I7-F	10	HDAC	0.405306	0

626	7-O8W-I19	100	HDAC	0.420995	0
627	7-O8W-J7-I	1	HDAC	0.199517	0.734
628	7-O8W-J19	10	HDAC	0.520346	0
629	7-O8W-K4-	1	HDAC	0.423918	0.001
630	7-O8W-K11	1	HDAC	0.083129	0.936
631	7-O8W-K18	1	HDAC	0.152098	0.738
632	7-O8W-K19	1	HDAC	0.092227	0.945
633	7-O8W-L2-1	0.1	HDAC	0.062517	1
634	7-O8W-L4-I	10	HDAC	0.136125	0.709
635	7-O8W-L5-I	1	HDAC	0.121152	0.778
636	7-O8W-L8-,	1	HDAC	0.360862	0.001
637	7-O8W-L10	1	HDAC	0.436131	0
638	7-O8W-L11	10	HDAC	0.384207	0.003
639	7-O8W-L14	1	HDAC	0.093697	0.841
640	7-O8W-L16	1	HDAC	0.078206	0.915
641	7-O8W-L18	10	HDAC	0.132985	0.82
642	7-O8W-M2	1	HDAC	0.413683	0
643	7-O8W-M5	10	HDAC	0.251378	0.05
644	7-O8W-M8	10	HDAC	0.523679	0
645	7-O8W-M1	10	HDAC	0.458779	0
646	7-O8W-M1	100	HDAC	0.417587	0
647	7-O8W-M1	10	HDAC	0.418213	0
648	7-O8W-M1	10	HDAC	0.266368	0.357
649	7-O8W-M1	100	HDAC	0.396134	0
650	7-O8W-N2-	10	HDAC	0.465	0
651	7-O8W-N4-	100	HDAC	0.524012	0
652	7-O8W-N5-	100	HDAC	0.492478	0
653	7-O8W-N8-	100	HDAC	0.492131	0
654	7-O8W-N1(	100	HDAC	0.566462	0
655	7-O8W-N14	100	HDAC	0.438707	0.002
656	7-O8W-N1(	100	HDAC	0.038107	0.988
657	7-O8W-N18	1000	HDAC	0.189011	0.375
658	7-O8W-O2-	100	HDAC	0.346699	0.001
659	7-O8W-O4-	1000	HDAC	0.485017	0
660	7-O8W-O5-	1000	HDAC	0.491221	0.001

**661**	7-O8W-O8-	1000	HDAC	0.461781	0.003
**662**	7-O8W-O1(	1000	HDAC	0.511725	0
**663**	7-O8W-O1:	1000	HDAC	0.471174	0
**664**	7-O8W-O1‹	1000	HDAC	0.501414	0
**665**	7-O8W-O1(	1000	HDAC	0.463362	0
**666**	7-O8W-P2-	1000	HDAC	0.449745	0
**667**	7-O8W-P4-	10000	HDAC	0.464901	0
**668**	7-O8W-P5-	10000	HDAC	0.455972	0.027
**669**	7-O8W-P8-	10000	HDAC	0.549821	0
**670**	7-O8W-P1(	10000	HDAC	0.454971	0
**671**	7-O8W-P11	10000	HDAC	0.519025	0.005
**672**	7-O8W-P1‹	10000	HDAC	0.438474	0
**673**	7-O8W-P1(	10000	HDAC	0.489871	0
**674**	7-O8W-P18	10000	HDAC	0.416848	0.001
