# Supplementary material for: Evaluating feature extraction in ovarian cancer cell line co-cultures using deep neural networks
Source: Commun Biol. 2025 Feb 25;8:303. doi: 10.1038/s42003-025-07766-w (PMC11862010; doi:10.1038/s42003-025-07766-w)
Supplement: Supplementary file 8 — Supplementary Data 6 [file 42003_2025_7766_MOESM8_ESM.pdf]

	Well_annotati	_concentra	/ell_Catego	Highest_ES	Pvalue
0	2-KB-A16-G	10000	EGFR	0.276855	0
1	2-KB-A19-E	10000	EGFR	0.264271	0
2	2-KB-B19-E	1000	EGFR	0.282008	0
3	2-KB-C16-G	1000	EGFR	0.173692	0.022
4	2-KB-C19-E	100	EGFR	0.229139	0
5	2-KB-D16-G	100	EGFR	0.16314	0.032
6	2-KB-D19-E	10	EGFR	0.129143	0.12
7	2-KB-E16-G	10	EGFR	0.168673	0.032
8	2-KB-E19-E	1	EGFR	0.269155	0
9	2-KB-F16-G	1	EGFR	0.147355	0.045
10	2-KB-K11-A	0.1	EGFR	0.139199	0.073
11	2-KB-L11-A	1	EGFR	0.14383	0.08
12	2-KB-L16-O	0.25	EGFR	0.121709	0.151
13	2-KB-L19-L	0.1	EGFR	0.14832	0.075
14	2-KB-M11-/	10	EGFR	0.212517	0
15	2-KB-M16-(	2.5	EGFR	0.142304	0.08
16	2-KB-M19-I	1	EGFR	0.17451	0.019
17	2-KB-N16-C	25	EGFR	0.124761	0.136
18	2-KB-N19-L	10	EGFR	0.120735	0.189
19	2-KB-O11-A	100	EGFR	0.211633	0.003
20	2-KB-O16-C	250	EGFR	0.261178	0
21	2-KB-O19-L	100	EGFR	0.151939	0.05
22	2-KB-P11-A	1000	EGFR	0.133468	0.116
23	2-KB-P16-C	2500	EGFR	0.278558	0
24	2-KB-P19-L	1000	EGFR	0.149926	0.065
25	3-KB-F21-R	10000	EGFR	0.281448	0
26	3-KB-G20-N	1000	EGFR	0.276059	0
27	3-KB-G21-F	1000	EGFR	0.289032	0
28	3-KB-H20-N	100	EGFR	0.235534	0
29	3-KB-H21-F	100	EGFR	0.13176	0.119
30	3-KB-I20-N	10	EGFR	0.139004	0.086
31	3-KB-I21-R	10	EGFR	0.123887	0.141
32	3-KB-J20-N	1	EGFR	0.121907	0.156
33	3-KB-J21-R	1	EGFR	0.106538	0.283

**34**	3-KB-K4-Ca	1	EGFR	0.135526	0.09
**35**	3-KB-K18-D	0.1	EGFR	0.153419	0.047
**36**	3-KB-K20-N	0.1	EGFR	0.140877	0.084
**37**	3-KB-L4-Ca	10	EGFR	0.15332	0.051
**38**	3-KB-L18-D	1	EGFR	0.120526	0.166
**39**	3-KB-M18-I	10	EGFR	0.155088	0.037
**40**	3-KB-N4-Ca	100	EGFR	0.193496	0.006
**41**	3-KB-N18-D	100	EGFR	0.157536	0.037
**42**	3-KB-O4-Ca	1000	EGFR	0.283314	0
**43**	3-KB-P4-Ca	10000	EGFR	0.208914	0.001
**44**	3-KB-P18-D	1000	EGFR	0.217458	0.002
**45**	4-KB-F13-Sa	1000	EGFR	0.155598	0.054
**46**	4-KB-G13-S	100	EGFR	0.161031	0.039
**47**	4-KB-G16-V	10000	EGFR	0.175031	0.014
**48**	4-KB-H13-S	10	EGFR	0.151518	0.056
**49**	4-KB-H16-V	1000	EGFR	0.122816	0.167
**50**	4-KB-I13-Sa	1	EGFR	0.147725	0.056
**51**	4-KB-I16-Va	100	EGFR	0.133237	0.106
**52**	4-KB-J13-Sa	0.1	EGFR	0.134004	0.118
**53**	4-KB-J16-Va	10	EGFR	0.131804	0.132
**54**	4-KB-K7-Icc	1	EGFR	0.144802	0.071
**55**	4-KB-K13-T	0.1	EGFR	0.125898	0.119
**56**	4-KB-K16-V	1	EGFR	0.152467	0.048
**57**	4-KB-L7-Icc	10	EGFR	0.139168	0.105
**58**	4-KB-L13-Te	1	EGFR	0.147726	0.051
**59**	4-KB-M7-Ic	100	EGFR	0.127127	0.131
**60**	4-KB-M13-T	10	EGFR	0.143146	0.068
**61**	4-KB-N13-T	100	EGFR	0.142307	0.074
**62**	4-KB-O7-Icc	1000	EGFR	0.134462	0.094
**63**	4-KB-P7-Icc	10000	EGFR	0.145396	0.076
**64**	4-KB-P13-T	1000	EGFR	0.153106	0.044
**65**	5-KB-F4-Po	1000	EGFR	0.17808	0.013
**66**	5-KB-F7-AZ	1000	EGFR	0.152987	0.043
**67**	5-KB-G4-Pc	100	EGFR	0.1508	0.055
**68**	5-KB-G7-AZ	100	EGFR	0.139835	0.075

**69**	5-KB-H4-Pc	10	EGFR	0.150755	0.057
**70**	5-KB-H7-AZ	10	EGFR	0.136103	0.109
**71**	5-KB-I4-Po:	1	EGFR	0.140036	0.091
**72**	5-KB-I7-AZI	1	EGFR	0.13442	0.114
**73**	5-KB-J4-Po:	0.1	EGFR	0.129994	0.131
**74**	5-KB-J7-AZI	0.1	EGFR	0.141745	0.069
**75**	5-KB-K7-Ol	0.1	EGFR	0.134121	0.11
**76**	5-KB-L7-Ol	1	EGFR	0.137694	0.098
**77**	5-KB-M7-Ol	10	EGFR	0.128905	0.146
**78**	5-KB-O7-Ol	100	EGFR	0.145275	0.073
**79**	5-KB-P7-Ol	1000	EGFR	0.158899	0.028
**80**	2-KB-A15-L	2500	VEGFR	0.105962	0.195
**81**	2-KB-A17-N	10000	VEGFR	0.113093	0.176
**82**	2-KB-A20-T	10000	VEGFR	0.141898	0.046
**83**	2-KB-B15-L	250	VEGFR	0.10829	0.186
**84**	2-KB-B17-N	1000	VEGFR	0.147202	0.028
**85**	2-KB-B20-T	1000	VEGFR	0.13597	0.062
**86**	2-KB-C15-L	25	VEGFR	0.110447	0.185
**87**	2-KB-C17-N	100	VEGFR	0.127308	0.101
**88**	2-KB-D15-L	2.5	VEGFR	0.100687	0.225
**89**	2-KB-D17-N	10	VEGFR	0.100403	0.304
**90**	2-KB-D20-T	100	VEGFR	0.109316	0.199
**91**	2-KB-E17-N	1	VEGFR	0.110635	0.173
**92**	2-KB-E20-Ti	10	VEGFR	0.13567	0.071
**93**	2-KB-F13-A	10000	VEGFR	0.10034	0.259
**94**	2-KB-F15-L	0.25	VEGFR	0.102802	0.245
**95**	2-KB-F19-R	10000	VEGFR	0.130885	0.08
**96**	2-KB-F20-Ti	1	VEGFR	0.107747	0.18
**97**	2-KB-F21-V	10000	VEGFR	0.103873	0.211
**98**	2-KB-G10-A	10000	VEGFR	0.143893	0.04
**99**	2-KB-G13-A	1000	VEGFR	0.10967	0.161
**100**	2-KB-G19-R	1000	VEGFR	0.109004	0.184
**101**	2-KB-G21-V	1000	VEGFR	0.098721	0.28
**102**	2-KB-H10-A	1000	VEGFR	0.110701	0.207
**103**	2-KB-H13-A	100	VEGFR	0.122992	0.109

**104**	2-KB-H21-\	100	VEGFR	0.104862	0.214
**105**	2-KB-I10-A	100	VEGFR	0.096485	0.3
**106**	2-KB-I13-A	10	VEGFR	0.117786	0.15
**107**	2-KB-I19-R	100	VEGFR	0.107251	0.207
**108**	2-KB-I21-V	10	VEGFR	0.107393	0.203
**109**	2-KB-J10-A	10	VEGFR	0.114736	0.168
**110**	2-KB-J13-A	1	VEGFR	0.104002	0.206
**111**	2-KB-J19-R	10	VEGFR	0.101398	0.248
**112**	2-KB-J21-V	1	VEGFR	0.108435	0.204
**113**	2-KB-K10-A	1	VEGFR	0.09846	0.3
**114**	2-KB-K13-V	0.1	VEGFR	0.104946	0.245
**115**	2-KB-K17-P	1	VEGFR	0.103145	0.251
**116**	2-KB-K19-R	1	VEGFR	0.101763	0.251
**117**	2-KB-L12-S	0.1	VEGFR	0.101773	0.241
**118**	2-KB-L13-V	1	VEGFR	0.102108	0.261
**119**	2-KB-L21-C	0.1	VEGFR	0.11741	0.141
**120**	2-KB-M12-S	1	VEGFR	0.10942	0.198
**121**	2-KB-M13-\	10	VEGFR	0.103439	0.235
**122**	2-KB-M17-I	10	VEGFR	0.112433	0.176
**123**	2-KB-M21-(	1	VEGFR	0.113394	0.156
**124**	2-KB-N12-S	10	VEGFR	0.103362	0.243
**125**	2-KB-N13-V	100	VEGFR	0.102985	0.276
**126**	2-KB-N17-F	100	VEGFR	0.115761	0.164
**127**	2-KB-N21-C	10	VEGFR	0.100117	0.272
**128**	2-KB-O12-S	100	VEGFR	0.103485	0.244
**129**	2-KB-O17-F	1000	VEGFR	0.113082	0.171
**130**	2-KB-O21-C	100	VEGFR	0.110074	0.17
**131**	2-KB-P12-S	1000	VEGFR	0.102265	0.26
**132**	2-KB-P13-V	1000	VEGFR	0.106575	0.197
**133**	2-KB-P17-P	10000	VEGFR	0.116024	0.164
**134**	2-KB-P21-C	1000	VEGFR	0.124291	0.11
**135**	3-KB-A3-Ca	1000	VEGFR	0.117086	0.119
**136**	3-KB-A6-Fo	1000	VEGFR	0.10532	0.212
**137**	3-KB-A18-L	1000	VEGFR	0.108409	0.197
**138**	3-KB-B3-Ca	100	VEGFR	0.100091	0.279

**139**	3-KB-B6-Fo	100	VEGFR	0.118358	0.141
**140**	3-KB-B18-L	100	VEGFR	0.11068	0.191
**141**	3-KB-C3-Ca	10	VEGFR	0.095192	0.309
**142**	3-KB-C6-Fo	10	VEGFR	0.110154	0.201
**143**	3-KB-C18-L	10	VEGFR	0.106764	0.212
**144**	3-KB-D3-Ca	1	VEGFR	0.095809	0.307
**145**	3-KB-D6-Fo	1	VEGFR	0.098462	0.284
**146**	3-KB-D18-L	1	VEGFR	0.114701	0.176
**147**	3-KB-E3-Cal	0.1	VEGFR	0.097774	0.308
**148**	3-KB-E6-Fo	0.1	VEGFR	0.100137	0.261
**149**	3-KB-E18-Li	0.1	VEGFR	0.108962	0.207
**150**	3-KB-F18-B	1000	VEGFR	0.112148	0.162
**151**	3-KB-G18-E	100	VEGFR	0.10821	0.186
**152**	3-KB-H18-E	10	VEGFR	0.105434	0.229
**153**	3-KB-I18-Br	1	VEGFR	0.103086	0.246
**154**	3-KB-J18-B	0.1	VEGFR	0.101117	0.24
**155**	4-KB-A12-E	10000	VEGFR	0.091734	0.308
**156**	4-KB-A15-G	2500	VEGFR	0.105878	0.22
**157**	4-KB-A20-N	10000	VEGFR	0.105891	0.233
**158**	4-KB-B12-E	1000	VEGFR	0.111312	0.164
**159**	4-KB-B15-G	250	VEGFR	0.10975	0.206
**160**	4-KB-B20-N	1000	VEGFR	0.110831	0.17
**161**	4-KB-C15-G	25	VEGFR	0.102417	0.215
**162**	4-KB-D12-E	100	VEGFR	0.104749	0.198
**163**	4-KB-D15-G	2.5	VEGFR	0.104065	0.22
**164**	4-KB-D20-N	100	VEGFR	0.106745	0.189
**165**	4-KB-E12-E	10	VEGFR	0.104746	0.223
**166**	4-KB-E20-N	10	VEGFR	0.108056	0.198
**167**	4-KB-F12-E	1	VEGFR	0.103289	0.266
**168**	4-KB-F15-G	0.25	VEGFR	0.105436	0.206
**169**	4-KB-F20-N	1	VEGFR	0.105237	0.246
**170**	4-KB-L16-T	1	VEGFR	0.102953	0.253
**171**	4-KB-M16-	10	VEGFR	0.10558	0.189
**172**	4-KB-N16-T	100	VEGFR	0.105554	0.208
**173**	4-KB-O16-T	1000	VEGFR	0.109058	0.182

**174**	4-KB-P16-T	10000	VEGFR	0.11342	0.181
**175**	2-KB-L10-Ic	1	PI3K	0.209563	0
**176**	2-KB-M10-I	10	PI3K	0.197853	0
**177**	2-KB-N10-I	100	PI3K	0.24121	0
**178**	2-KB-O10-I	1000	PI3K	0.237908	0
**179**	2-KB-P10-Ic	10000	PI3K	0.204299	0
**180**	3-KB-A16-P	2500	PI3K	0.168026	0.004
**181**	3-KB-C16-P	250	PI3K	0.198506	0.002
**182**	3-KB-D16-F	25	PI3K	0.255993	0
**183**	3-KB-E16-P	2.5	PI3K	0.211508	0
**184**	3-KB-F16-P	0.25	PI3K	0.179674	0
**185**	3-KB-F17-N	100000	PI3K	0.19939	0
**186**	3-KB-F19-D	500	PI3K	0.217468	0
**187**	3-KB-G17-N	10000	PI3K	0.205435	0
**188**	3-KB-G19-D	50	PI3K	0.257843	0
**189**	3-KB-H17-N	1000	PI3K	0.178431	0.003
**190**	3-KB-I17-M	100	PI3K	0.236937	0
**191**	3-KB-I19-D	5	PI3K	0.217321	0.001
**192**	3-KB-J17-N	10	PI3K	0.1693	0.002
**193**	3-KB-J19-D	0.5	PI3K	0.167359	0.004
**194**	3-KB-K19-D	0.05	PI3K	0.180295	0.001
**195**	3-KB-L8-Pic	1	PI3K	0.220996	0
**196**	3-KB-L21-T	0.1	PI3K	0.17631	0.001
**197**	3-KB-M8-Pi	10	PI3K	0.199096	0
**198**	3-KB-M21-T	1	PI3K	0.222407	0
**199**	3-KB-N8-Pi	100	PI3K	0.263261	0
**200**	3-KB-N21-T	10	PI3K	0.228421	0
**201**	3-KB-O8-Pi	1000	PI3K	0.222937	0
**202**	3-KB-O21-T	100	PI3K	0.242882	0
**203**	3-KB-P8-Pic	10000	PI3K	0.172087	0.006
**204**	3-KB-P21-T	1000	PI3K	0.259287	0
**205**	4-KB-A19-A	2500	PI3K	0.188125	0
**206**	4-KB-B19-A	250	PI3K	0.201578	0
**207**	4-KB-C19-A	25	PI3K	0.186603	0
**208**	4-KB-D19-A	2.5	PI3K	0.195079	0

**209**	4-KB-E19-A	0.25	PI3K	0.196493	0
**210**	4-KB-F14-N	1000	PI3K	0.166032	0.003
**211**	4-KB-G2-TG	2500	PI3K	0.174919	0
**212**	4-KB-G5-So	10000	PI3K	0.196595	0
**213**	4-KB-G14-N	100	PI3K	0.258497	0
**214**	4-KB-G20-E	10000	PI3K	0.198409	0
**215**	4-KB-H2-TG	250	PI3K	0.187394	0
**216**	4-KB-H5-So	1000	PI3K	0.185083	0
**217**	4-KB-H14-N	10	PI3K	0.266744	0
**218**	4-KB-H20-E	1000	PI3K	0.239252	0
**219**	4-KB-I2-TGI	25	PI3K	0.220227	0
**220**	4-KB-I5-Sor	100	PI3K	0.187639	0.002
**221**	4-KB-I14-N'	1	PI3K	0.188712	0.001
**222**	4-KB-I20-Bu	100	PI3K	0.202632	0
**223**	4-KB-J2-TGI	2.5	PI3K	0.189573	0
**224**	4-KB-J5-Sor	10	PI3K	0.197515	0
**225**	4-KB-J20-Bu	10	PI3K	0.184366	0.001
**226**	4-KB-K2-TG	0.25	PI3K	0.187592	0.001
**227**	4-KB-K4-Da	0.1	PI3K	0.186438	0.001
**228**	4-KB-K5-So	1	PI3K	0.189981	0
**229**	4-KB-K14-N	0.1	PI3K	0.199217	0
**230**	4-KB-K20-B	1	PI3K	0.201229	0.001
**231**	4-KB-L4-Da	1	PI3K	0.208318	0
**232**	4-KB-L14-G	0.1	PI3K	0.180078	0.002
**233**	4-KB-L15-T	1	PI3K	0.204319	0
**234**	4-KB-L21-C	0.1	PI3K	0.202013	0
**235**	4-KB-M14-(	1	PI3K	0.185272	0.002
**236**	4-KB-M15-T	10	PI3K	0.187361	0
**237**	4-KB-M21-(	1	PI3K	0.197866	0.001
**238**	4-KB-N4-Da	10	PI3K	0.251165	0
**239**	4-KB-N14-G	10	PI3K	0.211374	0
**240**	4-KB-N15-T	100	PI3K	0.194401	0
**241**	4-KB-N21-C	10	PI3K	0.198189	0
**242**	4-KB-O4-Da	100	PI3K	0.237705	0
**243**	4-KB-O14-G	100	PI3K	0.255637	0

**244**	4-KB-O15-T	1000	PI3K	0.215985	0
**245**	4-KB-O21-C	100	PI3K	0.253855	0
**246**	4-KB-P4-Da	1000	PI3K	0.26766	0
**247**	4-KB-P14-G	1000	PI3K	0.246665	0
**248**	4-KB-P15-T	10000	PI3K	0.210652	0
**249**	4-KB-P21-C	1000	PI3K	0.212526	0
**250**	5-KB-A6-LY	2500	PI3K	0.249497	0
**251**	5-KB-A7-AN	1000	PI3K	0.210554	0
**252**	5-KB-A16-A	2500	PI3K	0.240464	0
**253**	5-KB-A17-P	10000	PI3K	0.183603	0.001
**254**	5-KB-B6-LY	250	PI3K	0.253371	0
**255**	5-KB-B7-AN	100	PI3K	0.192022	0
**256**	5-KB-B17-P	1000	PI3K	0.203444	0.001
**257**	5-KB-C6-LY	25	PI3K	0.204449	0
**258**	5-KB-C7-AN	10	PI3K	0.191015	0
**259**	5-KB-C16-A	250	PI3K	0.261188	0
**260**	5-KB-C17-P	100	PI3K	0.200546	0
**261**	5-KB-D6-LY	2.5	PI3K	0.184023	0
**262**	5-KB-D7-AN	1	PI3K	0.180877	0.001
**263**	5-KB-D16-A	25	PI3K	0.206103	0
**264**	5-KB-D17-P	10	PI3K	0.189692	0
**265**	5-KB-E6-LY	0.25	PI3K	0.177837	0
**266**	5-KB-E7-AN	0.1	PI3K	0.19204	0
**267**	5-KB-E16-A	2.5	PI3K	0.203611	0
**268**	5-KB-E17-P	1	PI3K	0.202767	0
**269**	5-KB-F11-G	10000	PI3K	0.257546	0
**270**	5-KB-F16-A	0.25	PI3K	0.185288	0
**271**	5-KB-G9-Se	10000	PI3K	0.225426	0
**272**	5-KB-G11-C	1000	PI3K	0.23641	0
**273**	5-KB-H9-Se	1000	PI3K	0.200178	0
**274**	5-KB-H11-C	100	PI3K	0.2112	0
**275**	5-KB-I9-Ser	100	PI3K	0.19813	0
**276**	5-KB-I11-G	10	PI3K	0.187314	0
**277**	5-KB-J9-Ser	10	PI3K	0.198795	0
**278**	5-KB-J11-G	1	PI3K	0.200228	0

**279**	5-KB-K9-Se	1	PI3K	0.194808	0
**280**	5-KB-L14-A	0.1	PI3K	0.194765	0.001
**281**	5-KB-L20-Z	1	PI3K	0.174919	0.001
**282**	5-KB-L23-O	0.1	PI3K	0.17797	0.001
**283**	5-KB-M14-A	1	PI3K	0.200986	0
**284**	5-KB-M20-Z	10	PI3K	0.194713	0.001
**285**	5-KB-M23-O	1	PI3K	0.197278	0
**286**	5-KB-N14-A	10	PI3K	0.237999	0
**287**	5-KB-N20-Z	100	PI3K	0.238954	0
**288**	5-KB-N23-C	10	PI3K	0.243548	0
**289**	5-KB-O14-A	100	PI3K	0.264063	0
**290**	5-KB-O20-Z	1000	PI3K	0.230624	0
**291**	5-KB-O23-C	100	PI3K	0.273262	0
**292**	5-KB-P14-A	1000	PI3K	0.235497	0
**293**	5-KB-P20-Z	10000	PI3K	0.247119	0
**294**	5-KB-P23-C	1000	PI3K	0.205489	0
**295**	6-KB-A8-TG	10000	PI3K	0.251727	0
**296**	6-KB-B8-TG	1000	PI3K	0.201796	0
**297**	6-KB-C8-TG	100	PI3K	0.203028	0
**298**	6-KB-D8-TG	10	PI3K	0.192176	0.002
**299**	6-KB-E8-TG	1	PI3K	0.188649	0.001
**300**	6-KB-L6-GD	1	PI3K	0.199475	0
**301**	6-KB-M6-GI	10	PI3K	0.193689	0.001
**302**	6-KB-N6-GI	100	PI3K	0.248983	0
**303**	6-KB-O6-GI	1000	PI3K	0.253481	0
**304**	6-KB-P6-GD	10000	PI3K	0.236997	0
**305**	1-KB-F11-A	10000	Topoisome	0.411616	0
**306**	1-KB-G11-A	1000	Topoisome	0.466046	0
**307**	1-KB-G20-E	1000	Topoisome	0.444872	0
**308**	1-KB-H11-A	100	Topoisome	0.319912	0
**309**	1-KB-H20-E	100	Topoisome	0.423063	0
**310**	1-KB-I11-Ar	10	Topoisome	0.036535	0.937
**311**	1-KB-I20-Ep	10	Topoisome	0.232439	0.007
**312**	1-KB-J11-Ar	1	Topoisome	0.04509	0.912
**313**	1-KB-J20-Ep	1	Topoisome	0.163586	0.072

**314**	1-KB-K11-S	1	Topoisome	0.289321	0
**315**	1-KB-K20-E	0.1	Topoisome	0.105282	0.408
**316**	1-KB-L11-Sl	10	Topoisome	0.325884	0
**317**	1-KB-L14-T	1	Topoisome	0.098865	0.433
**318**	1-KB-M11-S	100	Topoisome	0.385526	0
**319**	1-KB-M14-T	10	Topoisome	0.136175	0.175
**320**	1-KB-N14-T	100	Topoisome	0.309722	0
**321**	1-KB-O11-S	1000	Topoisome	0.390309	0
**322**	1-KB-O14-T	1000	Topoisome	0.366381	0
**323**	1-KB-P11-S	10000	Topoisome	0.370726	0
**324**	1-KB-P14-T	10000	Topoisome	0.393768	0
**325**	3-KB-A11-E	10000	Topoisome	0.091991	0.501
**326**	3-KB-B11-E	1000	Topoisome	0.170617	0.061
**327**	3-KB-C11-E	100	Topoisome	0.125591	0.235
**328**	3-KB-D11-E	10	Topoisome	0.052331	0.871
**329**	3-KB-E11-E	1	Topoisome	0.027798	0.984
**330**	3-KB-G9-Da	1000	Topoisome	0.248476	0.001
**331**	3-KB-G10-T	10000	Topoisome	0.032835	0.967
**332**	3-KB-H9-Da	100	Topoisome	0.149364	0.128
**333**	3-KB-H10-T	1000	Topoisome	0.060656	0.797
**334**	3-KB-I9-Dau	10	Topoisome	0.082419	0.59
**335**	3-KB-I10-Te	100	Topoisome	0.021588	0.991
**336**	3-KB-J9-Dau	1	Topoisome	0.088209	0.561
**337**	3-KB-J10-Te	10	Topoisome	0.077871	0.657
**338**	3-KB-K7-Ida	0.1	Topoisome	0.034509	0.967
**339**	3-KB-K9-Da	0.1	Topoisome	0.023526	0.99
**340**	3-KB-K10-T	1	Topoisome	0.022252	0.992
**341**	3-KB-L6-Do	0.1	Topoisome	0.022252	0.991
**342**	3-KB-L7-Ida	1	Topoisome	0.051253	0.88
**343**	3-KB-L9-Val	0.5	Topoisome	0.05059	0.879
**344**	3-KB-L10-M	0.1	Topoisome	0.058879	0.811
**345**	3-KB-L16-P	1	Topoisome	0.022252	0.992
**346**	3-KB-M6-Do	1	Topoisome	0.02192	0.995
**347**	3-KB-M7-Id	10	Topoisome	0.083947	0.588
**348**	3-KB-M9-Va	5	Topoisome	0.090855	0.55

**349**	3-KB-M10-I	1	Topoisome	0.022584	0.989
**350**	3-KB-M16-I	10	Topoisome	0.057001	0.795
**351**	3-KB-N6-Dc	10	Topoisome	0.03402	0.97
**352**	3-KB-N9-Va	50	Topoisome	0.134209	0.193
**353**	3-KB-N10-N	10	Topoisome	0.153309	0.098
**354**	3-KB-N16-F	100	Topoisome	0.033036	0.975
**355**	3-KB-O6-Dc	100	Topoisome	0.189081	0.031
**356**	3-KB-O7-Ida	100	Topoisome	0.178737	0.046
**357**	3-KB-O9-Va	500	Topoisome	0.162751	0.086
**358**	3-KB-O10-N	100	Topoisome	0.206184	0.016
**359**	3-KB-O16-F	1000	Topoisome	0.022252	0.99
**360**	3-KB-P6-Dc	1000	Topoisome	0.226502	0.002
**361**	3-KB-P7-Ida	1000	Topoisome	0.251544	0.002
**362**	3-KB-P9-Va	5000	Topoisome	0.249168	0
**363**	3-KB-P10-N	1000	Topoisome	0.252833	0.001
**364**	3-KB-P16-P	10000	Topoisome	0.032366	0.968
**365**	1-KB-A10-V	10000	Mitotic	0.817399	0
**366**	1-KB-A13-Ix	1000	Mitotic	0.77821	0
**367**	1-KB-A18-P	1000	Mitotic	0.764175	0
**368**	1-KB-B10-V	1000	Mitotic	0.721026	0
**369**	1-KB-B13-Ix	100	Mitotic	0.413211	0
**370**	1-KB-B18-P	100	Mitotic	0.699963	0
**371**	1-KB-C10-V	100	Mitotic	0.663362	0
**372**	1-KB-C13-Ix	10	Mitotic	0.236581	0.017
**373**	1-KB-C18-P	10	Mitotic	0.646067	0
**374**	1-KB-D10-V	10	Mitotic	0.429959	0
**375**	1-KB-D13-I:	1	Mitotic	0.109824	0.514
**376**	1-KB-D18-F	1	Mitotic	0.462531	0
**377**	1-KB-E10-V	1	Mitotic	0.031901	0.978
**378**	1-KB-E13-Ix	0.1	Mitotic	0.085494	0.681
**379**	1-KB-E18-P	0.1	Mitotic	0.236433	0.011
**380**	1-KB-F13-V	1000	Mitotic	0.567754	0
**381**	1-KB-G13-V	100	Mitotic	0.130768	0.317
**382**	1-KB-G15-E	1000	Mitotic	0.577002	0
**383**	1-KB-H13-V	10	Mitotic	0.153826	0.215

**384**	1-KB-H15-E	100	Mitotic	0.553551	0
**385**	1-KB-I13-Vi	1	Mitotic	0.110436	0.463
**386**	1-KB-I15-Er	10	Mitotic	0.429686	0
**387**	1-KB-J13-Vi	0.1	Mitotic	0.078268	0.749
**388**	1-KB-J15-Er	1	Mitotic	0.239165	0.013
**389**	1-KB-K7-Vir	0.1	Mitotic	0.029733	0.986
**390**	1-KB-K15-E	0.1	Mitotic	0.047066	0.931
**391**	1-KB-L7-Vir	1	Mitotic	0.028297	0.988
**392**	1-KB-L20-V	0.1	Mitotic	0.099966	0.591
**393**	1-KB-M7-Vi	10	Mitotic	0.075913	0.767
**394**	1-KB-M20-\	1	Mitotic	0.110337	0.459
**395**	1-KB-N20-\	10	Mitotic	0.096481	0.573
**396**	1-KB-O7-Vi	100	Mitotic	0.13888	0.265
**397**	1-KB-O20-\	100	Mitotic	0.087775	0.637
**398**	1-KB-P7-Vir	1000	Mitotic	0.309443	0.001
**399**	1-KB-P20-V	1000	Mitotic	0.239805	0.014
**400**	3-KB-A7-Dc	1000	Mitotic	0.092755	0.597
**401**	3-KB-B7-Dc	100	Mitotic	0.131293	0.318
**402**	3-KB-C7-Dc	10	Mitotic	0.126183	0.346
**403**	3-KB-D7-Dc	1	Mitotic	0.037206	0.973
**404**	3-KB-E7-Do	0.1	Mitotic	0.028339	0.988
**405**	6-KB-L19-A	1	Mitotic	0.055841	0.88
**406**	6-KB-M19-/	10	Mitotic	0.046446	0.918
**407**	6-KB-N19-A	100	Mitotic	0.050582	0.913
**408**	6-KB-O19-A	1000	Mitotic	0.027584	0.992
**409**	6-KB-P19-A	10000	Mitotic	0.051777	0.901
**410**	2-KB-A12-T	250	MEK1/2	0.397185	0
**411**	2-KB-B12-T	25	MEK1/2	0.304468	0.015
**412**	2-KB-D12-T	2.5	MEK1/2	0.361485	0.002
**413**	2-KB-E12-Ti	0.25	MEK1/2	0.106215	0.631
**414**	2-KB-F12-Ti	2.5000000	MEK1/2	0.098981	0.722
**415**	2-KB-F14-C	1000	MEK1/2	0.405185	0
**416**	2-KB-G14-C	100	MEK1/2	0.365929	0
**417**	2-KB-H14-C	10	MEK1/2	0.249638	0.04
**418**	2-KB-I14-Cc	1	MEK1/2	0.133755	0.475

**419**	2-KB-K14-C	0.1	MEK1/2	0.103913	0.683
**420**	2-KB-L20-S	1	MEK1/2	0.103913	0.66
**421**	2-KB-M20-S	10	MEK1/2	0.145118	0.41
**422**	2-KB-N20-S	100	MEK1/2	0.147163	0.382
**423**	2-KB-O20-S	1000	MEK1/2	0.26382	0.027
**424**	2-KB-P20-S	10000	MEK1/2	0.283753	0.015
**425**	4-KB-A10-B	1000	MEK1/2	0.194698	0.16
**426**	4-KB-A13-P	1000	MEK1/2	0.149144	0.39
**427**	4-KB-B10-B	100	MEK1/2	0.194338	0.158
**428**	4-KB-B13-P	100	MEK1/2	0.196333	0.156
**429**	4-KB-C10-B	10	MEK1/2	0.179567	0.204
**430**	4-KB-C13-P	10	MEK1/2	0.182828	0.221
**431**	4-KB-D10-B	1	MEK1/2	0.133818	0.492
**432**	4-KB-D13-P	1	MEK1/2	0.106873	0.665
**433**	4-KB-E10-B	0.1	MEK1/2	0.142712	0.402
**434**	4-KB-E13-P	0.1	MEK1/2	0.114844	0.6
**435**	4-KB-L19-G	0.25	MEK1/2	0.108188	0.652
**436**	4-KB-M19-G	2.5	MEK1/2	0.106544	0.662
**437**	4-KB-N19-G	25	MEK1/2	0.108583	0.654
**438**	4-KB-O19-G	250	MEK1/2	0.106215	0.634
**439**	4-KB-P19-G	2500	MEK1/2	0.128466	0.506
**440**	1-KB-L2-Ola	1	PARP	0.11745	0.66
**441**	1-KB-L6-Ru	1	PARP	0.410914	0
**442**	1-KB-M2-Ol	10	PARP	0.118906	0.629
**443**	1-KB-M6-Ru	10	PARP	0.326941	0.008
**444**	1-KB-N2-Ol	100	PARP	0.0794	0.853
**445**	1-KB-N6-Ru	100	PARP	0.184482	0.298
**446**	1-KB-O2-Ol	1000	PARP	0.417552	0
**447**	1-KB-O6-Ru	1000	PARP	0.339001	0.006
**448**	1-KB-P2-Ola	10000	PARP	0.396971	0.001
**449**	1-KB-P6-Ru	10000	PARP	0.327171	0.015
**450**	7-KB-A3-Tal	1000	PARP	0.359407	0.003
**451**	7-KB-B2-Ve	10000	PARP	0.316239	0.011
**452**	7-KB-B3-Tal	100	PARP	0.349856	0.002
**453**	7-KB-C2-Ve	1000	PARP	0.247111	0.085

**454**	7-KB-C3-Tal	10	PARP	0.21454	0.165
**455**	7-KB-D2-Ve	100	PARP	0.141899	0.518
**456**	7-KB-D3-Ta	1	PARP	0.17834	0.291
**457**	7-KB-E2-Vel	10	PARP	0.082049	0.865
**458**	7-KB-E3-Tal	0.1	PARP	0.286065	0.034
**459**	7-KB-F2-Vel	1	PARP	0.325575	0.008
**460**	7-KB-G2-Ni	10000	PARP	0.367008	0.003
**461**	7-KB-H2-Ni	1000	PARP	0.418581	0
**462**	7-KB-I2-Nir	100	PARP	0.325694	0.012
**463**	7-KB-J2-Nir	10	PARP	0.352278	0.004
**464**	7-KB-K2-Ni	1	PARP	0.299729	0.021
**465**	3-KB-A19-D	1000	CDK	0.326335	0
**466**	3-KB-B19-D	100	CDK	0.303919	0
**467**	3-KB-B23-A	2500	CDK	0.323328	0
**468**	3-KB-C19-D	10	CDK	0.30113	0
**469**	3-KB-C23-A	250	CDK	0.316203	0
**470**	3-KB-D19-D	1	CDK	0.173708	0.048
**471**	3-KB-D23-A	25	CDK	0.163299	0.075
**472**	3-KB-E19-D	0.1	CDK	0.113834	0.308
**473**	3-KB-E23-A	2.5	CDK	0.324966	0
**474**	3-KB-F23-A	0.25	CDK	0.097433	0.471
**475**	3-KB-K17-P	1	CDK	0.099523	0.454
**476**	3-KB-L19-R	1	CDK	0.095624	0.469
**477**	3-KB-M17-I	10	CDK	0.10762	0.378
**478**	3-KB-M19-I	10	CDK	0.11059	0.331
**479**	3-KB-N17-F	100	CDK	0.145717	0.143
**480**	3-KB-N19-F	100	CDK	0.102844	0.426
**481**	3-KB-O17-F	1000	CDK	0.148283	0.134
**482**	3-KB-O19-F	1000	CDK	0.125157	0.256
**483**	3-KB-P17-P	10000	CDK	0.191997	0.019
**484**	3-KB-P19-R	10000	CDK	0.220134	0.005
**485**	4-KB-A4-SN	10000	CDK	0.298637	0
**486**	4-KB-A8-Mi	10000	CDK	0.296533	0
**487**	4-KB-B4-SN	1000	CDK	0.297223	0
**488**	4-KB-B8-Mi	1000	CDK	0.288083	0

**489**	4-KB-C4-SN	100	CDK	0.229645	0.001
**490**	4-KB-C8-Mi	100	CDK	0.145905	0.141
**491**	4-KB-D4-SN	10	CDK	0.097683	0.469
**492**	4-KB-D8-Mi	10	CDK	0.089725	0.514
**493**	4-KB-E4-SN	1	CDK	0.09135	0.509
**494**	4-KB-E8-Mi	1	CDK	0.092833	0.515
**495**	4-KB-F4-Sel	10000	CDK	0.115286	0.3
**496**	4-KB-F22-A	10000	CDK	0.308986	0
**497**	4-KB-G4-Se	1000	CDK	0.092616	0.53
**498**	4-KB-G22-A	1000	CDK	0.305908	0
**499**	4-KB-H4-Se	100	CDK	0.089382	0.538
**500**	4-KB-H22-A	100	CDK	0.105117	0.4
**501**	4-KB-I4-Seli	10	CDK	0.094948	0.49
**502**	4-KB-I22-Al	10	CDK	0.094593	0.507
**503**	4-KB-J4-Sel	1	CDK	0.091438	0.514
**504**	4-KB-J22-Al	1	CDK	0.09327	0.496
**505**	5-KB-A19-A	10000	CDK	0.312109	0
**506**	5-KB-B19-A	1000	CDK	0.233707	0.003
**507**	5-KB-C19-A	100	CDK	0.1074	0.365
**508**	5-KB-D19-A	10	CDK	0.094682	0.487
**509**	5-KB-E19-A	1	CDK	0.094345	0.495
**510**	5-KB-K17-A	1	CDK	0.097213	0.456
**511**	5-KB-M17-A	10	CDK	0.093284	0.506
**512**	5-KB-N17-A	100	CDK	0.093483	0.527
**513**	5-KB-O17-A	1000	CDK	0.283486	0
**514**	5-KB-P17-A	10000	CDK	0.286211	0
**515**	6-KB-A17-S	1000	CDK	0.097519	0.465
**516**	6-KB-B17-S	100	CDK	0.095331	0.489
**517**	6-KB-C17-S	10	CDK	0.092646	0.518
**518**	6-KB-D17-S	1	CDK	0.090811	0.517
**519**	6-KB-E17-S	0.1	CDK	0.092016	0.527
**520**	6-KB-L15-T	1	CDK	0.089883	0.52
**521**	6-KB-M15-T	10	CDK	0.092392	0.528
**522**	6-KB-N15-T	100	CDK	0.105865	0.398
**523**	6-KB-O15-T	1000	CDK	0.263564	0.001

**524**	6-KB-P15-T	10000	CDK	0.269576	0
**525**	7-KB-A21-d	10000	BET	0.495097	0
**526**	7-KB-A22-P	30000	BET	0.472934	0
**527**	7-KB-B21-d	1000	BET	0.55019	0
**528**	7-KB-B22-P	3000	BET	0.562506	0
**529**	7-KB-C21-d	100	BET	0.529025	0
**530**	7-KB-C22-P	300	BET	0.467398	0
**531**	7-KB-D21-c	10	BET	0.417383	0
**532**	7-KB-D22-F	30	BET	0.525718	0
**533**	7-KB-E21-d	1	BET	0.419696	0
**534**	7-KB-E22-P	3	BET	0.460013	0
**535**	7-KB-G10-E	10000	BET	0.539636	0
**536**	7-KB-G15-I-	10000	BET	0.540476	0
**537**	7-KB-H10-E	1000	BET	0.523115	0
**538**	7-KB-H15-I-	1000	BET	0.539472	0
**539**	7-KB-I10-Bi	100	BET	0.38764	0
**540**	7-KB-I15-I-I	100	BET	0.368473	0
**541**	7-KB-J10-Bi	10	BET	0.373761	0
**542**	7-KB-J15-I-I	10	BET	0.359881	0
**543**	7-KB-K10-B	1	BET	0.384997	0
**544**	7-KB-K13-N	1	BET	0.400529	0
**545**	7-KB-K15-I-	1	BET	0.371117	0
**546**	7-KB-L12-N	1	BET	0.348315	0
**547**	7-KB-L13-N	10	BET	0.547411	0
**548**	7-KB-L20-J(	1	BET	0.364838	0
**549**	7-KB-L23-A	0.03	BET	0.377396	0
**550**	7-KB-M12-I	10	BET	0.362147	0
**551**	7-KB-M13-I	100	BET	0.552746	0
**552**	7-KB-M20-J	10	BET	0.361533	0
**553**	7-KB-M23-/	0.3	BET	0.369795	0
**554**	7-KB-N12-I	100	BET	0.397224	0
**555**	7-KB-N13-I	1000	BET	0.568416	0
**556**	7-KB-N20-J	100	BET	0.400859	0
**557**	7-KB-N23-A	3	BET	0.338037	0
**558**	7-KB-O12-I	1000	BET	0.591543	0

**559**	7-KB-O20-J	1000	BET	0.589963	0
**560**	7-KB-O23-A	30	BET	0.58633	0
**561**	7-KB-P12-N	10000	BET	0.553813	0
**562**	7-KB-P13-N	10000	BET	0.533423	0
**563**	7-KB-P20-J	10000	BET	0.556543	0
**564**	7-KB-P23-A	300	BET	0.520049	0
**565**	8-KB-K22-C	1	BET	0.385327	0
**566**	8-KB-L22-C	10	BET	0.364838	0
**567**	8-KB-M22-(	100	BET	0.360442	0.001
**568**	8-KB-N22-C	1000	BET	0.34464	0
**569**	8-KB-O22-C	10000	BET	0.348941	0
**570**	1-KB-A3-Vo	10000	HDAC	0.578593	0.38
**571**	1-KB-B3-Vo	1000	HDAC	0.203876	0.02
**572**	1-KB-C3-Vo	100	HDAC	0.159353	0.009
**573**	1-KB-D3-Vc	10	HDAC	0.178805	0.006
**574**	1-KB-E3-Vo	1	HDAC	0.085977	0.409
**575**	1-KB-L12-R	0.1	HDAC	0.110411	0.165
**576**	1-KB-M12-I	1	HDAC	0.278056	0
**577**	1-KB-N12-F	10	HDAC	0.177428	0.005
**578**	1-KB-O12-F	100	HDAC	0.182553	0.001
**579**	1-KB-P12-R	1000	HDAC	0.188463	0
**580**	3-KB-A4-Pa	1000	HDAC	0.164141	0.012
**581**	3-KB-B4-Pa	100	HDAC	0.217223	0.002
**582**	3-KB-C4-Pa	10	HDAC	0.173306	0.007
**583**	3-KB-D4-Pa	1	HDAC	0.121816	0.104
**584**	3-KB-E4-Pa	0.1	HDAC	0.109509	0.157
**585**	3-KB-F7-Qu	1000	HDAC	0.173034	0.004
**586**	3-KB-G7-Qu	100	HDAC	0.231153	0.001
**587**	3-KB-G12-V	1000000	HDAC	0.129885	0.071
**588**	3-KB-H7-Qu	10	HDAC	0.141472	0.04
**589**	3-KB-H12-V	100000	HDAC	0.266254	0
**590**	3-KB-I7-Qui	1	HDAC	0.165701	0.012
**591**	3-KB-I12-Va	10000	HDAC	0.121861	0.094
**592**	3-KB-J7-Qu	0.1	HDAC	0.128369	0.061
**593**	3-KB-J12-Va	1000	HDAC	0.101912	0.22

**594**	3-KB-K3-Be	1	HDAC	0.236567	0
**595**	3-KB-K12-V	100	HDAC	0.123701	0.11
**596**	3-KB-L3-Bel	10	HDAC	0.262328	0
**597**	3-KB-M3-Be	100	HDAC	0.314319	0
**598**	3-KB-N3-Be	1000	HDAC	0.235099	0
**599**	3-KB-O3-Be	10000	HDAC	0.177222	0.003
**600**	7-KB-A5-Mc	10000	HDAC	0.291177	0
**601**	7-KB-A7-CL	10000	HDAC	0.264846	0
**602**	7-KB-A9-Gi	1000	HDAC	0.292423	0
**603**	7-KB-A12-R	10000	HDAC	0.305266	0
**604**	7-KB-B5-Mc	1000	HDAC	0.340468	0
**605**	7-KB-B7-CL	1000	HDAC	0.266317	0
**606**	7-KB-B12-R	1000	HDAC	0.281576	0
**607**	7-KB-C5-Mc	100	HDAC	0.206053	0
**608**	7-KB-C7-CL	100	HDAC	0.303218	0
**609**	7-KB-C9-Gi	100	HDAC	0.272035	0
**610**	7-KB-D7-CL	10	HDAC	0.379847	0
**611**	7-KB-D9-Gi	10	HDAC	0.11254	0.17
**612**	7-KB-D12-F	100	HDAC	0.152609	0.03
**613**	7-KB-E5-Mc	10	HDAC	0.235797	0
**614**	7-KB-E7-CU	1	HDAC	0.331089	0
**615**	7-KB-E9-Giv	1	HDAC	0.135088	0.044
**616**	7-KB-E12-R	10	HDAC	0.244436	0
**617**	7-KB-F5-Mc	1	HDAC	0.201	0.001
**618**	7-KB-F7-Res	10000	HDAC	0.274761	0
**619**	7-KB-F9-Giv	0.1	HDAC	0.143856	0.043
**620**	7-KB-F12-R	1	HDAC	0.178727	0.001
**621**	7-KB-F19-P	10000	HDAC	0.319348	0
**622**	7-KB-G7-Re	1000	HDAC	0.341313	0
**623**	7-KB-G19-F	1000	HDAC	0.314475	0
**624**	7-KB-H7-Re	100	HDAC	0.227007	0
**625**	7-KB-I7-Res	10	HDAC	0.280949	0
**626**	7-KB-I19-P(	100	HDAC	0.295042	0
**627**	7-KB-J7-Res	1	HDAC	0.295357	0
**628**	7-KB-J19-P	10	HDAC	0.17091	0.004

**629**	7-KB-K4-En	1	HDAC	0.269085	0
**630**	7-KB-K11-A	1	HDAC	0.210792	0.001
**631**	7-KB-K18-T	1	HDAC	0.131736	0.047
**632**	7-KB-K19-P	1	HDAC	0.270403	0
**633**	7-KB-L2-Ta	0.1	HDAC	0.283356	0
**634**	7-KB-L4-En	10	HDAC	0.267225	0
**635**	7-KB-L5-Pra	1	HDAC	0.316959	0
**636**	7-KB-L8-Ab	1	HDAC	0.306495	0
**637**	7-KB-L10-T	1	HDAC	0.148978	0.029
**638**	7-KB-L11-A	10	HDAC	0.222297	0
**639**	7-KB-L14-T	1	HDAC	0.270469	0
**640**	7-KB-L16-R	1	HDAC	0.113026	0.14
**641**	7-KB-L18-T	10	HDAC	0.21179	0.001
**642**	7-KB-M2-Ta	1	HDAC	0.290394	0
**643**	7-KB-M5-Pr	10	HDAC	0.27474	0
**644**	7-KB-M8-Al	10	HDAC	0.302824	0
**645**	7-KB-M10-	10	HDAC	0.19498	0
**646**	7-KB-M11-	100	HDAC	0.261683	0
**647**	7-KB-M14-	10	HDAC	0.287295	0
**648**	7-KB-M16-I	10	HDAC	0.132074	0.067
**649**	7-KB-M18-	100	HDAC	0.298665	0
**650**	7-KB-N2-Ta	10	HDAC	0.286208	0
**651**	7-KB-N4-En	100	HDAC	0.285451	0
**652**	7-KB-N5-Pr	100	HDAC	0.168861	0.006
**653**	7-KB-N8-Ab	100	HDAC	0.363023	0
**654**	7-KB-N10-T	100	HDAC	0.287288	0
**655**	7-KB-N14-T	100	HDAC	0.111601	0.146
**656**	7-KB-N16-F	100	HDAC	0.112347	0.154
**657**	7-KB-N18-T	1000	HDAC	0.197613	0.002
**658**	7-KB-O2-Ta	100	HDAC	0.295655	0
**659**	7-KB-O4-En	1000	HDAC	0.309139	0
**660**	7-KB-O5-Pr	1000	HDAC	0.341263	0
**661**	7-KB-O8-Ab	1000	HDAC	0.341465	0
**662**	7-KB-O10-T	1000	HDAC	0.355281	0
**663**	7-KB-O11-A	1000	HDAC	0.315194	0

**664**	7-KB-O14-T	1000	HDAC	0.111931	0.145
**665**	7-KB-O16-F	1000	HDAC	0.180414	0.004
**666**	7-KB-P2-Ta	1000	HDAC	0.13093	0.062
**667**	7-KB-P4-En	10000	HDAC	0.348524	0
**668**	7-KB-P5-Pr	10000	HDAC	0.277619	0
**669**	7-KB-P8-Ab	10000	HDAC	0.269875	0
**670**	7-KB-P10-T	10000	HDAC	0.347636	0
**671**	7-KB-P11-A	10000	HDAC	0.272886	0
**672**	7-KB-P14-T	10000	HDAC	0.119878	0.104
**673**	7-KB-P16-R	10000	HDAC	0.319343	0
**674**	7-KB-P18-T	10000	HDAC	0.314026	0
**0**	2-KW-A16-	10000	EGFR	0.393641	0.15
**1**	2-KW-A19-	10000	EGFR	0.367807	0.053
**2**	2-KW-B19-	1000	EGFR	0.296084	0.14
**3**	2-KW-C16-	1000	EGFR	0.389351	0.003
**4**	2-KW-C19-	100	EGFR	0.331299	0.009
**5**	2-KW-D16-	100	EGFR	0.382183	0.002
**6**	2-KW-D19-	10	EGFR	0.149777	0.231
**7**	2-KW-E16-	10	EGFR	0.128615	0.408
**8**	2-KW-E19-	1	EGFR	0.405055	0.007
**9**	2-KW-F16-	1	EGFR	0.095282	0.986
**10**	2-KW-K11-	0.1	EGFR	0.201979	0.258
**11**	2-KW-L11-	1	EGFR	0.258873	0.061
**12**	2-KW-L16-	0.25	EGFR	0.30601	0.163
**13**	2-KW-L19-	0.1	EGFR	0.360552	0.077
**14**	2-KW-M11-	10	EGFR	0.326766	0.066
**15**	2-KW-M16-	2.5	EGFR	0.346429	0.038
**16**	2-KW-M19-	1	EGFR	0.310967	0.118
**17**	2-KW-N16-	25	EGFR	0.335533	0.041
**18**	2-KW-N19-	10	EGFR	0.28251	0.234
**19**	2-KW-O11-	100	EGFR	0.481305	0
**20**	2-KW-O16-	250	EGFR	0.266585	0.33
**21**	2-KW-O19-	100	EGFR	0.280081	0.305
**22**	2-KW-P11-	1000	EGFR	0.398378	0.036
**23**	2-KW-P16-	2500	EGFR	0.387221	0.074

**24**	2-KW-P19-I	1000	EGFR	0.420534	0.004
**25**	3-KW-F21-I	10000	EGFR	0.351094	0.215
**26**	3-KW-G20-	1000	EGFR	0.351391	0.167
**27**	3-KW-G21-	1000	EGFR	0.322073	0.142
**28**	3-KW-H20-	100	EGFR	0.401953	0.001
**29**	3-KW-H21-	100	EGFR	0.299092	0.032
**30**	3-KW-I20-N	10	EGFR	0.383658	0.007
**31**	3-KW-I21-F	10	EGFR	0.321885	0.115
**32**	3-KW-J20-N	1	EGFR	0.163042	0.256
**33**	3-KW-J21-F	1	EGFR	0.233346	0.56
**34**	3-KW-K4-C	1	EGFR	0.217955	0.68
**35**	3-KW-K18-I	0.1	EGFR	0.323715	0.062
**36**	3-KW-K20-I	0.1	EGFR	0.31886	0.154
**37**	3-KW-L4-C	10	EGFR	0.236805	0.572
**38**	3-KW-L18-I	1	EGFR	0.268689	0.445
**39**	3-KW-M18-	10	EGFR	0.347966	0.023
**40**	3-KW-N4-C	100	EGFR	0.336631	0.041
**41**	3-KW-N18-	100	EGFR	0.4572	0
**42**	3-KW-O4-C	1000	EGFR	0.366307	0.107
**43**	3-KW-P4-C	10000	EGFR	0.294393	0.353
**44**	3-KW-P18-I	1000	EGFR	0.34051	0.036
**45**	4-KW-F13-S	1000	EGFR	0.354293	0.005
**46**	4-KW-G13-	100	EGFR	0.450509	0
**47**	4-KW-G16-	10000	EGFR	0.399947	0.009
**48**	4-KW-H13-	10	EGFR	0.099736	0.671
**49**	4-KW-H16-	1000	EGFR	0.116697	0.609
**50**	4-KW-I13-S	1	EGFR	0.42911	0
**51**	4-KW-I16-V	100	EGFR	0.112518	0.661
**52**	4-KW-J13-S	0.1	EGFR	0.363808	0.033
**53**	4-KW-J16-V	10	EGFR	0.055852	0.946
**54**	4-KW-K7-Ic	1	EGFR	0.28778	0.3
**55**	4-KW-K13-T	0.1	EGFR	0.189997	0.229
**56**	4-KW-K16-V	1	EGFR	0.370032	0.01
**57**	4-KW-L7-Ic	10	EGFR	0.163954	0.625
**58**	4-KW-L13-T	1	EGFR	0.1472	0.661

**59**	4-KW-M7-I	100	EGFR	0.380363	0.009
**60**	4-KW-M13-	10	EGFR	0.491877	0
**61**	4-KW-N13-	100	EGFR	0.435597	0
**62**	4-KW-O7-I	1000	EGFR	0.344376	0.092
**63**	4-KW-P7-I	10000	EGFR	0.39818	0.009
**64**	4-KW-P13-	1000	EGFR	0.374347	0.024
**65**	5-KW-F4-P	1000	EGFR	0.319491	0.106
**66**	5-KW-F7-A	1000	EGFR	0.384352	0.002
**67**	5-KW-G4-P	100	EGFR	0.346267	0.119
**68**	5-KW-G7-A	100	EGFR	0.336476	0.064
**69**	5-KW-H4-P	10	EGFR	0.282959	0.218
**70**	5-KW-H7-A	10	EGFR	0.245316	0.39
**71**	5-KW-I4-P	1	EGFR	0.263979	0.403
**72**	5-KW-I7-A	1	EGFR	0.195679	0.487
**73**	5-KW-J4-P	0.1	EGFR	0.276219	0.227
**74**	5-KW-J7-A	0.1	EGFR	0.326288	0.049
**75**	5-KW-K7-O	0.1	EGFR	0.291192	0.184
**76**	5-KW-L7-O	1	EGFR	0.407254	0.006
**77**	5-KW-M7-C	10	EGFR	0.417621	0.014
**78**	5-KW-O7-O	100	EGFR	0.334161	0.065
**79**	5-KW-P7-O	1000	EGFR	0.354306	0.024
**80**	2-KW-A15-	2500	VEGFR	0.388264	0.054
**81**	2-KW-A17-	10000	VEGFR	0.291308	0.598
**82**	2-KW-A20-	10000	VEGFR	0.369972	0.281
**83**	2-KW-B15-	250	VEGFR	0.383065	0.001
**84**	2-KW-B17-	1000	VEGFR	0.251165	0.311
**85**	2-KW-B20-	1000	VEGFR	0.202936	0.818
**86**	2-KW-C15-	25	VEGFR	0.187703	0.159
**87**	2-KW-C17-	100	VEGFR	0.387789	0
**88**	2-KW-D15-	2.5	VEGFR	0.193993	0.011
**89**	2-KW-D17-	10	VEGFR	0.311583	0.001
**90**	2-KW-D20-	100	VEGFR	0.260816	0.288
**91**	2-KW-E17-	1	VEGFR	0.24272	0.001
**92**	2-KW-E20-	10	VEGFR	0.165135	0.966
**93**	2-KW-F13-	10000	VEGFR	0.163057	0.985

**94**	2-KW-F15-I	0.25	VEGFR	0.223473	0.028
**95**	2-KW-F19-I	10000	VEGFR	0.150605	0.999
**96**	2-KW-F20-7	1	VEGFR	0.265948	0.228
**97**	2-KW-F21-\	10000	VEGFR	0.232154	0.245
**98**	2-KW-G10-	10000	VEGFR	0.472854	0
**99**	2-KW-G13-	1000	VEGFR	0.371286	0.051
**100**	2-KW-G19-	1000	VEGFR	0.281794	0.254
**101**	2-KW-G21-	1000	VEGFR	0.297856	0.162
**102**	2-KW-H10-	1000	VEGFR	0.469463	0
**103**	2-KW-H13-	100	VEGFR	0.411166	0
**104**	2-KW-H21-	100	VEGFR	0.211259	0.075
**105**	2-KW-I10-A	100	VEGFR	0.324017	0.013
**106**	2-KW-I13-A	10	VEGFR	0.517664	0
**107**	2-KW-I19-F	100	VEGFR	0.217328	0.9
**108**	2-KW-I21-\	10	VEGFR	0.207412	0.736
**109**	2-KW-J10-A	10	VEGFR	0.323813	0.01
**110**	2-KW-J13-A	1	VEGFR	0.359921	0.005
**111**	2-KW-J19-F	10	VEGFR	0.058266	0.999
**112**	2-KW-J21-\	1	VEGFR	0.301732	0.109
**113**	2-KW-K10-,	1	VEGFR	0.209305	0.69
**114**	2-KW-K13-'	0.1	VEGFR	0.293213	0.064
**115**	2-KW-K17-I	1	VEGFR	0.367023	0.001
**116**	2-KW-K19-I	1	VEGFR	0.322557	0.084
**117**	2-KW-L12-S	0.1	VEGFR	0.258522	0.57
**118**	2-KW-L13-\	1	VEGFR	0.188843	0.054
**119**	2-KW-L21-(	0.1	VEGFR	0.259393	0.409
**120**	2-KW-M12-	1	VEGFR	0.286924	0.144
**121**	2-KW-M13-	10	VEGFR	0.459178	0
**122**	2-KW-M17-	10	VEGFR	0.296459	0.11
**123**	2-KW-M21-	1	VEGFR	0.218056	0.598
**124**	2-KW-N12-	10	VEGFR	0.245582	0.628
**125**	2-KW-N13-	100	VEGFR	0.204094	0.751
**126**	2-KW-N17-	100	VEGFR	0.316949	0.073
**127**	2-KW-N21-	10	VEGFR	0.290878	0.171
**128**	2-KW-O12-	100	VEGFR	0.229233	0.722

**129**	2-KW-O17-	1000	VEGFR	0.254465	0.451
**130**	2-KW-O21-	100	VEGFR	0.198186	0.762
**131**	2-KW-P12-!	1000	VEGFR	0.376179	0.025
**132**	2-KW-P13-'	1000	VEGFR	0.238784	0.866
**133**	2-KW-P17-I	10000	VEGFR	0.356043	0.346
**134**	2-KW-P21-(	1000	VEGFR	0.350965	0.518
**135**	3-KW-A3-C	1000	VEGFR	0.342487	0.162
**136**	3-KW-A6-F	1000	VEGFR	0.253349	0.811
**137**	3-KW-A18-I	1000	VEGFR	0.300373	0
**138**	3-KW-B3-C	100	VEGFR	0.386629	0.004
**139**	3-KW-B6-F	100	VEGFR	0.370688	0.018
**140**	3-KW-B18-I	100	VEGFR	0.225496	0.028
**141**	3-KW-C3-C	10	VEGFR	0.180653	0.045
**142**	3-KW-C6-F	10	VEGFR	0.262631	0.186
**143**	3-KW-C18-I	10	VEGFR	0.214176	0.641
**144**	3-KW-D3-C	1	VEGFR	0.189993	0.921
**145**	3-KW-D6-F	1	VEGFR	0.270008	0.086
**146**	3-KW-D18-	1	VEGFR	0.282024	0.232
**147**	3-KW-E3-C	0.1	VEGFR	0.263493	0.345
**148**	3-KW-E6-F	0.1	VEGFR	0.404111	0
**149**	3-KW-E18-I	0.1	VEGFR	0.23025	0.004
**150**	3-KW-F18-I	1000	VEGFR	0.360403	0
**151**	3-KW-G18-	100	VEGFR	0.342204	0.041
**152**	3-KW-H18-	10	VEGFR	0.240029	0.543
**153**	3-KW-I18-E	1	VEGFR	0.357606	0
**154**	3-KW-J18-E	0.1	VEGFR	0.273741	0.214
**155**	4-KW-A12-I	10000	VEGFR	0.334486	0.386
**156**	4-KW-A15-(	2500	VEGFR	0.39311	0.126
**157**	4-KW-A20-I	10000	VEGFR	0.227293	0.854
**158**	4-KW-B12-I	1000	VEGFR	0.328889	0.002
**159**	4-KW-B15-(	250	VEGFR	0.364824	0.001
**160**	4-KW-B20-I	1000	VEGFR	0.225057	0.051
**161**	4-KW-C15-(	25	VEGFR	0.208349	0.145
**162**	4-KW-D12-	100	VEGFR	0.193384	0.946
**163**	4-KW-D15-	2.5	VEGFR	0.162689	0.102

**164**	4-KW-D20-	100	VEGFR	0.185724	0.076
**165**	4-KW-E12-I	10	VEGFR	0.292184	0
**166**	4-KW-E20-I	10	VEGFR	0.229834	0.055
**167**	4-KW-F12-I	1	VEGFR	0.231913	0.013
**168**	4-KW-F15-(	0.25	VEGFR	0.194855	0.11
**169**	4-KW-F20-I	1	VEGFR	0.224661	0.011
**170**	4-KW-L16-T	1	VEGFR	0.283676	0.266
**171**	4-KW-M16-	10	VEGFR	0.279417	0.034
**172**	4-KW-N16-	100	VEGFR	0.365952	0.018
**173**	4-KW-O16-	1000	VEGFR	0.113362	0.997
**174**	4-KW-P16-T	10000	VEGFR	0.166226	0.988
**175**	2-KW-L10-I	1	PI3K	0.14939	0.962
**176**	2-KW-M10-	10	PI3K	0.282278	0.032
**177**	2-KW-N10-	100	PI3K	0.235594	0.24
**178**	2-KW-O10-	1000	PI3K	0.43061	0.001
**179**	2-KW-P10-I	10000	PI3K	0.320487	0.148
**180**	3-KW-A16-I	2500	PI3K	0.111758	0.234
**181**	3-KW-C16-I	250	PI3K	0.172618	0.857
**182**	3-KW-D16-	25	PI3K	0.152371	0.944
**183**	3-KW-E16-I	2.5	PI3K	0.087149	0.621
**184**	3-KW-F16-I	0.25	PI3K	0.092498	0.362
**185**	3-KW-F17-I	100000	PI3K	0.311875	0.222
**186**	3-KW-F19-I	500	PI3K	0.36333	0.005
**187**	3-KW-G17-	10000	PI3K	0.195574	0.576
**188**	3-KW-G19-	50	PI3K	0.252796	0.263
**189**	3-KW-H17-	1000	PI3K	0.190553	0.757
**190**	3-KW-I17-N	100	PI3K	0.279967	0.229
**191**	3-KW-I19-D	5	PI3K	0.184363	0.4
**192**	3-KW-J17-N	10	PI3K	0.248774	0.253
**193**	3-KW-J19-D	0.5	PI3K	0.251821	0.424
**194**	3-KW-K19-I	0.05	PI3K	0.097164	0.522
**195**	3-KW-L8-Pi	1	PI3K	0.302081	0.009
**196**	3-KW-L21-T	0.1	PI3K	0.152379	0.18
**197**	3-KW-M8-P	10	PI3K	0.197699	0.16
**198**	3-KW-M21-	1	PI3K	0.186715	0.799

**199**	3-KW-N8-P	100	PI3K	0.373092	0.008
**200**	3-KW-N21-	10	PI3K	0.179521	0.855
**201**	3-KW-O8-P	1000	PI3K	0.334662	0.079
**202**	3-KW-O21-	100	PI3K	0.394953	0.006
**203**	3-KW-P8-Pi	10000	PI3K	0.321128	0.206
**204**	3-KW-P21-T	1000	PI3K	0.418086	0.008
**205**	4-KW-A19-,	2500	PI3K	0.370214	0.003
**206**	4-KW-B19-,	250	PI3K	0.216481	0.675
**207**	4-KW-C19-,	25	PI3K	0.110187	0.246
**208**	4-KW-D19-	2.5	PI3K	0.087247	0.355
**209**	4-KW-E19-/	0.25	PI3K	0.303034	0.02
**210**	4-KW-F14-I	1000	PI3K	0.34993	0.11
**211**	4-KW-G2-T(	2500	PI3K	0.264498	0.112
**212**	4-KW-G5-S(	10000	PI3K	0.334874	0.013
**213**	4-KW-G14-	100	PI3K	0.415098	0.001
**214**	4-KW-G20-	10000	PI3K	0.33994	0.177
**215**	4-KW-H2-T(	250	PI3K	0.108621	0.305
**216**	4-KW-H5-S(	1000	PI3K	0.124995	0.223
**217**	4-KW-H14-	10	PI3K	0.275023	0.058
**218**	4-KW-H20-	1000	PI3K	0.31623	0.009
**219**	4-KW-I2-TG	25	PI3K	0.16595	0.743
**220**	4-KW-I5-So	100	PI3K	0.181873	0.58
**221**	4-KW-I14-N	1	PI3K	0.308222	0.035
**222**	4-KW-I20-E	100	PI3K	0.301354	0.039
**223**	4-KW-J2-TG	2.5	PI3K	0.161944	0.899
**224**	4-KW-J5-Sc	10	PI3K	0.279335	0.239
**225**	4-KW-J20-E	10	PI3K	0.25579	0.264
**226**	4-KW-K2-T(	0.25	PI3K	0.205712	0.693
**227**	4-KW-K4-D	0.1	PI3K	0.23668	0.531
**228**	4-KW-K5-S(	1	PI3K	0.286644	0.138
**229**	4-KW-K14-I	0.1	PI3K	0.37952	0
**230**	4-KW-K20-I	1	PI3K	0.33611	0.056
**231**	4-KW-L4-D:	1	PI3K	0.305248	0.09
**232**	4-KW-L14-(	0.1	PI3K	0.45659	0
**233**	4-KW-L15-T	1	PI3K	0.315951	0.011

**234**	4-KW-L21-(	0.1	PI3K	0.153805	0.153
**235**	4-KW-M14-	1	PI3K	0.48179	0
**236**	4-KW-M15-	10	PI3K	0.338869	0.001
**237**	4-KW-M21-	1	PI3K	0.292759	0.096
**238**	4-KW-N4-D	10	PI3K	0.310833	0.056
**239**	4-KW-N14-	10	PI3K	0.328597	0.019
**240**	4-KW-N15-	100	PI3K	0.426012	0
**241**	4-KW-N21-	10	PI3K	0.313027	0.083
**242**	4-KW-O4-D	100	PI3K	0.320219	0.048
**243**	4-KW-O14-	100	PI3K	0.380999	0.021
**244**	4-KW-O15-	1000	PI3K	0.274297	0.034
**245**	4-KW-O21-	100	PI3K	0.409868	0.002
**246**	4-KW-P4-D	1000	PI3K	0.344862	0.035
**247**	4-KW-P14-	1000	PI3K	0.367607	0.043
**248**	4-KW-P15-	10000	PI3K	0.369549	0.001
**249**	4-KW-P21-	1000	PI3K	0.380726	0.064
**250**	5-KW-A6-L	2500	PI3K	0.398995	0.003
**251**	5-KW-A7-A	1000	PI3K	0.178569	0.729
**252**	5-KW-A16-	2500	PI3K	0.357204	0.006
**253**	5-KW-A17-	10000	PI3K	0.243264	0.38
**254**	5-KW-B6-L	250	PI3K	0.334559	0.043
**255**	5-KW-B7-A	100	PI3K	0.087843	0.329
**256**	5-KW-B17-	1000	PI3K	0.178481	0.099
**257**	5-KW-C6-L	25	PI3K	0.216035	0.524
**258**	5-KW-C7-A	10	PI3K	0.082338	0.933
**259**	5-KW-C16-	250	PI3K	0.414304	0.003
**260**	5-KW-C17-	100	PI3K	0.326305	0.006
**261**	5-KW-D6-L	2.5	PI3K	0.169995	0.888
**262**	5-KW-D7-A	1	PI3K	0.093857	0.255
**263**	5-KW-D16-	25	PI3K	0.341506	0.017
**264**	5-KW-D17-	10	PI3K	0.141724	0.138
**265**	5-KW-E6-L	0.25	PI3K	0.203309	0.76
**266**	5-KW-E7-A	0.1	PI3K	0.200052	0.183
**267**	5-KW-E16-	2.5	PI3K	0.296114	0.043
**268**	5-KW-E17-	1	PI3K	0.287516	0.12

**269**	5-KW-F11-(	10000	PI3K	0.419949	0.001
**270**	5-KW-F16-/	0.25	PI3K	0.256336	0.343
**271**	5-KW-G9-S	10000	PI3K	0.371767	0.005
**272**	5-KW-G11-	1000	PI3K	0.419613	0
**273**	5-KW-H9-S	1000	PI3K	0.400375	0
**274**	5-KW-H11-	100	PI3K	0.377265	0.006
**275**	5-KW-I9-Se	100	PI3K	0.13495	0.288
**276**	5-KW-I11-G	10	PI3K	0.251835	0.028
**277**	5-KW-J9-Se	10	PI3K	0.447401	0
**278**	5-KW-J11-(	1	PI3K	0.151998	0.363
**279**	5-KW-K9-Se	1	PI3K	0.289325	0.076
**280**	5-KW-L14-/	0.1	PI3K	0.300689	0.039
**281**	5-KW-L20-2	1	PI3K	0.216626	0.592
**282**	5-KW-L23-(	0.1	PI3K	0.056068	0.995
**283**	5-KW-M14-	1	PI3K	0.193295	0.038
**284**	5-KW-M20-	10	PI3K	0.145372	0.953
**285**	5-KW-M23-	1	PI3K	0.165136	0.918
**286**	5-KW-N14-	10	PI3K	0.305505	0.077
**287**	5-KW-N20-	100	PI3K	0.333737	0.01
**288**	5-KW-N23-	10	PI3K	0.390549	0.002
**289**	5-KW-O14-	100	PI3K	0.35083	0.006
**290**	5-KW-O20-	1000	PI3K	0.389162	0.005
**291**	5-KW-O23-	100	PI3K	0.364447	0.02
**292**	5-KW-P14-/	1000	PI3K	0.382653	0
**293**	5-KW-P20-2	10000	PI3K	0.404303	0.002
**294**	5-KW-P23-(	1000	PI3K	0.356451	0.038
**295**	6-KW-A8-T(	10000	PI3K	0.331325	0.014
**296**	6-KW-B8-T(	1000	PI3K	0.184457	0.109
**297**	6-KW-C8-T(	100	PI3K	0.345536	0.091
**298**	6-KW-D8-T(	10	PI3K	0.221526	0.16
**299**	6-KW-E8-T(	1	PI3K	0.123954	0.23
**300**	6-KW-L6-G	1	PI3K	0.225339	0.617
**301**	6-KW-M6-G	10	PI3K	0.139496	0.582
**302**	6-KW-N6-G	100	PI3K	0.203017	0.734
**303**	6-KW-O6-G	1000	PI3K	0.348629	0.024

**304**	6-KW-P6-G	10000	PI3K	0.300376	0.33
**305**	1-KW-F11-	10000	Topoisome	0.644681	0
**306**	1-KW-G11-	1000	Topoisome	0.571465	0
**307**	1-KW-G20-	1000	Topoisome	0.611504	0
**308**	1-KW-H11-	100	Topoisome	0.618757	0
**309**	1-KW-H20-	100	Topoisome	0.612309	0
**310**	1-KW-I11-	10	Topoisome	0.329956	0.006
**311**	1-KW-I20-E	10	Topoisome	0.50268	0
**312**	1-KW-J11-	1	Topoisome	0.488638	0
**313**	1-KW-J20-E	1	Topoisome	0.160766	0.886
**314**	1-KW-K11-	1	Topoisome	0.643346	0
**315**	1-KW-K20-	0.1	Topoisome	0.267165	0.491
**316**	1-KW-L11-	10	Topoisome	0.65447	0
**317**	1-KW-L14-	1	Topoisome	0.545492	0
**318**	1-KW-M11	100	Topoisome	0.528976	0
**319**	1-KW-M14	10	Topoisome	0.639574	0
**320**	1-KW-N14-	100	Topoisome	0.632067	0
**321**	1-KW-O11-	1000	Topoisome	0.489653	0.004
**322**	1-KW-O14-	1000	Topoisome	0.630731	0
**323**	1-KW-P11-	10000	Topoisome	0.458031	0.017
**324**	1-KW-P14-	10000	Topoisome	0.478984	0.004
**325**	3-KW-A11-	10000	Topoisome	0.595487	0
**326**	3-KW-B11-	1000	Topoisome	0.632101	0
**327**	3-KW-C11-	100	Topoisome	0.198929	0.238
**328**	3-KW-D11-	10	Topoisome	0.153812	0.472
**329**	3-KW-E11-	1	Topoisome	0.574261	0
**330**	3-KW-G9-D	1000	Topoisome	0.462188	0.032
**331**	3-KW-G10-	10000	Topoisome	0.461934	0
**332**	3-KW-H9-D	100	Topoisome	0.604421	0
**333**	3-KW-H10-	1000	Topoisome	0.295645	0.277
**334**	3-KW-I9-Da	10	Topoisome	0.614804	0
**335**	3-KW-I10-T	100	Topoisome	0.168821	0.474
**336**	3-KW-J9-Da	1	Topoisome	0.164642	0.499
**337**	3-KW-J10-T	10	Topoisome	0.462572	0.003
**338**	3-KW-K7-Ic	0.1	Topoisome	0.162602	0.52

339	3-KW-K9-D	0.1	Topoisome	0.097948	0.837
340	3-KW-K10-	1	Topoisome	0.42471	0.005
341	3-KW-L6-D	0.1	Topoisome	0.27239	0.433
342	3-KW-L7-Id	1	Topoisome	0.335398	0.087
343	3-KW-L9-Va	0.5	Topoisome	0.494915	0
344	3-KW-L10-I	0.1	Topoisome	0.40166	0.038
345	3-KW-L16-I	1	Topoisome	0.238212	0.571
346	3-KW-M6-D	1	Topoisome	0.539763	0
347	3-KW-M7-I	10	Topoisome	0.215794	0.714
348	3-KW-M9-V	5	Topoisome	0.397247	0.036
349	3-KW-M10-	1	Topoisome	0.533011	0
350	3-KW-M16-	10	Topoisome	0.445951	0
351	3-KW-N6-D	10	Topoisome	0.250189	0.539
352	3-KW-N9-V	50	Topoisome	0.463621	0
353	3-KW-N10-	10	Topoisome	0.426572	0.001
354	3-KW-N16-	100	Topoisome	0.325635	0.155
355	3-KW-O6-D	100	Topoisome	0.57154	0
356	3-KW-O7-Ic	100	Topoisome	0.607239	0
357	3-KW-O9-V	500	Topoisome	0.56451	0
358	3-KW-O10-	100	Topoisome	0.551625	0
359	3-KW-O16-	1000	Topoisome	0.380664	0.039
360	3-KW-P6-D	1000	Topoisome	0.574736	0
361	3-KW-P7-Ic	1000	Topoisome	0.483894	0.008
362	3-KW-P9-V	5000	Topoisome	0.551594	0
363	3-KW-P10-I	1000	Topoisome	0.500072	0.002
364	3-KW-P16-I	10000	Topoisome	0.303895	0.294
365	1-KW-A10-	10000	Mitotic	0.672387	0
366	1-KW-A13-I	1000	Mitotic	0.618927	0
367	1-KW-A18-I	1000	Mitotic	0.610596	0
368	1-KW-B10-	1000	Mitotic	0.668443	0
369	1-KW-B13-I	100	Mitotic	0.61308	0
370	1-KW-B18-I	100	Mitotic	0.624725	0
371	1-KW-C10-	100	Mitotic	0.635609	0
372	1-KW-C13-I	10	Mitotic	0.606124	0
373	1-KW-C18-I	10	Mitotic	0.567519	0

**374**	1-KW-D10-	10	Mitotic	0.528084	0
**375**	1-KW-D13-	1	Mitotic	0.200547	0.487
**376**	1-KW-D18-	1	Mitotic	0.499621	0.003
**377**	1-KW-E10-\	1	Mitotic	0.241179	0.082
**378**	1-KW-E13-I	0.1	Mitotic	0.489611	0.001
**379**	1-KW-E18-I	0.1	Mitotic	0.415641	0.036
**380**	1-KW-F13-\	1000	Mitotic	0.663824	0
**381**	1-KW-G13-	100	Mitotic	0.179178	0.411
**382**	1-KW-G15-	1000	Mitotic	0.660719	0
**383**	1-KW-H13-	10	Mitotic	0.413792	0
**384**	1-KW-H15-	100	Mitotic	0.652549	0
**385**	1-KW-I13-\	1	Mitotic	0.463654	0.001
**386**	1-KW-I15-E	10	Mitotic	0.646148	0
**387**	1-KW-J13-\	0.1	Mitotic	0.558957	0.001
**388**	1-KW-J15-E	1	Mitotic	0.640363	0
**389**	1-KW-K7-Vi	0.1	Mitotic	0.600369	0
**390**	1-KW-K15-I	0.1	Mitotic	0.552001	0.001
**391**	1-KW-L7-Vi	1	Mitotic	0.328634	0.231
**392**	1-KW-L20-\	0.1	Mitotic	0.503876	0
**393**	1-KW-M7-\	10	Mitotic	0.585134	0
**394**	1-KW-M20-	1	Mitotic	0.453051	0.005
**395**	1-KW-N20-	10	Mitotic	0.485981	0
**396**	1-KW-O7-V	100	Mitotic	0.466926	0.006
**397**	1-KW-O20-	100	Mitotic	0.466852	0.002
**398**	1-KW-P7-Vi	1000	Mitotic	0.59832	0
**399**	1-KW-P20-\	1000	Mitotic	0.607089	0
**400**	3-KW-A7-D	1000	Mitotic	0.555983	0
**401**	3-KW-B7-D	100	Mitotic	0.682867	0
**402**	3-KW-C7-D	10	Mitotic	0.70239	0
**403**	3-KW-D7-D	1	Mitotic	0.083411	0.903
**404**	3-KW-E7-D	0.1	Mitotic	0.282897	0.386
**405**	6-KW-L19-/	1	Mitotic	0.390445	0.091
**406**	6-KW-M19-	10	Mitotic	0.238379	0.63
**407**	6-KW-N19-	100	Mitotic	0.280892	0.459
**408**	6-KW-O19-	1000	Mitotic	0.584941	0

**409**	6-KW-P19-/	10000	Mitotic	0.52365	0
**410**	2-KW-A12-1	250	MEK1/2	0.293585	0.816
**411**	2-KW-B12-1	25	MEK1/2	0.496686	0.011
**412**	2-KW-D12-	2.5	MEK1/2	0.409967	0.175
**413**	2-KW-E12-1	0.25	MEK1/2	0.463694	0
**414**	2-KW-F12-1	2.5000000	MEK1/2	0.363555	0.071
**415**	2-KW-F14-(	1000	MEK1/2	0.318785	0.626
**416**	2-KW-G14-	100	MEK1/2	0.472016	0.036
**417**	2-KW-H14-	10	MEK1/2	0.537045	0.001
**418**	2-KW-I14-C	1	MEK1/2	0.332719	0.15
**419**	2-KW-K14-(	0.1	MEK1/2	0.23016	0.819
**420**	2-KW-L20-S	1	MEK1/2	0.111789	1
**421**	2-KW-M20-	10	MEK1/2	0.10544	1
**422**	2-KW-N20-	100	MEK1/2	0.272602	0.678
**423**	2-KW-O20-	1000	MEK1/2	0.270271	0.772
**424**	2-KW-P20-S	10000	MEK1/2	0.311261	0.704
**425**	4-KW-A10-I	1000	MEK1/2	0.532458	0
**426**	4-KW-A13-I	1000	MEK1/2	0.535548	0.009
**427**	4-KW-B10-I	100	MEK1/2	0.407413	0.006
**428**	4-KW-B13-I	100	MEK1/2	0.395628	0.214
**429**	4-KW-C10-I	10	MEK1/2	0.484509	0.004
**430**	4-KW-C13-I	10	MEK1/2	0.638632	0
**431**	4-KW-D10-	1	MEK1/2	0.365007	0.017
**432**	4-KW-D13-	1	MEK1/2	0.408481	0.002
**433**	4-KW-E10-I	0.1	MEK1/2	0.406522	0.003
**434**	4-KW-E13-I	0.1	MEK1/2	0.372227	0.007
**435**	4-KW-L19-(	0.25	MEK1/2	0.245895	0.829
**436**	4-KW-M19-	2.5	MEK1/2	0.204151	0.862
**437**	4-KW-N19-	25	MEK1/2	0.350491	0.187
**438**	4-KW-O19-	250	MEK1/2	0.214094	0.878
**439**	4-KW-P19-(	2500	MEK1/2	0.558216	0.016
**440**	1-KW-L2-OI	1	PARP	0.523837	0.002
**441**	1-KW-L6-Ru	1	PARP	0.567092	0
**442**	1-KW-M2-C	10	PARP	0.636893	0
**443**	1-KW-M6-F	10	PARP	0.574751	0.001

**444**	1-KW-N2-O	100	PARP	0.499401	0.014
**445**	1-KW-N6-R	100	PARP	0.593053	0.001
**446**	1-KW-O2-O	1000	PARP	0.711228	0
**447**	1-KW-O6-R	1000	PARP	0.703008	0
**448**	1-KW-P2-O	10000	PARP	0.613693	0
**449**	1-KW-P6-R	10000	PARP	0.57706	0
**450**	7-KW-A3-Ta	1000	PARP	0.610768	0.001
**451**	7-KW-B2-V	10000	PARP	0.648179	0
**452**	7-KW-B3-Ta	100	PARP	0.582064	0.001
**453**	7-KW-C2-V	1000	PARP	0.631897	0
**454**	7-KW-C3-Ta	10	PARP	0.62363	0
**455**	7-KW-D2-V	100	PARP	0.587642	0
**456**	7-KW-D3-T	1	PARP	0.523195	0.01
**457**	7-KW-E2-Ve	10	PARP	0.612592	0.001
**458**	7-KW-E3-Ta	0.1	PARP	0.1537	0.773
**459**	7-KW-F2-Ve	1	PARP	0.67706	0
**460**	7-KW-G2-N	10000	PARP	0.611029	0
**461**	7-KW-H2-N	1000	PARP	0.597072	0.001
**462**	7-KW-I2-Ni	100	PARP	0.421754	0.242
**463**	7-KW-J2-Ni	10	PARP	0.611724	0
**464**	7-KW-K2-N	1	PARP	0.581681	0
**465**	3-KW-A19-	1000	CDK	0.411844	0.076
**466**	3-KW-B19-	100	CDK	0.412213	0.08
**467**	3-KW-B23-	2500	CDK	0.359586	0.078
**468**	3-KW-C19-	10	CDK	0.326058	0.09
**469**	3-KW-C23-	250	CDK	0.368889	0.007
**470**	3-KW-D19-	1	CDK	0.482253	0
**471**	3-KW-D23-	25	CDK	0.232265	0.589
**472**	3-KW-E19-	0.1	CDK	0.198303	0.081
**473**	3-KW-E23-	2.5	CDK	0.229128	0.683
**474**	3-KW-F23-	0.25	CDK	0.155202	0.93
**475**	3-KW-K17-	1	CDK	0.250277	0.185
**476**	3-KW-L19-	1	CDK	0.097318	0.941
**477**	3-KW-M17-	10	CDK	0.460938	0
**478**	3-KW-M19-	10	CDK	0.193344	0.84

479	3-KW-N17- 100	CDK	0.360564	0.052
480	3-KW-N19- 100	CDK	0.472791	0
481	3-KW-O17- 1000	CDK	0.322833	0.18
482	3-KW-O19- 1000	CDK	0.440901	0
483	3-KW-P17-I 10000	CDK	0.28239	0.324
484	3-KW-P19-I 10000	CDK	0.514229	0
485	4-KW-A4-SI 10000	CDK	0.42264	0.037
486	4-KW-A8-M 10000	CDK	0.446952	0.029
487	4-KW-B4-SI 1000	CDK	0.433357	0.036
488	4-KW-B8-M 1000	CDK	0.318702	0.029
489	4-KW-C4-SI 100	CDK	0.416844	0.009
490	4-KW-C8-M 100	CDK	0.206499	0.184
491	4-KW-D4-SI 10	CDK	0.242149	0.198
492	4-KW-D8-M 10	CDK	0.213551	0.228
493	4-KW-E4-SI 1	CDK	0.277532	0.07
494	4-KW-E8-M 1	CDK	0.176808	0.135
495	4-KW-F4-Se 10000	CDK	0.371041	0.047
496	4-KW-F22-/ 10000	CDK	0.423207	0.031
497	4-KW-G4-S 1000	CDK	0.176119	0.893
498	4-KW-G22- 1000	CDK	0.433491	0.025
499	4-KW-H4-S 100	CDK	0.172611	0.226
500	4-KW-H22- 100	CDK	0.286147	0.043
501	4-KW-I4-Se 10	CDK	0.152344	0.89
502	4-KW-I22-A 10	CDK	0.31504	0.006
503	4-KW-J4-Se 1	CDK	0.136128	0.969
504	4-KW-J22-/ 1	CDK	0.217775	0.164
505	5-KW-A19-, 10000	CDK	0.401734	0.129
506	5-KW-B19-, 1000	CDK	0.42003	0.053
507	5-KW-C19-, 100	CDK	0.293544	0.372
508	5-KW-D19- 10	CDK	0.153782	0.921
509	5-KW-E19-/ 1	CDK	0.369615	0.045
510	5-KW-K17-, 1	CDK	0.277144	0.366
511	5-KW-M17- 10	CDK	0.321968	0.167
512	5-KW-N17- 100	CDK	0.449251	0.011
513	5-KW-O17- 1000	CDK	0.410682	0.126

**514**	5-KW-P17-,	10000	CDK	0.401462	0.105
**515**	6-KW-A17-!	1000	CDK	0.278613	0.452
**516**	6-KW-B17-!	100	CDK	0.236373	0.297
**517**	6-KW-C17-!	10	CDK	0.233101	0.076
**518**	6-KW-D17-	1	CDK	0.269779	0.022
**519**	6-KW-E17-!	0.1	CDK	0.331309	0.158
**520**	6-KW-L15-"	1	CDK	0.247438	0.529
**521**	6-KW-M15-	10	CDK	0.291653	0.081
**522**	6-KW-N15-	100	CDK	0.391834	0.046
**523**	6-KW-O15-	1000	CDK	0.412619	0.069
**524**	6-KW-P15-"	10000	CDK	0.322372	0.345
**525**	7-KW-A21-	10000	BET	0.63494	0
**526**	7-KW-A22-I	30000	BET	0.691247	0
**527**	7-KW-B21-	1000	BET	0.195512	0.827
**528**	7-KW-B22-I	3000	BET	0.710641	0
**529**	7-KW-C21-	100	BET	0.351528	0.116
**530**	7-KW-C22-I	300	BET	0.231155	0.581
**531**	7-KW-D21-	10	BET	0.230874	0.401
**532**	7-KW-D22-	30	BET	0.42437	0.014
**533**	7-KW-E21-(	1	BET	0.389249	0.104
**534**	7-KW-E22-I	3	BET	0.374409	0.176
**535**	7-KW-G10-	10000	BET	0.629521	0
**536**	7-KW-G15-	10000	BET	0.712216	0
**537**	7-KW-H10-	1000	BET	0.68115	0
**538**	7-KW-H15-	1000	BET	0.701927	0
**539**	7-KW-I10-E	100	BET	0.54272	0
**540**	7-KW-I15-I	100	BET	0.459146	0.004
**541**	7-KW-J10-E	10	BET	0.449249	0.008
**542**	7-KW-J15-I	10	BET	0.46977	0.003
**543**	7-KW-K10-I	1	BET	0.434117	0.005
**544**	7-KW-K13-I	1	BET	0.526292	0
**545**	7-KW-K15-I	1	BET	0.478558	0.001
**546**	7-KW-L12-I	1	BET	0.483671	0.012
**547**	7-KW-L13-I	10	BET	0.545885	0
**548**	7-KW-L20-J	1	BET	0.542615	0

**549**	7-KW-L23-/	0.03	BET	0.387132	0.06
**550**	7-KW-M12-	10	BET	0.639143	0
**551**	7-KW-M13-	100	BET	0.656818	0
**552**	7-KW-M20-	10	BET	0.377415	0.067
**553**	7-KW-M23-	0.3	BET	0.433181	0.006
**554**	7-KW-N12-	100	BET	0.490685	0
**555**	7-KW-N13-	1000	BET	0.607392	0
**556**	7-KW-N20-	100	BET	0.554294	0
**557**	7-KW-N23-	3	BET	0.426148	0.004
**558**	7-KW-O12-	1000	BET	0.713231	0
**559**	7-KW-O20-	1000	BET	0.738162	0
**560**	7-KW-O23-	30	BET	0.526388	0
**561**	7-KW-P12-I	10000	BET	0.702881	0
**562**	7-KW-P13-I	10000	BET	0.56491	0
**563**	7-KW-P20-.	10000	BET	0.592298	0
**564**	7-KW-P23-,	300	BET	0.595212	0
**565**	8-KW-K22-(	1	BET	0.394448	0.057
**566**	8-KW-L22-(	10	BET	0.387231	0.041
**567**	8-KW-M22-	100	BET	0.352494	0.057
**568**	8-KW-N22-	1000	BET	0.601498	0
**569**	8-KW-O22-	10000	BET	0.578409	0
**570**	1-KW-A3-V	10000	HDAC	0.450859	0.002
**571**	1-KW-B3-V	1000	HDAC	0.487516	0
**572**	1-KW-C3-V	100	HDAC	0.177282	0.824
**573**	1-KW-D3-V	10	HDAC	0.042928	0.996
**574**	1-KW-E3-V	1	HDAC	0.080671	0.946
**575**	1-KW-L12-I	0.1	HDAC	0.377317	0
**576**	1-KW-M12-	1	HDAC	0.391071	0.002
**577**	1-KW-N12-	10	HDAC	0.465837	0
**578**	1-KW-O12-	100	HDAC	0.485221	0
**579**	1-KW-P12-I	1000	HDAC	0.485099	0
**580**	3-KW-A4-P	1000	HDAC	0.477953	0
**581**	3-KW-B4-P	100	HDAC	0.484783	0
**582**	3-KW-C4-P	10	HDAC	0.308876	0.044
**583**	3-KW-D4-P	1	HDAC	0.151225	0.965

**584**	3-KW-E4-Pa	0.1	HDAC	0.026642	0.997
**585**	3-KW-F7-Q	1000	HDAC	0.497201	0
**586**	3-KW-G7-Q	100	HDAC	0.455386	0
**587**	3-KW-G12-	1000000	HDAC	0.121065	0.979
**588**	3-KW-H7-Q	10	HDAC	0.42424	0
**589**	3-KW-H12-	100000	HDAC	0.26849	0.346
**590**	3-KW-I7-Qu	1	HDAC	0.406174	0
**591**	3-KW-I12-V	10000	HDAC	0.078312	0.744
**592**	3-KW-J7-Qu	0.1	HDAC	0.043694	0.881
**593**	3-KW-J12-V	1000	HDAC	0.199442	0.548
**594**	3-KW-K3-B	1	HDAC	0.099279	0.996
**595**	3-KW-K12-	100	HDAC	0.203188	0.807
**596**	3-KW-L3-Be	10	HDAC	0.304612	0.288
**597**	3-KW-M3-E	100	HDAC	0.357883	0.005
**598**	3-KW-N3-B	1000	HDAC	0.420363	0.01
**599**	3-KW-O3-B	10000	HDAC	0.474909	0
**600**	7-KW-A5-M	10000	HDAC	0.522749	0
**601**	7-KW-A7-C	10000	HDAC	0.503817	0
**602**	7-KW-A9-G	1000	HDAC	0.523401	0
**603**	7-KW-A12-	10000	HDAC	0.546931	0
**604**	7-KW-B5-M	1000	HDAC	0.520107	0
**605**	7-KW-B7-C	1000	HDAC	0.543213	0
**606**	7-KW-B12-	1000	HDAC	0.585535	0
**607**	7-KW-C5-M	100	HDAC	0.460114	0
**608**	7-KW-C7-C	100	HDAC	0.536382	0
**609**	7-KW-C9-G	100	HDAC	0.571129	0
**610**	7-KW-D7-C	10	HDAC	0.033734	1
**611**	7-KW-D9-G	10	HDAC	0.576483	0
**612**	7-KW-D12-	100	HDAC	0.519148	0
**613**	7-KW-E5-M	10	HDAC	0.350681	0.01
**614**	7-KW-E7-C	1	HDAC	0.382547	0
**615**	7-KW-E9-G	1	HDAC	0.179146	0.18
**616**	7-KW-E12-	10	HDAC	0.461192	0
**617**	7-KW-F5-M	1	HDAC	0.051734	0.951
**618**	7-KW-F7-R	10000	HDAC	0.564992	0

**619**	7-KW-F9-Gi	0.1	HDAC	0.072835	0.736
**620**	7-KW-F12-I	1	HDAC	0.500426	0
**621**	7-KW-F19-I	10000	HDAC	0.436472	0
**622**	7-KW-G7-R	1000	HDAC	0.503424	0
**623**	7-KW-G19-	1000	HDAC	0.411934	0.001
**624**	7-KW-H7-R	100	HDAC	0.545592	0
**625**	7-KW-I7-Re	10	HDAC	0.549054	0
**626**	7-KW-I19-P	100	HDAC	0.508512	0
**627**	7-KW-J7-Re	1	HDAC	0.536455	0
**628**	7-KW-J19-P	10	HDAC	0.058276	0.864
**629**	7-KW-K4-Er	1	HDAC	0.335898	0.018
**630**	7-KW-K11-,	1	HDAC	0.58244	0
**631**	7-KW-K18-'	1	HDAC	0.359392	0.003
**632**	7-KW-K19-I	1	HDAC	0.396414	0
**633**	7-KW-L2-Ta	0.1	HDAC	0.335812	0.026
**634**	7-KW-L4-Er	10	HDAC	0.460038	0
**635**	7-KW-L5-Pr	1	HDAC	0.515361	0
**636**	7-KW-L8-Al	1	HDAC	0.396614	0.001
**637**	7-KW-L10-'	1	HDAC	0.583989	0
**638**	7-KW-L11-/	10	HDAC	0.560193	0
**639**	7-KW-L14-'	1	HDAC	0.418255	0
**640**	7-KW-L16-I	1	HDAC	0.578917	0
**641**	7-KW-L18-'	10	HDAC	0.405843	0
**642**	7-KW-M2-T	1	HDAC	0.497317	0
**643**	7-KW-M5-P	10	HDAC	0.469929	0
**644**	7-KW-M8-A	10	HDAC	0.382782	0
**645**	7-KW-M10-	10	HDAC	0.238534	0.024
**646**	7-KW-M11-	100	HDAC	0.508883	0
**647**	7-KW-M14-	10	HDAC	0.43859	0
**648**	7-KW-M16-	10	HDAC	0.335695	0.076
**649**	7-KW-M18-	100	HDAC	0.639817	0
**650**	7-KW-N2-Ta	10	HDAC	0.280543	0.215
**651**	7-KW-N4-Er	100	HDAC	0.406486	0
**652**	7-KW-N5-P	100	HDAC	0.492572	0
**653**	7-KW-N8-A	100	HDAC	0.504208	0

**654**	7-KW-N10-	100	HDAC	0.474329	0
**655**	7-KW-N14-	100	HDAC	0.373767	0.001
**656**	7-KW-N16-	100	HDAC	0.43445	0
**657**	7-KW-N18-	1000	HDAC	0.366885	0.001
**658**	7-KW-O2-Ta	100	HDAC	0.585315	0
**659**	7-KW-O4-E	1000	HDAC	0.541732	0
**660**	7-KW-O5-P	1000	HDAC	0.506655	0
**661**	7-KW-O8-A	1000	HDAC	0.5206	0
**662**	7-KW-O10-	1000	HDAC	0.504888	0
**663**	7-KW-O11-	1000	HDAC	0.516828	0
**664**	7-KW-O14-	1000	HDAC	0.468555	0
**665**	7-KW-O16-	1000	HDAC	0.505527	0
**666**	7-KW-P2-Ta	1000	HDAC	0.481473	0
**667**	7-KW-P4-E	10000	HDAC	0.510513	0
**668**	7-KW-P5-P	10000	HDAC	0.537032	0
**669**	7-KW-P8-A	10000	HDAC	0.536751	0
**670**	7-KW-P10-	10000	HDAC	0.494862	0
**671**	7-KW-P11-	10000	HDAC	0.563743	0
**672**	7-KW-P14-	10000	HDAC	0.497198	0
**673**	7-KW-P16-	10000	HDAC	0.511904	0
**674**	7-KW-P18-	10000	HDAC	0.483671	0
**0**	2-MHB-A16	10000	EGFR	0.415395	0
**1**	2-MHB-A19	10000	EGFR	0.518737	0
**2**	2-MHB-B19	1000	EGFR	0.543465	0
**3**	2-MHB-C16	1000	EGFR	0.587569	0
**4**	2-MHB-C19	100	EGFR	0.537725	0
**5**	2-MHB-D16	100	EGFR	0.592316	0
**6**	2-MHB-D19	10	EGFR	0.040451	0.989
**7**	2-MHB-E16	10	EGFR	0.588391	0
**8**	2-MHB-E19	1	EGFR	0.065001	0.968
**9**	2-MHB-F16	1	EGFR	0.563832	0
**10**	2-MHB-K11	0.1	EGFR	0.453875	0
**11**	2-MHB-L11	1	EGFR	0.576058	0
**12**	2-MHB-L16	0.25	EGFR	0.060734	0.95
**13**	2-MHB-L19	0.1	EGFR	0.646042	0

**14**	2-MHB-M1	10	EGFR	0.603682	0
**15**	2-MHB-M1	2.5	EGFR	0.626126	0
**16**	2-MHB-M1	1	EGFR	0.660793	0
**17**	2-MHB-N1	25	EGFR	0.599585	0
**18**	2-MHB-N1	10	EGFR	0.610436	0
**19**	2-MHB-O11	100	EGFR	0.523567	0
**20**	2-MHB-O1	250	EGFR	0.541841	0
**21**	2-MHB-O1	100	EGFR	0.604981	0
**22**	2-MHB-P11	1000	EGFR	0.510561	0
**23**	2-MHB-P1	2500	EGFR	0.541324	0
**24**	2-MHB-P1	1000	EGFR	0.382458	0
**25**	3-MHB-F21	10000	EGFR	0.474898	0
**26**	3-MHB-G2	1000	EGFR	0.495425	0
**27**	3-MHB-G21	1000	EGFR	0.547898	0
**28**	3-MHB-H2	100	EGFR	0.563954	0
**29**	3-MHB-H21	100	EGFR	0.087338	0.983
**30**	3-MHB-I20-	10	EGFR	0.526904	0
**31**	3-MHB-I21-	10	EGFR	0.043237	0.971
**32**	3-MHB-J20	1	EGFR	0.026029	1
**33**	3-MHB-J21	1	EGFR	0.089839	0.866
**34**	3-MHB-K4-	1	EGFR	0.403866	0.002
**35**	3-MHB-K18	0.1	EGFR	0.44281	0
**36**	3-MHB-K2	0.1	EGFR	0.535938	0
**37**	3-MHB-L4-	10	EGFR	0.542417	0
**38**	3-MHB-L18	1	EGFR	0.509537	0
**39**	3-MHB-M1	10	EGFR	0.543349	0
**40**	3-MHB-N4-	100	EGFR	0.548918	0
**41**	3-MHB-N18	100	EGFR	0.57989	0
**42**	3-MHB-O4-	1000	EGFR	0.533196	0
**43**	3-MHB-P4-	10000	EGFR	0.499248	0
**44**	3-MHB-P18	1000	EGFR	0.520998	0
**45**	4-MHB-F13	1000	EGFR	0.512784	0
**46**	4-MHB-G1	100	EGFR	0.580266	0
**47**	4-MHB-G1	10000	EGFR	0.570796	0
**48**	4-MHB-H1	10	EGFR	0.572465	0

**49**	4-MHB-H16	1000	EGFR	0.523638	0
**50**	4-MHB-I13-	1	EGFR	0.556025	0
**51**	4-MHB-I16-	100	EGFR	0.577534	0
**52**	4-MHB-J13	0.1	EGFR	0.334244	0.006
**53**	4-MHB-J16	10	EGFR	0.081568	0.76
**54**	4-MHB-K7-I	1	EGFR	0.53173	0
**55**	4-MHB-K13	0.1	EGFR	0.511345	0
**56**	4-MHB-K16	1	EGFR	0.046548	0.937
**57**	4-MHB-L7-I	10	EGFR	0.578397	0
**58**	4-MHB-L13	1	EGFR	0.586312	0
**59**	4-MHB-M7-	100	EGFR	0.568478	0
**60**	4-MHB-M1	10	EGFR	0.589874	0
**61**	4-MHB-N13	100	EGFR	0.589162	0
**62**	4-MHB-O7-	1000	EGFR	0.579189	0
**63**	4-MHB-P7-I	10000	EGFR	0.568736	0
**64**	4-MHB-P13	1000	EGFR	0.504155	0
**65**	5-MHB-F4-I	1000	EGFR	0.506156	0
**66**	5-MHB-F7-/	1000	EGFR	0.582357	0
**67**	5-MHB-G4-	100	EGFR	0.52206	0
**68**	5-MHB-G7-	100	EGFR	0.568228	0
**69**	5-MHB-H4-	10	EGFR	0.535581	0
**70**	5-MHB-H7-	10	EGFR	0.555254	0
**71**	5-MHB-I4-F	1	EGFR	0.53332	0
**72**	5-MHB-I7-A	1	EGFR	0.049225	0.909
**73**	5-MHB-J4-F	0.1	EGFR	0.39181	0.014
**74**	5-MHB-J7-/	0.1	EGFR	0.585283	0
**75**	5-MHB-K7-	0.1	EGFR	0.509631	0
**76**	5-MHB-L7-(	1	EGFR	0.041529	0.945
**77**	5-MHB-M7-	10	EGFR	0.321234	0.111
**78**	5-MHB-O7-	100	EGFR	0.224358	0.469
**79**	5-MHB-P7-	1000	EGFR	0.506567	0
**80**	2-MHB-A15	2500	VEGFR	0.148606	0.837
**81**	2-MHB-A17	10000	VEGFR	0.156045	0.824
**82**	2-MHB-A2C	10000	VEGFR	0.096146	0.992
**83**	2-MHB-B15	250	VEGFR	0.155416	0.791

**84**	2-MHB-B17	1000	VEGFR	0.151847	0.841
**85**	2-MHB-B2(	1000	VEGFR	0.207427	0.695
**86**	2-MHB-C15	25	VEGFR	0.156122	0.797
**87**	2-MHB-C17	100	VEGFR	0.199576	0.751
**88**	2-MHB-D1!	2.5	VEGFR	0.232296	0.163
**89**	2-MHB-D17	10	VEGFR	0.079208	0.997
**90**	2-MHB-D2(	100	VEGFR	0.271209	0
**91**	2-MHB-E17	1	VEGFR	0.333634	0.003
**92**	2-MHB-E20	10	VEGFR	0.296117	0
**93**	2-MHB-F13	10000	VEGFR	0.240129	0.3
**94**	2-MHB-F15	0.25	VEGFR	0.299707	0
**95**	2-MHB-F19	10000	VEGFR	0.116474	0.959
**96**	2-MHB-F20	1	VEGFR	0.276335	0
**97**	2-MHB-F21	10000	VEGFR	0.213187	0.017
**98**	2-MHB-G1(	10000	VEGFR	0.224447	0.006
**99**	2-MHB-G1:	1000	VEGFR	0.205163	0.527
**100**	2-MHB-G1!	1000	VEGFR	0.205228	0.769
**101**	2-MHB-G21	1000	VEGFR	0.19613	0.143
**102**	2-MHB-H1(	1000	VEGFR	0.334069	0.001
**103**	2-MHB-H1:	100	VEGFR	0.257749	0.075
**104**	2-MHB-H21	100	VEGFR	0.29373	0.052
**105**	2-MHB-I10-	100	VEGFR	0.251033	0.004
**106**	2-MHB-I13-	10	VEGFR	0.273671	0.227
**107**	2-MHB-I19-	100	VEGFR	0.341884	0.038
**108**	2-MHB-I21-	10	VEGFR	0.29765	0
**109**	2-MHB-J10	10	VEGFR	0.293567	0
**110**	2-MHB-J13	1	VEGFR	0.405336	0
**111**	2-MHB-J19	10	VEGFR	0.297064	0.037
**112**	2-MHB-J21	1	VEGFR	0.258477	0.001
**113**	2-MHB-K1(	1	VEGFR	0.283741	0.001
**114**	2-MHB-K13	0.1	VEGFR	0.301881	0.017
**115**	2-MHB-K17	1	VEGFR	0.27963	0.059
**116**	2-MHB-K1!	1	VEGFR	0.245302	0.001
**117**	2-MHB-L12	0.1	VEGFR	0.327558	0.001
**118**	2-MHB-L13	1	VEGFR	0.203121	0.577

**119**	2-MHB-L21	0.1	VEGFR	0.243136	0.097
**120**	2-MHB-M1:	1	VEGFR	0.165637	0.7
**121**	2-MHB-M1:	10	VEGFR	0.328492	0
**122**	2-MHB-M1'	10	VEGFR	0.156814	0.821
**123**	2-MHB-M2:	1	VEGFR	0.21979	0.002
**124**	2-MHB-N12	10	VEGFR	0.324756	0
**125**	2-MHB-N13	100	VEGFR	0.132217	0.911
**126**	2-MHB-N17	100	VEGFR	0.292289	0
**127**	2-MHB-N21	10	VEGFR	0.244924	0.052
**128**	2-MHB-O12	100	VEGFR	0.175711	0.594
**129**	2-MHB-O17	1000	VEGFR	0.232976	0.029
**130**	2-MHB-O21	100	VEGFR	0.276291	0.001
**131**	2-MHB-P12	1000	VEGFR	0.156071	0.167
**132**	2-MHB-P13	1000	VEGFR	0.137237	0.928
**133**	2-MHB-P17	10000	VEGFR	0.195016	0.02
**134**	2-MHB-P21	1000	VEGFR	0.185015	0.82
**135**	3-MHB-A3-(	1000	VEGFR	0.194549	0.331
**136**	3-MHB-A6-I	1000	VEGFR	0.029904	1
**137**	3-MHB-A18	1000	VEGFR	0.151135	0.335
**138**	3-MHB-B3-(	100	VEGFR	0.126837	0.96
**139**	3-MHB-B6-I	100	VEGFR	0.16957	0.925
**140**	3-MHB-B18	100	VEGFR	0.090129	0.993
**141**	3-MHB-C3-(	10	VEGFR	0.130163	0.986
**142**	3-MHB-C6-I	10	VEGFR	0.182611	0.737
**143**	3-MHB-C18	10	VEGFR	0.062894	1
**144**	3-MHB-D3-	1	VEGFR	0.115344	0.88
**145**	3-MHB-D6-	1	VEGFR	0.206828	0.746
**146**	3-MHB-D18	1	VEGFR	0.154342	0.822
**147**	3-MHB-E3-(	0.1	VEGFR	0.102514	0.512
**148**	3-MHB-E6-I	0.1	VEGFR	0.267797	0.006
**149**	3-MHB-E18	0.1	VEGFR	0.131345	0.946
**150**	3-MHB-F18	1000	VEGFR	0.139551	0.965
**151**	3-MHB-G18	100	VEGFR	0.150547	0.218
**152**	3-MHB-H18	10	VEGFR	0.197946	0.017
**153**	3-MHB-I18-	1	VEGFR	0.167916	0.036

**154**	3-MHB-J18	0.1	VEGFR	0.200445	0.022
**155**	4-MHB-A12	10000	VEGFR	0.141175	0.957
**156**	4-MHB-A15	2500	VEGFR	0.168716	0.828
**157**	4-MHB-A2(	10000	VEGFR	0.168809	0.517
**158**	4-MHB-B12	1000	VEGFR	0.14482	0.92
**159**	4-MHB-B15	250	VEGFR	0.171802	0.846
**160**	4-MHB-B2(	1000	VEGFR	0.149275	0.91
**161**	4-MHB-C15	25	VEGFR	0.185251	0.768
**162**	4-MHB-D12	100	VEGFR	0.295632	0.055
**163**	4-MHB-D15	2.5	VEGFR	0.324478	0.057
**164**	4-MHB-D2(	100	VEGFR	0.14487	0.964
**165**	4-MHB-E12	10	VEGFR	0.280645	0
**166**	4-MHB-E20	10	VEGFR	0.112024	0.981
**167**	4-MHB-F12	1	VEGFR	0.367612	0
**168**	4-MHB-F15	0.25	VEGFR	0.168689	0.884
**169**	4-MHB-F20	1	VEGFR	0.124415	0.934
**170**	4-MHB-L16	1	VEGFR	0.243787	0.309
**171**	4-MHB-M1	10	VEGFR	0.230773	0.33
**172**	4-MHB-N16	100	VEGFR	0.143306	0.928
**173**	4-MHB-O16	1000	VEGFR	0.246549	0.391
**174**	4-MHB-P16	10000	VEGFR	0.180591	0.169
**175**	2-MHB-L10	1	PI3K	0.231399	0.157
**176**	2-MHB-M1	10	PI3K	0.05331	0.892
**177**	2-MHB-N1(	100	PI3K	0.133602	0.909
**178**	2-MHB-O1(	1000	PI3K	0.145994	0.89
**179**	2-MHB-P1(	10000	PI3K	0.132632	0.952
**180**	3-MHB-A16	2500	PI3K	0.231171	0.29
**181**	3-MHB-C16	250	PI3K	0.241444	0.273
**182**	3-MHB-D16	25	PI3K	0.069438	0.902
**183**	3-MHB-E16	2.5	PI3K	0.225068	0.297
**184**	3-MHB-F16	0.25	PI3K	0.196463	0.446
**185**	3-MHB-F17	100000	PI3K	0.233407	0.326
**186**	3-MHB-F19	500	PI3K	0.205848	0.45
**187**	3-MHB-G17	10000	PI3K	0.094132	0.418
**188**	3-MHB-G19	50	PI3K	0.146743	0.877

**189**	3-MHB-H17	1000	PI3K	0.060042	0.794
**190**	3-MHB-I17-	100	PI3K	0.125683	0.225
**191**	3-MHB-I19-	5	PI3K	0.23453	0.347
**192**	3-MHB-J17	10	PI3K	0.083863	0.521
**193**	3-MHB-J19	0.5	PI3K	0.33352	0.001
**194**	3-MHB-K19	0.05	PI3K	0.152908	0.135
**195**	3-MHB-L8-I	1	PI3K	0.254185	0.291
**196**	3-MHB-L21	0.1	PI3K	0.286438	0.021
**197**	3-MHB-M8-	10	PI3K	0.252721	0.168
**198**	3-MHB-M2	1	PI3K	0.199737	0.163
**199**	3-MHB-N8-	100	PI3K	0.208567	0.404
**200**	3-MHB-N21	10	PI3K	0.278509	0.042
**201**	3-MHB-O8-	1000	PI3K	0.225521	0.413
**202**	3-MHB-O21	100	PI3K	0.183893	0.712
**203**	3-MHB-P8-I	10000	PI3K	0.268897	0.133
**204**	3-MHB-P21	1000	PI3K	0.185161	0.721
**205**	4-MHB-A19	2500	PI3K	0.196992	0.643
**206**	4-MHB-B19	250	PI3K	0.188063	0.676
**207**	4-MHB-C19	25	PI3K	0.159528	0.178
**208**	4-MHB-D19	2.5	PI3K	0.260824	0.315
**209**	4-MHB-E19	0.25	PI3K	0.316225	0.035
**210**	4-MHB-G2-	2500	PI3K	0.137621	0.931
**211**	4-MHB-G5-	10000	PI3K	0.276639	0.077
**212**	4-MHB-G14	100	PI3K	0.230214	0.301
**213**	4-MHB-G20	10000	PI3K	0.229019	0.418
**214**	4-MHB-H2-	250	PI3K	0.303095	0.218
**215**	4-MHB-H5-	1000	PI3K	0.243993	0.186
**216**	4-MHB-H14	10	PI3K	0.214807	0.449
**217**	4-MHB-H20	1000	PI3K	0.232159	0.33
**218**	4-MHB-I2-T	25	PI3K	0.142379	0.309
**219**	4-MHB-I5-S	100	PI3K	0.248366	0.173
**220**	4-MHB-I14-	1	PI3K	0.103368	0.337
**221**	4-MHB-I20-	100	PI3K	0.269528	0.083
**222**	4-MHB-J2-T	2.5	PI3K	0.267036	0.13
**223**	4-MHB-J5-S	10	PI3K	0.22112	0.017

**224**	4-MHB-J20	10	PI3K	0.339816	0.003
**225**	4-MHB-K2-[illegible]	0.25	PI3K	0.232783	0.62
**226**	4-MHB-K4-[illegible]	0.1	PI3K	0.291107	0.004
**227**	4-MHB-K5-[illegible]	1	PI3K	0.206828	0.202
**228**	4-MHB-K14	0.1	PI3K	0.208036	0.09
**229**	4-MHB-K2C	1	PI3K	0.320253	0.002
**230**	4-MHB-L4-[illegible]	1	PI3K	0.142032	0.223
**231**	4-MHB-L14	0.1	PI3K	0.257795	0.084
**232**	4-MHB-L15	1	PI3K	0.109036	0.495
**233**	4-MHB-L21	0.1	PI3K	0.335465	0.003
**234**	4-MHB-M1[illegible]	1	PI3K	0.269832	0.004
**235**	4-MHB-M1[illegible]	10	PI3K	0.263982	0.094
**236**	4-MHB-M2[illegible]	1	PI3K	0.255931	0.008
**237**	4-MHB-N4-	10	PI3K	0.200398	0.598
**238**	4-MHB-N1[illegible]	10	PI3K	0.403374	0
**239**	4-MHB-N1[illegible]	100	PI3K	0.258774	0.116
**240**	4-MHB-N21	10	PI3K	0.24472	0.242
**241**	4-MHB-O4-	100	PI3K	0.216149	0.462
**242**	4-MHB-O1[illegible]	100	PI3K	0.150103	0.864
**243**	4-MHB-O1[illegible]	1000	PI3K	0.269561	0.065
**244**	4-MHB-O21	100	PI3K	0.243518	0.257
**245**	4-MHB-P4-[illegible]	1000	PI3K	0.194258	0.67
**246**	4-MHB-P14	1000	PI3K	0.191954	0.706
**247**	4-MHB-P15	10000	PI3K	0.197412	0.762
**248**	4-MHB-P21	1000	PI3K	0.192534	0.64
**249**	5-MHB-A6-[illegible]	2500	PI3K	0.412927	0
**250**	5-MHB-A7-[illegible]	1000	PI3K	0.243584	0.001
**251**	5-MHB-A16	2500	PI3K	0.137363	0.895
**252**	5-MHB-A17	10000	PI3K	0.196166	0.546
**253**	5-MHB-B6-[illegible]	250	PI3K	0.201479	0.505
**254**	5-MHB-B7-[illegible]	100	PI3K	0.2263	0.082
**255**	5-MHB-B17	1000	PI3K	0.224544	0.283
**256**	5-MHB-C6-[illegible]	25	PI3K	0.230015	0.052
**257**	5-MHB-C7-[illegible]	10	PI3K	0.205599	0.014
**258**	5-MHB-C16	250	PI3K	0.205141	0.011

**259**	5-MHB-C17	100	PI3K	0.20095	0.018
**260**	5-MHB-D6-	2.5	PI3K	0.303073	0.003
**261**	5-MHB-D7-	1	PI3K	0.33228	0.001
**262**	5-MHB-D16	25	PI3K	0.159111	0.189
**263**	5-MHB-D17	10	PI3K	0.135209	0.176
**264**	5-MHB-E6-I	0.25	PI3K	0.196288	0.016
**265**	5-MHB-E7-/	0.1	PI3K	0.234696	0.002
**266**	5-MHB-E16	2.5	PI3K	0.161594	0.032
**267**	5-MHB-E17	1	PI3K	0.228455	0
**268**	5-MHB-F11	10000	PI3K	0.230562	0.037
**269**	5-MHB-F16	0.25	PI3K	0.224545	0.004
**270**	5-MHB-G9-	10000	PI3K	0.205799	0.519
**271**	5-MHB-G11	1000	PI3K	0.22463	0.001
**272**	5-MHB-H9-	1000	PI3K	0.249234	0.383
**273**	5-MHB-H11	100	PI3K	0.250698	0.365
**274**	5-MHB-I9-S	100	PI3K	0.271626	0.036
**275**	5-MHB-I11-	10	PI3K	0.127829	0.806
**276**	5-MHB-J9-S	10	PI3K	0.252766	0
**277**	5-MHB-J11	1	PI3K	0.086452	0.998
**278**	5-MHB-K9-S	1	PI3K	0.221704	0.216
**279**	5-MHB-L14	0.1	PI3K	0.167551	0.046
**280**	5-MHB-L20	1	PI3K	0.286546	0.005
**281**	5-MHB-L23	0.1	PI3K	0.150453	0.201
**282**	5-MHB-M14	1	PI3K	0.18613	0.437
**283**	5-MHB-M20	10	PI3K	0.161851	0.039
**284**	5-MHB-M23	1	PI3K	0.197268	0.719
**285**	5-MHB-N14	10	PI3K	0.231457	0.182
**286**	5-MHB-N20	100	PI3K	0.35176	0.002
**287**	5-MHB-N23	10	PI3K	0.181006	0.701
**288**	5-MHB-O14	100	PI3K	0.292711	0.02
**289**	5-MHB-O20	1000	PI3K	0.218538	0.386
**290**	5-MHB-O23	100	PI3K	0.17527	0.76
**291**	5-MHB-P14	1000	PI3K	0.161819	0.922
**292**	5-MHB-P20	10000	PI3K	0.181828	0.745
**293**	5-MHB-P23	1000	PI3K	0.17162	0.744

**294**	6-MHB-A8-7	10000	PI3K	0.141708	0.918
**295**	6-MHB-B8-7	1000	PI3K	0.18635	0.709
**296**	6-MHB-C8-7	100	PI3K	0.08779	0.996
**297**	6-MHB-D8-	10	PI3K	0.226787	0.399
**298**	6-MHB-E8-7	1	PI3K	0.102187	0.436
**299**	6-MHB-L6-(	1	PI3K	0.138208	0.919
**300**	6-MHB-M6-	10	PI3K	0.227976	0.324
**301**	6-MHB-N6-	100	PI3K	0.152527	0.868
**302**	6-MHB-O6-	1000	PI3K	0.185765	0.743
**303**	6-MHB-P6-	10000	PI3K	0.273802	0.091
**304**	1-MHB-F11	10000	Topoisome	0.35413	0.036
**305**	1-MHB-G11	1000	Topoisome	0.512407	0
**306**	1-MHB-G2(	1000	Topoisome	0.36037	0.023
**307**	1-MHB-H11	100	Topoisome	0.538253	0
**308**	1-MHB-H2(	100	Topoisome	0.464342	0
**309**	1-MHB-I11-	10	Topoisome	0.116262	0.525
**310**	1-MHB-I20-	10	Topoisome	0.106006	0.586
**311**	1-MHB-J11	1	Topoisome	0.425816	0.002
**312**	1-MHB-J20	1	Topoisome	0.090287	0.631
**313**	1-MHB-K11	1	Topoisome	0.622133	0
**314**	1-MHB-K2(	0.1	Topoisome	0.224737	0.529
**315**	1-MHB-L11	10	Topoisome	0.497771	0
**316**	1-MHB-L14	1	Topoisome	0.507228	0
**317**	1-MHB-M1	100	Topoisome	0.374186	0.008
**318**	1-MHB-M1	10	Topoisome	0.486848	0
**319**	1-MHB-N14	100	Topoisome	0.547079	0
**320**	1-MHB-O11	1000	Topoisome	0.346753	0.052
**321**	1-MHB-O14	1000	Topoisome	0.414394	0.002
**322**	1-MHB-P11	10000	Topoisome	0.349453	0.036
**323**	1-MHB-P14	10000	Topoisome	0.335613	0.059
**324**	3-MHB-A11	10000	Topoisome	0.345953	0.025
**325**	3-MHB-B11	1000	Topoisome	0.38717	0.004
**326**	3-MHB-C11	100	Topoisome	0.397447	0.009
**327**	3-MHB-D11	10	Topoisome	0.597124	0
**328**	3-MHB-E11	1	Topoisome	0.430094	0

**329**	3-MHB-G9-	1000	Topoisome	0.362935	0.024
**330**	3-MHB-G1(	10000	Topoisome	0.526526	0
**331**	3-MHB-H9-	100	Topoisome	0.493114	0
**332**	3-MHB-H1(	1000	Topoisome	0.50804	0
**333**	3-MHB-I9-D	10	Topoisome	0.526406	0
**334**	3-MHB-I10-	100	Topoisome	0.562185	0
**335**	3-MHB-J9-D	1	Topoisome	0.124217	0.474
**336**	3-MHB-J10	10	Topoisome	0.131726	0.383
**337**	3-MHB-K7-I	0.1	Topoisome	0.362348	0.023
**338**	3-MHB-K9-I	0.1	Topoisome	0.402731	0.001
**339**	3-MHB-K1C	1	Topoisome	0.534539	0
**340**	3-MHB-L6-I	0.1	Topoisome	0.433169	0.007
**341**	3-MHB-L7-I	1	Topoisome	0.263336	0.227
**342**	3-MHB-L9-\	0.5	Topoisome	0.196661	0.332
**343**	3-MHB-L10	0.1	Topoisome	0.543689	0
**344**	3-MHB-L16	1	Topoisome	0.566145	0
**345**	3-MHB-M6-	1	Topoisome	0.482735	0.002
**346**	3-MHB-M7-	10	Topoisome	0.417361	0.002
**347**	3-MHB-M9-	5	Topoisome	0.408749	0.001
**348**	3-MHB-M1(	1	Topoisome	0.22333	0.211
**349**	3-MHB-M1(	10	Topoisome	0.492264	0
**350**	3-MHB-N6-	10	Topoisome	0.407599	0.018
**351**	3-MHB-N9-	50	Topoisome	0.512543	0
**352**	3-MHB-N1(	10	Topoisome	0.465387	0
**353**	3-MHB-N1E	100	Topoisome	0.127394	0.622
**354**	3-MHB-O6-	100	Topoisome	0.555037	0
**355**	3-MHB-O7-	100	Topoisome	0.536263	0
**356**	3-MHB-O9-	500	Topoisome	0.481051	0
**357**	3-MHB-O1(	100	Topoisome	0.540452	0
**358**	3-MHB-O1E	1000	Topoisome	0.414046	0
**359**	3-MHB-P6-I	1000	Topoisome	0.380654	0.012
**360**	3-MHB-P7-I	1000	Topoisome	0.350683	0.031
**361**	3-MHB-P9-\	5000	Topoisome	0.349649	0.034
**362**	3-MHB-P1C	1000	Topoisome	0.386201	0.005
**363**	3-MHB-P1E	10000	Topoisome	0.391006	0.005

**364**	1-MHB-A1C	10000	Mitotic	0.574776	0
**365**	1-MHB-A13	1000	Mitotic	0.56274	0
**366**	1-MHB-A18	1000	Mitotic	0.570697	0
**367**	1-MHB-B1C	1000	Mitotic	0.574341	0
**368**	1-MHB-B13	100	Mitotic	0.436164	0.019
**369**	1-MHB-B18	100	Mitotic	0.600817	0
**370**	1-MHB-C1C	100	Mitotic	0.592691	0
**371**	1-MHB-C13	10	Mitotic	0.114625	0.706
**372**	1-MHB-C18	10	Mitotic	0.512366	0
**373**	1-MHB-D1(	10	Mitotic	0.294462	0.454
**374**	1-MHB-D13	1	Mitotic	0.325991	0.095
**375**	1-MHB-D18	1	Mitotic	0.513072	0
**376**	1-MHB-E10	1	Mitotic	0.450567	0.005
**377**	1-MHB-E13	0.1	Mitotic	0.154016	0.518
**378**	1-MHB-E18	0.1	Mitotic	0.128937	0.68
**379**	1-MHB-F13	1000	Mitotic	0.527276	0
**380**	1-MHB-G13	100	Mitotic	0.560179	0
**381**	1-MHB-G15	1000	Mitotic	0.583482	0
**382**	1-MHB-H13	10	Mitotic	0.545016	0
**383**	1-MHB-H15	100	Mitotic	0.569671	0
**384**	1-MHB-I13-	1	Mitotic	0.195582	0.294
**385**	1-MHB-I15-	10	Mitotic	0.530869	0
**386**	1-MHB-J13	0.1	Mitotic	0.24845	0.387
**387**	1-MHB-J15	1	Mitotic	0.51002	0.001
**388**	1-MHB-K7-'	0.1	Mitotic	0.133585	0.562
**389**	1-MHB-K15	0.1	Mitotic	0.156628	0.519
**390**	1-MHB-L7-\	1	Mitotic	0.467809	0.001
**391**	1-MHB-L20	0.1	Mitotic	0.165494	0.47
**392**	1-MHB-M7-	10	Mitotic	0.408042	0.006
**393**	1-MHB-M2(	1	Mitotic	0.22921	0.187
**394**	1-MHB-N2(	10	Mitotic	0.32133	0.062
**395**	1-MHB-O7-	100	Mitotic	0.532773	0
**396**	1-MHB-O2(	100	Mitotic	0.142345	0.487
**397**	1-MHB-P7-'	1000	Mitotic	0.552311	0
**398**	1-MHB-P2C	1000	Mitotic	0.433691	0.004

**399**	3-MHB-A7-I	1000	Mitotic	0.427732	0.002
**400**	3-MHB-B7-I	100	Mitotic	0.450149	0.002
**401**	3-MHB-C7-I	10	Mitotic	0.444567	0.003
**402**	3-MHB-D7-	1	Mitotic	0.488368	0
**403**	3-MHB-E7-I	0.1	Mitotic	0.233707	0.613
**404**	6-MHB-L19	1	Mitotic	0.238195	0.263
**405**	6-MHB-M1	10	Mitotic	0.237001	0.244
**406**	6-MHB-N1	100	Mitotic	0.258522	0.573
**407**	6-MHB-O1	1000	Mitotic	0.471403	0.001
**408**	6-MHB-P1	10000	Mitotic	0.545034	0
**409**	2-MHB-A12	250	MEK1/2	0.671607	0
**410**	2-MHB-B12	25	MEK1/2	0.694014	0
**411**	2-MHB-D1	2.5	MEK1/2	0.681907	0
**412**	2-MHB-E12	0.25	MEK1/2	0.751405	0
**413**	2-MHB-F12	2.5000000	MEK1/2	0.705286	0
**414**	2-MHB-F14	1000	MEK1/2	0.604566	0
**415**	2-MHB-G1	100	MEK1/2	0.640627	0
**416**	2-MHB-H1	10	MEK1/2	0.696374	0
**417**	2-MHB-I14	1	MEK1/2	0.737426	0
**418**	2-MHB-K14	0.1	MEK1/2	0.716832	0
**419**	2-MHB-L20	1	MEK1/2	0.131205	0.931
**420**	2-MHB-M2	10	MEK1/2	0.633553	0
**421**	2-MHB-N2	100	MEK1/2	0.624644	0
**422**	2-MHB-O2	1000	MEK1/2	0.649475	0
**423**	2-MHB-P2	10000	MEK1/2	0.554183	0
**424**	4-MHB-A1	1000	MEK1/2	0.612399	0
**425**	4-MHB-A13	1000	MEK1/2	0.689079	0
**426**	4-MHB-B1	100	MEK1/2	0.680306	0
**427**	4-MHB-B13	100	MEK1/2	0.666902	0
**428**	4-MHB-C1	10	MEK1/2	0.678117	0
**429**	4-MHB-C13	10	MEK1/2	0.67472	0
**430**	4-MHB-D1	1	MEK1/2	0.683097	0
**431**	4-MHB-D1	1	MEK1/2	0.632832	0
**432**	4-MHB-E10	0.1	MEK1/2	0.801399	0
**433**	4-MHB-E13	0.1	MEK1/2	0.659606	0

**434**	4-MHB-L19	0.25	MEK1/2	0.676097	0
**435**	4-MHB-M1!	2.5	MEK1/2	0.097857	0.966
**436**	4-MHB-N1!	25	MEK1/2	0.714337	0
**437**	4-MHB-O1!	250	MEK1/2	0.684548	0
**438**	4-MHB-P1!	2500	MEK1/2	0.629482	0
**439**	1-MHB-L2-(	1	PARP	0.601037	0.009
**440**	1-MHB-L6-I	1	PARP	0.397769	0.13
**441**	1-MHB-M2-	10	PARP	0.17295	0.625
**442**	1-MHB-M6-	10	PARP	0.527589	0.018
**443**	1-MHB-N2-	100	PARP	0.575394	0.002
**444**	1-MHB-N6-	100	PARP	0.584953	0.001
**445**	1-MHB-O2-	1000	PARP	0.472826	0.023
**446**	1-MHB-O6-	1000	PARP	0.538789	0.033
**447**	1-MHB-P2-(	10000	PARP	0.457638	0.033
**448**	1-MHB-P6-I	10000	PARP	0.54485	0.004
**449**	7-MHB-A3-T	1000	PARP	0.45856	0.027
**450**	7-MHB-B2-V	10000	PARP	0.330085	0.075
**451**	7-MHB-B3-T	100	PARP	0.34687	0.233
**452**	7-MHB-C2-V	1000	PARP	0.313043	0.101
**453**	7-MHB-C3-T	10	PARP	0.234072	0.725
**454**	7-MHB-D2-	100	PARP	0.565394	0.002
**455**	7-MHB-D3-	1	PARP	0.54471	0.003
**456**	7-MHB-E2-V	10	PARP	0.543649	0.015
**457**	7-MHB-E3-T	0.1	PARP	0.512258	0.005
**458**	7-MHB-F2-V	1	PARP	0.523723	0.006
**459**	7-MHB-G2-	10000	PARP	0.388685	0.134
**460**	7-MHB-H2-	1000	PARP	0.472648	0.018
**461**	7-MHB-I2-N	100	PARP	0.358774	0.029
**462**	7-MHB-J2-N	10	PARP	0.590894	0.001
**463**	7-MHB-K2-I	1	PARP	0.423011	0.014
**464**	3-MHB-A1!	1000	CDK	0.350308	0.04
**465**	3-MHB-B1!	100	CDK	0.339713	0.066
**466**	3-MHB-B23	2500	CDK	0.38523	0.012
**467**	3-MHB-C1!	10	CDK	0.270201	0.268
**468**	3-MHB-C23	250	CDK	0.26957	0.281

**469**	3-MHB-D19	1	CDK	0.187564	0.425
**470**	3-MHB-D23	25	CDK	0.28766	0.231
**471**	3-MHB-E19	0.1	CDK	0.182334	0.829
**472**	3-MHB-E23	2.5	CDK	0.347544	0.236
**473**	3-MHB-F23	0.25	CDK	0.482476	0
**474**	3-MHB-K17	1	CDK	0.31981	0.158
**475**	3-MHB-L19	1	CDK	0.304828	0.138
**476**	3-MHB-M17	10	CDK	0.283871	0.244
**477**	3-MHB-M19	10	CDK	0.106373	0.504
**478**	3-MHB-N17	100	CDK	0.21217	0.493
**479**	3-MHB-N19	100	CDK	0.283167	0.269
**480**	3-MHB-O17	1000	CDK	0.219855	0.587
**481**	3-MHB-O19	1000	CDK	0.217558	0.384
**482**	3-MHB-P17	10000	CDK	0.217891	0.64
**483**	3-MHB-P19	10000	CDK	0.402029	0.054
**484**	4-MHB-A4-	10000	CDK	0.265096	0.359
**485**	4-MHB-A8-	10000	CDK	0.35186	0.04
**486**	4-MHB-B4-	1000	CDK	0.27767	0.332
**487**	4-MHB-B8-	1000	CDK	0.243781	0.395
**488**	4-MHB-C4-	100	CDK	0.191809	0.171
**489**	4-MHB-C8-	100	CDK	0.21653	0.552
**490**	4-MHB-D4-	10	CDK	0.220438	0.099
**491**	4-MHB-D8-	10	CDK	0.197062	0.779
**492**	4-MHB-E4-	1	CDK	0.203548	0.074
**493**	4-MHB-E8-	1	CDK	0.141879	0.803
**494**	4-MHB-F4-	10000	CDK	0.155513	0.912
**495**	4-MHB-F22	10000	CDK	0.342778	0.069
**496**	4-MHB-G4-	1000	CDK	0.234318	0.054
**497**	4-MHB-G22	1000	CDK	0.341741	0.057
**498**	4-MHB-H4-	100	CDK	0.22468	0.132
**499**	4-MHB-H22	100	CDK	0.344428	0.047
**500**	4-MHB-I4-S	10	CDK	0.260283	0.154
**501**	4-MHB-I22-	10	CDK	0.168918	0.461
**502**	4-MHB-J4-S	1	CDK	0.23932	0.273
**503**	4-MHB-J22	1	CDK	0.212503	0.49

**504**	5-MHB-A19	10000	CDK	0.329321	0.078
**505**	5-MHB-B19	1000	CDK	0.245464	0.385
**506**	5-MHB-C19	100	CDK	0.235371	0.634
**507**	5-MHB-D19	10	CDK	0.137188	0.353
**508**	5-MHB-E19	1	CDK	0.167854	0.405
**509**	5-MHB-K17	1	CDK	0.213583	0.443
**510**	5-MHB-M1	10	CDK	0.177743	0.635
**511**	5-MHB-N17	100	CDK	0.115505	0.579
**512**	5-MHB-O17	1000	CDK	0.301443	0.172
**513**	5-MHB-P17	10000	CDK	0.346614	0.039
**514**	6-MHB-A17	1000	CDK	0.240106	0.454
**515**	6-MHB-B17	100	CDK	0.164135	0.884
**516**	6-MHB-C17	10	CDK	0.142764	0.389
**517**	6-MHB-D17	1	CDK	0.110526	0.567
**518**	6-MHB-E17	0.1	CDK	0.157701	0.794
**519**	6-MHB-L15	1	CDK	0.258951	0.221
**520**	6-MHB-M1	10	CDK	0.131088	0.8
**521**	6-MHB-N15	100	CDK	0.253945	0.386
**522**	6-MHB-O15	1000	CDK	0.308266	0.143
**523**	6-MHB-P15	10000	CDK	0.304965	0.154
**524**	7-MHB-A21	10000	BET	0.574469	0
**525**	7-MHB-A22	30000	BET	0.588995	0
**526**	7-MHB-B21	1000	BET	0.450709	0.007
**527**	7-MHB-B22	3000	BET	0.561892	0
**528**	7-MHB-C21	100	BET	0.13533	0.455
**529**	7-MHB-C22	300	BET	0.136128	0.538
**530**	7-MHB-D21	10	BET	0.282303	0.04
**531**	7-MHB-D22	30	BET	0.24515	0.112
**532**	7-MHB-E21	1	BET	0.503841	0
**533**	7-MHB-E22	3	BET	0.371356	0.122
**534**	7-MHB-G10	10000	BET	0.614252	0
**535**	7-MHB-G15	10000	BET	0.605665	0
**536**	7-MHB-H10	1000	BET	0.599116	0
**537**	7-MHB-H15	1000	BET	0.594456	0
**538**	7-MHB-I10-	100	BET	0.506809	0

**539**	7-MHB-I15-	100	BET	0.250812	0.332
**540**	7-MHB-J10	10	BET	0.144525	0.347
**541**	7-MHB-J15	10	BET	0.544678	0
**542**	7-MHB-K1C	1	BET	0.12433	0.564
**543**	7-MHB-K13	1	BET	0.547763	0
**544**	7-MHB-K15	1	BET	0.51608	0
**545**	7-MHB-L12	1	BET	0.476587	0
**546**	7-MHB-L13	10	BET	0.603857	0
**547**	7-MHB-L20	1	BET	0.526166	0
**548**	7-MHB-L23	0.03	BET	0.525559	0.001
**549**	7-MHB-M1:	10	BET	0.336061	0.332
**550**	7-MHB-M1:	100	BET	0.571037	0
**551**	7-MHB-M2(	10	BET	0.351422	0.105
**552**	7-MHB-M2:	0.3	BET	0.197423	0.159
**553**	7-MHB-N12	100	BET	0.127633	0.931
**554**	7-MHB-N13	1000	BET	0.545322	0
**555**	7-MHB-N2(	100	BET	0.56763	0
**556**	7-MHB-N23	3	BET	0.198467	0.19
**557**	7-MHB-O12	1000	BET	0.586336	0
**558**	7-MHB-O2(	1000	BET	0.550346	0
**559**	7-MHB-O23	30	BET	0.516224	0
**560**	7-MHB-P12	10000	BET	0.540709	0
**561**	7-MHB-P13	10000	BET	0.506254	0.001
**562**	7-MHB-P2C	10000	BET	0.600221	0
**563**	7-MHB-P23	300	BET	0.587294	0
**564**	8-MHB-K22	1	BET	0.093802	0.939
**565**	8-MHB-L22	10	BET	0.238161	0.827
**566**	8-MHB-M2:	100	BET	0.208221	0.849
**567**	8-MHB-N22	1000	BET	0.410842	0.008
**568**	8-MHB-O22	10000	BET	0.429254	0.003
**569**	1-MHB-A3-'	10000	HDAC	0.266826	0.178
**570**	1-MHB-B3-'	1000	HDAC	0.275399	0.101
**571**	1-MHB-C3-'	100	HDAC	0.104723	0.462
**572**	1-MHB-D3-	10	HDAC	0.278969	0.109
**573**	1-MHB-E3-\	1	HDAC	0.294401	0.181

**574**	1-MHB-L12	0.1	HDAC	0.161543	0.108
**575**	1-MHB-M1	1	HDAC	0.27397	0.169
**576**	1-MHB-N12	10	HDAC	0.248994	0.346
**577**	1-MHB-O12	100	HDAC	0.12086	0.97
**578**	1-MHB-P12	1000	HDAC	0.273627	0.185
**579**	3-MHB-A4-	1000	HDAC	0.216481	0.534
**580**	3-MHB-B4-	100	HDAC	0.269906	0.149
**581**	3-MHB-C4-	10	HDAC	0.403304	0
**582**	3-MHB-D4-	1	HDAC	0.068823	0.798
**583**	3-MHB-E4-	0.1	HDAC	0.333986	0.006
**584**	3-MHB-F7-	1000	HDAC	0.282356	0.119
**585**	3-MHB-G7-	100	HDAC	0.27057	0.128
**586**	3-MHB-G12	1000000	HDAC	0.111286	0.264
**587**	3-MHB-H7-	10	HDAC	0.375306	0.002
**588**	3-MHB-H12	100000	HDAC	0.336573	0.01
**589**	3-MHB-I7-	1	HDAC	0.089267	0.531
**590**	3-MHB-I12-	10000	HDAC	0.224541	0.052
**591**	3-MHB-J7-	0.1	HDAC	0.186164	0.143
**592**	3-MHB-J12	1000	HDAC	0.200522	0.027
**593**	3-MHB-K3-	1	HDAC	0.309305	0.022
**594**	3-MHB-K12	100	HDAC	0.328795	0.038
**595**	3-MHB-L3-	10	HDAC	0.128832	0.407
**596**	3-MHB-M3-	100	HDAC	0.210013	0.504
**597**	3-MHB-N3-	1000	HDAC	0.265675	0.149
**598**	3-MHB-O3-	10000	HDAC	0.237562	0.448
**599**	7-MHB-A5-	10000	HDAC	0.363863	0.002
**600**	7-MHB-A7-	10000	HDAC	0.359529	0.001
**601**	7-MHB-A9-	1000	HDAC	0.378534	0
**602**	7-MHB-A12	10000	HDAC	0.399428	0
**603**	7-MHB-B5-	1000	HDAC	0.363754	0.003
**604**	7-MHB-B12	1000	HDAC	0.405295	0
**605**	7-MHB-C5-	100	HDAC	0.027631	0.999
**606**	7-MHB-C7-	100	HDAC	0.411236	0
**607**	7-MHB-C9-	100	HDAC	0.336735	0.005
**608**	7-MHB-D7-	10	HDAC	0.363643	0

**609**	7-MHB-D9-	10	HDAC	0.193558	0.028
**610**	7-MHB-D12	100	HDAC	0.162221	0.65
**611**	7-MHB-E5-I	10	HDAC	0.153364	0.268
**612**	7-MHB-E7-(	1	HDAC	0.190025	0.156
**613**	7-MHB-E9-(	1	HDAC	0.430471	0
**614**	7-MHB-E12	10	HDAC	0.146659	0.097
**615**	7-MHB-F5-I	1	HDAC	0.292462	0.033
**616**	7-MHB-F7-I	10000	HDAC	0.416501	0
**617**	7-MHB-F9-(	0.1	HDAC	0.433375	0
**618**	7-MHB-F12	1	HDAC	0.378632	0
**619**	7-MHB-F19	10000	HDAC	0.325633	0.007
**620**	7-MHB-G7-	1000	HDAC	0.375487	0
**621**	7-MHB-G19	1000	HDAC	0.160195	0.054
**622**	7-MHB-H7-	100	HDAC	0.42028	0
**623**	7-MHB-I7-F	10	HDAC	0.409083	0
**624**	7-MHB-I19-	100	HDAC	0.211096	0.044
**625**	7-MHB-J7-F	1	HDAC	0.118439	0.211
**626**	7-MHB-J19	10	HDAC	0.391488	0
**627**	7-MHB-K4-I	1	HDAC	0.262572	0.038
**628**	7-MHB-K11	1	HDAC	0.431261	0
**629**	7-MHB-K18	1	HDAC	0.440428	0
**630**	7-MHB-K19	1	HDAC	0.112668	0.275
**631**	7-MHB-L2-T	0.1	HDAC	0.170443	0.176
**632**	7-MHB-L4-I	10	HDAC	0.114169	0.99
**633**	7-MHB-L5-I	1	HDAC	0.221425	0.129
**634**	7-MHB-L8-/	1	HDAC	0.438606	0
**635**	7-MHB-L10	1	HDAC	0.357142	0
**636**	7-MHB-L11	10	HDAC	0.395841	0
**637**	7-MHB-L14	1	HDAC	0.421832	0.001
**638**	7-MHB-L16	1	HDAC	0.153234	0.135
**639**	7-MHB-L18	10	HDAC	0.129328	0.177
**640**	7-MHB-M2-	1	HDAC	0.34368	0.033
**641**	7-MHB-M5-	10	HDAC	0.284502	0.009
**642**	7-MHB-M8-	10	HDAC	0.415244	0
**643**	7-MHB-M1(	10	HDAC	0.402069	0

**644**	7-MHB-M1	100	HDAC	0.43573	0
**645**	7-MHB-M1	10	HDAC	0.479643	0
**646**	7-MHB-M1	10	HDAC	0.374133	0
**647**	7-MHB-M1	100	HDAC	0.385862	0.011
**648**	7-MHB-N2-	10	HDAC	0.36104	0.018
**649**	7-MHB-N4-	100	HDAC	0.363033	0.001
**650**	7-MHB-N5-	100	HDAC	0.467078	0
**651**	7-MHB-N8-	100	HDAC	0.350599	0
**652**	7-MHB-N1	100	HDAC	0.395545	0
**653**	7-MHB-N1	100	HDAC	0.428601	0
**654**	7-MHB-N1	100	HDAC	0.282499	0.037
**655**	7-MHB-N1	1000	HDAC	0.134416	0.168
**656**	7-MHB-O2-	100	HDAC	0.443774	0.001
**657**	7-MHB-O4-	1000	HDAC	0.293635	0.074
**658**	7-MHB-O5-	1000	HDAC	0.389702	0.001
**659**	7-MHB-O8-	1000	HDAC	0.368275	0.001
**660**	7-MHB-O1	1000	HDAC	0.428566	0
**661**	7-MHB-O1	1000	HDAC	0.338768	0.005
**662**	7-MHB-O1	1000	HDAC	0.421348	0
**663**	7-MHB-O1	1000	HDAC	0.148183	0.624
**664**	7-MHB-P2-	1000	HDAC	0.316279	0.054
**665**	7-MHB-P4-	10000	HDAC	0.385546	0.001
**666**	7-MHB-P5-	10000	HDAC	0.379437	0
**667**	7-MHB-P8-	10000	HDAC	0.366731	0.002
**668**	7-MHB-P1	10000	HDAC	0.344734	0.009
**669**	7-MHB-P1	10000	HDAC	0.367632	0.004
**670**	7-MHB-P14	10000	HDAC	0.399509	0.012
**671**	7-MHB-P1	10000	HDAC	0.433295	0
**672**	7-MHB-P1	10000	HDAC	0.437645	0
**0**	2-O3B-A16	10000	EGFR	0.50015	0
**1**	2-O3B-A19	10000	EGFR	0.521851	0
**2**	2-O3B-B19	1000	EGFR	0.615813	0
**3**	2-O3B-C16	1000	EGFR	0.619047	0
**4**	2-O3B-C19	100	EGFR	0.459813	0
**5**	2-O3B-D16	100	EGFR	0.622486	0

**6**	2-O3B-D19	10	EGFR	0.120112	0.951
**7**	2-O3B-E16-	10	EGFR	0.421641	0.022
**8**	2-O3B-E19-	1	EGFR	0.155325	0.893
**9**	2-O3B-F16-	1	EGFR	0.369791	0.026
**10**	2-O3B-K11-	0.1	EGFR	0.502312	0
**11**	2-O3B-L11-	1	EGFR	0.467732	0
**12**	2-O3B-L16-	0.25	EGFR	0.483486	0
**13**	2-O3B-L19-	0.1	EGFR	0.462351	0
**14**	2-O3B-M11	10	EGFR	0.670587	0
**15**	2-O3B-M16	2.5	EGFR	0.477477	0
**16**	2-O3B-M19	1	EGFR	0.20337	0.779
**17**	2-O3B-N16	25	EGFR	0.488673	0
**18**	2-O3B-N19	10	EGFR	0.58666	0
**19**	2-O3B-O11	100	EGFR	0.627529	0
**20**	2-O3B-O16	250	EGFR	0.531853	0
**21**	2-O3B-O19	100	EGFR	0.626208	0
**22**	2-O3B-P11-	1000	EGFR	0.592366	0
**23**	2-O3B-P16-	2500	EGFR	0.541537	0
**24**	2-O3B-P19-	1000	EGFR	0.622021	0
**25**	3-O3B-F21-	10000	EGFR	0.525751	0
**26**	3-O3B-G20	1000	EGFR	0.489108	0
**27**	3-O3B-G21	1000	EGFR	0.494312	0
**28**	3-O3B-H20	100	EGFR	0.590155	0
**29**	3-O3B-H21	100	EGFR	0.222445	0.178
**30**	3-O3B-I20-	10	EGFR	0.577108	0
**31**	3-O3B-I21-	10	EGFR	0.576285	0
**32**	3-O3B-J20-	1	EGFR	0.212674	0.322
**33**	3-O3B-J21-	1	EGFR	0.103929	0.789
**34**	3-O3B-K4-(	1	EGFR	0.118624	0.977
**35**	3-O3B-K18-	0.1	EGFR	0.513087	0
**36**	3-O3B-K20-	0.1	EGFR	0.327205	0.029
**37**	3-O3B-L4-C	10	EGFR	0.484456	0
**38**	3-O3B-L18-	1	EGFR	0.046116	0.998
**39**	3-O3B-M18	10	EGFR	0.512933	0
**40**	3-O3B-N4-(	100	EGFR	0.300769	0.117

**41**	3-O3B-N18	100	EGFR	0.552188	0
**42**	3-O3B-O4-(	1000	EGFR	0.511778	0
**43**	3-O3B-P4-(	10000	EGFR	0.436576	0.004
**44**	3-O3B-P18-	1000	EGFR	0.559705	0
**45**	4-O3B-F13-	1000	EGFR	0.583786	0
**46**	4-O3B-G13	100	EGFR	0.645408	0
**47**	4-O3B-G16	10000	EGFR	0.502536	0
**48**	4-O3B-H13	10	EGFR	0.597721	0
**49**	4-O3B-H16	1000	EGFR	0.641802	0
**50**	4-O3B-I13-!	1	EGFR	0.227519	0.103
**51**	4-O3B-I16-'	100	EGFR	0.58099	0
**52**	4-O3B-J13-	0.1	EGFR	0.460156	0
**53**	4-O3B-J16-	10	EGFR	0.096093	0.609
**54**	4-O3B-K7-I(	1	EGFR	0.489383	0
**55**	4-O3B-K13-	0.1	EGFR	0.524036	0
**56**	4-O3B-K16-	1	EGFR	0.467597	0
**57**	4-O3B-L7-I(	10	EGFR	0.540468	0
**58**	4-O3B-L13-	1	EGFR	0.569633	0
**59**	4-O3B-M7-I	100	EGFR	0.495861	0
**60**	4-O3B-M13	10	EGFR	0.601772	0
**61**	4-O3B-N13	100	EGFR	0.644634	0
**62**	4-O3B-O7-I	1000	EGFR	0.525742	0
**63**	4-O3B-P7-I(	10000	EGFR	0.629103	0
**64**	4-O3B-P13-	1000	EGFR	0.46812	0
**65**	5-O3B-F4-P	1000	EGFR	0.515243	0
**66**	5-O3B-F7-A	1000	EGFR	0.634583	0
**67**	5-O3B-G4-F	100	EGFR	0.596024	0
**68**	5-O3B-G7-/	100	EGFR	0.488666	0
**69**	5-O3B-H4-F	10	EGFR	0.668625	0
**70**	5-O3B-H7-/	10	EGFR	0.148499	0.573
**71**	5-O3B-I4-P(	1	EGFR	0.533067	0
**72**	5-O3B-I7-A:	1	EGFR	0.359941	0.033
**73**	5-O3B-J4-P	0.1	EGFR	0.340265	0.177
**74**	5-O3B-J7-A	0.1	EGFR	0.234069	0.669
**75**	5-O3B-K7-(	0.1	EGFR	0.272565	0.21

**76**	5-O3B-L7-C	1	EGFR	0.190024	0.379
**77**	5-O3B-M7-	10	EGFR	0.566641	0
**78**	5-O3B-O7-C	100	EGFR	0.567216	0
**79**	5-O3B-P7-C	1000	EGFR	0.586693	0
**80**	2-O3B-A15-	2500	VEGFR	0.521119	0
**81**	2-O3B-A17-	10000	VEGFR	0.255981	0.593
**82**	2-O3B-A20-	10000	VEGFR	0.238428	0.751
**83**	2-O3B-B15-	250	VEGFR	0.435743	0
**84**	2-O3B-B17-	1000	VEGFR	0.310601	0.144
**85**	2-O3B-B20-	1000	VEGFR	0.459078	0
**86**	2-O3B-C15-	25	VEGFR	0.102759	0.961
**87**	2-O3B-C17-	100	VEGFR	0.47793	0
**88**	2-O3B-D15	2.5	VEGFR	0.404714	0.001
**89**	2-O3B-D17	10	VEGFR	0.431266	0
**90**	2-O3B-D20	100	VEGFR	0.409513	0.001
**91**	2-O3B-E17-	1	VEGFR	0.462754	0
**92**	2-O3B-E20-	10	VEGFR	0.435713	0
**93**	2-O3B-F13-	10000	VEGFR	0.286845	0.354
**94**	2-O3B-F15-	0.25	VEGFR	0.572709	0
**95**	2-O3B-F19-	10000	VEGFR	0.239428	0.721
**96**	2-O3B-F20-	1	VEGFR	0.424919	0
**97**	2-O3B-F21-	10000	VEGFR	0.200205	0.808
**98**	2-O3B-G10	10000	VEGFR	0.439351	0
**99**	2-O3B-G13	1000	VEGFR	0.54824	0
**100**	2-O3B-G19	1000	VEGFR	0.454546	0
**101**	2-O3B-G21	1000	VEGFR	0.390075	0
**102**	2-O3B-H10	1000	VEGFR	0.328059	0.004
**103**	2-O3B-H13	100	VEGFR	0.520301	0
**104**	2-O3B-H21	100	VEGFR	0.284021	0.069
**105**	2-O3B-I10-	100	VEGFR	0.380295	0.008
**106**	2-O3B-I13-	10	VEGFR	0.434682	0
**107**	2-O3B-I19-	100	VEGFR	0.388168	0
**108**	2-O3B-I21-	10	VEGFR	0.403985	0.001
**109**	2-O3B-J10-	10	VEGFR	0.386957	0.001
**110**	2-O3B-J13-	1	VEGFR	0.167778	0.905

**111**	2-O3B-J19-	10	VEGFR	0.390121	0
**112**	2-O3B-J21-	1	VEGFR	0.426825	0
**113**	2-O3B-K10-	1	VEGFR	0.37801	0
**114**	2-O3B-K13-	0.1	VEGFR	0.531035	0
**115**	2-O3B-K17-	1	VEGFR	0.109382	0.912
**116**	2-O3B-K19-	1	VEGFR	0.375115	0.005
**117**	2-O3B-L12-	0.1	VEGFR	0.35221	0.001
**118**	2-O3B-L13-	1	VEGFR	0.36461	0.043
**119**	2-O3B-L21-	0.1	VEGFR	0.474019	0
**120**	2-O3B-M12	1	VEGFR	0.523192	0
**121**	2-O3B-M13	10	VEGFR	0.284875	0.002
**122**	2-O3B-M17	10	VEGFR	0.391729	0.018
**123**	2-O3B-M21	1	VEGFR	0.318598	0.033
**124**	2-O3B-N12	10	VEGFR	0.026685	1
**125**	2-O3B-N13	100	VEGFR	0.144049	0.883
**126**	2-O3B-N17	100	VEGFR	0.198768	0.645
**127**	2-O3B-N21	10	VEGFR	0.072456	0.787
**128**	2-O3B-O12	100	VEGFR	0.230374	0.006
**129**	2-O3B-O17	1000	VEGFR	0.445692	0
**130**	2-O3B-O21	100	VEGFR	0.363281	0.005
**131**	2-O3B-P12-	1000	VEGFR	0.422608	0
**132**	2-O3B-P13-	1000	VEGFR	0.198273	0.868
**133**	2-O3B-P17-	10000	VEGFR	0.310352	0.168
**134**	2-O3B-P21-	1000	VEGFR	0.446187	0
**135**	3-O3B-A3-C	1000	VEGFR	0.366417	0.07
**136**	3-O3B-A6-F	1000	VEGFR	0.24082	0.707
**137**	3-O3B-A18-	1000	VEGFR	0.39294	0.001
**138**	3-O3B-B3-C	100	VEGFR	0.422471	0
**139**	3-O3B-B6-F	100	VEGFR	0.489699	0
**140**	3-O3B-B18-	100	VEGFR	0.3942	0
**141**	3-O3B-C3-C	10	VEGFR	0.127657	0.974
**142**	3-O3B-C6-F	10	VEGFR	0.416971	0
**143**	3-O3B-C18-	10	VEGFR	0.285994	0.048
**144**	3-O3B-D3-C	1	VEGFR	0.09703	0.656
**145**	3-O3B-D6-F	1	VEGFR	0.099718	0.699

**146**	3-O3B-D18	1	VEGFR	0.193629	0.143
**147**	3-O3B-E3-C	0.1	VEGFR	0.383691	0.002
**148**	3-O3B-E6-F	0.1	VEGFR	0.286894	0.005
**149**	3-O3B-E18-	0.1	VEGFR	0.221178	0.382
**150**	3-O3B-F18-	1000	VEGFR	0.120585	0.952
**151**	3-O3B-G18	100	VEGFR	0.292488	0.029
**152**	3-O3B-H18	10	VEGFR	0.114542	0.737
**153**	3-O3B-I18-I	1	VEGFR	0.12842	0.72
**154**	3-O3B-J18-	0.1	VEGFR	0.151826	0.802
**155**	4-O3B-A12-	10000	VEGFR	0.187076	0.909
**156**	4-O3B-A15-	2500	VEGFR	0.42368	0
**157**	4-O3B-A20-	10000	VEGFR	0.350699	0.012
**158**	4-O3B-B12-	1000	VEGFR	0.266042	0.427
**159**	4-O3B-B15-	250	VEGFR	0.113105	0.47
**160**	4-O3B-B20-	1000	VEGFR	0.353152	0.013
**161**	4-O3B-C15-	25	VEGFR	0.195642	0.486
**162**	4-O3B-D12	100	VEGFR	0.404282	0.001
**163**	4-O3B-D15	2.5	VEGFR	0.097447	0.969
**164**	4-O3B-D20	100	VEGFR	0.268399	0.128
**165**	4-O3B-E12-	10	VEGFR	0.30884	0.065
**166**	4-O3B-E20-	10	VEGFR	0.279399	0.119
**167**	4-O3B-F12-	1	VEGFR	0.146157	0.444
**168**	4-O3B-F15-	0.25	VEGFR	0.161537	0.39
**169**	4-O3B-F20-	1	VEGFR	0.076331	0.697
**170**	4-O3B-L16-	1	VEGFR	0.432328	0
**171**	4-O3B-M16	10	VEGFR	0.208678	0.528
**172**	4-O3B-N16	100	VEGFR	0.126178	0.964
**173**	4-O3B-O16	1000	VEGFR	0.474165	0
**174**	4-O3B-P16-	10000	VEGFR	0.368872	0.011
**175**	2-O3B-L10-	1	PI3K	0.053103	0.988
**176**	2-O3B-M1C	10	PI3K	0.298205	0.083
**177**	2-O3B-N10	100	PI3K	0.367625	0.003
**178**	2-O3B-O10	1000	PI3K	0.417746	0
**179**	2-O3B-P10-	10000	PI3K	0.398581	0.001
**180**	3-O3B-A16-	2500	PI3K	0.066335	0.979

**181**	3-O3B-C16-	250	PI3K	0.446972	0
**182**	3-O3B-D16	25	PI3K	0.041109	0.957
**183**	3-O3B-E16-	2.5	PI3K	0.021942	1
**184**	3-O3B-F16-	0.25	PI3K	0.369929	0.004
**185**	3-O3B-F17-	100000	PI3K	0.372872	0
**186**	3-O3B-F19-	500	PI3K	0.431037	0
**187**	3-O3B-G17	10000	PI3K	0.048304	0.954
**188**	3-O3B-G19	50	PI3K	0.373107	0.001
**189**	3-O3B-H17	1000	PI3K	0.163251	0.765
**190**	3-O3B-I17-I	100	PI3K	0.249729	0.108
**191**	3-O3B-I19-I	5	PI3K	0.440381	0
**192**	3-O3B-J17-	10	PI3K	0.248989	0.107
**193**	3-O3B-J19-	0.5	PI3K	0.143404	0.486
**194**	3-O3B-K19-	0.05	PI3K	0.179608	0.561
**195**	3-O3B-L8-P	1	PI3K	0.084885	0.625
**196**	3-O3B-L21-	0.1	PI3K	0.06076	0.928
**197**	3-O3B-M8-I	10	PI3K	0.322851	0.02
**198**	3-O3B-M21	1	PI3K	0.255978	0.087
**199**	3-O3B-N8-F	100	PI3K	0.362095	0
**200**	3-O3B-N21	10	PI3K	0.064254	0.998
**201**	3-O3B-O8-F	1000	PI3K	0.39555	0
**202**	3-O3B-O21	100	PI3K	0.333077	0.012
**203**	3-O3B-P8-F	10000	PI3K	0.370681	0.007
**204**	3-O3B-P21-	1000	PI3K	0.331116	0.013
**205**	4-O3B-A19-	2500	PI3K	0.471381	0
**206**	4-O3B-B19-	250	PI3K	0.176357	0.583
**207**	4-O3B-C19-	25	PI3K	0.291747	0.038
**208**	4-O3B-D19	2.5	PI3K	0.400059	0
**209**	4-O3B-E19-	0.25	PI3K	0.458724	0
**210**	4-O3B-F14-	1000	PI3K	0.3694	0.017
**211**	4-O3B-G2-T	2500	PI3K	0.198672	0.728
**212**	4-O3B-G5-S	10000	PI3K	0.452866	0
**213**	4-O3B-G14	100	PI3K	0.382685	0.001
**214**	4-O3B-G20	10000	PI3K	0.364433	0.011
**215**	4-O3B-H2-T	250	PI3K	0.068619	0.989

216	4-O3B-H5-S	1000	PI3K	0.296665	0.01
217	4-O3B-H14	10	PI3K	0.452607	0
218	4-O3B-H20	1000	PI3K	0.39141	0.001
219	4-O3B-I2-T(	25	PI3K	0.130014	0.914
220	4-O3B-I5-S(	100	PI3K	0.292416	0.126
221	4-O3B-I14-I	1	PI3K	0.413347	0
222	4-O3B-I20-I	100	PI3K	0.040624	0.966
223	4-O3B-J2-T(	2.5	PI3K	0.019431	1
224	4-O3B-J5-S(	10	PI3K	0.256661	0.232
225	4-O3B-J20-	10	PI3K	0.136931	0.621
226	4-O3B-K2-T	0.25	PI3K	0.103977	0.963
227	4-O3B-K4-D	0.1	PI3K	0.442651	0
228	4-O3B-K5-S	1	PI3K	0.430581	0
229	4-O3B-K14-	0.1	PI3K	0.226492	0.331
230	4-O3B-K20-	1	PI3K	0.364234	0.001
231	4-O3B-L4-D	1	PI3K	0.435784	0
232	4-O3B-L14-	0.1	PI3K	0.41646	0
233	4-O3B-L15-	1	PI3K	0.146936	0.311
234	4-O3B-L21-	0.1	PI3K	0.308942	0.011
235	4-O3B-M14	1	PI3K	0.218546	0.193
236	4-O3B-M15	10	PI3K	0.439589	0
237	4-O3B-M21	1	PI3K	0.187562	0.339
238	4-O3B-N4-D	10	PI3K	0.42289	0
239	4-O3B-N14	10	PI3K	0.441719	0
240	4-O3B-N15	100	PI3K	0.369911	0.001
241	4-O3B-N21	10	PI3K	0.392233	0
242	4-O3B-O4-D	100	PI3K	0.38912	0.003
243	4-O3B-O14	100	PI3K	0.380252	0.003
244	4-O3B-O15	1000	PI3K	0.430406	0
245	4-O3B-O21	100	PI3K	0.41397	0.001
246	4-O3B-P4-D	1000	PI3K	0.395236	0
247	4-O3B-P14-	1000	PI3K	0.384324	0.001
248	4-O3B-P15-	10000	PI3K	0.426256	0
249	4-O3B-P21-	1000	PI3K	0.348952	0.007
250	5-O3B-A6-L	2500	PI3K	0.353853	0.017

**251**	5-O3B-A7-A	1000	PI3K	0.398026	0
**252**	5-O3B-A16-	2500	PI3K	0.457966	0
**253**	5-O3B-A17-	10000	PI3K	0.217642	0.099
**254**	5-O3B-B6-L	250	PI3K	0.45647	0
**255**	5-O3B-B7-A	100	PI3K	0.153891	0.559
**256**	5-O3B-B17-	1000	PI3K	0.469679	0
**257**	5-O3B-C6-L	25	PI3K	0.283603	0.024
**258**	5-O3B-C7-A	10	PI3K	0.456816	0
**259**	5-O3B-C16-	250	PI3K	0.444954	0
**260**	5-O3B-C17-	100	PI3K	0.423731	0
**261**	5-O3B-D6-L	2.5	PI3K	0.345676	0.001
**262**	5-O3B-D7-A	1	PI3K	0.473551	0
**263**	5-O3B-D16	25	PI3K	0.431897	0
**264**	5-O3B-D17	10	PI3K	0.330793	0.004
**265**	5-O3B-E6-L	0.25	PI3K	0.098776	0.742
**266**	5-O3B-E7-A	0.1	PI3K	0.175413	0.213
**267**	5-O3B-E16-	2.5	PI3K	0.277259	0.033
**268**	5-O3B-E17-	1	PI3K	0.291877	0.015
**269**	5-O3B-F11-	10000	PI3K	0.484308	0
**270**	5-O3B-F16-	0.25	PI3K	0.186252	0.209
**271**	5-O3B-G9-S	10000	PI3K	0.458241	0
**272**	5-O3B-G11	1000	PI3K	0.464693	0
**273**	5-O3B-H9-S	1000	PI3K	0.428191	0
**274**	5-O3B-H11	100	PI3K	0.411845	0
**275**	5-O3B-I9-Se	100	PI3K	0.056307	0.979
**276**	5-O3B-I11-	10	PI3K	0.160265	0.391
**277**	5-O3B-J9-S	10	PI3K	0.484921	0
**278**	5-O3B-J11-	1	PI3K	0.089134	0.752
**279**	5-O3B-K9-S	1	PI3K	0.42681	0
**280**	5-O3B-L14-	0.1	PI3K	0.438823	0
**281**	5-O3B-L20-	1	PI3K	0.499853	0
**282**	5-O3B-L23-	0.1	PI3K	0.186024	0.703
**283**	5-O3B-M14	1	PI3K	0.423661	0
**284**	5-O3B-M20	10	PI3K	0.489317	0
**285**	5-O3B-M23	1	PI3K	0.440998	0

**286**	5-O3B-N14	10	PI3K	0.419784	0
**287**	5-O3B-N20	100	PI3K	0.446535	0
**288**	5-O3B-N23	10	PI3K	0.476455	0
**289**	5-O3B-O14	100	PI3K	0.385409	0
**290**	5-O3B-O20	1000	PI3K	0.392889	0.003
**291**	5-O3B-O23	100	PI3K	0.367647	0.003
**292**	5-O3B-P14·	1000	PI3K	0.445924	0
**293**	5-O3B-P20·	10000	PI3K	0.373868	0.005
**294**	5-O3B-P23·	1000	PI3K	0.343913	0.01
**295**	6-O3B-A8-T	10000	PI3K	0.415302	0
**296**	6-O3B-B8-T	1000	PI3K	0.408884	0
**297**	6-O3B-C8-T	100	PI3K	0.216469	0.398
**298**	6-O3B-D8-T	10	PI3K	0.116935	0.973
**299**	6-O3B-E8-T	1	PI3K	0.025015	1
**300**	6-O3B-L6-G	1	PI3K	0.157753	0.801
**301**	6-O3B-M6-(	10	PI3K	0.073172	0.986
**302**	6-O3B-N6-(	100	PI3K	0.22845	0.263
**303**	6-O3B-O6-(	1000	PI3K	0.384718	0
**304**	6-O3B-P6-G	10000	PI3K	0.363019	0.011
**305**	1-O3B-F11-	10000	Topoisome	0.45138	0.002
**306**	1-O3B-G11	1000	Topoisome	0.634821	0
**307**	1-O3B-G20	1000	Topoisome	0.423835	0.007
**308**	1-O3B-H11	100	Topoisome	0.63454	0
**309**	1-O3B-H20	100	Topoisome	0.595764	0
**310**	1-O3B-I11-.	10	Topoisome	0.296918	0.08
**311**	1-O3B-I20-I	10	Topoisome	0.260214	0.328
**312**	1-O3B-J11-	1	Topoisome	0.11952	0.657
**313**	1-O3B-J20-	1	Topoisome	0.21073	0.461
**314**	1-O3B-K11·	1	Topoisome	0.571072	0
**315**	1-O3B-K20·	0.1	Topoisome	0.257563	0.358
**316**	1-O3B-L11-	10	Topoisome	0.477192	0
**317**	1-O3B-L14-	1	Topoisome	0.317243	0.067
**318**	1-O3B-M11	100	Topoisome	0.553345	0
**319**	1-O3B-M14	10	Topoisome	0.53553	0
**320**	1-O3B-N14	100	Topoisome	0.642375	0

**321**	1-O3B-O11	1000	Topoisome	0.439942	0.003
**322**	1-O3B-O14	1000	Topoisome	0.564288	0
**323**	1-O3B-P11·	10000	Topoisome	0.443875	0.004
**324**	1-O3B-P14·	10000	Topoisome	0.430531	0.003
**325**	3-O3B-A11·	10000	Topoisome	0.553391	0
**326**	3-O3B-B11·	1000	Topoisome	0.595349	0
**327**	3-O3B-C11·	100	Topoisome	0.561992	0
**328**	3-O3B-D11	10	Topoisome	0.446045	0.017
**329**	3-O3B-E11-	1	Topoisome	0.212862	0.201
**330**	3-O3B-G9-[	1000	Topoisome	0.456722	0.001
**331**	3-O3B-G10	10000	Topoisome	0.47362	0
**332**	3-O3B-H9-[	100	Topoisome	0.495438	0
**333**	3-O3B-H10	1000	Topoisome	0.134386	0.575
**334**	3-O3B-I9-D	10	Topoisome	0.281389	0.545
**335**	3-O3B-I10-'	100	Topoisome	0.140513	0.647
**336**	3-O3B-J9-D	1	Topoisome	0.171996	0.705
**337**	3-O3B-J10-	10	Topoisome	0.157002	0.635
**338**	3-O3B-K7-I	0.1	Topoisome	0.418519	0.024
**339**	3-O3B-K9-[	0.1	Topoisome	0.439255	0.001
**340**	3-O3B-K10·	1	Topoisome	0.078317	0.793
**341**	3-O3B-L6-[	0.1	Topoisome	0.11623	0.892
**342**	3-O3B-L7-I	1	Topoisome	0.470685	0.008
**343**	3-O3B-L9-V	0.5	Topoisome	0.434403	0
**344**	3-O3B-L10-	0.1	Topoisome	0.098121	0.753
**345**	3-O3B-L16-	1	Topoisome	0.32384	0.243
**346**	3-O3B-M6-I	1	Topoisome	0.245588	0.317
**347**	3-O3B-M7-I	10	Topoisome	0.512287	0
**348**	3-O3B-M9-'	5	Topoisome	0.362362	0.09
**349**	3-O3B-M1C	1	Topoisome	0.384063	0.008
**350**	3-O3B-M16	10	Topoisome	0.350406	0.028
**351**	3-O3B-N6-[	10	Topoisome	0.420137	0
**352**	3-O3B-N9-\	50	Topoisome	0.602419	0
**353**	3-O3B-N10	10	Topoisome	0.586425	0
**354**	3-O3B-N16	100	Topoisome	0.232733	0.347
**355**	3-O3B-O6-[	100	Topoisome	0.574049	0

**356**	3-O3B-O7-I	100	Topoisome	0.576718	0
**357**	3-O3B-O9-\	500	Topoisome	0.586458	0
**358**	3-O3B-O10	100	Topoisome	0.598868	0
**359**	3-O3B-O16	1000	Topoisome	0.491643	0
**360**	3-O3B-P6-D	1000	Topoisome	0.453009	0.001
**361**	3-O3B-P7-I	1000	Topoisome	0.493498	0
**362**	3-O3B-P9-V	5000	Topoisome	0.433171	0.01
**363**	3-O3B-P10-	1000	Topoisome	0.450894	0.002
**364**	3-O3B-P16-	10000	Topoisome	0.40924	0.007
**365**	1-O3B-A10-	10000	Mitotic	0.613548	0
**366**	1-O3B-A13-	1000	Mitotic	0.622696	0
**367**	1-O3B-A18-	1000	Mitotic	0.669149	0
**368**	1-O3B-B10-	1000	Mitotic	0.617237	0
**369**	1-O3B-B13-	100	Mitotic	0.066988	0.919
**370**	1-O3B-B18-	100	Mitotic	0.630871	0
**371**	1-O3B-C10-	100	Mitotic	0.653479	0
**372**	1-O3B-C13-	10	Mitotic	0.359186	0.057
**373**	1-O3B-C18-	10	Mitotic	0.627394	0
**374**	1-O3B-D10	10	Mitotic	0.61288	0
**375**	1-O3B-D13	1	Mitotic	0.278059	0.126
**376**	1-O3B-D18	1	Mitotic	0.528444	0
**377**	1-O3B-E10-	1	Mitotic	0.377082	0.019
**378**	1-O3B-E13-	0.1	Mitotic	0.27982	0.402
**379**	1-O3B-E18-	0.1	Mitotic	0.34613	0.095
**380**	1-O3B-F13-	1000	Mitotic	0.656621	0
**381**	1-O3B-G13	100	Mitotic	0.542092	0
**382**	1-O3B-G15	1000	Mitotic	0.678197	0
**383**	1-O3B-H13	10	Mitotic	0.333576	0.138
**384**	1-O3B-H15	100	Mitotic	0.662445	0
**385**	1-O3B-I13-'	1	Mitotic	0.490575	0.002
**386**	1-O3B-I15-I	10	Mitotic	0.578946	0
**387**	1-O3B-J13-	0.1	Mitotic	0.227777	0.187
**388**	1-O3B-J15-	1	Mitotic	0.613132	0
**389**	1-O3B-K7-V	0.1	Mitotic	0.133917	0.851
**390**	1-O3B-K15-	0.1	Mitotic	0.596184	0

**391**	1-O3B-L7-V	1	Mitotic	0.123579	0.993
**392**	1-O3B-L20-	0.1	Mitotic	0.438932	0.012
**393**	1-O3B-M7-'	10	Mitotic	0.530696	0.001
**394**	1-O3B-M2C	1	Mitotic	0.581623	0
**395**	1-O3B-N20	10	Mitotic	0.461803	0.001
**396**	1-O3B-O7-\	100	Mitotic	0.609405	0
**397**	1-O3B-O20	100	Mitotic	0.469824	0.003
**398**	1-O3B-P7-\	1000	Mitotic	0.605704	0
**399**	1-O3B-P20-	1000	Mitotic	0.570464	0
**400**	3-O3B-A7-D	1000	Mitotic	0.625511	0
**401**	3-O3B-B7-D	100	Mitotic	0.581004	0
**402**	3-O3B-C7-D	10	Mitotic	0.593418	0
**403**	3-O3B-D7-D	1	Mitotic	0.469389	0.002
**404**	3-O3B-E7-D	0.1	Mitotic	0.064708	1
**405**	6-O3B-L19-	1	Mitotic	0.358497	0.176
**406**	6-O3B-M19	10	Mitotic	0.468564	0
**407**	6-O3B-N19	100	Mitotic	0.070338	0.988
**408**	6-O3B-O19	1000	Mitotic	0.612598	0
**409**	6-O3B-P19-	10000	Mitotic	0.618442	0
**410**	2-O3B-A12-	250	MEK1/2	0.640991	0
**411**	2-O3B-B12-	25	MEK1/2	0.628944	0
**412**	2-O3B-D12	2.5	MEK1/2	0.710066	0
**413**	2-O3B-E12-	0.25	MEK1/2	0.30376	0.311
**414**	2-O3B-F12-	2.5000000	MEK1/2	0.67247	0
**415**	2-O3B-F14-	1000	MEK1/2	0.638246	0
**416**	2-O3B-G14	100	MEK1/2	0.729319	0
**417**	2-O3B-H14	10	MEK1/2	0.779972	0
**418**	2-O3B-I14-	1	MEK1/2	0.651878	0
**419**	2-O3B-K14-	0.1	MEK1/2	0.538219	0.001
**420**	2-O3B-L20-	1	MEK1/2	0.331386	0.652
**421**	2-O3B-M2C	10	MEK1/2	0.48658	0.01
**422**	2-O3B-N20	100	MEK1/2	0.743515	0
**423**	2-O3B-O20	1000	MEK1/2	0.617542	0
**424**	2-O3B-P20-	10000	MEK1/2	0.659134	0
**425**	4-O3B-A10-	1000	MEK1/2	0.682266	0

**426**	4-O3B-A13-	1000	MEK1/2	0.655164	0
**427**	4-O3B-B10-	100	MEK1/2	0.666302	0
**428**	4-O3B-B13-	100	MEK1/2	0.668165	0
**429**	4-O3B-C10-	10	MEK1/2	0.694098	0
**430**	4-O3B-C13-	10	MEK1/2	0.675535	0
**431**	4-O3B-D10	1	MEK1/2	0.427464	0.02
**432**	4-O3B-D13	1	MEK1/2	0.606221	0
**433**	4-O3B-E10-	0.1	MEK1/2	0.390874	0.025
**434**	4-O3B-E13-	0.1	MEK1/2	0.658493	0
**435**	4-O3B-L19-	0.25	MEK1/2	0.379977	0.144
**436**	4-O3B-M19	2.5	MEK1/2	0.577134	0.001
**437**	4-O3B-N19	25	MEK1/2	0.695338	0
**438**	4-O3B-O19	250	MEK1/2	0.497644	0.005
**439**	4-O3B-P19-	2500	MEK1/2	0.694652	0
**440**	1-O3B-L2-C	1	PARP	0.428901	0.061
**441**	1-O3B-L6-R	1	PARP	0.450973	0.042
**442**	1-O3B-M2-	10	PARP	0.461582	0.029
**443**	1-O3B-M6-	10	PARP	0.404471	0.125
**444**	1-O3B-N2-C	100	PARP	0.699445	0
**445**	1-O3B-N6-F	100	PARP	0.754929	0
**446**	1-O3B-O2-C	1000	PARP	0.618724	0
**447**	1-O3B-O6-F	1000	PARP	0.73773	0
**448**	1-O3B-P2-C	10000	PARP	0.566343	0.001
**449**	1-O3B-P6-R	10000	PARP	0.649515	0
**450**	7-O3B-A3-T	1000	PARP	0.626132	0
**451**	7-O3B-B2-V	10000	PARP	0.722614	0
**452**	7-O3B-B3-T	100	PARP	0.654341	0
**453**	7-O3B-C2-V	1000	PARP	0.472534	0.038
**454**	7-O3B-C3-T	10	PARP	0.690509	0
**455**	7-O3B-D2-V	100	PARP	0.185545	0.762
**456**	7-O3B-D3-T	1	PARP	0.824773	0
**457**	7-O3B-E2-V	10	PARP	0.459101	0.051
**458**	7-O3B-E3-T	0.1	PARP	0.24319	0.696
**459**	7-O3B-F2-V	1	PARP	0.169859	0.732
**460**	7-O3B-G2-N	10000	PARP	0.670517	0

**461**	7-O3B-H2-N	1000	PARP	0.621724	0
**462**	7-O3B-I2-N	100	PARP	0.660939	0
**463**	7-O3B-J2-N	10	PARP	0.521171	0.008
**464**	7-O3B-K2-N	1	PARP	0.333233	0.649
**465**	3-O3B-A19-	1000	CDK	0.465402	0
**466**	3-O3B-B19-	100	CDK	0.460476	0.001
**467**	3-O3B-B23-	2500	CDK	0.403893	0.012
**468**	3-O3B-C19-	10	CDK	0.490941	0
**469**	3-O3B-C23-	250	CDK	0.399587	0.007
**470**	3-O3B-D19	1	CDK	0.164913	0.667
**471**	3-O3B-D23	25	CDK	0.424979	0
**472**	3-O3B-E19-	0.1	CDK	0.114831	0.779
**473**	3-O3B-E23-	2.5	CDK	0.326745	0.086
**474**	3-O3B-F23-	0.25	CDK	0.35876	0.028
**475**	3-O3B-K17-	1	CDK	0.20935	0.764
**476**	3-O3B-L19-	1	CDK	0.162745	0.78
**477**	3-O3B-M17	10	CDK	0.388254	0.02
**478**	3-O3B-M19	10	CDK	0.342238	0.071
**479**	3-O3B-N17	100	CDK	0.368379	0.031
**480**	3-O3B-N19	100	CDK	0.411443	0.031
**481**	3-O3B-O17	1000	CDK	0.368348	0.031
**482**	3-O3B-O19	1000	CDK	0.363492	0.051
**483**	3-O3B-P17-	10000	CDK	0.372619	0.034
**484**	3-O3B-P19-	10000	CDK	0.403332	0.007
**485**	4-O3B-A4-S	10000	CDK	0.479491	0
**486**	4-O3B-A8-N	10000	CDK	0.472147	0.003
**487**	4-O3B-B4-S	1000	CDK	0.476848	0
**488**	4-O3B-B8-N	1000	CDK	0.411779	0.036
**489**	4-O3B-C4-S	100	CDK	0.448142	0.005
**490**	4-O3B-C8-N	100	CDK	0.367135	0.042
**491**	4-O3B-D4-S	10	CDK	0.291523	0.622
**492**	4-O3B-D8-N	10	CDK	0.118535	0.94
**493**	4-O3B-E4-S	1	CDK	0.256649	0.495
**494**	4-O3B-E8-N	1	CDK	0.371501	0.089
**495**	4-O3B-F4-S	10000	CDK	0.456626	0.002

**496**	4-O3B-F22-	10000	CDK	0.492963	0
**497**	4-O3B-G4-S	1000	CDK	0.346935	0.103
**498**	4-O3B-G22	1000	CDK	0.489368	0
**499**	4-O3B-H4-S	100	CDK	0.340488	0.149
**500**	4-O3B-H22	100	CDK	0.41125	0.001
**501**	4-O3B-I4-Se	10	CDK	0.378659	0.03
**502**	4-O3B-I22-,	10	CDK	0.339952	0.078
**503**	4-O3B-J4-S	1	CDK	0.358983	0.014
**504**	4-O3B-J22-	1	CDK	0.348747	0.05
**505**	5-O3B-A19-	10000	CDK	0.453044	0.001
**506**	5-O3B-B19-	1000	CDK	0.434636	0.003
**507**	5-O3B-C19-	100	CDK	0.427647	0.003
**508**	5-O3B-D19	10	CDK	0.046366	0.984
**509**	5-O3B-E19-	1	CDK	0.231373	0.447
**510**	5-O3B-K17-	1	CDK	0.345444	0.036
**511**	5-O3B-M17	10	CDK	0.112544	0.924
**512**	5-O3B-N17	100	CDK	0.354778	0.036
**513**	5-O3B-O17	1000	CDK	0.436295	0.009
**514**	5-O3B-P17-	10000	CDK	0.45348	0.002
**515**	6-O3B-A17-	1000	CDK	0.244781	0.35
**516**	6-O3B-B17-	100	CDK	0.491807	0
**517**	6-O3B-C17-	10	CDK	0.323815	0.132
**518**	6-O3B-D17	1	CDK	0.048978	0.988
**519**	6-O3B-E17-	0.1	CDK	0.342864	0.172
**520**	6-O3B-L15-	1	CDK	0.087811	0.917
**521**	6-O3B-M15	10	CDK	0.350239	0.127
**522**	6-O3B-N15	100	CDK	0.441766	0.005
**523**	6-O3B-O15	1000	CDK	0.447828	0.002
**524**	6-O3B-P15-	10000	CDK	0.42925	0.002
**525**	7-O3B-A21-	10000	BET	0.430671	0.026
**526**	7-O3B-A22-	30000	BET	0.559425	0
**527**	7-O3B-B21-	1000	BET	0.513701	0.001
**528**	7-O3B-B22-	3000	BET	0.432313	0
**529**	7-O3B-C21-	100	BET	0.136263	0.767
**530**	7-O3B-C22-	300	BET	0.456977	0

**531**	7-O3B-D21	10	BET	0.180556	0.378
**532**	7-O3B-D22	30	BET	0.470149	0.001
**533**	7-O3B-E21-	1	BET	0.210816	0.424
**534**	7-O3B-E22-	3	BET	0.157899	0.821
**535**	7-O3B-G10	10000	BET	0.589174	0
**536**	7-O3B-G15	10000	BET	0.56268	0
**537**	7-O3B-H10	1000	BET	0.578154	0
**538**	7-O3B-H15	1000	BET	0.580773	0
**539**	7-O3B-I10-I	100	BET	0.709524	0
**540**	7-O3B-I15-I	100	BET	0.38857	0.096
**541**	7-O3B-J10-	10	BET	0.62451	0
**542**	7-O3B-J15-	10	BET	0.291087	0.207
**543**	7-O3B-K10-	1	BET	0.32623	0.006
**544**	7-O3B-K13-	1	BET	0.412057	0
**545**	7-O3B-K15-	1	BET	0.388055	0.003
**546**	7-O3B-L12-	1	BET	0.276281	0.467
**547**	7-O3B-L13-	10	BET	0.617088	0
**548**	7-O3B-L20-	1	BET	0.163837	0.887
**549**	7-O3B-L23-	0.03	BET	0.202523	0.377
**550**	7-O3B-M12	10	BET	0.704336	0
**551**	7-O3B-M13	100	BET	0.609909	0
**552**	7-O3B-M2C	10	BET	0.430536	0.011
**553**	7-O3B-M23	0.3	BET	0.310087	0.32
**554**	7-O3B-N12	100	BET	0.424393	0
**555**	7-O3B-N13	1000	BET	0.515964	0
**556**	7-O3B-N20	100	BET	0.612927	0
**557**	7-O3B-N23	3	BET	0.606148	0
**558**	7-O3B-O12	1000	BET	0.643059	0
**559**	7-O3B-O20	1000	BET	0.571896	0
**560**	7-O3B-O23	30	BET	0.519521	0
**561**	7-O3B-P12-	10000	BET	0.548811	0
**562**	7-O3B-P13-	10000	BET	0.455199	0.014
**563**	7-O3B-P20-	10000	BET	0.473185	0.009
**564**	7-O3B-P23-	300	BET	0.606285	0
**565**	8-O3B-K22-	1	BET	0.068993	0.998

**566**	8-O3B-L22-	10	BET	0.172449	0.8
**567**	8-O3B-M22	100	BET	0.390922	0.023
**568**	8-O3B-N22	1000	BET	0.472688	0.001
**569**	8-O3B-O22	10000	BET	0.434101	0.001
**570**	1-O3B-A3-\	10000	HDAC	0.405111	0.001
**571**	1-O3B-B3-\	1000	HDAC	0.418124	0
**572**	1-O3B-C3-\	100	HDAC	0.380819	0.003
**573**	1-O3B-D3-\	10	HDAC	0.254424	0.187
**574**	1-O3B-E3-V	1	HDAC	0.288526	0.089
**575**	1-O3B-L12-	0.1	HDAC	0.381666	0
**576**	1-O3B-M12	1	HDAC	0.442621	0
**577**	1-O3B-N12	10	HDAC	0.399605	0.001
**578**	1-O3B-O12	100	HDAC	0.373841	0.003
**579**	1-O3B-P12-	1000	HDAC	0.388245	0.002
**580**	3-O3B-A4-F	1000	HDAC	0.371626	0.01
**581**	3-O3B-B4-F	100	HDAC	0.417464	0.001
**582**	3-O3B-C4-F	10	HDAC	0.372753	0.002
**583**	3-O3B-D4-F	1	HDAC	0.341926	0.009
**584**	3-O3B-E4-P	0.1	HDAC	0.312938	0.085
**585**	3-O3B-F7-C	1000	HDAC	0.399297	0.001
**586**	3-O3B-G7-(	100	HDAC	0.361295	0.004
**587**	3-O3B-G12	1000000	HDAC	0.329675	0.021
**588**	3-O3B-H7-(	10	HDAC	0.266177	0.375
**589**	3-O3B-H12	100000	HDAC	0.079968	0.954
**590**	3-O3B-I7-Q	1	HDAC	0.132169	0.931
**591**	3-O3B-I12-'	10000	HDAC	0.113131	0.967
**592**	3-O3B-J7-Q	0.1	HDAC	0.084417	0.945
**593**	3-O3B-J12-	1000	HDAC	0.10422	0.913
**594**	3-O3B-K3-E	1	HDAC	0.389866	0.001
**595**	3-O3B-K12-	100	HDAC	0.343283	0
**596**	3-O3B-L3-B	10	HDAC	0.043569	0.998
**597**	3-O3B-M3-I	100	HDAC	0.401391	0.002
**598**	3-O3B-N3-E	1000	HDAC	0.383578	0.018
**599**	3-O3B-O3-E	10000	HDAC	0.378358	0.004
**600**	7-O3B-A5-N	10000	HDAC	0.470177	0

**601**	7-O3B-A7-C	10000	HDAC	0.408078	0
**602**	7-O3B-A9-G	1000	HDAC	0.435369	0.002
**603**	7-O3B-A12-	10000	HDAC	0.427038	0.001
**604**	7-O3B-B5-N	1000	HDAC	0.465805	0
**605**	7-O3B-B7-C	1000	HDAC	0.430789	0.001
**606**	7-O3B-B12-	1000	HDAC	0.47198	0
**607**	7-O3B-C5-N	100	HDAC	0.231678	0.333
**608**	7-O3B-C7-C	100	HDAC	0.444706	0
**609**	7-O3B-C9-G	100	HDAC	0.274381	0.113
**610**	7-O3B-D7-C	10	HDAC	0.359168	0.004
**611**	7-O3B-D9-C	10	HDAC	0.389122	0.007
**612**	7-O3B-D12	100	HDAC	0.047895	0.888
**613**	7-O3B-E5-N	10	HDAC	0.126269	0.64
**614**	7-O3B-E7-C	1	HDAC	0.324089	0.049
**615**	7-O3B-E9-G	1	HDAC	0.086194	0.962
**616**	7-O3B-E12-	10	HDAC	0.191269	0.166
**617**	7-O3B-F5-N	1	HDAC	0.342301	0.002
**618**	7-O3B-F7-R	10000	HDAC	0.466264	0
**619**	7-O3B-F9-G	0.1	HDAC	0.279584	0.007
**620**	7-O3B-F12-	1	HDAC	0.472879	0
**621**	7-O3B-F19-	10000	HDAC	0.459286	0
**622**	7-O3B-G7-F	1000	HDAC	0.450722	0
**623**	7-O3B-G19	1000	HDAC	0.369313	0.002
**624**	7-O3B-H7-F	100	HDAC	0.254787	0.339
**625**	7-O3B-I7-R	10	HDAC	0.387664	0
**626**	7-O3B-I19-I	100	HDAC	0.379791	0.001
**627**	7-O3B-J7-R	1	HDAC	0.307922	0.149
**628**	7-O3B-J19-	10	HDAC	0.147023	0.402
**629**	7-O3B-K4-E	1	HDAC	0.351742	0.003
**630**	7-O3B-K11-	1	HDAC	0.297751	0.022
**631**	7-O3B-K18-	1	HDAC	0.337687	0.001
**632**	7-O3B-K19-	1	HDAC	0.418962	0
**633**	7-O3B-L2-T	0.1	HDAC	0.463987	0
**634**	7-O3B-L4-E	10	HDAC	0.294852	0.042
**635**	7-O3B-L5-P	1	HDAC	0.45528	0

**636**	7-O3B-L8-A	1	HDAC	0.393504	0.001
**637**	7-O3B-L10-	1	HDAC	0.173901	0.045
**638**	7-O3B-L11-	10	HDAC	0.416455	0
**639**	7-O3B-L14-	1	HDAC	0.431456	0
**640**	7-O3B-L16-	1	HDAC	0.405456	0
**641**	7-O3B-L18-	10	HDAC	0.165091	0.815
**642**	7-O3B-M2-T	1	HDAC	0.318472	0.008
**643**	7-O3B-M5-I	10	HDAC	0.428755	0
**644**	7-O3B-M8-,	10	HDAC	0.454394	0
**645**	7-O3B-M1C	10	HDAC	0.477474	0
**646**	7-O3B-M11	100	HDAC	0.467211	0
**647**	7-O3B-M14	10	HDAC	0.093784	0.731
**648**	7-O3B-M1€	10	HDAC	0.508327	0
**649**	7-O3B-M18	100	HDAC	0.454919	0
**650**	7-O3B-N2-T	10	HDAC	0.291907	0.026
**651**	7-O3B-N4-E	100	HDAC	0.486558	0
**652**	7-O3B-N5-F	100	HDAC	0.464564	0
**653**	7-O3B-N8-/	100	HDAC	0.453541	0
**654**	7-O3B-N10	100	HDAC	0.523689	0
**655**	7-O3B-N14	100	HDAC	0.160439	0.446
**656**	7-O3B-N16	100	HDAC	0.166559	0.464
**657**	7-O3B-N18	1000	HDAC	0.558185	0
**658**	7-O3B-O2-T	100	HDAC	0.029715	0.998
**659**	7-O3B-O4-E	1000	HDAC	0.552022	0
**660**	7-O3B-O5-F	1000	HDAC	0.474116	0
**661**	7-O3B-O8-/	1000	HDAC	0.476989	0
**662**	7-O3B-O10	1000	HDAC	0.505442	0
**663**	7-O3B-O11	1000	HDAC	0.430608	0
**664**	7-O3B-O14	1000	HDAC	0.043203	0.939
**665**	7-O3B-O16	1000	HDAC	0.417939	0
**666**	7-O3B-P2-T	1000	HDAC	0.422814	0
**667**	7-O3B-P4-E	10000	HDAC	0.442075	0
**668**	7-O3B-P5-F	10000	HDAC	0.413202	0
**669**	7-O3B-P8-A	10000	HDAC	0.392066	0
**670**	7-O3B-P10-	10000	HDAC	0.315697	0.101

**671**	7-O3B-P11·	10000	HDAC	0.408793	0
**672**	7-O3B-P14·	10000	HDAC	0.375854	0
**673**	7-O3B-P16·	10000	HDAC	0.44421	0
**674**	7-O3B-P18·	10000	HDAC	0.432607	0
**0**	2-O8W-A16	10000	EGFR	0.169723	0.782
**1**	2-O8W-A19	10000	EGFR	0.284322	0.235
**2**	2-O8W-B19	1000	EGFR	0.096635	0.969
**3**	2-O8W-C16	1000	EGFR	0.190077	0.552
**4**	2-O8W-C19	100	EGFR	0.131179	0.835
**5**	2-O8W-D16	100	EGFR	0.099338	0.949
**6**	2-O8W-D19	10	EGFR	0.115588	0.966
**7**	2-O8W-E16	10	EGFR	0.090516	0.948
**8**	2-O8W-E19	1	EGFR	0.155315	0.867
**9**	2-O8W-F16	1	EGFR	0.234949	0.392
**10**	2-O8W-K11	0.1	EGFR	0.264115	0.03
**11**	2-O8W-L11	1	EGFR	0.226009	0.228
**12**	2-O8W-L16	0.25	EGFR	0.300406	0.056
**13**	2-O8W-L19	0.1	EGFR	0.188571	0.639
**14**	2-O8W-M1	10	EGFR	0.278582	0.103
**15**	2-O8W-M1	2.5	EGFR	0.253689	0.033
**16**	2-O8W-M1	1	EGFR	0.207669	0.486
**17**	2-O8W-N16	25	EGFR	0.327416	0.002
**18**	2-O8W-N19	10	EGFR	0.299211	0.045
**19**	2-O8W-O11	100	EGFR	0.283364	0.049
**20**	2-O8W-O16	250	EGFR	0.3083	0.118
**21**	2-O8W-O19	100	EGFR	0.147967	0.815
**22**	2-O8W-P11	1000	EGFR	0.281212	0.121
**23**	2-O8W-P16	2500	EGFR	0.236149	0.345
**24**	2-O8W-P19	1000	EGFR	0.157139	0.889
**25**	3-O8W-F21	10000	EGFR	0.086126	0.996
**26**	3-O8W-G20	1000	EGFR	0.175161	0.808
**27**	3-O8W-G21	1000	EGFR	0.261817	0.493
**28**	3-O8W-H20	100	EGFR	0.130941	0.822
**29**	3-O8W-H21	100	EGFR	0.204565	0.4
**30**	3-O8W-I20	10	EGFR	0.127337	0.884

**31**	3-O8W-I21	10	EGFR	0.18138	0.47
**32**	3-O8W-J20	1	EGFR	0.345191	0.024
**33**	3-O8W-J21	1	EGFR	0.27712	0.197
**34**	3-O8W-K4-	1	EGFR	0.277476	0.239
**35**	3-O8W-K1ξ	0.1	EGFR	0.227475	0.212
**36**	3-O8W-K2(	0.1	EGFR	0.155939	0.366
**37**	3-O8W-L4-(	10	EGFR	0.188168	0.695
**38**	3-O8W-L18	1	EGFR	0.20816	0.105
**39**	3-O8W-M1	10	EGFR	0.335517	0.091
**40**	3-O8W-N4-	100	EGFR	0.32353	0.031
**41**	3-O8W-N1ξ	100	EGFR	0.442456	0
**42**	3-O8W-O4-	1000	EGFR	0.146574	0.9
**43**	3-O8W-P4-	10000	EGFR	0.068138	0.988
**44**	3-O8W-P1ξ	1000	EGFR	0.328751	0.06
**45**	4-O8W-F13	1000	EGFR	0.239718	0.126
**46**	4-O8W-G1:	100	EGFR	0.383683	0.005
**47**	4-O8W-G1(	10000	EGFR	0.153769	0.817
**48**	4-O8W-H1:	10	EGFR	0.249807	0.112
**49**	4-O8W-H1(	1000	EGFR	0.211232	0.312
**50**	4-O8W-I13	1	EGFR	0.315693	0.004
**51**	4-O8W-I16	100	EGFR	0.306812	0.012
**52**	4-O8W-J13	0.1	EGFR	0.342986	0
**53**	4-O8W-J16	10	EGFR	0.441726	0
**54**	4-O8W-K7-	1	EGFR	0.325785	0.005
**55**	4-O8W-K1:	0.1	EGFR	0.164342	0.529
**56**	4-O8W-K1(	1	EGFR	0.276138	0.021
**57**	4-O8W-L7-I	10	EGFR	0.22714	0.11
**58**	4-O8W-L13	1	EGFR	0.349695	0.003
**59**	4-O8W-M7	100	EGFR	0.224342	0.112
**60**	4-O8W-M1	10	EGFR	0.35682	0.001
**61**	4-O8W-N1:	100	EGFR	0.241109	0.079
**62**	4-O8W-O7-	1000	EGFR	0.243128	0.096
**63**	4-O8W-P7-	10000	EGFR	0.216884	0.293
**64**	4-O8W-P1:	1000	EGFR	0.103609	0.915
**65**	5-O8W-F4-I	1000	EGFR	0.251899	0.371

**66**	5-O8W-F7-	1000	EGFR	0.225805	0.224
**67**	5-O8W-G4-	100	EGFR	0.156341	0.723
**68**	5-O8W-G7-	100	EGFR	0.231798	0.294
**69**	5-O8W-H4-	10	EGFR	0.156894	0.733
**70**	5-O8W-H7-	10	EGFR	0.254417	0.146
**71**	5-O8W-I4-	1	EGFR	0.25662	0.155
**72**	5-O8W-I7-	1	EGFR	0.274831	0.162
**73**	5-O8W-J4-	0.1	EGFR	0.197859	0.436
**74**	5-O8W-J7-	0.1	EGFR	0.298316	0.021
**75**	5-O8W-K7-	0.1	EGFR	0.263862	0.19
**76**	5-O8W-L7-	1	EGFR	0.258089	0.045
**77**	5-O8W-M7	10	EGFR	0.321091	0.015
**78**	5-O8W-O7-	100	EGFR	0.270918	0.04
**79**	5-O8W-P7-	1000	EGFR	0.289287	0.184
**80**	2-O8W-A15	2500	VEGFR	0.157657	0.73
**81**	2-O8W-A17	10000	VEGFR	0.197876	0.801
**82**	2-O8W-A20	10000	VEGFR	0.266443	0.176
**83**	2-O8W-B15	250	VEGFR	0.335242	0.093
**84**	2-O8W-B17	1000	VEGFR	0.259283	0.224
**85**	2-O8W-B20	1000	VEGFR	0.22041	0.55
**86**	2-O8W-C15	25	VEGFR	0.290545	0.048
**87**	2-O8W-C17	100	VEGFR	0.317589	0.016
**88**	2-O8W-D15	2.5	VEGFR	0.307031	0.017
**89**	2-O8W-D17	10	VEGFR	0.124045	0.769
**90**	2-O8W-D20	100	VEGFR	0.269159	0.123
**91**	2-O8W-E17	1	VEGFR	0.352176	0.006
**92**	2-O8W-E20	10	VEGFR	0.284396	0.103
**93**	2-O8W-F13	10000	VEGFR	0.28044	0.144
**94**	2-O8W-F15	0.25	VEGFR	0.272328	0.045
**95**	2-O8W-F19	10000	VEGFR	0.25398	0.261
**96**	2-O8W-F20	1	VEGFR	0.171511	0.87
**97**	2-O8W-F21	10000	VEGFR	0.05854	0.969
**98**	2-O8W-G10	10000	VEGFR	0.349272	0.001
**99**	2-O8W-G13	1000	VEGFR	0.38211	0.038
**100**	2-O8W-G19	1000	VEGFR	0.247665	0.147

**101**	2-O8W-G2:	1000	VEGFR	0.284671	0.095
**102**	2-O8W-H1(	1000	VEGFR	0.29162	0.02
**103**	2-O8W-H1:	100	VEGFR	0.257162	0.216
**104**	2-O8W-H2:	100	VEGFR	0.23058	0.455
**105**	2-O8W-I10	100	VEGFR	0.357138	0.001
**106**	2-O8W-I13	10	VEGFR	0.318998	0.006
**107**	2-O8W-I19	100	VEGFR	0.279081	0.067
**108**	2-O8W-I21	10	VEGFR	0.32405	0.014
**109**	2-O8W-J10	10	VEGFR	0.277388	0.028
**110**	2-O8W-J13	1	VEGFR	0.117849	0.722
**111**	2-O8W-J19	10	VEGFR	0.316554	0.002
**112**	2-O8W-J21	1	VEGFR	0.295855	0.045
**113**	2-O8W-K1(	1	VEGFR	0.305298	0.017
**114**	2-O8W-K1:	0.1	VEGFR	0.248182	0.105
**115**	2-O8W-K17	1	VEGFR	0.336992	0
**116**	2-O8W-K1!	1	VEGFR	0.318783	0.003
**117**	2-O8W-L12	0.1	VEGFR	0.264551	0.069
**118**	2-O8W-L13	1	VEGFR	0.300754	0.017
**119**	2-O8W-L21	0.1	VEGFR	0.320006	0.043
**120**	2-O8W-M1	1	VEGFR	0.335088	0.002
**121**	2-O8W-M1	10	VEGFR	0.156248	0.525
**122**	2-O8W-M1	10	VEGFR	0.319187	0.003
**123**	2-O8W-M2	1	VEGFR	0.229014	0.291
**124**	2-O8W-N1:	10	VEGFR	0.342594	0.003
**125**	2-O8W-N1:	100	VEGFR	0.27672	0.014
**126**	2-O8W-N1:	100	VEGFR	0.402304	0.002
**127**	2-O8W-N2:	10	VEGFR	0.097589	0.852
**128**	2-O8W-O1:	100	VEGFR	0.244509	0.35
**129**	2-O8W-O1:	1000	VEGFR	0.36335	0.001
**130**	2-O8W-O2:	100	VEGFR	0.221789	0.276
**131**	2-O8W-P1:	1000	VEGFR	0.30716	0.041
**132**	2-O8W-P1:	1000	VEGFR	0.206584	0.628
**133**	2-O8W-P1:	10000	VEGFR	0.309262	0.01
**134**	2-O8W-P21	1000	VEGFR	0.163469	0.703
**135**	3-O8W-A3-	1000	VEGFR	0.218807	0.64

**136**	3-O8W-A6-	1000	VEGFR	0.231735	0.487
**137**	3-O8W-A18	1000	VEGFR	0.229199	0.578
**138**	3-O8W-B3-	100	VEGFR	0.108026	0.921
**139**	3-O8W-B6-	100	VEGFR	0.195562	0.652
**140**	3-O8W-B18	100	VEGFR	0.265528	0.134
**141**	3-O8W-C3-	10	VEGFR	0.129562	0.842
**142**	3-O8W-C6-	10	VEGFR	0.195113	0.484
**143**	3-O8W-C18	10	VEGFR	0.194131	0.583
**144**	3-O8W-D3-	1	VEGFR	0.117922	0.955
**145**	3-O8W-D6-	1	VEGFR	0.175726	0.536
**146**	3-O8W-D18	1	VEGFR	0.242669	0.273
**147**	3-O8W-E3-	0.1	VEGFR	0.115829	0.991
**148**	3-O8W-E6-	0.1	VEGFR	0.147036	0.937
**149**	3-O8W-E18	0.1	VEGFR	0.295287	0.028
**150**	3-O8W-F18	1000	VEGFR	0.368161	0.004
**151**	3-O8W-G18	100	VEGFR	0.29535	0.041
**152**	3-O8W-H18	10	VEGFR	0.221765	0.099
**153**	3-O8W-I18	1	VEGFR	0.250137	0.372
**154**	3-O8W-J18	0.1	VEGFR	0.292497	0.011
**155**	4-O8W-A12	10000	VEGFR	0.223425	0.452
**156**	4-O8W-A15	2500	VEGFR	0.175535	0.696
**157**	4-O8W-A20	10000	VEGFR	0.211298	0.645
**158**	4-O8W-B12	1000	VEGFR	0.265275	0.126
**159**	4-O8W-B15	250	VEGFR	0.079105	0.94
**160**	4-O8W-B20	1000	VEGFR	0.210188	0.337
**161**	4-O8W-C15	25	VEGFR	0.128011	0.703
**162**	4-O8W-D12	100	VEGFR	0.120134	0.769
**163**	4-O8W-D15	2.5	VEGFR	0.270991	0.03
**164**	4-O8W-D20	100	VEGFR	0.226774	0.234
**165**	4-O8W-E12	10	VEGFR	0.209084	0.51
**166**	4-O8W-E20	10	VEGFR	0.178385	0.523
**167**	4-O8W-F12	1	VEGFR	0.078088	0.933
**168**	4-O8W-F15	0.25	VEGFR	0.292735	0.066
**169**	4-O8W-F20	1	VEGFR	0.13367	0.723
**170**	4-O8W-L16	1	VEGFR	0.134945	0.611

**171**	4-O8W-M1	10	VEGFR	0.273886	0.054
**172**	4-O8W-N1(	100	VEGFR	0.200708	0.305
**173**	4-O8W-O1(	1000	VEGFR	0.205582	0.317
**174**	4-O8W-P1(	10000	VEGFR	0.24966	0.146
**175**	2-O8W-L1C	1	PI3K	0.333441	0.001
**176**	2-O8W-M1	10	PI3K	0.345459	0
**177**	2-O8W-N1(	100	PI3K	0.041693	0.998
**178**	2-O8W-O1(	1000	PI3K	0.366265	0.001
**179**	2-O8W-P1(	10000	PI3K	0.108033	0.947
**180**	3-O8W-A1(	2500	PI3K	0.332319	0
**181**	3-O8W-C1(	250	PI3K	0.206624	0.022
**182**	3-O8W-D1(	25	PI3K	0.427813	0
**183**	3-O8W-E1(	2.5	PI3K	0.348446	0.001
**184**	3-O8W-F1(	0.25	PI3K	0.302141	0
**185**	3-O8W-F17	100000	PI3K	0.017858	1
**186**	3-O8W-F1(	500	PI3K	0.378189	0
**187**	3-O8W-G1	10000	PI3K	0.13844	0.84
**188**	3-O8W-G1(	50	PI3K	0.12535	0.846
**189**	3-O8W-H1	1000	PI3K	0.247327	0.102
**190**	3-O8W-I17	100	PI3K	0.068162	0.983
**191**	3-O8W-I19	5	PI3K	0.299539	0
**192**	3-O8W-J17	10	PI3K	0.234826	0.004
**193**	3-O8W-J19	0.5	PI3K	0.303764	0
**194**	3-O8W-K1(	0.05	PI3K	0.107521	0.949
**195**	3-O8W-L8-I	1	PI3K	0.173762	0.608
**196**	3-O8W-L21	0.1	PI3K	0.100212	0.953
**197**	3-O8W-M8	10	PI3K	0.323018	0.01
**198**	3-O8W-M2	1	PI3K	0.174411	0.88
**199**	3-O8W-N8-	100	PI3K	0.363844	0
**200**	3-O8W-N2	10	PI3K	0.044066	0.997
**201**	3-O8W-O8-	1000	PI3K	0.2675	0.058
**202**	3-O8W-O2	100	PI3K	0.298342	0.011
**203**	3-O8W-P8-	10000	PI3K	0.139743	0.917
**204**	3-O8W-P21	1000	PI3K	0.365224	0.003
**205**	4-O8W-A1(	2500	PI3K	0.342709	0

**206**	4-O8W-B1‹	250	PI3K	0.284675	0.016
**207**	4-O8W-C1‹	25	PI3K	0.385699	0.001
**208**	4-O8W-D1‹	2.5	PI3K	0.065998	1
**209**	4-O8W-E1‹	0.25	PI3K	0.050004	0.999
**210**	4-O8W-F14	1000	PI3K	0.034987	1
**211**	4-O8W-G2-	2500	PI3K	0.405346	0
**212**	4-O8W-G5-	10000	PI3K	0.188119	0.619
**213**	4-O8W-G1‹	100	PI3K	0.189901	0.724
**214**	4-O8W-G2(	10000	PI3K	0.04318	1
**215**	4-O8W-H2-	250	PI3K	0.222523	0.126
**216**	4-O8W-H5-	1000	PI3K	0.091176	0.985
**217**	4-O8W-H1‹	10	PI3K	0.424295	0
**218**	4-O8W-H2(	1000	PI3K	0.354978	0
**219**	4-O8W-I2-1	25	PI3K	0.336846	0.001
**220**	4-O8W-I5-‹	100	PI3K	0.408747	0
**221**	4-O8W-I14	1	PI3K	0.366302	0
**222**	4-O8W-I20	100	PI3K	0.355123	0
**223**	4-O8W-J2-1	2.5	PI3K	0.298301	0.001
**224**	4-O8W-J5-‹	10	PI3K	0.315734	0.025
**225**	4-O8W-J20	10	PI3K	0.23456	0.09
**226**	4-O8W-K2-	0.25	PI3K	0.377372	0
**227**	4-O8W-K4-	0.1	PI3K	0.414002	0
**228**	4-O8W-K5-	1	PI3K	0.334023	0.002
**229**	4-O8W-K1‹	0.1	PI3K	0.443418	0
**230**	4-O8W-K2(	1	PI3K	0.355395	0
**231**	4-O8W-L4-|	1	PI3K	0.334828	0.001
**232**	4-O8W-L14	0.1	PI3K	0.315813	0
**233**	4-O8W-L1‹	1	PI3K	0.429128	0
**234**	4-O8W-L21	0.1	PI3K	0.31351	0
**235**	4-O8W-M1	1	PI3K	0.2991	0.013
**236**	4-O8W-M1	10	PI3K	0.363752	0
**237**	4-O8W-M2	1	PI3K	0.267113	0.003
**238**	4-O8W-N4-	10	PI3K	0.438039	0
**239**	4-O8W-N1‹	10	PI3K	0.432969	0
**240**	4-O8W-N1‹	100	PI3K	0.257621	0

**241**	4-O8W-N2	10	PI3K	0.253698	0.066
**242**	4-O8W-O4-	100	PI3K	0.367768	0.005
**243**	4-O8W-O1	100	PI3K	0.435327	0
**244**	4-O8W-O1	1000	PI3K	0.431838	0
**245**	4-O8W-O2	100	PI3K	0.474206	0
**246**	4-O8W-P4-	1000	PI3K	0.275886	0.406
**247**	4-O8W-P1	1000	PI3K	0.228749	0.582
**248**	4-O8W-P1	10000	PI3K	0.430377	0
**249**	4-O8W-P21	1000	PI3K	0.233715	0.237
**250**	5-O8W-A6-	2500	PI3K	0.169805	0.884
**251**	5-O8W-A7-	1000	PI3K	0.361113	0
**252**	5-O8W-A1	2500	PI3K	0.398299	0
**253**	5-O8W-A1	10000	PI3K	0.28878	0.122
**254**	5-O8W-B6-	250	PI3K	0.422389	0
**255**	5-O8W-B7-	100	PI3K	0.395108	0
**256**	5-O8W-B1	1000	PI3K	0.094263	0.971
**257**	5-O8W-C6-	25	PI3K	0.366732	0
**258**	5-O8W-C7-	10	PI3K	0.117698	0.965
**259**	5-O8W-C1	250	PI3K	0.436097	0
**260**	5-O8W-C1	100	PI3K	0.113326	0.946
**261**	5-O8W-D6-	2.5	PI3K	0.331593	0
**262**	5-O8W-D7-	1	PI3K	0.397076	0
**263**	5-O8W-D1	25	PI3K	0.232345	0.405
**264**	5-O8W-D1	10	PI3K	0.384403	0
**265**	5-O8W-E6-	0.25	PI3K	0.108698	0.965
**266**	5-O8W-E7-	0.1	PI3K	0.17742	0.614
**267**	5-O8W-E1	2.5	PI3K	0.106297	0.947
**268**	5-O8W-E17	1	PI3K	0.064035	0.993
**269**	5-O8W-F11	10000	PI3K	0.214082	0.193
**270**	5-O8W-F1	0.25	PI3K	0.287303	0.01
**271**	5-O8W-G9-	10000	PI3K	0.499849	0
**272**	5-O8W-G1	1000	PI3K	0.310563	0.002
**273**	5-O8W-H9-	1000	PI3K	0.320661	0
**274**	5-O8W-H1	100	PI3K	0.397954	0
**275**	5-O8W-I9-	100	PI3K	0.327144	0

276	5-O8W-I11	10	PI3K	0.308147	0
277	5-O8W-J9-!	10	PI3K	0.374821	0
278	5-O8W-J11	1	PI3K	0.277827	0.001
279	5-O8W-K9-	1	PI3K	0.310899	0
280	5-O8W-L14	0.1	PI3K	0.287418	0.021
281	5-O8W-L2(	1	PI3K	0.335559	0.002
282	5-O8W-L23	0.1	PI3K	0.091253	0.985
283	5-O8W-M1	1	PI3K	0.435584	0
284	5-O8W-M2	10	PI3K	0.303494	0.185
285	5-O8W-M2	1	PI3K	0.23704	0.056
286	5-O8W-N1	10	PI3K	0.462851	0
287	5-O8W-N2(	100	PI3K	0.207509	0.237
288	5-O8W-N2:	10	PI3K	0.425045	0
289	5-O8W-O1	100	PI3K	0.487917	0
290	5-O8W-O2(	1000	PI3K	0.437755	0
291	5-O8W-O2:	100	PI3K	0.100244	0.983
292	5-O8W-P1	1000	PI3K	0.36608	0.002
293	5-O8W-P2(	10000	PI3K	0.154577	0.829
294	5-O8W-P2:	1000	PI3K	0.130662	0.969
295	6-O8W-A8-	10000	PI3K	0.341711	0
296	6-O8W-B8-	1000	PI3K	0.029509	1
297	6-O8W-C8-	100	PI3K	0.140695	0.881
298	6-O8W-D8-	10	PI3K	0.331852	0
299	6-O8W-E8-	1	PI3K	0.349923	0
300	6-O8W-L6-	1	PI3K	0.262837	0.003
301	6-O8W-M6	10	PI3K	0.336327	0
302	6-O8W-N6-	100	PI3K	0.217401	0.035
303	6-O8W-O6-	1000	PI3K	0.448756	0
304	6-O8W-P6-	10000	PI3K	0.025929	1
305	1-O8W-F11	10000	Topoisome	0.557284	0
306	1-O8W-G1:	1000	Topoisome	0.540074	0
307	1-O8W-G2(	1000	Topoisome	0.463874	0.002
308	1-O8W-H1:	100	Topoisome	0.516997	0
309	1-O8W-H2(	100	Topoisome	0.54334	0
310	1-O8W-I11	10	Topoisome	0.536185	0

311	1-O8W-I20	10	Topoisome	0.08957	0.985
312	1-O8W-J11	1	Topoisome	0.151274	0.69
313	1-O8W-J20	1	Topoisome	0.169172	0.713
314	1-O8W-K1	1	Topoisome	0.299003	0.111
315	1-O8W-K2(	0.1	Topoisome	0.036837	1
316	1-O8W-L11	10	Topoisome	0.567291	0
317	1-O8W-L14	1	Topoisome	0.297991	0.024
318	1-O8W-M1	100	Topoisome	0.466866	0
319	1-O8W-M1	10	Topoisome	0.316819	0.063
320	1-O8W-N1	100	Topoisome	0.595093	0
321	1-O8W-O1	1000	Topoisome	0.44382	0.001
322	1-O8W-O1	1000	Topoisome	0.557319	0
323	1-O8W-P1	10000	Topoisome	0.390458	0.003
324	1-O8W-P1	10000	Topoisome	0.426899	0.001
325	3-O8W-A1	10000	Topoisome	0.500531	0
326	3-O8W-B1	1000	Topoisome	0.618754	0
327	3-O8W-C1	100	Topoisome	0.627366	0
328	3-O8W-D1	10	Topoisome	0.597471	0
329	3-O8W-E11	1	Topoisome	0.235816	0.197
330	3-O8W-G9-	1000	Topoisome	0.391527	0.002
331	3-O8W-G1(	10000	Topoisome	0.588843	0
332	3-O8W-H9-	100	Topoisome	0.516561	0
333	3-O8W-H1(	1000	Topoisome	0.516891	0
334	3-O8W-I9-[	10	Topoisome	0.278969	0.075
335	3-O8W-I10	100	Topoisome	0.512535	0
336	3-O8W-J9-I	1	Topoisome	0.600611	0
337	3-O8W-J10	10	Topoisome	0.622036	0
338	3-O8W-K7-	0.1	Topoisome	0.597284	0
339	3-O8W-K9-	0.1	Topoisome	0.04893	0.99
340	3-O8W-K1(	1	Topoisome	0.44329	0
341	3-O8W-L6-I	0.1	Topoisome	0.506215	0
342	3-O8W-L7-I	1	Topoisome	0.29091	0.091
343	3-O8W-L9-	0.5	Topoisome	0.642815	0
344	3-O8W-L1C	0.1	Topoisome	0.364952	0.015
345	3-O8W-L16	1	Topoisome	0.53696	0

**346**	3-O8W-M6	1	Topoisome	0.526215	0
**347**	3-O8W-M7	10	Topoisome	0.578904	0
**348**	3-O8W-M9	5	Topoisome	0.554466	0
**349**	3-O8W-M1	1	Topoisome	0.472006	0
**350**	3-O8W-M1	10	Topoisome	0.672593	0
**351**	3-O8W-N6-	10	Topoisome	0.091983	0.895
**352**	3-O8W-N9-	50	Topoisome	0.483916	0
**353**	3-O8W-N1(	10	Topoisome	0.66761	0
**354**	3-O8W-N1(	100	Topoisome	0.059148	0.996
**355**	3-O8W-O6-	100	Topoisome	0.459501	0
**356**	3-O8W-O7-	100	Topoisome	0.514098	0
**357**	3-O8W-O9-	500	Topoisome	0.541371	0
**358**	3-O8W-O1(	100	Topoisome	0.558839	0
**359**	3-O8W-O1(	1000	Topoisome	0.119943	0.982
**360**	3-O8W-P6-	1000	Topoisome	0.471867	0
**361**	3-O8W-P7-	1000	Topoisome	0.426448	0
**362**	3-O8W-P9-	5000	Topoisome	0.493433	0
**363**	3-O8W-P1(	1000	Topoisome	0.53617	0
**364**	3-O8W-P1(	10000	Topoisome	0.624672	0
**365**	1-O8W-A1(	10000	Mitotic	0.53689	0
**366**	1-O8W-A1:	1000	Mitotic	0.507795	0
**367**	1-O8W-A1{	1000	Mitotic	0.522958	0
**368**	1-O8W-B1(	1000	Mitotic	0.569766	0
**369**	1-O8W-B1:	100	Mitotic	0.144457	0.787
**370**	1-O8W-B1{	100	Mitotic	0.560866	0
**371**	1-O8W-C1(	100	Mitotic	0.521263	0
**372**	1-O8W-C1:	10	Mitotic	0.647063	0
**373**	1-O8W-C1{	10	Mitotic	0.536171	0
**374**	1-O8W-D1(	10	Mitotic	0.494627	0
**375**	1-O8W-D1:	1	Mitotic	0.132987	0.817
**376**	1-O8W-D1{	1	Mitotic	0.426897	0.022
**377**	1-O8W-E1C	1	Mitotic	0.475704	0
**378**	1-O8W-E13	0.1	Mitotic	0.486623	0.001
**379**	1-O8W-E18	0.1	Mitotic	0.483726	0.001
**380**	1-O8W-F13	1000	Mitotic	0.530264	0

**381**	1-O8W-G1:	100	Mitotic	0.524659	0
**382**	1-O8W-G1!	1000	Mitotic	0.563465	0
**383**	1-O8W-H1:	10	Mitotic	0.533245	0
**384**	1-O8W-H1!	100	Mitotic	0.493464	0
**385**	1-O8W-I13	1	Mitotic	0.423017	0.053
**386**	1-O8W-I15	10	Mitotic	0.518518	0
**387**	1-O8W-J13	0.1	Mitotic	0.180871	0.581
**388**	1-O8W-J15	1	Mitotic	0.505406	0
**389**	1-O8W-K7-	0.1	Mitotic	0.456101	0.006
**390**	1-O8W-K1!	0.1	Mitotic	0.082821	0.966
**391**	1-O8W-L7-'	1	Mitotic	0.177599	0.736
**392**	1-O8W-L2(	0.1	Mitotic	0.042106	1
**393**	1-O8W-M7	10	Mitotic	0.104521	0.899
**394**	1-O8W-M2	1	Mitotic	0.357529	0.346
**395**	1-O8W-N2(	10	Mitotic	0.450165	0.009
**396**	1-O8W-O7-	100	Mitotic	0.555829	0
**397**	1-O8W-O2(	100	Mitotic	0.12969	0.925
**398**	1-O8W-P7-	1000	Mitotic	0.518526	0
**399**	1-O8W-P2(	1000	Mitotic	0.51079	0
**400**	3-O8W-A7-	1000	Mitotic	0.511147	0.001
**401**	3-O8W-B7-	100	Mitotic	0.505214	0
**402**	3-O8W-C7-	10	Mitotic	0.511521	0.001
**403**	3-O8W-D7-	1	Mitotic	0.418726	0.02
**404**	3-O8W-E7-I	0.1	Mitotic	0.390951	0.028
**405**	6-O8W-L1!	1	Mitotic	0.177593	0.696
**406**	6-O8W-M1	10	Mitotic	0.14405	0.915
**407**	6-O8W-N1!	100	Mitotic	0.09797	0.886
**408**	6-O8W-O1!	1000	Mitotic	0.55019	0
**409**	6-O8W-P1!	10000	Mitotic	0.437815	0.002
**410**	2-O8W-A1:	250	MEK1/2	0.390377	0.161
**411**	2-O8W-B1:	25	MEK1/2	0.664098	0
**412**	2-O8W-D1:	2.5	MEK1/2	0.705994	0
**413**	2-O8W-E12	0.25	MEK1/2	0.660185	0
**414**	2-O8W-F12	2.5000000	MEK1/2	0.304434	0.296
**415**	2-O8W-F14	1000	MEK1/2	0.497168	0.026

**416**	2-O8W-G14	100	MEK1/2	0.689784	0
**417**	2-O8W-H14	10	MEK1/2	0.652456	0
**418**	2-O8W-I14	1	MEK1/2	0.40942	0.129
**419**	2-O8W-K14	0.1	MEK1/2	0.344879	0.248
**420**	2-O8W-L20	1	MEK1/2	0.099069	0.982
**421**	2-O8W-M2	10	MEK1/2	0.181184	0.899
**422**	2-O8W-N20	100	MEK1/2	0.564044	0
**423**	2-O8W-O20	1000	MEK1/2	0.594976	0
**424**	2-O8W-P20	10000	MEK1/2	0.616221	0
**425**	4-O8W-A10	1000	MEK1/2	0.648386	0
**426**	4-O8W-A13	1000	MEK1/2	0.725392	0
**427**	4-O8W-B10	100	MEK1/2	0.745287	0
**428**	4-O8W-B13	100	MEK1/2	0.715522	0
**429**	4-O8W-C10	10	MEK1/2	0.700316	0
**430**	4-O8W-C13	10	MEK1/2	0.645337	0
**431**	4-O8W-D10	1	MEK1/2	0.532178	0
**432**	4-O8W-D13	1	MEK1/2	0.661255	0
**433**	4-O8W-E10	0.1	MEK1/2	0.15652	0.944
**434**	4-O8W-E13	0.1	MEK1/2	0.303033	0.352
**435**	4-O8W-L19	0.25	MEK1/2	0.523394	0
**436**	4-O8W-M1	2.5	MEK1/2	0.41208	0.053
**437**	4-O8W-N19	25	MEK1/2	0.182725	0.848
**438**	4-O8W-O19	250	MEK1/2	0.604363	0
**439**	4-O8W-P19	2500	MEK1/2	0.681231	0
**440**	1-O8W-L2-	1	PARP	0.246983	0.542
**441**	1-O8W-L6-	1	PARP	0.188492	0.839
**442**	1-O8W-M2	10	PARP	0.297352	0.586
**443**	1-O8W-M6	10	PARP	0.174191	0.898
**444**	1-O8W-N2-	100	PARP	0.363285	0.161
**445**	1-O8W-N6-	100	PARP	0.211563	0.564
**446**	1-O8W-O2-	1000	PARP	0.567968	0.033
**447**	1-O8W-O6-	1000	PARP	0.436078	0.034
**448**	1-O8W-P2-	10000	PARP	0.671608	0
**449**	1-O8W-P6-	10000	PARP	0.596062	0
**450**	7-O8W-A3-	1000	PARP	0.713914	0

**451**	7-O8W-B2-	10000	PARP	0.644152	0
**452**	7-O8W-B3-	100	PARP	0.647516	0.004
**453**	7-O8W-C2-	1000	PARP	0.426537	0.074
**454**	7-O8W-C3-	10	PARP	0.723529	0
**455**	7-O8W-D2-	100	PARP	0.566949	0
**456**	7-O8W-D3-	1	PARP	0.601868	0.001
**457**	7-O8W-E2-'	10	PARP	0.316418	0.313
**458**	7-O8W-E3-'	0.1	PARP	0.378822	0.125
**459**	7-O8W-F2-'	1	PARP	0.115142	1
**460**	7-O8W-G2-	10000	PARP	0.712599	0
**461**	7-O8W-H2-	1000	PARP	0.583209	0.006
**462**	7-O8W-I2-N	100	PARP	0.642739	0
**463**	7-O8W-J2-I	10	PARP	0.340183	0.342
**464**	7-O8W-K2-	1	PARP	0.501281	0.012
**465**	3-O8W-A19	1000	CDK	0.313547	0.076
**466**	3-O8W-B19	100	CDK	0.275583	0.268
**467**	3-O8W-B23	2500	CDK	0.456905	0.006
**468**	3-O8W-C19	10	CDK	0.372949	0.08
**469**	3-O8W-C23	250	CDK	0.327543	0.065
**470**	3-O8W-D19	1	CDK	0.261057	0.112
**471**	3-O8W-D23	25	CDK	0.233813	0.528
**472**	3-O8W-E19	0.1	CDK	0.44702	0.017
**473**	3-O8W-E23	2.5	CDK	0.306921	0.41
**474**	3-O8W-F23	0.25	CDK	0.192339	0.563
**475**	3-O8W-K17	1	CDK	0.218284	0.42
**476**	3-O8W-L19	1	CDK	0.259464	0.217
**477**	3-O8W-M1	10	CDK	0.319684	0.218
**478**	3-O8W-M1	10	CDK	0.229615	0.319
**479**	3-O8W-N17	100	CDK	0.253629	0.062
**480**	3-O8W-N19	100	CDK	0.269663	0.096
**481**	3-O8W-O17	1000	CDK	0.382474	0.016
**482**	3-O8W-O19	1000	CDK	0.479527	0
**483**	3-O8W-P17	10000	CDK	0.305319	0.222
**484**	3-O8W-P19	10000	CDK	0.501571	0.003
**485**	4-O8W-A4-	10000	CDK	0.330902	0.043

**486**	4-O8W-A8-	10000	CDK	0.328848	0.04
**487**	4-O8W-B4-	1000	CDK	0.33879	0.04
**488**	4-O8W-B8-	1000	CDK	0.183542	0.71
**489**	4-O8W-C4-	100	CDK	0.193005	0.605
**490**	4-O8W-C8-	100	CDK	0.210818	0.775
**491**	4-O8W-D4-	10	CDK	0.223013	0.504
**492**	4-O8W-D8-	10	CDK	0.184274	0.622
**493**	4-O8W-E4-[illegible]	1	CDK	0.13483	0.844
**494**	4-O8W-E8-[illegible]	1	CDK	0.135878	0.823
**495**	4-O8W-F4-[illegible]	10000	CDK	0.41358	0.08
**496**	4-O8W-F22	10000	CDK	0.340945	0.023
**497**	4-O8W-G4-	1000	CDK	0.374071	0.015
**498**	4-O8W-G2[illegible]	1000	CDK	0.291761	0.183
**499**	4-O8W-H4-	100	CDK	0.305398	0.133
**500**	4-O8W-H2[illegible]	100	CDK	0.375108	0.013
**501**	4-O8W-I4-[illegible]	10	CDK	0.15429	0.759
**502**	4-O8W-I22	10	CDK	0.34465	0.071
**503**	4-O8W-J4-[illegible]	1	CDK	0.24984	0.546
**504**	4-O8W-J22	1	CDK	0.304657	0.175
**505**	5-O8W-A1[illegible]	10000	CDK	0.323994	0.068
**506**	5-O8W-B1[illegible]	1000	CDK	0.270417	0.276
**507**	5-O8W-C1[illegible]	100	CDK	0.37378	0.141
**508**	5-O8W-D1[illegible]	10	CDK	0.326749	0.414
**509**	5-O8W-E1[illegible]	1	CDK	0.216794	0.818
**510**	5-O8W-K1[illegible]	1	CDK	0.079477	0.895
**511**	5-O8W-M1	10	CDK	0.108343	0.78
**512**	5-O8W-N1[illegible]	100	CDK	0.219876	0.327
**513**	5-O8W-O1[illegible]	1000	CDK	0.307403	0.136
**514**	5-O8W-P1[illegible]	10000	CDK	0.29731	0.116
**515**	6-O8W-A1[illegible]	1000	CDK	0.132033	0.819
**516**	6-O8W-B1[illegible]	100	CDK	0.07997	0.985
**517**	6-O8W-C1[illegible]	10	CDK	0.231323	0.377
**518**	6-O8W-D1[illegible]	1	CDK	0.299986	0.32
**519**	6-O8W-E17	0.1	CDK	0.220625	0.396
**520**	6-O8W-L1[illegible]	1	CDK	0.123324	0.809

**521**	6-O8W-M1 10	CDK	0.271926	0.1
**522**	6-O8W-N1! 100	CDK	0.279199	0.14
**523**	6-O8W-O1! 1000	CDK	0.268131	0.215
**524**	6-O8W-P1! 10000	CDK	0.330208	0.054
**525**	7-O8W-A21 10000	BET	0.35801	0.075
**526**	7-O8W-A22 30000	BET	0.58943	0
**527**	7-O8W-B21 1000	BET	0.391529	0.15
**528**	7-O8W-B22 3000	BET	0.369472	0.004
**529**	7-O8W-C21 100	BET	0.474768	0
**530**	7-O8W-C22 300	BET	0.32546	0.102
**531**	7-O8W-D2: 10	BET	0.157236	0.917
**532**	7-O8W-D2: 30	BET	0.19585	0.604
**533**	7-O8W-E21 1	BET	0.239078	0.191
**534**	7-O8W-E22 3	BET	0.230868	0.175
**535**	7-O8W-G1(10000	BET	0.384007	0.061
**536**	7-O8W-G1! 10000	BET	0.537067	0
**537**	7-O8W-H1(1000	BET	0.47899	0.011
**538**	7-O8W-H1! 1000	BET	0.485137	0
**539**	7-O8W-I10 100	BET	0.207597	0.362
**540**	7-O8W-I15 100	BET	0.292696	0.074
**541**	7-O8W-J10 10	BET	0.234238	0.406
**542**	7-O8W-J15 10	BET	0.19107	0.601
**543**	7-O8W-K1(1	BET	0.303766	0.128
**544**	7-O8W-K1: 1	BET	0.396043	0.018
**545**	7-O8W-K1! 1	BET	0.234392	0.175
**546**	7-O8W-L12 1	BET	0.328899	0.106
**547**	7-O8W-L13 10	BET	0.62463	0
**548**	7-O8W-L2(1	BET	0.269898	0.171
**549**	7-O8W-L23 0.03	BET	0.368935	0.02
**550**	7-O8W-M1 10	BET	0.1411	0.791
**551**	7-O8W-M1 100	BET	0.535356	0
**552**	7-O8W-M2 10	BET	0.432849	0.032
**553**	7-O8W-M2 0.3	BET	0.425003	0.003
**554**	7-O8W-N1: 100	BET	0.531477	0.004
**555**	7-O8W-N1: 1000	BET	0.475282	0.004

**556**	7-O8W-N2(	100	BET	0.322437	0.053
**557**	7-O8W-N2:	3	BET	0.251598	0.228
**558**	7-O8W-O1:	1000	BET	0.435972	0.033
**559**	7-O8W-O2(	1000	BET	0.44721	0.015
**560**	7-O8W-O2:	30	BET	0.402879	0.028
**561**	7-O8W-P1:	10000	BET	0.542008	0
**562**	7-O8W-P1:	10000	BET	0.337087	0.126
**563**	7-O8W-P2(	10000	BET	0.467465	0.002
**564**	7-O8W-P2:	300	BET	0.341641	0.173
**565**	8-O8W-K2:	1	BET	0.199415	0.426
**566**	8-O8W-L22	10	BET	0.264759	0.422
**567**	8-O8W-M2	100	BET	0.255585	0.305
**568**	8-O8W-N2:	1000	BET	0.575167	0
**569**	8-O8W-O2:	10000	BET	0.418294	0.031
**570**	1-O8W-A3-	10000	HDAC	0.343536	0.006
**571**	1-O8W-B3-	1000	HDAC	0.444012	0.001
**572**	1-O8W-C3-	100	HDAC	0.144127	0.962
**573**	1-O8W-D3-	10	HDAC	0.301903	0.056
**574**	1-O8W-E3-'	1	HDAC	0.341699	0.004
**575**	1-O8W-L12	0.1	HDAC	0.037824	0.997
**576**	1-O8W-M1	1	HDAC	0.158141	0.556
**577**	1-O8W-N1:	10	HDAC	0.426476	0
**578**	1-O8W-O1:	100	HDAC	0.36571	0.005
**579**	1-O8W-P1:	1000	HDAC	0.357365	0
**580**	3-O8W-A4-	1000	HDAC	0.433567	0
**581**	3-O8W-B4-	100	HDAC	0.350575	0.043
**582**	3-O8W-C4-	10	HDAC	0.388358	0.016
**583**	3-O8W-D4-	1	HDAC	0.216698	0.644
**584**	3-O8W-E4-I	0.1	HDAC	0.303982	0.014
**585**	3-O8W-F7-(	1000	HDAC	0.337743	0.005
**586**	3-O8W-G7-	100	HDAC	0.468102	0
**587**	3-O8W-G1:	1000000	HDAC	0.312086	0.007
**588**	3-O8W-H7-	10	HDAC	0.325054	0.042
**589**	3-O8W-H1:	100000	HDAC	0.121421	0.752
**590**	3-O8W-I7-(	1	HDAC	0.161636	0.409

**591**	3-O8W-I12	10000	HDAC	0.211185	0.166
**592**	3-O8W-J7-(	0.1	HDAC	0.366101	0
**593**	3-O8W-J12	1000	HDAC	0.197492	0.304
**594**	3-O8W-K3-	1	HDAC	0.070319	0.997
**595**	3-O8W-K12	100	HDAC	0.211893	0.344
**596**	3-O8W-L3-I	10	HDAC	0.233127	0.3
**597**	3-O8W-M3	100	HDAC	0.313676	0.201
**598**	3-O8W-N3-	1000	HDAC	0.375236	0.007
**599**	3-O8W-O3-	10000	HDAC	0.308716	0.011
**600**	7-O8W-A5-	10000	HDAC	0.337181	0.007
**601**	7-O8W-A7-	10000	HDAC	0.369278	0.001
**602**	7-O8W-A9-	1000	HDAC	0.428502	0.001
**603**	7-O8W-A12	10000	HDAC	0.395431	0
**604**	7-O8W-B5-	1000	HDAC	0.478752	0
**605**	7-O8W-B7-	1000	HDAC	0.389001	0
**606**	7-O8W-B12	1000	HDAC	0.43859	0
**607**	7-O8W-C5-	100	HDAC	0.41931	0
**608**	7-O8W-C7-	100	HDAC	0.363212	0
**609**	7-O8W-C9-	100	HDAC	0.415355	0.003
**610**	7-O8W-D7-	10	HDAC	0.514779	0
**611**	7-O8W-D9-	10	HDAC	0.168356	0.314
**612**	7-O8W-D12	100	HDAC	0.108568	0.845
**613**	7-O8W-E5-I	10	HDAC	0.403838	0
**614**	7-O8W-E7-(	1	HDAC	0.432277	0.004
**615**	7-O8W-E9-(	1	HDAC	0.111273	0.583
**616**	7-O8W-E12	10	HDAC	0.132587	0.653
**617**	7-O8W-F5-I	1	HDAC	0.45395	0
**618**	7-O8W-F7-I	10000	HDAC	0.369238	0.001
**619**	7-O8W-F9-(	0.1	HDAC	0.278487	0.054
**620**	7-O8W-F12	1	HDAC	0.384548	0
**621**	7-O8W-F19	10000	HDAC	0.349314	0.006
**622**	7-O8W-G7-	1000	HDAC	0.49084	0
**623**	7-O8W-G19	1000	HDAC	0.371012	0.001
**624**	7-O8W-H7-	100	HDAC	0.052754	0.994
**625**	7-O8W-I7-F	10	HDAC	0.357232	0

**626**	7-O8W-I19	100	HDAC	0.109195	0.682
**627**	7-O8W-J7-	1	HDAC	0.469692	0
**628**	7-O8W-J19	10	HDAC	0.113486	0.72
**629**	7-O8W-K4-	1	HDAC	0.156868	0.869
**630**	7-O8W-K11	1	HDAC	0.088133	0.855
**631**	7-O8W-K18	1	HDAC	0.02686	0.995
**632**	7-O8W-K19	1	HDAC	0.12998	0.735
**633**	7-O8W-L2-	0.1	HDAC	0.045474	0.993
**634**	7-O8W-L4-	10	HDAC	0.060489	0.95
**635**	7-O8W-L5-	1	HDAC	0.113754	0.758
**636**	7-O8W-L8-	1	HDAC	0.205523	0.304
**637**	7-O8W-L10	1	HDAC	0.309708	0.01
**638**	7-O8W-L11	10	HDAC	0.197285	0.213
**639**	7-O8W-L14	1	HDAC	0.305335	0.015
**640**	7-O8W-L16	1	HDAC	0.06323	0.94
**641**	7-O8W-L18	10	HDAC	0.065322	0.989
**642**	7-O8W-M2	1	HDAC	0.163114	0.496
**643**	7-O8W-M5	10	HDAC	0.352757	0.005
**644**	7-O8W-M8	10	HDAC	0.365879	0.001
**645**	7-O8W-M1	10	HDAC	0.349909	0.003
**646**	7-O8W-M1	100	HDAC	0.262174	0.065
**647**	7-O8W-M1	10	HDAC	0.342954	0.037
**648**	7-O8W-M1	10	HDAC	0.385013	0
**649**	7-O8W-M1	100	HDAC	0.269274	0.239
**650**	7-O8W-N2-	10	HDAC	0.431273	0
**651**	7-O8W-N4-	100	HDAC	0.266114	0.053
**652**	7-O8W-N5-	100	HDAC	0.472102	0
**653**	7-O8W-N8-	100	HDAC	0.353573	0.006
**654**	7-O8W-N10	100	HDAC	0.425697	0
**655**	7-O8W-N14	100	HDAC	0.288388	0.058
**656**	7-O8W-N16	100	HDAC	0.350618	0
**657**	7-O8W-N18	1000	HDAC	0.263236	0.04
**658**	7-O8W-O2-	100	HDAC	0.414287	0
**659**	7-O8W-O4-	1000	HDAC	0.473954	0
**660**	7-O8W-O5-	1000	HDAC	0.39439	0

**661**	7-O8W-O8-	1000	HDAC	0.394179	0.001
**662**	7-O8W-O1(	1000	HDAC	0.432705	0.002
**663**	7-O8W-O1:	1000	HDAC	0.450805	0
**664**	7-O8W-O1‹	1000	HDAC	0.49596	0
**665**	7-O8W-O1(	1000	HDAC	0.435879	0
**666**	7-O8W-P2-	1000	HDAC	0.163054	0.463
**667**	7-O8W-P4-	10000	HDAC	0.408346	0
**668**	7-O8W-P5-	10000	HDAC	0.420482	0
**669**	7-O8W-P8-	10000	HDAC	0.38604	0.004
**670**	7-O8W-P1(	10000	HDAC	0.40655	0.001
**671**	7-O8W-P1:	10000	HDAC	0.336979	0.002
**672**	7-O8W-P1‹	10000	HDAC	0.489013	0
**673**	7-O8W-P1(	10000	HDAC	0.454866	0
**674**	7-O8W-P1{	10000	HDAC	0.39471	0
