# Supplementary material for: Evaluating feature extraction in ovarian cancer cell line co-cultures using deep neural networks
Source: Commun Biol. 2025 Feb 25;8:303. doi: 10.1038/s42003-025-07766-w (PMC11862010; doi:10.1038/s42003-025-07766-w)
Supplement: Supplementary file 9 — Supplementary Data 7 [file 42003_2025_7766_MOESM9_ESM.pdf]

	Well_annotati	_concentra	ell_Catego	Highest_ES	Pvalue
**0**	2-KB-A16-G	10000	EGFR	0.274026	0
**1**	2-KB-A19-E	10000	EGFR	0.177613	0.072
**2**	2-KB-B19-E	1000	EGFR	0.135688	0.311
**3**	2-KB-C16-G	1000	EGFR	0.259275	0.001
**4**	2-KB-C19-E	100	EGFR	0.199833	0.035
**5**	2-KB-D16-G	100	EGFR	0.136872	0.292
**6**	2-KB-D19-E	10	EGFR	0.153407	0.162
**7**	2-KB-E16-G	10	EGFR	0.173473	0.083
**8**	2-KB-E19-E	1	EGFR	0.144499	0.248
**9**	2-KB-F16-G	1	EGFR	0.222092	0.007
**10**	2-KB-K11-A	0.1	EGFR	0.14937	0.19
**11**	2-KB-L11-A	1	EGFR	0.175032	0.083
**12**	2-KB-L16-O	0.25	EGFR	0.199655	0.026
**13**	2-KB-L19-L	0.1	EGFR	0.225026	0.005
**14**	2-KB-M11-A	10	EGFR	0.170583	0.084
**15**	2-KB-M16-O	2.5	EGFR	0.210316	0.022
**16**	2-KB-M19-L	1	EGFR	0.214578	0.008
**17**	2-KB-N16-O	25	EGFR	0.190518	0.042
**18**	2-KB-N19-L	10	EGFR	0.242973	0.002
**19**	2-KB-O11-A	100	EGFR	0.252724	0.001
**20**	2-KB-O16-O	250	EGFR	0.276852	0.001
**21**	2-KB-O19-L	100	EGFR	0.23756	0.002
**22**	2-KB-P11-A	1000	EGFR	0.269009	0
**23**	2-KB-P16-C	2500	EGFR	0.227857	0.001
**24**	2-KB-P19-L	1000	EGFR	0.161093	0.117
**25**	3-KB-F21-R	10000	EGFR	0.153781	0.21
**26**	3-KB-G20-N	1000	EGFR	0.235005	0.002
**27**	3-KB-G21-R	1000	EGFR	0.286496	0.001
**28**	3-KB-H20-N	100	EGFR	0.294286	0
**29**	3-KB-H21-R	100	EGFR	0.23537	0.002
**30**	3-KB-I20-N	10	EGFR	0.191545	0.025
**31**	3-KB-I21-R	10	EGFR	0.165426	0.118
**32**	3-KB-J20-N	1	EGFR	0.263974	0
**33**	3-KB-J21-R	1	EGFR	0.294581	0

**34**	3-KB-K4-Ca	1	EGFR	0.1358	0.295
**35**	3-KB-K18-D	0.1	EGFR	0.171983	0.084
**36**	3-KB-K20-N	0.1	EGFR	0.20952	0.013
**37**	3-KB-L4-Ca	10	EGFR	0.133372	0.347
**38**	3-KB-L18-D	1	EGFR	0.143977	0.218
**39**	3-KB-M18-I	10	EGFR	0.291701	0
**40**	3-KB-N4-Ca	100	EGFR	0.180627	0.056
**41**	3-KB-N18-D	100	EGFR	0.299994	0
**42**	3-KB-O4-Ca	1000	EGFR	0.236414	0.005
**43**	3-KB-P4-Ca	10000	EGFR	0.195302	0.955
**44**	3-KB-P18-D	1000	EGFR	0.286102	0
**45**	4-KB-F13-Sa	1000	EGFR	0.232989	0.001
**46**	4-KB-G13-S	100	EGFR	0.253365	0
**47**	4-KB-G16-V	10000	EGFR	0.255313	0
**48**	4-KB-H13-S	10	EGFR	0.164267	0.116
**49**	4-KB-H16-V	1000	EGFR	0.142828	0.265
**50**	4-KB-I13-Sa	1	EGFR	0.159094	0.128
**51**	4-KB-I16-Va	100	EGFR	0.136141	0.312
**52**	4-KB-J13-Sa	0.1	EGFR	0.135499	0.292
**53**	4-KB-J16-Va	10	EGFR	0.135908	0.285
**54**	4-KB-K7-Ico	1	EGFR	0.166128	0.112
**55**	4-KB-K13-T	0.1	EGFR	0.135754	0.35
**56**	4-KB-K16-V	1	EGFR	0.168272	0.102
**57**	4-KB-L7-Ico	10	EGFR	0.1536	0.189
**58**	4-KB-L13-T	1	EGFR	0.136612	0.289
**59**	4-KB-M7-Ic	100	EGFR	0.171888	0.094
**60**	4-KB-M13-T	10	EGFR	0.137115	0.313
**61**	4-KB-N13-T	100	EGFR	0.181639	0.061
**62**	4-KB-O7-Ico	1000	EGFR	0.163935	0.115
**63**	4-KB-P7-Ico	10000	EGFR	0.136425	0.328
**64**	4-KB-P13-T	1000	EGFR	0.268981	0
**65**	5-KB-F4-Po	1000	EGFR	0.295786	0
**66**	5-KB-F7-AZ	1000	EGFR	0.219514	0.006
**67**	5-KB-G4-Po	100	EGFR	0.180305	0.061
**68**	5-KB-G7-AZ	100	EGFR	0.158013	0.159

**69**	5-KB-H4-Pc	10	EGFR	0.198416	0.034
**70**	5-KB-H7-AZ	10	EGFR	0.153152	0.198
**71**	5-KB-I4-Po:	1	EGFR	0.135239	0.317
**72**	5-KB-I7-AZI	1	EGFR	0.135628	0.314
**73**	5-KB-J4-Po:	0.1	EGFR	0.133521	0.339
**74**	5-KB-J7-AZI	0.1	EGFR	0.13359	0.338
**75**	5-KB-K7-Ol	0.1	EGFR	0.134878	0.349
**76**	5-KB-L7-Ol	1	EGFR	0.135787	0.35
**77**	5-KB-M7-Ol	10	EGFR	0.135296	0.35
**78**	5-KB-O7-Ol	100	EGFR	0.138927	0.286
**79**	5-KB-P7-Ol	1000	EGFR	0.136891	0.339
**80**	2-KB-A15-L	2500	VEGFR	0.224941	0.001
**81**	2-KB-A17-N	10000	VEGFR	0.160215	0.084
**82**	2-KB-A20-T	10000	VEGFR	0.274733	0
**83**	2-KB-B15-L	250	VEGFR	0.174701	0.045
**84**	2-KB-B17-N	1000	VEGFR	0.289443	0
**85**	2-KB-B20-T	1000	VEGFR	0.170221	0.052
**86**	2-KB-C15-L	25	VEGFR	0.168909	0.054
**87**	2-KB-C17-N	100	VEGFR	0.171641	0.048
**88**	2-KB-D15-L	2.5	VEGFR	0.243833	0
**89**	2-KB-D17-N	10	VEGFR	0.16952	0.071
**90**	2-KB-D20-T	100	VEGFR	0.217206	0.007
**91**	2-KB-E17-N	1	VEGFR	0.175643	0.05
**92**	2-KB-E20-Ti	10	VEGFR	0.173493	0.057
**93**	2-KB-F13-A	10000	VEGFR	0.202077	0.004
**94**	2-KB-F15-L	0.25	VEGFR	0.171132	0.052
**95**	2-KB-F19-R	10000	VEGFR	0.173048	0.032
**96**	2-KB-F20-Ti	1	VEGFR	0.175508	0.052
**97**	2-KB-F21-V	10000	VEGFR	0.243865	0.001
**98**	2-KB-G10-A	10000	VEGFR	0.172365	0.05
**99**	2-KB-G13-A	1000	VEGFR	0.286638	0
**100**	2-KB-G19-R	1000	VEGFR	0.26457	0
**101**	2-KB-G21-V	1000	VEGFR	0.2462	0.001
**102**	2-KB-H10-A	1000	VEGFR	0.279395	0
**103**	2-KB-H13-A	100	VEGFR	0.291237	0

**104**	2-KB-H21-V	100	VEGFR	0.286705	0
**105**	2-KB-I10-A	100	VEGFR	0.176198	0.055
**106**	2-KB-I13-A	10	VEGFR	0.219948	0.004
**107**	2-KB-I19-R	100	VEGFR	0.310172	0
**108**	2-KB-I21-V	10	VEGFR	0.213986	0.008
**109**	2-KB-J10-A	10	VEGFR	0.254444	0
**110**	2-KB-J13-A	1	VEGFR	0.267226	0
**111**	2-KB-J19-R	10	VEGFR	0.23815	0
**112**	2-KB-J21-V	1	VEGFR	0.217214	0.004
**113**	2-KB-K10-A	1	VEGFR	0.171916	0.061
**114**	2-KB-K13-V	0.1	VEGFR	0.273322	0
**115**	2-KB-K17-P	1	VEGFR	0.222721	0.004
**116**	2-KB-K19-R	1	VEGFR	0.250586	0
**117**	2-KB-L12-S	0.1	VEGFR	0.19688	0.018
**118**	2-KB-L13-V	1	VEGFR	0.172832	0.066
**119**	2-KB-L21-C	0.1	VEGFR	0.213794	0.003
**120**	2-KB-M12-S	1	VEGFR	0.206544	0.003
**121**	2-KB-M13-V	10	VEGFR	0.247479	0.001
**122**	2-KB-M17-P	10	VEGFR	0.170295	0.056
**123**	2-KB-M21-C	1	VEGFR	0.289513	0
**124**	2-KB-N12-S	10	VEGFR	0.171534	0.065
**125**	2-KB-N13-V	100	VEGFR	0.250464	0.002
**126**	2-KB-N17-P	100	VEGFR	0.222393	0.004
**127**	2-KB-N21-C	10	VEGFR	0.170254	0.062
**128**	2-KB-O12-S	100	VEGFR	0.277592	0
**129**	2-KB-O17-P	1000	VEGFR	0.277971	0
**130**	2-KB-O21-C	100	VEGFR	0.298976	0
**131**	2-KB-P12-S	1000	VEGFR	0.202146	0.011
**132**	2-KB-P13-V	1000	VEGFR	0.225986	0.004
**133**	2-KB-P17-P	10000	VEGFR	0.17957	0.039
**134**	2-KB-P21-C	1000	VEGFR	0.17092	0.057
**135**	3-KB-A3-Ca	1000	VEGFR	0.24582	0
**136**	3-KB-A6-Fo	1000	VEGFR	0.187753	0.013
**137**	3-KB-A18-L	1000	VEGFR	0.172221	0.049
**138**	3-KB-B3-Ca	100	VEGFR	0.173252	0.045

**139**	3-KB-B6-Fo	100	VEGFR	0.168712	0.056
**140**	3-KB-B18-L	100	VEGFR	0.172841	0.041
**141**	3-KB-C3-Ca	10	VEGFR	0.175128	0.045
**142**	3-KB-C6-Fo	10	VEGFR	0.176632	0.036
**143**	3-KB-C18-L	10	VEGFR	0.174941	0.04
**144**	3-KB-D3-Ca	1	VEGFR	0.17272	0.058
**145**	3-KB-D6-Fo	1	VEGFR	0.173474	0.063
**146**	3-KB-D18-L	1	VEGFR	0.177452	0.047
**147**	3-KB-E3-Cal	0.1	VEGFR	0.174465	0.06
**148**	3-KB-E6-For	0.1	VEGFR	0.174901	0.038
**149**	3-KB-E18-Li	0.1	VEGFR	0.217439	0.002
**150**	3-KB-F18-B	1000	VEGFR	0.167851	0.094
**151**	3-KB-G18-E	100	VEGFR	0.167784	0.069
**152**	3-KB-H18-E	10	VEGFR	0.255148	0
**153**	3-KB-I18-Br	1	VEGFR	0.175935	0.05
**154**	3-KB-J18-Br	0.1	VEGFR	0.259827	0
**155**	4-KB-A12-E	10000	VEGFR	0.037934	0.982
**156**	4-KB-A15-G	2500	VEGFR	0.176858	0.053
**157**	4-KB-A20-N	10000	VEGFR	0.171466	0.046
**158**	4-KB-B12-E	1000	VEGFR	0.225554	0.002
**159**	4-KB-B15-G	250	VEGFR	0.17519	0.044
**160**	4-KB-B20-N	1000	VEGFR	0.175757	0.055
**161**	4-KB-C15-G	25	VEGFR	0.176193	0.044
**162**	4-KB-D12-E	100	VEGFR	0.178274	0.038
**163**	4-KB-D15-G	2.5	VEGFR	0.173581	0.064
**164**	4-KB-D20-N	100	VEGFR	0.174349	0.05
**165**	4-KB-E12-El	10	VEGFR	0.176104	0.041
**166**	4-KB-E20-M	10	VEGFR	0.176396	0.048
**167**	4-KB-F12-El	1	VEGFR	0.176011	0.043
**168**	4-KB-F15-G	0.25	VEGFR	0.190442	0.023
**169**	4-KB-F20-M	1	VEGFR	0.170247	0.052
**170**	4-KB-L16-T	1	VEGFR	0.173898	0.065
**171**	4-KB-M16-T	10	VEGFR	0.177119	0.054
**172**	4-KB-N16-T	100	VEGFR	0.175442	0.057
**173**	4-KB-O16-T	1000	VEGFR	0.171552	0.056

**174**	4-KB-P16-T	10000	VEGFR	0.167072	0.07
**175**	2-KB-L10-Ic	1	PI3K	0.092425	0.67
**176**	2-KB-M10-I	10	PI3K	0.091938	0.655
**177**	2-KB-N10-I	100	PI3K	0.115963	0.321
**178**	2-KB-O10-I	1000	PI3K	0.151182	0.062
**179**	2-KB-P10-Ic	10000	PI3K	0.180891	0.008
**180**	3-KB-A16-P	2500	PI3K	0.093027	0.669
**181**	3-KB-C16-P	250	PI3K	0.093012	0.668
**182**	3-KB-D16-F	25	PI3K	0.092916	0.627
**183**	3-KB-E16-P	2.5	PI3K	0.092902	0.692
**184**	3-KB-F16-P	0.25	PI3K	0.095721	0.714
**185**	3-KB-F17-N	100000	PI3K	0.285257	0.053
**186**	3-KB-F19-D	500	PI3K	0.182784	0.008
**187**	3-KB-G17-N	10000	PI3K	0.101836	0.489
**188**	3-KB-G19-D	50	PI3K	0.155514	0.039
**189**	3-KB-H17-N	1000	PI3K	0.121316	0.285
**190**	3-KB-I17-M	100	PI3K	0.09184	0.68
**191**	3-KB-I19-D	5	PI3K	0.115926	0.307
**192**	3-KB-J17-N	10	PI3K	0.146944	0.054
**193**	3-KB-J19-D	0.5	PI3K	0.167809	0.016
**194**	3-KB-K19-D	0.05	PI3K	0.092071	0.706
**195**	3-KB-L8-Pic	1	PI3K	0.092599	0.626
**196**	3-KB-L21-T	0.1	PI3K	0.093001	0.691
**197**	3-KB-M8-Pi	10	PI3K	0.09297	0.687
**198**	3-KB-M21-T	1	PI3K	0.09195	0.658
**199**	3-KB-N8-Pi	100	PI3K	0.147185	0.058
**200**	3-KB-N21-T	10	PI3K	0.09821	0.556
**201**	3-KB-O8-Pi	1000	PI3K	0.247465	0
**202**	3-KB-O21-T	100	PI3K	0.150964	0.056
**203**	3-KB-P8-Pic	10000	PI3K	0.219999	0
**204**	3-KB-P21-T	1000	PI3K	0.241156	0
**205**	4-KB-A19-A	2500	PI3K	0.09283	0.666
**206**	4-KB-B19-A	250	PI3K	0.093042	0.658
**207**	4-KB-C19-A	25	PI3K	0.09272	0.71
**208**	4-KB-D19-A	2.5	PI3K	0.092739	0.673

**209**	4-KB-E19-A	0.25	PI3K	0.092463	0.679
**210**	4-KB-F14-N	1000	PI3K	0.261977	0.926
**211**	4-KB-G2-TG	2500	PI3K	0.093308	0.669
**212**	4-KB-G5-So	10000	PI3K	0.13054	0.159
**213**	4-KB-G14-N	100	PI3K	0.246919	0
**214**	4-KB-G20-E	10000	PI3K	0.209924	0
**215**	4-KB-H2-TG	250	PI3K	0.093126	0.655
**216**	4-KB-H5-So	1000	PI3K	0.092761	0.668
**217**	4-KB-H14-N	10	PI3K	0.154755	0.041
**218**	4-KB-H20-E	1000	PI3K	0.174443	0.012
**219**	4-KB-I2-TGI	25	PI3K	0.092927	0.672
**220**	4-KB-I5-Sor	100	PI3K	0.107229	0.457
**221**	4-KB-I14-N'	1	PI3K	0.09244	0.692
**222**	4-KB-I20-Bu	100	PI3K	0.092181	0.72
**223**	4-KB-J2-TGI	2.5	PI3K	0.093144	0.69
**224**	4-KB-J5-Sor	10	PI3K	0.092986	0.681
**225**	4-KB-J20-Bu	10	PI3K	0.092975	0.69
**226**	4-KB-K2-TG	0.25	PI3K	0.092921	0.663
**227**	4-KB-K4-Da	0.1	PI3K	0.092888	0.732
**228**	4-KB-K5-So	1	PI3K	0.103229	0.505
**229**	4-KB-K14-N	0.1	PI3K	0.092917	0.692
**230**	4-KB-K20-B	1	PI3K	0.092295	0.711
**231**	4-KB-L4-Da	1	PI3K	0.092757	0.679
**232**	4-KB-L14-G	0.1	PI3K	0.092982	0.694
**233**	4-KB-L15-T	1	PI3K	0.096392	0.603
**234**	4-KB-L21-C	0.1	PI3K	0.108217	0.42
**235**	4-KB-M14-G	1	PI3K	0.09201	0.656
**236**	4-KB-M15-T	10	PI3K	0.101419	0.555
**237**	4-KB-M21-C	1	PI3K	0.125202	0.224
**238**	4-KB-N4-Da	10	PI3K	0.110346	0.4
**239**	4-KB-N14-G	10	PI3K	0.148574	0.065
**240**	4-KB-N15-T	100	PI3K	0.092714	0.667
**241**	4-KB-N21-C	10	PI3K	0.11385	0.354
**242**	4-KB-O4-Da	100	PI3K	0.220189	0.001
**243**	4-KB-O14-G	100	PI3K	0.222588	0

**244**	4-KB-O15-T	1000	PI3K	0.092878	0.668
**245**	4-KB-O21-C	100	PI3K	0.205117	0
**246**	4-KB-P4-Da	1000	PI3K	0.201127	0.001
**247**	4-KB-P14-G	1000	PI3K	0.240735	0
**248**	4-KB-P15-T	10000	PI3K	0.143755	0.072
**249**	4-KB-P21-C	1000	PI3K	0.251833	0
**250**	5-KB-A6-LY	2500	PI3K	0.236785	0
**251**	5-KB-A7-AN	1000	PI3K	0.16126	0.031
**252**	5-KB-A16-A	2500	PI3K	0.206323	0.002
**253**	5-KB-A17-P	10000	PI3K	0.156711	0.043
**254**	5-KB-B6-LY	250	PI3K	0.221375	0
**255**	5-KB-B7-AN	100	PI3K	0.092746	0.661
**256**	5-KB-B17-P	1000	PI3K	0.092749	0.687
**257**	5-KB-C6-LY	25	PI3K	0.092733	0.688
**258**	5-KB-C7-AN	10	PI3K	0.09277	0.72
**259**	5-KB-C16-A	250	PI3K	0.19325	0.002
**260**	5-KB-C17-P	100	PI3K	0.092568	0.644
**261**	5-KB-D6-LY	2.5	PI3K	0.093096	0.675
**262**	5-KB-D7-AN	1	PI3K	0.092161	0.679
**263**	5-KB-D16-A	25	PI3K	0.140725	0.084
**264**	5-KB-D17-P	10	PI3K	0.092906	0.654
**265**	5-KB-E6-LY	0.25	PI3K	0.091941	0.678
**266**	5-KB-E7-AN	0.1	PI3K	0.092581	0.671
**267**	5-KB-E16-A	2.5	PI3K	0.099427	0.588
**268**	5-KB-E17-P	1	PI3K	0.105855	0.467
**269**	5-KB-F11-G	10000	PI3K	0.190209	0.002
**270**	5-KB-F16-A	0.25	PI3K	0.105129	0.501
**271**	5-KB-G9-Se	10000	PI3K	0.202614	0.003
**272**	5-KB-G11-G	1000	PI3K	0.149276	0.07
**273**	5-KB-H9-Se	1000	PI3K	0.134882	0.136
**274**	5-KB-H11-G	100	PI3K	0.146546	0.072
**275**	5-KB-I9-Ser	100	PI3K	0.092711	0.656
**276**	5-KB-I11-G	10	PI3K	0.099729	0.566
**277**	5-KB-J9-Ser	10	PI3K	0.092812	0.689
**278**	5-KB-J11-G	1	PI3K	0.092809	0.69

**279**	5-KB-K9-Se	1	PI3K	0.10203	0.541
**280**	5-KB-L14-A	0.1	PI3K	0.093082	0.665
**281**	5-KB-L20-Z	1	PI3K	0.09297	0.672
**282**	5-KB-L23-O	0.1	PI3K	0.093691	0.667
**283**	5-KB-M14-A	1	PI3K	0.116698	0.321
**284**	5-KB-M20-Z	10	PI3K	0.093005	0.669
**285**	5-KB-M23-O	1	PI3K	0.092784	0.665
**286**	5-KB-N14-A	10	PI3K	0.110467	0.385
**287**	5-KB-N20-Z	100	PI3K	0.174207	0.015
**288**	5-KB-N23-C	10	PI3K	0.19237	0.004
**289**	5-KB-O14-A	100	PI3K	0.178283	0.011
**290**	5-KB-O20-Z	1000	PI3K	0.230503	0
**291**	5-KB-O23-C	100	PI3K	0.233976	0
**292**	5-KB-P14-A	1000	PI3K	0.191032	0.003
**293**	5-KB-P20-Z	10000	PI3K	0.247341	0
**294**	5-KB-P23-C	1000	PI3K	0.235579	0.003
**295**	6-KB-A8-TG	10000	PI3K	0.195846	0.003
**296**	6-KB-B8-TG	1000	PI3K	0.168405	0.013
**297**	6-KB-C8-TG	100	PI3K	0.138429	0.108
**298**	6-KB-D8-TG	10	PI3K	0.095854	0.57
**299**	6-KB-E8-TG	1	PI3K	0.09251	0.675
**300**	6-KB-L6-GD	1	PI3K	0.092063	0.676
**301**	6-KB-M6-GI	10	PI3K	0.093235	0.684
**302**	6-KB-N6-GI	100	PI3K	0.091989	0.657
**303**	6-KB-O6-GI	1000	PI3K	0.246788	0
**304**	6-KB-P6-GD	10000	PI3K	0.226985	0
**305**	1-KB-F11-A	10000	Topoisome	0.420199	0
**306**	1-KB-G11-A	1000	Topoisome	0.344731	0
**307**	1-KB-G20-E	1000	Topoisome	0.432143	0
**308**	1-KB-H11-A	100	Topoisome	0.18691	0.099
**309**	1-KB-H20-E	100	Topoisome	0.339425	0
**310**	1-KB-I11-Ar	10	Topoisome	0.048821	1
**311**	1-KB-I20-Ep	10	Topoisome	0.048821	1
**312**	1-KB-J11-Ar	1	Topoisome	0.048821	1
**313**	1-KB-J20-Ep	1	Topoisome	0.049153	1

**314**	1-KB-K11-S	1	Topoisome	0.166934	0.176
**315**	1-KB-K20-E	0.1	Topoisome	0.048821	0.998
**316**	1-KB-L11-Sl	10	Topoisome	0.388757	0
**317**	1-KB-L14-T	1	Topoisome	0.049153	0.999
**318**	1-KB-M11-S	100	Topoisome	0.423208	0
**319**	1-KB-M14-T	10	Topoisome	0.065189	0.979
**320**	1-KB-N14-T	100	Topoisome	0.342816	0
**321**	1-KB-O11-S	1000	Topoisome	0.439937	0
**322**	1-KB-O14-T	1000	Topoisome	0.418495	0
**323**	1-KB-P11-S	10000	Topoisome	0.449644	0
**324**	1-KB-P14-T	10000	Topoisome	0.436931	0
**325**	3-KB-A11-E	10000	Topoisome	0.360707	0
**326**	3-KB-B11-E	1000	Topoisome	0.304609	0.001
**327**	3-KB-C11-E	100	Topoisome	0.049153	0.999
**328**	3-KB-D11-E	10	Topoisome	0.049153	1
**329**	3-KB-E11-E	1	Topoisome	0.105368	0.717
**330**	3-KB-G9-Da	1000	Topoisome	0.512414	0
**331**	3-KB-G10-T	10000	Topoisome	0.060631	0.988
**332**	3-KB-H9-Da	100	Topoisome	0.361066	0
**333**	3-KB-H10-T	1000	Topoisome	0.075549	0.952
**334**	3-KB-I9-Dau	10	Topoisome	0.152021	0.309
**335**	3-KB-I10-Te	100	Topoisome	0.054162	0.996
**336**	3-KB-J9-Dau	1	Topoisome	0.170248	0.17
**337**	3-KB-J10-Te	10	Topoisome	0.170238	0.156
**338**	3-KB-K7-Ida	0.1	Topoisome	0.07706	0.936
**339**	3-KB-K9-Da	0.1	Topoisome	0.144022	0.313
**340**	3-KB-K10-T	1	Topoisome	0.171824	0.15
**341**	3-KB-L6-Do	0.1	Topoisome	0.048821	0.999
**342**	3-KB-L7-Ida	1	Topoisome	0.167818	0.156
**343**	3-KB-L9-Val	0.5	Topoisome	0.070541	0.962
**344**	3-KB-L10-M	0.1	Topoisome	0.047825	1
**345**	3-KB-L16-P	1	Topoisome	0.166283	0.187
**346**	3-KB-M6-Do	1	Topoisome	0.048821	1
**347**	3-KB-M7-Id	10	Topoisome	0.259119	0.003
**348**	3-KB-M9-Va	5	Topoisome	0.11881	0.583

**349**	3-KB-M10-I	1	Topoisome	0.161855	0.206
**350**	3-KB-M16-I	10	Topoisome	0.048489	1
**351**	3-KB-N6-Dc	10	Topoisome	0.135004	0.444
**352**	3-KB-N9-Va	50	Topoisome	0.298778	0
**353**	3-KB-N10-N	10	Topoisome	0.168462	0.139
**354**	3-KB-N16-F	100	Topoisome	0.139953	0.372
**355**	3-KB-O6-Dc	100	Topoisome	0.296132	0
**356**	3-KB-O7-Ida	100	Topoisome	0.39356	0
**357**	3-KB-O9-Va	500	Topoisome	0.417241	0
**358**	3-KB-O10-N	100	Topoisome	0.370366	0
**359**	3-KB-O16-F	1000	Topoisome	0.054216	0.996
**360**	3-KB-P6-Dc	1000	Topoisome	0.497754	0
**361**	3-KB-P7-Ida	1000	Topoisome	0.516083	0
**362**	3-KB-P9-Va	5000	Topoisome	0.432735	0
**363**	3-KB-P10-N	1000	Topoisome	0.452462	0
**364**	3-KB-P16-P	10000	Topoisome	0.217798	0.029
**365**	1-KB-A10-V	10000	Mitotic	0.549619	0
**366**	1-KB-A13-I	1000	Mitotic	0.458068	0
**367**	1-KB-A18-P	1000	Mitotic	0.563789	0
**368**	1-KB-B10-V	1000	Mitotic	0.569646	0
**369**	1-KB-B13-I	100	Mitotic	0.15512	0.372
**370**	1-KB-B18-P	100	Mitotic	0.523082	0
**371**	1-KB-C10-V	100	Mitotic	0.550839	0
**372**	1-KB-C13-I	10	Mitotic	0.051223	0.996
**373**	1-KB-C18-P	10	Mitotic	0.446564	0
**374**	1-KB-D10-V	10	Mitotic	0.191135	0.118
**375**	1-KB-D13-I	1	Mitotic	0.10313	0.79
**376**	1-KB-D18-F	1	Mitotic	0.169581	0.275
**377**	1-KB-E10-V	1	Mitotic	0.114195	0.704
**378**	1-KB-E13-Ix	0.1	Mitotic	0.181501	0.237
**379**	1-KB-E18-P	0.1	Mitotic	0.054972	0.998
**380**	1-KB-F13-V	1000	Mitotic	0.586816	0
**381**	1-KB-G13-V	100	Mitotic	0.135373	0.526
**382**	1-KB-G15-E	1000	Mitotic	0.585709	0
**383**	1-KB-H13-V	10	Mitotic	0.166664	0.285

**384**	1-KB-H15-E	100	Mitotic	0.597451	0
**385**	1-KB-I13-Vi	1	Mitotic	0.18745	0.181
**386**	1-KB-I15-Er	10	Mitotic	0.577301	0
**387**	1-KB-J13-Vi	0.1	Mitotic	0.181768	0.194
**388**	1-KB-J15-Er	1	Mitotic	0.166234	0.245
**389**	1-KB-K7-Vir	0.1	Mitotic	0.08598	0.918
**390**	1-KB-K15-E	0.1	Mitotic	0.137316	0.495
**391**	1-KB-L7-Vir	1	Mitotic	0.204926	0.094
**392**	1-KB-L20-V	0.1	Mitotic	0.063551	0.983
**393**	1-KB-M7-Vi	10	Mitotic	0.100907	0.804
**394**	1-KB-M20-\	1	Mitotic	0.098509	0.825
**395**	1-KB-N20-V	10	Mitotic	0.065192	0.99
**396**	1-KB-O7-Vi	100	Mitotic	0.163574	0.296
**397**	1-KB-O20-V	100	Mitotic	0.051223	0.999
**398**	1-KB-P7-Vir	1000	Mitotic	0.584694	0
**399**	1-KB-P20-V	1000	Mitotic	0.467152	0
**400**	3-KB-A7-Dc	1000	Mitotic	0.476475	0
**401**	3-KB-B7-Dc	100	Mitotic	0.519043	0
**402**	3-KB-C7-Dc	10	Mitotic	0.471703	0
**403**	3-KB-D7-Dc	1	Mitotic	0.109893	0.636
**404**	3-KB-E7-Do	0.1	Mitotic	0.051223	0.998
**405**	6-KB-L19-A	1	Mitotic	0.051223	0.997
**406**	6-KB-M19-A	10	Mitotic	0.084737	0.909
**407**	6-KB-N19-A	100	Mitotic	0.078752	0.946
**408**	6-KB-O19-A	1000	Mitotic	0.44533	0
**409**	6-KB-P19-A	10000	Mitotic	0.517575	0
**410**	2-KB-A12-T	250	MEK1/2	0.371864	0.002
**411**	2-KB-B12-T	25	MEK1/2	0.38819	0
**412**	2-KB-D12-T	2.5	MEK1/2	0.23986	0.103
**413**	2-KB-E12-Ti	0.25	MEK1/2	0.241508	0.122
**414**	2-KB-F12-Ti	2.5000000	MEK1/2	0.181953	0.385
**415**	2-KB-F14-C	1000	MEK1/2	0.357664	0.003
**416**	2-KB-G14-C	100	MEK1/2	0.371979	0.001
**417**	2-KB-H14-C	10	MEK1/2	0.400512	0
**418**	2-KB-I14-Cc	1	MEK1/2	0.130549	0.708

**419**	2-KB-K14-C	0.1	MEK1/2	0.254229	0.08
**420**	2-KB-L20-S	1	MEK1/2	0.225876	0.168
**421**	2-KB-M20-S	10	MEK1/2	0.310044	0.022
**422**	2-KB-N20-S	100	MEK1/2	0.396913	0.001
**423**	2-KB-O20-S	1000	MEK1/2	0.360258	0.002
**424**	2-KB-P20-S	10000	MEK1/2	0.368966	0.001
**425**	4-KB-A10-B	1000	MEK1/2	0.344371	0.008
**426**	4-KB-A13-P	1000	MEK1/2	0.39208	0
**427**	4-KB-B10-B	100	MEK1/2	0.304307	0.018
**428**	4-KB-B13-P	100	MEK1/2	0.36897	0.001
**429**	4-KB-C10-B	10	MEK1/2	0.133509	0.688
**430**	4-KB-C13-P	10	MEK1/2	0.309802	0.014
**431**	4-KB-D10-B	1	MEK1/2	0.13187	0.711
**432**	4-KB-D13-P	1	MEK1/2	0.191354	0.327
**433**	4-KB-E10-B	0.1	MEK1/2	0.16464	0.482
**434**	4-KB-E13-P	0.1	MEK1/2	0.213497	0.198
**435**	4-KB-L19-G	0.25	MEK1/2	0.259626	0.07
**436**	4-KB-M19-G	2.5	MEK1/2	0.131536	0.695
**437**	4-KB-N19-G	25	MEK1/2	0.218089	0.193
**438**	4-KB-O19-G	250	MEK1/2	0.152101	0.553
**439**	4-KB-P19-G	2500	MEK1/2	0.335333	0.004
**440**	1-KB-L2-Ola	1	PARP	0.290587	0.053
**441**	1-KB-L6-Ru	1	PARP	0.154027	0.628
**442**	1-KB-M2-Ol	10	PARP	0.324836	0.025
**443**	1-KB-M6-Ru	10	PARP	0.348437	0.005
**444**	1-KB-N2-Ol	100	PARP	0.336303	0.018
**445**	1-KB-N6-Ru	100	PARP	0.147078	0.647
**446**	1-KB-O2-Ol	1000	PARP	0.14872	0.646
**447**	1-KB-O6-Ru	1000	PARP	0.14675	0.644
**448**	1-KB-P2-Ola	10000	PARP	0.191645	0.335
**449**	1-KB-P6-Ru	10000	PARP	0.189306	0.361
**450**	7-KB-A3-Tal	1000	PARP	0.220629	0.206
**451**	7-KB-B2-Ve	10000	PARP	0.147406	0.641
**452**	7-KB-B3-Tal	100	PARP	0.155686	0.567
**453**	7-KB-C2-Ve	1000	PARP	0.17104	0.507

**454**	7-KB-C3-Tal	10	PARP	0.15047	0.586
**455**	7-KB-D2-Ve	100	PARP	0.335339	0.019
**456**	7-KB-D3-Ta	1	PARP	0.158632	0.605
**457**	7-KB-E2-Vel	10	PARP	0.145108	0.671
**458**	7-KB-E3-Tal	0.1	PARP	0.147078	0.668
**459**	7-KB-F2-Vel	1	PARP	0.31652	0.028
**460**	7-KB-G2-Ni	10000	PARP	0.208934	0.283
**461**	7-KB-H2-Ni	1000	PARP	0.148063	0.667
**462**	7-KB-I2-Nir	100	PARP	0.306931	0.036
**463**	7-KB-J2-Nir	10	PARP	0.335754	0.015
**464**	7-KB-K2-Ni	1	PARP	0.337347	0.02
**465**	3-KB-A19-D	1000	CDK	0.451006	0
**466**	3-KB-B19-D	100	CDK	0.444044	0
**467**	3-KB-B23-A	2500	CDK	0.250562	0.005
**468**	3-KB-C19-D	10	CDK	0.15058	0.27
**469**	3-KB-C23-A	250	CDK	0.228845	0.012
**470**	3-KB-D19-D	1	CDK	0.118558	0.597
**471**	3-KB-D23-A	25	CDK	0.128584	0.478
**472**	3-KB-E19-D	0.1	CDK	0.103512	0.724
**473**	3-KB-E23-A	2.5	CDK	0.052507	0.995
**474**	3-KB-F23-A	0.25	CDK	0.220952	0.026
**475**	3-KB-K17-P	1	CDK	0.197816	0.074
**476**	3-KB-L19-R	1	CDK	0.160361	0.226
**477**	3-KB-M17-I	10	CDK	0.144349	0.351
**478**	3-KB-M19-I	10	CDK	0.223319	0.026
**479**	3-KB-N17-F	100	CDK	0.220708	0.032
**480**	3-KB-N19-F	100	CDK	0.144177	0.354
**481**	3-KB-O17-F	1000	CDK	0.232265	0.013
**482**	3-KB-O19-F	1000	CDK	0.236018	0.017
**483**	3-KB-P17-P	10000	CDK	0.238131	0.009
**484**	3-KB-P19-R	10000	CDK	0.214393	0.033
**485**	4-KB-A4-SN	10000	CDK	0.384916	0
**486**	4-KB-A8-Mi	10000	CDK	0.368158	0
**487**	4-KB-B4-SN	1000	CDK	0.378846	0
**488**	4-KB-B8-Mi	1000	CDK	0.221474	0.014

**489**	4-KB-C4-SN	100	CDK	0.221178	0.015
**490**	4-KB-C8-Mi	100	CDK	0.074031	0.914
**491**	4-KB-D4-SN	10	CDK	0.140511	0.374
**492**	4-KB-D8-Mi	10	CDK	0.133907	0.435
**493**	4-KB-E4-SN	1	CDK	0.092873	0.83
**494**	4-KB-E8-Mi	1	CDK	0.114346	0.619
**495**	4-KB-F4-Sel	10000	CDK	0.267732	0
**496**	4-KB-F22-A	10000	CDK	0.393075	0
**497**	4-KB-G4-Se	1000	CDK	0.132089	0.455
**498**	4-KB-G22-A	1000	CDK	0.386891	0
**499**	4-KB-H4-Se	100	CDK	0.1045	0.732
**500**	4-KB-H22-A	100	CDK	0.246312	0.008
**501**	4-KB-I4-Seli	10	CDK	0.099698	0.778
**502**	4-KB-I22-Al	10	CDK	0.137633	0.367
**503**	4-KB-J4-Sel	1	CDK	0.114492	0.623
**504**	4-KB-J22-Al	1	CDK	0.131241	0.478
**505**	5-KB-A19-A	10000	CDK	0.449109	0
**506**	5-KB-B19-A	1000	CDK	0.346419	0
**507**	5-KB-C19-A	100	CDK	0.236082	0.009
**508**	5-KB-D19-A	10	CDK	0.081133	0.915
**509**	5-KB-E19-A	1	CDK	0.172838	0.173
**510**	5-KB-K17-A	1	CDK	0.10716	0.692
**511**	5-KB-M17-A	10	CDK	0.127268	0.507
**512**	5-KB-N17-A	100	CDK	0.123657	0.524
**513**	5-KB-O17-A	1000	CDK	0.439415	0
**514**	5-KB-P17-A	10000	CDK	0.429555	0
**515**	6-KB-A17-S	1000	CDK	0.057643	0.995
**516**	6-KB-B17-S	100	CDK	0.097122	0.79
**517**	6-KB-C17-S	10	CDK	0.092676	0.807
**518**	6-KB-D17-S	1	CDK	0.075098	0.953
**519**	6-KB-E17-S	0.1	CDK	0.089574	0.839
**520**	6-KB-L15-T	1	CDK	0.156116	0.244
**521**	6-KB-M15-T	10	CDK	0.187396	0.099
**522**	6-KB-N15-T	100	CDK	0.231846	0.011
**523**	6-KB-O15-T	1000	CDK	0.433259	0

**524**	6-KB-P15-T 10000	CDK	0.475654	0
**525**	7-KB-A21-d 10000	BET	0.365168	0
**526**	7-KB-A22-P 30000	BET	0.387701	0
**527**	7-KB-B21-d 1000	BET	0.20069	0.118
**528**	7-KB-B22-P 3000	BET	0.383694	0
**529**	7-KB-C21-d 100	BET	0.255449	0.029
**530**	7-KB-C22-P 300	BET	0.248188	0.024
**531**	7-KB-D21-c 10	BET	0.101065	0.803
**532**	7-KB-D22-F 30	BET	0.100463	0.806
**533**	7-KB-E21-d 1	BET	0.280613	0.005
**534**	7-KB-E22-P 3	BET	0.100637	0.798
**535**	7-KB-G10-E 10000	BET	0.373472	0
**536**	7-KB-G15-I 10000	BET	0.369353	0
**537**	7-KB-H10-E 1000	BET	0.383731	0
**538**	7-KB-H15-I 1000	BET	0.398304	0
**539**	7-KB-I10-Bi 100	BET	0.182547	0.202
**540**	7-KB-I15-I-I 100	BET	0.100769	0.793
**541**	7-KB-J10-Bi 10	BET	0.100848	0.822
**542**	7-KB-J15-I-I 10	BET	0.12368	0.623
**543**	7-KB-K10-B 1	BET	0.100679	0.825
**544**	7-KB-K13-N 1	BET	0.209481	0.108
**545**	7-KB-K15-I- 1	BET	0.118479	0.689
**546**	7-KB-L12-N 1	BET	0.101251	0.818
**547**	7-KB-L13-N 10	BET	0.349869	0
**548**	7-KB-L20-J(1	BET	0.100876	0.812
**549**	7-KB-L23-A 0.03	BET	0.12129	0.661
**550**	7-KB-M12-I 10	BET	0.112205	0.734
**551**	7-KB-M13-I 100	BET	0.409941	0
**552**	7-KB-M20-J 10	BET	0.101245	0.82
**553**	7-KB-M23-A 0.3	BET	0.131672	0.596
**554**	7-KB-N12-N 100	BET	0.158148	0.361
**555**	7-KB-N13-N 1000	BET	0.386734	0
**556**	7-KB-N20-J 100	BET	0.19744	0.126
**557**	7-KB-N23-A 3	BET	0.200708	0.101
**558**	7-KB-O12-N 1000	BET	0.318268	0.001

**559**	7-KB-O20-J	1000	BET	0.419901	0
**560**	7-KB-O23-A	30	BET	0.274005	0.006
**561**	7-KB-P12-N	10000	BET	0.419393	0
**562**	7-KB-P13-N	10000	BET	0.367202	0
**563**	7-KB-P20-J	10000	BET	0.427135	0
**564**	7-KB-P23-A	300	BET	0.36889	0
**565**	8-KB-K22-C	1	BET	0.100024	0.824
**566**	8-KB-L22-C	10	BET	0.10088	0.812
**567**	8-KB-M22-(	100	BET	0.156003	0.369
**568**	8-KB-N22-C	1000	BET	0.100517	0.84
**569**	8-KB-O22-C	10000	BET	0.101091	0.82
**570**	1-KB-A3-Vo	10000	HDAC	0.178488	0.02
**571**	1-KB-B3-Vo	1000	HDAC	0.065755	0.848
**572**	1-KB-C3-Vo	100	HDAC	0.072131	0.935
**573**	1-KB-D3-Vc	10	HDAC	0.143704	0.174
**574**	1-KB-E3-Vo	1	HDAC	0.16037	0.076
**575**	1-KB-L12-R	0.1	HDAC	0.120662	0.348
**576**	1-KB-M12-I	1	HDAC	0.182114	0.021
**577**	1-KB-N12-R	10	HDAC	0.340736	0
**578**	1-KB-O12-R	100	HDAC	0.374931	0.001
**579**	1-KB-P12-R	1000	HDAC	0.390363	0
**580**	3-KB-A4-Pa	1000	HDAC	0.418942	0
**581**	3-KB-B4-Pa	100	HDAC	0.284813	0.001
**582**	3-KB-C4-Pa	10	HDAC	0.205814	0.01
**583**	3-KB-D4-Pa	1	HDAC	0.138343	0.198
**584**	3-KB-E4-Pa	0.1	HDAC	0.114671	0.438
**585**	3-KB-F7-Qu	1000	HDAC	0.453875	0
**586**	3-KB-G7-Qu	100	HDAC	0.281992	0
**587**	3-KB-G12-V	1000000	HDAC	0.079429	0.88
**588**	3-KB-H7-Qu	10	HDAC	0.106807	0.503
**589**	3-KB-H12-V	100000	HDAC	0.057463	0.993
**590**	3-KB-I7-Qui	1	HDAC	0.103077	0.577
**591**	3-KB-I12-Va	10000	HDAC	0.105545	0.528
**592**	3-KB-J7-Qu	0.1	HDAC	0.036962	1
**593**	3-KB-J12-Va	1000	HDAC	0.035052	1

**594**	3-KB-K3-Be	1	HDAC	0.156629	0.09
**595**	3-KB-K12-V	100	HDAC	0.036957	1
**596**	3-KB-L3-Bel	10	HDAC	0.136237	0.202
**597**	3-KB-M3-Be	100	HDAC	0.080675	0.863
**598**	3-KB-N3-Be	1000	HDAC	0.279553	0
**599**	3-KB-O3-Be	10000	HDAC	0.600075	0
**600**	7-KB-A5-Mc	10000	HDAC	0.305331	0
**601**	7-KB-A7-CL	10000	HDAC	0.456367	0
**602**	7-KB-A9-Gi	1000	HDAC	0.292206	0
**603**	7-KB-A12-R	10000	HDAC	0.24661	0.001
**604**	7-KB-B5-Mc	1000	HDAC	0.135298	0.155
**605**	7-KB-B7-CL	1000	HDAC	0.450349	0
**606**	7-KB-B12-R	1000	HDAC	0.145126	0.163
**607**	7-KB-C5-Mc	100	HDAC	0.14134	0.172
**608**	7-KB-C7-CL	100	HDAC	0.363774	0
**609**	7-KB-C9-Gi	100	HDAC	0.124675	0.285
**610**	7-KB-D7-CL	10	HDAC	0.109104	0.396
**611**	7-KB-D9-Gi	10	HDAC	0.145525	0.129
**612**	7-KB-D12-R	100	HDAC	0.145622	0.159
**613**	7-KB-E5-Mc	10	HDAC	0.149811	0.108
**614**	7-KB-E7-CU	1	HDAC	0.17461	0.034
**615**	7-KB-E9-Gi	1	HDAC	0.175559	0.035
**616**	7-KB-E12-R	10	HDAC	0.119953	0.365
**617**	7-KB-F5-Mc	1	HDAC	0.039004	1
**618**	7-KB-F7-Re	10000	HDAC	0.422886	0
**619**	7-KB-F9-Gi	0.1	HDAC	0.121377	0.356
**620**	7-KB-F12-R	1	HDAC	0.091947	0.748
**621**	7-KB-F19-P	10000	HDAC	0.063153	0.879
**622**	7-KB-G7-Re	1000	HDAC	0.115063	0.311
**623**	7-KB-G19-P	1000	HDAC	0.056159	0.995
**624**	7-KB-H7-Re	100	HDAC	0.189845	0.018
**625**	7-KB-I7-Res	10	HDAC	0.161911	0.059
**626**	7-KB-I19-P	100	HDAC	0.11754	0.386
**627**	7-KB-J7-Res	1	HDAC	0.147884	0.142
**628**	7-KB-J19-P	10	HDAC	0.164285	0.059

629	7-KB-K4-En	1	HDAC	0.175573	0.038
630	7-KB-K11-A	1	HDAC	0.134395	0.252
631	7-KB-K18-T	1	HDAC	0.072356	0.933
632	7-KB-K19-P	1	HDAC	0.13227	0.253
633	7-KB-L2-Ta	0.1	HDAC	0.147053	0.147
634	7-KB-L4-En	10	HDAC	0.125433	0.31
635	7-KB-L5-Pra	1	HDAC	0.140609	0.19
636	7-KB-L8-Ab	1	HDAC	0.136333	0.224
637	7-KB-L10-T	1	HDAC	0.147524	0.121
638	7-KB-L11-A	10	HDAC	0.141718	0.166
639	7-KB-L14-T	1	HDAC	0.161595	0.058
640	7-KB-L16-R	1	HDAC	0.114657	0.423
641	7-KB-L18-T	10	HDAC	0.078924	0.884
642	7-KB-M2-Ta	1	HDAC	0.136986	0.225
643	7-KB-M5-Pr	10	HDAC	0.174904	0.031
644	7-KB-M8-Al	10	HDAC	0.185396	0.02
645	7-KB-M10-	10	HDAC	0.13037	0.238
646	7-KB-M11-	100	HDAC	0.172125	0.033
647	7-KB-M14-	10	HDAC	0.093173	0.703
648	7-KB-M16-	10	HDAC	0.110909	0.497
649	7-KB-M18-	100	HDAC	0.101537	0.56
650	7-KB-N2-Ta	10	HDAC	0.113673	0.473
651	7-KB-N4-En	100	HDAC	0.175467	0.028
652	7-KB-N5-Pr	100	HDAC	0.152055	0.095
653	7-KB-N8-Ab	100	HDAC	0.190426	0.01
654	7-KB-N10-T	100	HDAC	0.149192	0.131
655	7-KB-N14-T	100	HDAC	0.111497	0.46
656	7-KB-N16-R	100	HDAC	0.108743	0.481
657	7-KB-N18-T	1000	HDAC	0.119947	0.403
658	7-KB-O2-Ta	100	HDAC	0.125601	0.303
659	7-KB-O4-En	1000	HDAC	0.234418	0
660	7-KB-O5-Pr	1000	HDAC	0.195391	0.02
661	7-KB-O8-Ab	1000	HDAC	0.217895	0.01
662	7-KB-O10-T	1000	HDAC	0.187937	0.014
663	7-KB-O11-A	1000	HDAC	0.106214	0.375

**664**	7-KB-O14-T	1000	HDAC	0.035857	1
**665**	7-KB-O16-F	1000	HDAC	0.179848	0.034
**666**	7-KB-P2-Ta	1000	HDAC	0.079043	0.876
**667**	7-KB-P4-En	10000	HDAC	0.106063	0.377
**668**	7-KB-P5-Pr	10000	HDAC	0.424521	0
**669**	7-KB-P8-Ab	10000	HDAC	0.493762	0
**670**	7-KB-P10-T	10000	HDAC	0.104536	0.394
**671**	7-KB-P11-A	10000	HDAC	0.399658	0
**672**	7-KB-P14-T	10000	HDAC	0.094039	0.736
**673**	7-KB-P16-R	10000	HDAC	0.119997	0.278
**674**	7-KB-P18-T	10000	HDAC	0.058094	0.962
**0**	2-KW-A16-	10000	EGFR	0.239816	0.242
**1**	2-KW-A19-	10000	EGFR	0.350691	0.015
**2**	2-KW-B19-	1000	EGFR	0.27675	0.156
**3**	2-KW-C16-	1000	EGFR	0.080988	0.957
**4**	2-KW-C19-	100	EGFR	0.43231	0
**5**	2-KW-D16-	100	EGFR	0.291728	0.19
**6**	2-KW-D19-	10	EGFR	0.353371	0.008
**7**	2-KW-E16-	10	EGFR	0.258642	0.079
**8**	2-KW-E19-	1	EGFR	0.403248	0.005
**9**	2-KW-F16-	1	EGFR	0.075056	0.979
**10**	2-KW-K11-	0.1	EGFR	0.167147	0.589
**11**	2-KW-L11-	1	EGFR	0.303615	0.027
**12**	2-KW-L16-	0.25	EGFR	0.333484	0.072
**13**	2-KW-L19-	0.1	EGFR	0.46761	0
**14**	2-KW-M11-	10	EGFR	0.413706	0.001
**15**	2-KW-M16-	2.5	EGFR	0.367107	0.002
**16**	2-KW-M19-	1	EGFR	0.443957	0.001
**17**	2-KW-N16-	25	EGFR	0.423506	0.001
**18**	2-KW-N19-	10	EGFR	0.108937	0.859
**19**	2-KW-O11-	100	EGFR	0.378037	0
**20**	2-KW-O16-	250	EGFR	0.127842	0.756
**21**	2-KW-O19-	100	EGFR	0.28201	0.156
**22**	2-KW-P11-	1000	EGFR	0.236855	0.167
**23**	2-KW-P16-	2500	EGFR	0.319228	0.021

**24**	2-KW-P19-I	1000	EGFR	0.348296	0.01
**25**	3-KW-F21-I	10000	EGFR	0.251604	0.248
**26**	3-KW-G20-	1000	EGFR	0.338366	0.004
**27**	3-KW-G21-	1000	EGFR	0.232556	0.15
**28**	3-KW-H20-	100	EGFR	0.392708	0
**29**	3-KW-H21-	100	EGFR	0.361882	0.003
**30**	3-KW-I20-N	10	EGFR	0.324859	0.008
**31**	3-KW-I21-F	10	EGFR	0.232228	0.219
**32**	3-KW-J20-I	1	EGFR	0.470063	0
**33**	3-KW-J21-F	1	EGFR	0.281999	0.254
**34**	3-KW-K4-C	1	EGFR	0.080251	0.955
**35**	3-KW-K18-I	0.1	EGFR	0.39641	0.001
**36**	3-KW-K20-I	0.1	EGFR	0.114464	0.925
**37**	3-KW-L4-C	10	EGFR	0.07767	0.98
**38**	3-KW-L18-I	1	EGFR	0.295925	0.17
**39**	3-KW-M18-	10	EGFR	0.238552	0.266
**40**	3-KW-N4-C	100	EGFR	0.400962	0.012
**41**	3-KW-N18-	100	EGFR	0.396657	0
**42**	3-KW-O4-C	1000	EGFR	0.30997	0.019
**43**	3-KW-P4-C	10000	EGFR	0.084995	1
**44**	3-KW-P18-I	1000	EGFR	0.17071	0.503
**45**	4-KW-F13-S	1000	EGFR	0.315153	0.01
**46**	4-KW-G13-	100	EGFR	0.34884	0.002
**47**	4-KW-G16-	10000	EGFR	0.304441	0.016
**48**	4-KW-H13-	10	EGFR	0.257834	0.095
**49**	4-KW-H16-	1000	EGFR	0.267916	0.052
**50**	4-KW-I13-S	1	EGFR	0.347085	0.003
**51**	4-KW-I16-V	100	EGFR	0.234969	0.277
**52**	4-KW-J13-S	0.1	EGFR	0.083199	0.914
**53**	4-KW-J16-V	10	EGFR	0.088391	0.943
**54**	4-KW-K7-Ic	1	EGFR	0.077675	0.973
**55**	4-KW-K13-	0.1	EGFR	0.291512	0.018
**56**	4-KW-K16-	1	EGFR	0.361251	0
**57**	4-KW-L7-Ic	10	EGFR	0.260049	0.139
**58**	4-KW-L13-	1	EGFR	0.152684	0.624

**59**	4-KW-M7-I	100	EGFR	0.18134	0.431
**60**	4-KW-M13-	10	EGFR	0.355906	0.001
**61**	4-KW-N13-	100	EGFR	0.364313	0.005
**62**	4-KW-O7-Ic	1000	EGFR	0.091768	0.95
**63**	4-KW-P7-Ic	10000	EGFR	0.288859	0.09
**64**	4-KW-P13-	1000	EGFR	0.307442	0.016
**65**	5-KW-F4-Pc	1000	EGFR	0.407604	0.004
**66**	5-KW-F7-AZ	1000	EGFR	0.151241	0.844
**67**	5-KW-G4-P	100	EGFR	0.383248	0.003
**68**	5-KW-G7-A	100	EGFR	0.372746	0.013
**69**	5-KW-H4-P	10	EGFR	0.165978	0.583
**70**	5-KW-H7-A	10	EGFR	0.156762	0.835
**71**	5-KW-I4-Pc	1	EGFR	0.207635	0.846
**72**	5-KW-I7-AZ	1	EGFR	0.315945	0.017
**73**	5-KW-J4-Pc	0.1	EGFR	0.109316	0.871
**74**	5-KW-J7-AZ	0.1	EGFR	0.366949	0.003
**75**	5-KW-K7-O	0.1	EGFR	0.091696	0.935
**76**	5-KW-L7-OI	1	EGFR	0.377374	0
**77**	5-KW-M7-C	10	EGFR	0.356981	0.023
**78**	5-KW-O7-O	100	EGFR	0.320026	0.08
**79**	5-KW-P7-O	1000	EGFR	0.329004	0.02
**80**	2-KW-A15-I	2500	VEGFR	0.384045	0.003
**81**	2-KW-A17-I	10000	VEGFR	0.300617	0.028
**82**	2-KW-A20-	10000	VEGFR	0.270749	0.078
**83**	2-KW-B15-I	250	VEGFR	0.215488	0.167
**84**	2-KW-B17-I	1000	VEGFR	0.240113	0.039
**85**	2-KW-B20-	1000	VEGFR	0.182873	0.805
**86**	2-KW-C15-I	25	VEGFR	0.227924	0.08
**87**	2-KW-C17-I	100	VEGFR	0.280886	0.014
**88**	2-KW-D15-	2.5	VEGFR	0.319874	0
**89**	2-KW-D17-	10	VEGFR	0.34929	0.009
**90**	2-KW-D20-	100	VEGFR	0.300201	0.1
**91**	2-KW-E17-I	1	VEGFR	0.315117	0.015
**92**	2-KW-E20-	10	VEGFR	0.185283	0.695
**93**	2-KW-F13-/	10000	VEGFR	0.289256	0.136

**94**	2-KW-F15-I	0.25	VEGFR	0.203674	0.251
**95**	2-KW-F19-I	10000	VEGFR	0.250155	0.24
**96**	2-KW-F20-T	1	VEGFR	0.149983	0.663
**97**	2-KW-F21-\	10000	VEGFR	0.325944	0.464
**98**	2-KW-G10-	10000	VEGFR	0.367628	0.001
**99**	2-KW-G13-	1000	VEGFR	0.301365	0.03
**100**	2-KW-G19-	1000	VEGFR	0.253039	0.208
**101**	2-KW-G21-	1000	VEGFR	0.239936	0.378
**102**	2-KW-H10-	1000	VEGFR	0.313286	0.124
**103**	2-KW-H13-	100	VEGFR	0.307668	0.017
**104**	2-KW-H21-	100	VEGFR	0.294929	0.032
**105**	2-KW-I10-/	100	VEGFR	0.235504	0.099
**106**	2-KW-I13-/	10	VEGFR	0.396863	0
**107**	2-KW-I19-F	100	VEGFR	0.161485	0.735
**108**	2-KW-I21-\	10	VEGFR	0.218402	0.769
**109**	2-KW-J10-/	10	VEGFR	0.293428	0.146
**110**	2-KW-J13-/	1	VEGFR	0.357141	0.001
**111**	2-KW-J19-F	10	VEGFR	0.230695	0.386
**112**	2-KW-J21-\	1	VEGFR	0.273088	0.491
**113**	2-KW-K10-/	1	VEGFR	0.20913	0.489
**114**	2-KW-K13-'	0.1	VEGFR	0.291255	0.011
**115**	2-KW-K17-I	1	VEGFR	0.272585	0.062
**116**	2-KW-K19-I	1	VEGFR	0.205535	0.471
**117**	2-KW-L12-S	0.1	VEGFR	0.299437	0.037
**118**	2-KW-L13-'	1	VEGFR	0.249971	0.073
**119**	2-KW-L21-(	0.1	VEGFR	0.273815	0.188
**120**	2-KW-M12-	1	VEGFR	0.251223	0.086
**121**	2-KW-M13-	10	VEGFR	0.397726	0.001
**122**	2-KW-M17-	10	VEGFR	0.229747	0.415
**123**	2-KW-M21-	1	VEGFR	0.263246	0.3
**124**	2-KW-N12-	10	VEGFR	0.292283	0.042
**125**	2-KW-N13-	100	VEGFR	0.157491	0.585
**126**	2-KW-N17-	100	VEGFR	0.235788	0.153
**127**	2-KW-N21-	10	VEGFR	0.250602	0.187
**128**	2-KW-O12-	100	VEGFR	0.181791	0.706

**129**	2-KW-O17-	1000	VEGFR	0.232998	0.619
**130**	2-KW-O21-	100	VEGFR	0.188335	0.61
**131**	2-KW-P12-!	1000	VEGFR	0.215278	0.397
**132**	2-KW-P13-'	1000	VEGFR	0.268365	0.034
**133**	2-KW-P17-I	10000	VEGFR	0.259419	0.042
**134**	2-KW-P21-(	1000	VEGFR	0.332855	0.009
**135**	3-KW-A3-C	1000	VEGFR	0.374589	0
**136**	3-KW-A6-F	1000	VEGFR	0.313333	0.066
**137**	3-KW-A18-I	1000	VEGFR	0.305605	0.002
**138**	3-KW-B3-C	100	VEGFR	0.366579	0
**139**	3-KW-B6-F	100	VEGFR	0.334529	0.015
**140**	3-KW-B18-I	100	VEGFR	0.315212	0.003
**141**	3-KW-C3-C	10	VEGFR	0.199022	0.351
**142**	3-KW-C6-F	10	VEGFR	0.225504	0.183
**143**	3-KW-C18-I	10	VEGFR	0.141267	0.798
**144**	3-KW-D3-C	1	VEGFR	0.132388	0.962
**145**	3-KW-D6-F	1	VEGFR	0.114055	0.83
**146**	3-KW-D18-	1	VEGFR	0.176348	0.62
**147**	3-KW-E3-C	0.1	VEGFR	0.147086	0.695
**148**	3-KW-E6-F	0.1	VEGFR	0.401202	0.01
**149**	3-KW-E18-I	0.1	VEGFR	0.225228	0.095
**150**	3-KW-F18-I	1000	VEGFR	0.324726	0.002
**151**	3-KW-G18-	100	VEGFR	0.241732	0.41
**152**	3-KW-H18-	10	VEGFR	0.175019	0.592
**153**	3-KW-I18-E	1	VEGFR	0.433215	0
**154**	3-KW-J18-E	0.1	VEGFR	0.136143	0.65
**155**	4-KW-A12-I	10000	VEGFR	0.254863	0.603
**156**	4-KW-A15-(	2500	VEGFR	0.359582	0.001
**157**	4-KW-A20-I	10000	VEGFR	0.202899	0.376
**158**	4-KW-B12-I	1000	VEGFR	0.255481	0.119
**159**	4-KW-B15-(	250	VEGFR	0.21614	0.093
**160**	4-KW-B20-I	1000	VEGFR	0.257327	0.065
**161**	4-KW-C15-(	25	VEGFR	0.145912	0.375
**162**	4-KW-D12-	100	VEGFR	0.34321	0.017
**163**	4-KW-D15-	2.5	VEGFR	0.244416	0.088

**164**	4-KW-D20-	100	VEGFR	0.07902	0.929
**165**	4-KW-E12-I	10	VEGFR	0.344487	0
**166**	4-KW-E20-I	10	VEGFR	0.233244	0.186
**167**	4-KW-F12-I	1	VEGFR	0.242695	0.113
**168**	4-KW-F15-(	0.25	VEGFR	0.215036	0.189
**169**	4-KW-F20-I	1	VEGFR	0.144089	0.561
**170**	4-KW-L16-˜	1	VEGFR	0.152493	0.783
**171**	4-KW-M16·	10	VEGFR	0.264877	0.035
**172**	4-KW-N16-	100	VEGFR	0.271887	0.074
**173**	4-KW-O16-	1000	VEGFR	0.316685	0.007
**174**	4-KW-P16-˜	10000	VEGFR	0.314595	0.006
**175**	2-KW-L10-I	1	PI3K	0.177112	0.921
**176**	2-KW-M10·	10	PI3K	0.328524	0
**177**	2-KW-N10-	100	PI3K	0.303561	0.002
**178**	2-KW-O10-	1000	PI3K	0.276567	0.016
**179**	2-KW-P10-I	10000	PI3K	0.34101	0.001
**180**	3-KW-A16-I	2500	PI3K	0.3654	0
**181**	3-KW-C16-I	250	PI3K	0.060615	0.983
**182**	3-KW-D16-	25	PI3K	0.103973	0.855
**183**	3-KW-E16-I	2.5	PI3K	0.149758	0.878
**184**	3-KW-F16-I	0.25	PI3K	0.221734	0.057
**185**	3-KW-F17-I	100000	PI3K	0.298683	0.262
**186**	3-KW-F19-I	500	PI3K	0.306114	0.001
**187**	3-KW-G17-	10000	PI3K	0.205137	0.199
**188**	3-KW-G19-	50	PI3K	0.277619	0.137
**189**	3-KW-H17-	1000	PI3K	0.224744	0.323
**190**	3-KW-I17-N	100	PI3K	0.118526	0.982
**191**	3-KW-I19-D	5	PI3K	0.146424	0.279
**192**	3-KW-J17-N	10	PI3K	0.127337	0.938
**193**	3-KW-J19-D	0.5	PI3K	0.143838	0.859
**194**	3-KW-K19-I	0.05	PI3K	0.177628	0.605
**195**	3-KW-L8-Pi	1	PI3K	0.20848	0.379
**196**	3-KW-L21-˜	0.1	PI3K	0.324269	0
**197**	3-KW-M8-P	10	PI3K	0.271614	0.006
**198**	3-KW-M21·	1	PI3K	0.148968	0.813

**199**	3-KW-N8-P	100	PI3K	0.391619	0
**200**	3-KW-N21-	10	PI3K	0.111169	0.852
**201**	3-KW-O8-P	1000	PI3K	0.370161	0
**202**	3-KW-O21-	100	PI3K	0.348973	0
**203**	3-KW-P8-Pi	10000	PI3K	0.344461	0.001
**204**	3-KW-P21-T	1000	PI3K	0.364851	0
**205**	4-KW-A19-/	2500	PI3K	0.376397	0
**206**	4-KW-B19-/	250	PI3K	0.165315	0.127
**207**	4-KW-C19-/	25	PI3K	0.107283	0.668
**208**	4-KW-D19-	2.5	PI3K	0.117712	0.571
**209**	4-KW-E19-/	0.25	PI3K	0.307922	0.013
**210**	4-KW-F14-I	1000	PI3K	0.366422	0.006
**211**	4-KW-G2-T	2500	PI3K	0.203909	0.341
**212**	4-KW-G5-S	10000	PI3K	0.347732	0.004
**213**	4-KW-G14-	100	PI3K	0.352276	0.001
**214**	4-KW-G20-	10000	PI3K	0.385942	0.001
**215**	4-KW-H2-T	250	PI3K	0.362012	0
**216**	4-KW-H5-S	1000	PI3K	0.356223	0
**217**	4-KW-H14-	10	PI3K	0.380267	0
**218**	4-KW-H20-	1000	PI3K	0.394139	0
**219**	4-KW-I2-TG	25	PI3K	0.105892	0.838
**220**	4-KW-I5-So	100	PI3K	0.186254	0.369
**221**	4-KW-I14-N	1	PI3K	0.309217	0.003
**222**	4-KW-I20-E	100	PI3K	0.231793	0.061
**223**	4-KW-J2-TG	2.5	PI3K	0.078248	0.978
**224**	4-KW-J5-Sc	10	PI3K	0.237516	0.354
**225**	4-KW-J20-E	10	PI3K	0.102725	0.965
**226**	4-KW-K2-T	0.25	PI3K	0.079363	0.947
**227**	4-KW-K4-D	0.1	PI3K	0.160408	0.464
**228**	4-KW-K5-S	1	PI3K	0.39718	0
**229**	4-KW-K14-I	0.1	PI3K	0.312072	0.001
**230**	4-KW-K20-I	1	PI3K	0.391196	0
**231**	4-KW-L4-D	1	PI3K	0.290716	0.089
**232**	4-KW-L14-(	0.1	PI3K	0.344425	0
**233**	4-KW-L15-T	1	PI3K	0.139686	0.617

**234**	4-KW-L21-(0.1	PI3K	0.388129	0
**235**	4-KW-M14- 1	PI3K	0.33589	0
**236**	4-KW-M15- 10	PI3K	0.365484	0
**237**	4-KW-M21- 1	PI3K	0.144229	0.908
**238**	4-KW-N4-D 10	PI3K	0.209438	0.564
**239**	4-KW-N14- 10	PI3K	0.265816	0.08
**240**	4-KW-N15- 100	PI3K	0.406231	0
**241**	4-KW-N21- 10	PI3K	0.361667	0
**242**	4-KW-O4-D 100	PI3K	0.316	0.021
**243**	4-KW-O14- 100	PI3K	0.346813	0.001
**244**	4-KW-O15- 1000	PI3K	0.4018	0
**245**	4-KW-O21- 100	PI3K	0.402414	0
**246**	4-KW-P4-D 1000	PI3K	0.319599	0.013
**247**	4-KW-P14-(1000	PI3K	0.379749	0
**248**	4-KW-P15-1 10000	PI3K	0.344317	0
**249**	4-KW-P21-(1000	PI3K	0.369839	0.001
**250**	5-KW-A6-LY 2500	PI3K	0.31644	0.045
**251**	5-KW-A7-Al 1000	PI3K	0.181466	0.126
**252**	5-KW-A16-, 2500	PI3K	0.347177	0
**253**	5-KW-A17-I 10000	PI3K	0.131749	0.722
**254**	5-KW-B6-LY 250	PI3K	0.253364	0.181
**255**	5-KW-B7-Al 100	PI3K	0.132523	0.383
**256**	5-KW-B17-I 1000	PI3K	0.329583	0
**257**	5-KW-C6-LY 25	PI3K	0.104823	0.958
**258**	5-KW-C7-Al 10	PI3K	0.071939	0.938
**259**	5-KW-C16-, 250	PI3K	0.290886	0.002
**260**	5-KW-C17-I 100	PI3K	0.120284	0.664
**261**	5-KW-D6-LY 2.5	PI3K	0.057759	0.994
**262**	5-KW-D7-A 1	PI3K	0.273298	0.003
**263**	5-KW-D16- 25	PI3K	0.115194	0.75
**264**	5-KW-D17- 10	PI3K	0.279247	0.023
**265**	5-KW-E6-LY 0.25	PI3K	0.125203	0.639
**266**	5-KW-E7-Al 0.1	PI3K	0.257335	0.081
**267**	5-KW-E16-/ 2.5	PI3K	0.081097	1
**268**	5-KW-E17-I 1	PI3K	0.101624	0.96

**269**	5-KW-F11-(	10000	PI3K	0.325757	0
**270**	5-KW-F16-/	0.25	PI3K	0.085706	0.979
**271**	5-KW-G9-S	10000	PI3K	0.35185	0
**272**	5-KW-G11-	1000	PI3K	0.32099	0
**273**	5-KW-H9-S	1000	PI3K	0.343094	0.004
**274**	5-KW-H11-	100	PI3K	0.351016	0.003
**275**	5-KW-I9-Se	100	PI3K	0.225572	0.043
**276**	5-KW-I11-(	10	PI3K	0.22618	0.229
**277**	5-KW-J9-Se	10	PI3K	0.294512	0.011
**278**	5-KW-J11-(	1	PI3K	0.327671	0.022
**279**	5-KW-K9-Se	1	PI3K	0.09825	0.88
**280**	5-KW-L14-/	0.1	PI3K	0.13197	0.953
**281**	5-KW-L20-:	1	PI3K	0.04609	0.996
**282**	5-KW-L23-(	0.1	PI3K	0.131277	0.659
**283**	5-KW-M14-	1	PI3K	0.24671	0.153
**284**	5-KW-M20-	10	PI3K	0.218562	0.501
**285**	5-KW-M23-	1	PI3K	0.066805	0.983
**286**	5-KW-N14-	10	PI3K	0.240184	0.392
**287**	5-KW-N20-	100	PI3K	0.304306	0.053
**288**	5-KW-N23-	10	PI3K	0.359431	0
**289**	5-KW-O14-	100	PI3K	0.226241	0.099
**290**	5-KW-O20-	1000	PI3K	0.390042	0
**291**	5-KW-O23-	100	PI3K	0.349176	0.001
**292**	5-KW-P14-/	1000	PI3K	0.305156	0.023
**293**	5-KW-P20-:	10000	PI3K	0.360477	0
**294**	5-KW-P23-(	1000	PI3K	0.367156	0.004
**295**	6-KW-A8-T(	10000	PI3K	0.363215	0
**296**	6-KW-B8-T(	1000	PI3K	0.377989	0
**297**	6-KW-C8-T(	100	PI3K	0.045193	1
**298**	6-KW-D8-T(	10	PI3K	0.090468	0.996
**299**	6-KW-E8-T(	1	PI3K	0.275312	0.002
**300**	6-KW-L6-G	1	PI3K	0.116635	0.965
**301**	6-KW-M6-(	10	PI3K	0.282434	0.006
**302**	6-KW-N6-G	100	PI3K	0.220448	0.545
**303**	6-KW-O6-G	1000	PI3K	0.387281	0

**304**	6-KW-P6-G	10000	PI3K	0.396711	0
**305**	1-KW-F11-/	10000	Topoisome	0.587181	0
**306**	1-KW-G11-	1000	Topoisome	0.617767	0
**307**	1-KW-G20-	1000	Topoisome	0.54956	0
**308**	1-KW-H11-	100	Topoisome	0.587999	0
**309**	1-KW-H20-	100	Topoisome	0.600476	0
**310**	1-KW-I11-A	10	Topoisome	0.480262	0
**311**	1-KW-I20-E	10	Topoisome	0.512929	0
**312**	1-KW-J11-/	1	Topoisome	0.416199	0.001
**313**	1-KW-J20-E	1	Topoisome	0.112715	0.994
**314**	1-KW-K11-!	1	Topoisome	0.54466	0
**315**	1-KW-K20-I	0.1	Topoisome	0.042032	1
**316**	1-KW-L11-S	10	Topoisome	0.590345	0
**317**	1-KW-L14-T	1	Topoisome	0.473866	0
**318**	1-KW-M11-	100	Topoisome	0.536343	0
**319**	1-KW-M14-	10	Topoisome	0.498811	0
**320**	1-KW-N14-	100	Topoisome	0.604771	0
**321**	1-KW-O11-	1000	Topoisome	0.540373	0
**322**	1-KW-O14-	1000	Topoisome	0.571967	0
**323**	1-KW-P11-!	10000	Topoisome	0.530059	0
**324**	1-KW-P14-T	10000	Topoisome	0.535917	0
**325**	3-KW-A11-I	10000	Topoisome	0.603507	0
**326**	3-KW-B11-I	1000	Topoisome	0.591561	0
**327**	3-KW-C11-I	100	Topoisome	0.463364	0
**328**	3-KW-D11-	10	Topoisome	0.34066	0.007
**329**	3-KW-E11-I	1	Topoisome	0.155008	0.817
**330**	3-KW-G9-D	1000	Topoisome	0.520073	0
**331**	3-KW-G10-	10000	Topoisome	0.051274	0.995
**332**	3-KW-H9-D	100	Topoisome	0.58075	0
**333**	3-KW-H10-	1000	Topoisome	0.196866	0.47
**334**	3-KW-I9-Da	10	Topoisome	0.547992	0
**335**	3-KW-I10-T	100	Topoisome	0.269265	0.102
**336**	3-KW-J9-Da	1	Topoisome	0.480412	0.001
**337**	3-KW-J10-T	10	Topoisome	0.361602	0.038
**338**	3-KW-K7-Ic	0.1	Topoisome	0.253027	0.437

**339**	3-KW-K9-D	0.1	Topoisome	0.491	0
**340**	3-KW-K10-1	1	Topoisome	0.285831	0.139
**341**	3-KW-L6-D	0.1	Topoisome	0.032031	1
**342**	3-KW-L7-Id	1	Topoisome	0.105475	0.939
**343**	3-KW-L9-Va	0.5	Topoisome	0.495583	0
**344**	3-KW-L10-I	0.1	Topoisome	0.491237	0
**345**	3-KW-L16-I	1	Topoisome	0.379607	0.017
**346**	3-KW-M6-D	1	Topoisome	0.339907	0.078
**347**	3-KW-M7-I	10	Topoisome	0.407435	0.008
**348**	3-KW-M9-V	5	Topoisome	0.40156	0.004
**349**	3-KW-M10-	1	Topoisome	0.312097	0.038
**350**	3-KW-M16-	10	Topoisome	0.516687	0
**351**	3-KW-N6-D	10	Topoisome	0.418566	0.023
**352**	3-KW-N9-V	50	Topoisome	0.584136	0
**353**	3-KW-N10-	10	Topoisome	0.501627	0
**354**	3-KW-N16-	100	Topoisome	0.100826	0.948
**355**	3-KW-O6-D	100	Topoisome	0.583843	0
**356**	3-KW-O7-Ic	100	Topoisome	0.617282	0
**357**	3-KW-O9-V	500	Topoisome	0.608549	0
**358**	3-KW-O10-	100	Topoisome	0.599696	0
**359**	3-KW-O16-	1000	Topoisome	0.193488	0.443
**360**	3-KW-P6-D	1000	Topoisome	0.500192	0
**361**	3-KW-P7-Ic	1000	Topoisome	0.523191	0
**362**	3-KW-P9-Va	5000	Topoisome	0.531129	0
**363**	3-KW-P10-I	1000	Topoisome	0.481595	0
**364**	3-KW-P16-I	10000	Topoisome	0.089885	0.949
**365**	1-KW-A10-Y	10000	Mitotic	0.684501	0
**366**	1-KW-A13-I	1000	Mitotic	0.58643	0
**367**	1-KW-A18-I	1000	Mitotic	0.681113	0
**368**	1-KW-B10-Y	1000	Mitotic	0.682845	0
**369**	1-KW-B13-I	100	Mitotic	0.312111	0.274
**370**	1-KW-B18-I	100	Mitotic	0.598643	0
**371**	1-KW-C10-Y	100	Mitotic	0.673069	0
**372**	1-KW-C13-I	10	Mitotic	0.412848	0.015
**373**	1-KW-C18-I	10	Mitotic	0.566112	0

**374**	1-KW-D10-	10	Mitotic	0.568905	0
**375**	1-KW-D13-	1	Mitotic	0.17555	0.702
**376**	1-KW-D18-	1	Mitotic	0.549328	0
**377**	1-KW-E10-\	1	Mitotic	0.497643	0
**378**	1-KW-E13-I	0.1	Mitotic	0.551268	0
**379**	1-KW-E18-I	0.1	Mitotic	0.357865	0.078
**380**	1-KW-F13-\	1000	Mitotic	0.63314	0
**381**	1-KW-G13-	100	Mitotic	0.476298	0
**382**	1-KW-G15-	1000	Mitotic	0.680082	0
**383**	1-KW-H13-	10	Mitotic	0.466759	0
**384**	1-KW-H15-	100	Mitotic	0.687977	0
**385**	1-KW-I13-\	1	Mitotic	0.509524	0
**386**	1-KW-I15-E	10	Mitotic	0.680632	0
**387**	1-KW-J13-\	0.1	Mitotic	0.059988	0.999
**388**	1-KW-J15-E	1	Mitotic	0.605369	0
**389**	1-KW-K7-Vi	0.1	Mitotic	0.207563	0.77
**390**	1-KW-K15-I	0.1	Mitotic	0.53103	0
**391**	1-KW-L7-Vi	1	Mitotic	0.270391	0.42
**392**	1-KW-L20-\	0.1	Mitotic	0.261562	0.373
**393**	1-KW-M7-\	10	Mitotic	0.46903	0
**394**	1-KW-M20-	1	Mitotic	0.287472	0.254
**395**	1-KW-N20-	10	Mitotic	0.411059	0.008
**396**	1-KW-O7-V	100	Mitotic	0.098448	0.987
**397**	1-KW-O20-	100	Mitotic	0.335178	0.384
**398**	1-KW-P7-Vi	1000	Mitotic	0.665851	0
**399**	1-KW-P20-'	1000	Mitotic	0.540085	0
**400**	3-KW-A7-D	1000	Mitotic	0.637024	0
**401**	3-KW-B7-D	100	Mitotic	0.650438	0
**402**	3-KW-C7-D	10	Mitotic	0.662713	0
**403**	3-KW-D7-D	1	Mitotic	0.235154	0.337
**404**	3-KW-E7-D	0.1	Mitotic	0.16359	0.817
**405**	6-KW-L19-/	1	Mitotic	0.393467	0.046
**406**	6-KW-M19-	10	Mitotic	0.216724	0.681
**407**	6-KW-N19-	100	Mitotic	0.229966	0.706
**408**	6-KW-O19-	1000	Mitotic	0.647013	0

**409**	6-KW-P19-/	10000	Mitotic	0.641955	0
**410**	2-KW-A12-1	250	MEK1/2	0.488103	0.006
**411**	2-KW-B12-1	25	MEK1/2	0.492105	0.005
**412**	2-KW-D12-	2.5	MEK1/2	0.540189	0.001
**413**	2-KW-E12-1	0.25	MEK1/2	0.409851	0.011
**414**	2-KW-F12-1	2.5000000	MEK1/2	0.260174	0.559
**415**	2-KW-F14-(	1000	MEK1/2	0.496041	0.001
**416**	2-KW-G14-	100	MEK1/2	0.524341	0.001
**417**	2-KW-H14-	10	MEK1/2	0.521222	0.003
**418**	2-KW-I14-C	1	MEK1/2	0.440258	0.01
**419**	2-KW-K14-	0.1	MEK1/2	0.23999	0.847
**420**	2-KW-L20-S	1	MEK1/2	0.223172	0.814
**421**	2-KW-M20-	10	MEK1/2	0.210457	0.977
**422**	2-KW-N20-	100	MEK1/2	0.291601	0.665
**423**	2-KW-O20-	1000	MEK1/2	0.422305	0.149
**424**	2-KW-P20-S	10000	MEK1/2	0.476015	0.01
**425**	4-KW-A10-I	1000	MEK1/2	0.649502	0
**426**	4-KW-A13-I	1000	MEK1/2	0.608811	0
**427**	4-KW-B10-I	100	MEK1/2	0.640188	0
**428**	4-KW-B13-I	100	MEK1/2	0.617471	0
**429**	4-KW-C10-I	10	MEK1/2	0.528846	0.001
**430**	4-KW-C13-I	10	MEK1/2	0.568604	0.001
**431**	4-KW-D10-	1	MEK1/2	0.518144	0.005
**432**	4-KW-D13-	1	MEK1/2	0.439513	0.014
**433**	4-KW-E10-I	0.1	MEK1/2	0.563138	0
**434**	4-KW-E13-I	0.1	MEK1/2	0.553215	0
**435**	4-KW-L19-(	0.25	MEK1/2	0.209942	0.723
**436**	4-KW-M19-	2.5	MEK1/2	0.175603	0.952
**437**	4-KW-N19-	25	MEK1/2	0.265009	0.362
**438**	4-KW-O19-	250	MEK1/2	0.195615	0.702
**439**	4-KW-P19-	2500	MEK1/2	0.61914	0
**440**	1-KW-L2-OI	1	PARP	0.339992	0.121
**441**	1-KW-L6-Ru	1	PARP	0.559433	0.007
**442**	1-KW-M2-C	10	PARP	0.427302	0.028
**443**	1-KW-M6-F	10	PARP	0.426069	0.175

**444**	1-KW-N2-O	100	PARP	0.700768	0
**445**	1-KW-N6-R	100	PARP	0.552142	0.006
**446**	1-KW-O2-O	1000	PARP	0.688653	0
**447**	1-KW-O6-R	1000	PARP	0.440039	0.062
**448**	1-KW-P2-O	10000	PARP	0.4412	0.104
**449**	1-KW-P6-R	10000	PARP	0.348173	0.298
**450**	7-KW-A3-Ta	1000	PARP	0.596599	0.001
**451**	7-KW-B2-Ve	10000	PARP	0.709796	0
**452**	7-KW-B3-Ta	100	PARP	0.540057	0.014
**453**	7-KW-C2-Ve	1000	PARP	0.606215	0.001
**454**	7-KW-C3-Ta	10	PARP	0.705996	0
**455**	7-KW-D2-V	100	PARP	0.560509	0.001
**456**	7-KW-D3-Ta	1	PARP	0.420745	0.024
**457**	7-KW-E2-Ve	10	PARP	0.456532	0.125
**458**	7-KW-E3-Ta	0.1	PARP	0.308131	0.217
**459**	7-KW-F2-Ve	1	PARP	0.376914	0.101
**460**	7-KW-G2-N	10000	PARP	0.608393	0.002
**461**	7-KW-H2-N	1000	PARP	0.693609	0
**462**	7-KW-I2-Ni	100	PARP	0.52679	0.009
**463**	7-KW-J2-Ni	10	PARP	0.392202	0.067
**464**	7-KW-K2-N	1	PARP	0.372727	0.067
**465**	3-KW-A19-	1000	CDK	0.453412	0.01
**466**	3-KW-B19-	100	CDK	0.434897	0.013
**467**	3-KW-B23-	2500	CDK	0.43927	0
**468**	3-KW-C19-	10	CDK	0.435049	0.001
**469**	3-KW-C23-	250	CDK	0.381838	0.005
**470**	3-KW-D19-	1	CDK	0.32194	0.143
**471**	3-KW-D23-	25	CDK	0.331309	0.202
**472**	3-KW-E19-	0.1	CDK	0.425978	0
**473**	3-KW-E23-	2.5	CDK	0.080995	0.975
**474**	3-KW-F23-	0.25	CDK	0.07144	0.989
**475**	3-KW-K17-	1	CDK	0.365231	0.007
**476**	3-KW-L19-	1	CDK	0.269558	0.332
**477**	3-KW-M17-	10	CDK	0.390062	0.002
**478**	3-KW-M19-	10	CDK	0.328176	0.17

**479**	3-KW-N17-	100	CDK	0.459303	0
**480**	3-KW-N19-	100	CDK	0.396385	0.003
**481**	3-KW-O17-	1000	CDK	0.37854	0.002
**482**	3-KW-O19-	1000	CDK	0.371786	0.007
**483**	3-KW-P17-I	10000	CDK	0.399125	0.01
**484**	3-KW-P19-I	10000	CDK	0.399365	0.002
**485**	4-KW-A4-SI	10000	CDK	0.508392	0.001
**486**	4-KW-A8-M	10000	CDK	0.516403	0.001
**487**	4-KW-B4-SI	1000	CDK	0.493343	0.003
**488**	4-KW-B8-M	1000	CDK	0.389623	0.003
**489**	4-KW-C4-SI	100	CDK	0.432093	0.005
**490**	4-KW-C8-M	100	CDK	0.33942	0.025
**491**	4-KW-D4-SI	10	CDK	0.057945	0.981
**492**	4-KW-D8-M	10	CDK	0.143882	0.721
**493**	4-KW-E4-SI	1	CDK	0.252651	0.307
**494**	4-KW-E8-M	1	CDK	0.271749	0.142
**495**	4-KW-F4-Se	10000	CDK	0.417126	0.003
**496**	4-KW-F22-/	10000	CDK	0.498819	0.003
**497**	4-KW-G4-S	1000	CDK	0.063947	0.998
**498**	4-KW-G22-	1000	CDK	0.48003	0.004
**499**	4-KW-H4-S	100	CDK	0.392437	0.006
**500**	4-KW-H22-	100	CDK	0.42611	0.001
**501**	4-KW-I4-Se	10	CDK	0.101439	0.911
**502**	4-KW-I22-A	10	CDK	0.381434	0.002
**503**	4-KW-J4-Se	1	CDK	0.079744	0.962
**504**	4-KW-J22-/	1	CDK	0.271118	0.309
**505**	5-KW-A19-,	10000	CDK	0.478879	0.01
**506**	5-KW-B19-,	1000	CDK	0.413321	0.011
**507**	5-KW-C19-,	100	CDK	0.374148	0.017
**508**	5-KW-D19-	10	CDK	0.075925	0.979
**509**	5-KW-E19-/	1	CDK	0.229997	0.374
**510**	5-KW-K17-,	1	CDK	0.138524	0.942
**511**	5-KW-M17-	10	CDK	0.33903	0.098
**512**	5-KW-N17-	100	CDK	0.351539	0.016
**513**	5-KW-O17-	1000	CDK	0.442009	0.021

514	5-KW-P17-/	10000	CDK	0.481018	0.009
515	6-KW-A17-!	1000	CDK	0.324559	0.144
516	6-KW-B17-!	100	CDK	0.364181	0.05
517	6-KW-C17-!	10	CDK	0.345981	0.016
518	6-KW-D17-	1	CDK	0.356212	0.002
519	6-KW-E17-!	0.1	CDK	0.294823	0.085
520	6-KW-L15-7	1	CDK	0.276027	0.206
521	6-KW-M15-	10	CDK	0.315803	0.202
522	6-KW-N15-	100	CDK	0.414338	0.001
523	6-KW-O15-	1000	CDK	0.489951	0.005
524	6-KW-P15-7	10000	CDK	0.485113	0.039
525	7-KW-A21-	10000	BET	0.471982	0.002
526	7-KW-A22-I	30000	BET	0.572135	0
527	7-KW-B21-	1000	BET	0.441765	0.004
528	7-KW-B22-I	3000	BET	0.523965	0
529	7-KW-C21-	100	BET	0.244843	0.315
530	7-KW-C22-I	300	BET	0.281201	0.071
531	7-KW-D21-	10	BET	0.630812	0
532	7-KW-D22-	30	BET	0.322543	0.201
533	7-KW-E21-	1	BET	0.312234	0.395
534	7-KW-E22-I	3	BET	0.335418	0.283
535	7-KW-G10-	10000	BET	0.610148	0
536	7-KW-G15-	10000	BET	0.618987	0
537	7-KW-H10-	1000	BET	0.620308	0
538	7-KW-H15-	1000	BET	0.612876	0
539	7-KW-I10-E	100	BET	0.567857	0
540	7-KW-I15-I	100	BET	0.420551	0.002
541	7-KW-J10-E	10	BET	0.260556	0.24
542	7-KW-J15-I	10	BET	0.25787	0.487
543	7-KW-K10-I	1	BET	0.183947	0.729
544	7-KW-K13-I	1	BET	0.277321	0.533
545	7-KW-K15-I	1	BET	0.240175	0.65
546	7-KW-L12-I	1	BET	0.253075	0.546
547	7-KW-L13-I	10	BET	0.290608	0.167
548	7-KW-L20-J	1	BET	0.201087	0.784

**549**	7-KW-L23-/	0.03	BET	0.200026	0.619
**550**	7-KW-M12-	10	BET	0.471888	0.002
**551**	7-KW-M13-	100	BET	0.609031	0
**552**	7-KW-M20-	10	BET	0.137776	0.953
**553**	7-KW-M23-	0.3	BET	0.192785	0.766
**554**	7-KW-N12-	100	BET	0.286046	0.496
**555**	7-KW-N13-	1000	BET	0.58755	0
**556**	7-KW-N20-	100	BET	0.466207	0
**557**	7-KW-N23-	3	BET	0.201823	0.45
**558**	7-KW-O12-	1000	BET	0.5611	0
**559**	7-KW-O20-	1000	BET	0.623996	0
**560**	7-KW-O23-	30	BET	0.697575	0
**561**	7-KW-P12-I	10000	BET	0.609333	0
**562**	7-KW-P13-I	10000	BET	0.572058	0.001
**563**	7-KW-P20-.	10000	BET	0.598034	0
**564**	7-KW-P23-,	300	BET	0.592099	0
**565**	8-KW-K22-(	1	BET	0.162729	0.581
**566**	8-KW-L22-(	10	BET	0.213001	0.426
**567**	8-KW-M22-	100	BET	0.165085	0.828
**568**	8-KW-N22-	1000	BET	0.381479	0.051
**569**	8-KW-O22-	10000	BET	0.435294	0.003
**570**	1-KW-A3-V	10000	HDAC	0.412281	0.002
**571**	1-KW-B3-V	1000	HDAC	0.34861	0.001
**572**	1-KW-C3-V	100	HDAC	0.204846	0.37
**573**	1-KW-D3-V	10	HDAC	0.109171	0.901
**574**	1-KW-E3-V	1	HDAC	0.054447	0.973
**575**	1-KW-L12-I	0.1	HDAC	0.256814	0.028
**576**	1-KW-M12-	1	HDAC	0.40261	0
**577**	1-KW-N12-	10	HDAC	0.414832	0.006
**578**	1-KW-O12-	100	HDAC	0.481821	0
**579**	1-KW-P12-I	1000	HDAC	0.465935	0.001
**580**	3-KW-A4-P	1000	HDAC	0.416174	0.003
**581**	3-KW-B4-P	100	HDAC	0.352114	0.011
**582**	3-KW-C4-P	10	HDAC	0.366931	0.011
**583**	3-KW-D4-P	1	HDAC	0.050078	0.994

**584**	3-KW-E4-Pa	0.1	HDAC	0.283574	0.047
**585**	3-KW-F7-Q	1000	HDAC	0.432759	0.002
**586**	3-KW-G7-Q	100	HDAC	0.276607	0.124
**587**	3-KW-G12-	1000000	HDAC	0.112579	0.788
**588**	3-KW-H7-Q	10	HDAC	0.349493	0.002
**589**	3-KW-H12-	100000	HDAC	0.106429	0.735
**590**	3-KW-I7-Qu	1	HDAC	0.257845	0.031
**591**	3-KW-I12-V	10000	HDAC	0.113325	0.735
**592**	3-KW-J7-Qu	0.1	HDAC	0.274312	0.045
**593**	3-KW-J12-V	1000	HDAC	0.171818	0.369
**594**	3-KW-K3-B	1	HDAC	0.071442	0.993
**595**	3-KW-K12-	100	HDAC	0.37194	0
**596**	3-KW-L3-Be	10	HDAC	0.104726	0.978
**597**	3-KW-M3-E	100	HDAC	0.305639	0.012
**598**	3-KW-N3-B	1000	HDAC	0.296602	0.089
**599**	3-KW-O3-B	10000	HDAC	0.488427	0.001
**600**	7-KW-A5-M	10000	HDAC	0.562281	0
**601**	7-KW-A7-C	10000	HDAC	0.621916	0
**602**	7-KW-A9-G	1000	HDAC	0.490352	0
**603**	7-KW-A12-	10000	HDAC	0.488073	0
**604**	7-KW-B5-M	1000	HDAC	0.44979	0
**605**	7-KW-B7-C	1000	HDAC	0.575689	0
**606**	7-KW-B12-	1000	HDAC	0.642562	0
**607**	7-KW-C5-M	100	HDAC	0.583436	0
**608**	7-KW-C7-C	100	HDAC	0.533727	0
**609**	7-KW-C9-G	100	HDAC	0.514078	0
**610**	7-KW-D7-C	10	HDAC	0.094672	0.867
**611**	7-KW-D9-G	10	HDAC	0.479835	0
**612**	7-KW-D12-	100	HDAC	0.415988	0
**613**	7-KW-E5-M	10	HDAC	0.317596	0.007
**614**	7-KW-E7-C	1	HDAC	0.276113	0.028
**615**	7-KW-E9-G	1	HDAC	0.350922	0
**616**	7-KW-E12-	10	HDAC	0.198458	0.272
**617**	7-KW-F5-M	1	HDAC	0.1114	0.748
**618**	7-KW-F7-Re	10000	HDAC	0.567958	0

**619**	7-KW-F9-Gi	0.1	HDAC	0.206708	0.174
**620**	7-KW-F12-I	1	HDAC	0.559612	0
**621**	7-KW-F19-I	10000	HDAC	0.295949	0.043
**622**	7-KW-G7-R	1000	HDAC	0.467803	0
**623**	7-KW-G19-	1000	HDAC	0.198125	0.448
**624**	7-KW-H7-R	100	HDAC	0.438397	0
**625**	7-KW-I7-Re	10	HDAC	0.260504	0.097
**626**	7-KW-I19-P	100	HDAC	0.26051	0.113
**627**	7-KW-J7-Re	1	HDAC	0.513905	0
**628**	7-KW-J19-F	10	HDAC	0.04867	0.98
**629**	7-KW-K4-Er	1	HDAC	0.316534	0.028
**630**	7-KW-K11-/	1	HDAC	0.442308	0
**631**	7-KW-K18-'	1	HDAC	0.339618	0.001
**632**	7-KW-K19-I	1	HDAC	0.435447	0
**633**	7-KW-L2-Ta	0.1	HDAC	0.184981	0.311
**634**	7-KW-L4-Er	10	HDAC	0.585484	0
**635**	7-KW-L5-Pr	1	HDAC	0.15852	0.443
**636**	7-KW-L8-Al	1	HDAC	0.070174	0.98
**637**	7-KW-L10-'	1	HDAC	0.453074	0
**638**	7-KW-L11-/	10	HDAC	0.526912	0
**639**	7-KW-L14-'	1	HDAC	0.353161	0
**640**	7-KW-L16-I	1	HDAC	0.515528	0
**641**	7-KW-L18-'	10	HDAC	0.503877	0
**642**	7-KW-M2-T	1	HDAC	0.125612	0.926
**643**	7-KW-M5-F	10	HDAC	0.118697	0.968
**644**	7-KW-M8-A	10	HDAC	0.273851	0.012
**645**	7-KW-M10-	10	HDAC	0.453044	0
**646**	7-KW-M11-	100	HDAC	0.514668	0
**647**	7-KW-M14-	10	HDAC	0.39227	0.005
**648**	7-KW-M16-	10	HDAC	0.616368	0
**649**	7-KW-M18-	100	HDAC	0.600572	0
**650**	7-KW-N2-Ta	10	HDAC	0.223965	0.128
**651**	7-KW-N4-Er	100	HDAC	0.45281	0
**652**	7-KW-N5-P	100	HDAC	0.412871	0
**653**	7-KW-N8-A	100	HDAC	0.503993	0

**654**	7-KW-N10-	100	HDAC	0.451212	0
**655**	7-KW-N14-	100	HDAC	0.133111	0.695
**656**	7-KW-N16-	100	HDAC	0.215629	0.25
**657**	7-KW-N18-	1000	HDAC	0.32932	0.009
**658**	7-KW-O2-Ta	100	HDAC	0.502322	0
**659**	7-KW-O4-Er	1000	HDAC	0.467521	0
**660**	7-KW-O5-P	1000	HDAC	0.457954	0.001
**661**	7-KW-O8-A	1000	HDAC	0.463142	0.001
**662**	7-KW-O10-	1000	HDAC	0.488676	0
**663**	7-KW-O11-	1000	HDAC	0.418993	0.001
**664**	7-KW-O14-	1000	HDAC	0.404635	0
**665**	7-KW-O16-	1000	HDAC	0.44198	0
**666**	7-KW-P2-Ta	1000	HDAC	0.543865	0
**667**	7-KW-P4-Er	10000	HDAC	0.447507	0.001
**668**	7-KW-P5-Pr	10000	HDAC	0.556805	0
**669**	7-KW-P8-Al	10000	HDAC	0.542978	0
**670**	7-KW-P10-T	10000	HDAC	0.436844	0
**671**	7-KW-P11-A	10000	HDAC	0.57875	0
**672**	7-KW-P14-T	10000	HDAC	0.489033	0
**673**	7-KW-P16-I	10000	HDAC	0.407911	0.001
**674**	7-KW-P18-T	10000	HDAC	0.455479	0
**0**	2-MHB-A16	10000	EGFR	0.589009	0
**1**	2-MHB-A19	10000	EGFR	0.591109	0
**2**	2-MHB-B19	1000	EGFR	0.54727	0
**3**	2-MHB-C16	1000	EGFR	0.61128	0
**4**	2-MHB-C19	100	EGFR	0.538754	0
**5**	2-MHB-D16	100	EGFR	0.644239	0
**6**	2-MHB-D19	10	EGFR	0.080064	0.992
**7**	2-MHB-E16	10	EGFR	0.493124	0
**8**	2-MHB-E19	1	EGFR	0.08861	0.986
**9**	2-MHB-F16	1	EGFR	0.522831	0
**10**	2-MHB-K11	0.1	EGFR	0.400526	0
**11**	2-MHB-L11	1	EGFR	0.470372	0
**12**	2-MHB-L16	0.25	EGFR	0.258147	0.067
**13**	2-MHB-L19	0.1	EGFR	0.609086	0

**14**	2-MHB-M1	10	EGFR	0.64331	0
**15**	2-MHB-M1	2.5	EGFR	0.553633	0
**16**	2-MHB-M1	1	EGFR	0.425439	0
**17**	2-MHB-N1	25	EGFR	0.16202	0.402
**18**	2-MHB-N1	10	EGFR	0.621288	0
**19**	2-MHB-O1	100	EGFR	0.604028	0
**20**	2-MHB-O1	250	EGFR	0.676303	0
**21**	2-MHB-O1	100	EGFR	0.666587	0
**22**	2-MHB-P11	1000	EGFR	0.601429	0
**23**	2-MHB-P1	2500	EGFR	0.583381	0
**24**	2-MHB-P1	1000	EGFR	0.604638	0
**25**	3-MHB-F21	10000	EGFR	0.14674	0.953
**26**	3-MHB-G2	1000	EGFR	0.590581	0
**27**	3-MHB-G2	1000	EGFR	0.63796	0
**28**	3-MHB-H2	100	EGFR	0.616028	0
**29**	3-MHB-H2	100	EGFR	0.050062	0.996
**30**	3-MHB-I20	10	EGFR	0.593161	0
**31**	3-MHB-I21	10	EGFR	0.032122	1
**32**	3-MHB-J20	1	EGFR	0.050281	0.971
**33**	3-MHB-J21	1	EGFR	0.056536	1
**34**	3-MHB-K4-	1	EGFR	0.335411	0.035
**35**	3-MHB-K18	0.1	EGFR	0.48239	0
**36**	3-MHB-K2	0.1	EGFR	0.484437	0
**37**	3-MHB-L4-	10	EGFR	0.467995	0
**38**	3-MHB-L18	1	EGFR	0.564721	0
**39**	3-MHB-M1	10	EGFR	0.590791	0
**40**	3-MHB-N4-	100	EGFR	0.520808	0
**41**	3-MHB-N18	100	EGFR	0.579013	0
**42**	3-MHB-O4-	1000	EGFR	0.587182	0
**43**	3-MHB-P4-	10000	EGFR	0.180208	0.937
**44**	3-MHB-P18	1000	EGFR	0.616872	0
**45**	4-MHB-F13	1000	EGFR	0.594996	0
**46**	4-MHB-G1	100	EGFR	0.648775	0
**47**	4-MHB-G1	10000	EGFR	0.609493	0
**48**	4-MHB-H1	10	EGFR	0.643844	0

**49**	4-MHB-H16	1000	EGFR	0.637034	0
**50**	4-MHB-I13-	1	EGFR	0.519601	0
**51**	4-MHB-I16-	100	EGFR	0.611197	0
**52**	4-MHB-J13	0.1	EGFR	0.200772	0.144
**53**	4-MHB-J16	10	EGFR	0.069186	0.87
**54**	4-MHB-K7-I	1	EGFR	0.452043	0
**55**	4-MHB-K13	0.1	EGFR	0.13675	0.529
**56**	4-MHB-K16	1	EGFR	0.082188	0.823
**57**	4-MHB-L7-I	10	EGFR	0.492871	0
**58**	4-MHB-L13	1	EGFR	0.585472	0
**59**	4-MHB-M7-	100	EGFR	0.643093	0
**60**	4-MHB-M1	10	EGFR	0.682412	0
**61**	4-MHB-N13	100	EGFR	0.600323	0
**62**	4-MHB-O7-	1000	EGFR	0.616828	0
**63**	4-MHB-P7-I	10000	EGFR	0.590227	0
**64**	4-MHB-P13	1000	EGFR	0.591234	0
**65**	5-MHB-F4-I	1000	EGFR	0.510697	0
**66**	5-MHB-F7-A	1000	EGFR	0.592934	0
**67**	5-MHB-G4-	100	EGFR	0.561128	0
**68**	5-MHB-G7-	100	EGFR	0.643433	0
**69**	5-MHB-H4-	10	EGFR	0.530778	0
**70**	5-MHB-H7-	10	EGFR	0.512869	0
**71**	5-MHB-I4-F	1	EGFR	0.541699	0
**72**	5-MHB-I7-A	1	EGFR	0.198961	0.118
**73**	5-MHB-J4-F	0.1	EGFR	0.188892	0.686
**74**	5-MHB-J7-A	0.1	EGFR	0.089379	0.819
**75**	5-MHB-K7-	0.1	EGFR	0.458432	0
**76**	5-MHB-L7-(	1	EGFR	0.069052	0.885
**77**	5-MHB-M7-	10	EGFR	0.386271	0
**78**	5-MHB-O7-	100	EGFR	0.057052	0.962
**79**	5-MHB-P7-	1000	EGFR	0.449813	0
**80**	2-MHB-A15	2500	VEGFR	0.131375	0.943
**81**	2-MHB-A17	10000	VEGFR	0.123015	0.969
**82**	2-MHB-A20	10000	VEGFR	0.102158	0.997
**83**	2-MHB-B15	250	VEGFR	0.238331	0.532

**84**	2-MHB-B17	1000	VEGFR	0.187059	0.685
**85**	2-MHB-B2(	1000	VEGFR	0.244841	0.503
**86**	2-MHB-C15	25	VEGFR	0.283751	0.143
**87**	2-MHB-C17	100	VEGFR	0.251663	0.343
**88**	2-MHB-D1!	2.5	VEGFR	0.370219	0
**89**	2-MHB-D1;	10	VEGFR	0.225452	0.394
**90**	2-MHB-D2(	100	VEGFR	0.338255	0.001
**91**	2-MHB-E17	1	VEGFR	0.398354	0
**92**	2-MHB-E20	10	VEGFR	0.374892	0
**93**	2-MHB-F13	10000	VEGFR	0.094884	0.996
**94**	2-MHB-F15	0.25	VEGFR	0.406625	0
**95**	2-MHB-F19	10000	VEGFR	0.076441	1
**96**	2-MHB-F20	1	VEGFR	0.41606	0
**97**	2-MHB-F21	10000	VEGFR	0.211527	0.104
**98**	2-MHB-G1(	10000	VEGFR	0.319075	0
**99**	2-MHB-G1:	1000	VEGFR	0.160658	0.927
**100**	2-MHB-G1!	1000	VEGFR	0.282589	0.163
**101**	2-MHB-G2:	1000	VEGFR	0.310248	0.017
**102**	2-MHB-H1(	1000	VEGFR	0.358178	0.002
**103**	2-MHB-H1:	100	VEGFR	0.37243	0.001
**104**	2-MHB-H2:	100	VEGFR	0.415236	0
**105**	2-MHB-I10-	100	VEGFR	0.301357	0.104
**106**	2-MHB-I13-	10	VEGFR	0.365295	0
**107**	2-MHB-I19-	100	VEGFR	0.107582	0.995
**108**	2-MHB-I21-	10	VEGFR	0.363591	0
**109**	2-MHB-J10	10	VEGFR	0.382048	0
**110**	2-MHB-J13	1	VEGFR	0.388401	0
**111**	2-MHB-J19	10	VEGFR	0.388254	0
**112**	2-MHB-J21	1	VEGFR	0.318031	0
**113**	2-MHB-K1(	1	VEGFR	0.373127	0
**114**	2-MHB-K13	0.1	VEGFR	0.354872	0
**115**	2-MHB-K17	1	VEGFR	0.388635	0
**116**	2-MHB-K1!	1	VEGFR	0.133397	0.887
**117**	2-MHB-L12	0.1	VEGFR	0.391863	0
**118**	2-MHB-L13	1	VEGFR	0.405564	0

**119**	2-MHB-L21	0.1	VEGFR	0.369587	0
**120**	2-MHB-M1	1	VEGFR	0.404104	0
**121**	2-MHB-M1	10	VEGFR	0.401966	0
**122**	2-MHB-M1	10	VEGFR	0.371671	0
**123**	2-MHB-M2	1	VEGFR	0.356227	0
**124**	2-MHB-N12	10	VEGFR	0.407281	0
**125**	2-MHB-N13	100	VEGFR	0.302242	0.13
**126**	2-MHB-N17	100	VEGFR	0.281641	0.009
**127**	2-MHB-N21	10	VEGFR	0.245309	0.071
**128**	2-MHB-O12	100	VEGFR	0.137366	0.955
**129**	2-MHB-O17	1000	VEGFR	0.205865	0.176
**130**	2-MHB-O21	100	VEGFR	0.273771	0
**131**	2-MHB-P12	1000	VEGFR	0.189382	0.764
**132**	2-MHB-P13	1000	VEGFR	0.059226	1
**133**	2-MHB-P17	10000	VEGFR	0.203788	0.687
**134**	2-MHB-P21	1000	VEGFR	0.074184	0.993
**135**	3-MHB-A3-	1000	VEGFR	0.154668	0.703
**136**	3-MHB-A6-	1000	VEGFR	0.093359	1
**137**	3-MHB-A18	1000	VEGFR	0.115712	0.941
**138**	3-MHB-B3-	100	VEGFR	0.189138	0.65
**139**	3-MHB-B6-	100	VEGFR	0.087616	0.999
**140**	3-MHB-B18	100	VEGFR	0.203404	0.561
**141**	3-MHB-C3-	10	VEGFR	0.08537	0.995
**142**	3-MHB-C6-	10	VEGFR	0.123362	0.962
**143**	3-MHB-C18	10	VEGFR	0.113225	0.967
**144**	3-MHB-D3-	1	VEGFR	0.089937	0.964
**145**	3-MHB-D6-	1	VEGFR	0.098372	0.949
**146**	3-MHB-D18	1	VEGFR	0.115846	0.913
**147**	3-MHB-E3-	0.1	VEGFR	0.162624	0.826
**148**	3-MHB-E6-	0.1	VEGFR	0.312009	0.002
**149**	3-MHB-E18	0.1	VEGFR	0.101022	0.994
**150**	3-MHB-F18	1000	VEGFR	0.141493	0.957
**151**	3-MHB-G18	100	VEGFR	0.274114	0.156
**152**	3-MHB-H18	10	VEGFR	0.208248	0.045
**153**	3-MHB-I18-	1	VEGFR	0.1026	0.858

**154**	3-MHB-J18	0.1	VEGFR	0.17417	0.235
**155**	4-MHB-A12	10000	VEGFR	0.317887	0.549
**156**	4-MHB-A15	2500	VEGFR	0.177023	0.96
**157**	4-MHB-A2(	10000	VEGFR	0.225623	0.471
**158**	4-MHB-B12	1000	VEGFR	0.082297	0.999
**159**	4-MHB-B15	250	VEGFR	0.323371	0
**160**	4-MHB-B2(	1000	VEGFR	0.237255	0.153
**161**	4-MHB-C15	25	VEGFR	0.249895	0.018
**162**	4-MHB-D12	100	VEGFR	0.274565	0.025
**163**	4-MHB-D15	2.5	VEGFR	0.286234	0.061
**164**	4-MHB-D2(	100	VEGFR	0.053661	0.995
**165**	4-MHB-E12	10	VEGFR	0.312023	0
**166**	4-MHB-E20	10	VEGFR	0.277401	0.265
**167**	4-MHB-F12	1	VEGFR	0.32448	0.002
**168**	4-MHB-F15	0.25	VEGFR	0.293521	0.009
**169**	4-MHB-F20	1	VEGFR	0.225962	0.496
**170**	4-MHB-L16	1	VEGFR	0.323303	0.016
**171**	4-MHB-M1	10	VEGFR	0.242171	0.243
**172**	4-MHB-N16	100	VEGFR	0.314926	0.003
**173**	4-MHB-O16	1000	VEGFR	0.307485	0
**174**	4-MHB-P16	10000	VEGFR	0.182494	0.471
**175**	2-MHB-L10	1	PI3K	0.152414	0.826
**176**	2-MHB-M1	10	PI3K	0.167908	0.701
**177**	2-MHB-N1(	100	PI3K	0.178001	0.35
**178**	2-MHB-O1(	1000	PI3K	0.327506	0
**179**	2-MHB-P1(	10000	PI3K	0.377601	0.002
**180**	3-MHB-A16	2500	PI3K	0.20248	0.673
**181**	3-MHB-C16	250	PI3K	0.070864	0.971
**182**	3-MHB-D16	25	PI3K	0.106708	0.622
**183**	3-MHB-E16	2.5	PI3K	0.202645	0.373
**184**	3-MHB-F16	0.25	PI3K	0.233273	0.413
**185**	3-MHB-F17	100000	PI3K	0.179071	0.975
**186**	3-MHB-F19	500	PI3K	0.300509	0.059
**187**	3-MHB-G17	10000	PI3K	0.214889	0.17
**188**	3-MHB-G1!	50	PI3K	0.08458	0.959

**189**	3-MHB-H1[illegible]	1000	PI3K	0.156273	0.568
**190**	3-MHB-I17[illegible]	100	PI3K	0.113117	0.592
**191**	3-MHB-I19[illegible]	5	PI3K	0.091746	0.99
**192**	3-MHB-J17	10	PI3K	0.463546	0
**193**	3-MHB-J19	0.5	PI3K	0.154223	0.726
**194**	3-MHB-K1[illegible]	0.05	PI3K	0.181743	0.136
**195**	3-MHB-L8-[illegible]	1	PI3K	0.195246	0.409
**196**	3-MHB-L21	0.1	PI3K	0.281511	0.06
**197**	3-MHB-M8[illegible]	10	PI3K	0.223495	0.026
**198**	3-MHB-M2[illegible]	1	PI3K	0.115579	0.948
**199**	3-MHB-N8-	100	PI3K	0.355295	0.007
**200**	3-MHB-N2[illegible]	10	PI3K	0.477409	0
**201**	3-MHB-O8-	1000	PI3K	0.273922	0.425
**202**	3-MHB-O2[illegible]	100	PI3K	0.39456	0.002
**203**	3-MHB-P8-[illegible]	10000	PI3K	0.32148	0.09
**204**	3-MHB-P21	1000	PI3K	0.391254	0
**205**	4-MHB-A1[illegible]	2500	PI3K	0.433239	0
**206**	4-MHB-B1[illegible]	250	PI3K	0.361959	0.022
**207**	4-MHB-C1[illegible]	25	PI3K	0.141938	0.593
**208**	4-MHB-D1[illegible]	2.5	PI3K	0.107109	0.749
**209**	4-MHB-E19	0.25	PI3K	0.156522	0.258
**210**	4-MHB-G2-	2500	PI3K	0.06188	0.999
**211**	4-MHB-G5-	10000	PI3K	0.247568	0.514
**212**	4-MHB-G1[illegible]	100	PI3K	0.419035	0
**213**	4-MHB-G2[illegible]	10000	PI3K	0.215602	0.868
**214**	4-MHB-H2-	250	PI3K	0.14187	0.683
**215**	4-MHB-H5-	1000	PI3K	0.217437	0.542
**216**	4-MHB-H1[illegible]	10	PI3K	0.362771	0.006
**217**	4-MHB-H2[illegible]	1000	PI3K	0.378039	0.018
**218**	4-MHB-I2-T	25	PI3K	0.051711	1
**219**	4-MHB-I5-S	100	PI3K	0.189962	0.723
**220**	4-MHB-I14[illegible]	1	PI3K	0.290396	0
**221**	4-MHB-I20[illegible]	100	PI3K	0.352485	0
**222**	4-MHB-J2-[illegible]	2.5	PI3K	0.083581	0.98
**223**	4-MHB-J5-[illegible]	10	PI3K	0.241823	0.231

**224**	4-MHB-J20	10	PI3K	0.181211	0.077
**225**	4-MHB-K2-[illegible]	0.25	PI3K	0.101218	0.984
**226**	4-MHB-K4-[illegible]	0.1	PI3K	0.109362	0.887
**227**	4-MHB-K5-[illegible]	1	PI3K	0.178285	0.253
**228**	4-MHB-K14	0.1	PI3K	0.172793	0.063
**229**	4-MHB-K2[illegible]	1	PI3K	0.213784	0.033
**230**	4-MHB-L4-[illegible]	1	PI3K	0.155497	0.659
**231**	4-MHB-L14	0.1	PI3K	0.277024	0.008
**232**	4-MHB-L15	1	PI3K	0.251457	0.002
**233**	4-MHB-L21	0.1	PI3K	0.324634	0
**234**	4-MHB-M1[illegible]	1	PI3K	0.237718	0.003
**235**	4-MHB-M1[illegible]	10	PI3K	0.205673	0.06
**236**	4-MHB-M2[illegible]	1	PI3K	0.166229	0.109
**237**	4-MHB-N4-	10	PI3K	0.301608	0.043
**238**	4-MHB-N1[illegible]	10	PI3K	0.39095	0
**239**	4-MHB-N1[illegible]	100	PI3K	0.246147	0.005
**240**	4-MHB-N21	10	PI3K	0.469573	0
**241**	4-MHB-O4-	100	PI3K	0.313899	0.196
**242**	4-MHB-O1[illegible]	100	PI3K	0.360923	0.016
**243**	4-MHB-O1[illegible]	1000	PI3K	0.216541	0.009
**244**	4-MHB-O21	100	PI3K	0.403588	0
**245**	4-MHB-P4-[illegible]	1000	PI3K	0.327825	0.122
**246**	4-MHB-P14	1000	PI3K	0.362316	0.002
**247**	4-MHB-P15	10000	PI3K	0.341096	0.003
**248**	4-MHB-P21	1000	PI3K	0.428387	0.001
**249**	5-MHB-A6-[illegible]	2500	PI3K	0.365034	0.002
**250**	5-MHB-A7-[illegible]	1000	PI3K	0.245532	0.421
**251**	5-MHB-A16	2500	PI3K	0.416123	0
**252**	5-MHB-A17	10000	PI3K	0.03664	1
**253**	5-MHB-B6-[illegible]	250	PI3K	0.296407	0.265
**254**	5-MHB-B7-[illegible]	100	PI3K	0.137648	0.922
**255**	5-MHB-B17	1000	PI3K	0.190665	0.423
**256**	5-MHB-C6-[illegible]	25	PI3K	0.132853	0.954
**257**	5-MHB-C7-[illegible]	10	PI3K	0.165237	0.662
**258**	5-MHB-C1[illegible]	250	PI3K	0.192449	0.547

**259**	5-MHB-C17	100	PI3K	0.086269	0.987
**260**	5-MHB-D6-	2.5	PI3K	0.21416	0.668
**261**	5-MHB-D7-	1	PI3K	0.131568	0.935
**262**	5-MHB-D16	25	PI3K	0.136789	0.924
**263**	5-MHB-D17	10	PI3K	0.122913	0.666
**264**	5-MHB-E6-I	0.25	PI3K	0.123864	0.921
**265**	5-MHB-E7-/	0.1	PI3K	0.219404	0.822
**266**	5-MHB-E16	2.5	PI3K	0.148147	0.365
**267**	5-MHB-E17	1	PI3K	0.280383	0.044
**268**	5-MHB-F11	10000	PI3K	0.285295	0.003
**269**	5-MHB-F16	0.25	PI3K	0.283576	0.023
**270**	5-MHB-G9-	10000	PI3K	0.439489	0
**271**	5-MHB-G11	1000	PI3K	0.210999	0.058
**272**	5-MHB-H9-	1000	PI3K	0.437378	0.001
**273**	5-MHB-H11	100	PI3K	0.228001	0.049
**274**	5-MHB-I9-S	100	PI3K	0.362115	0
**275**	5-MHB-I11-	10	PI3K	0.184575	0.177
**276**	5-MHB-J9-S	10	PI3K	0.209759	0.044
**277**	5-MHB-J11	1	PI3K	0.137001	0.86
**278**	5-MHB-K9-S	1	PI3K	0.363988	0.001
**279**	5-MHB-L14	0.1	PI3K	0.238693	0.006
**280**	5-MHB-L20	1	PI3K	0.220357	0.026
**281**	5-MHB-L23	0.1	PI3K	0.18786	0.146
**282**	5-MHB-M14	1	PI3K	0.243125	0.09
**283**	5-MHB-M20	10	PI3K	0.15417	0.132
**284**	5-MHB-M23	1	PI3K	0.315032	0.008
**285**	5-MHB-N14	10	PI3K	0.201362	0.23
**286**	5-MHB-N20	100	PI3K	0.290538	0
**287**	5-MHB-N23	10	PI3K	0.48663	0
**288**	5-MHB-O14	100	PI3K	0.375449	0
**289**	5-MHB-O20	1000	PI3K	0.396625	0.003
**290**	5-MHB-O23	100	PI3K	0.471658	0
**291**	5-MHB-P14	1000	PI3K	0.295548	0.124
**292**	5-MHB-P20	10000	PI3K	0.366105	0.019
**293**	5-MHB-P23	1000	PI3K	0.197873	0.948

**294**	6-MHB-A8-	10000	PI3K	0.323844	0.029
**295**	6-MHB-B8-	1000	PI3K	0.116747	0.961
**296**	6-MHB-C8-	100	PI3K	0.147993	0.931
**297**	6-MHB-D8-	10	PI3K	0.156656	0.94
**298**	6-MHB-E8-	1	PI3K	0.158724	0.884
**299**	6-MHB-L6-	1	PI3K	0.224573	0.5
**300**	6-MHB-M6-	10	PI3K	0.171286	0.693
**301**	6-MHB-N6-	100	PI3K	0.324252	0.019
**302**	6-MHB-O6-	1000	PI3K	0.361796	0.025
**303**	6-MHB-P6-	10000	PI3K	0.040697	1
**304**	1-MHB-F11	10000	Topoisome	0.52025	0
**305**	1-MHB-G11	1000	Topoisome	0.642819	0
**306**	1-MHB-G2	1000	Topoisome	0.289604	0.432
**307**	1-MHB-H11	100	Topoisome	0.606328	0
**308**	1-MHB-H2	100	Topoisome	0.560494	0
**309**	1-MHB-I11-	10	Topoisome	0.237847	0.081
**310**	1-MHB-I20-	10	Topoisome	0.163544	0.372
**311**	1-MHB-J11	1	Topoisome	0.30234	0.324
**312**	1-MHB-J20	1	Topoisome	0.171678	0.328
**313**	1-MHB-K11	1	Topoisome	0.542269	0
**314**	1-MHB-K2	0.1	Topoisome	0.569588	0
**315**	1-MHB-L11	10	Topoisome	0.630323	0
**316**	1-MHB-L14	1	Topoisome	0.494534	0
**317**	1-MHB-M1	100	Topoisome	0.459042	0.003
**318**	1-MHB-M1	10	Topoisome	0.475532	0
**319**	1-MHB-N1	100	Topoisome	0.656312	0
**320**	1-MHB-O11	1000	Topoisome	0.375002	0.021
**321**	1-MHB-O1	1000	Topoisome	0.562719	0.001
**322**	1-MHB-P11	10000	Topoisome	0.388142	0.104
**323**	1-MHB-P14	10000	Topoisome	0.359453	0.117
**324**	3-MHB-A11	10000	Topoisome	0.657367	0
**325**	3-MHB-B11	1000	Topoisome	0.637193	0
**326**	3-MHB-C11	100	Topoisome	0.584343	0
**327**	3-MHB-D11	10	Topoisome	0.439711	0
**328**	3-MHB-E11	1	Topoisome	0.52895	0

**329**	3-MHB-G9-	1000	Topoisome	0.320817	0.358
**330**	3-MHB-G1(	10000	Topoisome	0.320251	0.142
**331**	3-MHB-H9-	100	Topoisome	0.595899	0
**332**	3-MHB-H1(	1000	Topoisome	0.498896	0
**333**	3-MHB-I9-[	10	Topoisome	0.411564	0.012
**334**	3-MHB-I10-	100	Topoisome	0.137378	0.874
**335**	3-MHB-J9-[	1	Topoisome	0.378801	0.001
**336**	3-MHB-J10	10	Topoisome	0.333958	0.005
**337**	3-MHB-K7-I	0.1	Topoisome	0.068394	1
**338**	3-MHB-K9-I	0.1	Topoisome	0.394078	0.006
**339**	3-MHB-K1(	1	Topoisome	0.427123	0.001
**340**	3-MHB-L6-I	0.1	Topoisome	0.08697	0.995
**341**	3-MHB-L7-I	1	Topoisome	0.394204	0.009
**342**	3-MHB-L9-\	0.5	Topoisome	0.438213	0
**343**	3-MHB-L10	0.1	Topoisome	0.387848	0.004
**344**	3-MHB-L16	1	Topoisome	0.341272	0.01
**345**	3-MHB-M6-	1	Topoisome	0.421151	0
**346**	3-MHB-M7-	10	Topoisome	0.291498	0.228
**347**	3-MHB-M9-	5	Topoisome	0.307235	0.168
**348**	3-MHB-M1(	1	Topoisome	0.39831	0.001
**349**	3-MHB-M1(	10	Topoisome	0.379295	0.017
**350**	3-MHB-N6-	10	Topoisome	0.379774	0.008
**351**	3-MHB-N9-	50	Topoisome	0.524108	0
**352**	3-MHB-N1(	10	Topoisome	0.536501	0
**353**	3-MHB-N16	100	Topoisome	0.318434	0.004
**354**	3-MHB-O6-	100	Topoisome	0.642883	0
**355**	3-MHB-O7-	100	Topoisome	0.656106	0
**356**	3-MHB-O9-	500	Topoisome	0.613552	0
**357**	3-MHB-O1(	100	Topoisome	0.670924	0
**358**	3-MHB-O16	1000	Topoisome	0.14058	0.93
**359**	3-MHB-P6-I	1000	Topoisome	0.320401	0.162
**360**	3-MHB-P7-I	1000	Topoisome	0.348464	0.293
**361**	3-MHB-P9-\	5000	Topoisome	0.361191	0.305
**362**	3-MHB-P1(	1000	Topoisome	0.524107	0
**363**	3-MHB-P16	10000	Topoisome	0.326654	0.185

**364**	1-MHB-A1(	10000	Mitotic	0.717846	0
**365**	1-MHB-A13	1000	Mitotic	0.620868	0
**366**	1-MHB-A18	1000	Mitotic	0.634438	0
**367**	1-MHB-B1(	1000	Mitotic	0.487199	0.002
**368**	1-MHB-B13	100	Mitotic	0.083097	1
**369**	1-MHB-B18	100	Mitotic	0.658404	0
**370**	1-MHB-C1(	100	Mitotic	0.594743	0
**371**	1-MHB-C13	10	Mitotic	0.345183	0.113
**372**	1-MHB-C18	10	Mitotic	0.393368	0.094
**373**	1-MHB-D1(	10	Mitotic	0.321857	0.25
**374**	1-MHB-D13	1	Mitotic	0.411984	0.013
**375**	1-MHB-D18	1	Mitotic	0.227957	0.671
**376**	1-MHB-E10	1	Mitotic	0.402506	0.006
**377**	1-MHB-E13	0.1	Mitotic	0.310306	0.033
**378**	1-MHB-E18	0.1	Mitotic	0.354583	0.256
**379**	1-MHB-F13	1000	Mitotic	0.483058	0.003
**380**	1-MHB-G13	100	Mitotic	0.723835	0
**381**	1-MHB-G15	1000	Mitotic	0.670376	0
**382**	1-MHB-H13	10	Mitotic	0.424425	0.017
**383**	1-MHB-H15	100	Mitotic	0.690497	0
**384**	1-MHB-I13-	1	Mitotic	0.299697	0.032
**385**	1-MHB-I15-	10	Mitotic	0.481056	0.004
**386**	1-MHB-J13	0.1	Mitotic	0.279885	0.112
**387**	1-MHB-J15	1	Mitotic	0.383494	0.005
**388**	1-MHB-K7-	0.1	Mitotic	0.398892	0.044
**389**	1-MHB-K15	0.1	Mitotic	0.28764	0.054
**390**	1-MHB-L7-	1	Mitotic	0.400834	0.026
**391**	1-MHB-L20	0.1	Mitotic	0.338055	0.021
**392**	1-MHB-M7-	10	Mitotic	0.434229	0.006
**393**	1-MHB-M2(	1	Mitotic	0.327287	0.011
**394**	1-MHB-N2(	10	Mitotic	0.273916	0.122
**395**	1-MHB-O7-	100	Mitotic	0.43469	0.045
**396**	1-MHB-O2(	100	Mitotic	0.226332	0.271
**397**	1-MHB-P7-	1000	Mitotic	0.570419	0
**398**	1-MHB-P2(	1000	Mitotic	0.415308	0.041

**399**	3-MHB-A7-I	1000	Mitotic	0.508816	0.004
**400**	3-MHB-B7-I	100	Mitotic	0.585854	0.001
**401**	3-MHB-C7-I	10	Mitotic	0.444303	0.033
**402**	3-MHB-D7-	1	Mitotic	0.304289	0.407
**403**	3-MHB-E7-I	0.1	Mitotic	0.23803	0.552
**404**	6-MHB-L19	1	Mitotic	0.244034	0.14
**405**	6-MHB-M1!	10	Mitotic	0.19996	0.603
**406**	6-MHB-N1!	100	Mitotic	0.259913	0.313
**407**	6-MHB-O1!	1000	Mitotic	0.44888	0.024
**408**	6-MHB-P1!	10000	Mitotic	0.650282	0
**409**	2-MHB-A12	250	MEK1/2	0.717733	0
**410**	2-MHB-B12	25	MEK1/2	0.854678	0
**411**	2-MHB-D1:	2.5	MEK1/2	0.846058	0
**412**	2-MHB-E12	0.25	MEK1/2	0.694167	0
**413**	2-MHB-F12	2.5000000	MEK1/2	0.628854	0
**414**	2-MHB-F14	1000	MEK1/2	0.635239	0
**415**	2-MHB-G14	100	MEK1/2	0.683297	0
**416**	2-MHB-H14	10	MEK1/2	0.835983	0
**417**	2-MHB-I14-	1	MEK1/2	0.749892	0
**418**	2-MHB-K14	0.1	MEK1/2	0.635611	0
**419**	2-MHB-L20	1	MEK1/2	0.283367	0.234
**420**	2-MHB-M2(	10	MEK1/2	0.889044	0
**421**	2-MHB-N2(	100	MEK1/2	0.708776	0
**422**	2-MHB-O2(	1000	MEK1/2	0.724706	0
**423**	2-MHB-P2(	10000	MEK1/2	0.674328	0
**424**	4-MHB-A1(	1000	MEK1/2	0.743983	0
**425**	4-MHB-A13	1000	MEK1/2	0.708438	0
**426**	4-MHB-B1(	100	MEK1/2	0.857706	0
**427**	4-MHB-B13	100	MEK1/2	0.756805	0
**428**	4-MHB-C1(	10	MEK1/2	0.69007	0
**429**	4-MHB-C13	10	MEK1/2	0.871522	0
**430**	4-MHB-D1(	1	MEK1/2	0.562979	0.001
**431**	4-MHB-D1:	1	MEK1/2	0.819445	0
**432**	4-MHB-E10	0.1	MEK1/2	0.727925	0
**433**	4-MHB-E13	0.1	MEK1/2	0.363919	0.063

**434**	4-MHB-L19	0.25	MEK1/2	0.350585	0.041
**435**	4-MHB-M1	2.5	MEK1/2	0.178813	0.539
**436**	4-MHB-N1	25	MEK1/2	0.692068	0
**437**	4-MHB-O1	250	MEK1/2	0.706507	0
**438**	4-MHB-P1	2500	MEK1/2	0.676444	0
**439**	1-MHB-L2-	1	PARP	0.814737	0
**440**	1-MHB-L6-	1	PARP	0.483685	0.042
**441**	1-MHB-M2	10	PARP	0.219186	0.504
**442**	1-MHB-M6	10	PARP	0.274779	0.379
**443**	1-MHB-N2-	100	PARP	0.729089	0
**444**	1-MHB-N6-	100	PARP	0.355925	0.086
**445**	1-MHB-O2-	1000	PARP	0.655816	0
**446**	1-MHB-O6-	1000	PARP	0.737756	0
**447**	1-MHB-P2-	10000	PARP	0.650194	0.001
**448**	1-MHB-P6-	10000	PARP	0.698262	0
**449**	7-MHB-A3-	1000	PARP	0.653245	0.001
**450**	7-MHB-B2-	10000	PARP	0.642333	0
**451**	7-MHB-B3-	100	PARP	0.563925	0.007
**452**	7-MHB-C2-	1000	PARP	0.491526	0.01
**453**	7-MHB-C3-	10	PARP	0.514521	0.007
**454**	7-MHB-D2-	100	PARP	0.535557	0.002
**455**	7-MHB-D3-	1	PARP	0.373109	0.227
**456**	7-MHB-E2-	10	PARP	0.562888	0.001
**457**	7-MHB-E3-	0.1	PARP	0.423259	0.095
**458**	7-MHB-F2-	1	PARP	0.629238	0
**459**	7-MHB-G2-	10000	PARP	0.69709	0
**460**	7-MHB-H2-	1000	PARP	0.782578	0
**461**	7-MHB-I2-	100	PARP	0.489649	0.008
**462**	7-MHB-J2-	10	PARP	0.475613	0.028
**463**	7-MHB-K2-	1	PARP	0.481652	0.04
**464**	3-MHB-A1	1000	CDK	0.418669	0.001
**465**	3-MHB-B1	100	CDK	0.473373	0.003
**466**	3-MHB-B2	2500	CDK	0.406371	0.001
**467**	3-MHB-C1	10	CDK	0.379497	0.053
**468**	3-MHB-C2	250	CDK	0.266847	0.294

**469**	3-MHB-D1	1	CDK	0.321655	0.031
**470**	3-MHB-D2	25	CDK	0.308104	0.089
**471**	3-MHB-E19	0.1	CDK	0.161383	0.912
**472**	3-MHB-E23	2.5	CDK	0.385487	0.016
**473**	3-MHB-F23	0.25	CDK	0.330317	0.05
**474**	3-MHB-K17	1	CDK	0.155501	0.774
**475**	3-MHB-L19	1	CDK	0.066459	1
**476**	3-MHB-M1	10	CDK	0.329486	0.061
**477**	3-MHB-M1	10	CDK	0.212035	0.176
**478**	3-MHB-N1	100	CDK	0.276481	0.187
**479**	3-MHB-N1	100	CDK	0.186989	0.545
**480**	3-MHB-O1	1000	CDK	0.429041	0.003
**481**	3-MHB-O1	1000	CDK	0.278592	0.048
**482**	3-MHB-P17	10000	CDK	0.282713	0.451
**483**	3-MHB-P1	10000	CDK	0.325593	0.113
**484**	4-MHB-A4-	10000	CDK	0.477238	0.009
**485**	4-MHB-A8-	10000	CDK	0.433905	0.002
**486**	4-MHB-B4-	1000	CDK	0.502515	0
**487**	4-MHB-B8-	1000	CDK	0.237255	0.744
**488**	4-MHB-C4-	100	CDK	0.260215	0.256
**489**	4-MHB-C8-	100	CDK	0.12987	0.952
**490**	4-MHB-D4-	10	CDK	0.223379	0.428
**491**	4-MHB-D8-	10	CDK	0.139553	0.904
**492**	4-MHB-E4-	1	CDK	0.26201	0.123
**493**	4-MHB-E8-	1	CDK	0.208916	0.742
**494**	4-MHB-F4-	10000	CDK	0.328699	0.364
**495**	4-MHB-F22	10000	CDK	0.497074	0.003
**496**	4-MHB-G4-	1000	CDK	0.374008	0.089
**497**	4-MHB-G2	1000	CDK	0.492258	0.005
**498**	4-MHB-H4-	100	CDK	0.224388	0.203
**499**	4-MHB-H2	100	CDK	0.350469	0.13
**500**	4-MHB-I4-S	10	CDK	0.180895	0.715
**501**	4-MHB-I22	10	CDK	0.307112	0.009
**502**	4-MHB-J4-S	1	CDK	0.182364	0.801
**503**	4-MHB-J22	1	CDK	0.257735	0.062

**504**	5-MHB-A19	10000	CDK	0.360117	0.272
**505**	5-MHB-B19	1000	CDK	0.311297	0.381
**506**	5-MHB-C19	100	CDK	0.429994	0.005
**507**	5-MHB-D19	10	CDK	0.244592	0.1
**508**	5-MHB-E19	1	CDK	0.303728	0.249
**509**	5-MHB-K17	1	CDK	0.110794	0.945
**510**	5-MHB-M1	10	CDK	0.204035	0.618
**511**	5-MHB-N17	100	CDK	0.294909	0.026
**512**	5-MHB-O17	1000	CDK	0.317558	0.438
**513**	5-MHB-P17	10000	CDK	0.502427	0.002
**514**	6-MHB-A17	1000	CDK	0.392556	0.085
**515**	6-MHB-B17	100	CDK	0.375483	0.02
**516**	6-MHB-C17	10	CDK	0.25277	0.444
**517**	6-MHB-D17	1	CDK	0.220454	0.571
**518**	6-MHB-E17	0.1	CDK	0.240894	0.714
**519**	6-MHB-L15	1	CDK	0.100484	0.937
**520**	6-MHB-M1	10	CDK	0.14279	0.822
**521**	6-MHB-N15	100	CDK	0.329478	0.3
**522**	6-MHB-O15	1000	CDK	0.478199	0.002
**523**	6-MHB-P15	10000	CDK	0.459628	0.004
**524**	7-MHB-A21	10000	BET	0.626828	0
**525**	7-MHB-A22	30000	BET	0.57289	0
**526**	7-MHB-B21	1000	BET	0.406256	0.044
**527**	7-MHB-B22	3000	BET	0.64252	0
**528**	7-MHB-C21	100	BET	0.146503	0.734
**529**	7-MHB-C22	300	BET	0.139021	0.732
**530**	7-MHB-D21	10	BET	0.31625	0.148
**531**	7-MHB-D22	30	BET	0.397021	0.066
**532**	7-MHB-E21	1	BET	0.522302	0
**533**	7-MHB-E22	3	BET	0.552139	0
**534**	7-MHB-G10	10000	BET	0.604068	0
**535**	7-MHB-G15	10000	BET	0.591529	0
**536**	7-MHB-H10	1000	BET	0.610242	0
**537**	7-MHB-H15	1000	BET	0.631655	0
**538**	7-MHB-I10	100	BET	0.486427	0.001

**539**	7-MHB-I15-	100	BET	0.307646	0.194
**540**	7-MHB-J10	10	BET	0.089979	0.92
**541**	7-MHB-J15	10	BET	0.090898	0.902
**542**	7-MHB-K10	1	BET	0.200143	0.625
**543**	7-MHB-K13	1	BET	0.239684	0.492
**544**	7-MHB-K15	1	BET	0.197628	0.604
**545**	7-MHB-L12	1	BET	0.342003	0.103
**546**	7-MHB-L13	10	BET	0.597861	0
**547**	7-MHB-L20	1	BET	0.376479	0.083
**548**	7-MHB-L23	0.03	BET	0.304268	0.141
**549**	7-MHB-M1:	10	BET	0.45571	0.004
**550**	7-MHB-M1:	100	BET	0.617421	0
**551**	7-MHB-M2(	10	BET	0.281568	0.277
**552**	7-MHB-M2:	0.3	BET	0.186952	0.663
**553**	7-MHB-N12	100	BET	0.325582	0.226
**554**	7-MHB-N13	1000	BET	0.487814	0.052
**555**	7-MHB-N2(	100	BET	0.604694	0
**556**	7-MHB-N23	3	BET	0.255926	0.374
**557**	7-MHB-O12	1000	BET	0.62748	0
**558**	7-MHB-O2(	1000	BET	0.615356	0
**559**	7-MHB-O23	30	BET	0.453056	0.002
**560**	7-MHB-P12	10000	BET	0.617879	0
**561**	7-MHB-P13	10000	BET	0.39745	0.21
**562**	7-MHB-P20	10000	BET	0.609263	0
**563**	7-MHB-P23	300	BET	0.620858	0
**564**	8-MHB-K22	1	BET	0.161789	0.813
**565**	8-MHB-L22	10	BET	0.53921	0
**566**	8-MHB-M2:	100	BET	0.176831	0.782
**567**	8-MHB-N22	1000	BET	0.592483	0
**568**	8-MHB-O22	10000	BET	0.55597	0
**569**	1-MHB-A3-'	10000	HDAC	0.320417	0.403
**570**	1-MHB-B3-'	1000	HDAC	0.388297	0.005
**571**	1-MHB-C3-'	100	HDAC	0.225225	0.051
**572**	1-MHB-D3-	10	HDAC	0.094806	0.988
**573**	1-MHB-E3-\	1	HDAC	0.118619	0.965

**574**	1-MHB-L12	0.1	HDAC	0.301681	0.005
**575**	1-MHB-M1:	1	HDAC	0.48501	0
**576**	1-MHB-N12	10	HDAC	0.307112	0.019
**577**	1-MHB-O12	100	HDAC	0.240641	0.216
**578**	1-MHB-P12	1000	HDAC	0.374148	0.025
**579**	3-MHB-A4-I	1000	HDAC	0.272955	0.587
**580**	3-MHB-B4-I	100	HDAC	0.320487	0.152
**581**	3-MHB-C4-I	10	HDAC	0.258735	0.185
**582**	3-MHB-D4-	1	HDAC	0.080594	0.984
**583**	3-MHB-E4-I	0.1	HDAC	0.311457	0.037
**584**	3-MHB-F7-(	1000	HDAC	0.218733	0.841
**585**	3-MHB-G7-	100	HDAC	0.325673	0.175
**586**	3-MHB-G12	1000000	HDAC	0.112731	0.528
**587**	3-MHB-H7-	10	HDAC	0.491269	0
**588**	3-MHB-H12	100000	HDAC	0.398003	0
**589**	3-MHB-I7-C	1	HDAC	0.301081	0.001
**590**	3-MHB-I12-	10000	HDAC	0.267505	0.001
**591**	3-MHB-J7-C	0.1	HDAC	0.171509	0.784
**592**	3-MHB-J12	1000	HDAC	0.351904	0
**593**	3-MHB-K3-I	1	HDAC	0.143994	0.907
**594**	3-MHB-K12	100	HDAC	0.369481	0
**595**	3-MHB-L3-I	10	HDAC	0.168514	0.767
**596**	3-MHB-M3-	100	HDAC	0.244855	0.375
**597**	3-MHB-N3-	1000	HDAC	0.321873	0.232
**598**	3-MHB-O3-	10000	HDAC	0.246022	0.157
**599**	7-MHB-A5-I	10000	HDAC	0.381015	0.042
**600**	7-MHB-A7-	10000	HDAC	0.429712	0.002
**601**	7-MHB-A9-	1000	HDAC	0.366595	0.11
**602**	7-MHB-A12	10000	HDAC	0.334748	0.235
**603**	7-MHB-B5-I	1000	HDAC	0.404533	0.008
**604**	7-MHB-B12	1000	HDAC	0.537128	0
**605**	7-MHB-C5-I	100	HDAC	0.029645	0.999
**606**	7-MHB-C7-	100	HDAC	0.384696	0.015
**607**	7-MHB-C9-	100	HDAC	0.47916	0
**608**	7-MHB-D7-	10	HDAC	0.367344	0.059

**609**	7-MHB-D9-	10	HDAC	0.560495	0
**610**	7-MHB-D12	100	HDAC	0.132691	0.625
**611**	7-MHB-E5-I	10	HDAC	0.289437	0.08
**612**	7-MHB-E7-(	1	HDAC	0.545666	0
**613**	7-MHB-E9-(	1	HDAC	0.437054	0
**614**	7-MHB-E12	10	HDAC	0.232586	0.014
**615**	7-MHB-F5-I	1	HDAC	0.298415	0.113
**616**	7-MHB-F7-I	10000	HDAC	0.342465	0.011
**617**	7-MHB-F9-(	0.1	HDAC	0.489687	0
**618**	7-MHB-F12	1	HDAC	0.402915	0
**619**	7-MHB-F19	10000	HDAC	0.455887	0
**620**	7-MHB-G7-	1000	HDAC	0.499224	0
**621**	7-MHB-G19	1000	HDAC	0.183831	0.064
**622**	7-MHB-H7-	100	HDAC	0.508324	0
**623**	7-MHB-I7-F	10	HDAC	0.403373	0
**624**	7-MHB-I19-	100	HDAC	0.2445	0.021
**625**	7-MHB-J7-F	1	HDAC	0.419991	0
**626**	7-MHB-J19	10	HDAC	0.420668	0
**627**	7-MHB-K4-I	1	HDAC	0.108019	0.975
**628**	7-MHB-K11	1	HDAC	0.343987	0.006
**629**	7-MHB-K18	1	HDAC	0.395622	0
**630**	7-MHB-K19	1	HDAC	0.137681	0.309
**631**	7-MHB-L2-7	0.1	HDAC	0.083942	0.976
**632**	7-MHB-L4-I	10	HDAC	0.100219	0.926
**633**	7-MHB-L5-I	1	HDAC	0.140257	0.788
**634**	7-MHB-L8-/	1	HDAC	0.426213	0
**635**	7-MHB-L10	1	HDAC	0.501392	0
**636**	7-MHB-L11	10	HDAC	0.367995	0
**637**	7-MHB-L14	1	HDAC	0.444354	0
**638**	7-MHB-L16	1	HDAC	0.472603	0
**639**	7-MHB-L18	10	HDAC	0.145856	0.221
**640**	7-MHB-M2-	1	HDAC	0.211866	0.584
**641**	7-MHB-M5-	10	HDAC	0.246273	0.346
**642**	7-MHB-M8-	10	HDAC	0.545898	0
**643**	7-MHB-M1(	10	HDAC	0.490877	0

**644**	7-MHB-M1	100	HDAC	0.509179	0
**645**	7-MHB-M1	10	HDAC	0.429491	0
**646**	7-MHB-M1	10	HDAC	0.448781	0
**647**	7-MHB-M1	100	HDAC	0.461284	0
**648**	7-MHB-N2-	10	HDAC	0.10924	0.975
**649**	7-MHB-N4-	100	HDAC	0.477158	0
**650**	7-MHB-N5-	100	HDAC	0.496806	0
**651**	7-MHB-N8-	100	HDAC	0.486585	0
**652**	7-MHB-N1	100	HDAC	0.488673	0
**653**	7-MHB-N1	100	HDAC	0.484141	0
**654**	7-MHB-N1	100	HDAC	0.381118	0
**655**	7-MHB-N1	1000	HDAC	0.208504	0.47
**656**	7-MHB-O2-	100	HDAC	0.100018	0.969
**657**	7-MHB-O4-	1000	HDAC	0.503323	0
**658**	7-MHB-O5-	1000	HDAC	0.33606	0.248
**659**	7-MHB-O8-	1000	HDAC	0.337011	0.318
**660**	7-MHB-O1	1000	HDAC	0.560643	0
**661**	7-MHB-O1	1000	HDAC	0.345711	0.16
**662**	7-MHB-O1	1000	HDAC	0.4577	0
**663**	7-MHB-O1	1000	HDAC	0.34706	0
**664**	7-MHB-P2-	1000	HDAC	0.362873	0
**665**	7-MHB-P4-	10000	HDAC	0.31724	0.322
**666**	7-MHB-P5-	10000	HDAC	0.395082	0.05
**667**	7-MHB-P8-	10000	HDAC	0.39213	0.026
**668**	7-MHB-P1	10000	HDAC	0.34189	0.177
**669**	7-MHB-P11	10000	HDAC	0.385118	0.027
**670**	7-MHB-P14	10000	HDAC	0.398945	0.003
**671**	7-MHB-P1	10000	HDAC	0.475635	0
**672**	7-MHB-P18	10000	HDAC	0.448121	0
**0**	2-O3B-A16-	10000	EGFR	0.558174	0
**1**	2-O3B-A19-	10000	EGFR	0.539178	0
**2**	2-O3B-B19-	1000	EGFR	0.575406	0
**3**	2-O3B-C16-	1000	EGFR	0.603696	0
**4**	2-O3B-C19-	100	EGFR	0.617767	0
**5**	2-O3B-D16	100	EGFR	0.644597	0

**6**	2-O3B-D19	10	EGFR	0.040134	0.999
**7**	2-O3B-E16-	10	EGFR	0.563494	0
**8**	2-O3B-E19-	1	EGFR	0.079018	0.98
**9**	2-O3B-F16-	1	EGFR	0.32218	0.158
**10**	2-O3B-K11-	0.1	EGFR	0.351111	0.001
**11**	2-O3B-L11-	1	EGFR	0.486531	0
**12**	2-O3B-L16-	0.25	EGFR	0.189124	0.839
**13**	2-O3B-L19-	0.1	EGFR	0.531241	0
**14**	2-O3B-M11	10	EGFR	0.665806	0
**15**	2-O3B-M16	2.5	EGFR	0.527742	0
**16**	2-O3B-M19	1	EGFR	0.432778	0.001
**17**	2-O3B-N16	25	EGFR	0.553882	0
**18**	2-O3B-N19	10	EGFR	0.512499	0
**19**	2-O3B-O11	100	EGFR	0.609231	0
**20**	2-O3B-O16	250	EGFR	0.608081	0
**21**	2-O3B-O19	100	EGFR	0.554445	0
**22**	2-O3B-P11-	1000	EGFR	0.546275	0
**23**	2-O3B-P16-	2500	EGFR	0.546912	0
**24**	2-O3B-P19-	1000	EGFR	0.649167	0
**25**	3-O3B-F21-	10000	EGFR	0.534957	0
**26**	3-O3B-G20	1000	EGFR	0.567336	0
**27**	3-O3B-G21	1000	EGFR	0.572121	0
**28**	3-O3B-H20	100	EGFR	0.603155	0
**29**	3-O3B-H21	100	EGFR	0.509913	0
**30**	3-O3B-I20-	10	EGFR	0.549114	0
**31**	3-O3B-I21-	10	EGFR	0.46069	0
**32**	3-O3B-J20-	1	EGFR	0.478956	0
**33**	3-O3B-J21-	1	EGFR	0.439699	0.001
**34**	3-O3B-K4-C	1	EGFR	0.049726	0.972
**35**	3-O3B-K18-	0.1	EGFR	0.178749	0.684
**36**	3-O3B-K20-	0.1	EGFR	0.08038	0.859
**37**	3-O3B-L4-C	10	EGFR	0.59935	0
**38**	3-O3B-L18-	1	EGFR	0.048055	0.984
**39**	3-O3B-M18	10	EGFR	0.711693	0
**40**	3-O3B-N4-C	100	EGFR	0.078061	0.979

**41**	3-O3B-N18	100	EGFR	0.639757	0
**42**	3-O3B-O4-	1000	EGFR	0.624931	0
**43**	3-O3B-P4-	10000	EGFR	0.374727	0.395
**44**	3-O3B-P18	1000	EGFR	0.544569	0
**45**	4-O3B-F13	1000	EGFR	0.527185	0
**46**	4-O3B-G13	100	EGFR	0.626235	0
**47**	4-O3B-G16	10000	EGFR	0.512878	0
**48**	4-O3B-H13	10	EGFR	0.58835	0
**49**	4-O3B-H16	1000	EGFR	0.613091	0
**50**	4-O3B-I13-	1	EGFR	0.516488	0
**51**	4-O3B-I16-	100	EGFR	0.47769	0
**52**	4-O3B-J13-	0.1	EGFR	0.39825	0.002
**53**	4-O3B-J16-	10	EGFR	0.087177	0.991
**54**	4-O3B-K7-	1	EGFR	0.453307	0
**55**	4-O3B-K13	0.1	EGFR	0.210541	0.202
**56**	4-O3B-K16	1	EGFR	0.479243	0
**57**	4-O3B-L7-	10	EGFR	0.435162	0.001
**58**	4-O3B-L13-	1	EGFR	0.533864	0
**59**	4-O3B-M7-	100	EGFR	0.488155	0
**60**	4-O3B-M13	10	EGFR	0.536816	0
**61**	4-O3B-N13	100	EGFR	0.562651	0
**62**	4-O3B-O7-	1000	EGFR	0.555714	0
**63**	4-O3B-P7-	10000	EGFR	0.56819	0
**64**	4-O3B-P13	1000	EGFR	0.512529	0
**65**	5-O3B-F4-P	1000	EGFR	0.506118	0
**66**	5-O3B-F7-A	1000	EGFR	0.541117	0
**67**	5-O3B-G4-	100	EGFR	0.558365	0
**68**	5-O3B-G7-	100	EGFR	0.470669	0
**69**	5-O3B-H4-	10	EGFR	0.57601	0
**70**	5-O3B-H7-	10	EGFR	0.381034	0.005
**71**	5-O3B-I4-P	1	EGFR	0.519057	0
**72**	5-O3B-I7-A	1	EGFR	0.36955	0.004
**73**	5-O3B-J4-P	0.1	EGFR	0.46682	0
**74**	5-O3B-J7-A	0.1	EGFR	0.262943	0.425
**75**	5-O3B-K7-	0.1	EGFR	0.193249	0.9

**76**	5-O3B-L7-C	1	EGFR	0.353427	0.009
**77**	5-O3B-M7-	10	EGFR	0.37972	0.002
**78**	5-O3B-O7-(	100	EGFR	0.424007	0
**79**	5-O3B-P7-C	1000	EGFR	0.564216	0
**80**	2-O3B-A15-	2500	VEGFR	0.263221	0.318
**81**	2-O3B-A17-	10000	VEGFR	0.193658	0.956
**82**	2-O3B-A20-	10000	VEGFR	0.192433	0.939
**83**	2-O3B-B15-	250	VEGFR	0.417233	0
**84**	2-O3B-B17-	1000	VEGFR	0.21866	0.874
**85**	2-O3B-B20-	1000	VEGFR	0.213098	0.4
**86**	2-O3B-C15-	25	VEGFR	0.336818	0
**87**	2-O3B-C17-	100	VEGFR	0.313425	0.01
**88**	2-O3B-D15	2.5	VEGFR	0.482277	0
**89**	2-O3B-D17	10	VEGFR	0.299506	0.004
**90**	2-O3B-D20	100	VEGFR	0.205461	0.142
**91**	2-O3B-E17-	1	VEGFR	0.316464	0.082
**92**	2-O3B-E20-	10	VEGFR	0.425995	0
**93**	2-O3B-F13-	10000	VEGFR	0.152649	0.987
**94**	2-O3B-F15-	0.25	VEGFR	0.356894	0
**95**	2-O3B-F19-	10000	VEGFR	0.16639	0.995
**96**	2-O3B-F20-	1	VEGFR	0.312927	0
**97**	2-O3B-F21-	10000	VEGFR	0.274111	0.31
**98**	2-O3B-G10	10000	VEGFR	0.30058	0.239
**99**	2-O3B-G13	1000	VEGFR	0.084551	0.999
**100**	2-O3B-G19	1000	VEGFR	0.384724	0.004
**101**	2-O3B-G21	1000	VEGFR	0.283138	0.001
**102**	2-O3B-H10	1000	VEGFR	0.434741	0
**103**	2-O3B-H13	100	VEGFR	0.359165	0.01
**104**	2-O3B-H21	100	VEGFR	0.319415	0
**105**	2-O3B-I10-,	100	VEGFR	0.302046	0.185
**106**	2-O3B-I13-,	10	VEGFR	0.467735	0
**107**	2-O3B-I19-I	100	VEGFR	0.420953	0
**108**	2-O3B-I21-'	10	VEGFR	0.330143	0.031
**109**	2-O3B-J10-	10	VEGFR	0.366728	0
**110**	2-O3B-J13-	1	VEGFR	0.299518	0.001

**111**	2-O3B-J19-	10	VEGFR	0.262104	0.216
**112**	2-O3B-J21-	1	VEGFR	0.277818	0.032
**113**	2-O3B-K10-	1	VEGFR	0.307242	0
**114**	2-O3B-K13-	0.1	VEGFR	0.410316	0
**115**	2-O3B-K17-	1	VEGFR	0.106847	0.961
**116**	2-O3B-K19-	1	VEGFR	0.203864	0.496
**117**	2-O3B-L12-	0.1	VEGFR	0.313698	0.008
**118**	2-O3B-L13-	1	VEGFR	0.259525	0.222
**119**	2-O3B-L21-	0.1	VEGFR	0.257458	0.343
**120**	2-O3B-M12	1	VEGFR	0.282663	0.181
**121**	2-O3B-M13	10	VEGFR	0.416577	0
**122**	2-O3B-M17	10	VEGFR	0.315429	0.048
**123**	2-O3B-M21	1	VEGFR	0.205837	0.546
**124**	2-O3B-N12	10	VEGFR	0.264055	0.006
**125**	2-O3B-N13	100	VEGFR	0.220236	0.318
**126**	2-O3B-N17	100	VEGFR	0.095783	0.976
**127**	2-O3B-N21	10	VEGFR	0.298199	0.006
**128**	2-O3B-O12	100	VEGFR	0.291734	0
**129**	2-O3B-O17	1000	VEGFR	0.208235	0.845
**130**	2-O3B-O21	100	VEGFR	0.194844	0.801
**131**	2-O3B-P12-	1000	VEGFR	0.344772	0
**132**	2-O3B-P13-	1000	VEGFR	0.147206	0.981
**133**	2-O3B-P17-	10000	VEGFR	0.211783	0.475
**134**	2-O3B-P21-	1000	VEGFR	0.148928	0.871
**135**	3-O3B-A3-(	1000	VEGFR	0.124681	0.976
**136**	3-O3B-A6-F	1000	VEGFR	0.114471	0.999
**137**	3-O3B-A18-	1000	VEGFR	0.326146	0.002
**138**	3-O3B-B3-(	100	VEGFR	0.367976	0.007
**139**	3-O3B-B6-F	100	VEGFR	0.314555	0.011
**140**	3-O3B-B18-	100	VEGFR	0.254005	0.014
**141**	3-O3B-C3-(	10	VEGFR	0.143455	0.847
**142**	3-O3B-C6-F	10	VEGFR	0.374775	0.001
**143**	3-O3B-C18-	10	VEGFR	0.264463	0
**144**	3-O3B-D3-(	1	VEGFR	0.327997	0.005
**145**	3-O3B-D6-F	1	VEGFR	0.247674	0.024

**146**	3-O3B-D18	1	VEGFR	0.223332	0.02
**147**	3-O3B-E3-C	0.1	VEGFR	0.278356	0.006
**148**	3-O3B-E6-F	0.1	VEGFR	0.34299	0
**149**	3-O3B-E18-	0.1	VEGFR	0.280374	0.117
**150**	3-O3B-F18-	1000	VEGFR	0.209216	0.815
**151**	3-O3B-G18	100	VEGFR	0.204128	0.084
**152**	3-O3B-H18	10	VEGFR	0.208789	0.787
**153**	3-O3B-I18-	1	VEGFR	0.097961	0.982
**154**	3-O3B-J18-	0.1	VEGFR	0.109444	0.969
**155**	4-O3B-A12-	10000	VEGFR	0.113156	1
**156**	4-O3B-A15-	2500	VEGFR	0.296495	0.005
**157**	4-O3B-A20-	10000	VEGFR	0.203057	0.661
**158**	4-O3B-B12-	1000	VEGFR	0.114216	0.998
**159**	4-O3B-B15-	250	VEGFR	0.282577	0.001
**160**	4-O3B-B20-	1000	VEGFR	0.242785	0.025
**161**	4-O3B-C15-	25	VEGFR	0.128219	0.968
**162**	4-O3B-D12	100	VEGFR	0.105947	0.964
**163**	4-O3B-D15	2.5	VEGFR	0.089775	0.995
**164**	4-O3B-D20	100	VEGFR	0.213653	0.483
**165**	4-O3B-E12-	10	VEGFR	0.224351	0.495
**166**	4-O3B-E20-	10	VEGFR	0.270924	0.022
**167**	4-O3B-F12-	1	VEGFR	0.267882	0.122
**168**	4-O3B-F15-	0.25	VEGFR	0.045144	0.997
**169**	4-O3B-F20-	1	VEGFR	0.225636	0.032
**170**	4-O3B-L16-	1	VEGFR	0.375453	0
**171**	4-O3B-M16	10	VEGFR	0.0776	0.994
**172**	4-O3B-N16	100	VEGFR	0.076358	0.981
**173**	4-O3B-O16	1000	VEGFR	0.387864	0.001
**174**	4-O3B-P16-	10000	VEGFR	0.409992	0.001
**175**	2-O3B-L10-	1	PI3K	0.024959	0.997
**176**	2-O3B-M10	10	PI3K	0.220505	0.338
**177**	2-O3B-N10	100	PI3K	0.230843	0.316
**178**	2-O3B-O10	1000	PI3K	0.414279	0
**179**	2-O3B-P10-	10000	PI3K	0.38745	0.006
**180**	3-O3B-A16-	2500	PI3K	0.043392	0.988

**181**	3-O3B-C16-	250	PI3K	0.229042	0.088
**182**	3-O3B-D16	25	PI3K	0.057051	0.937
**183**	3-O3B-E16-	2.5	PI3K	0.053966	0.878
**184**	3-O3B-F16-	0.25	PI3K	0.264903	0.129
**185**	3-O3B-F17-	100000	PI3K	0.346278	0.13
**186**	3-O3B-F19-	500	PI3K	0.432824	0
**187**	3-O3B-G17	10000	PI3K	0.039471	0.978
**188**	3-O3B-G19	50	PI3K	0.442967	0
**189**	3-O3B-H17	1000	PI3K	0.446354	0
**190**	3-O3B-I17-I	100	PI3K	0.446892	0
**191**	3-O3B-I19-I	5	PI3K	0.449102	0
**192**	3-O3B-J17-	10	PI3K	0.399575	0
**193**	3-O3B-J19-	0.5	PI3K	0.450347	0
**194**	3-O3B-K19-	0.05	PI3K	0.048846	0.912
**195**	3-O3B-L8-P	1	PI3K	0.474229	0
**196**	3-O3B-L21-	0.1	PI3K	0.03793	0.964
**197**	3-O3B-M8-I	10	PI3K	0.337703	0.001
**198**	3-O3B-M21	1	PI3K	0.072707	0.895
**199**	3-O3B-N8-F	100	PI3K	0.516596	0
**200**	3-O3B-N21	10	PI3K	0.055415	0.972
**201**	3-O3B-O8-F	1000	PI3K	0.388034	0.007
**202**	3-O3B-O21	100	PI3K	0.211231	0.402
**203**	3-O3B-P8-F	10000	PI3K	0.417102	0.005
**204**	3-O3B-P21-	1000	PI3K	0.414931	0
**205**	4-O3B-A19-	2500	PI3K	0.379904	0
**206**	4-O3B-B19-	250	PI3K	0.250526	0.483
**207**	4-O3B-C19-	25	PI3K	0.384193	0
**208**	4-O3B-D19	2.5	PI3K	0.132459	0.524
**209**	4-O3B-E19-	0.25	PI3K	0.101283	0.92
**210**	4-O3B-F14-	1000	PI3K	0.402502	0.014
**211**	4-O3B-G2-T	2500	PI3K	0.411894	0.001
**212**	4-O3B-G5-S	10000	PI3K	0.486502	0
**213**	4-O3B-G14	100	PI3K	0.391367	0.01
**214**	4-O3B-G20	10000	PI3K	0.403253	0.014
**215**	4-O3B-H2-T	250	PI3K	0.167814	0.914

**216**	4-O3B-H5-S	1000	PI3K	0.391457	0
**217**	4-O3B-H14	10	PI3K	0.399368	0
**218**	4-O3B-H20	1000	PI3K	0.409164	0
**219**	4-O3B-I2-T(	25	PI3K	0.084672	0.976
**220**	4-O3B-I5-S(	100	PI3K	0.399582	0
**221**	4-O3B-I14-I	1	PI3K	0.408032	0
**222**	4-O3B-I20-I	100	PI3K	0.142602	0.898
**223**	4-O3B-J2-T(	2.5	PI3K	0.040724	0.978
**224**	4-O3B-J5-S(	10	PI3K	0.464943	0
**225**	4-O3B-J20-	10	PI3K	0.138935	0.84
**226**	4-O3B-K2-T	0.25	PI3K	0.049333	0.981
**227**	4-O3B-K4-D	0.1	PI3K	0.434966	0
**228**	4-O3B-K5-S	1	PI3K	0.428077	0
**229**	4-O3B-K14-	0.1	PI3K	0.05227	0.982
**230**	4-O3B-K20-	1	PI3K	0.211334	0.603
**231**	4-O3B-L4-D	1	PI3K	0.418236	0
**232**	4-O3B-L14-	0.1	PI3K	0.303289	0.005
**233**	4-O3B-L15-	1	PI3K	0.323271	0.001
**234**	4-O3B-L21-	0.1	PI3K	0.207397	0.547
**235**	4-O3B-M14	1	PI3K	0.411954	0
**236**	4-O3B-M15	10	PI3K	0.450027	0
**237**	4-O3B-M21	1	PI3K	0.431475	0
**238**	4-O3B-N4-D	10	PI3K	0.403463	0
**239**	4-O3B-N14	10	PI3K	0.364796	0.001
**240**	4-O3B-N15	100	PI3K	0.257386	0.07
**241**	4-O3B-N21	10	PI3K	0.441137	0
**242**	4-O3B-O4-D	100	PI3K	0.383773	0.005
**243**	4-O3B-O14	100	PI3K	0.399546	0.002
**244**	4-O3B-O15	1000	PI3K	0.407248	0
**245**	4-O3B-O21	100	PI3K	0.386027	0
**246**	4-O3B-P4-D	1000	PI3K	0.376824	0.004
**247**	4-O3B-P14-	1000	PI3K	0.4037	0.014
**248**	4-O3B-P15-	10000	PI3K	0.407665	0
**249**	4-O3B-P21-	1000	PI3K	0.400434	0.016
**250**	5-O3B-A6-L	2500	PI3K	0.409078	0.018

**251**	5-O3B-A7-A	1000	PI3K	0.441887	0
**252**	5-O3B-A16-	2500	PI3K	0.424295	0
**253**	5-O3B-A17-	10000	PI3K	0.406224	0
**254**	5-O3B-B6-L	250	PI3K	0.364631	0.019
**255**	5-O3B-B7-A	100	PI3K	0.310488	0.248
**256**	5-O3B-B17-	1000	PI3K	0.45722	0
**257**	5-O3B-C6-L	25	PI3K	0.388975	0
**258**	5-O3B-C7-A	10	PI3K	0.395997	0.001
**259**	5-O3B-C16-	250	PI3K	0.520259	0
**260**	5-O3B-C17-	100	PI3K	0.30434	0.02
**261**	5-O3B-D6-L	2.5	PI3K	0.269634	0.104
**262**	5-O3B-D7-A	1	PI3K	0.46793	0
**263**	5-O3B-D16	25	PI3K	0.421519	0
**264**	5-O3B-D17	10	PI3K	0.346154	0
**265**	5-O3B-E6-L	0.25	PI3K	0.398955	0.001
**266**	5-O3B-E7-A	0.1	PI3K	0.377972	0
**267**	5-O3B-E16-	2.5	PI3K	0.484953	0
**268**	5-O3B-E17-	1	PI3K	0.372814	0
**269**	5-O3B-F11-	10000	PI3K	0.458224	0
**270**	5-O3B-F16-	0.25	PI3K	0.335009	0.004
**271**	5-O3B-G9-S	10000	PI3K	0.490371	0
**272**	5-O3B-G11	1000	PI3K	0.427327	0
**273**	5-O3B-H9-S	1000	PI3K	0.373534	0
**274**	5-O3B-H11	100	PI3K	0.404296	0
**275**	5-O3B-I9-Se	100	PI3K	0.269302	0.413
**276**	5-O3B-I11-	10	PI3K	0.402583	0
**277**	5-O3B-J9-S	10	PI3K	0.42642	0
**278**	5-O3B-J11-	1	PI3K	0.407868	0
**279**	5-O3B-K9-S	1	PI3K	0.473922	0
**280**	5-O3B-L14-	0.1	PI3K	0.447889	0
**281**	5-O3B-L20-	1	PI3K	0.46326	0
**282**	5-O3B-L23-	0.1	PI3K	0.236427	0.753
**283**	5-O3B-M14	1	PI3K	0.34767	0.001
**284**	5-O3B-M20	10	PI3K	0.415281	0
**285**	5-O3B-M23	1	PI3K	0.418875	0

**286**	5-O3B-N14	10	PI3K	0.410629	0
**287**	5-O3B-N20	100	PI3K	0.428904	0
**288**	5-O3B-N23	10	PI3K	0.417332	0
**289**	5-O3B-O14	100	PI3K	0.390813	0
**290**	5-O3B-O20	1000	PI3K	0.380002	0.023
**291**	5-O3B-O23	100	PI3K	0.398394	0.021
**292**	5-O3B-P14-	1000	PI3K	0.384805	0.001
**293**	5-O3B-P20-	10000	PI3K	0.391501	0.021
**294**	5-O3B-P23-	1000	PI3K	0.391629	0.046
**295**	6-O3B-A8-T	10000	PI3K	0.37397	0.003
**296**	6-O3B-B8-T	1000	PI3K	0.395686	0
**297**	6-O3B-C8-T	100	PI3K	0.357915	0.001
**298**	6-O3B-D8-T	10	PI3K	0.05048	0.997
**299**	6-O3B-E8-T	1	PI3K	0.037461	0.995
**300**	6-O3B-L6-G	1	PI3K	0.035604	0.988
**301**	6-O3B-M6-	10	PI3K	0.036829	0.984
**302**	6-O3B-N6-C	100	PI3K	0.100969	0.996
**303**	6-O3B-O6-C	1000	PI3K	0.366151	0.003
**304**	6-O3B-P6-G	10000	PI3K	0.384974	0.026
**305**	1-O3B-F11-	10000	Topoisome	0.544393	0
**306**	1-O3B-G11	1000	Topoisome	0.596465	0
**307**	1-O3B-G20	1000	Topoisome	0.510805	0.004
**308**	1-O3B-H11	100	Topoisome	0.605628	0
**309**	1-O3B-H20	100	Topoisome	0.614907	0
**310**	1-O3B-I11-	10	Topoisome	0.473988	0
**311**	1-O3B-I20-	10	Topoisome	0.257165	0.235
**312**	1-O3B-J11-	1	Topoisome	0.109541	0.859
**313**	1-O3B-J20-	1	Topoisome	0.169478	0.489
**314**	1-O3B-K11-	1	Topoisome	0.589087	0
**315**	1-O3B-K20-	0.1	Topoisome	0.216333	0.481
**316**	1-O3B-L11-	10	Topoisome	0.56818	0
**317**	1-O3B-L14-	1	Topoisome	0.259145	0.256
**318**	1-O3B-M11	100	Topoisome	0.573914	0
**319**	1-O3B-M14	10	Topoisome	0.583141	0
**320**	1-O3B-N14	100	Topoisome	0.630913	0

**321**	1-O3B-O11	1000	Topoisome	0.52793	0
**322**	1-O3B-O14	1000	Topoisome	0.566032	0
**323**	1-O3B-P11·	10000	Topoisome	0.533833	0.003
**324**	1-O3B-P14·	10000	Topoisome	0.532349	0
**325**	3-O3B-A11·	10000	Topoisome	0.553963	0
**326**	3-O3B-B11·	1000	Topoisome	0.588509	0
**327**	3-O3B-C11·	100	Topoisome	0.53124	0
**328**	3-O3B-D11	10	Topoisome	0.389405	0.016
**329**	3-O3B-E11-	1	Topoisome	0.198005	0.316
**330**	3-O3B-G9-[	1000	Topoisome	0.425689	0.089
**331**	3-O3B-G10	10000	Topoisome	0.183705	0.707
**332**	3-O3B-H9-[	100	Topoisome	0.53399	0
**333**	3-O3B-H10	1000	Topoisome	0.17554	0.811
**334**	3-O3B-I9-D	10	Topoisome	0.244213	0.585
**335**	3-O3B-I10-'	100	Topoisome	0.168742	0.823
**336**	3-O3B-J9-D	1	Topoisome	0.158606	0.876
**337**	3-O3B-J10-	10	Topoisome	0.163231	0.829
**338**	3-O3B-K7-I	0.1	Topoisome	0.180771	0.698
**339**	3-O3B-K9-[	0.1	Topoisome	0.297546	0.027
**340**	3-O3B-K10·	1	Topoisome	0.239149	0.108
**341**	3-O3B-L6-[	0.1	Topoisome	0.086936	0.94
**342**	3-O3B-L7-I	1	Topoisome	0.212227	0.831
**343**	3-O3B-L9-V	0.5	Topoisome	0.224497	0.19
**344**	3-O3B-L10-	0.1	Topoisome	0.138575	0.462
**345**	3-O3B-L16-	1	Topoisome	0.17055	0.471
**346**	3-O3B-M6-I	1	Topoisome	0.08693	0.824
**347**	3-O3B-M7-I	10	Topoisome	0.45596	0.006
**348**	3-O3B-M9-'	5	Topoisome	0.337603	0.011
**349**	3-O3B-M1C	1	Topoisome	0.220684	0.355
**350**	3-O3B-M16	10	Topoisome	0.296171	0.138
**351**	3-O3B-N6-[	10	Topoisome	0.259298	0.133
**352**	3-O3B-N9-\	50	Topoisome	0.655131	0
**353**	3-O3B-N10	10	Topoisome	0.602497	0
**354**	3-O3B-N16	100	Topoisome	0.256368	0.088
**355**	3-O3B-O6-[	100	Topoisome	0.599992	0

**356**	3-O3B-O7-I	100	Topoisome	0.574986	0
**357**	3-O3B-O9-\	500	Topoisome	0.566181	0
**358**	3-O3B-O10	100	Topoisome	0.588928	0
**359**	3-O3B-O16	1000	Topoisome	0.41633	0.007
**360**	3-O3B-P6-D	1000	Topoisome	0.509794	0.001
**361**	3-O3B-P7-I	1000	Topoisome	0.522236	0.003
**362**	3-O3B-P9-V	5000	Topoisome	0.494846	0.011
**363**	3-O3B-P10-	1000	Topoisome	0.490447	0.003
**364**	3-O3B-P16-	10000	Topoisome	0.417531	0.003
**365**	1-O3B-A10-	10000	Mitotic	0.756165	0
**366**	1-O3B-A13-	1000	Mitotic	0.760604	0
**367**	1-O3B-A18-	1000	Mitotic	0.755062	0
**368**	1-O3B-B10-	1000	Mitotic	0.758333	0
**369**	1-O3B-B13-	100	Mitotic	0.264432	0.089
**370**	1-O3B-B18-	100	Mitotic	0.762567	0
**371**	1-O3B-C10-	100	Mitotic	0.762003	0
**372**	1-O3B-C13-	10	Mitotic	0.306691	0.531
**373**	1-O3B-C18-	10	Mitotic	0.762852	0
**374**	1-O3B-D10	10	Mitotic	0.76713	0
**375**	1-O3B-D13	1	Mitotic	0.404645	0.005
**376**	1-O3B-D18	1	Mitotic	0.404998	0.014
**377**	1-O3B-E10-	1	Mitotic	0.42086	0.009
**378**	1-O3B-E13-	0.1	Mitotic	0.480851	0.001
**379**	1-O3B-E18-	0.1	Mitotic	0.18424	0.887
**380**	1-O3B-F13-	1000	Mitotic	0.761124	0
**381**	1-O3B-G13	100	Mitotic	0.496122	0
**382**	1-O3B-G15	1000	Mitotic	0.757896	0
**383**	1-O3B-H13	10	Mitotic	0.143776	0.826
**384**	1-O3B-H15	100	Mitotic	0.762685	0
**385**	1-O3B-I13-'	1	Mitotic	0.521465	0.002
**386**	1-O3B-I15-I	10	Mitotic	0.762945	0
**387**	1-O3B-J13-	0.1	Mitotic	0.410827	0.018
**388**	1-O3B-J15-	1	Mitotic	0.761849	0
**389**	1-O3B-K7-V	0.1	Mitotic	0.076826	0.988
**390**	1-O3B-K15-	0.1	Mitotic	0.531795	0

**391**	1-O3B-L7-V	1	Mitotic	0.326569	0.448
**392**	1-O3B-L20-	0.1	Mitotic	0.447743	0.006
**393**	1-O3B-M7-'	10	Mitotic	0.345491	0.095
**394**	1-O3B-M2C	1	Mitotic	0.552367	0
**395**	1-O3B-N20	10	Mitotic	0.38492	0.009
**396**	1-O3B-O7-\	100	Mitotic	0.55076	0
**397**	1-O3B-O20	100	Mitotic	0.437595	0
**398**	1-O3B-P7-\	1000	Mitotic	0.755405	0
**399**	1-O3B-P20-	1000	Mitotic	0.766495	0
**400**	3-O3B-A7-D	1000	Mitotic	0.762946	0
**401**	3-O3B-B7-D	100	Mitotic	0.757348	0
**402**	3-O3B-C7-D	10	Mitotic	0.751095	0
**403**	3-O3B-D7-D	1	Mitotic	0.50065	0
**404**	3-O3B-E7-D	0.1	Mitotic	0.225289	0.865
**405**	6-O3B-L19-	1	Mitotic	0.285059	0.417
**406**	6-O3B-M19	10	Mitotic	0.093661	1
**407**	6-O3B-N19	100	Mitotic	0.328045	0.105
**408**	6-O3B-O19	1000	Mitotic	0.726624	0
**409**	6-O3B-P19-	10000	Mitotic	0.725601	0
**410**	2-O3B-A12-	250	MEK1/2	0.655	0
**411**	2-O3B-B12-	25	MEK1/2	0.705604	0
**412**	2-O3B-D12	2.5	MEK1/2	0.792121	0
**413**	2-O3B-E12-	0.25	MEK1/2	0.780571	0
**414**	2-O3B-F12-	2.5000000	MEK1/2	0.623457	0
**415**	2-O3B-F14-	1000	MEK1/2	0.64702	0
**416**	2-O3B-G14	100	MEK1/2	0.746005	0
**417**	2-O3B-H14	10	MEK1/2	0.786247	0
**418**	2-O3B-I14-	1	MEK1/2	0.666942	0
**419**	2-O3B-K14-	0.1	MEK1/2	0.705622	0
**420**	2-O3B-L20-	1	MEK1/2	0.113042	0.965
**421**	2-O3B-M2C	10	MEK1/2	0.719602	0
**422**	2-O3B-N20	100	MEK1/2	0.813017	0
**423**	2-O3B-O20	1000	MEK1/2	0.777253	0
**424**	2-O3B-P20-	10000	MEK1/2	0.650472	0
**425**	4-O3B-A10-	1000	MEK1/2	0.712649	0

**426**	4-O3B-A13-	1000	MEK1/2	0.705724	0
**427**	4-O3B-B10-	100	MEK1/2	0.710699	0
**428**	4-O3B-B13-	100	MEK1/2	0.720202	0
**429**	4-O3B-C10-	10	MEK1/2	0.749595	0
**430**	4-O3B-C13-	10	MEK1/2	0.651729	0
**431**	4-O3B-D10	1	MEK1/2	0.596349	0
**432**	4-O3B-D13	1	MEK1/2	0.732308	0
**433**	4-O3B-E10-	0.1	MEK1/2	0.581745	0
**434**	4-O3B-E13-	0.1	MEK1/2	0.513617	0.001
**435**	4-O3B-L19-	0.25	MEK1/2	0.680522	0
**436**	4-O3B-M19	2.5	MEK1/2	0.568112	0
**437**	4-O3B-N19	25	MEK1/2	0.761728	0
**438**	4-O3B-O19	250	MEK1/2	0.719964	0
**439**	4-O3B-P19-	2500	MEK1/2	0.738797	0
**440**	1-O3B-L2-C	1	PARP	0.628304	0
**441**	1-O3B-L6-R	1	PARP	0.613883	0.006
**442**	1-O3B-M2-	10	PARP	0.70191	0
**443**	1-O3B-M6-	10	PARP	0.635763	0.001
**444**	1-O3B-N2-(	100	PARP	0.792692	0
**445**	1-O3B-N6-F	100	PARP	0.774134	0
**446**	1-O3B-O2-(	1000	PARP	0.771268	0
**447**	1-O3B-O6-F	1000	PARP	0.746713	0
**448**	1-O3B-P2-C	10000	PARP	0.714549	0
**449**	1-O3B-P6-F	10000	PARP	0.728165	0
**450**	7-O3B-A3-T	1000	PARP	0.743921	0
**451**	7-O3B-B2-V	10000	PARP	0.759708	0
**452**	7-O3B-B3-T	100	PARP	0.732633	0
**453**	7-O3B-C2-V	1000	PARP	0.629163	0.002
**454**	7-O3B-C3-T	10	PARP	0.705224	0
**455**	7-O3B-D2-V	100	PARP	0.299831	0.298
**456**	7-O3B-D3-T	1	PARP	0.786184	0
**457**	7-O3B-E2-V	10	PARP	0.338671	0.48
**458**	7-O3B-E3-T	0.1	PARP	0.4718	0.043
**459**	7-O3B-F2-V	1	PARP	0.387274	0.057
**460**	7-O3B-G2-N	10000	PARP	0.765674	0

**461**	7-O3B-H2-N	1000	PARP	0.740539	0
**462**	7-O3B-I2-N	100	PARP	0.796172	0
**463**	7-O3B-J2-N	10	PARP	0.609283	0
**464**	7-O3B-K2-N	1	PARP	0.560882	0.002
**465**	3-O3B-A19-	1000	CDK	0.483954	0.003
**466**	3-O3B-B19-	100	CDK	0.475562	0.006
**467**	3-O3B-B23-	2500	CDK	0.30667	0.447
**468**	3-O3B-C19-	10	CDK	0.232103	0.426
**469**	3-O3B-C23-	250	CDK	0.263694	0.853
**470**	3-O3B-D19	1	CDK	0.180561	0.299
**471**	3-O3B-D23	25	CDK	0.172791	0.439
**472**	3-O3B-E19-	0.1	CDK	0.167884	0.608
**473**	3-O3B-E23-	2.5	CDK	0.19623	0.645
**474**	3-O3B-F23-	0.25	CDK	0.160683	0.425
**475**	3-O3B-K17-	1	CDK	0.314925	0.164
**476**	3-O3B-L19-	1	CDK	0.148426	0.33
**477**	3-O3B-M17	10	CDK	0.221405	0.501
**478**	3-O3B-M19	10	CDK	0.206343	0.532
**479**	3-O3B-N17	100	CDK	0.280692	0.208
**480**	3-O3B-N19	100	CDK	0.229729	0.318
**481**	3-O3B-O17	1000	CDK	0.297825	0.388
**482**	3-O3B-O19	1000	CDK	0.137287	0.597
**483**	3-O3B-P17-	10000	CDK	0.283812	0.353
**484**	3-O3B-P19-	10000	CDK	0.187019	0.569
**485**	4-O3B-A4-S	10000	CDK	0.52031	0
**486**	4-O3B-A8-N	10000	CDK	0.469114	0.018
**487**	4-O3B-B4-S	1000	CDK	0.516537	0.001
**488**	4-O3B-B8-N	1000	CDK	0.443631	0.018
**489**	4-O3B-C4-S	100	CDK	0.351185	0.098
**490**	4-O3B-C8-N	100	CDK	0.243381	0.426
**491**	4-O3B-D4-S	10	CDK	0.22233	0.493
**492**	4-O3B-D8-N	10	CDK	0.392152	0.014
**493**	4-O3B-E4-S	1	CDK	0.17498	0.829
**494**	4-O3B-E8-N	1	CDK	0.260571	0.361
**495**	4-O3B-F4-S	10000	CDK	0.422589	0.01

**496**	4-O3B-F22-	10000	CDK	0.514214	0.002
**497**	4-O3B-G4-S	1000	CDK	0.234325	0.75
**498**	4-O3B-G22	1000	CDK	0.505409	0
**499**	4-O3B-H4-S	100	CDK	0.226246	0.702
**500**	4-O3B-H22	100	CDK	0.141997	0.767
**501**	4-O3B-I4-Se	10	CDK	0.230503	0.665
**502**	4-O3B-I22-,	10	CDK	0.121732	0.777
**503**	4-O3B-J4-Se	1	CDK	0.180306	0.888
**504**	4-O3B-J22-	1	CDK	0.146899	0.611
**505**	5-O3B-A19-	10000	CDK	0.499769	0.006
**506**	5-O3B-B19-	1000	CDK	0.399746	0.053
**507**	5-O3B-C19-	100	CDK	0.228745	0.527
**508**	5-O3B-D19	10	CDK	0.257724	0.437
**509**	5-O3B-E19-	1	CDK	0.245301	0.403
**510**	5-O3B-K17-	1	CDK	0.247131	0.39
**511**	5-O3B-M17	10	CDK	0.240362	0.46
**512**	5-O3B-N17	100	CDK	0.218722	0.517
**513**	5-O3B-O17	1000	CDK	0.513662	0.002
**514**	5-O3B-P17-	10000	CDK	0.501217	0.004
**515**	6-O3B-A17-	1000	CDK	0.272877	0.356
**516**	6-O3B-B17-	100	CDK	0.273809	0.24
**517**	6-O3B-C17-	10	CDK	0.415416	0.011
**518**	6-O3B-D17	1	CDK	0.087184	0.904
**519**	6-O3B-E17-	0.1	CDK	0.121856	0.998
**520**	6-O3B-L15-	1	CDK	0.233089	0.384
**521**	6-O3B-M15	10	CDK	0.270315	0.164
**522**	6-O3B-N15	100	CDK	0.436467	0.022
**523**	6-O3B-O15	1000	CDK	0.500338	0.005
**524**	6-O3B-P15-	10000	CDK	0.509149	0.004
**525**	7-O3B-A21-	10000	BET	0.591642	0
**526**	7-O3B-A22-	30000	BET	0.625149	0
**527**	7-O3B-B21-	1000	BET	0.492383	0
**528**	7-O3B-B22-	3000	BET	0.735638	0
**529**	7-O3B-C21-	100	BET	0.426484	0.004
**530**	7-O3B-C22-	300	BET	0.505892	0

**531**	7-O3B-D21	10	BET	0.189813	0.932
**532**	7-O3B-D22	30	BET	0.694106	0
**533**	7-O3B-E21-	1	BET	0.143898	0.944
**534**	7-O3B-E22-	3	BET	0.158651	0.978
**535**	7-O3B-G10	10000	BET	0.645612	0
**536**	7-O3B-G15	10000	BET	0.6494	0
**537**	7-O3B-H10	1000	BET	0.636324	0
**538**	7-O3B-H15	1000	BET	0.628092	0
**539**	7-O3B-I10-	100	BET	0.658024	0
**540**	7-O3B-I15-	100	BET	0.656782	0
**541**	7-O3B-J10-	10	BET	0.76883	0
**542**	7-O3B-J15-	10	BET	0.45892	0.002
**543**	7-O3B-K10-	1	BET	0.259812	0.225
**544**	7-O3B-K13-	1	BET	0.60736	0
**545**	7-O3B-K15-	1	BET	0.57081	0
**546**	7-O3B-L12-	1	BET	0.414961	0.01
**547**	7-O3B-L13-	10	BET	0.646053	0
**548**	7-O3B-L20-	1	BET	0.093082	0.934
**549**	7-O3B-L23-	0.03	BET	0.15913	0.786
**550**	7-O3B-M12	10	BET	0.749937	0
**551**	7-O3B-M13	100	BET	0.635142	0
**552**	7-O3B-M20	10	BET	0.587989	0
**553**	7-O3B-M23	0.3	BET	0.586273	0
**554**	7-O3B-N12	100	BET	0.565007	0
**555**	7-O3B-N13	1000	BET	0.666358	0
**556**	7-O3B-N20	100	BET	0.679669	0
**557**	7-O3B-N23	3	BET	0.542976	0
**558**	7-O3B-O12	1000	BET	0.619865	0
**559**	7-O3B-O20	1000	BET	0.643628	0
**560**	7-O3B-O23	30	BET	0.545195	0
**561**	7-O3B-P12-	10000	BET	0.645894	0
**562**	7-O3B-P13-	10000	BET	0.657807	0
**563**	7-O3B-P20-	10000	BET	0.641947	0
**564**	7-O3B-P23-	300	BET	0.630692	0
**565**	8-O3B-K22-	1	BET	0.101395	0.996

**566**	8-O3B-L22-	10	BET	0.157786	0.623
**567**	8-O3B-M22	100	BET	0.448096	0.002
**568**	8-O3B-N22	1000	BET	0.445863	0.003
**569**	8-O3B-O22	10000	BET	0.553633	0
**570**	1-O3B-A3-\	10000	HDAC	0.45755	0.015
**571**	1-O3B-B3-\	1000	HDAC	0.406744	0.001
**572**	1-O3B-C3-\	100	HDAC	0.288254	0.041
**573**	1-O3B-D3-\	10	HDAC	0.379941	0.021
**574**	1-O3B-E3-V	1	HDAC	0.039727	1
**575**	1-O3B-L12-	0.1	HDAC	0.357034	0.002
**576**	1-O3B-M12	1	HDAC	0.46074	0
**577**	1-O3B-N12	10	HDAC	0.456038	0.013
**578**	1-O3B-O12	100	HDAC	0.346174	0.062
**579**	1-O3B-P12-	1000	HDAC	0.354802	0.021
**580**	3-O3B-A4-F	1000	HDAC	0.389337	0.018
**581**	3-O3B-B4-F	100	HDAC	0.396696	0.013
**582**	3-O3B-C4-F	10	HDAC	0.527153	0
**583**	3-O3B-D4-F	1	HDAC	0.189579	0.783
**584**	3-O3B-E4-P	0.1	HDAC	0.080834	0.98
**585**	3-O3B-F7-C	1000	HDAC	0.373157	0.079
**586**	3-O3B-G7-(	100	HDAC	0.28523	0.054
**587**	3-O3B-G12	1000000	HDAC	0.038017	0.977
**588**	3-O3B-H7-(	10	HDAC	0.263368	0.233
**589**	3-O3B-H12	100000	HDAC	0.221083	0.471
**590**	3-O3B-I7-Q	1	HDAC	0.195961	0.781
**591**	3-O3B-I12-'	10000	HDAC	0.209257	0.593
**592**	3-O3B-J7-Q	0.1	HDAC	0.183585	0.763
**593**	3-O3B-J12-	1000	HDAC	0.184148	0.816
**594**	3-O3B-K3-E	1	HDAC	0.106607	0.968
**595**	3-O3B-K12-	100	HDAC	0.08838	0.888
**596**	3-O3B-L3-B	10	HDAC	0.077536	0.965
**597**	3-O3B-M3-I	100	HDAC	0.445441	0
**598**	3-O3B-N3-E	1000	HDAC	0.401016	0.007
**599**	3-O3B-O3-E	10000	HDAC	0.347488	0.095
**600**	7-O3B-A5-N	10000	HDAC	0.467253	0.002

**601**	7-O3B-A7-C	10000	HDAC	0.363323	0.059
**602**	7-O3B-A9-G	1000	HDAC	0.437475	0
**603**	7-O3B-A12-	10000	HDAC	0.440837	0
**604**	7-O3B-B5-M	1000	HDAC	0.449344	0
**605**	7-O3B-B7-C	1000	HDAC	0.363892	0.052
**606**	7-O3B-B12-	1000	HDAC	0.438412	0
**607**	7-O3B-C5-M	100	HDAC	0.452164	0
**608**	7-O3B-C7-C	100	HDAC	0.495774	0
**609**	7-O3B-C9-G	100	HDAC	0.434662	0
**610**	7-O3B-D7-C	10	HDAC	0.317556	0.065
**611**	7-O3B-D9-C	10	HDAC	0.172207	0.874
**612**	7-O3B-D12	100	HDAC	0.497666	0
**613**	7-O3B-E5-M	10	HDAC	0.28464	0.098
**614**	7-O3B-E7-C	1	HDAC	0.133966	0.986
**615**	7-O3B-E9-G	1	HDAC	0.305857	0.068
**616**	7-O3B-E12-	10	HDAC	0.4469	0
**617**	7-O3B-F5-M	1	HDAC	0.117262	0.782
**618**	7-O3B-F7-R	10000	HDAC	0.480933	0
**619**	7-O3B-F9-G	0.1	HDAC	0.478194	0
**620**	7-O3B-F12-	1	HDAC	0.532427	0
**621**	7-O3B-F19-	10000	HDAC	0.42819	0
**622**	7-O3B-G7-F	1000	HDAC	0.435816	0
**623**	7-O3B-G19	1000	HDAC	0.36935	0.078
**624**	7-O3B-H7-F	100	HDAC	0.288153	0.111
**625**	7-O3B-I7-R	10	HDAC	0.299629	0.089
**626**	7-O3B-I19-	100	HDAC	0.349277	0.119
**627**	7-O3B-J7-R	1	HDAC	0.400743	0.004
**628**	7-O3B-J19-	10	HDAC	0.387918	0.001
**629**	7-O3B-K4-E	1	HDAC	0.278982	0.098
**630**	7-O3B-K11-	1	HDAC	0.497802	0
**631**	7-O3B-K18-	1	HDAC	0.3291	0.001
**632**	7-O3B-K19-	1	HDAC	0.224286	0.597
**633**	7-O3B-L2-T	0.1	HDAC	0.150851	0.767
**634**	7-O3B-L4-E	10	HDAC	0.037015	0.986
**635**	7-O3B-L5-P	1	HDAC	0.334112	0

**636**	7-O3B-L8-A	1	HDAC	0.216801	0.418
**637**	7-O3B-L10-	1	HDAC	0.42353	0
**638**	7-O3B-L11-	10	HDAC	0.380395	0.017
**639**	7-O3B-L14-	1	HDAC	0.252071	0.254
**640**	7-O3B-L16-	1	HDAC	0.389818	0.001
**641**	7-O3B-L18-	10	HDAC	0.227043	0.395
**642**	7-O3B-M2-1	1	HDAC	0.097303	0.734
**643**	7-O3B-M5-I	10	HDAC	0.158415	0.507
**644**	7-O3B-M8-,	10	HDAC	0.172004	0.308
**645**	7-O3B-M1C	10	HDAC	0.419676	0
**646**	7-O3B-M11	100	HDAC	0.458848	0
**647**	7-O3B-M14	10	HDAC	0.343433	0.016
**648**	7-O3B-M16	10	HDAC	0.350017	0.001
**649**	7-O3B-M18	100	HDAC	0.323435	0.006
**650**	7-O3B-N2-1	10	HDAC	0.124851	0.609
**651**	7-O3B-N4-E	100	HDAC	0.525962	0
**652**	7-O3B-N5-F	100	HDAC	0.592777	0
**653**	7-O3B-N8-/	100	HDAC	0.579592	0
**654**	7-O3B-N10	100	HDAC	0.627078	0
**655**	7-O3B-N14	100	HDAC	0.405128	0
**656**	7-O3B-N16	100	HDAC	0.457962	0
**657**	7-O3B-N18	1000	HDAC	0.524529	0
**658**	7-O3B-O2-1	100	HDAC	0.09277	0.862
**659**	7-O3B-O4-E	1000	HDAC	0.579223	0
**660**	7-O3B-O5-F	1000	HDAC	0.434015	0
**661**	7-O3B-O8-/	1000	HDAC	0.424643	0
**662**	7-O3B-O10	1000	HDAC	0.500361	0
**663**	7-O3B-O11	1000	HDAC	0.461456	0
**664**	7-O3B-O14	1000	HDAC	0.268162	0.003
**665**	7-O3B-O16	1000	HDAC	0.42793	0
**666**	7-O3B-P2-T	1000	HDAC	0.373438	0
**667**	7-O3B-P4-E	10000	HDAC	0.448763	0
**668**	7-O3B-P5-F	10000	HDAC	0.388823	0.009
**669**	7-O3B-P8-A	10000	HDAC	0.400011	0.005
**670**	7-O3B-P10-	10000	HDAC	0.433284	0

**671**	7-O3B-P11-	10000	HDAC	0.360933	0.067
**672**	7-O3B-P14-	10000	HDAC	0.08272	0.737
**673**	7-O3B-P16-	10000	HDAC	0.479376	0
**674**	7-O3B-P18-	10000	HDAC	0.45617	0.002
**0**	2-O8W-A16	10000	EGFR	0.328347	0.034
**1**	2-O8W-A19	10000	EGFR	0.244094	0.307
**2**	2-O8W-B19	1000	EGFR	0.088729	0.901
**3**	2-O8W-C16	1000	EGFR	0.143295	0.377
**4**	2-O8W-C19	100	EGFR	0.138336	0.501
**5**	2-O8W-D16	100	EGFR	0.189684	0.613
**6**	2-O8W-D19	10	EGFR	0.229808	0.341
**7**	2-O8W-E16	10	EGFR	0.311434	0.103
**8**	2-O8W-E19	1	EGFR	0.275554	0.084
**9**	2-O8W-F16	1	EGFR	0.285103	0.092
**10**	2-O8W-K11	0.1	EGFR	0.213301	0.466
**11**	2-O8W-L11	1	EGFR	0.280982	0.118
**12**	2-O8W-L16	0.25	EGFR	0.309695	0.063
**13**	2-O8W-L19	0.1	EGFR	0.165436	0.455
**14**	2-O8W-M1	10	EGFR	0.127871	0.952
**15**	2-O8W-M1	2.5	EGFR	0.326406	0.016
**16**	2-O8W-M1	1	EGFR	0.382492	0.001
**17**	2-O8W-N1(	25	EGFR	0.289741	0.022
**18**	2-O8W-N19	10	EGFR	0.249714	0.083
**19**	2-O8W-O1:	100	EGFR	0.203537	0.385
**20**	2-O8W-O1(	250	EGFR	0.227	0.238
**21**	2-O8W-O19	100	EGFR	0.375248	0.02
**22**	2-O8W-P11	1000	EGFR	0.208671	0.388
**23**	2-O8W-P16	2500	EGFR	0.230325	0.43
**24**	2-O8W-P19	1000	EGFR	0.320697	0.081
**25**	3-O8W-F21	10000	EGFR	0.142337	0.993
**26**	3-O8W-G2(	1000	EGFR	0.217836	0.387
**27**	3-O8W-G2:	1000	EGFR	0.204977	0.382
**28**	3-O8W-H2(	100	EGFR	0.124082	0.913
**29**	3-O8W-H2:	100	EGFR	0.199357	0.597
**30**	3-O8W-I20	10	EGFR	0.21729	0.22

**31**	3-O8W-I21	10	EGFR	0.124113	0.664
**32**	3-O8W-J20	1	EGFR	0.423275	0
**33**	3-O8W-J21	1	EGFR	0.268039	0.073
**34**	3-O8W-K4-	1	EGFR	0.213216	0.343
**35**	3-O8W-K1[illegible]	0.1	EGFR	0.391964	0.001
**36**	3-O8W-K2[illegible]	0.1	EGFR	0.124853	0.645
**37**	3-O8W-L4-[illegible]	10	EGFR	0.145789	0.665
**38**	3-O8W-L1[illegible]	1	EGFR	0.095316	0.868
**39**	3-O8W-M1	10	EGFR	0.368544	0.001
**40**	3-O8W-N4-	100	EGFR	0.263233	0.117
**41**	3-O8W-N1[illegible]	100	EGFR	0.382959	0.002
**42**	3-O8W-O4-	1000	EGFR	0.167952	0.593
**43**	3-O8W-P4-	10000	EGFR	0.075376	1
**44**	3-O8W-P1[illegible]	1000	EGFR	0.25774	0.117
**45**	4-O8W-F13	1000	EGFR	0.250079	0.134
**46**	4-O8W-G1[illegible]	100	EGFR	0.211495	0.378
**47**	4-O8W-G1[illegible]	10000	EGFR	0.194361	0.67
**48**	4-O8W-H1[illegible]	10	EGFR	0.204586	0.617
**49**	4-O8W-H1[illegible]	1000	EGFR	0.101403	0.86
**50**	4-O8W-I13	1	EGFR	0.172443	0.433
**51**	4-O8W-I16	100	EGFR	0.155569	0.852
**52**	4-O8W-J13	0.1	EGFR	0.378599	0.001
**53**	4-O8W-J16	10	EGFR	0.30378	0.036
**54**	4-O8W-K7-	1	EGFR	0.140126	0.98
**55**	4-O8W-K1[illegible]	0.1	EGFR	0.155775	0.391
**56**	4-O8W-K1[illegible]	1	EGFR	0.260827	0.077
**57**	4-O8W-L7-[illegible]	10	EGFR	0.244577	0.169
**58**	4-O8W-L13	1	EGFR	0.217269	0.395
**59**	4-O8W-M7	100	EGFR	0.317584	0.048
**60**	4-O8W-M1	10	EGFR	0.255198	0.17
**61**	4-O8W-N1[illegible]	100	EGFR	0.138847	0.496
**62**	4-O8W-O7-	1000	EGFR	0.132642	0.918
**63**	4-O8W-P7-	10000	EGFR	0.150985	0.64
**64**	4-O8W-P1[illegible]	1000	EGFR	0.186109	0.566
**65**	5-O8W-F4-[illegible]	1000	EGFR	0.214748	0.408

**66**	5-O8W-F7-/	1000	EGFR	0.185126	0.616
**67**	5-O8W-G4-	100	EGFR	0.254543	0.376
**68**	5-O8W-G7-	100	EGFR	0.188106	0.535
**69**	5-O8W-H4-	10	EGFR	0.136368	0.741
**70**	5-O8W-H7-	10	EGFR	0.419432	0.001
**71**	5-O8W-I4-F	1	EGFR	0.22921	0.486
**72**	5-O8W-I7-/	1	EGFR	0.269312	0.247
**73**	5-O8W-J4-I	0.1	EGFR	0.212216	0.252
**74**	5-O8W-J7-/	0.1	EGFR	0.212447	0.678
**75**	5-O8W-K7-	0.1	EGFR	0.182131	0.696
**76**	5-O8W-L7-	1	EGFR	0.310015	0.162
**77**	5-O8W-M7	10	EGFR	0.18197	0.824
**78**	5-O8W-O7-	100	EGFR	0.191093	0.257
**79**	5-O8W-P7-	1000	EGFR	0.241348	0.288
**80**	2-O8W-A1!	2500	VEGFR	0.274853	0.103
**81**	2-O8W-A17	10000	VEGFR	0.313133	0.043
**82**	2-O8W-A2(	10000	VEGFR	0.147222	0.982
**83**	2-O8W-B1!	250	VEGFR	0.459396	0
**84**	2-O8W-B17	1000	VEGFR	0.333161	0.001
**85**	2-O8W-B2(	1000	VEGFR	0.211814	0.351
**86**	2-O8W-C1!	25	VEGFR	0.153879	0.684
**87**	2-O8W-C17	100	VEGFR	0.403588	0
**88**	2-O8W-D1!	2.5	VEGFR	0.438098	0.001
**89**	2-O8W-D1;	10	VEGFR	0.158816	0.6
**90**	2-O8W-D2(	100	VEGFR	0.322472	0
**91**	2-O8W-E17	1	VEGFR	0.384829	0
**92**	2-O8W-E2C	10	VEGFR	0.159843	0.806
**93**	2-O8W-F13	10000	VEGFR	0.180052	0.894
**94**	2-O8W-F15	0.25	VEGFR	0.316059	0.001
**95**	2-O8W-F1S	10000	VEGFR	0.123667	0.987
**96**	2-O8W-F2C	1	VEGFR	0.332658	0
**97**	2-O8W-F21	10000	VEGFR	0.152123	0.925
**98**	2-O8W-G1(	10000	VEGFR	0.402293	0.002
**99**	2-O8W-G1:	1000	VEGFR	0.441532	0.001
**100**	2-O8W-G1!	1000	VEGFR	0.114163	0.634

**101**	2-O8W-G2:	1000	VEGFR	0.46452	0
**102**	2-O8W-H1(	1000	VEGFR	0.105315	0.914
**103**	2-O8W-H1:	100	VEGFR	0.26948	0.141
**104**	2-O8W-H2:	100	VEGFR	0.090945	0.935
**105**	2-O8W-I10	100	VEGFR	0.3647	0.003
**106**	2-O8W-I13	10	VEGFR	0.34041	0.049
**107**	2-O8W-I19	100	VEGFR	0.376639	0.001
**108**	2-O8W-I21	10	VEGFR	0.215276	0.305
**109**	2-O8W-J10	10	VEGFR	0.22047	0.442
**110**	2-O8W-J13	1	VEGFR	0.496639	0
**111**	2-O8W-J19	10	VEGFR	0.453386	0
**112**	2-O8W-J21	1	VEGFR	0.440784	0
**113**	2-O8W-K1(	1	VEGFR	0.395232	0
**114**	2-O8W-K1:	0.1	VEGFR	0.128429	0.982
**115**	2-O8W-K1:	1	VEGFR	0.444262	0
**116**	2-O8W-K1:	1	VEGFR	0.201414	0.121
**117**	2-O8W-L12	0.1	VEGFR	0.202547	0.292
**118**	2-O8W-L13	1	VEGFR	0.50736	0
**119**	2-O8W-L21	0.1	VEGFR	0.352949	0.011
**120**	2-O8W-M1	1	VEGFR	0.217062	0.28
**121**	2-O8W-M1	10	VEGFR	0.380845	0.011
**122**	2-O8W-M1	10	VEGFR	0.434153	0
**123**	2-O8W-M2	1	VEGFR	0.182131	0.21
**124**	2-O8W-N1:	10	VEGFR	0.384652	0
**125**	2-O8W-N1:	100	VEGFR	0.247517	0.101
**126**	2-O8W-N1:	100	VEGFR	0.416522	0
**127**	2-O8W-N2:	10	VEGFR	0.207547	0.157
**128**	2-O8W-O1:	100	VEGFR	0.19594	0.458
**129**	2-O8W-O1:	1000	VEGFR	0.266951	0.43
**130**	2-O8W-O2:	100	VEGFR	0.3705	0.014
**131**	2-O8W-P1:	1000	VEGFR	0.223979	0.383
**132**	2-O8W-P1:	1000	VEGFR	0.171862	0.67
**133**	2-O8W-P1:	10000	VEGFR	0.173964	0.922
**134**	2-O8W-P2:	1000	VEGFR	0.469772	0
**135**	3-O8W-A3-	1000	VEGFR	0.223447	0.295

**136**	3-O8W-A6- 1000	VEGFR	0.149439	0.879
**137**	3-O8W-A18 1000	VEGFR	0.184116	0.569
**138**	3-O8W-B3- 100	VEGFR	0.146983	0.681
**139**	3-O8W-B6- 100	VEGFR	0.269604	0.11
**140**	3-O8W-B18 100	VEGFR	0.156573	0.857
**141**	3-O8W-C3- 10	VEGFR	0.142366	0.828
**142**	3-O8W-C6- 10	VEGFR	0.228371	0.307
**143**	3-O8W-C18 10	VEGFR	0.142416	0.778
**144**	3-O8W-D3- 1	VEGFR	0.106254	0.918
**145**	3-O8W-D6- 1	VEGFR	0.279811	0.048
**146**	3-O8W-D18 1	VEGFR	0.142104	0.869
**147**	3-O8W-E3- 0.1	VEGFR	0.164393	0.697
**148**	3-O8W-E6- 0.1	VEGFR	0.152848	0.735
**149**	3-O8W-E18 0.1	VEGFR	0.39345	0
**150**	3-O8W-F18 1000	VEGFR	0.318543	0.005
**151**	3-O8W-G18 100	VEGFR	0.25245	0.07
**152**	3-O8W-H18 10	VEGFR	0.177718	0.235
**153**	3-O8W-I18 1	VEGFR	0.157671	0.815
**154**	3-O8W-J18 0.1	VEGFR	0.156517	0.425
**155**	4-O8W-A12 10000	VEGFR	0.164114	0.999
**156**	4-O8W-A15 2500	VEGFR	0.057653	0.982
**157**	4-O8W-A20 10000	VEGFR	0.384531	0.008
**158**	4-O8W-B12 1000	VEGFR	0.148925	0.794
**159**	4-O8W-B15 250	VEGFR	0.125205	0.94
**160**	4-O8W-B20 1000	VEGFR	0.385766	0
**161**	4-O8W-C15 25	VEGFR	0.365941	0.003
**162**	4-O8W-D12 100	VEGFR	0.423493	0.001
**163**	4-O8W-D15 2.5	VEGFR	0.328555	0.006
**164**	4-O8W-D20 100	VEGFR	0.124935	0.815
**165**	4-O8W-E12 10	VEGFR	0.169168	0.574
**166**	4-O8W-E20 10	VEGFR	0.401391	0
**167**	4-O8W-F12 1	VEGFR	0.346527	0.014
**168**	4-O8W-F15 0.25	VEGFR	0.1627	0.807
**169**	4-O8W-F20 1	VEGFR	0.257251	0.219
**170**	4-O8W-L16 1	VEGFR	0.089141	0.879

**171**	4-O8W-M1	10	VEGFR	0.229197	0.276
**172**	4-O8W-N1(	100	VEGFR	0.295011	0.003
**173**	4-O8W-O1(	1000	VEGFR	0.305393	0.003
**174**	4-O8W-P1€	10000	VEGFR	0.102982	0.973
**175**	2-O8W-L1C	1	PI3K	0.293749	0.034
**176**	2-O8W-M1	10	PI3K	0.398325	0
**177**	2-O8W-N1(	100	PI3K	0.295689	0.051
**178**	2-O8W-O1(	1000	PI3K	0.37094	0
**179**	2-O8W-P1(	10000	PI3K	0.373268	0.001
**180**	3-O8W-A1€	2500	PI3K	0.051225	0.997
**181**	3-O8W-C1€	250	PI3K	0.074404	0.881
**182**	3-O8W-D1(	25	PI3K	0.230246	0.131
**183**	3-O8W-E1€	2.5	PI3K	0.311886	0.002
**184**	3-O8W-F1€	0.25	PI3K	0.289582	0.011
**185**	3-O8W-F17	100000	PI3K	0.190682	0.976
**186**	3-O8W-F1¢	500	PI3K	0.219617	0.024
**187**	3-O8W-G1;	10000	PI3K	0.138384	0.922
**188**	3-O8W-G1¢	50	PI3K	0.228308	0.168
**189**	3-O8W-H1;	1000	PI3K	0.43965	0
**190**	3-O8W-I17	100	PI3K	0.129604	0.548
**191**	3-O8W-I19	5	PI3K	0.084508	0.889
**192**	3-O8W-J17	10	PI3K	0.108623	0.792
**193**	3-O8W-J19	0.5	PI3K	0.176935	0.644
**194**	3-O8W-K1¢	0.05	PI3K	0.313405	0.04
**195**	3-O8W-L8-I	1	PI3K	0.234088	0.113
**196**	3-O8W-L21	0.1	PI3K	0.039993	0.998
**197**	3-O8W-M8	10	PI3K	0.26391	0.028
**198**	3-O8W-M2	1	PI3K	0.285928	0.013
**199**	3-O8W-N8-	100	PI3K	0.191944	0.077
**200**	3-O8W-N2:	10	PI3K	0.113144	0.774
**201**	3-O8W-O8-	1000	PI3K	0.257728	0.056
**202**	3-O8W-O2:	100	PI3K	0.155176	0.419
**203**	3-O8W-P8-	10000	PI3K	0.274652	0.101
**204**	3-O8W-P21	1000	PI3K	0.264647	0.05
**205**	4-O8W-A1¢	2500	PI3K	0.235077	0.076

**206**	4-O8W-B19	250	PI3K	0.201361	0.099
**207**	4-O8W-C19	25	PI3K	0.3471	0
**208**	4-O8W-D19	2.5	PI3K	0.243212	0.1
**209**	4-O8W-E19	0.25	PI3K	0.316728	0.069
**210**	4-O8W-F14	1000	PI3K	0.188525	0.981
**211**	4-O8W-G2-	2500	PI3K	0.313085	0.013
**212**	4-O8W-G5-	10000	PI3K	0.283952	0.018
**213**	4-O8W-G14	100	PI3K	0.302808	0.058
**214**	4-O8W-G20	10000	PI3K	0.333287	0.391
**215**	4-O8W-H2-	250	PI3K	0.115366	0.674
**216**	4-O8W-H5-	1000	PI3K	0.210234	0.3
**217**	4-O8W-H14	10	PI3K	0.361758	0.004
**218**	4-O8W-H20	1000	PI3K	0.281857	0.002
**219**	4-O8W-I2-T	25	PI3K	0.216143	0.223
**220**	4-O8W-I5-S	100	PI3K	0.292694	0.008
**221**	4-O8W-I14	1	PI3K	0.425305	0
**222**	4-O8W-I20	100	PI3K	0.349984	0.001
**223**	4-O8W-J2-T	2.5	PI3K	0.235441	0.088
**224**	4-O8W-J5-S	10	PI3K	0.3039	0.047
**225**	4-O8W-J20	10	PI3K	0.045013	0.997
**226**	4-O8W-K2-	0.25	PI3K	0.283566	0.011
**227**	4-O8W-K4-	0.1	PI3K	0.268932	0.022
**228**	4-O8W-K5-	1	PI3K	0.252568	0.065
**229**	4-O8W-K14	0.1	PI3K	0.442637	0
**230**	4-O8W-K20	1	PI3K	0.367713	0.002
**231**	4-O8W-L4-I	1	PI3K	0.172858	0.355
**232**	4-O8W-L14	0.1	PI3K	0.119991	0.667
**233**	4-O8W-L15	1	PI3K	0.396083	0
**234**	4-O8W-L21	0.1	PI3K	0.225652	0.275
**235**	4-O8W-M1	1	PI3K	0.201546	0.179
**236**	4-O8W-M1	10	PI3K	0.231501	0.201
**237**	4-O8W-M2	1	PI3K	0.134075	0.91
**238**	4-O8W-N4-	10	PI3K	0.344251	0.013
**239**	4-O8W-N14	10	PI3K	0.260172	0.015
**240**	4-O8W-N15	100	PI3K	0.124474	0.526

**241**	4-O8W-N2:	10	PI3K	0.159292	0.252
**242**	4-O8W-O4-	100	PI3K	0.304177	0.017
**243**	4-O8W-O14	100	PI3K	0.360058	0.001
**244**	4-O8W-O15	1000	PI3K	0.283317	0.022
**245**	4-O8W-O2:	100	PI3K	0.309419	0.009
**246**	4-O8W-P4-	1000	PI3K	0.309974	0.072
**247**	4-O8W-P14	1000	PI3K	0.317815	0.051
**248**	4-O8W-P15	10000	PI3K	0.42309	0
**249**	4-O8W-P21	1000	PI3K	0.297127	0.006
**250**	5-O8W-A6-	2500	PI3K	0.309541	0.116
**251**	5-O8W-A7-	1000	PI3K	0.441854	0
**252**	5-O8W-A16	2500	PI3K	0.316263	0.002
**253**	5-O8W-A17	10000	PI3K	0.278122	0.126
**254**	5-O8W-B6-	250	PI3K	0.282806	0.035
**255**	5-O8W-B7-	100	PI3K	0.302526	0.004
**256**	5-O8W-B17	1000	PI3K	0.208471	0.333
**257**	5-O8W-C6-	25	PI3K	0.338349	0.003
**258**	5-O8W-C7-	10	PI3K	0.214283	0.236
**259**	5-O8W-C16	250	PI3K	0.313759	0.015
**260**	5-O8W-C17	100	PI3K	0.310036	0
**261**	5-O8W-D6-	2.5	PI3K	0.168527	0.498
**262**	5-O8W-D7-	1	PI3K	0.266967	0.034
**263**	5-O8W-D16	25	PI3K	0.345878	0.002
**264**	5-O8W-D17	10	PI3K	0.282444	0.062
**265**	5-O8W-E6-I	0.25	PI3K	0.280197	0.02
**266**	5-O8W-E7-	0.1	PI3K	0.240547	0.147
**267**	5-O8W-E16	2.5	PI3K	0.169163	0.713
**268**	5-O8W-E17	1	PI3K	0.19777	0.257
**269**	5-O8W-F11	10000	PI3K	0.32377	0.002
**270**	5-O8W-F16	0.25	PI3K	0.211896	0.698
**271**	5-O8W-G9-	10000	PI3K	0.482175	0
**272**	5-O8W-G1:	1000	PI3K	0.322237	0.002
**273**	5-O8W-H9-	1000	PI3K	0.099311	0.712
**274**	5-O8W-H1:	100	PI3K	0.196311	0.233
**275**	5-O8W-I9-S	100	PI3K	0.357437	0.001

**276**	5-O8W-I11	10	PI3K	0.088493	0.778
**277**	5-O8W-J9-S	10	PI3K	0.231172	0.361
**278**	5-O8W-J11	1	PI3K	0.137909	0.763
**279**	5-O8W-K9-	1	PI3K	0.189524	0.592
**280**	5-O8W-L14	0.1	PI3K	0.150665	0.36
**281**	5-O8W-L2C	1	PI3K	0.210257	0.33
**282**	5-O8W-L23	0.1	PI3K	0.067168	0.938
**283**	5-O8W-M1	1	PI3K	0.298355	0.008
**284**	5-O8W-M2	10	PI3K	0.517455	0
**285**	5-O8W-M2	1	PI3K	0.113421	0.595
**286**	5-O8W-N14	10	PI3K	0.412518	0
**287**	5-O8W-N2(	100	PI3K	0.080204	0.869
**288**	5-O8W-N2:	10	PI3K	0.310285	0.006
**289**	5-O8W-O14	100	PI3K	0.414658	0
**290**	5-O8W-O2(	1000	PI3K	0.334777	0.002
**291**	5-O8W-O2:	100	PI3K	0.283638	0.028
**292**	5-O8W-P14	1000	PI3K	0.104893	0.666
**293**	5-O8W-P2(	10000	PI3K	0.364399	0
**294**	5-O8W-P2:	1000	PI3K	0.312453	0.161
**295**	6-O8W-A8-	10000	PI3K	0.268008	0.04
**296**	6-O8W-B8-	1000	PI3K	0.054804	0.996
**297**	6-O8W-C8-	100	PI3K	0.249591	0.107
**298**	6-O8W-D8-	10	PI3K	0.246461	0.071
**299**	6-O8W-E8-	1	PI3K	0.277683	0.041
**300**	6-O8W-L6-	1	PI3K	0.097928	0.783
**301**	6-O8W-M6	10	PI3K	0.180334	0.444
**302**	6-O8W-N6-	100	PI3K	0.091203	0.834
**303**	6-O8W-O6-	1000	PI3K	0.274569	0.043
**304**	6-O8W-P6-	10000	PI3K	0.297177	0.345
**305**	1-O8W-F11	10000	Topoisome	0.433779	0.012
**306**	1-O8W-G1:	1000	Topoisome	0.43907	0.003
**307**	1-O8W-G2(	1000	Topoisome	0.441837	0.026
**308**	1-O8W-H1:	100	Topoisome	0.463145	0
**309**	1-O8W-H2(	100	Topoisome	0.483589	0
**310**	1-O8W-I11	10	Topoisome	0.483321	0.001

311	1-O8W-I20	10	Topoisome	0.423294	0.001
312	1-O8W-J11	1	Topoisome	0.0456	0.99
313	1-O8W-J20	1	Topoisome	0.103102	0.854
314	1-O8W-K11	1	Topoisome	0.148913	0.697
315	1-O8W-K2(	0.1	Topoisome	0.101045	0.898
316	1-O8W-L11	10	Topoisome	0.42003	0.008
317	1-O8W-L14	1	Topoisome	0.340979	0.138
318	1-O8W-M1	100	Topoisome	0.455441	0.016
319	1-O8W-M1	10	Topoisome	0.443436	0
320	1-O8W-N1	100	Topoisome	0.459471	0
321	1-O8W-O1	1000	Topoisome	0.247681	0.783
322	1-O8W-O1	1000	Topoisome	0.435275	0.005
323	1-O8W-P11	10000	Topoisome	0.196545	0.912
324	1-O8W-P1	10000	Topoisome	0.464727	0.012
325	3-O8W-A11	10000	Topoisome	0.48402	0.001
326	3-O8W-B11	1000	Topoisome	0.514439	0
327	3-O8W-C11	100	Topoisome	0.43778	0
328	3-O8W-D1	10	Topoisome	0.282356	0.164
329	3-O8W-E11	1	Topoisome	0.211171	0.486
330	3-O8W-G9-	1000	Topoisome	0.259516	0.677
331	3-O8W-G1(	10000	Topoisome	0.563025	0
332	3-O8W-H9-	100	Topoisome	0.490529	0
333	3-O8W-H1(	1000	Topoisome	0.375723	0.015
334	3-O8W-I9-[	10	Topoisome	0.407246	0
335	3-O8W-I10	100	Topoisome	0.257668	0.303
336	3-O8W-J9-I	1	Topoisome	0.554996	0
337	3-O8W-J10	10	Topoisome	0.317976	0.066
338	3-O8W-K7-	0.1	Topoisome	0.353133	0.01
339	3-O8W-K9-	0.1	Topoisome	0.105953	0.906
340	3-O8W-K1(	1	Topoisome	0.328557	0.052
341	3-O8W-L6-	0.1	Topoisome	0.313958	0.055
342	3-O8W-L7-	1	Topoisome	0.503042	0
343	3-O8W-L9-	0.5	Topoisome	0.49462	0
344	3-O8W-L1(	0.1	Topoisome	0.497405	0
345	3-O8W-L1€	1	Topoisome	0.300838	0.169

**346**	3-O8W-M6	1	Topoisome	0.399508	0.003
**347**	3-O8W-M7	10	Topoisome	0.507303	0
**348**	3-O8W-M9	5	Topoisome	0.433762	0
**349**	3-O8W-M1	1	Topoisome	0.477117	0
**350**	3-O8W-M1	10	Topoisome	0.467717	0
**351**	3-O8W-N6-	10	Topoisome	0.268059	0.156
**352**	3-O8W-N9-	50	Topoisome	0.475614	0.001
**353**	3-O8W-N1(	10	Topoisome	0.48501	0
**354**	3-O8W-N1(	100	Topoisome	0.038207	0.998
**355**	3-O8W-O6-	100	Topoisome	0.51154	0
**356**	3-O8W-O7-	100	Topoisome	0.483138	0
**357**	3-O8W-O9-	500	Topoisome	0.492613	0
**358**	3-O8W-O1(	100	Topoisome	0.493464	0
**359**	3-O8W-O1(	1000	Topoisome	0.041208	0.999
**360**	3-O8W-P6-	1000	Topoisome	0.481233	0.001
**361**	3-O8W-P7-	1000	Topoisome	0.307891	0.498
**362**	3-O8W-P9-	5000	Topoisome	0.476857	0.006
**363**	3-O8W-P1(	1000	Topoisome	0.479169	0.009
**364**	3-O8W-P1(	10000	Topoisome	0.451522	0
**365**	1-O8W-A1(	10000	Mitotic	0.644444	0
**366**	1-O8W-A1:	1000	Mitotic	0.691684	0
**367**	1-O8W-A1{	1000	Mitotic	0.697305	0
**368**	1-O8W-B1(	1000	Mitotic	0.708493	0
**369**	1-O8W-B1:	100	Mitotic	0.371676	0.019
**370**	1-O8W-B1{	100	Mitotic	0.694618	0
**371**	1-O8W-C1(	100	Mitotic	0.68222	0
**372**	1-O8W-C1:	10	Mitotic	0.359213	0.048
**373**	1-O8W-C1{	10	Mitotic	0.656326	0
**374**	1-O8W-D1(	10	Mitotic	0.500592	0.001
**375**	1-O8W-D1:	1	Mitotic	0.475183	0
**376**	1-O8W-D1{	1	Mitotic	0.362956	0.026
**377**	1-O8W-E1C	1	Mitotic	0.404557	0.004
**378**	1-O8W-E13	0.1	Mitotic	0.133757	0.9
**379**	1-O8W-E18	0.1	Mitotic	0.351605	0.025
**380**	1-O8W-F13	1000	Mitotic	0.705258	0

**381**	1-O8W-G1	100	Mitotic	0.478035	0.001
**382**	1-O8W-G1	1000	Mitotic	0.642546	0
**383**	1-O8W-H1	10	Mitotic	0.367762	0.022
**384**	1-O8W-H1	100	Mitotic	0.685891	0
**385**	1-O8W-I13	1	Mitotic	0.475832	0.007
**386**	1-O8W-I15	10	Mitotic	0.660701	0
**387**	1-O8W-J13	0.1	Mitotic	0.112741	0.837
**388**	1-O8W-J15	1	Mitotic	0.449214	0.001
**389**	1-O8W-K7-	0.1	Mitotic	0.428619	0
**390**	1-O8W-K1	0.1	Mitotic	0.228275	0.716
**391**	1-O8W-L7-	1	Mitotic	0.460088	0.001
**392**	1-O8W-L2	0.1	Mitotic	0.170496	0.957
**393**	1-O8W-M7	10	Mitotic	0.080672	0.973
**394**	1-O8W-M2	1	Mitotic	0.39114	0.013
**395**	1-O8W-N2	10	Mitotic	0.406309	0.027
**396**	1-O8W-O7-	100	Mitotic	0.545074	0
**397**	1-O8W-O2	100	Mitotic	0.477906	0.006
**398**	1-O8W-P7-	1000	Mitotic	0.673995	0
**399**	1-O8W-P2	1000	Mitotic	0.615507	0
**400**	3-O8W-A7-	1000	Mitotic	0.648848	0
**401**	3-O8W-B7-	100	Mitotic	0.651179	0
**402**	3-O8W-C7-	10	Mitotic	0.604545	0
**403**	3-O8W-D7-	1	Mitotic	0.370093	0.02
**404**	3-O8W-E7-	0.1	Mitotic	0.32325	0.065
**405**	6-O8W-L1	1	Mitotic	0.121781	0.831
**406**	6-O8W-M1	10	Mitotic	0.111413	0.847
**407**	6-O8W-N1	100	Mitotic	0.113939	0.855
**408**	6-O8W-O1	1000	Mitotic	0.58769	0
**409**	6-O8W-P1	10000	Mitotic	0.655986	0
**410**	2-O8W-A1	250	MEK1/2	0.498743	0.001
**411**	2-O8W-B1	25	MEK1/2	0.592183	0
**412**	2-O8W-D1	2.5	MEK1/2	0.744836	0
**413**	2-O8W-E12	0.25	MEK1/2	0.304537	0.309
**414**	2-O8W-F12	2.5000000	MEK1/2	0.157088	0.907
**415**	2-O8W-F14	1000	MEK1/2	0.45622	0.017

**416**	2-O8W-G14	100	MEK1/2	0.68128	0
**417**	2-O8W-H14	10	MEK1/2	0.728044	0
**418**	2-O8W-I14	1	MEK1/2	0.243803	0.69
**419**	2-O8W-K14	0.1	MEK1/2	0.552111	0
**420**	2-O8W-L20	1	MEK1/2	0.247796	0.414
**421**	2-O8W-M2	10	MEK1/2	0.625359	0
**422**	2-O8W-N2(	100	MEK1/2	0.735688	0
**423**	2-O8W-O2(	1000	MEK1/2	0.78312	0
**424**	2-O8W-P2(	10000	MEK1/2	0.616495	0
**425**	4-O8W-A1(	1000	MEK1/2	0.739272	0
**426**	4-O8W-A13	1000	MEK1/2	0.581976	0
**427**	4-O8W-B1(	100	MEK1/2	0.788023	0
**428**	4-O8W-B13	100	MEK1/2	0.751005	0
**429**	4-O8W-C1(	10	MEK1/2	0.671696	0
**430**	4-O8W-C13	10	MEK1/2	0.766506	0
**431**	4-O8W-D1(	1	MEK1/2	0.550962	0
**432**	4-O8W-D13	1	MEK1/2	0.715502	0
**433**	4-O8W-E10	0.1	MEK1/2	0.209002	0.726
**434**	4-O8W-E13	0.1	MEK1/2	0.547932	0.001
**435**	4-O8W-L19	0.25	MEK1/2	0.462216	0.007
**436**	4-O8W-M1	2.5	MEK1/2	0.123896	0.945
**437**	4-O8W-N19	25	MEK1/2	0.094576	0.993
**438**	4-O8W-O19	250	MEK1/2	0.617024	0
**439**	4-O8W-P19	2500	MEK1/2	0.80809	0
**440**	1-O8W-L2-	1	PARP	0.56202	0.002
**441**	1-O8W-L6-	1	PARP	0.193741	0.996
**442**	1-O8W-M2	10	PARP	0.549035	0.005
**443**	1-O8W-M6	10	PARP	0.087906	1
**444**	1-O8W-N2-	100	PARP	0.566986	0
**445**	1-O8W-N6-	100	PARP	0.410687	0.246
**446**	1-O8W-O2-	1000	PARP	0.587744	0
**447**	1-O8W-O6-	1000	PARP	0.62363	0
**448**	1-O8W-P2-	10000	PARP	0.608497	0
**449**	1-O8W-P6-	10000	PARP	0.591803	0
**450**	7-O8W-A3-	1000	PARP	0.66603	0

**451**	7-O8W-B2-	10000	PARP	0.623742	0.001
**452**	7-O8W-B3-	100	PARP	0.62888	0
**453**	7-O8W-C2-	1000	PARP	0.569793	0
**454**	7-O8W-C3-	10	PARP	0.639834	0
**455**	7-O8W-D2-	100	PARP	0.586343	0
**456**	7-O8W-D3-	1	PARP	0.595072	0
**457**	7-O8W-E2-'	10	PARP	0.486461	0.004
**458**	7-O8W-E3-'	0.1	PARP	0.58436	0.004
**459**	7-O8W-F2-'	1	PARP	0.120885	0.997
**460**	7-O8W-G2-	10000	PARP	0.635535	0
**461**	7-O8W-H2-	1000	PARP	0.650894	0
**462**	7-O8W-I2-I	100	PARP	0.66351	0
**463**	7-O8W-J2-I	10	PARP	0.54592	0.002
**464**	7-O8W-K2-	1	PARP	0.518362	0.017
**465**	3-O8W-A1!	1000	CDK	0.387183	0.129
**466**	3-O8W-B1!	100	CDK	0.514518	0.02
**467**	3-O8W-B2:	2500	CDK	0.410885	0.012
**468**	3-O8W-C1!	10	CDK	0.394091	0.017
**469**	3-O8W-C2:	250	CDK	0.188609	0.353
**470**	3-O8W-D1!	1	CDK	0.312013	0.011
**471**	3-O8W-D2:	25	CDK	0.184387	0.512
**472**	3-O8W-E1!	0.1	CDK	0.320927	0.068
**473**	3-O8W-E23	2.5	CDK	0.132504	0.917
**474**	3-O8W-F23	0.25	CDK	0.112423	0.907
**475**	3-O8W-K17	1	CDK	0.216532	0.389
**476**	3-O8W-L1!	1	CDK	0.289054	0.028
**477**	3-O8W-M1	10	CDK	0.214287	0.187
**478**	3-O8W-M1	10	CDK	0.26692	0.314
**479**	3-O8W-N1:	100	CDK	0.34385	0.004
**480**	3-O8W-N1!	100	CDK	0.235664	0.121
**481**	3-O8W-O1:	1000	CDK	0.287045	0.085
**482**	3-O8W-O1!	1000	CDK	0.425852	0.005
**483**	3-O8W-P17	10000	CDK	0.387582	0.012
**484**	3-O8W-P1!	10000	CDK	0.435472	0.003
**485**	4-O8W-A4-	10000	CDK	0.422037	0.095

**486**	4-O8W-A8-	10000	CDK	0.4014	0.036
**487**	4-O8W-B4-	1000	CDK	0.461355	0.055
**488**	4-O8W-B8-	1000	CDK	0.179019	0.787
**489**	4-O8W-C4-	100	CDK	0.228359	0.346
**490**	4-O8W-C8-	100	CDK	0.139999	0.892
**491**	4-O8W-D4-	10	CDK	0.220134	0.307
**492**	4-O8W-D8-	10	CDK	0.094502	0.941
**493**	4-O8W-E4-!	1	CDK	0.275474	0.166
**494**	4-O8W-E8-I	1	CDK	0.313021	0.034
**495**	4-O8W-F4-!	10000	CDK	0.246479	0.424
**496**	4-O8W-F22	10000	CDK	0.489546	0.049
**497**	4-O8W-G4-	1000	CDK	0.361935	0.007
**498**	4-O8W-G2:	1000	CDK	0.427109	0.069
**499**	4-O8W-H4-	100	CDK	0.246609	0.52
**500**	4-O8W-H2:	100	CDK	0.394876	0.02
**501**	4-O8W-I4-S	10	CDK	0.214303	0.476
**502**	4-O8W-I22	10	CDK	0.369428	0.015
**503**	4-O8W-J4-!	1	CDK	0.145143	0.952
**504**	4-O8W-J22	1	CDK	0.252068	0.265
**505**	5-O8W-A1!	10000	CDK	0.39067	0.343
**506**	5-O8W-B1!	1000	CDK	0.242877	0.621
**507**	5-O8W-C1!	100	CDK	0.385016	0.012
**508**	5-O8W-D1!	10	CDK	0.327459	0.061
**509**	5-O8W-E1!	1	CDK	0.209302	0.609
**510**	5-O8W-K1:	1	CDK	0.182641	0.353
**511**	5-O8W-M1	10	CDK	0.236422	0.118
**512**	5-O8W-N1:	100	CDK	0.413619	0.01
**513**	5-O8W-O1:	1000	CDK	0.374795	0.326
**514**	5-O8W-P1:	10000	CDK	0.359652	0.284
**515**	6-O8W-A1:	1000	CDK	0.45306	0.001
**516**	6-O8W-B1:	100	CDK	0.159443	0.763
**517**	6-O8W-C1:	10	CDK	0.315646	0.16
**518**	6-O8W-D1:	1	CDK	0.311379	0.06
**519**	6-O8W-E17	0.1	CDK	0.117393	0.77
**520**	6-O8W-L1!	1	CDK	0.105426	0.972

521	6-O8W-M1	10	CDK	0.177333	0.446
522	6-O8W-N1!	100	CDK	0.302915	0.048
523	6-O8W-O1!	1000	CDK	0.426518	0.099
524	6-O8W-P1!	10000	CDK	0.372285	0.24
525	7-O8W-A21	10000	BET	0.56024	0.003
526	7-O8W-A22	30000	BET	0.498112	0
527	7-O8W-B21	1000	BET	0.263423	0.282
528	7-O8W-B22	3000	BET	0.31562	0.038
529	7-O8W-C21	100	BET	0.405363	0.008
530	7-O8W-C22	300	BET	0.656894	0
531	7-O8W-D2:	10	BET	0.280426	0.197
532	7-O8W-D2:	30	BET	0.460526	0.009
533	7-O8W-E21	1	BET	0.203254	0.56
534	7-O8W-E22	3	BET	0.286068	0.111
535	7-O8W-G1(	10000	BET	0.547135	0.001
536	7-O8W-G1!	10000	BET	0.489777	0.005
537	7-O8W-H1(	1000	BET	0.382363	0.024
538	7-O8W-H1!	1000	BET	0.465151	0
539	7-O8W-I10	100	BET	0.46094	0
540	7-O8W-I15	100	BET	0.587408	0
541	7-O8W-J10	10	BET	0.463911	0.006
542	7-O8W-J15	10	BET	0.358424	0.037
543	7-O8W-K1(	1	BET	0.201109	0.785
544	7-O8W-K1:	1	BET	0.545392	0
545	7-O8W-K1!	1	BET	0.726591	0
546	7-O8W-L12	1	BET	0.306606	0.326
547	7-O8W-L13	10	BET	0.482875	0
548	7-O8W-L2C	1	BET	0.214839	0.34
549	7-O8W-L23	0.03	BET	0.248228	0.175
550	7-O8W-M1	10	BET	0.381493	0.112
551	7-O8W-M1	100	BET	0.427922	0.017
552	7-O8W-M2	10	BET	0.275825	0.417
553	7-O8W-M2	0.3	BET	0.269473	0.102
554	7-O8W-N1:	100	BET	0.503149	0
555	7-O8W-N1:	1000	BET	0.568507	0.003

**556**	7-O8W-N2(	100	BET	0.528457	0
**557**	7-O8W-N2:	3	BET	0.529163	0
**558**	7-O8W-O1:	1000	BET	0.409686	0.003
**559**	7-O8W-O2(	1000	BET	0.522437	0.002
**560**	7-O8W-O2:	30	BET	0.512173	0.007
**561**	7-O8W-P1:	10000	BET	0.392038	0.045
**562**	7-O8W-P1:	10000	BET	0.252592	0.899
**563**	7-O8W-P2(	10000	BET	0.549371	0.001
**564**	7-O8W-P2:	300	BET	0.251443	0.926
**565**	8-O8W-K2:	1	BET	0.22107	0.229
**566**	8-O8W-L22	10	BET	0.174785	0.498
**567**	8-O8W-M2	100	BET	0.294825	0.061
**568**	8-O8W-N2:	1000	BET	0.375267	0.031
**569**	8-O8W-O2:	10000	BET	0.332503	0.17
**570**	1-O8W-A3-	10000	HDAC	0.44595	0.037
**571**	1-O8W-B3-	1000	HDAC	0.328868	0.008
**572**	1-O8W-C3-	100	HDAC	0.309595	0.005
**573**	1-O8W-D3-	10	HDAC	0.289193	0.007
**574**	1-O8W-E3-'	1	HDAC	0.262487	0.056
**575**	1-O8W-L12	0.1	HDAC	0.056056	0.974
**576**	1-O8W-M1	1	HDAC	0.166332	0.587
**577**	1-O8W-N1:	10	HDAC	0.343048	0.008
**578**	1-O8W-O1:	100	HDAC	0.41187	0.047
**579**	1-O8W-P1:	1000	HDAC	0.434527	0.005
**580**	3-O8W-A4-	1000	HDAC	0.384284	0.082
**581**	3-O8W-B4-	100	HDAC	0.39759	0.058
**582**	3-O8W-C4-	10	HDAC	0.347352	0
**583**	3-O8W-D4-	1	HDAC	0.26242	0.038
**584**	3-O8W-E4-I	0.1	HDAC	0.292216	0.022
**585**	3-O8W-F7-(	1000	HDAC	0.44331	0.008
**586**	3-O8W-G7-	100	HDAC	0.321689	0.016
**587**	3-O8W-G1:	1000000	HDAC	0.193051	0.27
**588**	3-O8W-H7-	10	HDAC	0.375162	0
**589**	3-O8W-H1:	100000	HDAC	0.182604	0.496
**590**	3-O8W-I7-(	1	HDAC	0.109465	0.81

**591**	3-O8W-I12	10000	HDAC	0.080779	0.888
**592**	3-O8W-J7-(	0.1	HDAC	0.330394	0.01
**593**	3-O8W-J12	1000	HDAC	0.086115	0.937
**594**	3-O8W-K3-	1	HDAC	0.241109	0.367
**595**	3-O8W-K12	100	HDAC	0.109194	0.861
**596**	3-O8W-L3-I	10	HDAC	0.103638	0.884
**597**	3-O8W-M3	100	HDAC	0.101344	0.95
**598**	3-O8W-N3-	1000	HDAC	0.360417	0.055
**599**	3-O8W-O3-	10000	HDAC	0.413966	0.04
**600**	7-O8W-A5-	10000	HDAC	0.454834	0.011
**601**	7-O8W-A7-	10000	HDAC	0.433896	0.036
**602**	7-O8W-A9-	1000	HDAC	0.433394	0.016
**603**	7-O8W-A12	10000	HDAC	0.424282	0.029
**604**	7-O8W-B5-	1000	HDAC	0.366937	0.001
**605**	7-O8W-B7-	1000	HDAC	0.422971	0.027
**606**	7-O8W-B12	1000	HDAC	0.417426	0
**607**	7-O8W-C5-	100	HDAC	0.327559	0.008
**608**	7-O8W-C7-	100	HDAC	0.451473	0.013
**609**	7-O8W-C9-	100	HDAC	0.41298	0
**610**	7-O8W-D7-	10	HDAC	0.368734	0.003
**611**	7-O8W-D9-	10	HDAC	0.414083	0
**612**	7-O8W-D12	100	HDAC	0.209404	0.537
**613**	7-O8W-E5-I	10	HDAC	0.322495	0.002
**614**	7-O8W-E7-(	1	HDAC	0.393682	0.009
**615**	7-O8W-E9-(	1	HDAC	0.460856	0
**616**	7-O8W-E12	10	HDAC	0.22011	0.447
**617**	7-O8W-F5-I	1	HDAC	0.335942	0.001
**618**	7-O8W-F7-I	10000	HDAC	0.430762	0.002
**619**	7-O8W-F9-(	0.1	HDAC	0.356342	0
**620**	7-O8W-F12	1	HDAC	0.361125	0
**621**	7-O8W-F19	10000	HDAC	0.362608	0.001
**622**	7-O8W-G7-	1000	HDAC	0.366303	0.002
**623**	7-O8W-G19	1000	HDAC	0.282766	0.052
**624**	7-O8W-H7-	100	HDAC	0.415384	0
**625**	7-O8W-I7-F	10	HDAC	0.305334	0.126

**626**	7-O8W-I19	100	HDAC	0.30727	0.019
**627**	7-O8W-J7-	1	HDAC	0.259578	0.226
**628**	7-O8W-J19	10	HDAC	0.171792	0.621
**629**	7-O8W-K4-	1	HDAC	0.352692	0.001
**630**	7-O8W-K11	1	HDAC	0.099377	0.83
**631**	7-O8W-K18	1	HDAC	0.075031	0.941
**632**	7-O8W-K19	1	HDAC	0.068482	0.967
**633**	7-O8W-L2-	0.1	HDAC	0.286009	0.014
**634**	7-O8W-L4-	10	HDAC	0.111901	0.666
**635**	7-O8W-L5-	1	HDAC	0.121111	0.801
**636**	7-O8W-L8-	1	HDAC	0.431229	0
**637**	7-O8W-L10	1	HDAC	0.408141	0
**638**	7-O8W-L11	10	HDAC	0.359875	0.002
**639**	7-O8W-L14	1	HDAC	0.087681	0.898
**640**	7-O8W-L16	1	HDAC	0.096763	0.888
**641**	7-O8W-L18	10	HDAC	0.121964	0.695
**642**	7-O8W-M2	1	HDAC	0.345369	0.002
**643**	7-O8W-M5	10	HDAC	0.341387	0.004
**644**	7-O8W-M8	10	HDAC	0.384809	0
**645**	7-O8W-M1	10	HDAC	0.377552	0
**646**	7-O8W-M1	100	HDAC	0.390059	0.001
**647**	7-O8W-M1	10	HDAC	0.340589	0
**648**	7-O8W-M1	10	HDAC	0.209455	0.604
**649**	7-O8W-M1	100	HDAC	0.31783	0.004
**650**	7-O8W-N2-	10	HDAC	0.424563	0
**651**	7-O8W-N4-	100	HDAC	0.426072	0.001
**652**	7-O8W-N5-	100	HDAC	0.442722	0
**653**	7-O8W-N8-	100	HDAC	0.460645	0
**654**	7-O8W-N1(	100	HDAC	0.335675	0.002
**655**	7-O8W-N14	100	HDAC	0.2846	0.028
**656**	7-O8W-N16	100	HDAC	0.035193	0.99
**657**	7-O8W-N18	1000	HDAC	0.141642	0.752
**658**	7-O8W-O2-	100	HDAC	0.387855	0
**659**	7-O8W-O4-	1000	HDAC	0.42848	0
**660**	7-O8W-O5-	1000	HDAC	0.431295	0.016

**661**	7-O8W-O8-	1000	HDAC	0.437845	0.014
**662**	7-O8W-O1(	1000	HDAC	0.393655	0.001
**663**	7-O8W-O1:	1000	HDAC	0.384123	0.001
**664**	7-O8W-O14	1000	HDAC	0.440466	0
**665**	7-O8W-O16	1000	HDAC	0.385518	0
**666**	7-O8W-P2-	1000	HDAC	0.171882	0.454
**667**	7-O8W-P4-	10000	HDAC	0.411145	0.009
**668**	7-O8W-P5-	10000	HDAC	0.438493	0.026
**669**	7-O8W-P8-	10000	HDAC	0.495754	0.002
**670**	7-O8W-P1(	10000	HDAC	0.380214	0.001
**671**	7-O8W-P11	10000	HDAC	0.412314	0.042
**672**	7-O8W-P14	10000	HDAC	0.37504	0
**673**	7-O8W-P16	10000	HDAC	0.465294	0
**674**	7-O8W-P18	10000	HDAC	0.360351	0.004
