# Supplementary material for: Evaluating feature extraction in ovarian cancer cell line co-cultures using deep neural networks
Source: Commun Biol. 2025 Feb 25;8:303. doi: 10.1038/s42003-025-07766-w (PMC11862010; doi:10.1038/s42003-025-07766-w)
Supplement: Supplementary file 10 — reporting-summary [file 42003_2025_7766_MOESM10_ESM.pdf]

nature portfolio

Corresponding author(s): DAPR
Manuscript number: COMMSBIO-24-4129A

Last updated by author(s): Dec 11, 2024

Reporting Summary

Nature Portfolio wishes to improve the reproducibility of the work that we publish. This form provides structure for consistency and transparency in reporting. For further information on Nature Portfolio policies, see our Editorial Policies and the Editorial Policy Checklist.

Statistics

For all statistical analyses, confirm that the following items are present in the figure legend, table legend, main text, or Methods section.

n/a	Confirmed	
☐	☒	The exact sample size (*n*) for each experimental group/condition, given as a discrete number and unit of measurement
☐	☒	A statement on whether measurements were taken from distinct samples or whether the same sample was measured repeatedly
☒	☐	The statistical test(s) used AND whether they are one- or two-sided<br>*Only common tests should be described solely by name; describe more complex techniques in the Methods section.*
☒	☐	A description of all covariates tested
☐	☒	A description of any assumptions or corrections, such as tests of normality and adjustment for multiple comparisons
☐	☒	A full description of the statistical parameters including central tendency (e.g. means) or other basic estimates (e.g. regression coefficient) AND variation (e.g. standard deviation) or associated estimates of uncertainty (e.g. confidence intervals)
☐	☒	For null hypothesis testing, the test statistic (e.g. *F*, *t*, *r*) with confidence intervals, effect sizes, degrees of freedom and *P* value noted<br>*Give P values as exact values whenever suitable.*
☒	☐	For Bayesian analysis, information on the choice of priors and Markov chain Monte Carlo settings
☐	☒	For hierarchical and complex designs, identification of the appropriate level for tests and full reporting of outcomes
☒	☐	Estimates of effect sizes (e.g. Cohen's *d*, Pearson's *r*), indicating how they were calculated

Our web collection on statistics for biologists contains articles on many of the points above.

Software and code

Policy information about availability of computer code

Data collection	The image dataset is available on: https://snd.se/en/catalogue/dataset/preview/836f1844-0987-40e1-9f1d-5c6f49fe08b1/1<br>Harmony Perkin Elmer V5.2<br>OperaPhenix, Revvity
Data analysis	The scipts and CellProfiler pipelines are available on: https://github.com/osharma08/ovarian_cancer_morphology<br>CellProfiler V4.2.4<br>RStudio V4.3.1<br>Python Version python3

For manuscripts utilizing custom algorithms or software that are central to the research but not yet described in published literature, software must be made available to editors and reviewers. We strongly encourage code deposition in a community repository (e.g. GitHub). See the Nature Portfolio guidelines for submitting code & software for further information.

Data

Policy information about availability of data

All manuscripts must include a data availability statement. This statement should provide the following information, where applicable:

- Accession codes, unique identifiers, or web links for publicly available datasets
- A description of any restrictions on data availability
- For clinical datasets or third party data, please ensure that the statement adheres to our policy

Link to the dataset: https://snd.se/en/catalogue/dataset/preview/836f1844-0987-40e1-9f1d-5c6f49fe08b1/1
Please note: This link is the "closed review"-link (not to be published as a URL to the dataset in a Data Accessibility Statement). Here is the DOI reserved for my dataset: https://doi.org/10.48723/srtg-ss33
The DOI will not resolve to the data description in the SND catalogue until the research work is accepted for publication.

Human research participants

Policy information about studies involving human research participants and Sex and Gender in Research.

Reporting on sex and gender	*Use the terms sex (biological attribute) and gender (shaped by social and cultural circumstances) carefully in order to avoid confusing both terms. Indicate if findings apply to only one sex or gender; describe whether sex and gender were considered in study design whether sex and/or gender was determined based on self-reporting or assigned and methods used. Provide in the source data disaggregated sex and gender data where this information has been collected, and consent has been obtained for sharing of individual-level data; provide overall numbers in this Reporting Summary. Please state if this information has not been collected. Report sex- and gender-based analyses where performed, justify reasons for lack of sex- and gender-based analysis.*
Population characteristics	*Describe the covariate-relevant population characteristics of the human research participants (e.g. age, genotypic information, past and current diagnosis and treatment categories). If you filled out the behavioural & social sciences study design questions and have nothing to add here, write "See above."*
Recruitment	*Describe how participants were recruited. Outline any potential self-selection bias or other biases that may be present and how these are likely to impact results.*
Ethics oversight	*Identify the organization(s) that approved the study protocol.*

Note that full information on the approval of the study protocol must also be provided in the manuscript.

Field-specific reporting

Please select the one below that is the best fit for your research. If you are not sure, read the appropriate sections before making your selection.

☒ Life sciences ☐ Behavioural & social sciences ☐ Ecological, evolutionary & environmental sciences

For a reference copy of the document with all sections, see nature.com/documents/nr-reporting-summary-flat.pdf

Life sciences study design

All studies must disclose on these points even when the disclosure is negative.

Sample size	We have 5 co-culture combinations of cancer cell lines along with fibroblasts. Each co-culture combination is treated with 528 drugs at 5 different concentrations. We acquired images at 10x resolution with 4 channels: red, green, blue, and brightfield, generating 245760 raw images.<br>Number of 384 well plates per combination: 8<br>Number of channels per image: 4<br>Field of view per image: 4<br>Total: 5*8*384*4*4 = 245760
Data exclusions	We excluded the Brightfield channel from the analysis and used only the red, green, and blue channels. Our analysis focused on observing changes in the cancer cells in the presence of fibroblasts, without explicitly analyzing the fibroblast cells. Instead, we used cancer-segmented masks to analyze the fibroblast channels.<br><br>Brightfield channel was excluded because the primary focus of our analysis was on fluorescent signals from the red, green, and blue channels, which provide specific information about the cellular and molecular changes under different treatments. The Brightfield channel was not necessary for this specific analysis and was excluded to streamline the data processing.
Replication	Single cell features were extracted and compared the abilities of CellProfiler and neural networks. All pipelines from CellProfiler and the codes

Replication	used for extracting features from these co-culture assays are available in provided GitHub repository. Additionally, R scripts for plots are also provided in the repository, making it possible to replicate our results.
Randomization	We ensured that the results observed were specifically due to treatment effects and not batch effects. We performed technical validations using Z' scores and checked for batch effects using UMAPs, as reported in the supplementary material. No batch effects were observed.
Blinding	Although I was aware of the treatment conditions, the feature extraction process was blinded to these conditions. During feature extraction, the network did not know which drug or concentration the images corresponded to (unsupervised learning). After features were extracted, they were annotated and mapped to the corresponding treatments for subsequent enrichment analysis.

Reporting for specific materials, systems and methods

We require information from authors about some types of materials, experimental systems and methods used in many studies. Here, indicate whether each material, system or method listed is relevant to your study. If you are not sure if a list item applies to your research, read the appropriate section before selecting a response.

Materials & experimental systems

n/a	Involved in the study	
☐	☒	Antibodies
☐	☒	Eukaryotic cell lines
☒	☐	Palaeontology and archaeology
☒	☐	Animals and other organisms
☒	☐	Clinical data
☒	☐	Dual use research of concern

Methods

n/a	Involved in the study	
☒	☐	ChIP-seq
☒	☐	Flow cytometry
☒	☐	MRI-based neuroimaging

Antibodies

Antibodies used	Primary Antibodies: (Antibody name, source, identifier)<br>Cytokeratin 8/18 (CK8/18), Agilent, M3652<br>Vimentin, Invitrogen, MA5-11883<br><br>Cytokeratin 8/18 primary antibody is a cocktail of two monoclonal IgG class antibodies produced in rabbit. These antibodies are of EP17/EP30 clone and have binding specificity to human protein.<br>Vimentin primary antibody is a monoclonal IgG1 kappa class antibody produced in mouse.<br><br>Both describes primary antibodies were used in a cocktail for co-staining. First each antibody was tested individualy on cells known to express the respective markers (Kuramochi for CK8/18, and BjHTERT for Vimentin). Final antibody concentrations and reactivity were optimized before generating the dataset for the study.<br><br>Seconday antibodies:(Antibody name, source, identifier)<br>Donkey anti-Rabbit Secondary Antibody, Alexa Fluor™ 568 Invitrogen, A10042<br>Donkey anti-Mouse Secondary Antibody, Alexa Fluor™ 488 Invitrogen, A21202<br>Donkey anti-Rabbit Secondary Antibody, Alexa Fluor™ 488 Invitrogen ,A21206<br>Donkey anti-Mouse Secondary Antibody, Alexa Fluor™ 568 Invitrogen, A10037
Validation	According to manufacturer, Cytokeratin 8/18 antibody's specificity was validated in Wester blotting assay using A431 cell lysates (epithelial carcinoma) (https://www.agilent.com/en/product/immunohistochemistry/antibodies-controls/primary-antibodies/cytokeratin-8-18-%28concentrate%29-76615).<br><br>The manufacturer validated Vimentin antibody for Western blotting, IHC, IF, IP (https://www.thermofisher.com/antibody/product/Vimentin-Antibody-clone-V9-Monoclonal/MA5-11883).

Eukaryotic cell lines

Policy information about cell lines and Sex and Gender in Research

Cell line source(s)	Cell lines (KURAMOCHI, OVCAR3, OVCAR8, MH, BjHTERT) were obtained from the Institute Molecular Medicine Finland (FIMM) and provided by Dr. Astrid Muramägi. WI38 cell lines was purchased from VWR, USA.
Authentication	All cell lines were routinely sent for short tandem repeat (STR) profiling using to Human Cell lIne Authentication Services (Eurofins). For this cell line pellet was collected, snap frozen and sent to the facility.
Mycoplasma contamination	Cell lines were routinely tested for mycoplasma using MycoAlert Mycoplasma Detection Kit (LT07-418).
Commonly misidentified lines<br>(See ICLAC register)	None of the cell lines are commonly misidentified.
